# Supplementary material for: PCPE-1, a brown adipose tissue-derived cytokine, promotes obesity-induced liver fibrosis
Source: EMBO J. 2024 Aug 19;43(21):4846–69. doi: 10.1038/s44318-024-00196-0 (PMC11535236; doi:10.1038/s44318-024-00196-0)
Supplement: Supplementary file 1 — Appendix [file 44318_2024_196_MOESM1_ESM.pdf]

PCPE-1, a brown adipose tissue-derived cytokine, promotes obesity-induced liver fibrosis

Manuscript: EMBOJ-2023-115949R

Appendix

Table of Contents

Appendix Figure S1 2
Appendix Figure S2 3
Appendix Figure S3 5
Appendix Figure S4 6

Appendix Figure S1

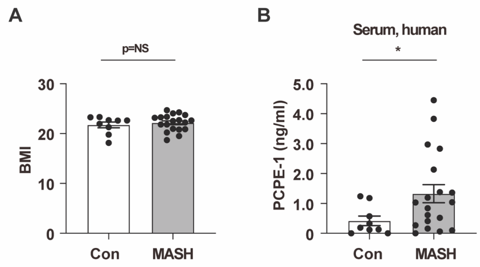

Appendix Figure S1. Related to Fig. 1.

(A) BMI of control (Con) or MASH patients showing BMI<25 (n = 9, 19). **(B)** ELISA for human PCPE-1 in serum from the BMI adjusted (BMI<25) control (Con) or MASH patients as demonstrated in Appendix Figure S1A (n = 9, 19). All data were analyzed by an independent-samples T-test. *P< 0.05, **P< 0.01. NS = not significant. Values represent the mean ± SEM. All data are from different biological replicates. Source data are available online for these figures.

Appendix Figure S2

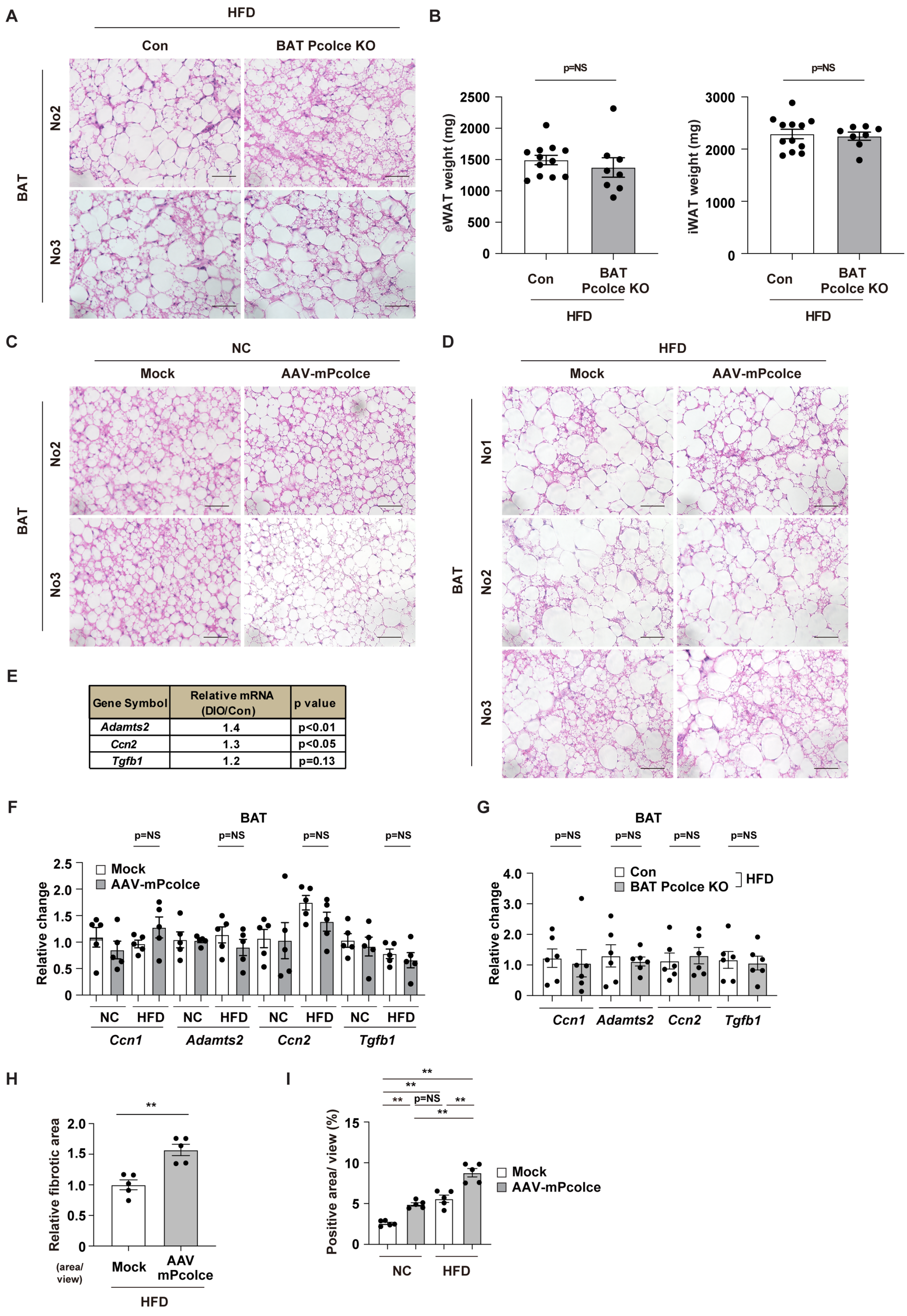

Appendix Figure S2. Related to Fig. 2.

(A) HE staining of the BAT from the Brown adipose tissue (BAT)-specific *Pcolce*

knockout (KO) mice (UCP1-Cre$^{+/-}$: *Pcolce*$^{flox/flox}$; BAT *Pcolce* KO) or littermate control mice (UCP1-Cre$^{-/-}$; *Pcolce*$^{flox/flox}$: Con) fed a high-fat diet (HFD). No1 mice are demonstrated in Figure EV2F. Scale bar = 50 μm. **(B)** Weight of epididymal white adipose tissue (eWAT) or inguinal white adipose tissue (iWAT) of the indicated mice aged 42-43 weeks of age (n=12,8). **(C, D)** HE staining of the BAT from the BAT AAV-mouse Pcolce injection model (AAV-mPcolce) maintained on a normal chow (NC)(C) or a high fat diet (HFD)(D). No1 mice for Appendix Figure S2C are demonstrated in Figure EV2M. Scale bar = 50 μm. **(E)** Results of DNA microarray datasets showing the level of transcripts *Adamts2*, *Ccn2* (Ctgf) and *Tgfb1* in brown adipose tissue (BAT) from high-fat diet (HFD) fed wild type mice (diet-induced obesity (DIO)) (GSE28440). **(F, G)** Results from quantitative PCR (qPCR) showing transcripts *Ccn1*, *Adamts2*, *Ccn2*, *Tgfb1* in BAT of the indicated mice (n=5,5,5,5)(F), (n=6,6)(G). **(H)** Relative fibrotic area in the liver of indicated mice, related to Figure EV2Q (n=5,5). **(I)** Positive area/ view (%) analyzing fibrotic area in the liver of indicated mice (n=5,5,5,5). The data in Figures F and I were analyzed by a two-way analysis of variance (ANOVA) followed by Tukey's multiple comparison test (F) or Dunnett test (I). Other data were analyzed by an independent-samples T-test. *$P < 0.05$, **$P < 0.01$. NS = not significant. Values represent the mean ± SEM. All data are from different biological replicates. Source data are available online for these figures.

Appendix Figure S3

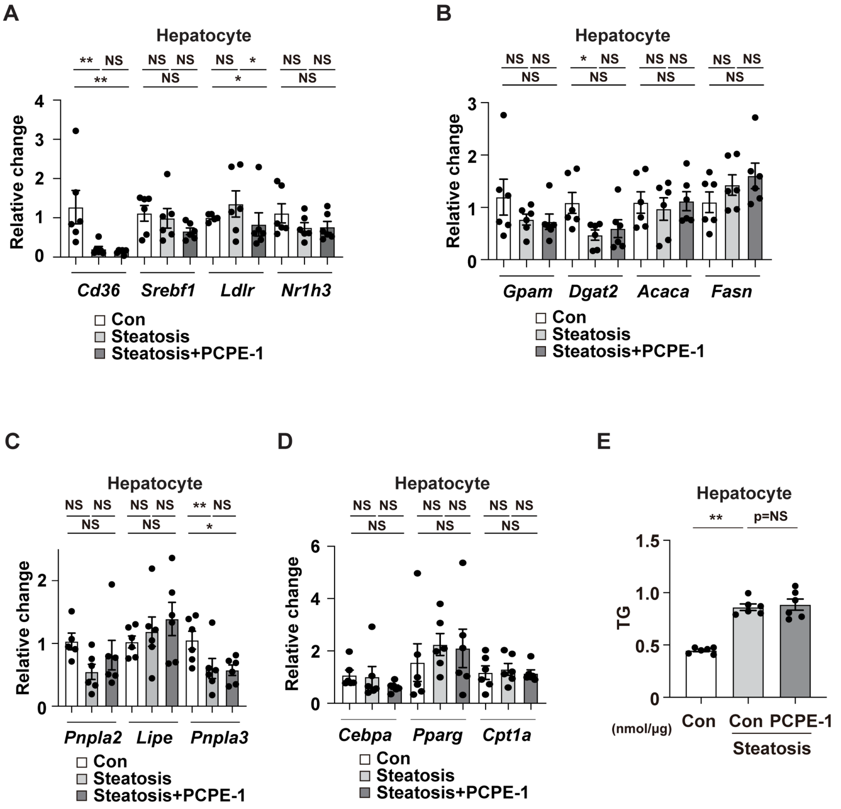

Appendix Figure S3. Related to Fig. 2.

(A-D) Results from quantitative PCR (qPCR) showing transcripts involved in the uptake (A), synthesis (B), lipolysis (C) and metabolism (D) of lipids in hepatocyte cell line (AML12) maintained under steatosis medium with or without recombinant PCPE-1 protein (n=6,6,6)(*Ldlr*: n=5,6,6, *Pnpla2*: n=5,6,6) **(E)** ELISA for triglyceride (TG) in hepatocyte cell line (AML12) maintained under steatosis medium with or without recombinant PCPE-1 protein(n=6,6,6). All data were analyzed by a two-way analysis of variance (ANOVA) followed by Tukey's multiple comparison test (B, *Pnpla2* and *Lipe*(C), *Cebpa* and *Pparg* (D)), or non-parametric Kruskal Wallis test (A, *Pnpla3*(C), *Cpt1a*(D), E). *P< 0.05, **P< 0.01. NS = not significant. Values represent the mean ± SEM. All data are from different biological replicates. Source data are available online for these figures.

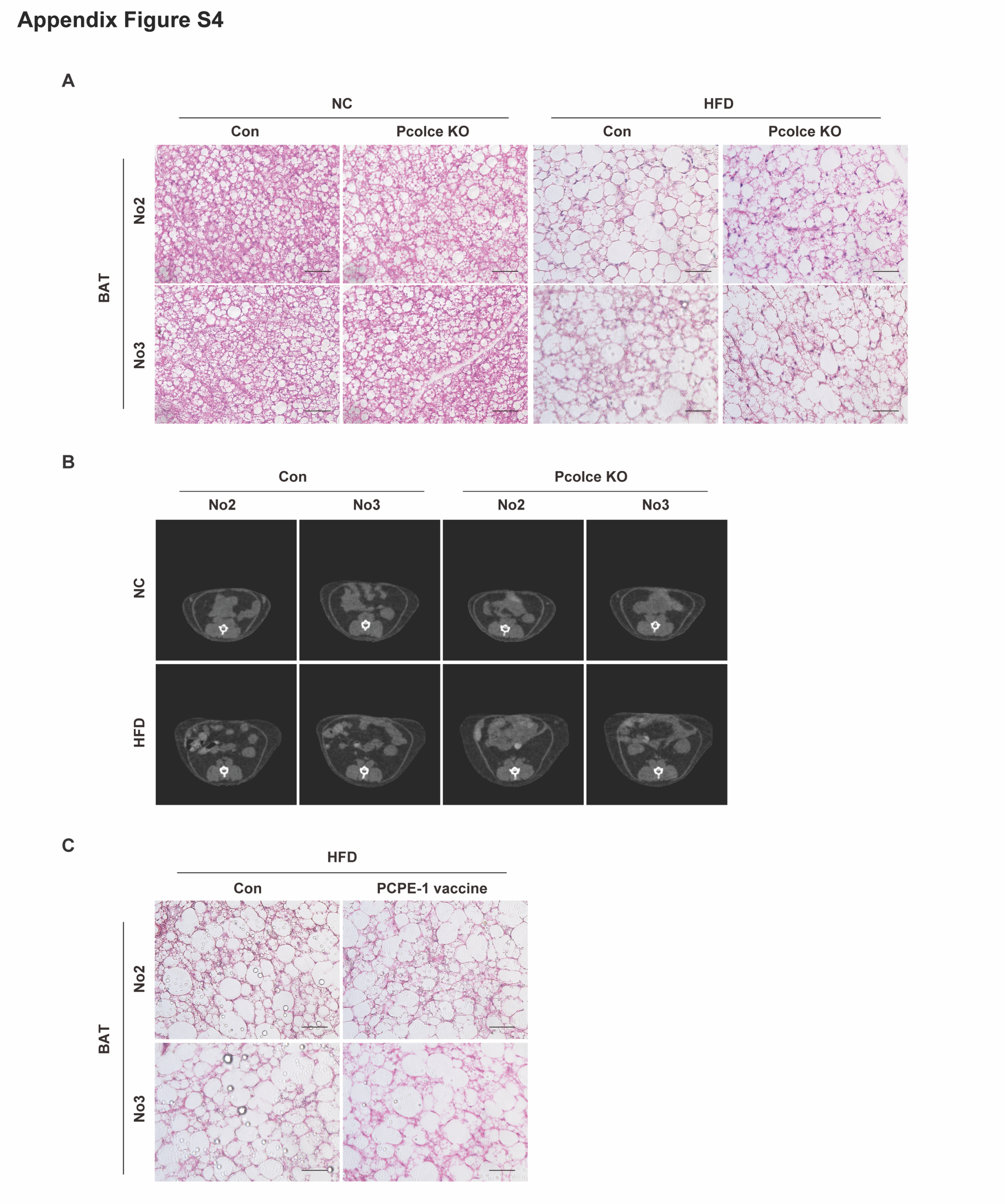

Appendix Figure S4. Related to Fig. 3.

(A, C) HE staining of the BAT from the systemic *Pcolce* knockout (*Pcolce* KO) or littermate control (Con) mice maintained on a normal chow (NC) or a high fat diet (HFD)(A), or from PCPE-1 vaccine therapy group maintained on HFD(C). Scale bar = 50 μm. Respective No1 mice are demonstrated in Figure EV3G or EV3O. **(B)** CT scan analyzing the abdominal area of indicated mice aged 29 weeks of age. No1 mice are demonstrated in Figure EV3H. Source data are available online for these figures.
