# Supplementary figures and images for: PCPE-1, a brown adipose tissue-derived cytokine, promotes obesity-induced liver fibrosis (part 2 of 6)
Source: EMBO J. 2024 Aug 19;43(21):4846–69. doi: 10.1038/s44318-024-00196-0 (PMC11535236; doi:10.1038/s44318-024-00196-0)

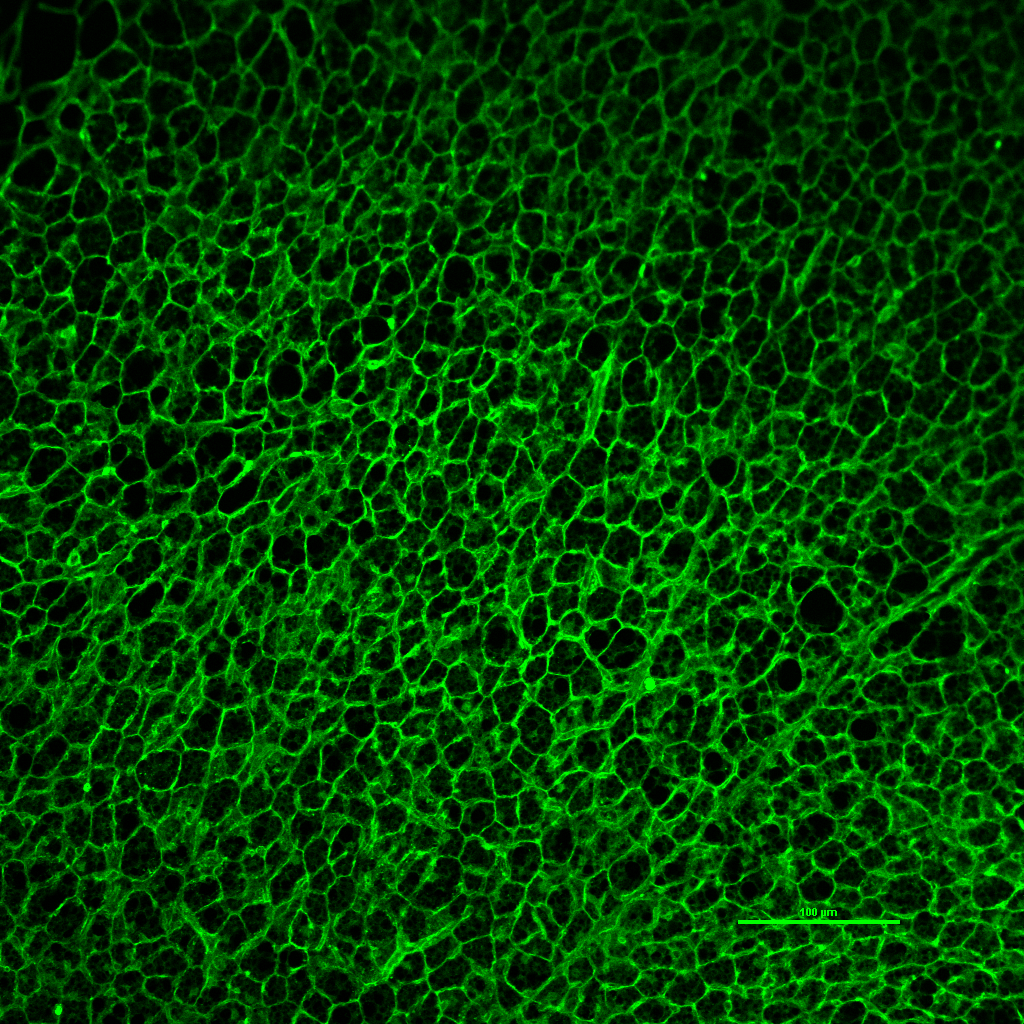

Supplement: Supplementary file 3 — Source data Fig. 1 [file 44318_2024_196_MOESM3_ESM.zip › Figure 1/Figure 1-D/Quantificated image/NC/no.3/NC BAT_no.3_RGB_WGA lectin_FITC-2.tif]

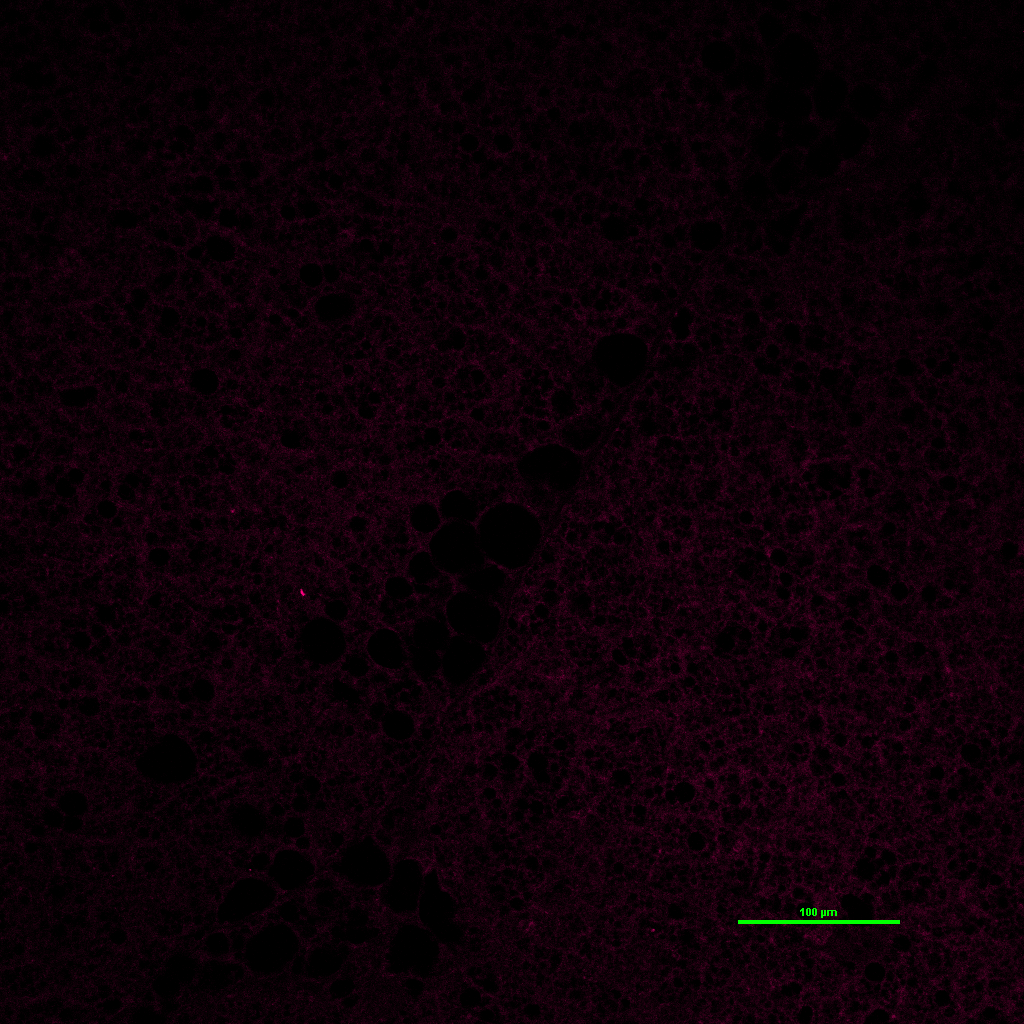

Supplement: Supplementary file 3 — Source data Fig. 1 [file 44318_2024_196_MOESM3_ESM.zip › Figure 1/Figure 1-D/Quantificated image/NC/no.3/NC BAT_no.3_RGB_PCPE-1_Cy5-4.tif]

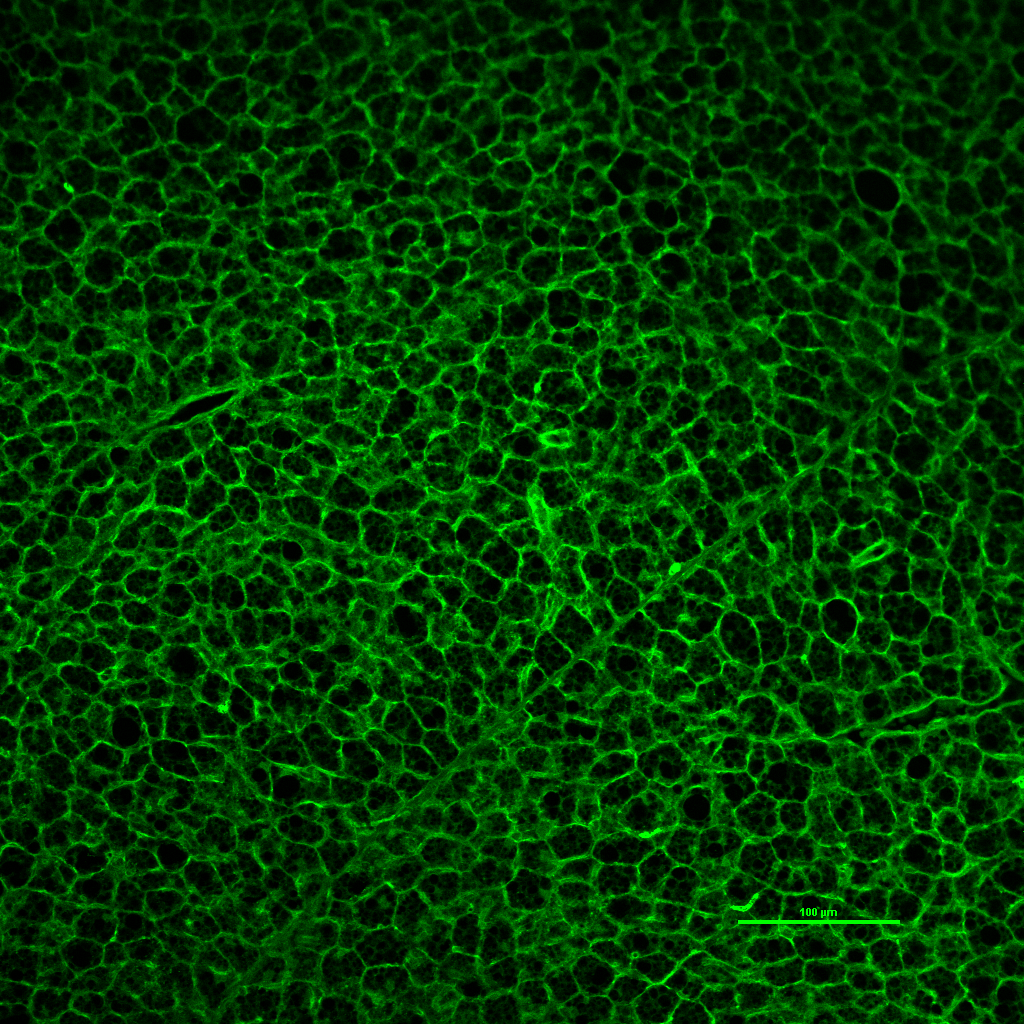

Supplement: Supplementary file 3 — Source data Fig. 1 [file 44318_2024_196_MOESM3_ESM.zip › Figure 1/Figure 1-D/Quantificated image/NC/no.3/NC BAT_no.3_RGB_WGA lectin_FITC-3.tif]

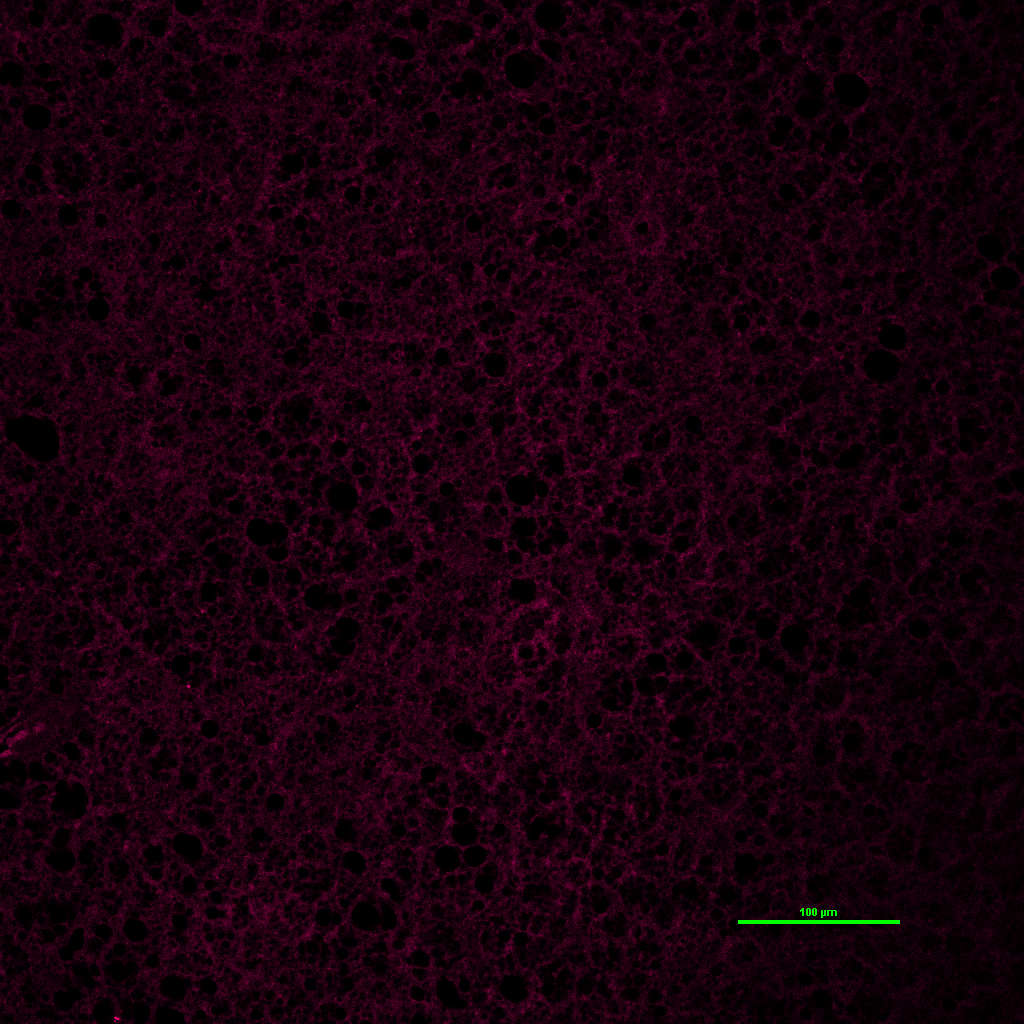

Supplement: Supplementary file 3 — Source data Fig. 1 [file 44318_2024_196_MOESM3_ESM.zip › Figure 1/Figure 1-D/Quantificated image/NC/no.3/NC BAT_no.3_RGB_PCPE-1_Cy5-1.tif]

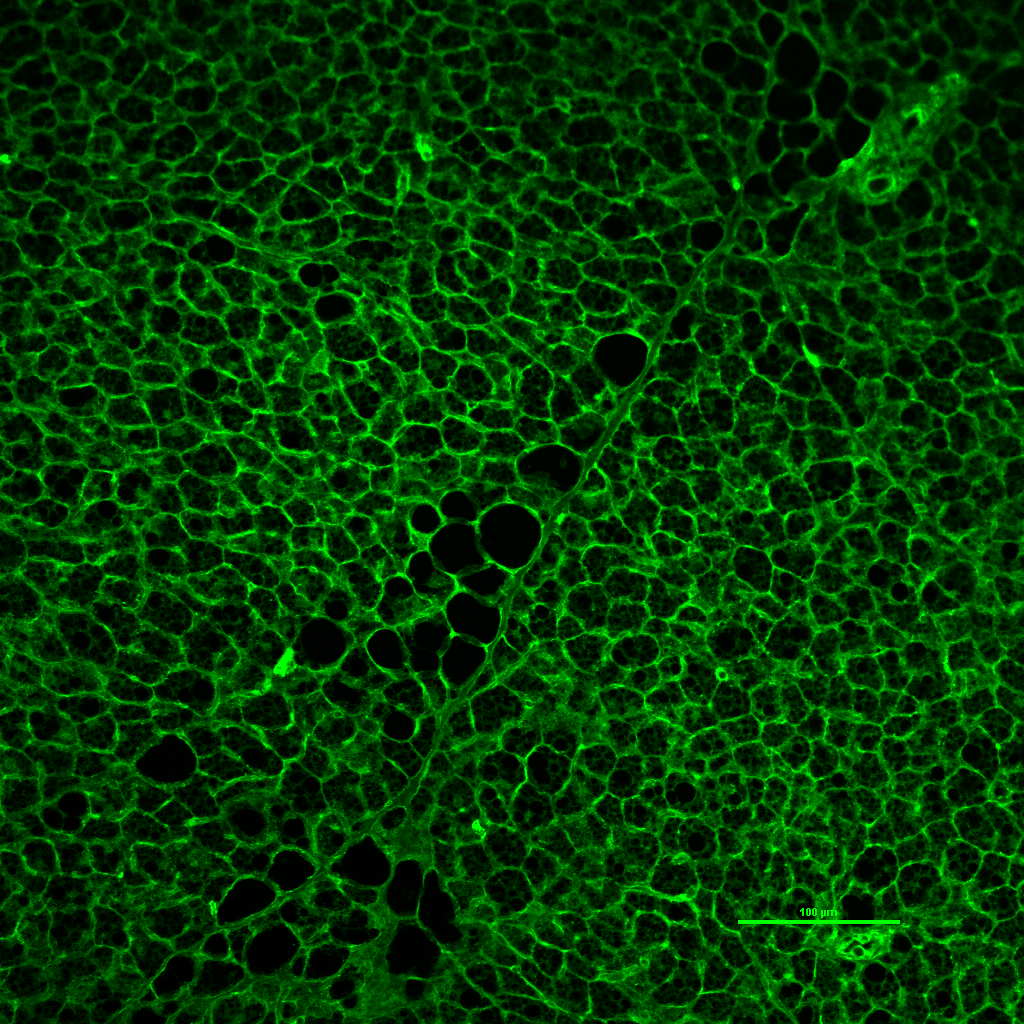

Supplement: Supplementary file 3 — Source data Fig. 1 [file 44318_2024_196_MOESM3_ESM.zip › Figure 1/Figure 1-D/Quantificated image/NC/no.3/NC BAT_no.3_RGB_WGA lectin_FITC-4.tif]

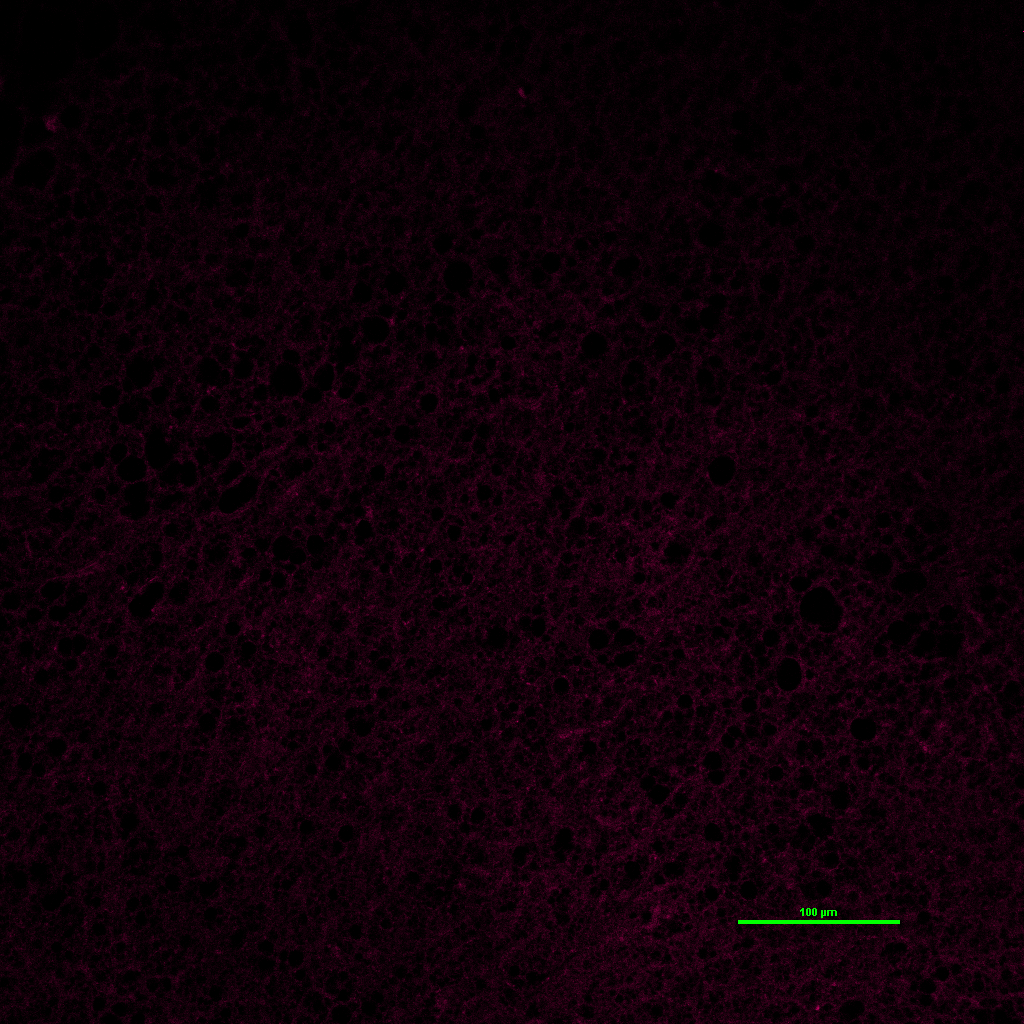

Supplement: Supplementary file 3 — Source data Fig. 1 [file 44318_2024_196_MOESM3_ESM.zip › Figure 1/Figure 1-D/Quantificated image/NC/no.3/NC BAT_no.3_RGB_PCPE-1_Cy5-2.tif]

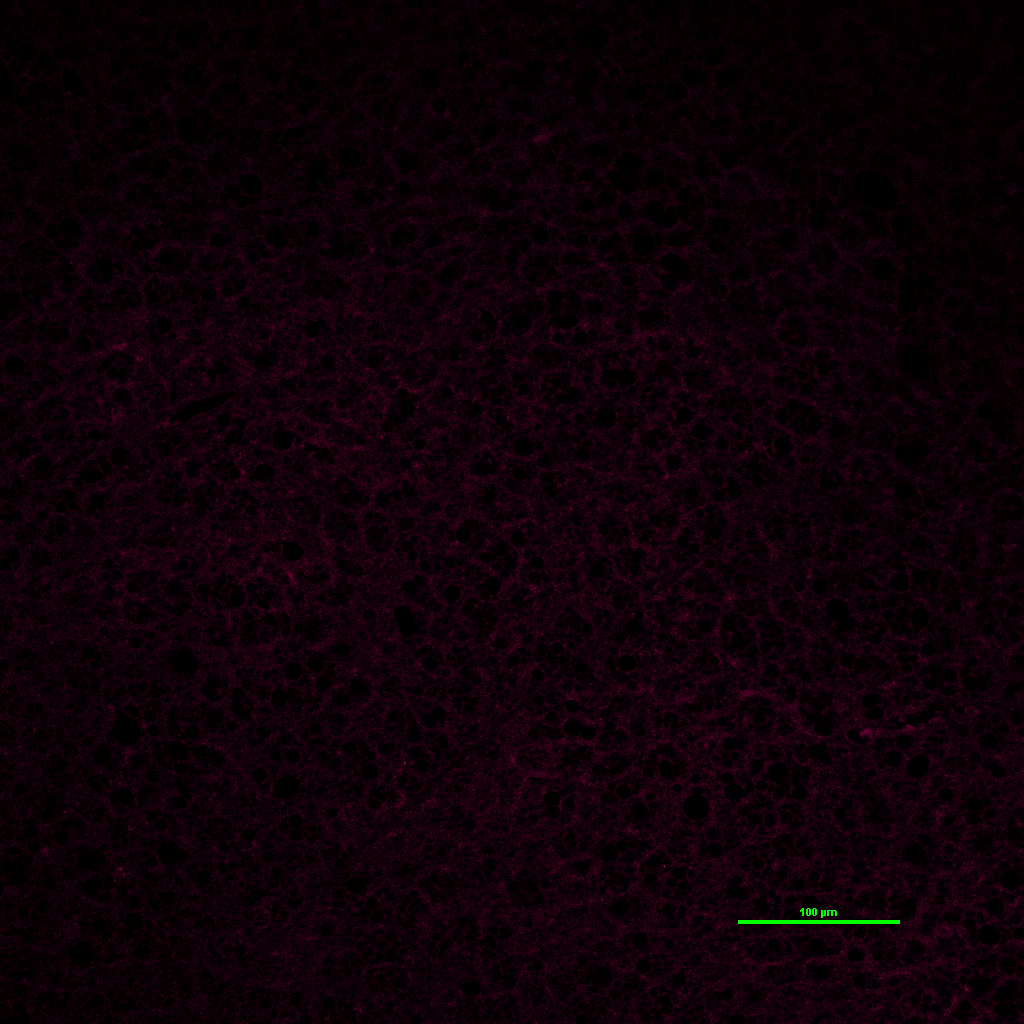

Supplement: Supplementary file 3 — Source data Fig. 1 [file 44318_2024_196_MOESM3_ESM.zip › Figure 1/Figure 1-D/Quantificated image/NC/no.3/NC BAT_no.3_RGB_PCPE-1_Cy5-3.tif]

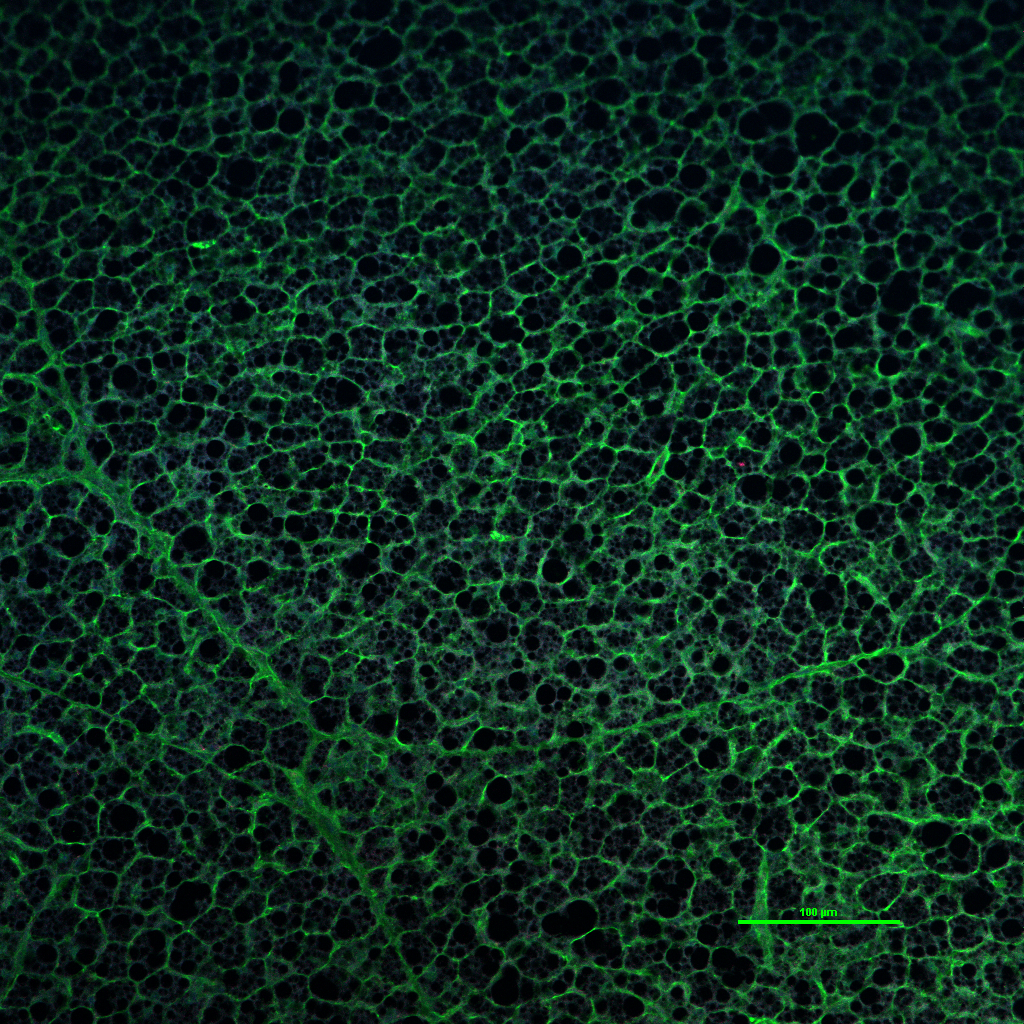

Supplement: Supplementary file 3 — Source data Fig. 1 [file 44318_2024_196_MOESM3_ESM.zip › Figure 1/Figure 1-D/Quantificated image/NC/no.4/NC BAT_no.4_RGB_Merge-4.tif]

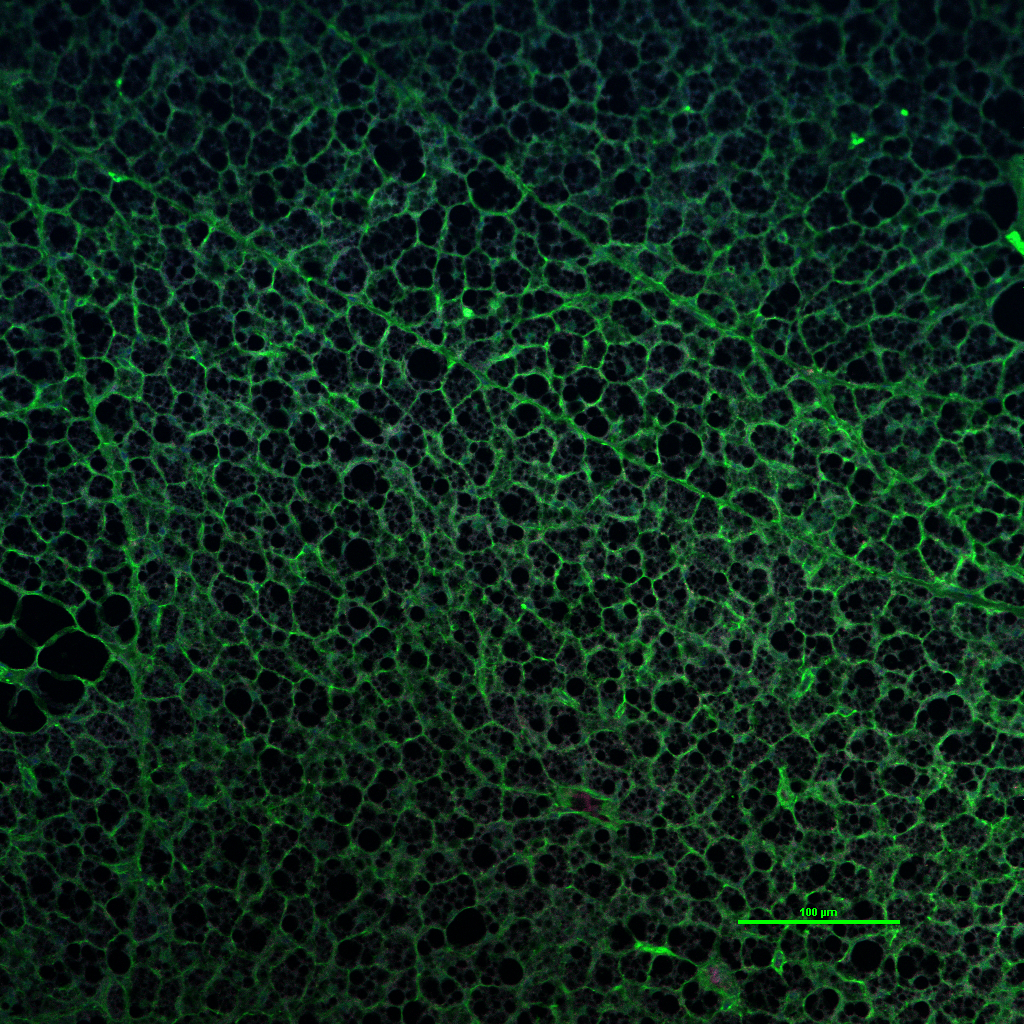

Supplement: Supplementary file 3 — Source data Fig. 1 [file 44318_2024_196_MOESM3_ESM.zip › Figure 1/Figure 1-D/Quantificated image/NC/no.4/NC BAT_no.4_RGB_Merge-1.tif]

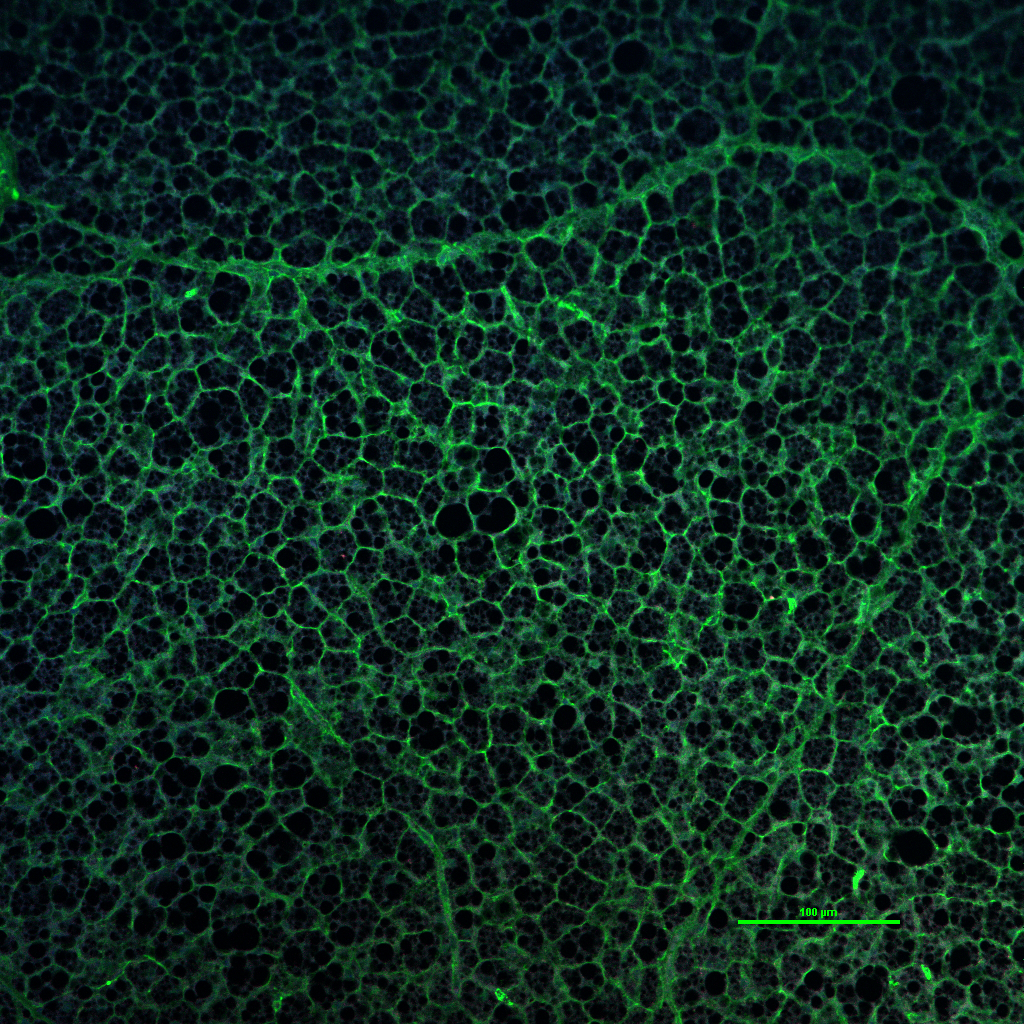

Supplement: Supplementary file 3 — Source data Fig. 1 [file 44318_2024_196_MOESM3_ESM.zip › Figure 1/Figure 1-D/Quantificated image/NC/no.4/NC BAT_no.4_RGB_Merge-2.tif]

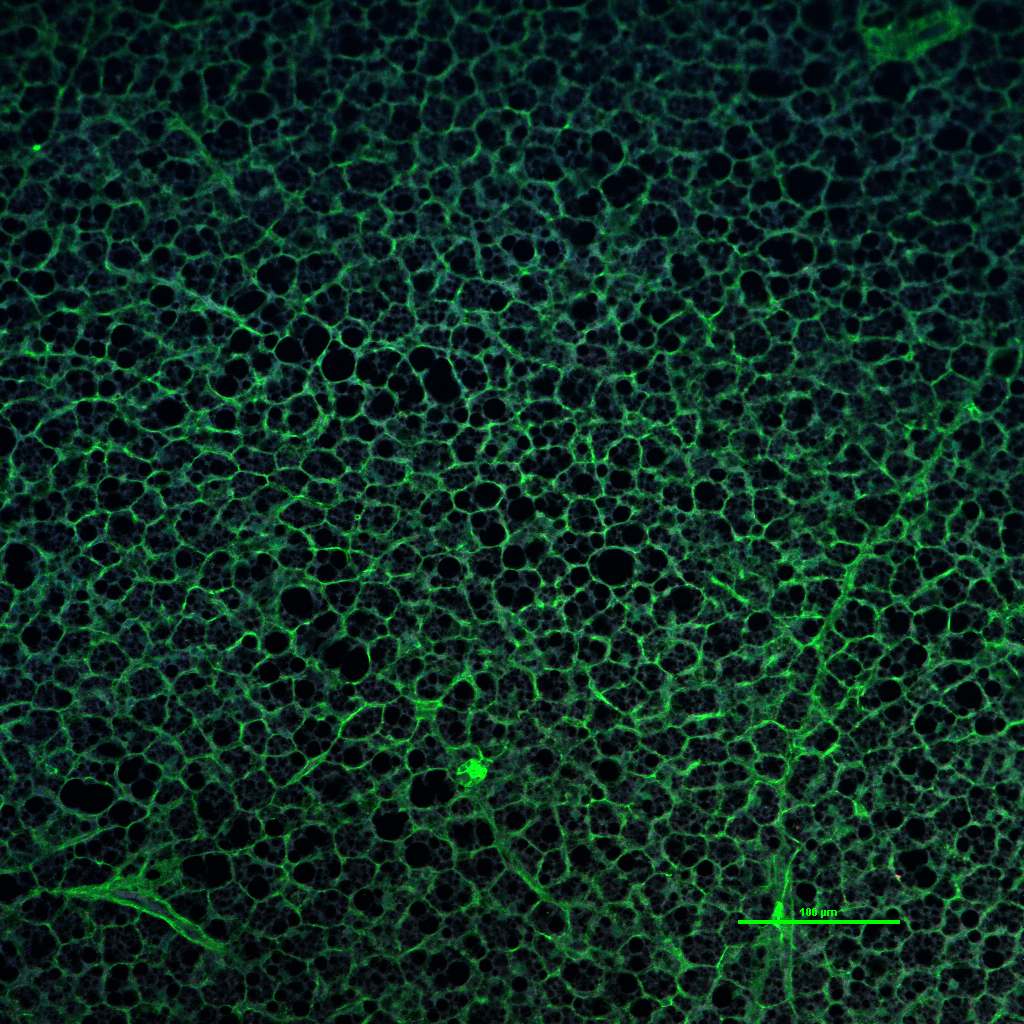

Supplement: Supplementary file 3 — Source data Fig. 1 [file 44318_2024_196_MOESM3_ESM.zip › Figure 1/Figure 1-D/Quantificated image/NC/no.4/NC BAT_no.4_RGB_Merge-3.tif]

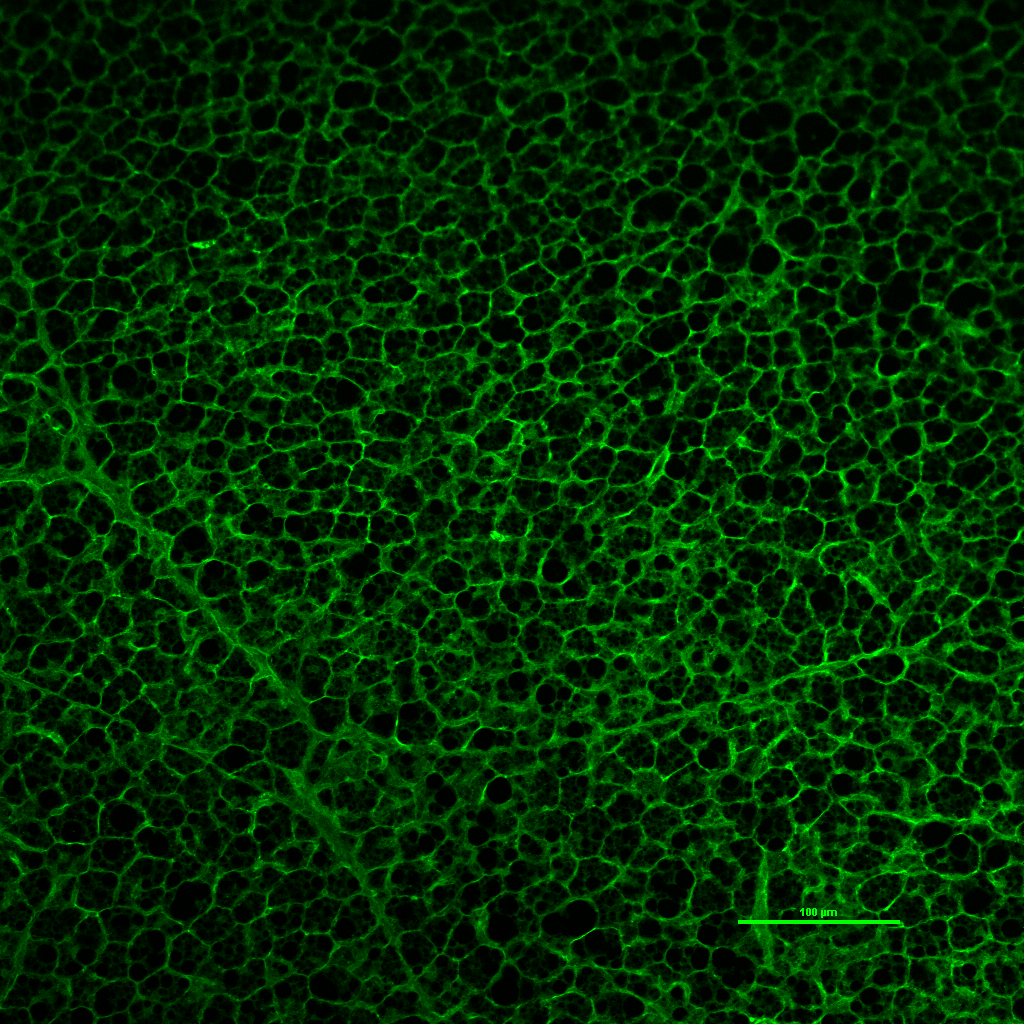

Supplement: Supplementary file 3 — Source data Fig. 1 [file 44318_2024_196_MOESM3_ESM.zip › Figure 1/Figure 1-D/Quantificated image/NC/no.4/NC BAT_no.4_RGB_WGA lectin_FITC-4.tif]

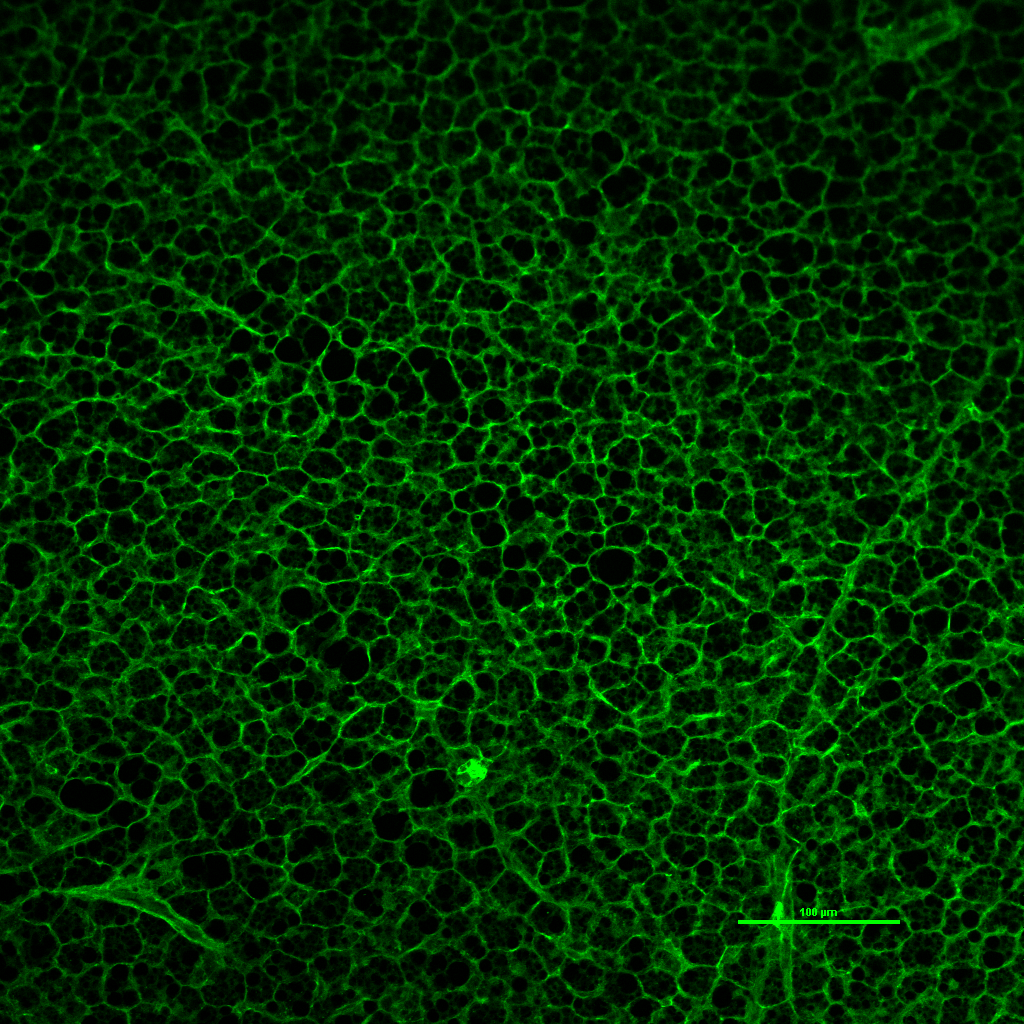

Supplement: Supplementary file 3 — Source data Fig. 1 [file 44318_2024_196_MOESM3_ESM.zip › Figure 1/Figure 1-D/Quantificated image/NC/no.4/NC BAT_no.4_RGB_WGA lectin_FITC-3.tif]

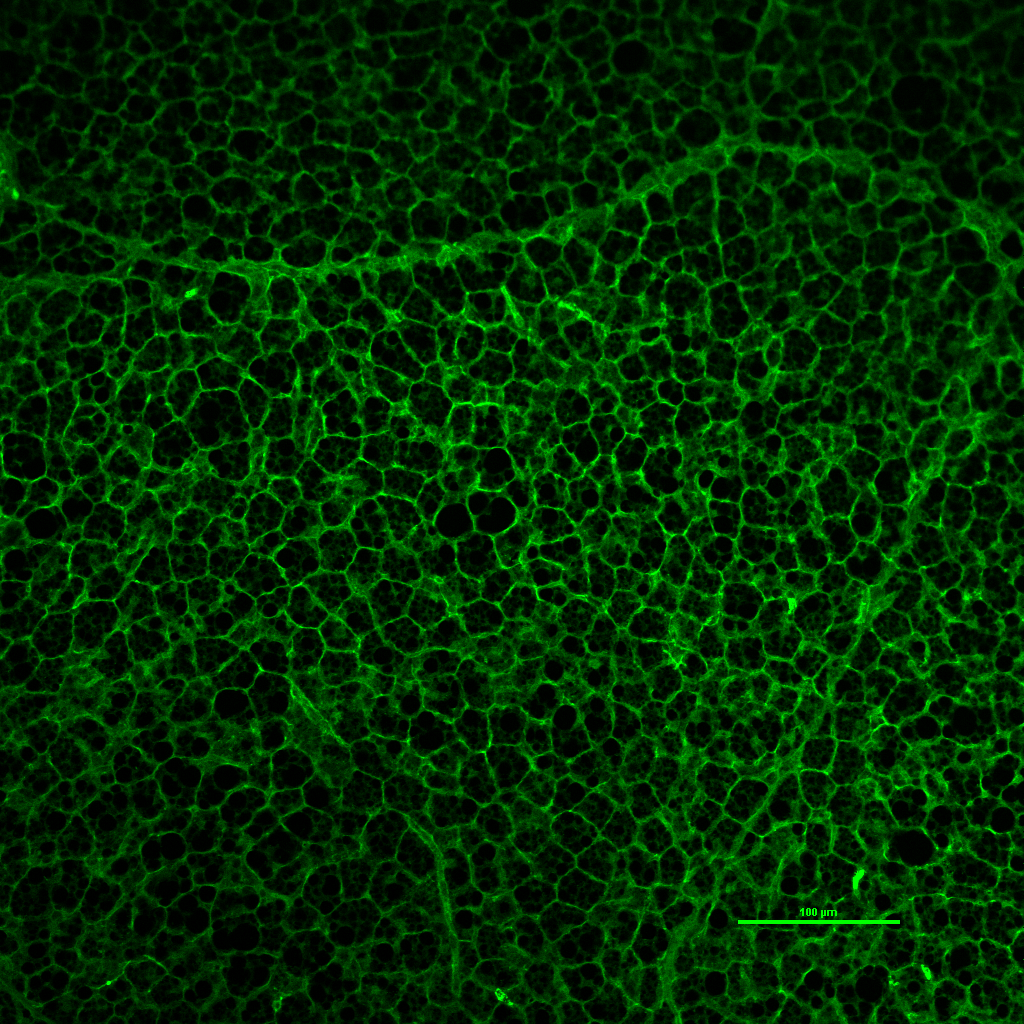

Supplement: Supplementary file 3 — Source data Fig. 1 [file 44318_2024_196_MOESM3_ESM.zip › Figure 1/Figure 1-D/Quantificated image/NC/no.4/NC BAT_no.4_RGB_WGA lectin_FITC-2.tif]

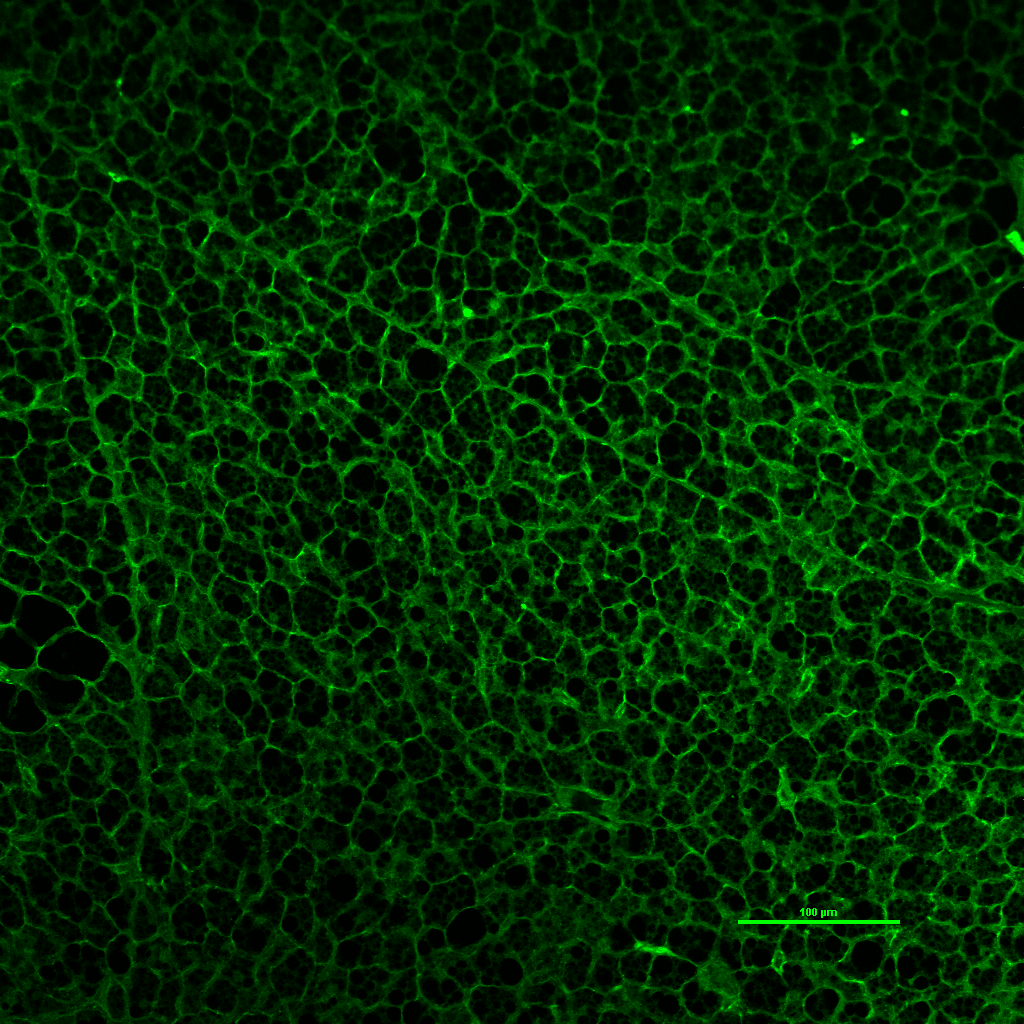

Supplement: Supplementary file 3 — Source data Fig. 1 [file 44318_2024_196_MOESM3_ESM.zip › Figure 1/Figure 1-D/Quantificated image/NC/no.4/NC BAT_no.4_RGB_WGA lectin_FITC-1.tif]

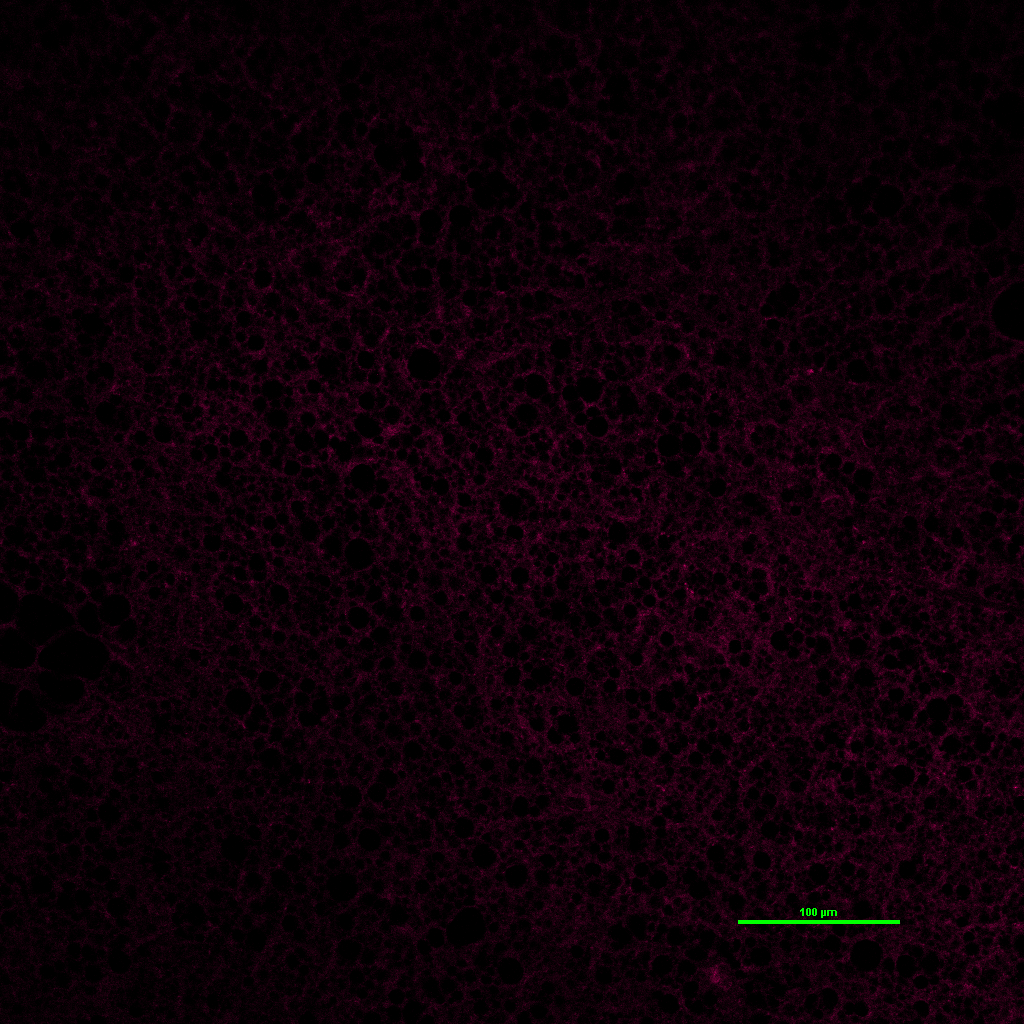

Supplement: Supplementary file 3 — Source data Fig. 1 [file 44318_2024_196_MOESM3_ESM.zip › Figure 1/Figure 1-D/Quantificated image/NC/no.4/NC BAT_no.4_RGB_PCPE-1_Cy5-1.tif]

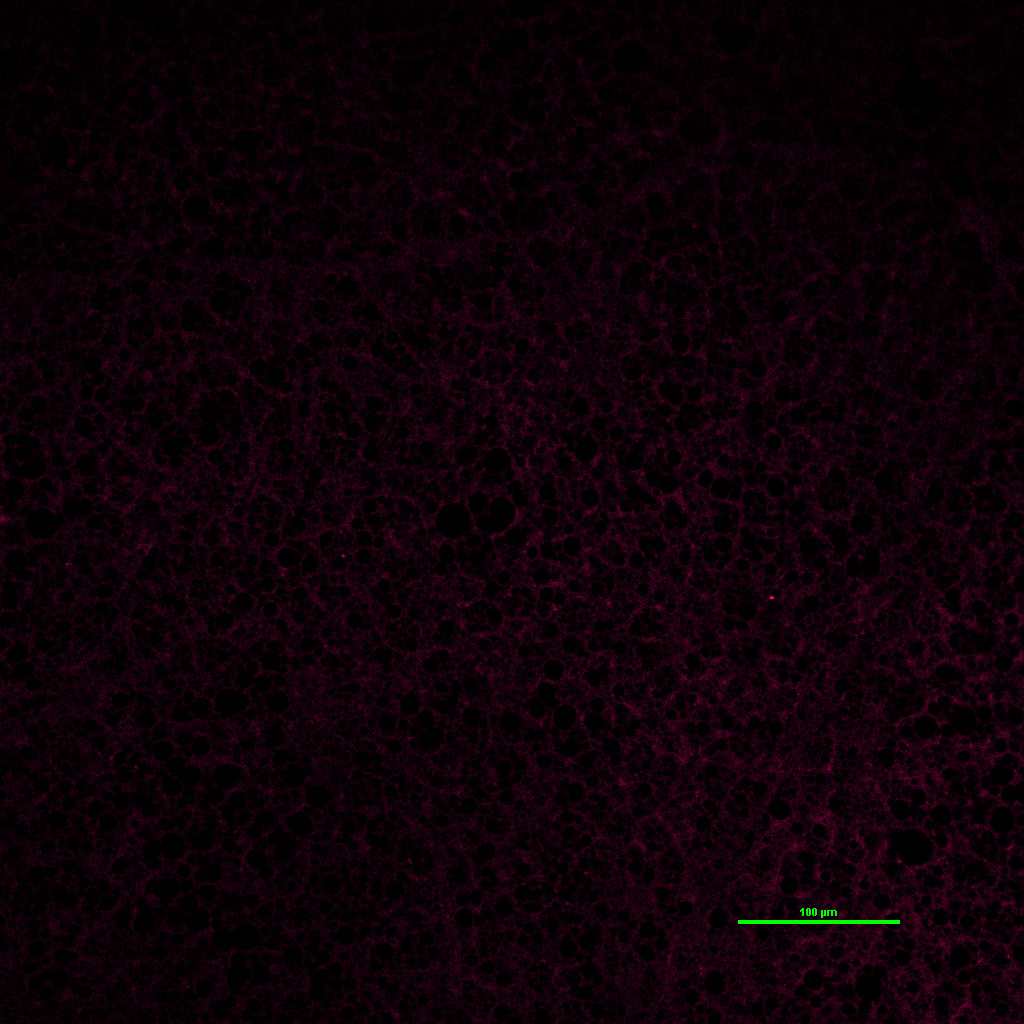

Supplement: Supplementary file 3 — Source data Fig. 1 [file 44318_2024_196_MOESM3_ESM.zip › Figure 1/Figure 1-D/Quantificated image/NC/no.4/NC BAT_no.4_RGB_PCPE-1_Cy5-2.tif]

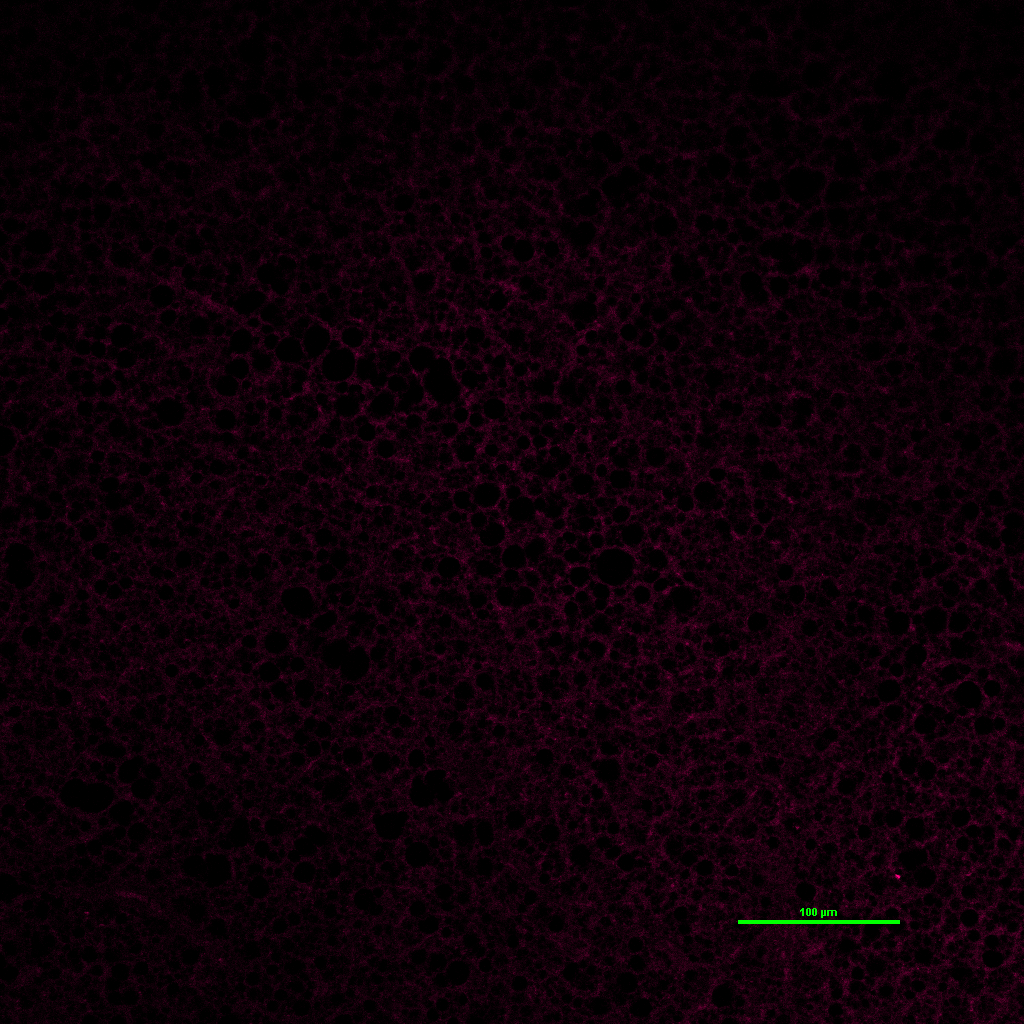

Supplement: Supplementary file 3 — Source data Fig. 1 [file 44318_2024_196_MOESM3_ESM.zip › Figure 1/Figure 1-D/Quantificated image/NC/no.4/NC BAT_no.4_RGB_PCPE-1_Cy5-3.tif]

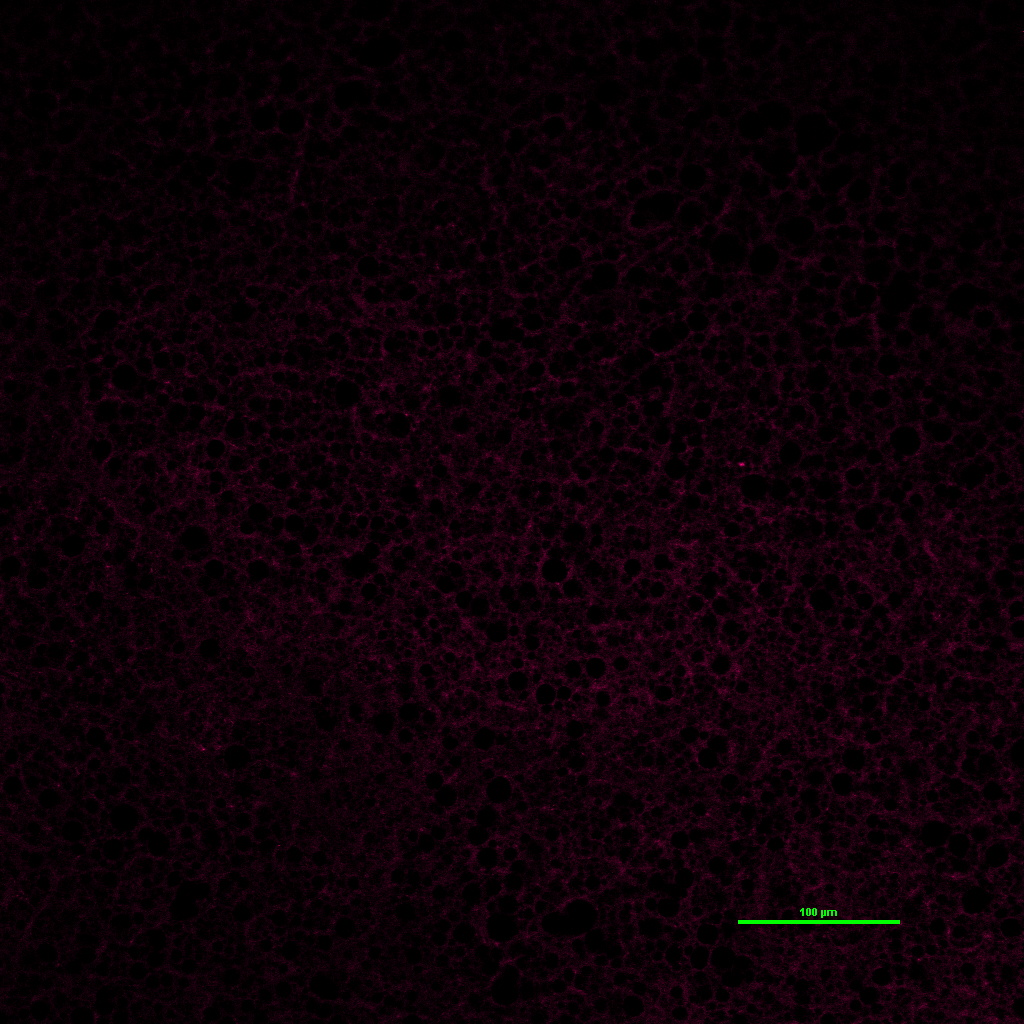

Supplement: Supplementary file 3 — Source data Fig. 1 [file 44318_2024_196_MOESM3_ESM.zip › Figure 1/Figure 1-D/Quantificated image/NC/no.4/NC BAT_no.4_RGB_PCPE-1_Cy5-4.tif]

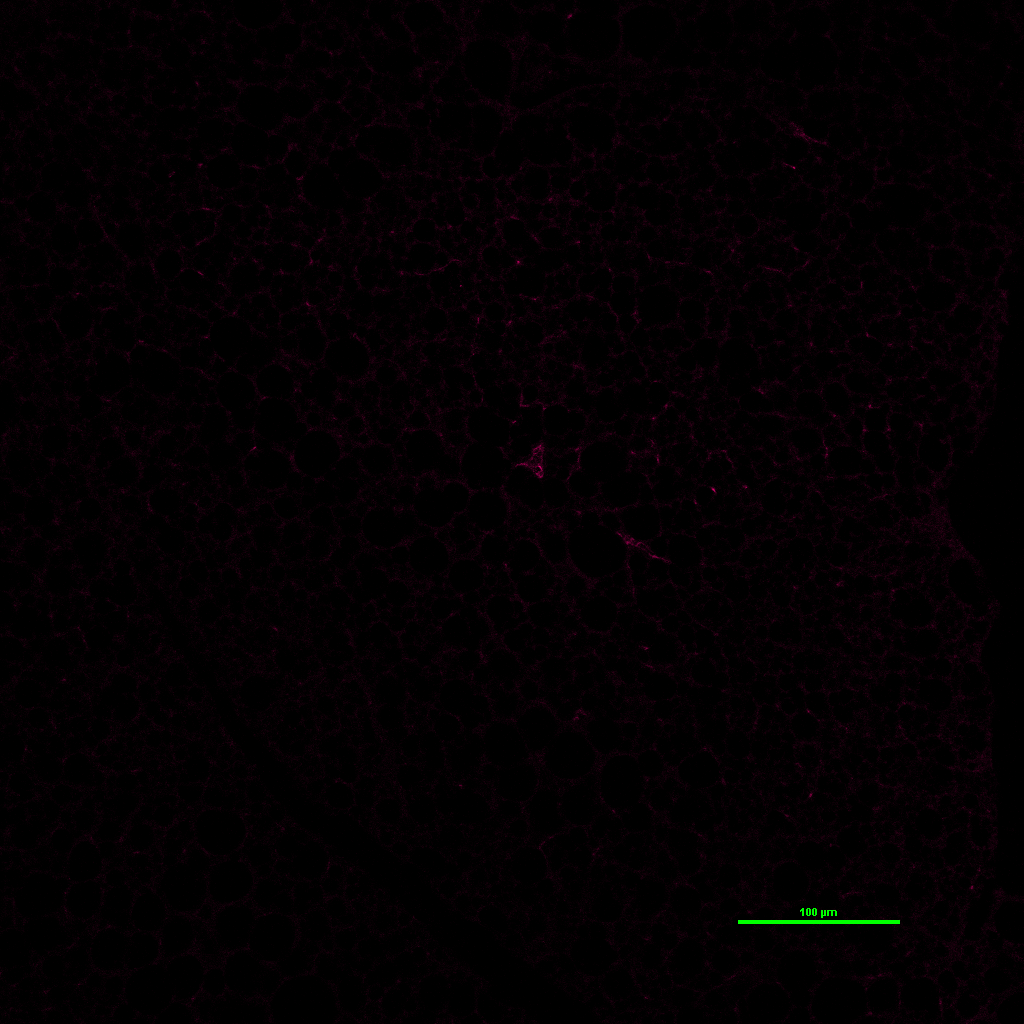

Supplement: Supplementary file 3 — Source data Fig. 1 [file 44318_2024_196_MOESM3_ESM.zip › Figure 1/Figure 1-D/Quantificated image/NC/no.2/NC BAT_no.2_RGB_PCPE-1_Cy5-4.tif]

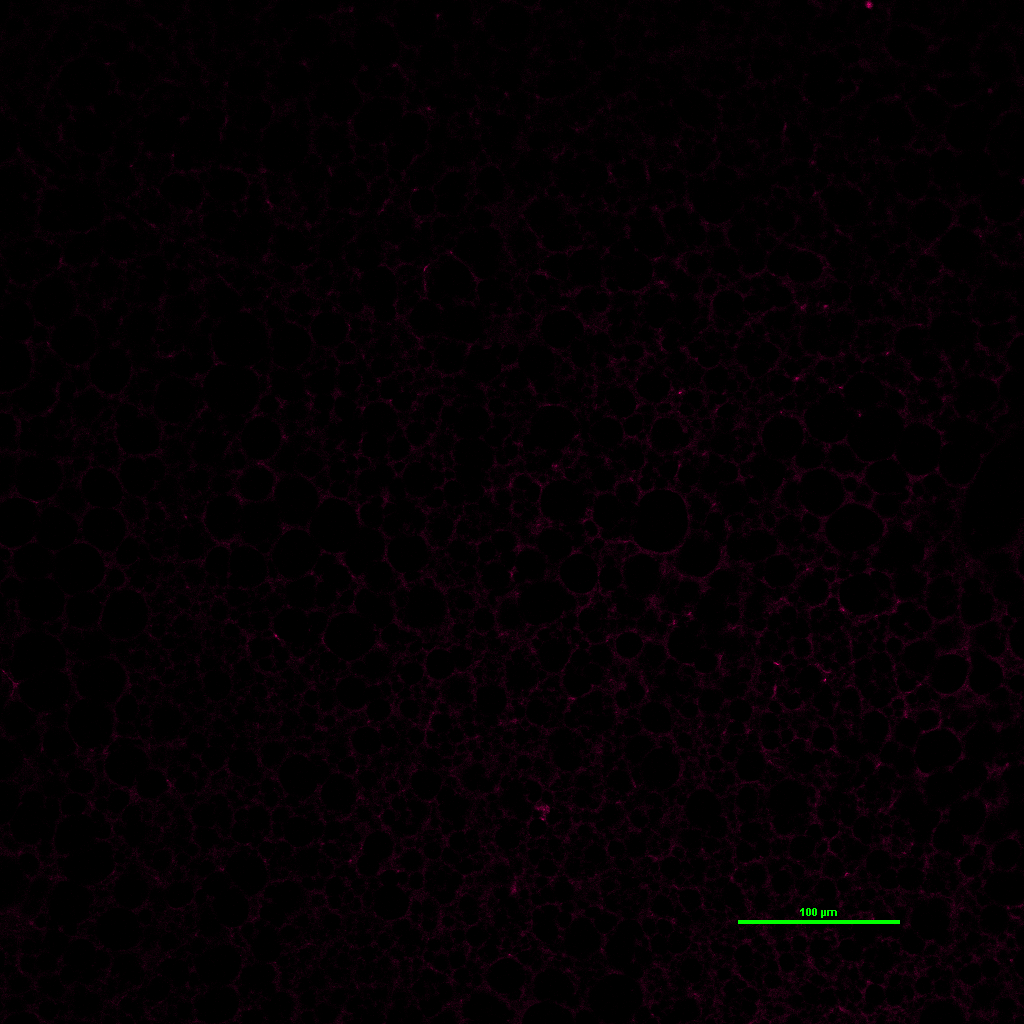

Supplement: Supplementary file 3 — Source data Fig. 1 [file 44318_2024_196_MOESM3_ESM.zip › Figure 1/Figure 1-D/Quantificated image/NC/no.2/NC BAT_no.2_RGB_PCPE-1_Cy5-2.tif]

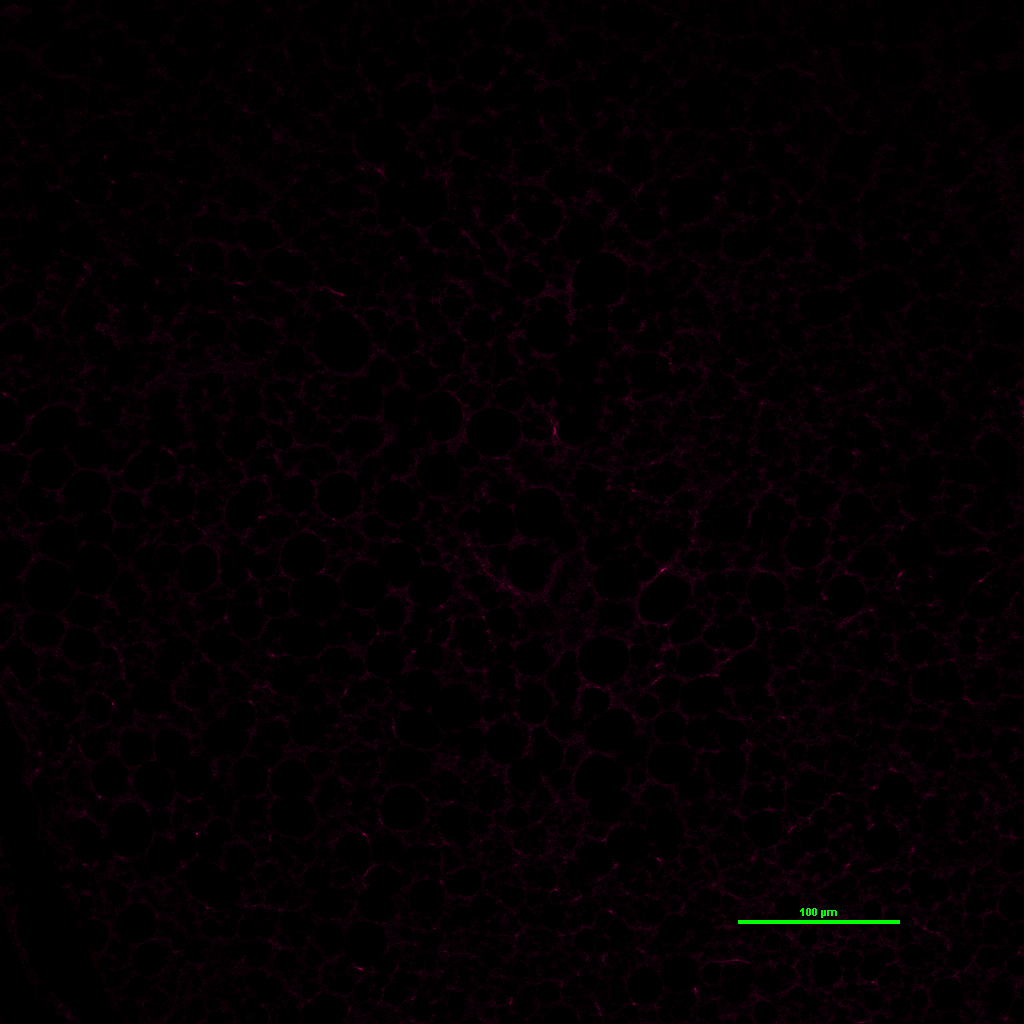

Supplement: Supplementary file 3 — Source data Fig. 1 [file 44318_2024_196_MOESM3_ESM.zip › Figure 1/Figure 1-D/Quantificated image/NC/no.2/NC BAT_no.2_RGB_PCPE-1_Cy5-3.tif]

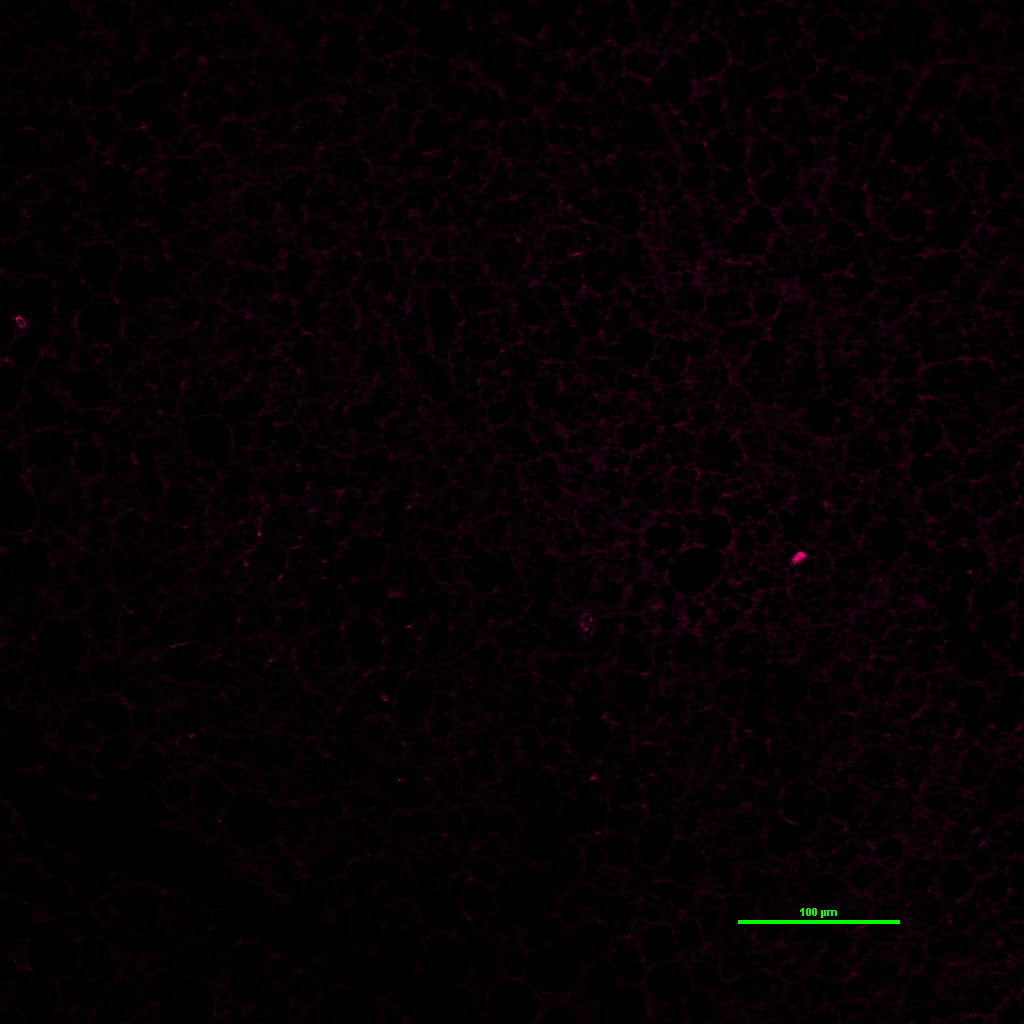

Supplement: Supplementary file 3 — Source data Fig. 1 [file 44318_2024_196_MOESM3_ESM.zip › Figure 1/Figure 1-D/Quantificated image/NC/no.2/NC BAT_no.2_RGB_PCPE-1_Cy5-1.tif]

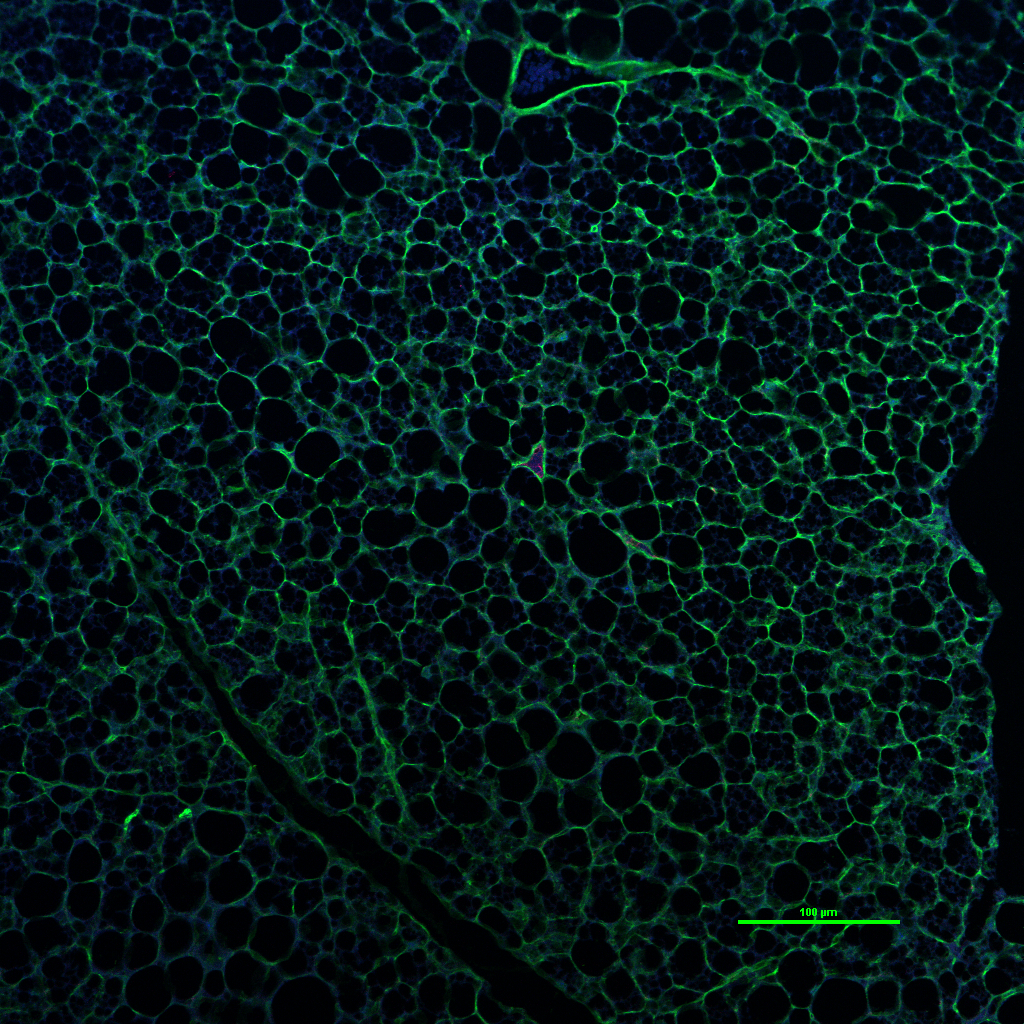

Supplement: Supplementary file 3 — Source data Fig. 1 [file 44318_2024_196_MOESM3_ESM.zip › Figure 1/Figure 1-D/Quantificated image/NC/no.2/NC BAT_no.2_RGB_Merge-4.tif]

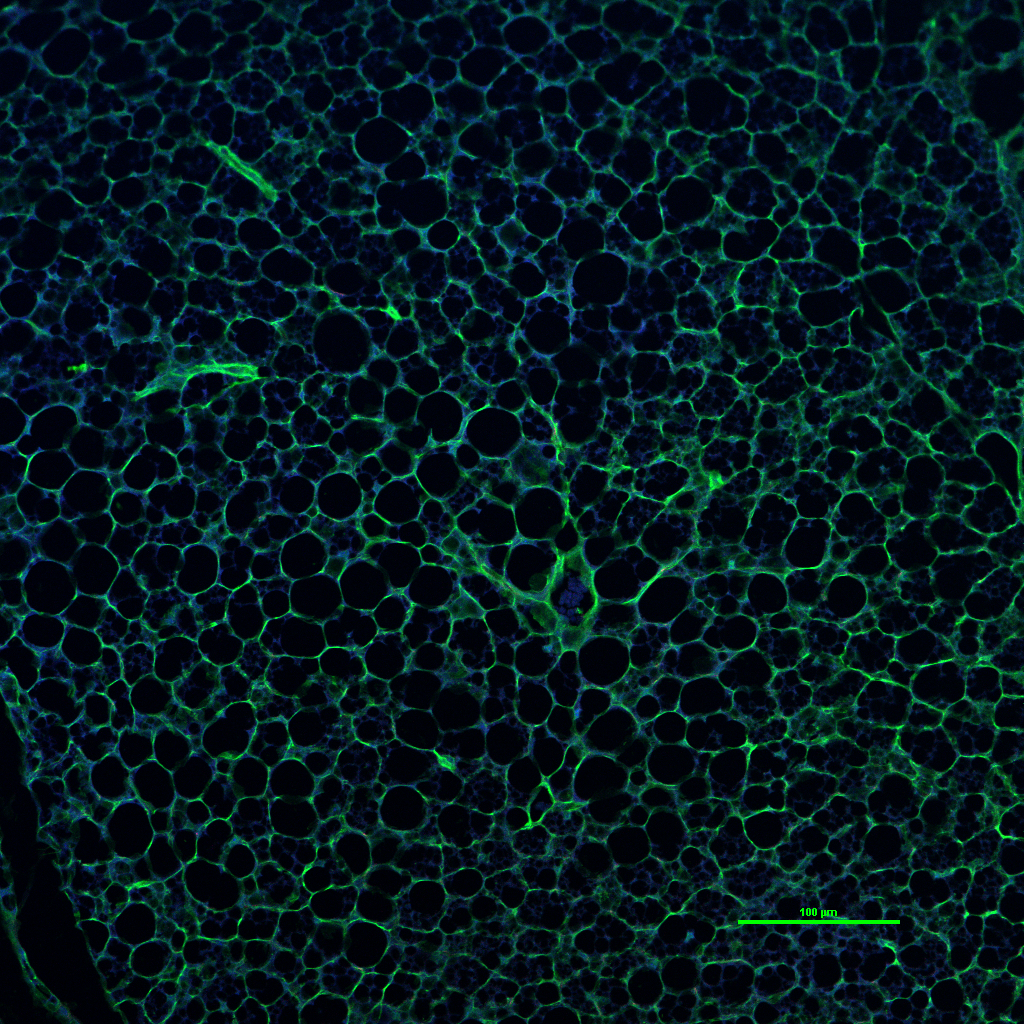

Supplement: Supplementary file 3 — Source data Fig. 1 [file 44318_2024_196_MOESM3_ESM.zip › Figure 1/Figure 1-D/Quantificated image/NC/no.2/NC BAT_no.2_RGB_Merge-3.tif]

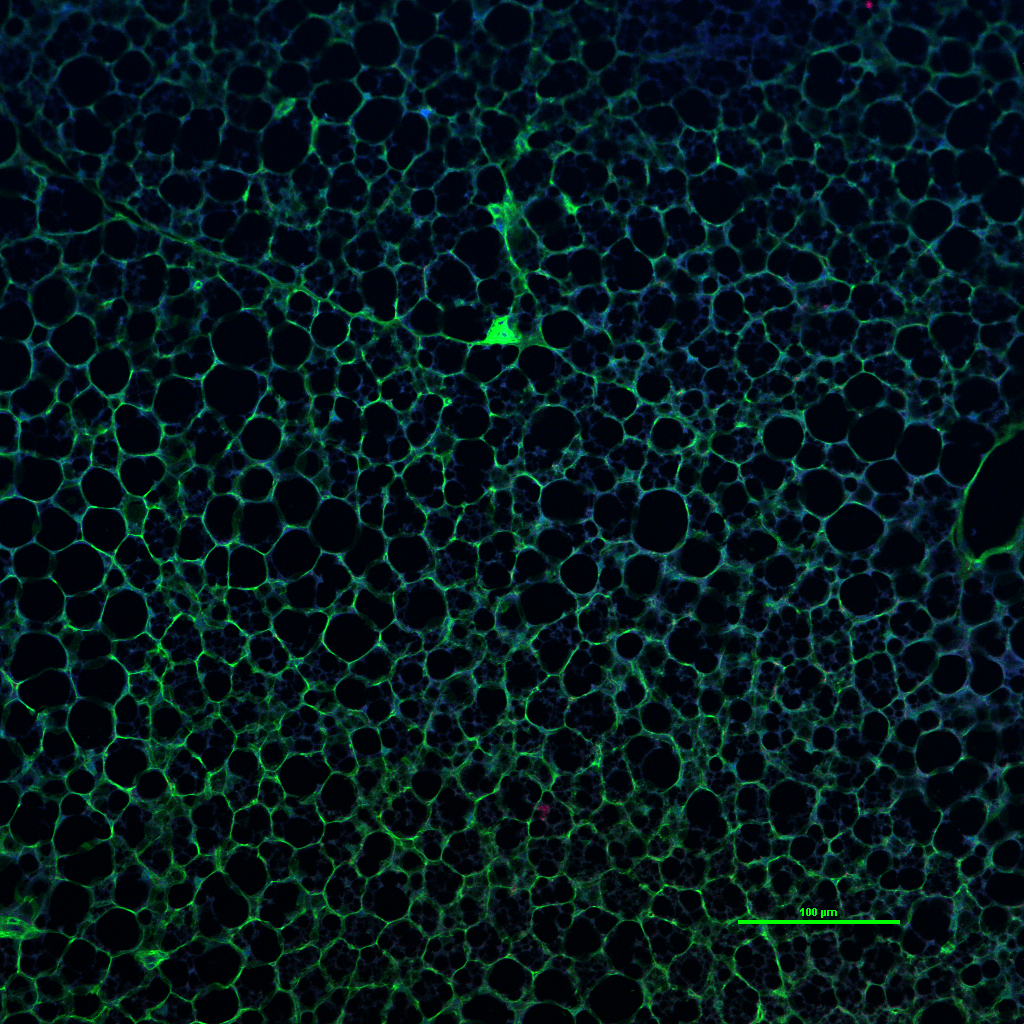

Supplement: Supplementary file 3 — Source data Fig. 1 [file 44318_2024_196_MOESM3_ESM.zip › Figure 1/Figure 1-D/Quantificated image/NC/no.2/NC BAT_no.2_RGB_Merge-2.tif]

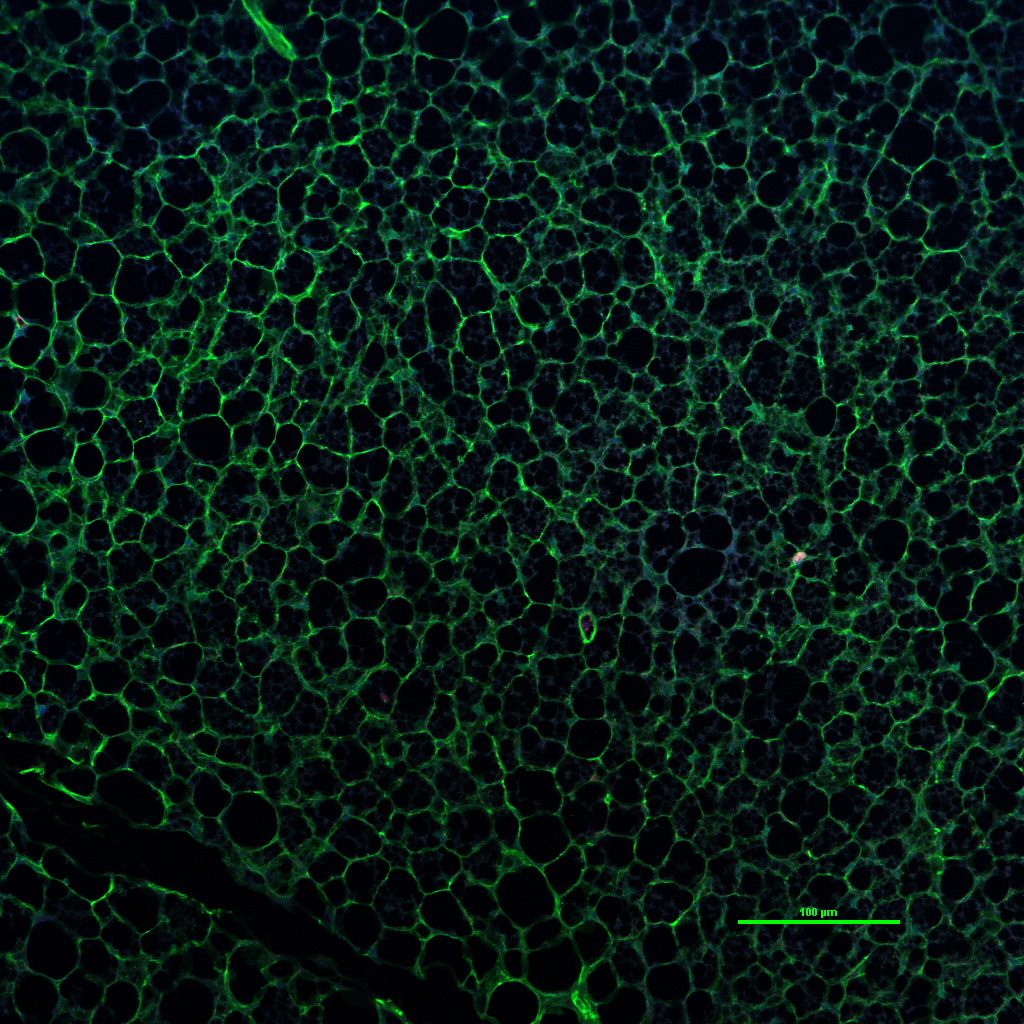

Supplement: Supplementary file 3 — Source data Fig. 1 [file 44318_2024_196_MOESM3_ESM.zip › Figure 1/Figure 1-D/Quantificated image/NC/no.2/NC BAT_no.2_RGB_Merge-1.tif]

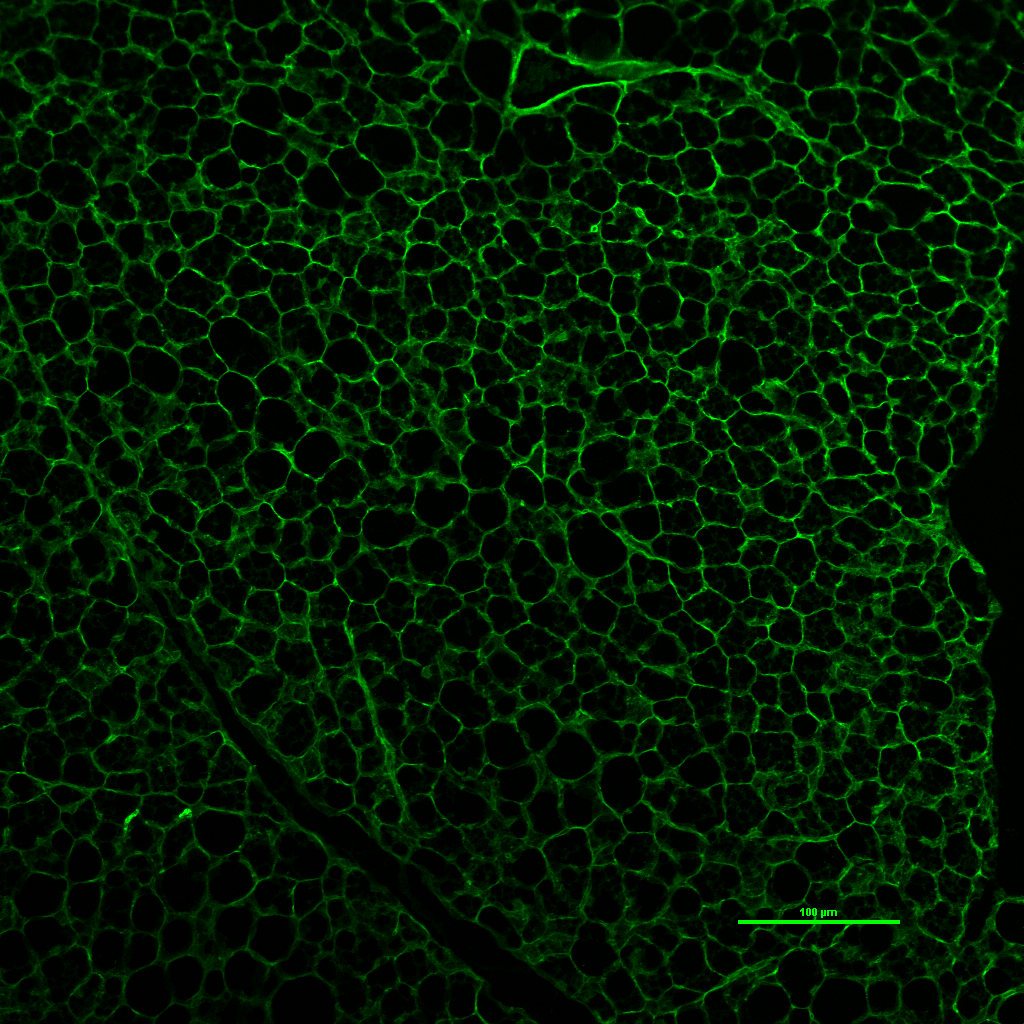

Supplement: Supplementary file 3 — Source data Fig. 1 [file 44318_2024_196_MOESM3_ESM.zip › Figure 1/Figure 1-D/Quantificated image/NC/no.2/NC BAT_no.2_RGB_WGA lectin_FITC-4.tif]

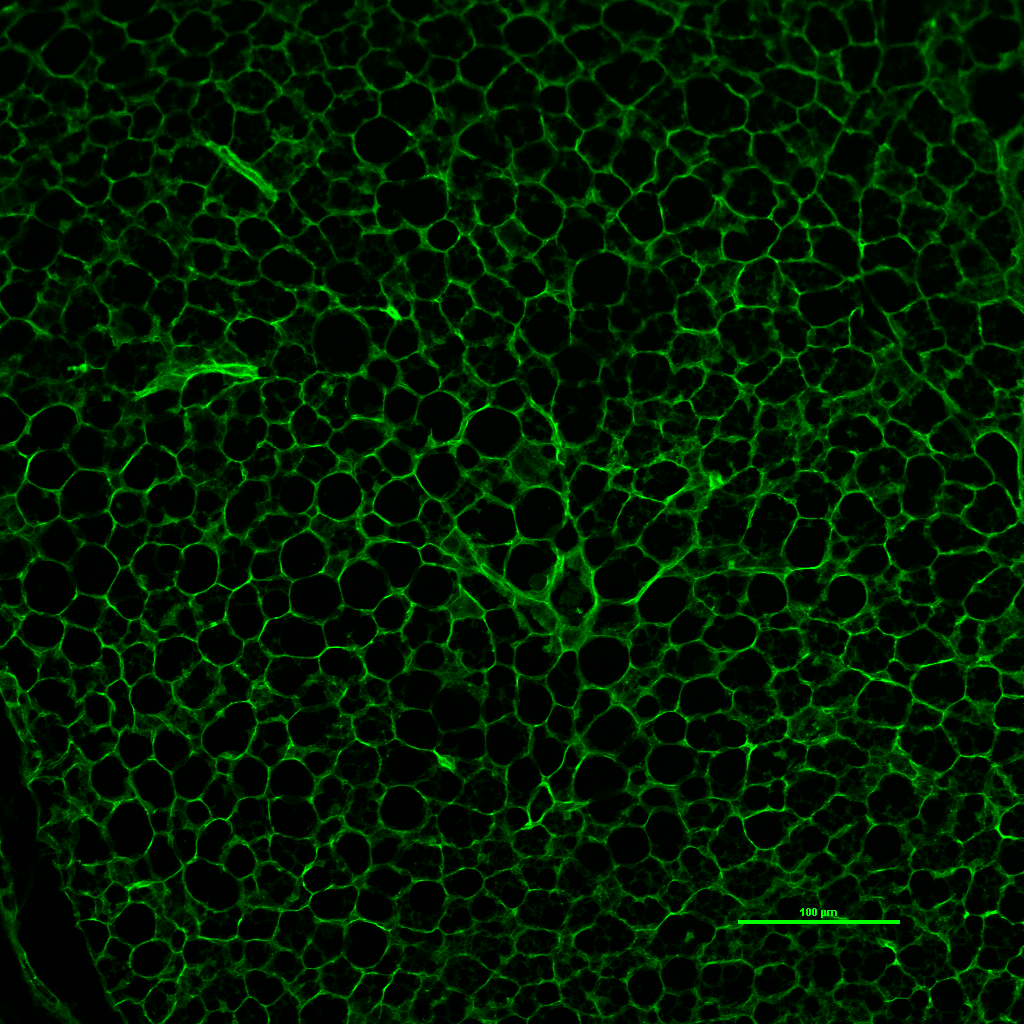

Supplement: Supplementary file 3 — Source data Fig. 1 [file 44318_2024_196_MOESM3_ESM.zip › Figure 1/Figure 1-D/Quantificated image/NC/no.2/NC BAT_no.2_RGB_WGA lectin_FITC-3.tif]

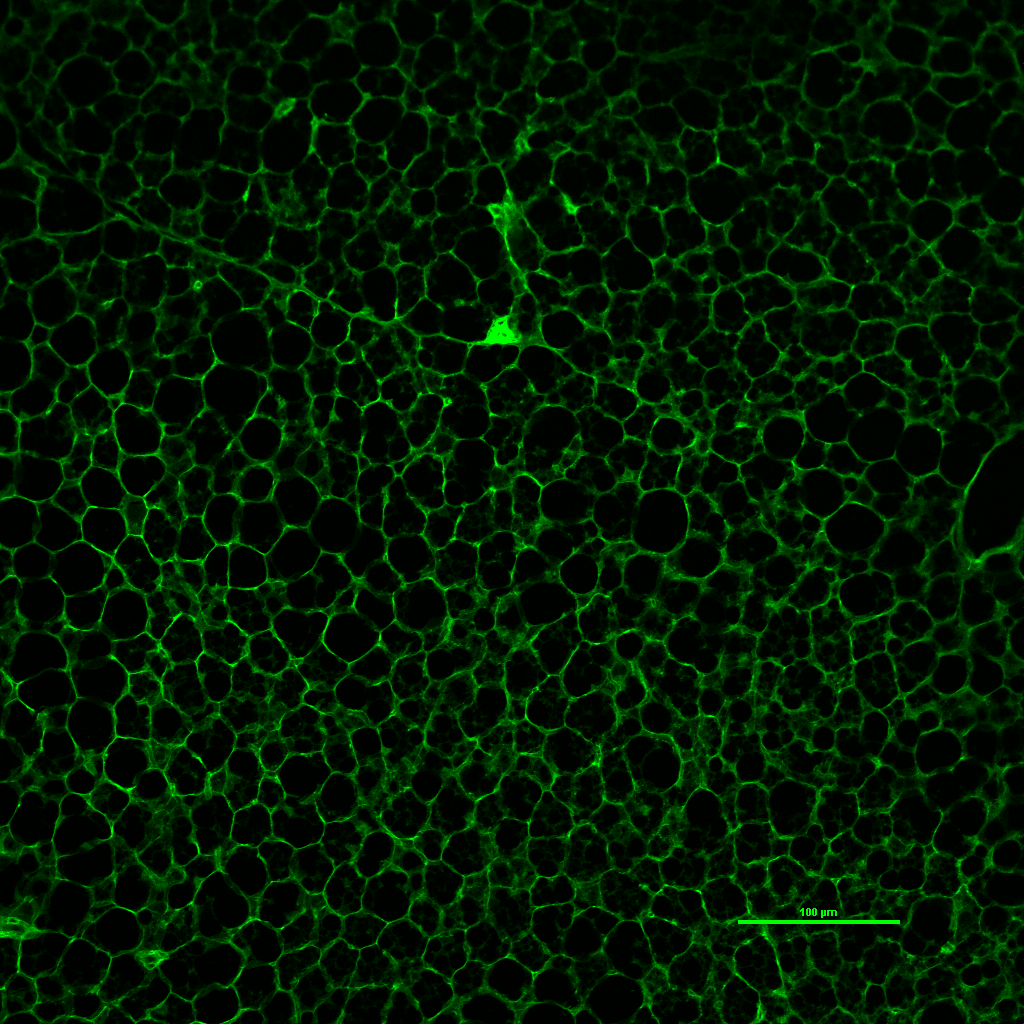

Supplement: Supplementary file 3 — Source data Fig. 1 [file 44318_2024_196_MOESM3_ESM.zip › Figure 1/Figure 1-D/Quantificated image/NC/no.2/NC BAT_no.2_RGB_WGA lectin_FITC-2.tif]

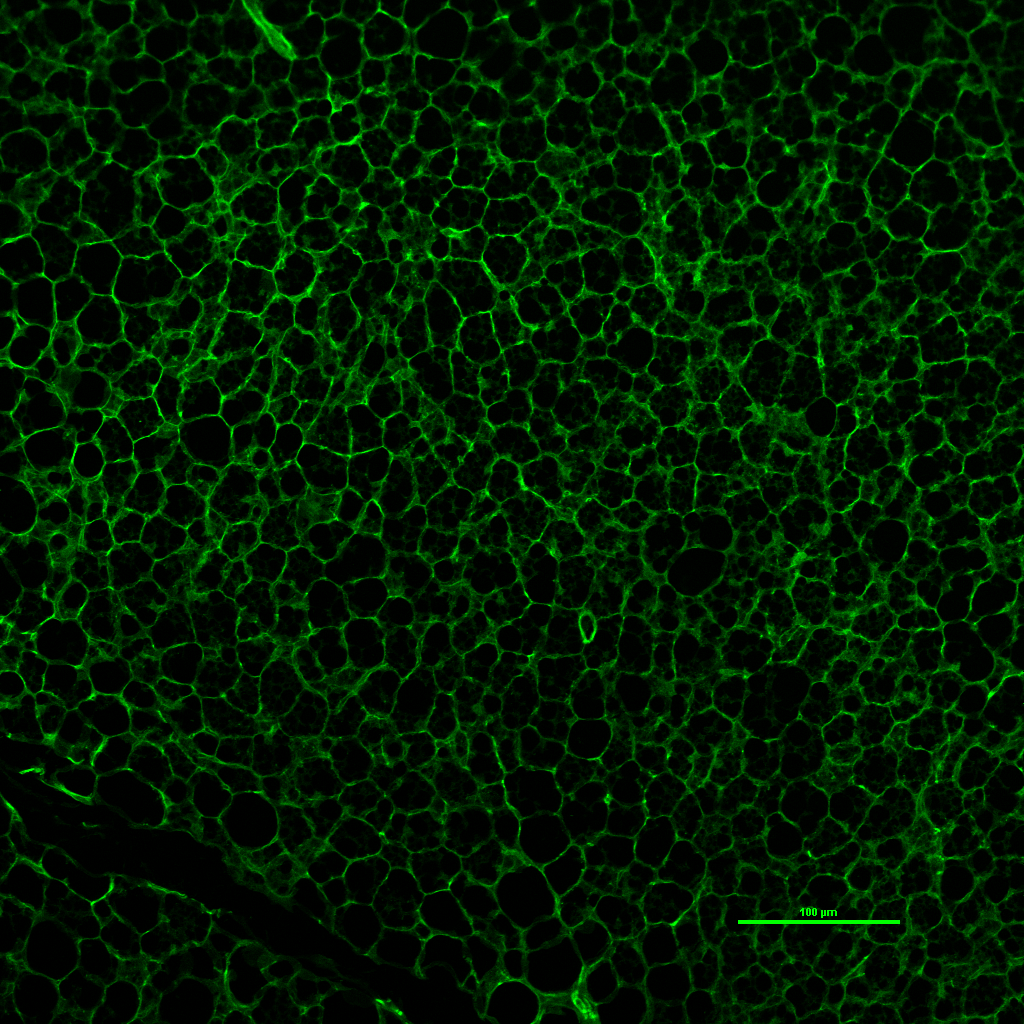

Supplement: Supplementary file 3 — Source data Fig. 1 [file 44318_2024_196_MOESM3_ESM.zip › Figure 1/Figure 1-D/Quantificated image/NC/no.2/NC BAT_no.2_RGB_WGA lectin_FITC-1.tif]

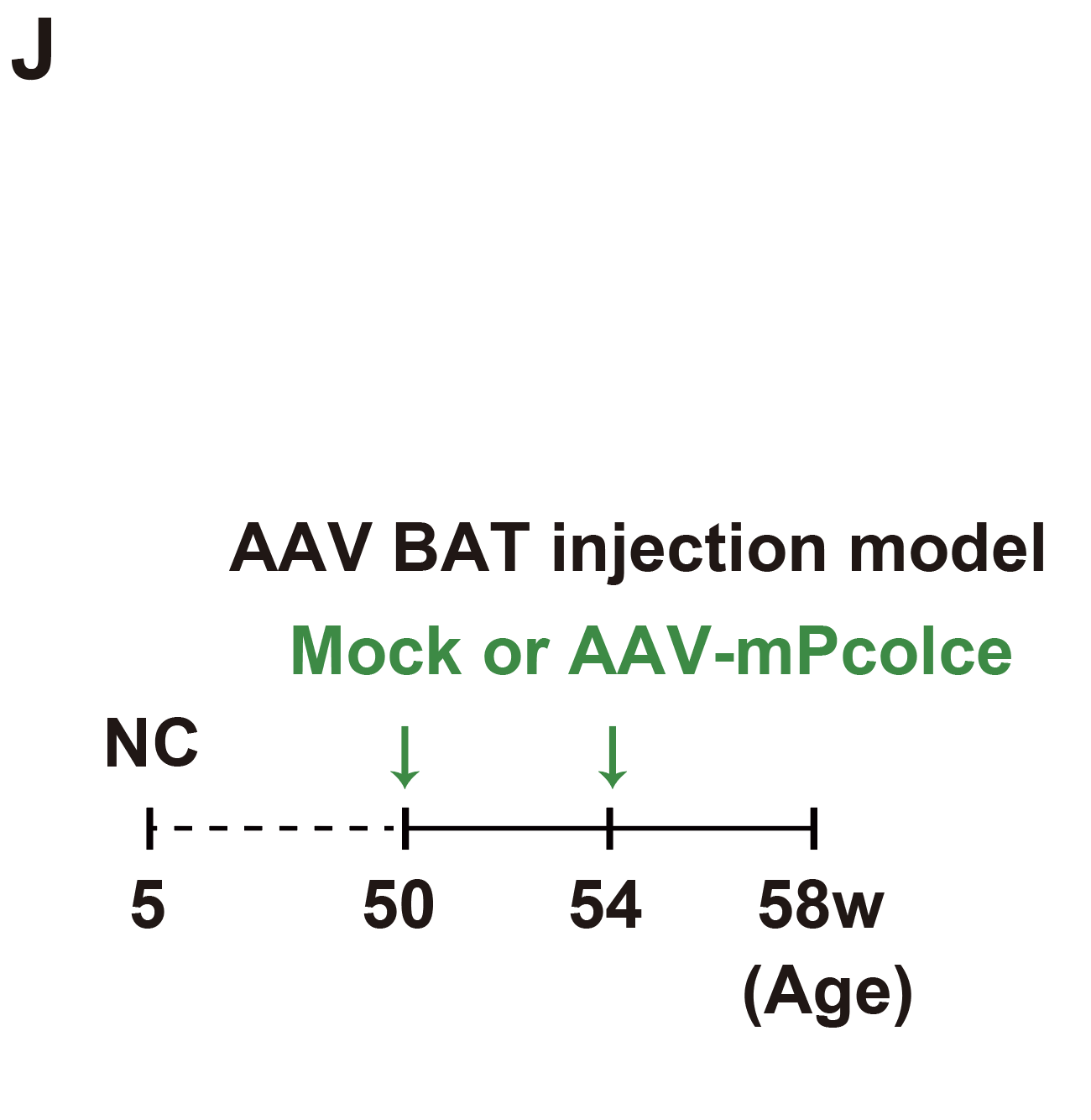

Supplement: Supplementary file 4 — Source data Fig. 2 [file 44318_2024_196_MOESM4_ESM.zip › Figure 2/Figure 2-J/Fig.2J.png]

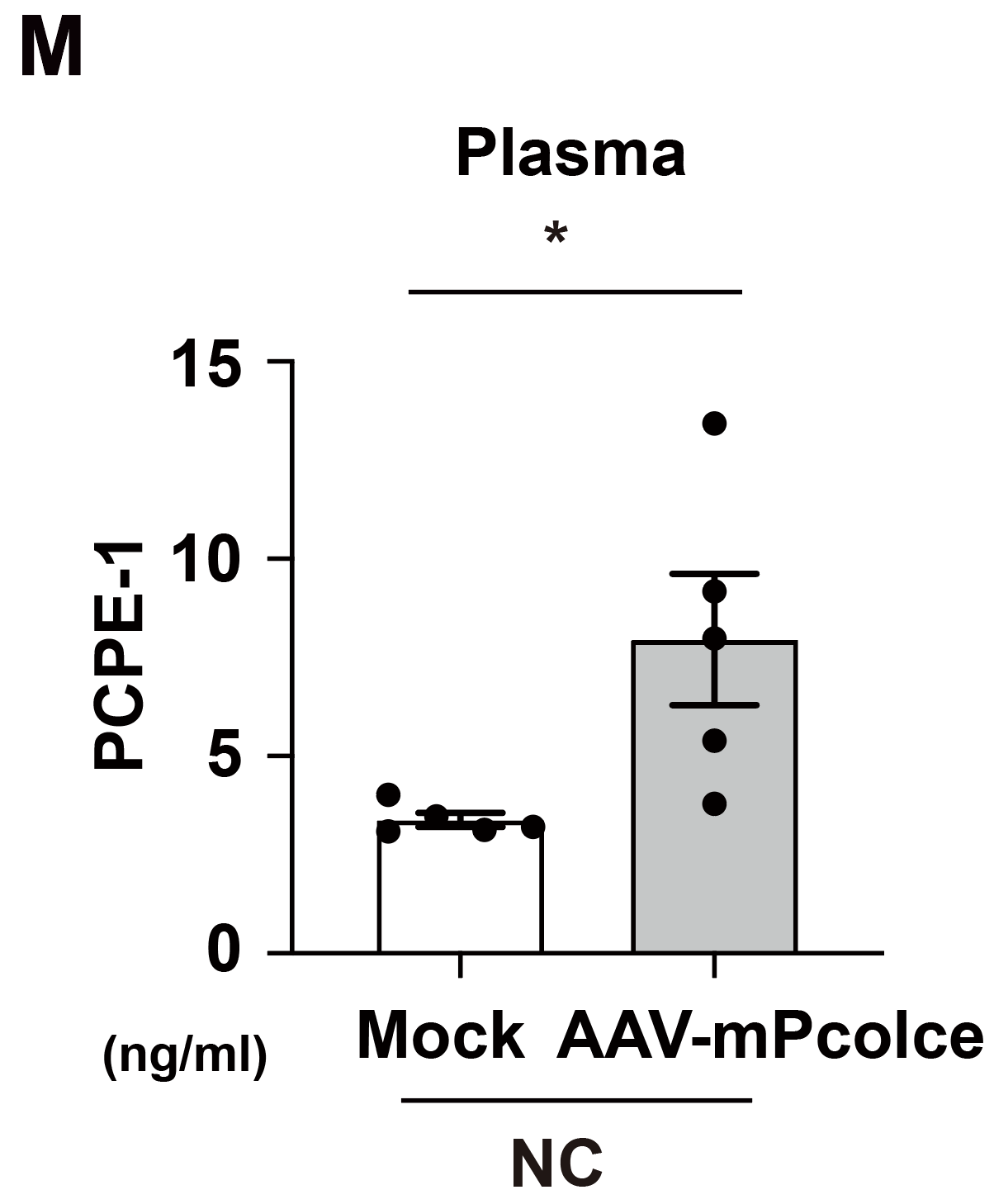

Supplement: Supplementary file 4 — Source data Fig. 2 [file 44318_2024_196_MOESM4_ESM.zip › Figure 2/Figure 2-M/Fig.2M.png]

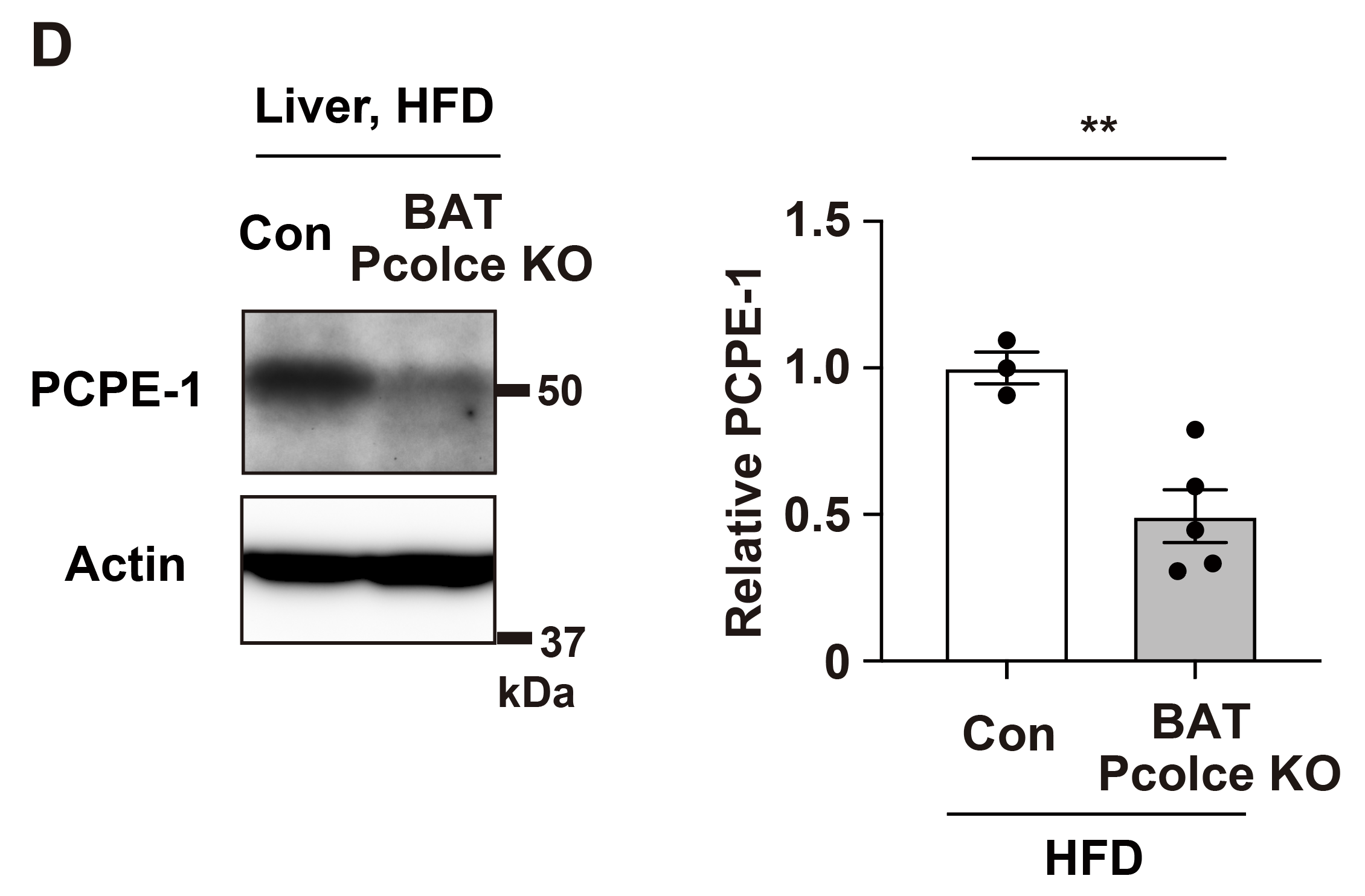

Supplement: Supplementary file 4 — Source data Fig. 2 [file 44318_2024_196_MOESM4_ESM.zip › Figure 2/Figure 2-D/Fig.2D.png]

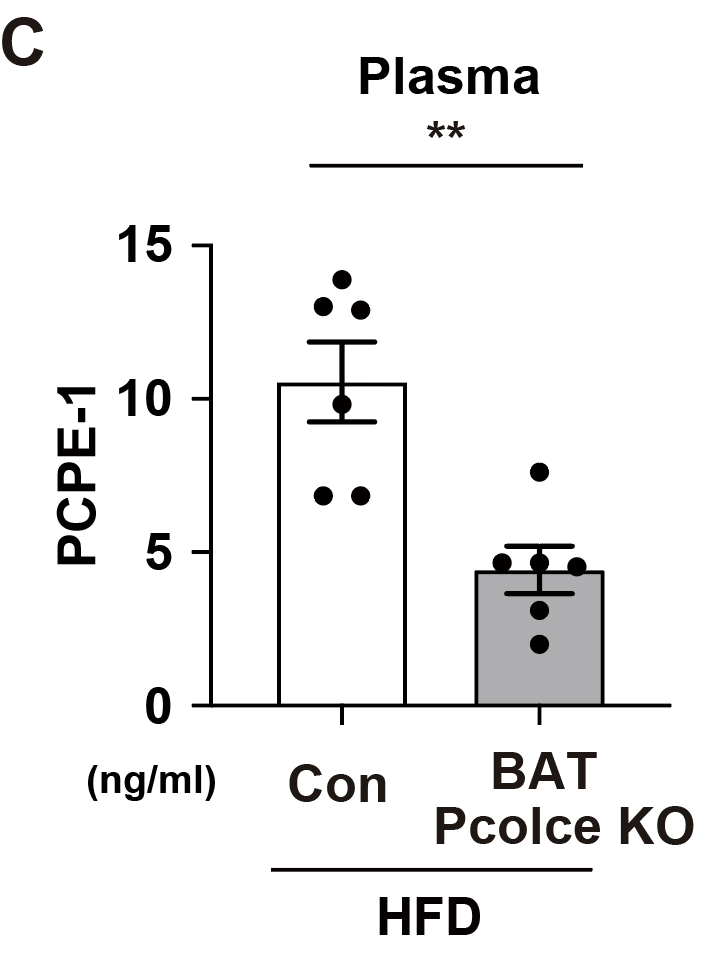

Supplement: Supplementary file 4 — Source data Fig. 2 [file 44318_2024_196_MOESM4_ESM.zip › Figure 2/Figure 2-C/Fig.2C.png]

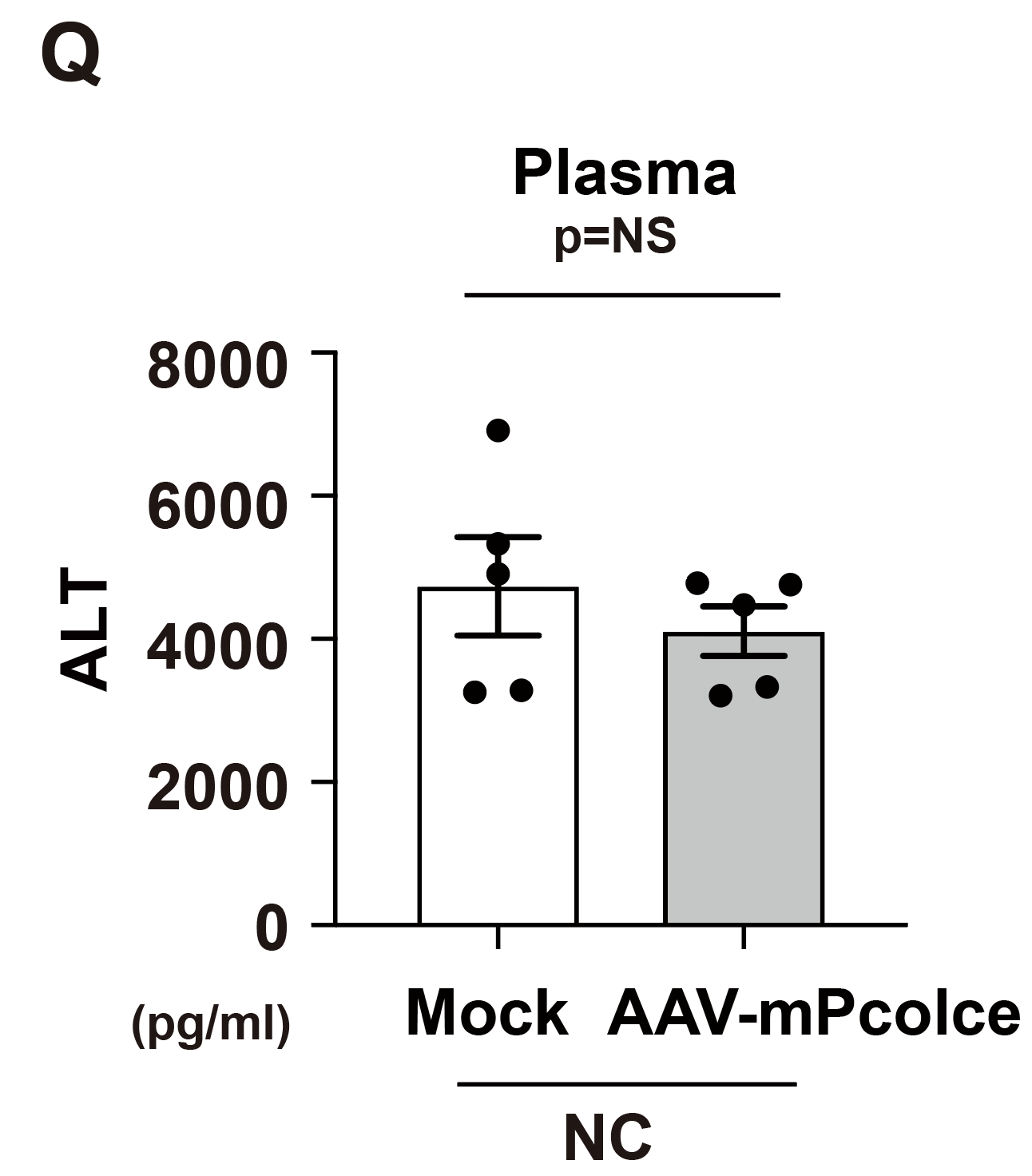

Supplement: Supplementary file 4 — Source data Fig. 2 [file 44318_2024_196_MOESM4_ESM.zip › Figure 2/Figure 2-Q/Fig.2Q.png]

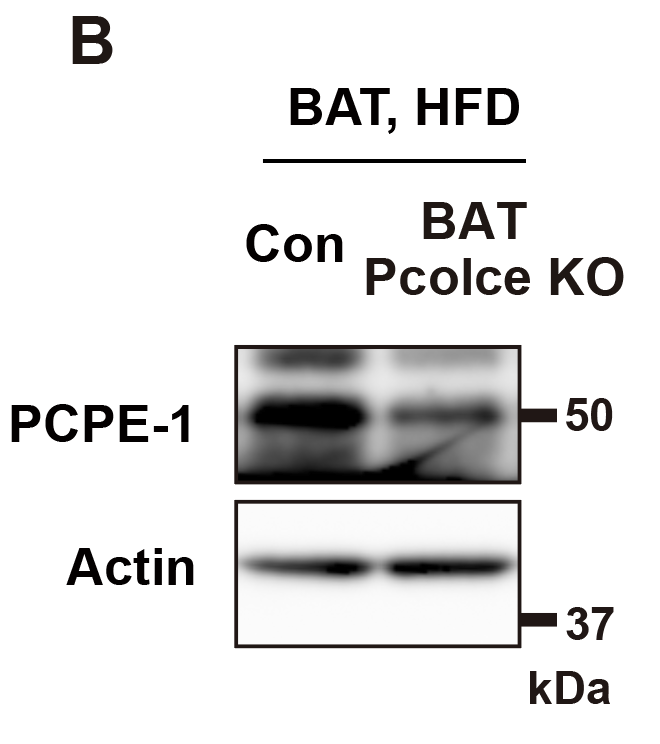

Supplement: Supplementary file 4 — Source data Fig. 2 [file 44318_2024_196_MOESM4_ESM.zip › Figure 2/Figure 2-B/Fig.2B.png]

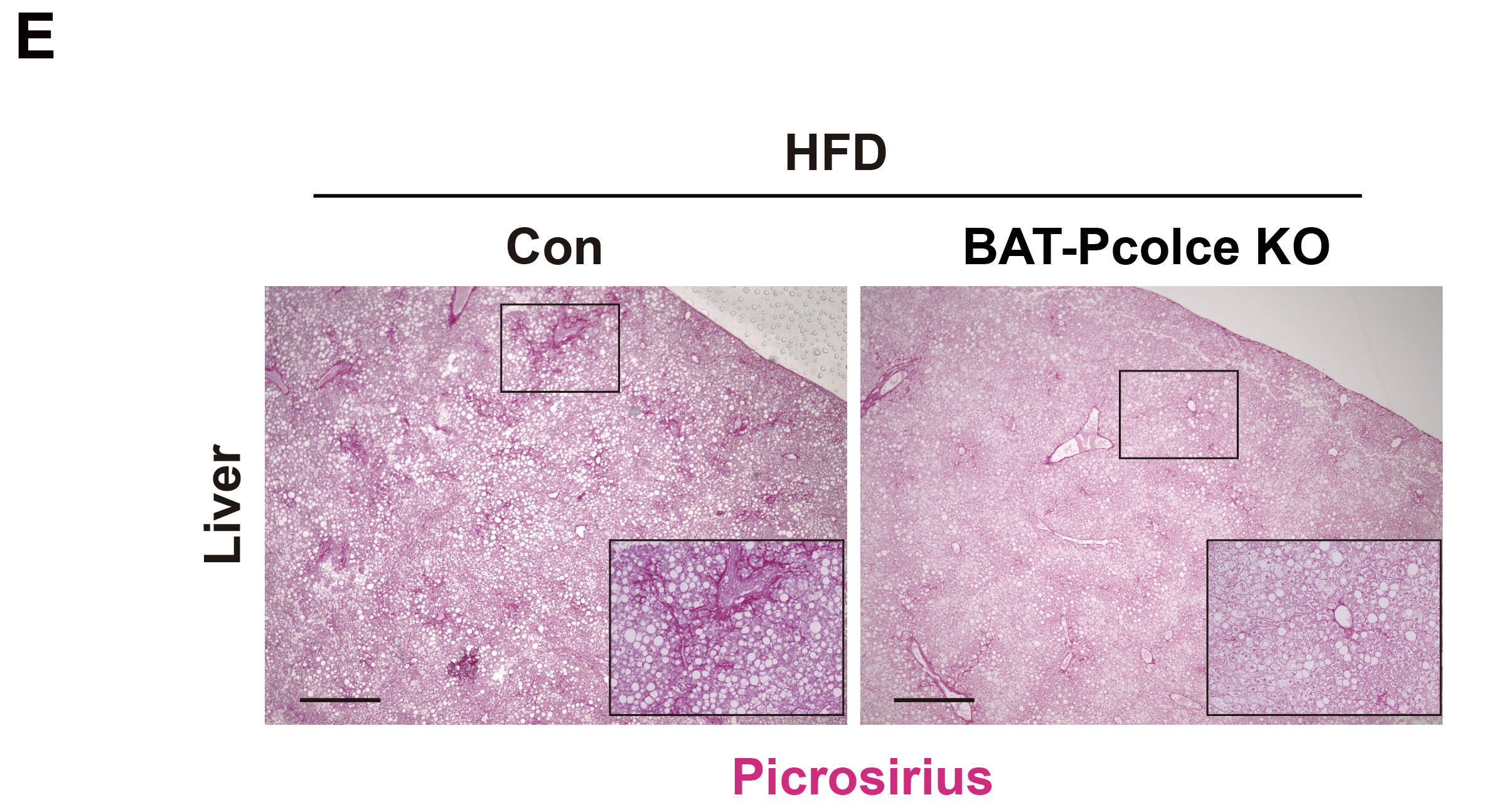

Supplement: Supplementary file 4 — Source data Fig. 2 [file 44318_2024_196_MOESM4_ESM.zip › Figure 2/Figure 2-E/Fig.2E.png]

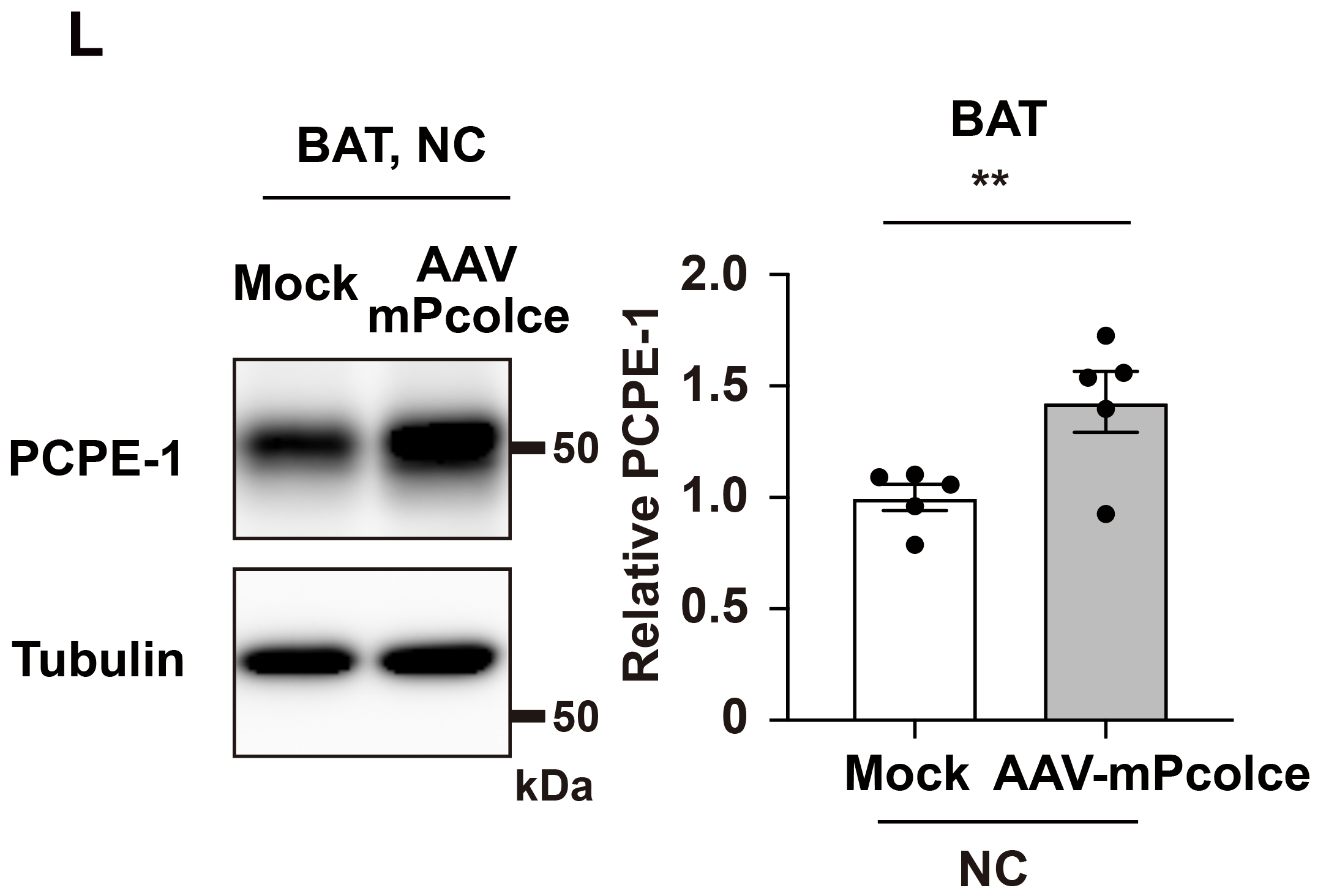

Supplement: Supplementary file 4 — Source data Fig. 2 [file 44318_2024_196_MOESM4_ESM.zip › Figure 2/Figure 2-L/Fig.2L.png]

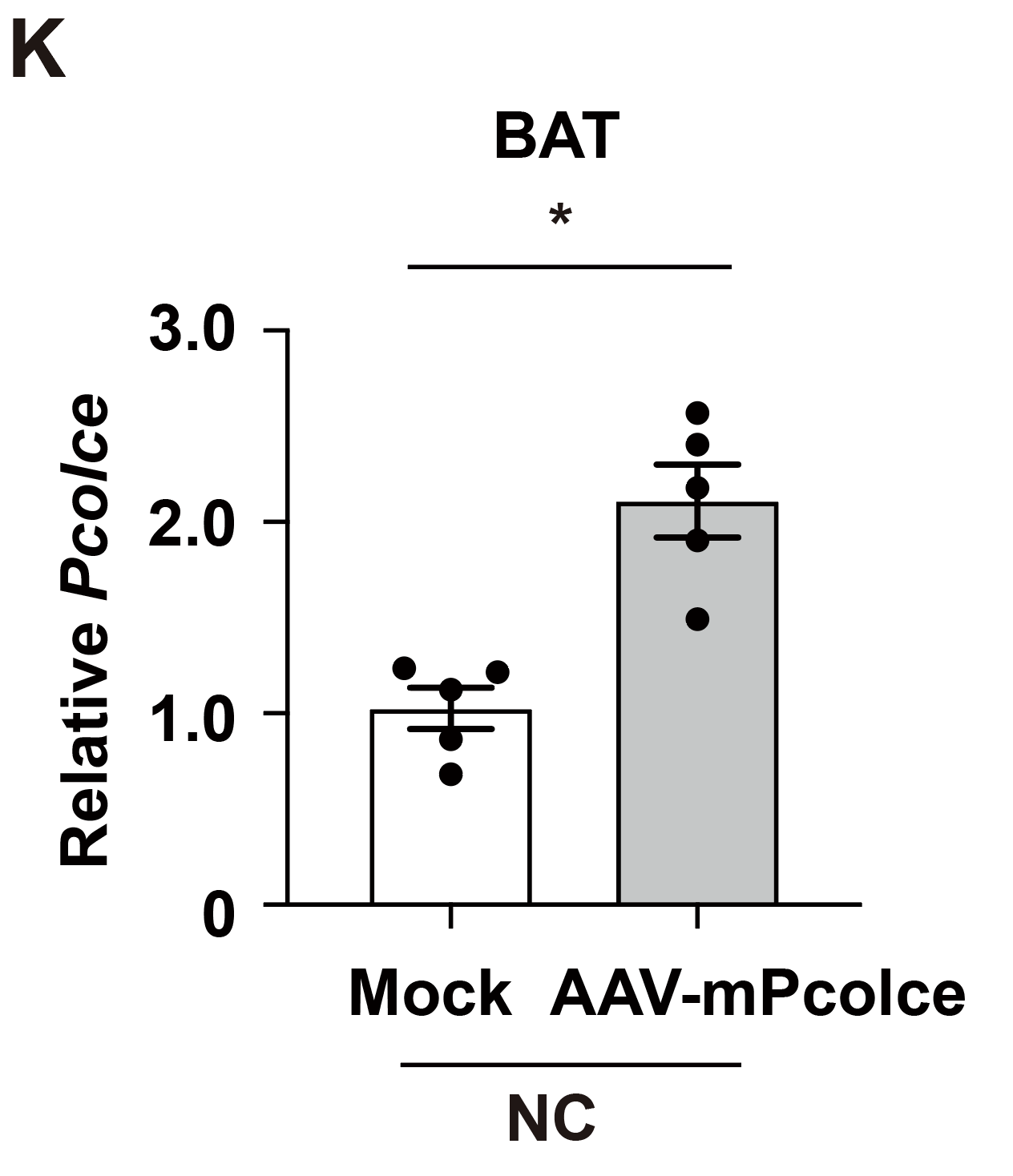

Supplement: Supplementary file 4 — Source data Fig. 2 [file 44318_2024_196_MOESM4_ESM.zip › Figure 2/Figure 2-K/Fig.2K.png]

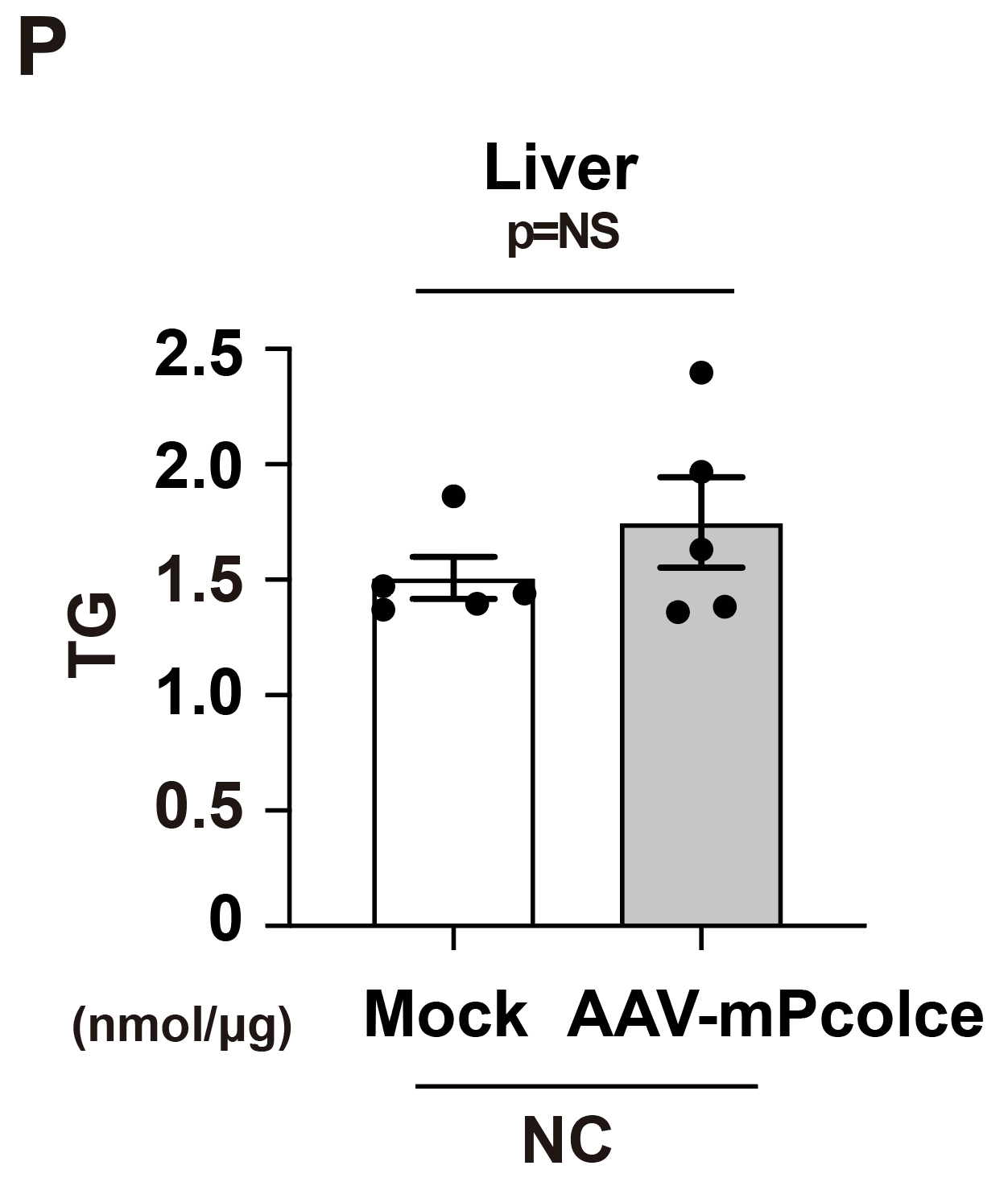

Supplement: Supplementary file 4 — Source data Fig. 2 [file 44318_2024_196_MOESM4_ESM.zip › Figure 2/Figure 2-P/Fig.2P.png]

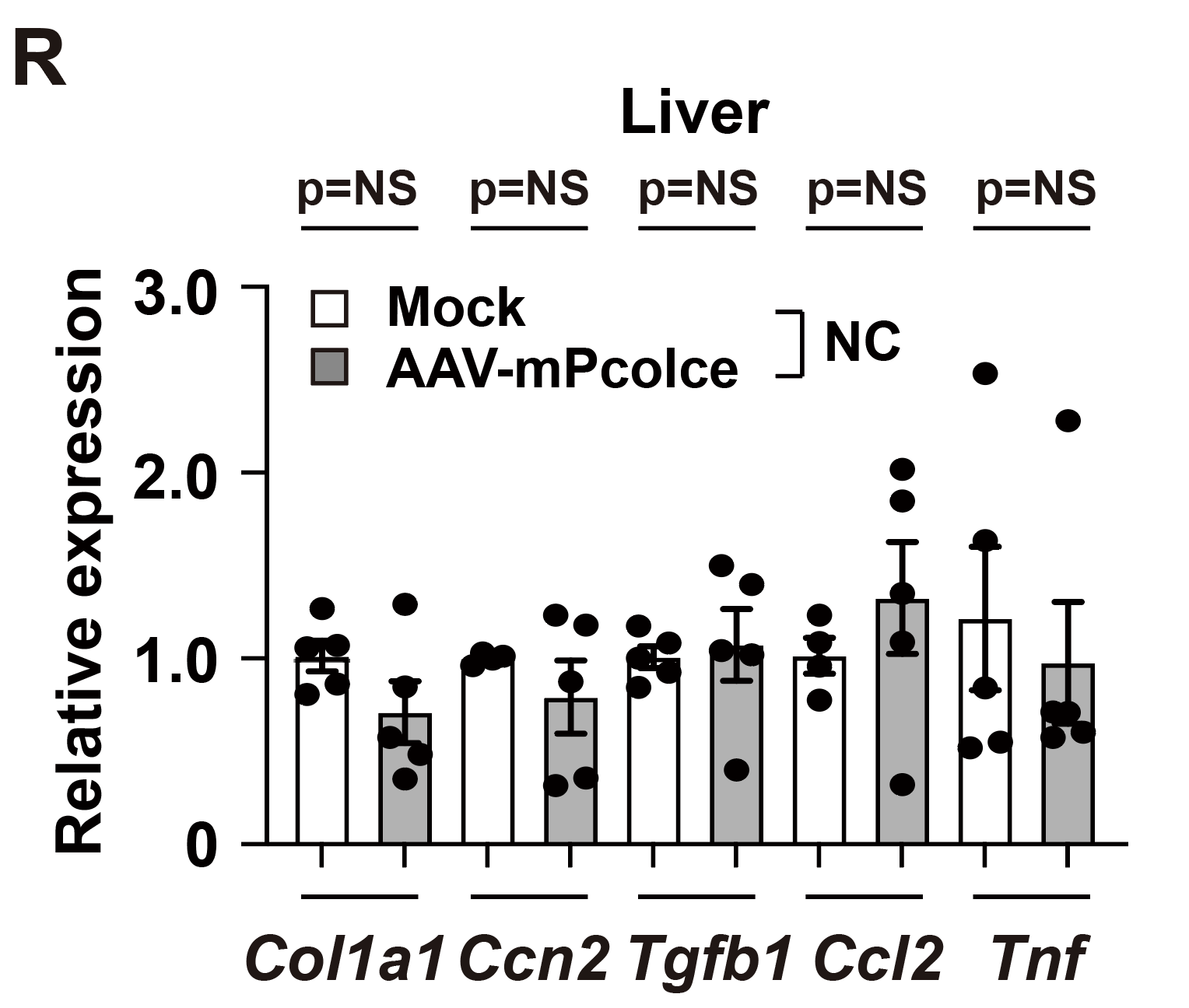

Supplement: Supplementary file 4 — Source data Fig. 2 [file 44318_2024_196_MOESM4_ESM.zip › Figure 2/Figure 2-R/Fig.2R.png]

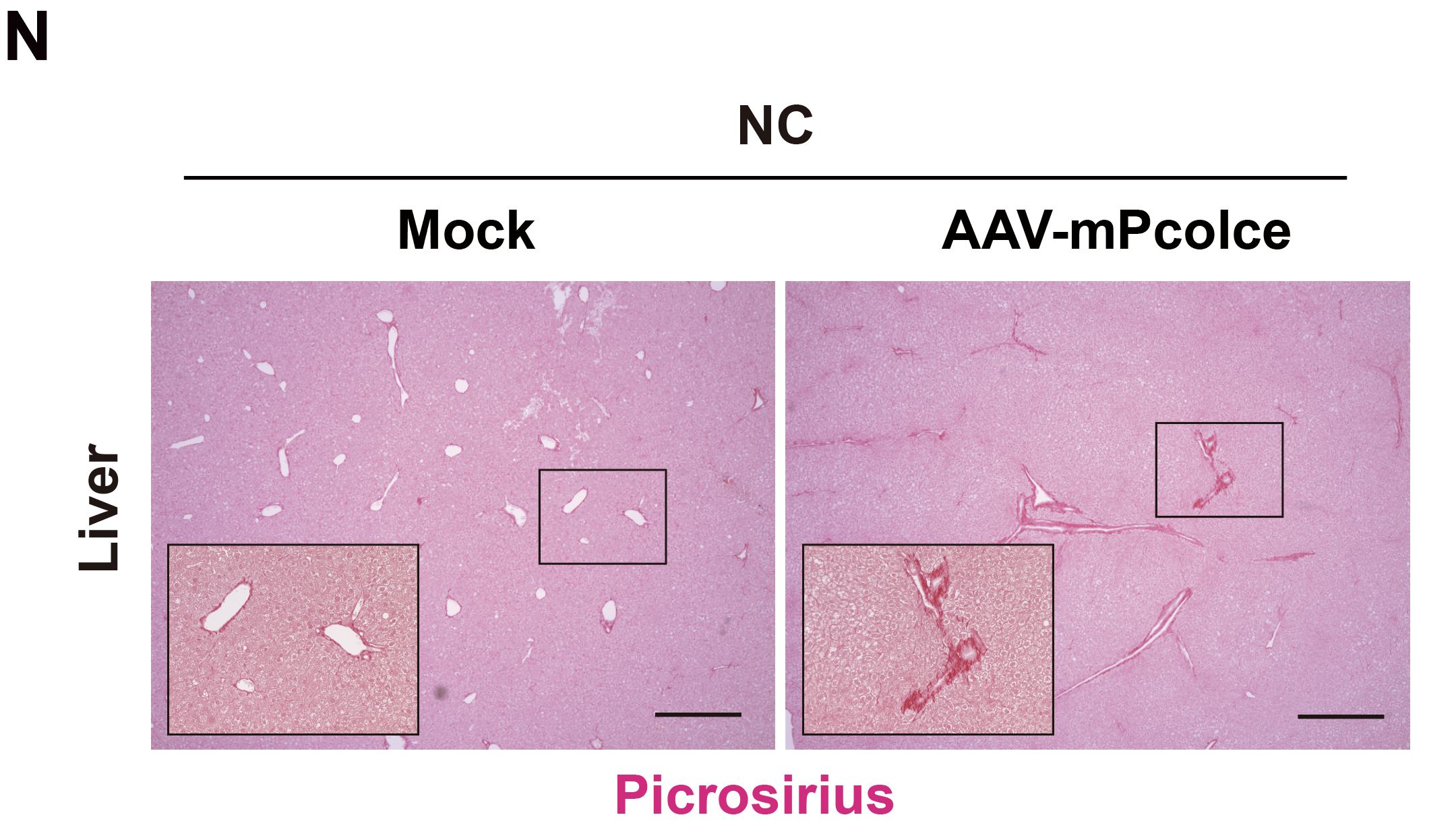

Supplement: Supplementary file 4 — Source data Fig. 2 [file 44318_2024_196_MOESM4_ESM.zip › Figure 2/Figure 2-N/Fig.2N.png]

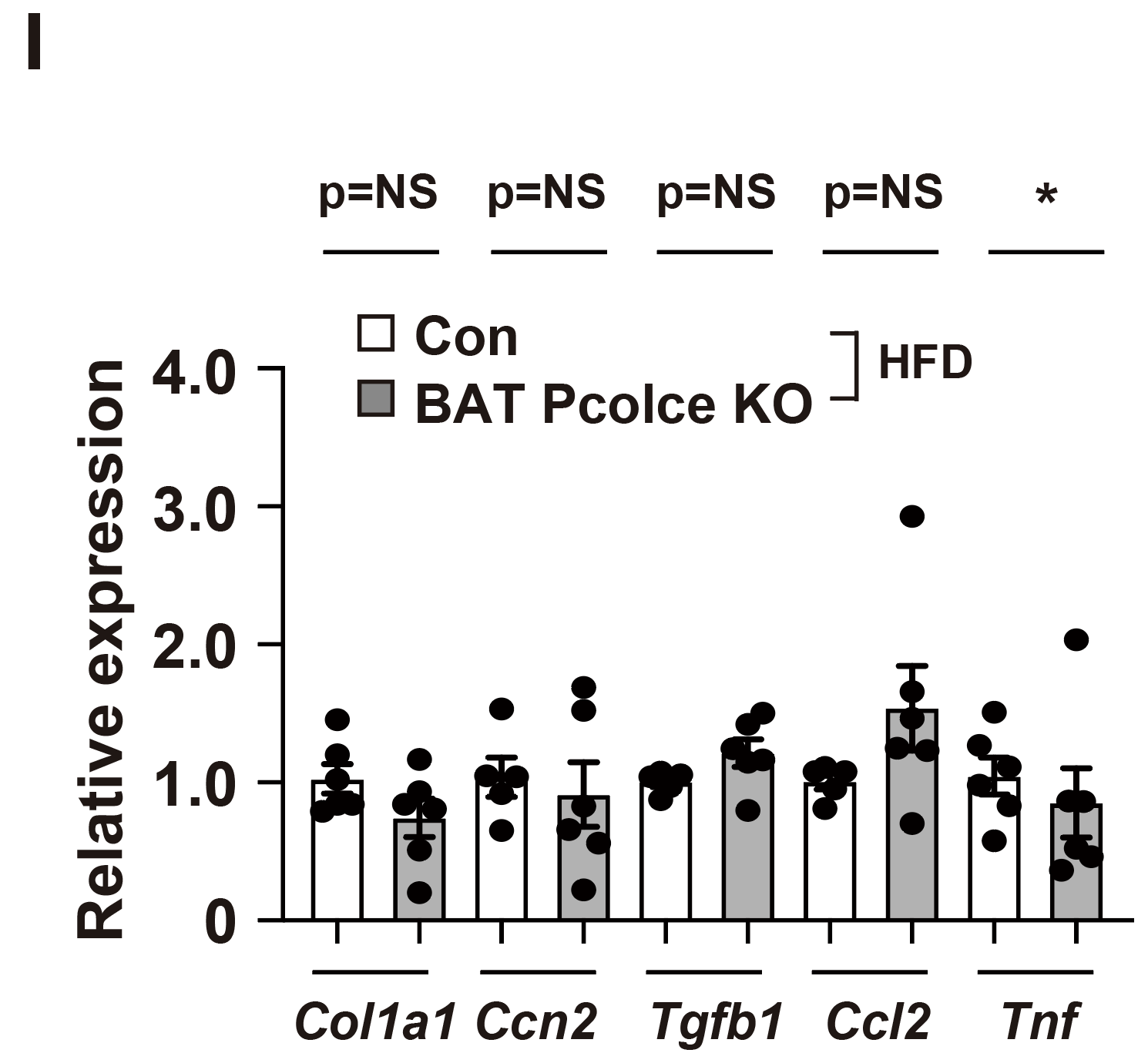

Supplement: Supplementary file 4 — Source data Fig. 2 [file 44318_2024_196_MOESM4_ESM.zip › Figure 2/Figure 2-I/Fig.2I.png]

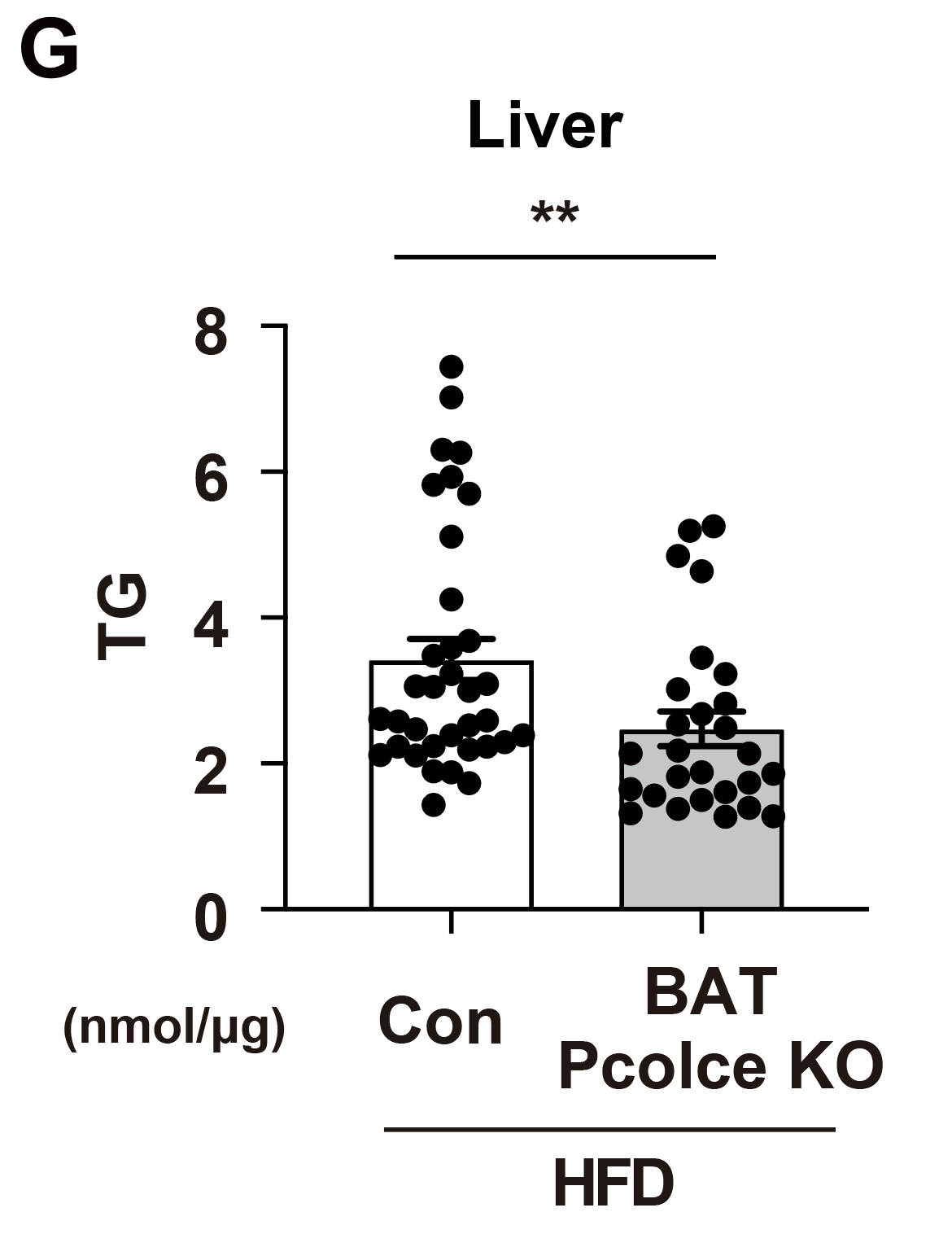

Supplement: Supplementary file 4 — Source data Fig. 2 [file 44318_2024_196_MOESM4_ESM.zip › Figure 2/Figure 2-G/Fig.2G.png]

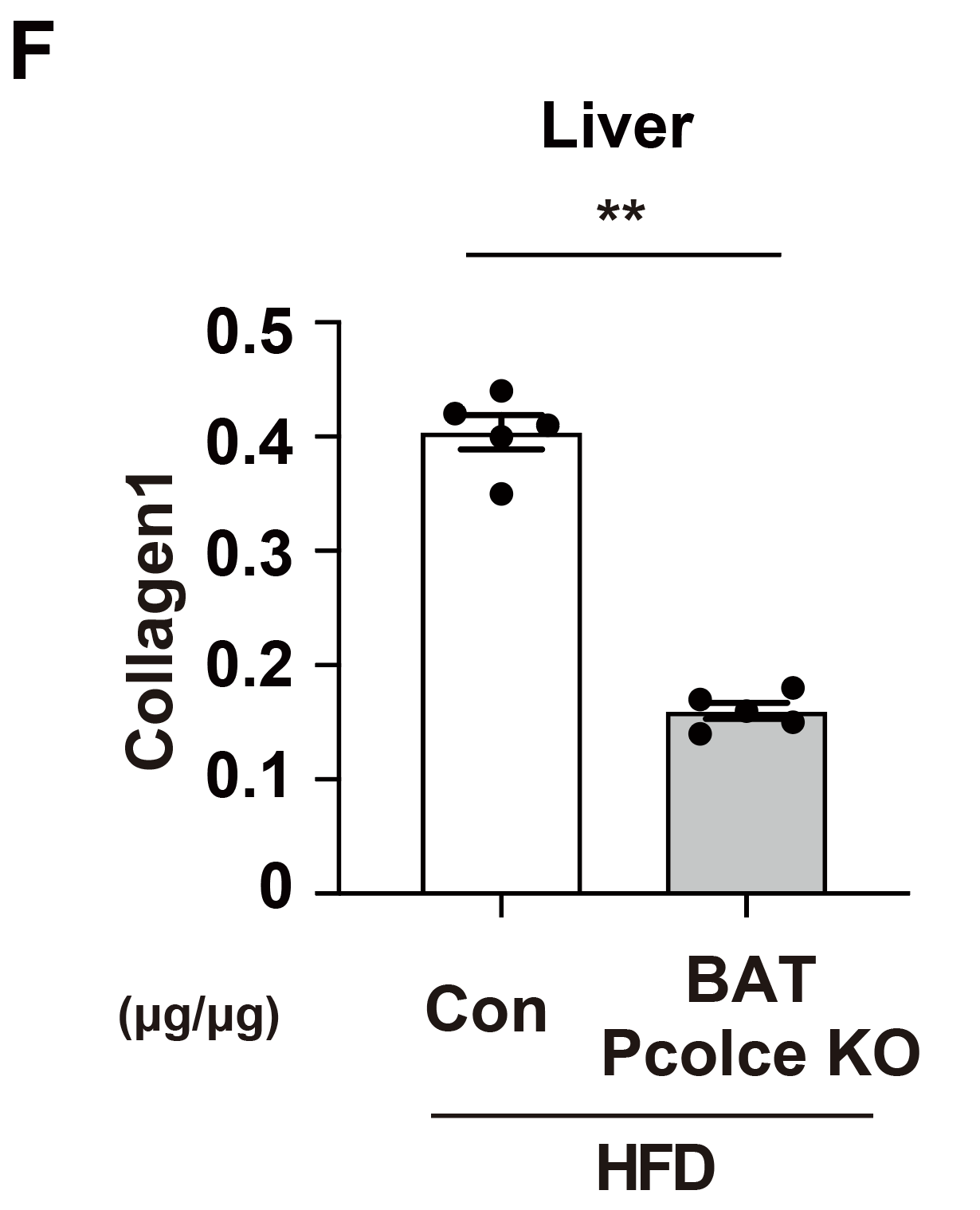

Supplement: Supplementary file 4 — Source data Fig. 2 [file 44318_2024_196_MOESM4_ESM.zip › Figure 2/Figure 2-F/Fig.2F.png]

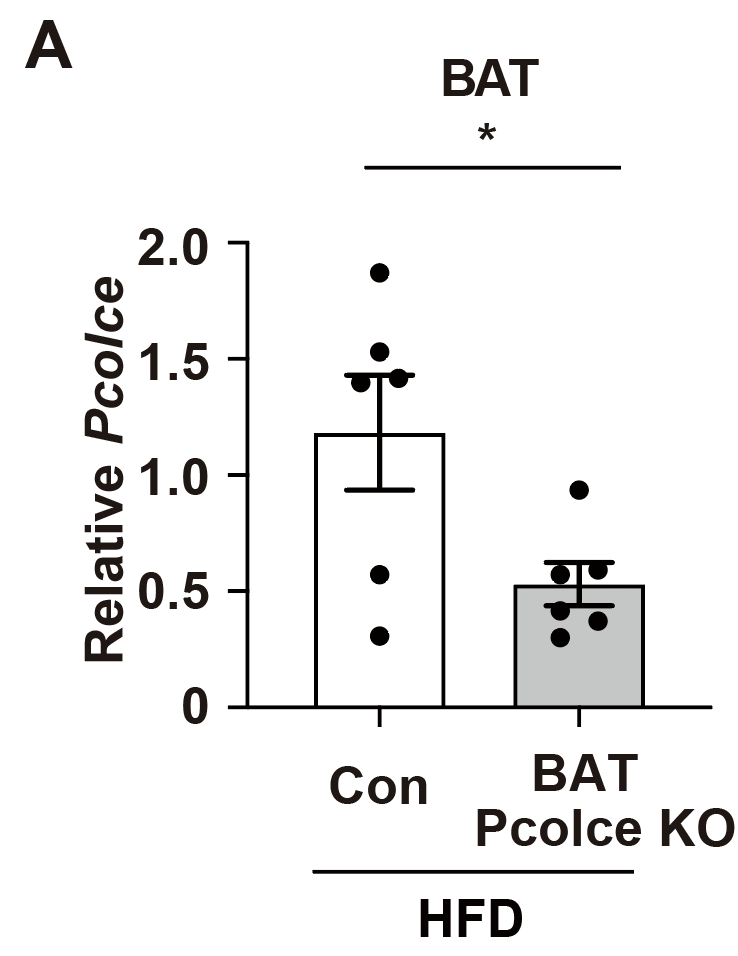

Supplement: Supplementary file 4 — Source data Fig. 2 [file 44318_2024_196_MOESM4_ESM.zip › Figure 2/Figure 2-A/Fig.2A.png]

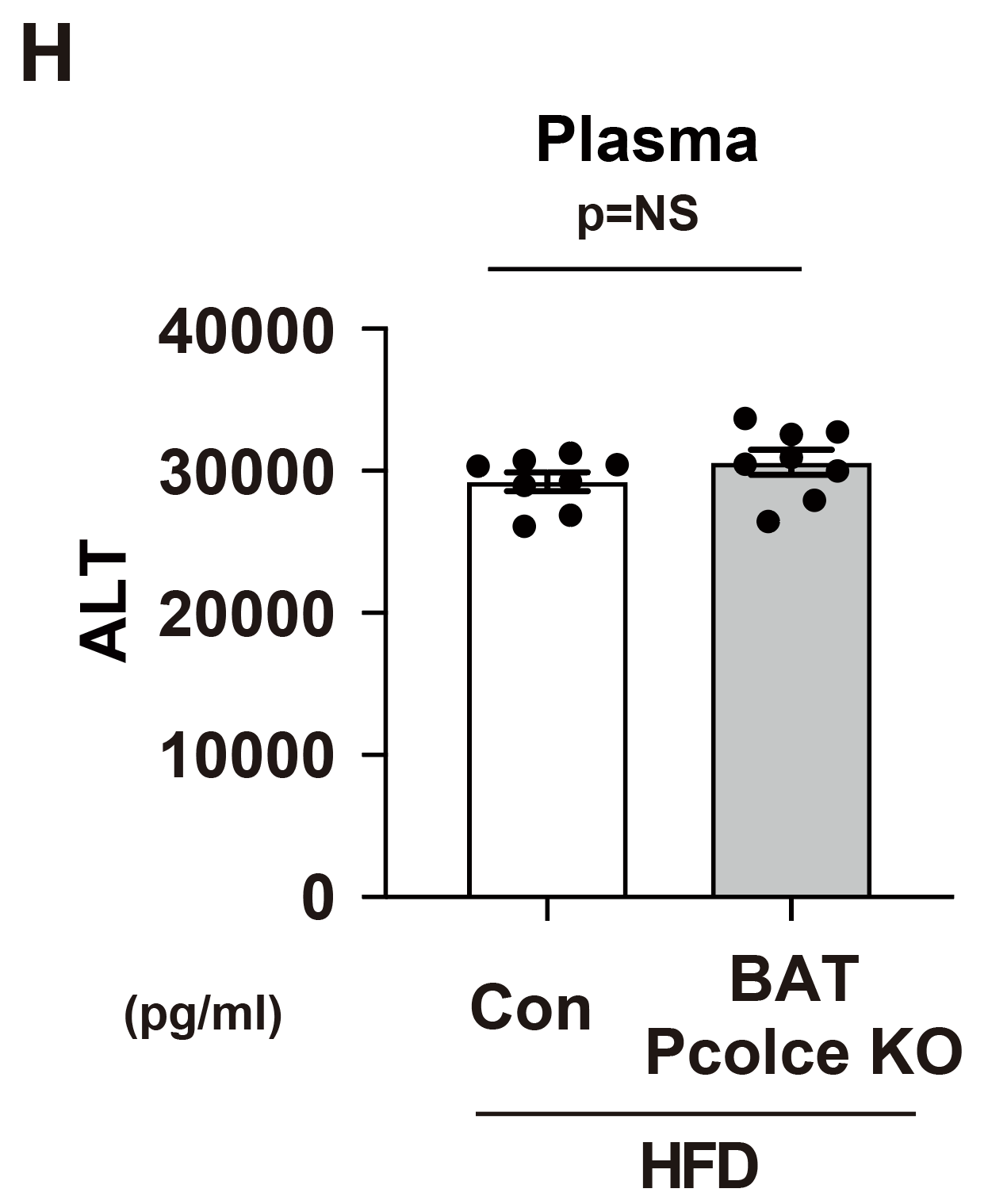

Supplement: Supplementary file 4 — Source data Fig. 2 [file 44318_2024_196_MOESM4_ESM.zip › Figure 2/Figure 2-H/Fig.2H.png]

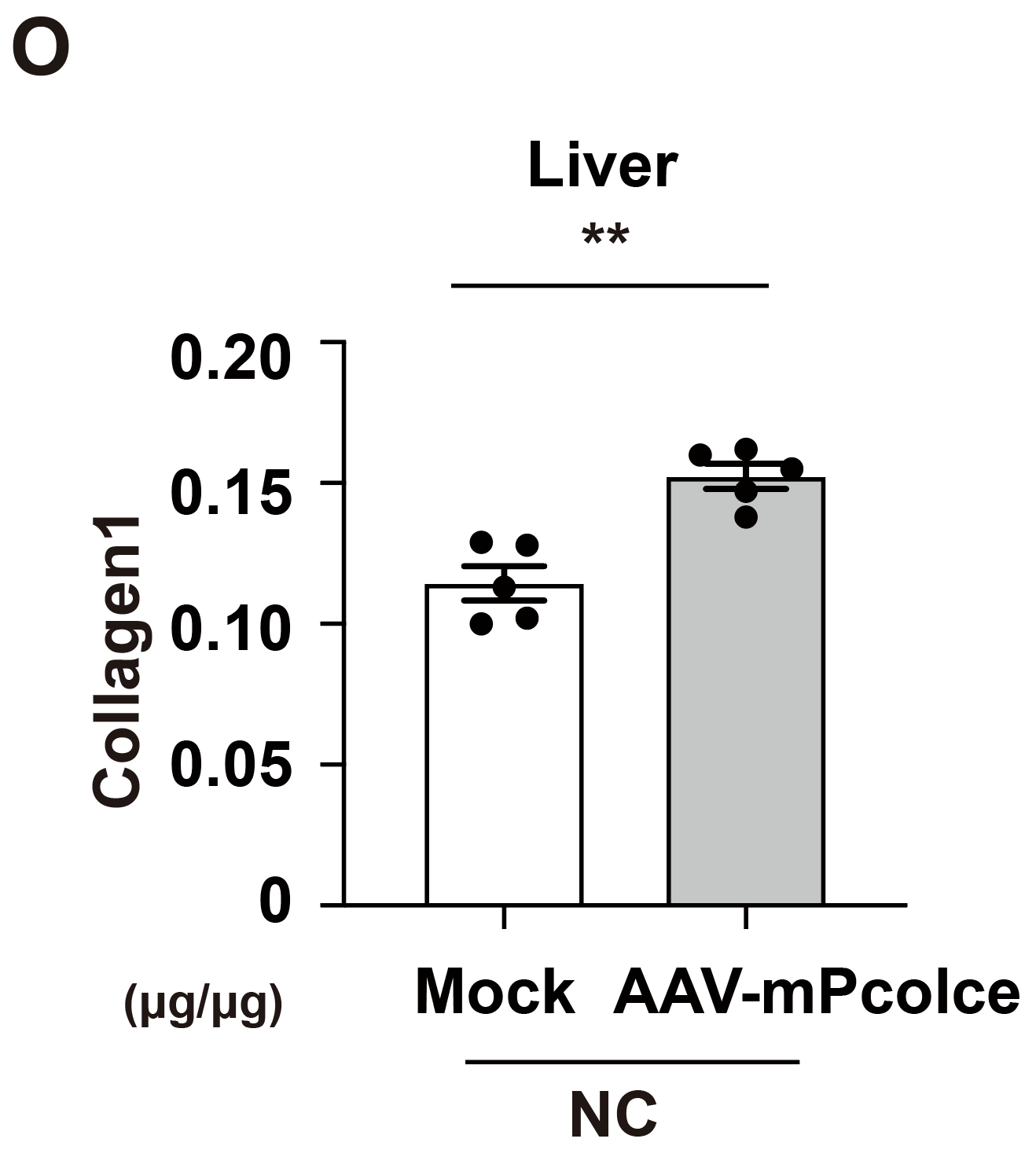

Supplement: Supplementary file 4 — Source data Fig. 2 [file 44318_2024_196_MOESM4_ESM.zip › Figure 2/Figure 2-O/Fig.2O.png]

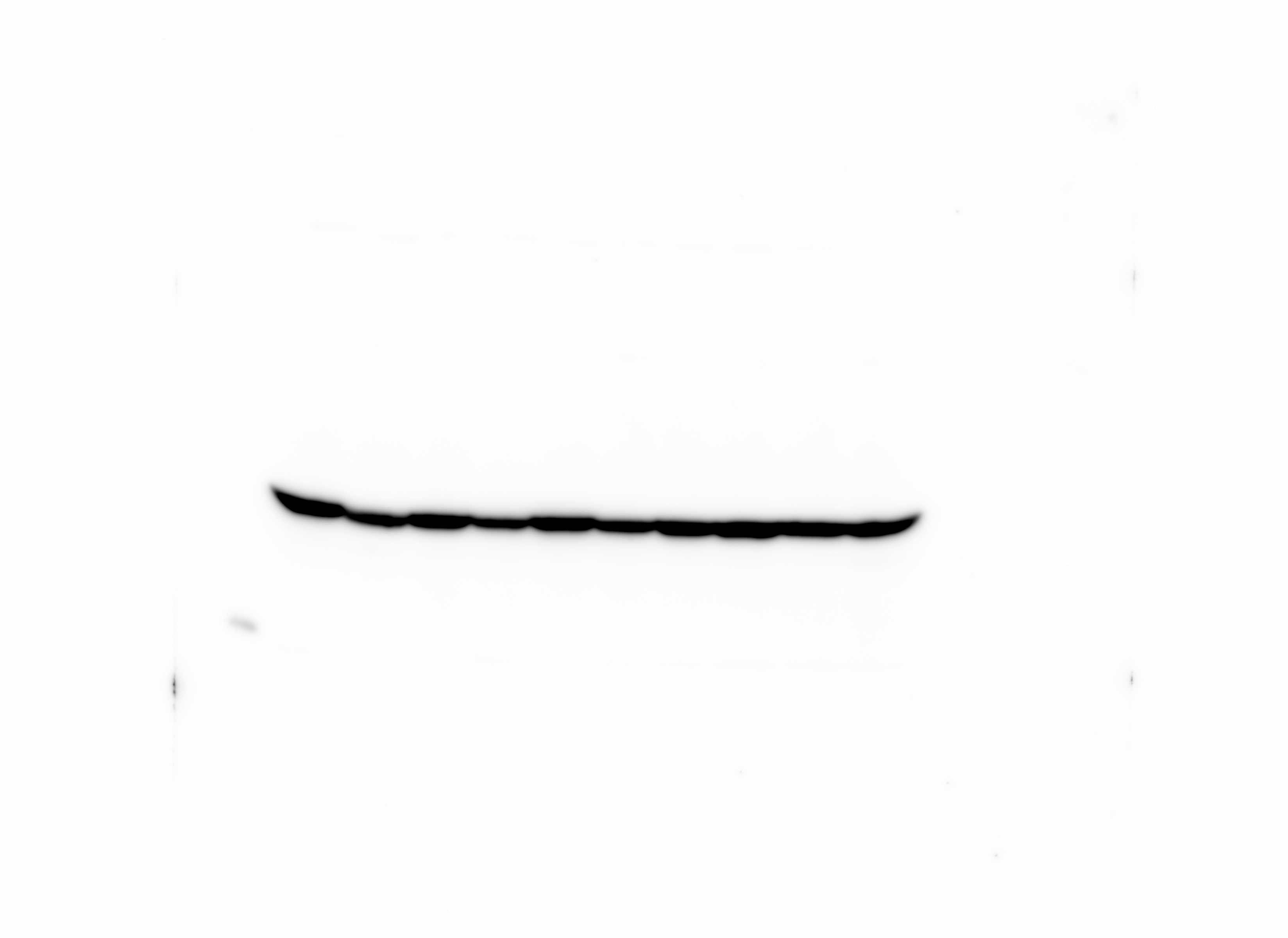

Supplement: Supplementary file 4 — Source data Fig. 2 [file 44318_2024_196_MOESM4_ESM.zip › Figure 2/Figure 2-D/Quantificated image/Actin.tif]

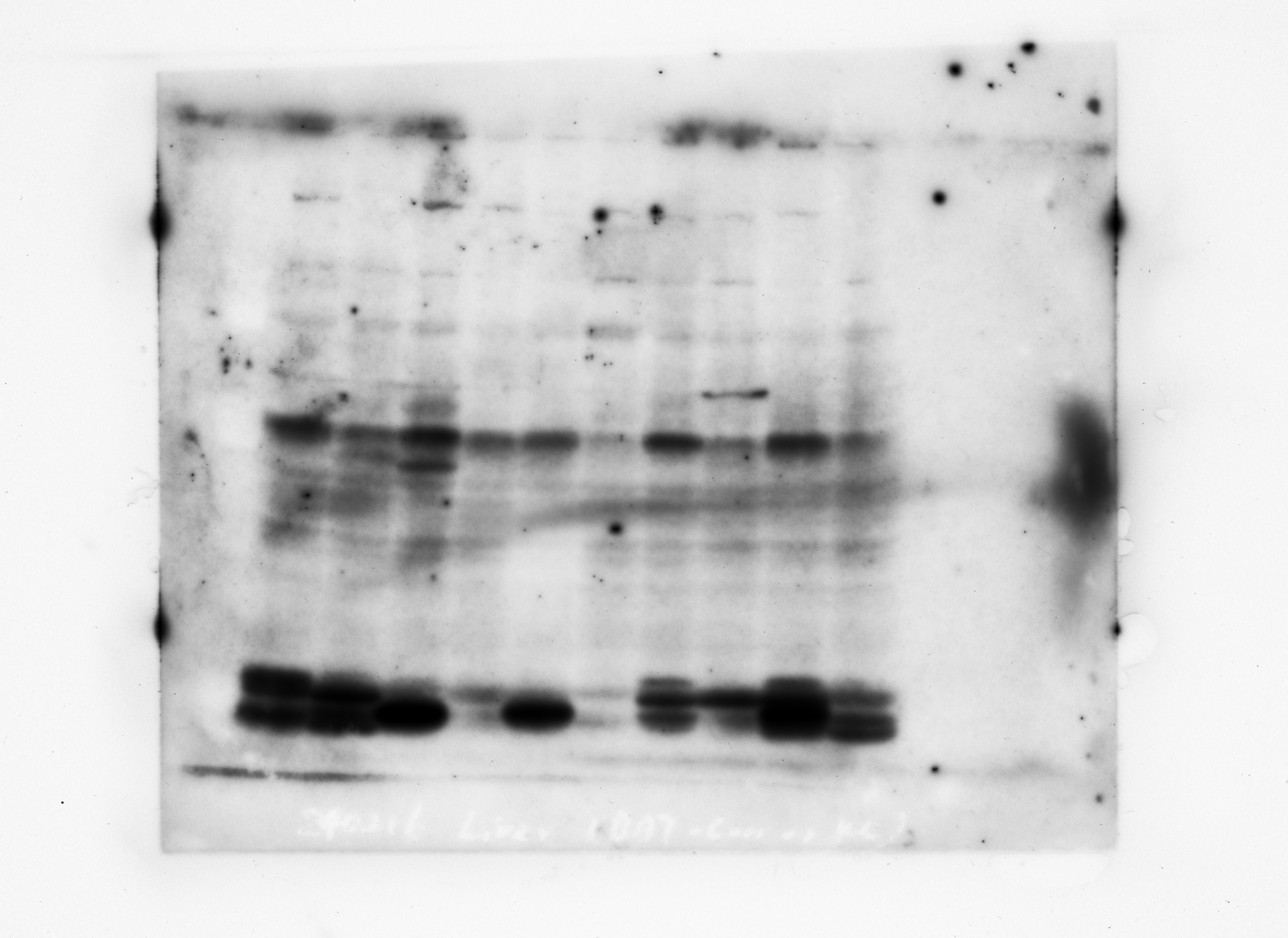

Supplement: Supplementary file 4 — Source data Fig. 2 [file 44318_2024_196_MOESM4_ESM.zip › Figure 2/Figure 2-D/Quantificated image/PCPE-1.tif]

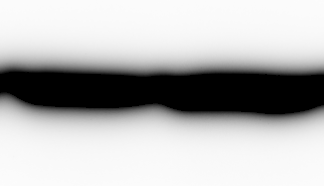

Supplement: Supplementary file 4 — Source data Fig. 2 [file 44318_2024_196_MOESM4_ESM.zip › Figure 2/Figure 2-D/Demonstrated image/Actin.tif]

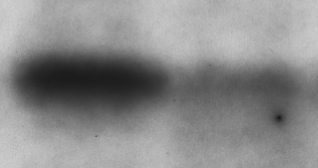

Supplement: Supplementary file 4 — Source data Fig. 2 [file 44318_2024_196_MOESM4_ESM.zip › Figure 2/Figure 2-D/Demonstrated image/PCPE-1.tif]

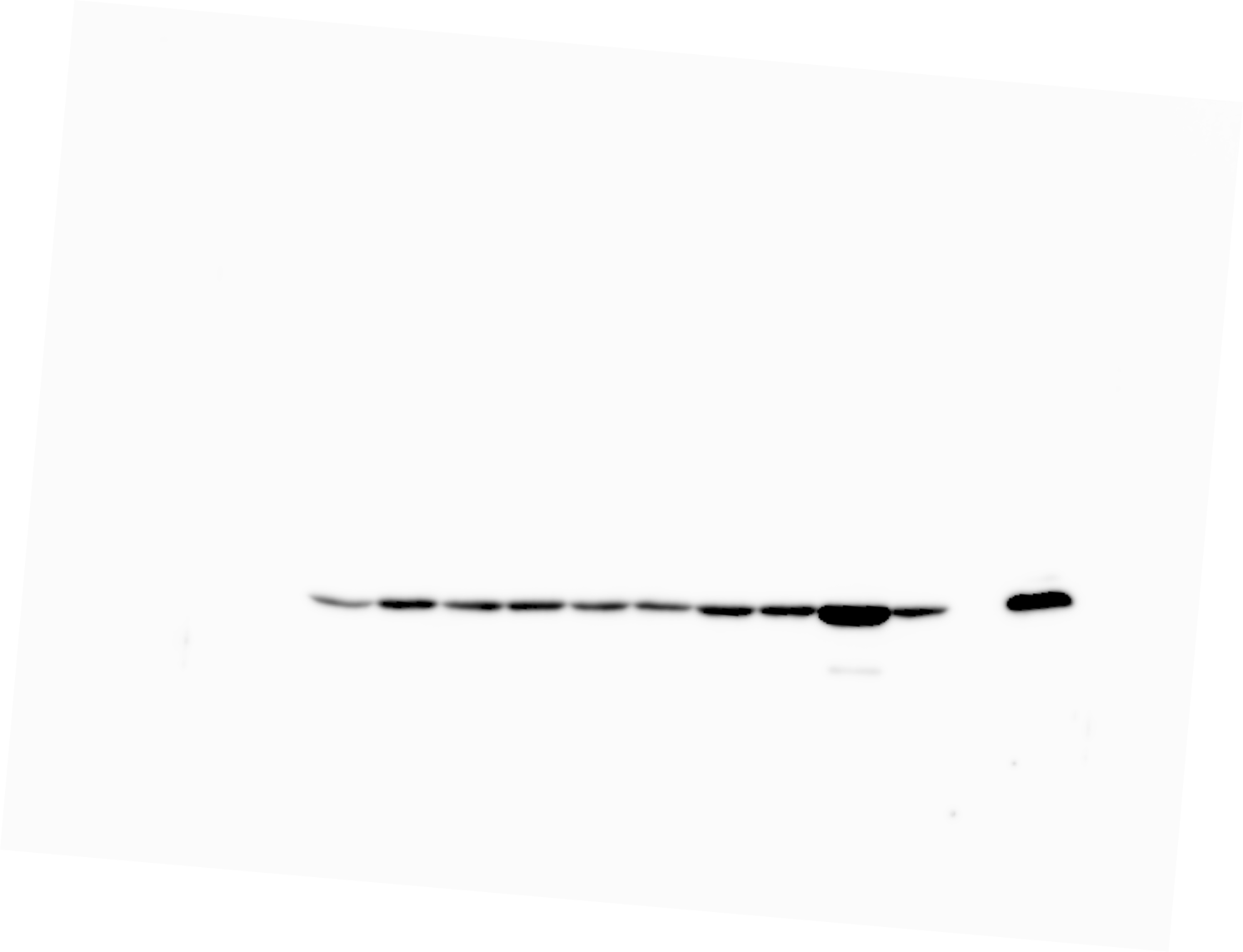

Supplement: Supplementary file 4 — Source data Fig. 2 [file 44318_2024_196_MOESM4_ESM.zip › Figure 2/Figure 2-B/Quantificated image/Actin.tif]

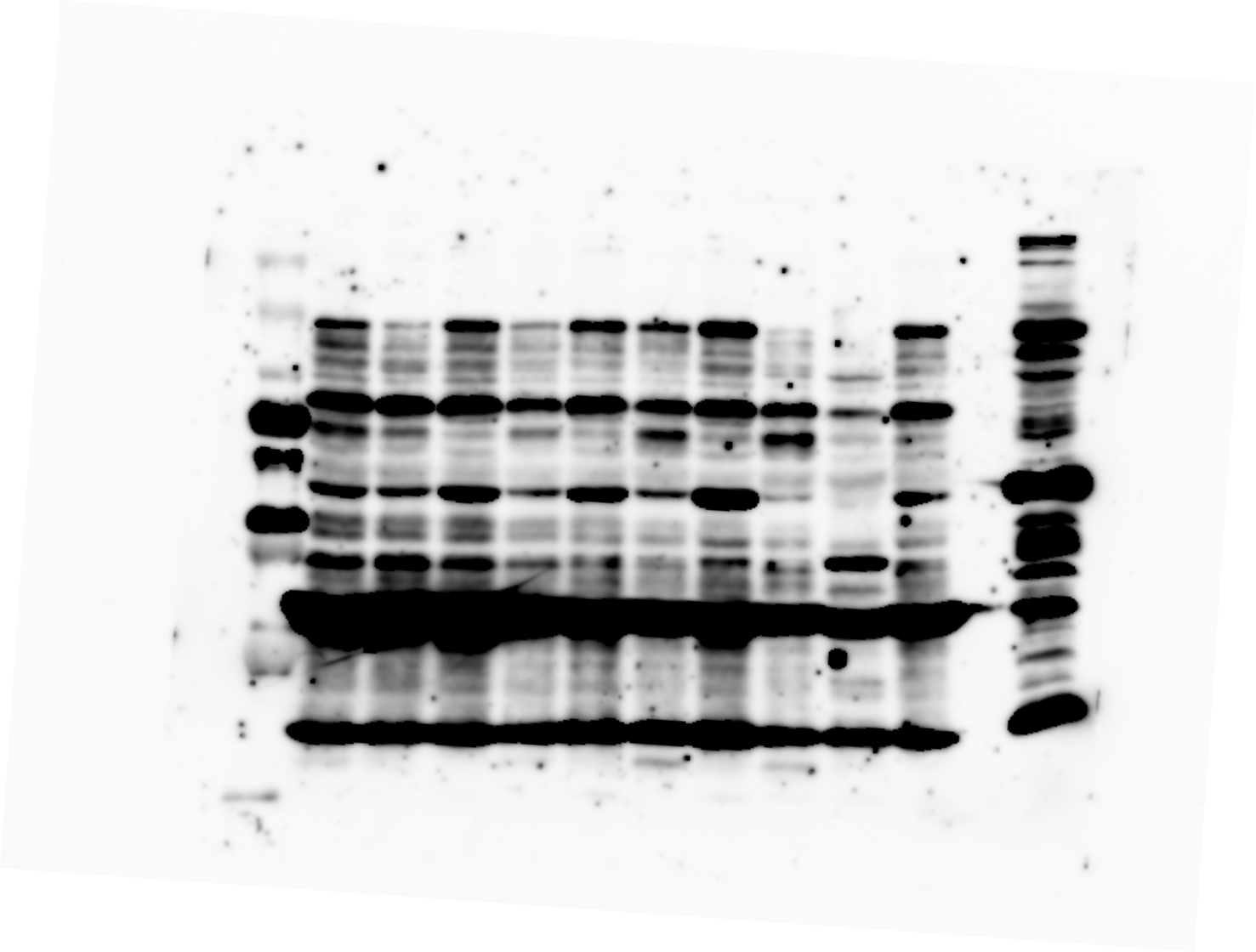

Supplement: Supplementary file 4 — Source data Fig. 2 [file 44318_2024_196_MOESM4_ESM.zip › Figure 2/Figure 2-B/Quantificated image/PCPE-1.tif]

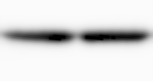

Supplement: Supplementary file 4 — Source data Fig. 2 [file 44318_2024_196_MOESM4_ESM.zip › Figure 2/Figure 2-B/Demonstrated image/Actin.tif]

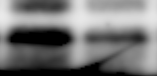

Supplement: Supplementary file 4 — Source data Fig. 2 [file 44318_2024_196_MOESM4_ESM.zip › Figure 2/Figure 2-B/Demonstrated image/PCPE-1.tif]

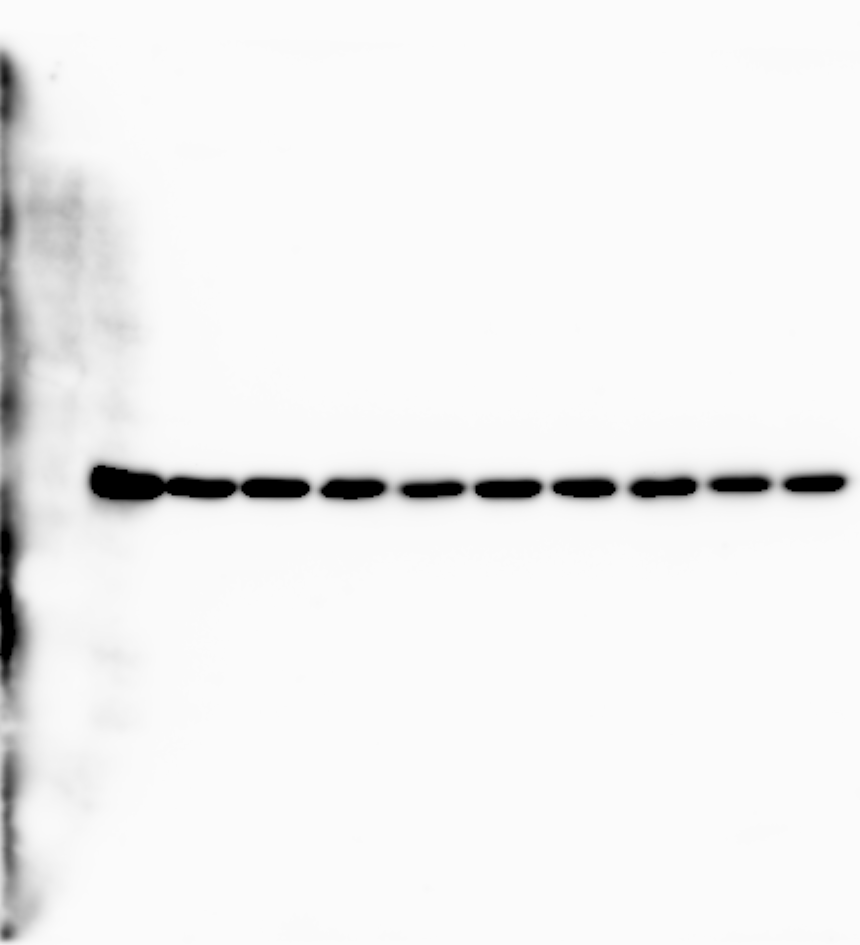

Supplement: Supplementary file 4 — Source data Fig. 2 [file 44318_2024_196_MOESM4_ESM.zip › Figure 2/Figure 2-L/Quantificated image/Tubulin.tif]

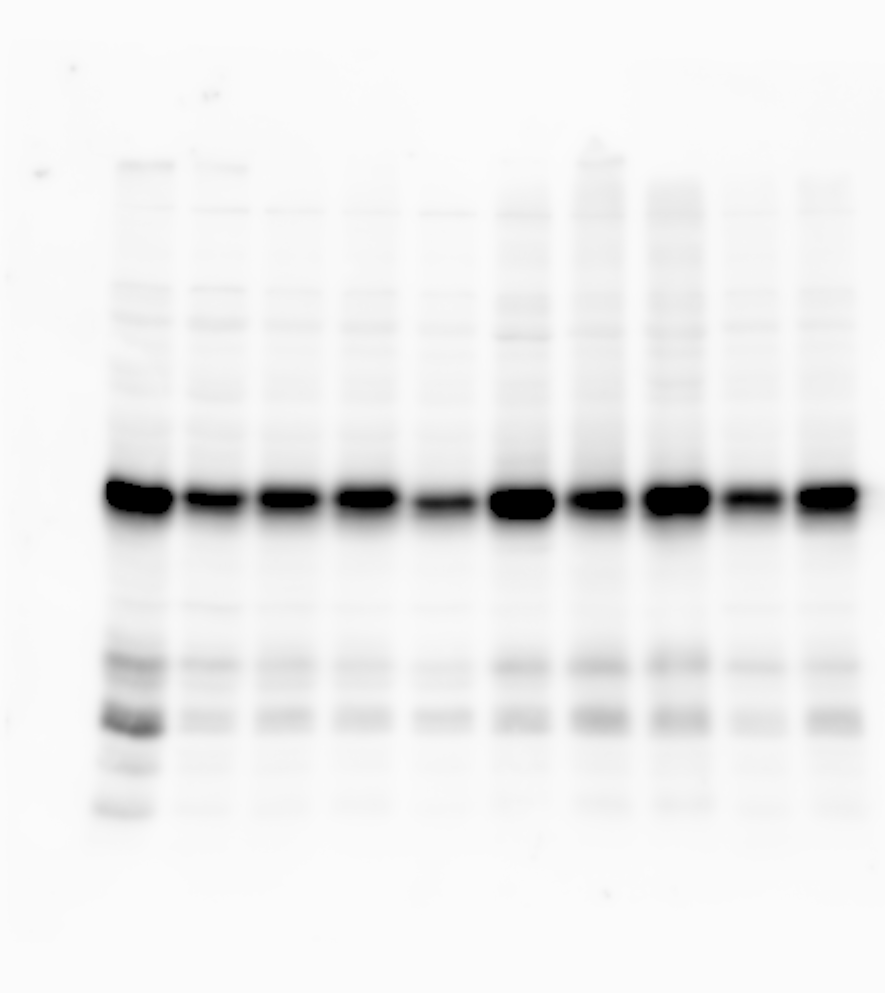

Supplement: Supplementary file 4 — Source data Fig. 2 [file 44318_2024_196_MOESM4_ESM.zip › Figure 2/Figure 2-L/Quantificated image/PCPE-1.tif]

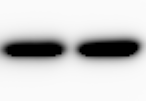

Supplement: Supplementary file 4 — Source data Fig. 2 [file 44318_2024_196_MOESM4_ESM.zip › Figure 2/Figure 2-L/Demonstrated image/Tubulin.tif]

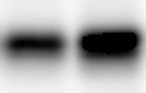

Supplement: Supplementary file 4 — Source data Fig. 2 [file 44318_2024_196_MOESM4_ESM.zip › Figure 2/Figure 2-L/Demonstrated image/PCPE-1.tif]

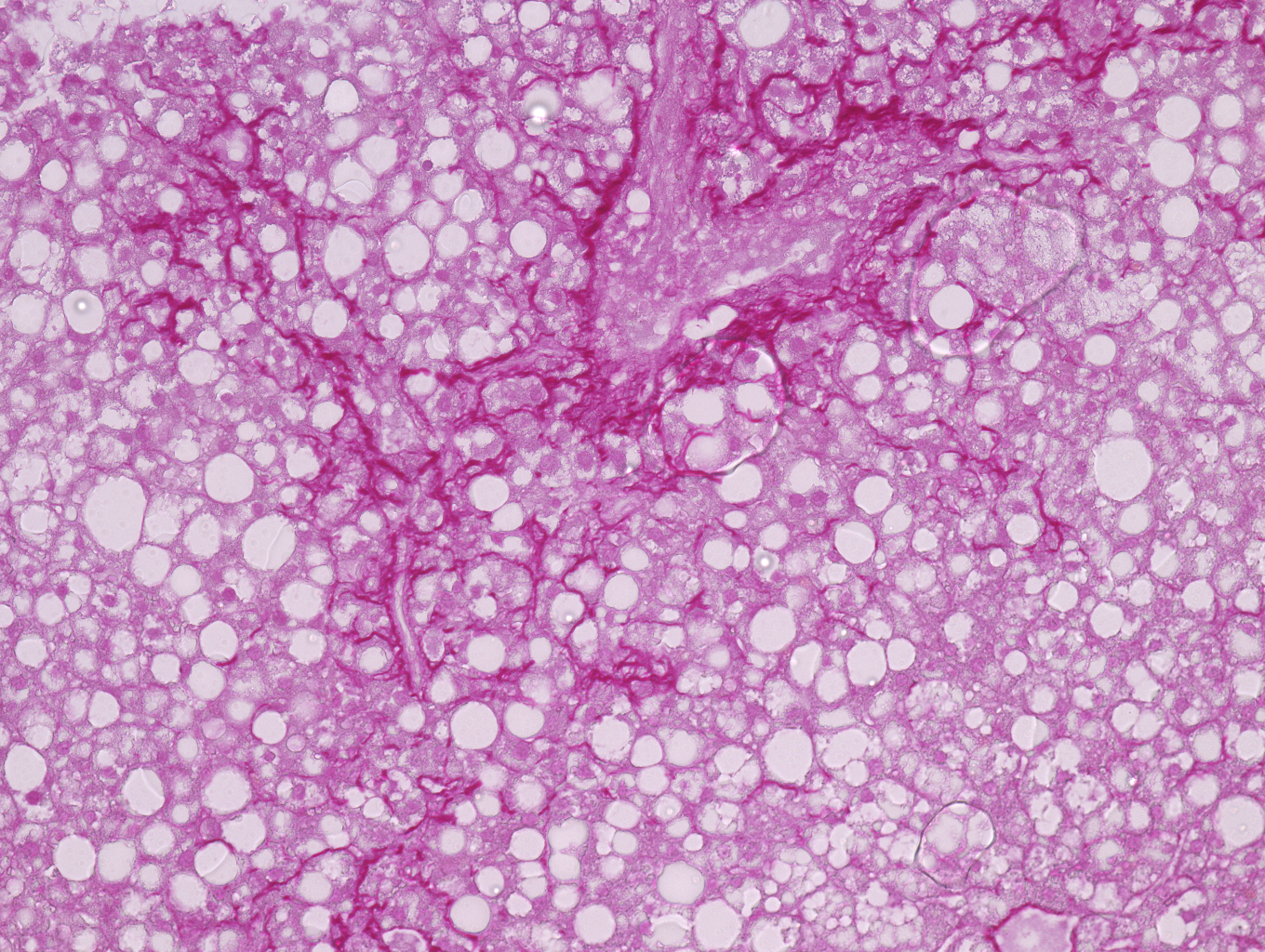

Supplement: Supplementary file 4 — Source data Fig. 2 [file 44318_2024_196_MOESM4_ESM.zip › Figure 2/Figure 2-E/Demonstrated image/HFD Con/HFD Liver Con high mag.tif]

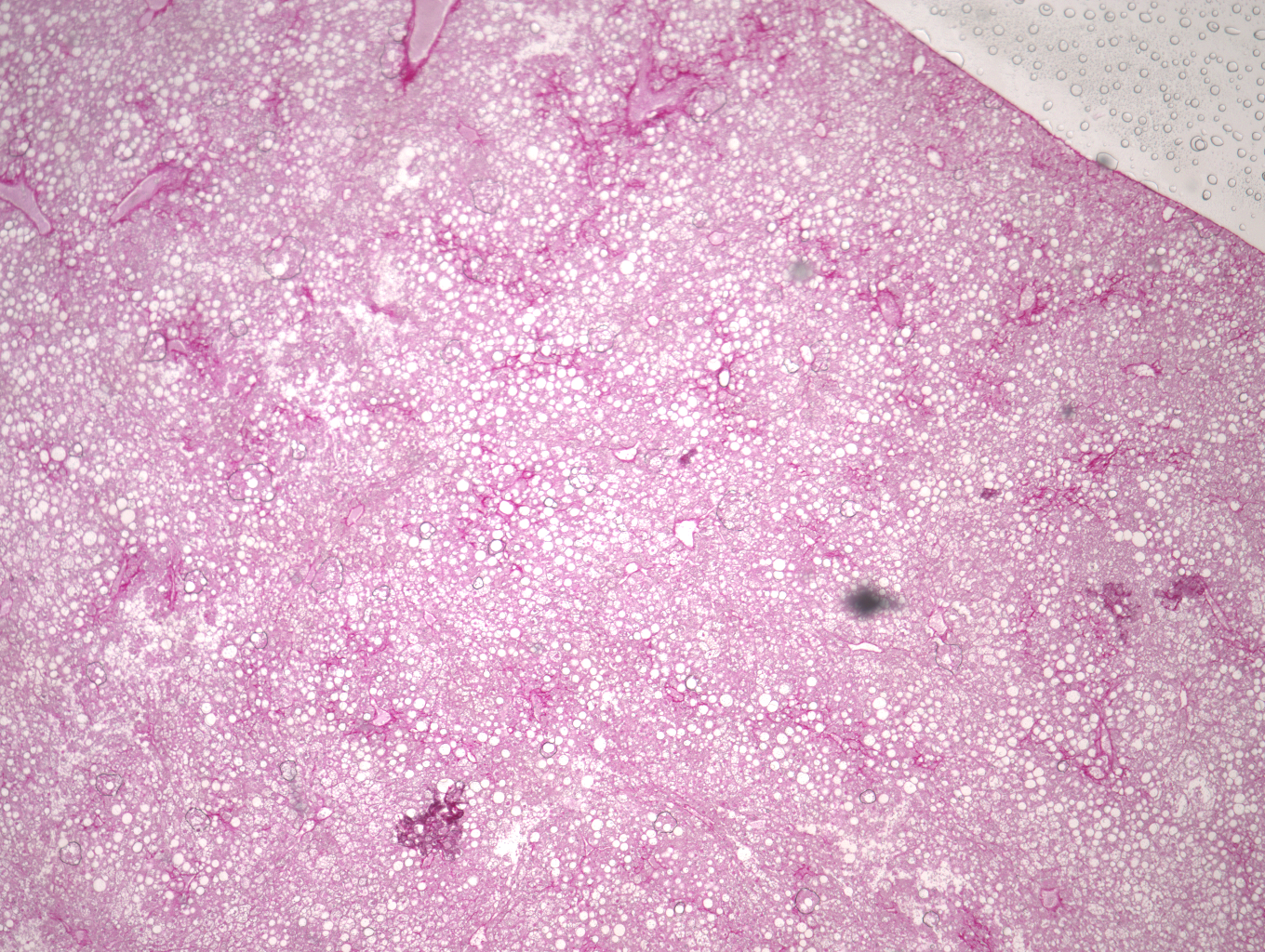

Supplement: Supplementary file 4 — Source data Fig. 2 [file 44318_2024_196_MOESM4_ESM.zip › Figure 2/Figure 2-E/Demonstrated image/HFD Con/HFD Liver Con.tiff]

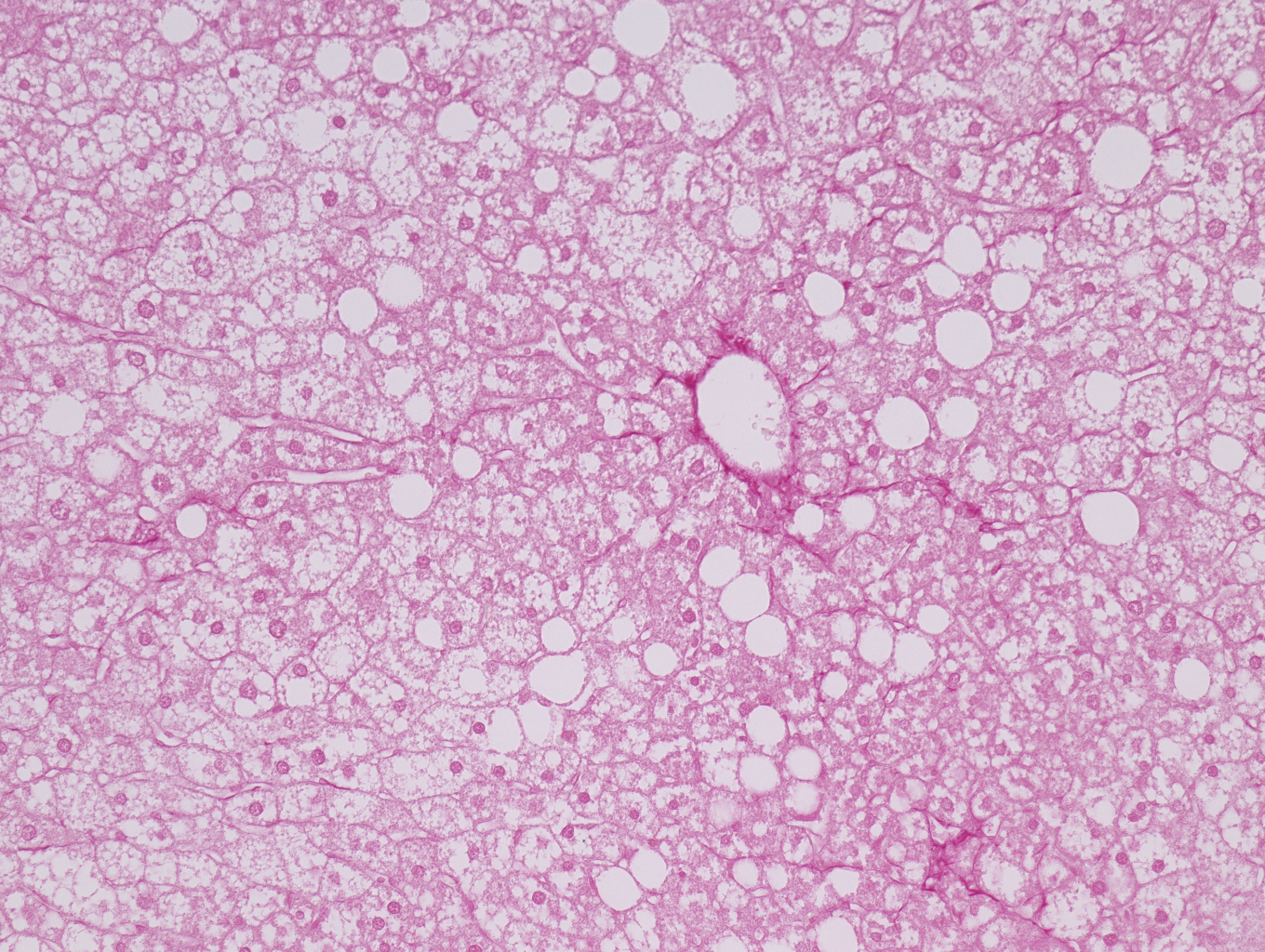

Supplement: Supplementary file 4 — Source data Fig. 2 [file 44318_2024_196_MOESM4_ESM.zip › Figure 2/Figure 2-E/Demonstrated image/HFD BAT Pcolce KO/BAT PcolceKO HFD high mag.tif]

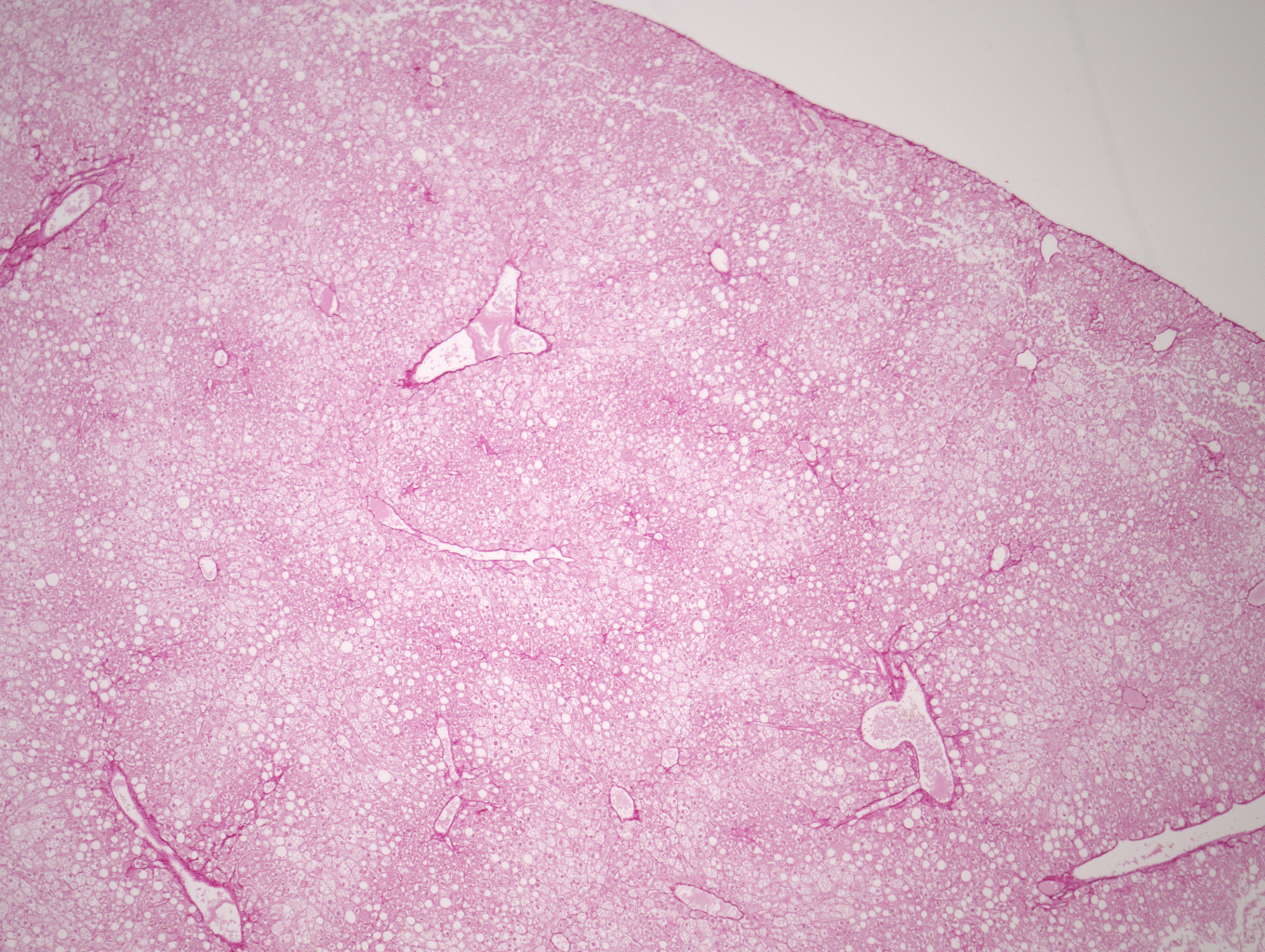

Supplement: Supplementary file 4 — Source data Fig. 2 [file 44318_2024_196_MOESM4_ESM.zip › Figure 2/Figure 2-E/Demonstrated image/HFD BAT Pcolce KO/BAT PcolceKO HFD.tif]

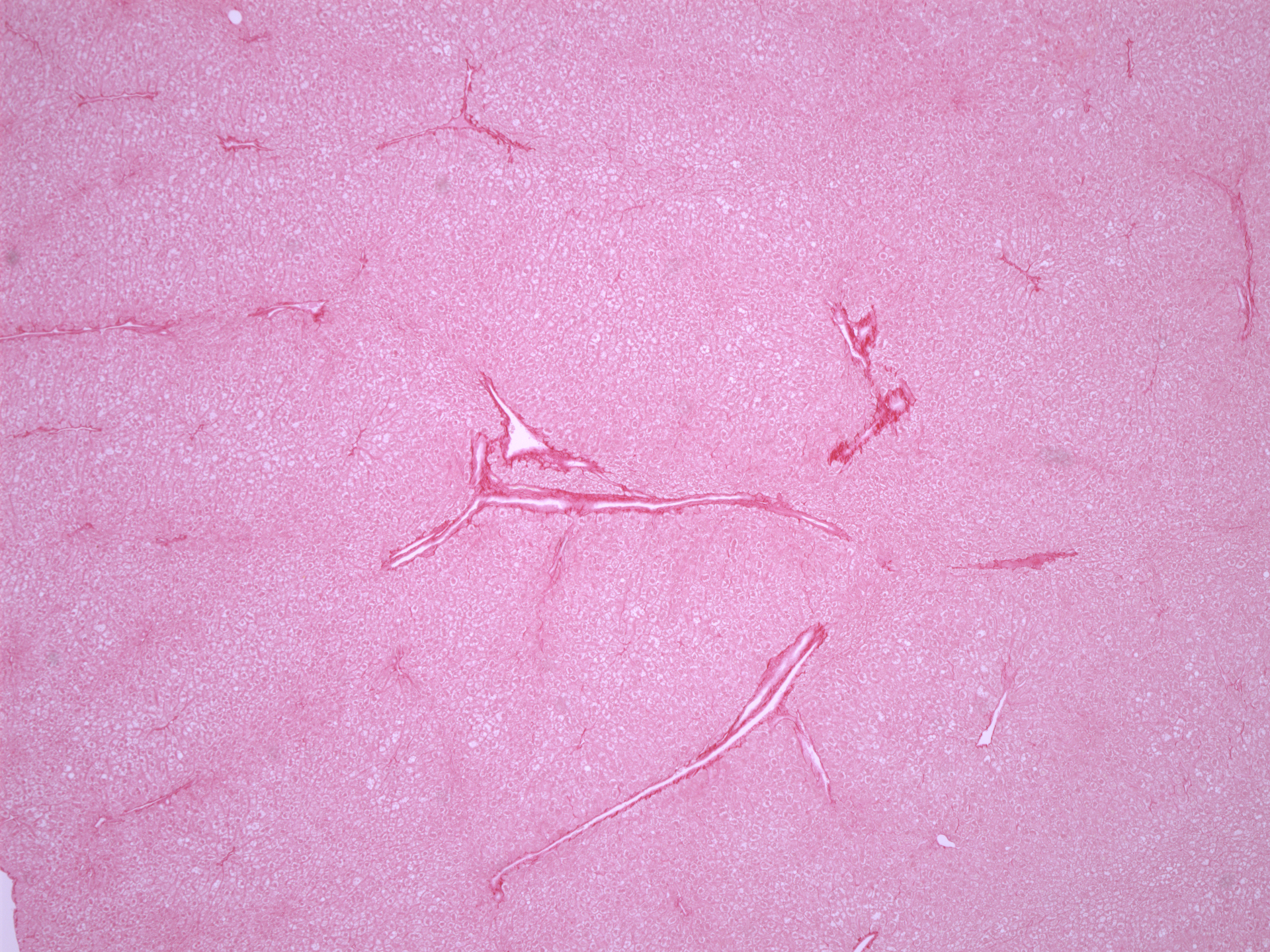

Supplement: Supplementary file 4 — Source data Fig. 2 [file 44318_2024_196_MOESM4_ESM.zip › Figure 2/Figure 2-N/Demonstrated image/NC AAV-mPcolce/NC-AAV-mPcolce-4x.tif]

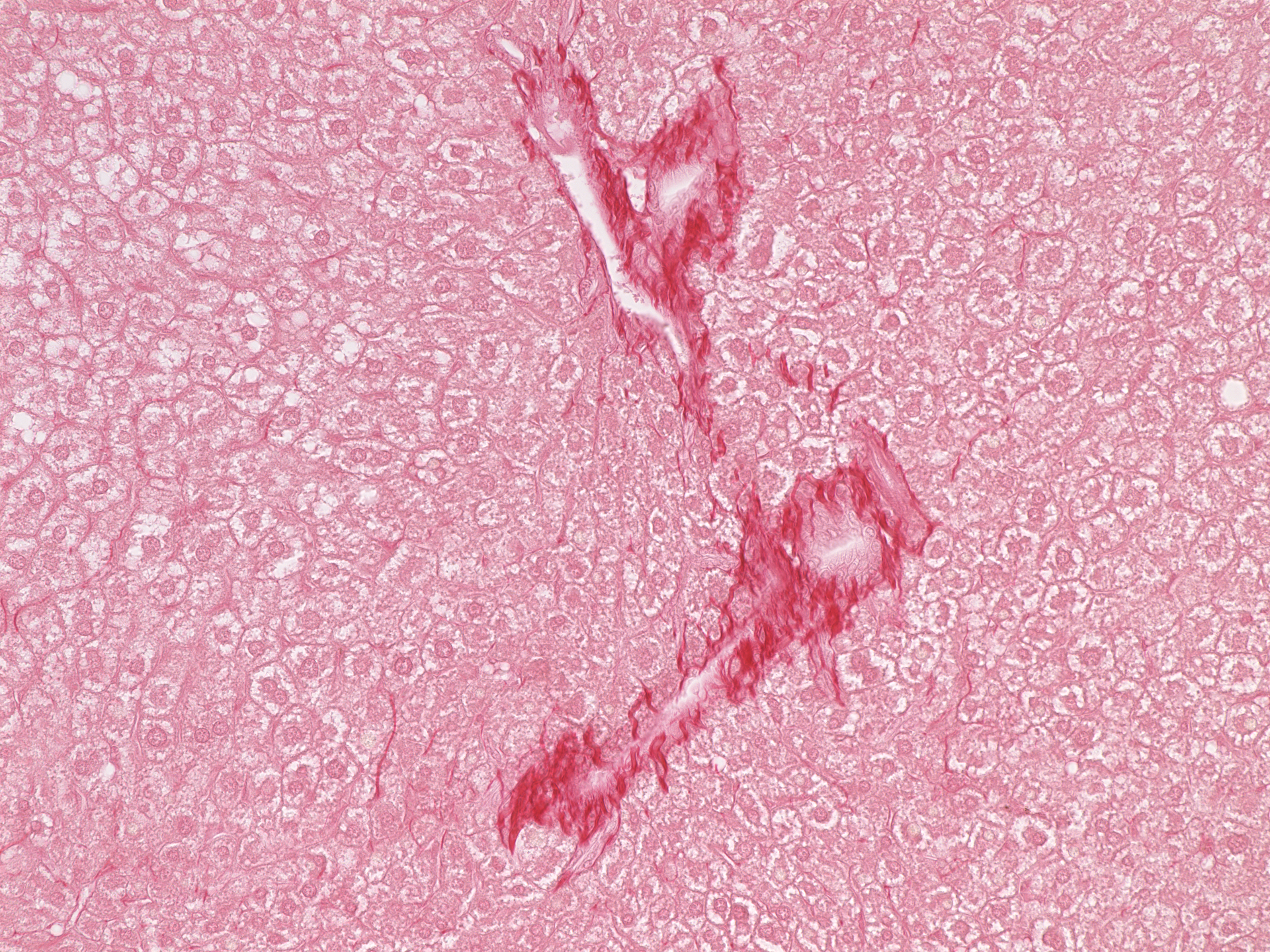

Supplement: Supplementary file 4 — Source data Fig. 2 [file 44318_2024_196_MOESM4_ESM.zip › Figure 2/Figure 2-N/Demonstrated image/NC AAV-mPcolce/NC-AAV-mPcolce-20x.tif]

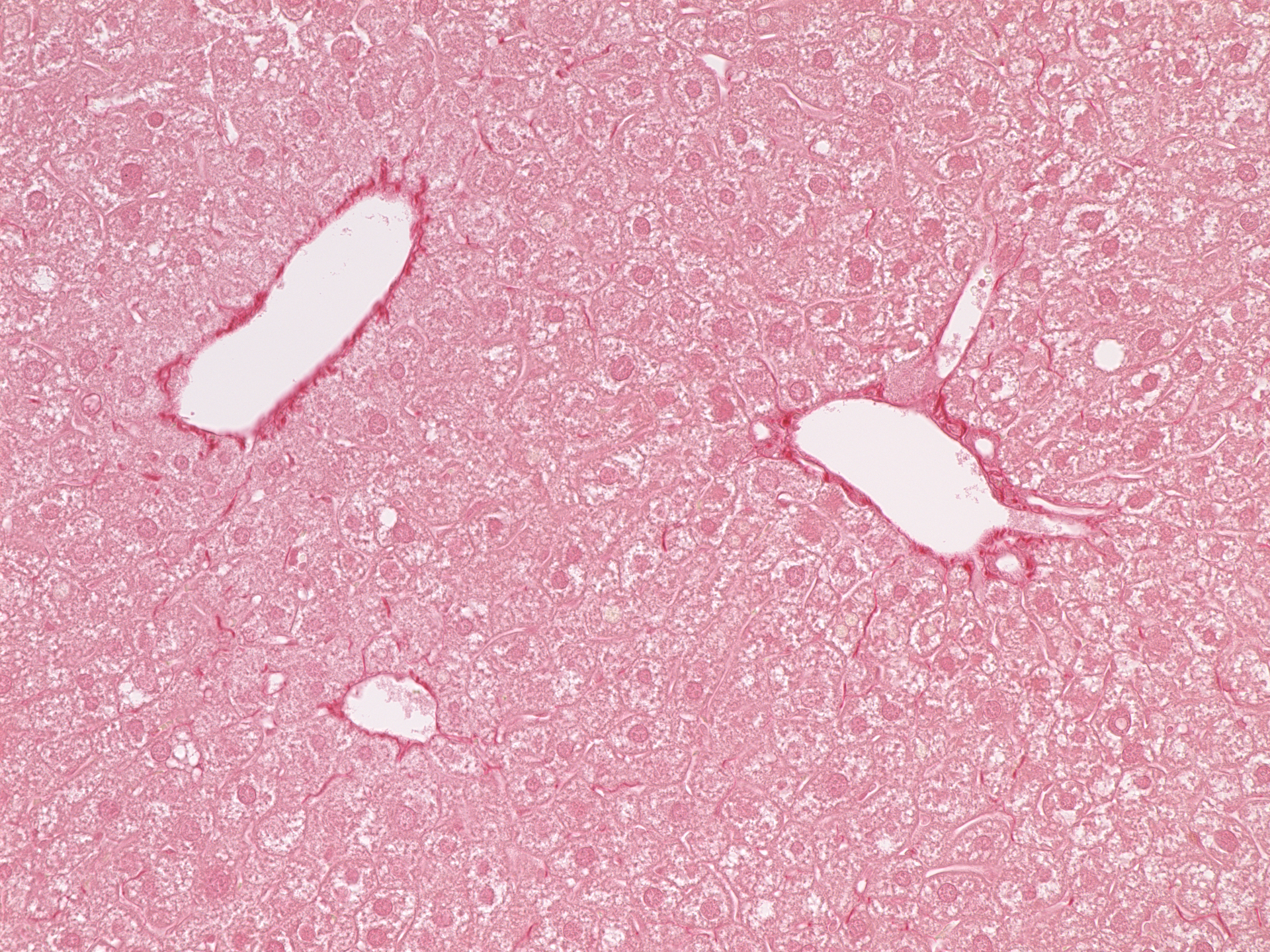

Supplement: Supplementary file 4 — Source data Fig. 2 [file 44318_2024_196_MOESM4_ESM.zip › Figure 2/Figure 2-N/Demonstrated image/NC Mock/NC-Mock-20x.tif]

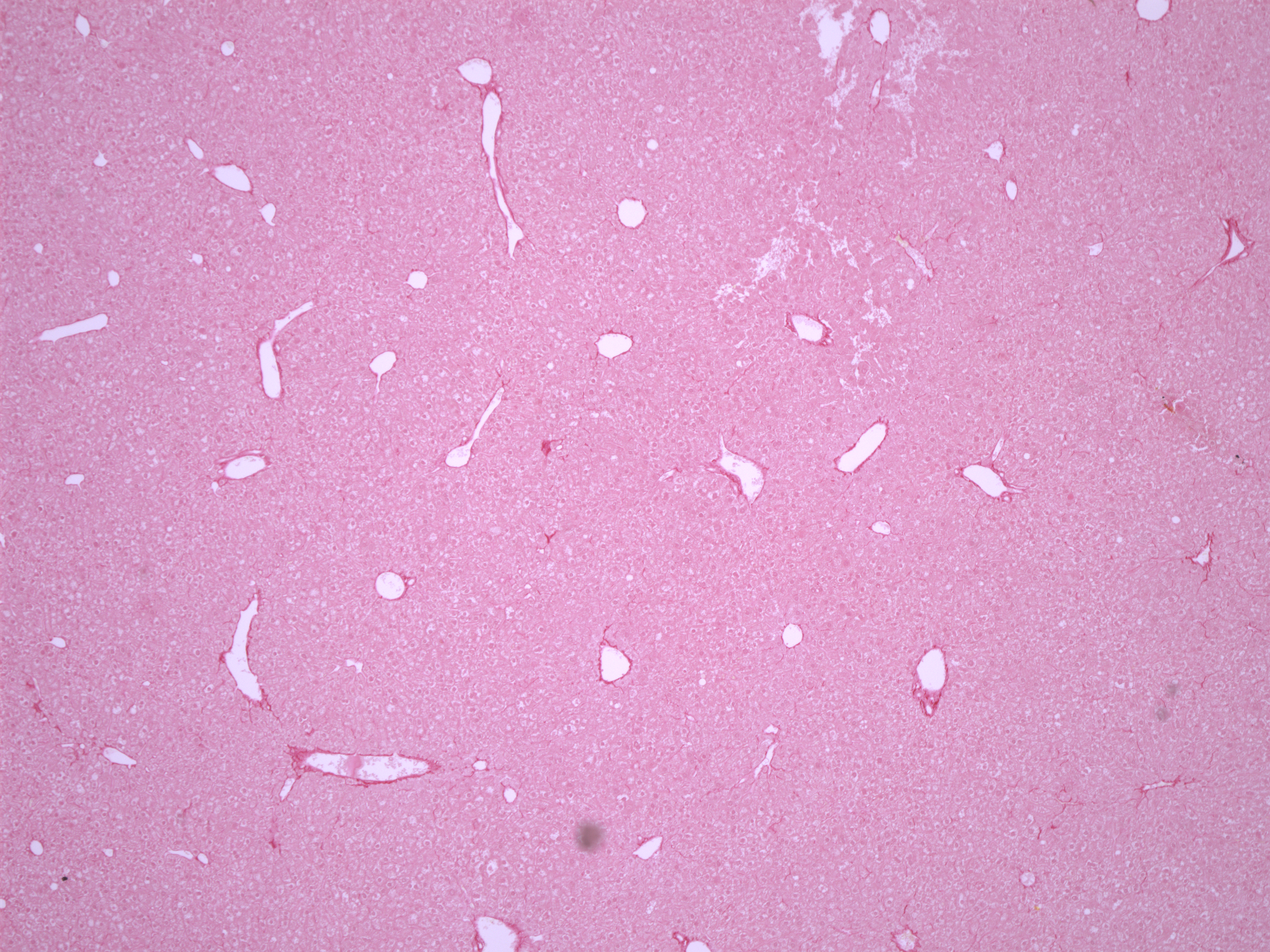

Supplement: Supplementary file 4 — Source data Fig. 2 [file 44318_2024_196_MOESM4_ESM.zip › Figure 2/Figure 2-N/Demonstrated image/NC Mock/NC-Mock-4x.tif]

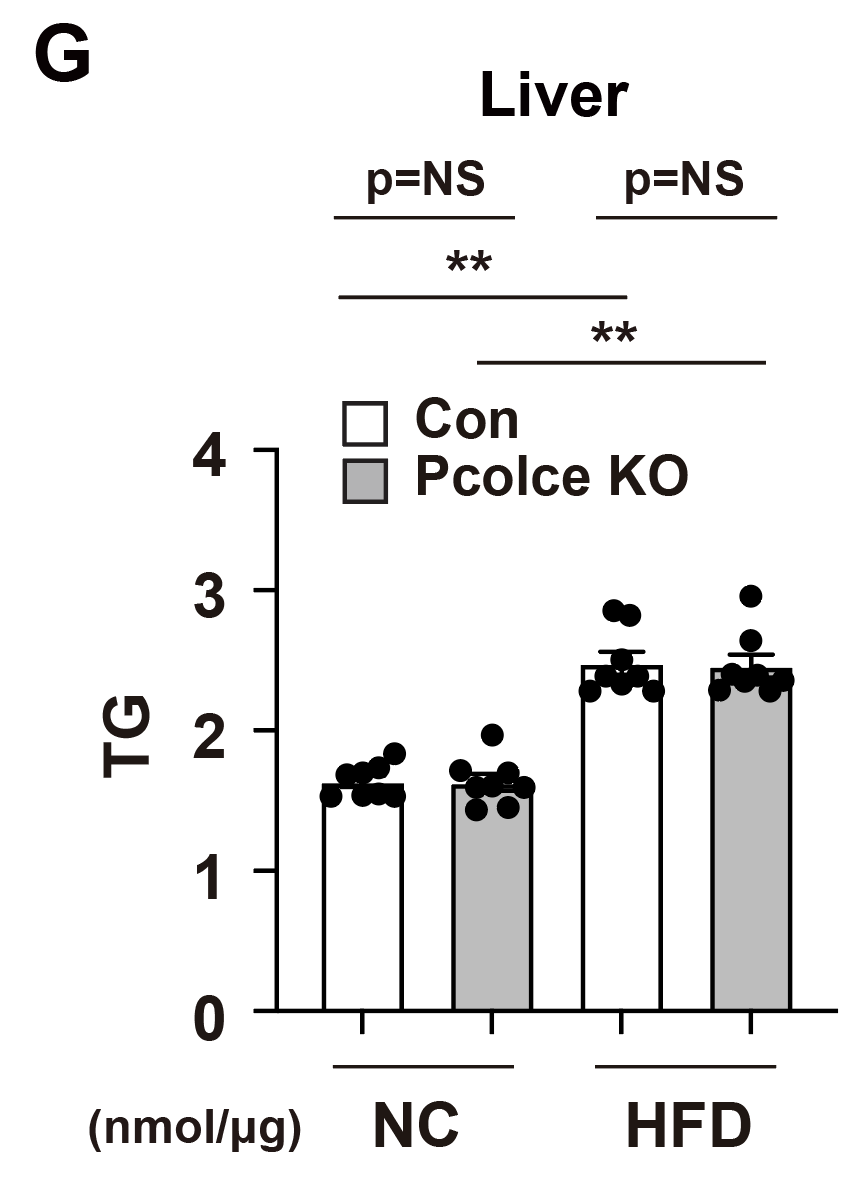

Supplement: Supplementary file 5 — Source data Fig. 3 [file 44318_2024_196_MOESM5_ESM.zip › Figure 3/Figure 3-G/Fig.3G.png]

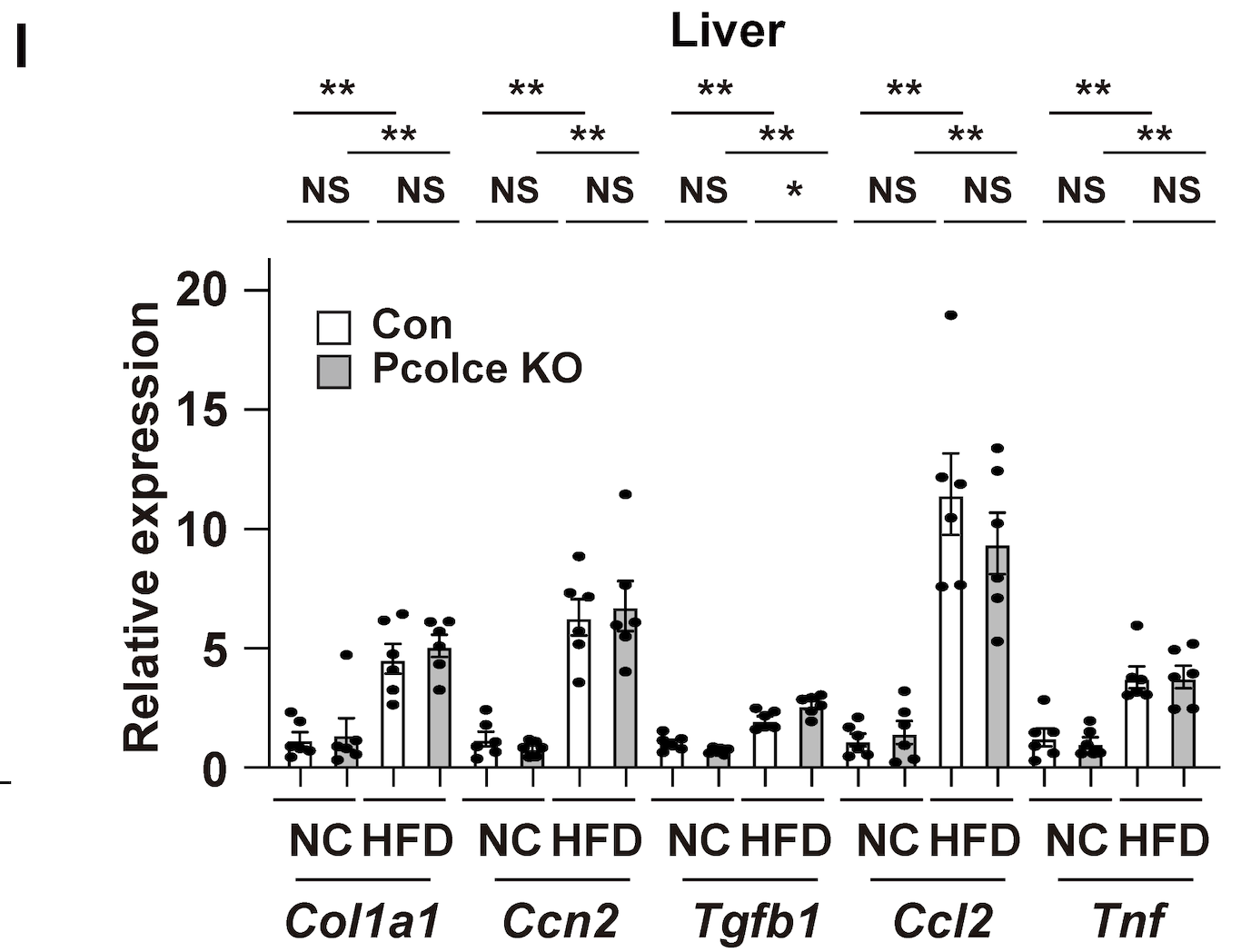

Supplement: Supplementary file 5 — Source data Fig. 3 [file 44318_2024_196_MOESM5_ESM.zip › Figure 3/Figure 3-I/Fig.3I.png]

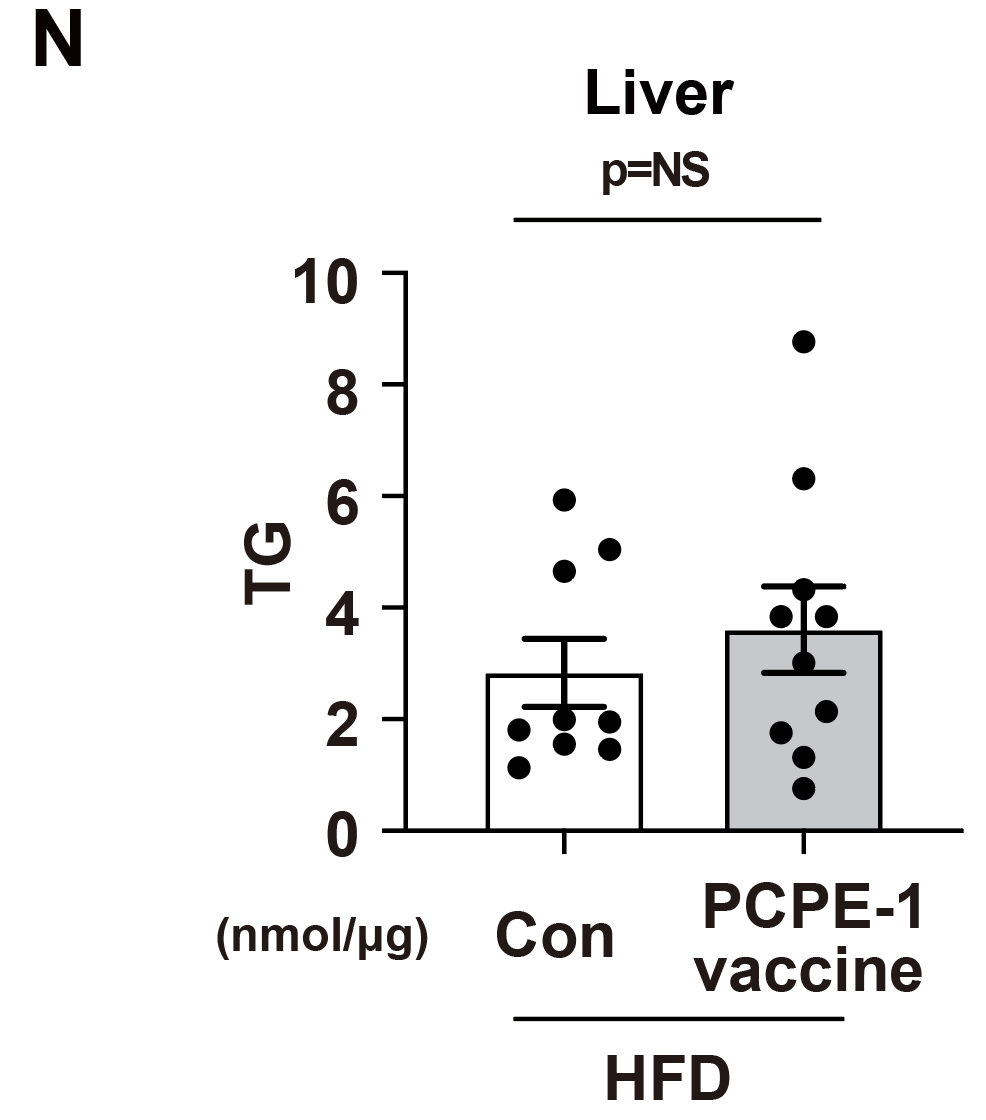

Supplement: Supplementary file 5 — Source data Fig. 3 [file 44318_2024_196_MOESM5_ESM.zip › Figure 3/Figure 3-N/Fig.3N.png]

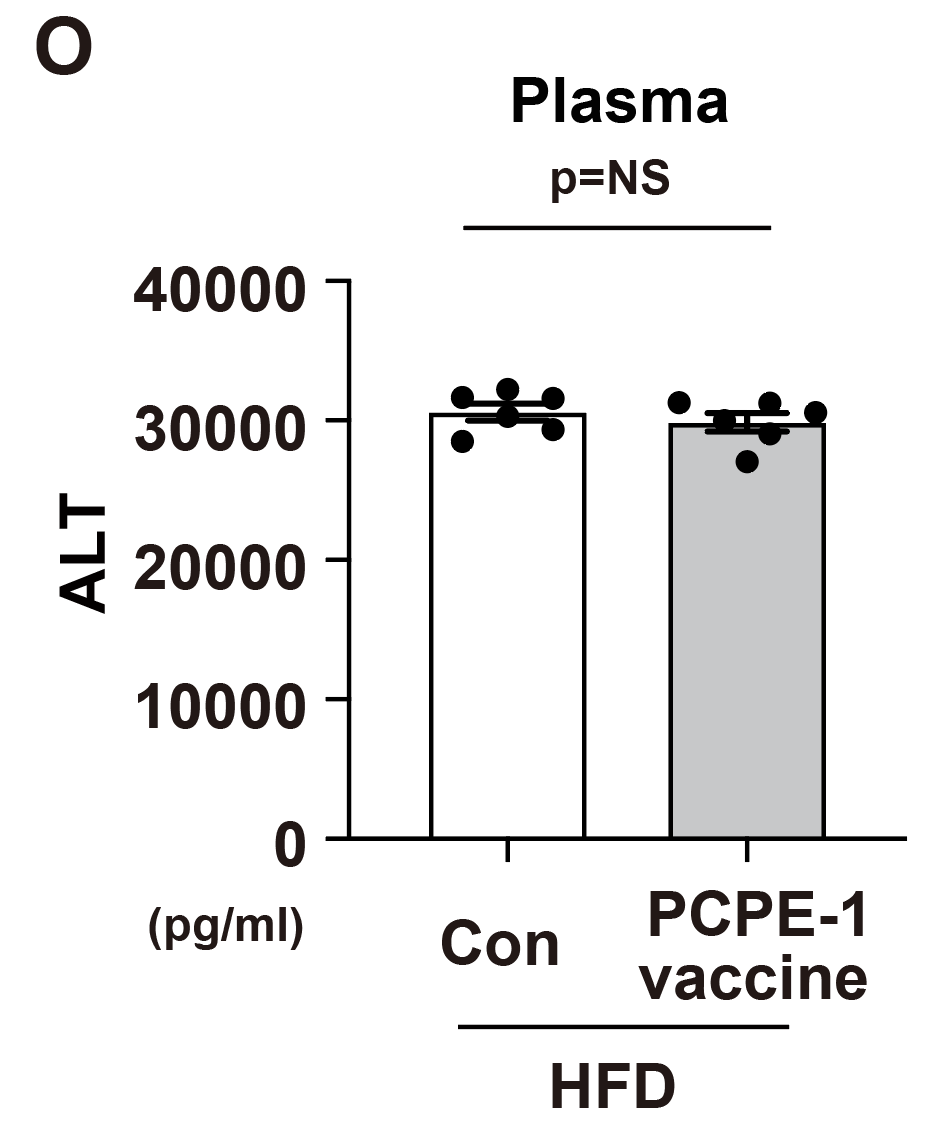

Supplement: Supplementary file 5 — Source data Fig. 3 [file 44318_2024_196_MOESM5_ESM.zip › Figure 3/Figure 3-O/Fig.3O.png]

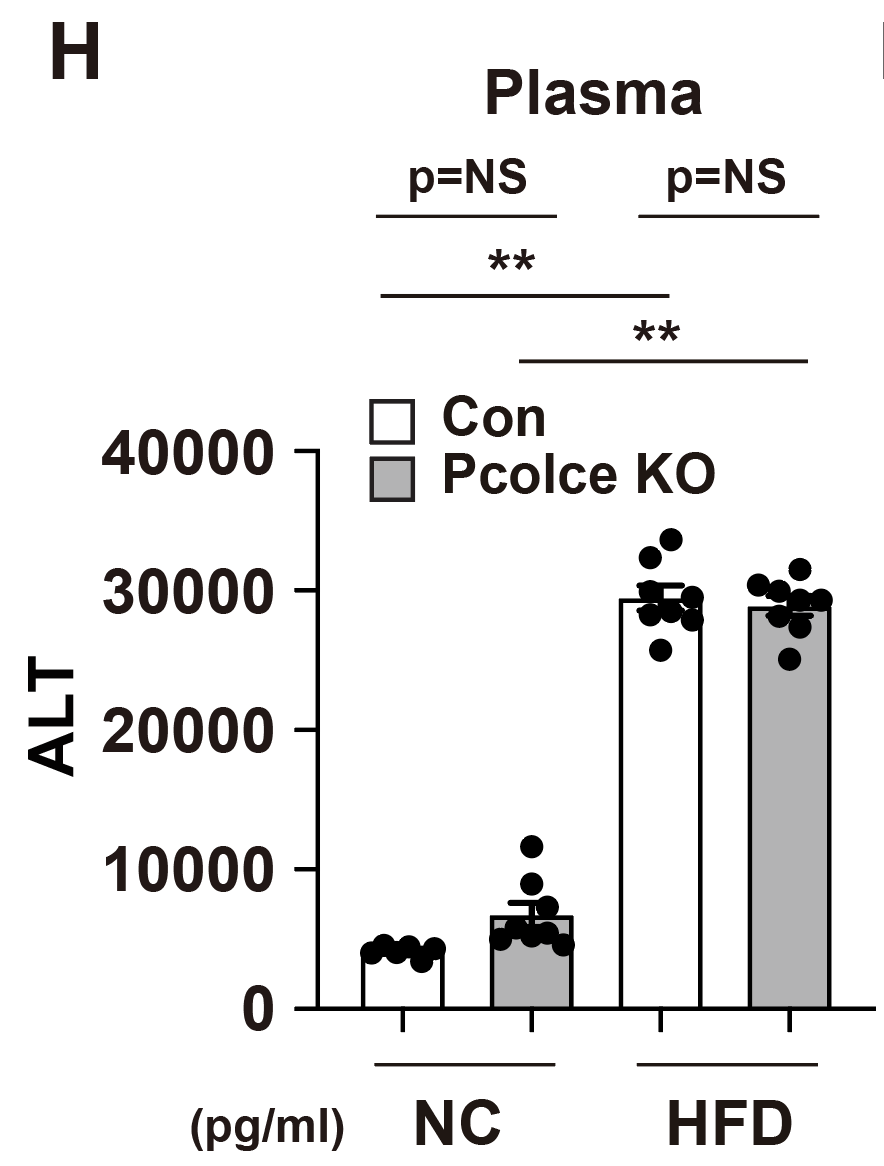

Supplement: Supplementary file 5 — Source data Fig. 3 [file 44318_2024_196_MOESM5_ESM.zip › Figure 3/Figure 3-H/Fig.3H.png]

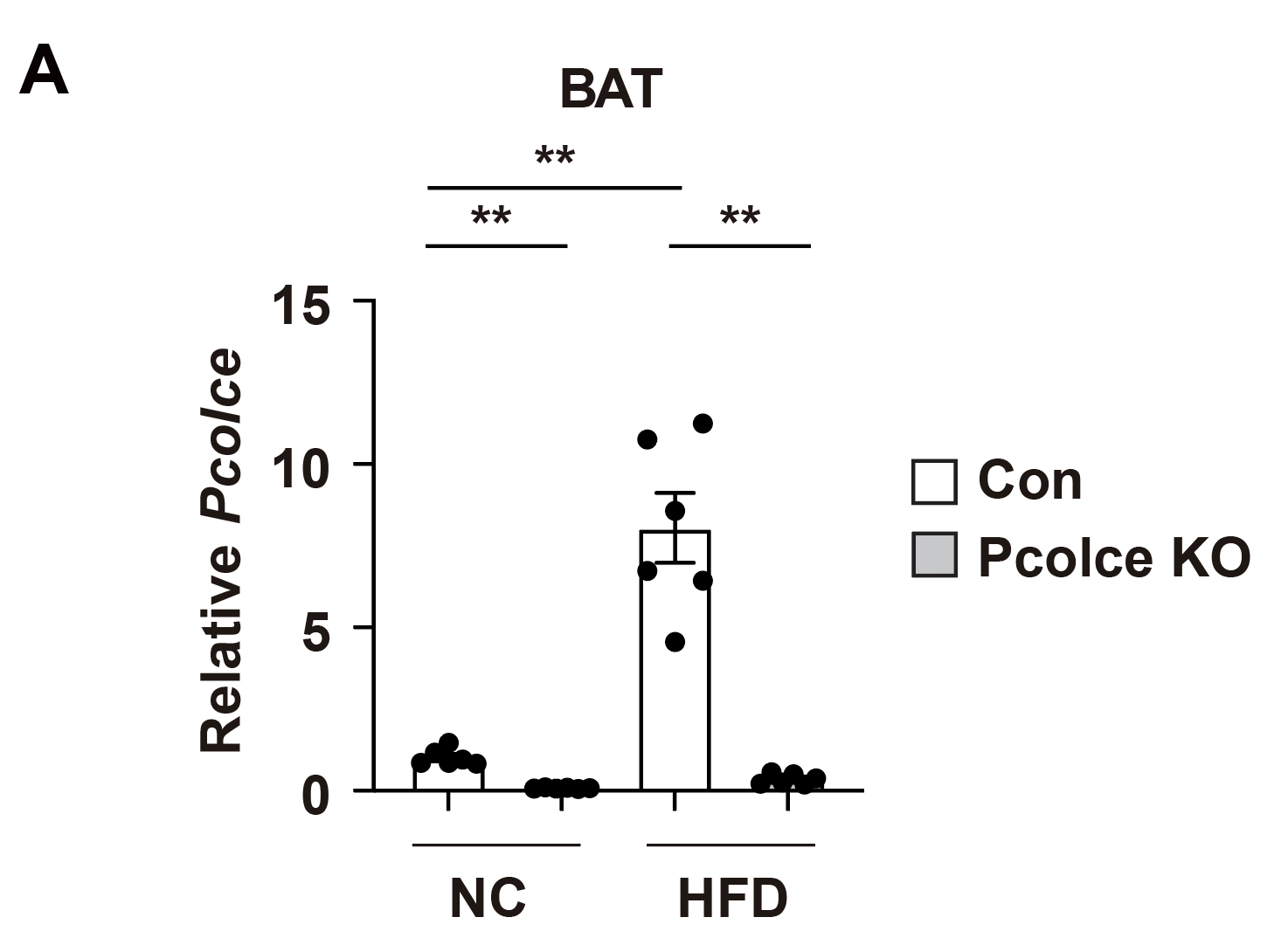

Supplement: Supplementary file 5 — Source data Fig. 3 [file 44318_2024_196_MOESM5_ESM.zip › Figure 3/Figure 3-A/Fig.3A.png]

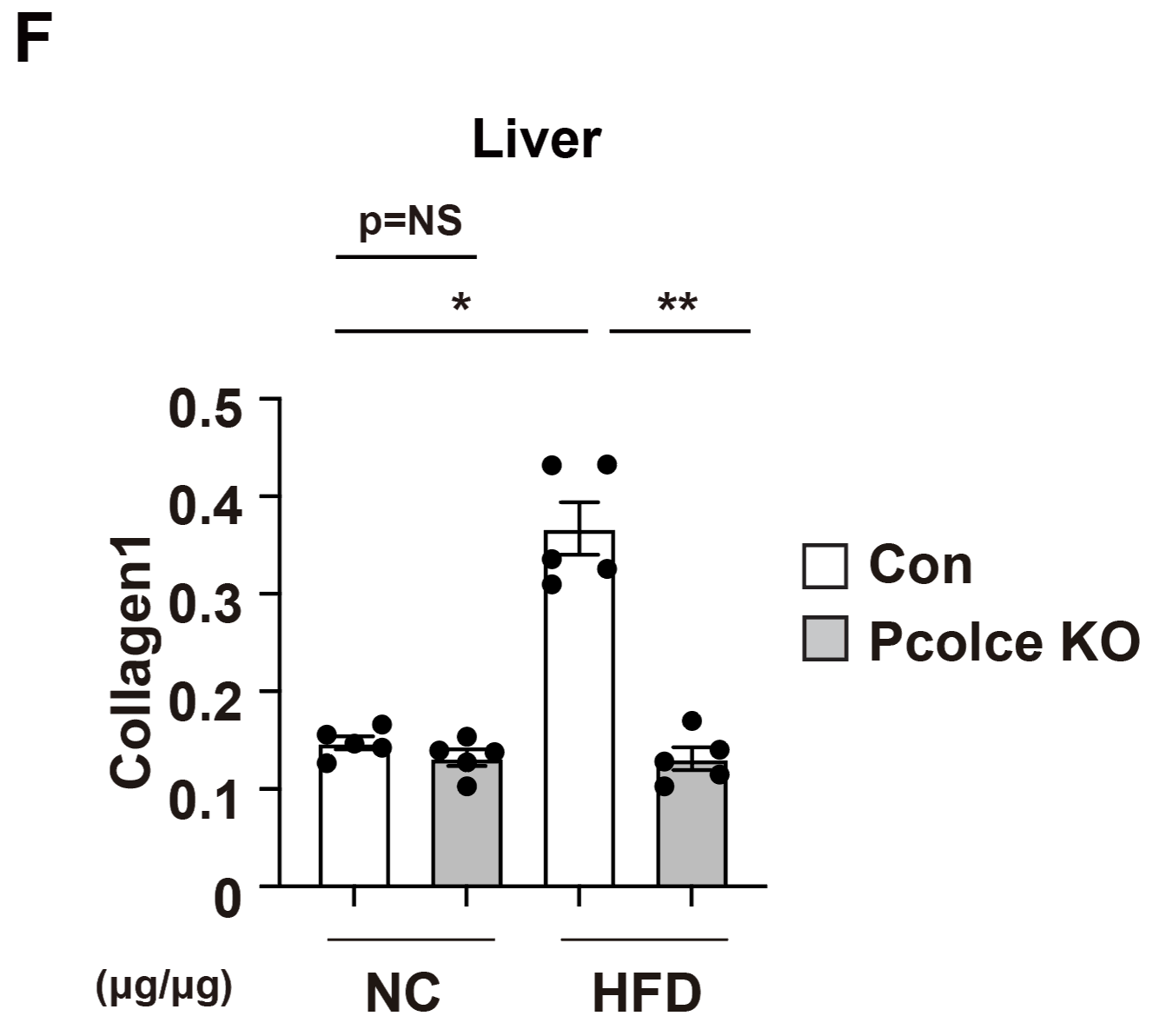

Supplement: Supplementary file 5 — Source data Fig. 3 [file 44318_2024_196_MOESM5_ESM.zip › Figure 3/Figure 3-F/Fig.3F.png]

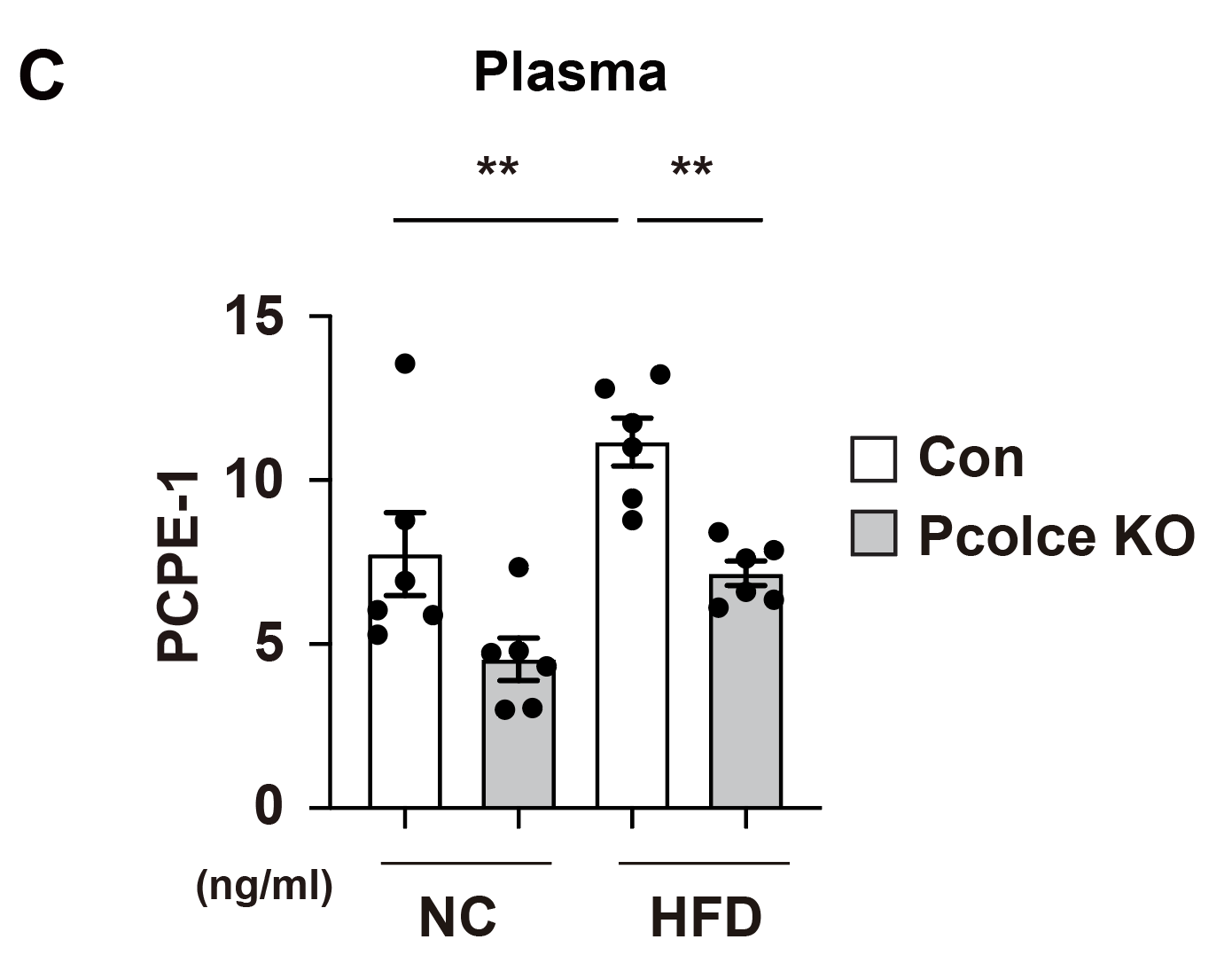

Supplement: Supplementary file 5 — Source data Fig. 3 [file 44318_2024_196_MOESM5_ESM.zip › Figure 3/Figure 3-C/Fig.3C.png]

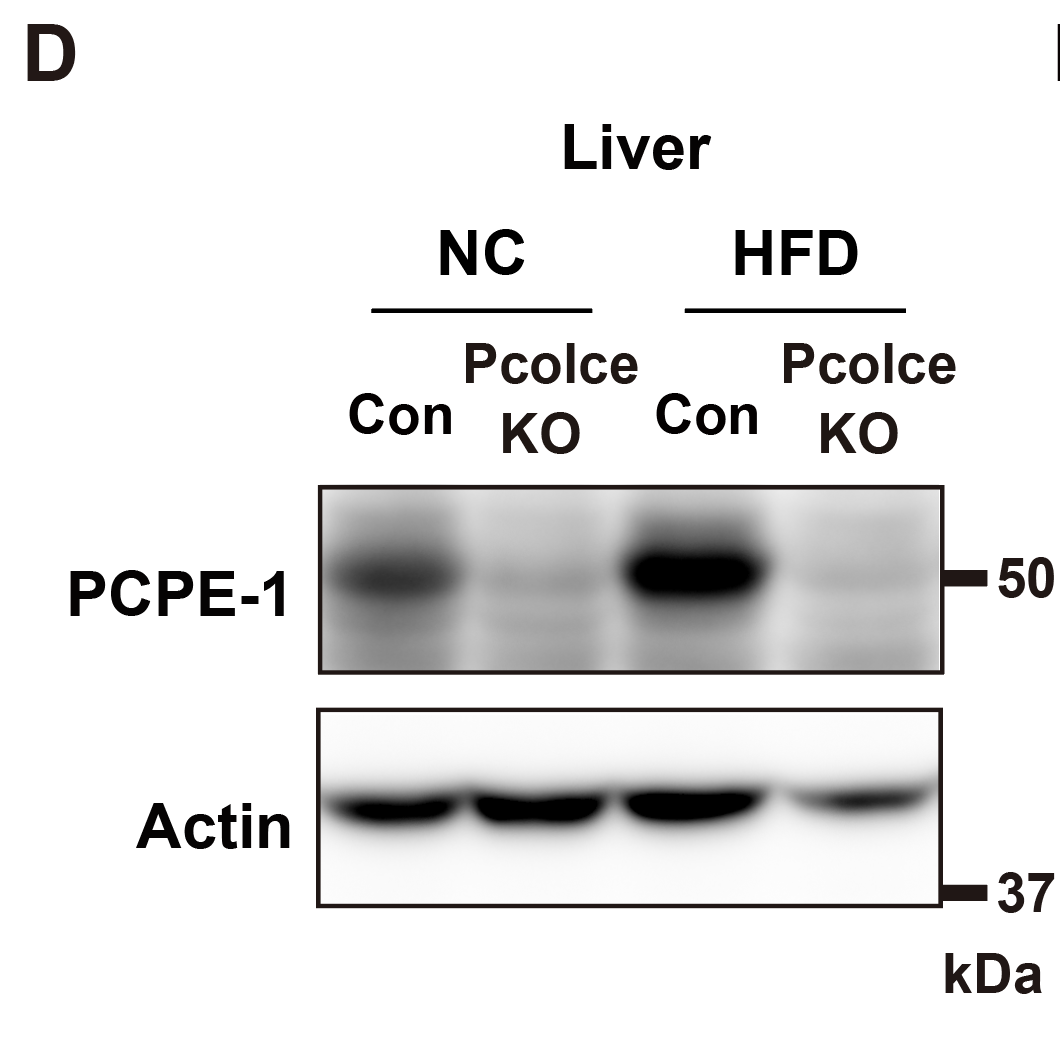

Supplement: Supplementary file 5 — Source data Fig. 3 [file 44318_2024_196_MOESM5_ESM.zip › Figure 3/Figure 3-D/Fig.3D.png]

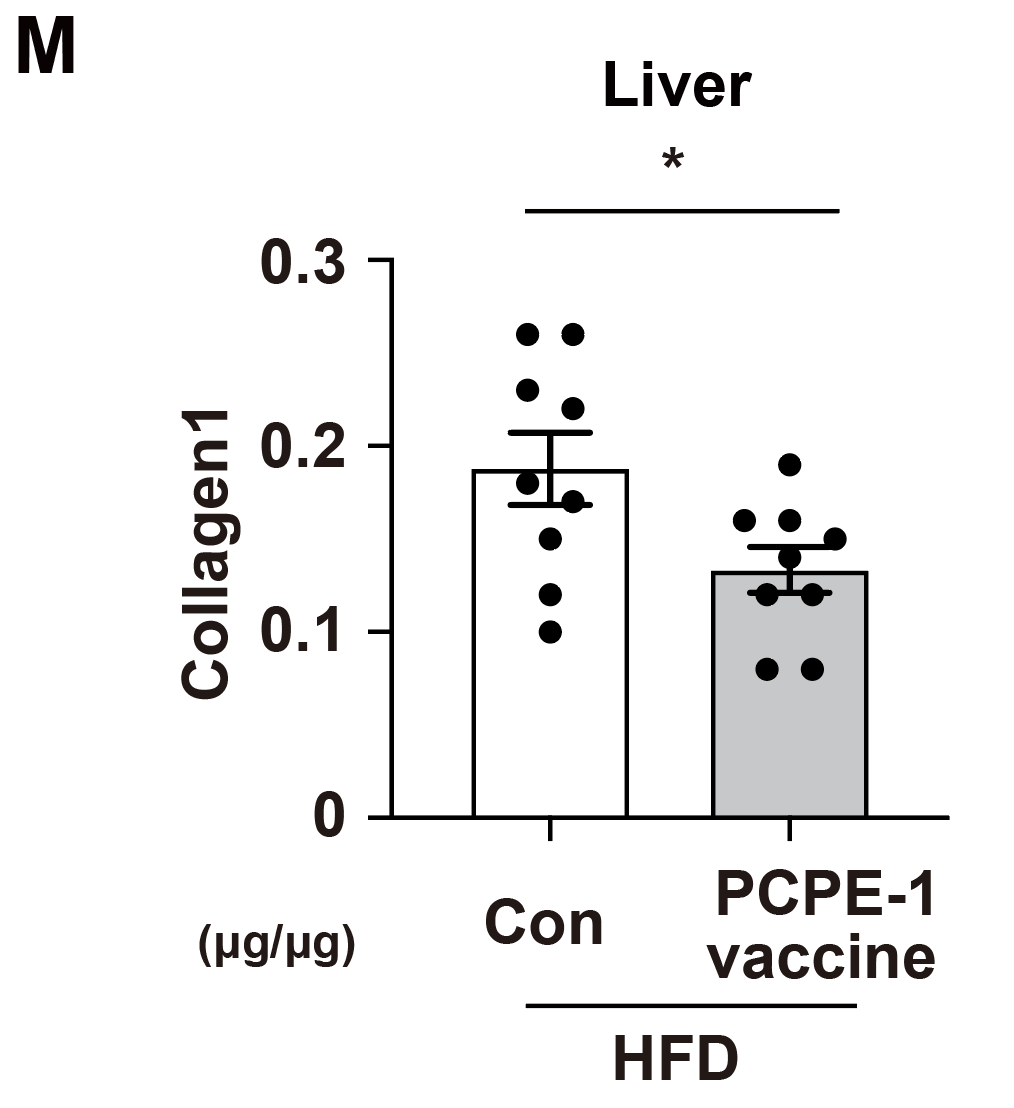

Supplement: Supplementary file 5 — Source data Fig. 3 [file 44318_2024_196_MOESM5_ESM.zip › Figure 3/Figure 3-M/Fig.3M.png]

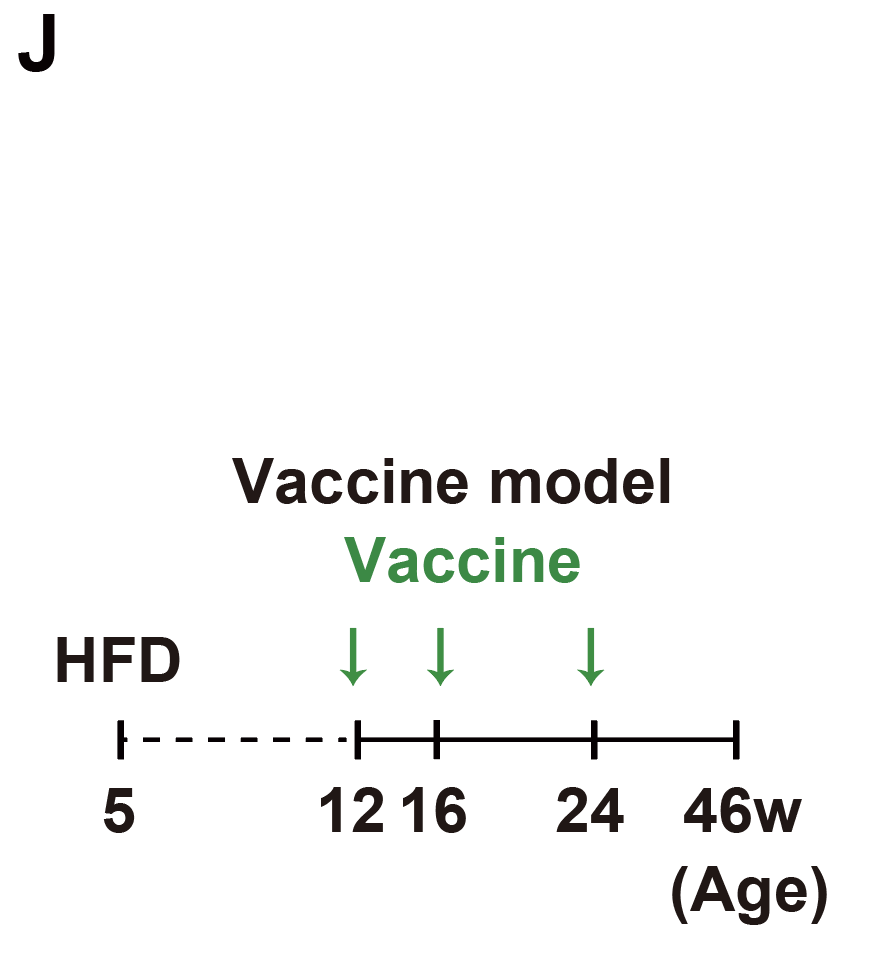

Supplement: Supplementary file 5 — Source data Fig. 3 [file 44318_2024_196_MOESM5_ESM.zip › Figure 3/Figure 3-J/Fig.3J.png]

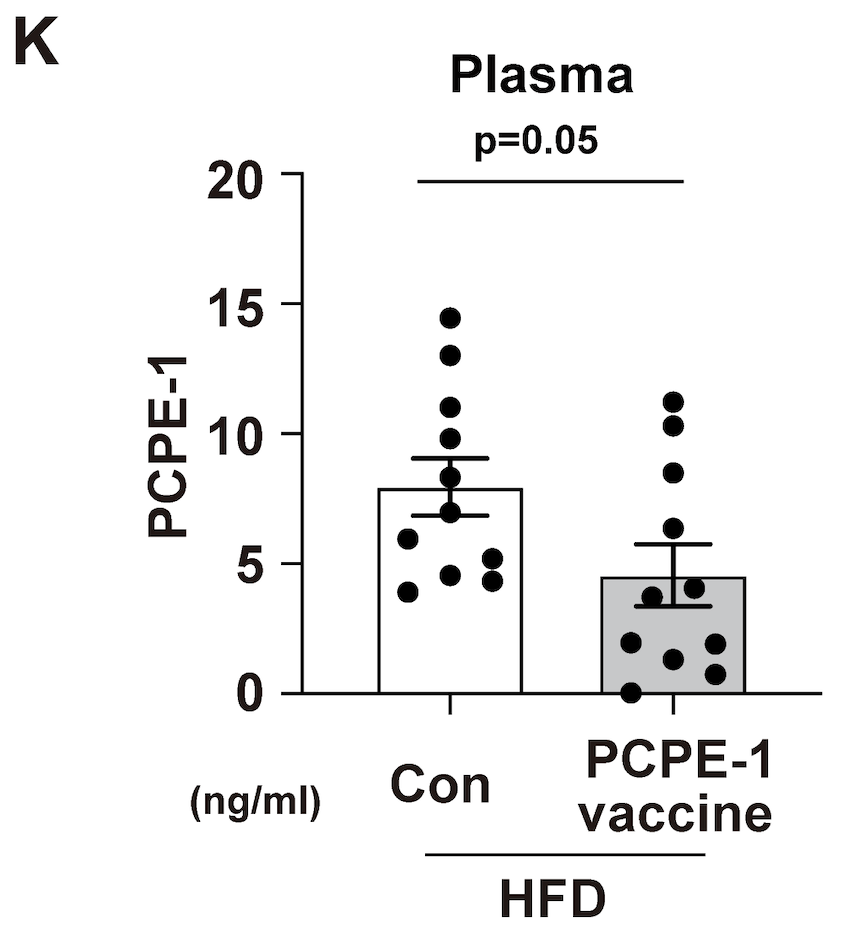

Supplement: Supplementary file 5 — Source data Fig. 3 [file 44318_2024_196_MOESM5_ESM.zip › Figure 3/Figure 3-K/Fig.3K.png]

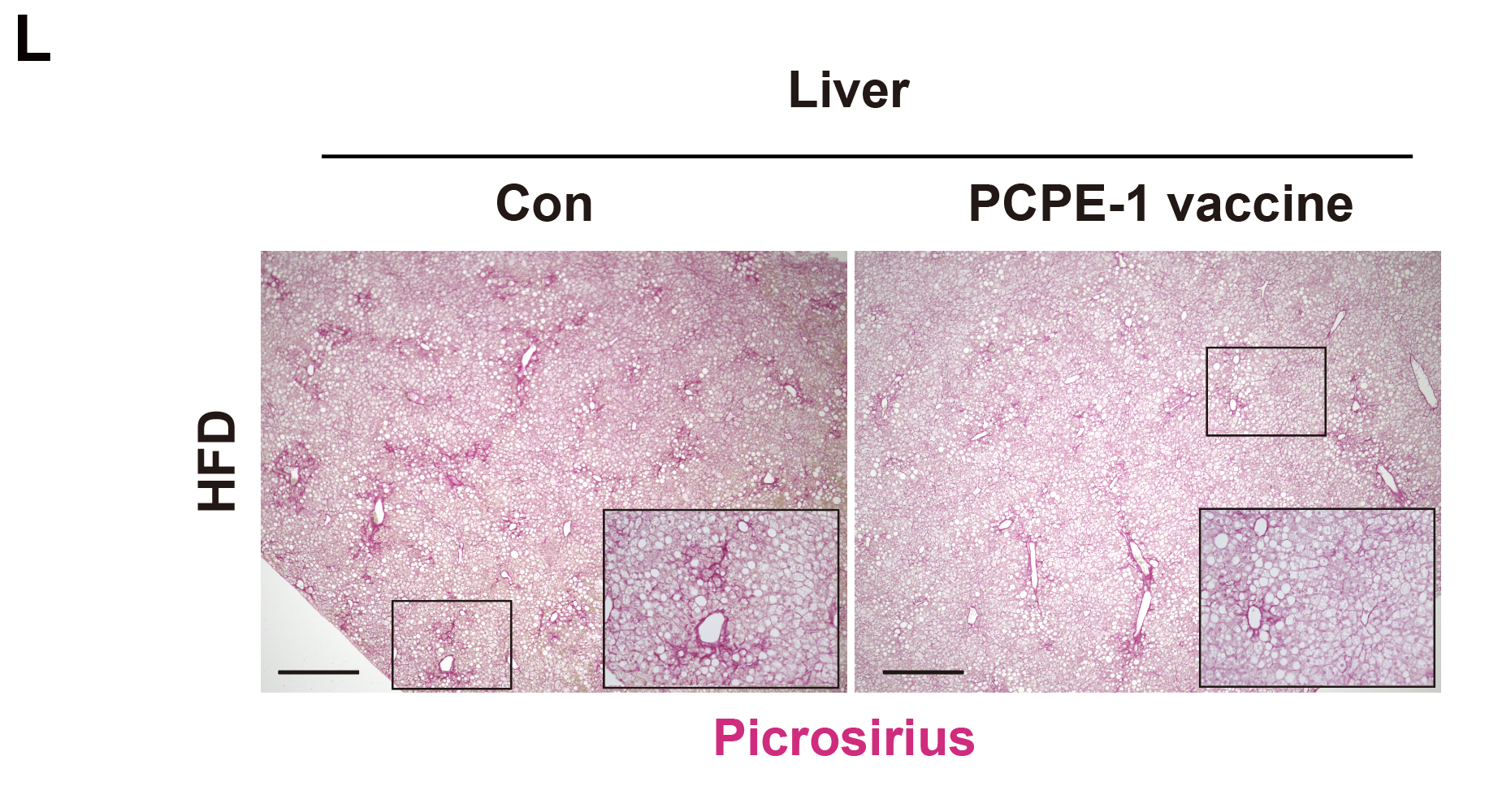

Supplement: Supplementary file 5 — Source data Fig. 3 [file 44318_2024_196_MOESM5_ESM.zip › Figure 3/Figure 3-L/Fig.3L.png]

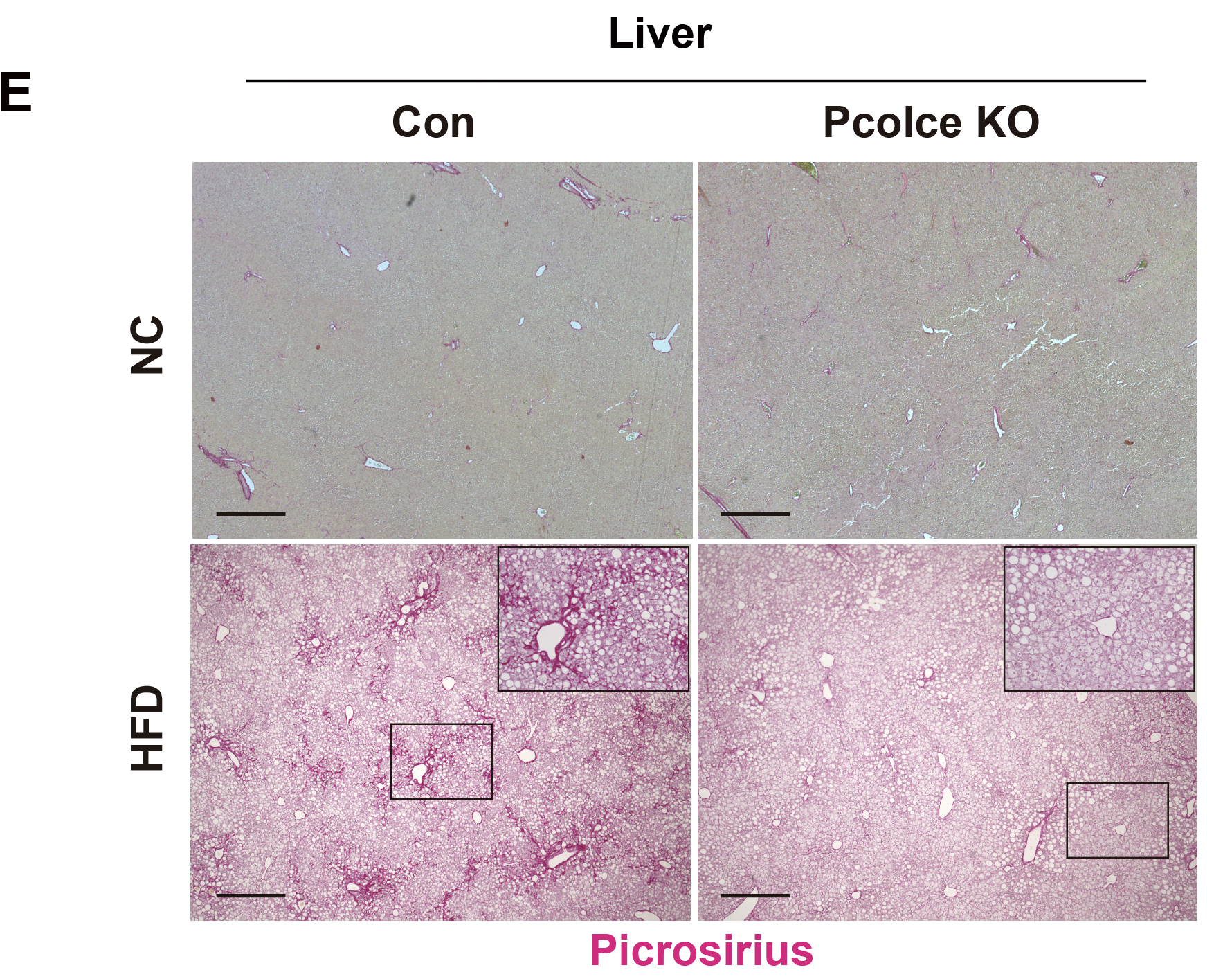

Supplement: Supplementary file 5 — Source data Fig. 3 [file 44318_2024_196_MOESM5_ESM.zip › Figure 3/Figure 3-E/Fig.3E.png]

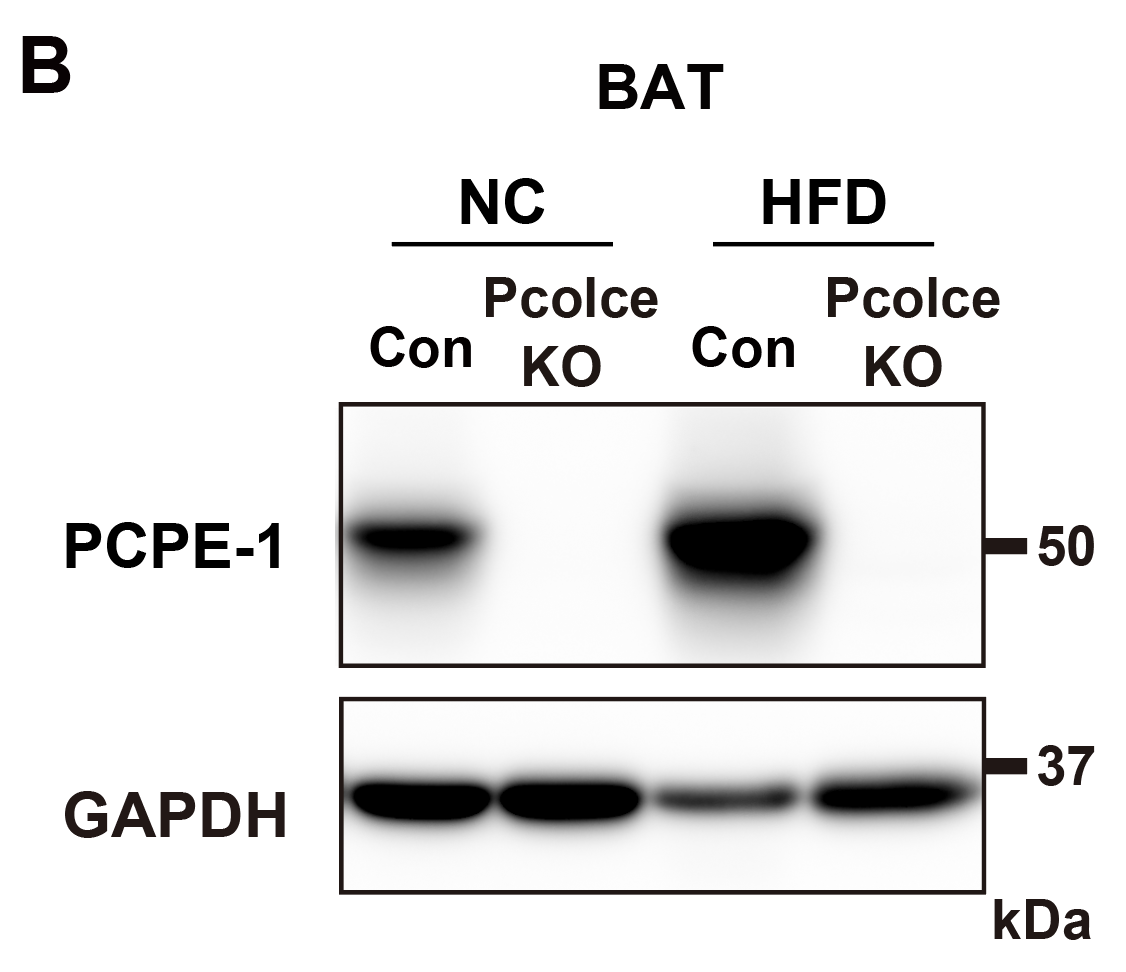

Supplement: Supplementary file 5 — Source data Fig. 3 [file 44318_2024_196_MOESM5_ESM.zip › Figure 3/Figure 3-B/Fig.3B.png]

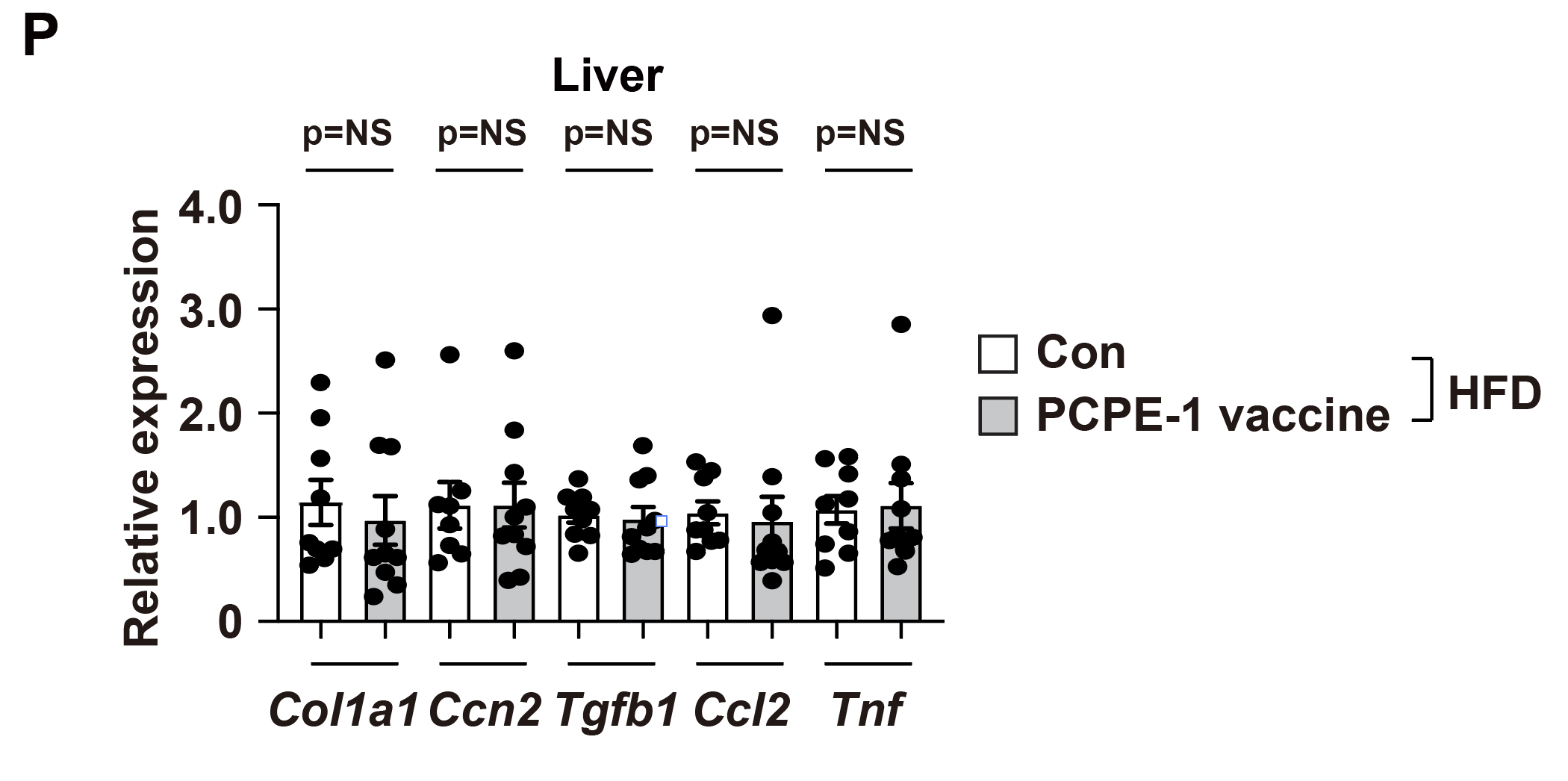

Supplement: Supplementary file 5 — Source data Fig. 3 [file 44318_2024_196_MOESM5_ESM.zip › Figure 3/Figure 3-P/Fig.3P.png]

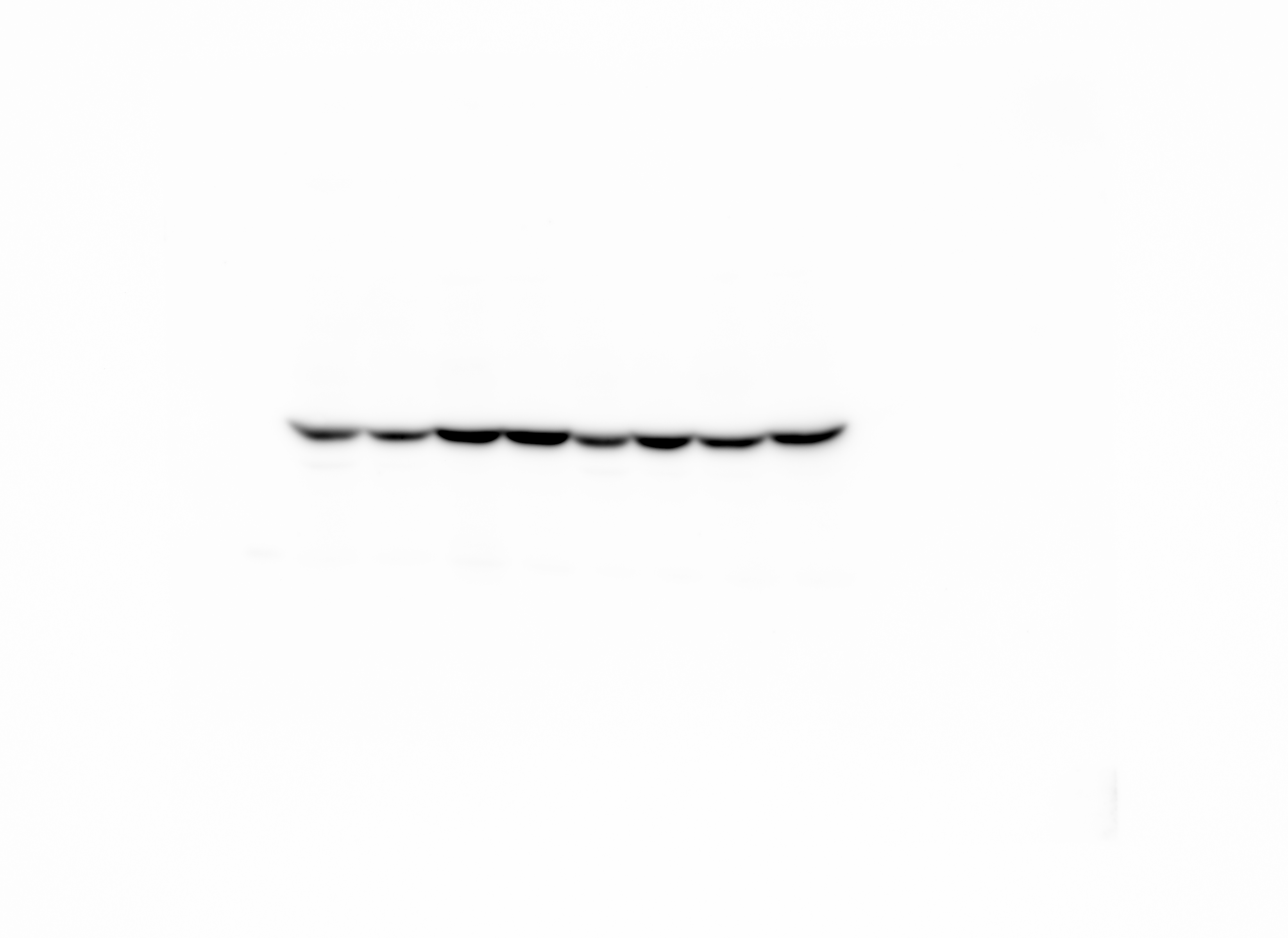

Supplement: Supplementary file 5 — Source data Fig. 3 [file 44318_2024_196_MOESM5_ESM.zip › Figure 3/Figure 3-D/Quantificated image/Actin membrane 2.tif]

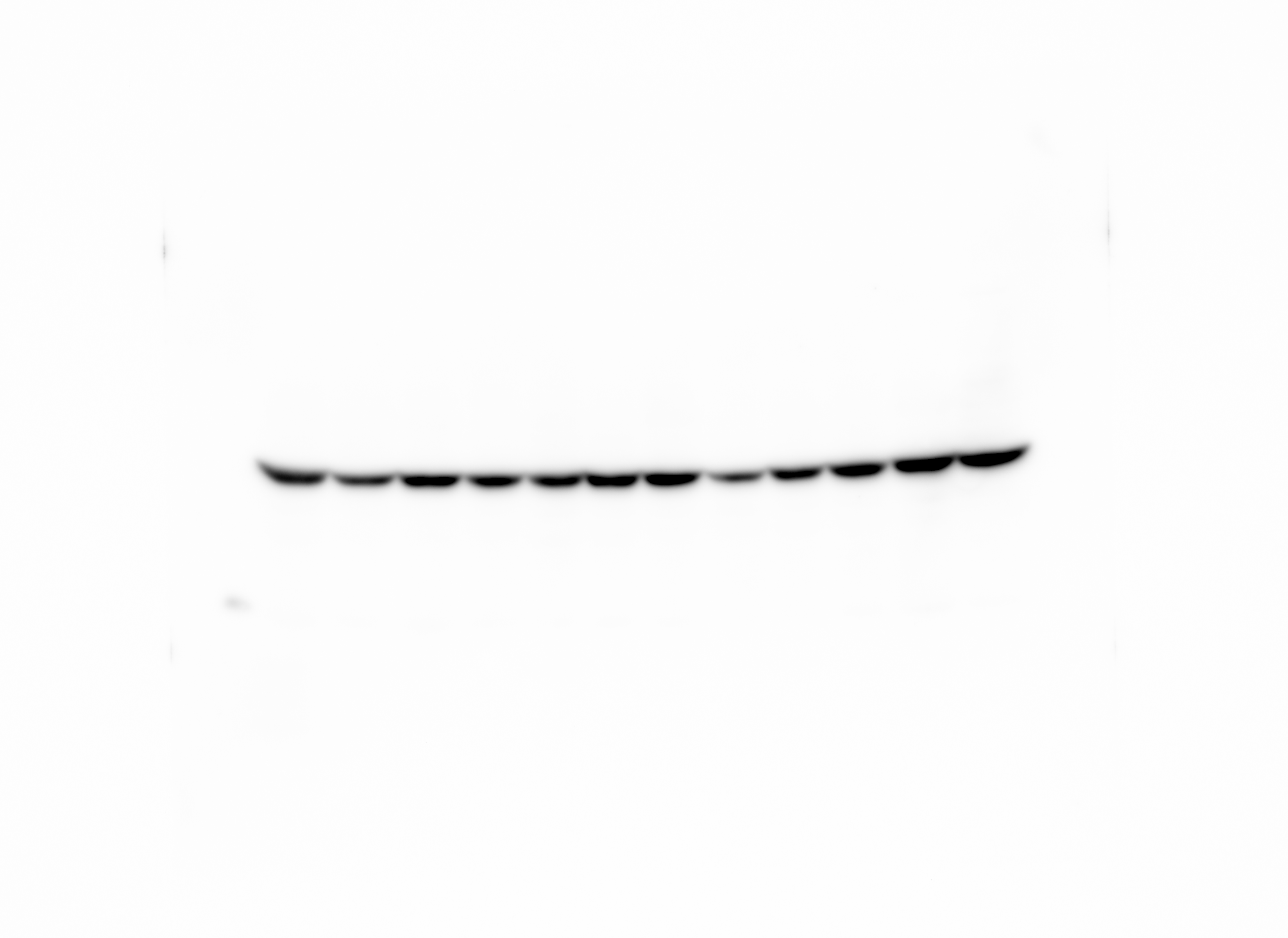

Supplement: Supplementary file 5 — Source data Fig. 3 [file 44318_2024_196_MOESM5_ESM.zip › Figure 3/Figure 3-D/Quantificated image/Actin membrane 1.tif]

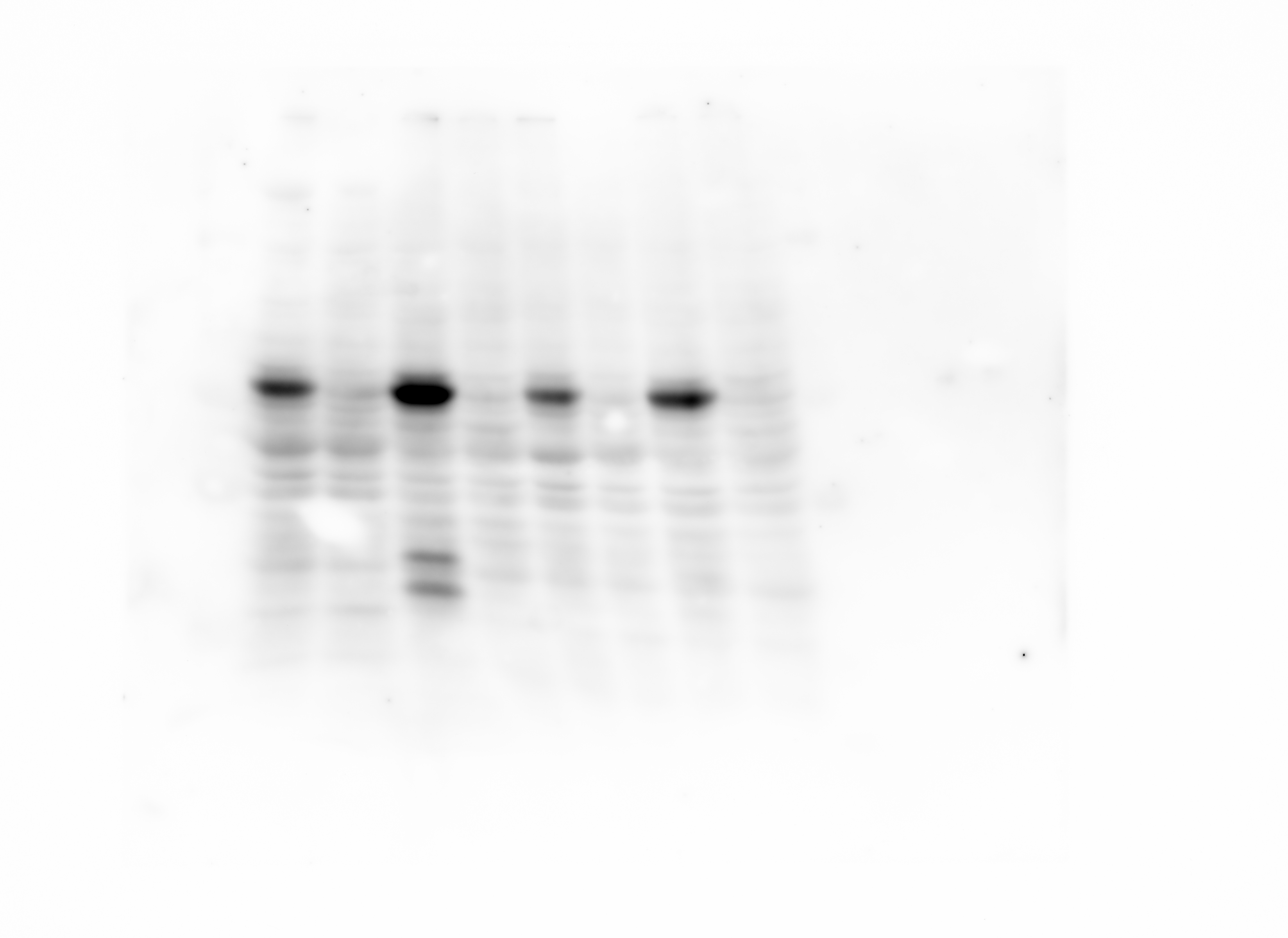

Supplement: Supplementary file 5 — Source data Fig. 3 [file 44318_2024_196_MOESM5_ESM.zip › Figure 3/Figure 3-D/Quantificated image/PCPE-1 membrane 2.tif]

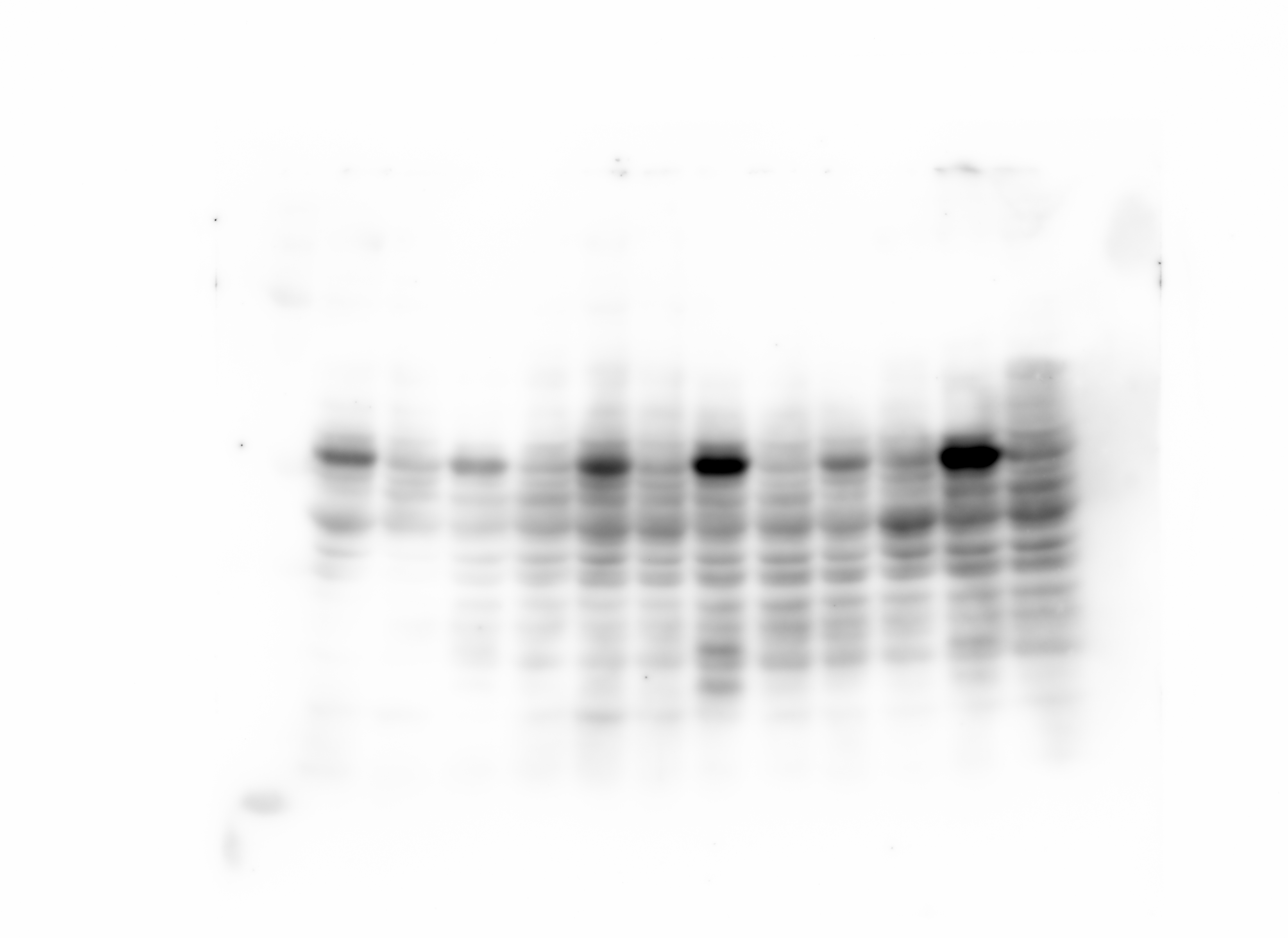

Supplement: Supplementary file 5 — Source data Fig. 3 [file 44318_2024_196_MOESM5_ESM.zip › Figure 3/Figure 3-D/Quantificated image/PCPE-1 membrane 1.tif]

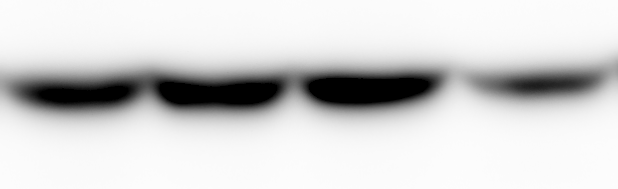

Supplement: Supplementary file 5 — Source data Fig. 3 [file 44318_2024_196_MOESM5_ESM.zip › Figure 3/Figure 3-D/Demonstrated image/Actin.tif]

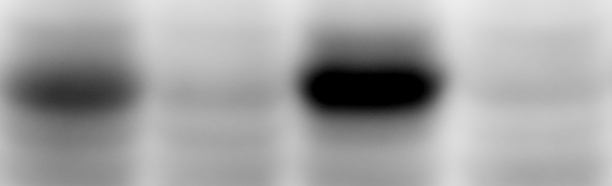

Supplement: Supplementary file 5 — Source data Fig. 3 [file 44318_2024_196_MOESM5_ESM.zip › Figure 3/Figure 3-D/Demonstrated image/PCPE-1.tif]

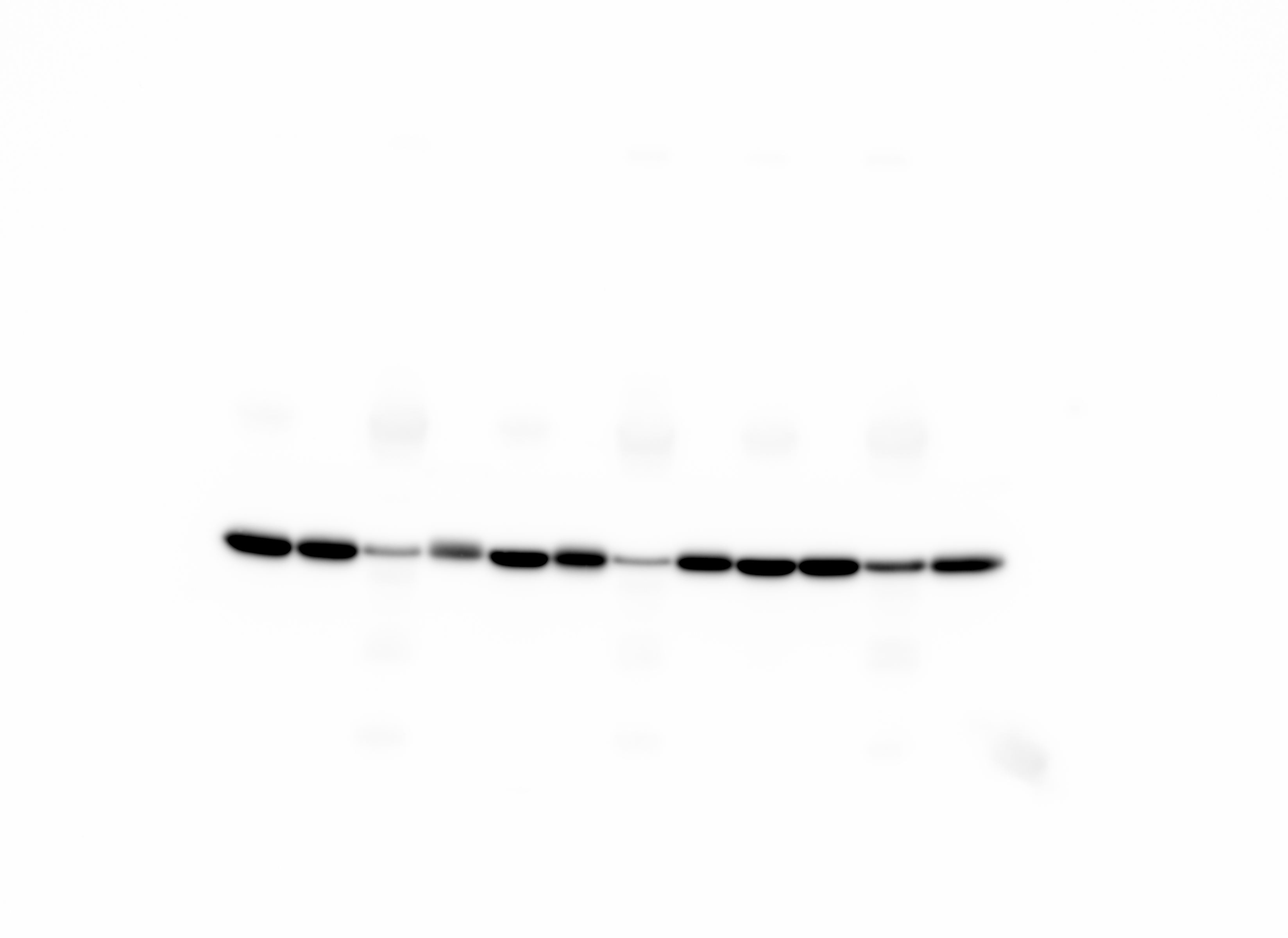

Supplement: Supplementary file 5 — Source data Fig. 3 [file 44318_2024_196_MOESM5_ESM.zip › Figure 3/Figure 3-B/Quantificated image/GAPDH membrane 1.tif]

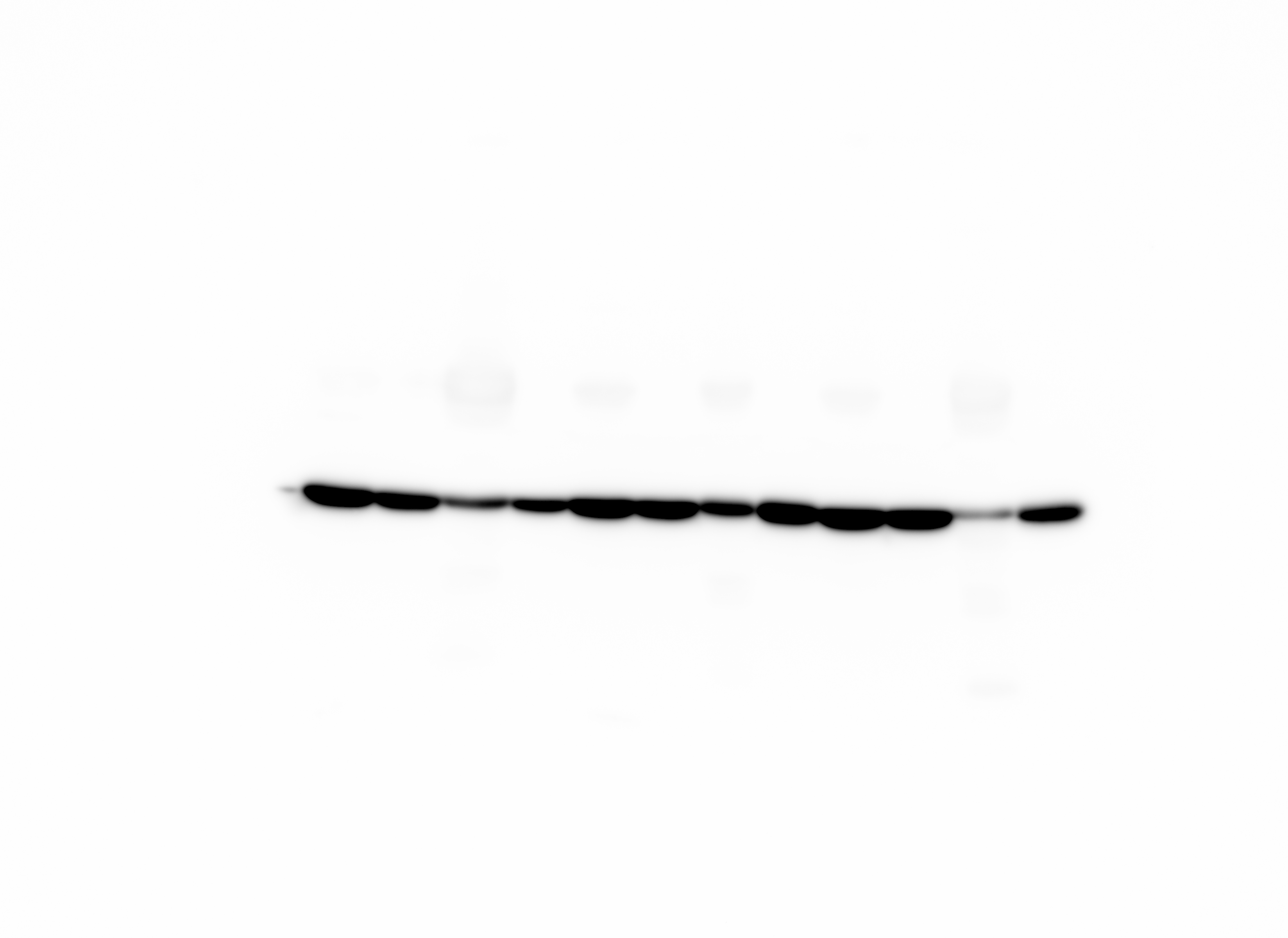

Supplement: Supplementary file 5 — Source data Fig. 3 [file 44318_2024_196_MOESM5_ESM.zip › Figure 3/Figure 3-B/Quantificated image/GAPDH membrane 2.tif]

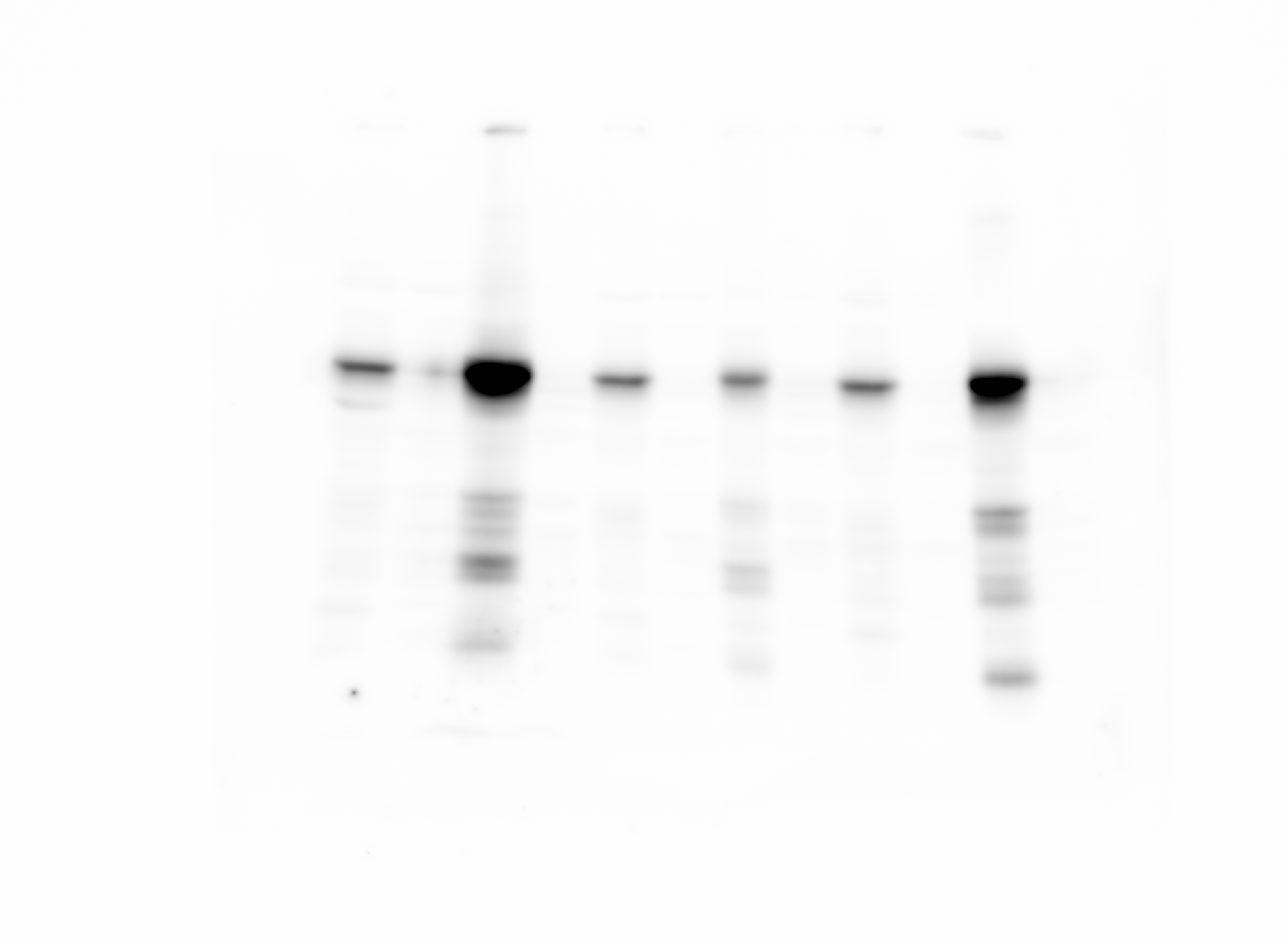

Supplement: Supplementary file 5 — Source data Fig. 3 [file 44318_2024_196_MOESM5_ESM.zip › Figure 3/Figure 3-B/Quantificated image/PCPE-1 membrane 2.tif]

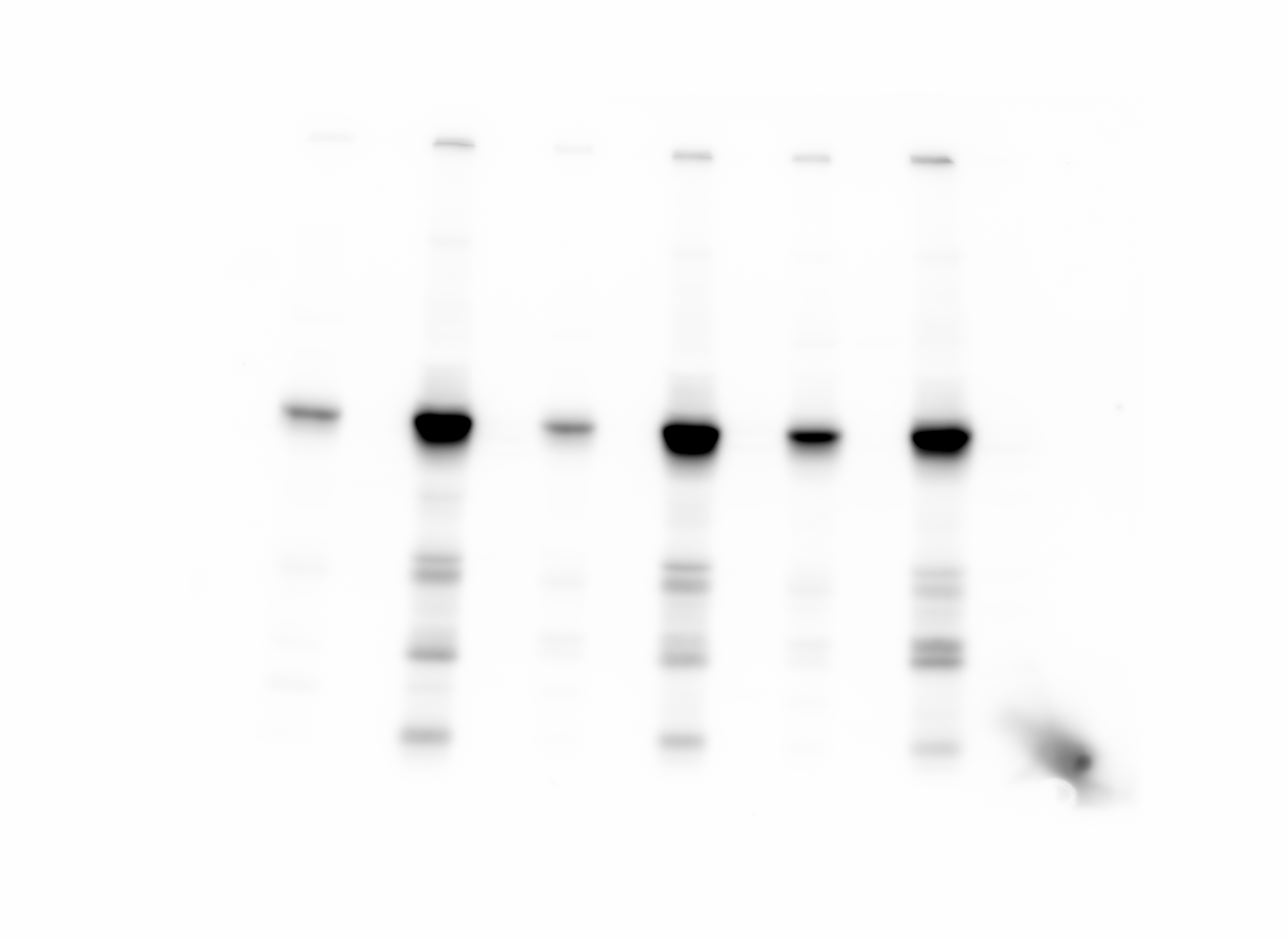

Supplement: Supplementary file 5 — Source data Fig. 3 [file 44318_2024_196_MOESM5_ESM.zip › Figure 3/Figure 3-B/Quantificated image/PCPE-1 membrane 1.tif]

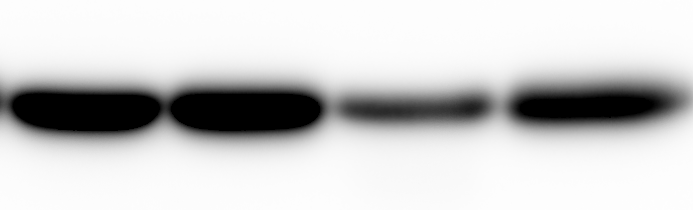

Supplement: Supplementary file 5 — Source data Fig. 3 [file 44318_2024_196_MOESM5_ESM.zip › Figure 3/Figure 3-B/Demonstrated image/GAPDH.tif]

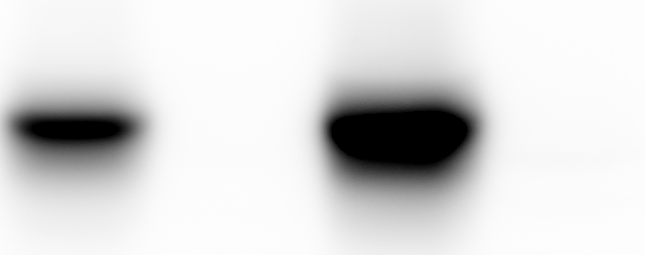

Supplement: Supplementary file 5 — Source data Fig. 3 [file 44318_2024_196_MOESM5_ESM.zip › Figure 3/Figure 3-B/Demonstrated image/PCPE-1.tif]

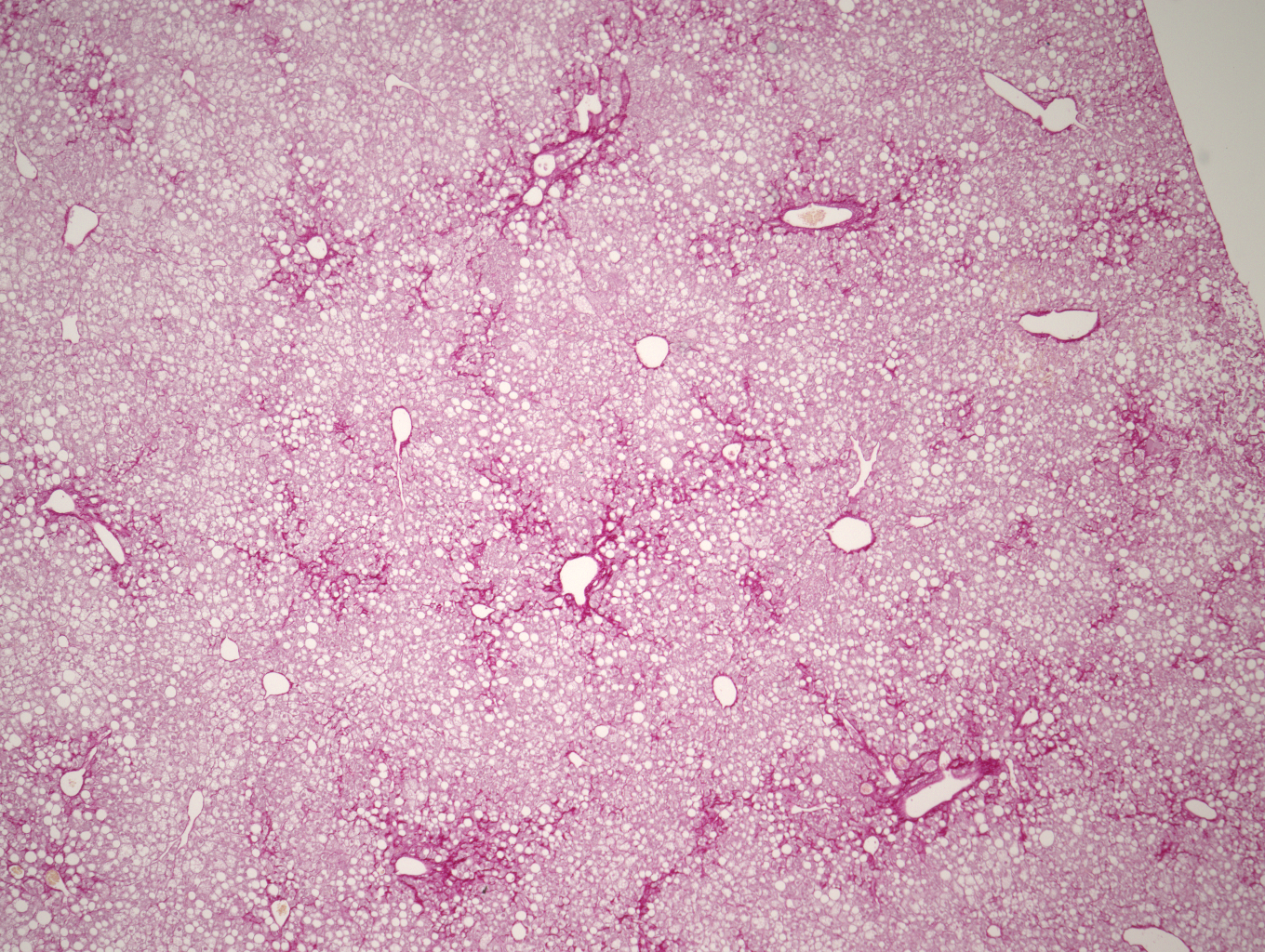

Supplement: Supplementary file 5 — Source data Fig. 3 [file 44318_2024_196_MOESM5_ESM.zip › Figure 3/Figure 3-E/Demonstrated image/HFD Con/HFD Con x4.tif]

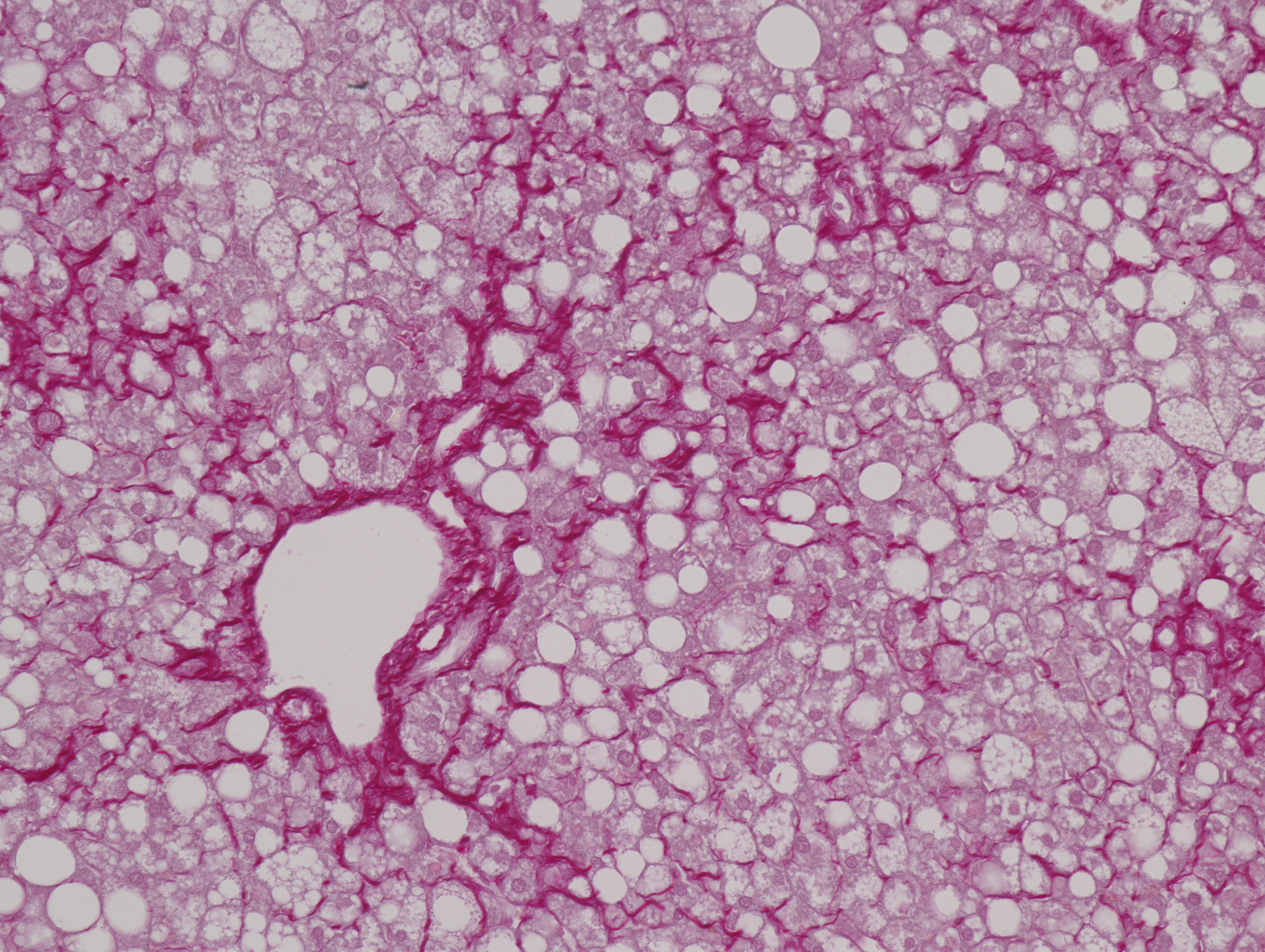

Supplement: Supplementary file 5 — Source data Fig. 3 [file 44318_2024_196_MOESM5_ESM.zip › Figure 3/Figure 3-E/Demonstrated image/HFD Con/HFD Con x20.tif]

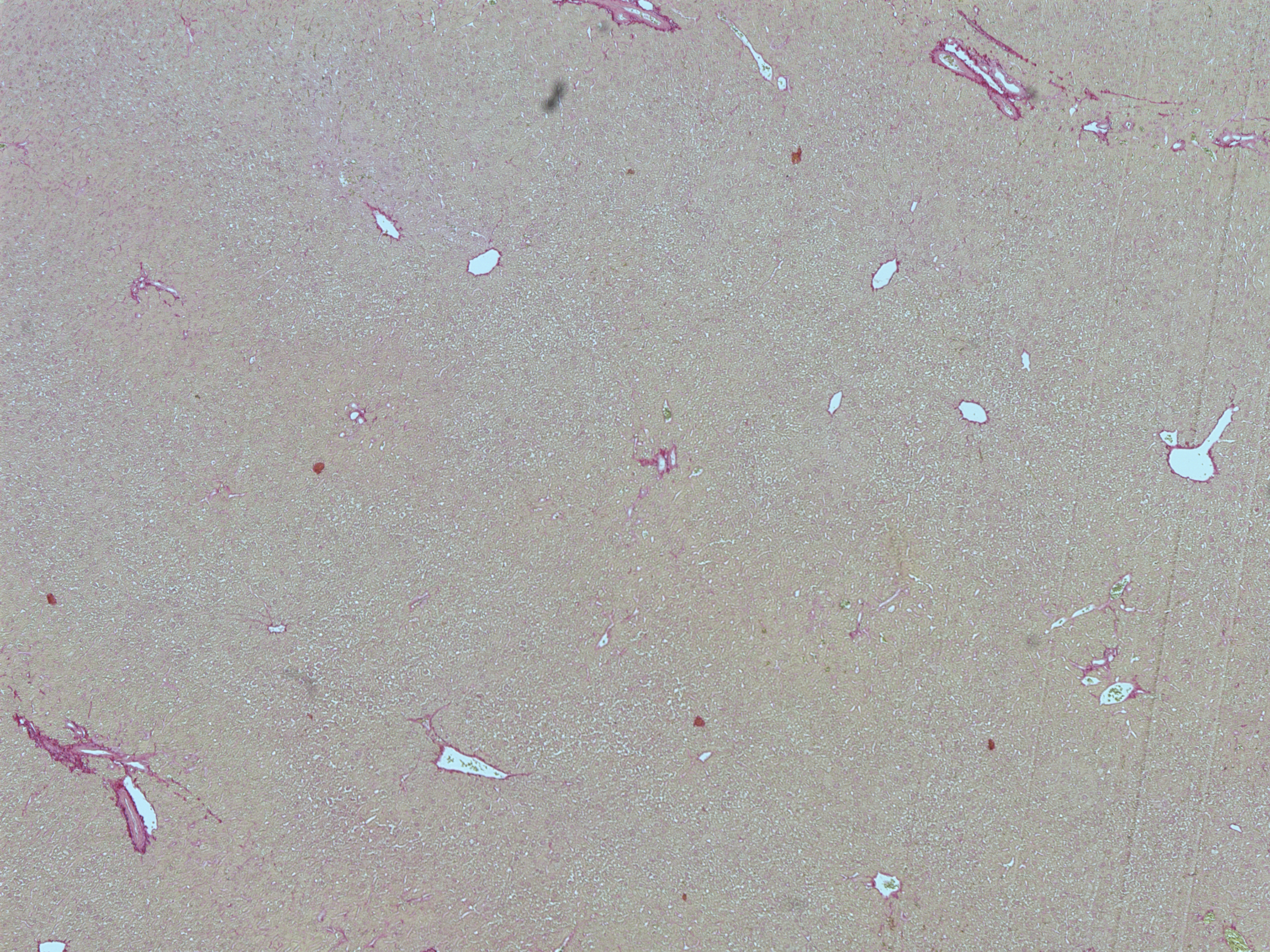

Supplement: Supplementary file 5 — Source data Fig. 3 [file 44318_2024_196_MOESM5_ESM.zip › Figure 3/Figure 3-E/Demonstrated image/NC Con/NC Con 4x.tif]
