# Supplementary figures and images for: PCPE-1, a brown adipose tissue-derived cytokine, promotes obesity-induced liver fibrosis (part 1 of 6)
Source: EMBO J. 2024 Aug 19;43(21):4846–69. doi: 10.1038/s44318-024-00196-0 (PMC11535236; doi:10.1038/s44318-024-00196-0)

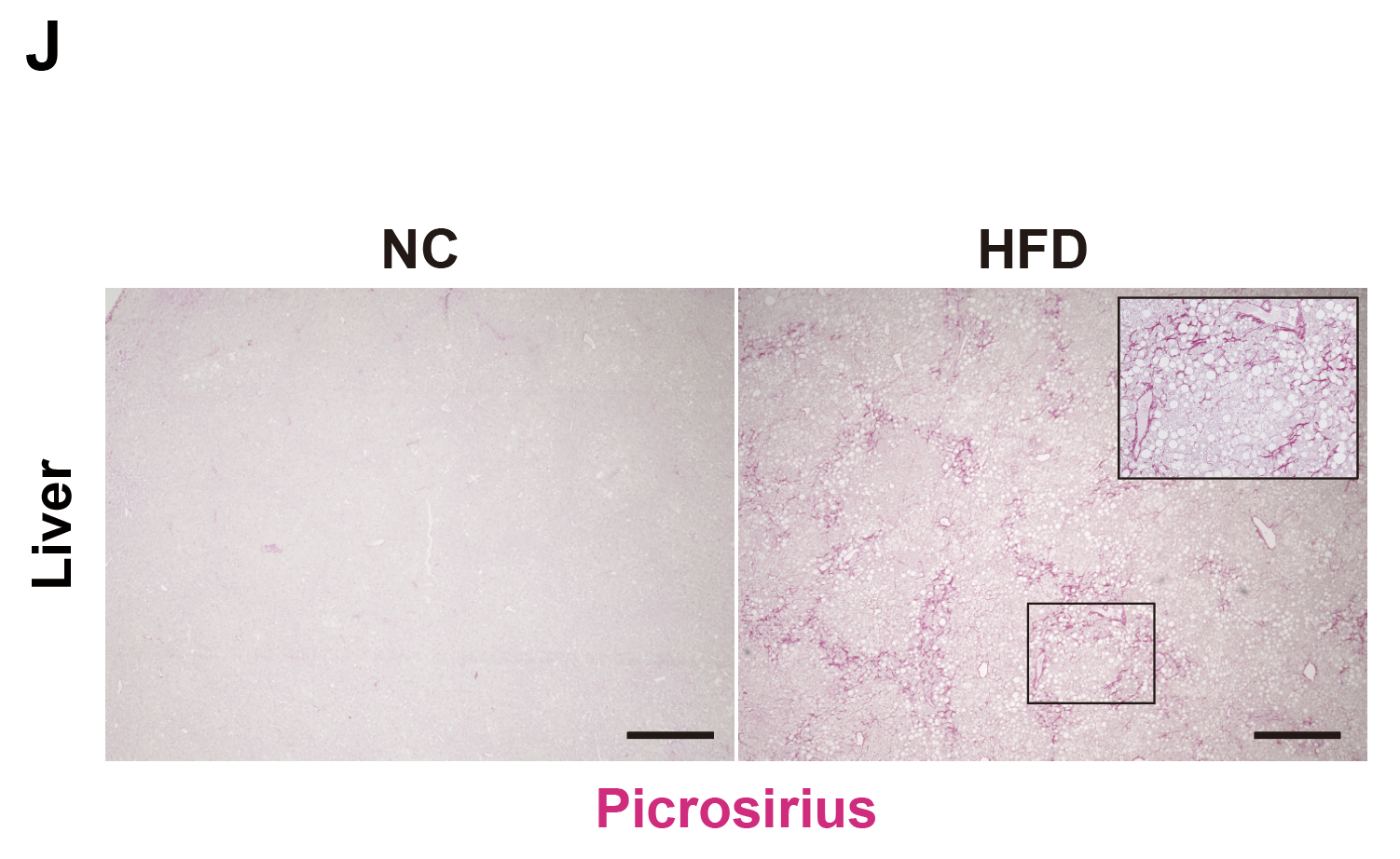

Supplement: Supplementary file 3 — Source data Fig. 1 [file 44318_2024_196_MOESM3_ESM.zip › Figure 1/Figure 1-J/Fig.1J.png]

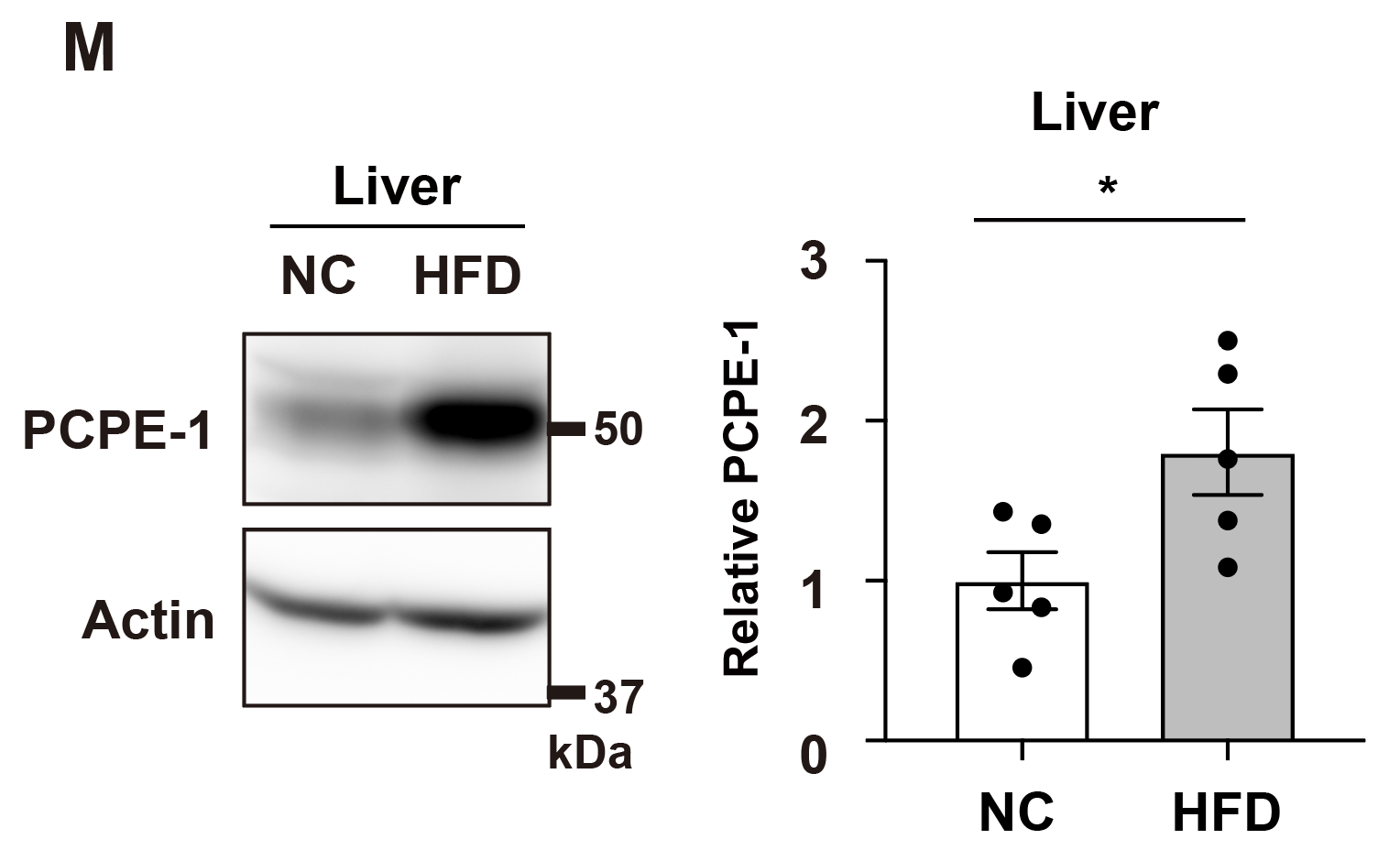

Supplement: Supplementary file 3 — Source data Fig. 1 [file 44318_2024_196_MOESM3_ESM.zip › Figure 1/Figure 1-M/Fig.1M.png]

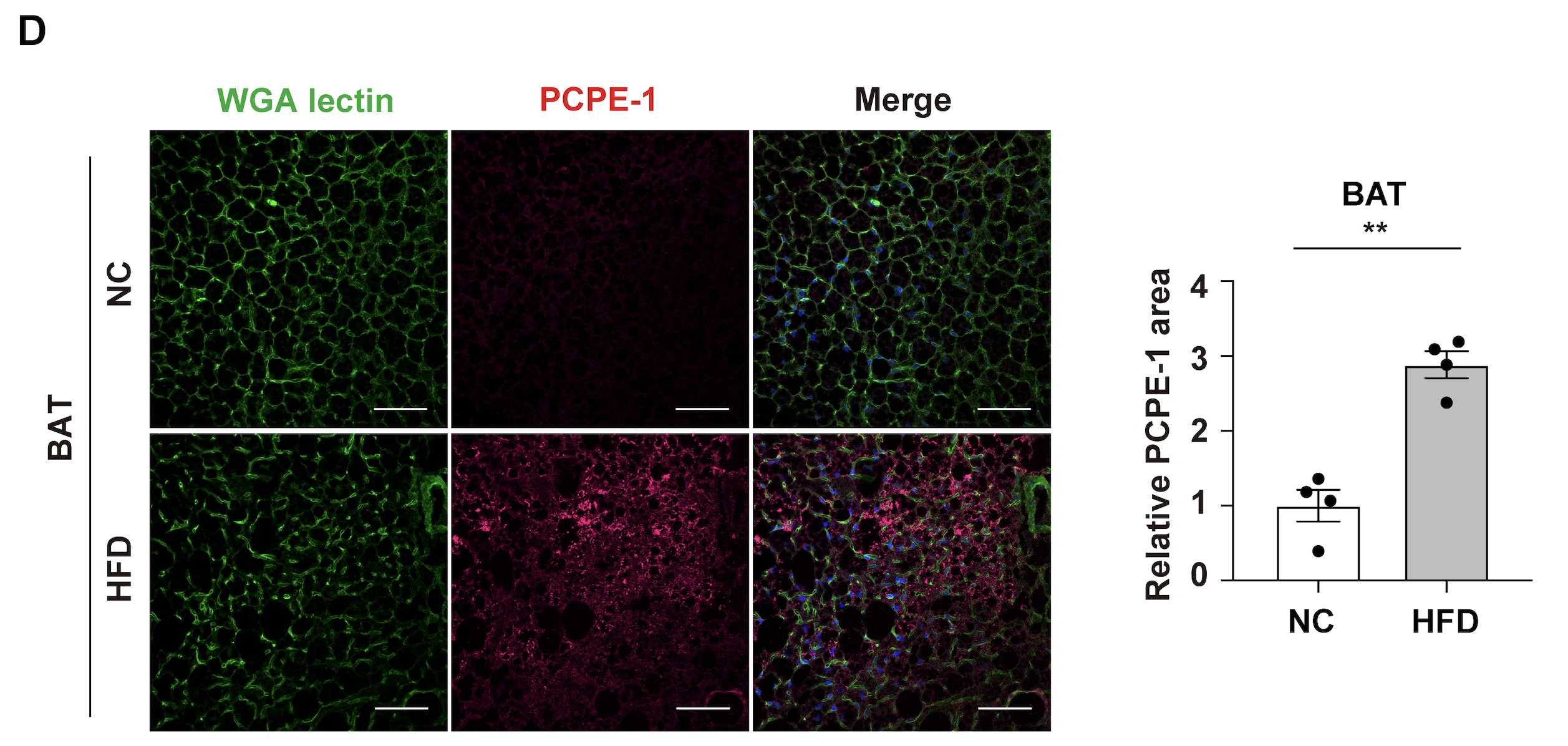

Supplement: Supplementary file 3 — Source data Fig. 1 [file 44318_2024_196_MOESM3_ESM.zip › Figure 1/Figure 1-D/Fig.1D.png]

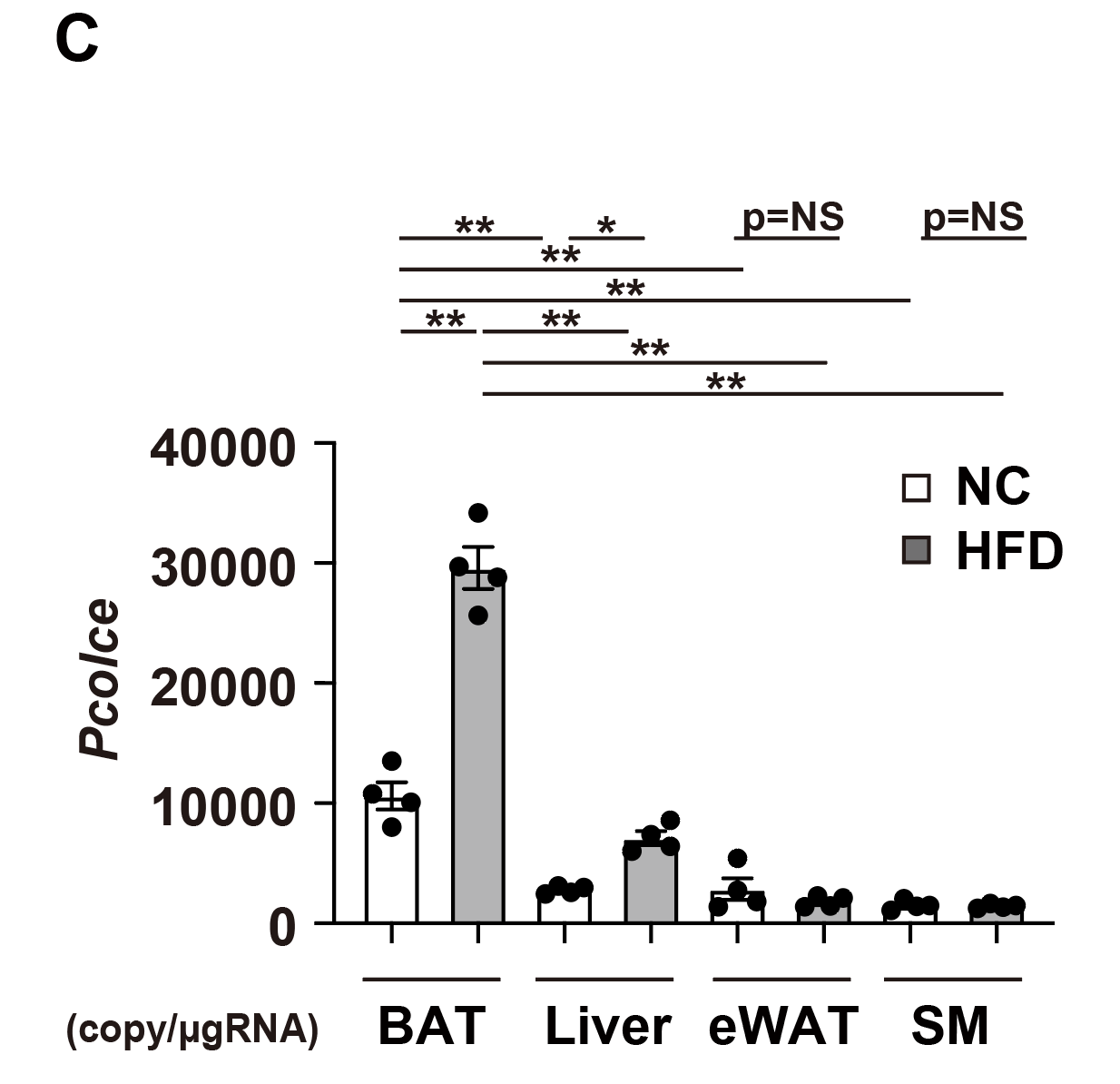

Supplement: Supplementary file 3 — Source data Fig. 1 [file 44318_2024_196_MOESM3_ESM.zip › Figure 1/Figure 1-C/Fig.1C.png]

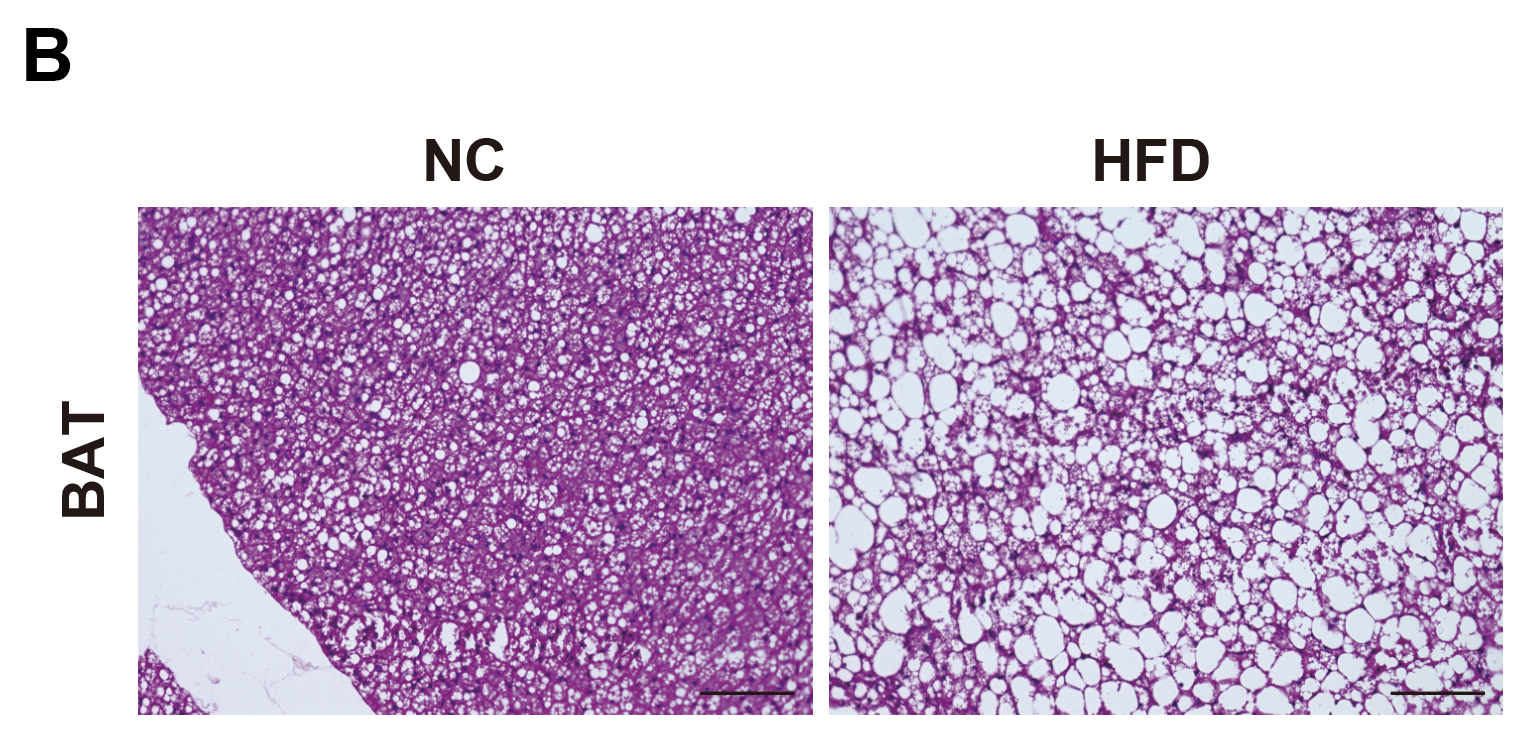

Supplement: Supplementary file 3 — Source data Fig. 1 [file 44318_2024_196_MOESM3_ESM.zip › Figure 1/Figure 1-B/Fig.1B.png]

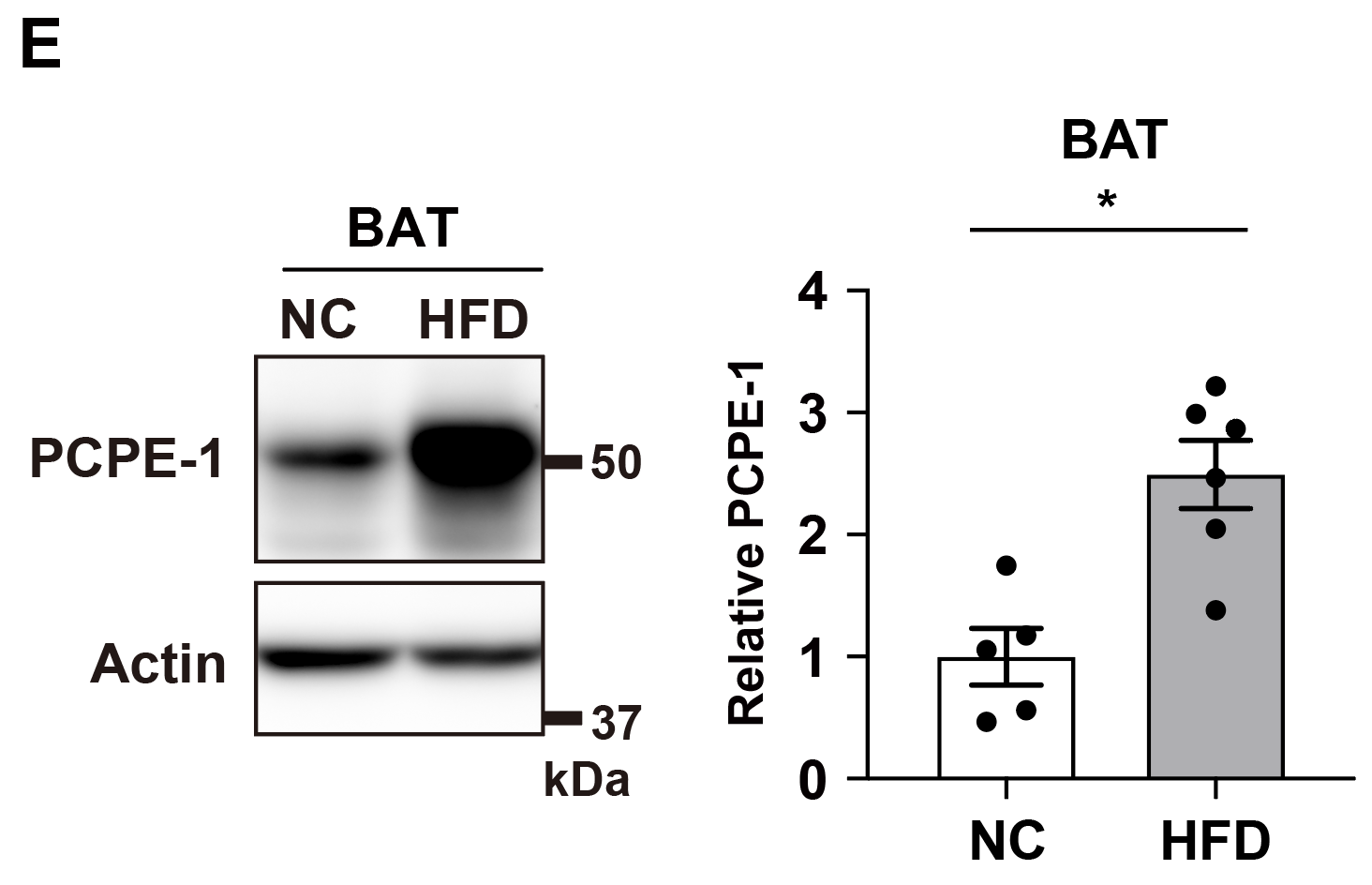

Supplement: Supplementary file 3 — Source data Fig. 1 [file 44318_2024_196_MOESM3_ESM.zip › Figure 1/Figure 1-E/Fig.1E.png]

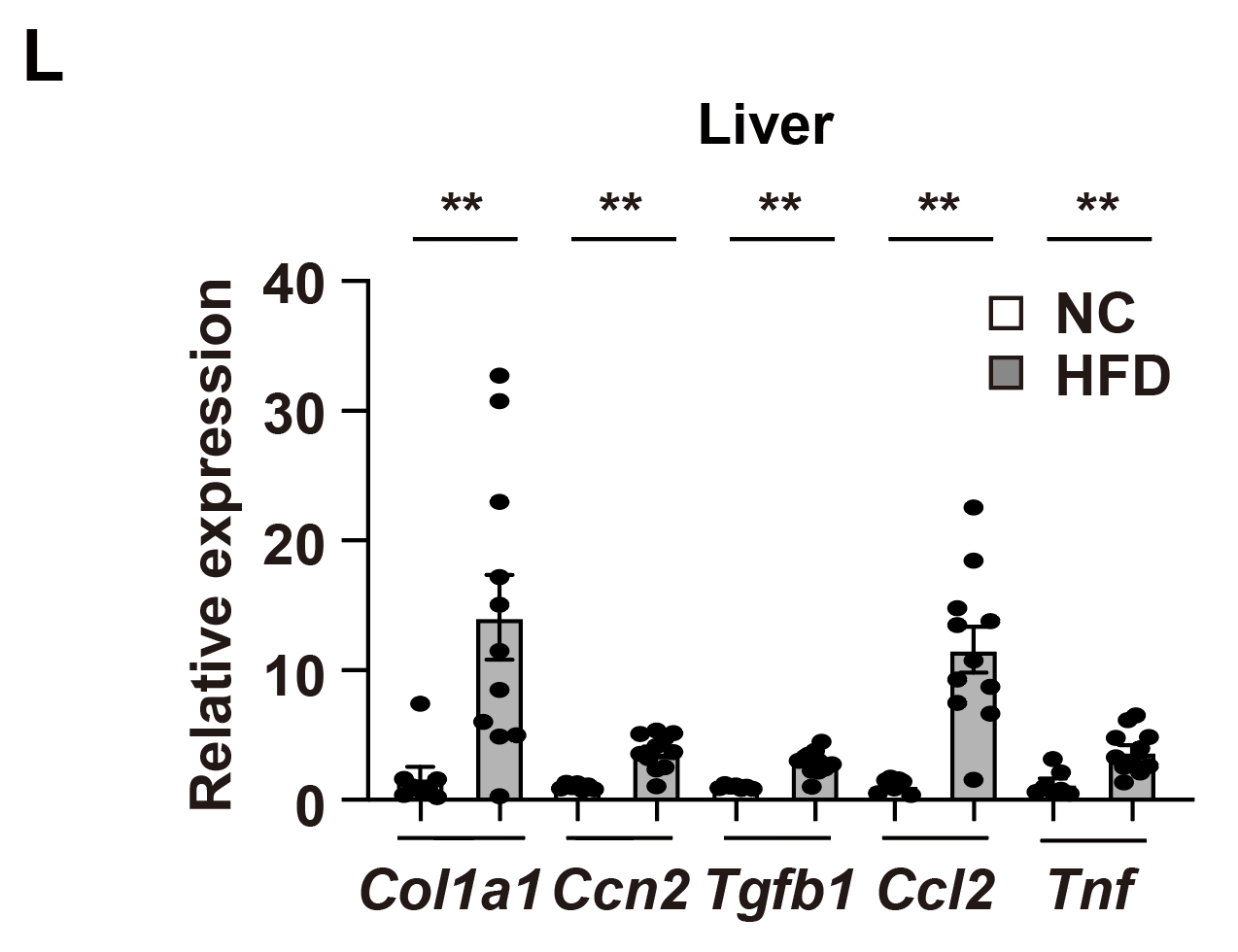

Supplement: Supplementary file 3 — Source data Fig. 1 [file 44318_2024_196_MOESM3_ESM.zip › Figure 1/Figure 1-L/Fig.1L.png]

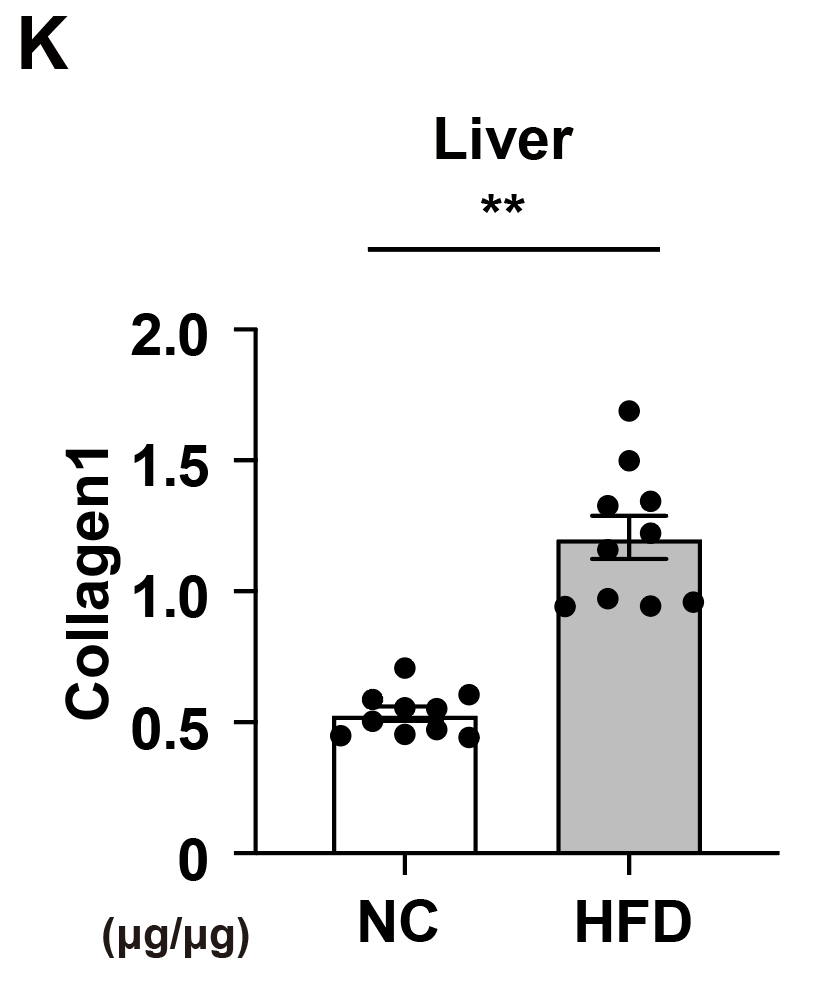

Supplement: Supplementary file 3 — Source data Fig. 1 [file 44318_2024_196_MOESM3_ESM.zip › Figure 1/Figure 1-K/Fig.1K.png]

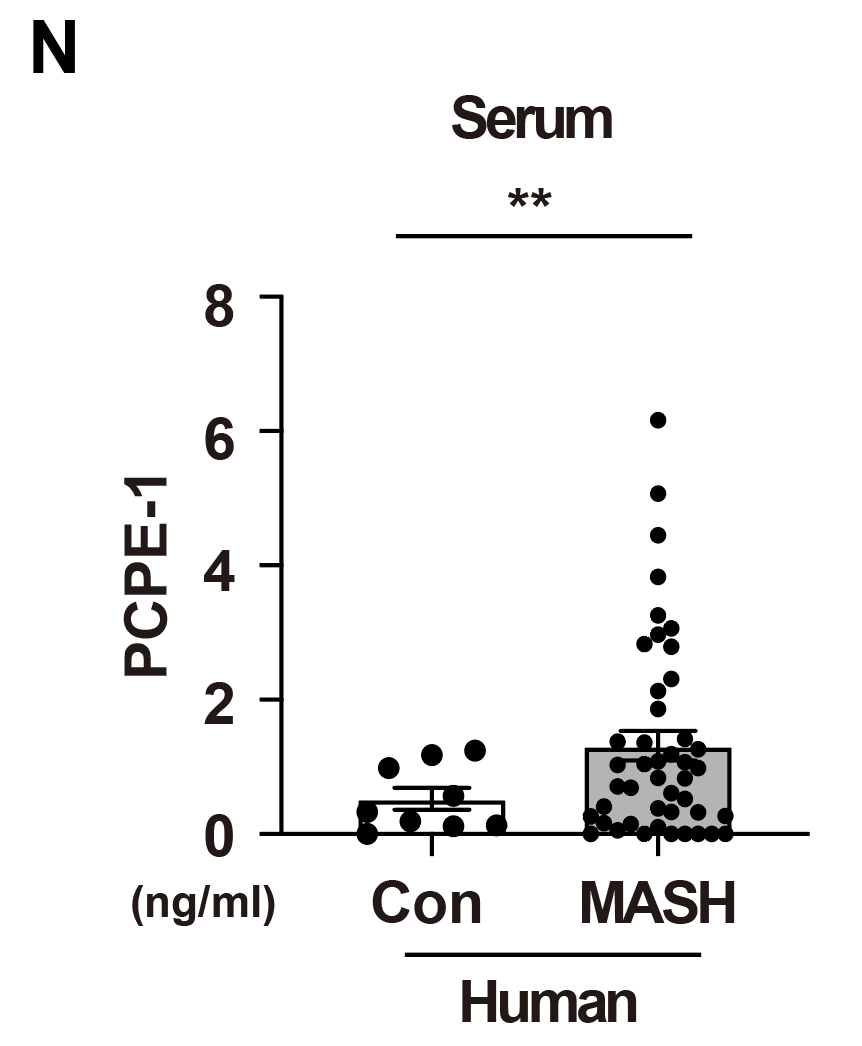

Supplement: Supplementary file 3 — Source data Fig. 1 [file 44318_2024_196_MOESM3_ESM.zip › Figure 1/Figure 1-N/Fig.1N.png]

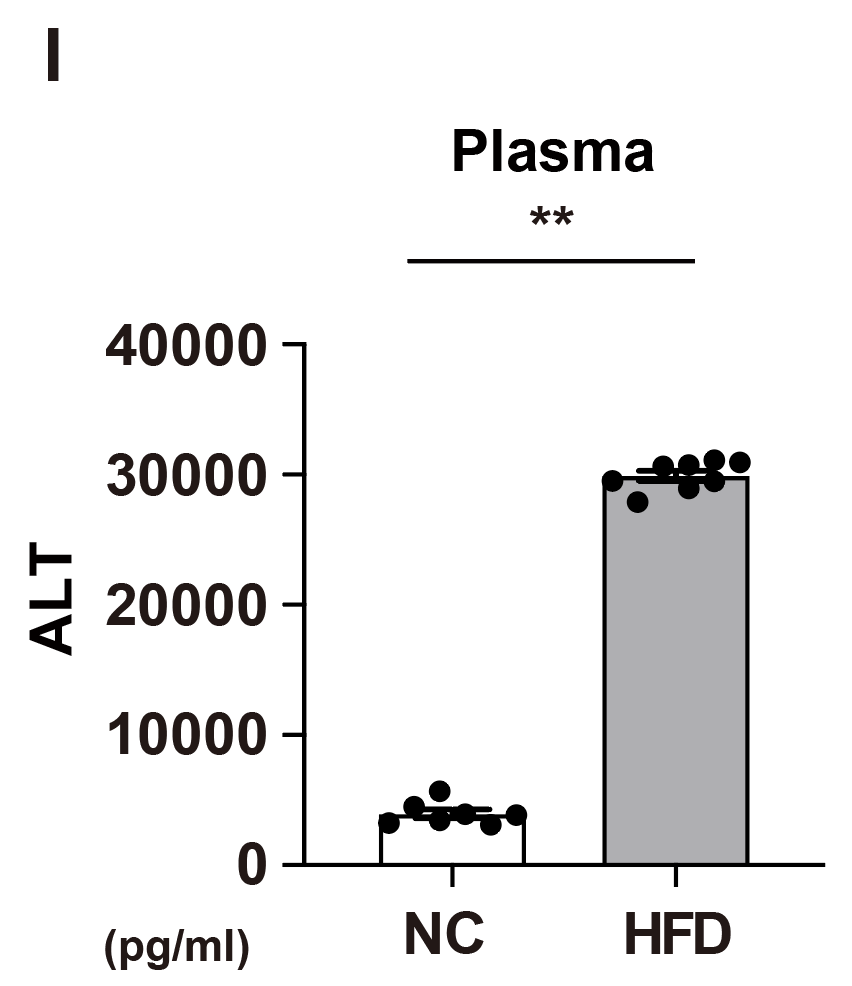

Supplement: Supplementary file 3 — Source data Fig. 1 [file 44318_2024_196_MOESM3_ESM.zip › Figure 1/Figure 1-I/Fig.1I.png]

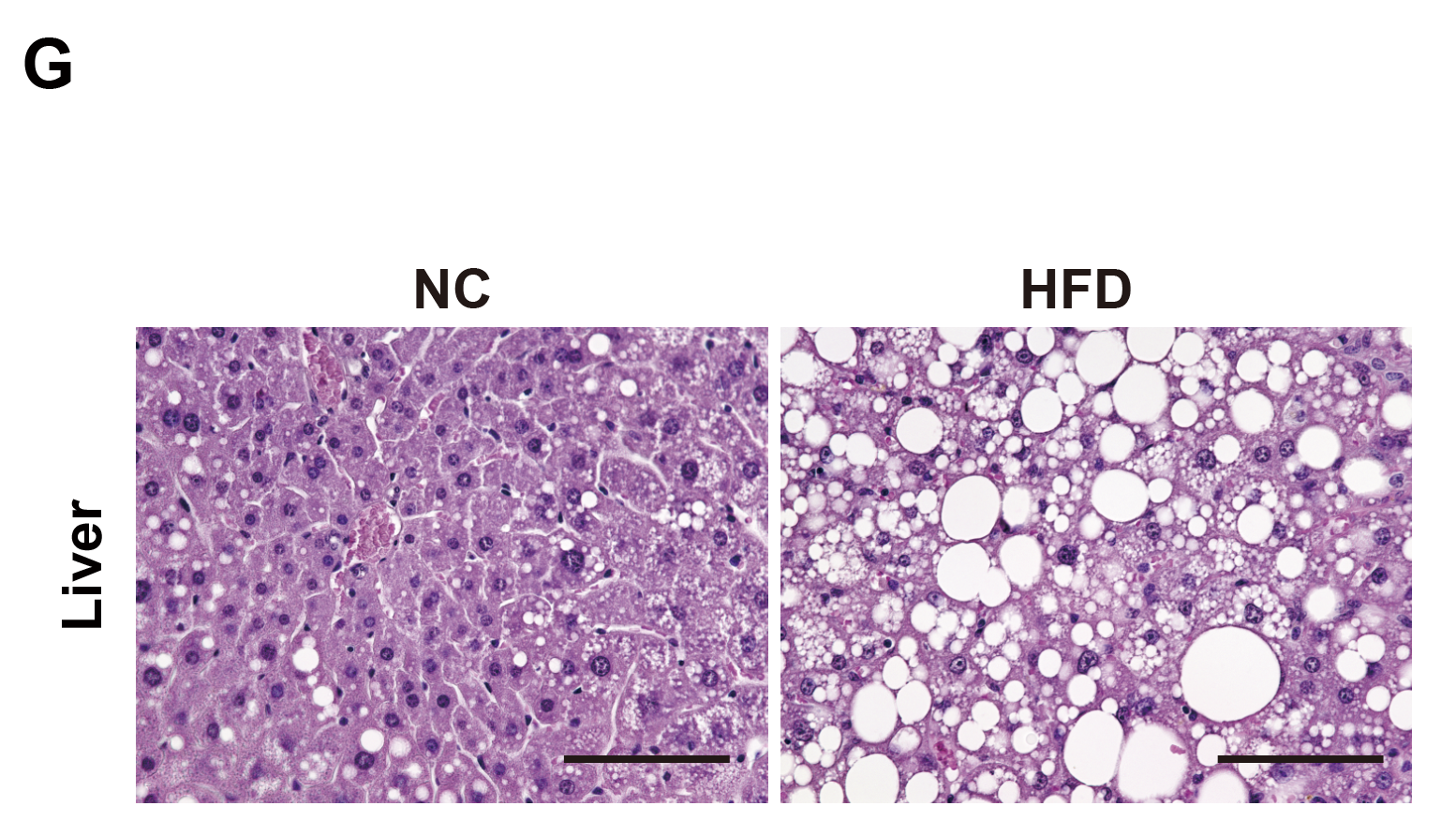

Supplement: Supplementary file 3 — Source data Fig. 1 [file 44318_2024_196_MOESM3_ESM.zip › Figure 1/Figure 1-G/Fig.1G.png]

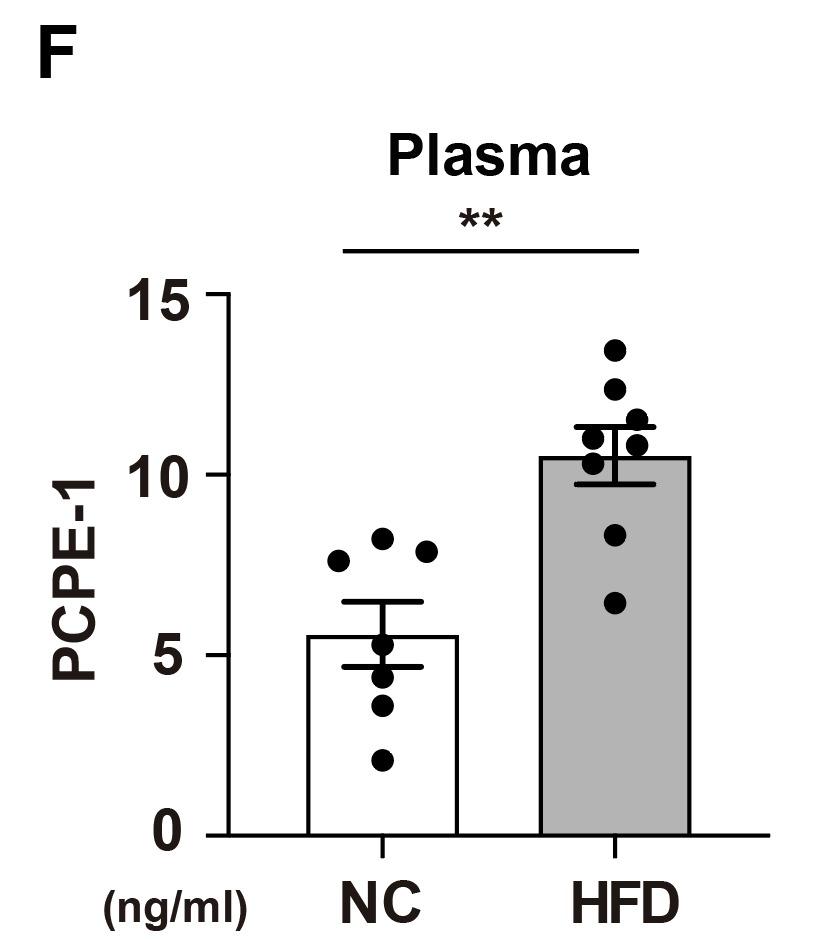

Supplement: Supplementary file 3 — Source data Fig. 1 [file 44318_2024_196_MOESM3_ESM.zip › Figure 1/Figure 1-F/Fig.1F.png]

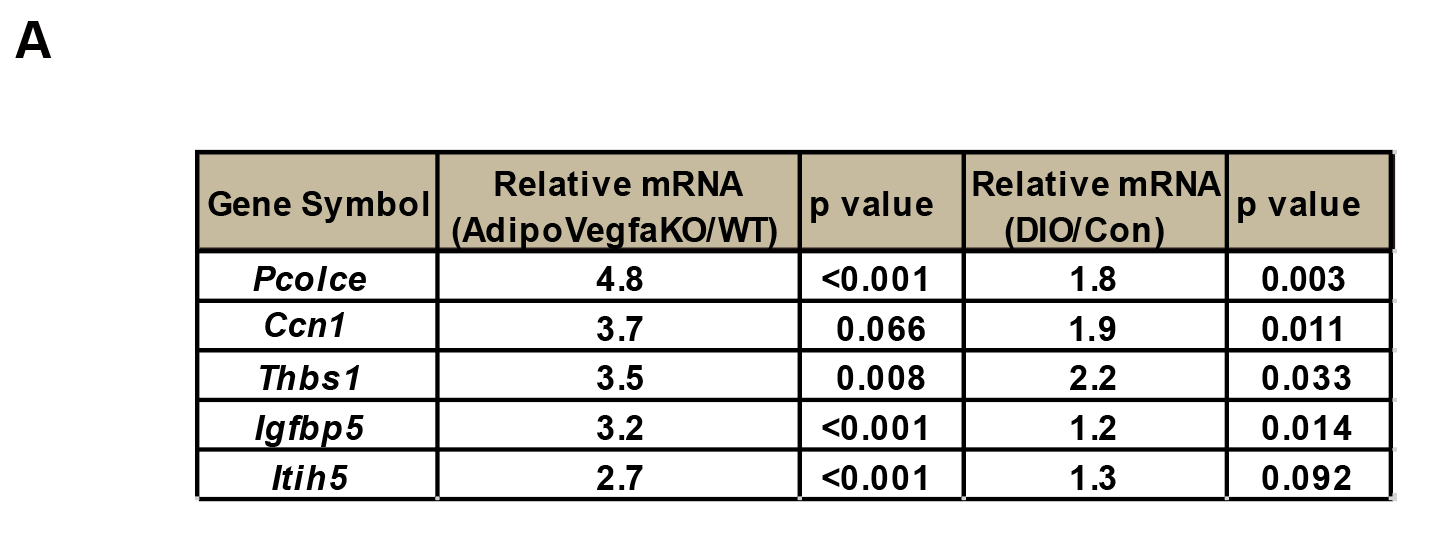

Supplement: Supplementary file 3 — Source data Fig. 1 [file 44318_2024_196_MOESM3_ESM.zip › Figure 1/Figure 1-A/Fig.1A.png]

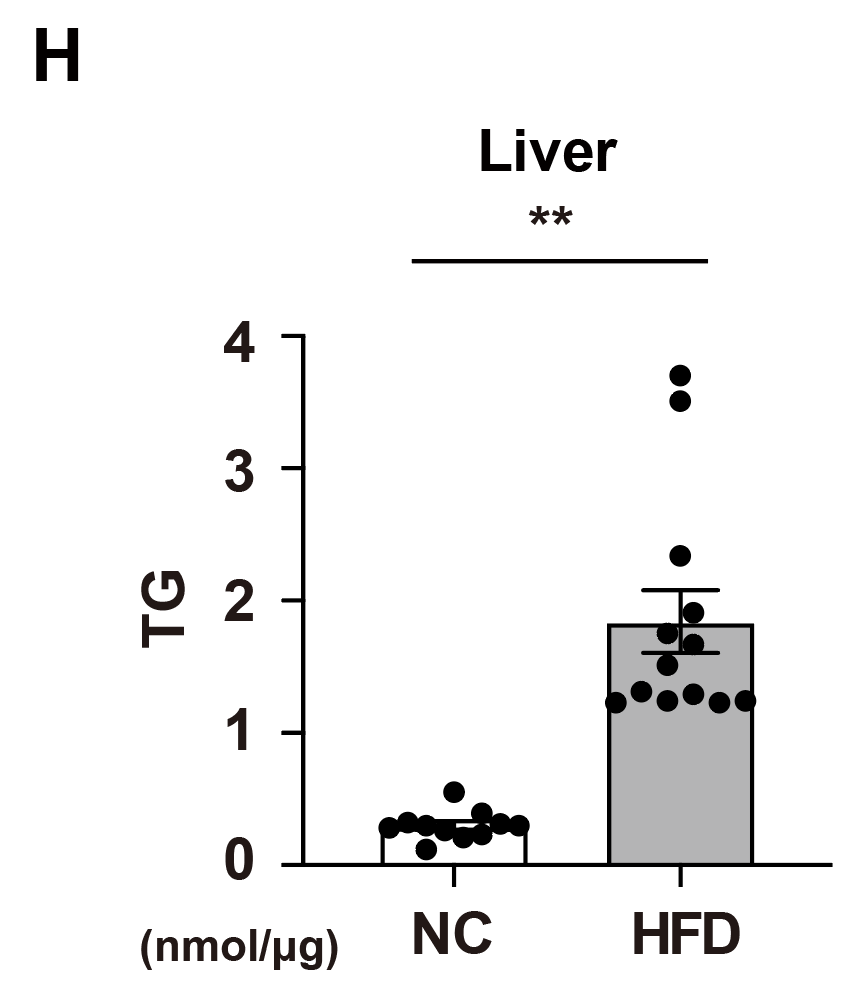

Supplement: Supplementary file 3 — Source data Fig. 1 [file 44318_2024_196_MOESM3_ESM.zip › Figure 1/Figure 1-H/Fig.1H.png]

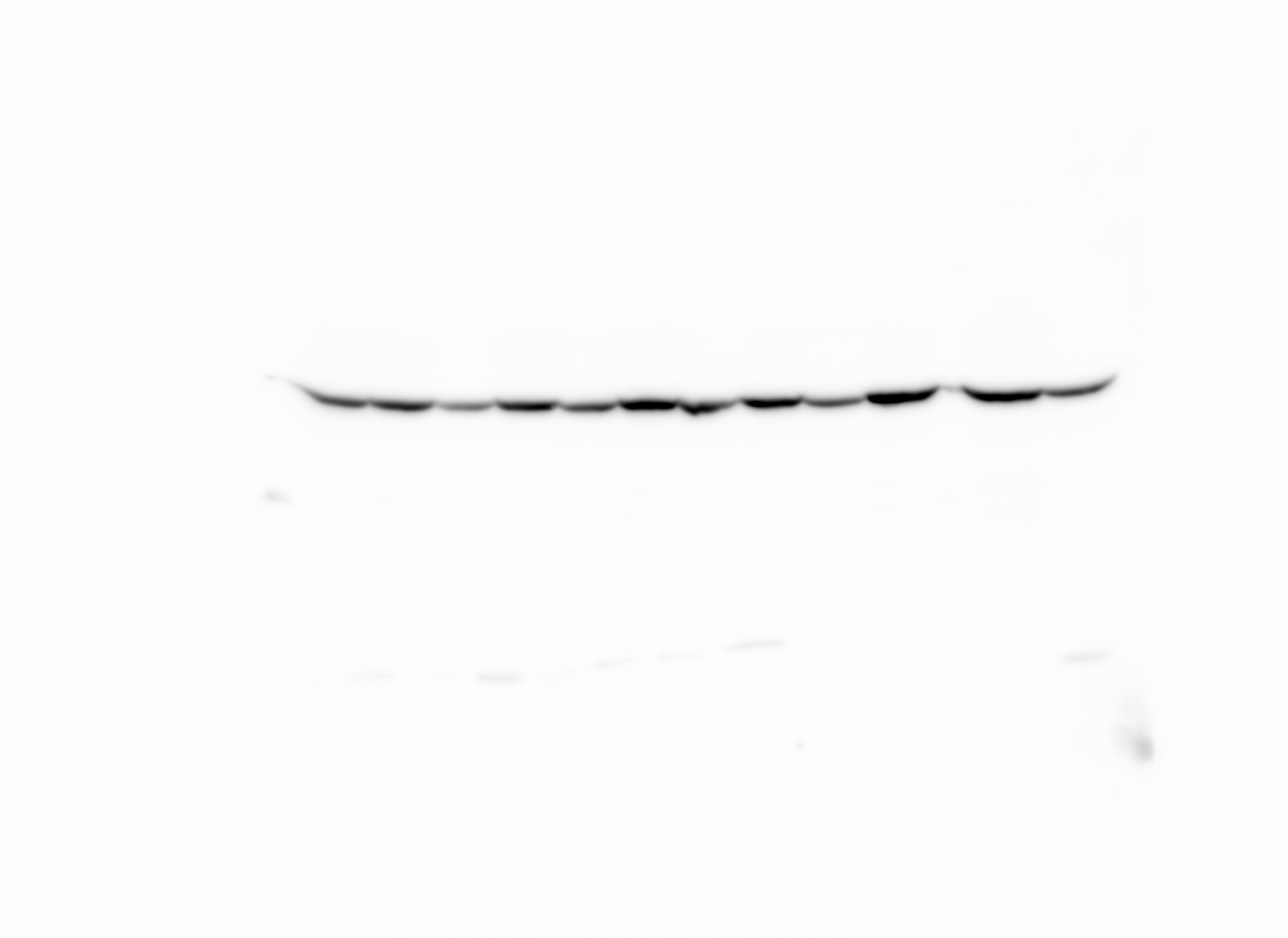

Supplement: Supplementary file 3 — Source data Fig. 1 [file 44318_2024_196_MOESM3_ESM.zip › Figure 1/Figure 1-M/Quantificated image/Actin.tif]

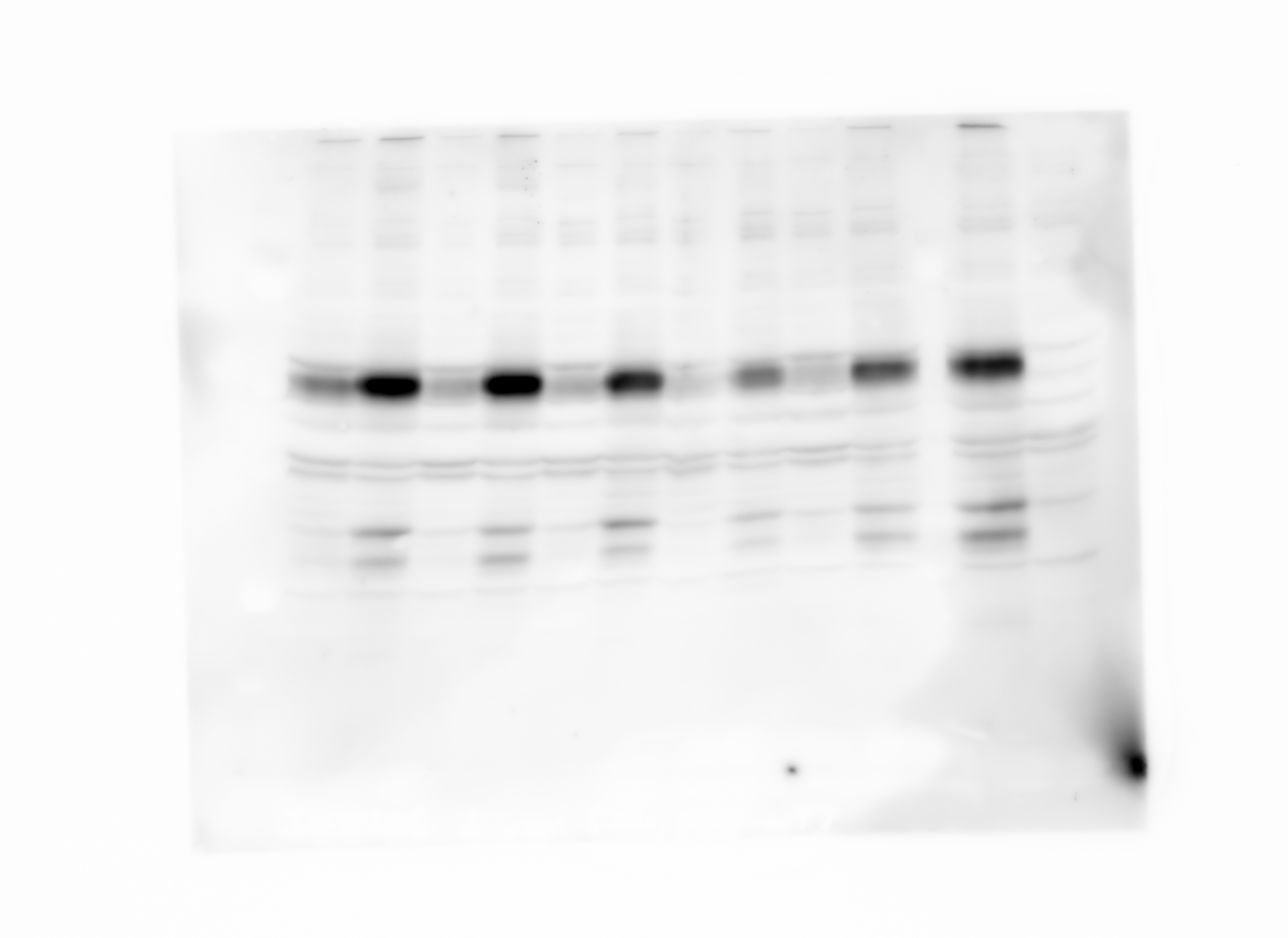

Supplement: Supplementary file 3 — Source data Fig. 1 [file 44318_2024_196_MOESM3_ESM.zip › Figure 1/Figure 1-M/Quantificated image/PCPE-1.tif]

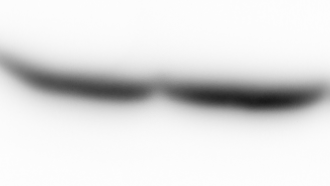

Supplement: Supplementary file 3 — Source data Fig. 1 [file 44318_2024_196_MOESM3_ESM.zip › Figure 1/Figure 1-M/Demonstrated image/Actin.tif]

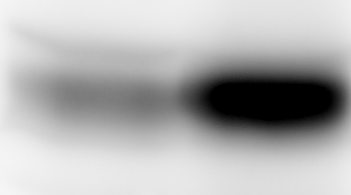

Supplement: Supplementary file 3 — Source data Fig. 1 [file 44318_2024_196_MOESM3_ESM.zip › Figure 1/Figure 1-M/Demonstrated image/PCPE-1.tif]

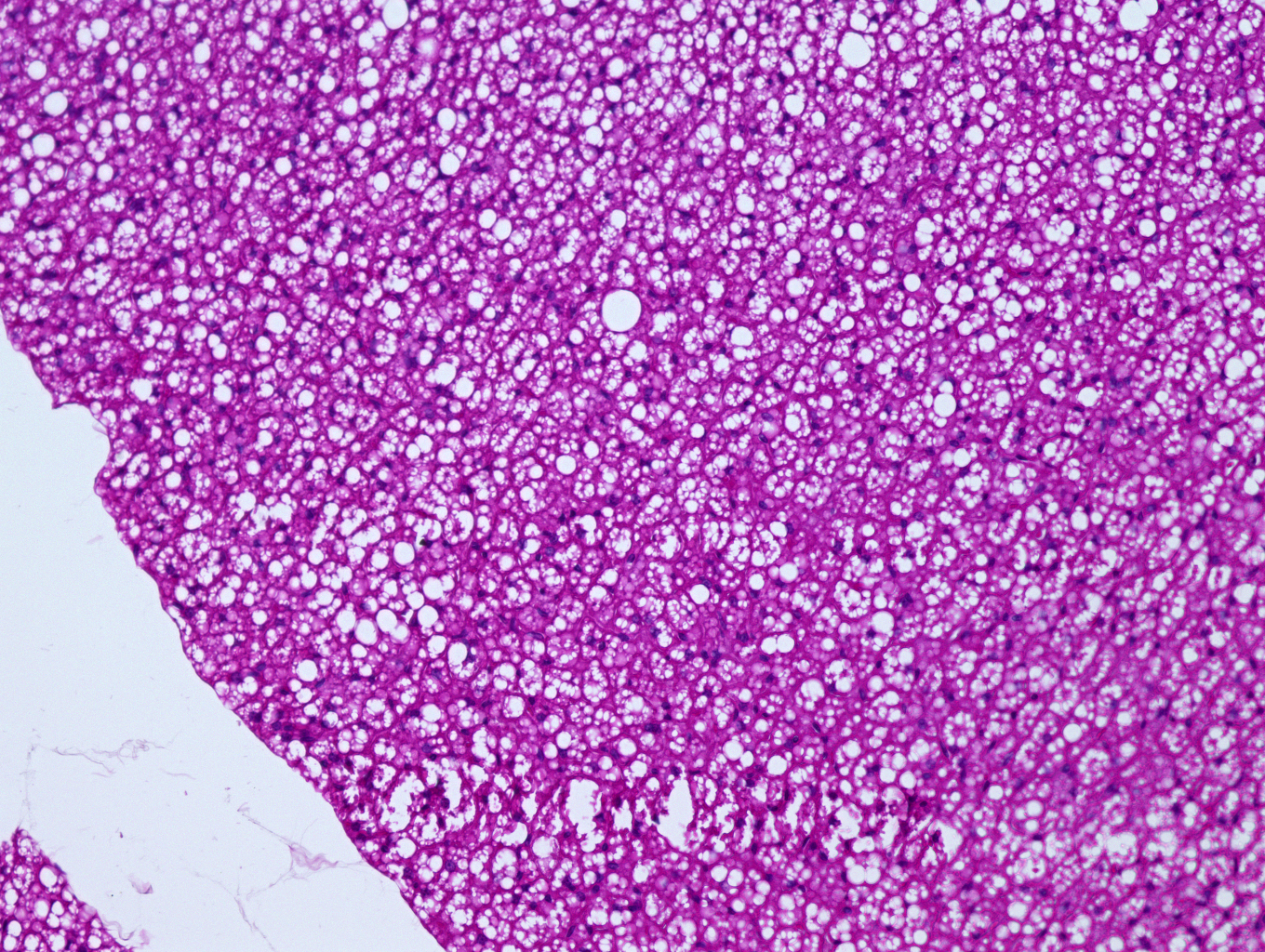

Supplement: Supplementary file 3 — Source data Fig. 1 [file 44318_2024_196_MOESM3_ESM.zip › Figure 1/Figure 1-B/Demonstrated data/Fig NC.tif]

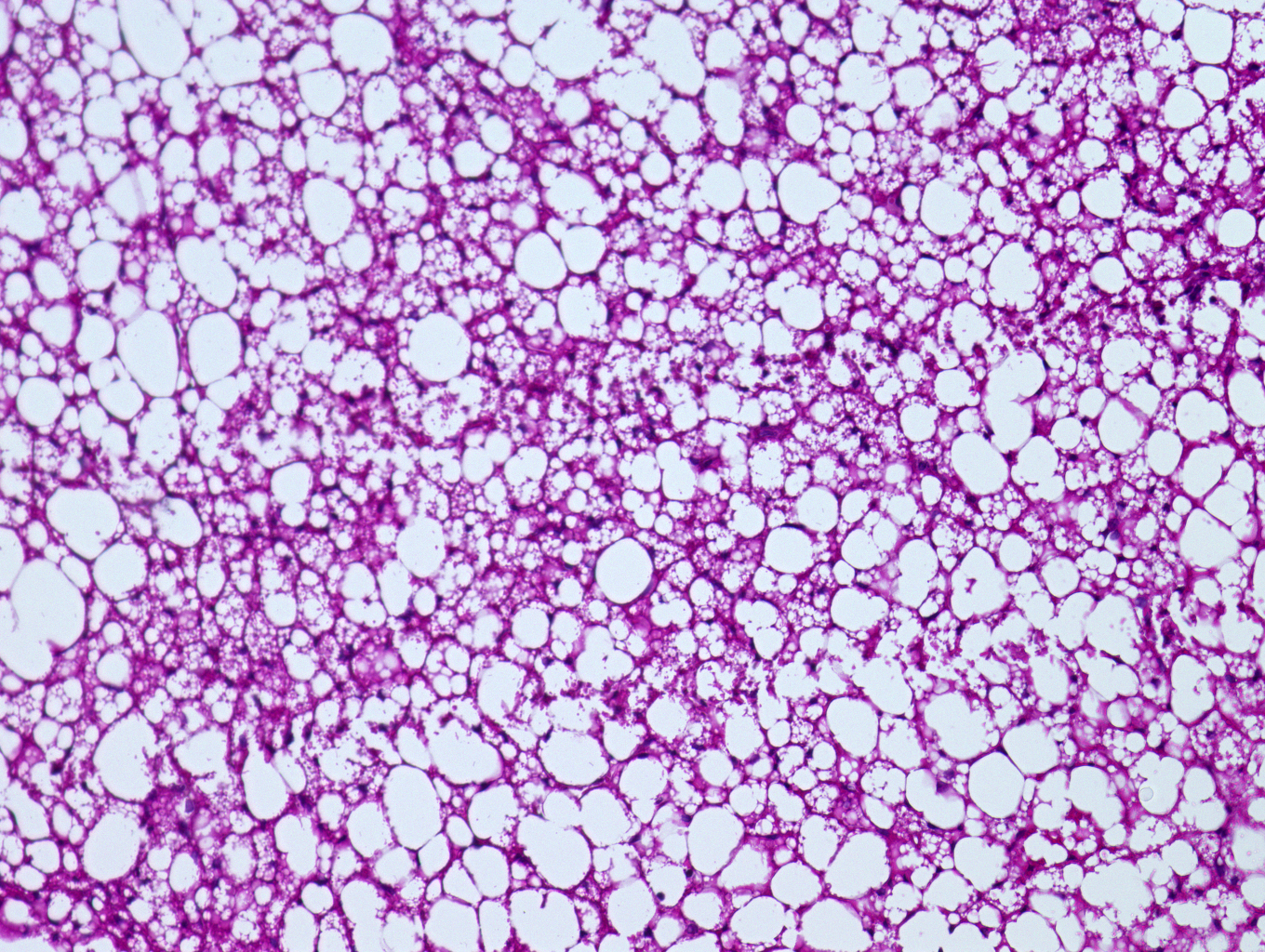

Supplement: Supplementary file 3 — Source data Fig. 1 [file 44318_2024_196_MOESM3_ESM.zip › Figure 1/Figure 1-B/Demonstrated data/Fig HFD.tif]

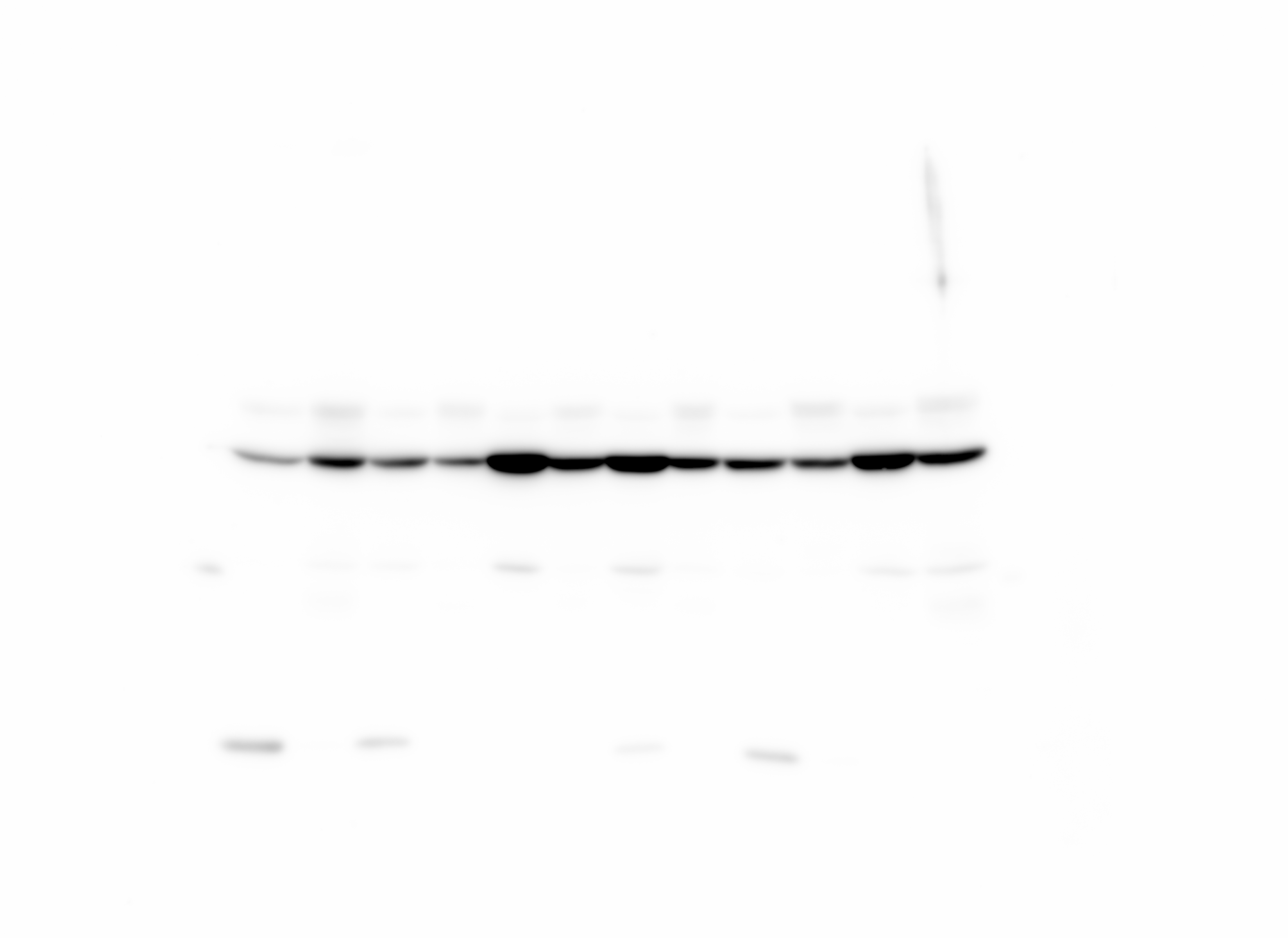

Supplement: Supplementary file 3 — Source data Fig. 1 [file 44318_2024_196_MOESM3_ESM.zip › Figure 1/Figure 1-E/Quantificated data/Actin.tif]

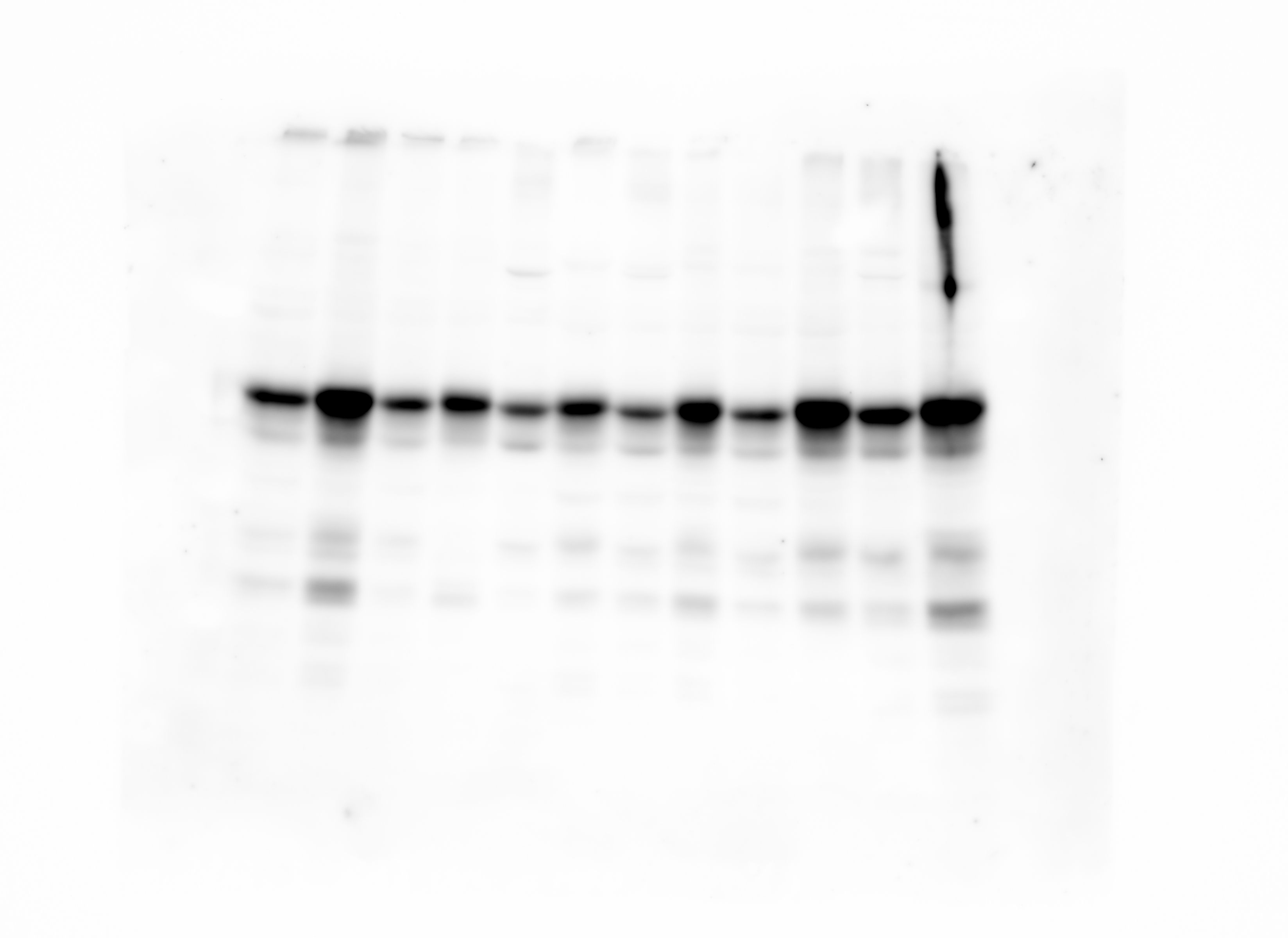

Supplement: Supplementary file 3 — Source data Fig. 1 [file 44318_2024_196_MOESM3_ESM.zip › Figure 1/Figure 1-E/Quantificated data/PCPE-1.tif]

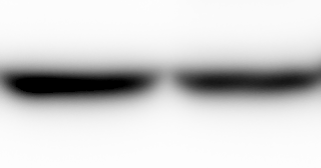

Supplement: Supplementary file 3 — Source data Fig. 1 [file 44318_2024_196_MOESM3_ESM.zip › Figure 1/Figure 1-E/Demonstrated data/Actin.tif]

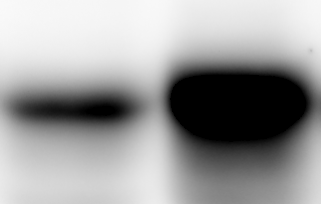

Supplement: Supplementary file 3 — Source data Fig. 1 [file 44318_2024_196_MOESM3_ESM.zip › Figure 1/Figure 1-E/Demonstrated data/PCPE-1.tif]

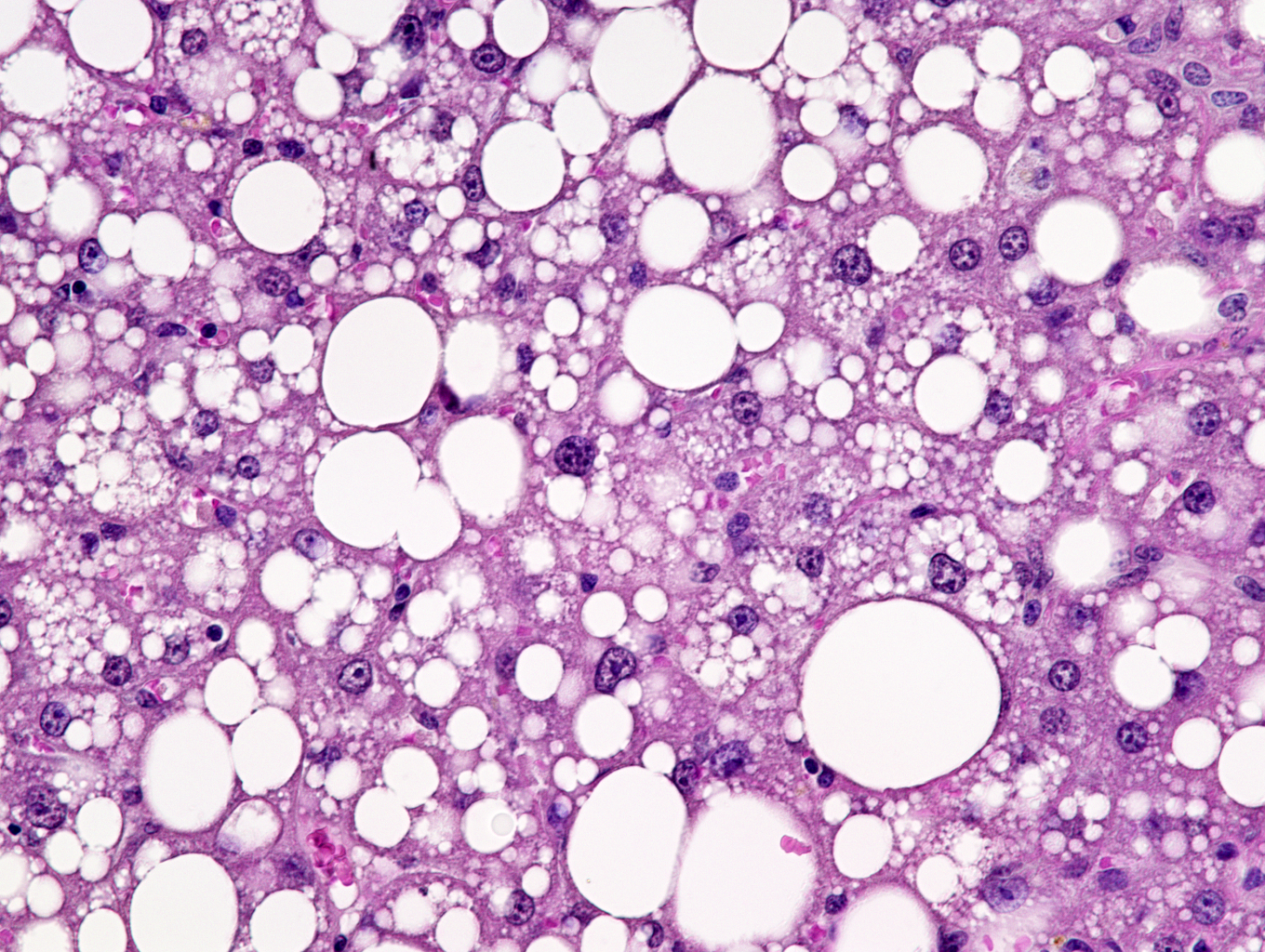

Supplement: Supplementary file 3 — Source data Fig. 1 [file 44318_2024_196_MOESM3_ESM.zip › Figure 1/Figure 1-G/Demonstrated image/HFD liver.tif]

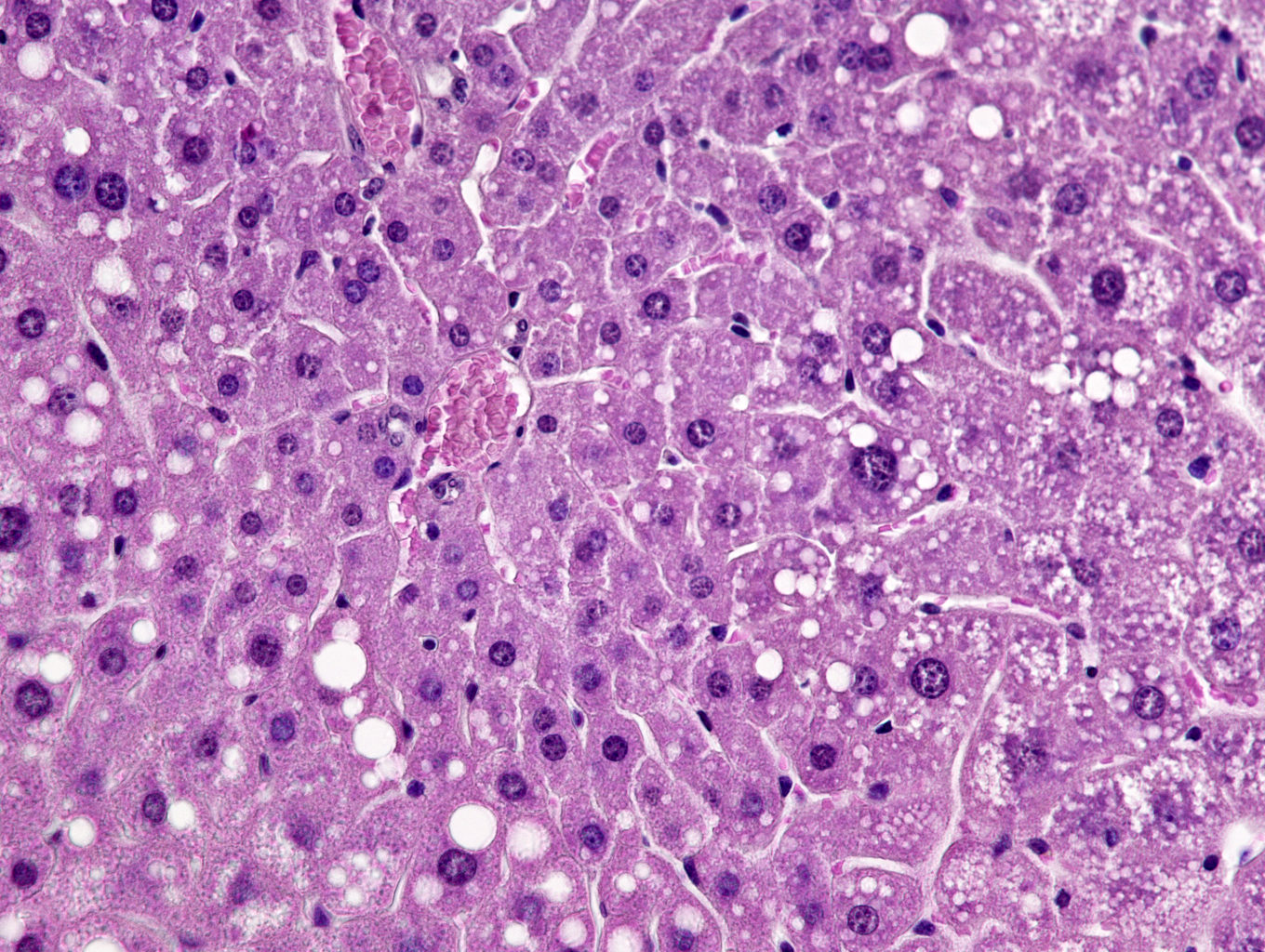

Supplement: Supplementary file 3 — Source data Fig. 1 [file 44318_2024_196_MOESM3_ESM.zip › Figure 1/Figure 1-G/Demonstrated image/NC liver.tif]

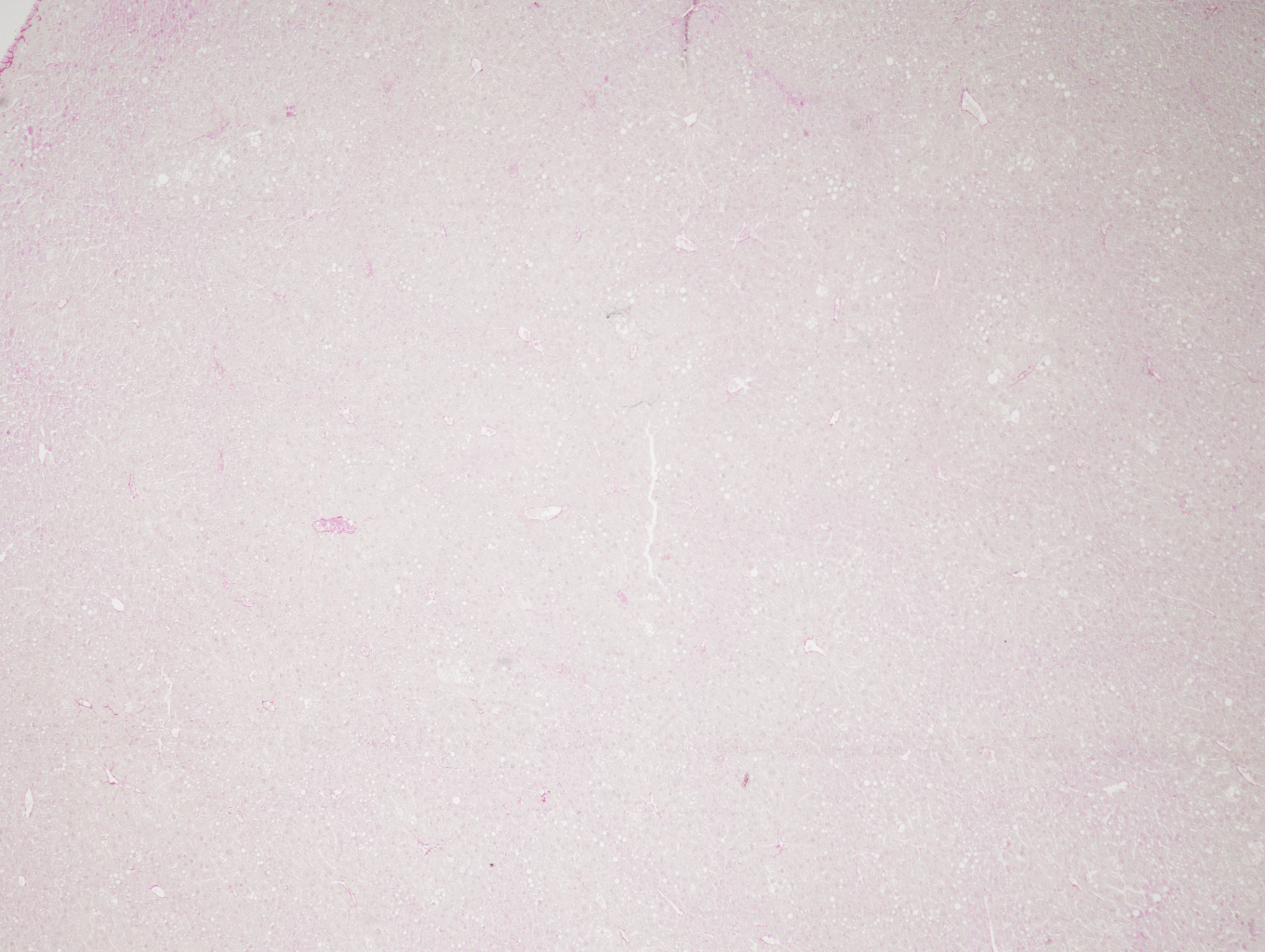

Supplement: Supplementary file 3 — Source data Fig. 1 [file 44318_2024_196_MOESM3_ESM.zip › Figure 1/Figure 1-J/Demonstrated image/NC /NC liver.tif]

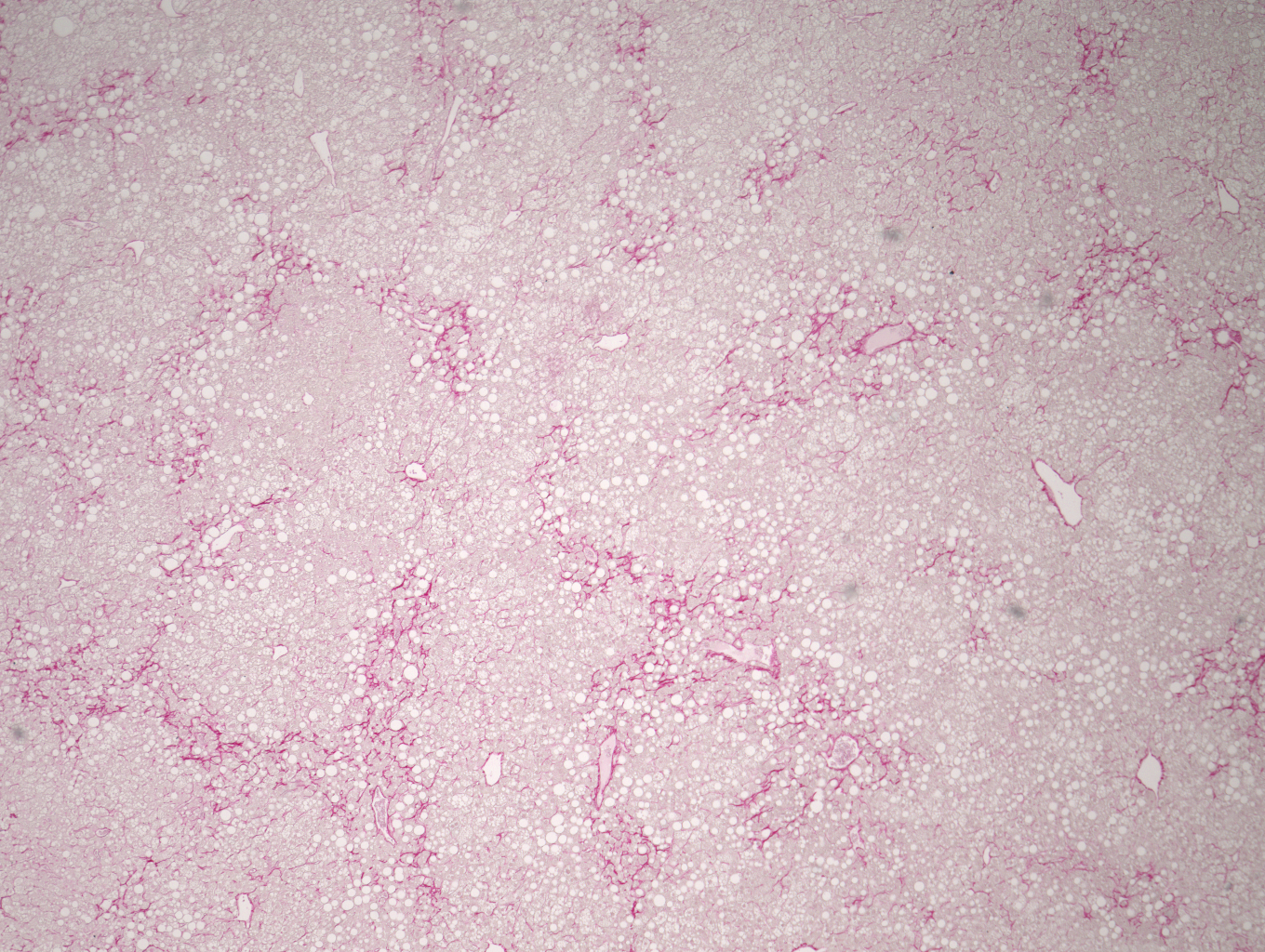

Supplement: Supplementary file 3 — Source data Fig. 1 [file 44318_2024_196_MOESM3_ESM.zip › Figure 1/Figure 1-J/Demonstrated image/HFD /HFD low mag.tif]

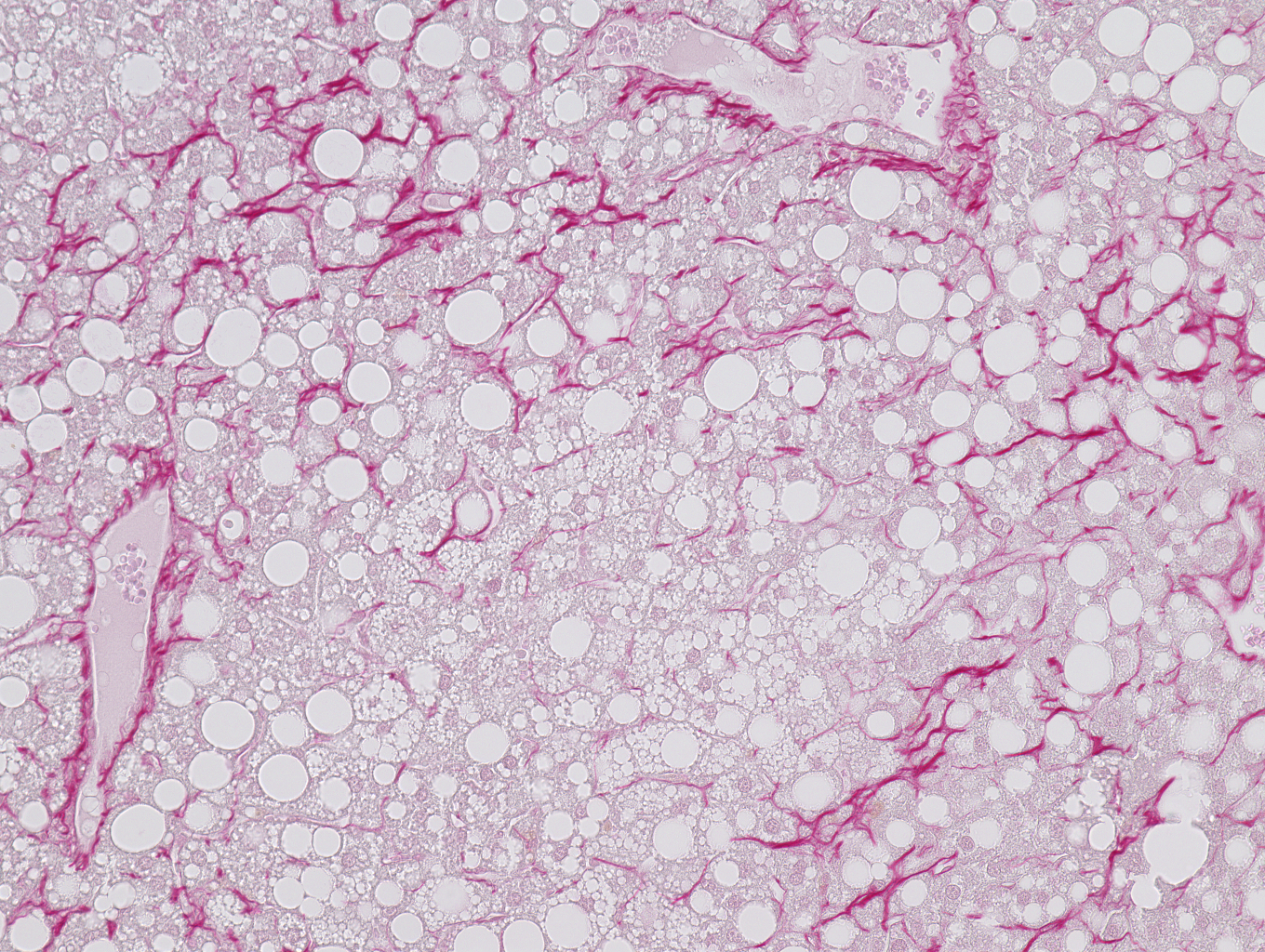

Supplement: Supplementary file 3 — Source data Fig. 1 [file 44318_2024_196_MOESM3_ESM.zip › Figure 1/Figure 1-J/Demonstrated image/HFD /HFD_Picrosirius.tif]

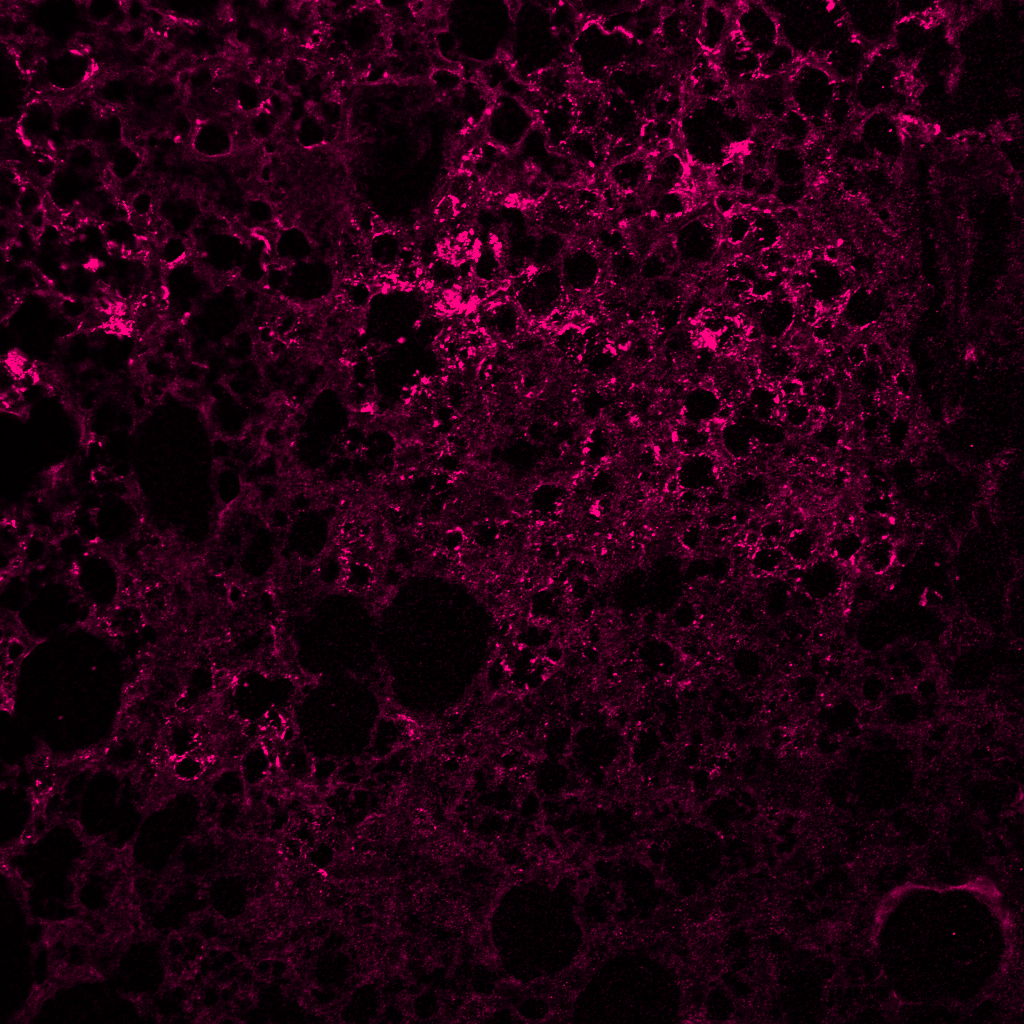

Supplement: Supplementary file 3 — Source data Fig. 1 [file 44318_2024_196_MOESM3_ESM.zip › Figure 1/Figure 1-D/Demonstrated image/HFD/HFD BAT_RGB_PCPE-1_Cy5.tif]

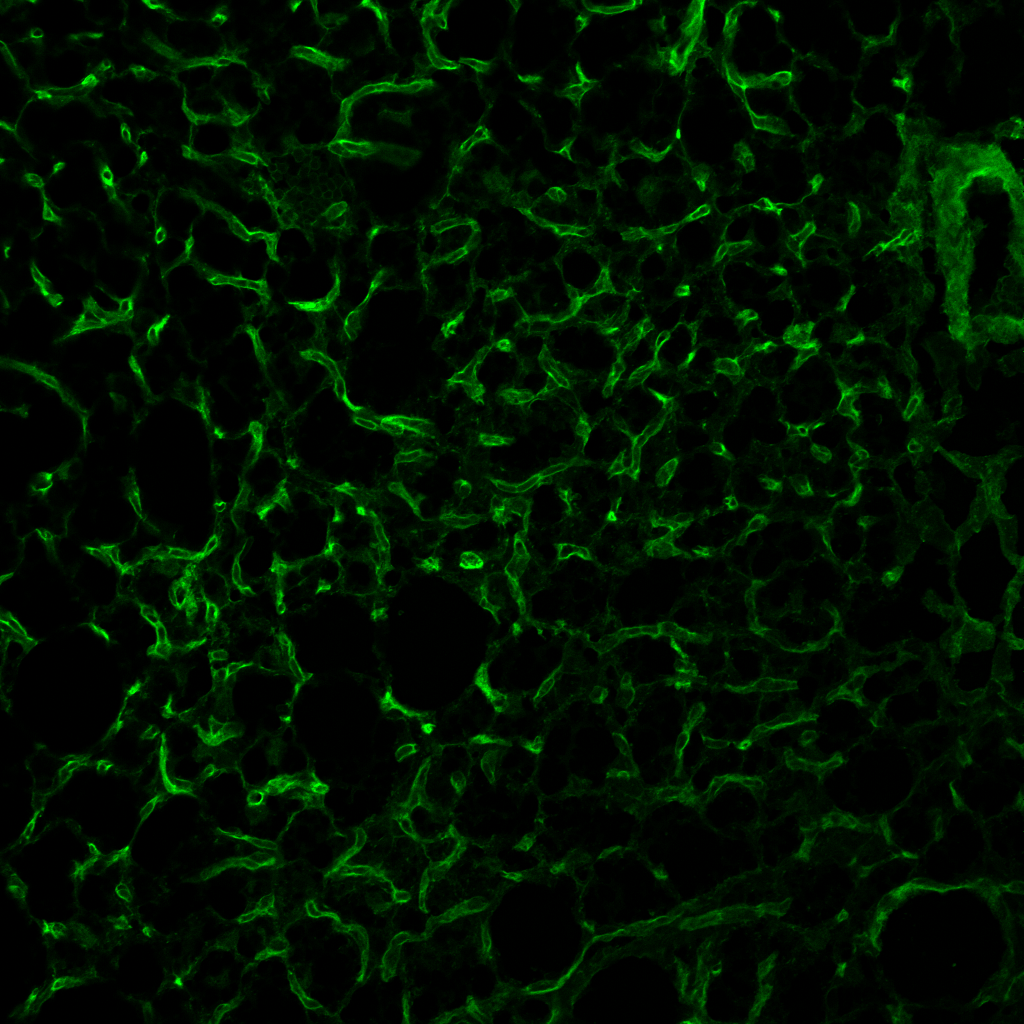

Supplement: Supplementary file 3 — Source data Fig. 1 [file 44318_2024_196_MOESM3_ESM.zip › Figure 1/Figure 1-D/Demonstrated image/HFD/HFD BAT_RGB_WGA lectin_FITC.tif]

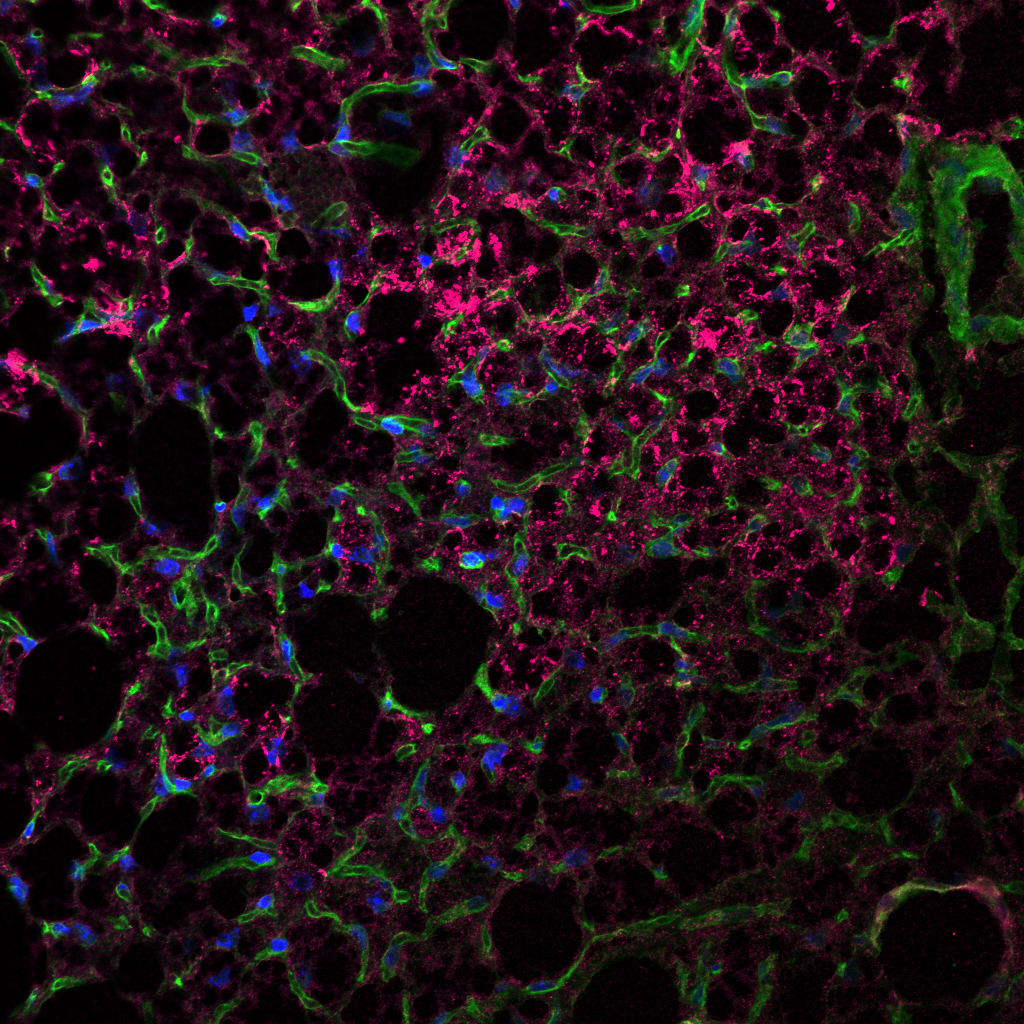

Supplement: Supplementary file 3 — Source data Fig. 1 [file 44318_2024_196_MOESM3_ESM.zip › Figure 1/Figure 1-D/Demonstrated image/HFD/HFD BAT_RGB_Merge.tif]

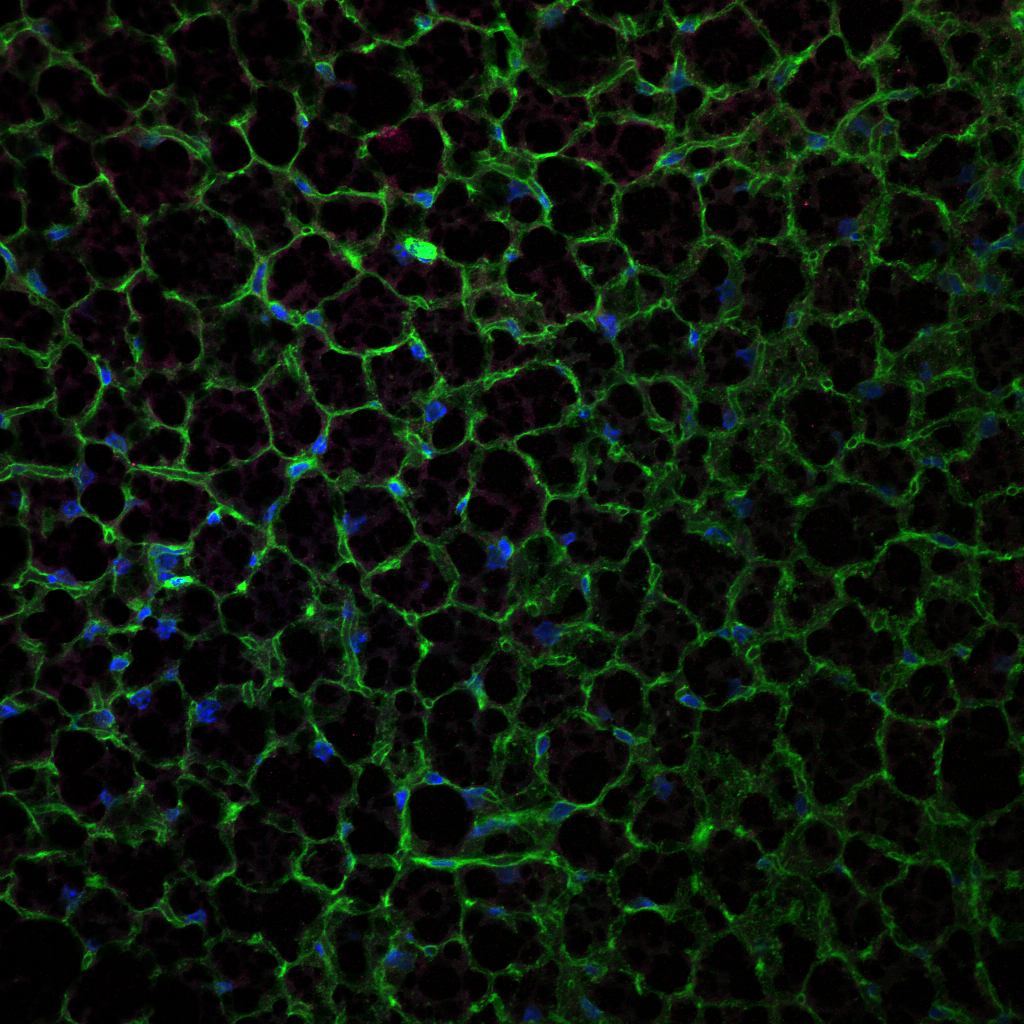

Supplement: Supplementary file 3 — Source data Fig. 1 [file 44318_2024_196_MOESM3_ESM.zip › Figure 1/Figure 1-D/Demonstrated image/NC/NC BAT_RGB_Merge.tif]

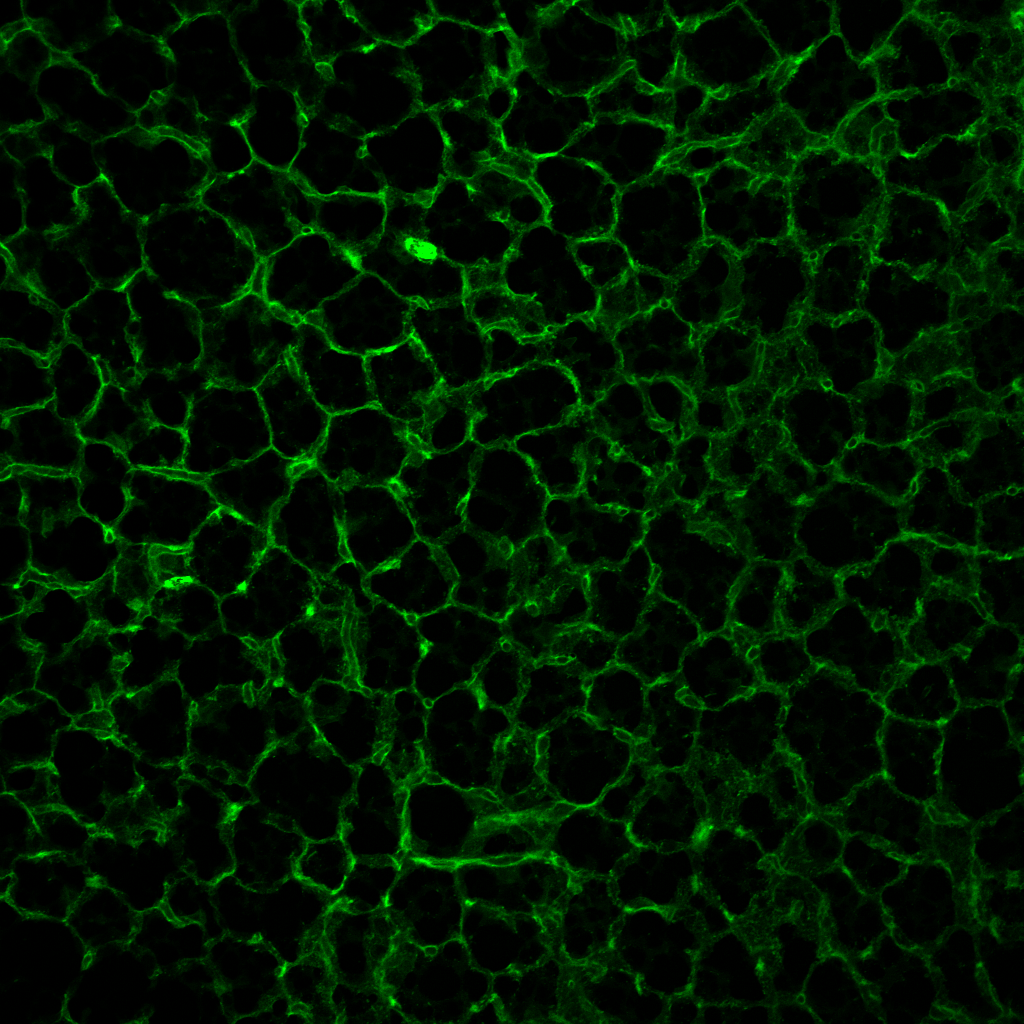

Supplement: Supplementary file 3 — Source data Fig. 1 [file 44318_2024_196_MOESM3_ESM.zip › Figure 1/Figure 1-D/Demonstrated image/NC/NC BAT_RGB_WGA lectin_FITC.tif]

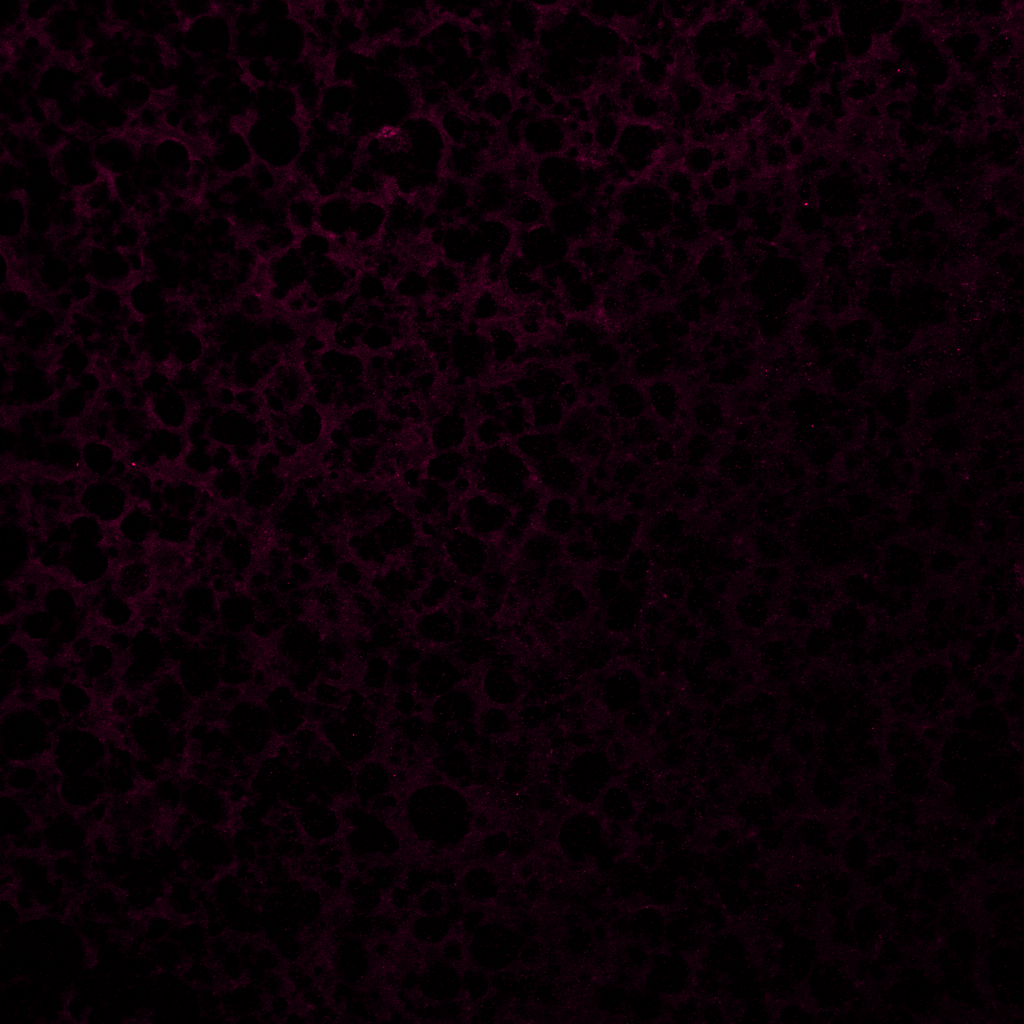

Supplement: Supplementary file 3 — Source data Fig. 1 [file 44318_2024_196_MOESM3_ESM.zip › Figure 1/Figure 1-D/Demonstrated image/NC/NC BAT_RGB_PCPE-1_Cy5.tif]

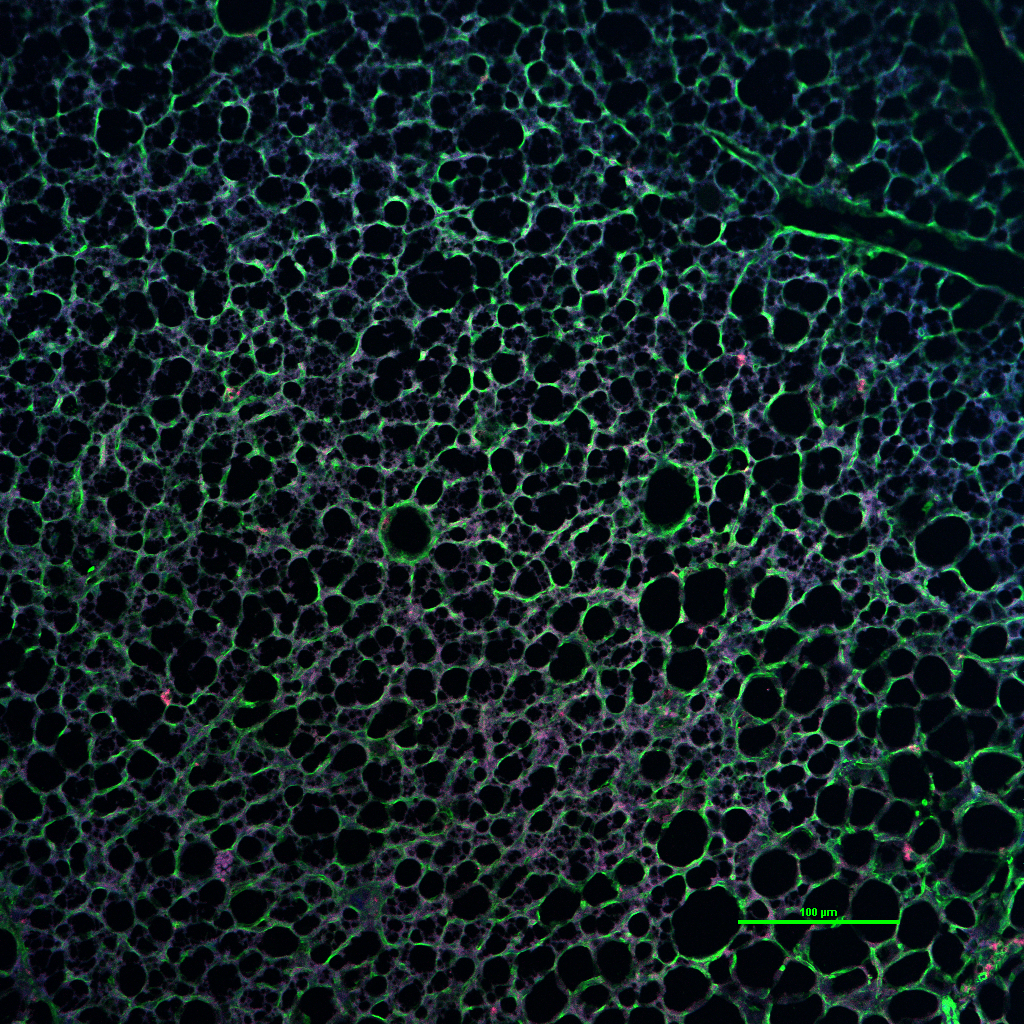

Supplement: Supplementary file 3 — Source data Fig. 1 [file 44318_2024_196_MOESM3_ESM.zip › Figure 1/Figure 1-D/Quantificated image/HFD/no.1/HFD BAT_no.1_RGB_Merge-1.tif]

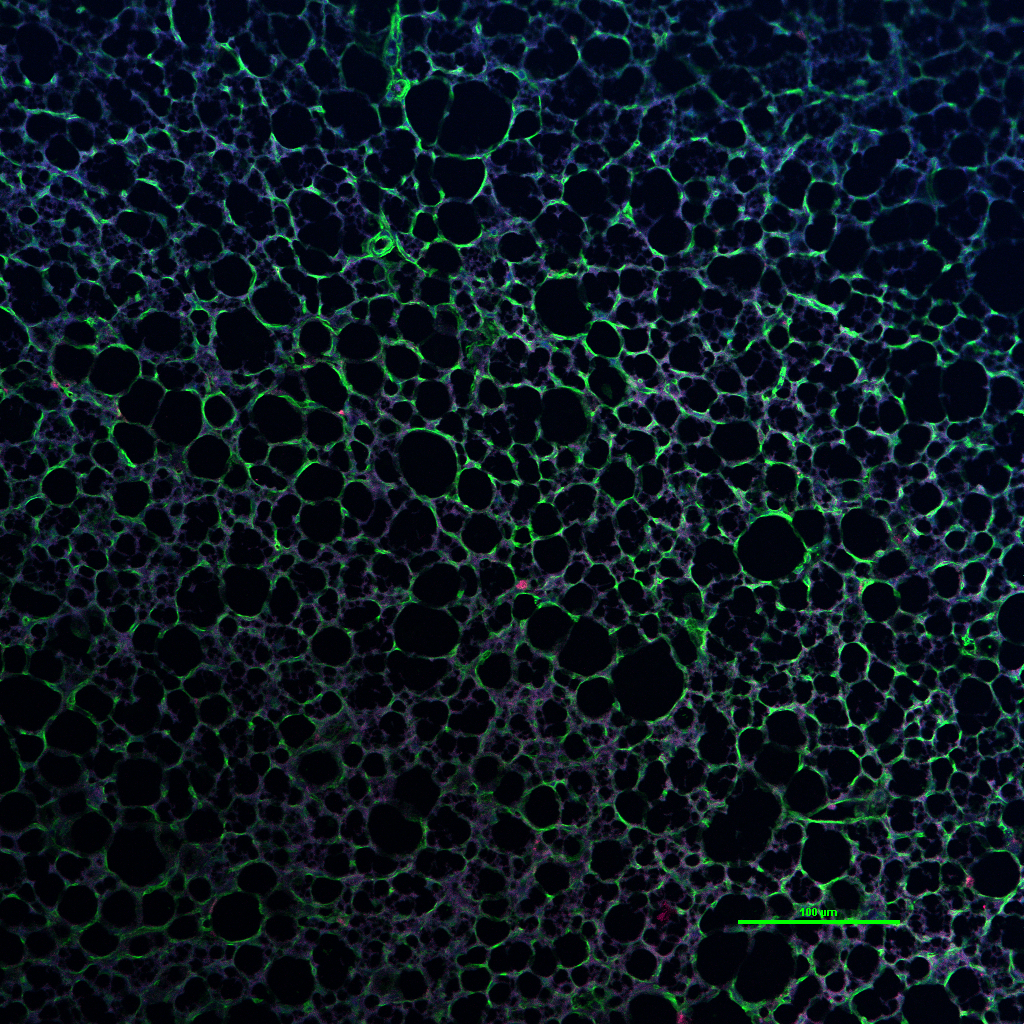

Supplement: Supplementary file 3 — Source data Fig. 1 [file 44318_2024_196_MOESM3_ESM.zip › Figure 1/Figure 1-D/Quantificated image/HFD/no.1/HFD BAT_no.1_RGB_Merge-2.tif]

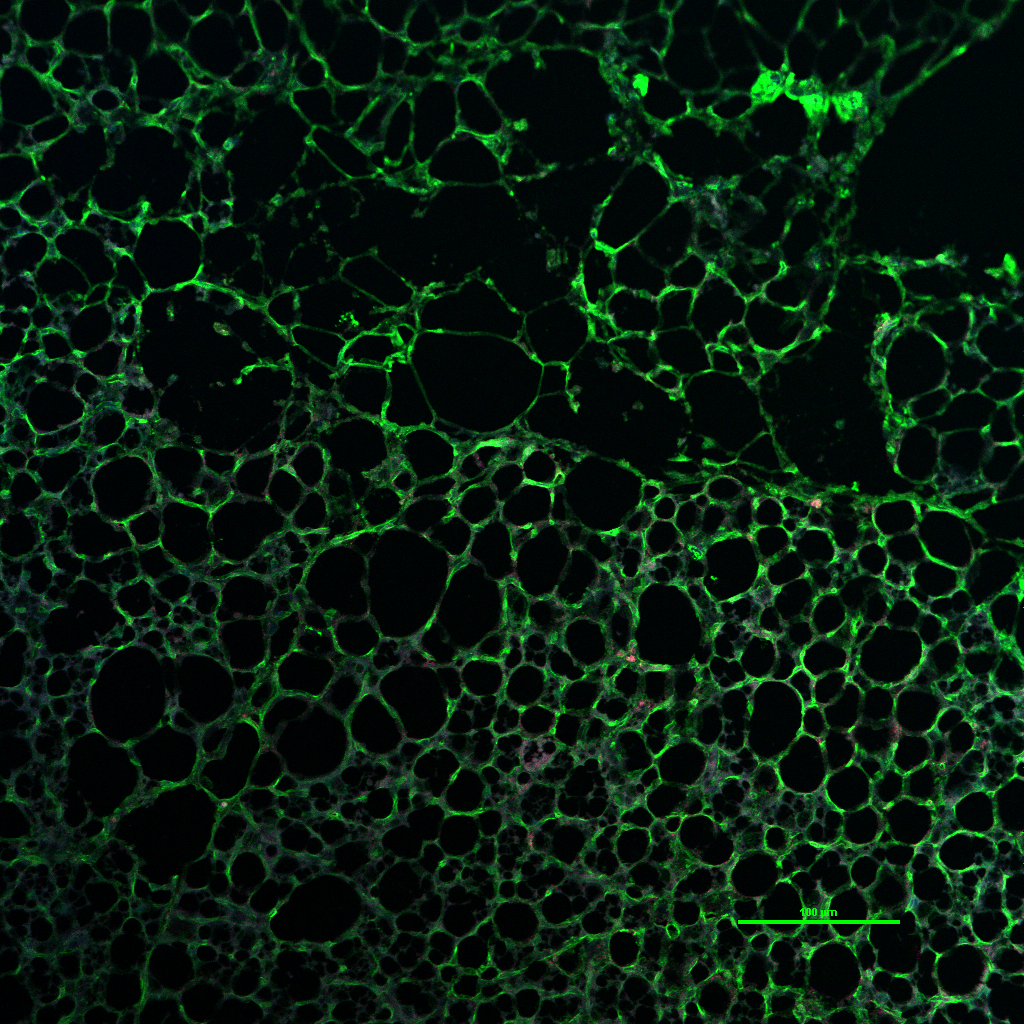

Supplement: Supplementary file 3 — Source data Fig. 1 [file 44318_2024_196_MOESM3_ESM.zip › Figure 1/Figure 1-D/Quantificated image/HFD/no.1/HFD BAT_no.1_RGB_Merge-3.tif]

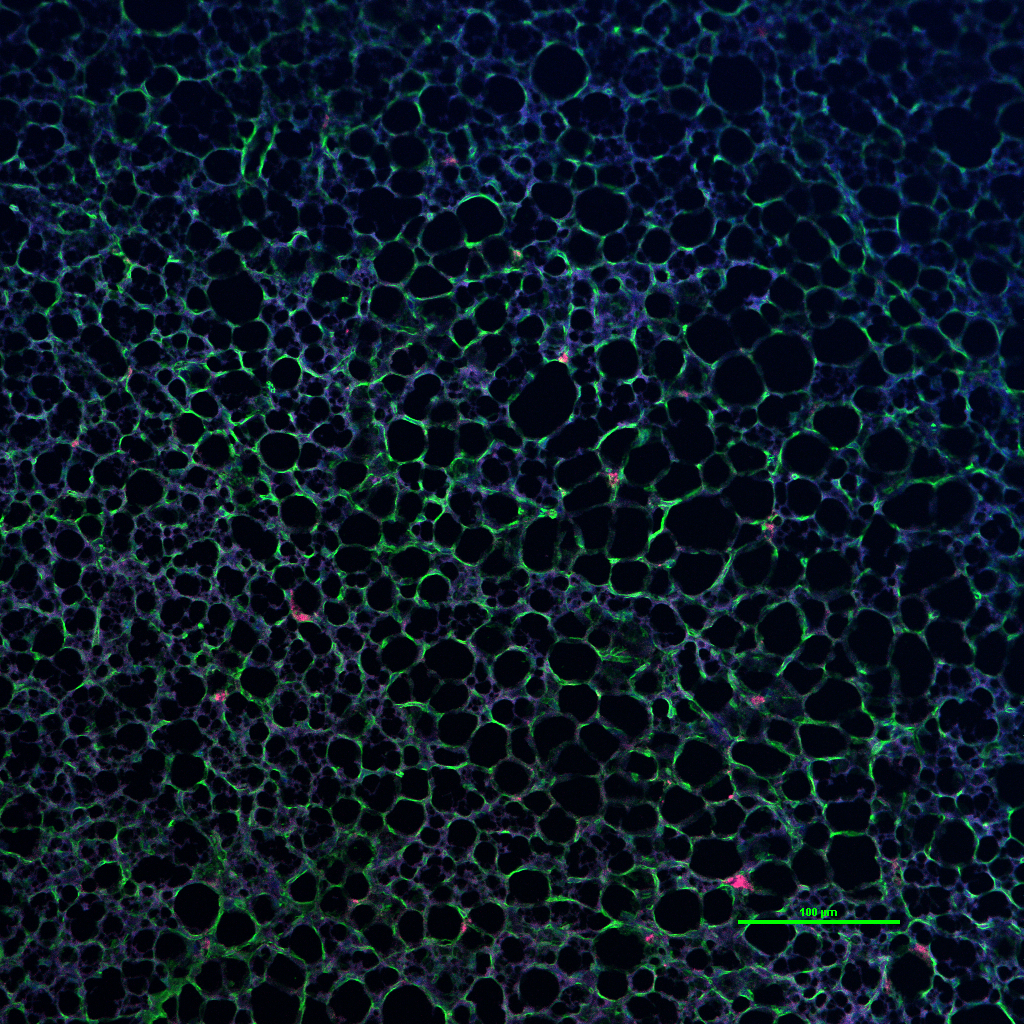

Supplement: Supplementary file 3 — Source data Fig. 1 [file 44318_2024_196_MOESM3_ESM.zip › Figure 1/Figure 1-D/Quantificated image/HFD/no.1/HFD BAT_no.1_RGB_Merge-4.tif]

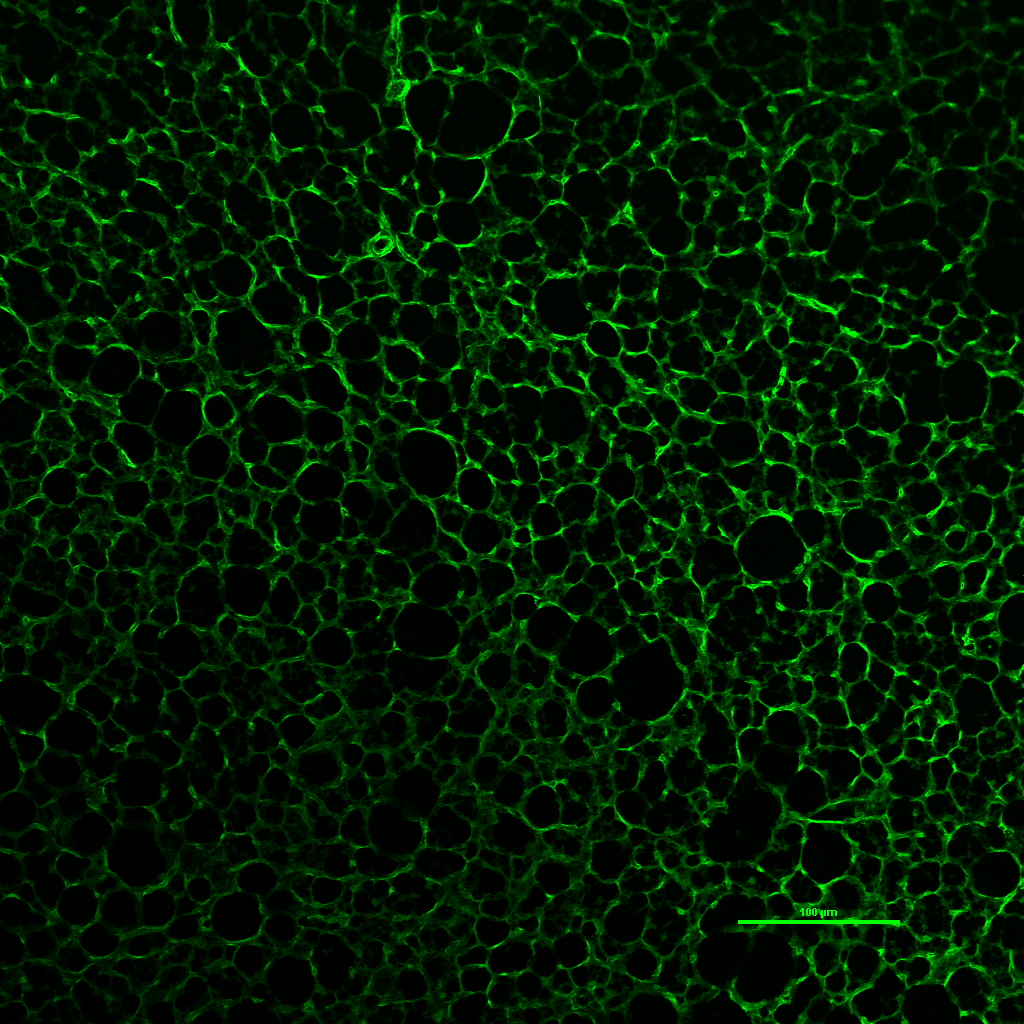

Supplement: Supplementary file 3 — Source data Fig. 1 [file 44318_2024_196_MOESM3_ESM.zip › Figure 1/Figure 1-D/Quantificated image/HFD/no.1/HFD BAT_no.1_RGB_WGA lectin_FITC-2.tif]

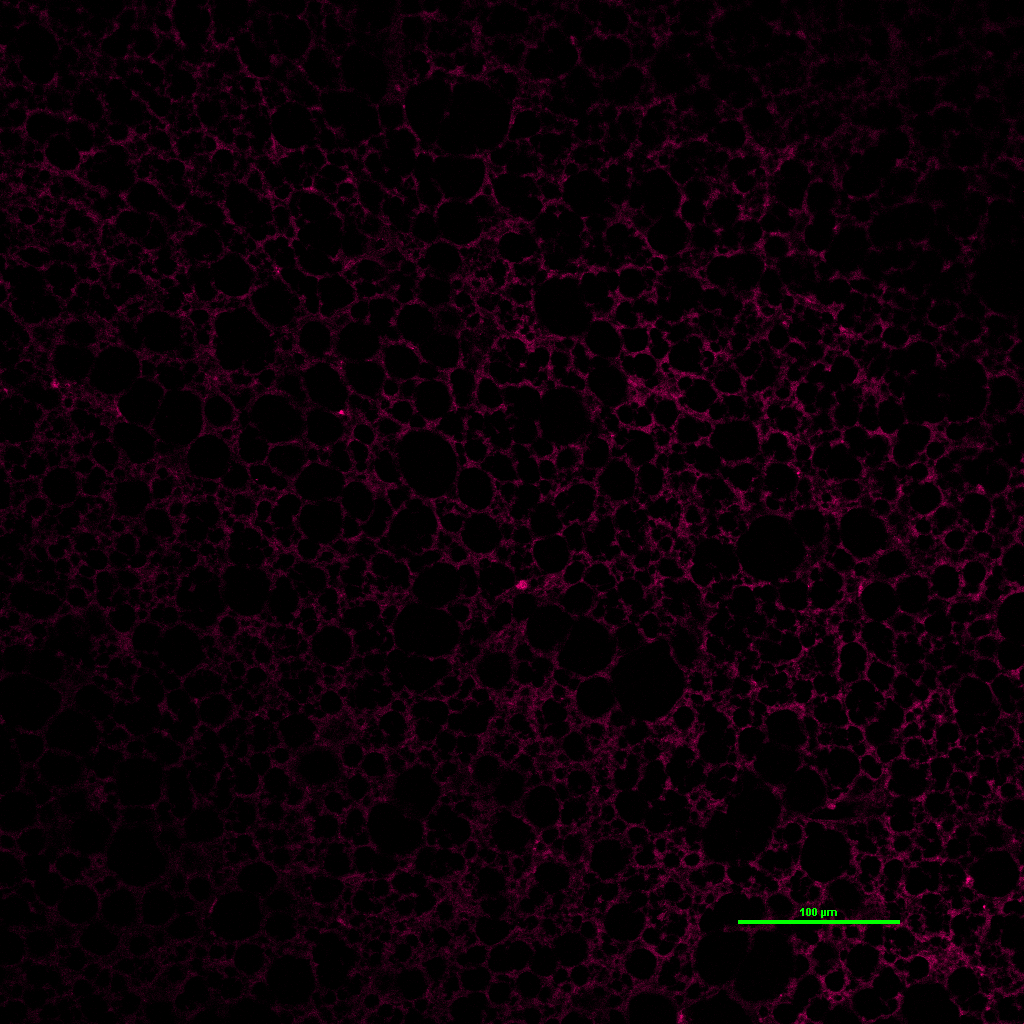

Supplement: Supplementary file 3 — Source data Fig. 1 [file 44318_2024_196_MOESM3_ESM.zip › Figure 1/Figure 1-D/Quantificated image/HFD/no.1/HFD BAT_no.1_RGB_PCPE-1_Cy5-2.tif]

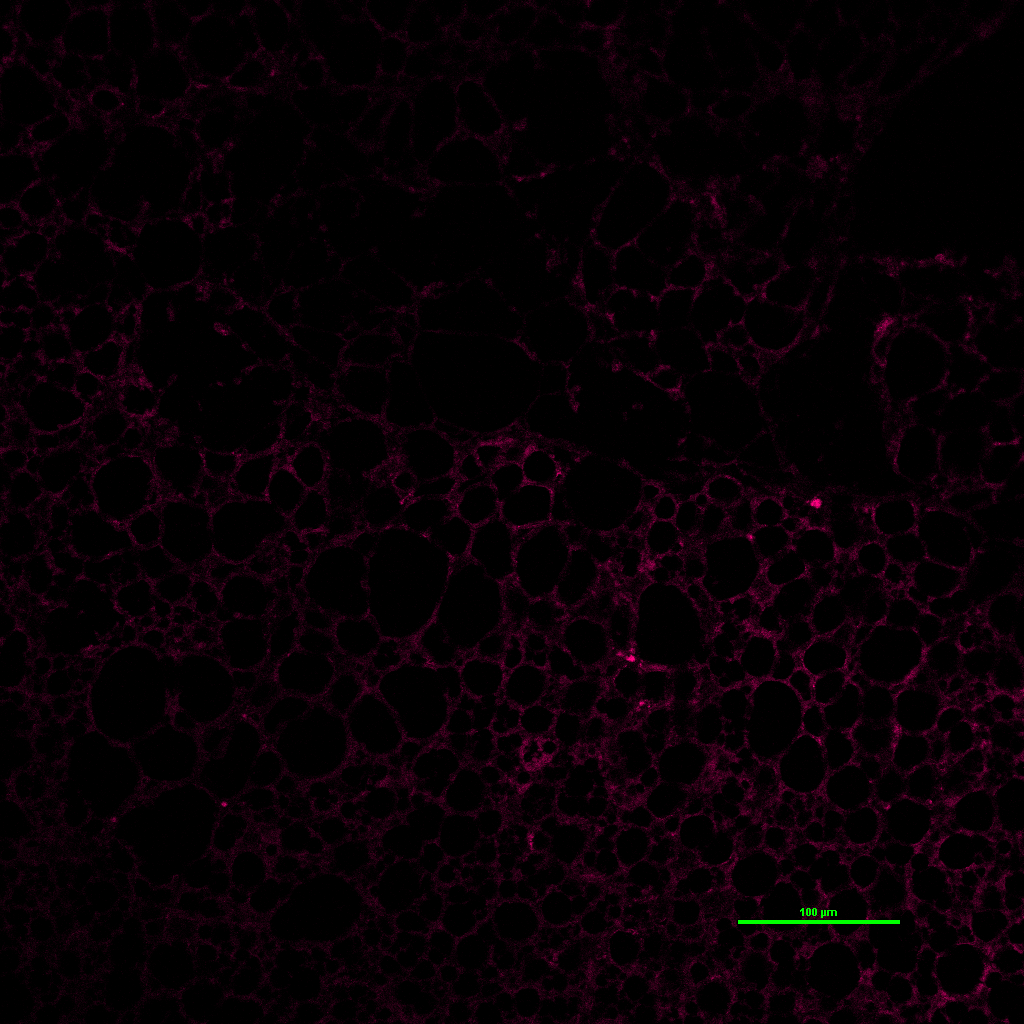

Supplement: Supplementary file 3 — Source data Fig. 1 [file 44318_2024_196_MOESM3_ESM.zip › Figure 1/Figure 1-D/Quantificated image/HFD/no.1/HFD BAT_no.1_RGB_PCPE-1_Cy5-3.tif]

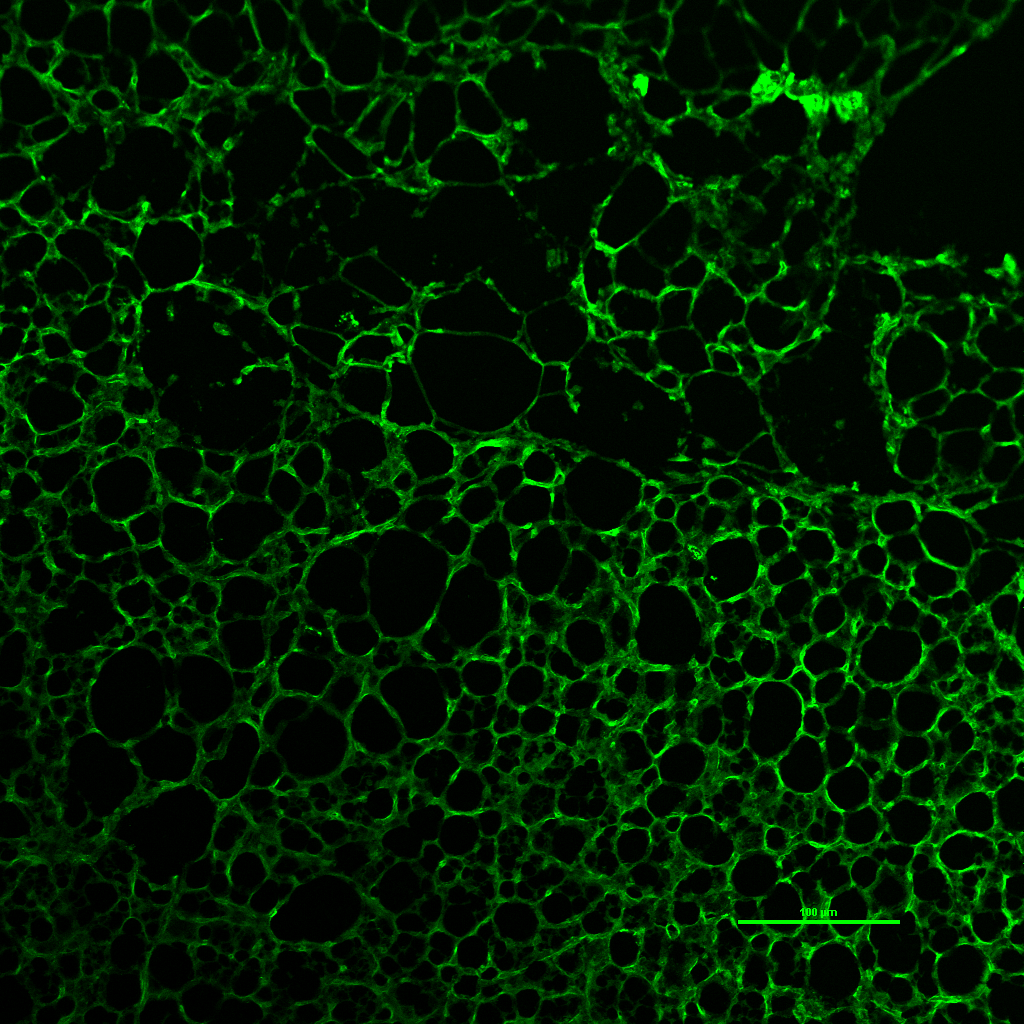

Supplement: Supplementary file 3 — Source data Fig. 1 [file 44318_2024_196_MOESM3_ESM.zip › Figure 1/Figure 1-D/Quantificated image/HFD/no.1/HFD BAT_no.1_RGB_WGA lectin_FITC-3.tif]

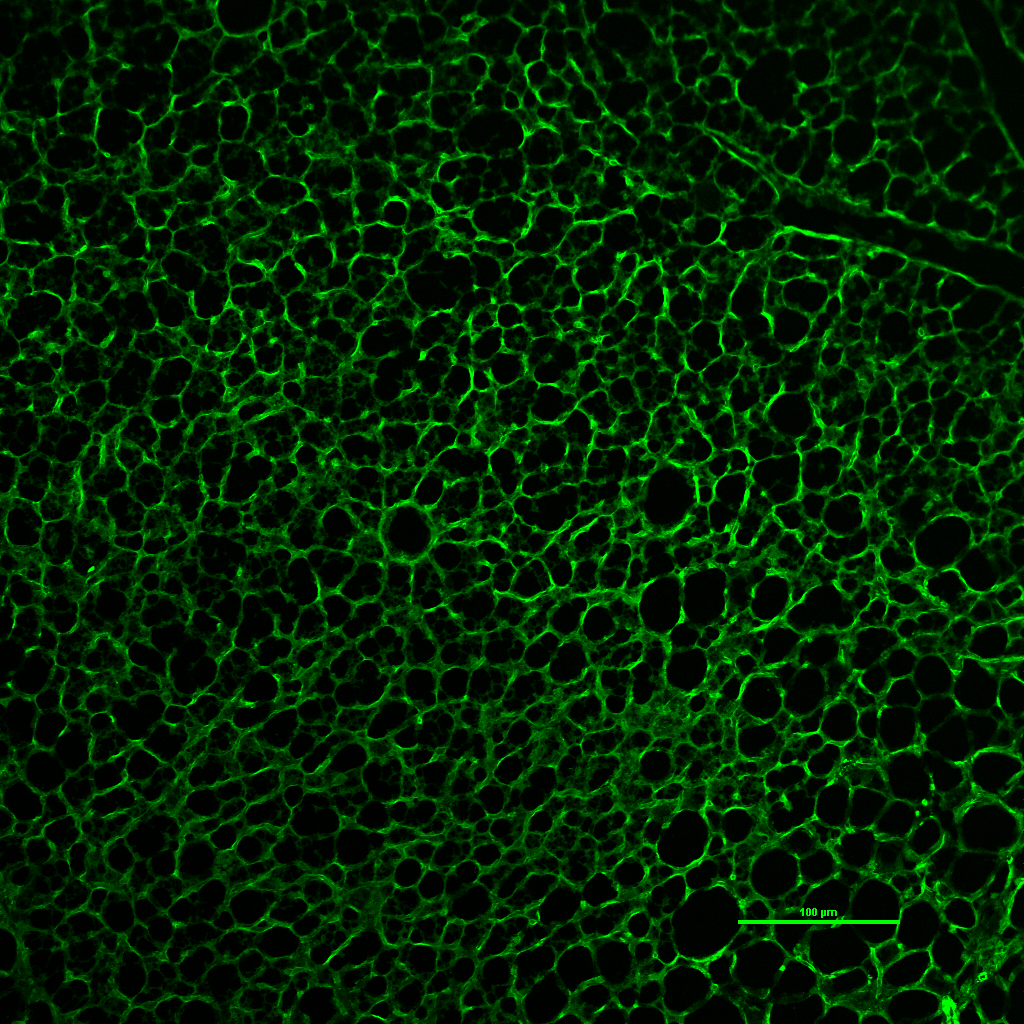

Supplement: Supplementary file 3 — Source data Fig. 1 [file 44318_2024_196_MOESM3_ESM.zip › Figure 1/Figure 1-D/Quantificated image/HFD/no.1/HFD BAT_no.1_RGB_WGA lectin_FITC-1.tif]

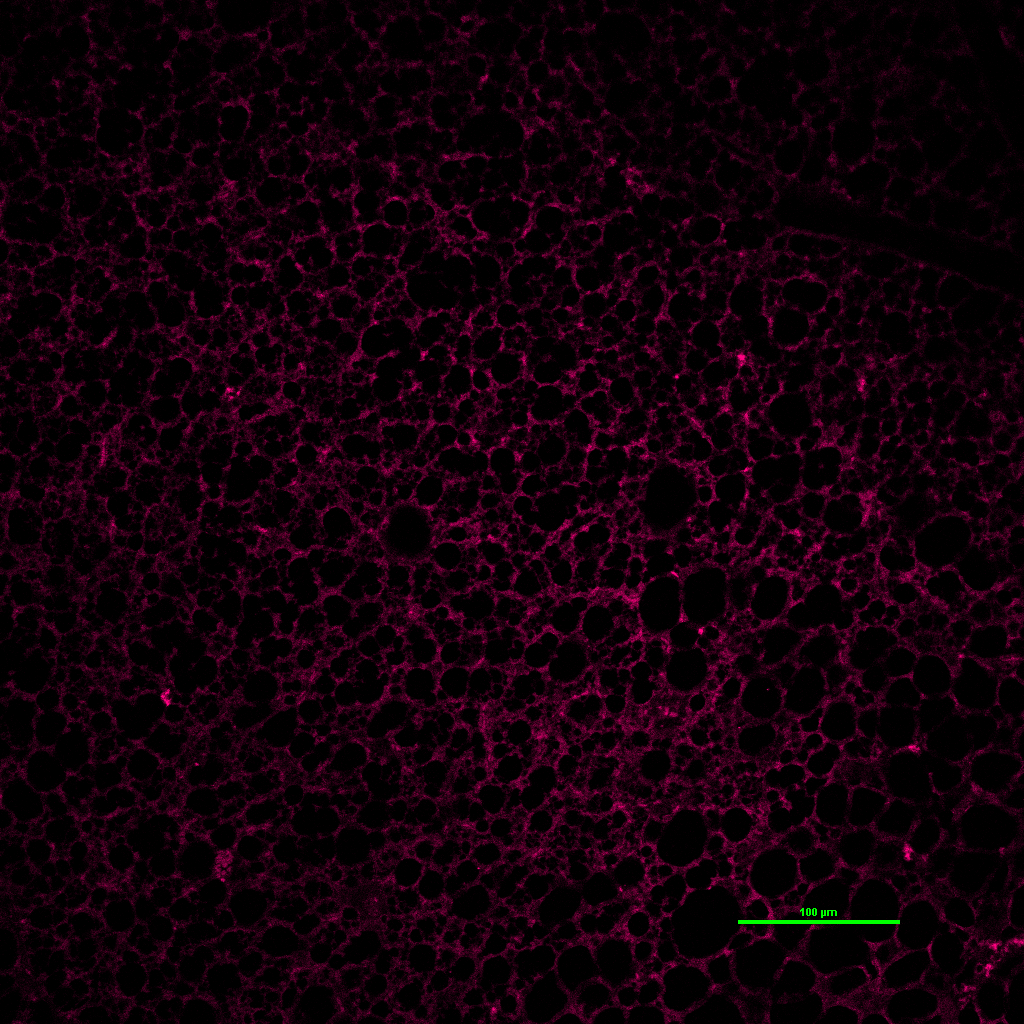

Supplement: Supplementary file 3 — Source data Fig. 1 [file 44318_2024_196_MOESM3_ESM.zip › Figure 1/Figure 1-D/Quantificated image/HFD/no.1/HFD BAT_no.1_RGB_PCPE-1_Cy5-1.tif]

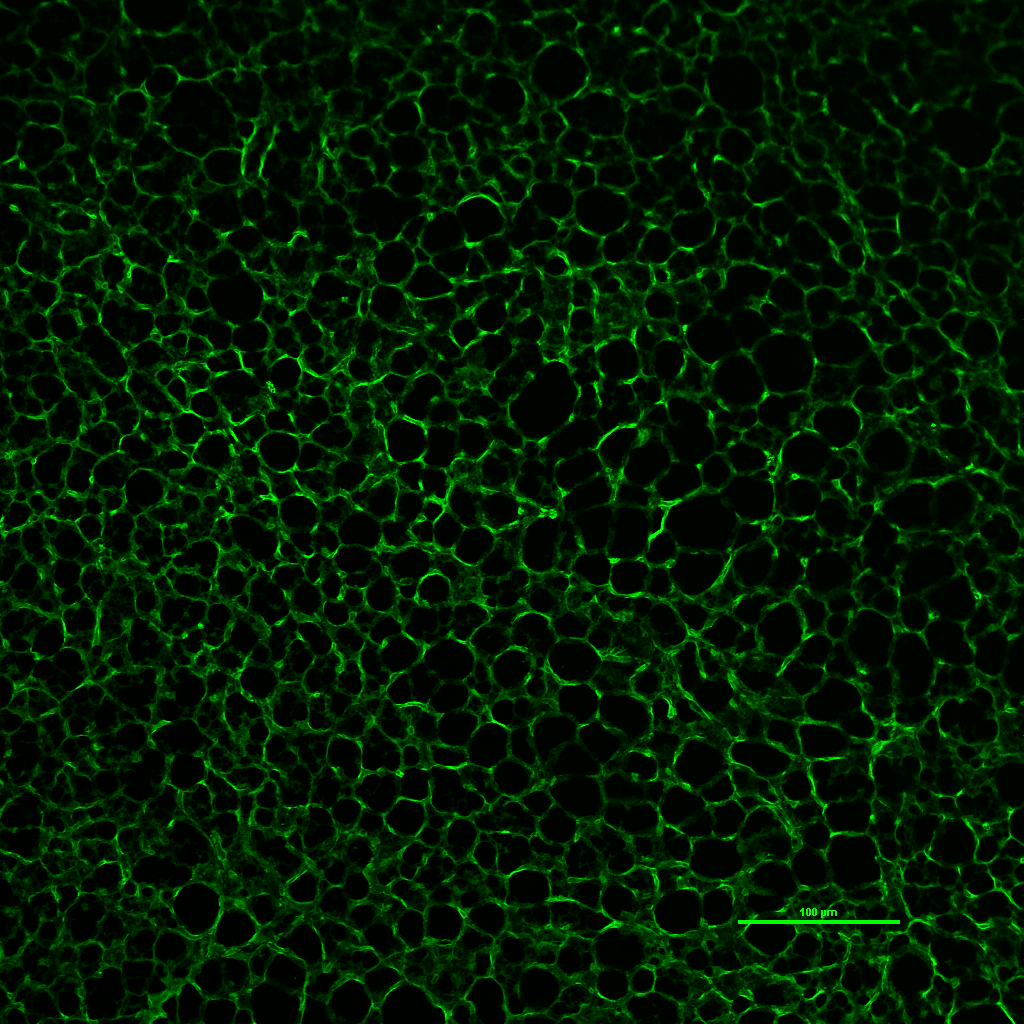

Supplement: Supplementary file 3 — Source data Fig. 1 [file 44318_2024_196_MOESM3_ESM.zip › Figure 1/Figure 1-D/Quantificated image/HFD/no.1/HFD BAT_no.1_RGB_WGA lectin_FITC-4.tif]

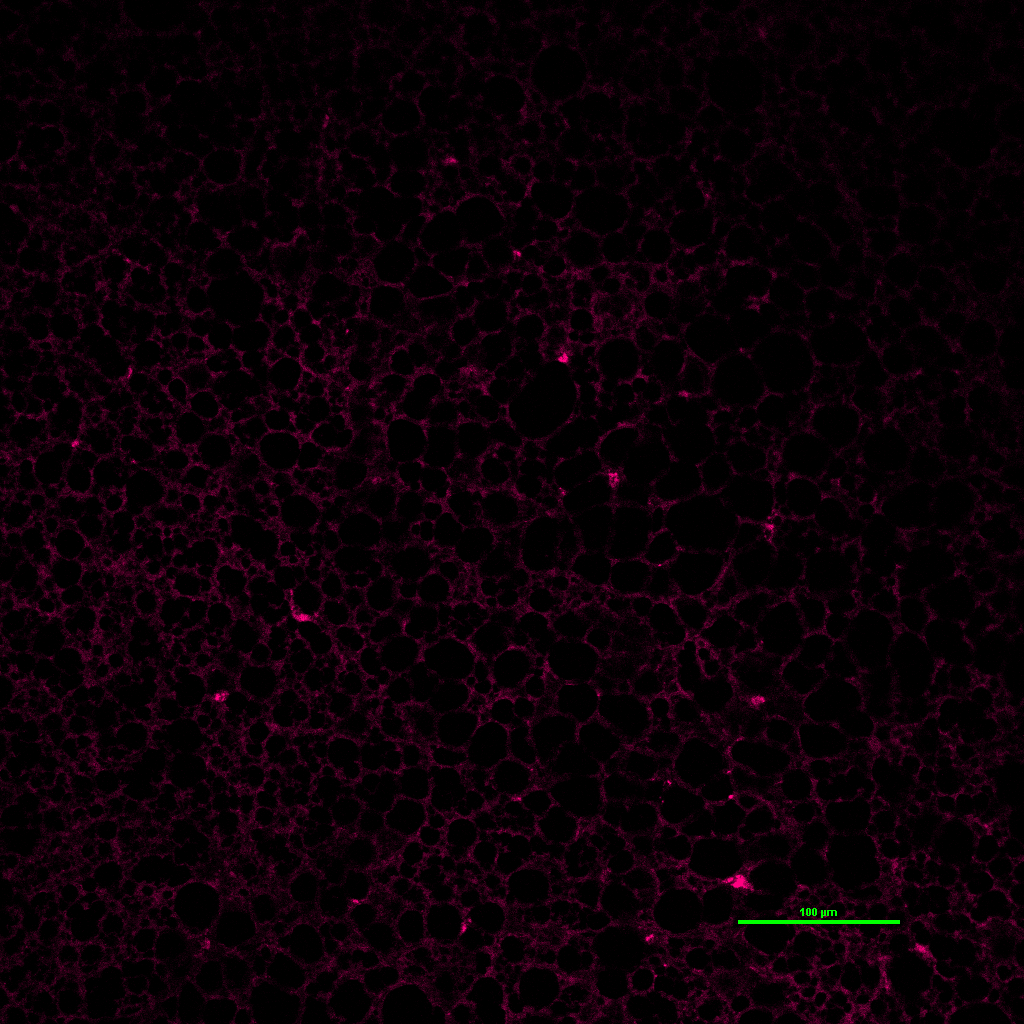

Supplement: Supplementary file 3 — Source data Fig. 1 [file 44318_2024_196_MOESM3_ESM.zip › Figure 1/Figure 1-D/Quantificated image/HFD/no.1/HFD BAT_no.1_RGB_PCPE-1_Cy5-4.tif]

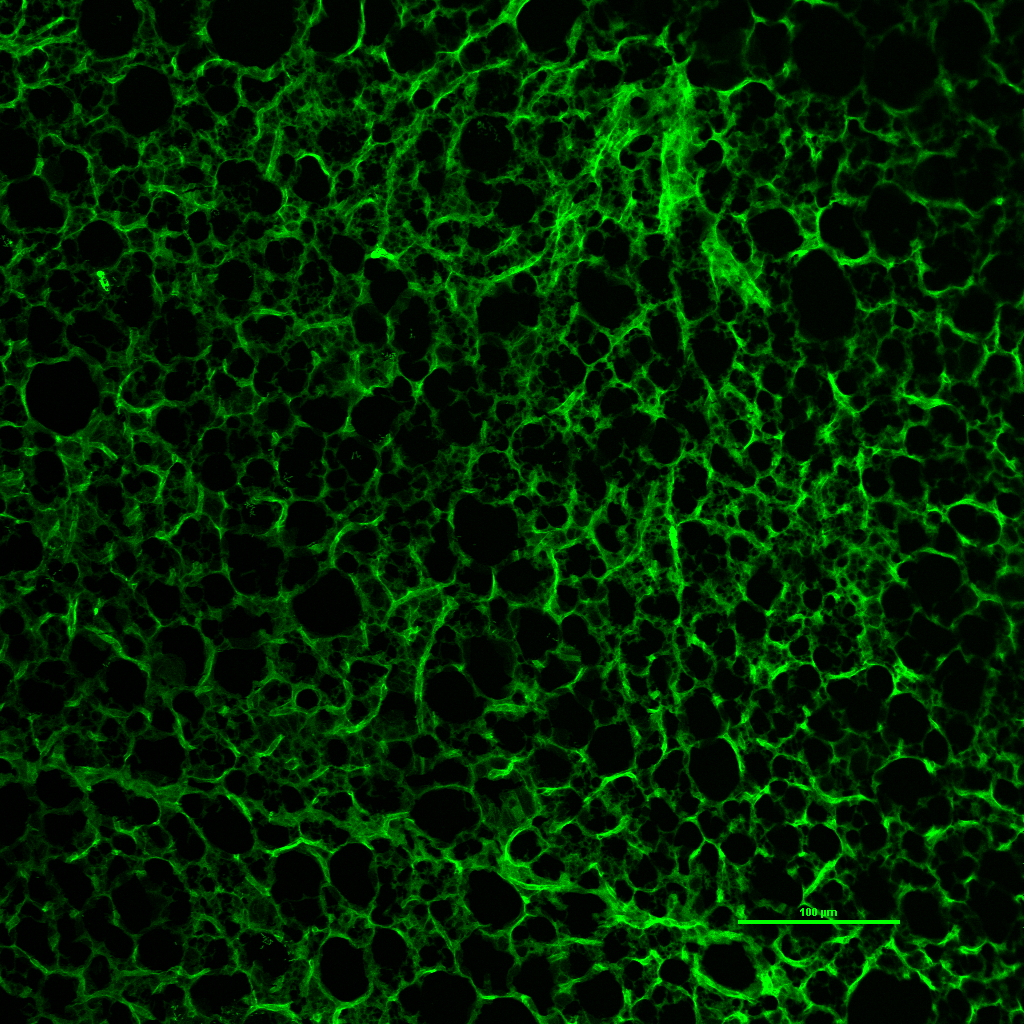

Supplement: Supplementary file 3 — Source data Fig. 1 [file 44318_2024_196_MOESM3_ESM.zip › Figure 1/Figure 1-D/Quantificated image/HFD/no.3/HFD BAT_no.3_RGB_WGA lectin_FITC-2.tif]

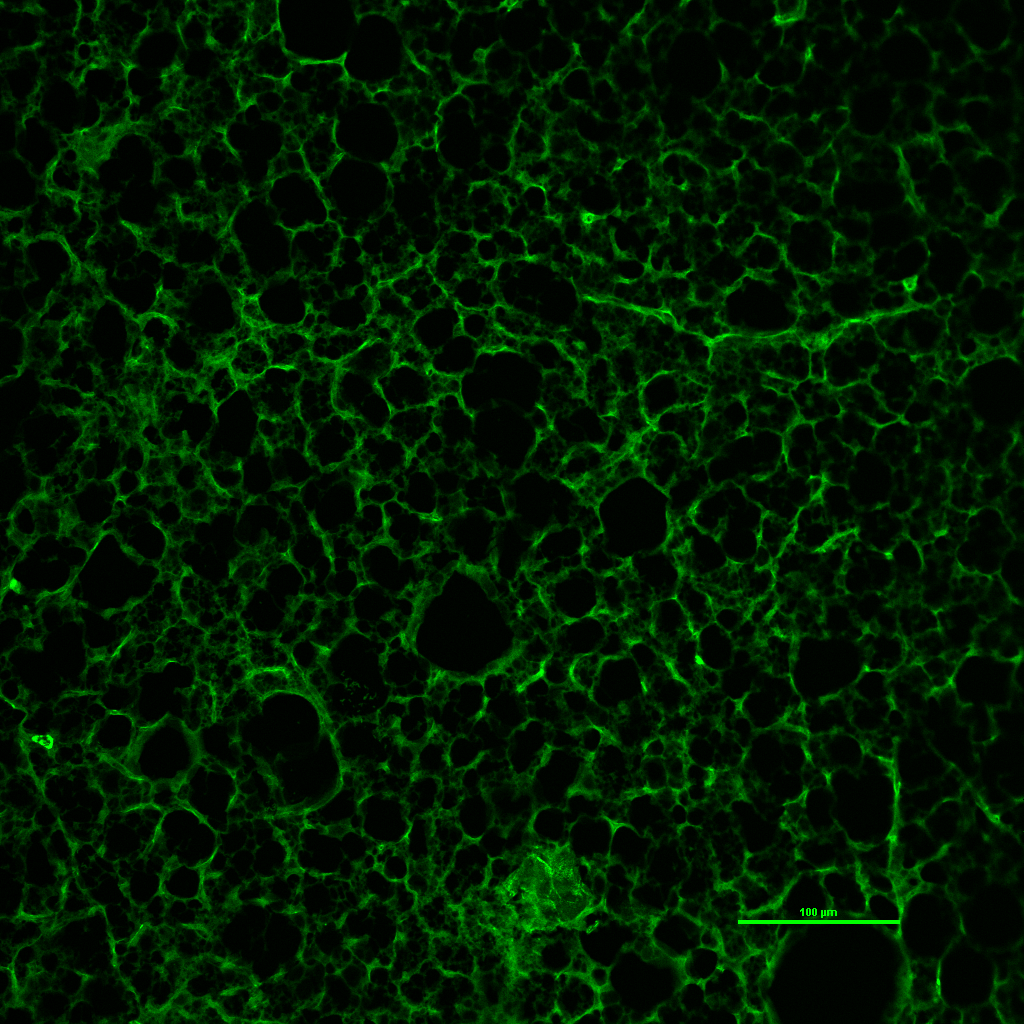

Supplement: Supplementary file 3 — Source data Fig. 1 [file 44318_2024_196_MOESM3_ESM.zip › Figure 1/Figure 1-D/Quantificated image/HFD/no.3/HFD BAT_no.3_RGB_WGA lectin_FITC-3.tif]

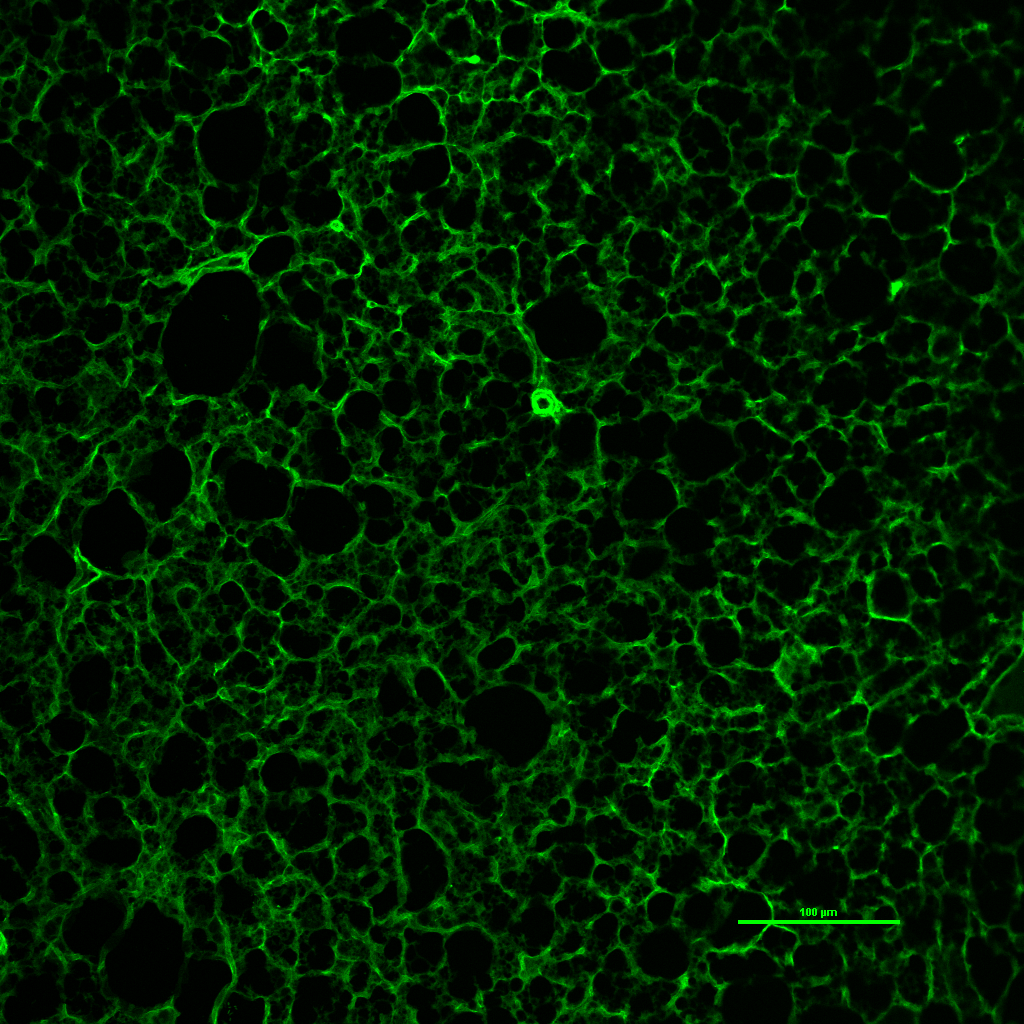

Supplement: Supplementary file 3 — Source data Fig. 1 [file 44318_2024_196_MOESM3_ESM.zip › Figure 1/Figure 1-D/Quantificated image/HFD/no.3/HFD BAT_no.3_RGB_WGA lectin_FITC-1.tif]

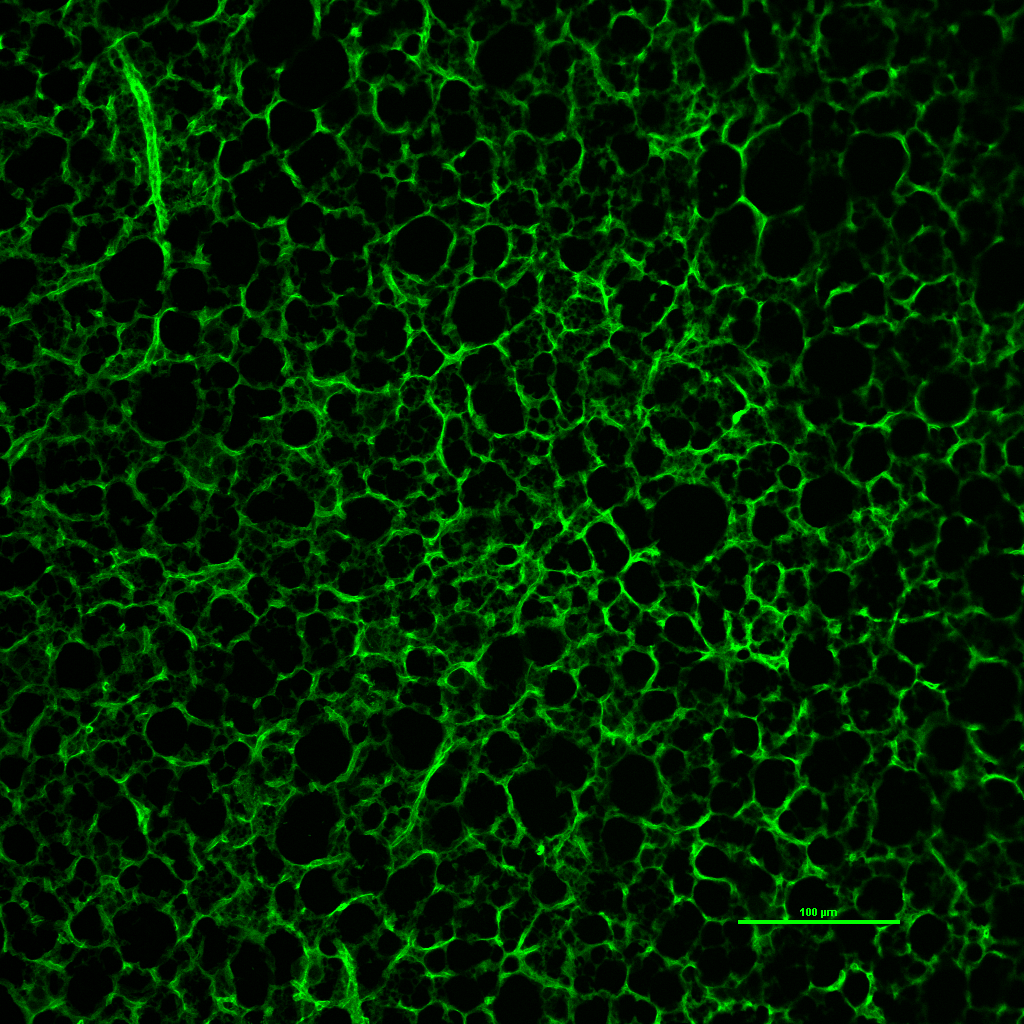

Supplement: Supplementary file 3 — Source data Fig. 1 [file 44318_2024_196_MOESM3_ESM.zip › Figure 1/Figure 1-D/Quantificated image/HFD/no.3/HFD BAT_no.3_RGB_WGA lectin_FITC-4.tif]

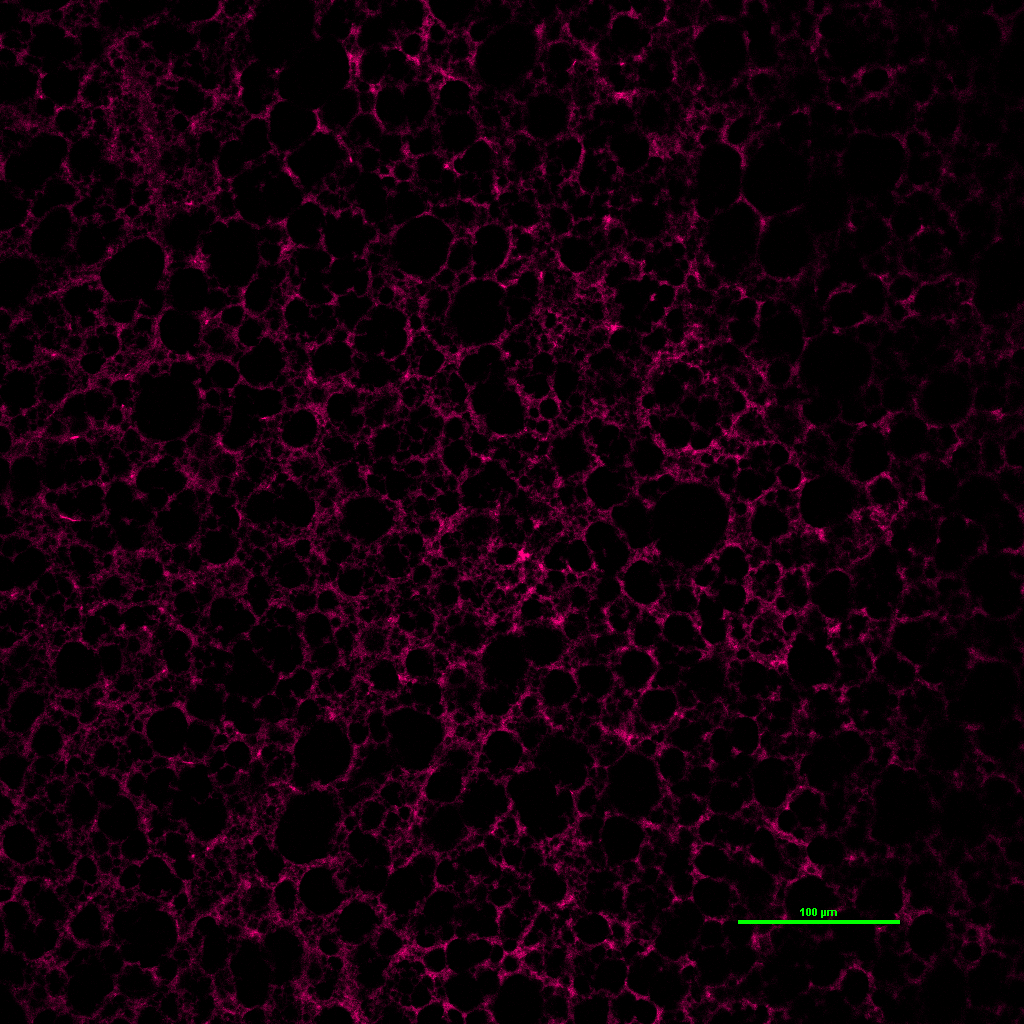

Supplement: Supplementary file 3 — Source data Fig. 1 [file 44318_2024_196_MOESM3_ESM.zip › Figure 1/Figure 1-D/Quantificated image/HFD/no.3/HFD BAT_no.3_RGB_PCPE-1_Cy5-4.tif]

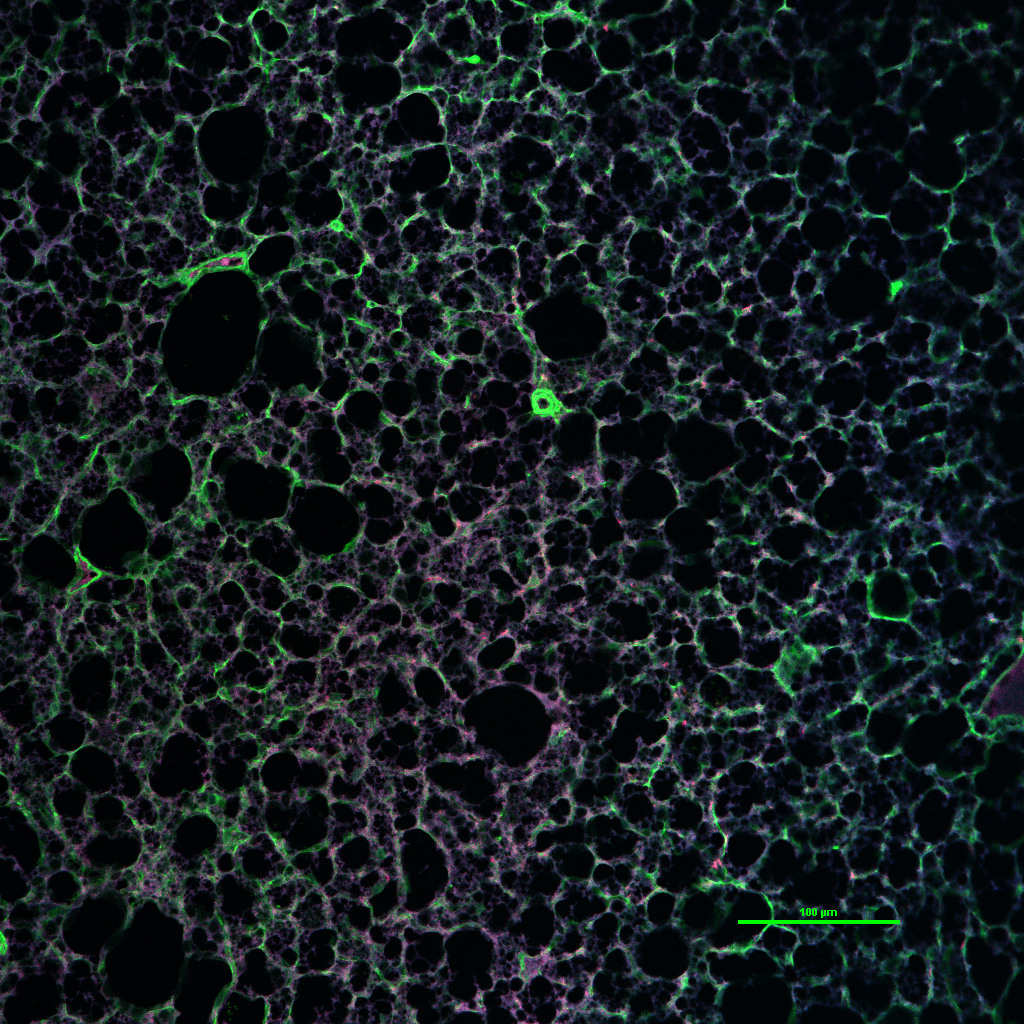

Supplement: Supplementary file 3 — Source data Fig. 1 [file 44318_2024_196_MOESM3_ESM.zip › Figure 1/Figure 1-D/Quantificated image/HFD/no.3/HFD BAT_no.3_RGB_Merge-1.tif]

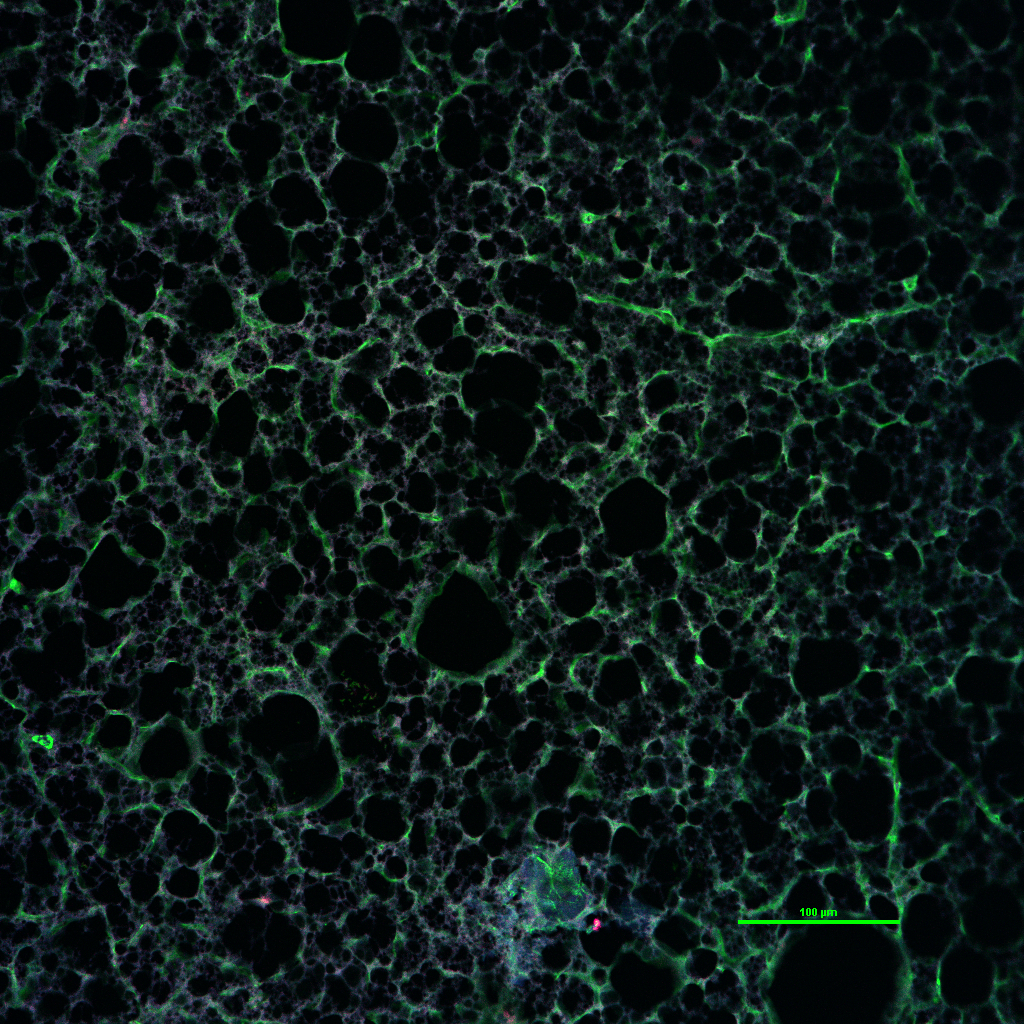

Supplement: Supplementary file 3 — Source data Fig. 1 [file 44318_2024_196_MOESM3_ESM.zip › Figure 1/Figure 1-D/Quantificated image/HFD/no.3/HFD BAT_no.3_RGB_Merge-3.tif]

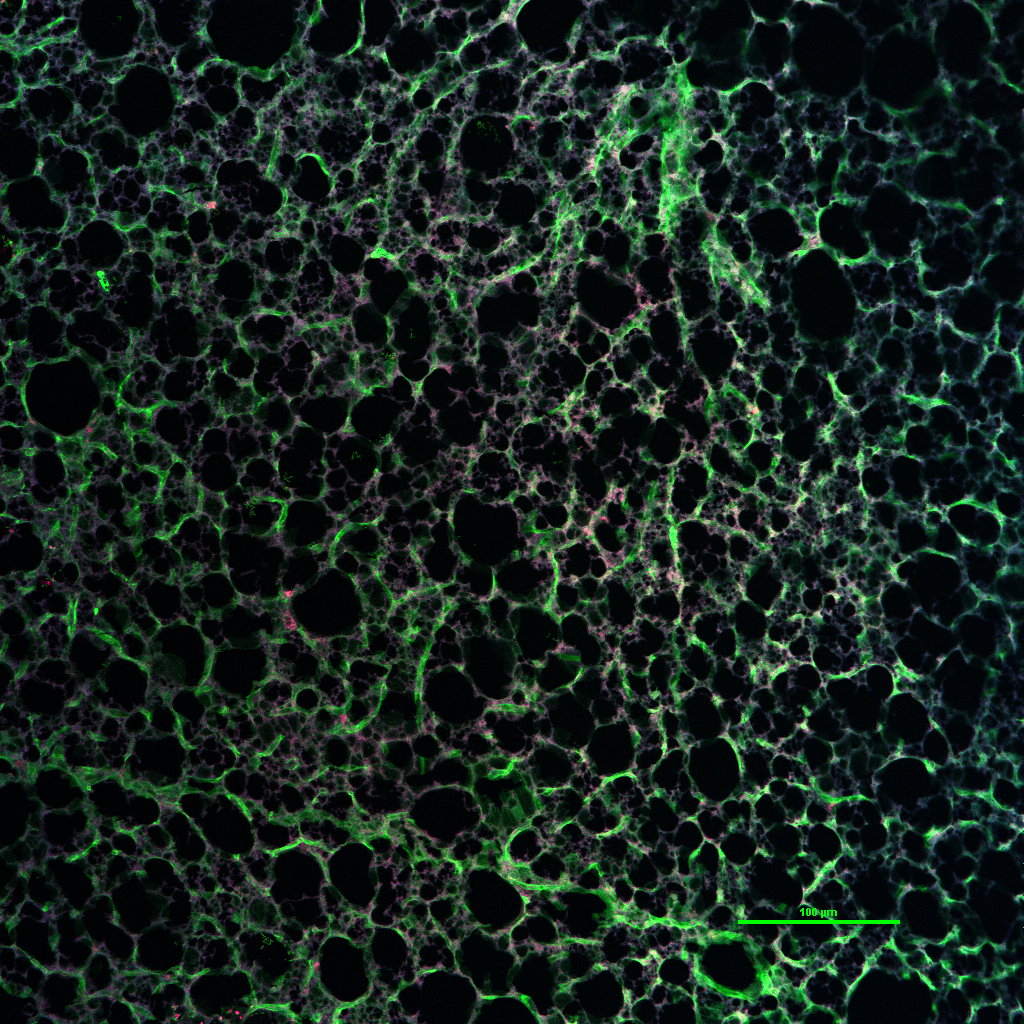

Supplement: Supplementary file 3 — Source data Fig. 1 [file 44318_2024_196_MOESM3_ESM.zip › Figure 1/Figure 1-D/Quantificated image/HFD/no.3/HFD BAT_no.3_RGB_Merge-2.tif]

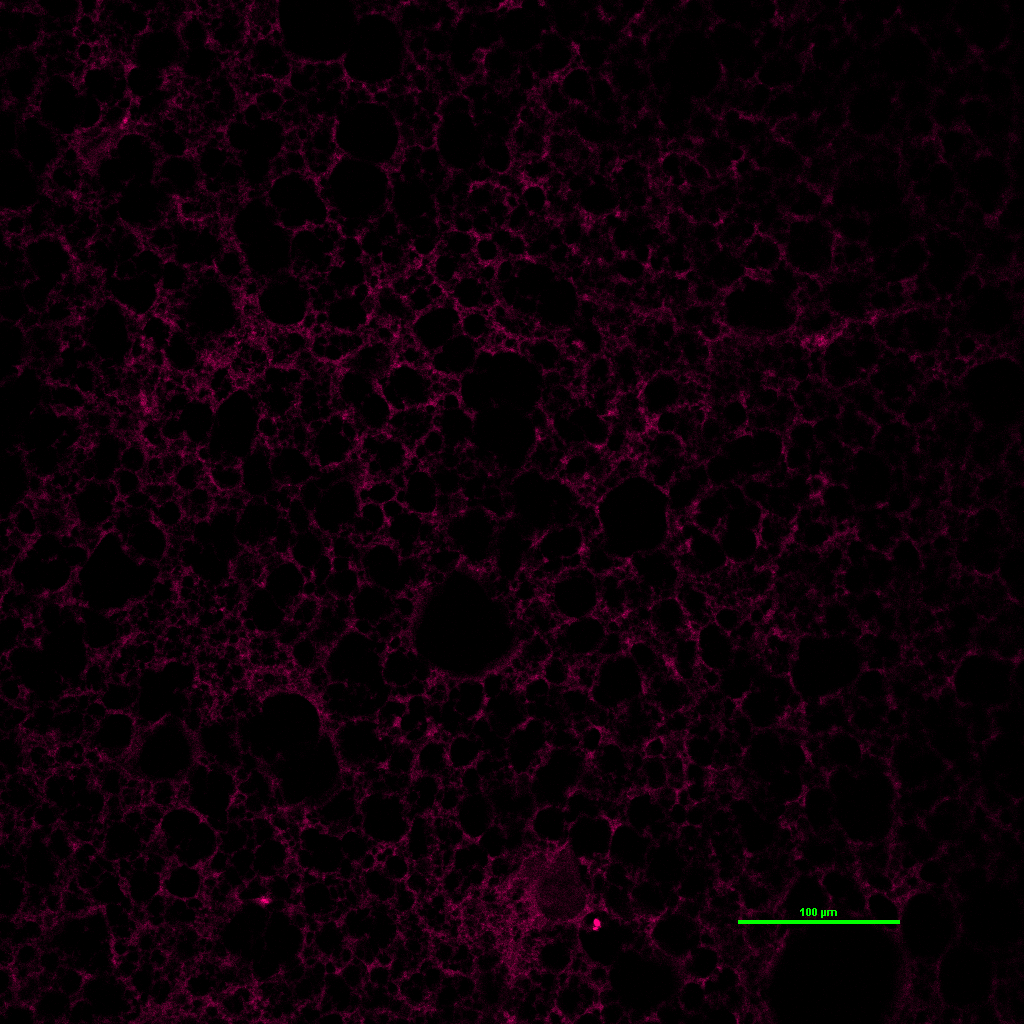

Supplement: Supplementary file 3 — Source data Fig. 1 [file 44318_2024_196_MOESM3_ESM.zip › Figure 1/Figure 1-D/Quantificated image/HFD/no.3/HFD BAT_no.3_RGB_PCPE-1_Cy5-3.tif]

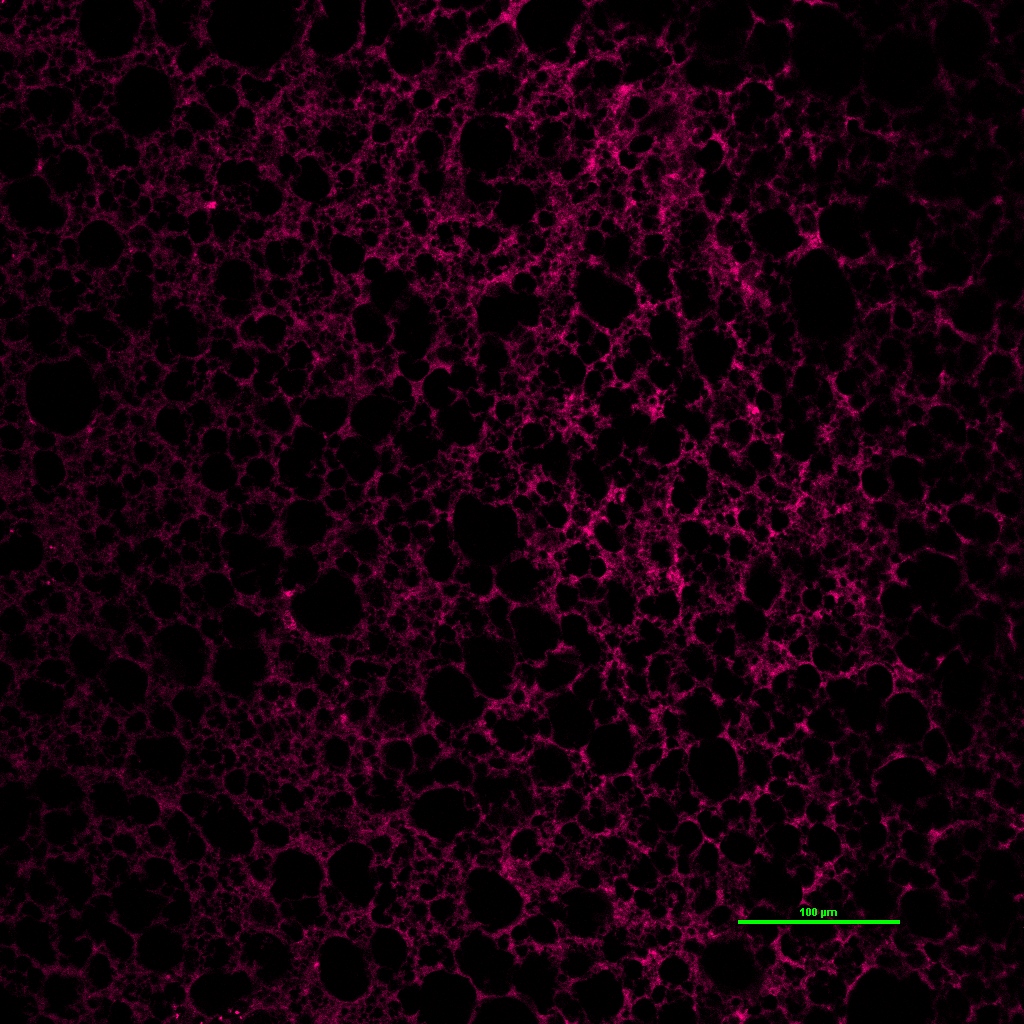

Supplement: Supplementary file 3 — Source data Fig. 1 [file 44318_2024_196_MOESM3_ESM.zip › Figure 1/Figure 1-D/Quantificated image/HFD/no.3/HFD BAT_no.3_RGB_PCPE-1_Cy5-2.tif]

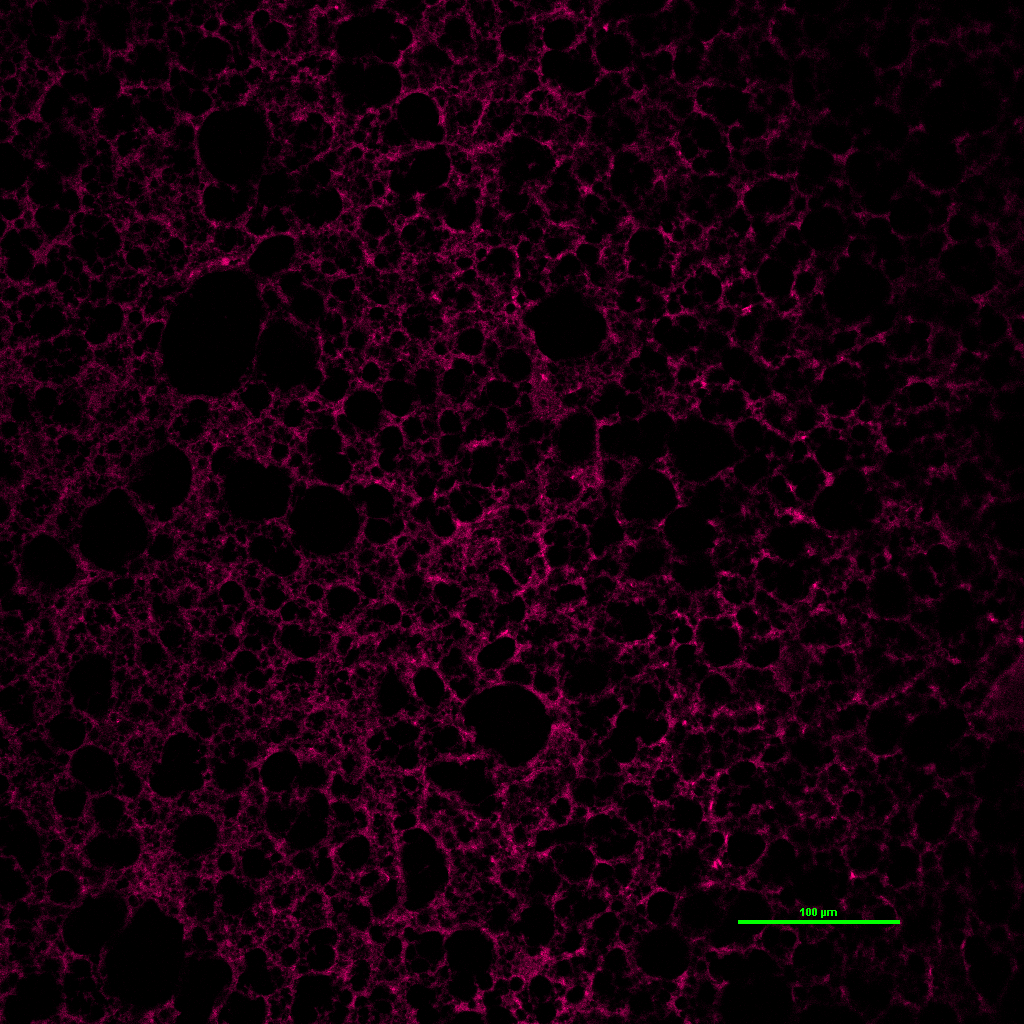

Supplement: Supplementary file 3 — Source data Fig. 1 [file 44318_2024_196_MOESM3_ESM.zip › Figure 1/Figure 1-D/Quantificated image/HFD/no.3/HFD BAT_no.3_RGB_PCPE-1_Cy5-1.tif]

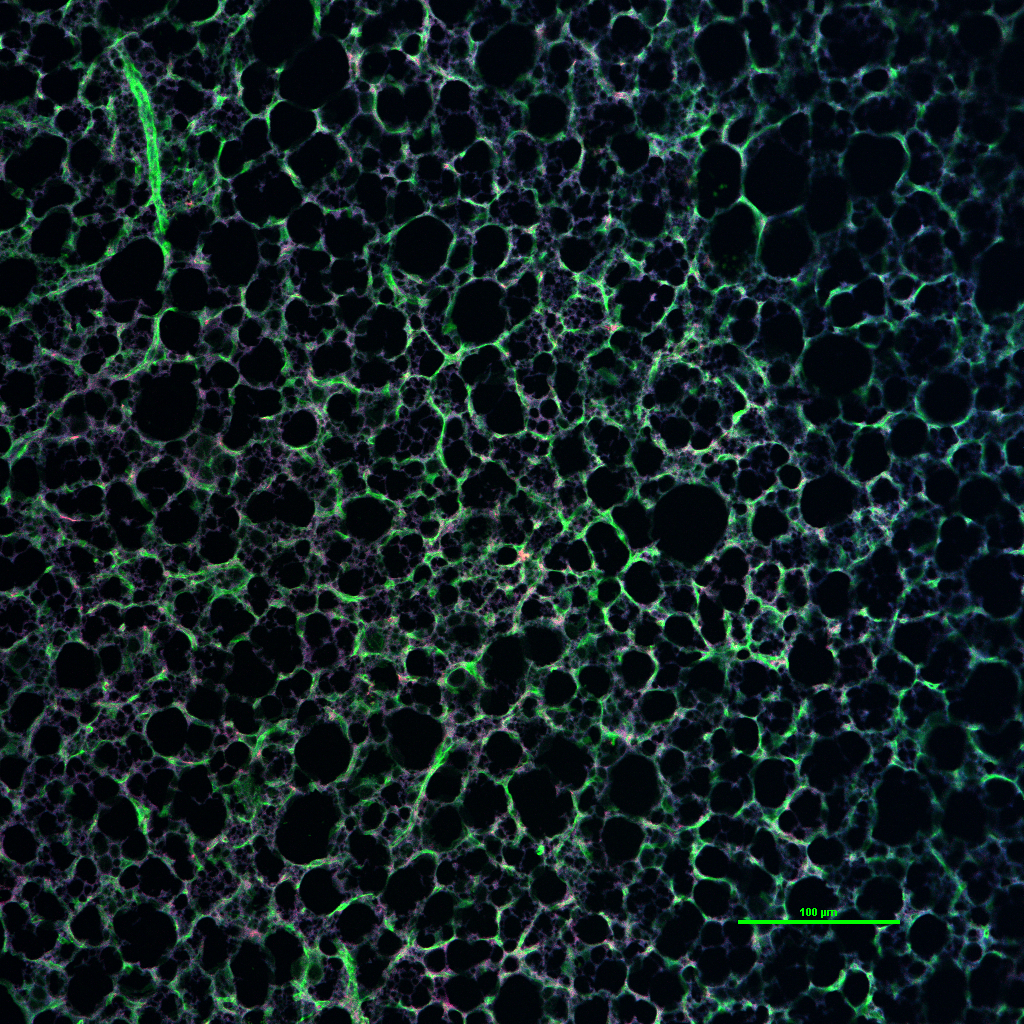

Supplement: Supplementary file 3 — Source data Fig. 1 [file 44318_2024_196_MOESM3_ESM.zip › Figure 1/Figure 1-D/Quantificated image/HFD/no.3/HFD BAT_no.3_RGB_Merge-4.tif]

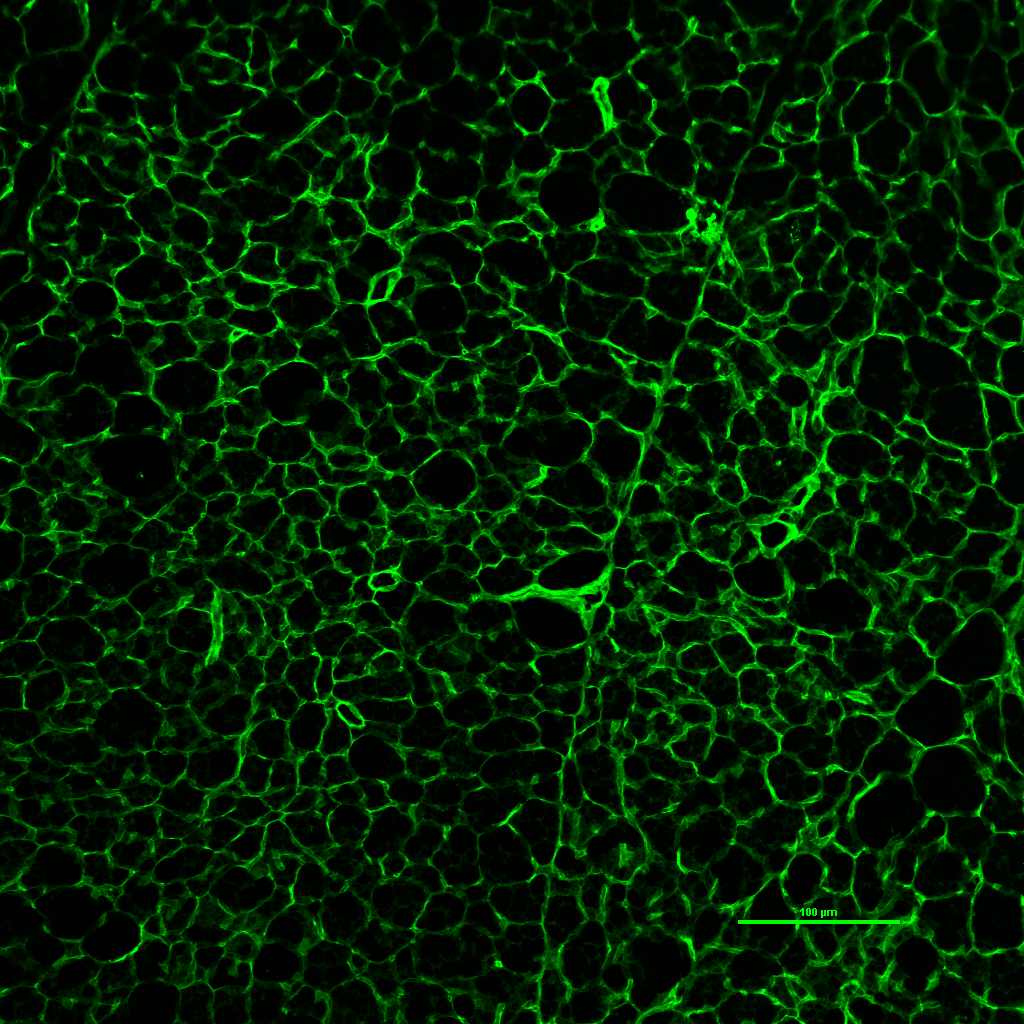

Supplement: Supplementary file 3 — Source data Fig. 1 [file 44318_2024_196_MOESM3_ESM.zip › Figure 1/Figure 1-D/Quantificated image/HFD/no.4/HFD BAT_no.4_RGB_WGA lectin_FITC-4.tif]

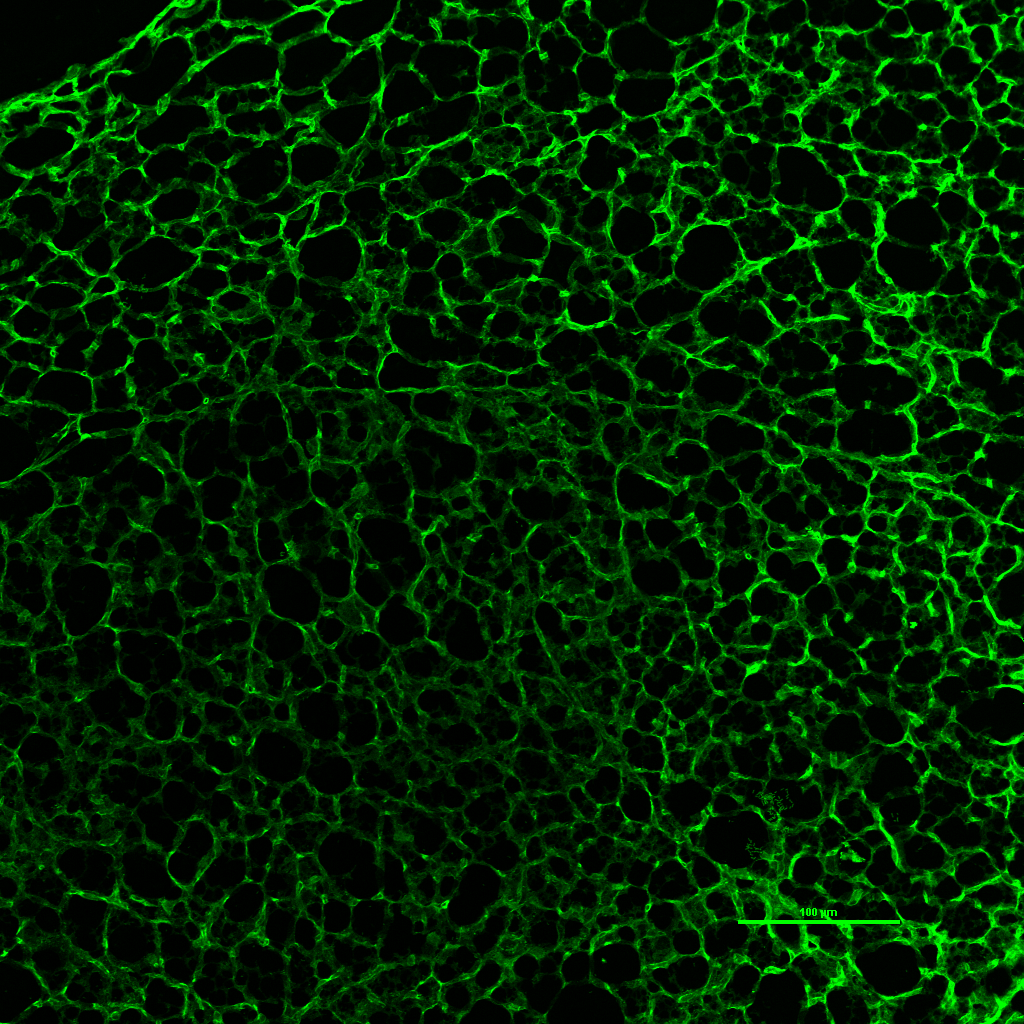

Supplement: Supplementary file 3 — Source data Fig. 1 [file 44318_2024_196_MOESM3_ESM.zip › Figure 1/Figure 1-D/Quantificated image/HFD/no.4/HFD BAT_no.4_RGB_WGA lectin_FITC-1.tif]

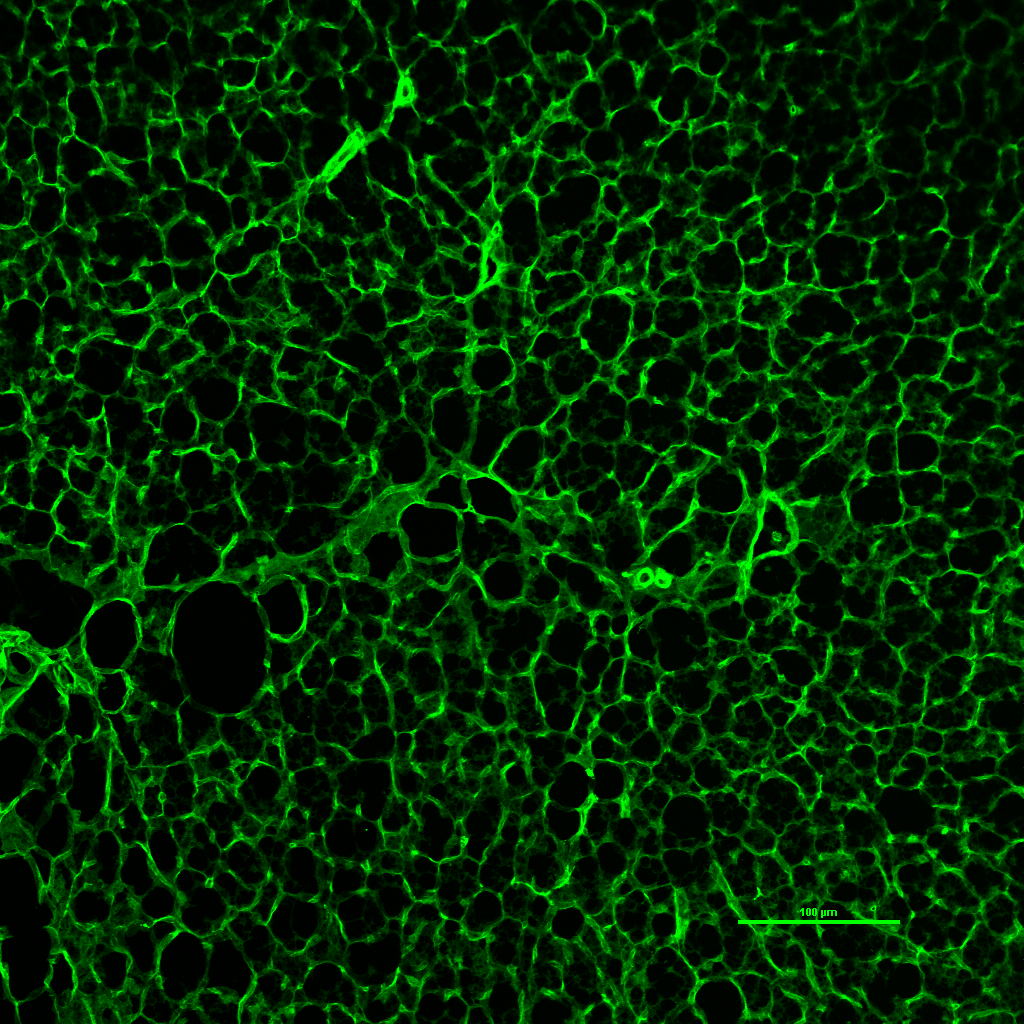

Supplement: Supplementary file 3 — Source data Fig. 1 [file 44318_2024_196_MOESM3_ESM.zip › Figure 1/Figure 1-D/Quantificated image/HFD/no.4/HFD BAT_no.4_RGB_WGA lectin_FITC-3.tif]

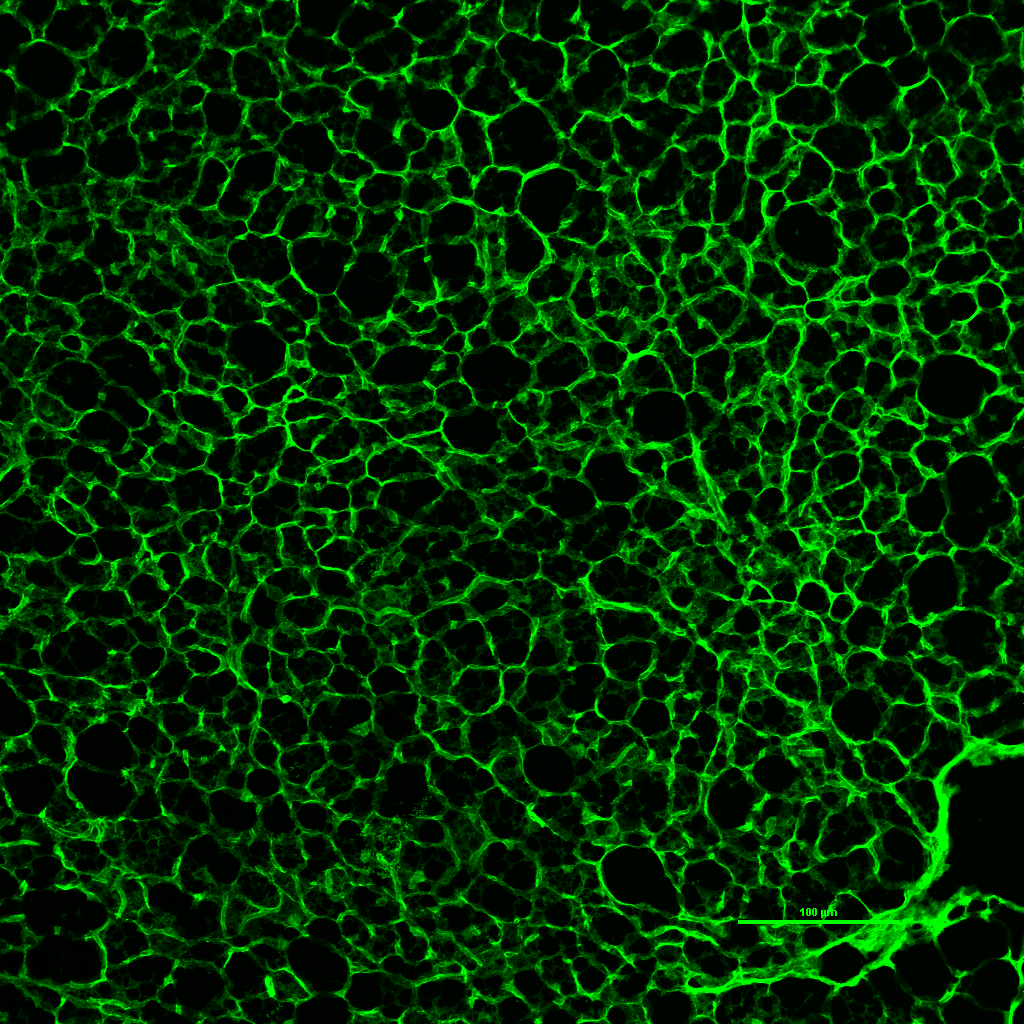

Supplement: Supplementary file 3 — Source data Fig. 1 [file 44318_2024_196_MOESM3_ESM.zip › Figure 1/Figure 1-D/Quantificated image/HFD/no.4/HFD BAT_no.4_RGB_WGA lectin_FITC-2.tif]

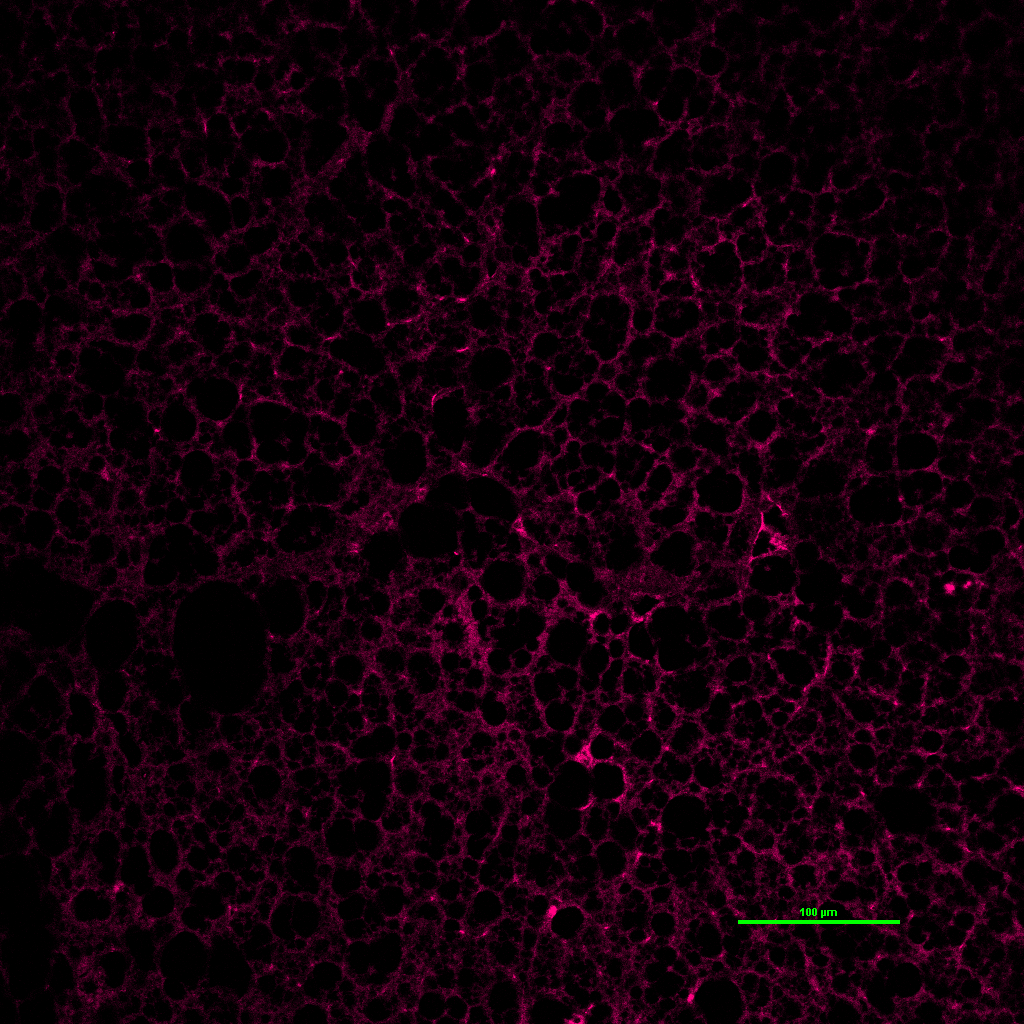

Supplement: Supplementary file 3 — Source data Fig. 1 [file 44318_2024_196_MOESM3_ESM.zip › Figure 1/Figure 1-D/Quantificated image/HFD/no.4/HFD BAT_no.4_RGB_PCPE-1_Cy5-3.tif]

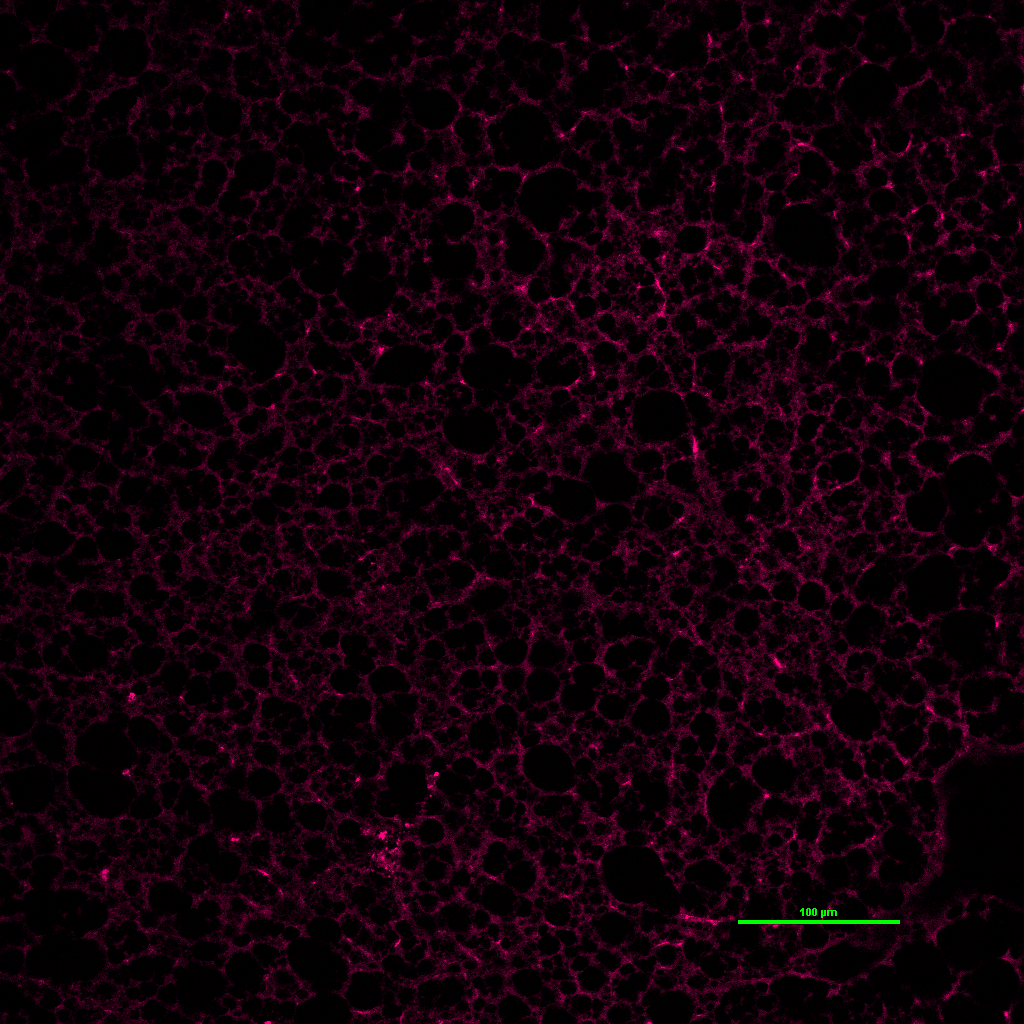

Supplement: Supplementary file 3 — Source data Fig. 1 [file 44318_2024_196_MOESM3_ESM.zip › Figure 1/Figure 1-D/Quantificated image/HFD/no.4/HFD BAT_no.4_RGB_PCPE-1_Cy5-2.tif]

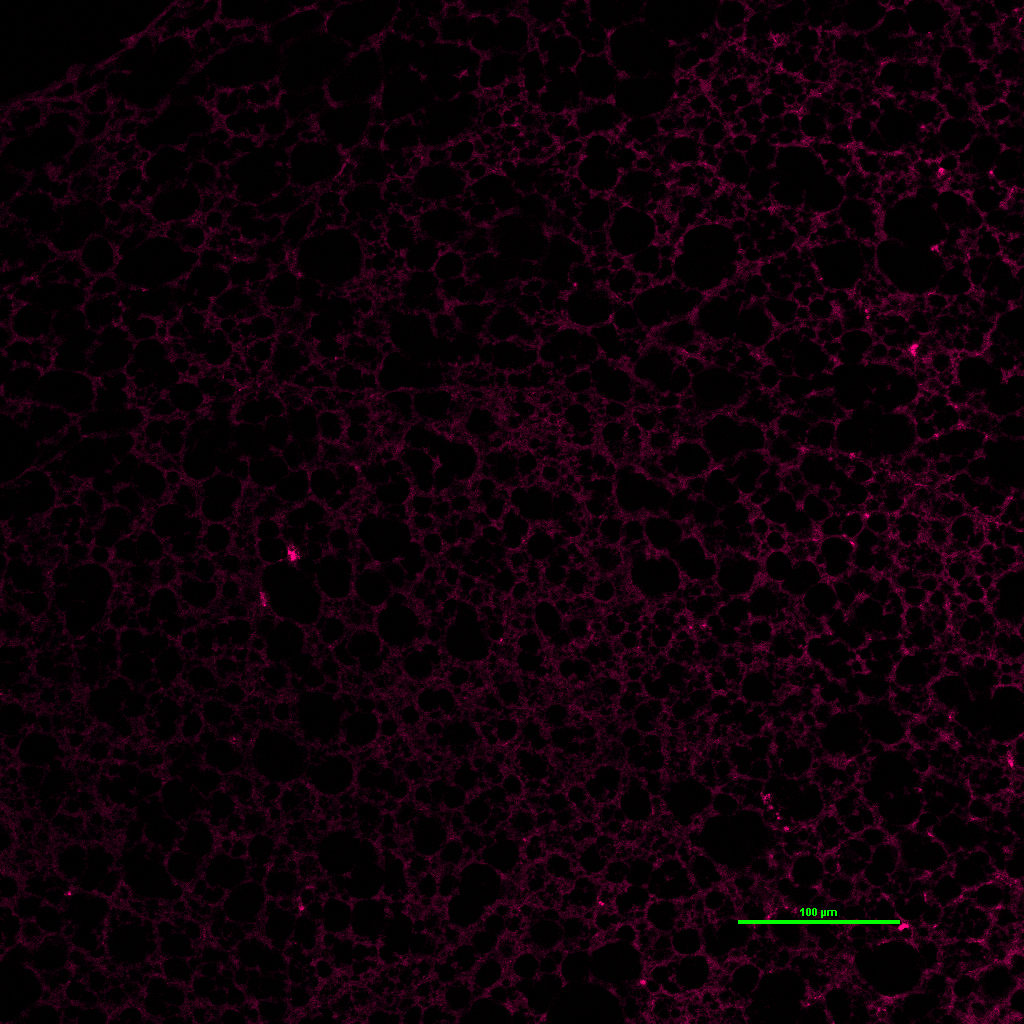

Supplement: Supplementary file 3 — Source data Fig. 1 [file 44318_2024_196_MOESM3_ESM.zip › Figure 1/Figure 1-D/Quantificated image/HFD/no.4/HFD BAT_no.4_RGB_PCPE-1_Cy5-1.tif]

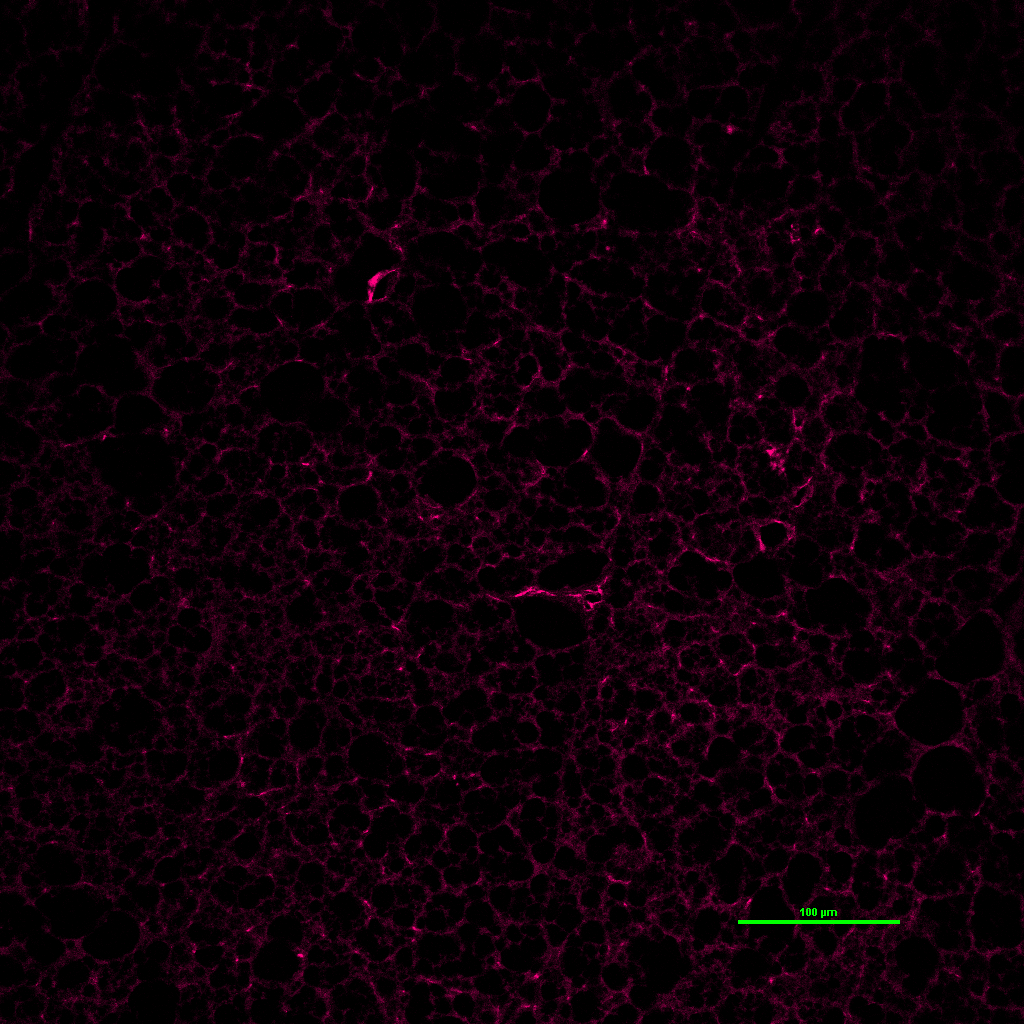

Supplement: Supplementary file 3 — Source data Fig. 1 [file 44318_2024_196_MOESM3_ESM.zip › Figure 1/Figure 1-D/Quantificated image/HFD/no.4/HFD BAT_no.4_RGB_PCPE-1_Cy5-4.tif]

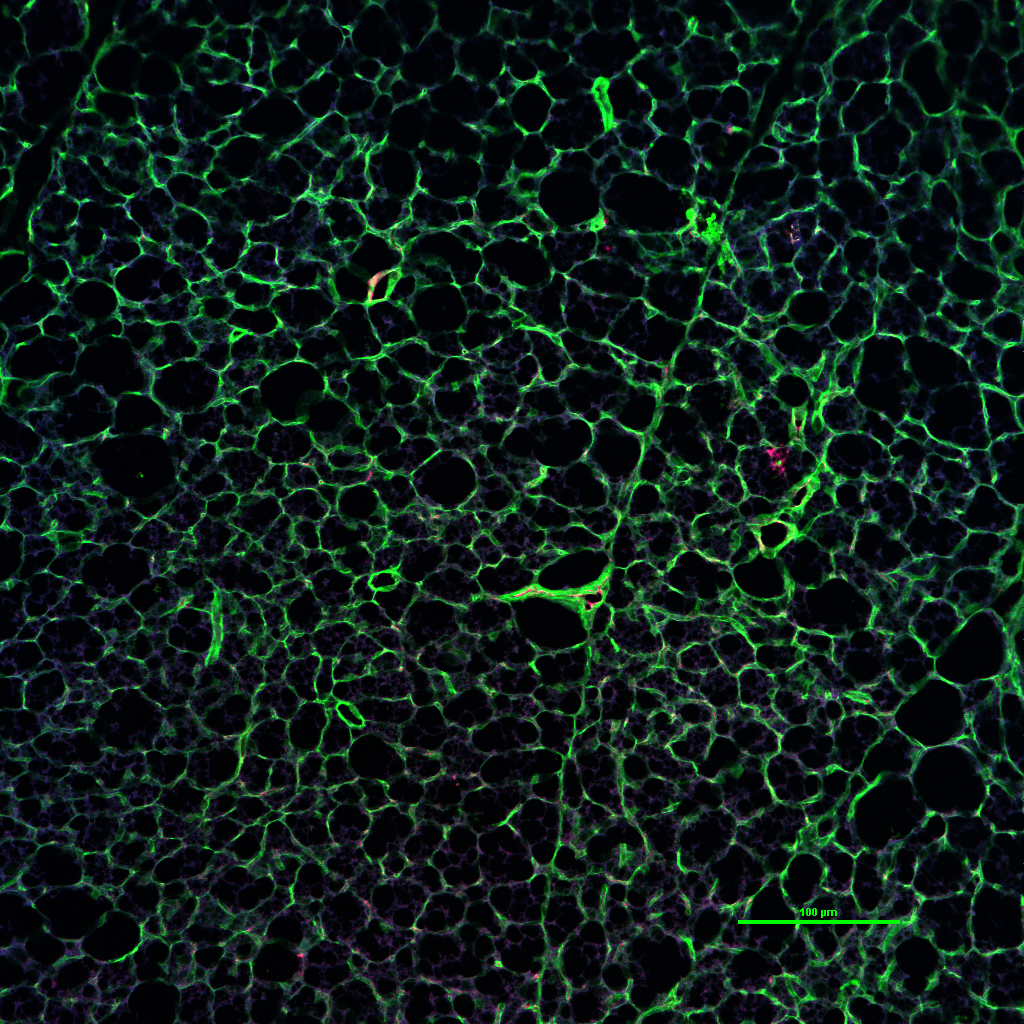

Supplement: Supplementary file 3 — Source data Fig. 1 [file 44318_2024_196_MOESM3_ESM.zip › Figure 1/Figure 1-D/Quantificated image/HFD/no.4/HFD BAT_no.4_RGB_Merge-4.tif]

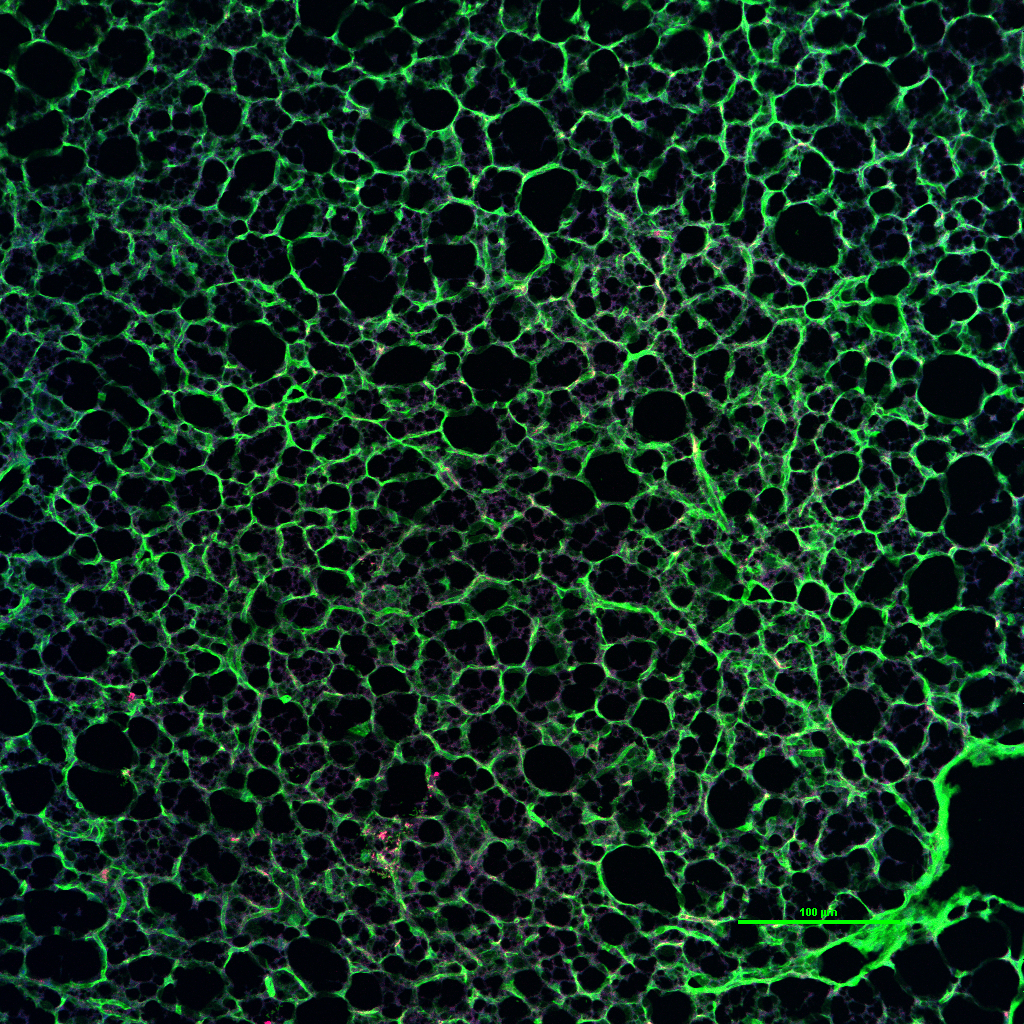

Supplement: Supplementary file 3 — Source data Fig. 1 [file 44318_2024_196_MOESM3_ESM.zip › Figure 1/Figure 1-D/Quantificated image/HFD/no.4/HFD BAT_no.4_RGB_Merge-2.tif]

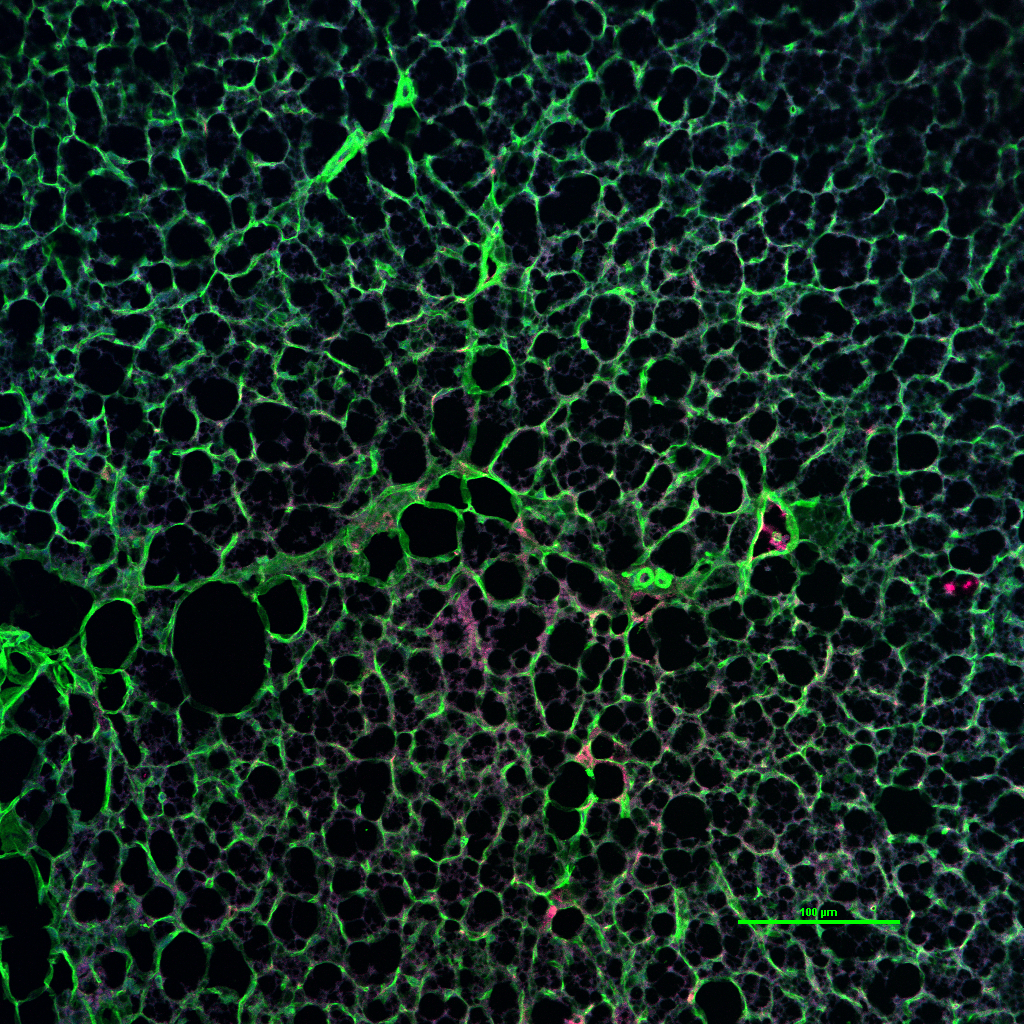

Supplement: Supplementary file 3 — Source data Fig. 1 [file 44318_2024_196_MOESM3_ESM.zip › Figure 1/Figure 1-D/Quantificated image/HFD/no.4/HFD BAT_no.4_RGB_Merge-3.tif]

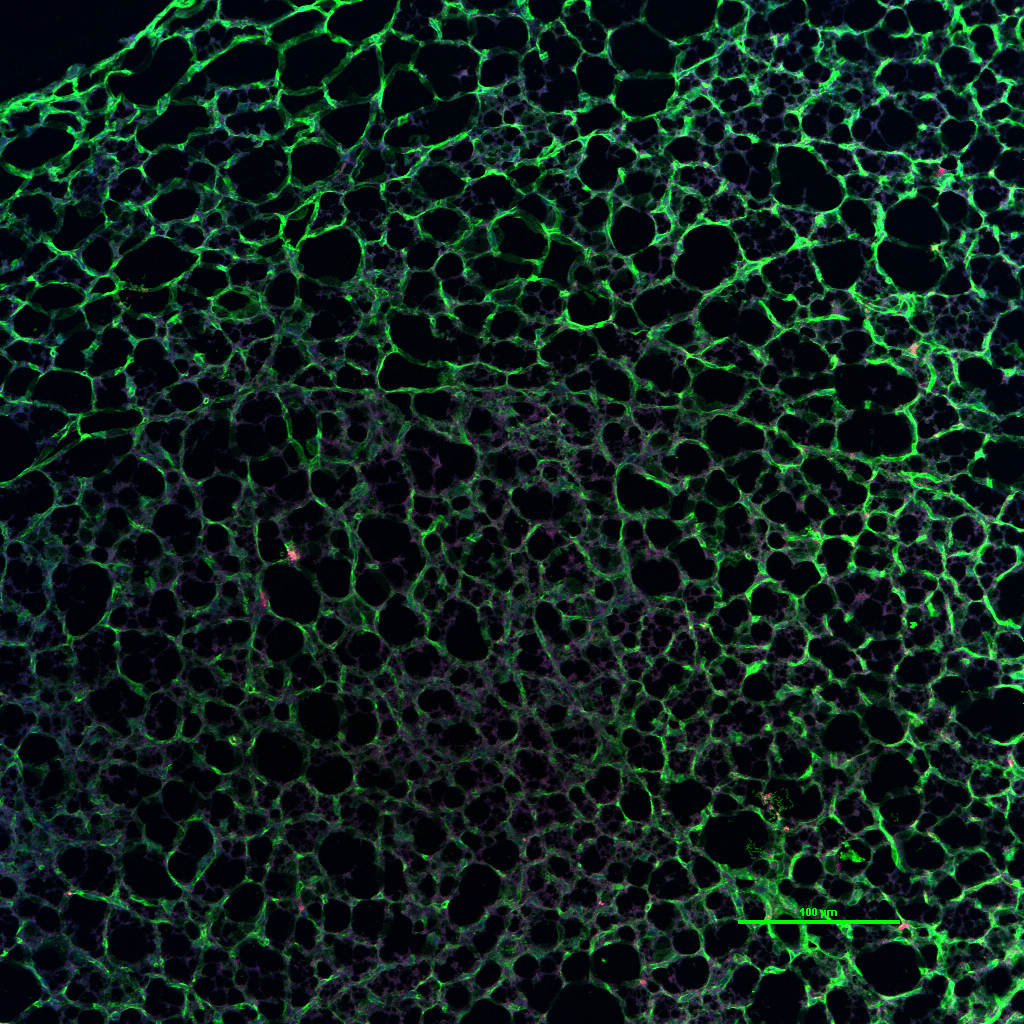

Supplement: Supplementary file 3 — Source data Fig. 1 [file 44318_2024_196_MOESM3_ESM.zip › Figure 1/Figure 1-D/Quantificated image/HFD/no.4/HFD BAT_no.4_RGB_Merge-1.tif]

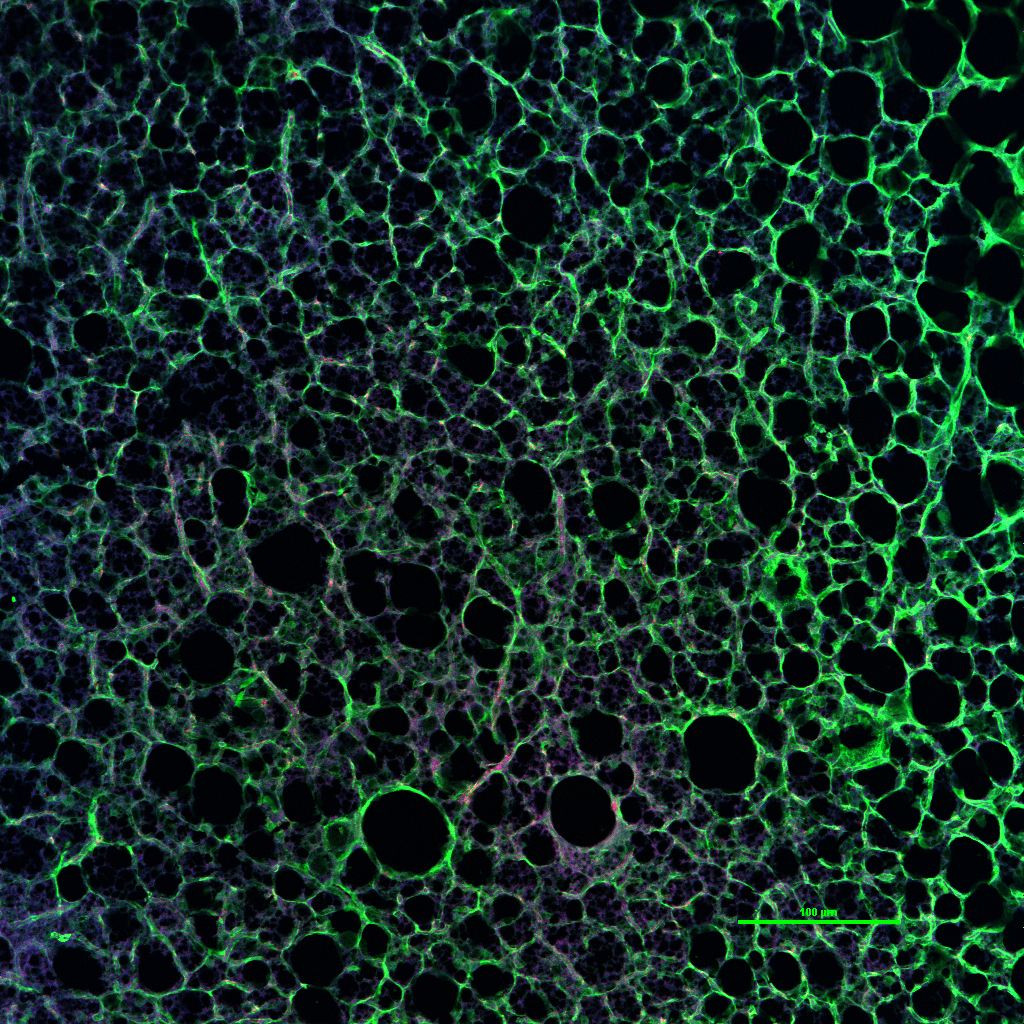

Supplement: Supplementary file 3 — Source data Fig. 1 [file 44318_2024_196_MOESM3_ESM.zip › Figure 1/Figure 1-D/Quantificated image/HFD/no.2/HFD BAT_no.2_RGB_Merge-4.tif]

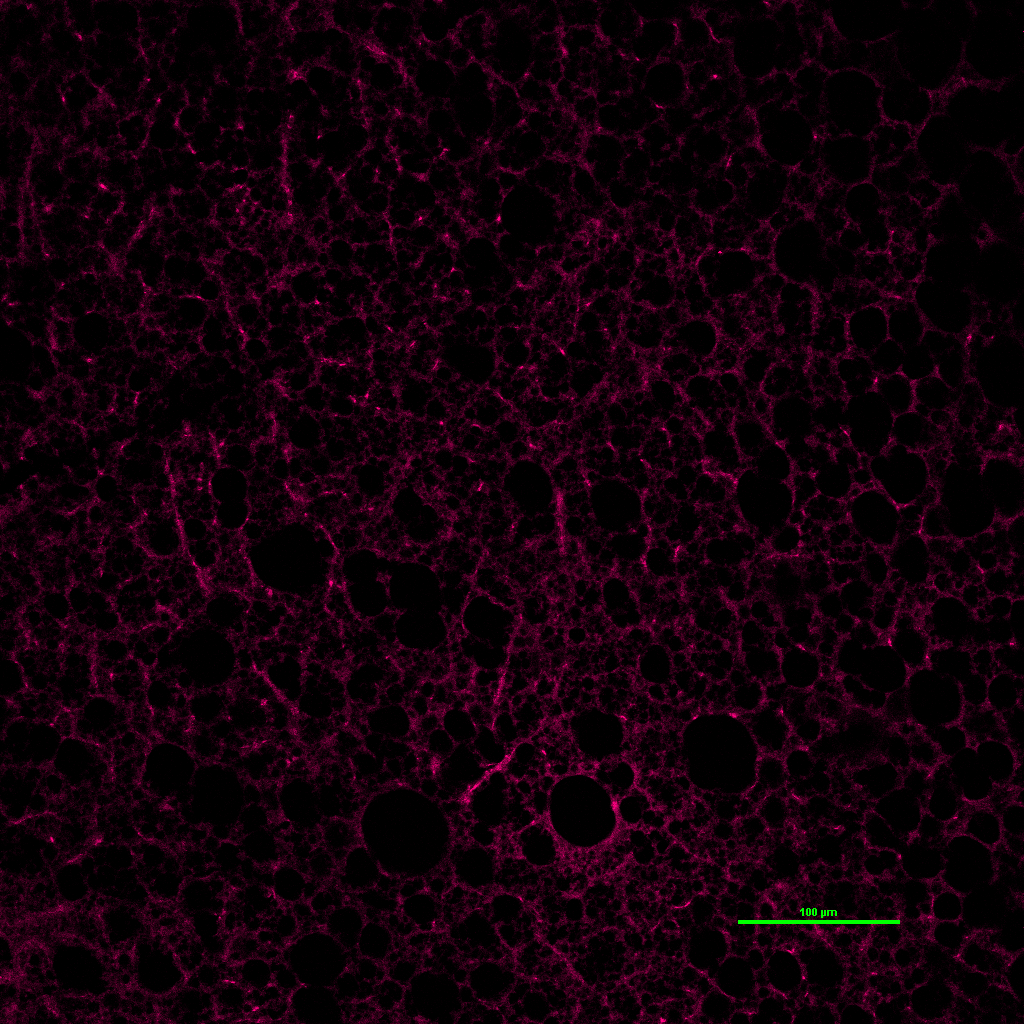

Supplement: Supplementary file 3 — Source data Fig. 1 [file 44318_2024_196_MOESM3_ESM.zip › Figure 1/Figure 1-D/Quantificated image/HFD/no.2/HFD BAT_no.2_RGB_PCPE-1_Cy5-4.tif]

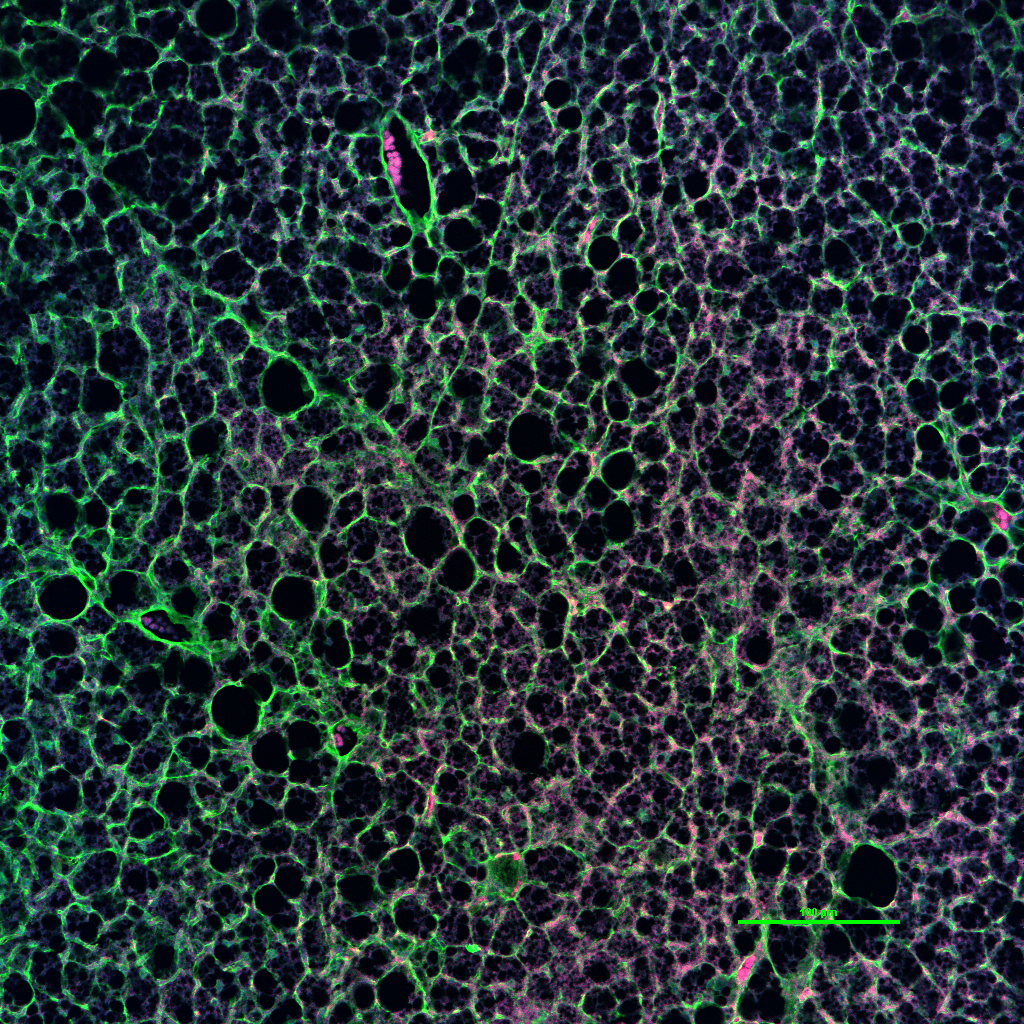

Supplement: Supplementary file 3 — Source data Fig. 1 [file 44318_2024_196_MOESM3_ESM.zip › Figure 1/Figure 1-D/Quantificated image/HFD/no.2/HFD BAT_no.2_RGB_Merge-1.tif]

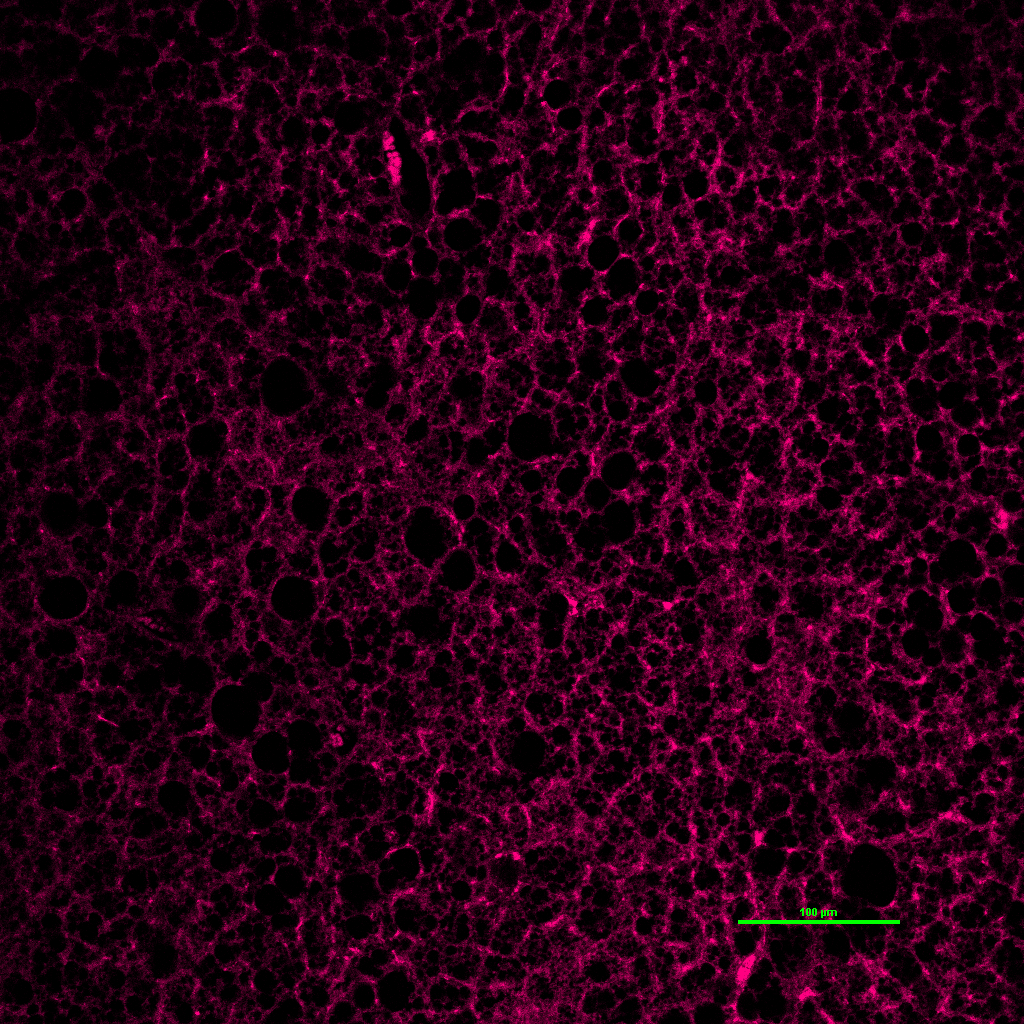

Supplement: Supplementary file 3 — Source data Fig. 1 [file 44318_2024_196_MOESM3_ESM.zip › Figure 1/Figure 1-D/Quantificated image/HFD/no.2/HFD BAT_no.2_RGB_PCPE-1_Cy5-1.tif]

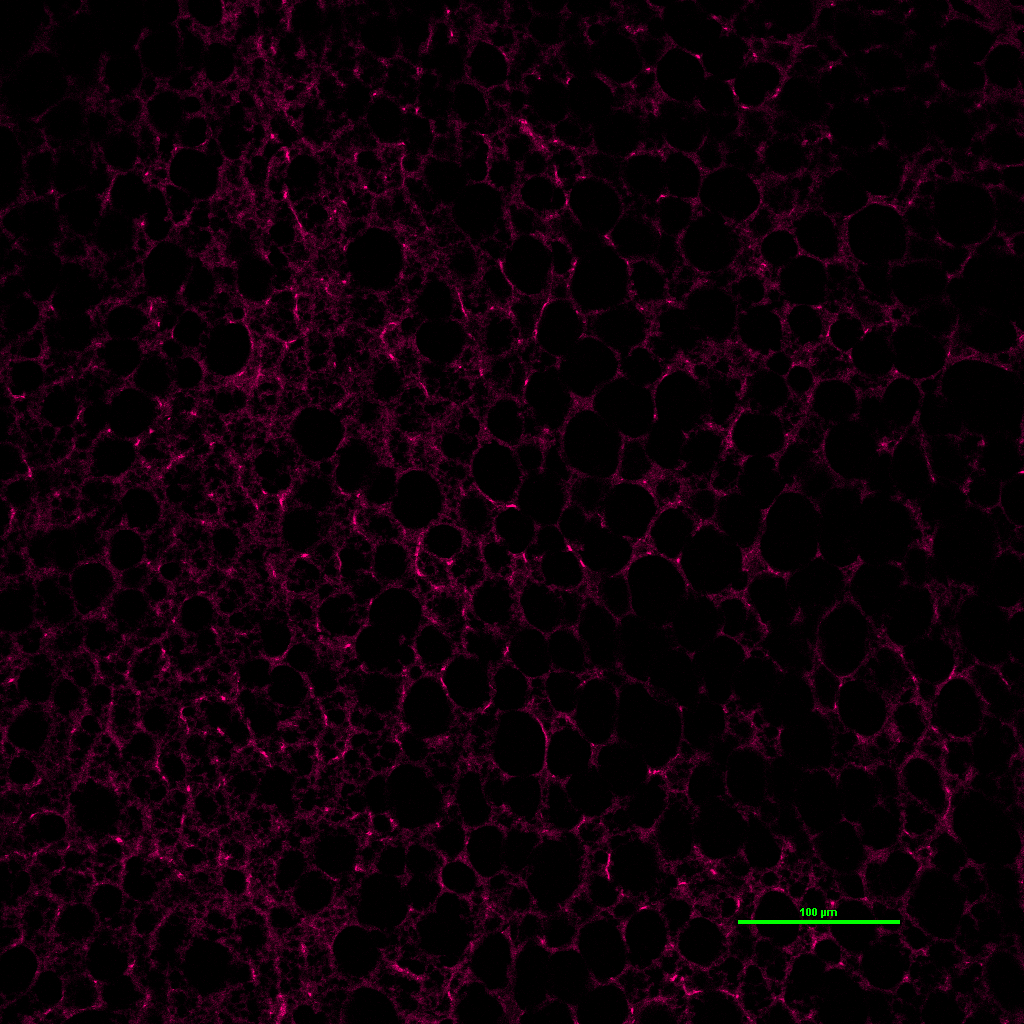

Supplement: Supplementary file 3 — Source data Fig. 1 [file 44318_2024_196_MOESM3_ESM.zip › Figure 1/Figure 1-D/Quantificated image/HFD/no.2/HFD BAT_no.2_RGB_PCPE-1_Cy5-3.tif]

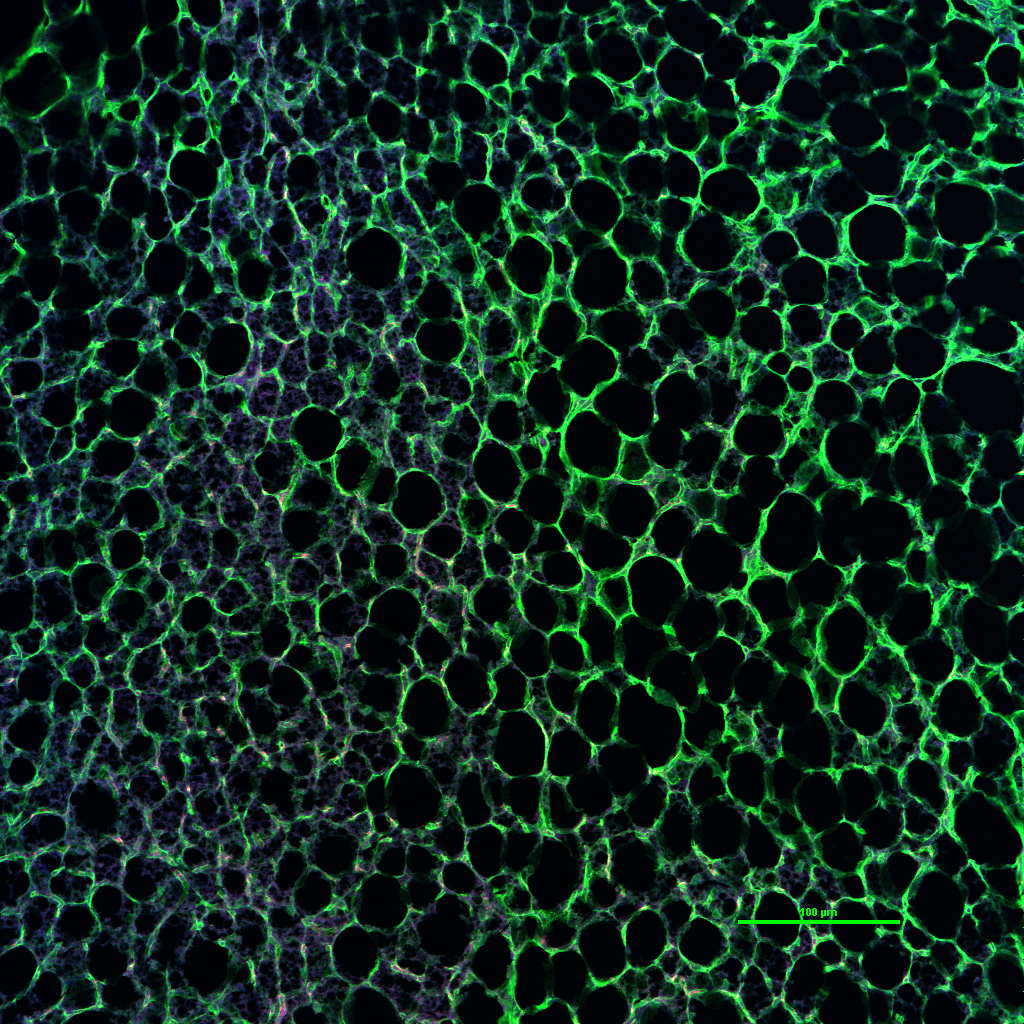

Supplement: Supplementary file 3 — Source data Fig. 1 [file 44318_2024_196_MOESM3_ESM.zip › Figure 1/Figure 1-D/Quantificated image/HFD/no.2/HFD BAT_no.2_RGB_Merge-3.tif]

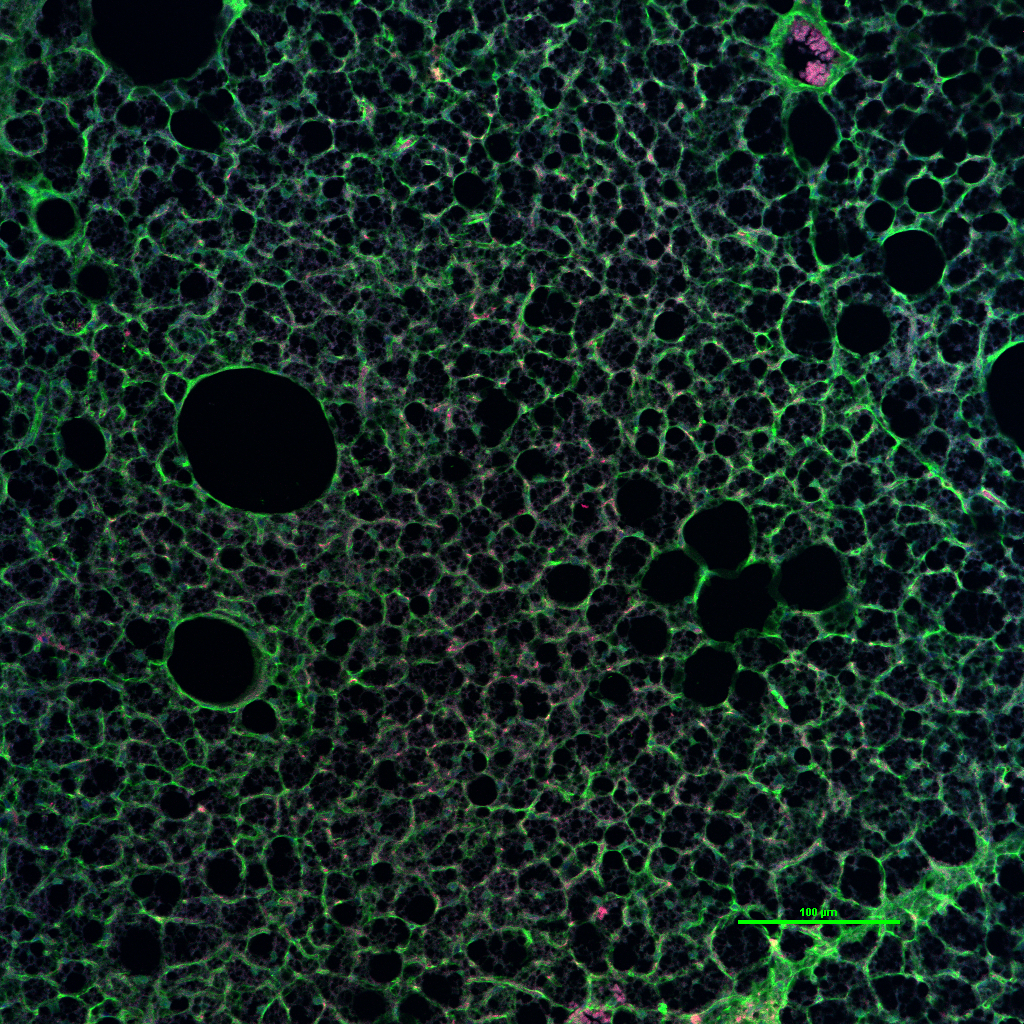

Supplement: Supplementary file 3 — Source data Fig. 1 [file 44318_2024_196_MOESM3_ESM.zip › Figure 1/Figure 1-D/Quantificated image/HFD/no.2/HFD BAT_no.2_RGB_Merge-2.tif]

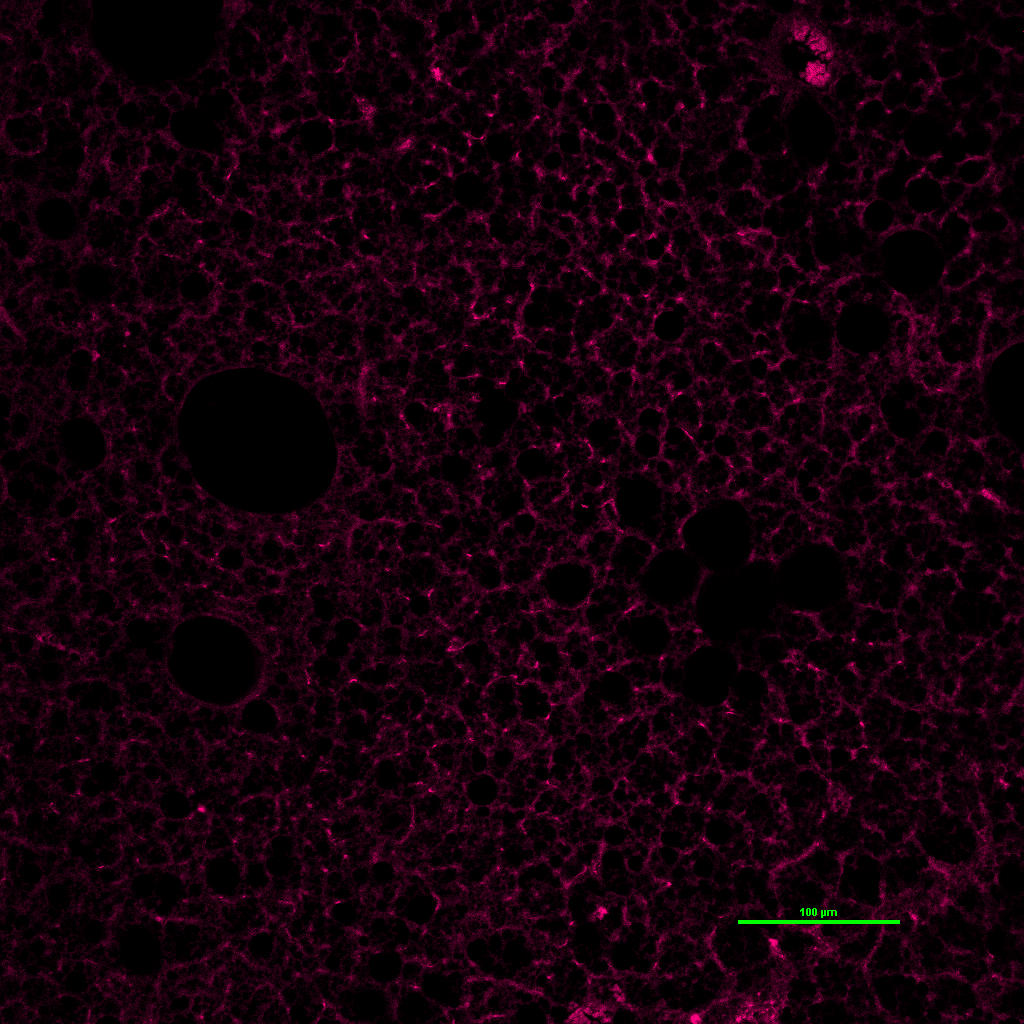

Supplement: Supplementary file 3 — Source data Fig. 1 [file 44318_2024_196_MOESM3_ESM.zip › Figure 1/Figure 1-D/Quantificated image/HFD/no.2/HFD BAT_no.2_RGB_PCPE-1_Cy5-2.tif]

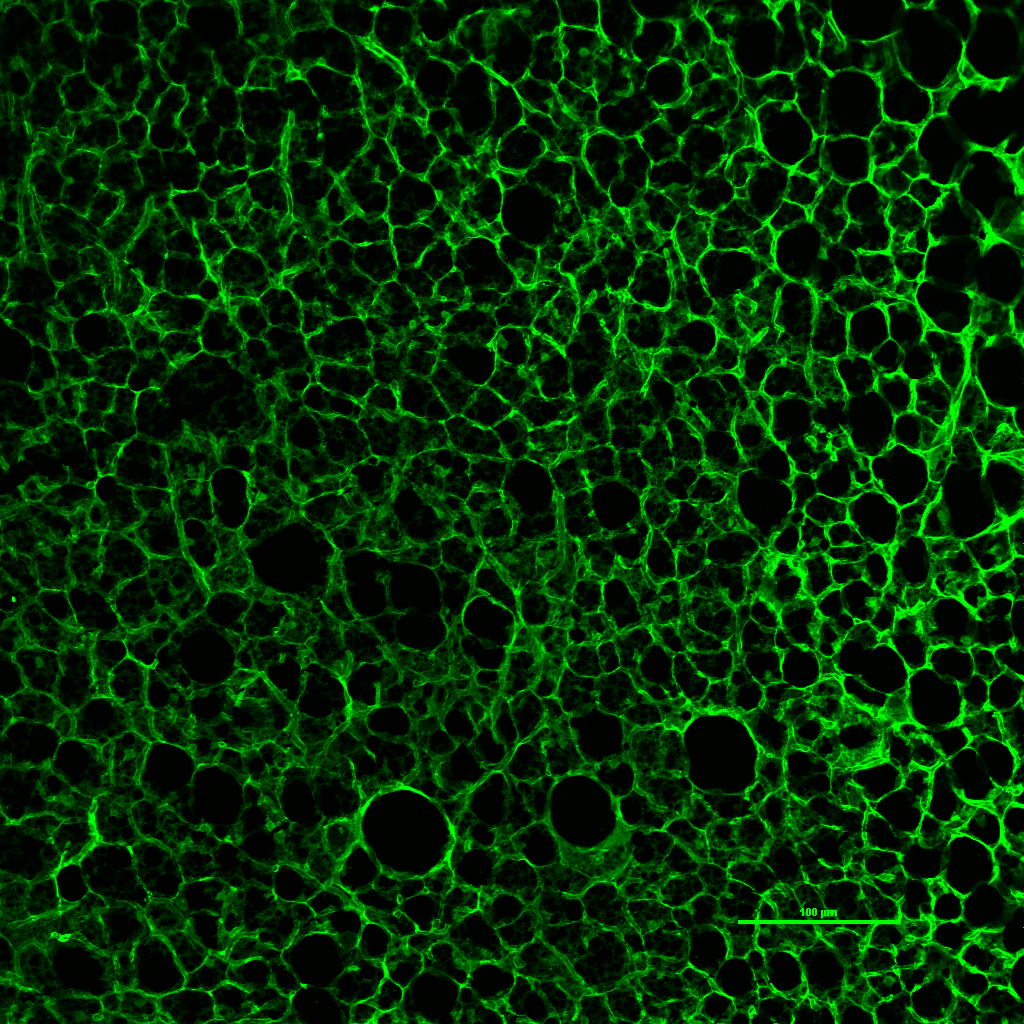

Supplement: Supplementary file 3 — Source data Fig. 1 [file 44318_2024_196_MOESM3_ESM.zip › Figure 1/Figure 1-D/Quantificated image/HFD/no.2/HFD BAT_no.2_RGB_WGA lectin_FITC-4.tif]

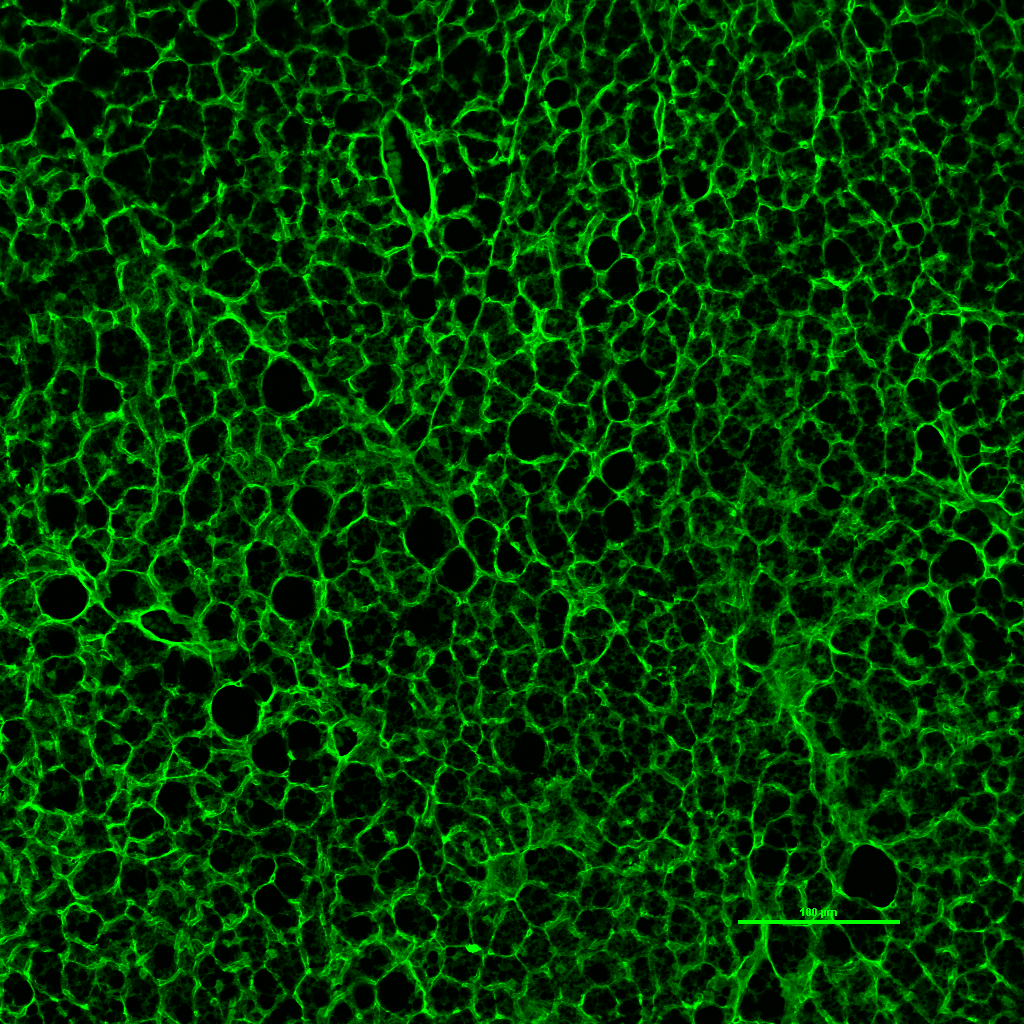

Supplement: Supplementary file 3 — Source data Fig. 1 [file 44318_2024_196_MOESM3_ESM.zip › Figure 1/Figure 1-D/Quantificated image/HFD/no.2/HFD BAT_no.2_RGB_WGA lectin_FITC-1.tif]

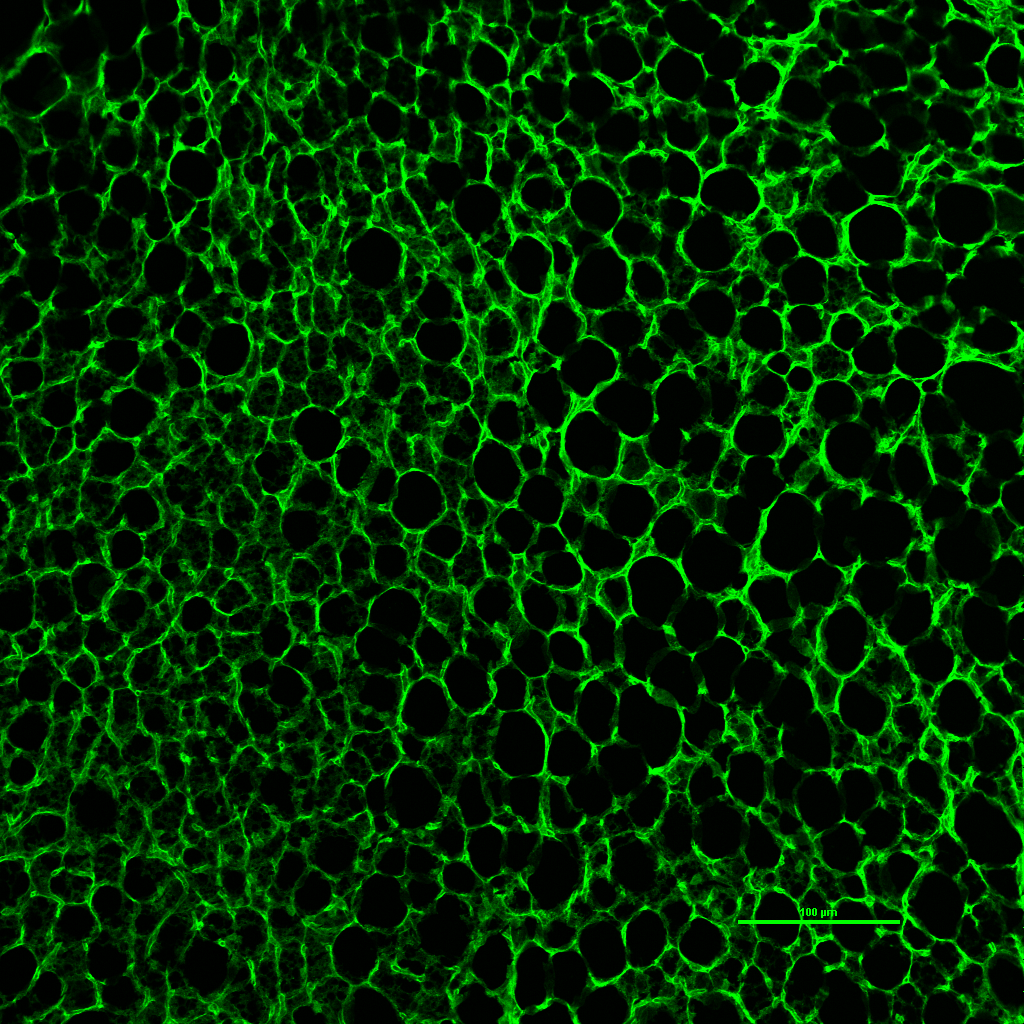

Supplement: Supplementary file 3 — Source data Fig. 1 [file 44318_2024_196_MOESM3_ESM.zip › Figure 1/Figure 1-D/Quantificated image/HFD/no.2/HFD BAT_no.2_RGB_WGA lectin_FITC-3.tif]

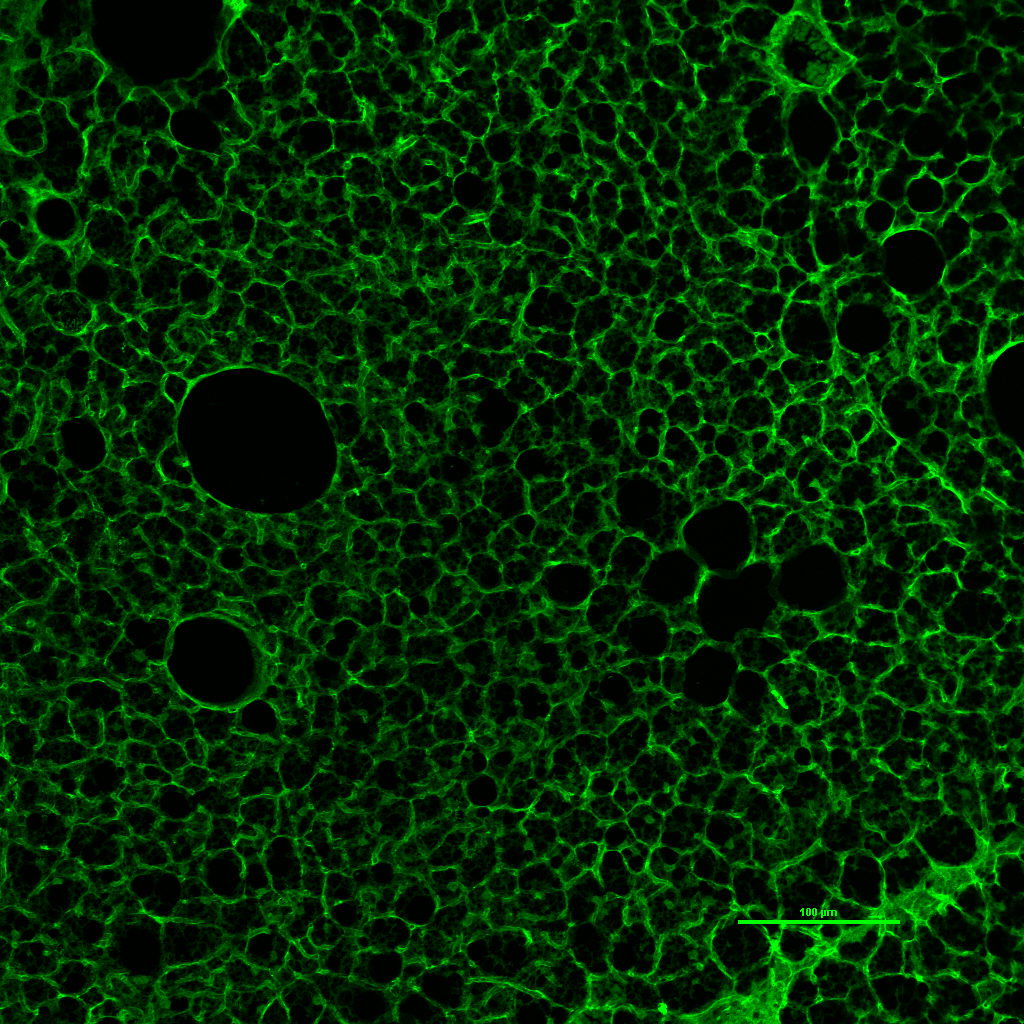

Supplement: Supplementary file 3 — Source data Fig. 1 [file 44318_2024_196_MOESM3_ESM.zip › Figure 1/Figure 1-D/Quantificated image/HFD/no.2/HFD BAT_no.2_RGB_WGA lectin_FITC-2.tif]

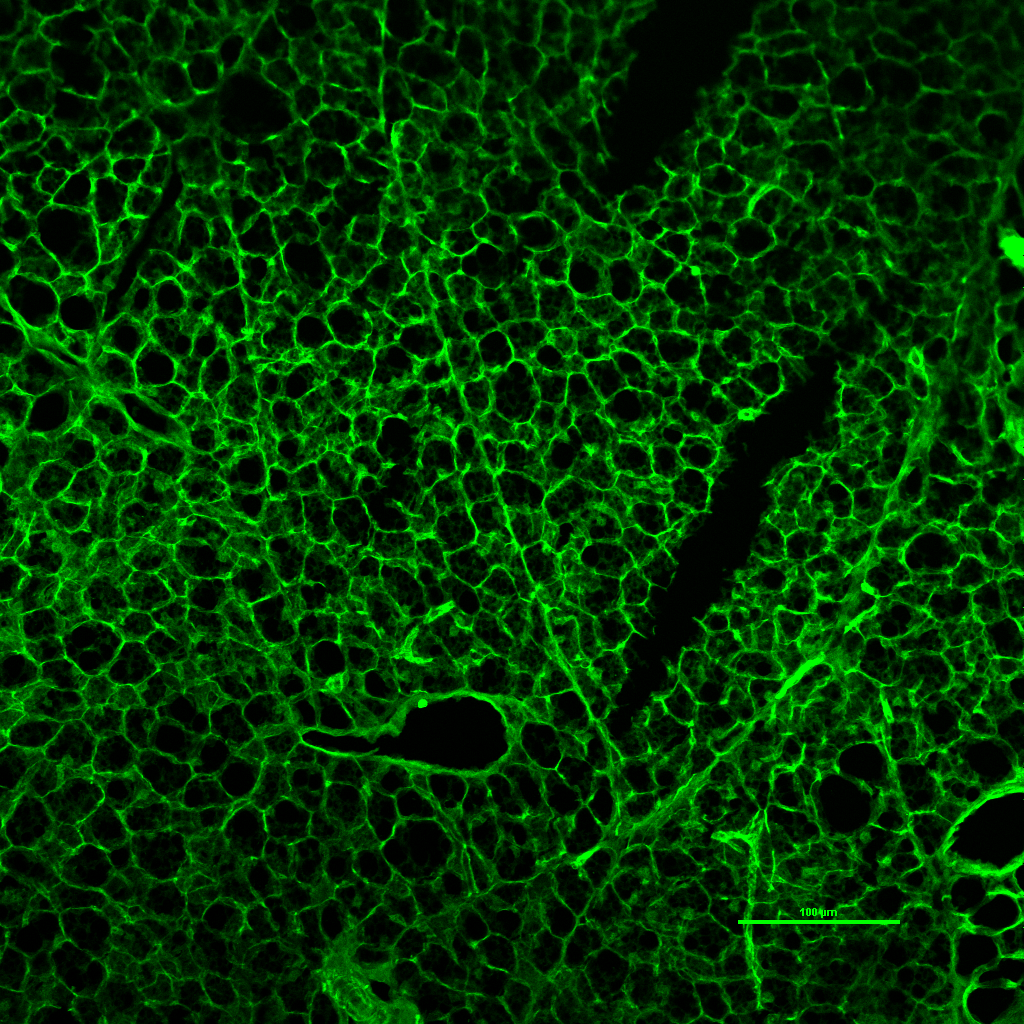

Supplement: Supplementary file 3 — Source data Fig. 1 [file 44318_2024_196_MOESM3_ESM.zip › Figure 1/Figure 1-D/Quantificated image/NC/no.1/NC BAT_no.1_RGB_WGA lectin_FITC-1.tif]

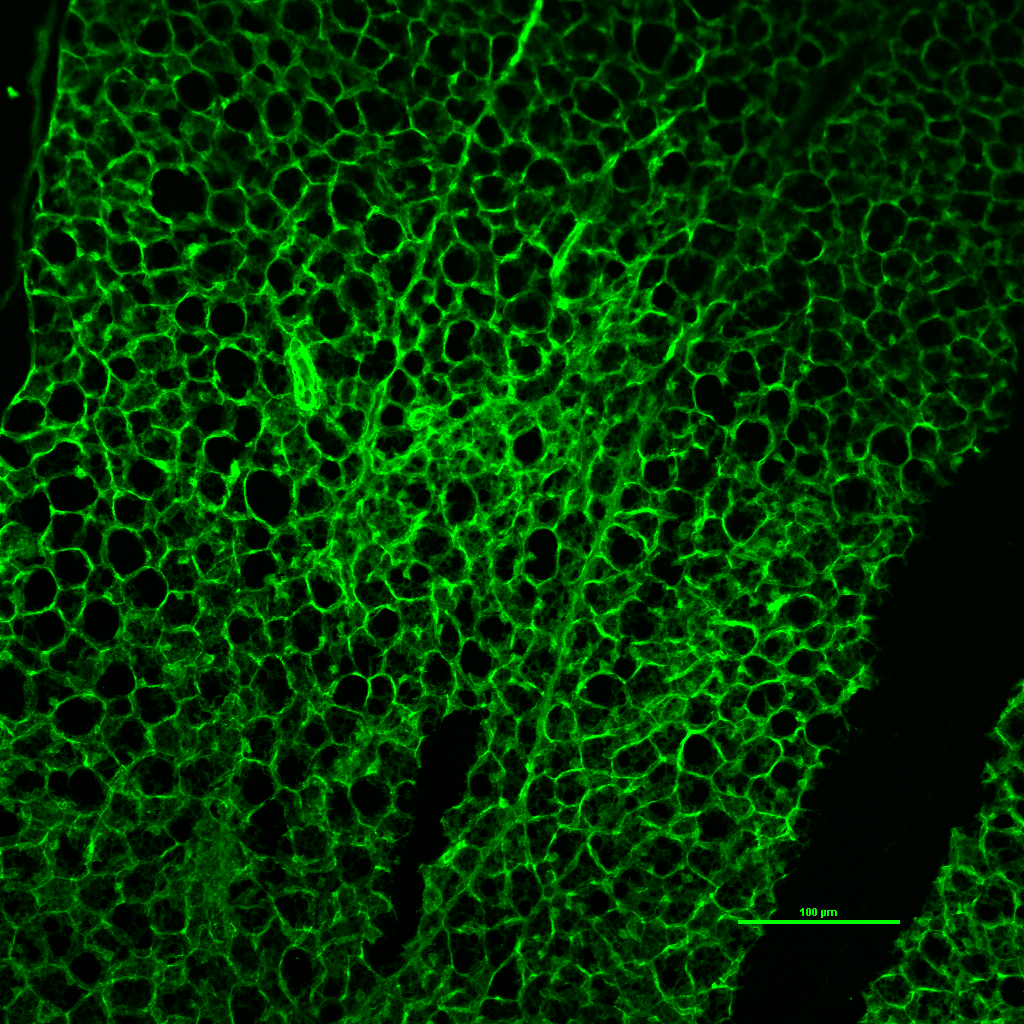

Supplement: Supplementary file 3 — Source data Fig. 1 [file 44318_2024_196_MOESM3_ESM.zip › Figure 1/Figure 1-D/Quantificated image/NC/no.1/NC BAT_no.1_RGB_WGA lectin_FITC-2.tif]

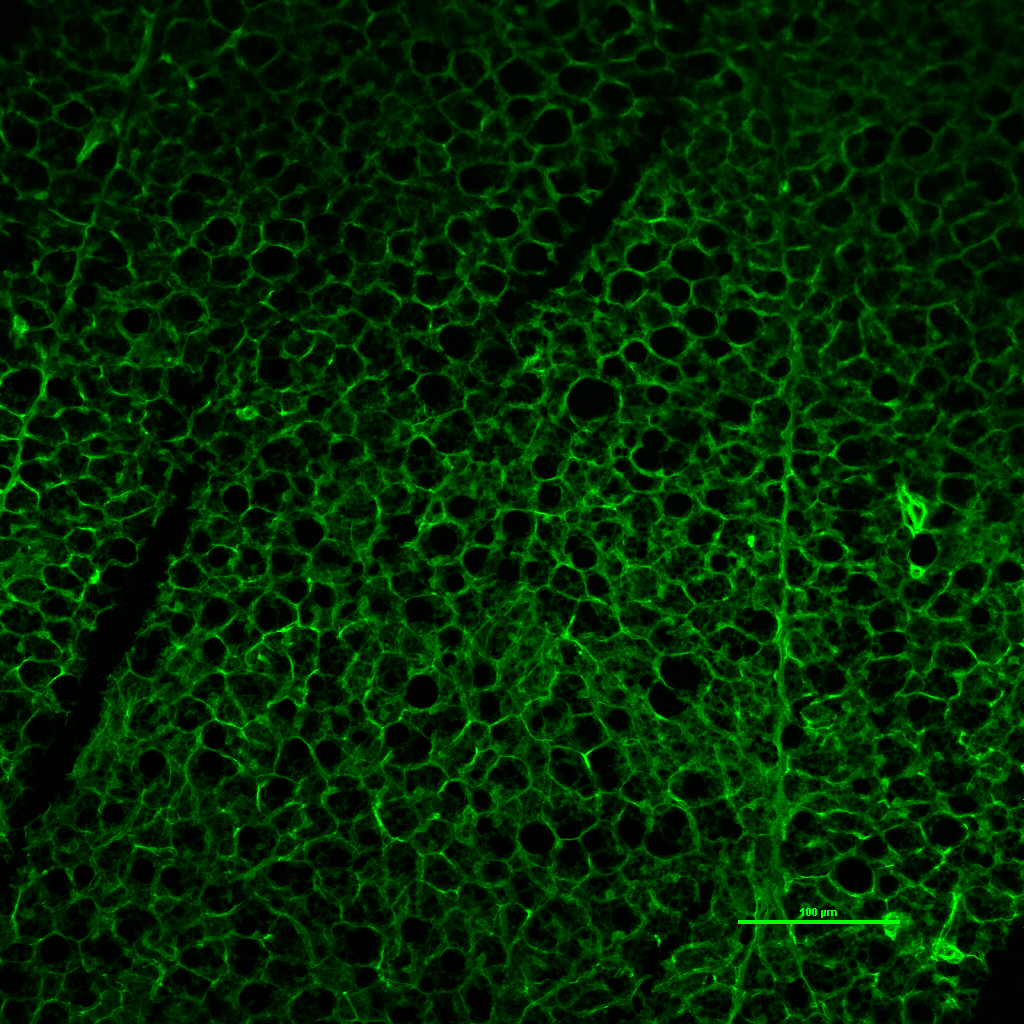

Supplement: Supplementary file 3 — Source data Fig. 1 [file 44318_2024_196_MOESM3_ESM.zip › Figure 1/Figure 1-D/Quantificated image/NC/no.1/NC BAT_no.1_RGB_WGA lectin_FITC-3.tif]

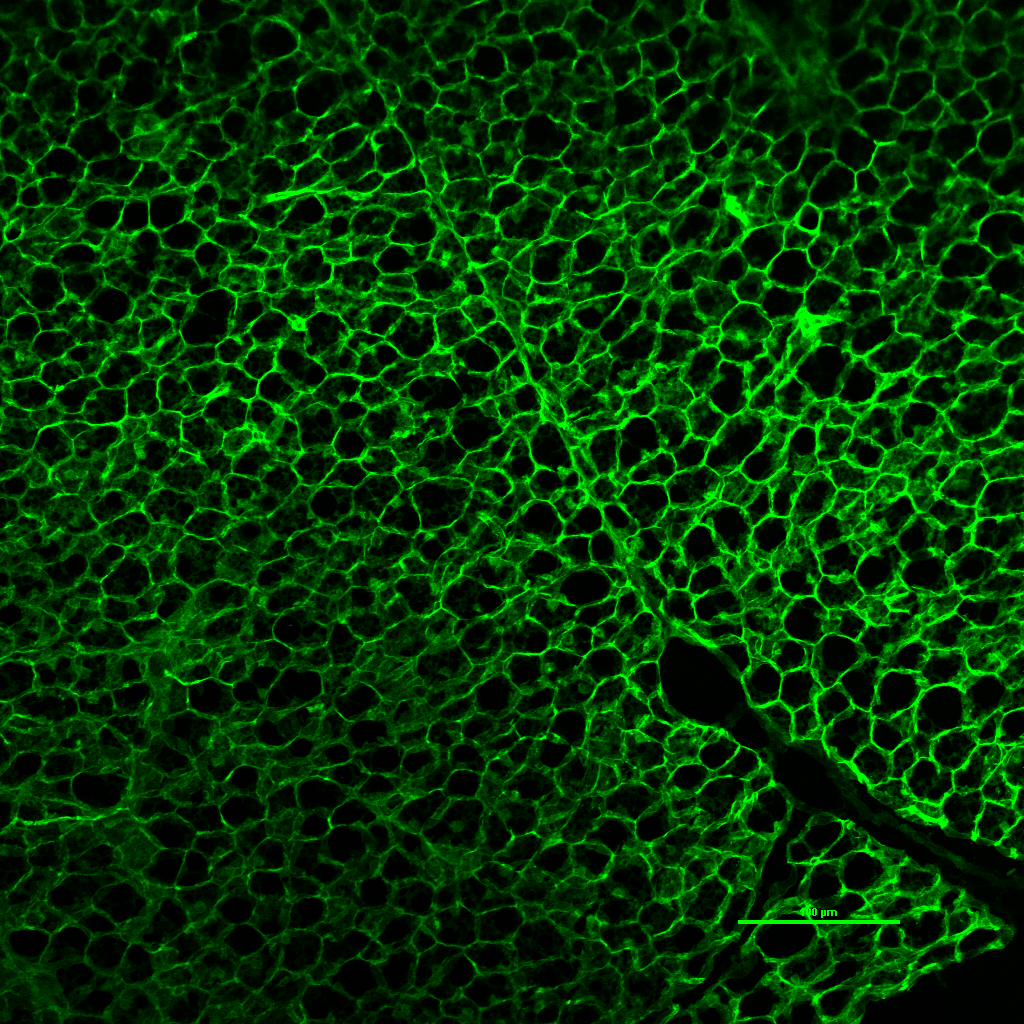

Supplement: Supplementary file 3 — Source data Fig. 1 [file 44318_2024_196_MOESM3_ESM.zip › Figure 1/Figure 1-D/Quantificated image/NC/no.1/NC BAT_no.1_RGB_WGA lectin_FITC-4.tif]

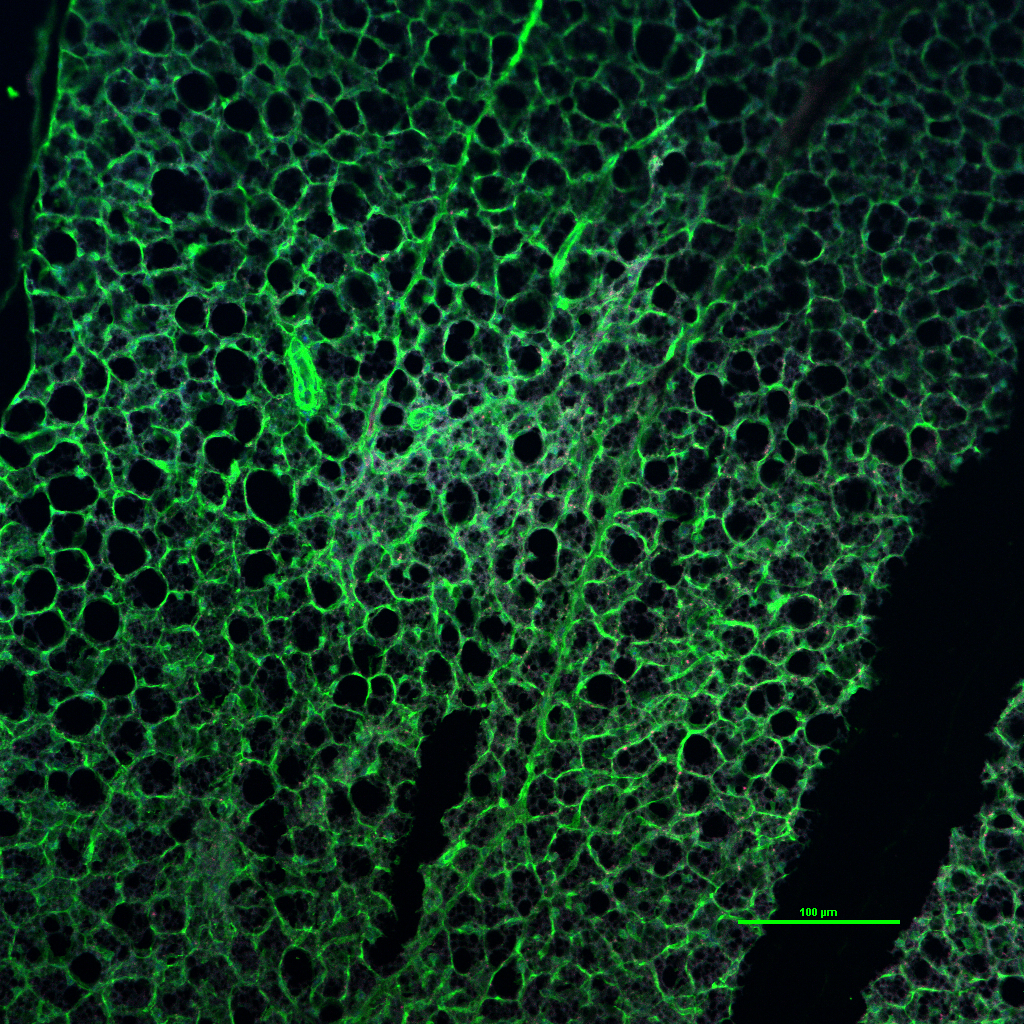

Supplement: Supplementary file 3 — Source data Fig. 1 [file 44318_2024_196_MOESM3_ESM.zip › Figure 1/Figure 1-D/Quantificated image/NC/no.1/NC BAT_no.1_RGB_Merge-2.tif]

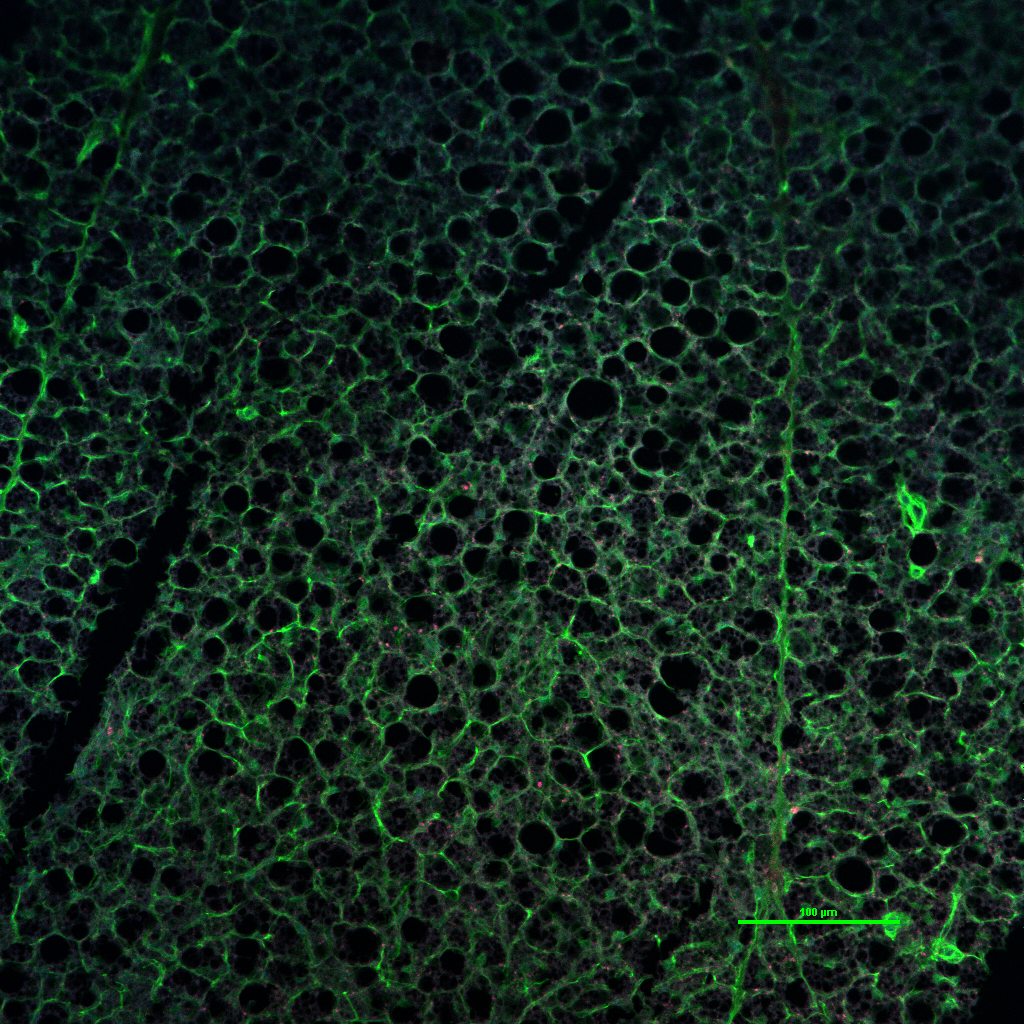

Supplement: Supplementary file 3 — Source data Fig. 1 [file 44318_2024_196_MOESM3_ESM.zip › Figure 1/Figure 1-D/Quantificated image/NC/no.1/NC BAT_no.1_RGB_Merge-3.tif]

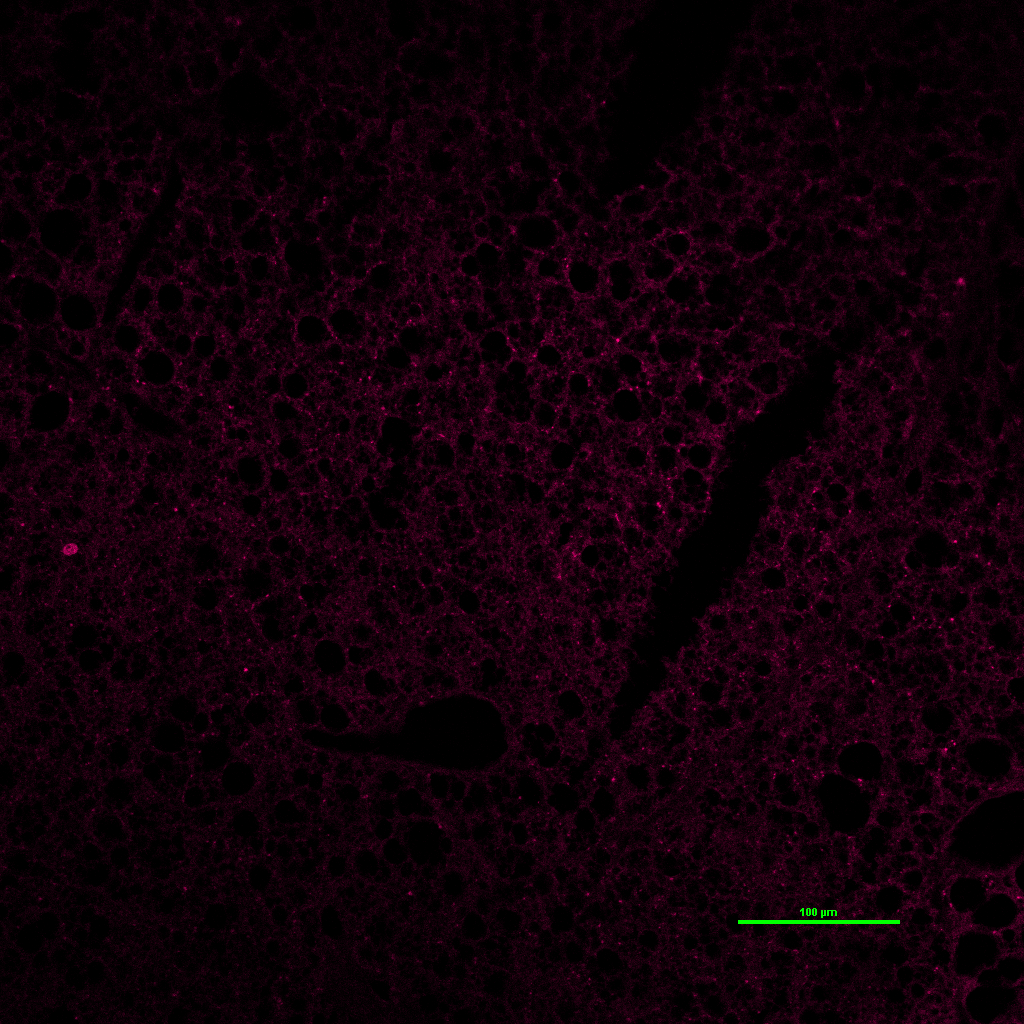

Supplement: Supplementary file 3 — Source data Fig. 1 [file 44318_2024_196_MOESM3_ESM.zip › Figure 1/Figure 1-D/Quantificated image/NC/no.1/NC BAT_no.1_RGB_PCPE-1_Cy5-1.tif]

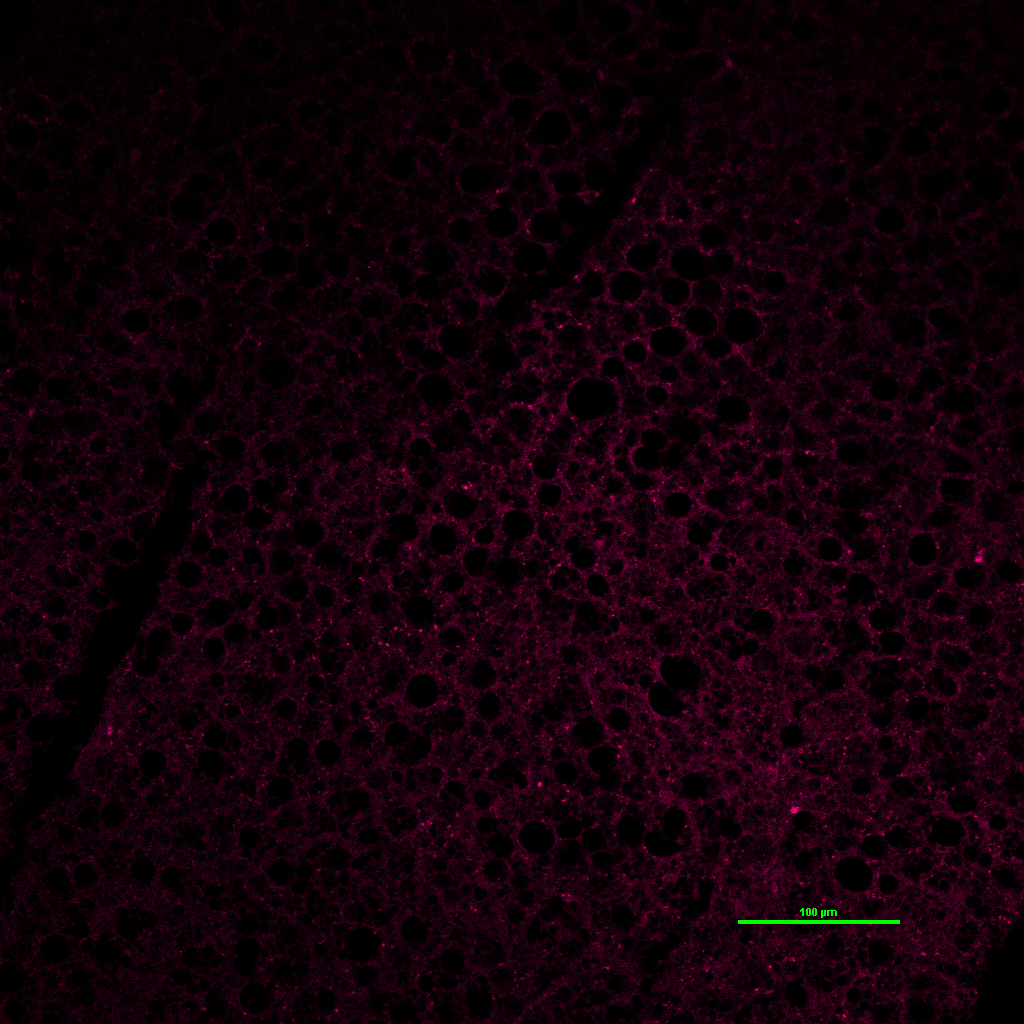

Supplement: Supplementary file 3 — Source data Fig. 1 [file 44318_2024_196_MOESM3_ESM.zip › Figure 1/Figure 1-D/Quantificated image/NC/no.1/NC BAT_no.1_RGB_PCPE-1_Cy5-3.tif]

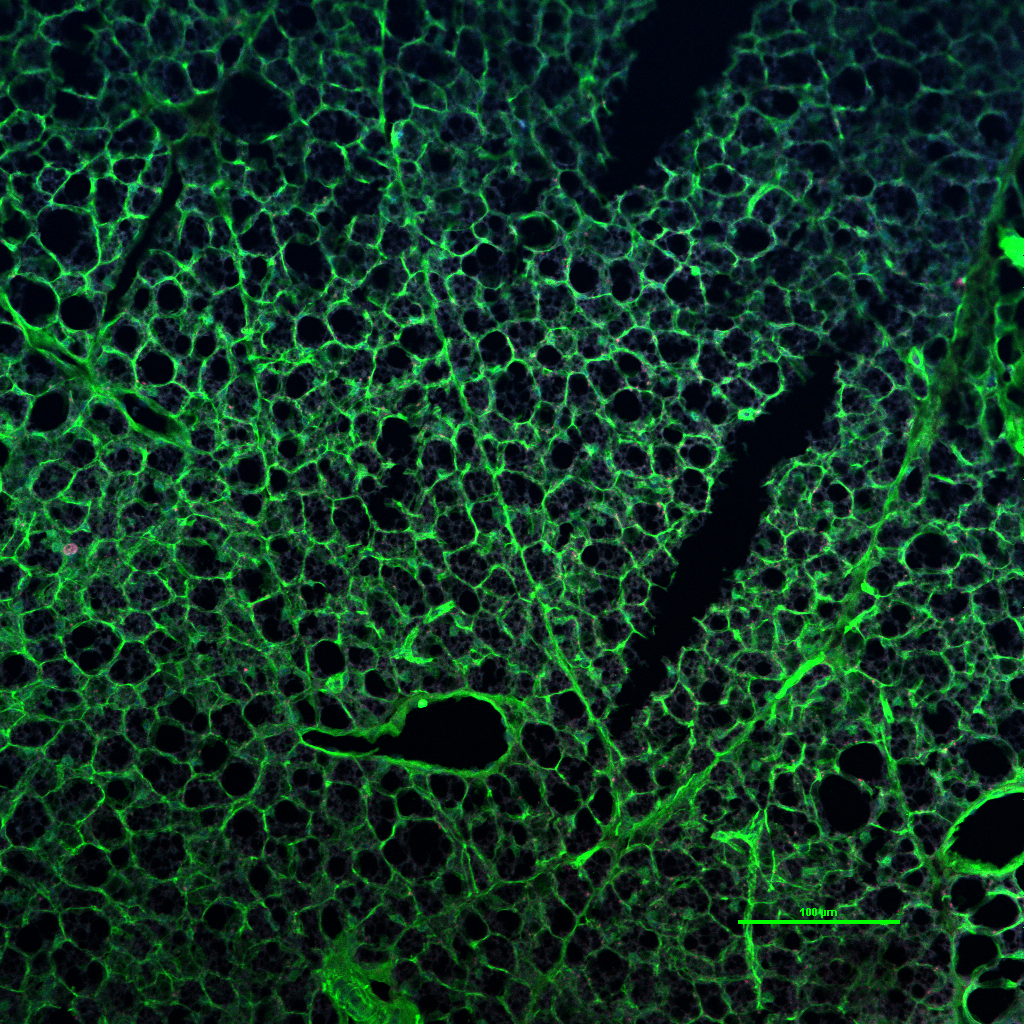

Supplement: Supplementary file 3 — Source data Fig. 1 [file 44318_2024_196_MOESM3_ESM.zip › Figure 1/Figure 1-D/Quantificated image/NC/no.1/NC BAT_no.1_RGB_Merge-1.tif]

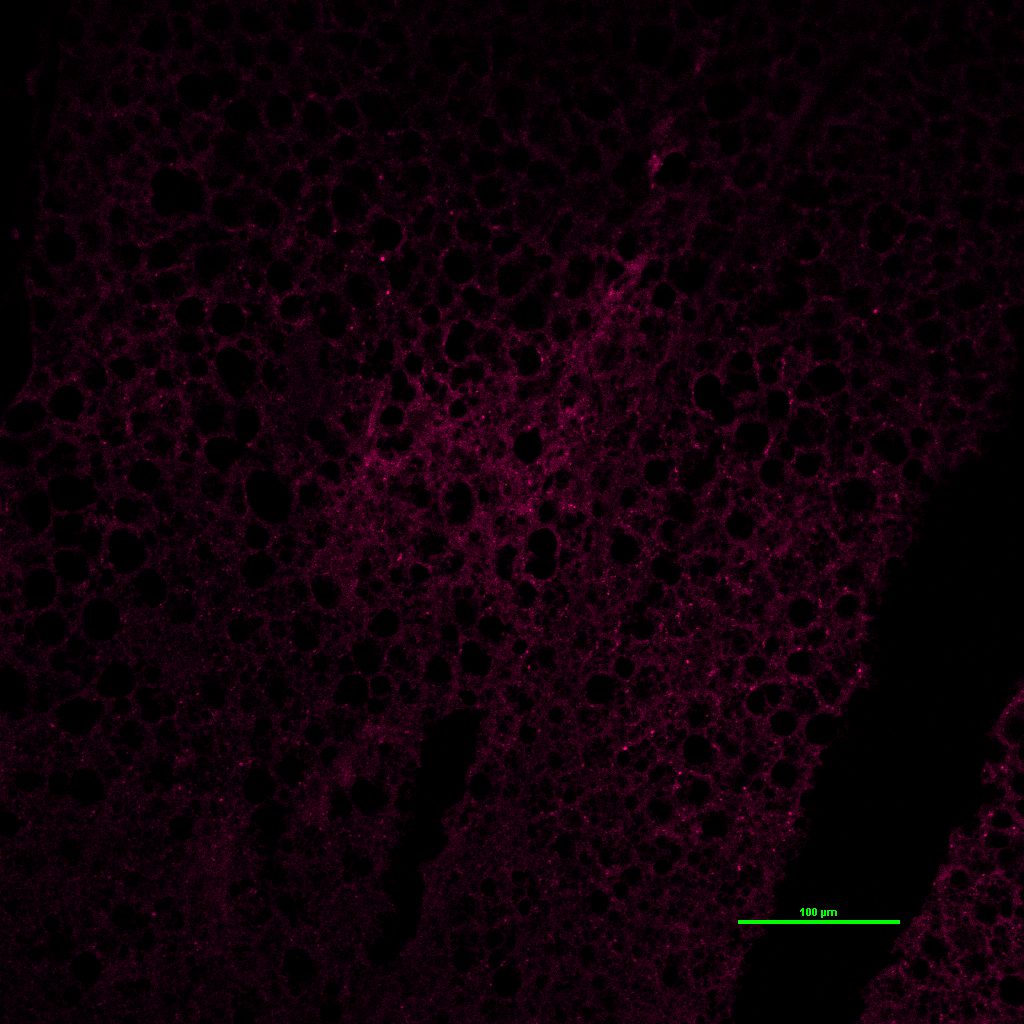

Supplement: Supplementary file 3 — Source data Fig. 1 [file 44318_2024_196_MOESM3_ESM.zip › Figure 1/Figure 1-D/Quantificated image/NC/no.1/NC BAT_no.1_RGB_PCPE-1_Cy5-2.tif]

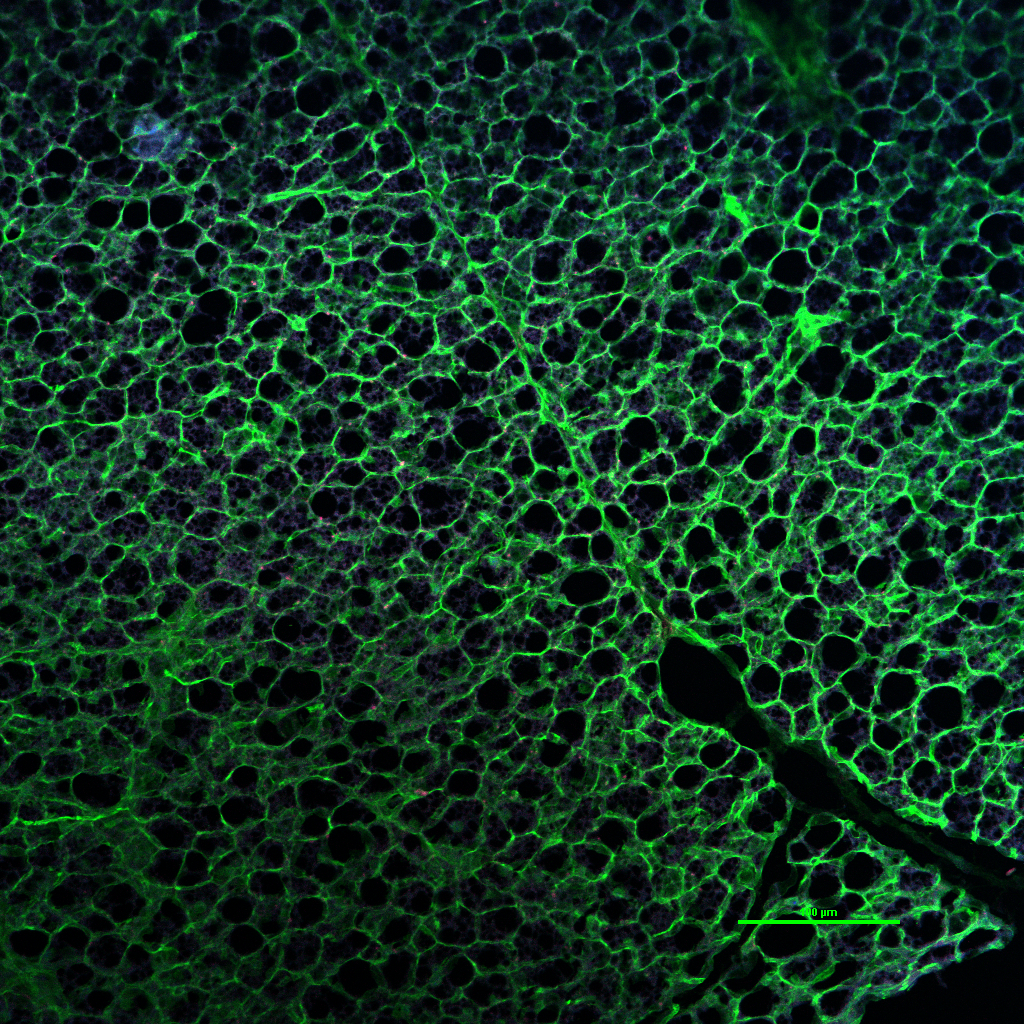

Supplement: Supplementary file 3 — Source data Fig. 1 [file 44318_2024_196_MOESM3_ESM.zip › Figure 1/Figure 1-D/Quantificated image/NC/no.1/NC BAT_no.1_RGB_Merge-4.tif]

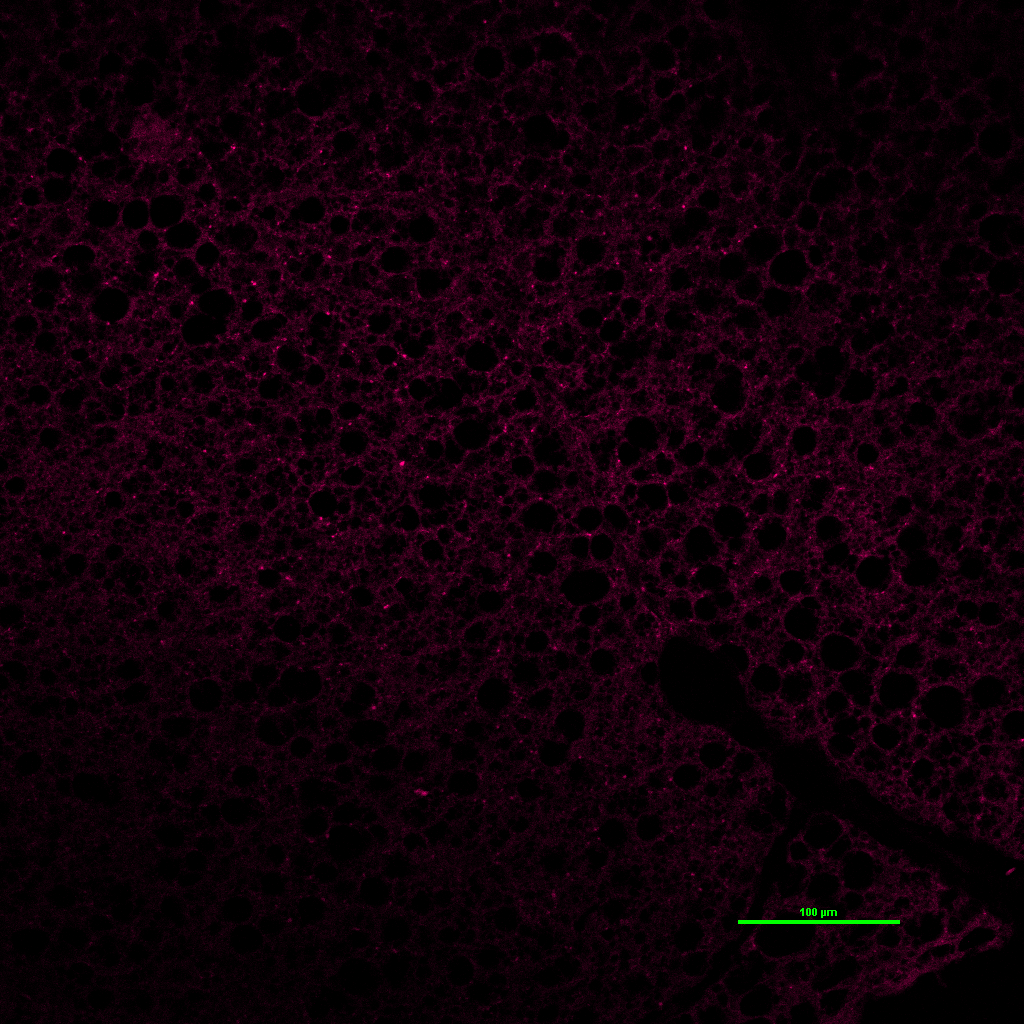

Supplement: Supplementary file 3 — Source data Fig. 1 [file 44318_2024_196_MOESM3_ESM.zip › Figure 1/Figure 1-D/Quantificated image/NC/no.1/NC BAT_no.1_RGB_PCPE-1_Cy5-4.tif]

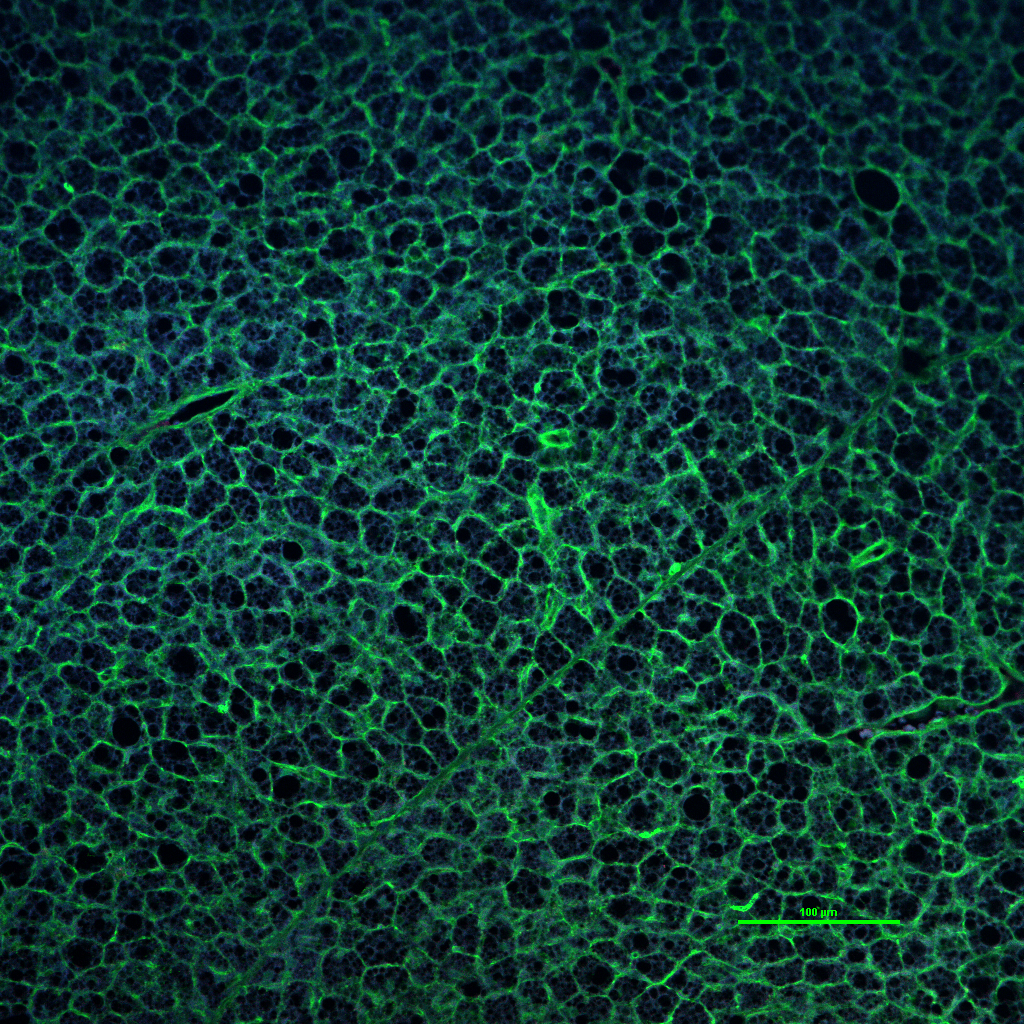

Supplement: Supplementary file 3 — Source data Fig. 1 [file 44318_2024_196_MOESM3_ESM.zip › Figure 1/Figure 1-D/Quantificated image/NC/no.3/NC BAT_no.3_RGB_Merge-3.tif]

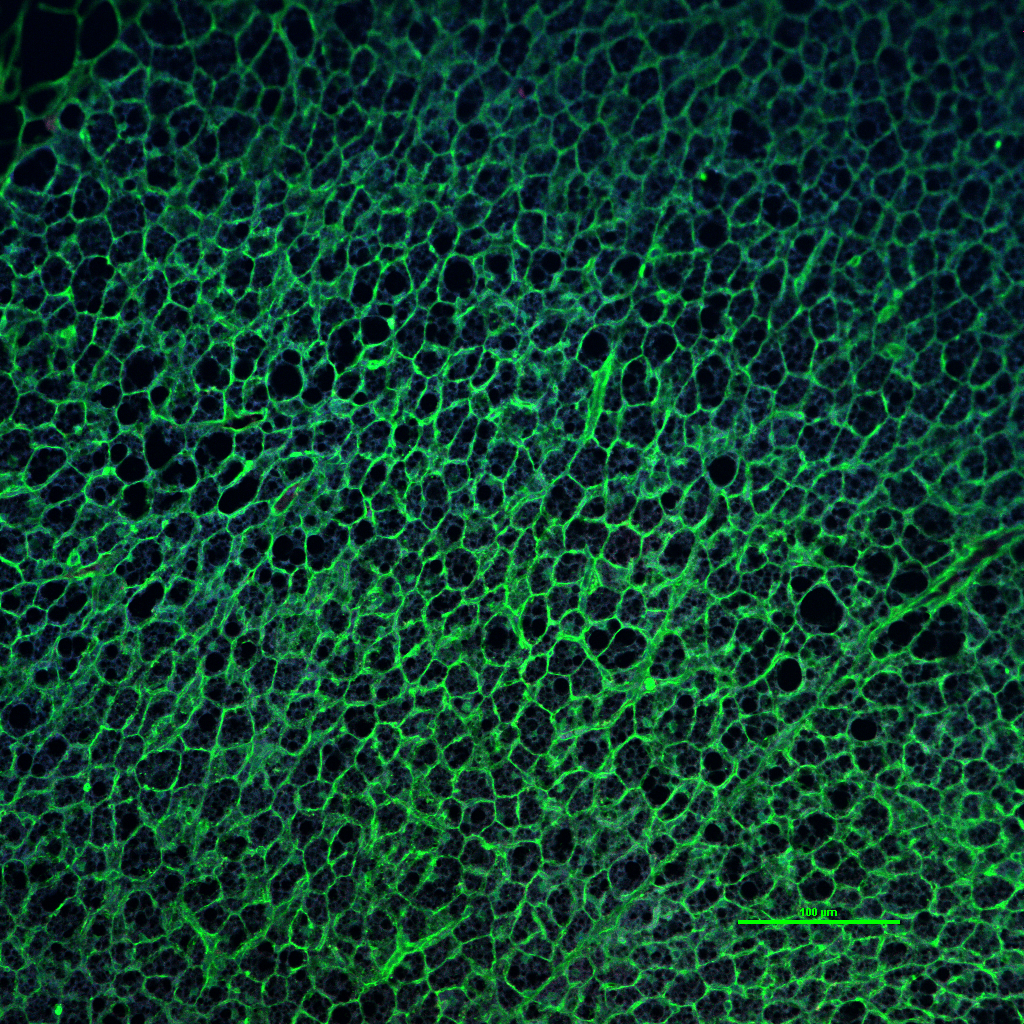

Supplement: Supplementary file 3 — Source data Fig. 1 [file 44318_2024_196_MOESM3_ESM.zip › Figure 1/Figure 1-D/Quantificated image/NC/no.3/NC BAT_no.3_RGB_Merge-2.tif]

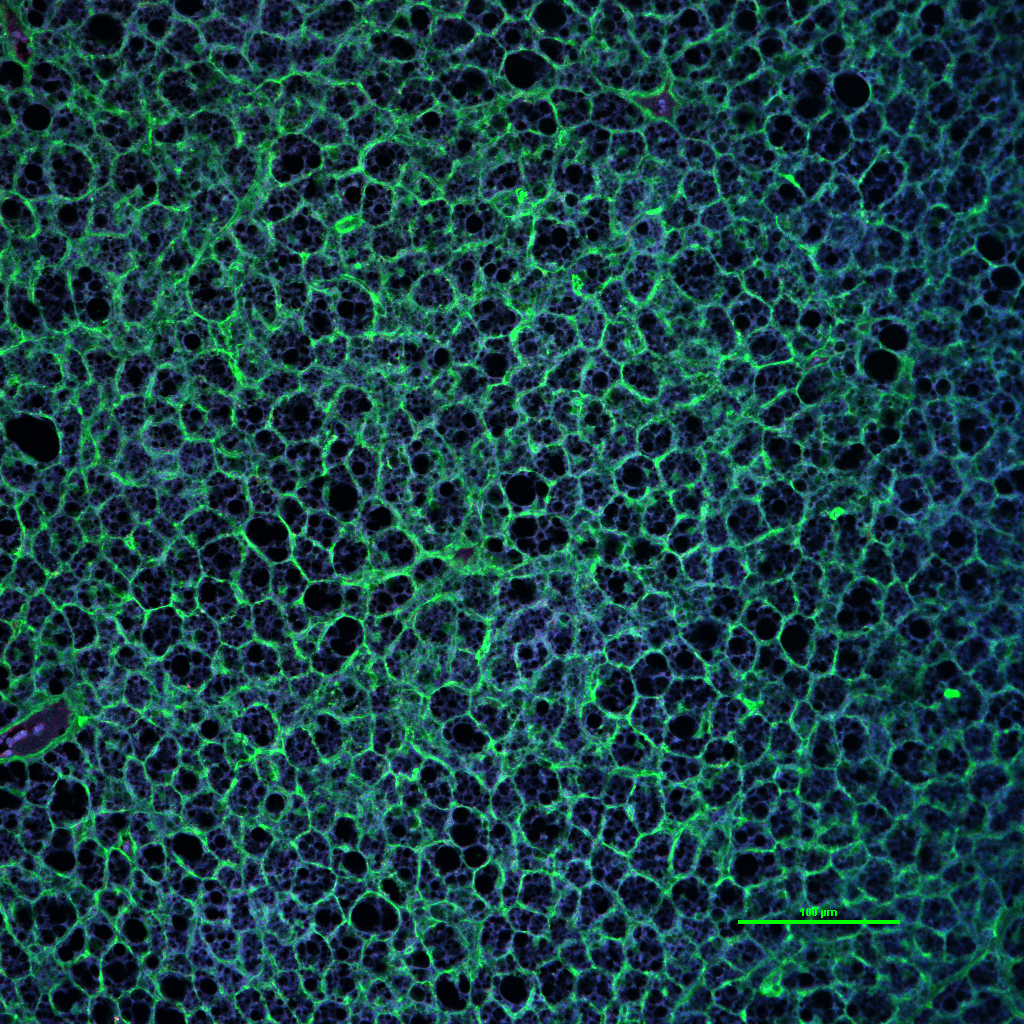

Supplement: Supplementary file 3 — Source data Fig. 1 [file 44318_2024_196_MOESM3_ESM.zip › Figure 1/Figure 1-D/Quantificated image/NC/no.3/NC BAT_no.3_RGB_Merge-1.tif]

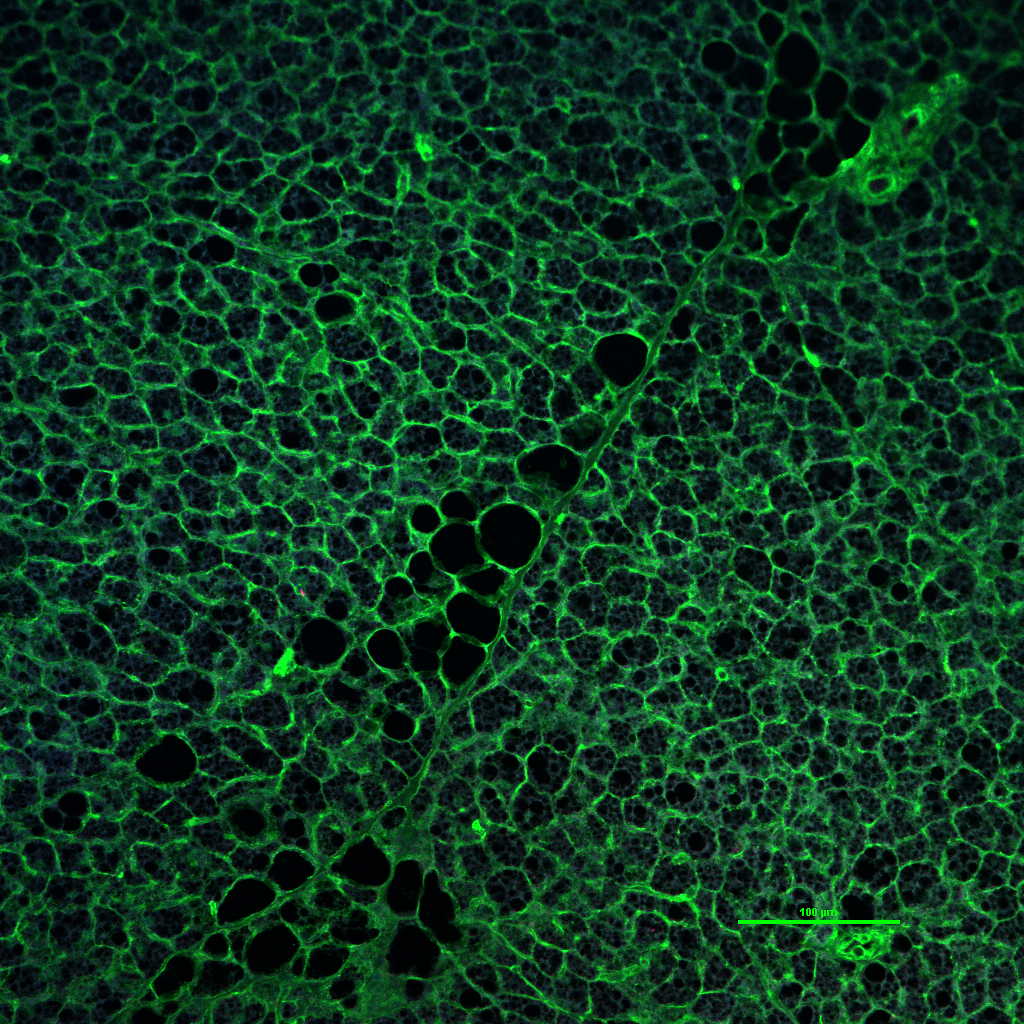

Supplement: Supplementary file 3 — Source data Fig. 1 [file 44318_2024_196_MOESM3_ESM.zip › Figure 1/Figure 1-D/Quantificated image/NC/no.3/NC BAT_no.3_RGB_Merge-4.tif]

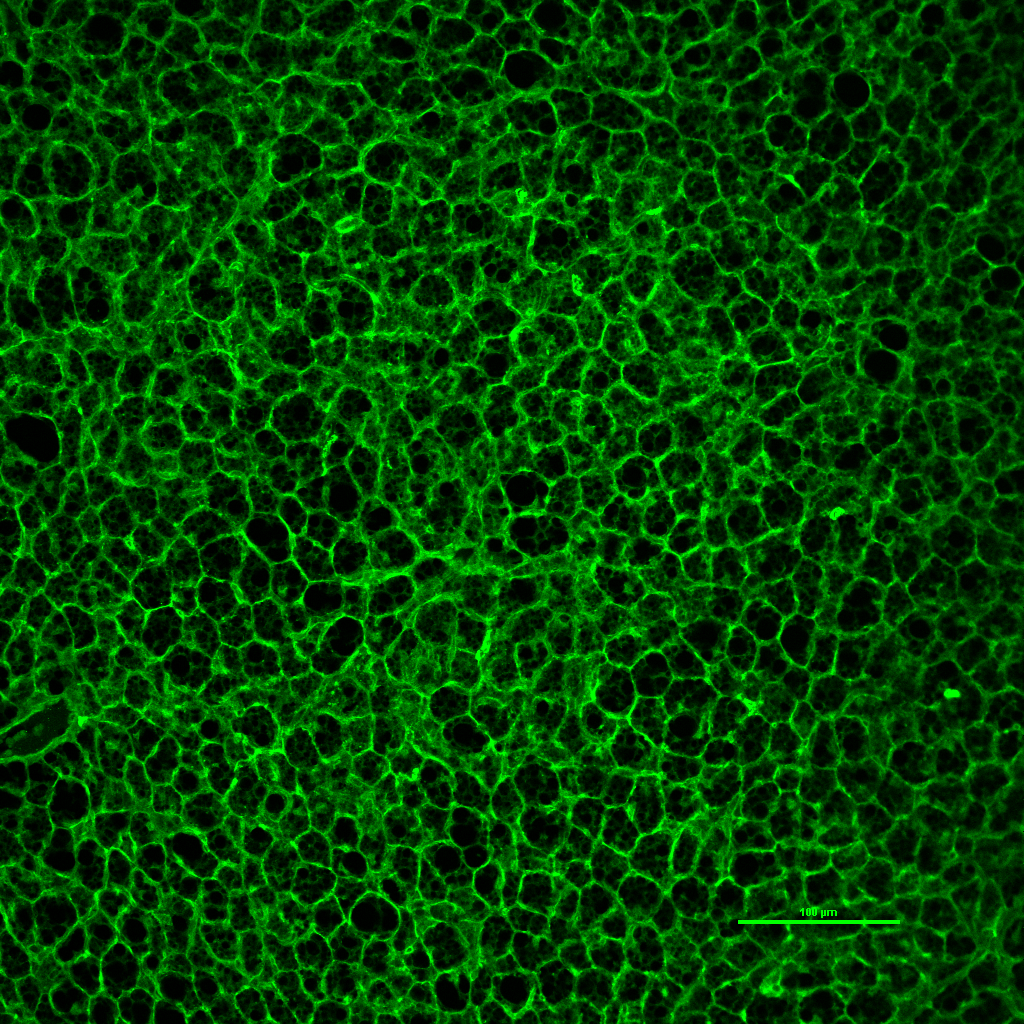

Supplement: Supplementary file 3 — Source data Fig. 1 [file 44318_2024_196_MOESM3_ESM.zip › Figure 1/Figure 1-D/Quantificated image/NC/no.3/NC BAT_no.3_RGB_WGA lectin_FITC-1.tif]
