# Supplementary figures and images for: PCPE-1, a brown adipose tissue-derived cytokine, promotes obesity-induced liver fibrosis (part 3 of 6)
Source: EMBO J. 2024 Aug 19;43(21):4846–69. doi: 10.1038/s44318-024-00196-0 (PMC11535236; doi:10.1038/s44318-024-00196-0)

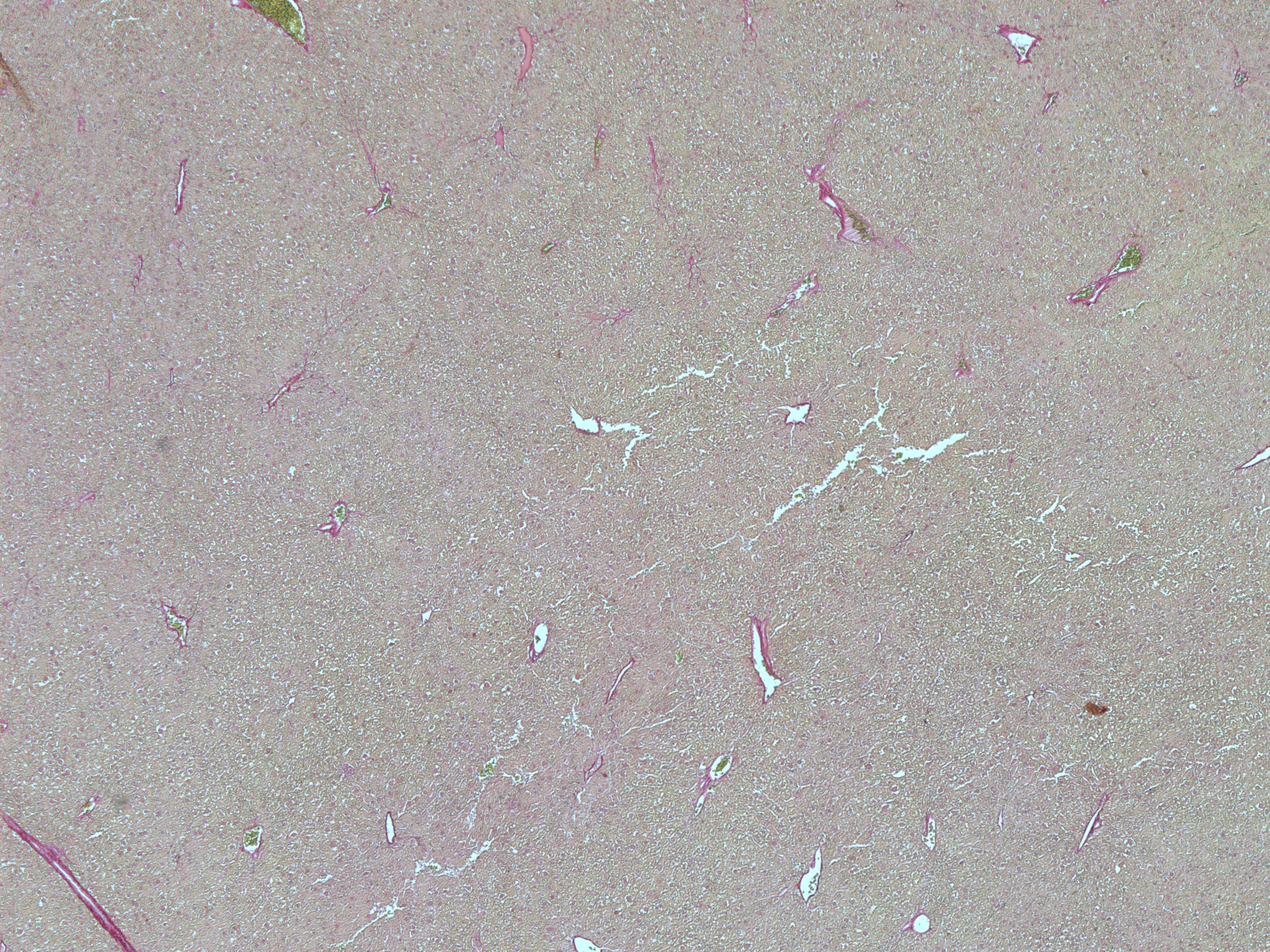

Supplement: Supplementary file 5 — Source data Fig. 3 [file 44318_2024_196_MOESM5_ESM.zip › Figure 3/Figure 3-E/Demonstrated image/NC Pcolce KO/NC Pcolce KO 4x.tif]

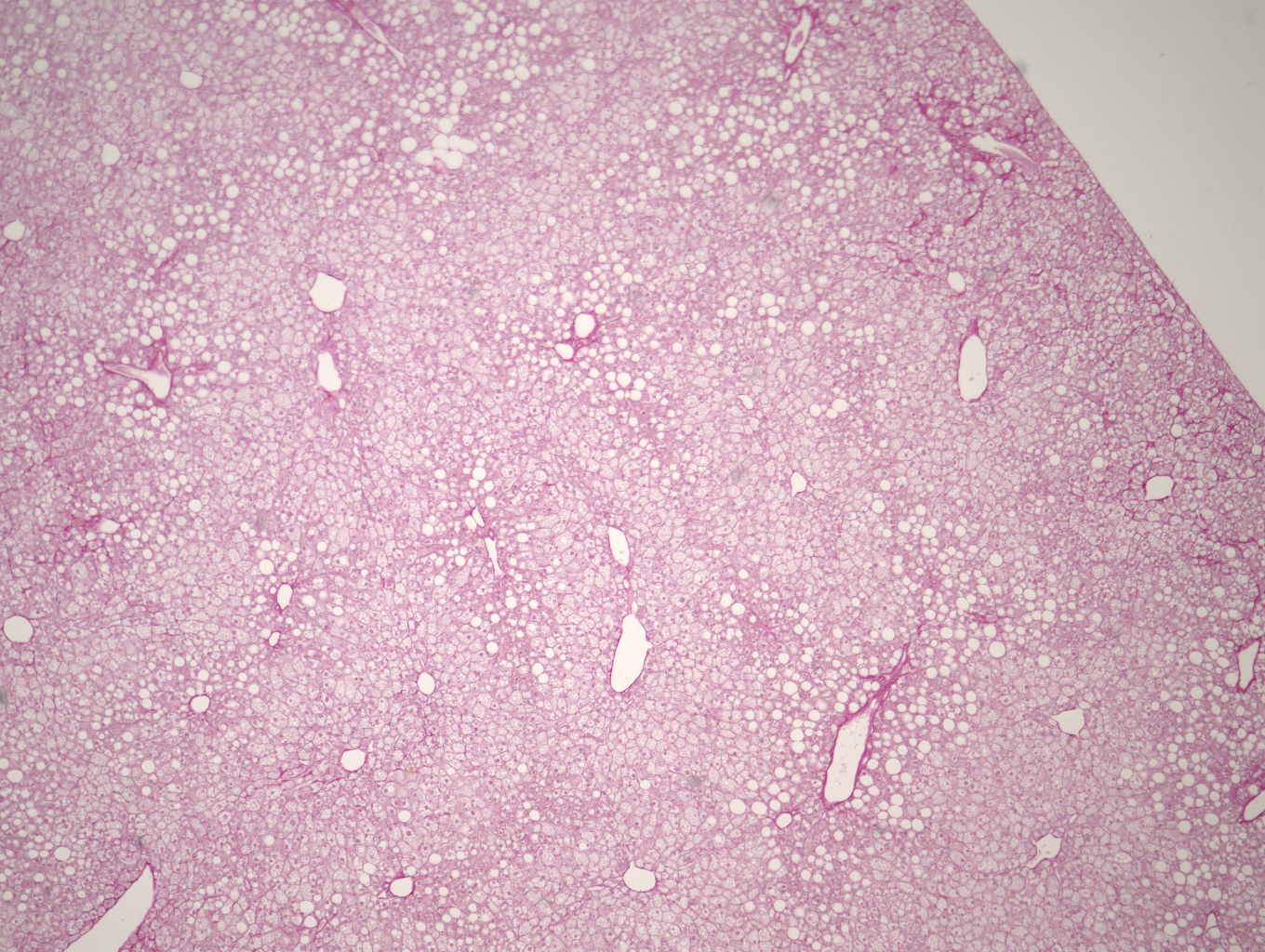

Supplement: Supplementary file 5 — Source data Fig. 3 [file 44318_2024_196_MOESM5_ESM.zip › Figure 3/Figure 3-E/Demonstrated image/HFD Pcolce KO/HFD Pcolce KO x4.tif]

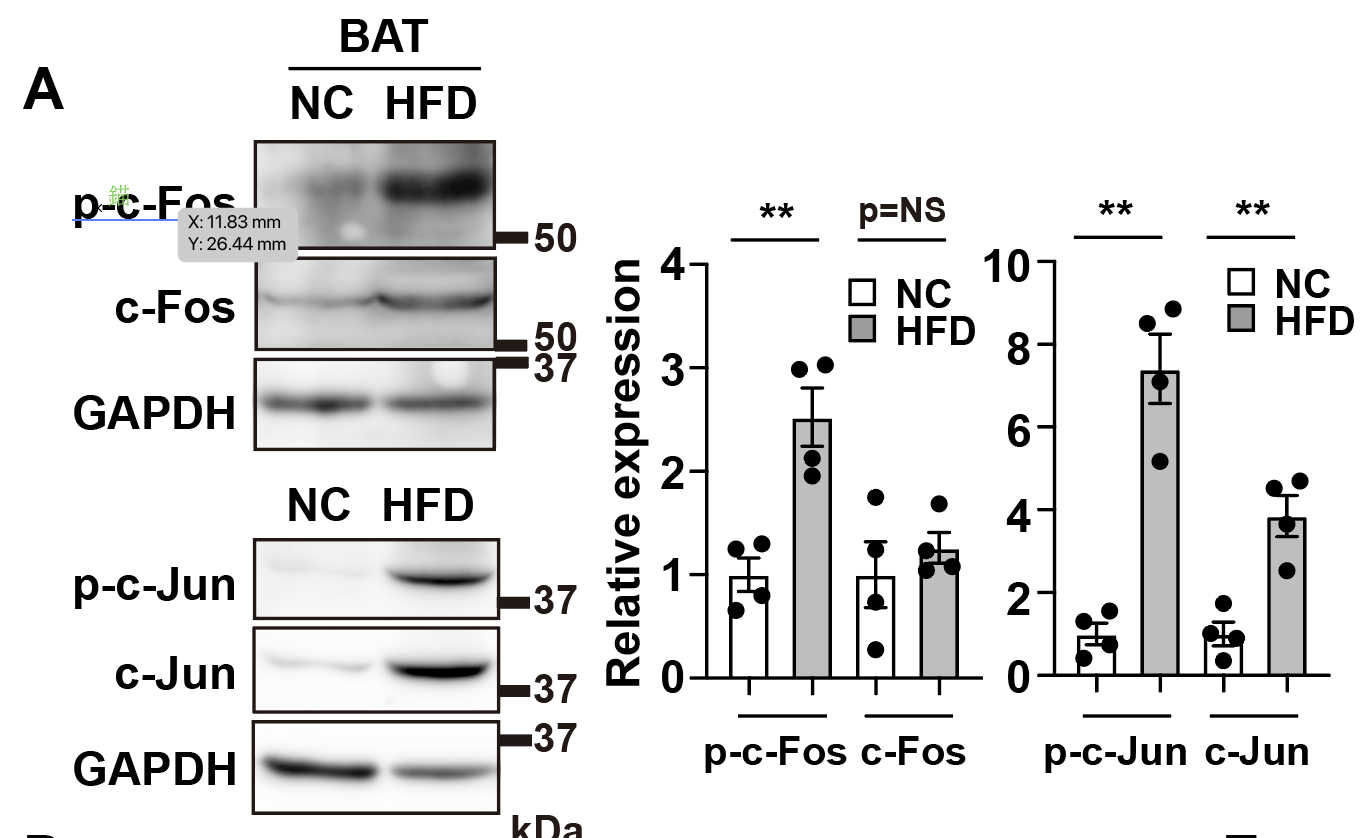

Supplement: Supplementary file 6 — Source data Fig. 4 [file 44318_2024_196_MOESM6_ESM.zip › Figure 4/Figure 4-A/Fig.4A.png]

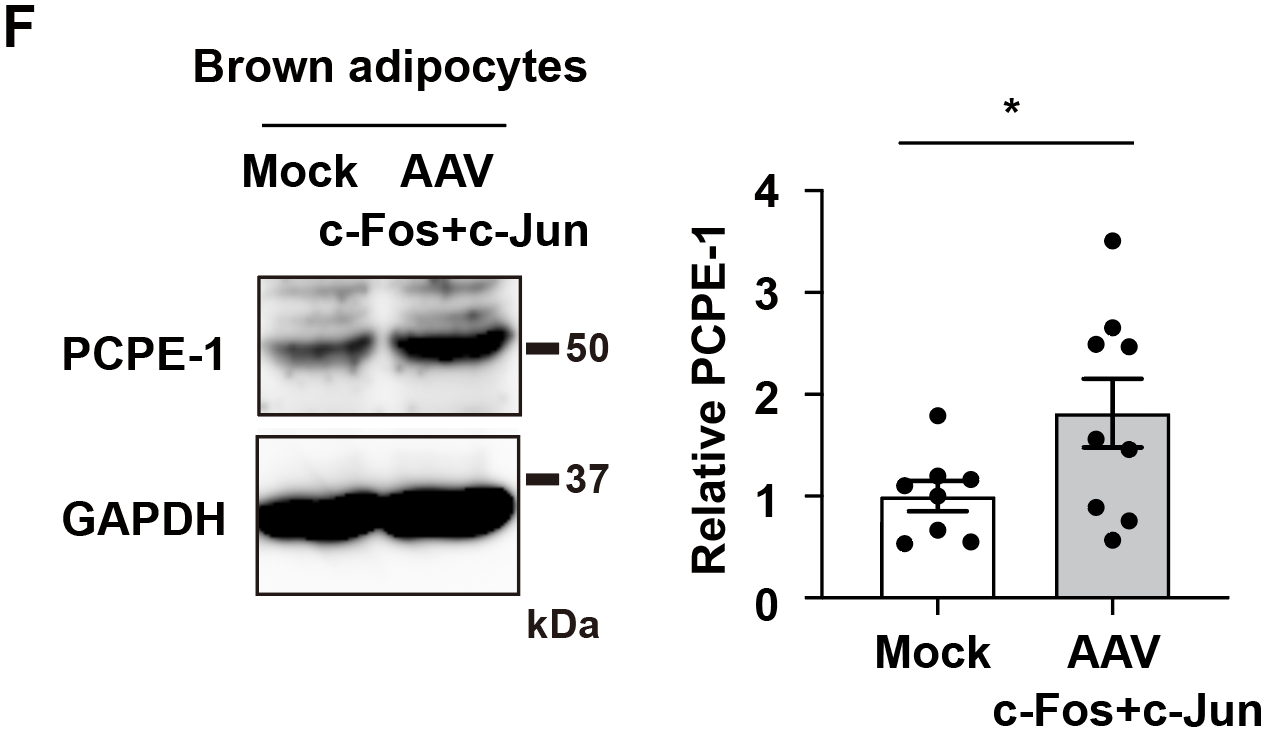

Supplement: Supplementary file 6 — Source data Fig. 4 [file 44318_2024_196_MOESM6_ESM.zip › Figure 4/Figure 4-F/Fig.4F.png]

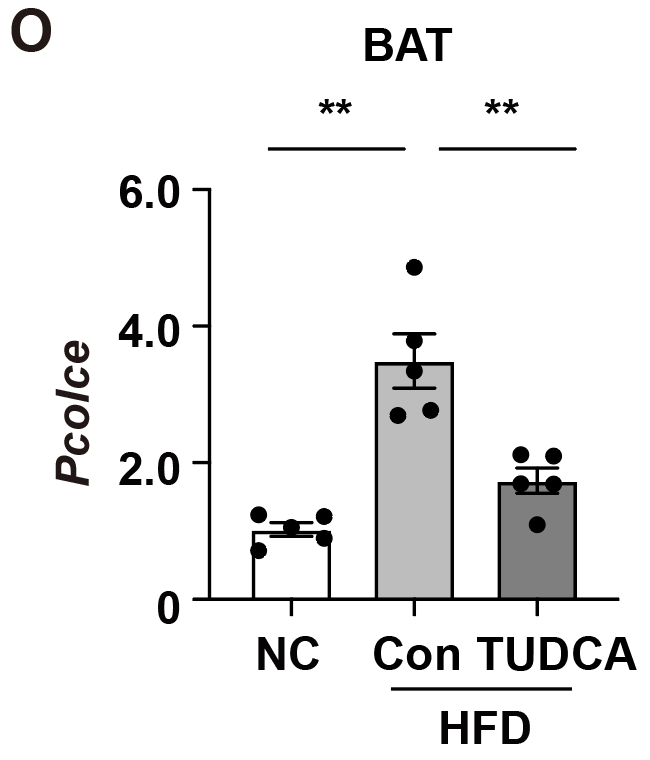

Supplement: Supplementary file 6 — Source data Fig. 4 [file 44318_2024_196_MOESM6_ESM.zip › Figure 4/Figure 4-O/Fig.4O.png]

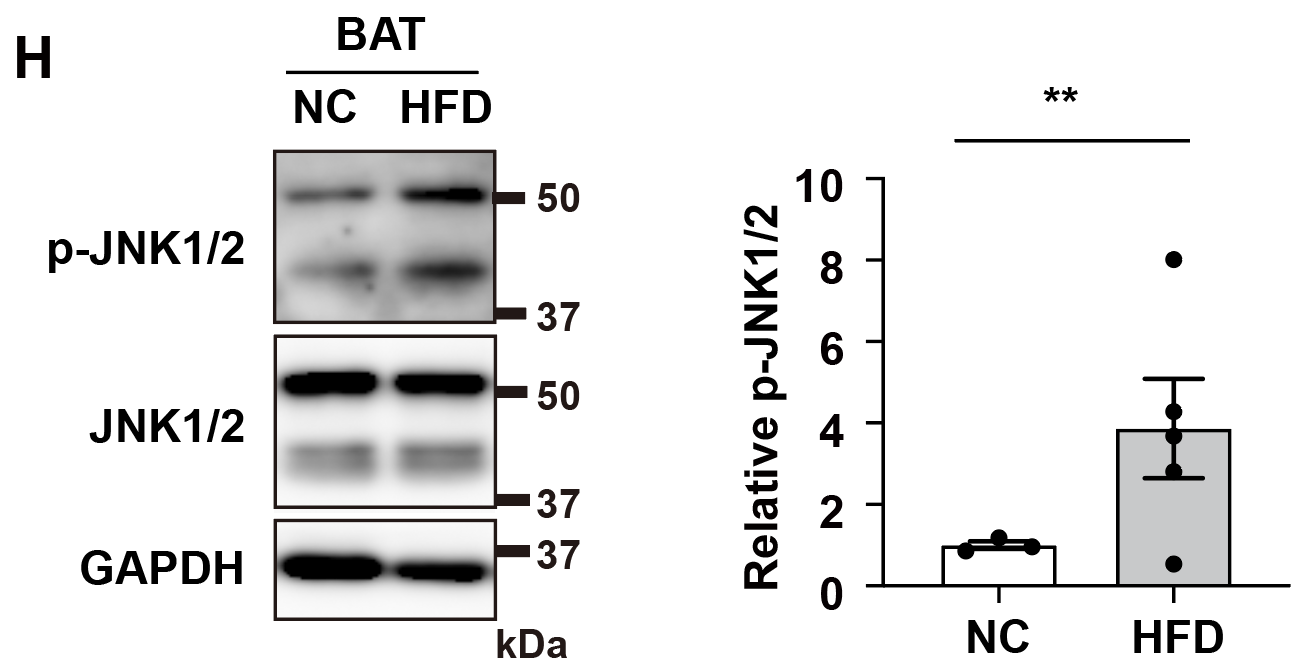

Supplement: Supplementary file 6 — Source data Fig. 4 [file 44318_2024_196_MOESM6_ESM.zip › Figure 4/Figure 4-H/Fig.4H.png]

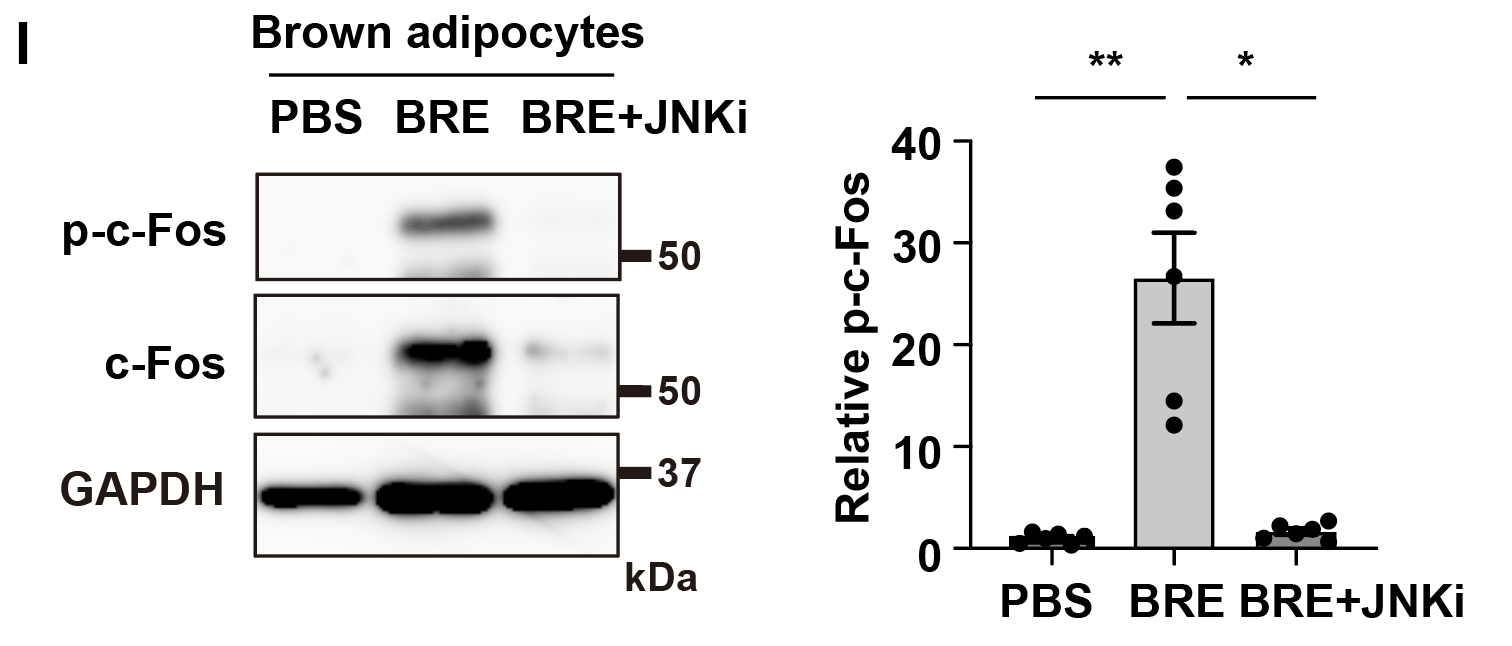

Supplement: Supplementary file 6 — Source data Fig. 4 [file 44318_2024_196_MOESM6_ESM.zip › Figure 4/Figure 4-I/Fig.4I.png]

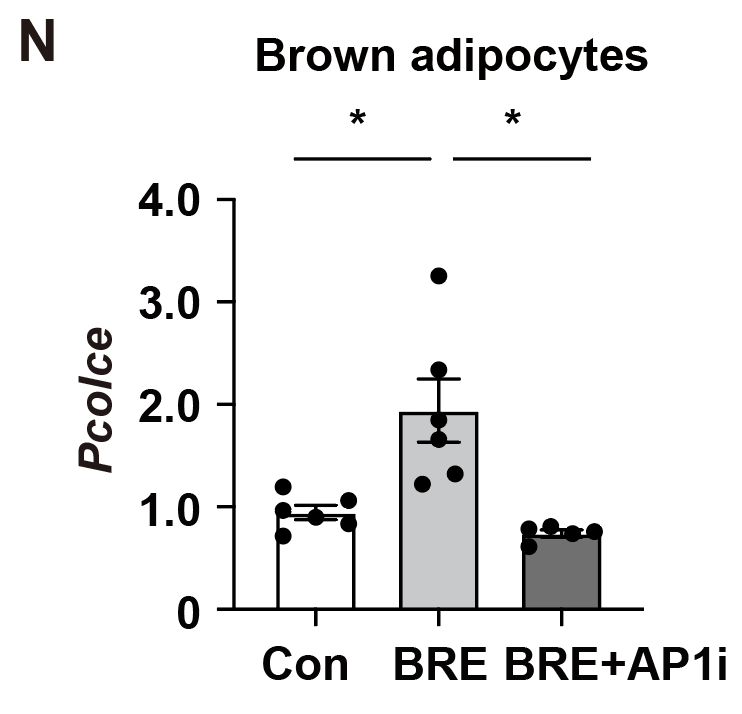

Supplement: Supplementary file 6 — Source data Fig. 4 [file 44318_2024_196_MOESM6_ESM.zip › Figure 4/Figure 4-N/Fig.4N.png]

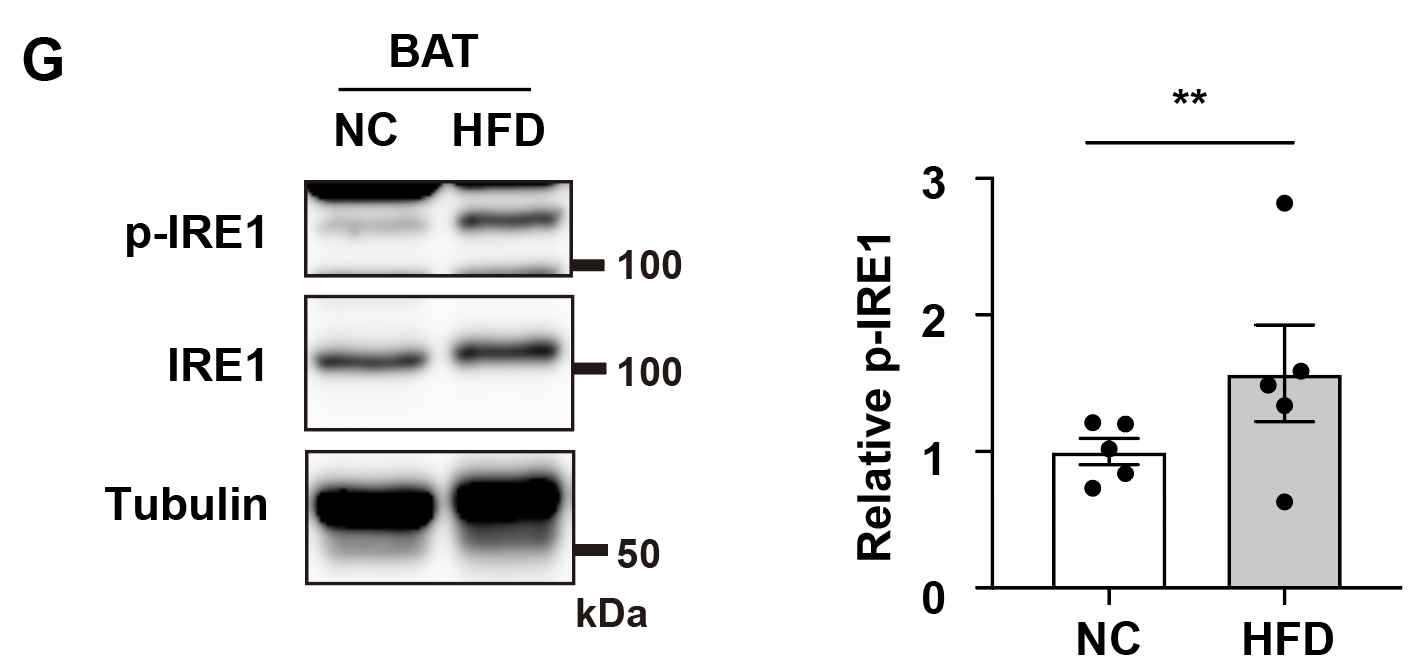

Supplement: Supplementary file 6 — Source data Fig. 4 [file 44318_2024_196_MOESM6_ESM.zip › Figure 4/Figure 4-G/Fig.4G.png]

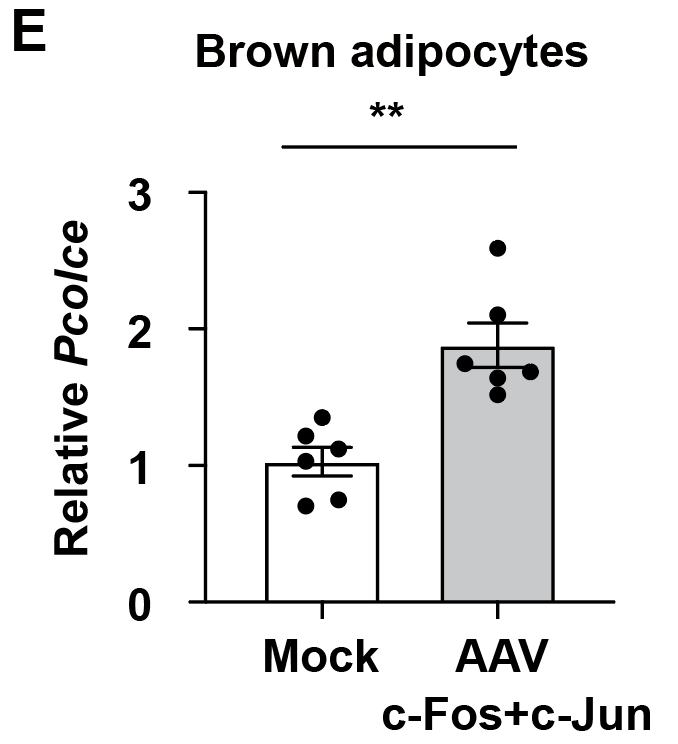

Supplement: Supplementary file 6 — Source data Fig. 4 [file 44318_2024_196_MOESM6_ESM.zip › Figure 4/Figure 4-E/Fig.4E.png]

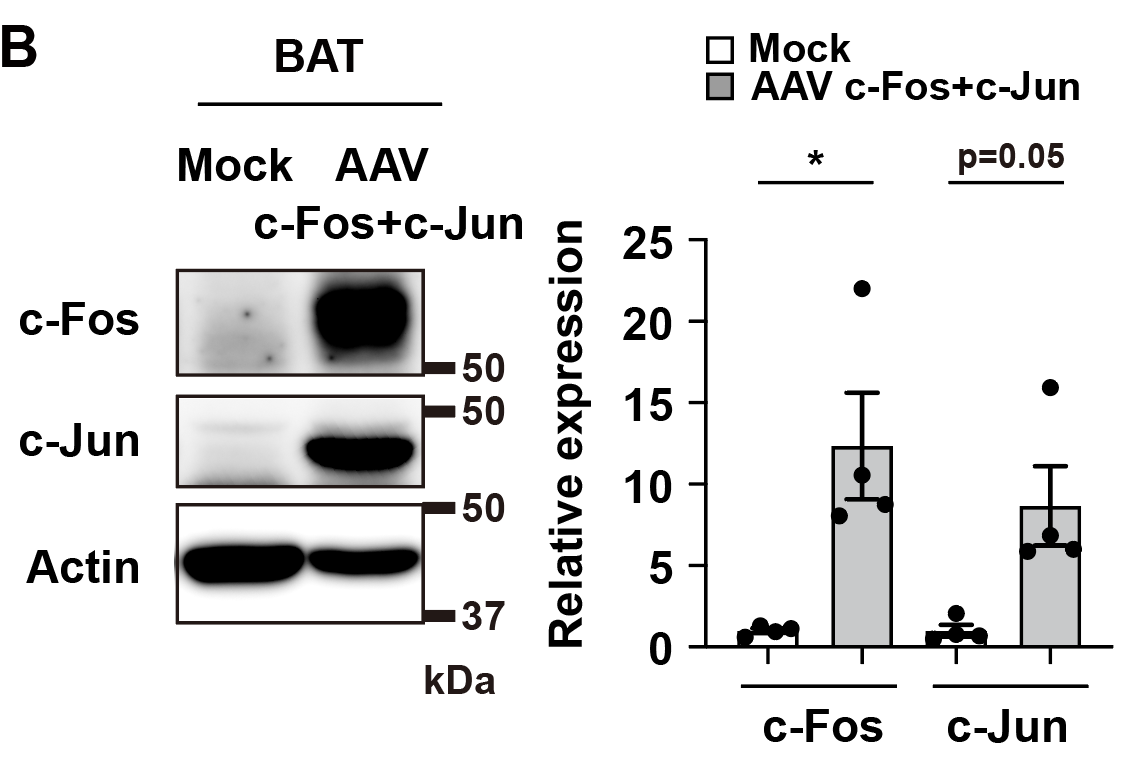

Supplement: Supplementary file 6 — Source data Fig. 4 [file 44318_2024_196_MOESM6_ESM.zip › Figure 4/Figure 4-B/Fig.4B.png]

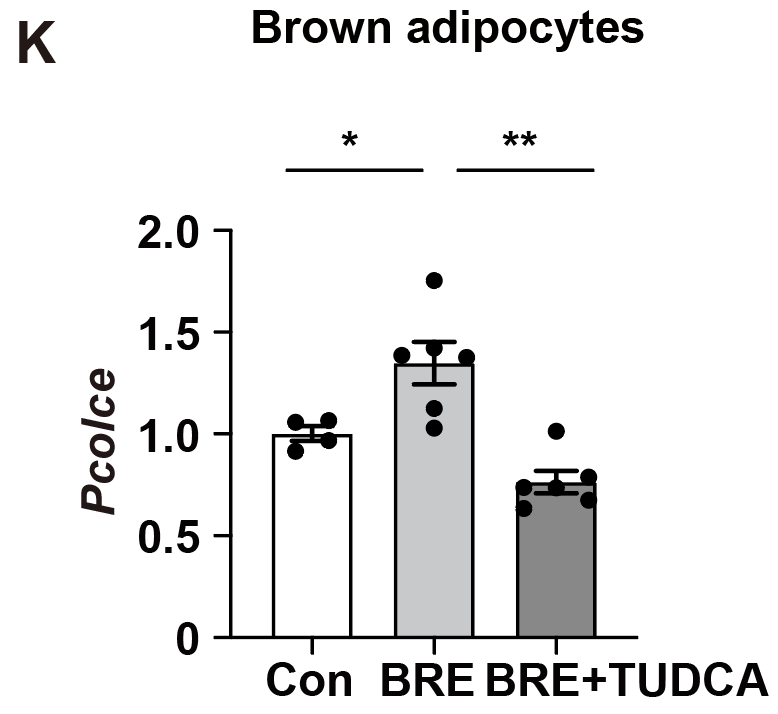

Supplement: Supplementary file 6 — Source data Fig. 4 [file 44318_2024_196_MOESM6_ESM.zip › Figure 4/Figure 4-K/Fig.4K.png]

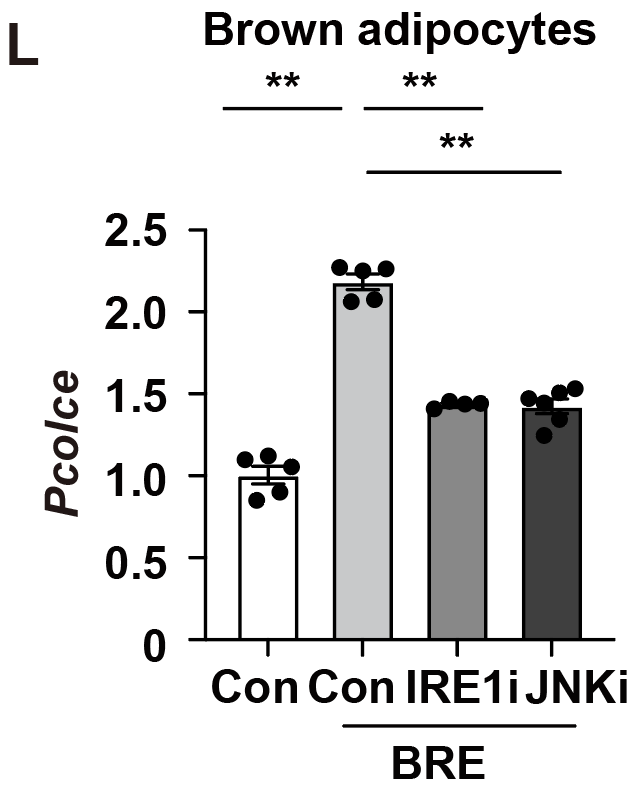

Supplement: Supplementary file 6 — Source data Fig. 4 [file 44318_2024_196_MOESM6_ESM.zip › Figure 4/Figure 4-L/Fig.4L.png]

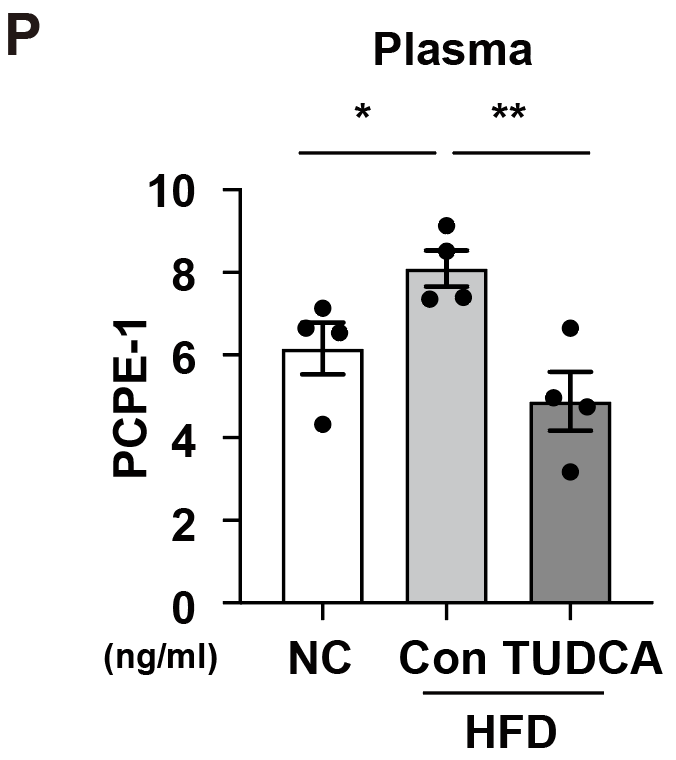

Supplement: Supplementary file 6 — Source data Fig. 4 [file 44318_2024_196_MOESM6_ESM.zip › Figure 4/Figure 4-P/Fig.4P.png]

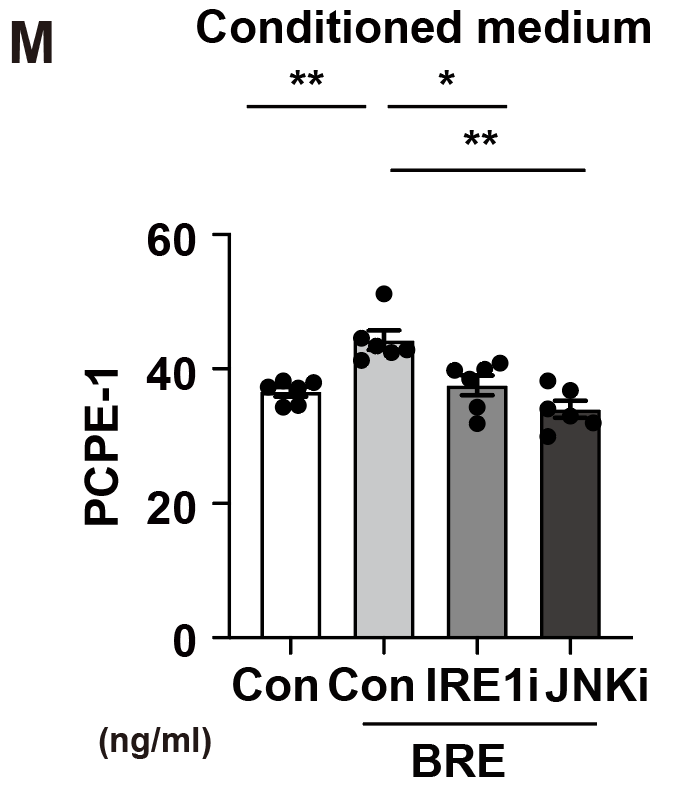

Supplement: Supplementary file 6 — Source data Fig. 4 [file 44318_2024_196_MOESM6_ESM.zip › Figure 4/Figure 4-M/Fig.4M.png]

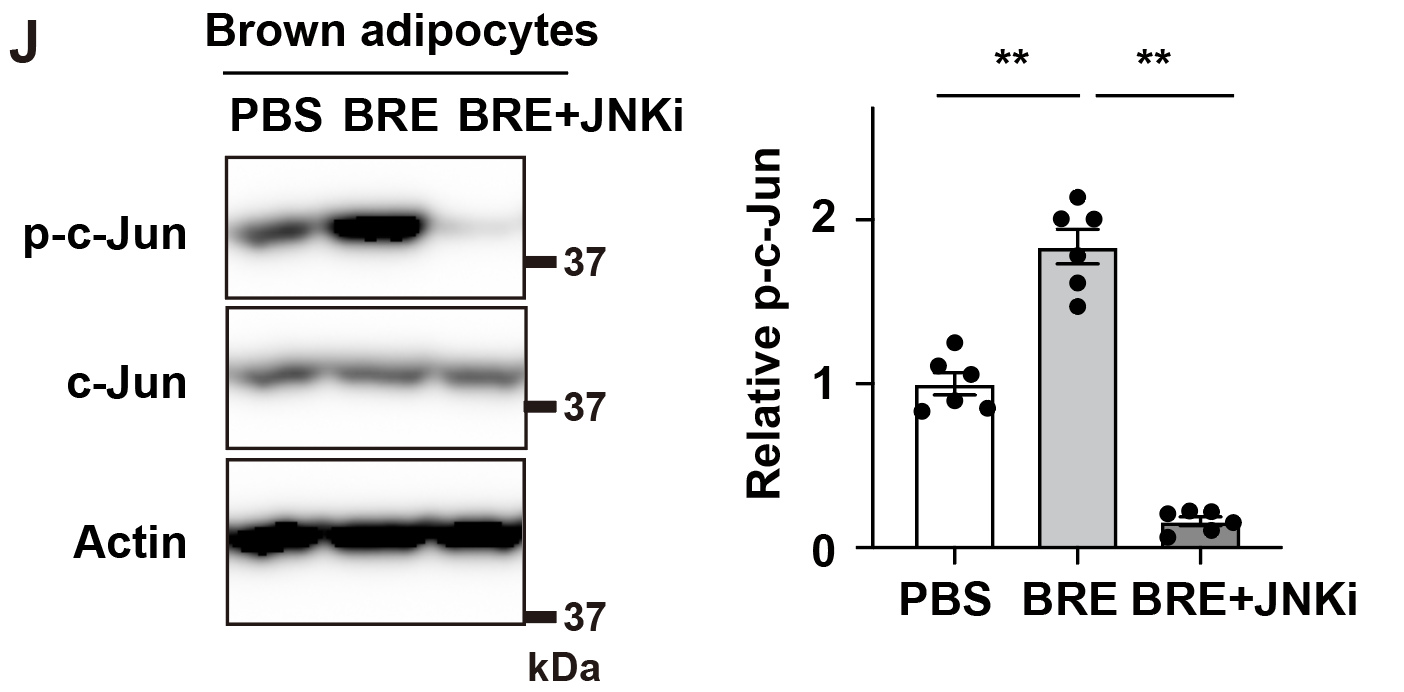

Supplement: Supplementary file 6 — Source data Fig. 4 [file 44318_2024_196_MOESM6_ESM.zip › Figure 4/Figure 4-J/Fig.4J.png]

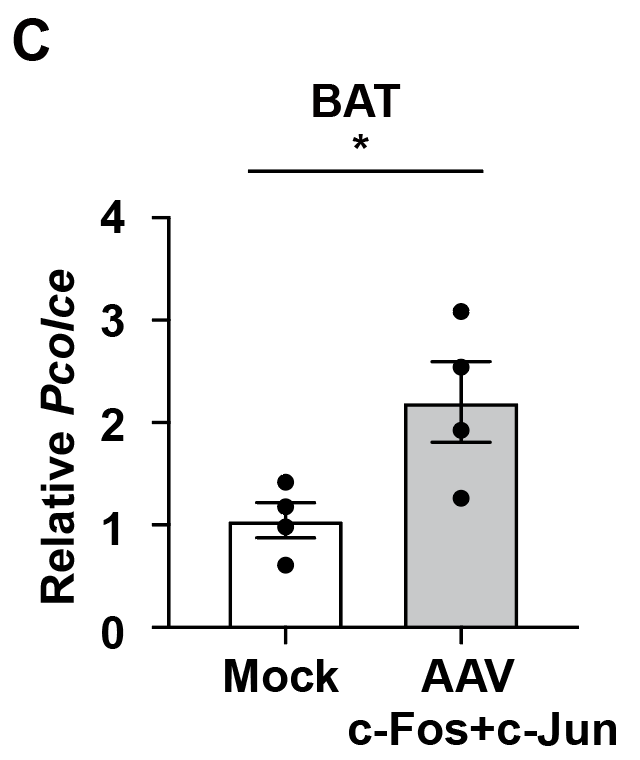

Supplement: Supplementary file 6 — Source data Fig. 4 [file 44318_2024_196_MOESM6_ESM.zip › Figure 4/Figure 4-C/Fig.4C.png]

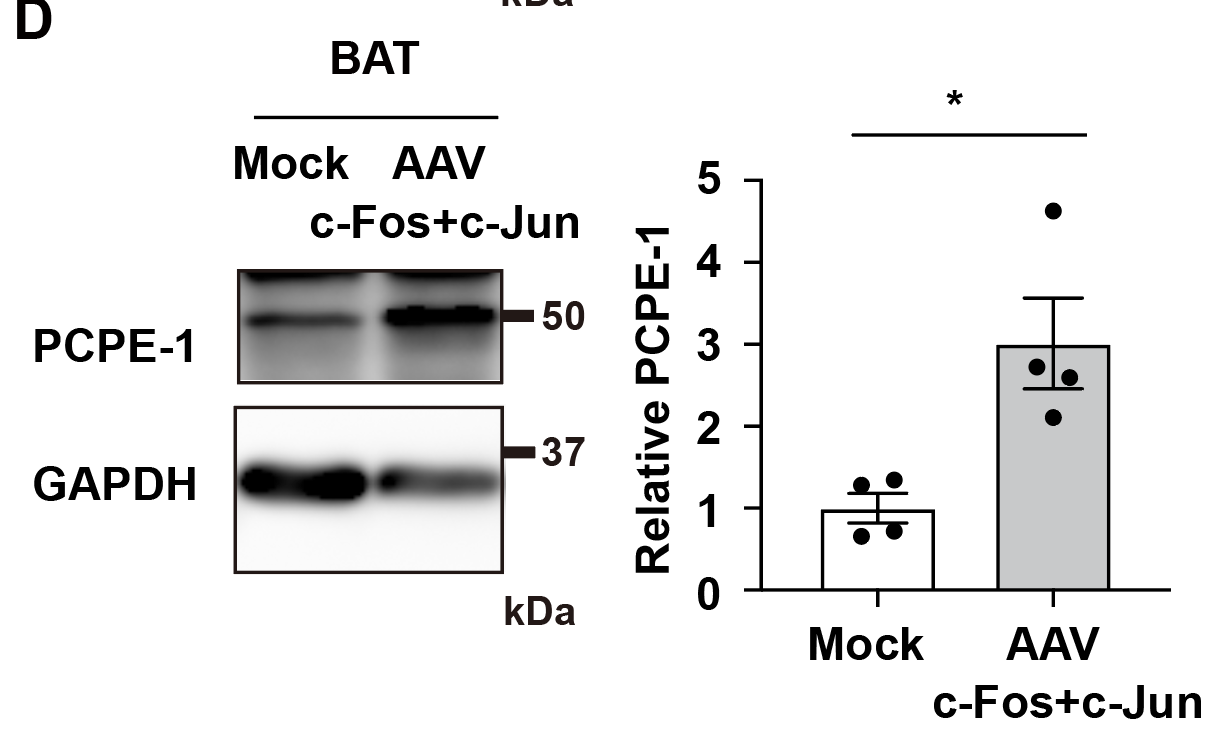

Supplement: Supplementary file 6 — Source data Fig. 4 [file 44318_2024_196_MOESM6_ESM.zip › Figure 4/Figure 4-D/Fig.4D.png]

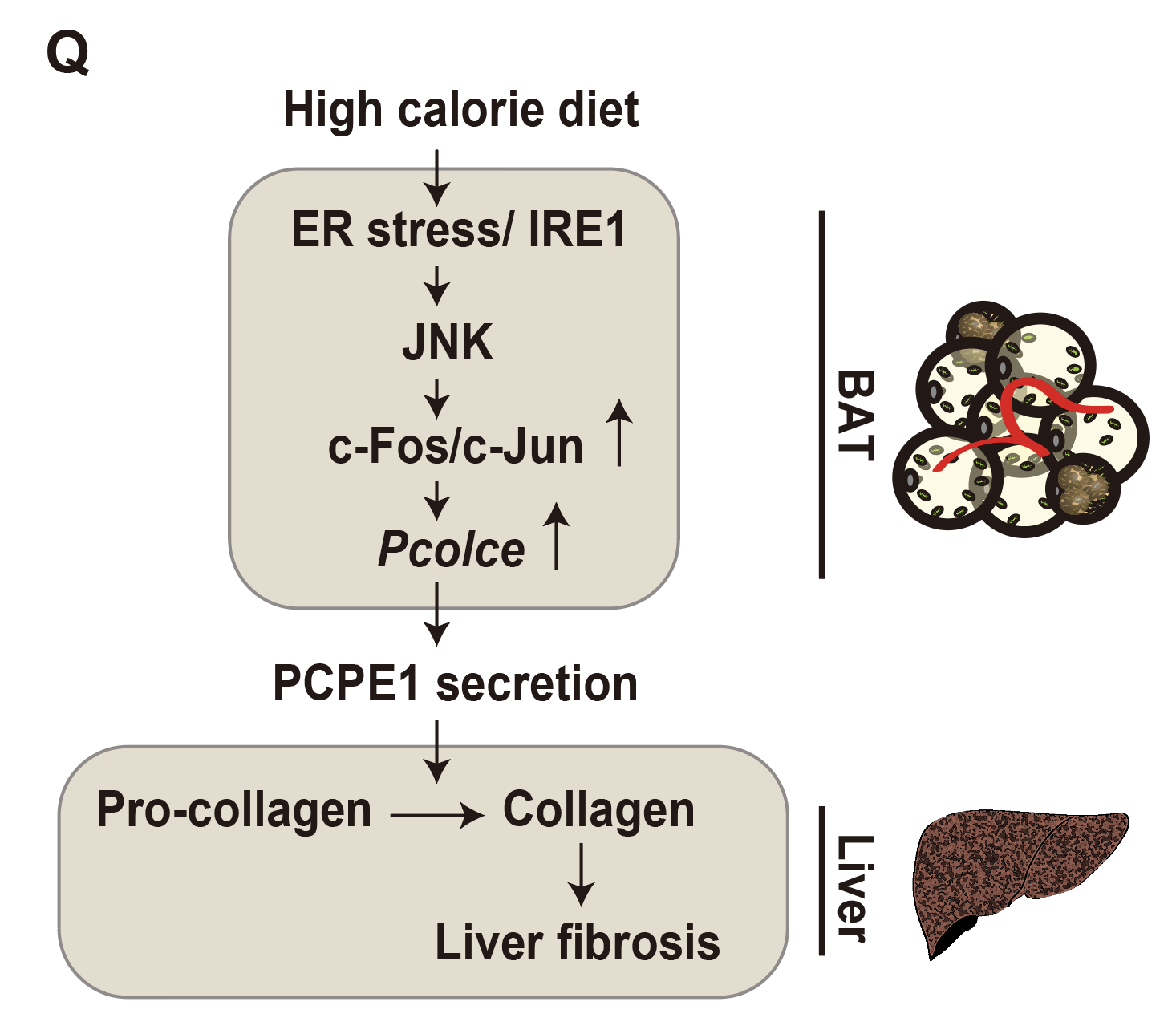

Supplement: Supplementary file 6 — Source data Fig. 4 [file 44318_2024_196_MOESM6_ESM.zip › Figure 4/Figure 4-Q/Fig.4Q.png]

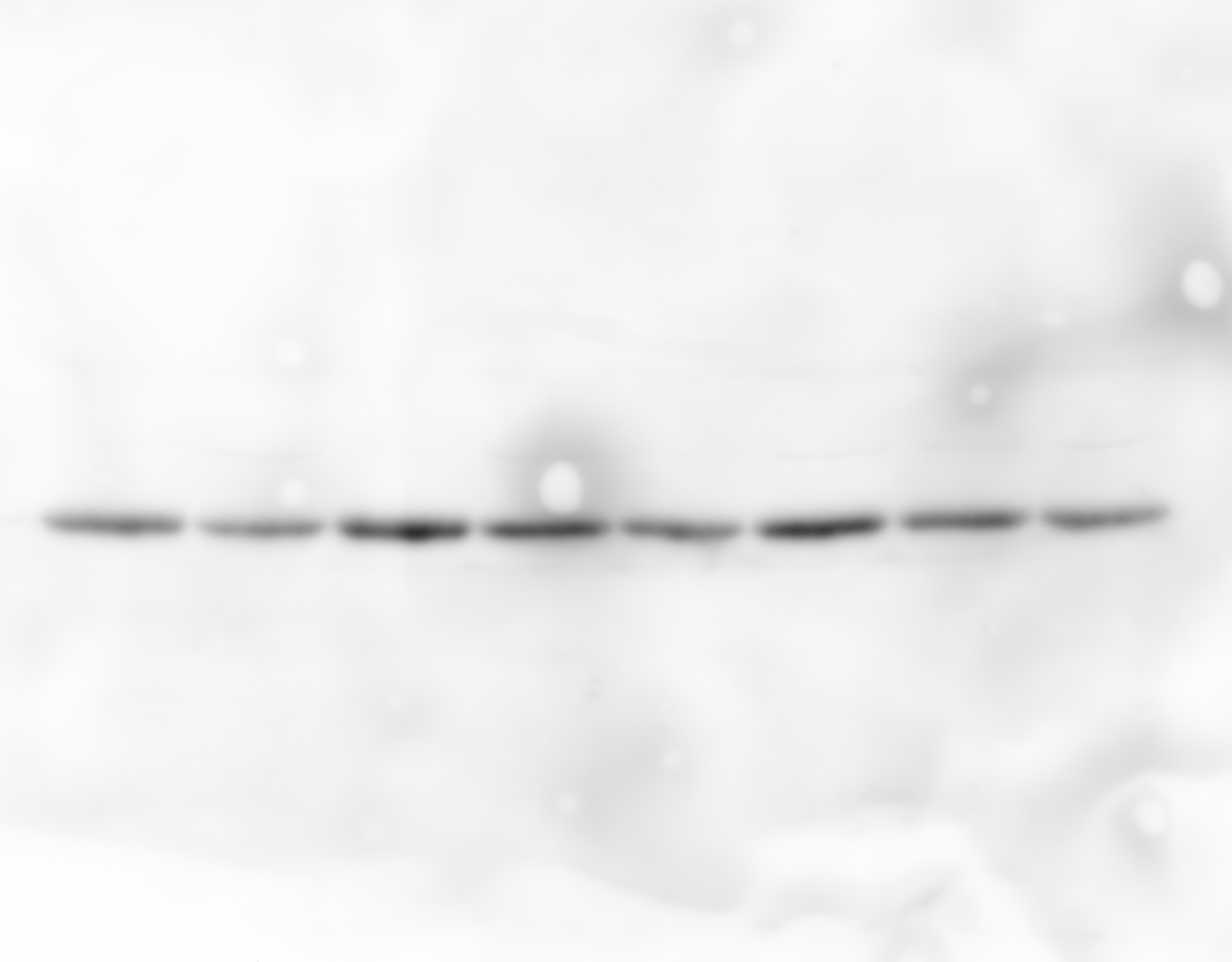

Supplement: Supplementary file 6 — Source data Fig. 4 [file 44318_2024_196_MOESM6_ESM.zip › Figure 4/Figure 4-A/Quantificated image/GAPDH (for p-c-Fos and c-Fos).tif]

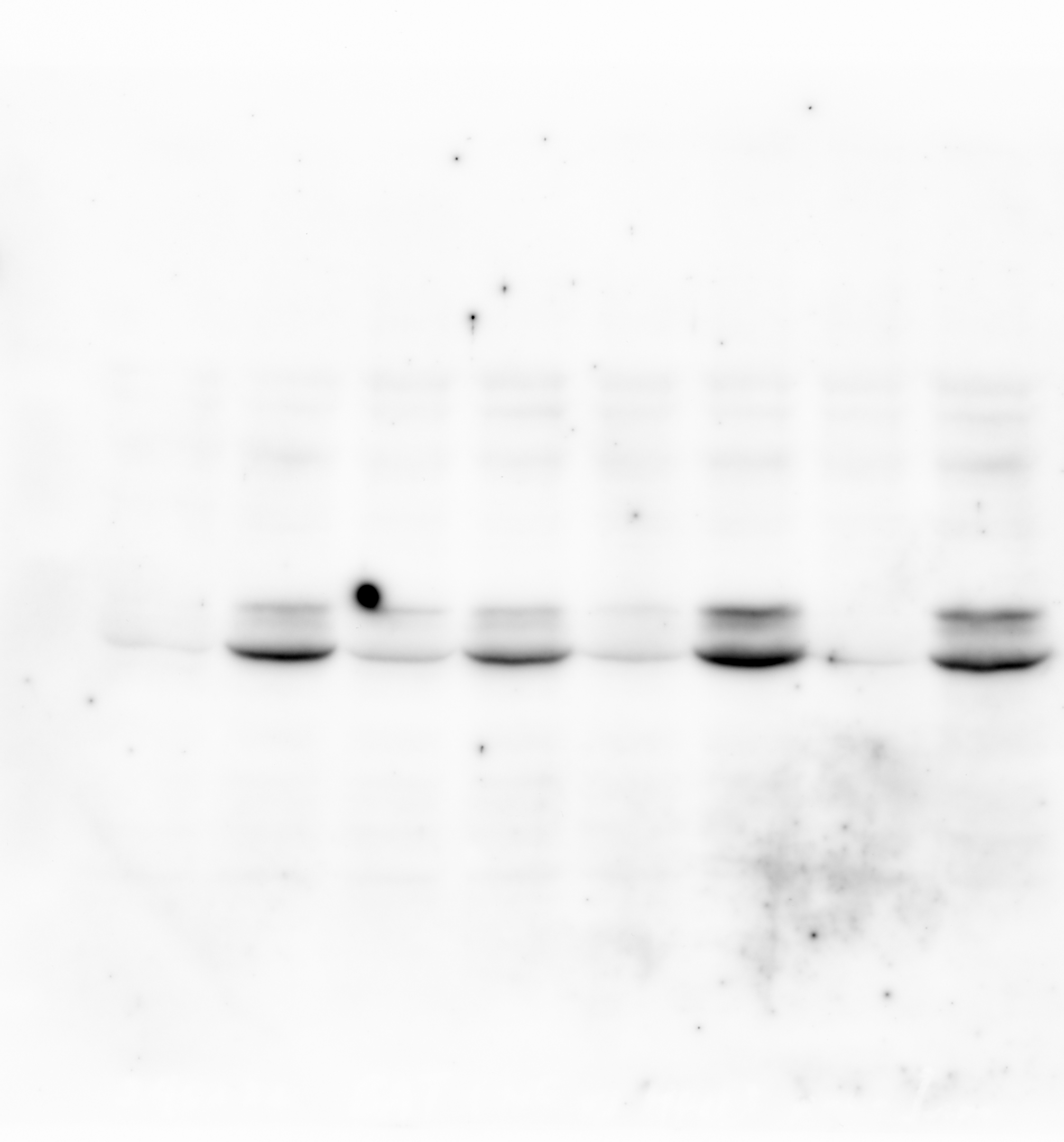

Supplement: Supplementary file 6 — Source data Fig. 4 [file 44318_2024_196_MOESM6_ESM.zip › Figure 4/Figure 4-A/Quantificated image/p-c-Jun.tif]

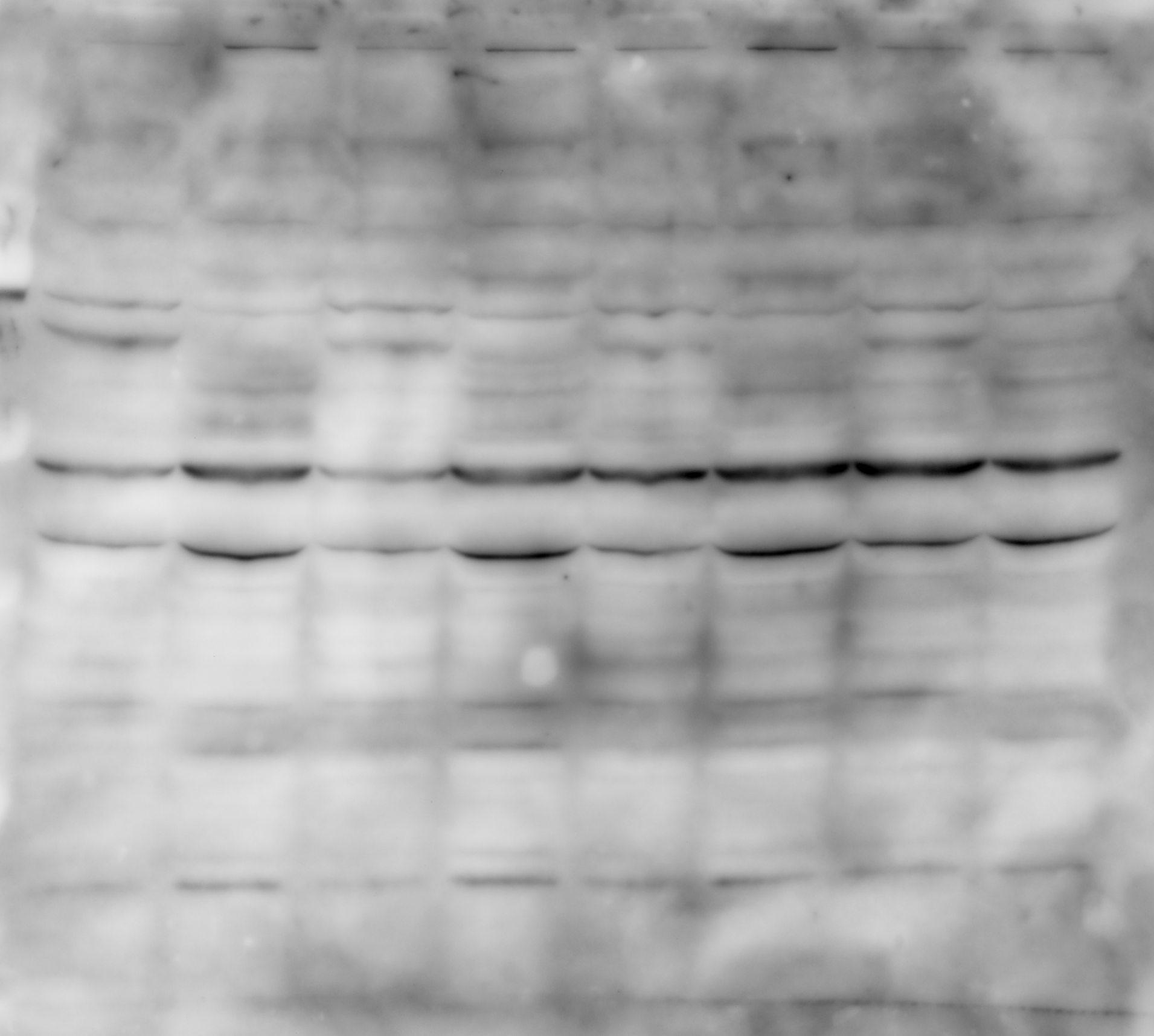

Supplement: Supplementary file 6 — Source data Fig. 4 [file 44318_2024_196_MOESM6_ESM.zip › Figure 4/Figure 4-A/Quantificated image/c-Fos.tif]

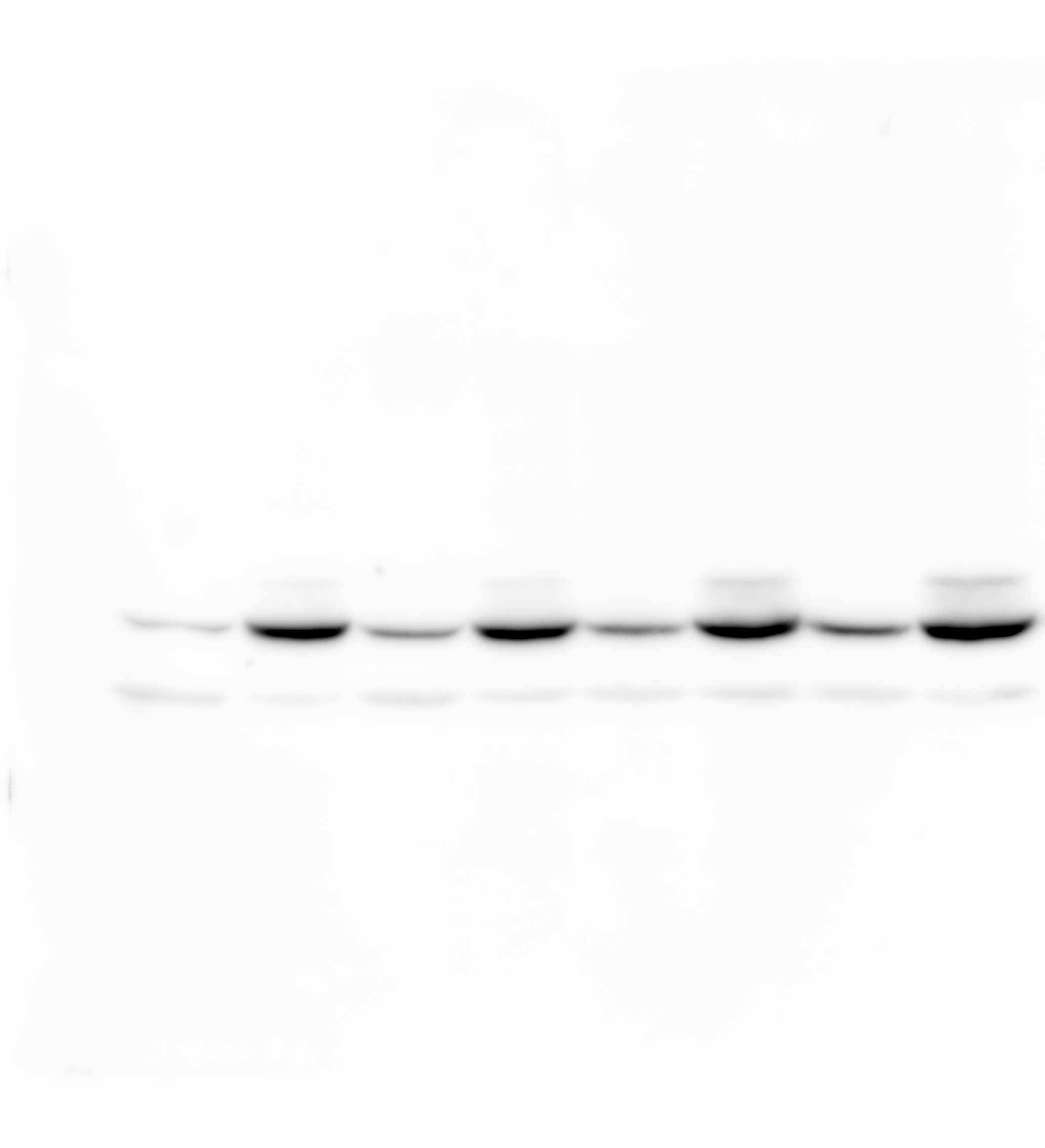

Supplement: Supplementary file 6 — Source data Fig. 4 [file 44318_2024_196_MOESM6_ESM.zip › Figure 4/Figure 4-A/Quantificated image/c-Jun.tif]

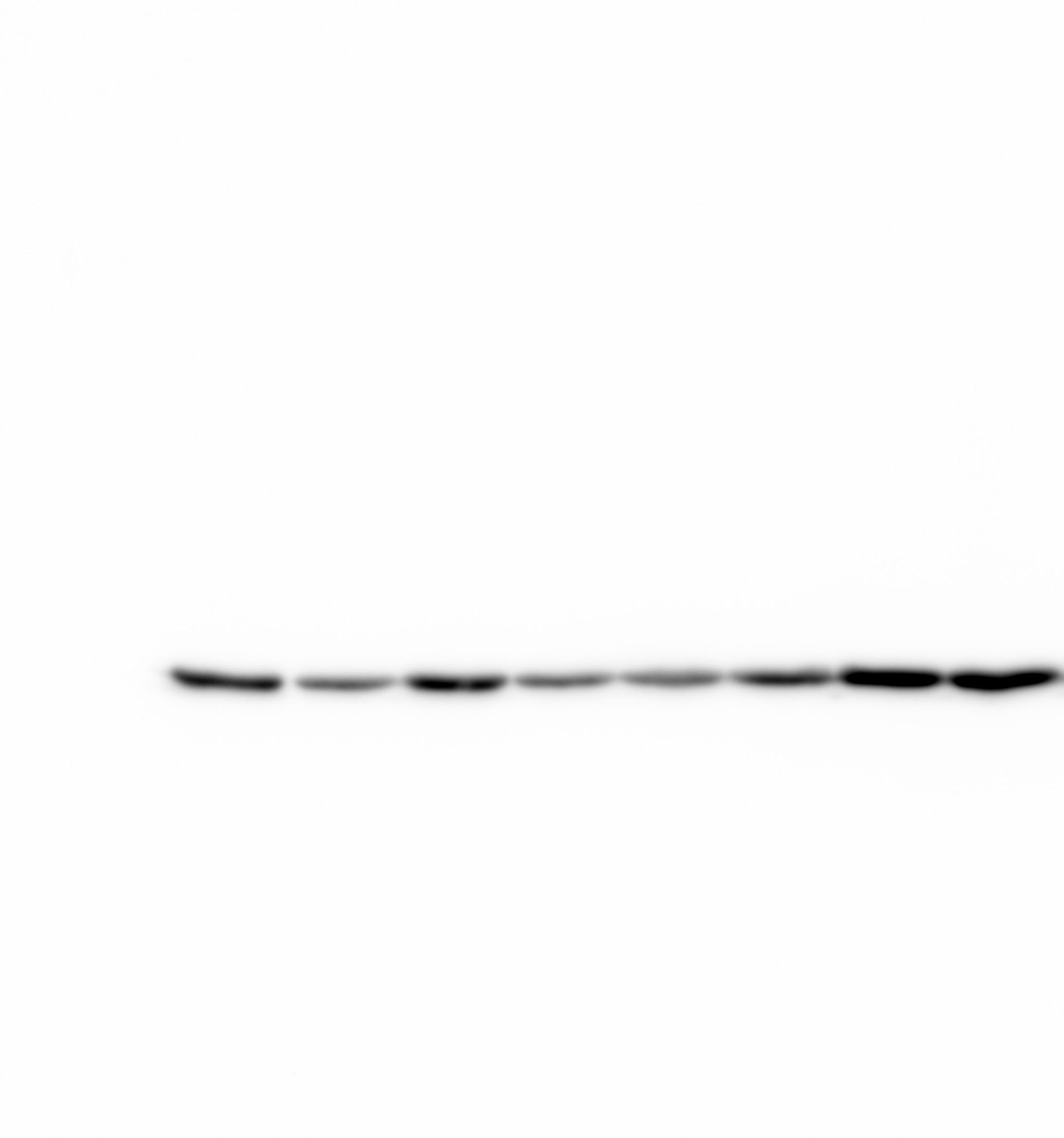

Supplement: Supplementary file 6 — Source data Fig. 4 [file 44318_2024_196_MOESM6_ESM.zip › Figure 4/Figure 4-A/Quantificated image/GAPDH (for p-c-Jun and c-Jun).tif]

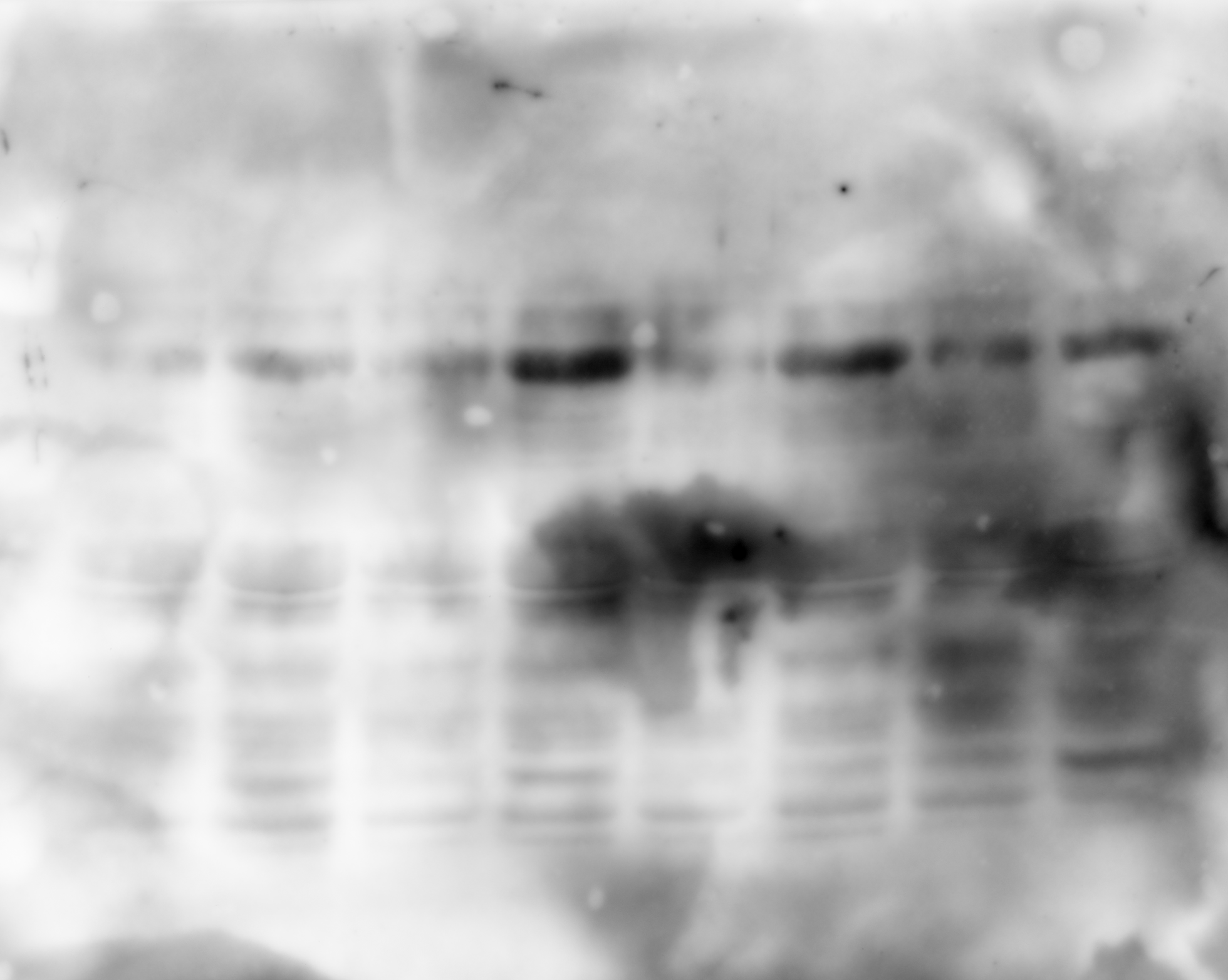

Supplement: Supplementary file 6 — Source data Fig. 4 [file 44318_2024_196_MOESM6_ESM.zip › Figure 4/Figure 4-A/Quantificated image/p-c-Fos.tif]

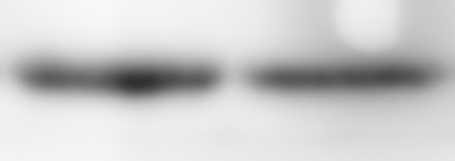

Supplement: Supplementary file 6 — Source data Fig. 4 [file 44318_2024_196_MOESM6_ESM.zip › Figure 4/Figure 4-A/Demonstrated image/GAPDH (for p-c-Fos and c-Fos).tif]

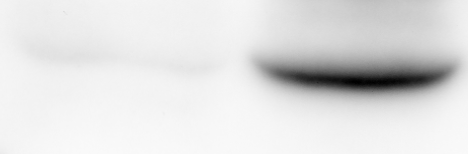

Supplement: Supplementary file 6 — Source data Fig. 4 [file 44318_2024_196_MOESM6_ESM.zip › Figure 4/Figure 4-A/Demonstrated image/p-c-Jun.tif]

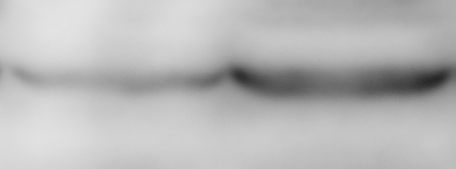

Supplement: Supplementary file 6 — Source data Fig. 4 [file 44318_2024_196_MOESM6_ESM.zip › Figure 4/Figure 4-A/Demonstrated image/c-Fos.tif]

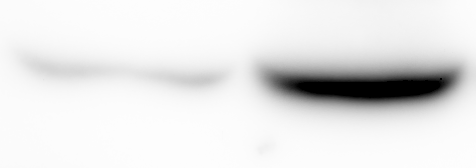

Supplement: Supplementary file 6 — Source data Fig. 4 [file 44318_2024_196_MOESM6_ESM.zip › Figure 4/Figure 4-A/Demonstrated image/c-Jun.tif]

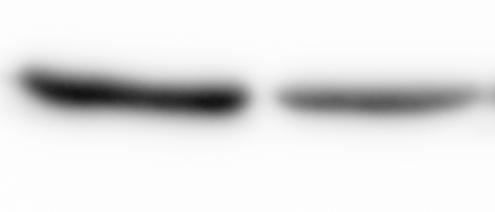

Supplement: Supplementary file 6 — Source data Fig. 4 [file 44318_2024_196_MOESM6_ESM.zip › Figure 4/Figure 4-A/Demonstrated image/GAPDH (for p-c-Jun and c-Jun).tif]

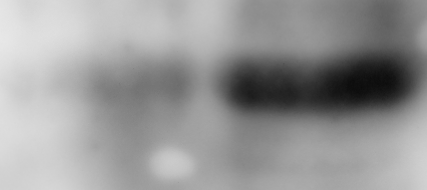

Supplement: Supplementary file 6 — Source data Fig. 4 [file 44318_2024_196_MOESM6_ESM.zip › Figure 4/Figure 4-A/Demonstrated image/p-c-Fos.tif]

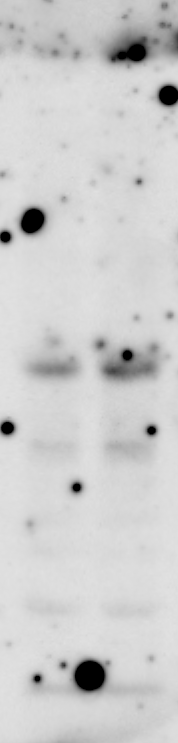

Supplement: Supplementary file 6 — Source data Fig. 4 [file 44318_2024_196_MOESM6_ESM.zip › Figure 4/Figure 4-F/Quantificated image/PCPE-1 rep 1.tif]

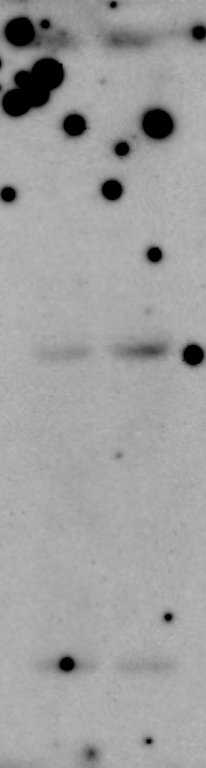

Supplement: Supplementary file 6 — Source data Fig. 4 [file 44318_2024_196_MOESM6_ESM.zip › Figure 4/Figure 4-F/Quantificated image/PCPE-1 rep 2.tif]

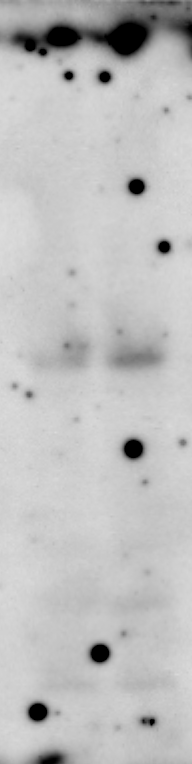

Supplement: Supplementary file 6 — Source data Fig. 4 [file 44318_2024_196_MOESM6_ESM.zip › Figure 4/Figure 4-F/Quantificated image/PCPE-1 rep 3.tif]

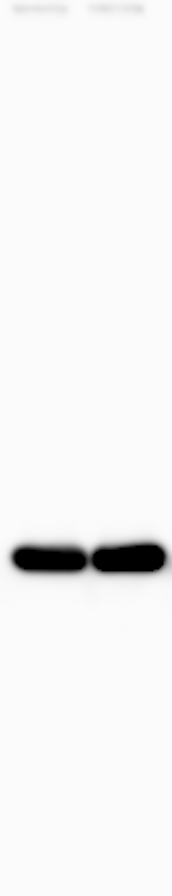

Supplement: Supplementary file 6 — Source data Fig. 4 [file 44318_2024_196_MOESM6_ESM.zip › Figure 4/Figure 4-F/Quantificated image/GAPDH rep 3.tif]

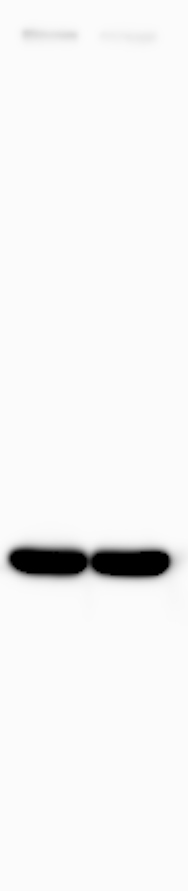

Supplement: Supplementary file 6 — Source data Fig. 4 [file 44318_2024_196_MOESM6_ESM.zip › Figure 4/Figure 4-F/Quantificated image/GAPDH rep 2.tif]

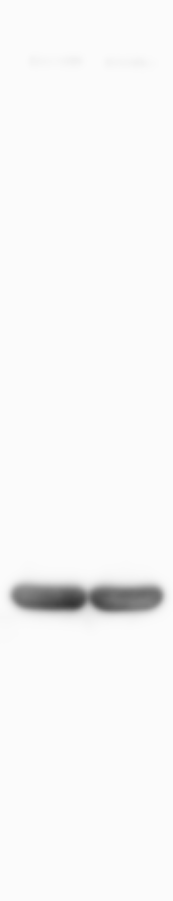

Supplement: Supplementary file 6 — Source data Fig. 4 [file 44318_2024_196_MOESM6_ESM.zip › Figure 4/Figure 4-F/Quantificated image/GAPDH rep 1.tif]

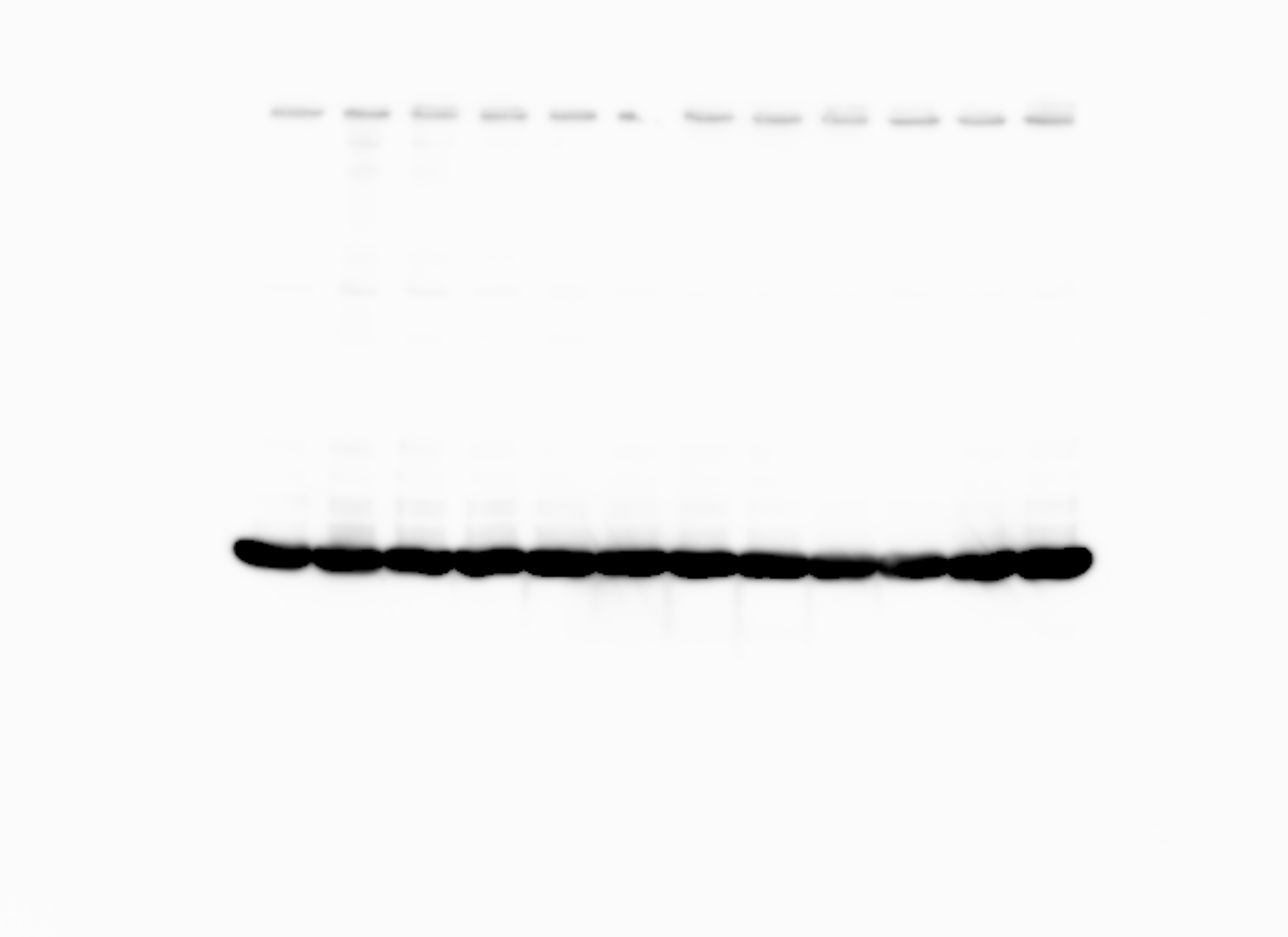

Supplement: Supplementary file 6 — Source data Fig. 4 [file 44318_2024_196_MOESM6_ESM.zip › Figure 4/Figure 4-F/Quantificated image/GAPDH.tif]

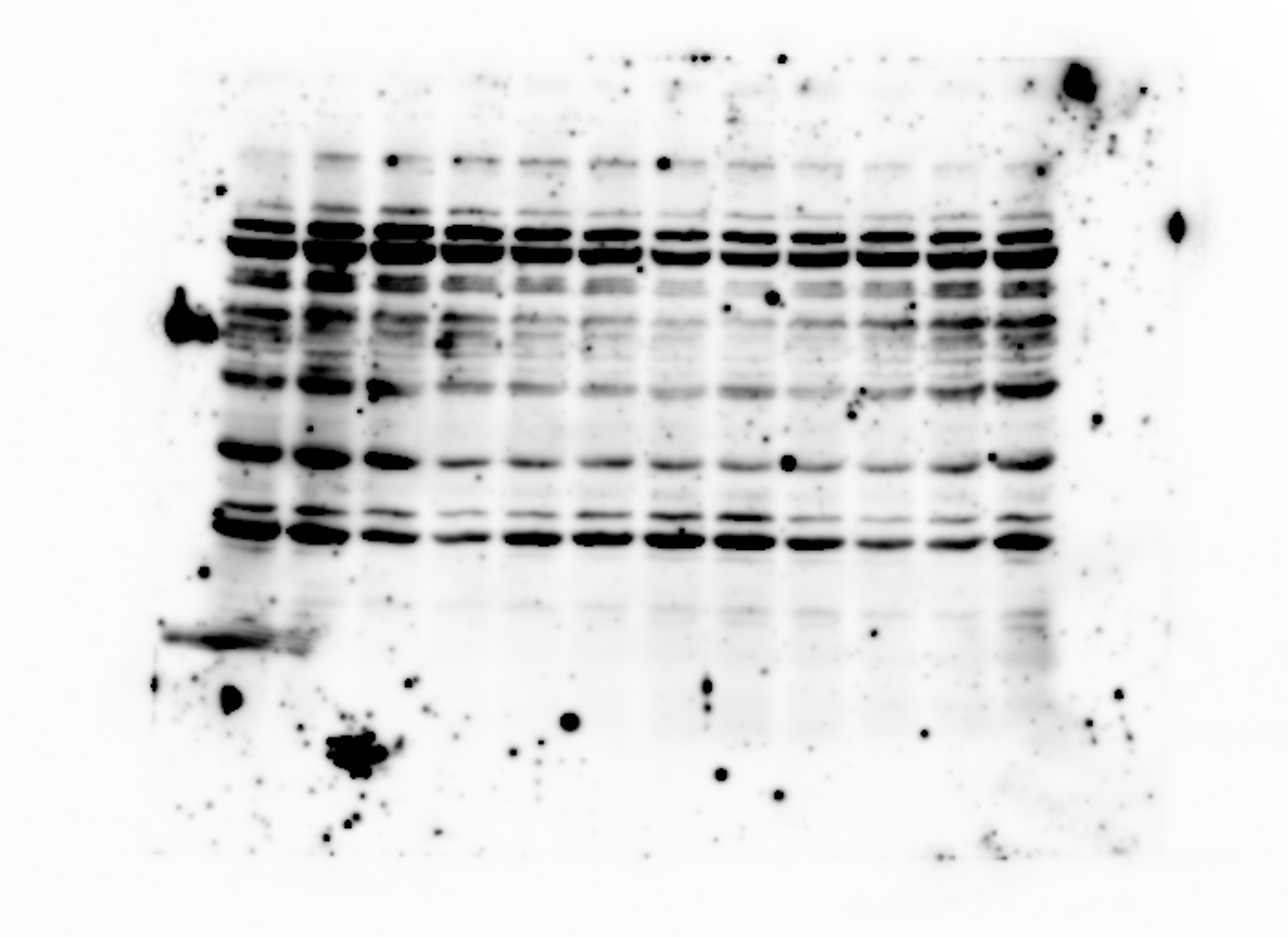

Supplement: Supplementary file 6 — Source data Fig. 4 [file 44318_2024_196_MOESM6_ESM.zip › Figure 4/Figure 4-F/Quantificated image/PCPE-1.tif]

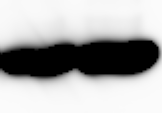

Supplement: Supplementary file 6 — Source data Fig. 4 [file 44318_2024_196_MOESM6_ESM.zip › Figure 4/Figure 4-F/Demonstrated image/GAPDH.tif]

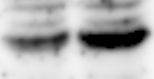

Supplement: Supplementary file 6 — Source data Fig. 4 [file 44318_2024_196_MOESM6_ESM.zip › Figure 4/Figure 4-F/Demonstrated image/PCPE-1.tif]

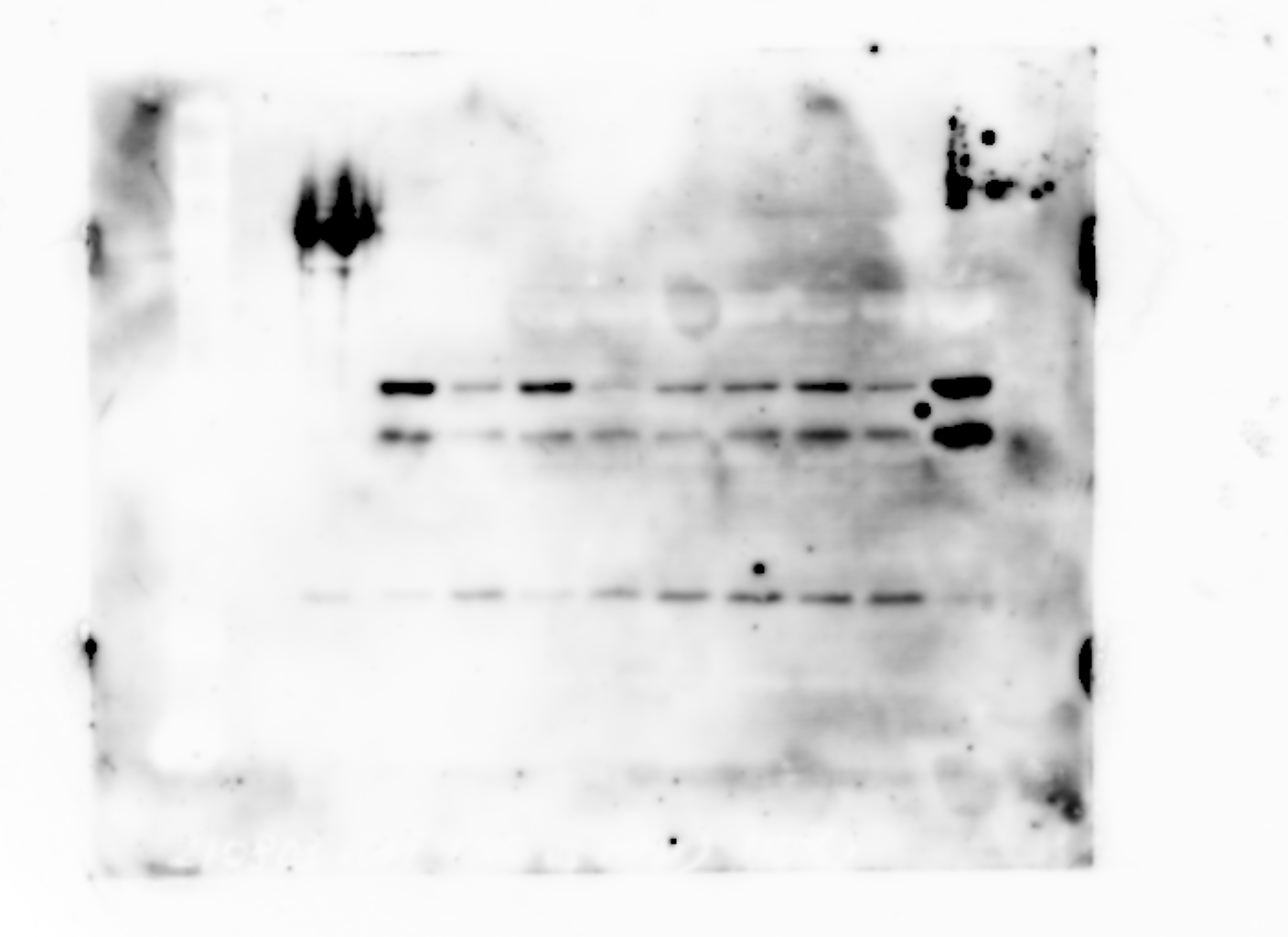

Supplement: Supplementary file 6 — Source data Fig. 4 [file 44318_2024_196_MOESM6_ESM.zip › Figure 4/Figure 4-H/Quantificated data/p-JNK1:2.tif]

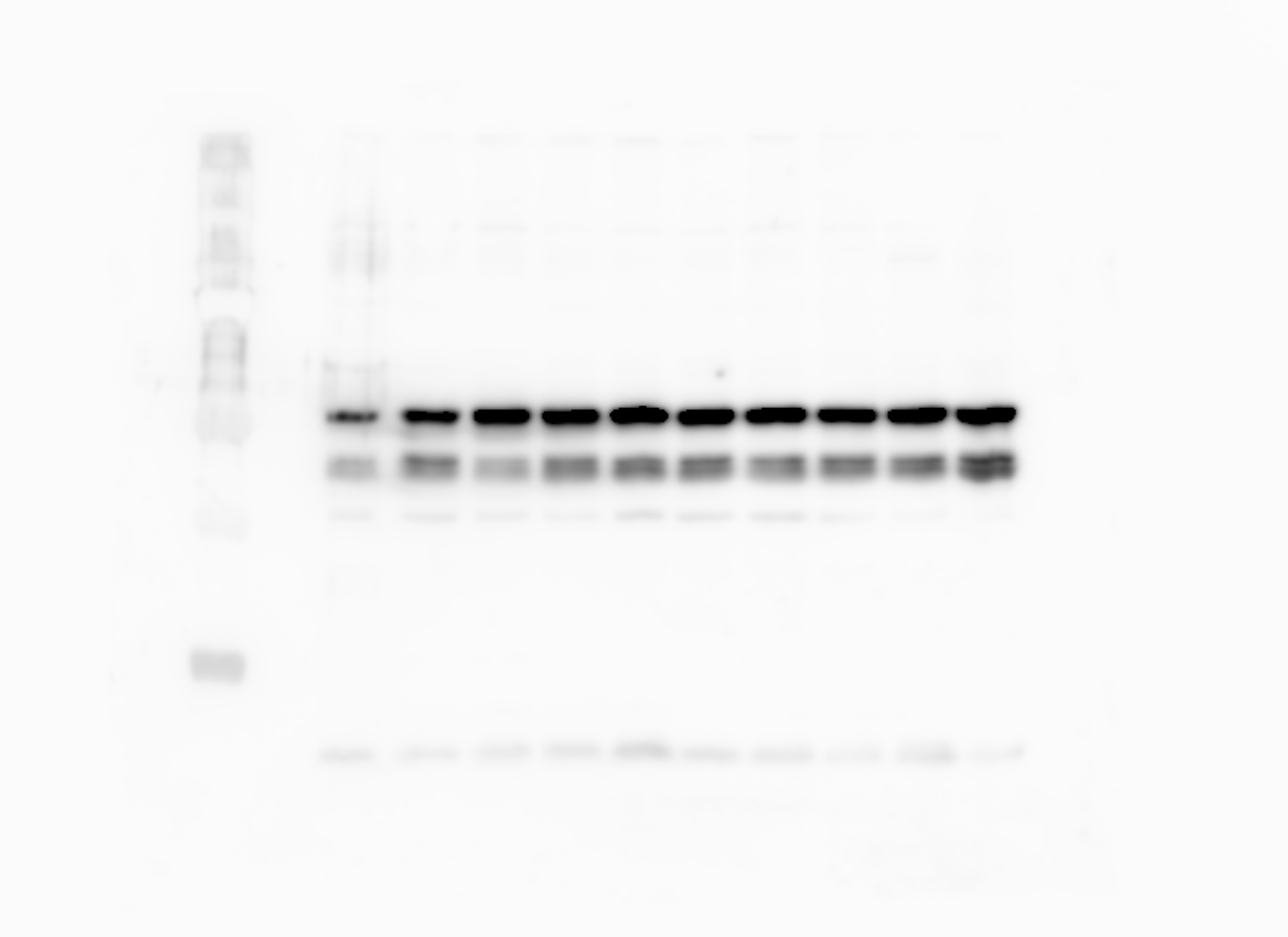

Supplement: Supplementary file 6 — Source data Fig. 4 [file 44318_2024_196_MOESM6_ESM.zip › Figure 4/Figure 4-H/Quantificated data/JNK1:2.tif]

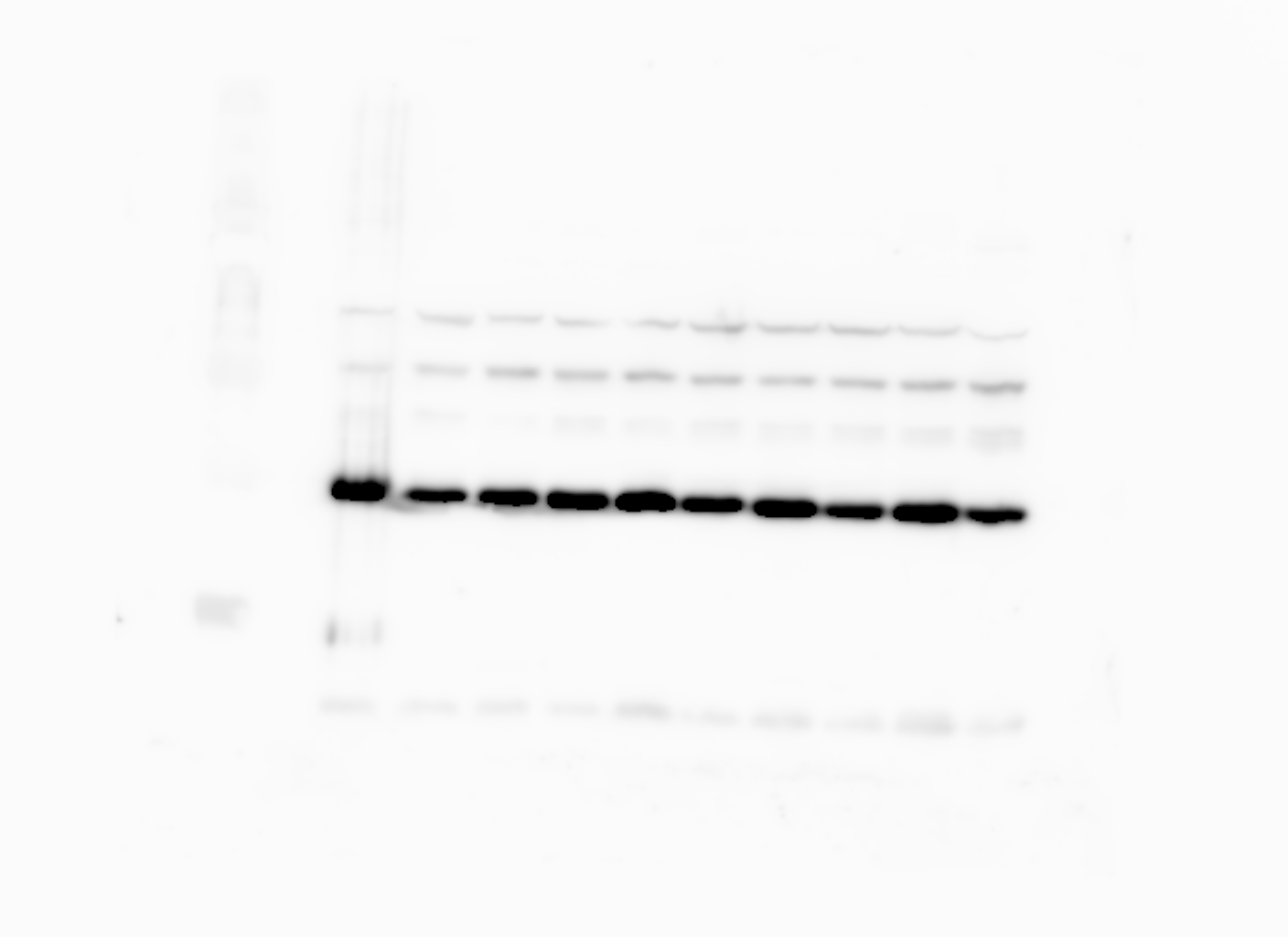

Supplement: Supplementary file 6 — Source data Fig. 4 [file 44318_2024_196_MOESM6_ESM.zip › Figure 4/Figure 4-H/Quantificated data/GAPDH.tif]

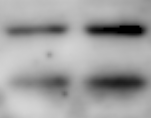

Supplement: Supplementary file 6 — Source data Fig. 4 [file 44318_2024_196_MOESM6_ESM.zip › Figure 4/Figure 4-H/Demonstrated data/p-JNK1:2.tif]

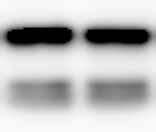

Supplement: Supplementary file 6 — Source data Fig. 4 [file 44318_2024_196_MOESM6_ESM.zip › Figure 4/Figure 4-H/Demonstrated data/JNK1:2.tif]

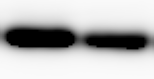

Supplement: Supplementary file 6 — Source data Fig. 4 [file 44318_2024_196_MOESM6_ESM.zip › Figure 4/Figure 4-H/Demonstrated data/GAPDH.tif]

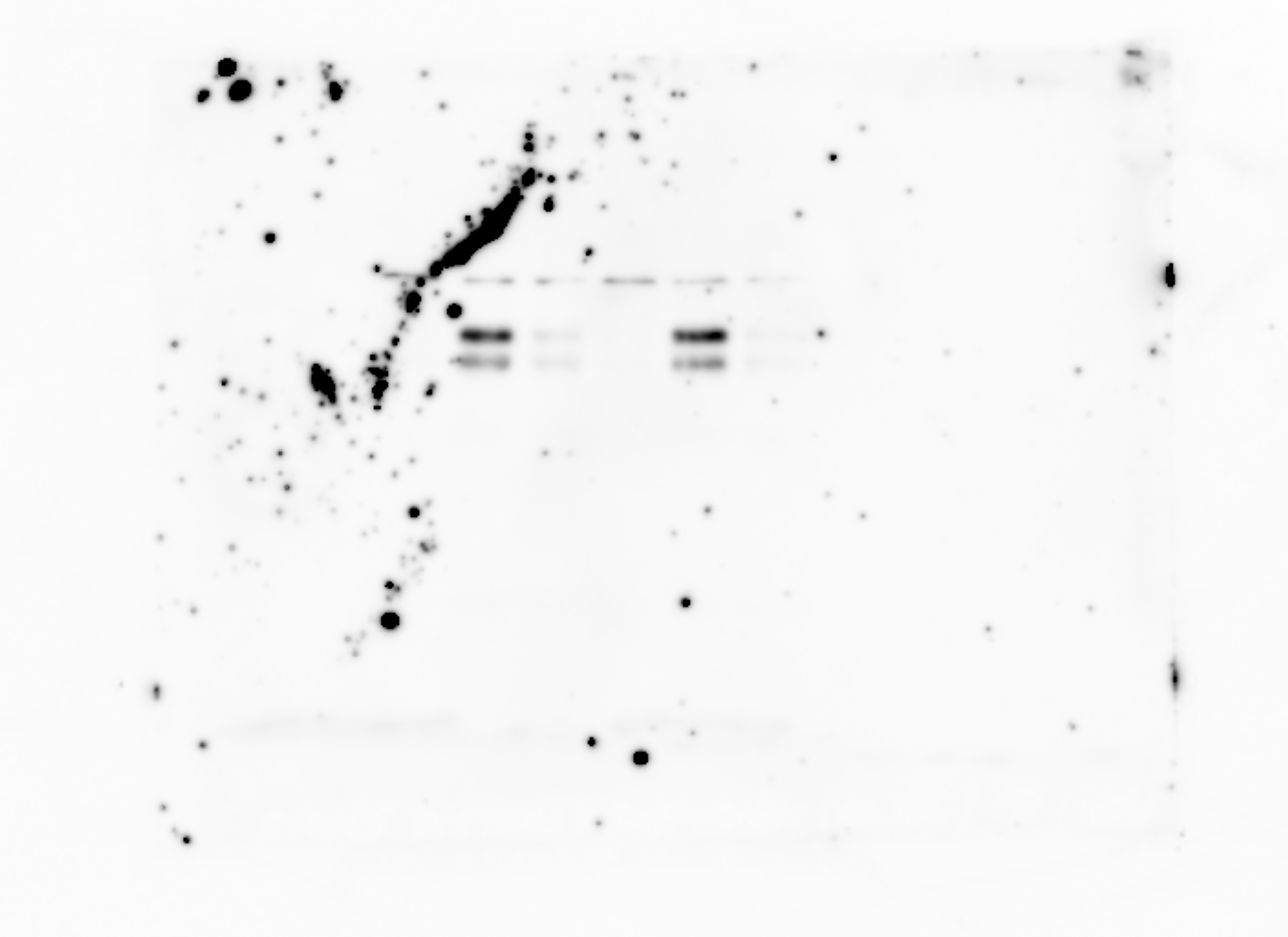

Supplement: Supplementary file 6 — Source data Fig. 4 [file 44318_2024_196_MOESM6_ESM.zip › Figure 4/Figure 4-I/Quantificated image/p-c-Fos membrane 2.tif]

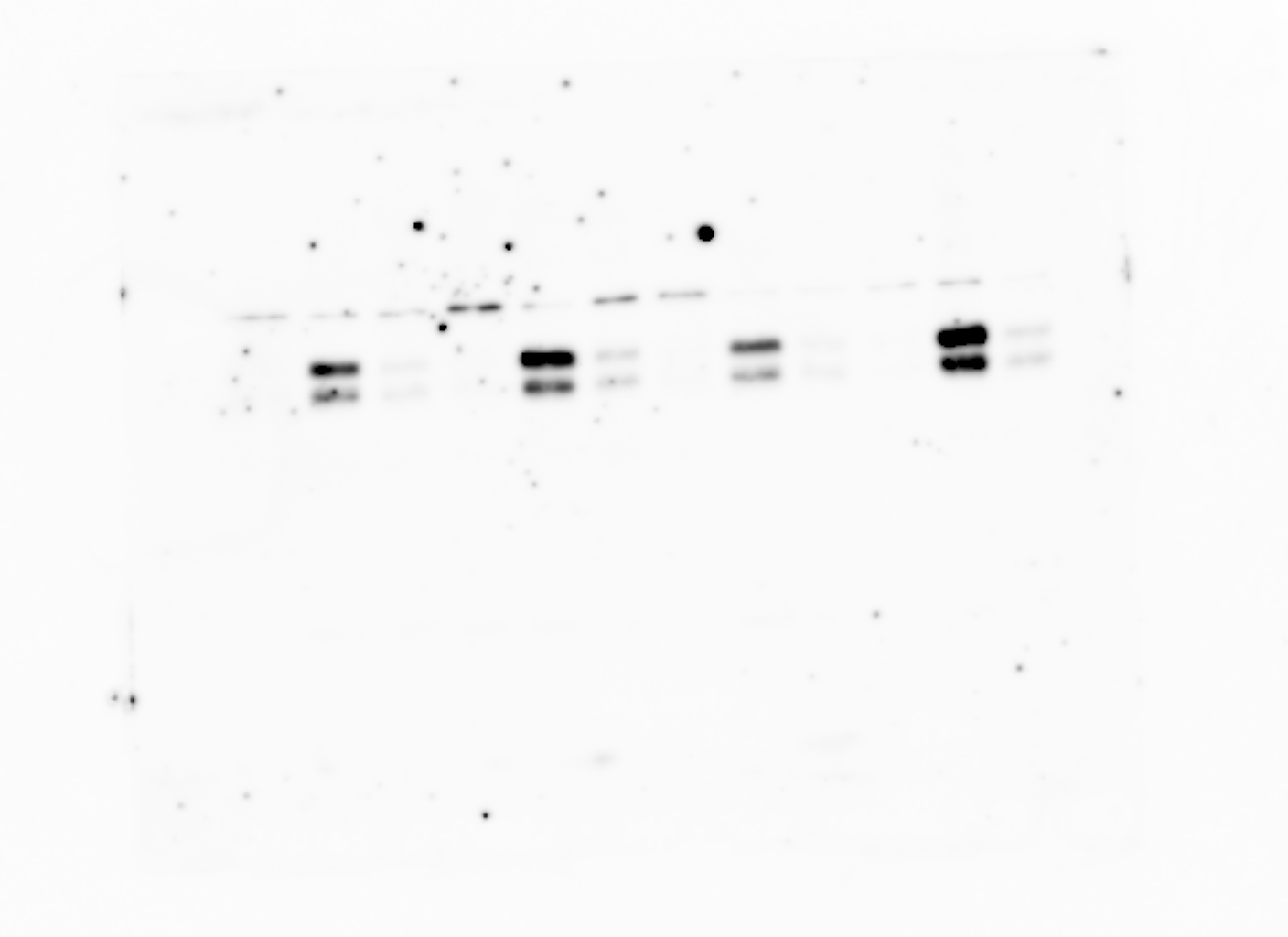

Supplement: Supplementary file 6 — Source data Fig. 4 [file 44318_2024_196_MOESM6_ESM.zip › Figure 4/Figure 4-I/Quantificated image/p-c-Fos membrane 1.tif]

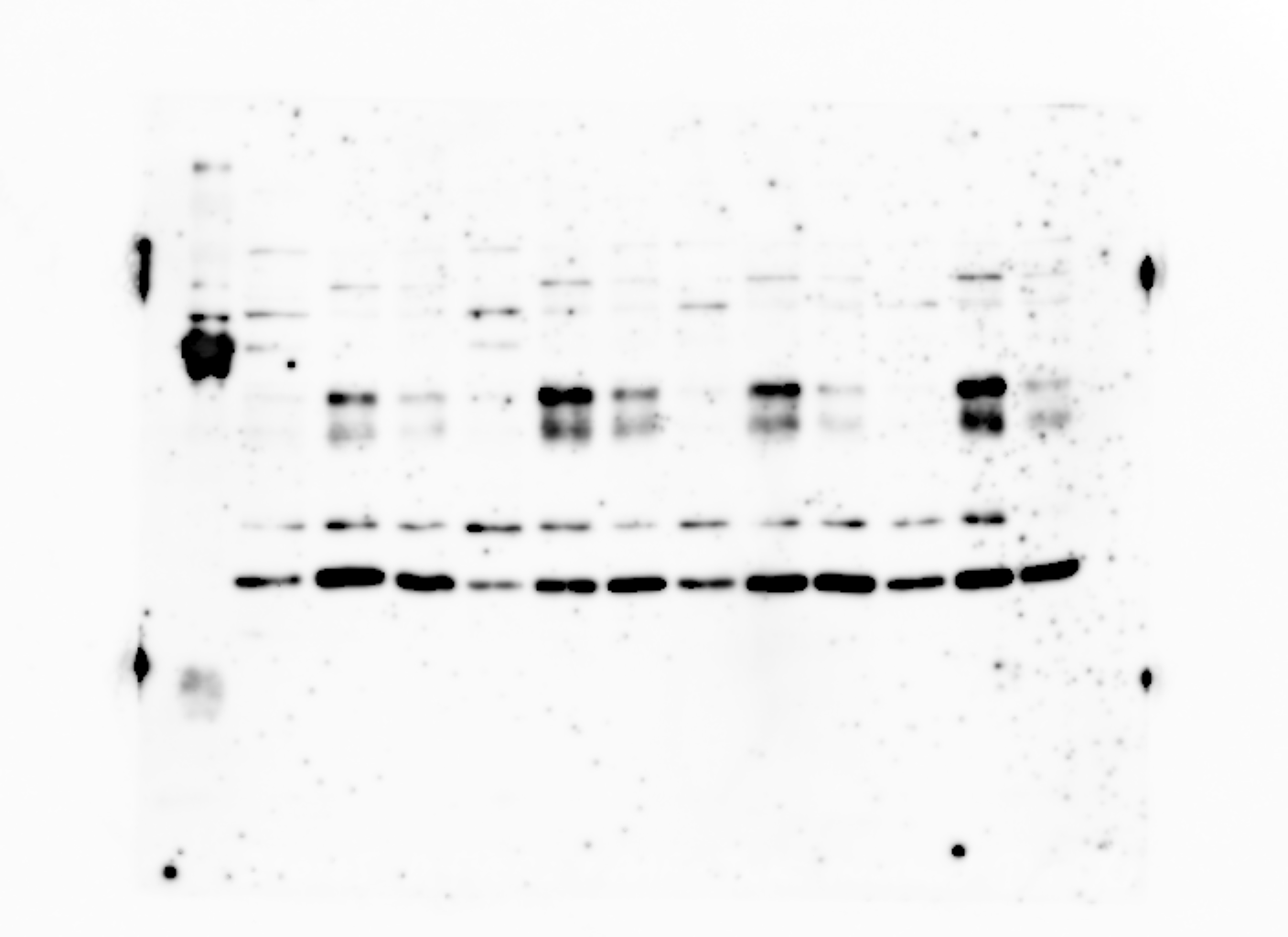

Supplement: Supplementary file 6 — Source data Fig. 4 [file 44318_2024_196_MOESM6_ESM.zip › Figure 4/Figure 4-I/Quantificated image/c-Fos membrane 1.tif]

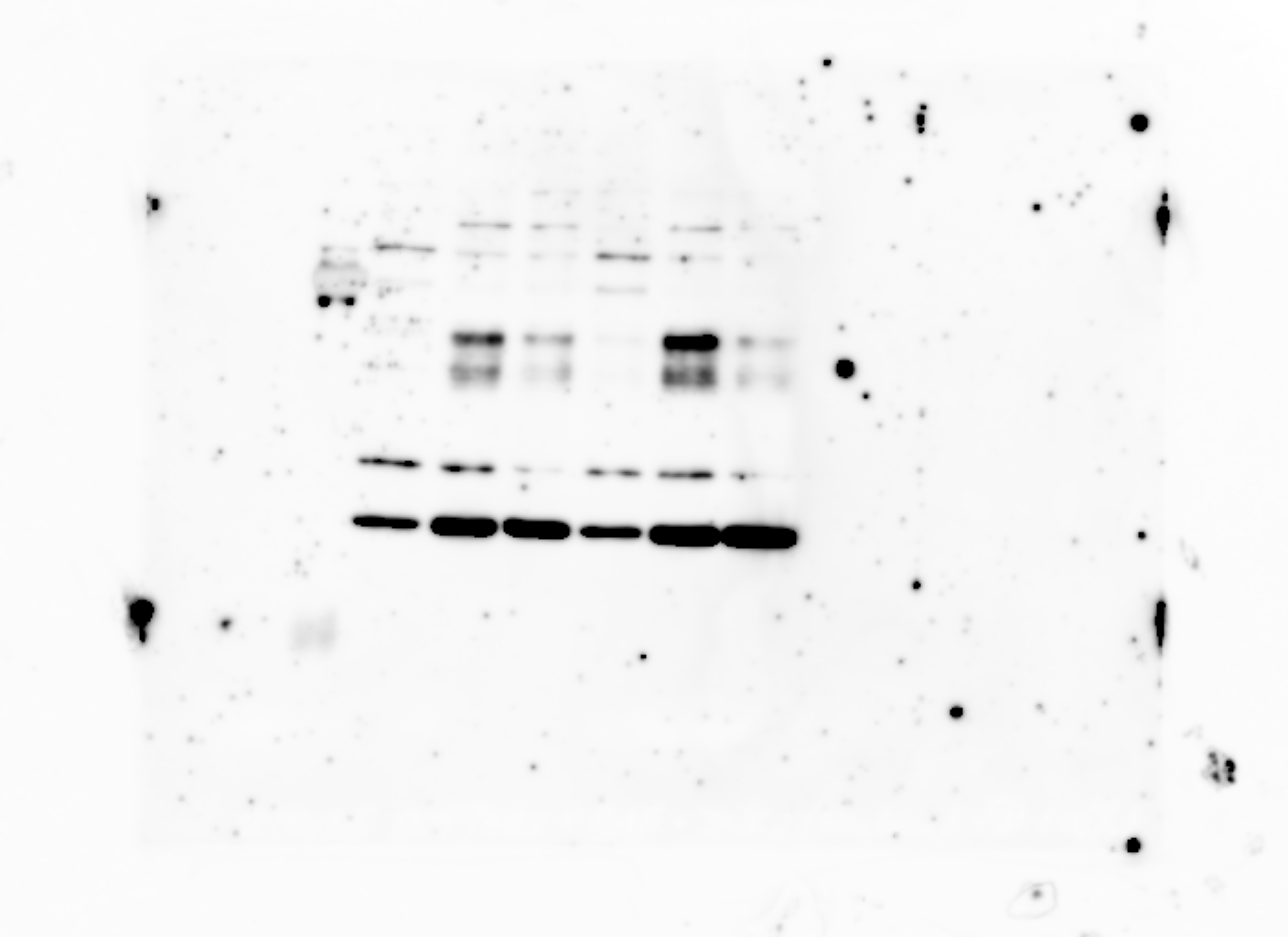

Supplement: Supplementary file 6 — Source data Fig. 4 [file 44318_2024_196_MOESM6_ESM.zip › Figure 4/Figure 4-I/Quantificated image/c-Fos membrane 2.tif]

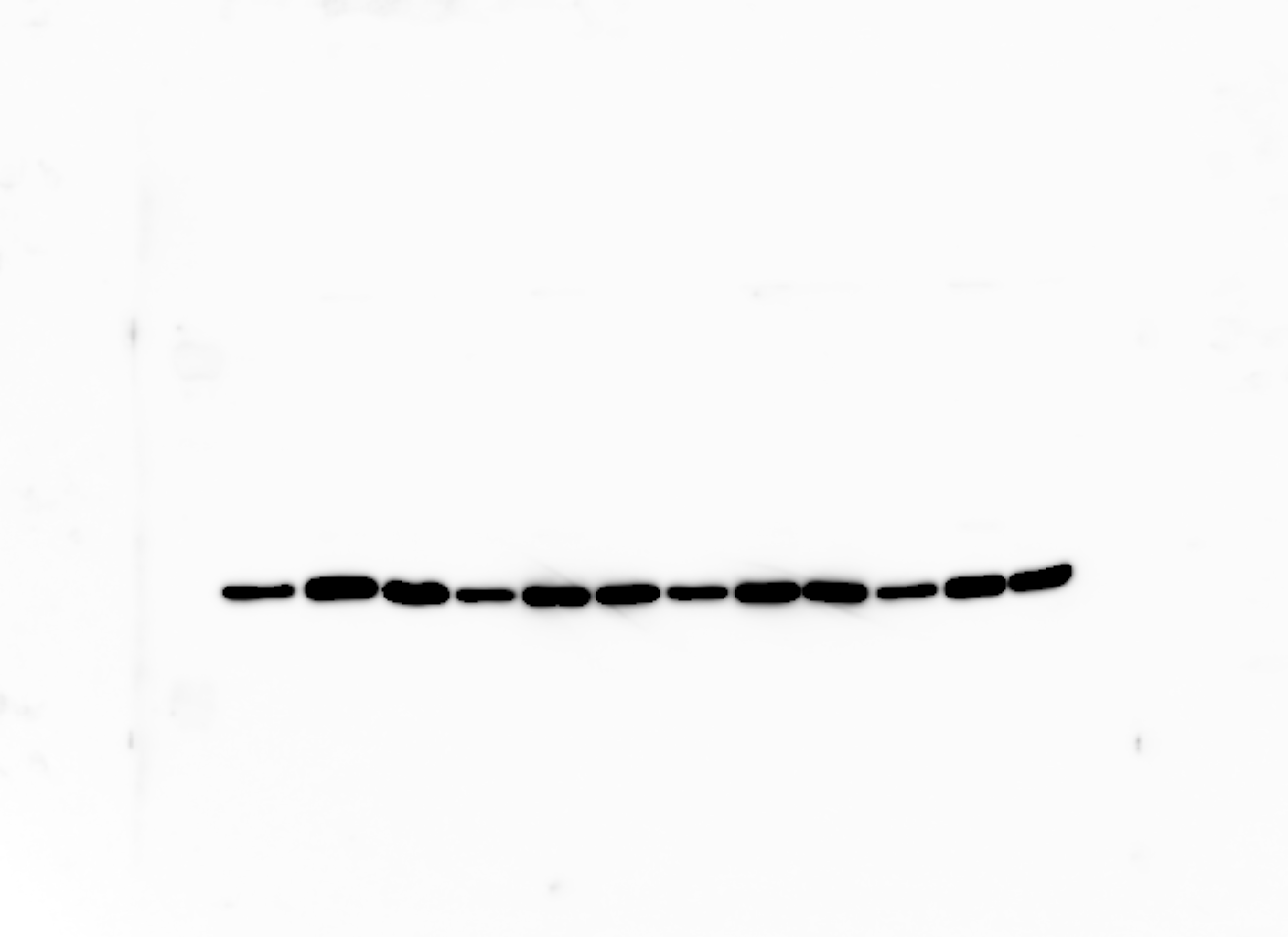

Supplement: Supplementary file 6 — Source data Fig. 4 [file 44318_2024_196_MOESM6_ESM.zip › Figure 4/Figure 4-I/Quantificated image/GAPDH membrane 1.tif]

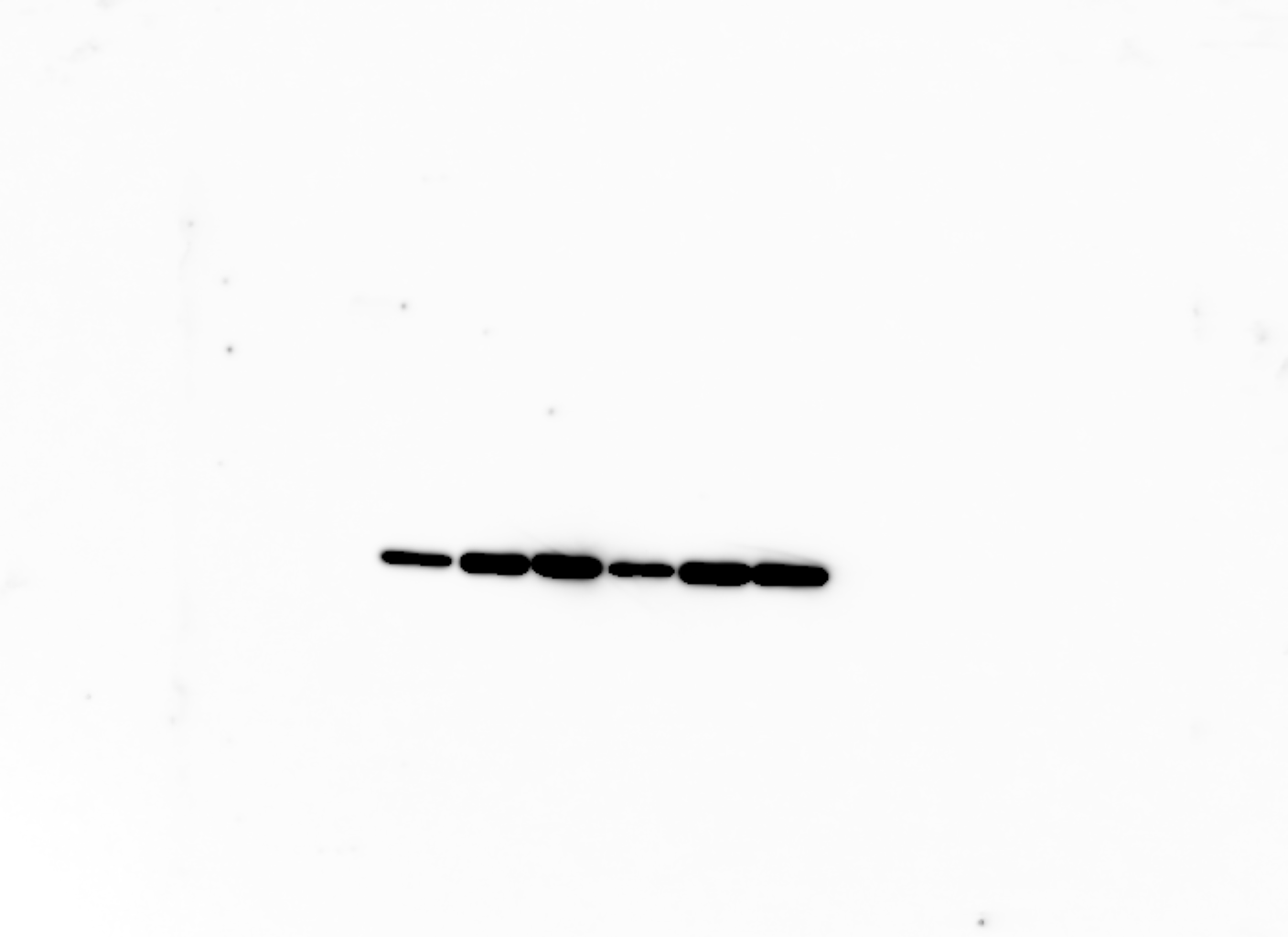

Supplement: Supplementary file 6 — Source data Fig. 4 [file 44318_2024_196_MOESM6_ESM.zip › Figure 4/Figure 4-I/Quantificated image/GAPDH membrane 2.tif]

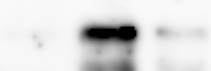

Supplement: Supplementary file 6 — Source data Fig. 4 [file 44318_2024_196_MOESM6_ESM.zip › Figure 4/Figure 4-I/Demonstrated image/c-Fos.tif]

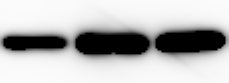

Supplement: Supplementary file 6 — Source data Fig. 4 [file 44318_2024_196_MOESM6_ESM.zip › Figure 4/Figure 4-I/Demonstrated image/GAPDH.tif]

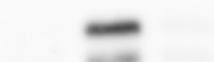

Supplement: Supplementary file 6 — Source data Fig. 4 [file 44318_2024_196_MOESM6_ESM.zip › Figure 4/Figure 4-I/Demonstrated image/p-c-Fos.tif]

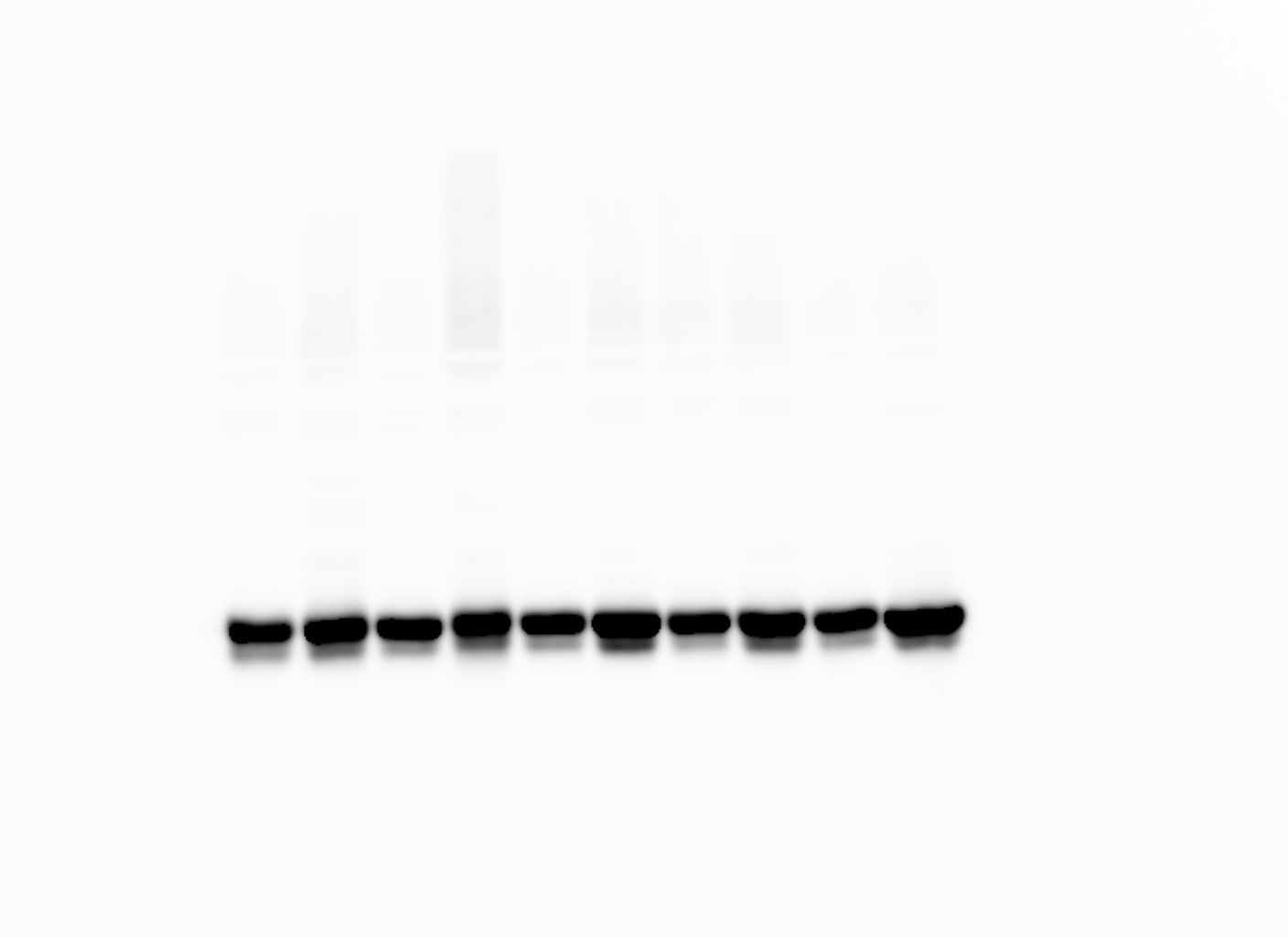

Supplement: Supplementary file 6 — Source data Fig. 4 [file 44318_2024_196_MOESM6_ESM.zip › Figure 4/Figure 4-G/Quantificated image/Tubulin.tif]

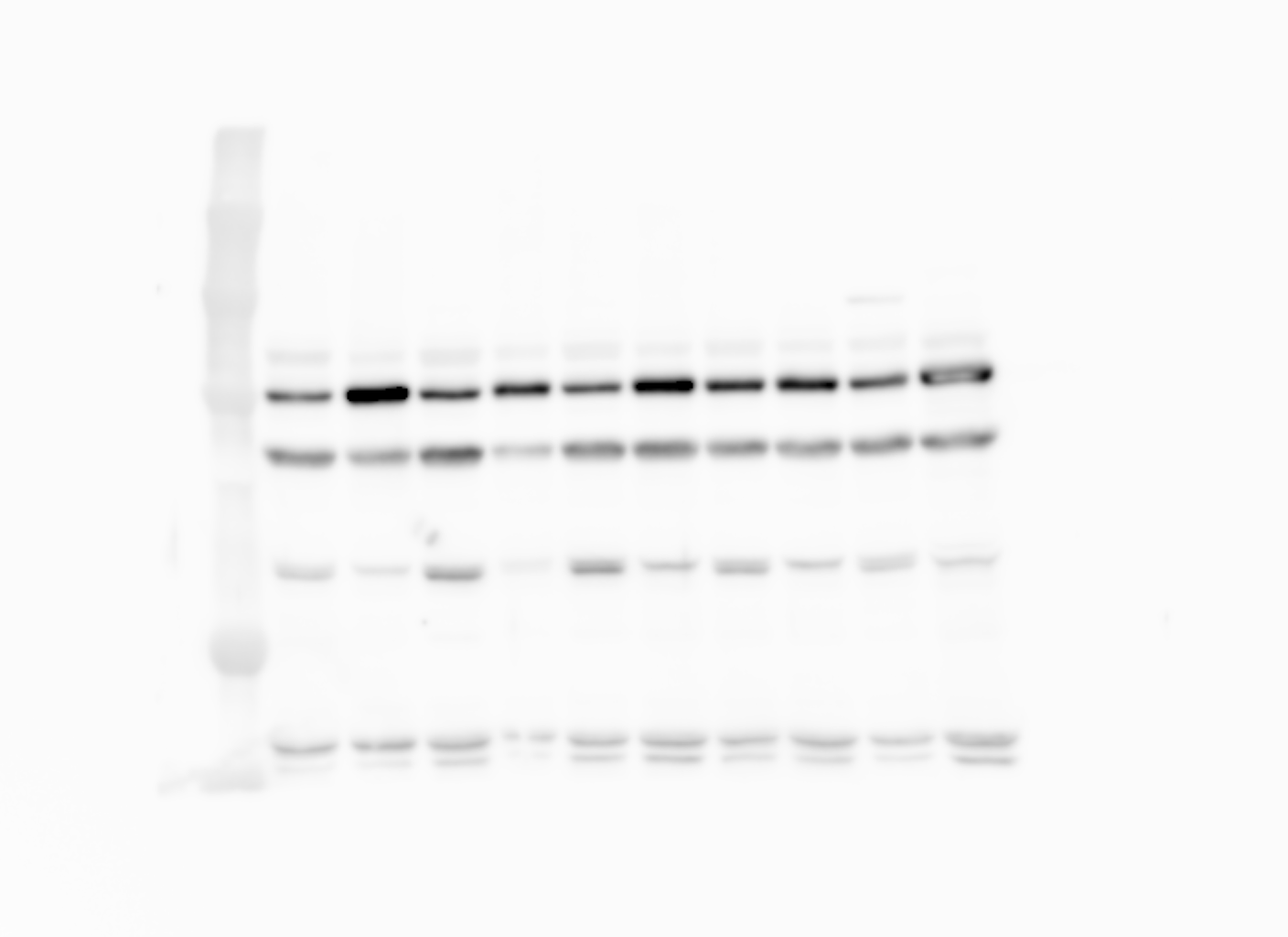

Supplement: Supplementary file 6 — Source data Fig. 4 [file 44318_2024_196_MOESM6_ESM.zip › Figure 4/Figure 4-G/Quantificated image/IRE1.tif]

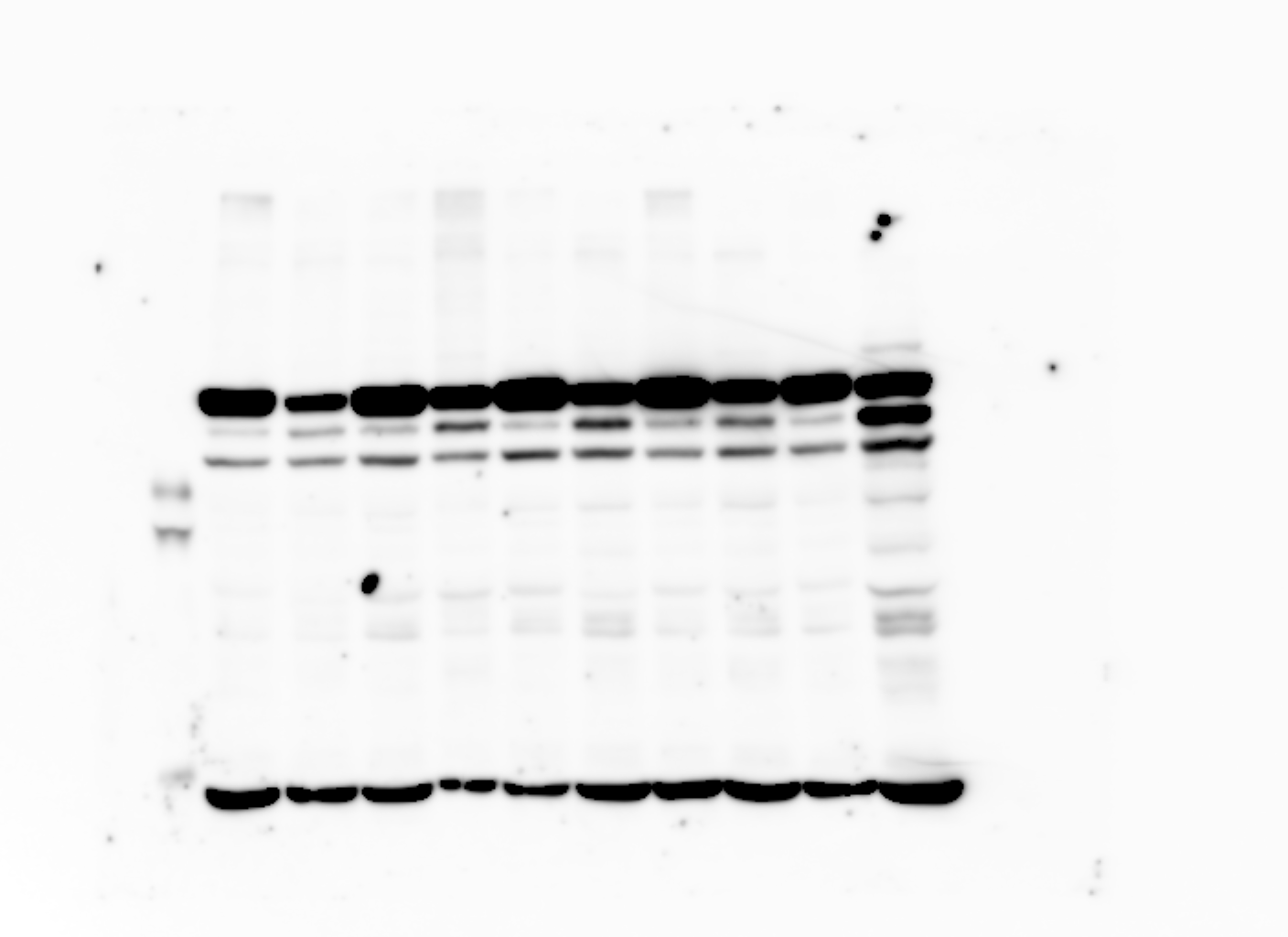

Supplement: Supplementary file 6 — Source data Fig. 4 [file 44318_2024_196_MOESM6_ESM.zip › Figure 4/Figure 4-G/Quantificated image/p-IRE1.tif]

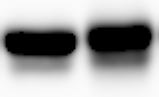

Supplement: Supplementary file 6 — Source data Fig. 4 [file 44318_2024_196_MOESM6_ESM.zip › Figure 4/Figure 4-G/Demonstrated image/Tubulin.tif]

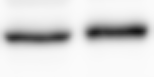

Supplement: Supplementary file 6 — Source data Fig. 4 [file 44318_2024_196_MOESM6_ESM.zip › Figure 4/Figure 4-G/Demonstrated image/IRE1.tif]

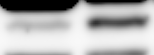

Supplement: Supplementary file 6 — Source data Fig. 4 [file 44318_2024_196_MOESM6_ESM.zip › Figure 4/Figure 4-G/Demonstrated image/p-IRE1.tif]

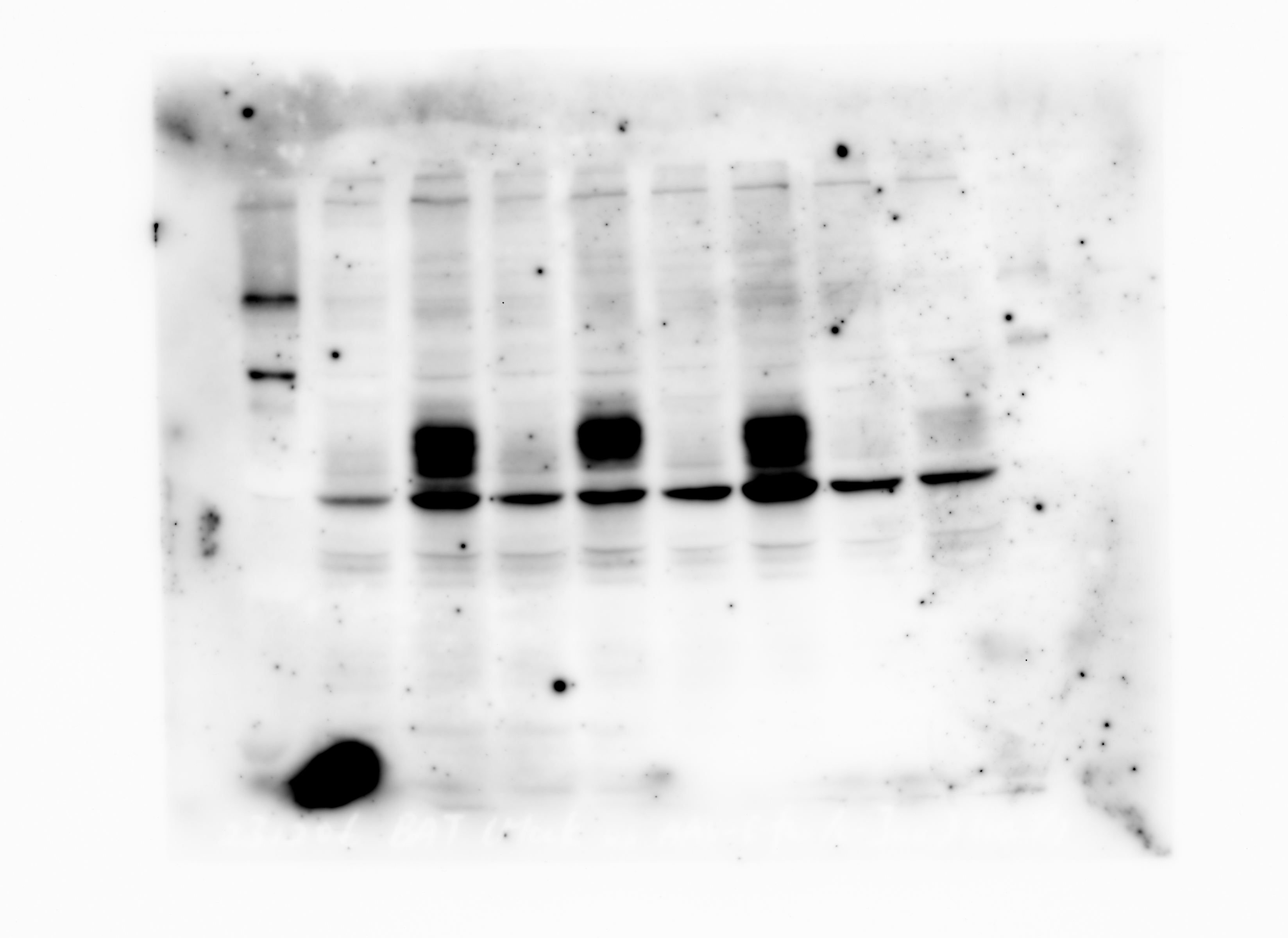

Supplement: Supplementary file 6 — Source data Fig. 4 [file 44318_2024_196_MOESM6_ESM.zip › Figure 4/Figure 4-B/Quantificated image/c-Fos.tif]

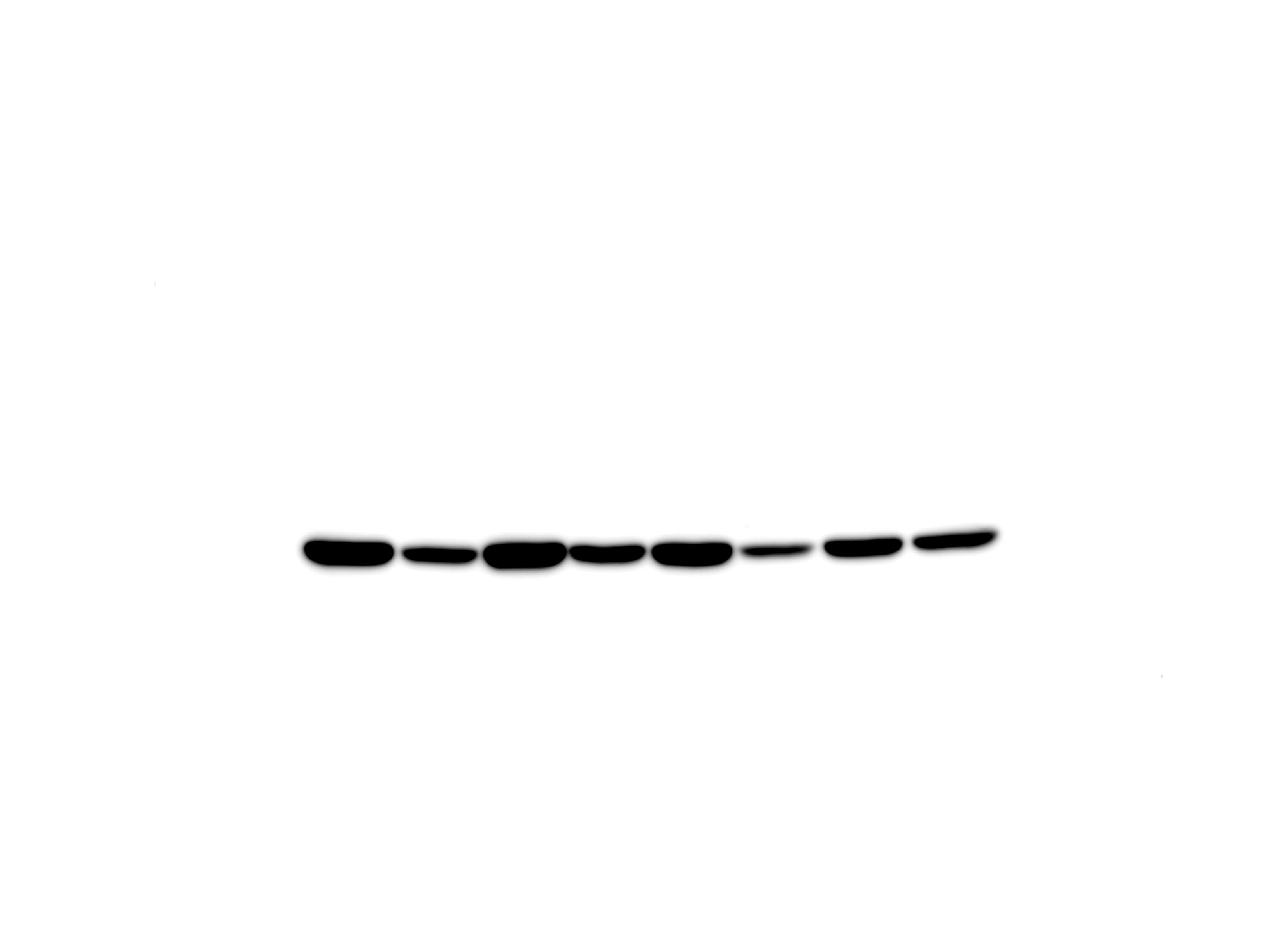

Supplement: Supplementary file 6 — Source data Fig. 4 [file 44318_2024_196_MOESM6_ESM.zip › Figure 4/Figure 4-B/Quantificated image/Actin.tif]

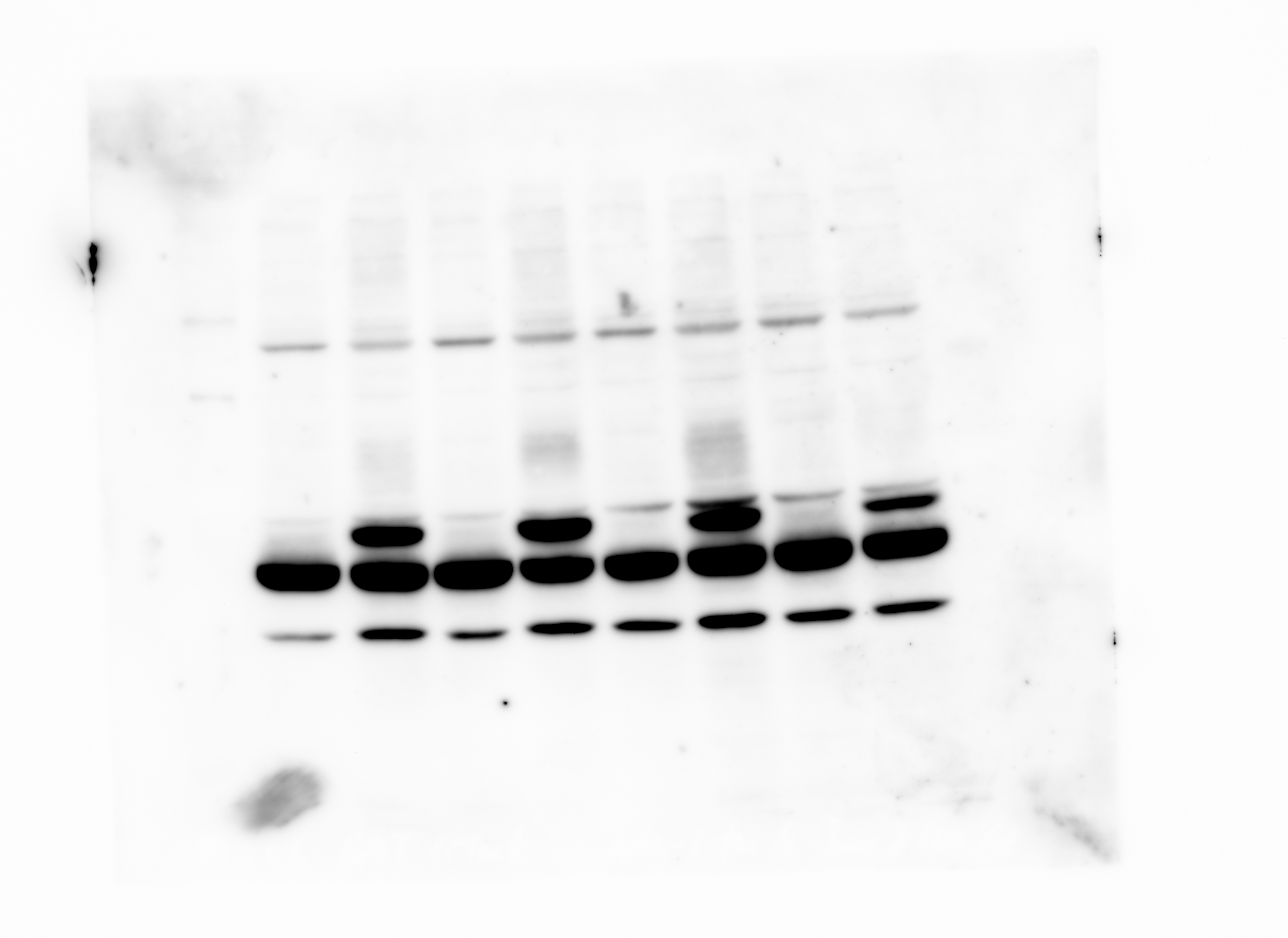

Supplement: Supplementary file 6 — Source data Fig. 4 [file 44318_2024_196_MOESM6_ESM.zip › Figure 4/Figure 4-B/Quantificated image/c-Jun.tif]

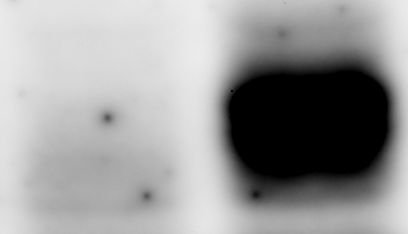

Supplement: Supplementary file 6 — Source data Fig. 4 [file 44318_2024_196_MOESM6_ESM.zip › Figure 4/Figure 4-B/Demonstrated image/c-Fos.tif]

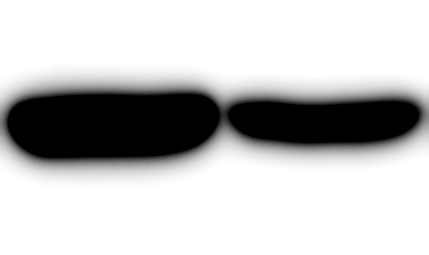

Supplement: Supplementary file 6 — Source data Fig. 4 [file 44318_2024_196_MOESM6_ESM.zip › Figure 4/Figure 4-B/Demonstrated image/Actin.tif]

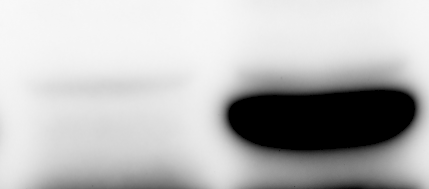

Supplement: Supplementary file 6 — Source data Fig. 4 [file 44318_2024_196_MOESM6_ESM.zip › Figure 4/Figure 4-B/Demonstrated image/c-Jun.tif]

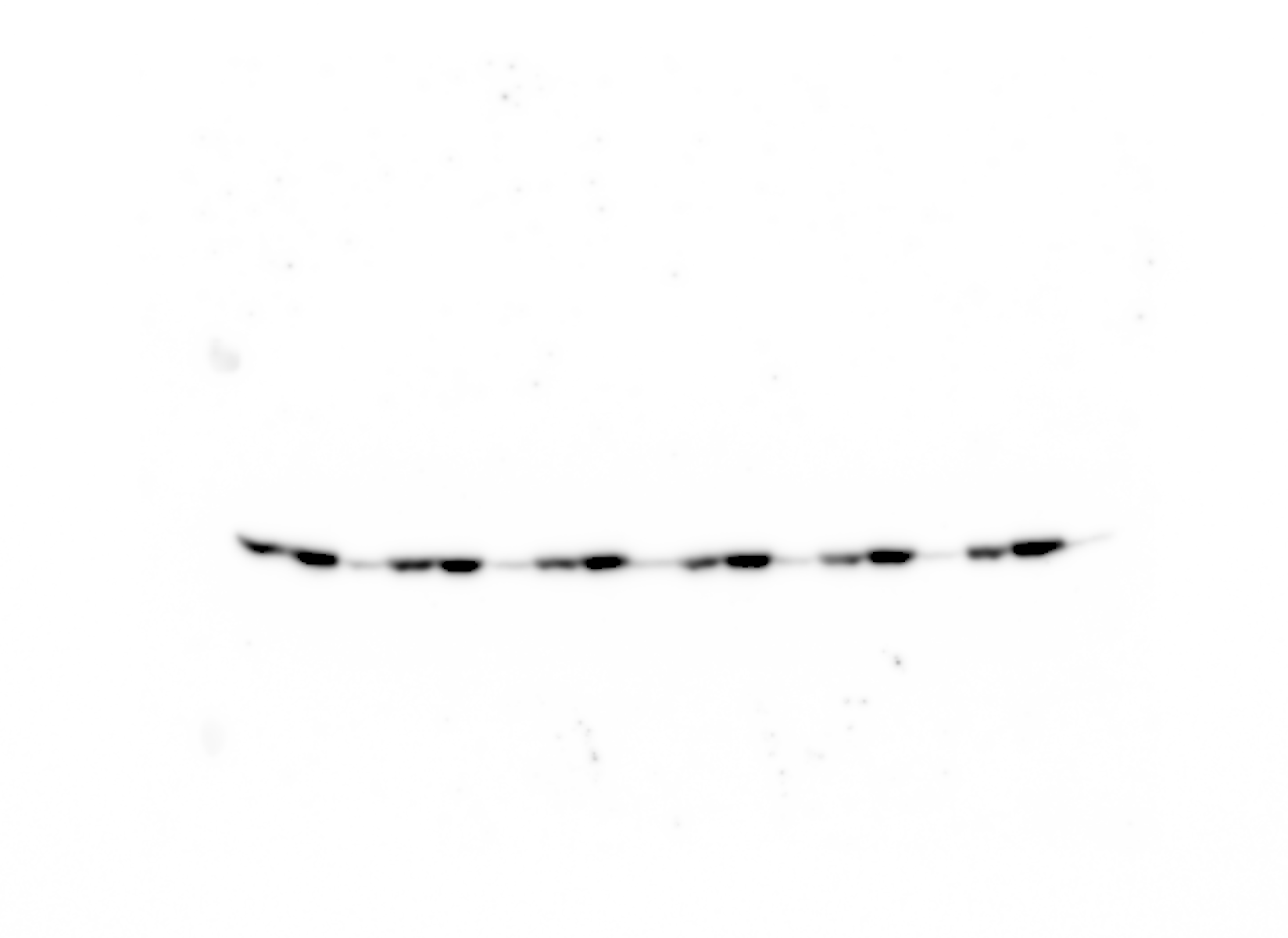

Supplement: Supplementary file 6 — Source data Fig. 4 [file 44318_2024_196_MOESM6_ESM.zip › Figure 4/Figure 4-J/Quantificated data/p-c-Jun.tif]

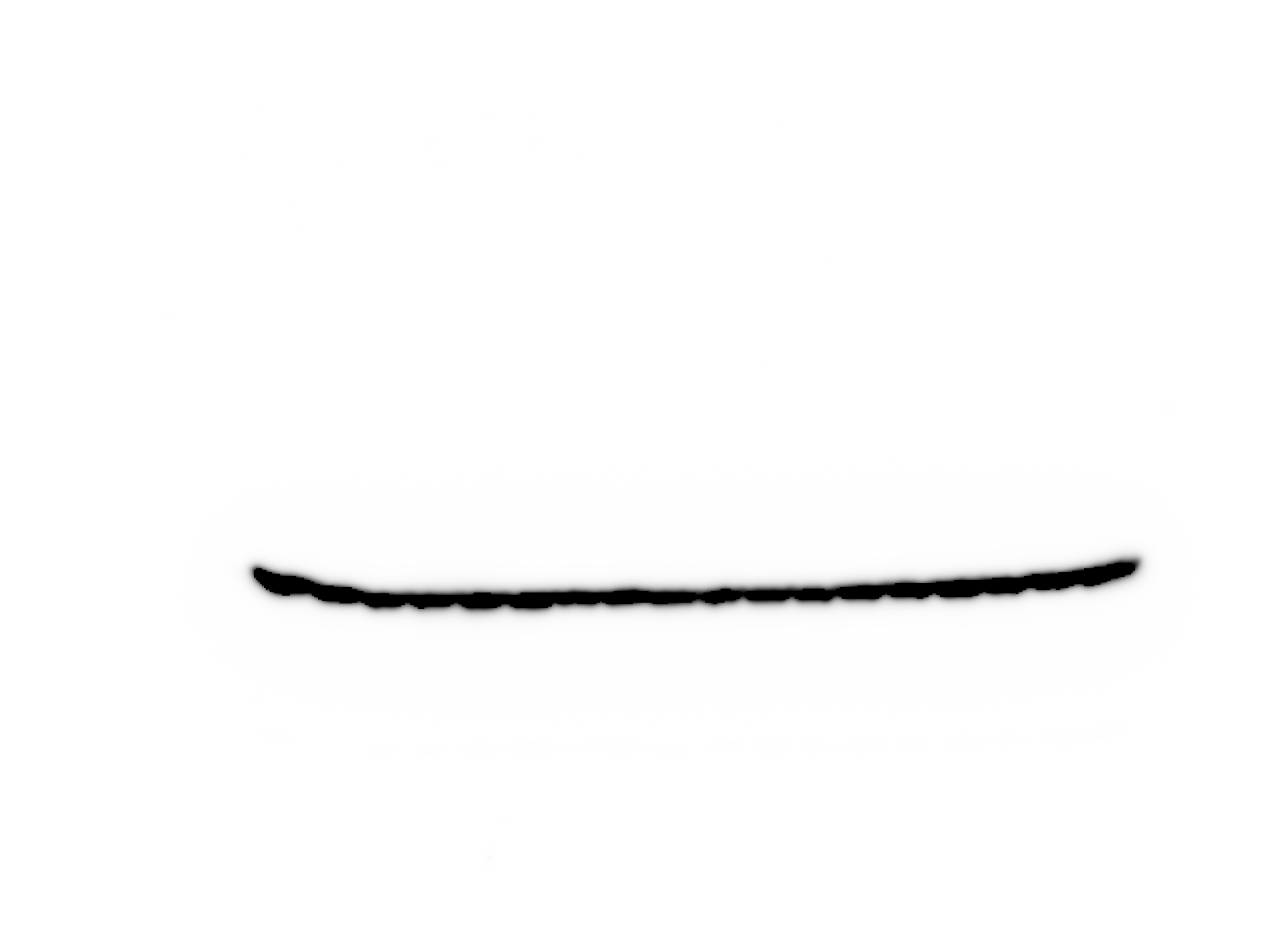

Supplement: Supplementary file 6 — Source data Fig. 4 [file 44318_2024_196_MOESM6_ESM.zip › Figure 4/Figure 4-J/Quantificated data/Actin.tif]

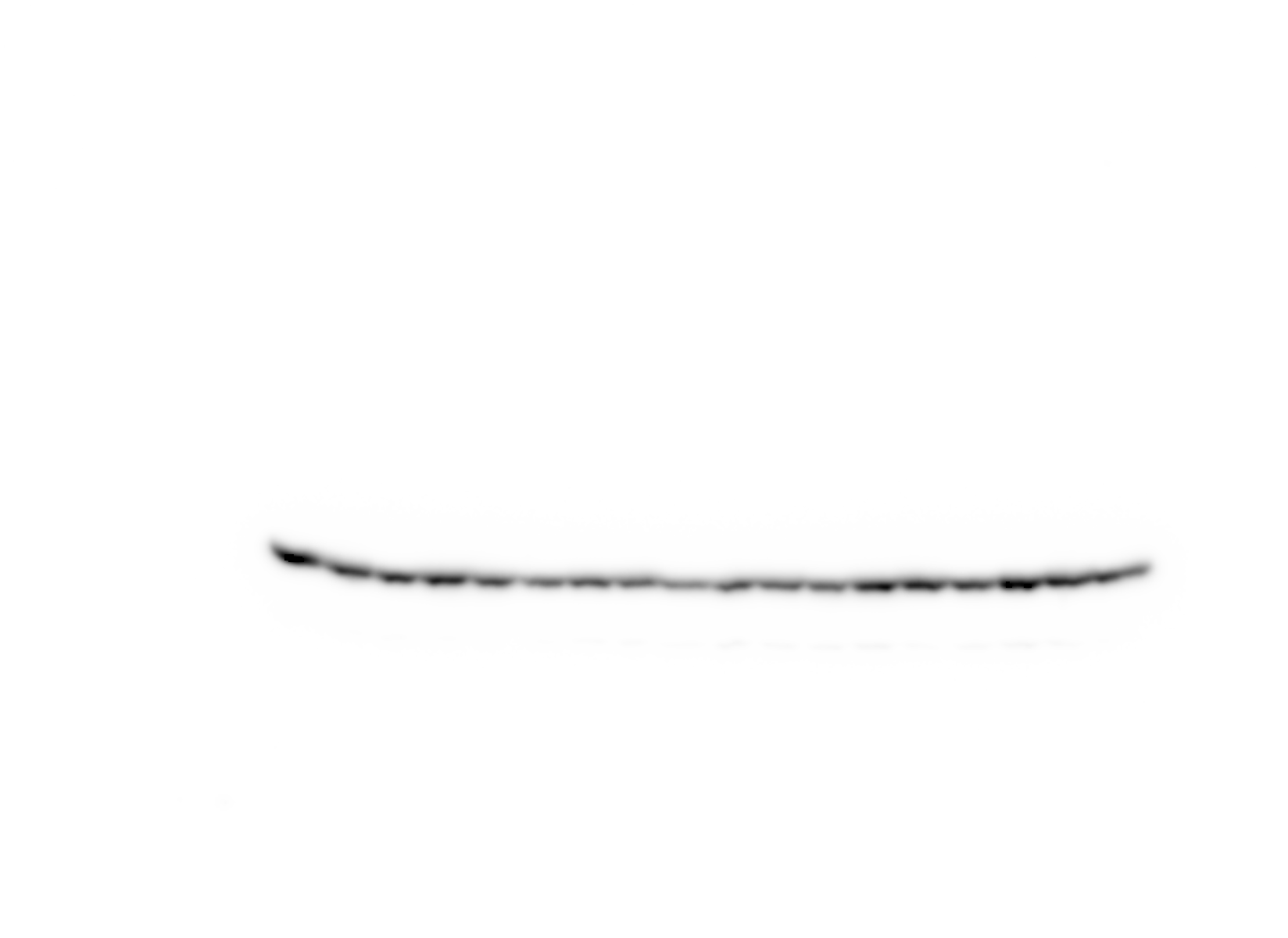

Supplement: Supplementary file 6 — Source data Fig. 4 [file 44318_2024_196_MOESM6_ESM.zip › Figure 4/Figure 4-J/Quantificated data/c-Jun.tif]

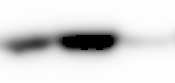

Supplement: Supplementary file 6 — Source data Fig. 4 [file 44318_2024_196_MOESM6_ESM.zip › Figure 4/Figure 4-J/Demonstrated data/p-c-Jun.tif]

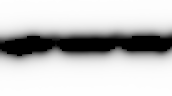

Supplement: Supplementary file 6 — Source data Fig. 4 [file 44318_2024_196_MOESM6_ESM.zip › Figure 4/Figure 4-J/Demonstrated data/Actin.tif]

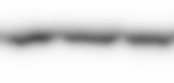

Supplement: Supplementary file 6 — Source data Fig. 4 [file 44318_2024_196_MOESM6_ESM.zip › Figure 4/Figure 4-J/Demonstrated data/c-Jun.tif]

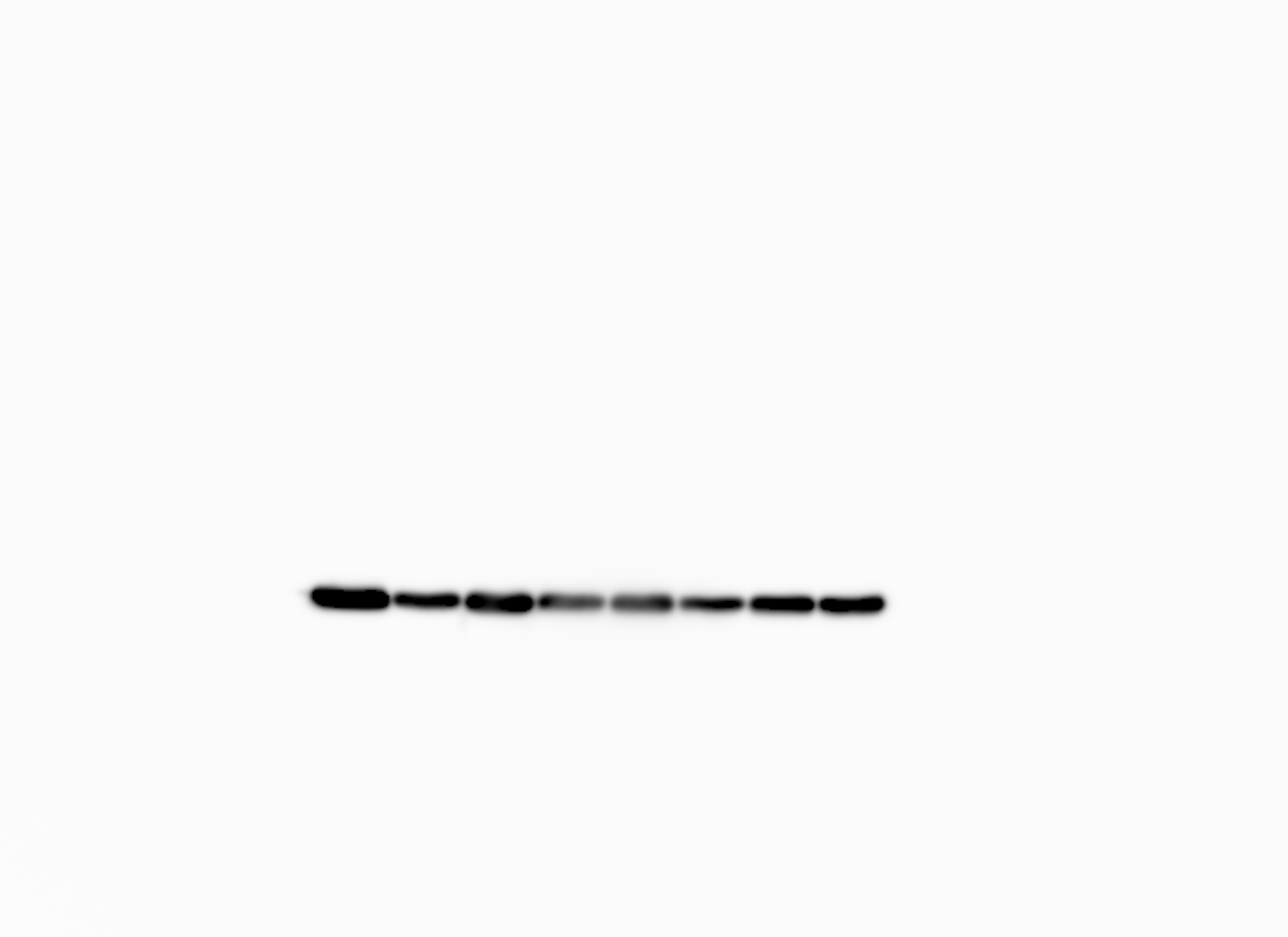

Supplement: Supplementary file 6 — Source data Fig. 4 [file 44318_2024_196_MOESM6_ESM.zip › Figure 4/Figure 4-D/Quantificated image/GAPDH.tif]

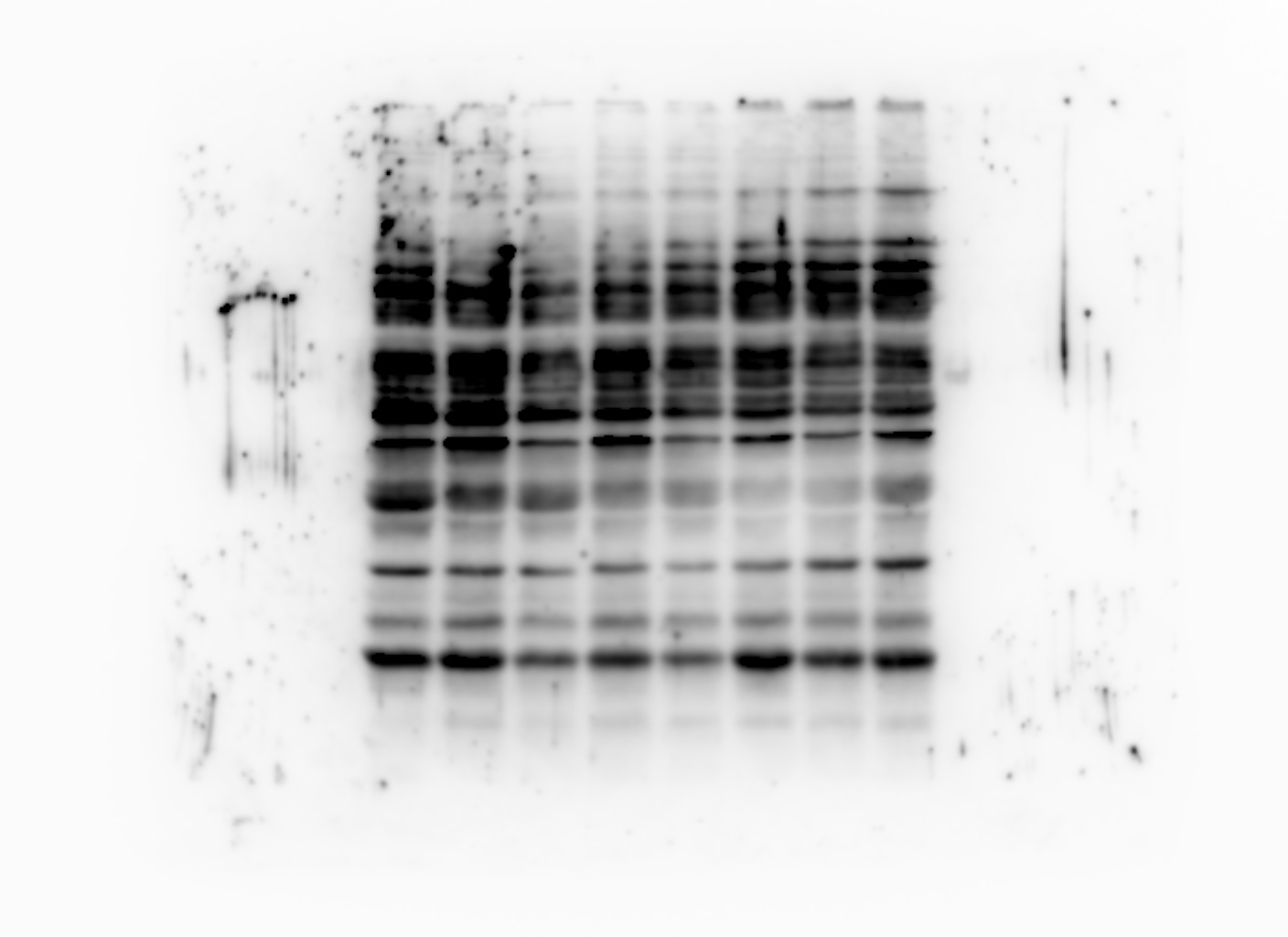

Supplement: Supplementary file 6 — Source data Fig. 4 [file 44318_2024_196_MOESM6_ESM.zip › Figure 4/Figure 4-D/Quantificated image/PCPE-1.tif]

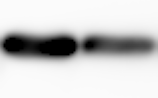

Supplement: Supplementary file 6 — Source data Fig. 4 [file 44318_2024_196_MOESM6_ESM.zip › Figure 4/Figure 4-D/Demonstrated image/GAPDH.tif]

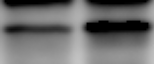

Supplement: Supplementary file 6 — Source data Fig. 4 [file 44318_2024_196_MOESM6_ESM.zip › Figure 4/Figure 4-D/Demonstrated image/PCPE-1.tif]

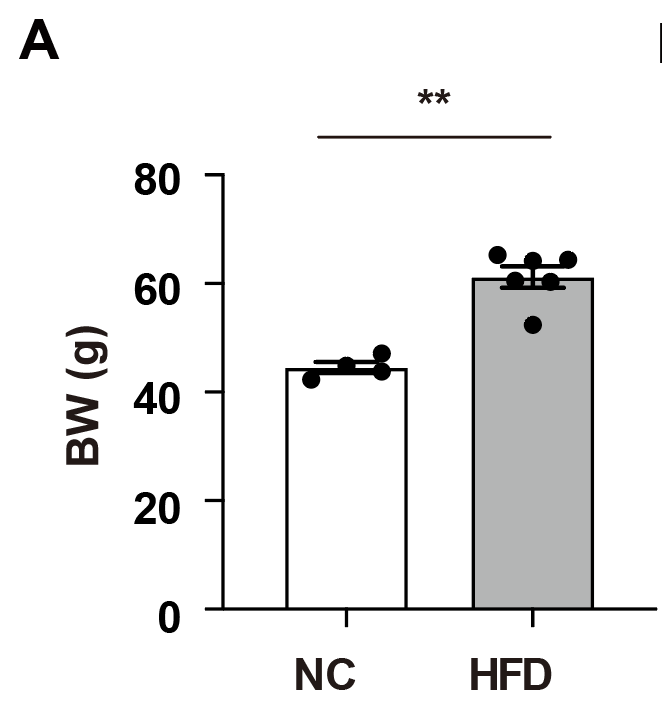

Supplement: Supplementary file 7 — Figure EV1 Source Data [file 44318_2024_196_MOESM7_ESM.zip › Figure EV1/Figure EV1-A/Fig EV1A.png]

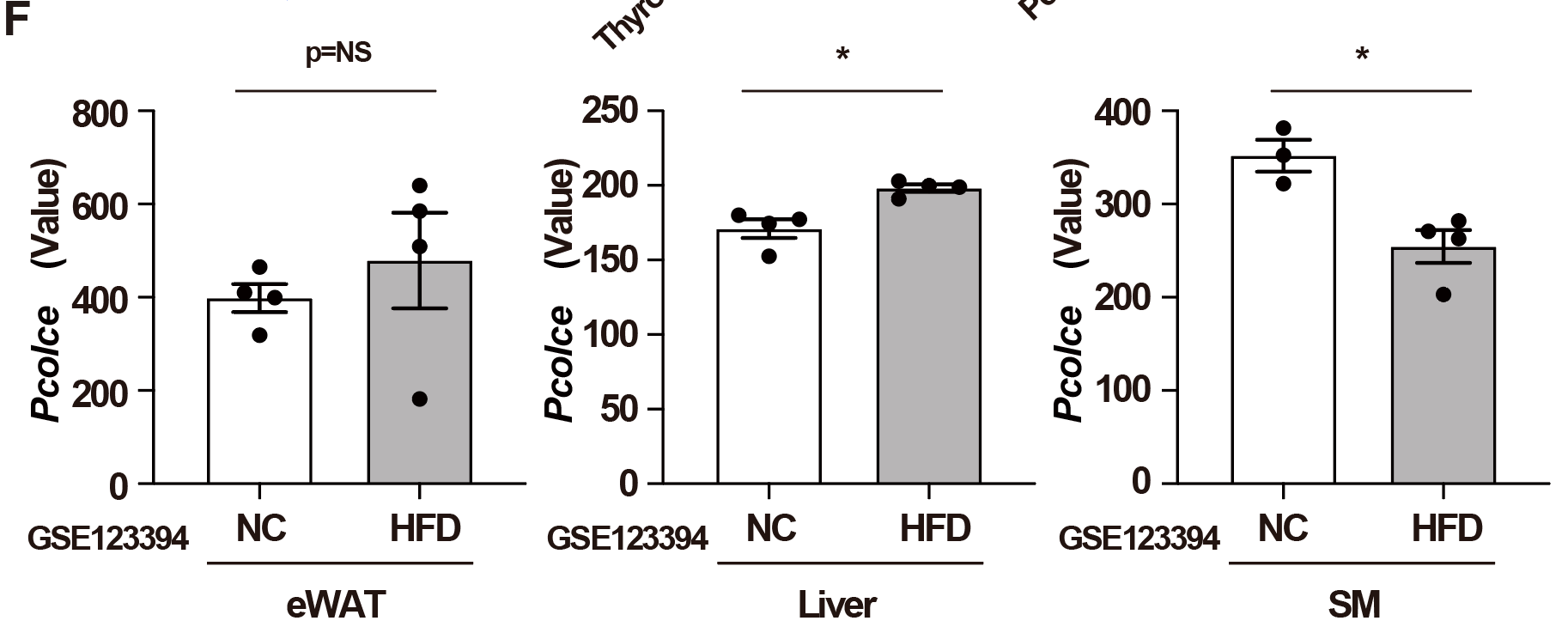

Supplement: Supplementary file 7 — Figure EV1 Source Data [file 44318_2024_196_MOESM7_ESM.zip › Figure EV1/Figure EV1-F/Fig. EV1F.png]

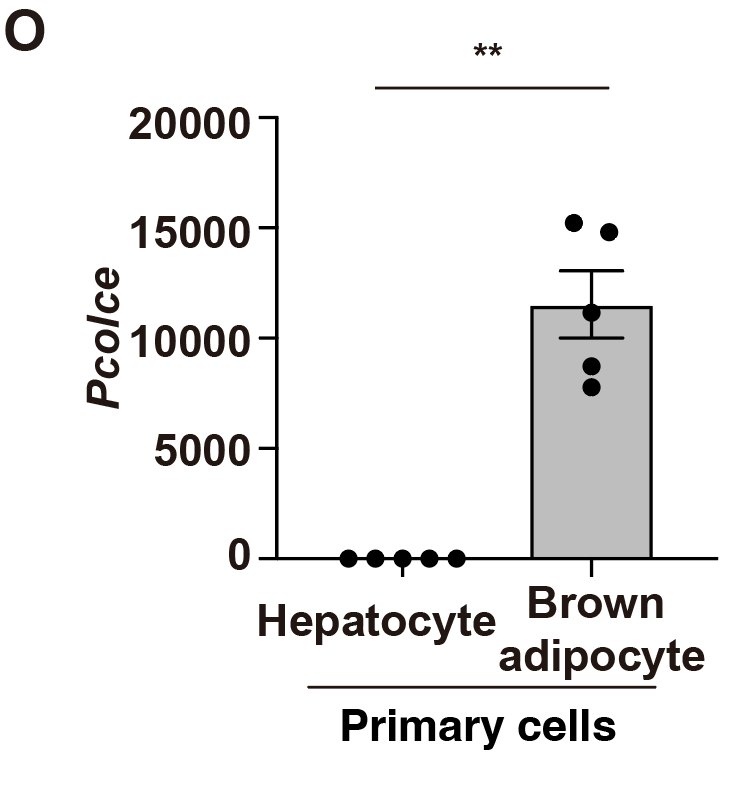

Supplement: Supplementary file 7 — Figure EV1 Source Data [file 44318_2024_196_MOESM7_ESM.zip › Figure EV1/Figure EV1-O/Fig.EV1O.png]

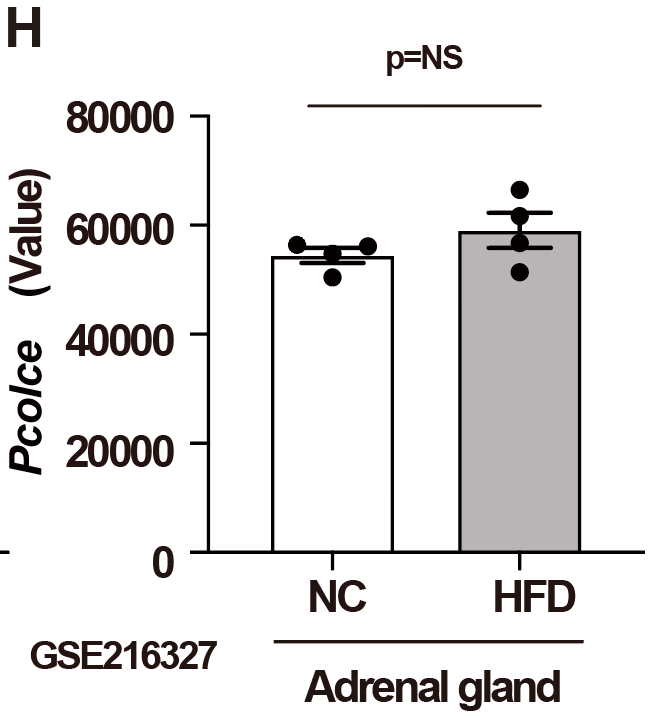

Supplement: Supplementary file 7 — Figure EV1 Source Data [file 44318_2024_196_MOESM7_ESM.zip › Figure EV1/Figure EV1-H/Fig.EV1H.png]

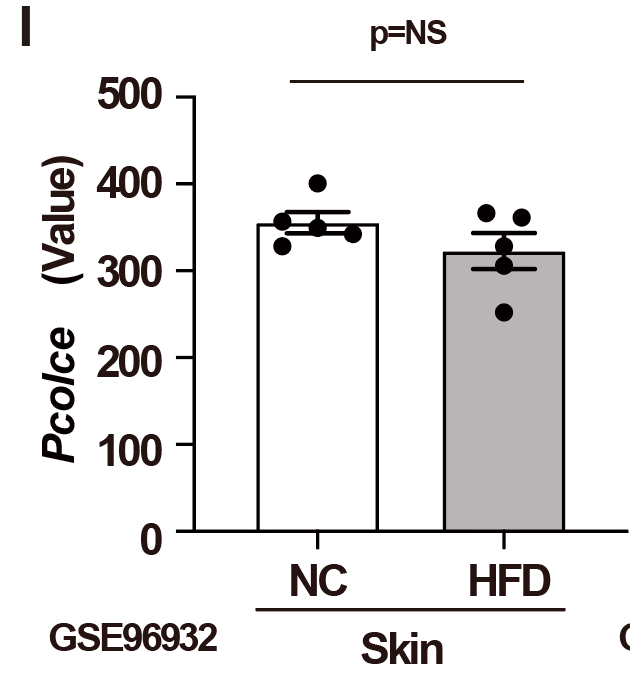

Supplement: Supplementary file 7 — Figure EV1 Source Data [file 44318_2024_196_MOESM7_ESM.zip › Figure EV1/Figure EV1-I/Fig.EV1I.png]

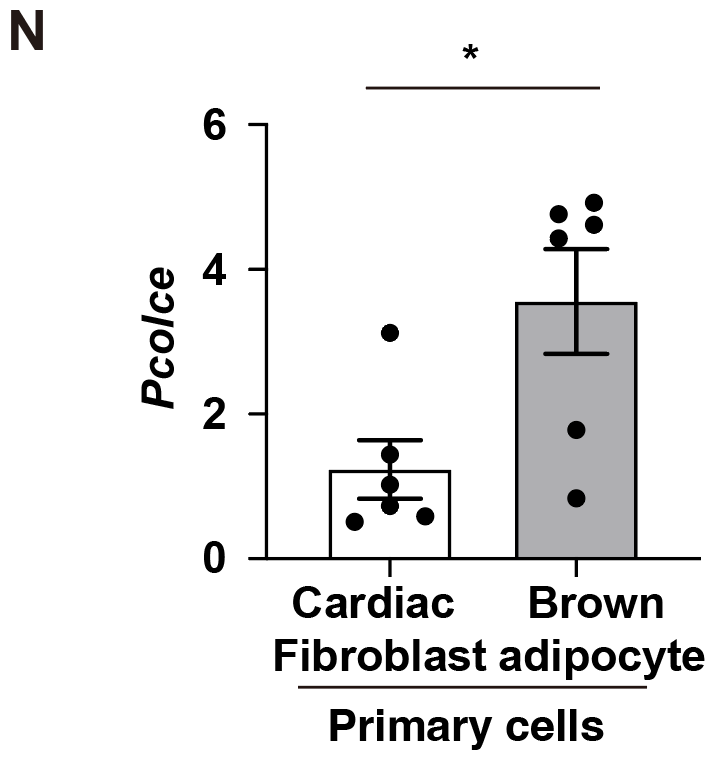

Supplement: Supplementary file 7 — Figure EV1 Source Data [file 44318_2024_196_MOESM7_ESM.zip › Figure EV1/Figure EV1-N/Fig.EV1N.png]

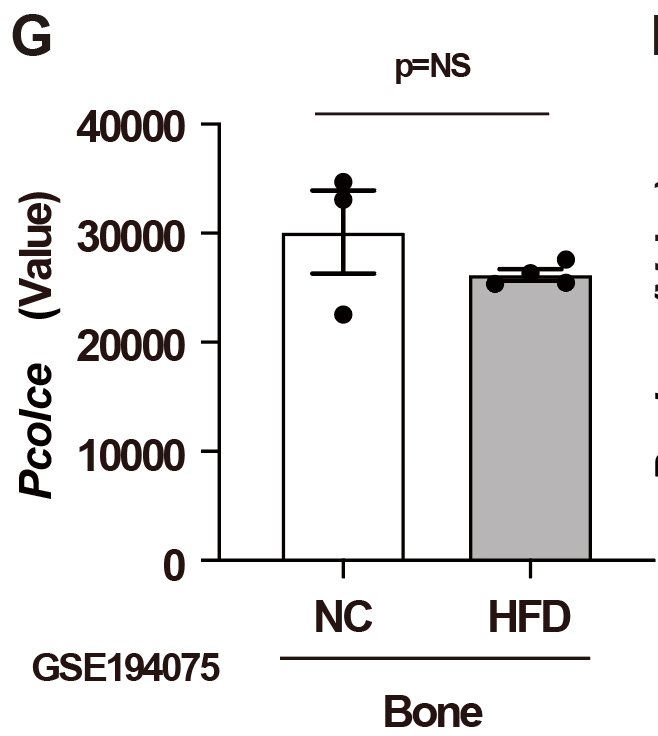

Supplement: Supplementary file 7 — Figure EV1 Source Data [file 44318_2024_196_MOESM7_ESM.zip › Figure EV1/Figure EV1-G/Fig.EV1G.png]

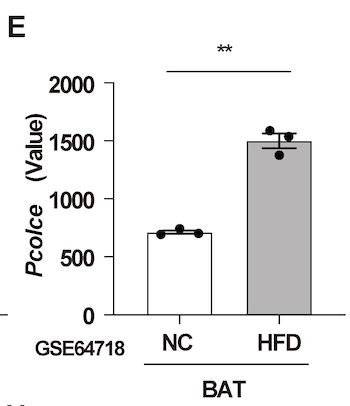

Supplement: Supplementary file 7 — Figure EV1 Source Data [file 44318_2024_196_MOESM7_ESM.zip › Figure EV1/Figure EV1-E/Fig.EV1E.png]

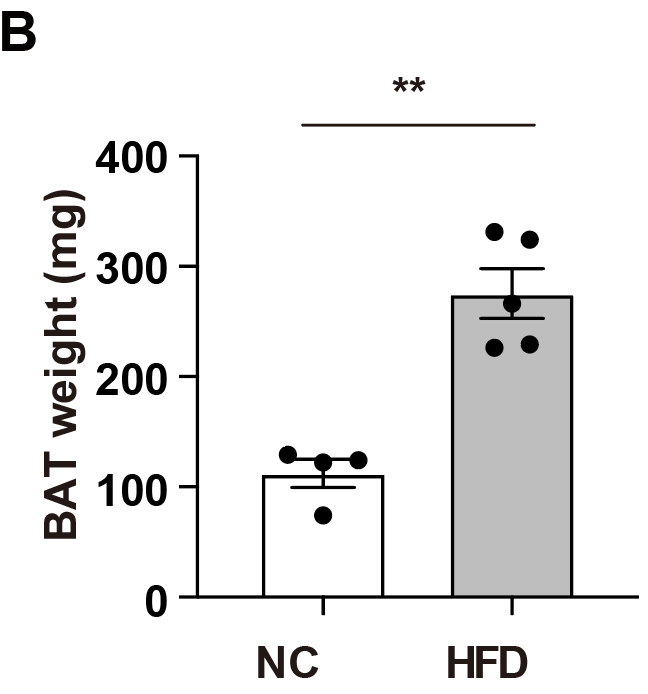

Supplement: Supplementary file 7 — Figure EV1 Source Data [file 44318_2024_196_MOESM7_ESM.zip › Figure EV1/Figure EV1-B/Fig. EV1B.png]

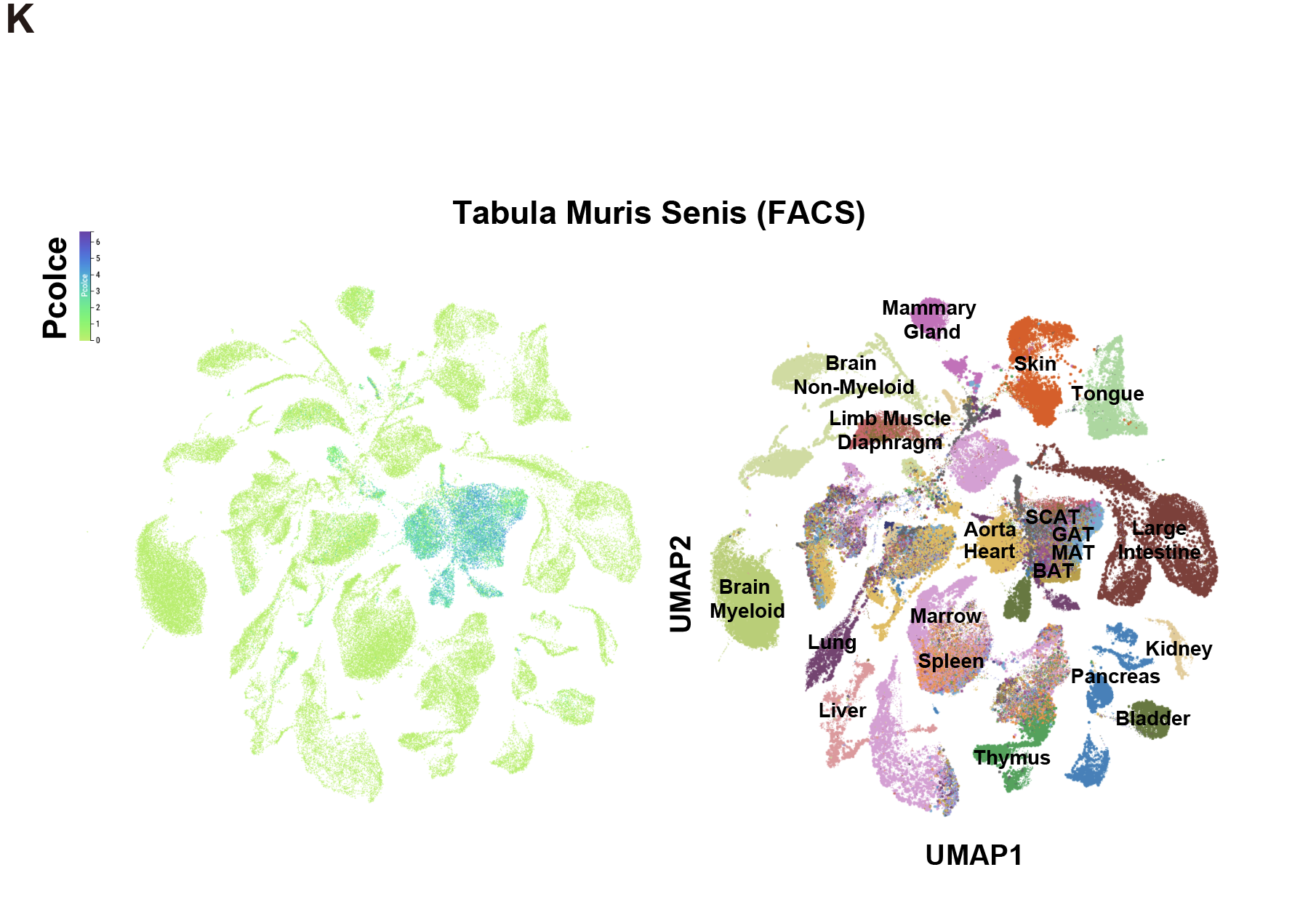

Supplement: Supplementary file 7 — Figure EV1 Source Data [file 44318_2024_196_MOESM7_ESM.zip › Figure EV1/Figure EV1-K/Fig.EV1K.png]

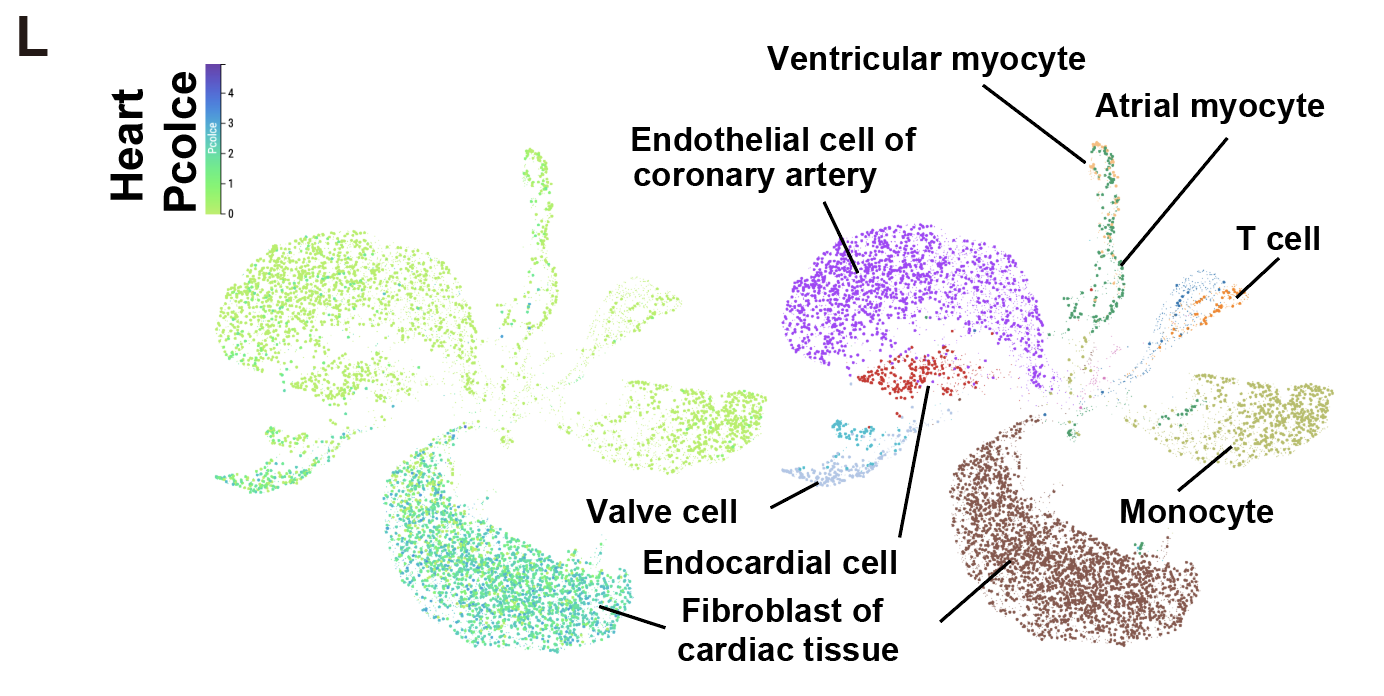

Supplement: Supplementary file 7 — Figure EV1 Source Data [file 44318_2024_196_MOESM7_ESM.zip › Figure EV1/Figure EV1-L/Fig.EV1L.png]

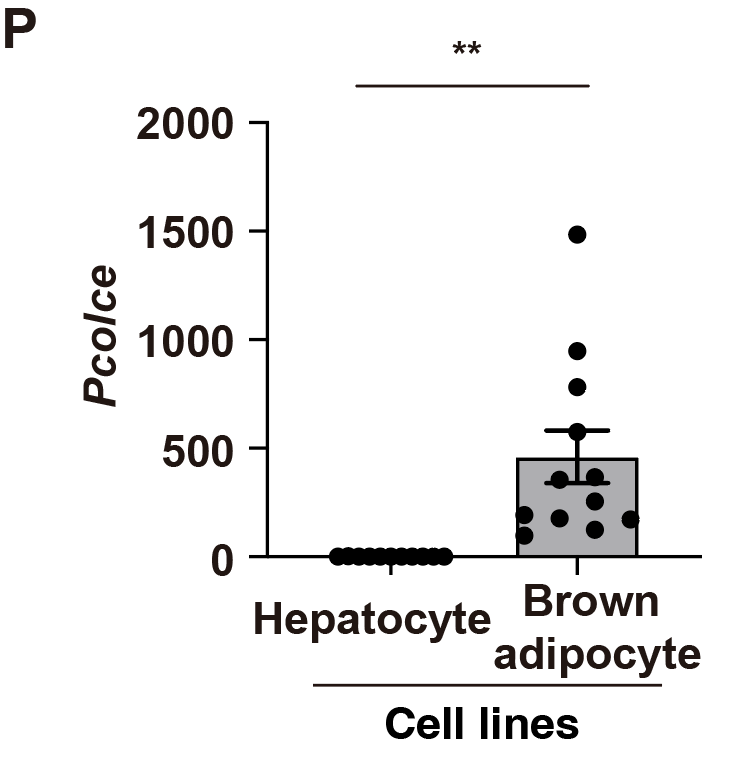

Supplement: Supplementary file 7 — Figure EV1 Source Data [file 44318_2024_196_MOESM7_ESM.zip › Figure EV1/Figure EV1-P/Fig.EV1P.png]

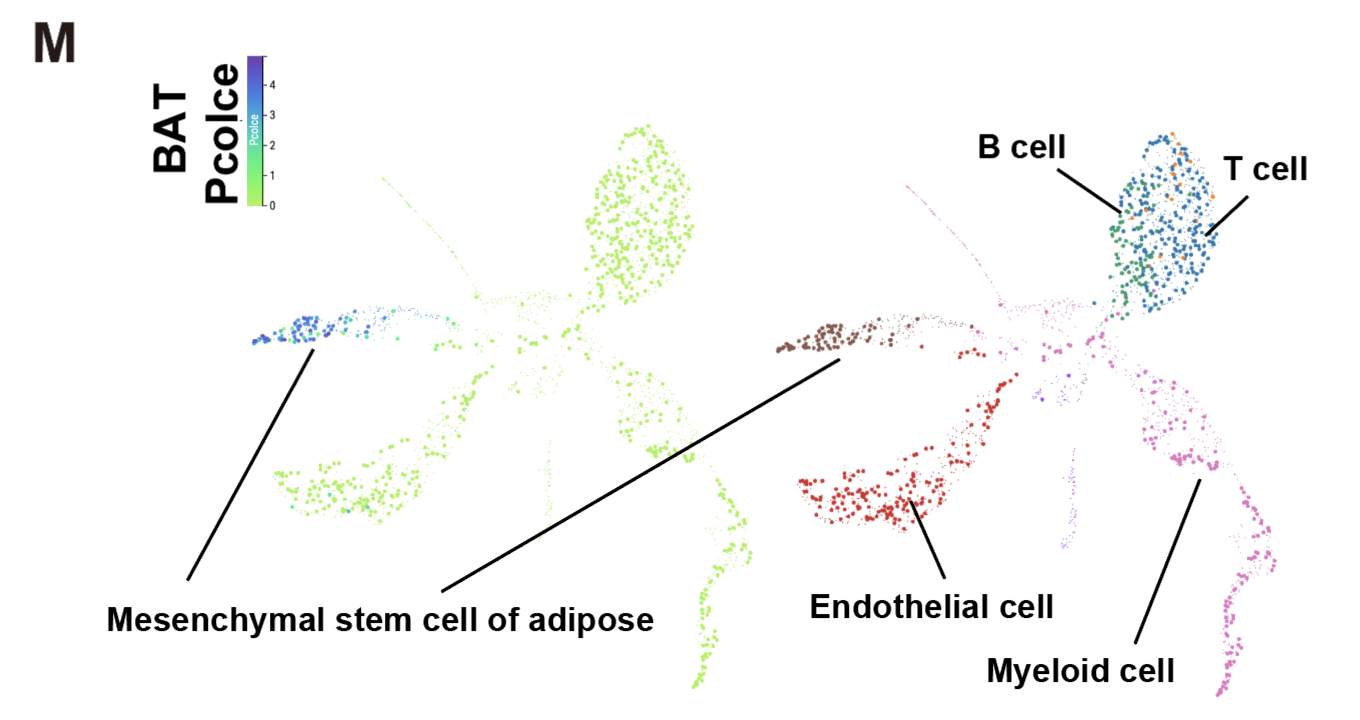

Supplement: Supplementary file 7 — Figure EV1 Source Data [file 44318_2024_196_MOESM7_ESM.zip › Figure EV1/Figure EV1-M/Fig.EV1M.png]

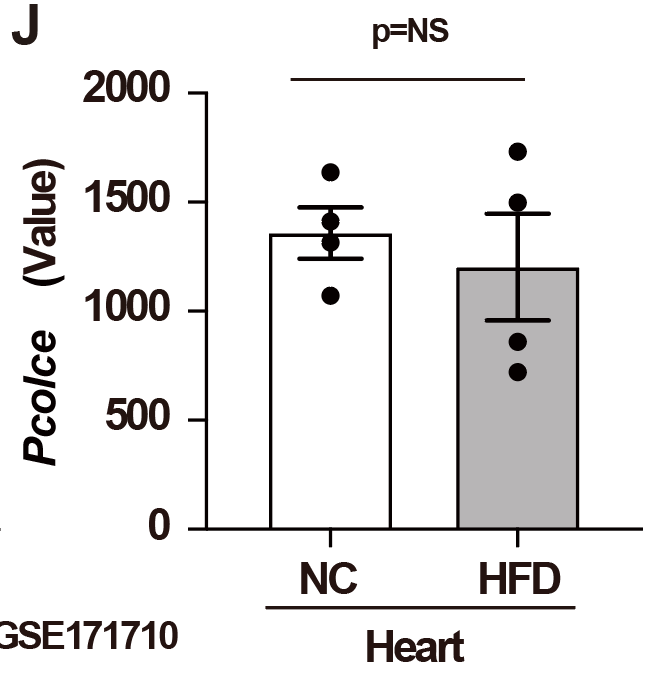

Supplement: Supplementary file 7 — Figure EV1 Source Data [file 44318_2024_196_MOESM7_ESM.zip › Figure EV1/Figure EV1-J/Fig.EV1J.png]

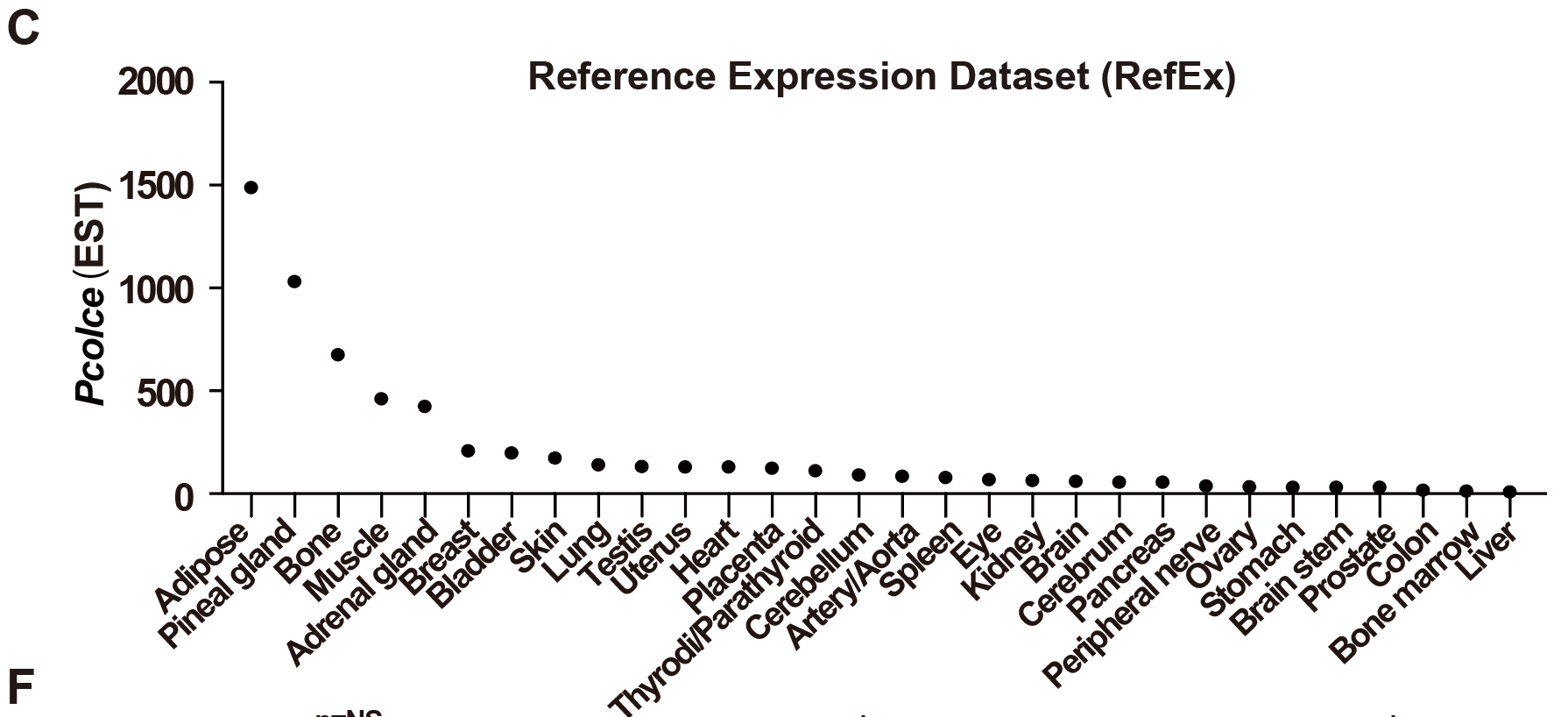

Supplement: Supplementary file 7 — Figure EV1 Source Data [file 44318_2024_196_MOESM7_ESM.zip › Figure EV1/Figure EV1-C/Fig.EV1C.png]

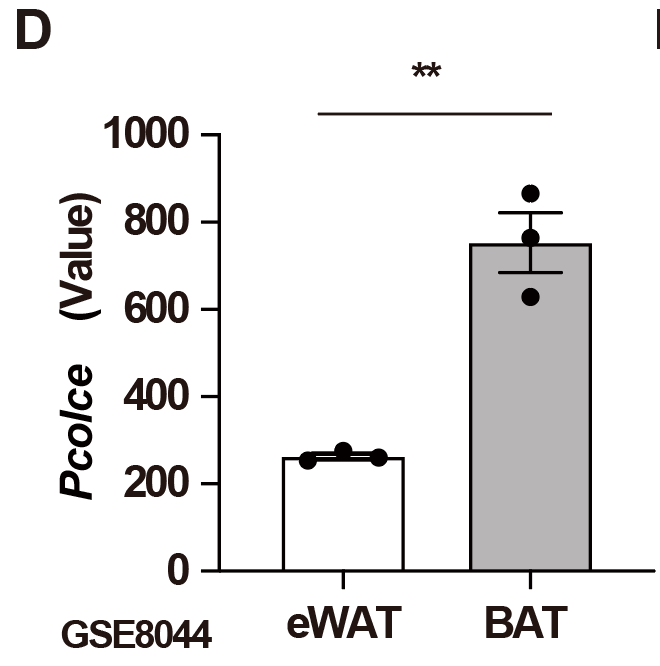

Supplement: Supplementary file 7 — Figure EV1 Source Data [file 44318_2024_196_MOESM7_ESM.zip › Figure EV1/Figure EV1-D/Fig.EV1D.png]

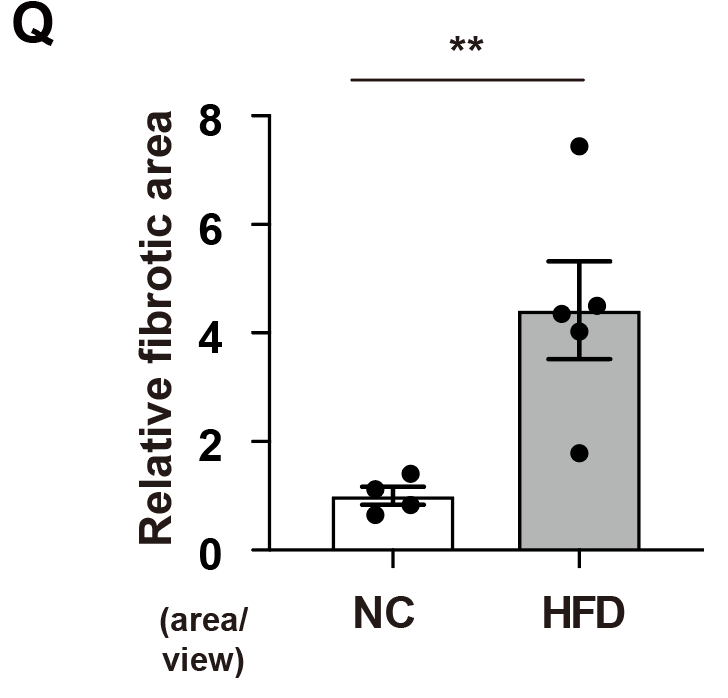

Supplement: Supplementary file 7 — Figure EV1 Source Data [file 44318_2024_196_MOESM7_ESM.zip › Figure EV1/Figure EV1-Q/Fig.EV1Q.png]

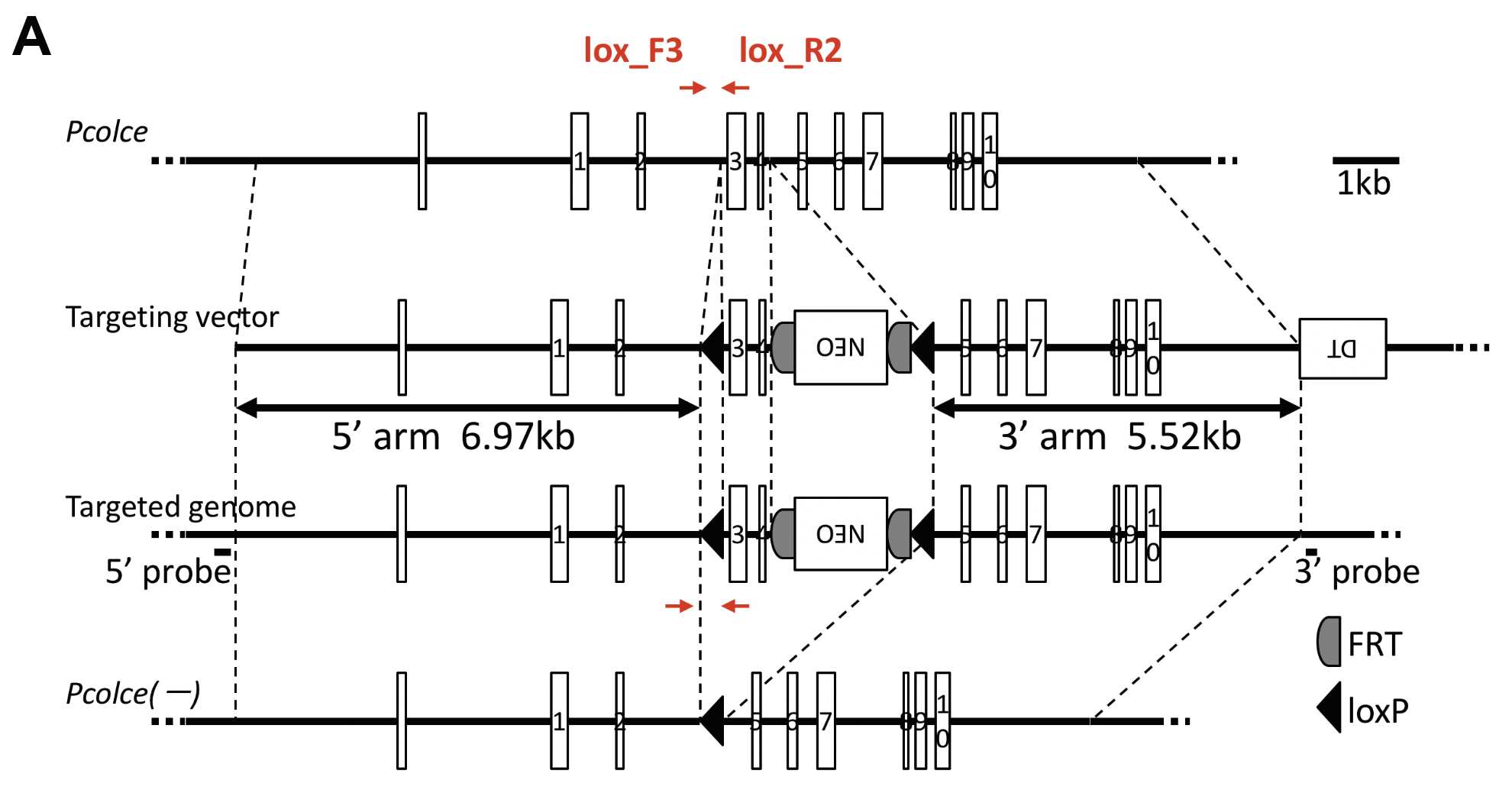

Supplement: Supplementary file 8 — Figure EV2 Source Data [file 44318_2024_196_MOESM8_ESM.zip › Figure EV2/Figure EV2-A/Fig.EV2A.png]

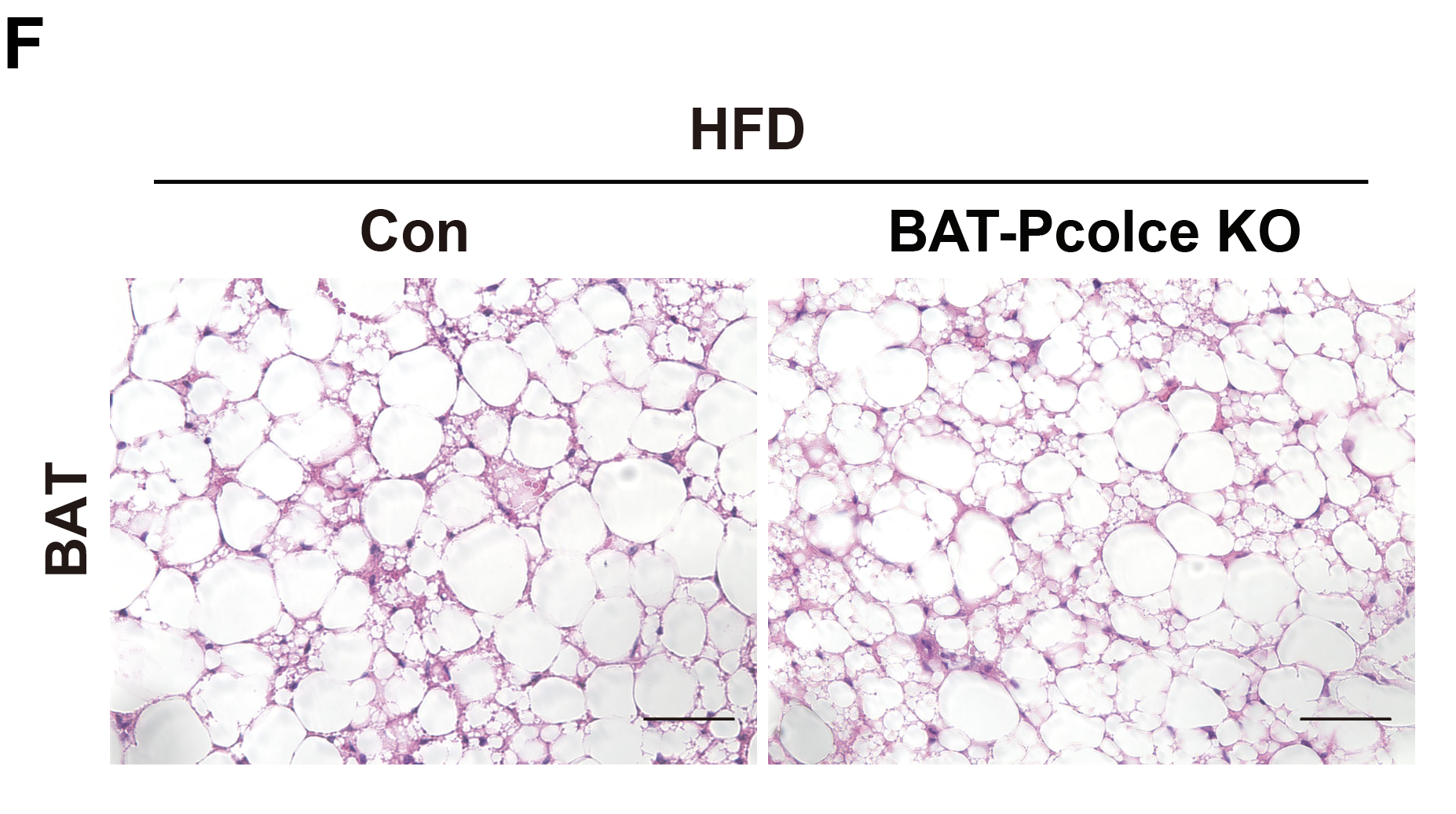

Supplement: Supplementary file 8 — Figure EV2 Source Data [file 44318_2024_196_MOESM8_ESM.zip › Figure EV2/Figure EV2-F/Fig.EV2F.png]

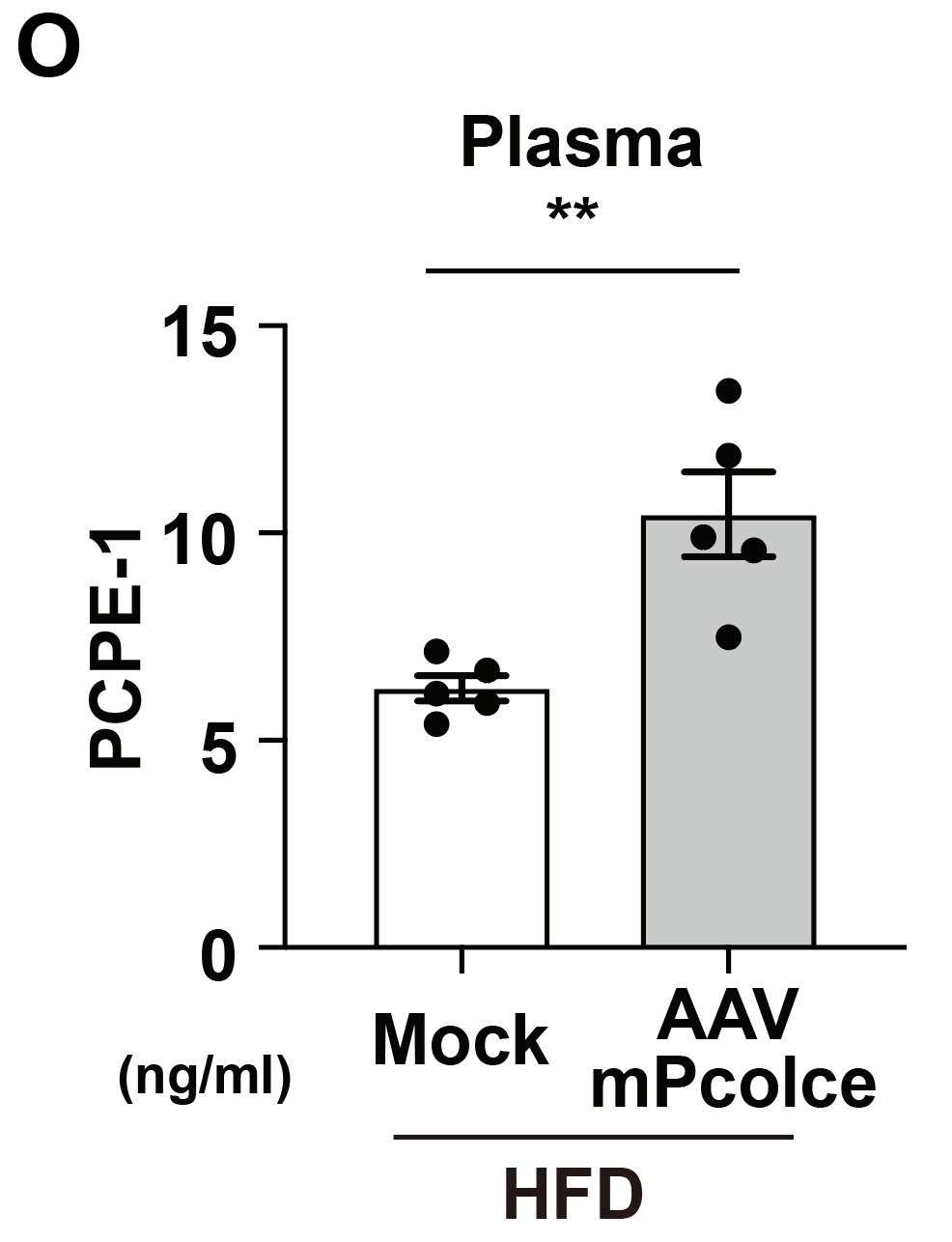

Supplement: Supplementary file 8 — Figure EV2 Source Data [file 44318_2024_196_MOESM8_ESM.zip › Figure EV2/Figure EV2-O/Fig.EV2O.png]

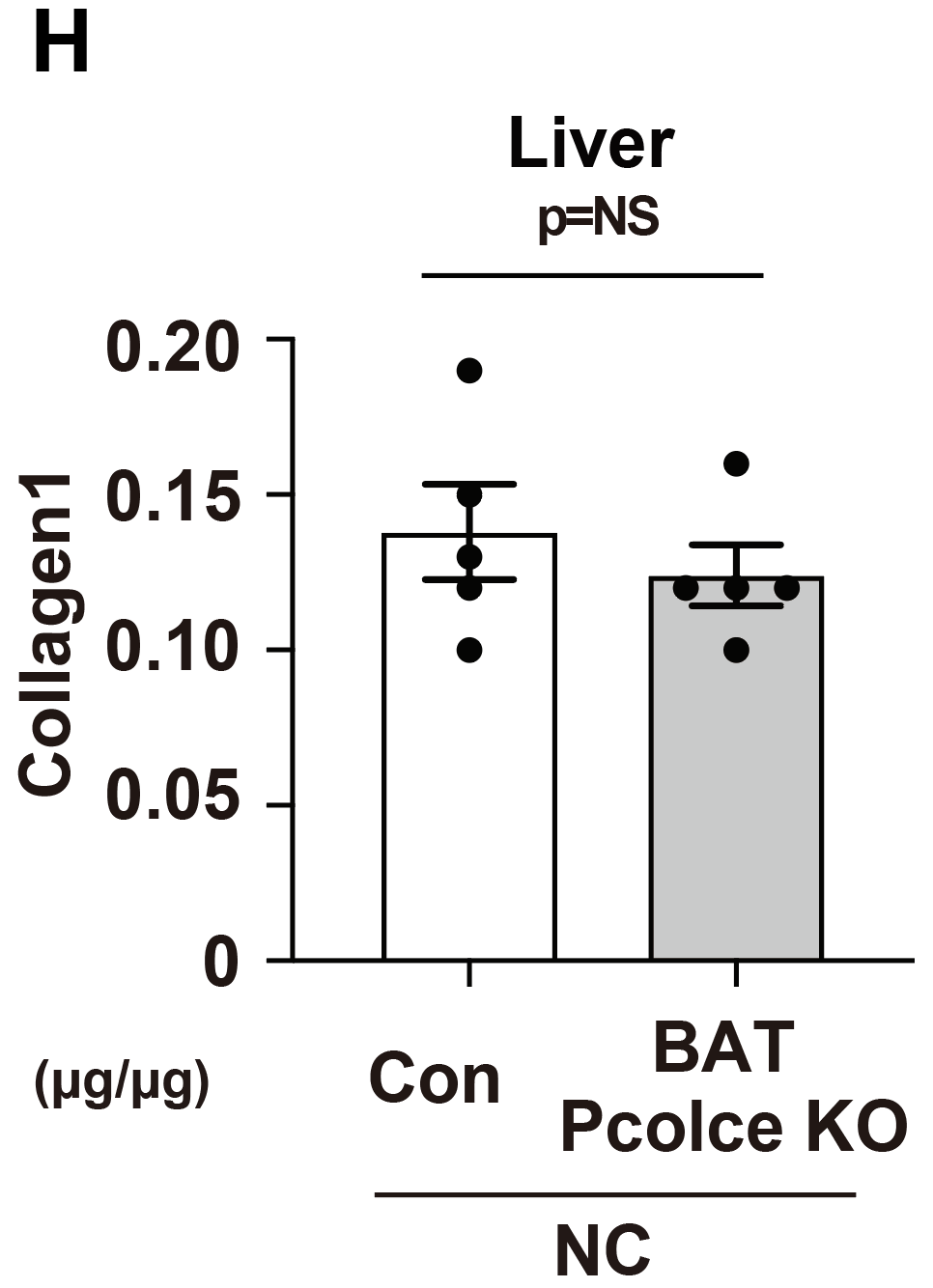

Supplement: Supplementary file 8 — Figure EV2 Source Data [file 44318_2024_196_MOESM8_ESM.zip › Figure EV2/Figure EV2-H/Fig.EV2H.png]

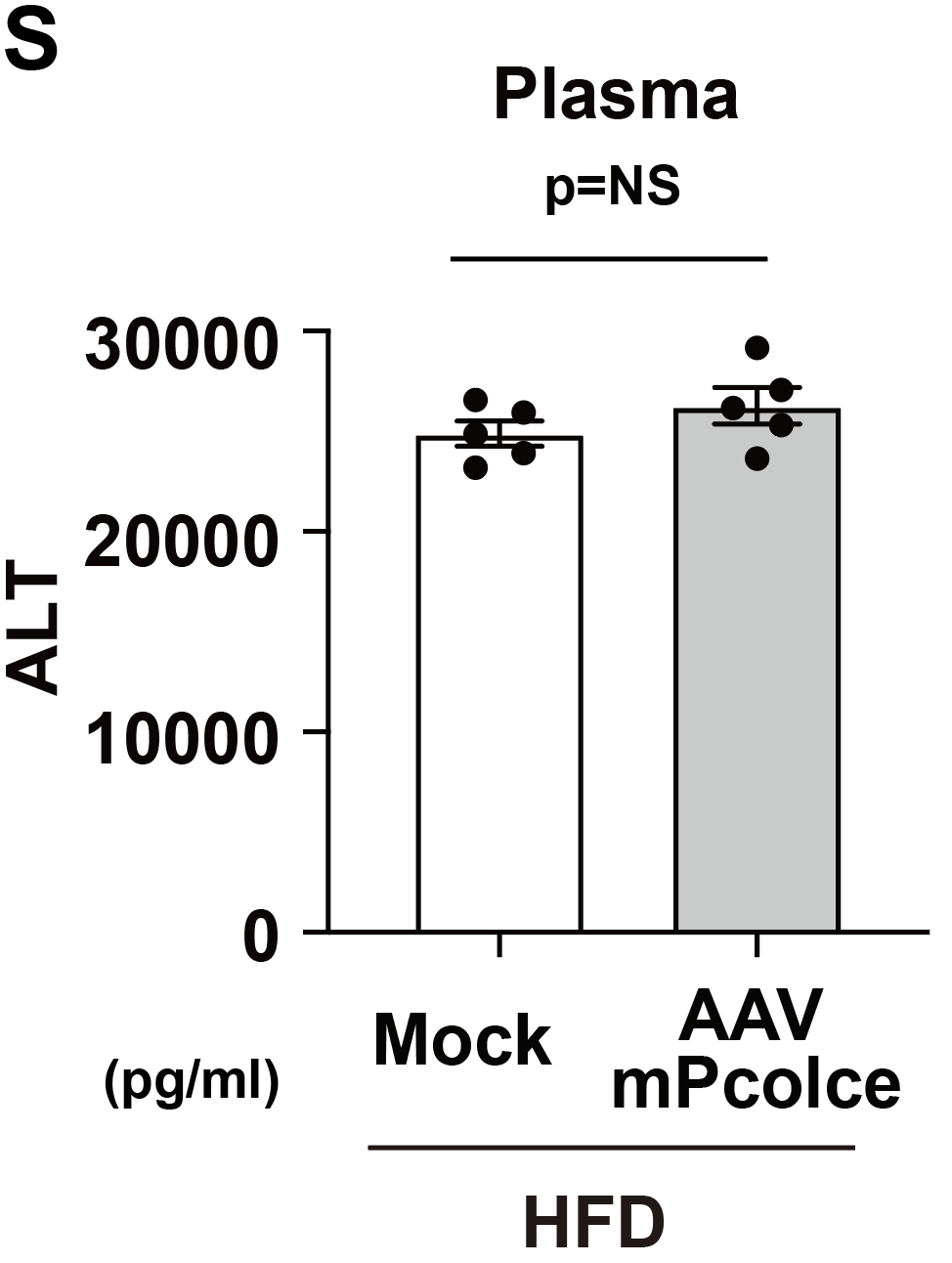

Supplement: Supplementary file 8 — Figure EV2 Source Data [file 44318_2024_196_MOESM8_ESM.zip › Figure EV2/Figure EV2-S/Fig.EV2S.png]
