# Supplementary figures and images for: PCPE-1, a brown adipose tissue-derived cytokine, promotes obesity-induced liver fibrosis (part 4 of 6)
Source: EMBO J. 2024 Aug 19;43(21):4846–69. doi: 10.1038/s44318-024-00196-0 (PMC11535236; doi:10.1038/s44318-024-00196-0)

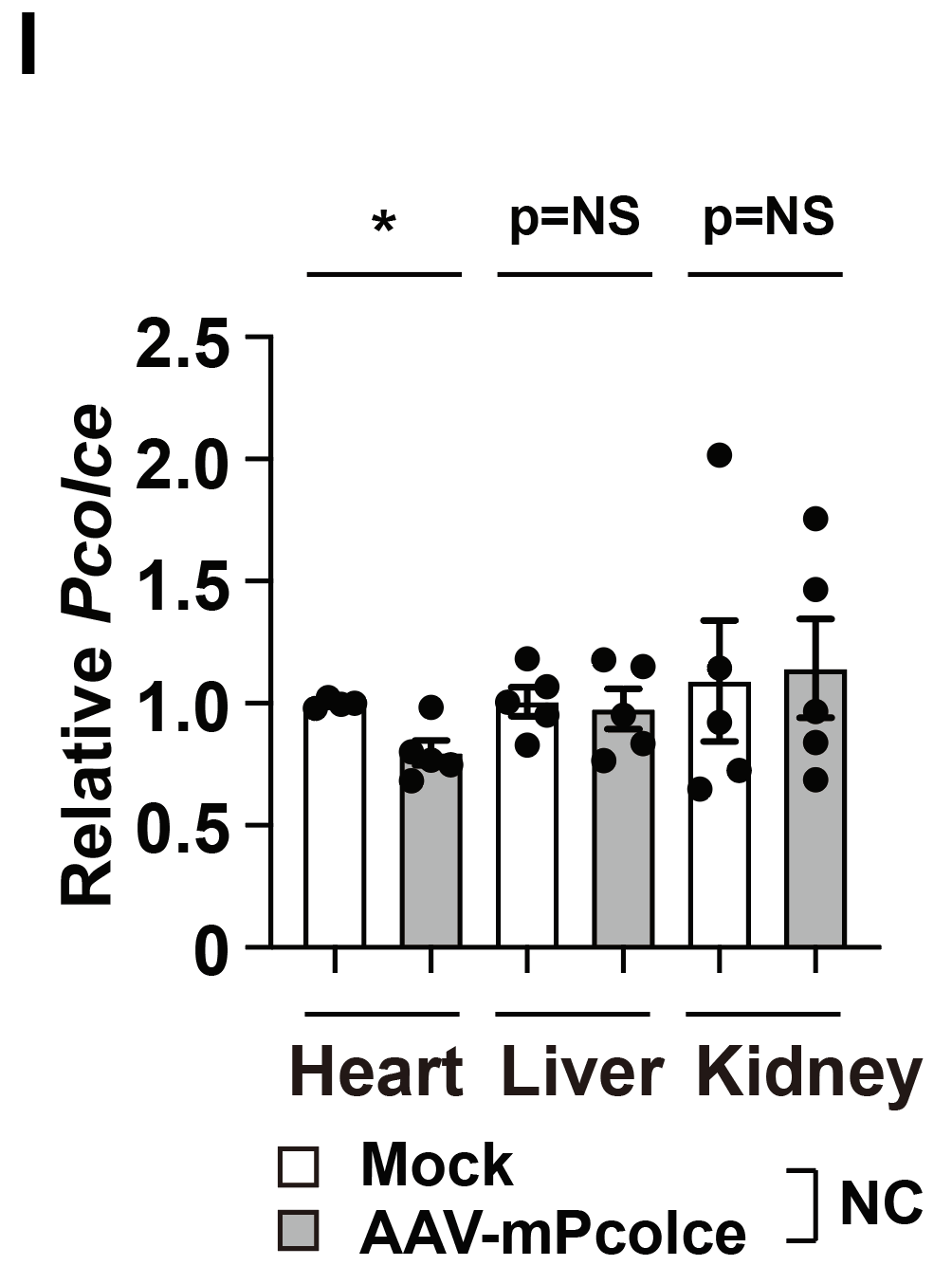

Supplement: Supplementary file 8 — Figure EV2 Source Data [file 44318_2024_196_MOESM8_ESM.zip › Figure EV2/Figure EV2-I/Fig.EV2I.png]

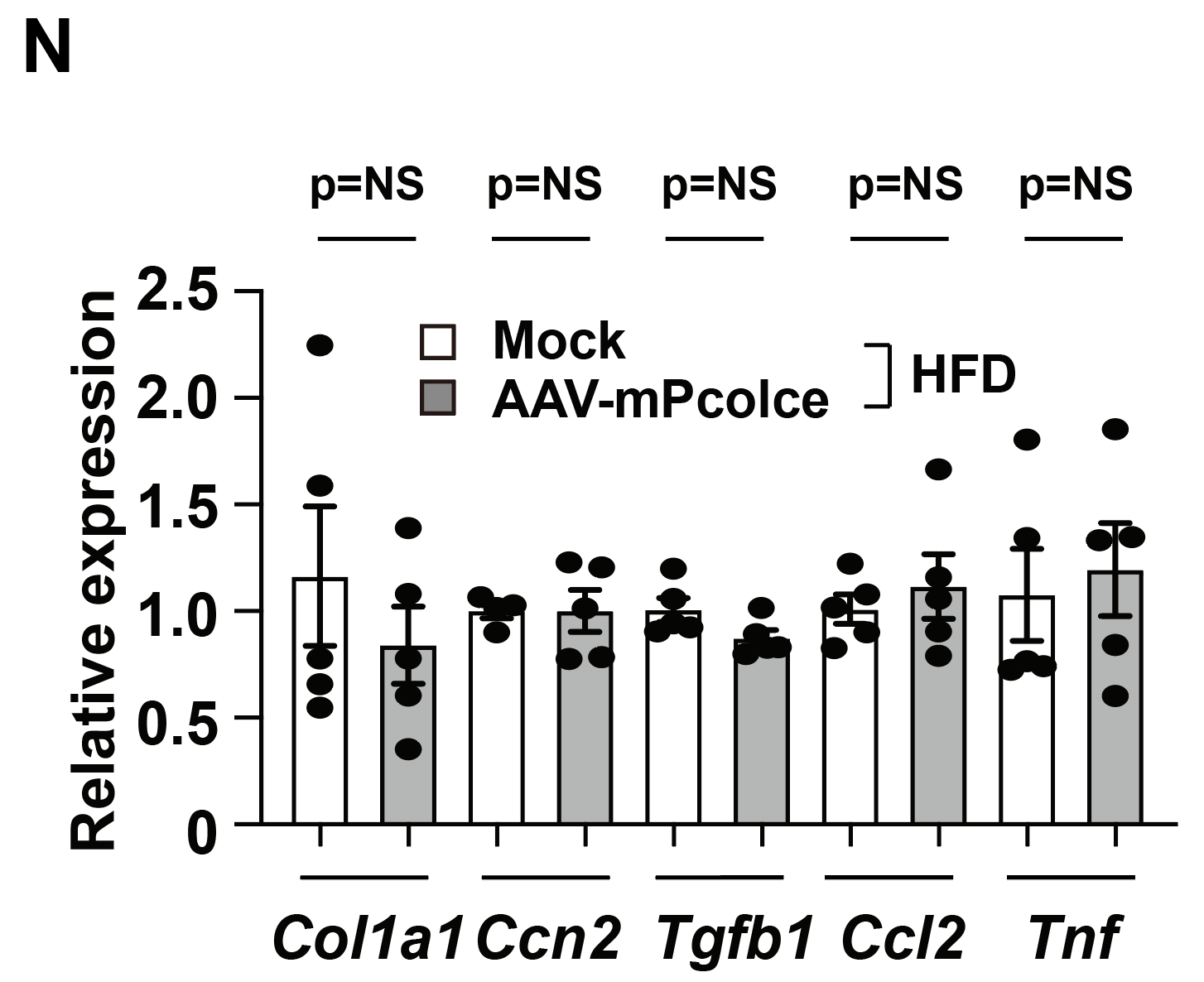

Supplement: Supplementary file 8 — Figure EV2 Source Data [file 44318_2024_196_MOESM8_ESM.zip › Figure EV2/Figure EV2-N/Fig.EV2N.png]

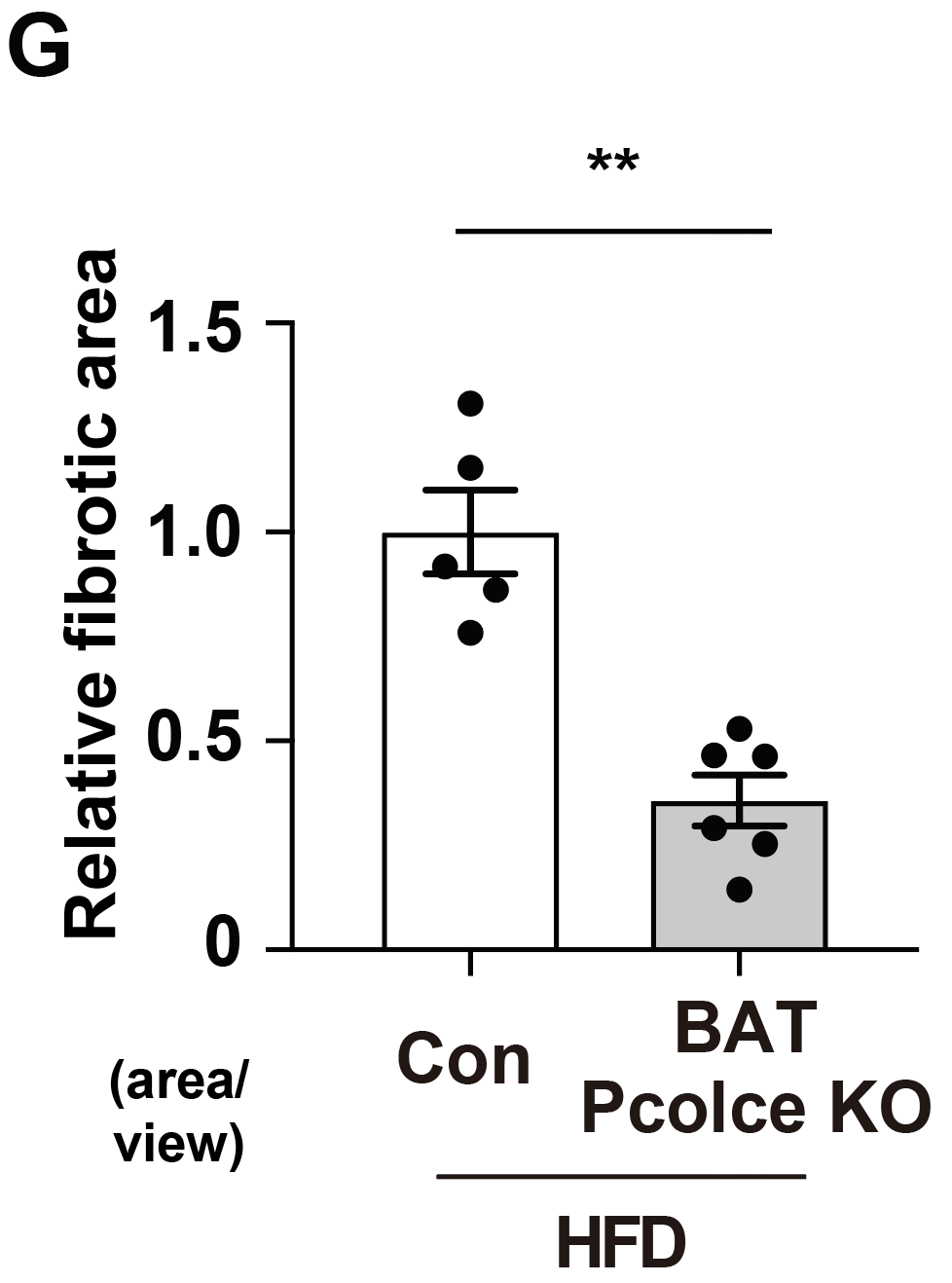

Supplement: Supplementary file 8 — Figure EV2 Source Data [file 44318_2024_196_MOESM8_ESM.zip › Figure EV2/Figure EV2-G/Fig.EV2G.png]

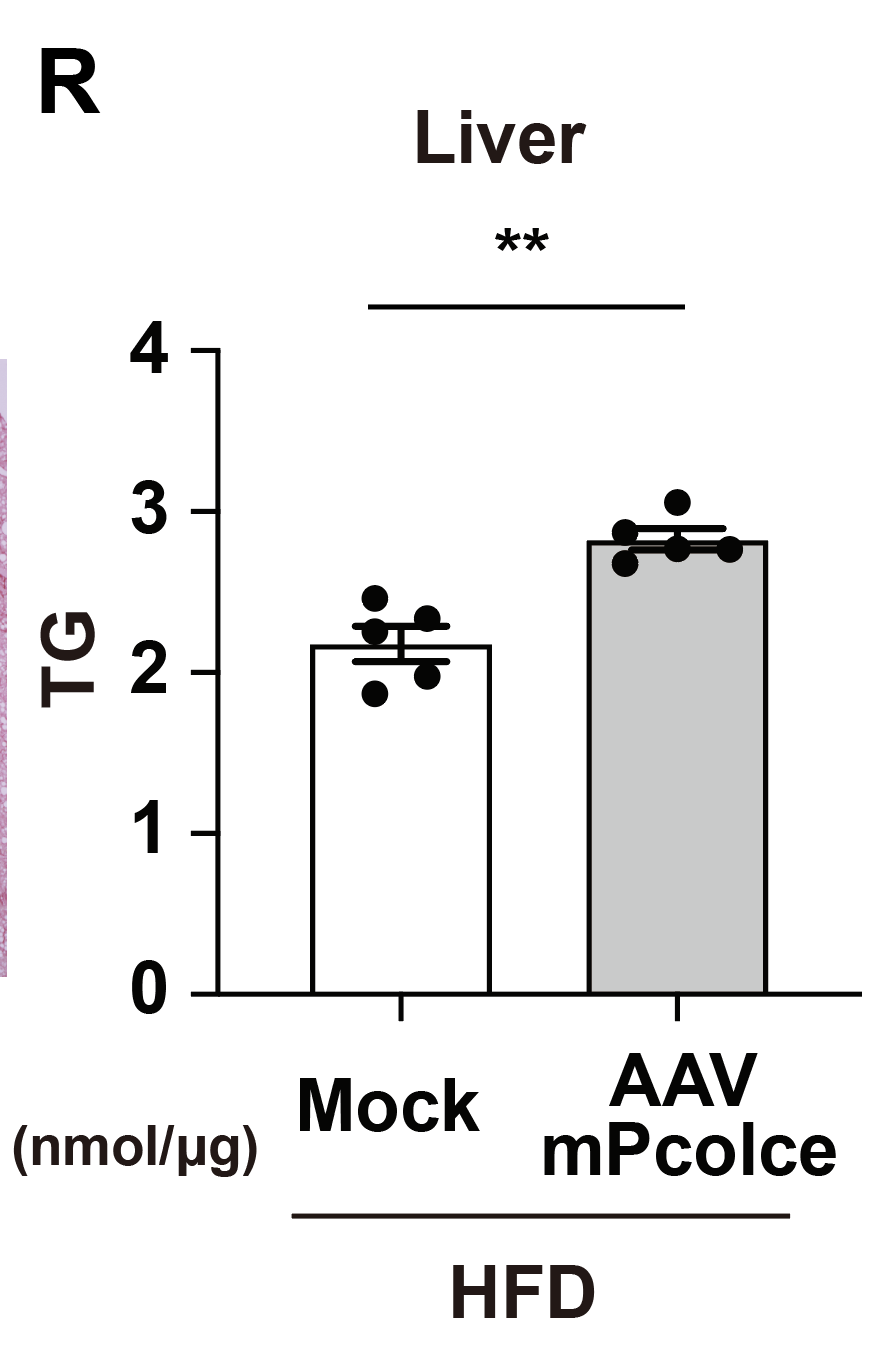

Supplement: Supplementary file 8 — Figure EV2 Source Data [file 44318_2024_196_MOESM8_ESM.zip › Figure EV2/Figure EV2-R/Fig.EV2R.png]

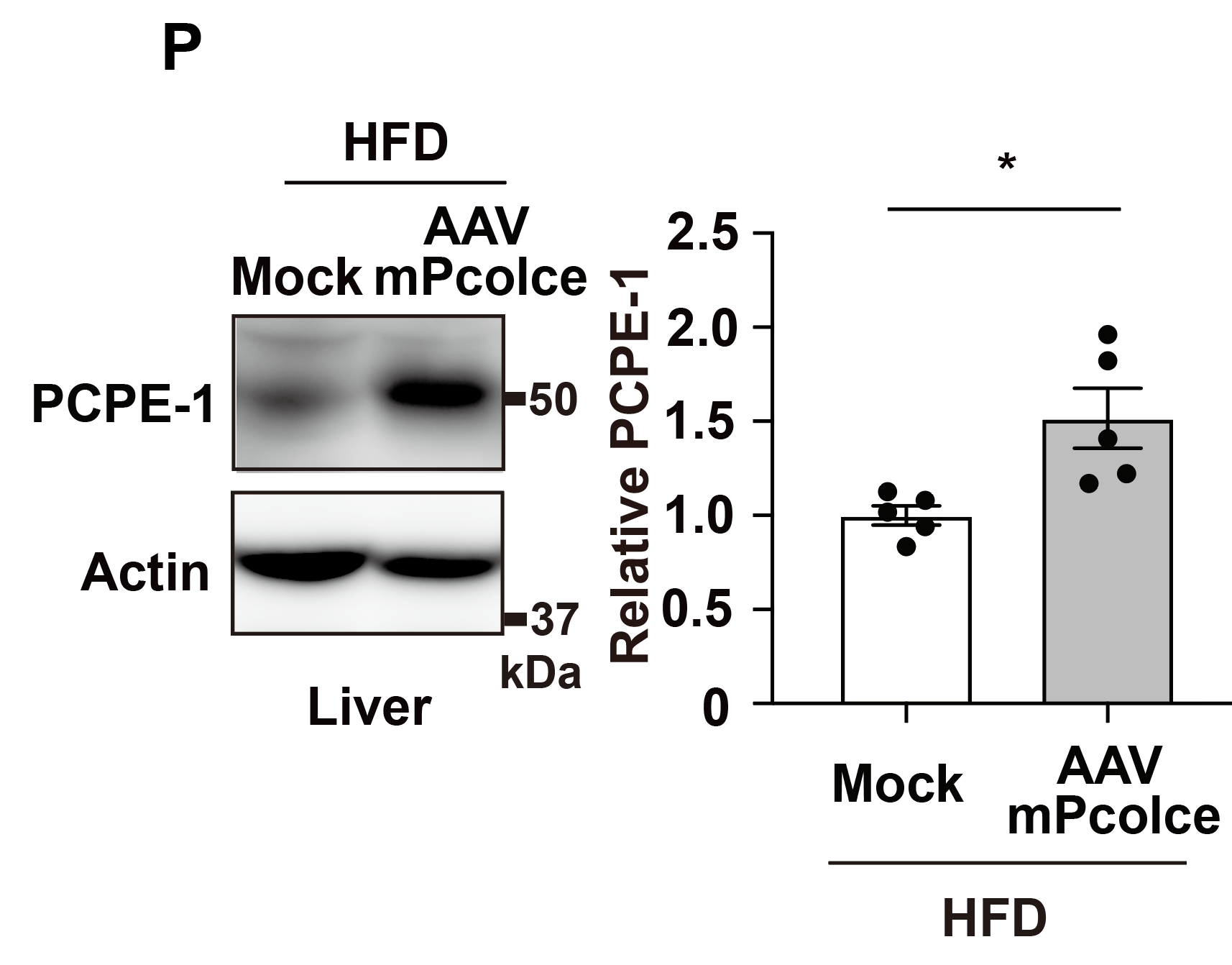

Supplement: Supplementary file 8 — Figure EV2 Source Data [file 44318_2024_196_MOESM8_ESM.zip › Figure EV2/Figure EV2-P/Fig.EV2P.png]

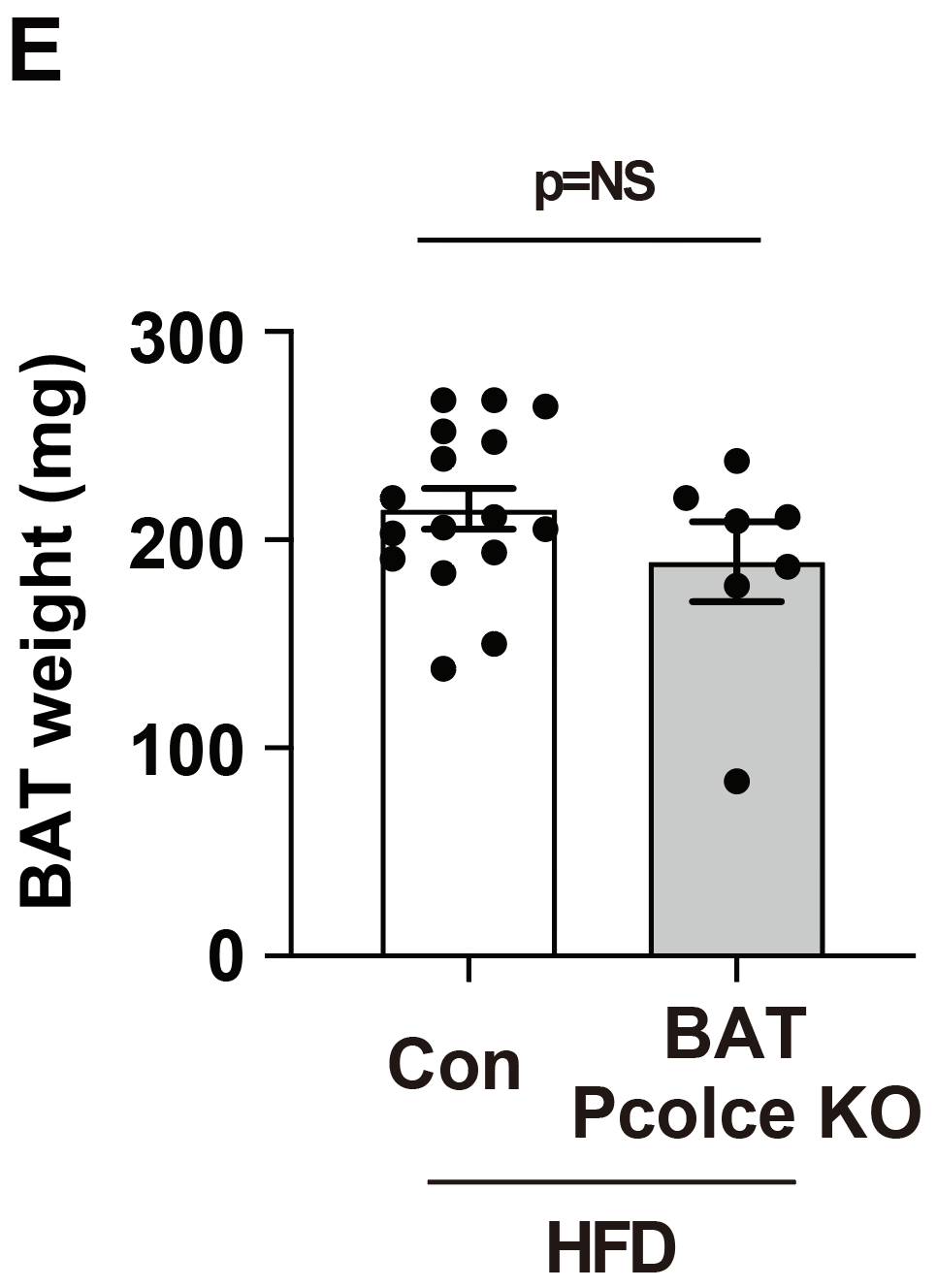

Supplement: Supplementary file 8 — Figure EV2 Source Data [file 44318_2024_196_MOESM8_ESM.zip › Figure EV2/Figure EV2-E/Fig.EV2E.png]

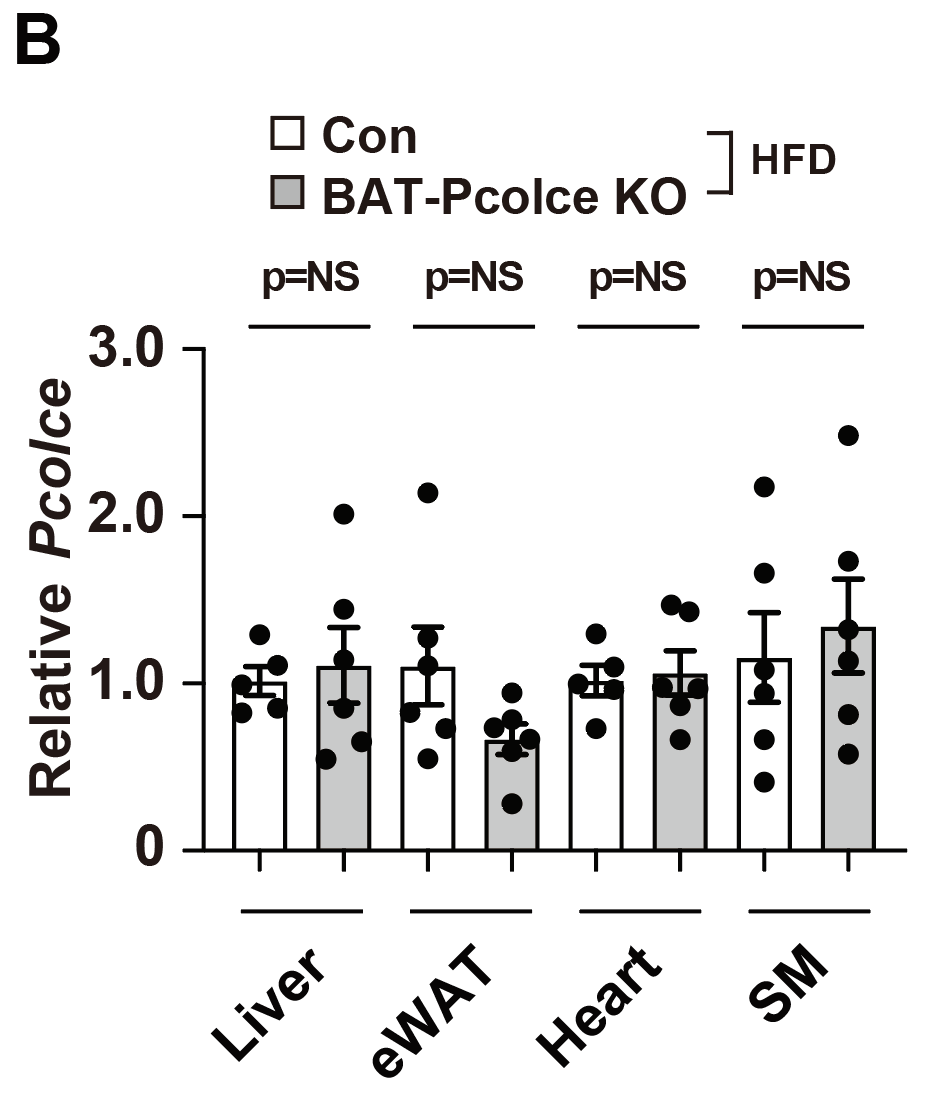

Supplement: Supplementary file 8 — Figure EV2 Source Data [file 44318_2024_196_MOESM8_ESM.zip › Figure EV2/Figure EV2-B/Fig.EV2B.png]

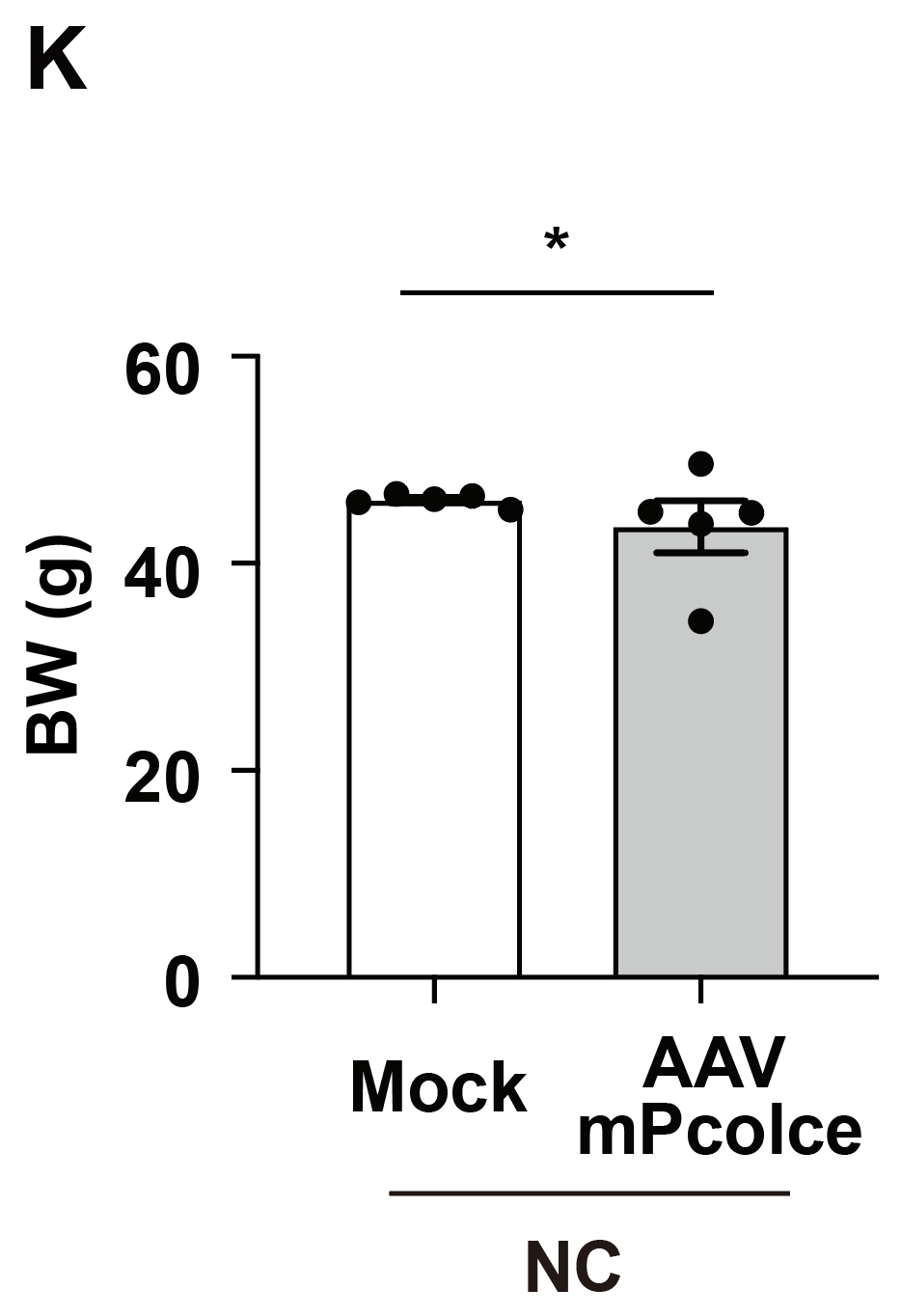

Supplement: Supplementary file 8 — Figure EV2 Source Data [file 44318_2024_196_MOESM8_ESM.zip › Figure EV2/Figure EV2-K/Fig.EV2K.png]

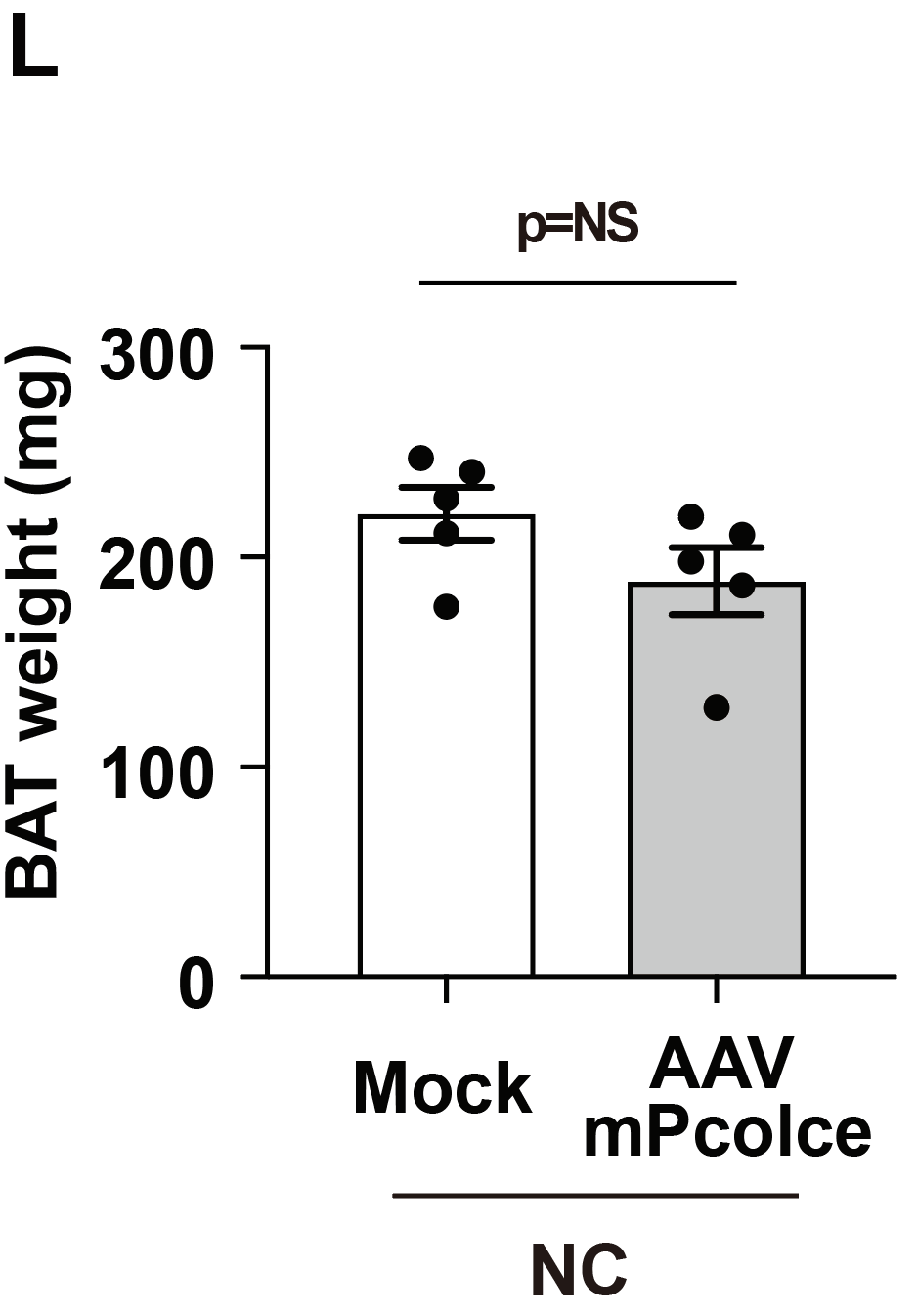

Supplement: Supplementary file 8 — Figure EV2 Source Data [file 44318_2024_196_MOESM8_ESM.zip › Figure EV2/Figure EV2-L/Fig.EV2L.png]

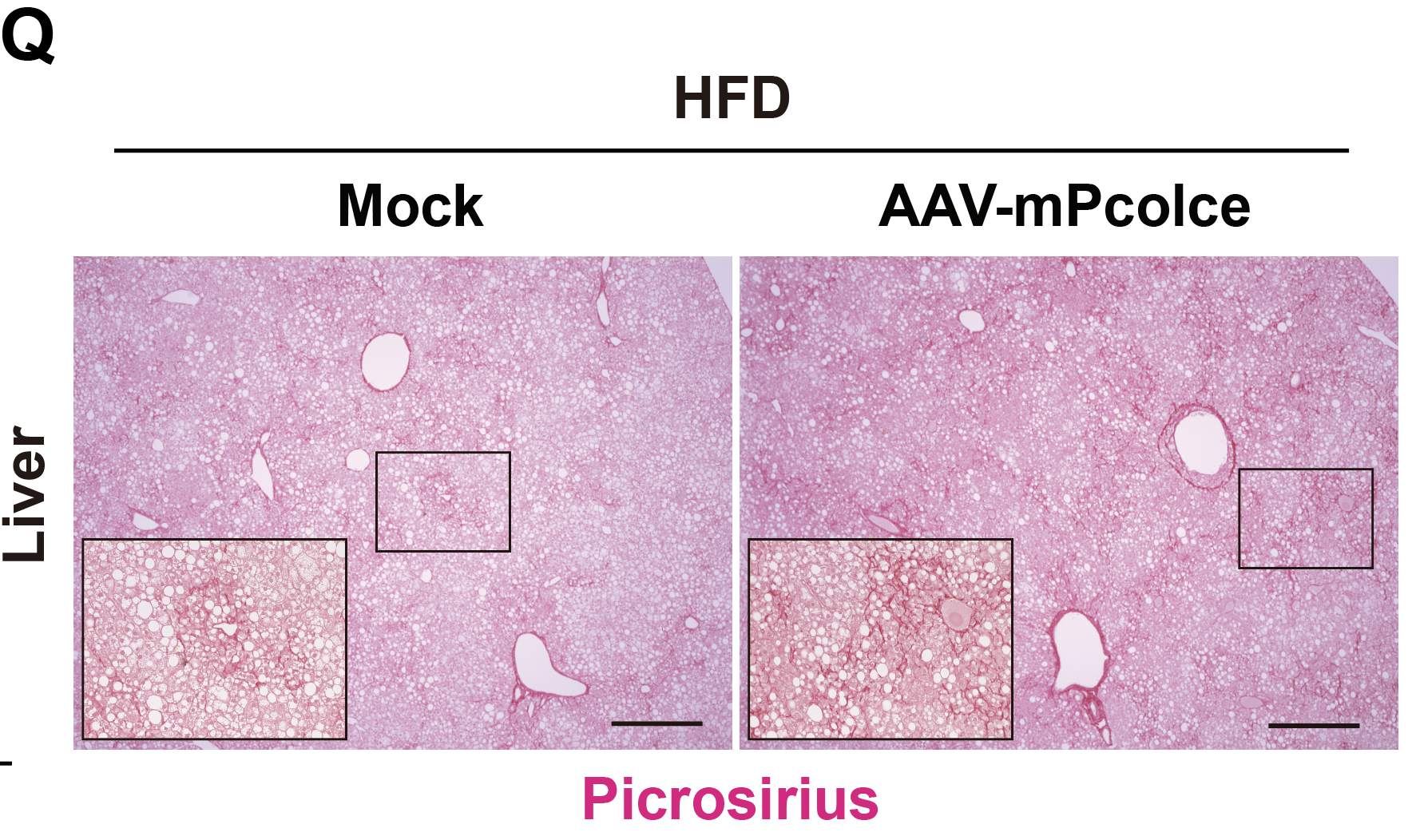

Supplement: Supplementary file 8 — Figure EV2 Source Data [file 44318_2024_196_MOESM8_ESM.zip › Figure EV2/Figure EV2-Q/Fig.EV2Q.png]

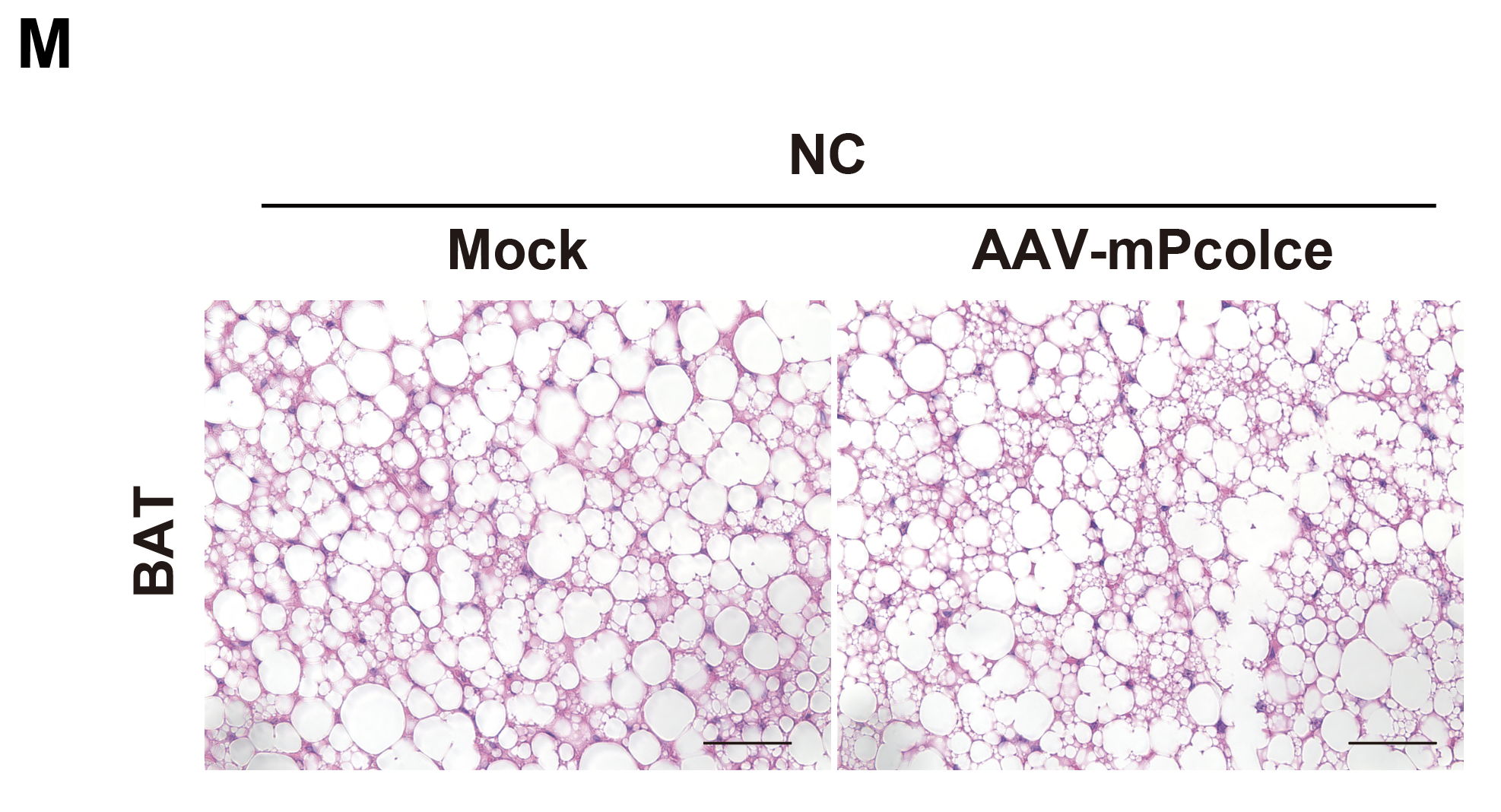

Supplement: Supplementary file 8 — Figure EV2 Source Data [file 44318_2024_196_MOESM8_ESM.zip › Figure EV2/Figure EV2-M/Fig.EV2M.png]

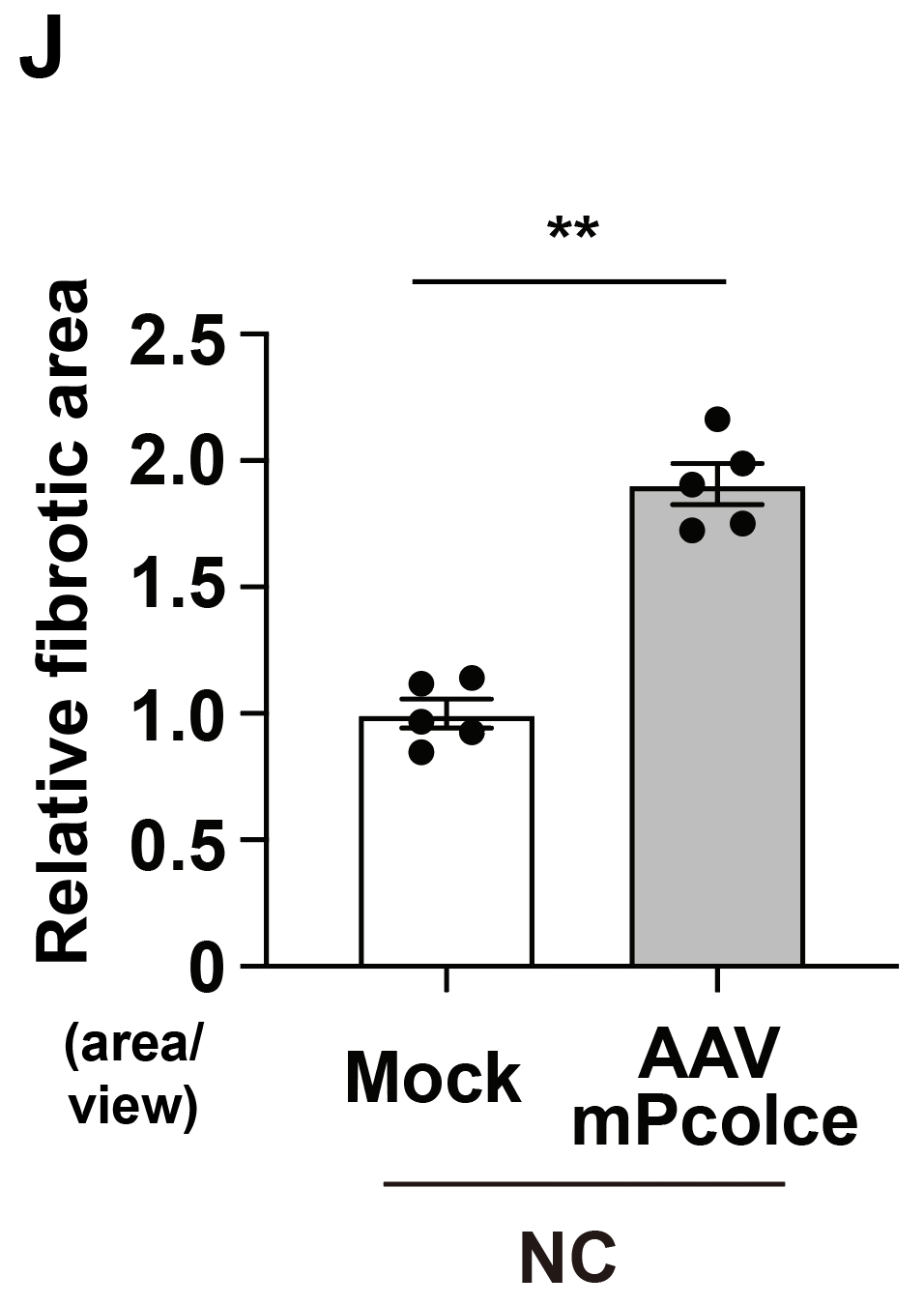

Supplement: Supplementary file 8 — Figure EV2 Source Data [file 44318_2024_196_MOESM8_ESM.zip › Figure EV2/Figure EV2-J/Figure EV2J.png]

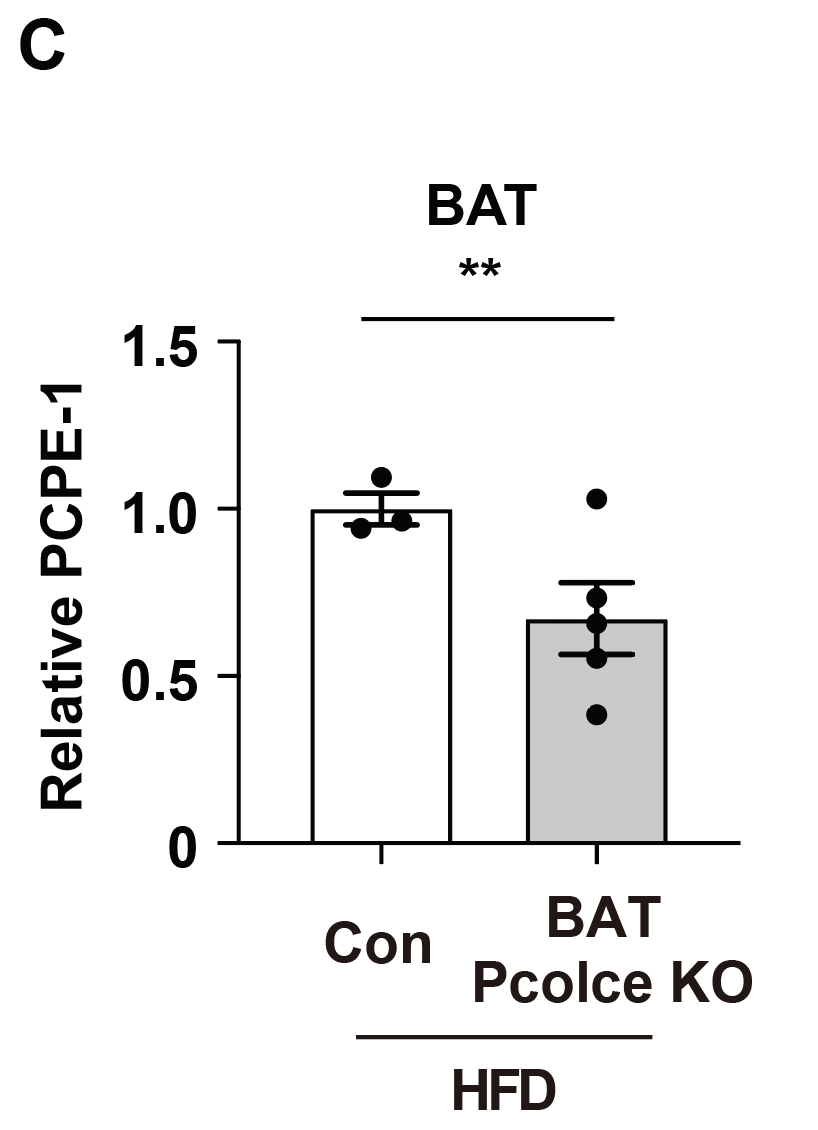

Supplement: Supplementary file 8 — Figure EV2 Source Data [file 44318_2024_196_MOESM8_ESM.zip › Figure EV2/Figure EV2-C/Fig.EV2C.png]

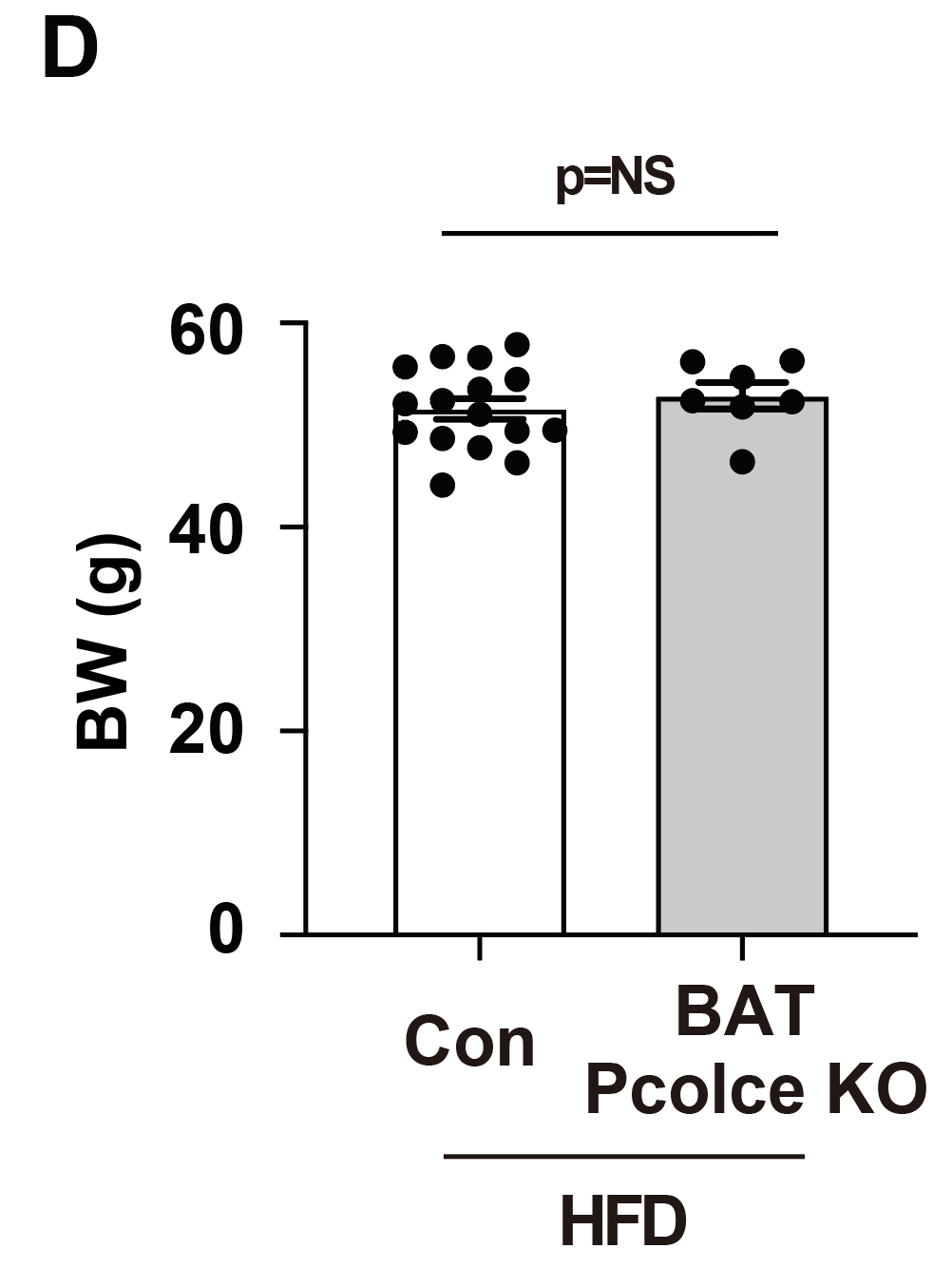

Supplement: Supplementary file 8 — Figure EV2 Source Data [file 44318_2024_196_MOESM8_ESM.zip › Figure EV2/Figure EV2-D/Fig.EV2D.png]

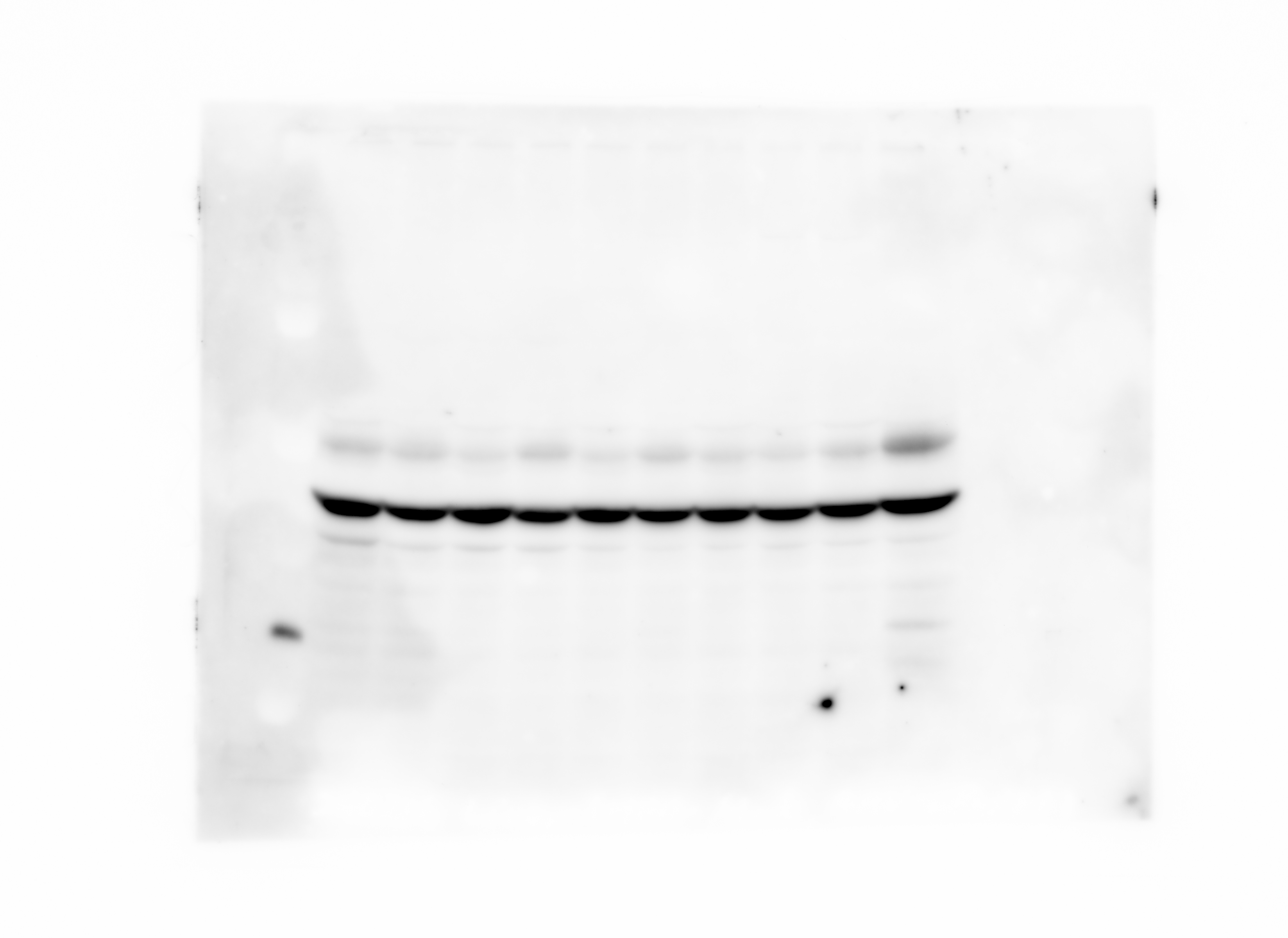

Supplement: Supplementary file 8 — Figure EV2 Source Data [file 44318_2024_196_MOESM8_ESM.zip › Figure EV2/Figure EV2-P/Quantificated image/Actin.tif]

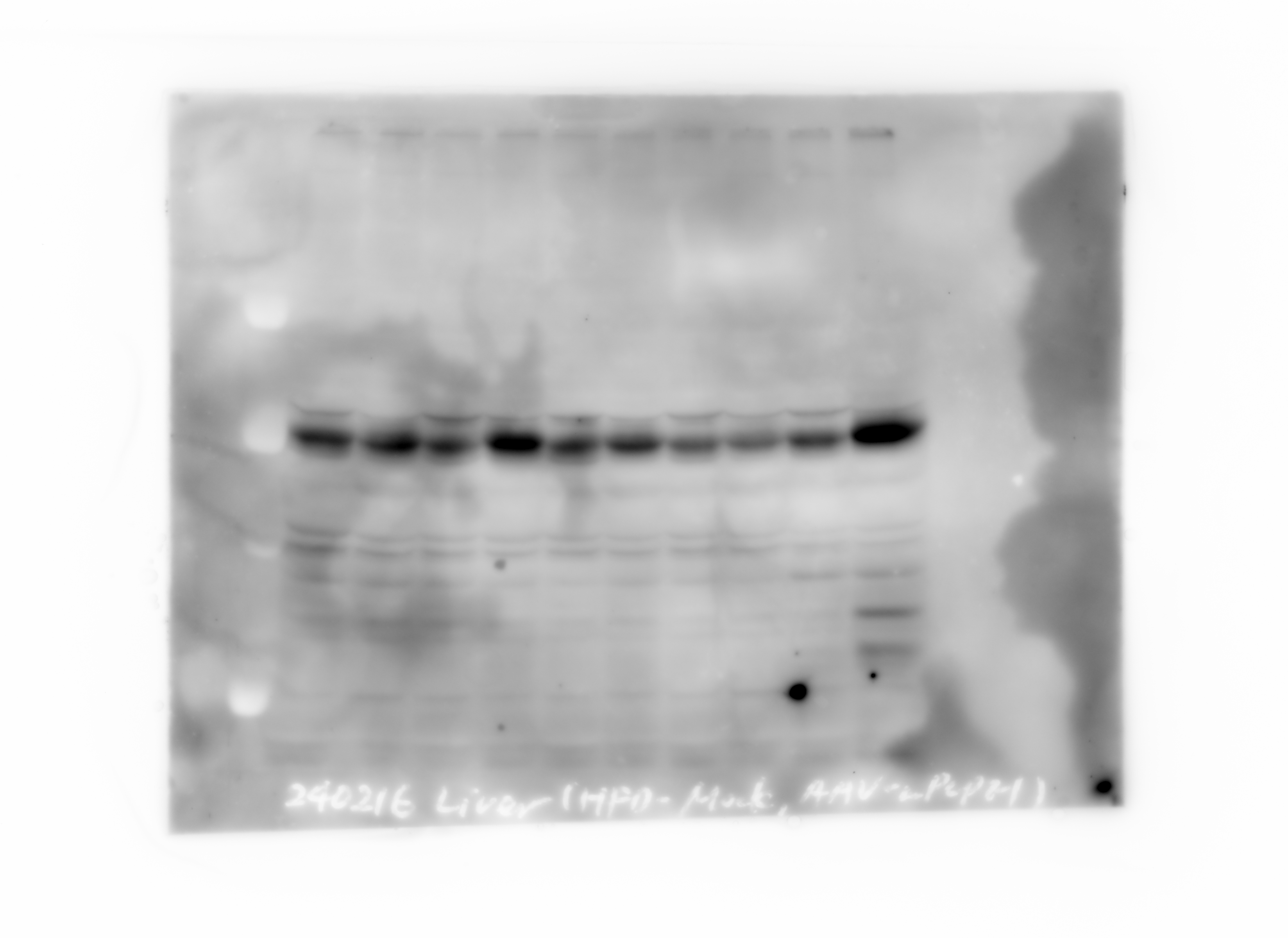

Supplement: Supplementary file 8 — Figure EV2 Source Data [file 44318_2024_196_MOESM8_ESM.zip › Figure EV2/Figure EV2-P/Quantificated image/PCPE-1.tif]

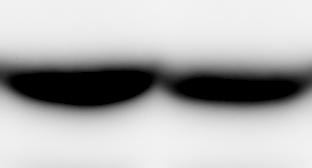

Supplement: Supplementary file 8 — Figure EV2 Source Data [file 44318_2024_196_MOESM8_ESM.zip › Figure EV2/Figure EV2-P/Demonstrated image/Actin.tif]

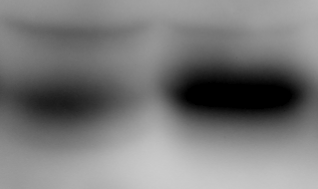

Supplement: Supplementary file 8 — Figure EV2 Source Data [file 44318_2024_196_MOESM8_ESM.zip › Figure EV2/Figure EV2-P/Demonstrated image/PCPE-1.tif]

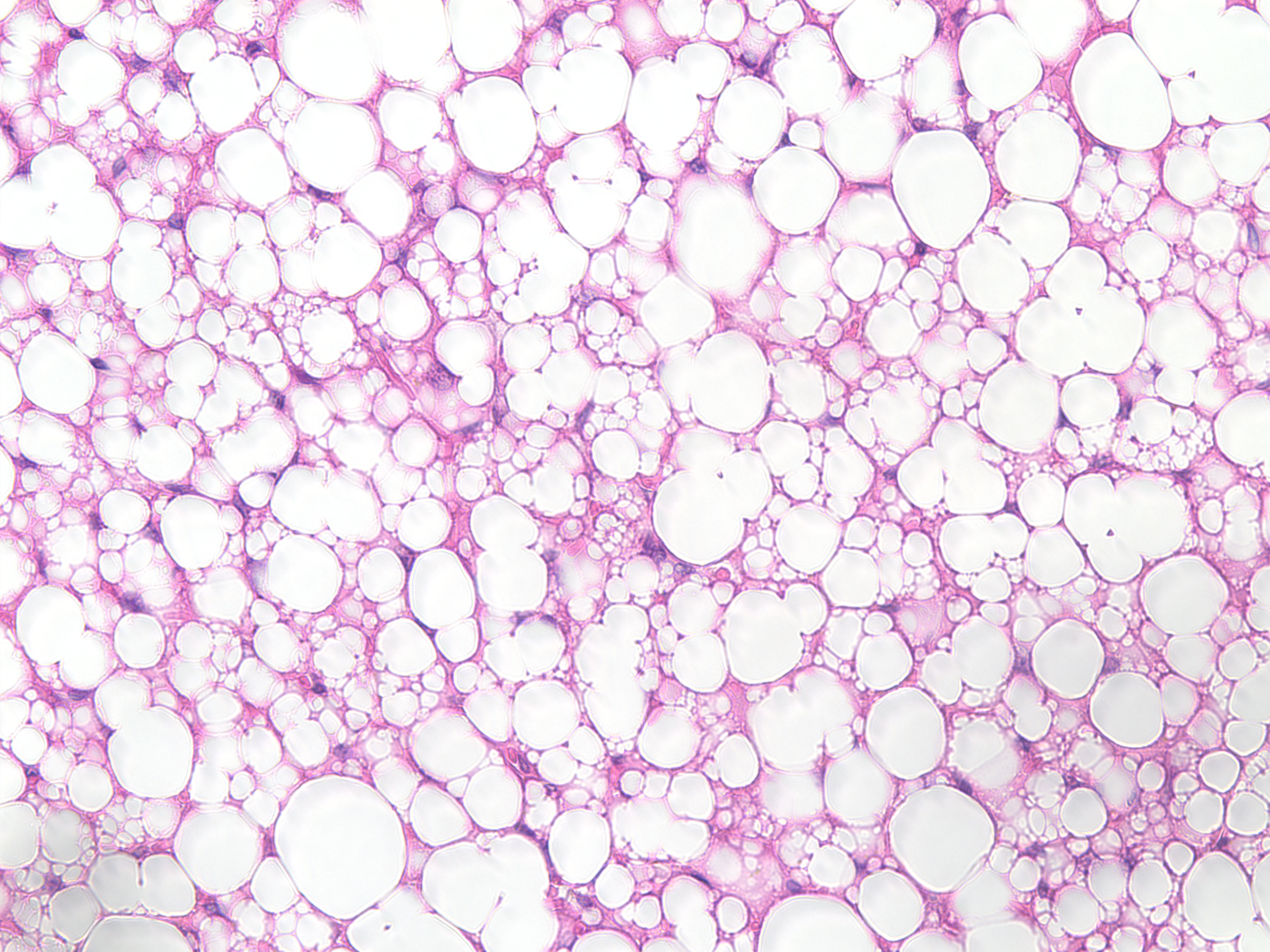

Supplement: Supplementary file 8 — Figure EV2 Source Data [file 44318_2024_196_MOESM8_ESM.zip › Figure EV2/Figure EV2-M/Demonstrated data/NC Mock.tif]

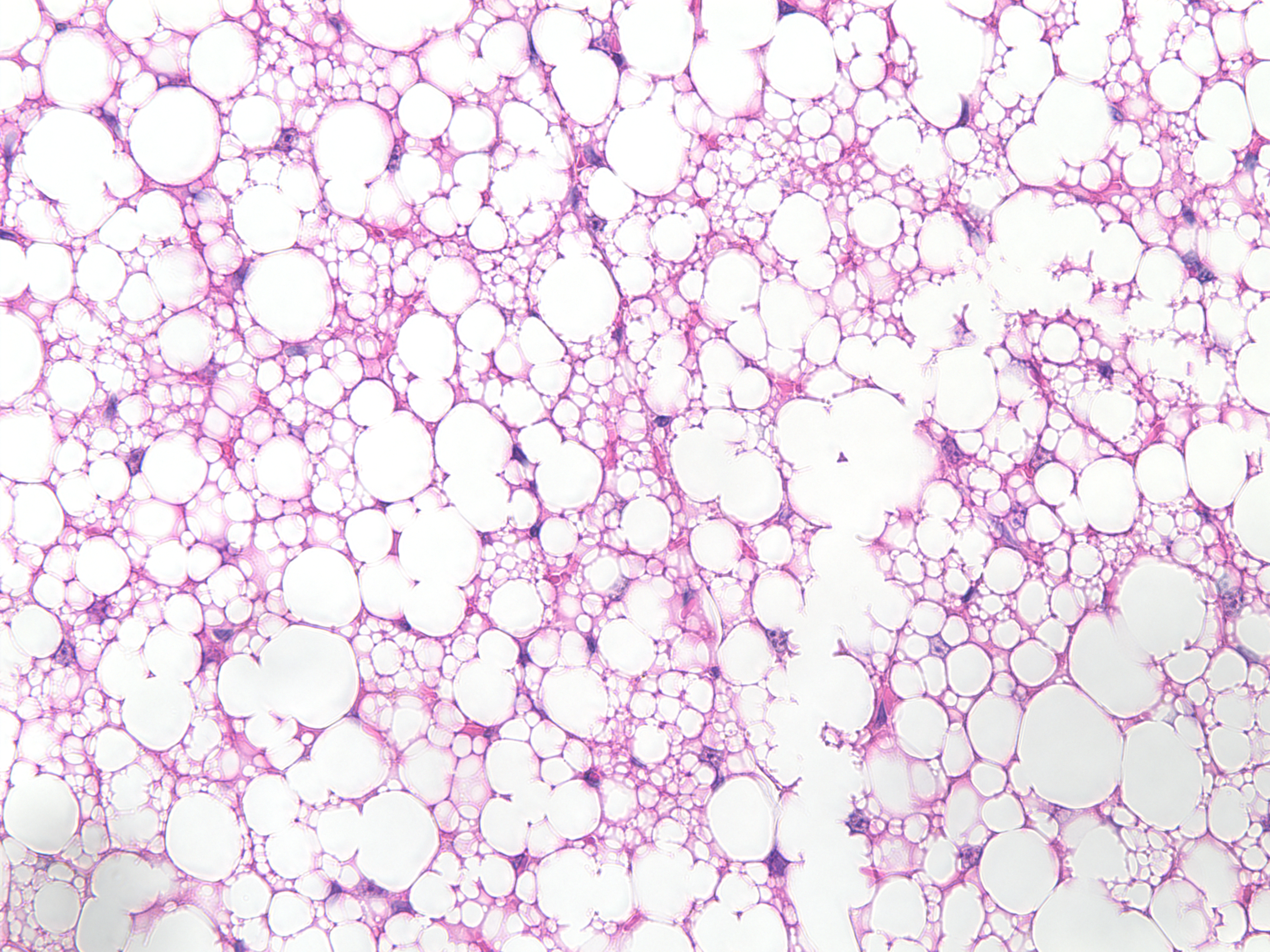

Supplement: Supplementary file 8 — Figure EV2 Source Data [file 44318_2024_196_MOESM8_ESM.zip › Figure EV2/Figure EV2-M/Demonstrated data/AAV Pcolce.tif]

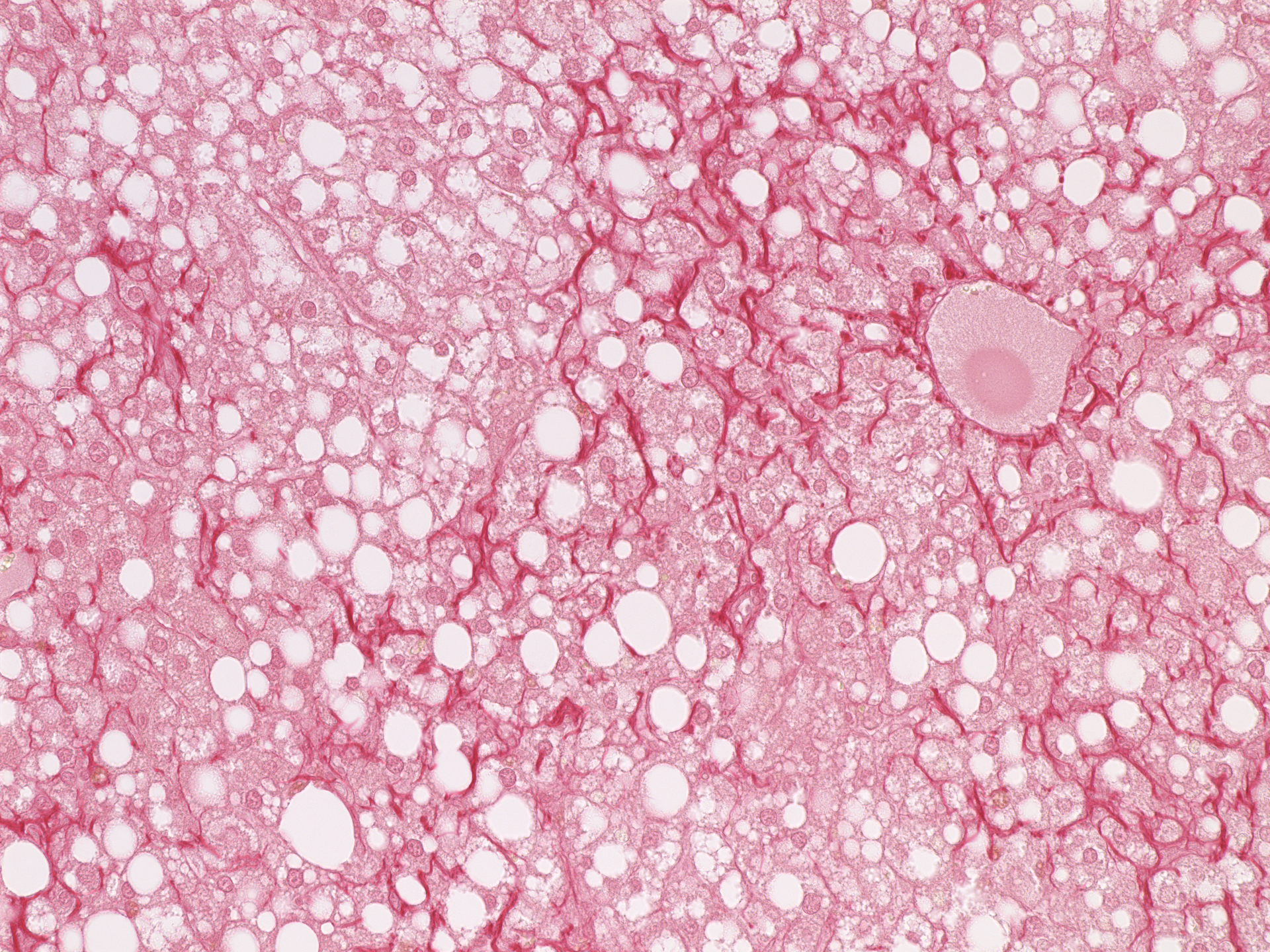

Supplement: Supplementary file 8 — Figure EV2 Source Data [file 44318_2024_196_MOESM8_ESM.zip › Figure EV2/Figure EV2-Q/Demonstrated image/HFD AAV-mPcolce/HFD-AAV-mPcolce-20x.tif]

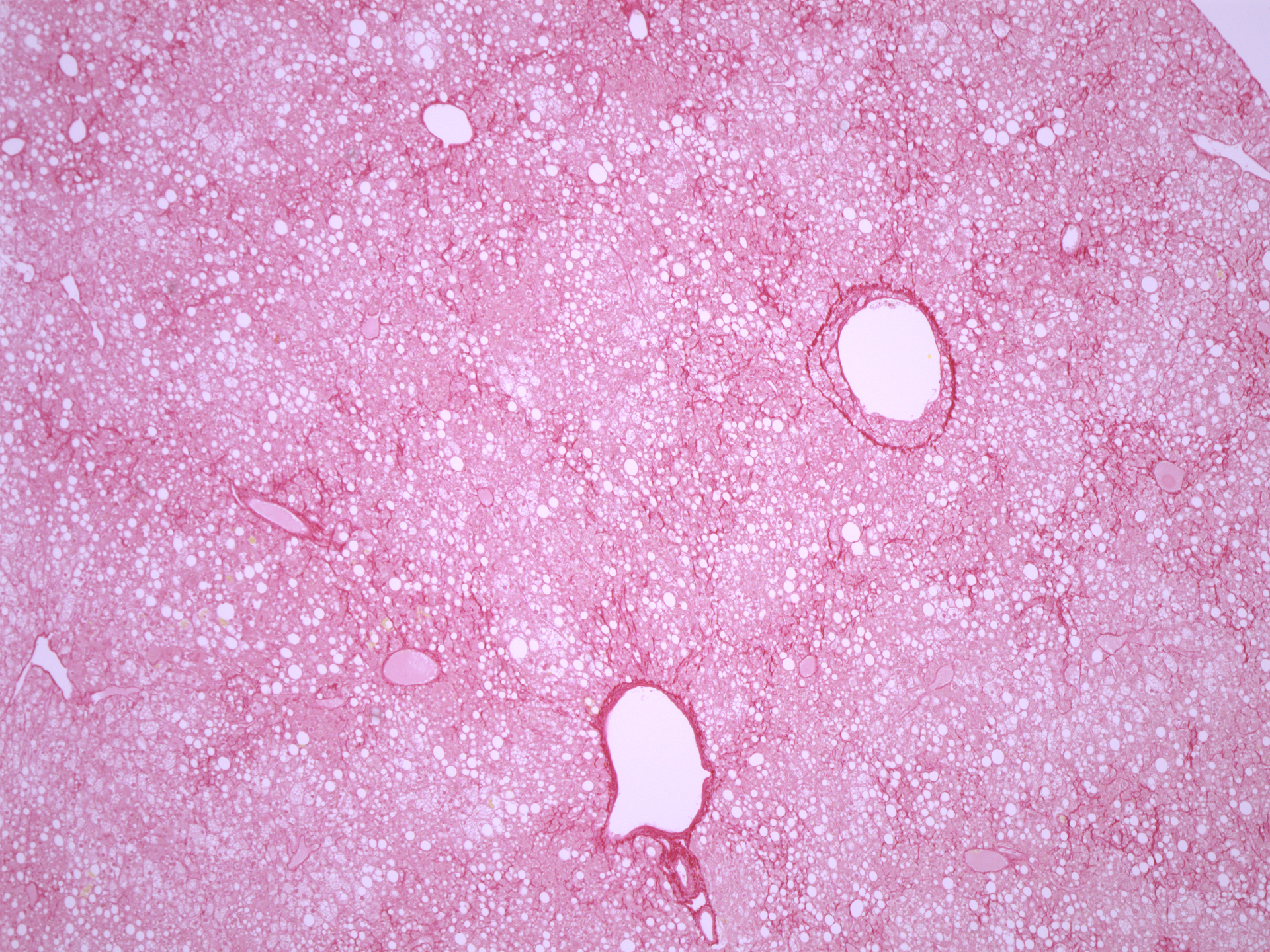

Supplement: Supplementary file 8 — Figure EV2 Source Data [file 44318_2024_196_MOESM8_ESM.zip › Figure EV2/Figure EV2-Q/Demonstrated image/HFD AAV-mPcolce/HFD-AAV-mPcolce-4x.tif]

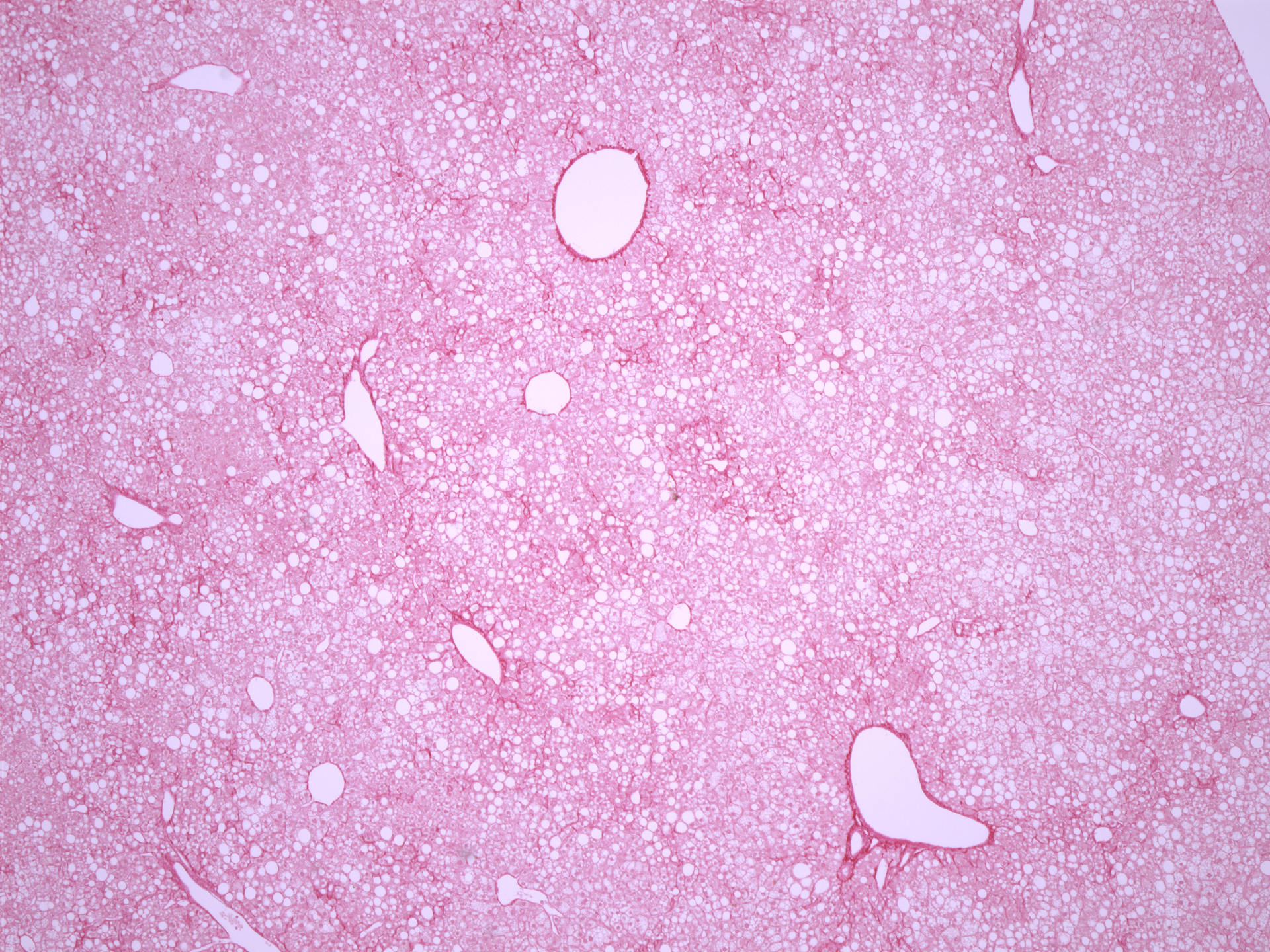

Supplement: Supplementary file 8 — Figure EV2 Source Data [file 44318_2024_196_MOESM8_ESM.zip › Figure EV2/Figure EV2-Q/Demonstrated image/HFD Mock/HFD-Mock-4x.tif]

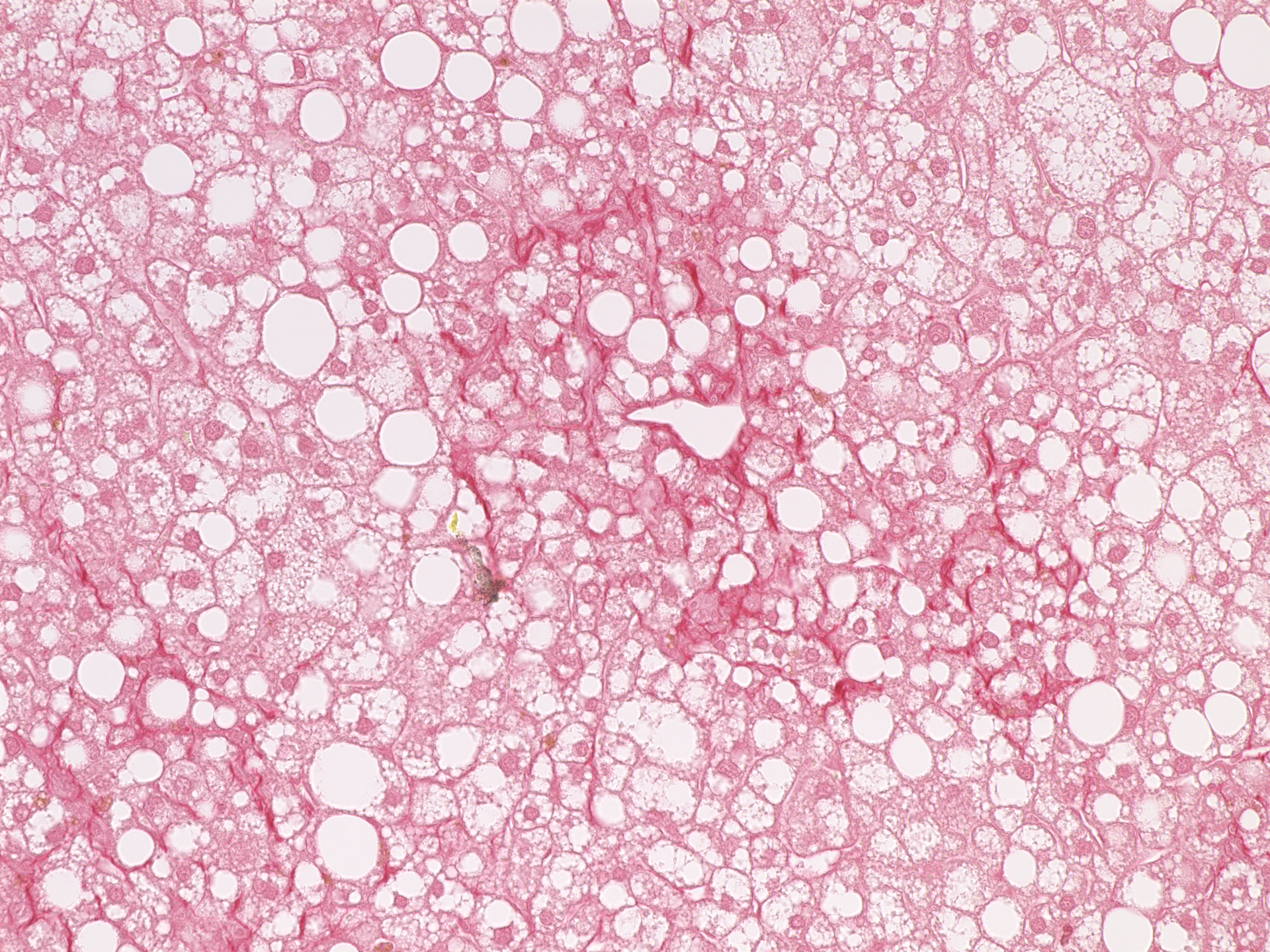

Supplement: Supplementary file 8 — Figure EV2 Source Data [file 44318_2024_196_MOESM8_ESM.zip › Figure EV2/Figure EV2-Q/Demonstrated image/HFD Mock/HFD-Mock-20x.tif]

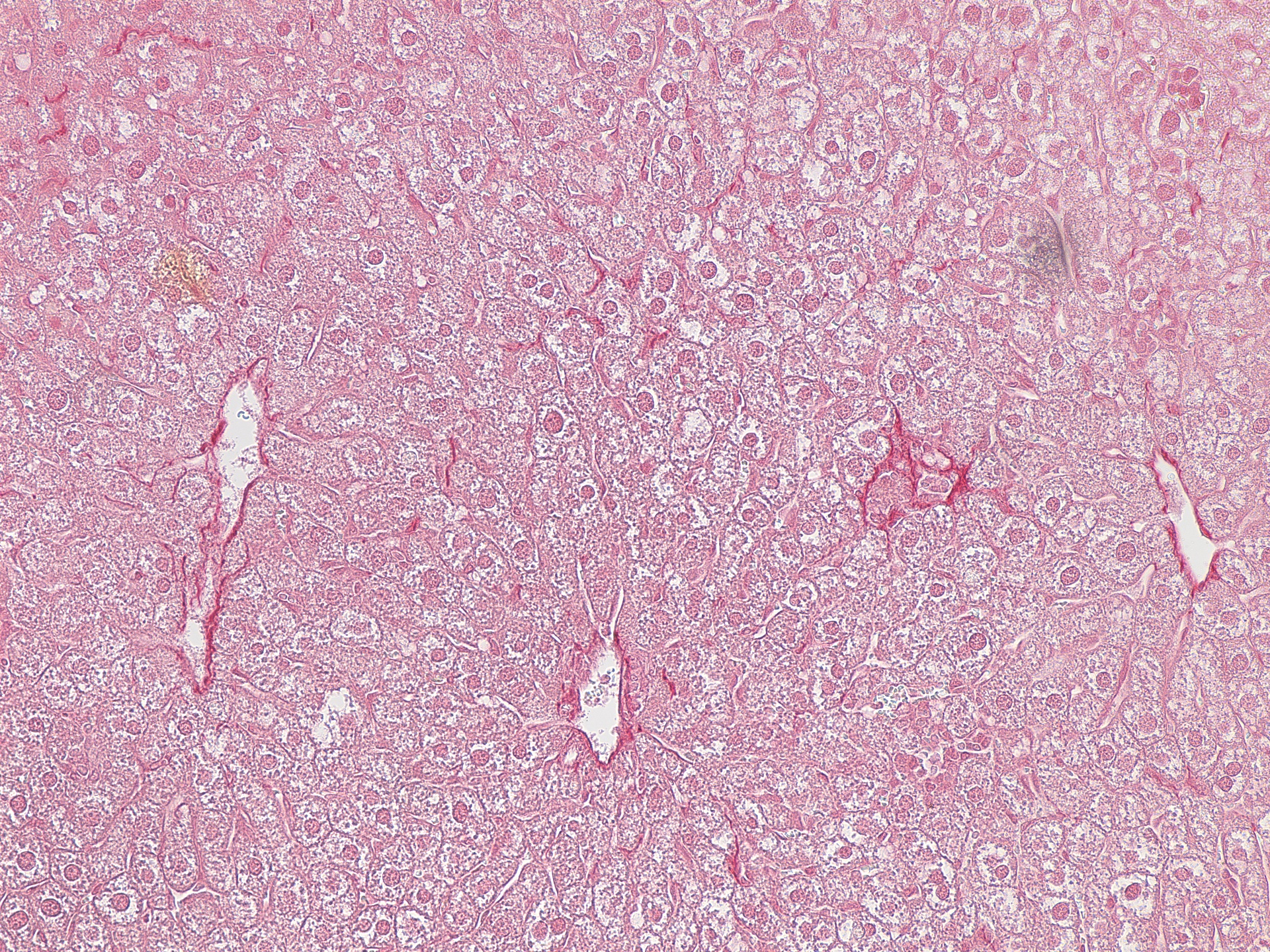

Supplement: Supplementary file 8 — Figure EV2 Source Data [file 44318_2024_196_MOESM8_ESM.zip › Figure EV2/Figure EV2-J/Quantificated image/NC AAV-mPcolce/no.1/NC-AAV-mPcolce-no.1-20x-4.jpg]

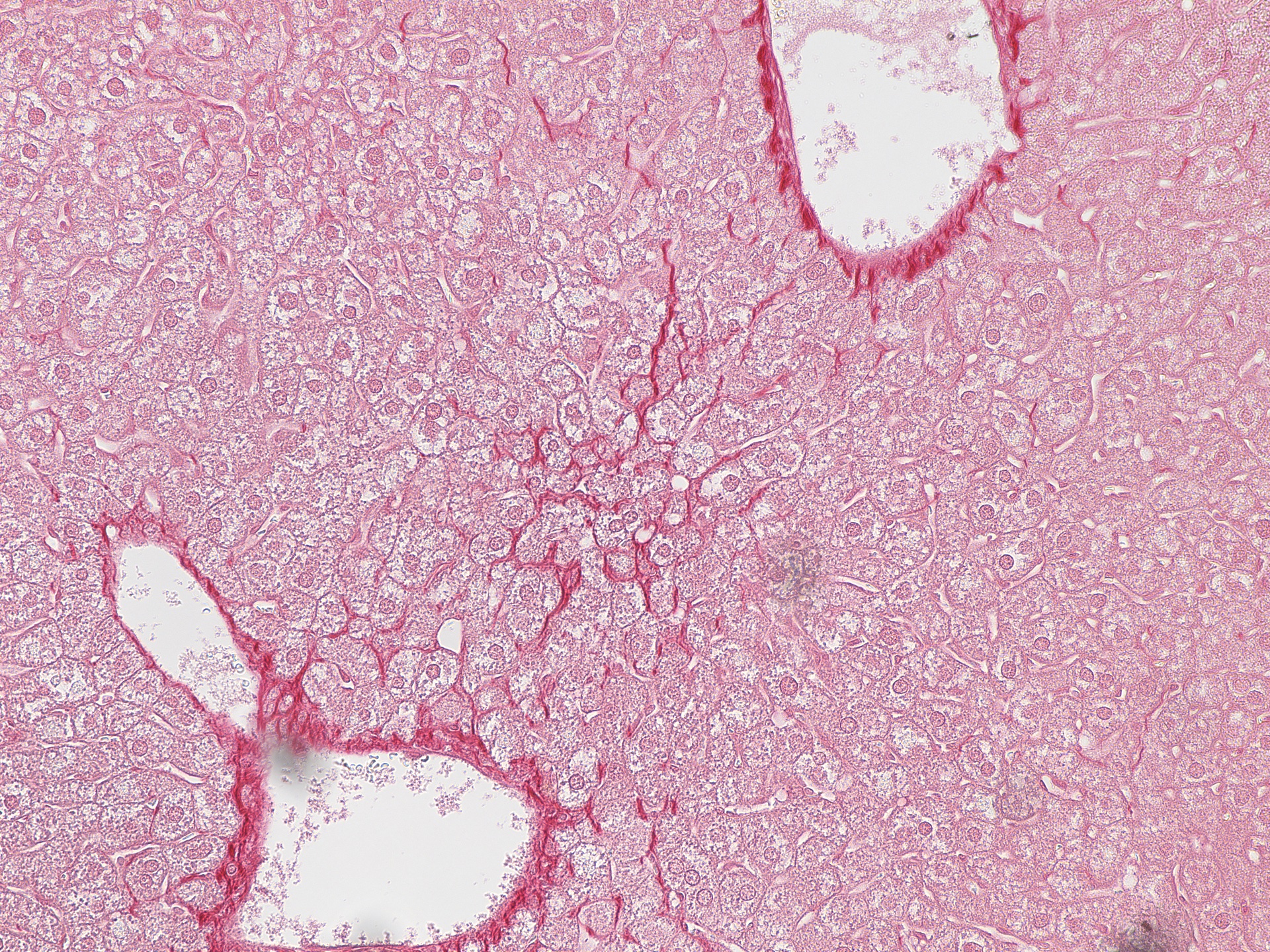

Supplement: Supplementary file 8 — Figure EV2 Source Data [file 44318_2024_196_MOESM8_ESM.zip › Figure EV2/Figure EV2-J/Quantificated image/NC AAV-mPcolce/no.1/NC-AAV-mPcolce-no.1-20x-5.jpg]

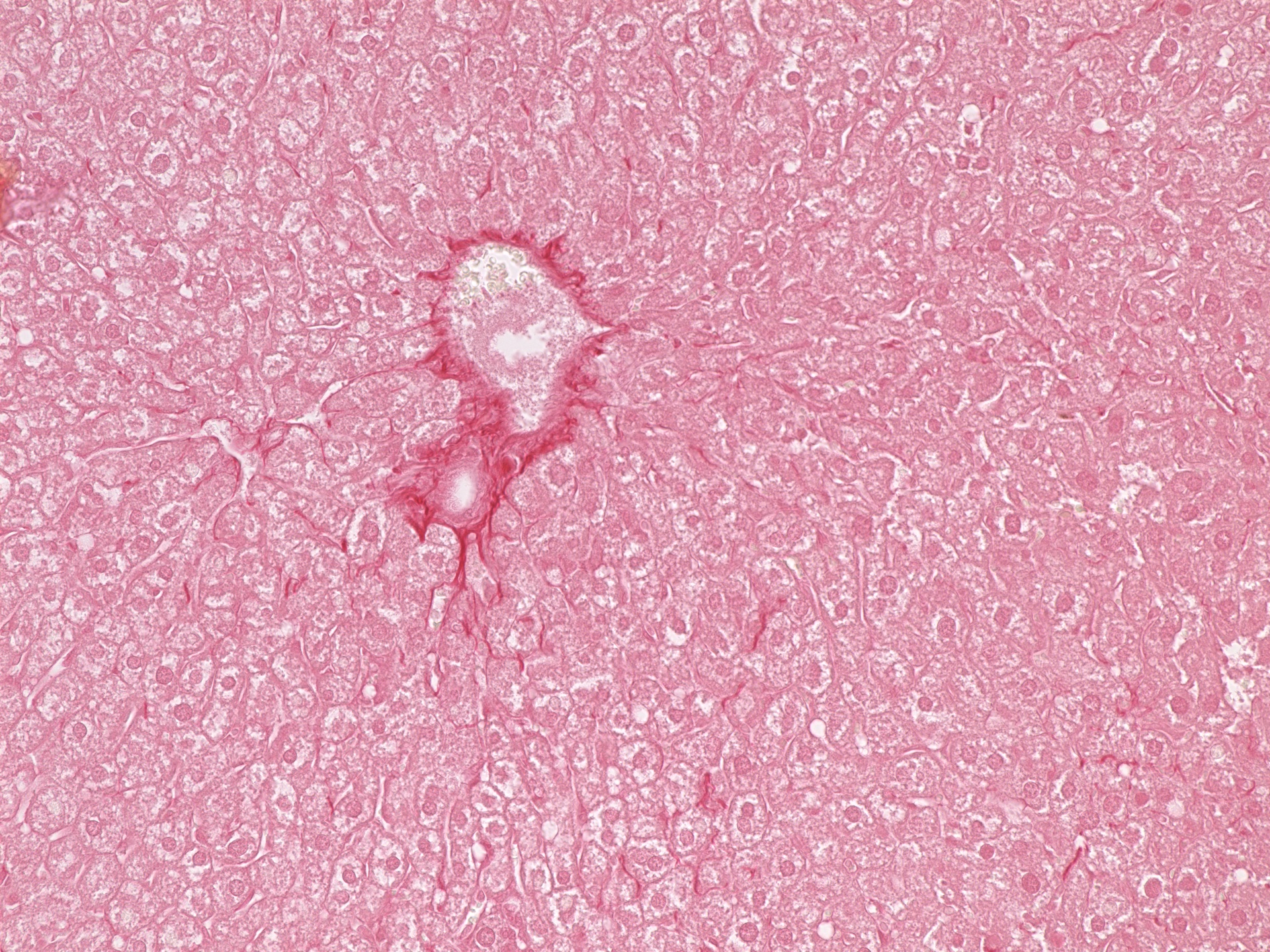

Supplement: Supplementary file 8 — Figure EV2 Source Data [file 44318_2024_196_MOESM8_ESM.zip › Figure EV2/Figure EV2-J/Quantificated image/NC AAV-mPcolce/no.1/NC-AAV-mPcolce-no.1-20x-2.jpg]

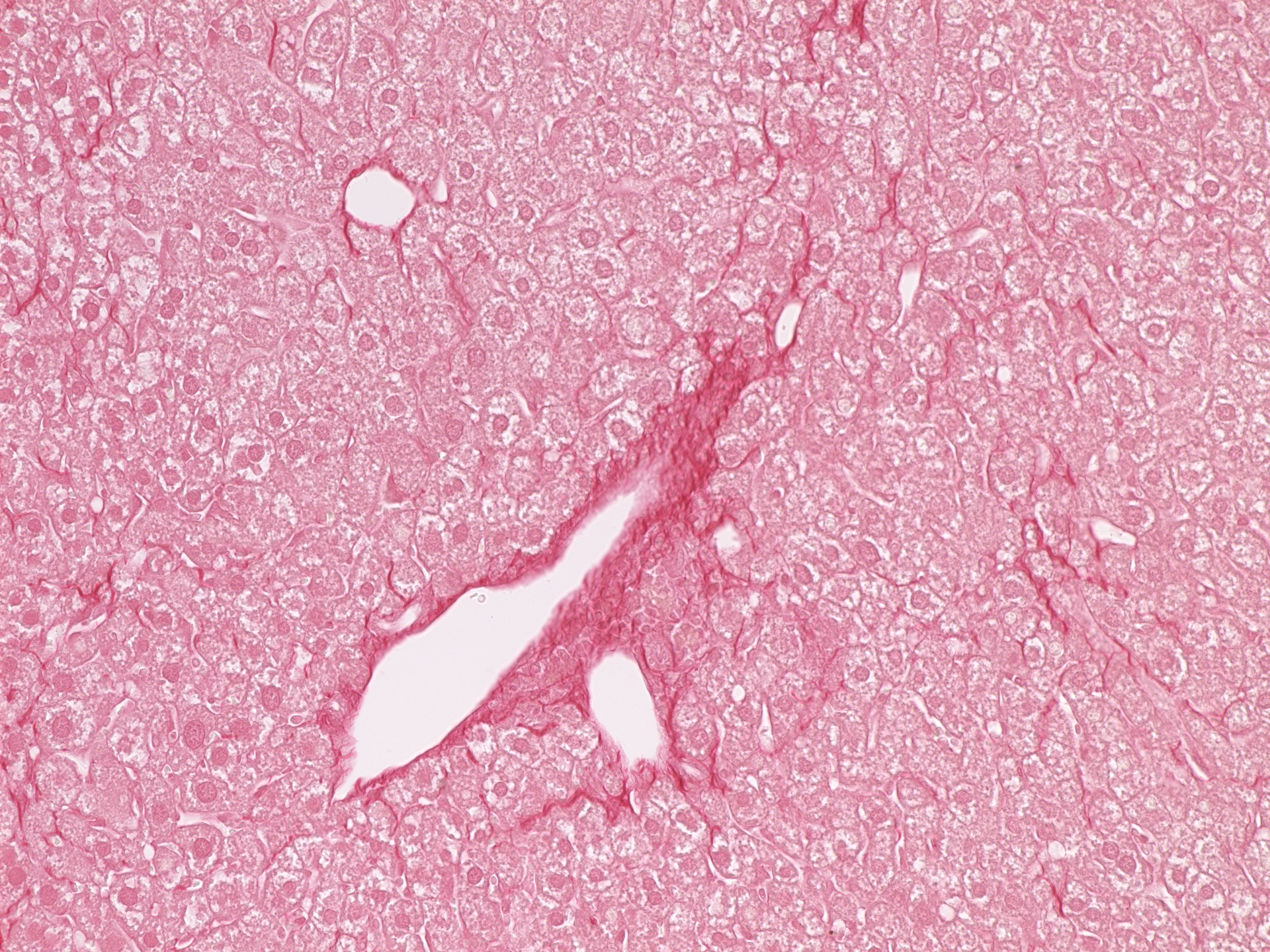

Supplement: Supplementary file 8 — Figure EV2 Source Data [file 44318_2024_196_MOESM8_ESM.zip › Figure EV2/Figure EV2-J/Quantificated image/NC AAV-mPcolce/no.1/NC-AAV-mPcolce-no.1-20x-3.jpg]

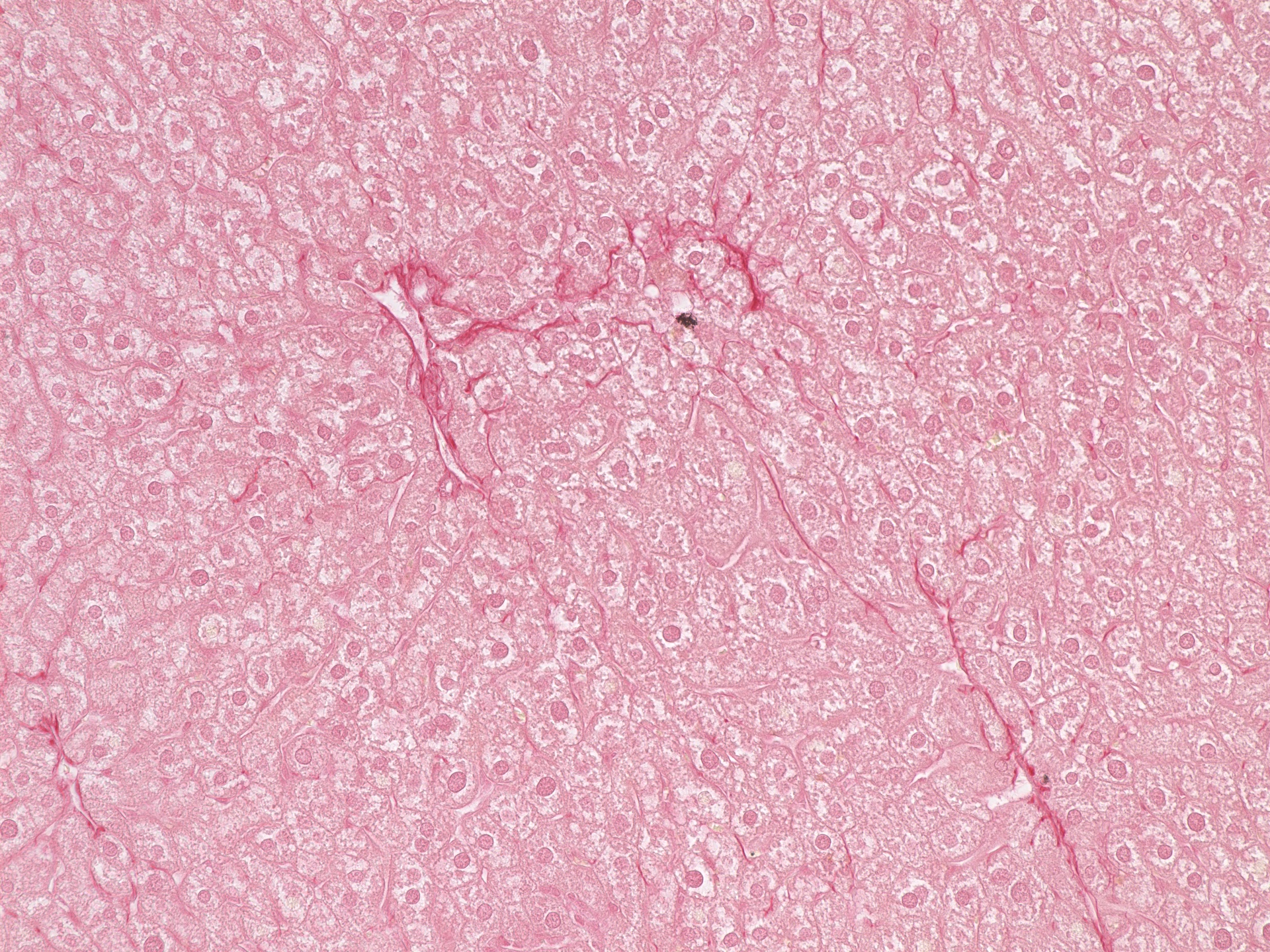

Supplement: Supplementary file 8 — Figure EV2 Source Data [file 44318_2024_196_MOESM8_ESM.zip › Figure EV2/Figure EV2-J/Quantificated image/NC AAV-mPcolce/no.1/NC-AAV-mPcolce-no.1-20x-1.jpg]

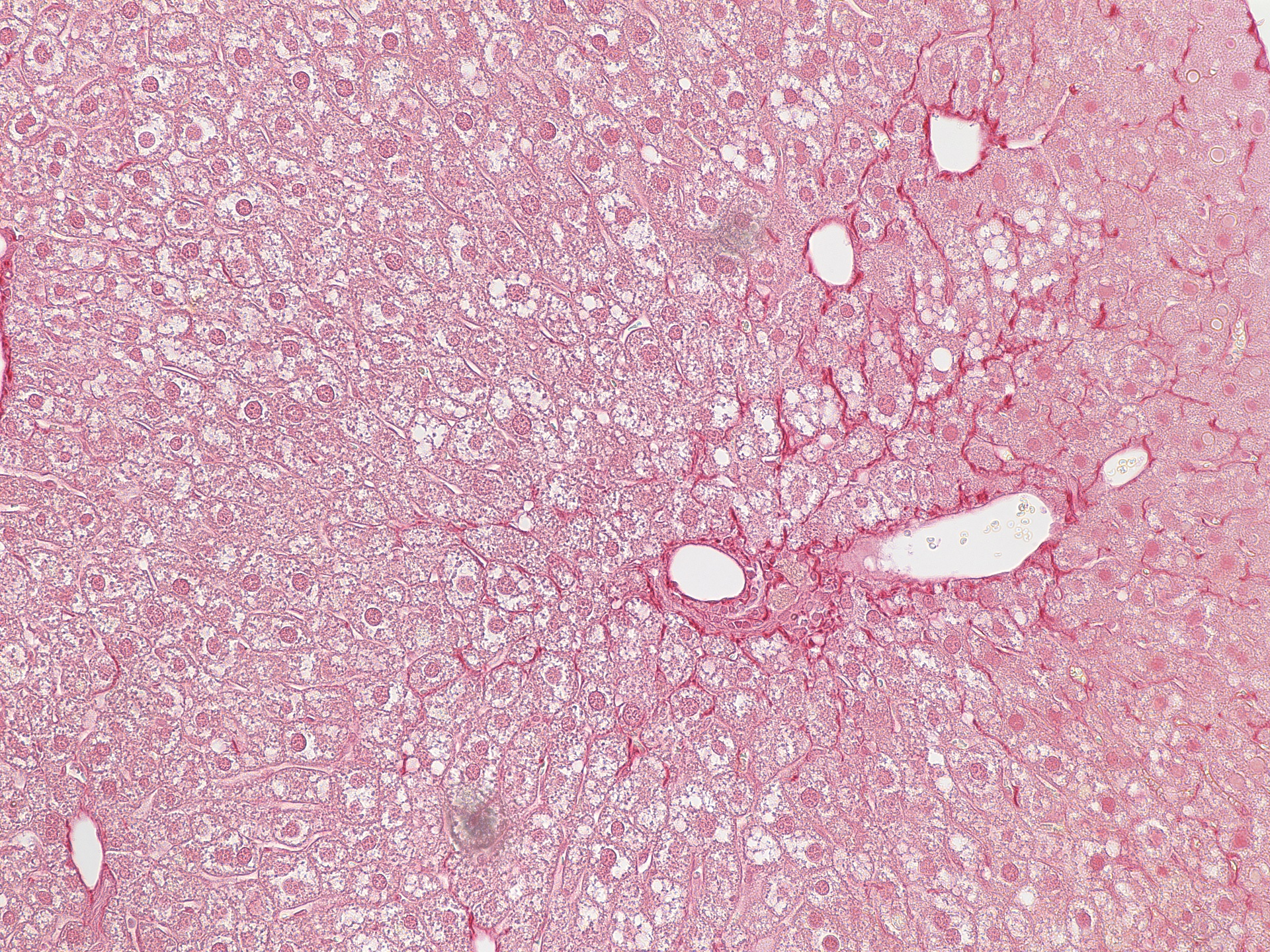

Supplement: Supplementary file 8 — Figure EV2 Source Data [file 44318_2024_196_MOESM8_ESM.zip › Figure EV2/Figure EV2-J/Quantificated image/NC AAV-mPcolce/no.3/NC-AAV-mPcolce-no.3-20x-5.jpg]

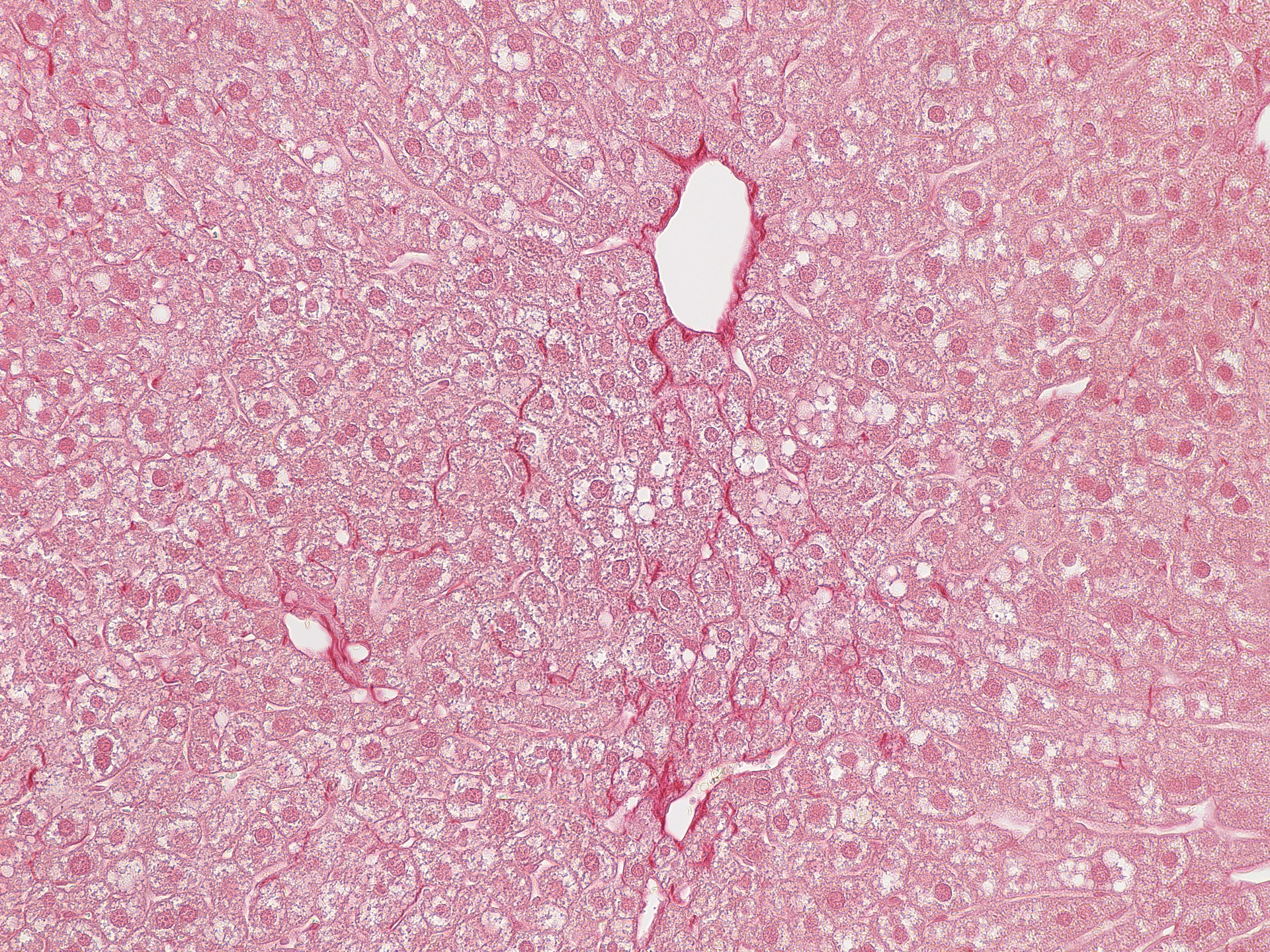

Supplement: Supplementary file 8 — Figure EV2 Source Data [file 44318_2024_196_MOESM8_ESM.zip › Figure EV2/Figure EV2-J/Quantificated image/NC AAV-mPcolce/no.3/NC-AAV-mPcolce-no.3-20x-4.jpg]

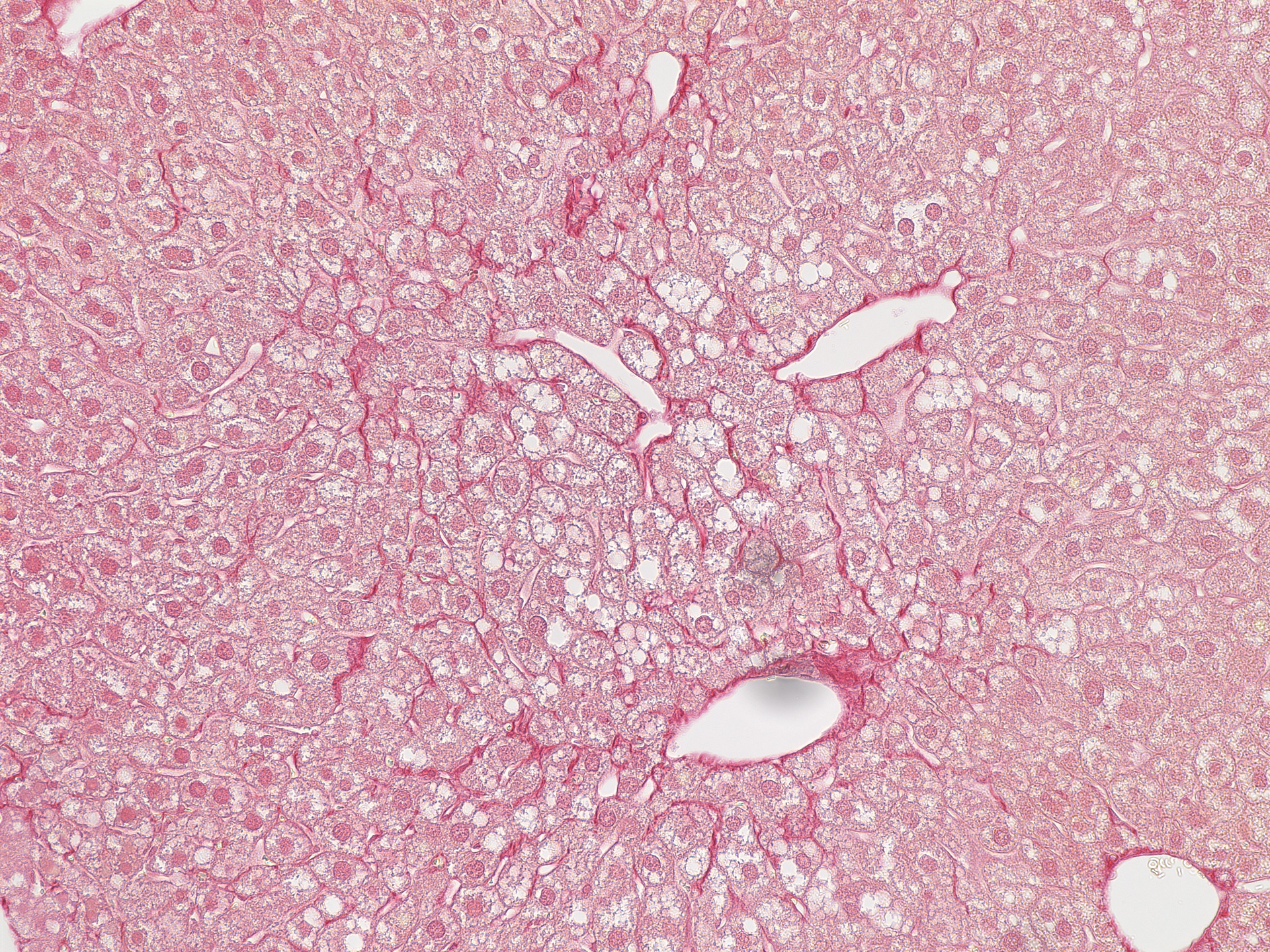

Supplement: Supplementary file 8 — Figure EV2 Source Data [file 44318_2024_196_MOESM8_ESM.zip › Figure EV2/Figure EV2-J/Quantificated image/NC AAV-mPcolce/no.3/NC-AAV-mPcolce-no.3-20x-3.jpg]

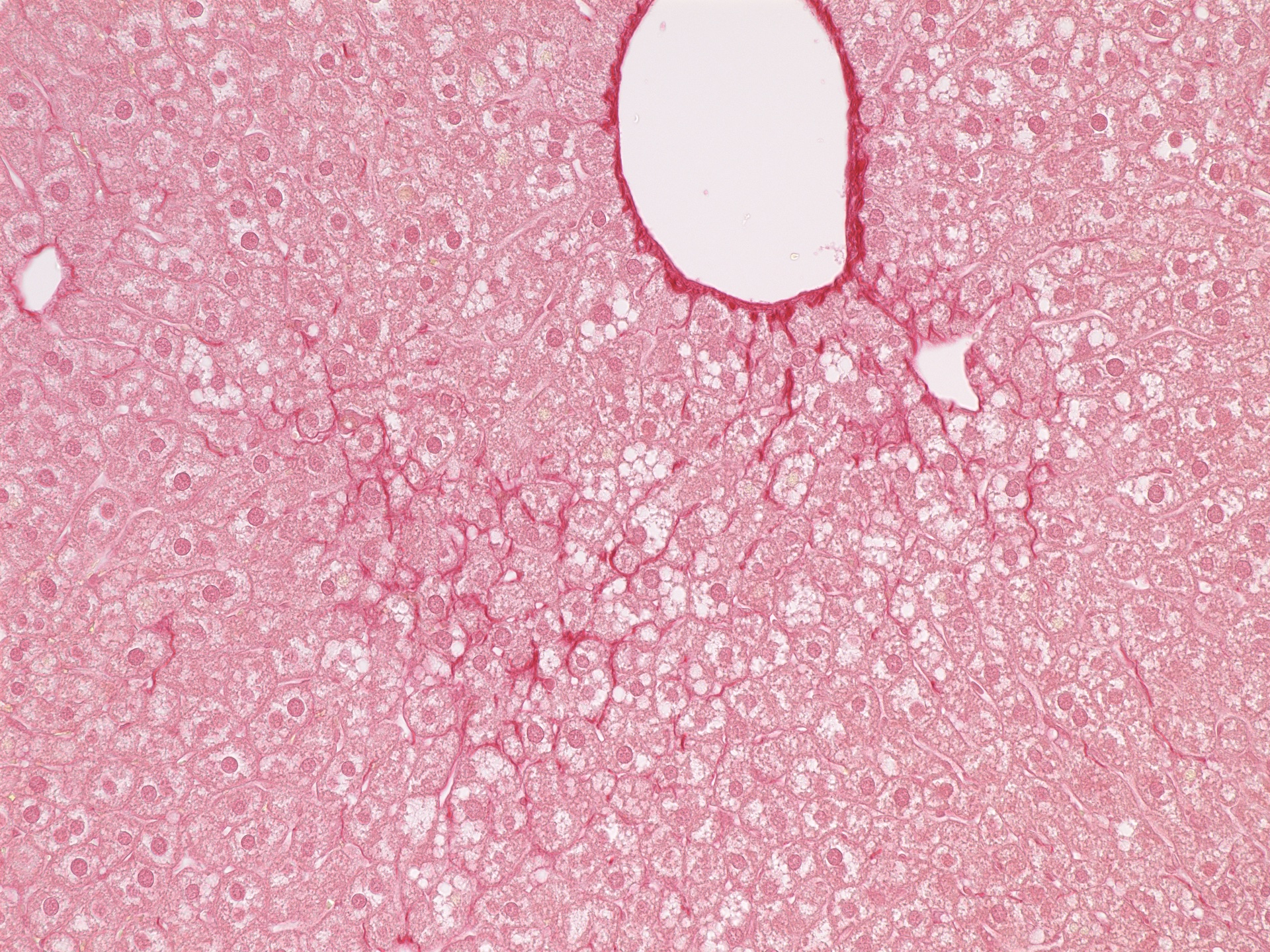

Supplement: Supplementary file 8 — Figure EV2 Source Data [file 44318_2024_196_MOESM8_ESM.zip › Figure EV2/Figure EV2-J/Quantificated image/NC AAV-mPcolce/no.3/NC-AAV-mPcolce-no.3-20x-2.jpg]

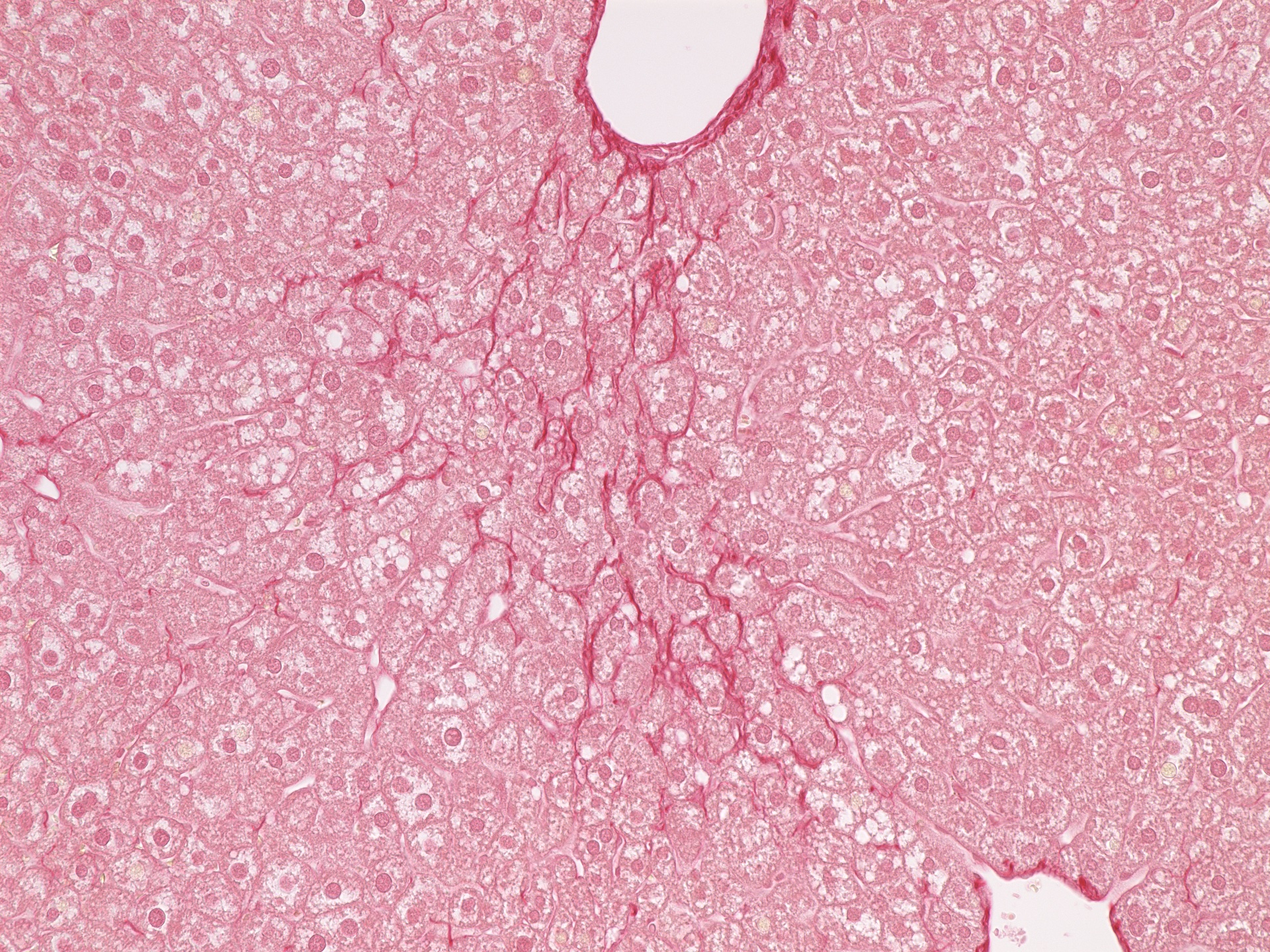

Supplement: Supplementary file 8 — Figure EV2 Source Data [file 44318_2024_196_MOESM8_ESM.zip › Figure EV2/Figure EV2-J/Quantificated image/NC AAV-mPcolce/no.3/NC-AAV-mPcolce-no.3-20x-1.jpg]

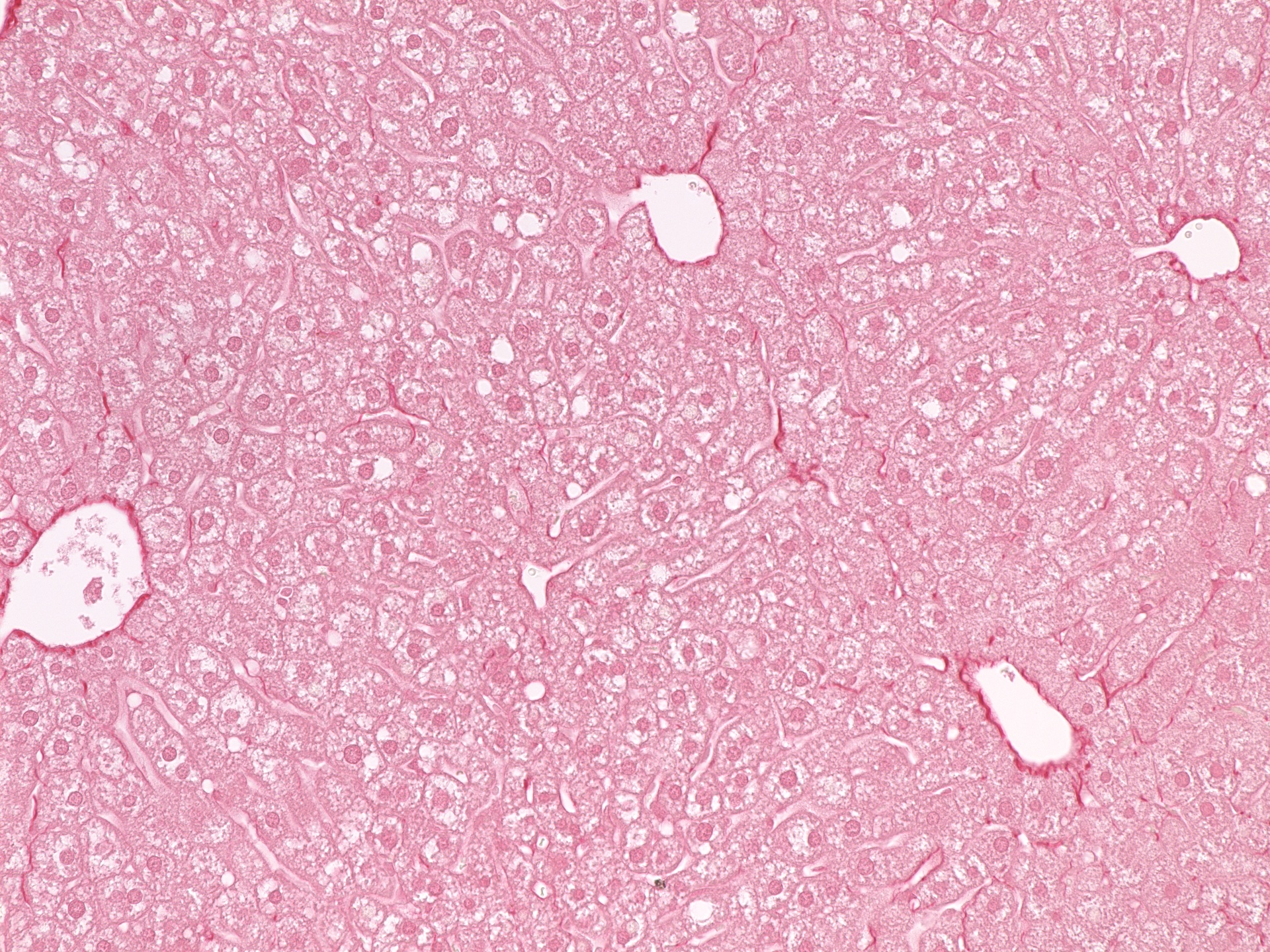

Supplement: Supplementary file 8 — Figure EV2 Source Data [file 44318_2024_196_MOESM8_ESM.zip › Figure EV2/Figure EV2-J/Quantificated image/NC AAV-mPcolce/no.4/NC-AAV-mPcolce-no.4-20x-2.jpg]

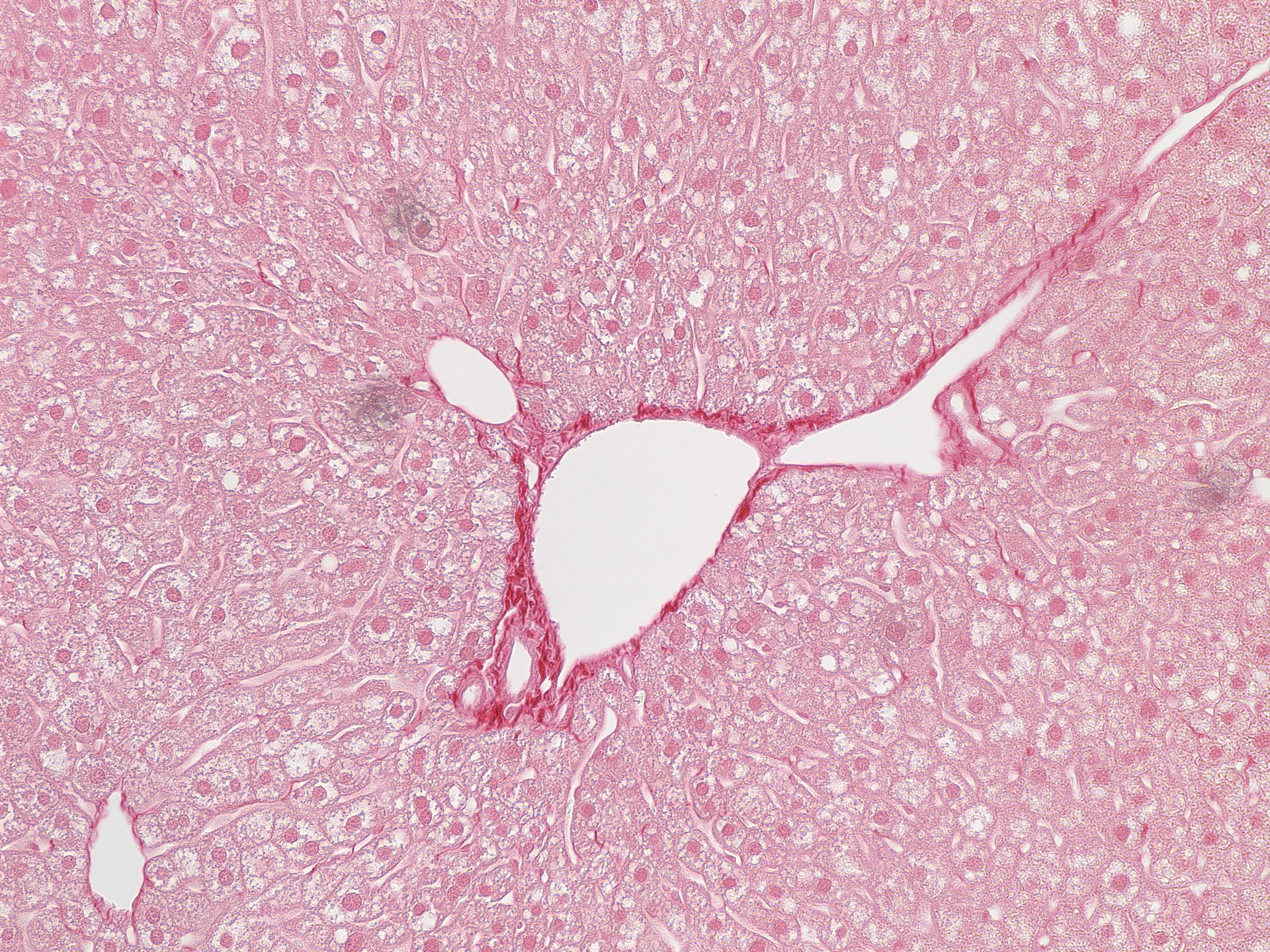

Supplement: Supplementary file 8 — Figure EV2 Source Data [file 44318_2024_196_MOESM8_ESM.zip › Figure EV2/Figure EV2-J/Quantificated image/NC AAV-mPcolce/no.4/NC-AAV-mPcolce-no.4-20x-3.jpg]

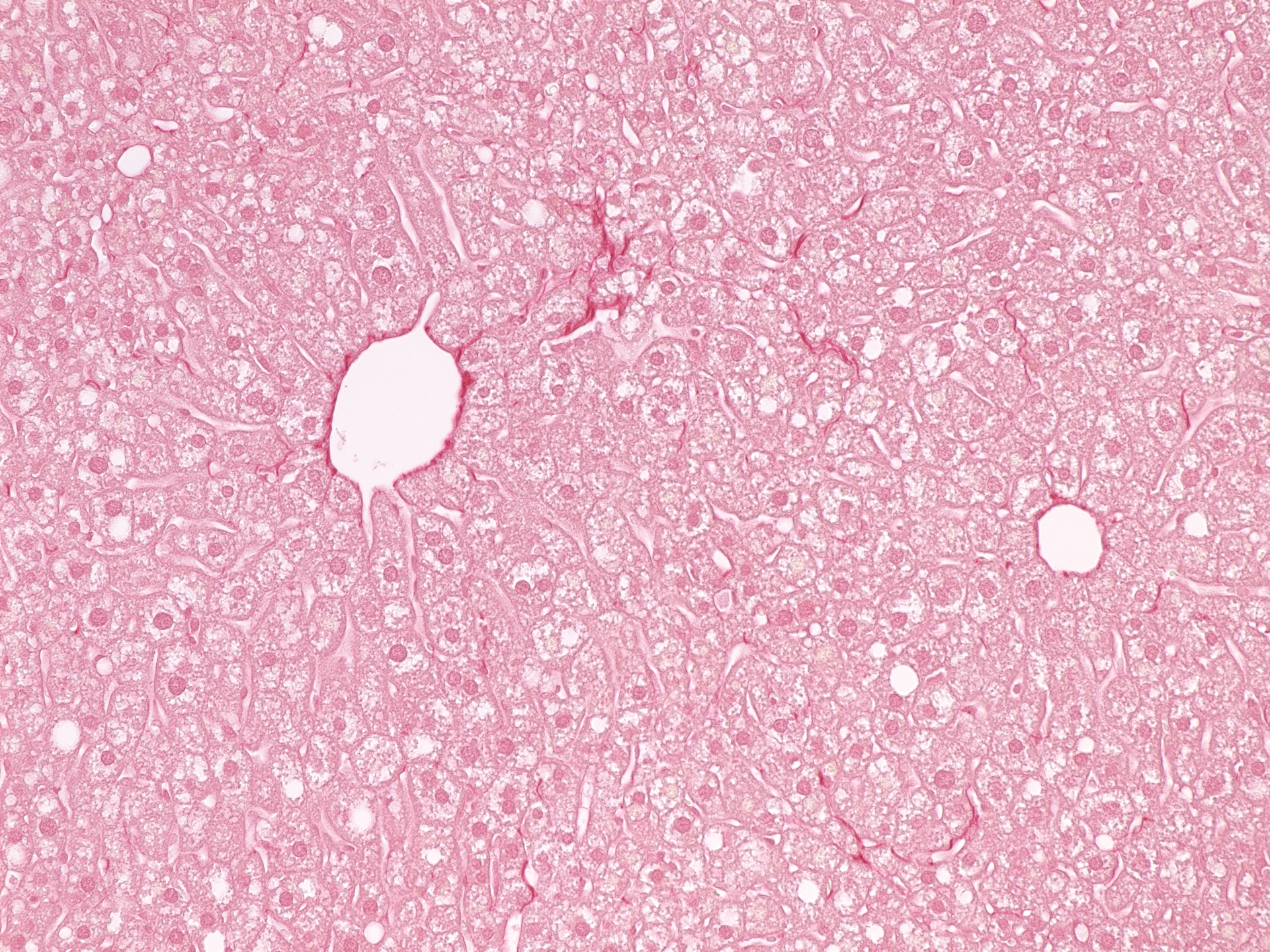

Supplement: Supplementary file 8 — Figure EV2 Source Data [file 44318_2024_196_MOESM8_ESM.zip › Figure EV2/Figure EV2-J/Quantificated image/NC AAV-mPcolce/no.4/NC-AAV-mPcolce-no.4-20x-1.jpg]

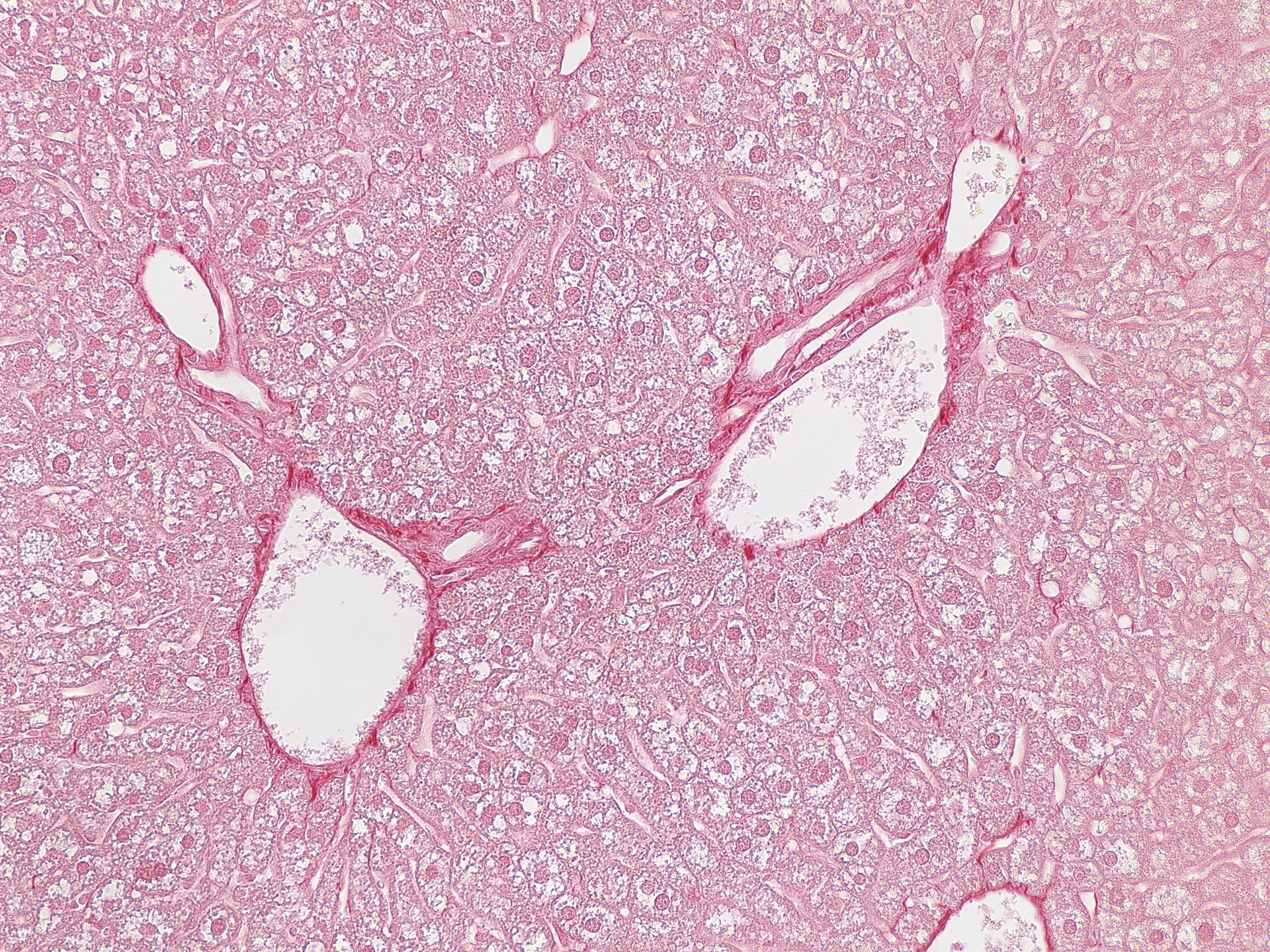

Supplement: Supplementary file 8 — Figure EV2 Source Data [file 44318_2024_196_MOESM8_ESM.zip › Figure EV2/Figure EV2-J/Quantificated image/NC AAV-mPcolce/no.4/NC-AAV-mPcolce-no.4-20x-4.jpg]

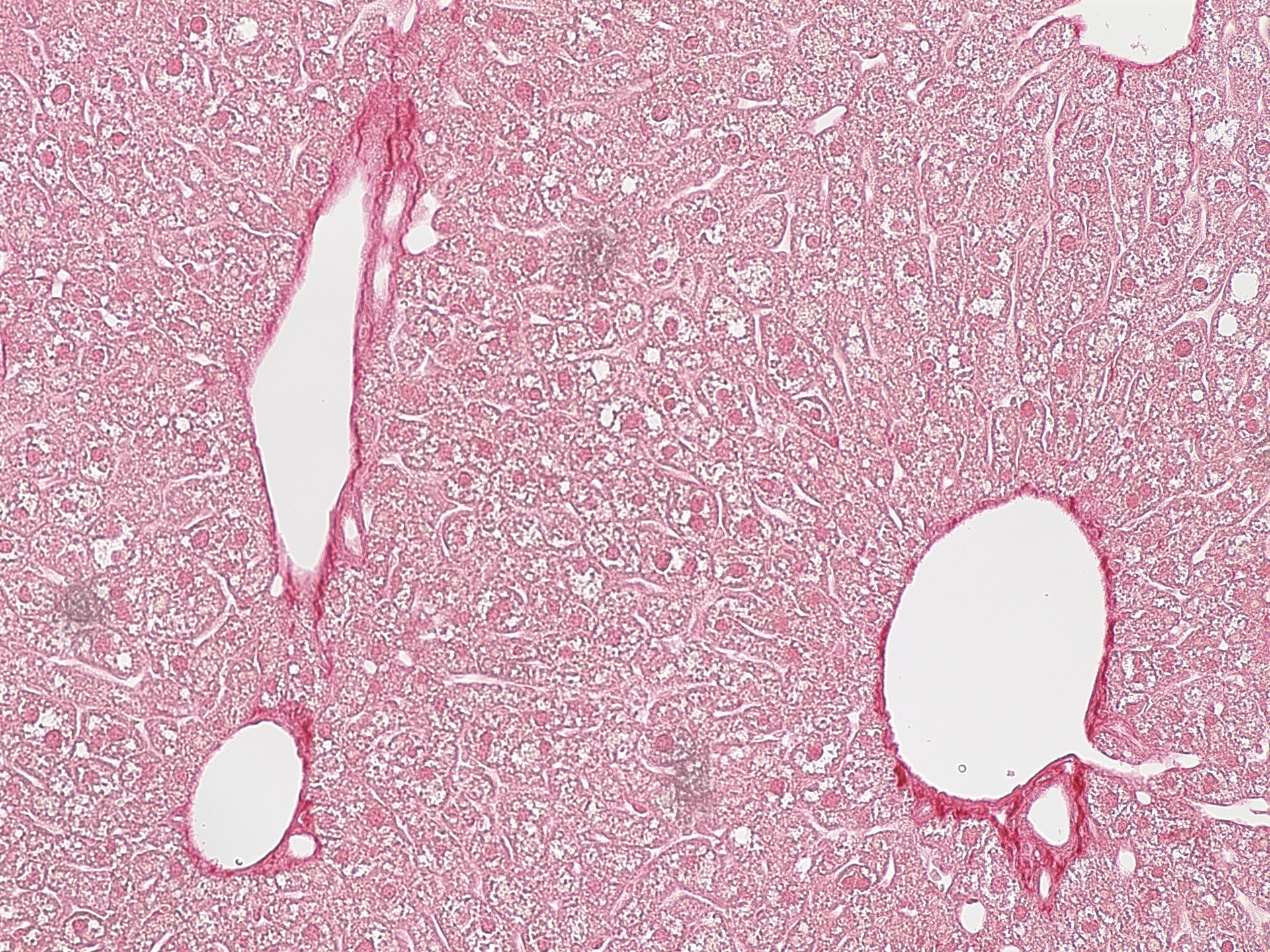

Supplement: Supplementary file 8 — Figure EV2 Source Data [file 44318_2024_196_MOESM8_ESM.zip › Figure EV2/Figure EV2-J/Quantificated image/NC AAV-mPcolce/no.4/NC-AAV-mPcolce-no.4-20x-5.jpg]

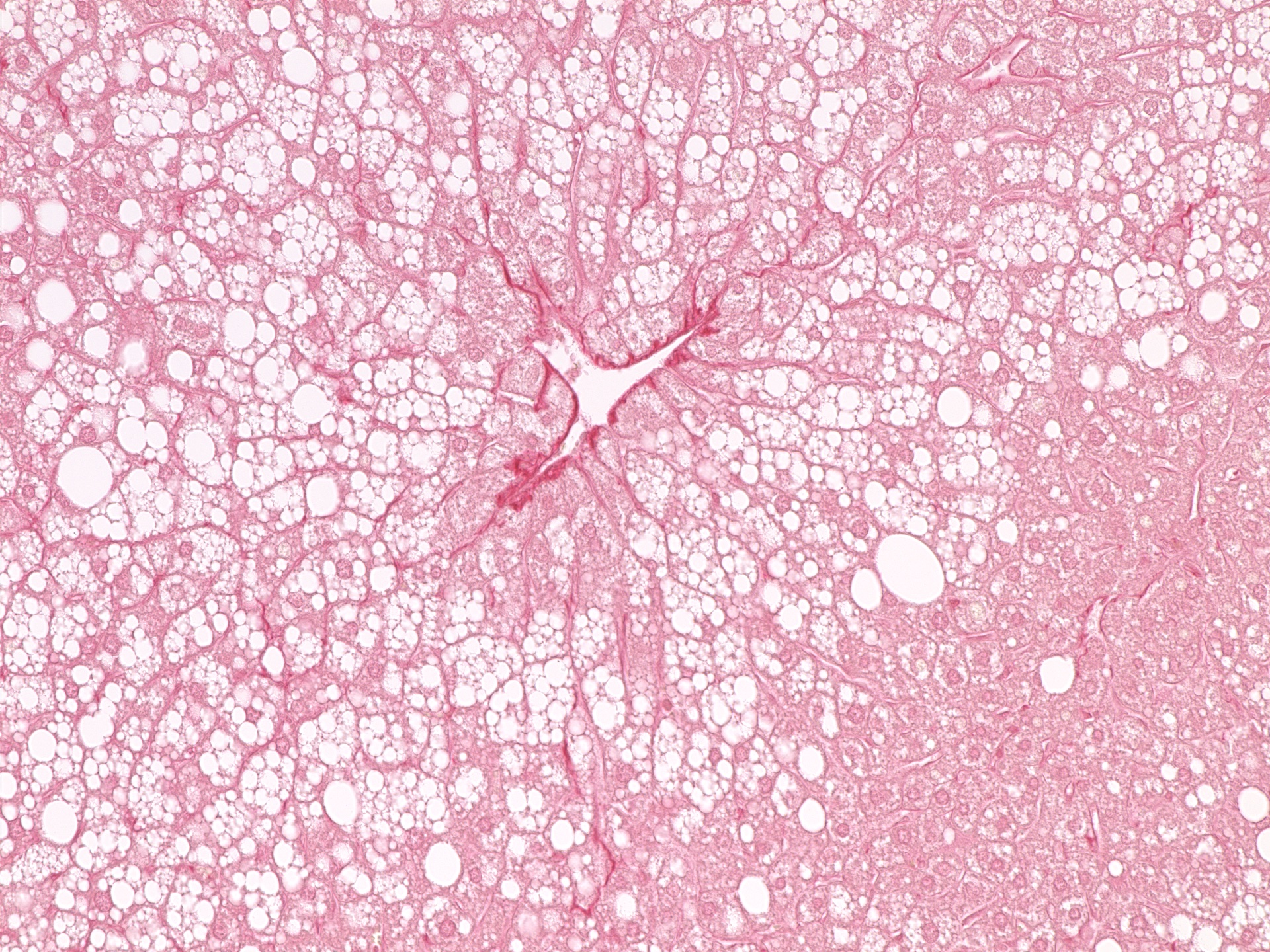

Supplement: Supplementary file 8 — Figure EV2 Source Data [file 44318_2024_196_MOESM8_ESM.zip › Figure EV2/Figure EV2-J/Quantificated image/NC AAV-mPcolce/no.5/NC-AAV-mPcolce-no.5-20x-2.jpg]

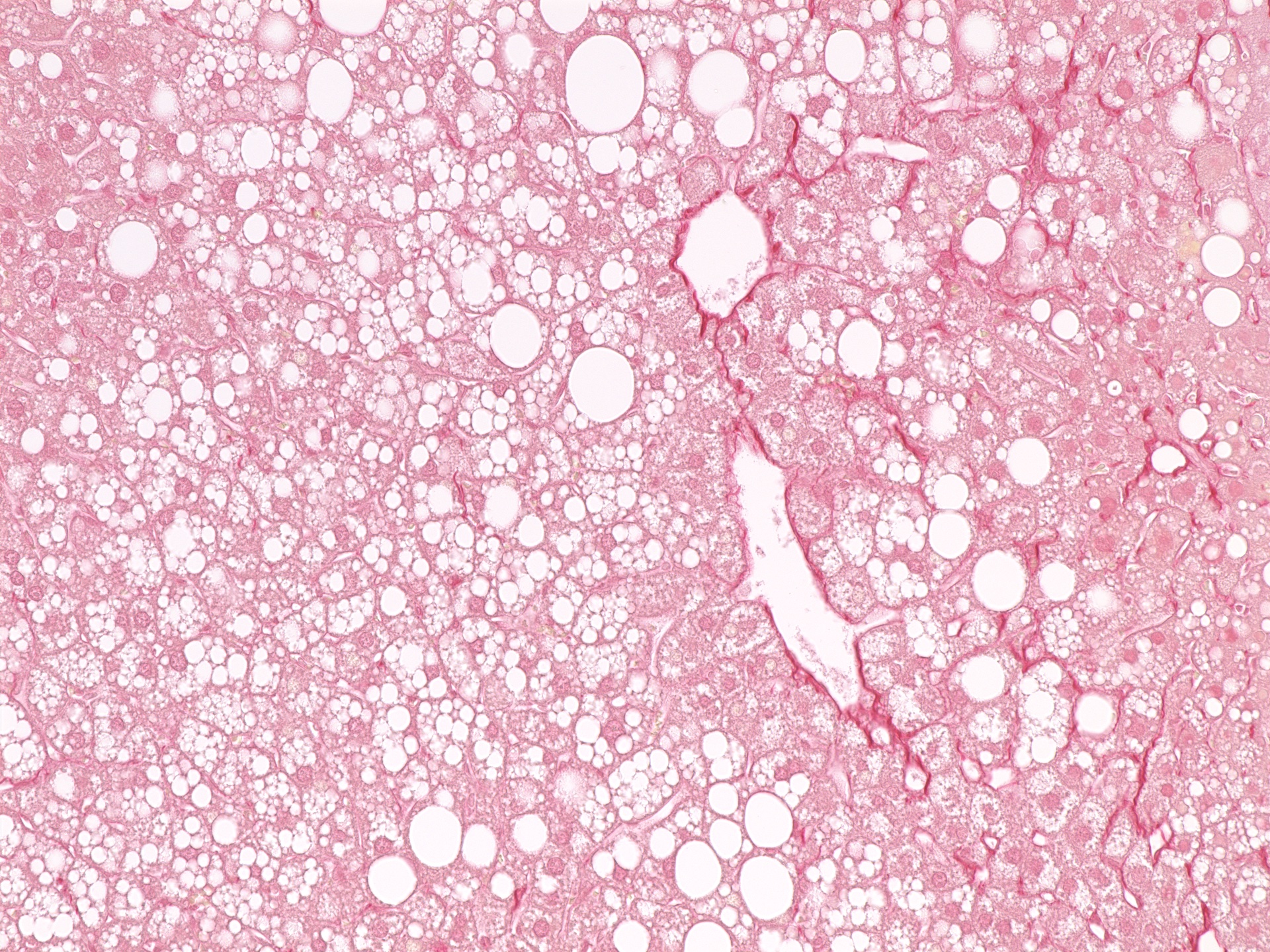

Supplement: Supplementary file 8 — Figure EV2 Source Data [file 44318_2024_196_MOESM8_ESM.zip › Figure EV2/Figure EV2-J/Quantificated image/NC AAV-mPcolce/no.5/NC-AAV-mPcolce-no.5-20x-3.jpg]

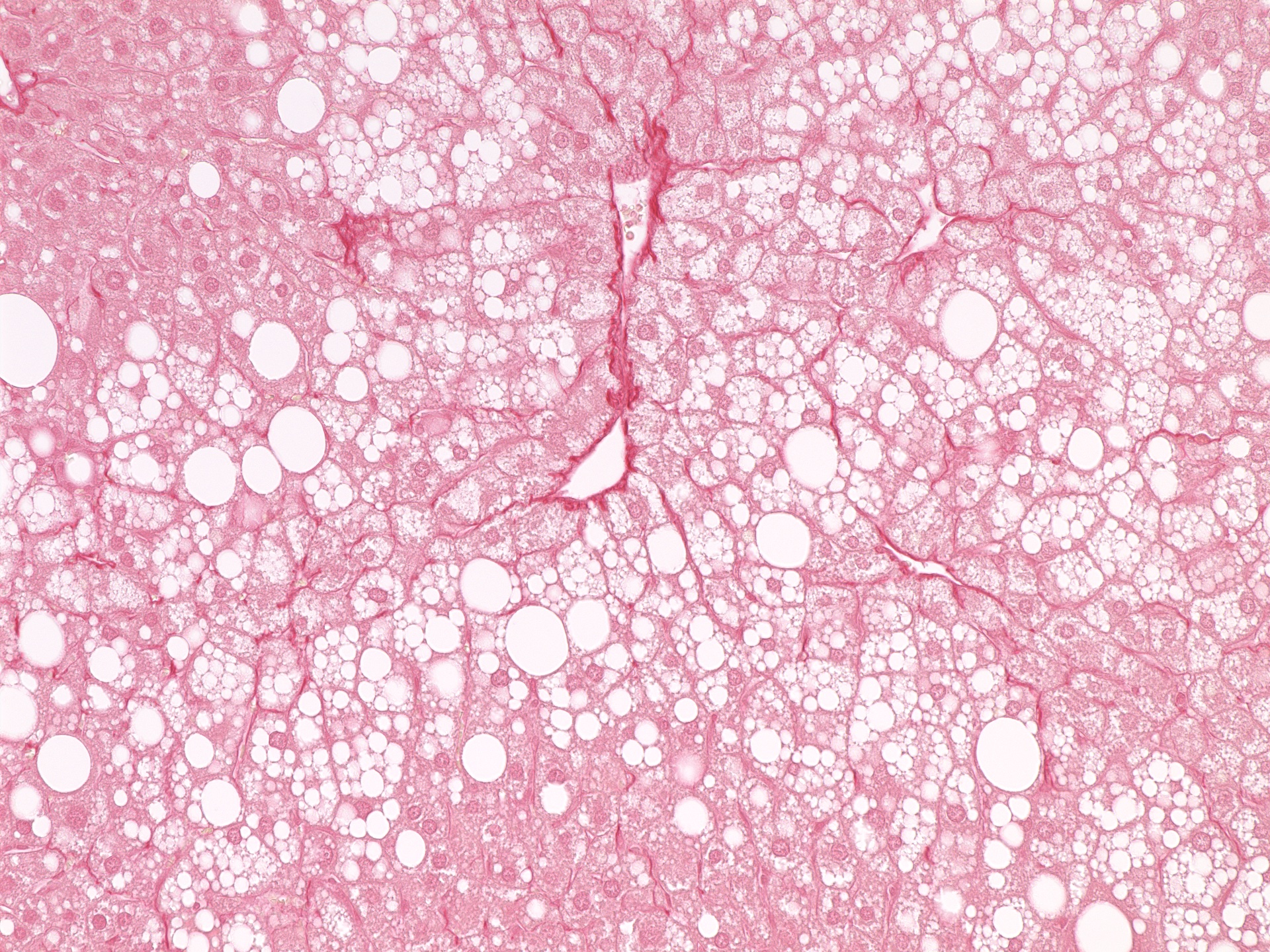

Supplement: Supplementary file 8 — Figure EV2 Source Data [file 44318_2024_196_MOESM8_ESM.zip › Figure EV2/Figure EV2-J/Quantificated image/NC AAV-mPcolce/no.5/NC-AAV-mPcolce-no.5-20x-1.jpg]

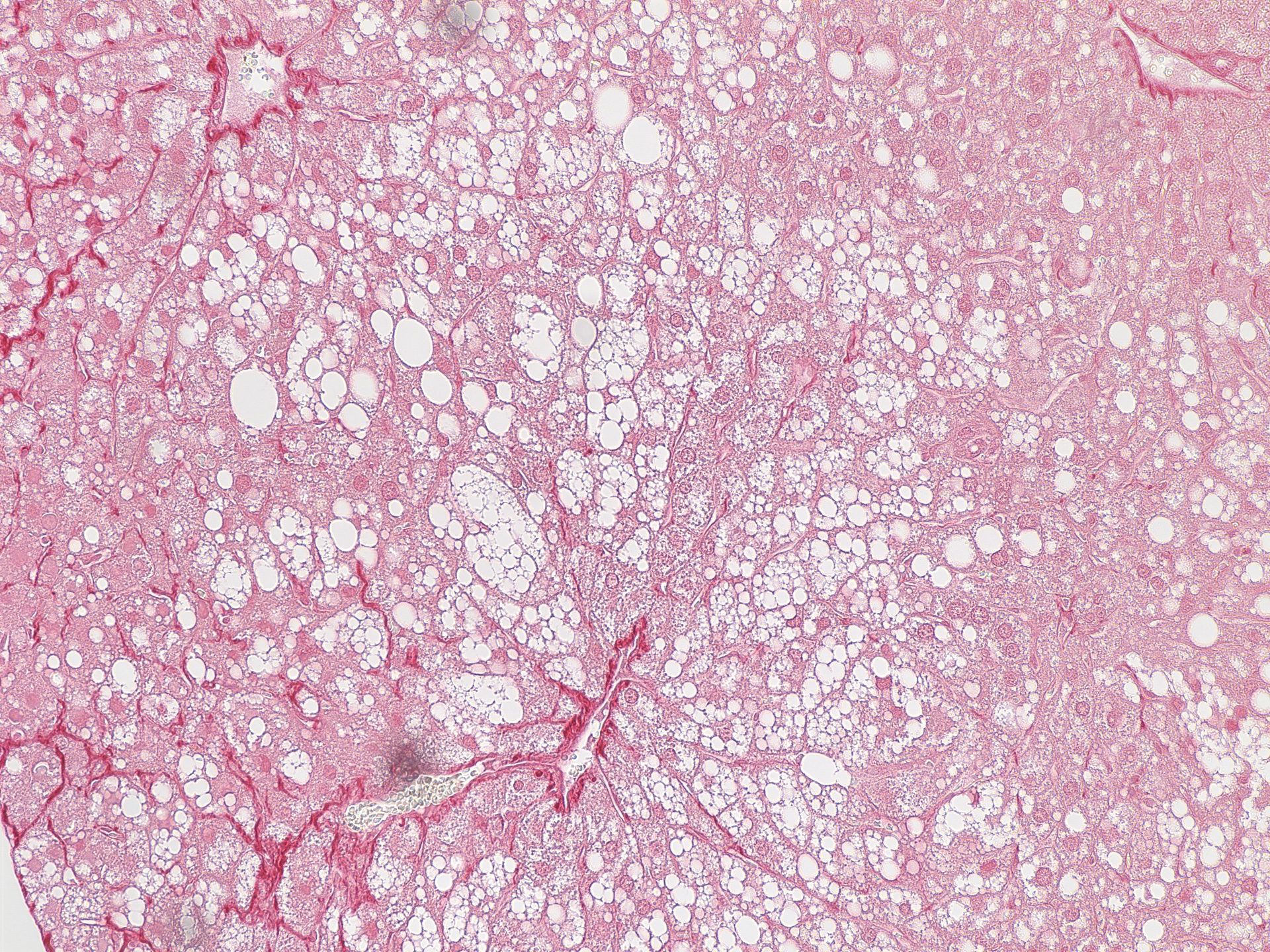

Supplement: Supplementary file 8 — Figure EV2 Source Data [file 44318_2024_196_MOESM8_ESM.zip › Figure EV2/Figure EV2-J/Quantificated image/NC AAV-mPcolce/no.5/NC-AAV-mPcolce-no.5-20x-4.jpg]

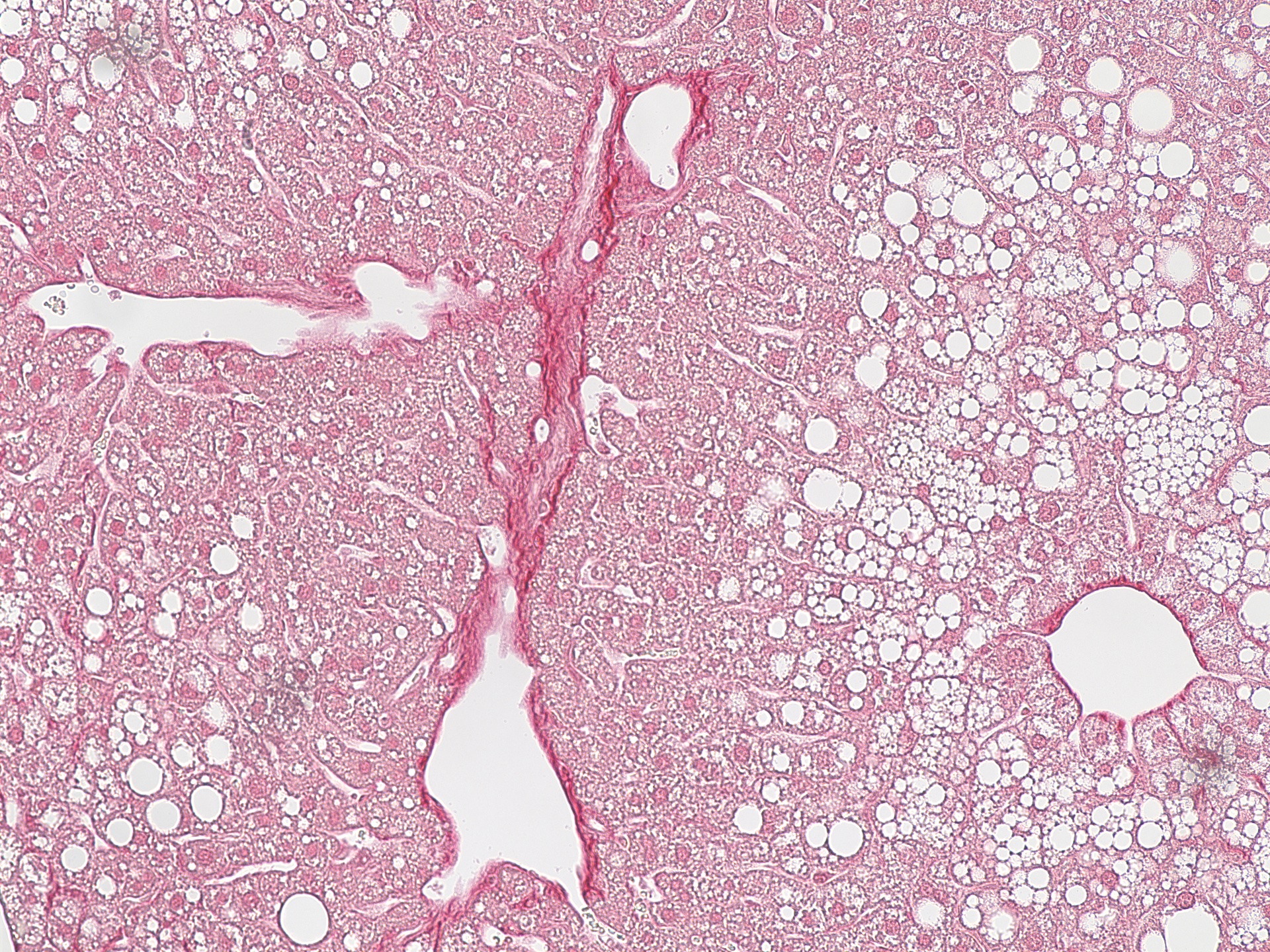

Supplement: Supplementary file 8 — Figure EV2 Source Data [file 44318_2024_196_MOESM8_ESM.zip › Figure EV2/Figure EV2-J/Quantificated image/NC AAV-mPcolce/no.5/NC-AAV-mPcolce-no.5-20x-5.jpg]

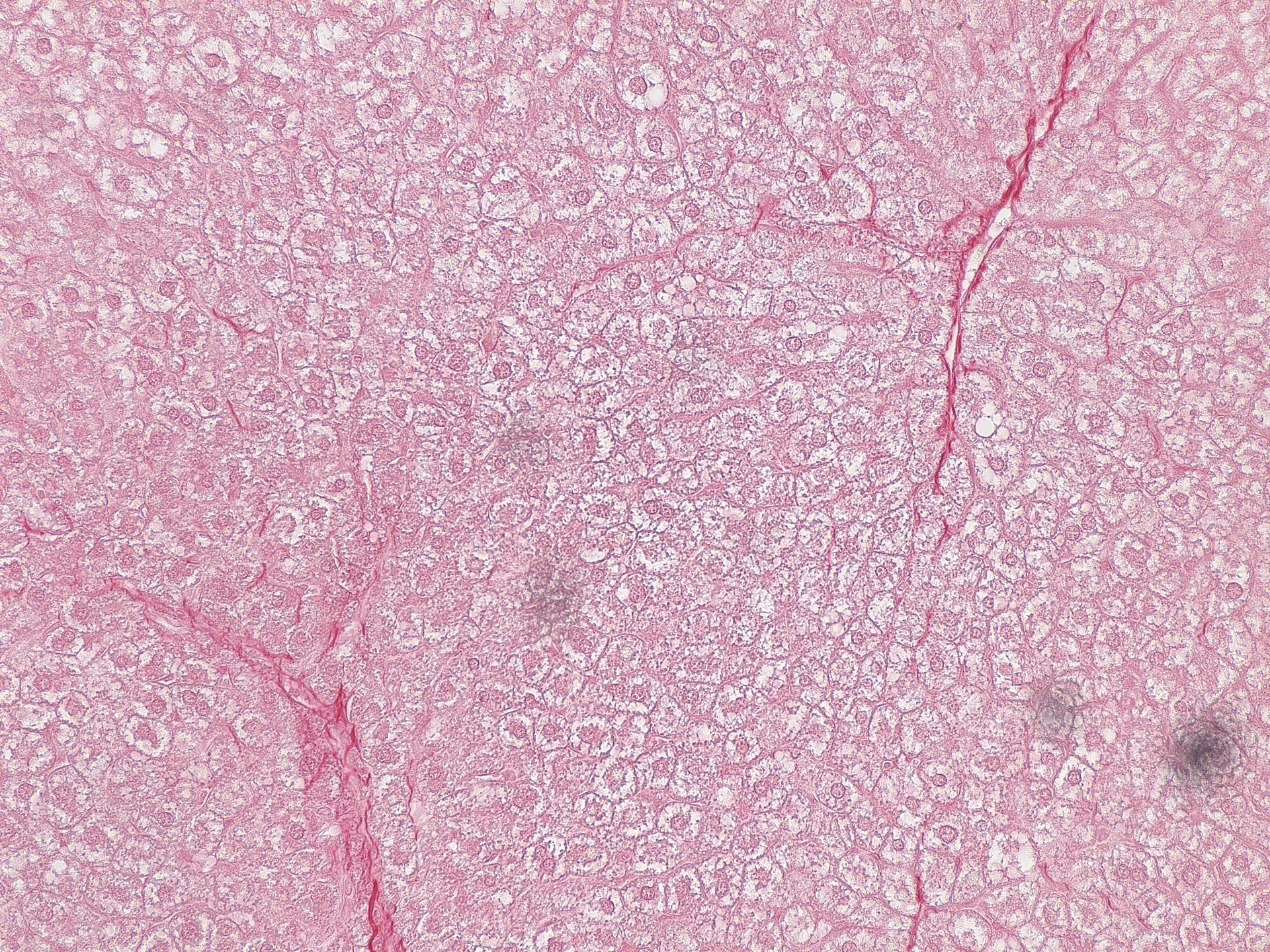

Supplement: Supplementary file 8 — Figure EV2 Source Data [file 44318_2024_196_MOESM8_ESM.zip › Figure EV2/Figure EV2-J/Quantificated image/NC AAV-mPcolce/no.2/NC-AAV-mPcolce-no.2-20x-5.jpg]

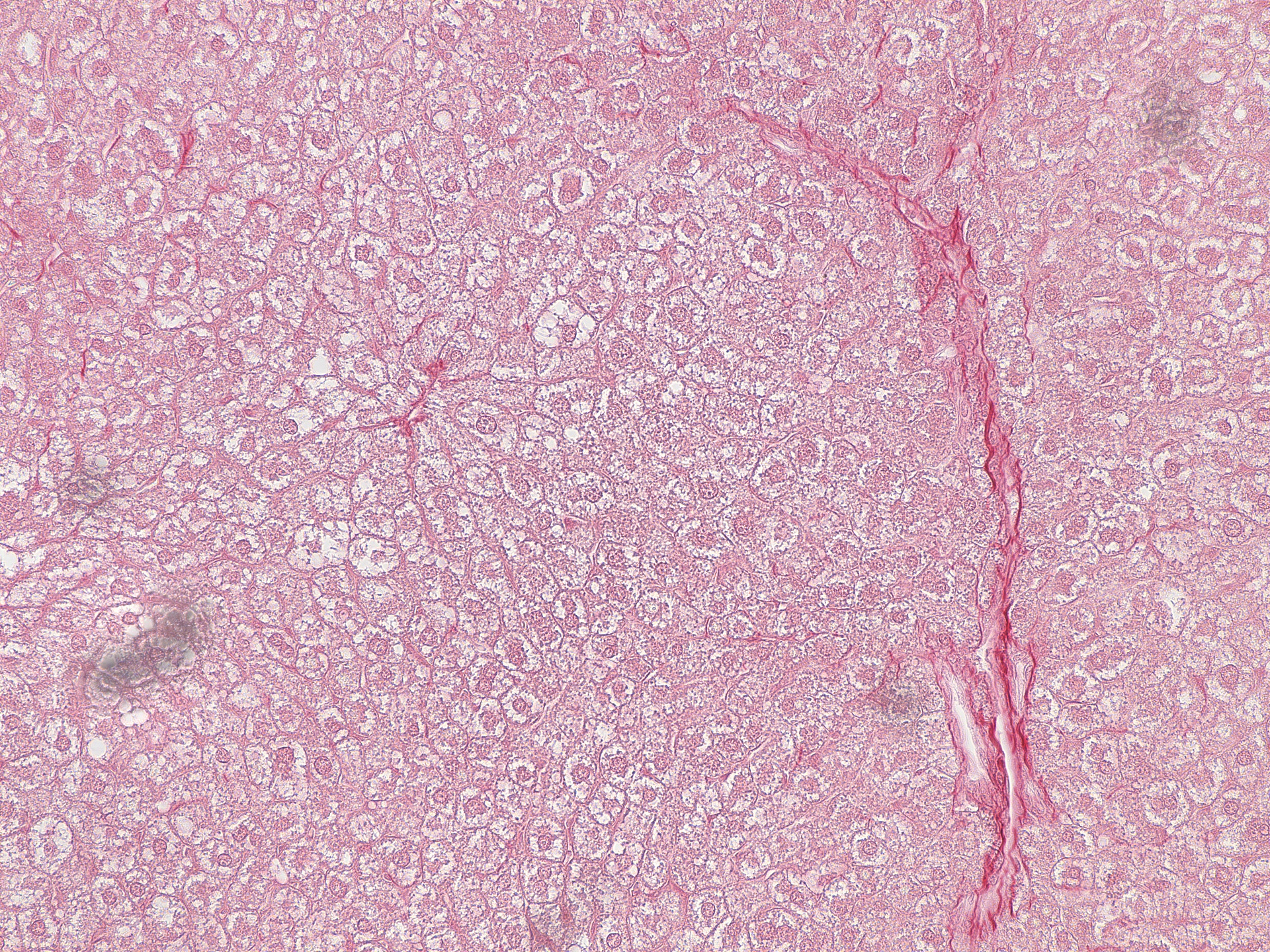

Supplement: Supplementary file 8 — Figure EV2 Source Data [file 44318_2024_196_MOESM8_ESM.zip › Figure EV2/Figure EV2-J/Quantificated image/NC AAV-mPcolce/no.2/NC-AAV-mPcolce-no.2-20x-4.jpg]

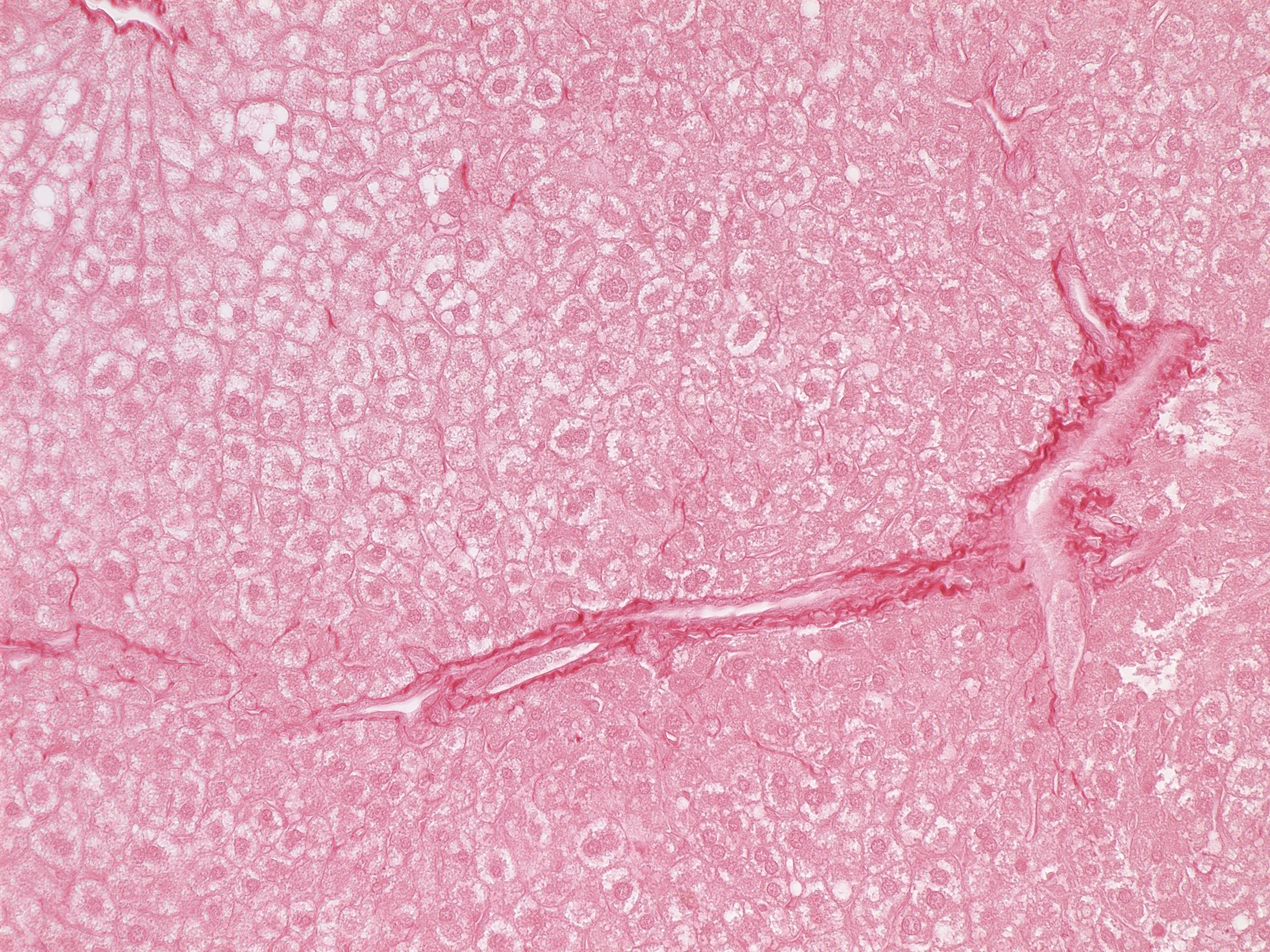

Supplement: Supplementary file 8 — Figure EV2 Source Data [file 44318_2024_196_MOESM8_ESM.zip › Figure EV2/Figure EV2-J/Quantificated image/NC AAV-mPcolce/no.2/NC-AAV-mPcolce-no.2-20x-3.jpg]

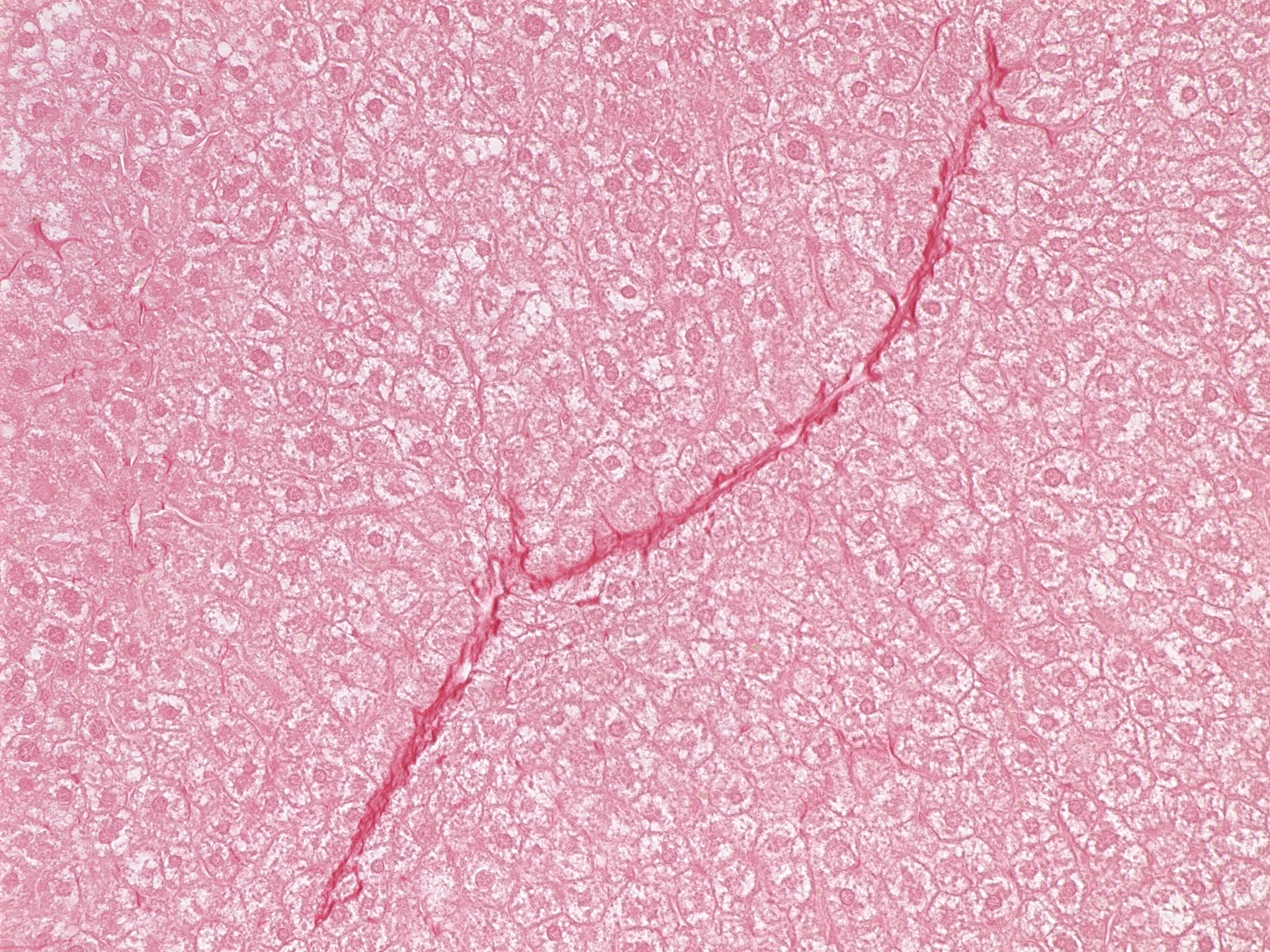

Supplement: Supplementary file 8 — Figure EV2 Source Data [file 44318_2024_196_MOESM8_ESM.zip › Figure EV2/Figure EV2-J/Quantificated image/NC AAV-mPcolce/no.2/NC-AAV-mPcolce-no.2-20x-2.jpg]

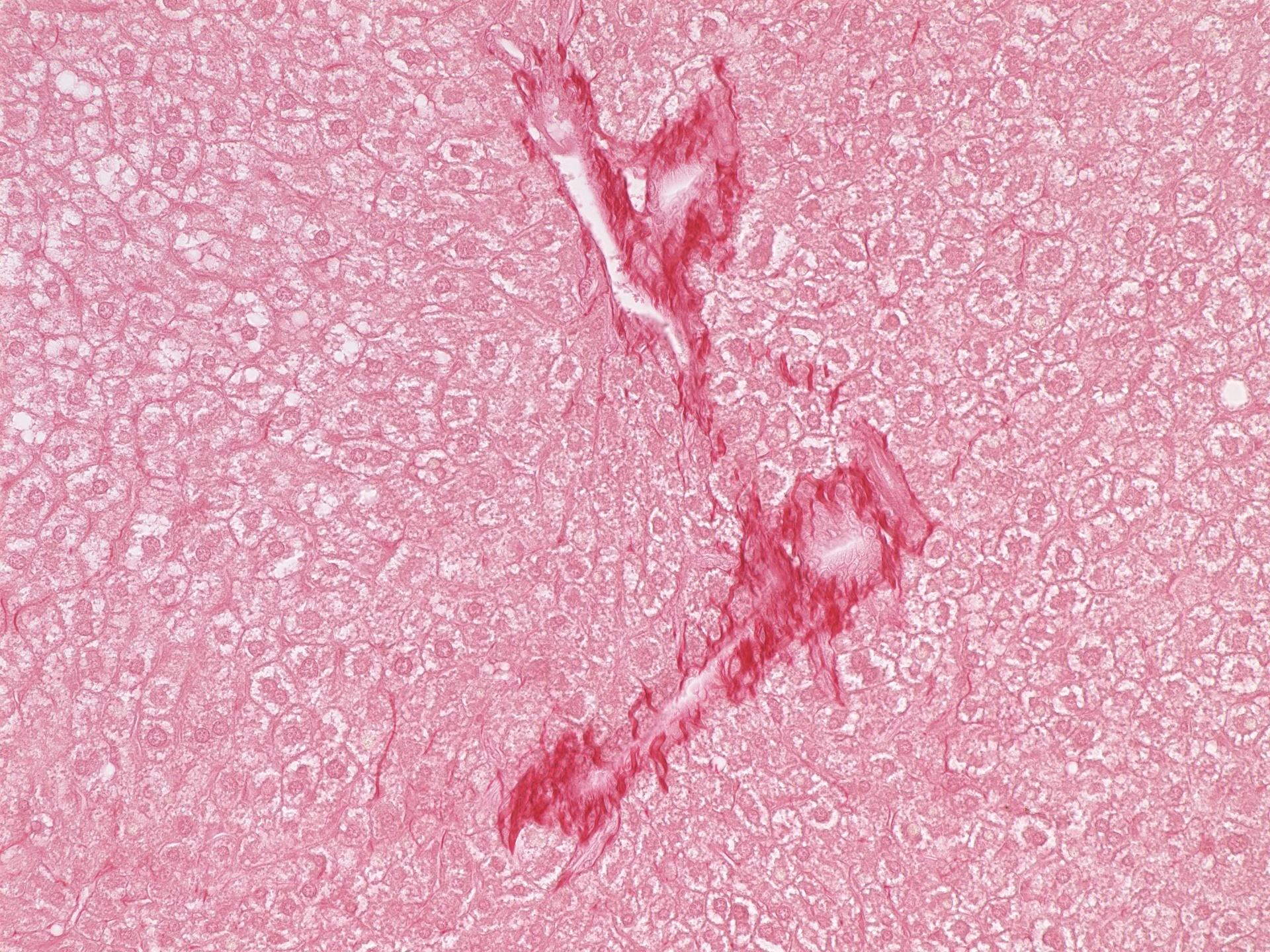

Supplement: Supplementary file 8 — Figure EV2 Source Data [file 44318_2024_196_MOESM8_ESM.zip › Figure EV2/Figure EV2-J/Quantificated image/NC AAV-mPcolce/no.2/NC-AAV-mPcolce-no.2-20x-1.jpg]

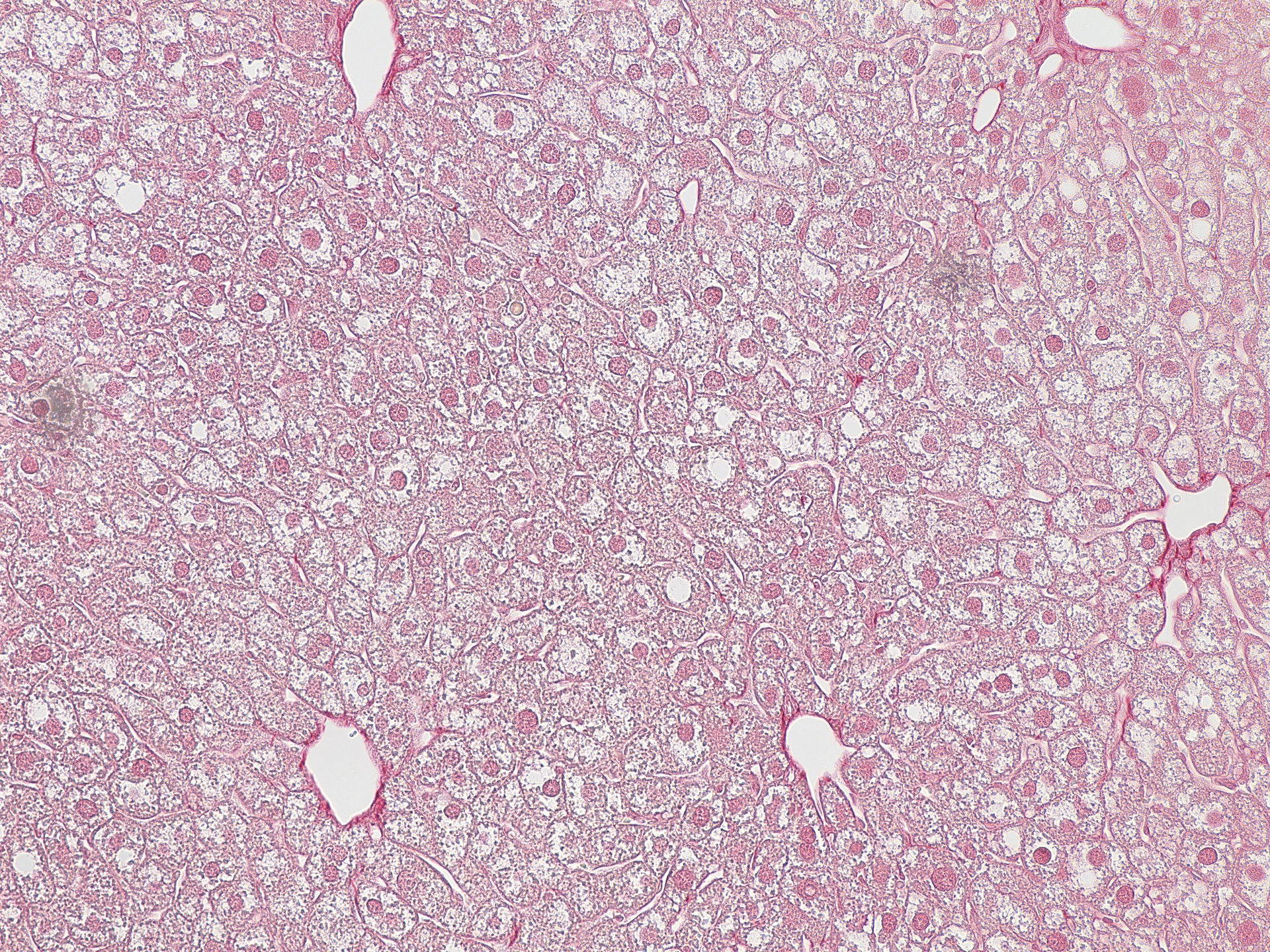

Supplement: Supplementary file 8 — Figure EV2 Source Data [file 44318_2024_196_MOESM8_ESM.zip › Figure EV2/Figure EV2-J/Quantificated image/NC Mock/no.1/NC-Mock-no.1-20x-5.jpg]

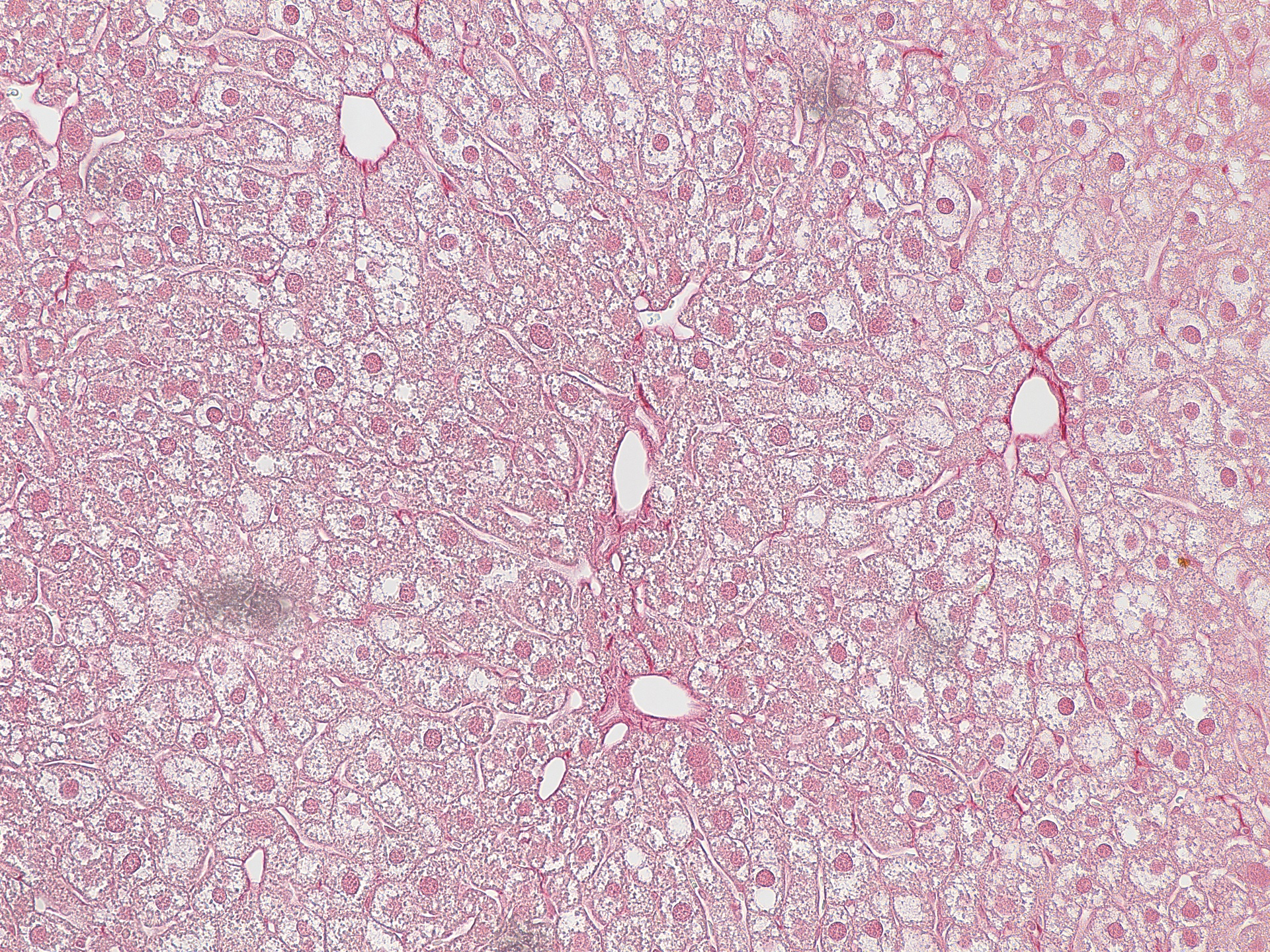

Supplement: Supplementary file 8 — Figure EV2 Source Data [file 44318_2024_196_MOESM8_ESM.zip › Figure EV2/Figure EV2-J/Quantificated image/NC Mock/no.1/NC-Mock-no.1-20x-4.jpg]

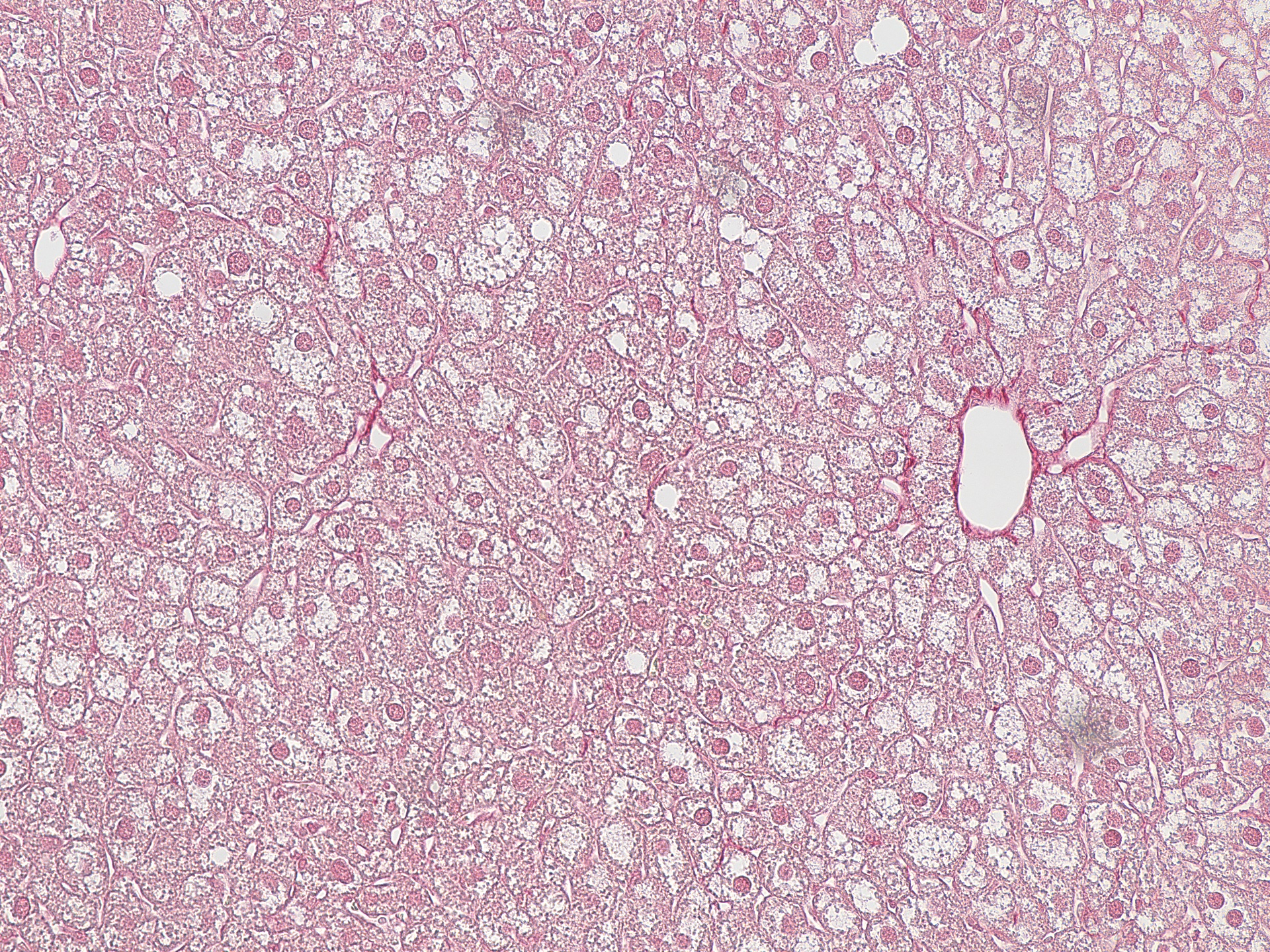

Supplement: Supplementary file 8 — Figure EV2 Source Data [file 44318_2024_196_MOESM8_ESM.zip › Figure EV2/Figure EV2-J/Quantificated image/NC Mock/no.1/NC-Mock-no.1-20x-3.jpg]

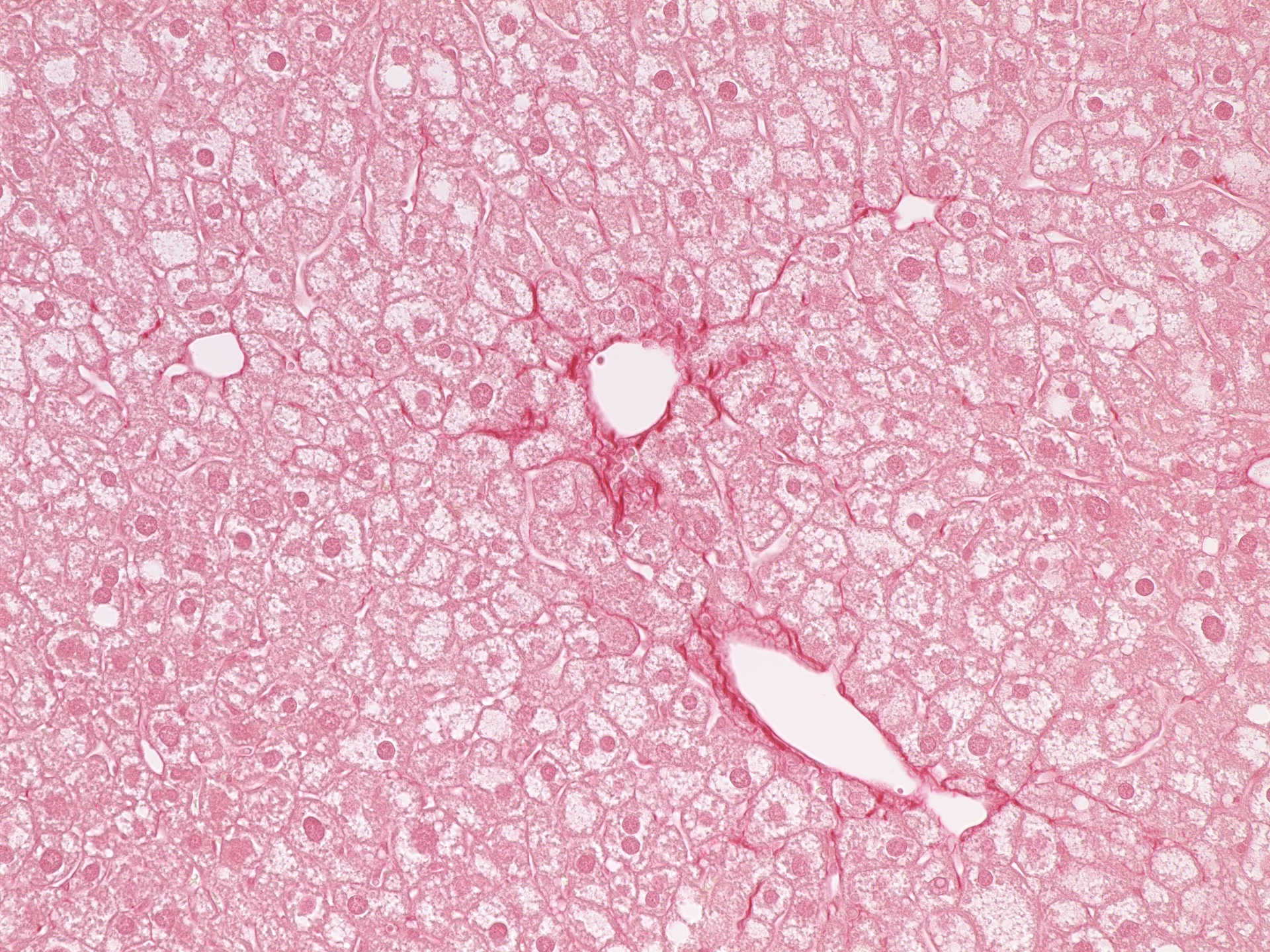

Supplement: Supplementary file 8 — Figure EV2 Source Data [file 44318_2024_196_MOESM8_ESM.zip › Figure EV2/Figure EV2-J/Quantificated image/NC Mock/no.1/NC-Mock-no.1-20x-2.jpg]

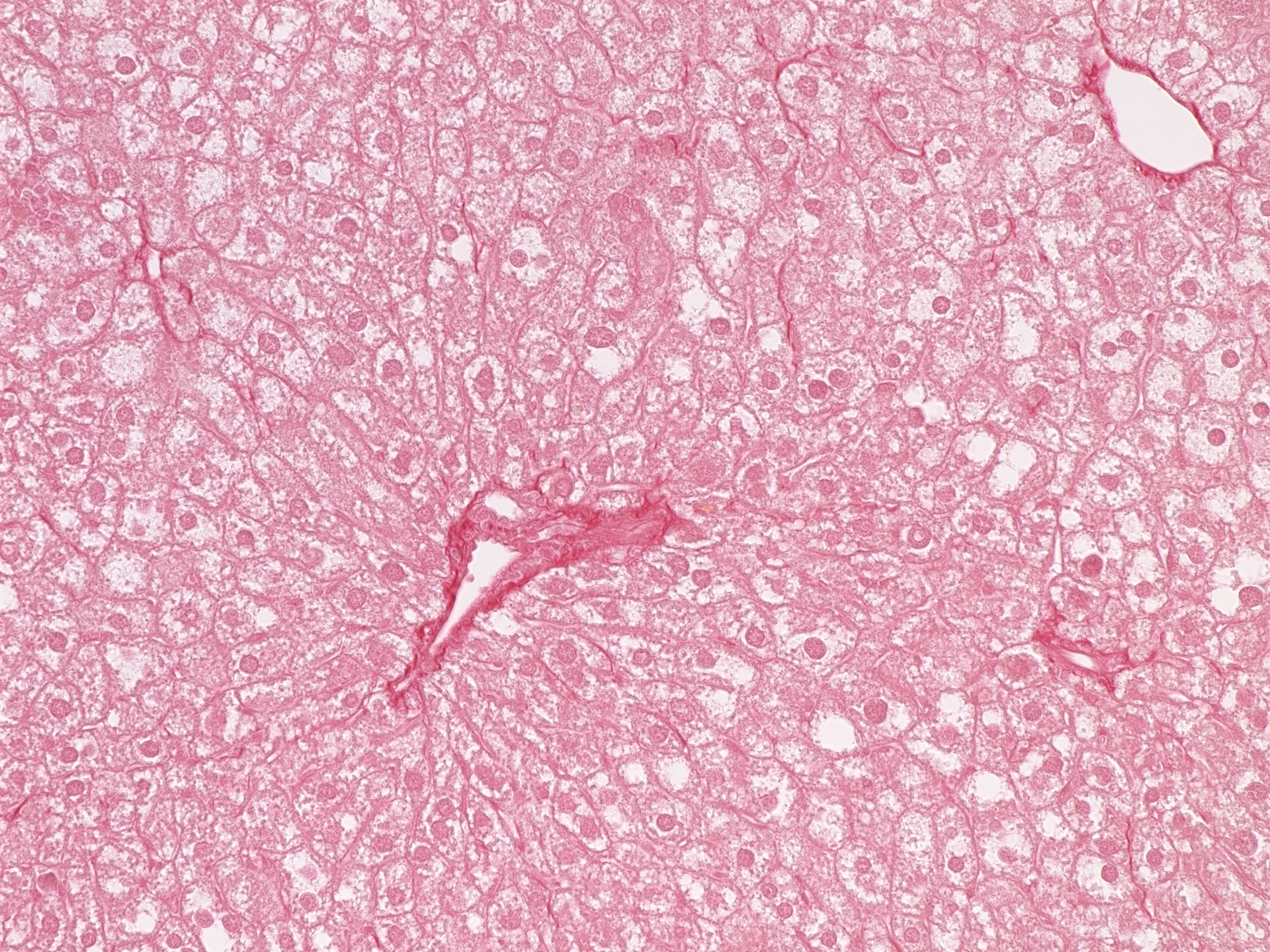

Supplement: Supplementary file 8 — Figure EV2 Source Data [file 44318_2024_196_MOESM8_ESM.zip › Figure EV2/Figure EV2-J/Quantificated image/NC Mock/no.1/NC-Mock-no.1-20x-1.jpg]

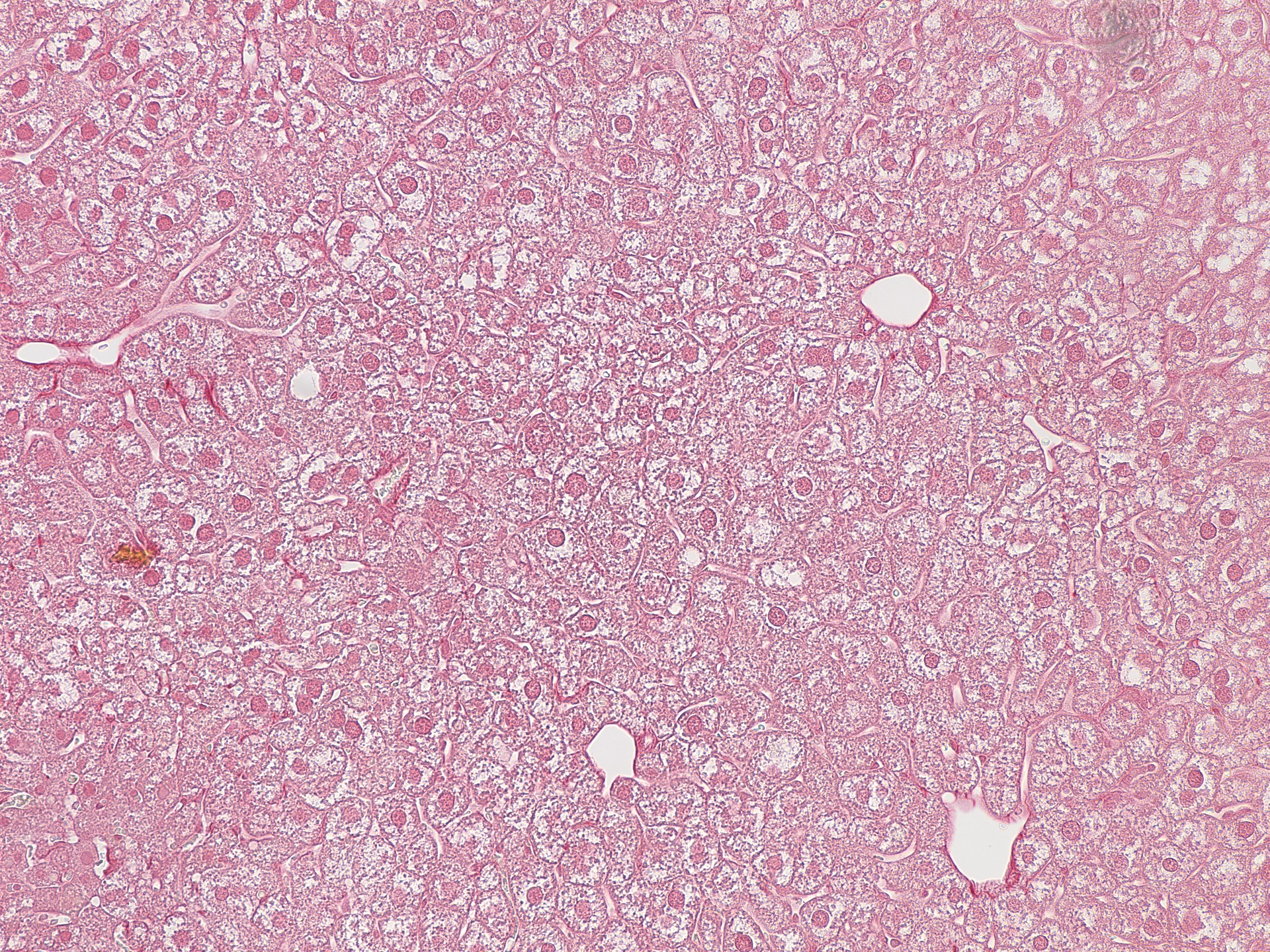

Supplement: Supplementary file 8 — Figure EV2 Source Data [file 44318_2024_196_MOESM8_ESM.zip › Figure EV2/Figure EV2-J/Quantificated image/NC Mock/no.3/NC-Mock-no.3-20x-4.jpg]

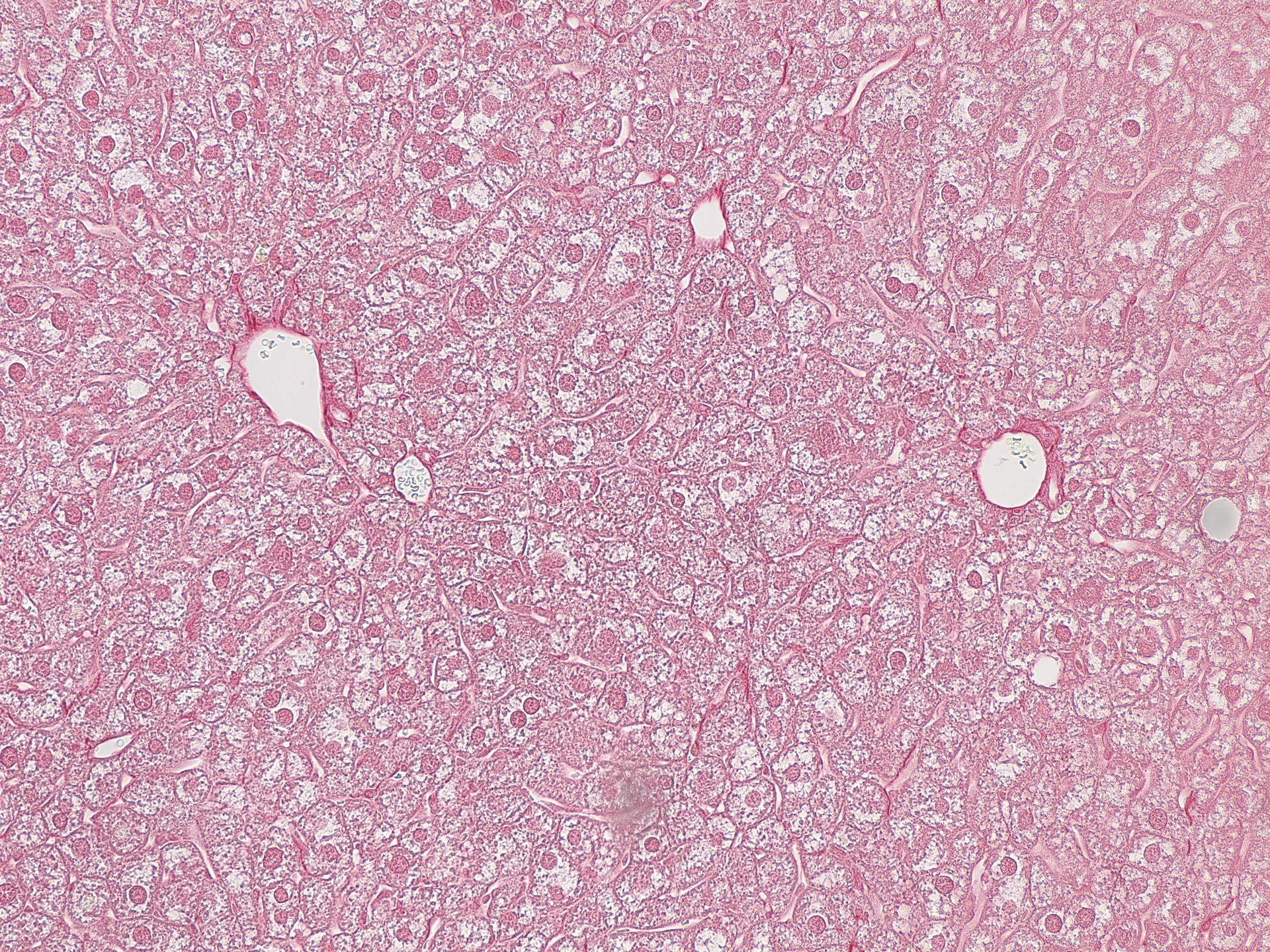

Supplement: Supplementary file 8 — Figure EV2 Source Data [file 44318_2024_196_MOESM8_ESM.zip › Figure EV2/Figure EV2-J/Quantificated image/NC Mock/no.3/NC-Mock-no.3-20x-5.jpg]

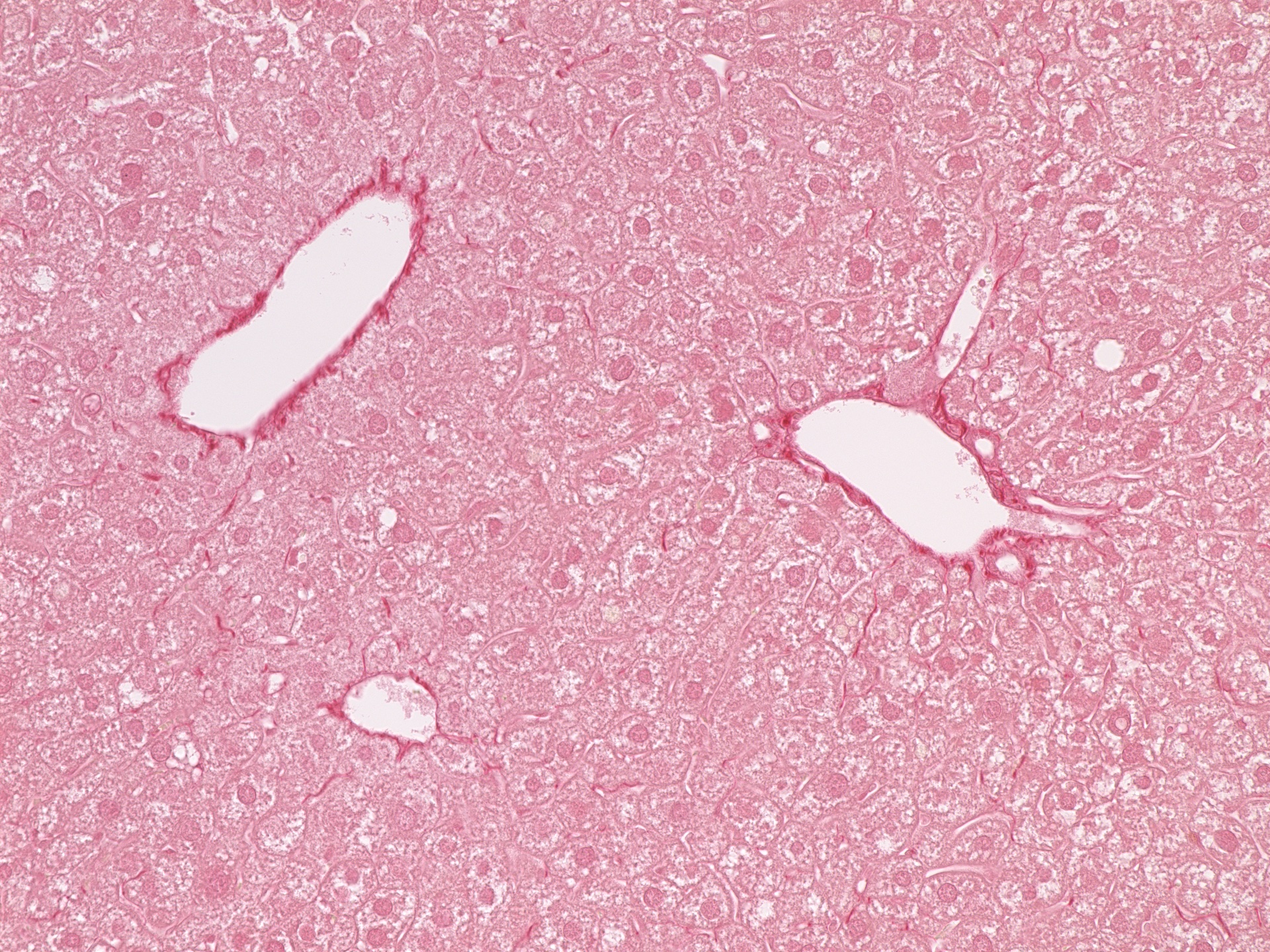

Supplement: Supplementary file 8 — Figure EV2 Source Data [file 44318_2024_196_MOESM8_ESM.zip › Figure EV2/Figure EV2-J/Quantificated image/NC Mock/no.3/NC-Mock-no.3-20x-2.jpg]

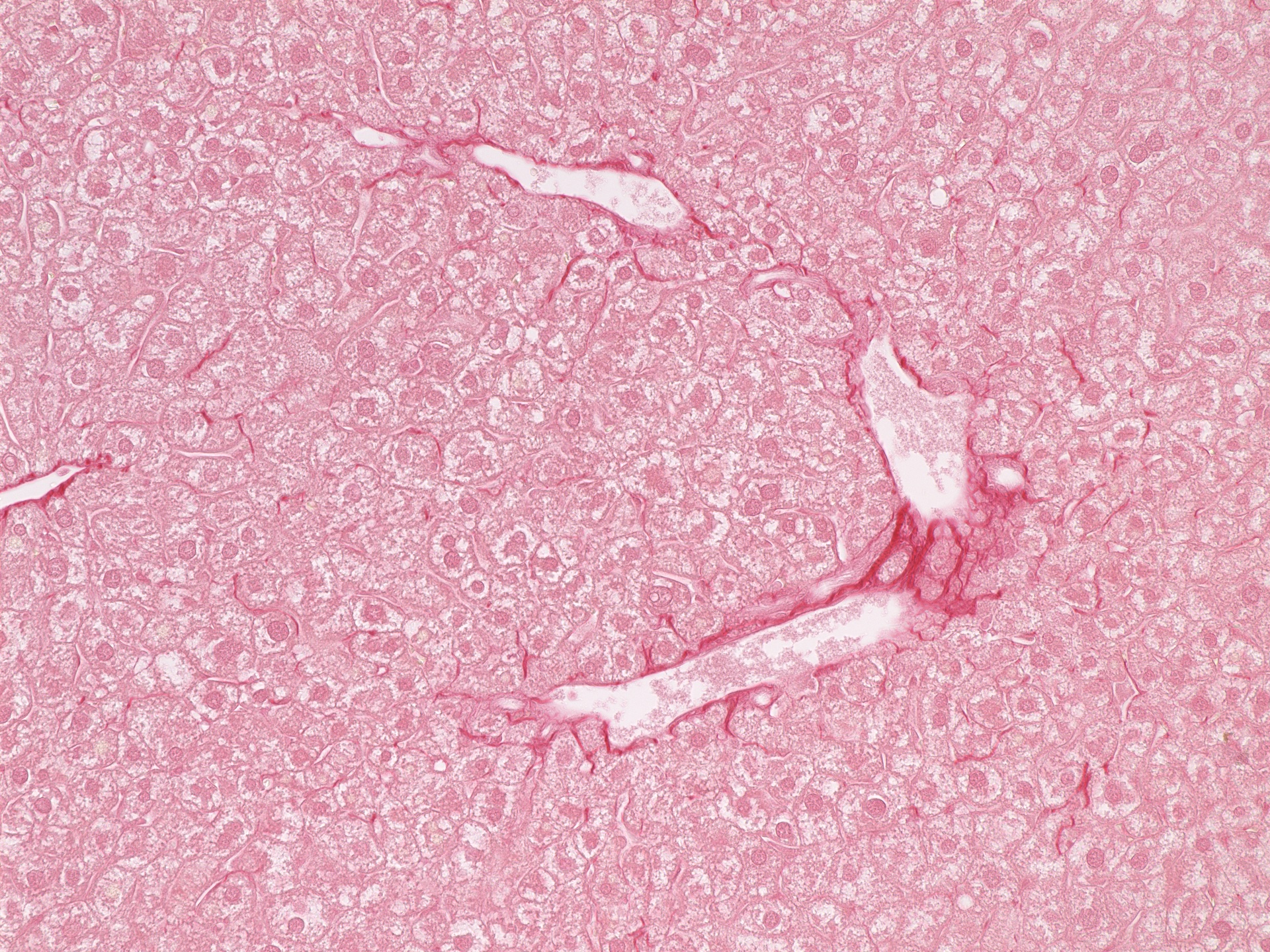

Supplement: Supplementary file 8 — Figure EV2 Source Data [file 44318_2024_196_MOESM8_ESM.zip › Figure EV2/Figure EV2-J/Quantificated image/NC Mock/no.3/NC-Mock-no.3-20x-3.jpg]

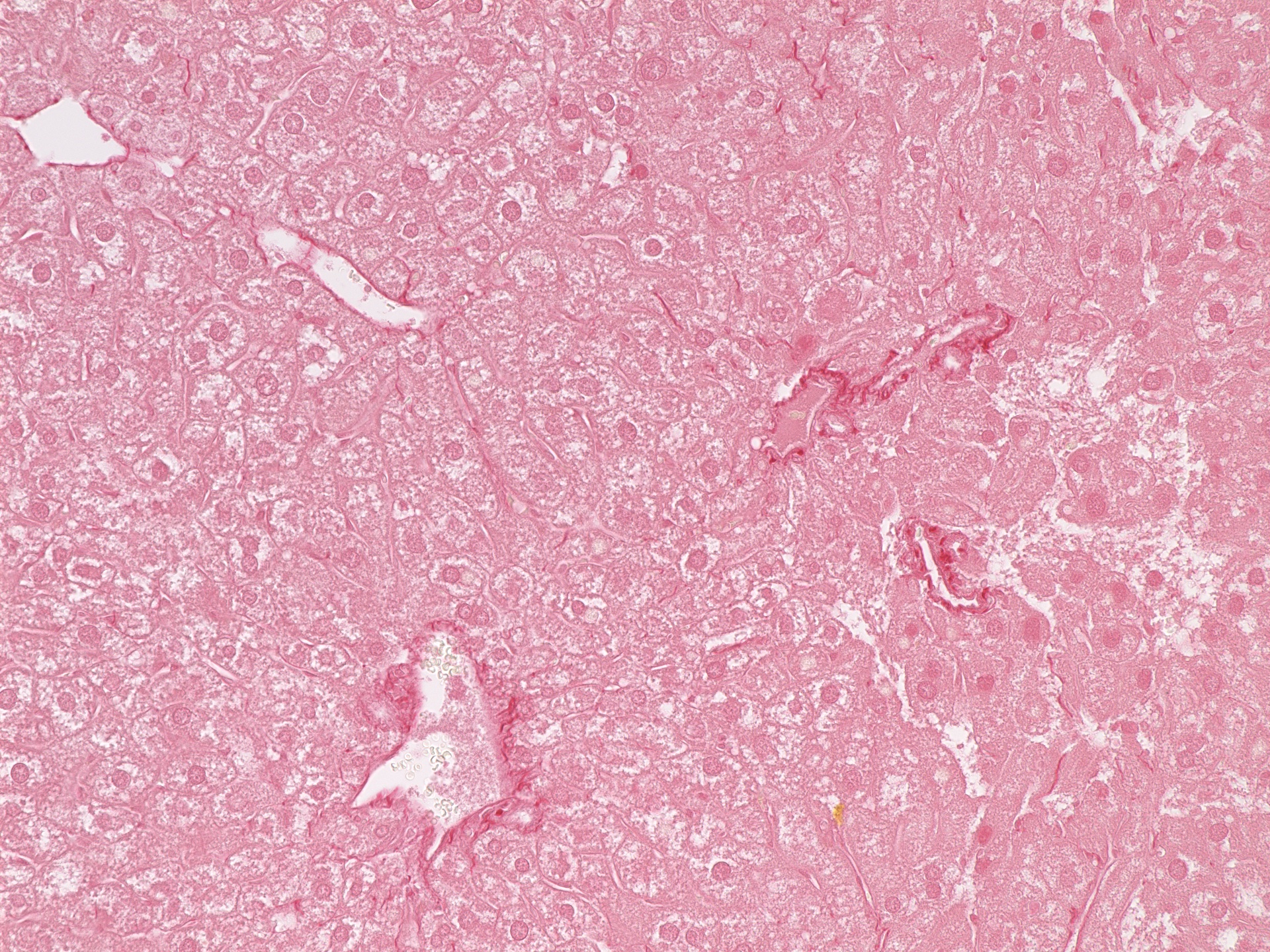

Supplement: Supplementary file 8 — Figure EV2 Source Data [file 44318_2024_196_MOESM8_ESM.zip › Figure EV2/Figure EV2-J/Quantificated image/NC Mock/no.3/NC-Mock-no.3-20x-1.jpg]

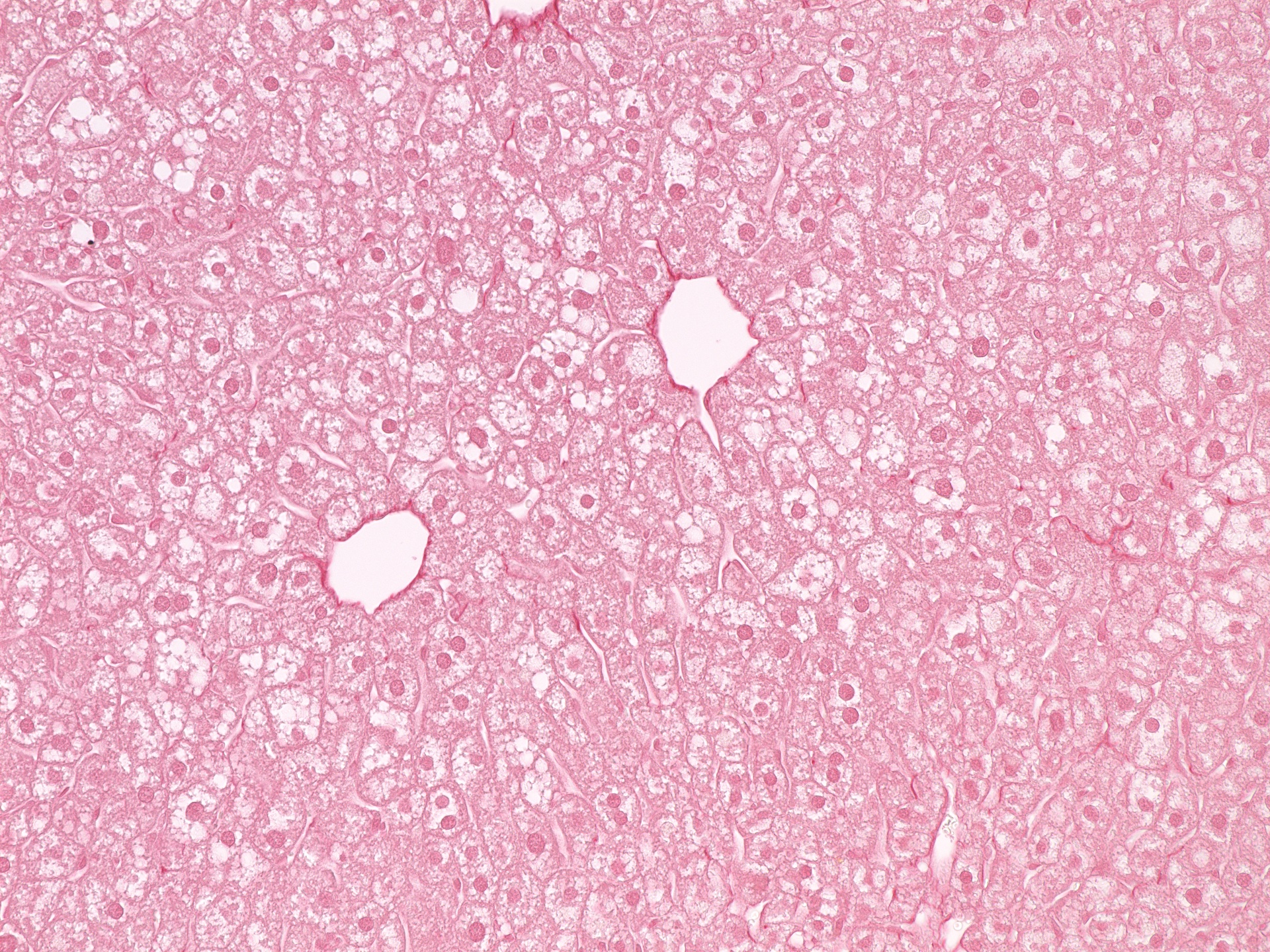

Supplement: Supplementary file 8 — Figure EV2 Source Data [file 44318_2024_196_MOESM8_ESM.zip › Figure EV2/Figure EV2-J/Quantificated image/NC Mock/no.4/NC-Mock-no.4-20x-3.jpg]

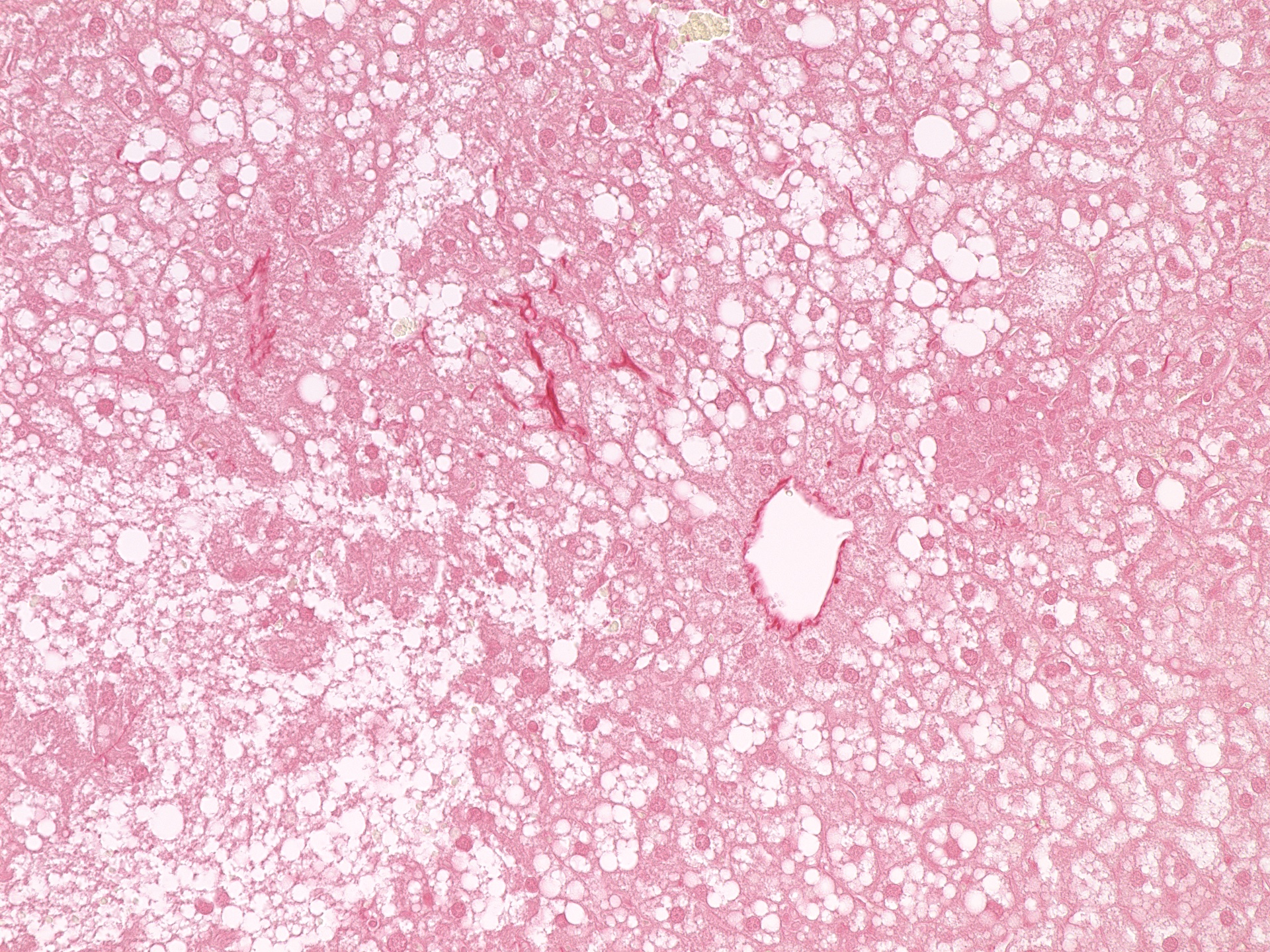

Supplement: Supplementary file 8 — Figure EV2 Source Data [file 44318_2024_196_MOESM8_ESM.zip › Figure EV2/Figure EV2-J/Quantificated image/NC Mock/no.4/NC-Mock-no.4-20x-2.jpg]

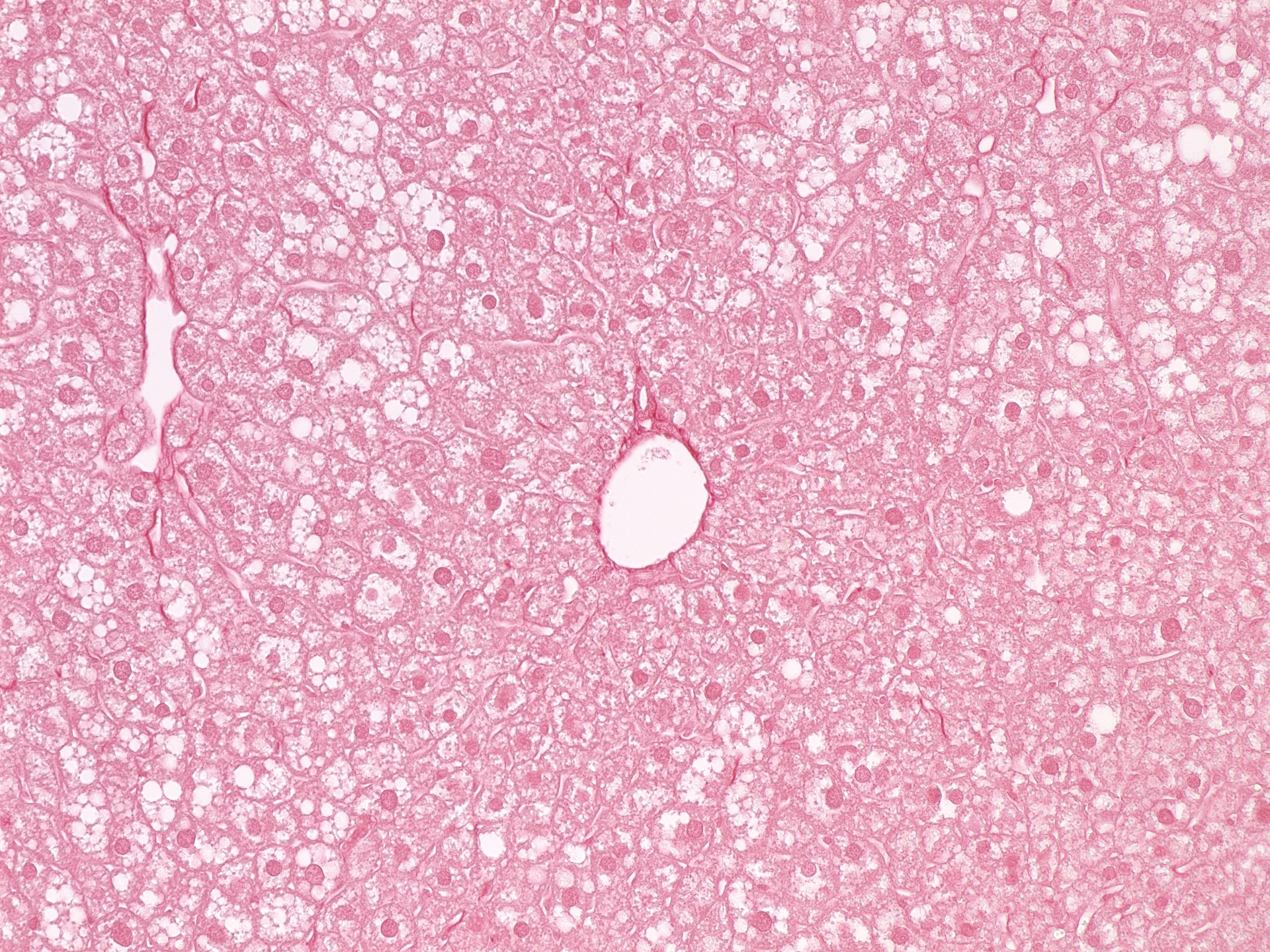

Supplement: Supplementary file 8 — Figure EV2 Source Data [file 44318_2024_196_MOESM8_ESM.zip › Figure EV2/Figure EV2-J/Quantificated image/NC Mock/no.4/NC-Mock-no.4-20x-1.jpg]

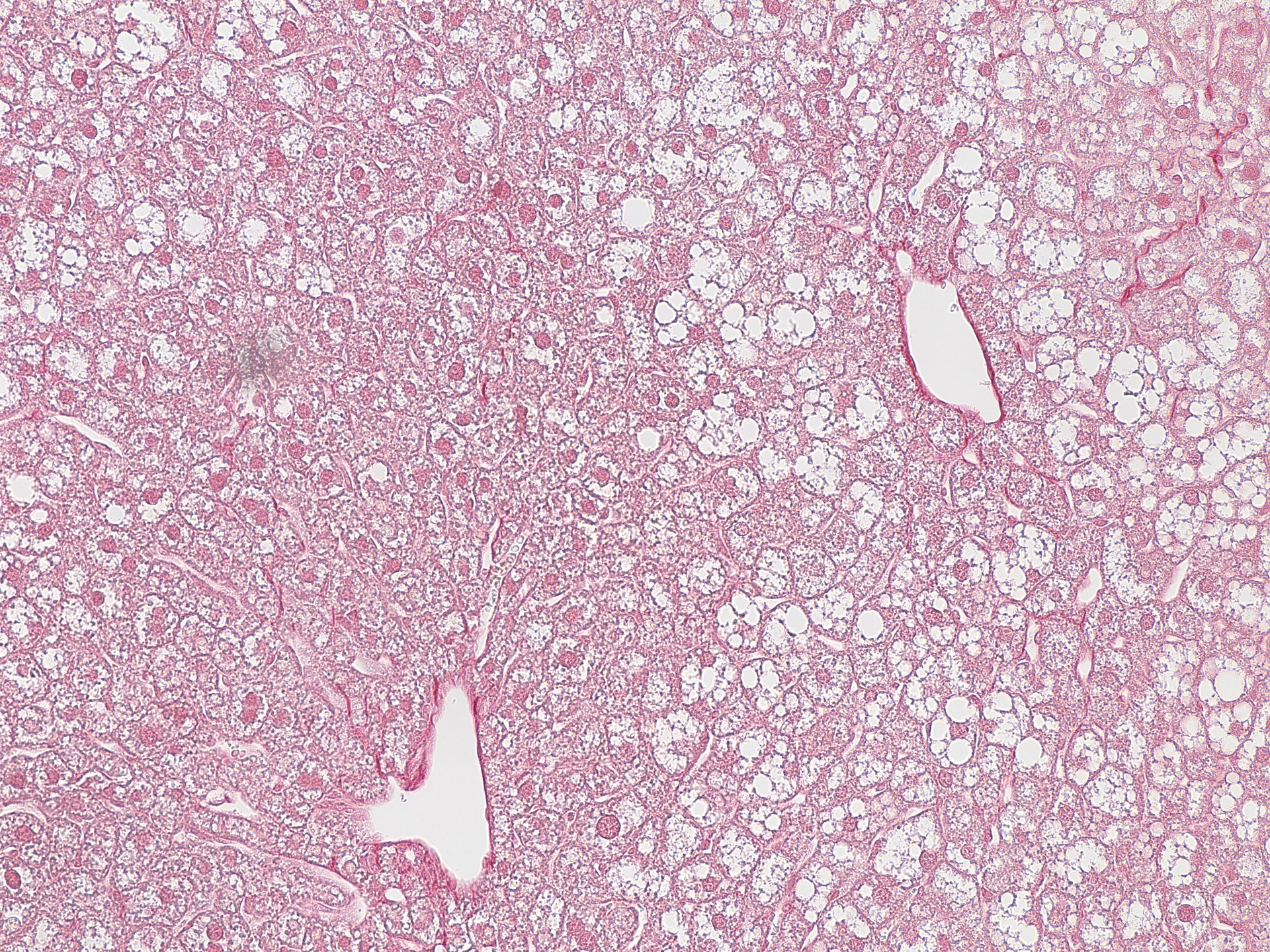

Supplement: Supplementary file 8 — Figure EV2 Source Data [file 44318_2024_196_MOESM8_ESM.zip › Figure EV2/Figure EV2-J/Quantificated image/NC Mock/no.4/NC-Mock-no.4-20x-5.jpg]

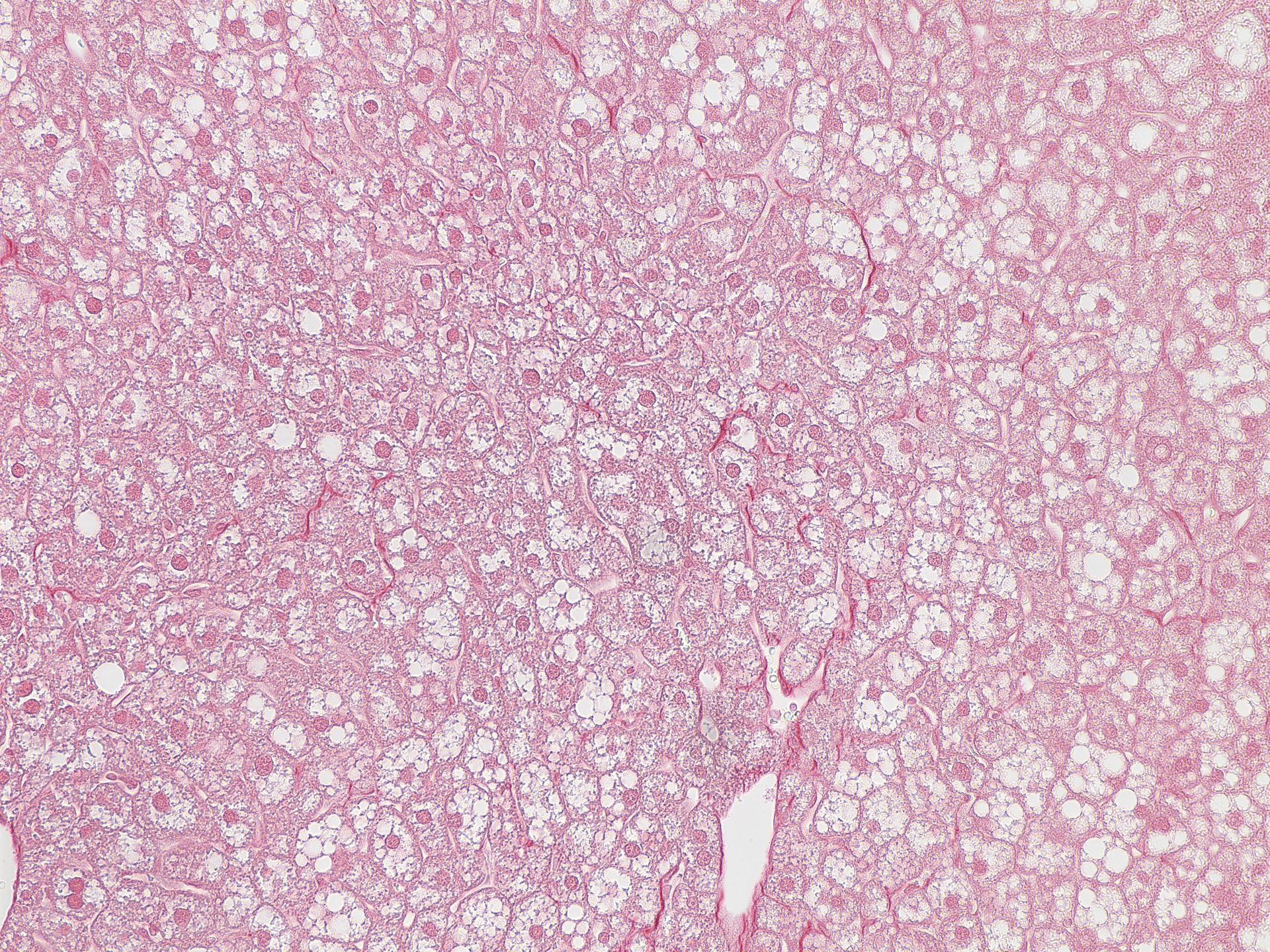

Supplement: Supplementary file 8 — Figure EV2 Source Data [file 44318_2024_196_MOESM8_ESM.zip › Figure EV2/Figure EV2-J/Quantificated image/NC Mock/no.4/NC-Mock-no.4-20x-4.jpg]

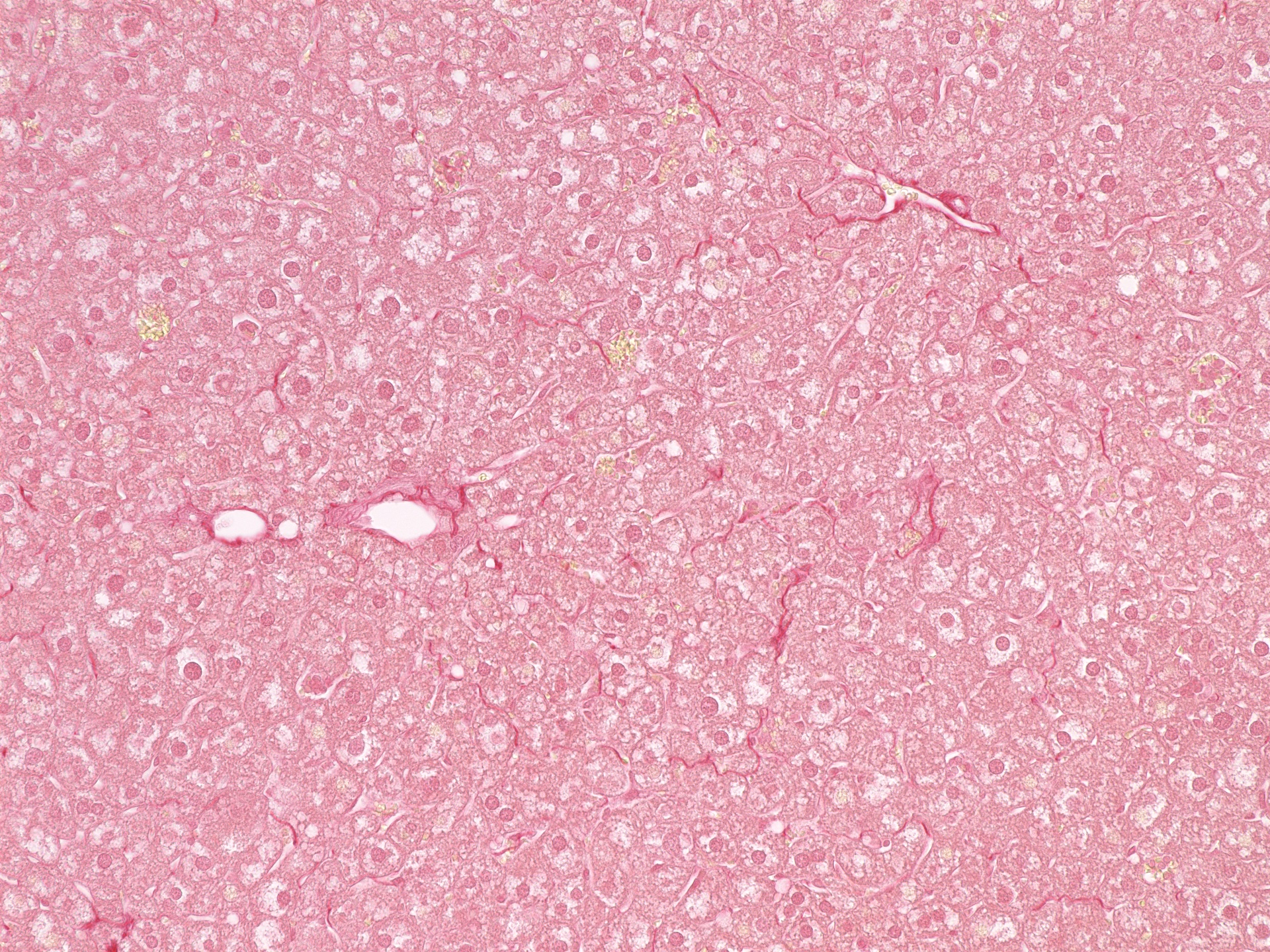

Supplement: Supplementary file 8 — Figure EV2 Source Data [file 44318_2024_196_MOESM8_ESM.zip › Figure EV2/Figure EV2-J/Quantificated image/NC Mock/no.5/NC-Mock-no.5-20x-3.jpg]

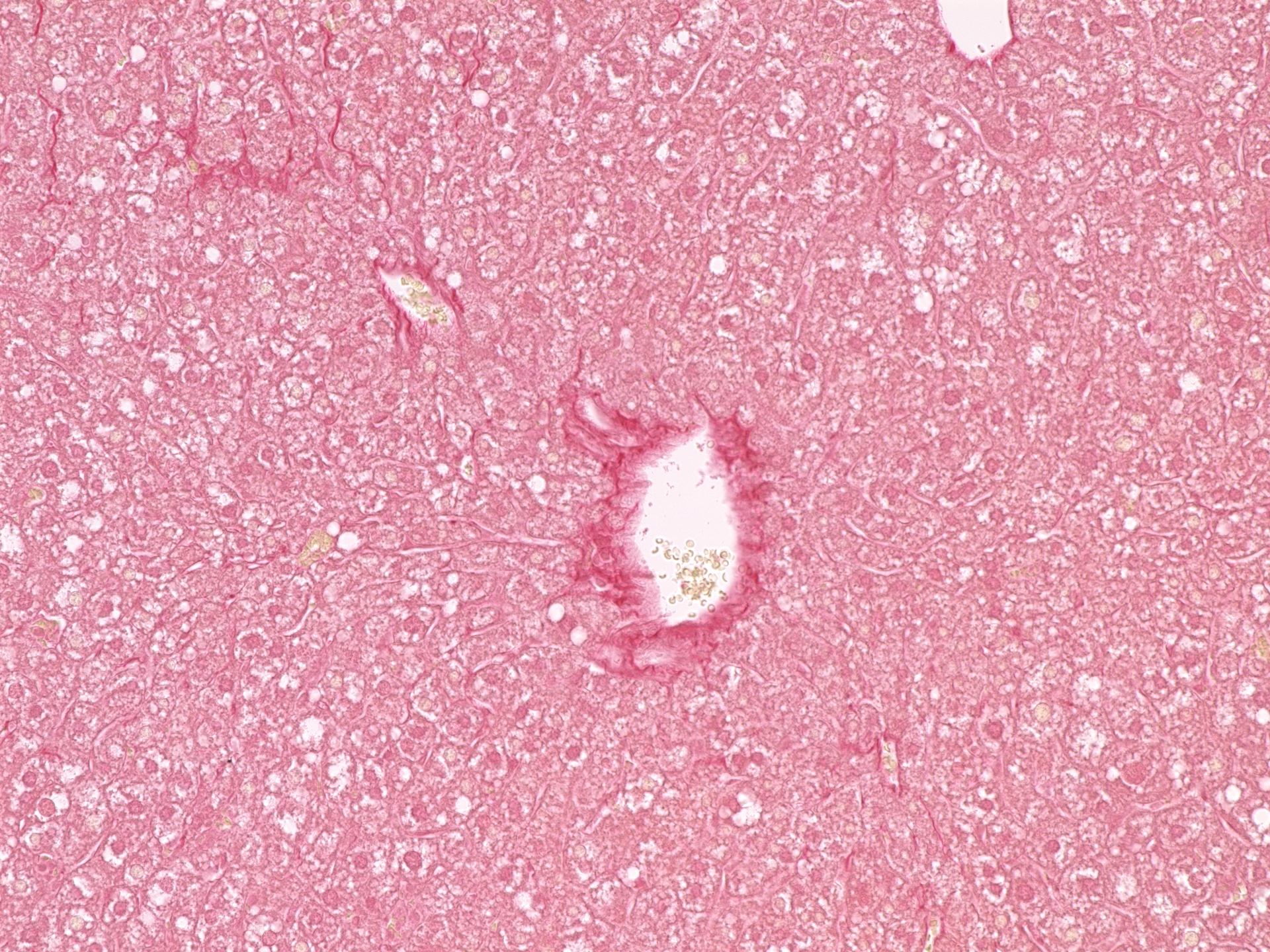

Supplement: Supplementary file 8 — Figure EV2 Source Data [file 44318_2024_196_MOESM8_ESM.zip › Figure EV2/Figure EV2-J/Quantificated image/NC Mock/no.5/NC-Mock-no.5-20x-2.jpg]

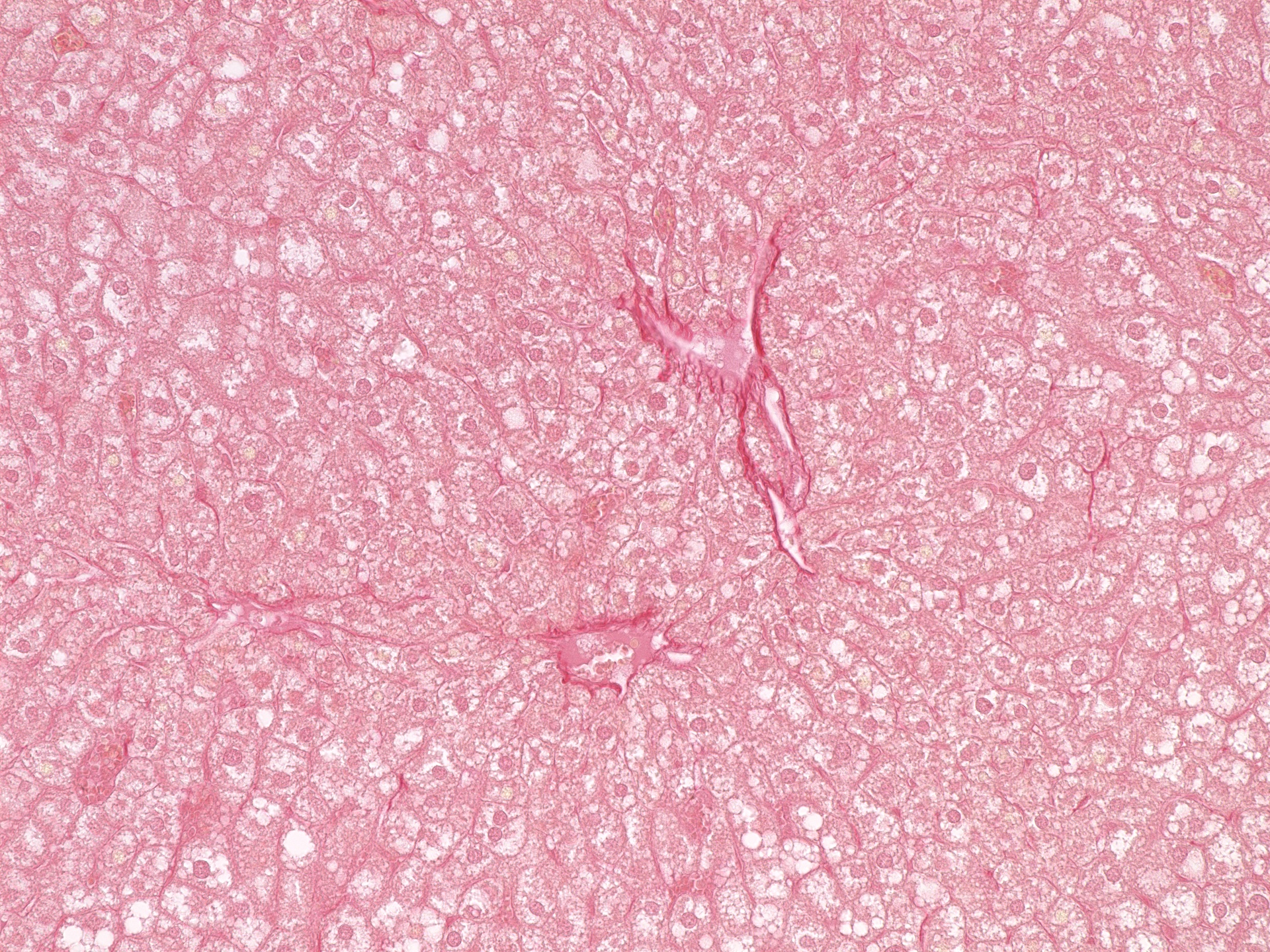

Supplement: Supplementary file 8 — Figure EV2 Source Data [file 44318_2024_196_MOESM8_ESM.zip › Figure EV2/Figure EV2-J/Quantificated image/NC Mock/no.5/NC-Mock-no.5-20x-1.jpg]

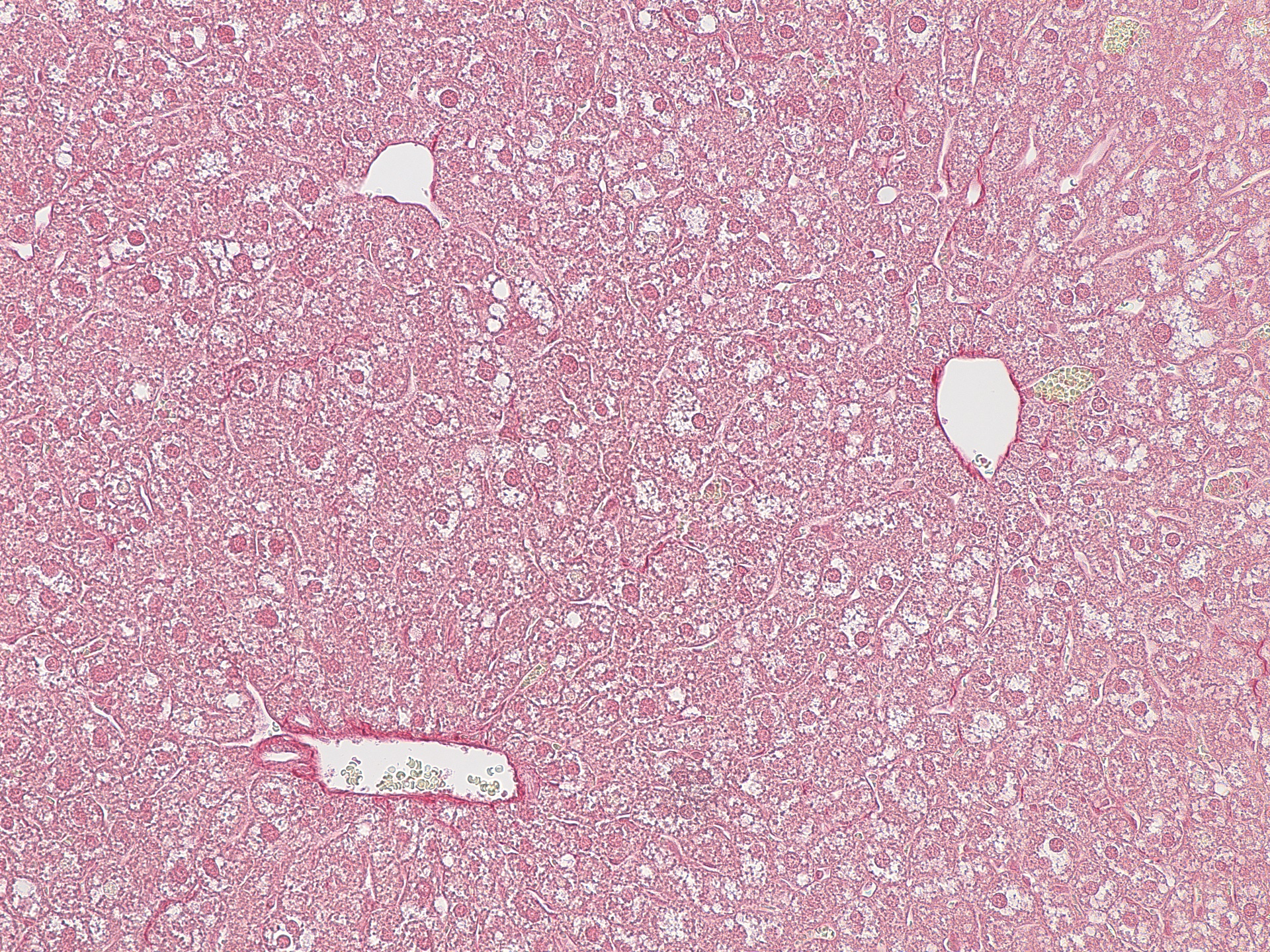

Supplement: Supplementary file 8 — Figure EV2 Source Data [file 44318_2024_196_MOESM8_ESM.zip › Figure EV2/Figure EV2-J/Quantificated image/NC Mock/no.5/NC-Mock-no.5-20x-5.jpg]

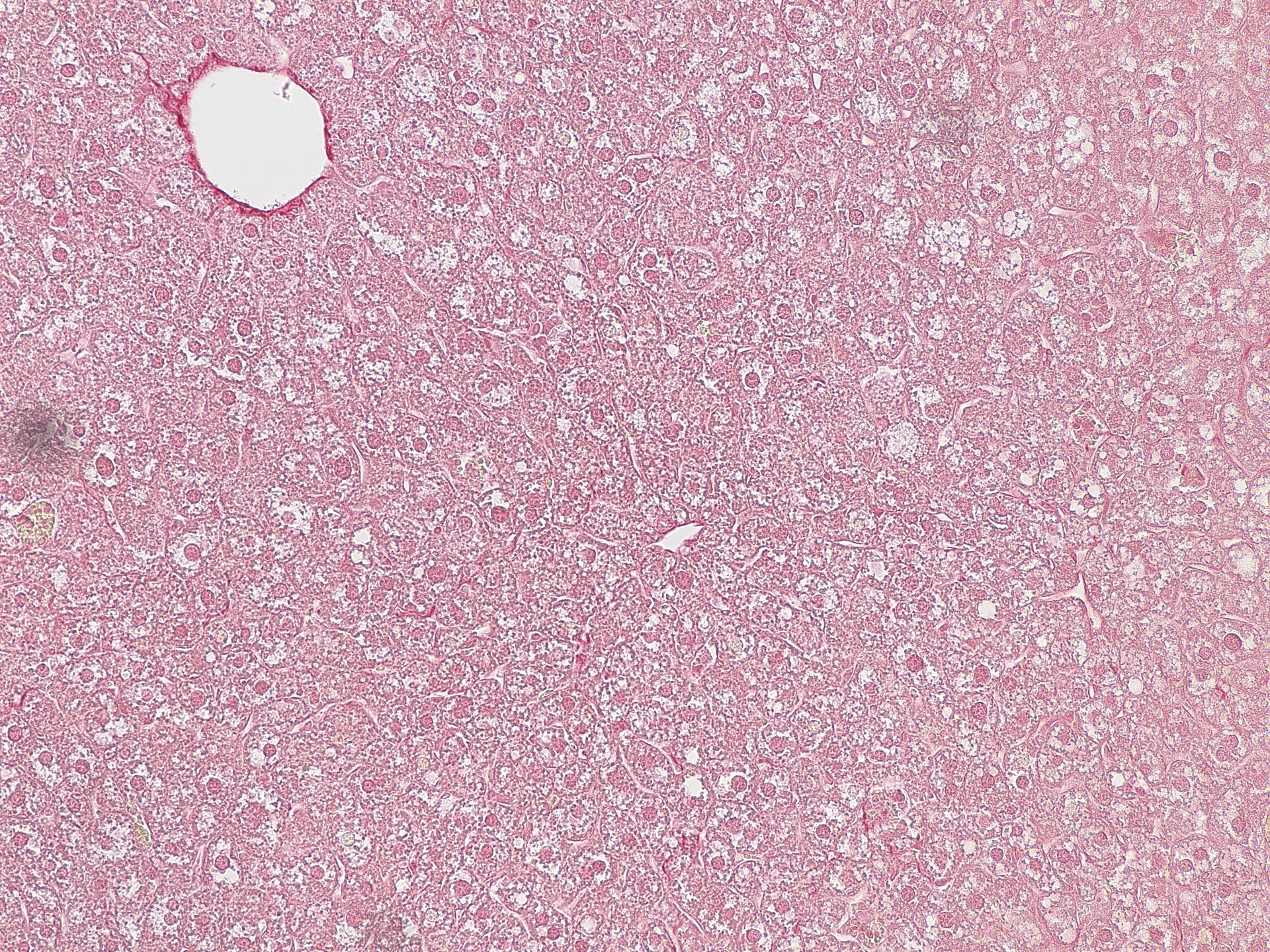

Supplement: Supplementary file 8 — Figure EV2 Source Data [file 44318_2024_196_MOESM8_ESM.zip › Figure EV2/Figure EV2-J/Quantificated image/NC Mock/no.5/NC-Mock-no.5-20x-4.jpg]

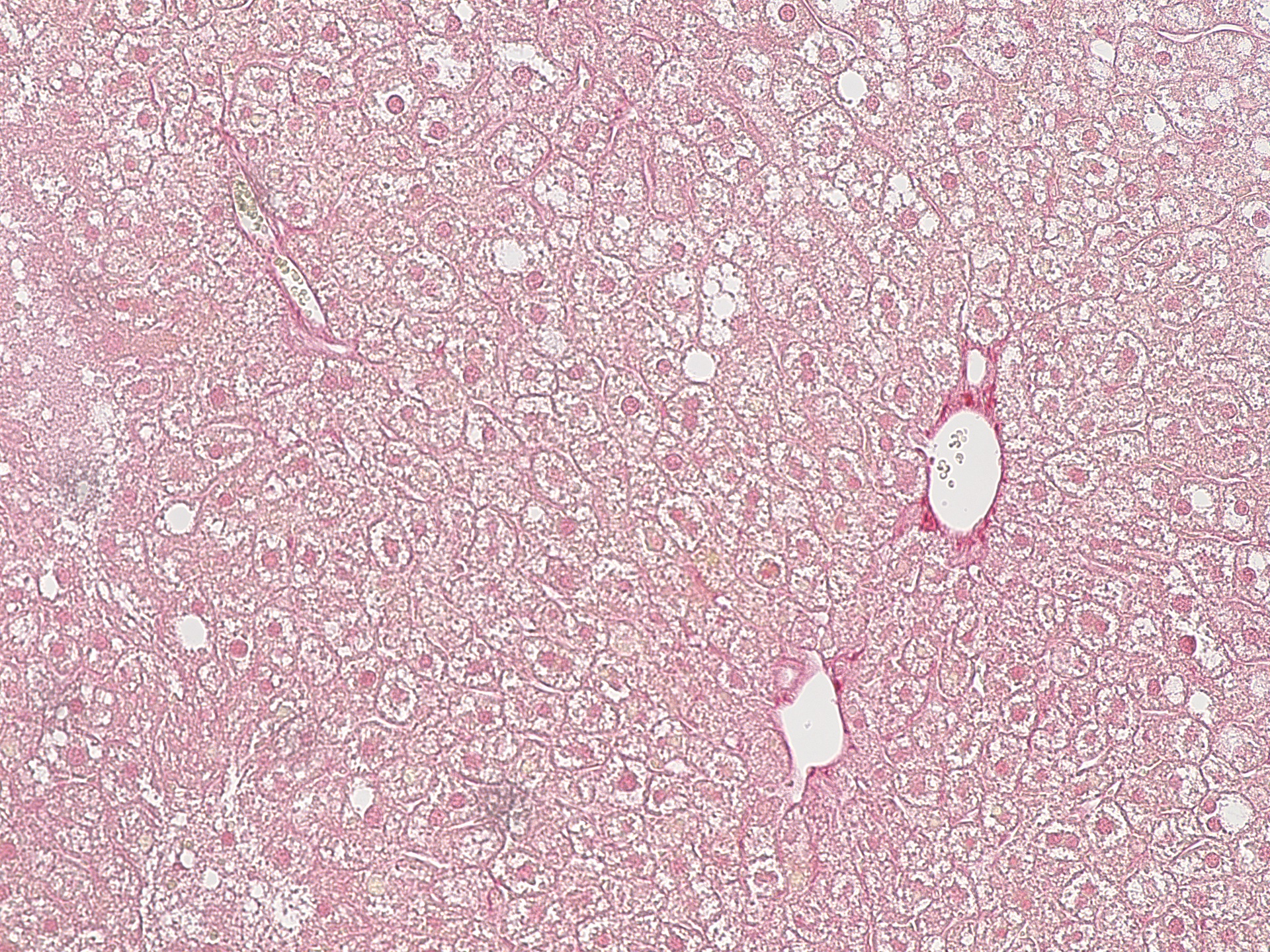

Supplement: Supplementary file 8 — Figure EV2 Source Data [file 44318_2024_196_MOESM8_ESM.zip › Figure EV2/Figure EV2-J/Quantificated image/NC Mock/no.2/NC-Mock-no.2-20x-4.jpg]

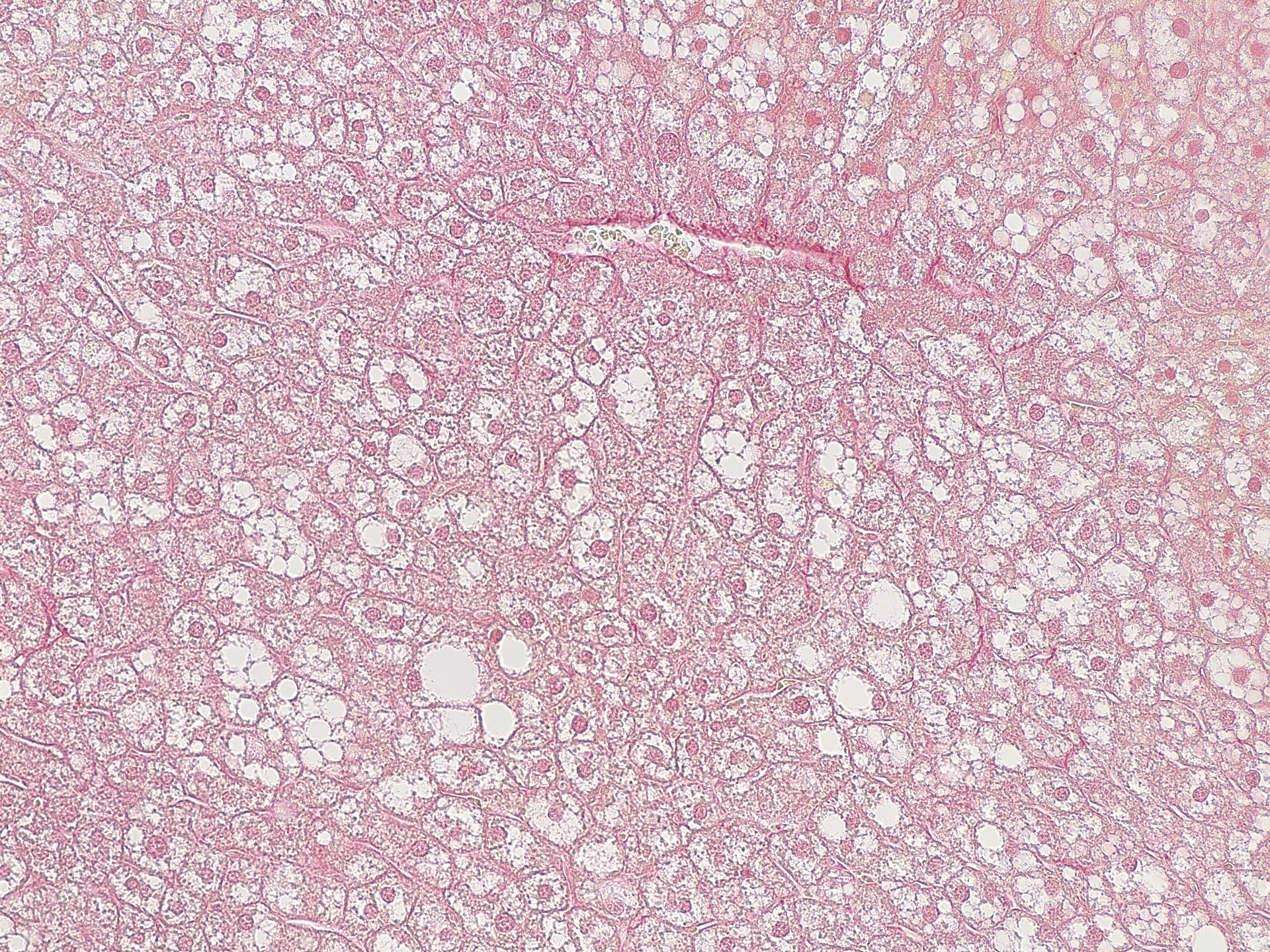

Supplement: Supplementary file 8 — Figure EV2 Source Data [file 44318_2024_196_MOESM8_ESM.zip › Figure EV2/Figure EV2-J/Quantificated image/NC Mock/no.2/NC-Mock-no.2-20x-5.jpg]

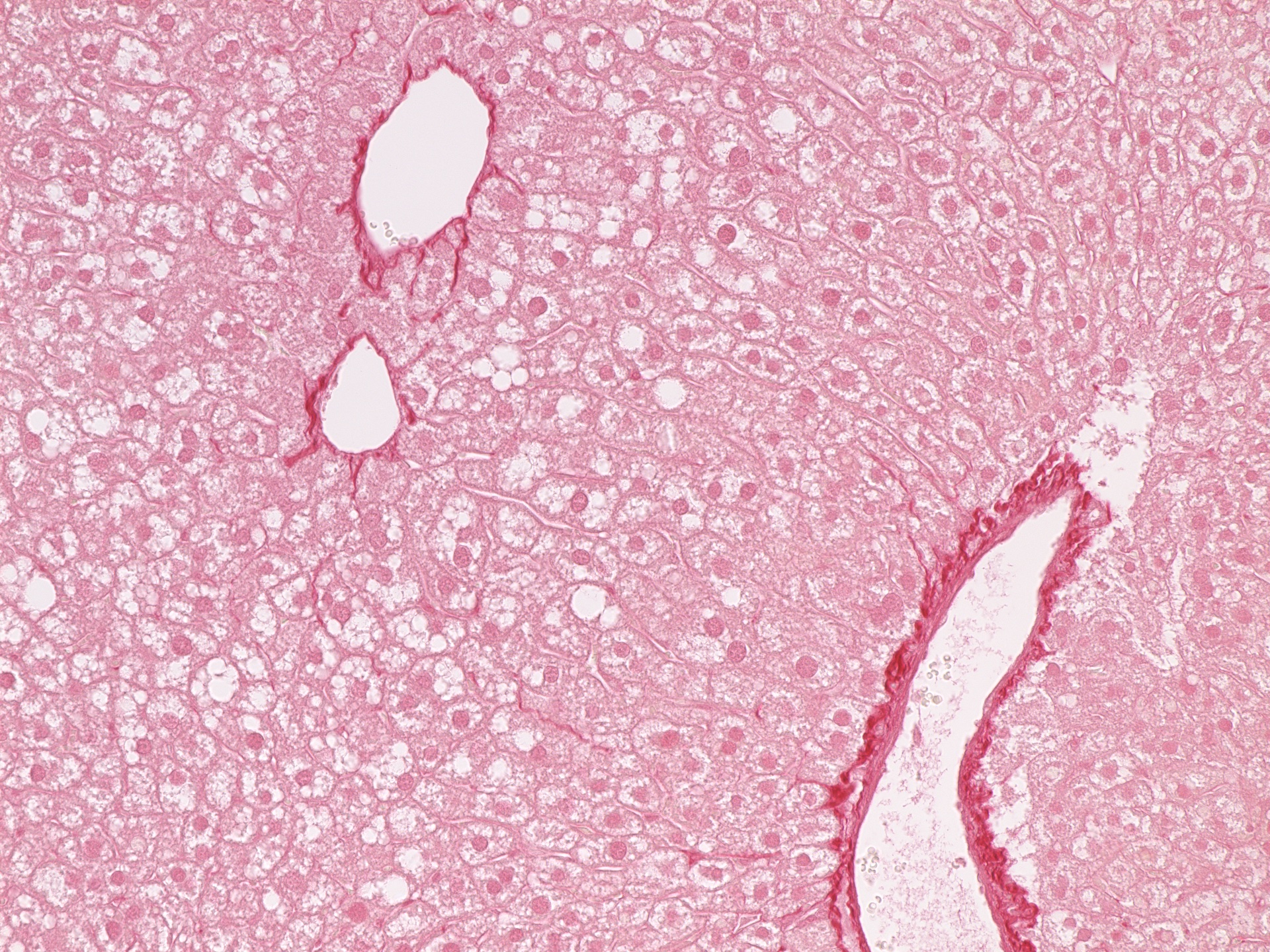

Supplement: Supplementary file 8 — Figure EV2 Source Data [file 44318_2024_196_MOESM8_ESM.zip › Figure EV2/Figure EV2-J/Quantificated image/NC Mock/no.2/NC-Mock-no.2-20x-2.jpg]

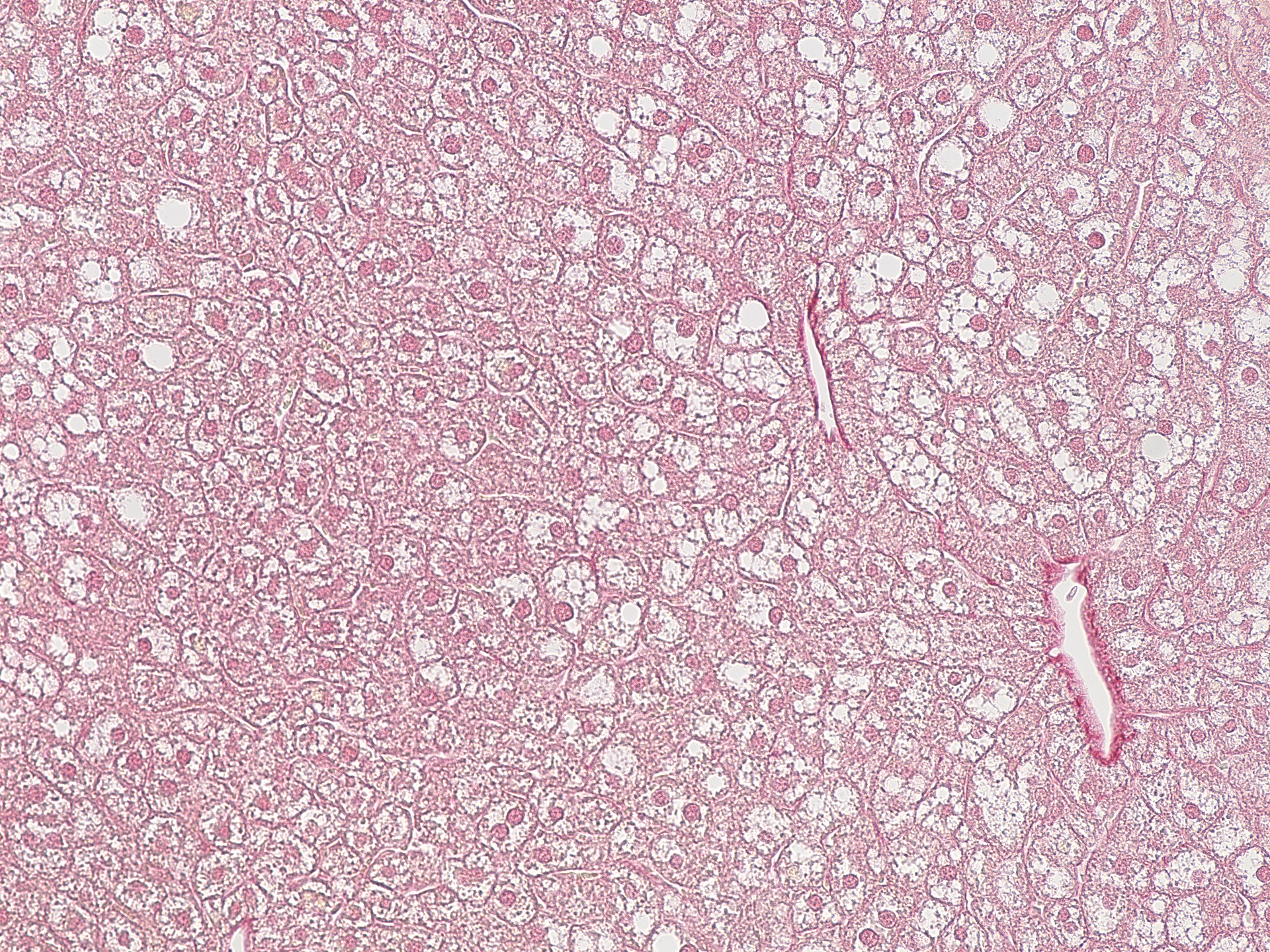

Supplement: Supplementary file 8 — Figure EV2 Source Data [file 44318_2024_196_MOESM8_ESM.zip › Figure EV2/Figure EV2-J/Quantificated image/NC Mock/no.2/NC-Mock-no.2-20x-3.jpg]

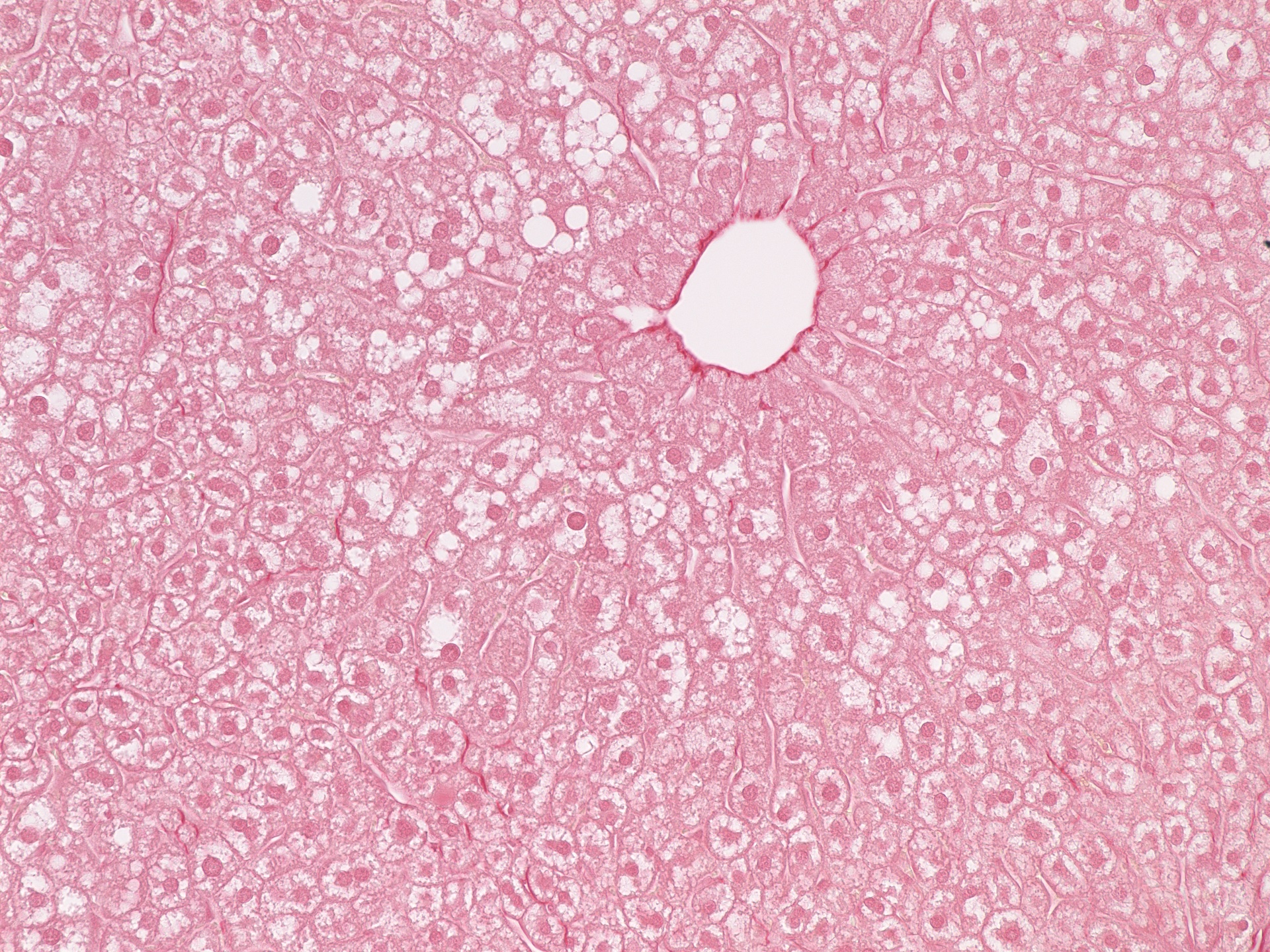

Supplement: Supplementary file 8 — Figure EV2 Source Data [file 44318_2024_196_MOESM8_ESM.zip › Figure EV2/Figure EV2-J/Quantificated image/NC Mock/no.2/NC-Mock-no.2-20x-1.jpg]

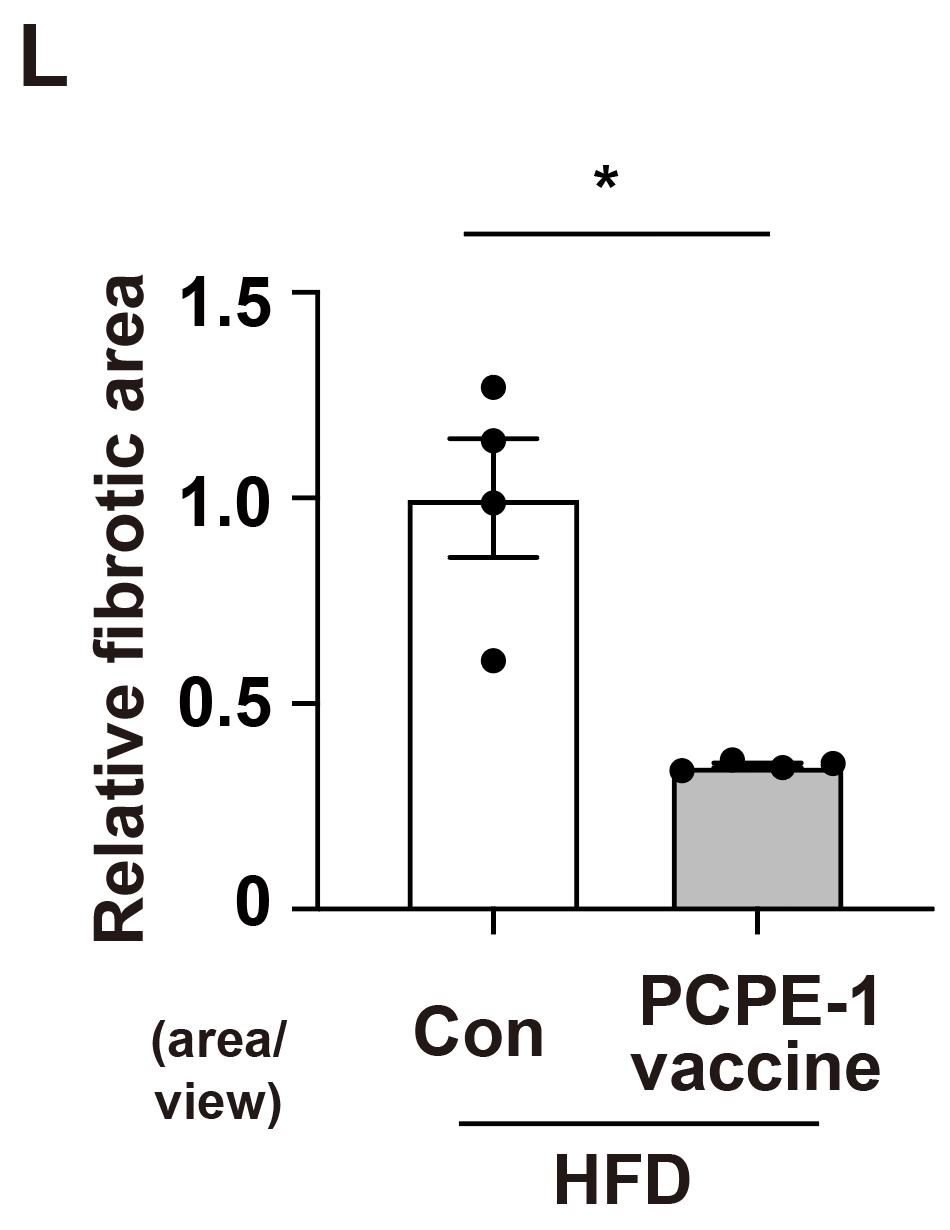

Supplement: Supplementary file 9 — Figure EV3 Source Data [file 44318_2024_196_MOESM9_ESM.zip › Figure EV3/Figure EV3-L/Fig.EV3L.png]

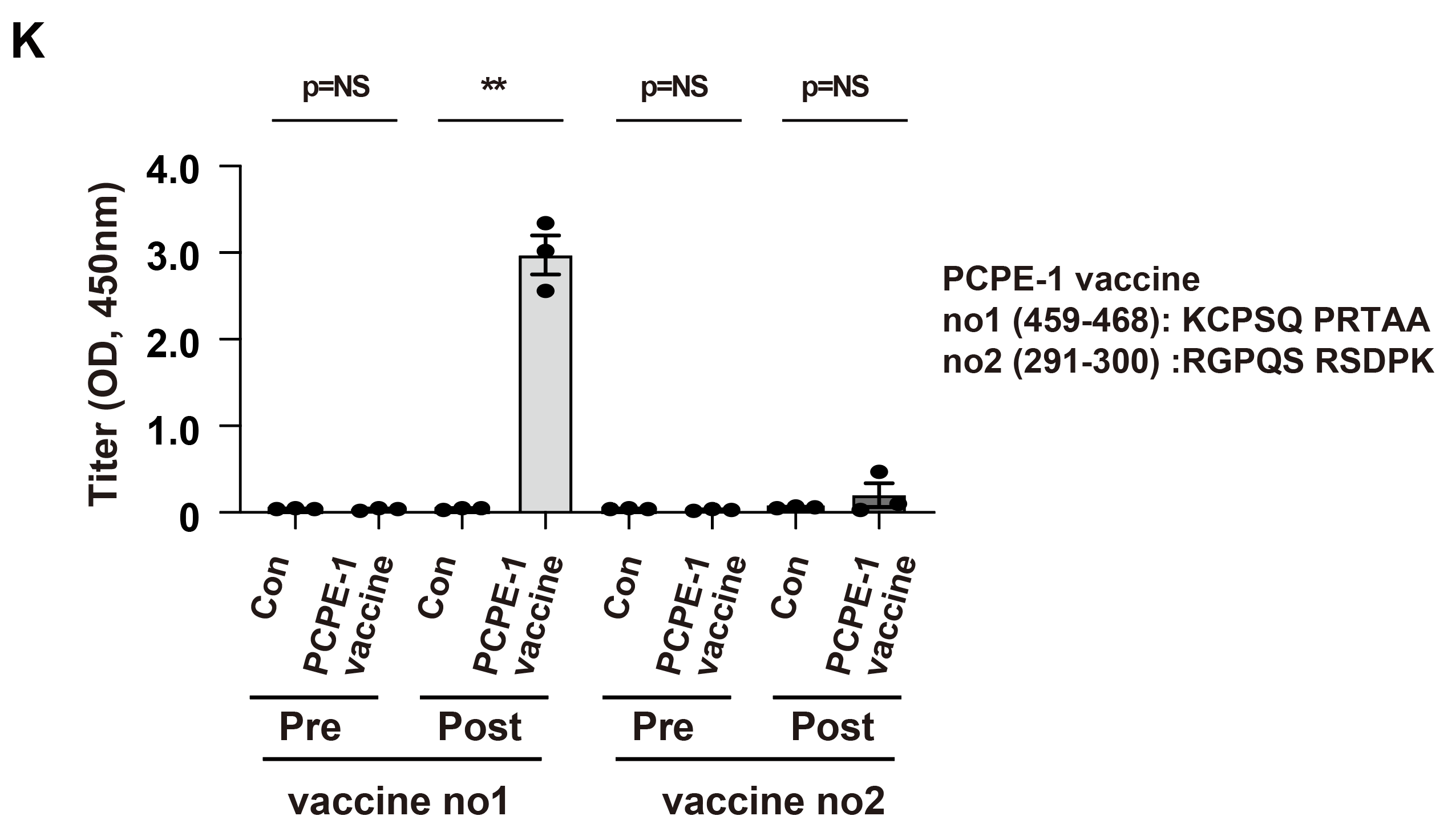

Supplement: Supplementary file 9 — Figure EV3 Source Data [file 44318_2024_196_MOESM9_ESM.zip › Figure EV3/Figure EV3-K/Fig.EV3K.png]

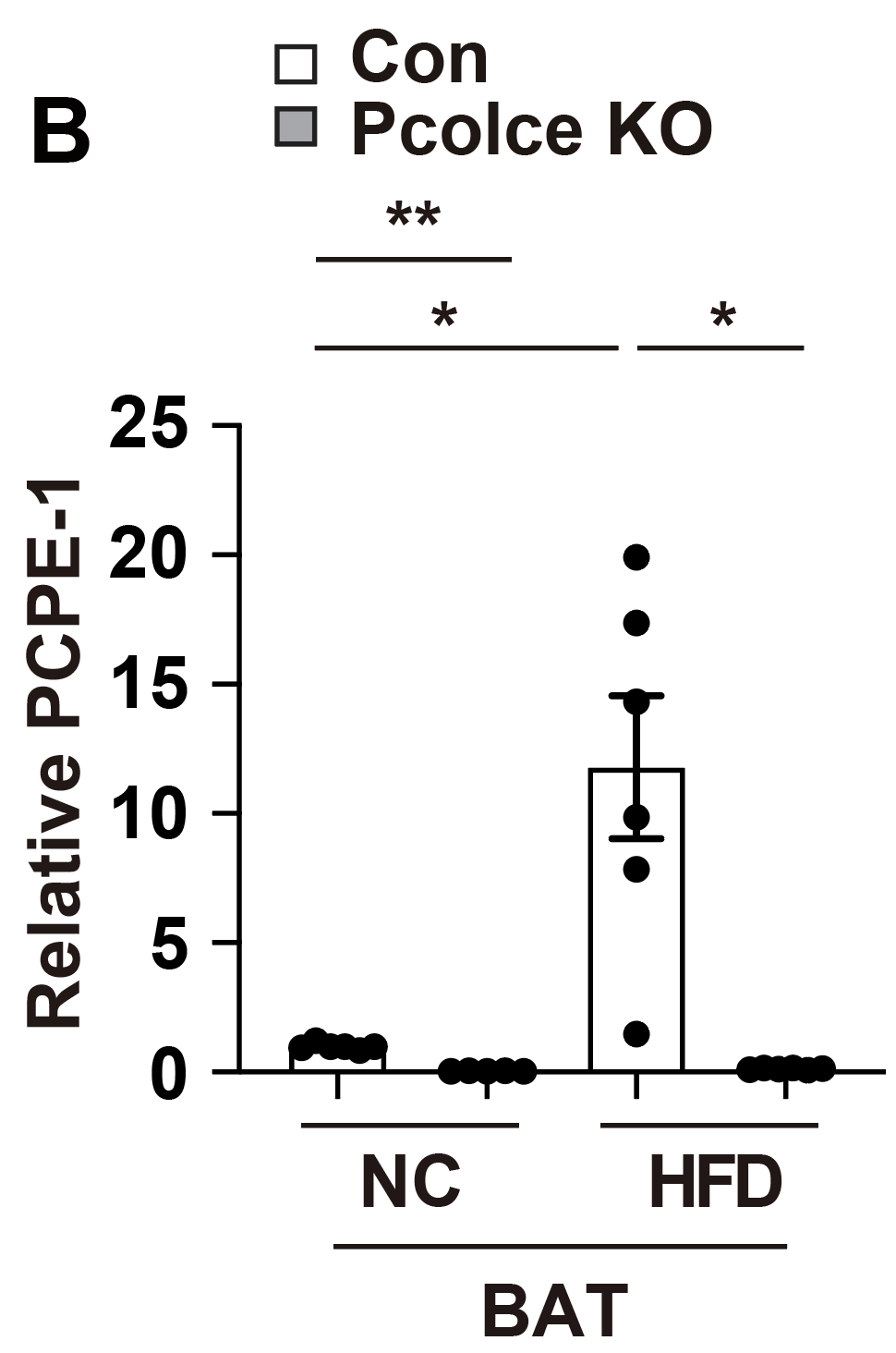

Supplement: Supplementary file 9 — Figure EV3 Source Data [file 44318_2024_196_MOESM9_ESM.zip › Figure EV3/Figure EV3-B/Fig.EV3B.png]

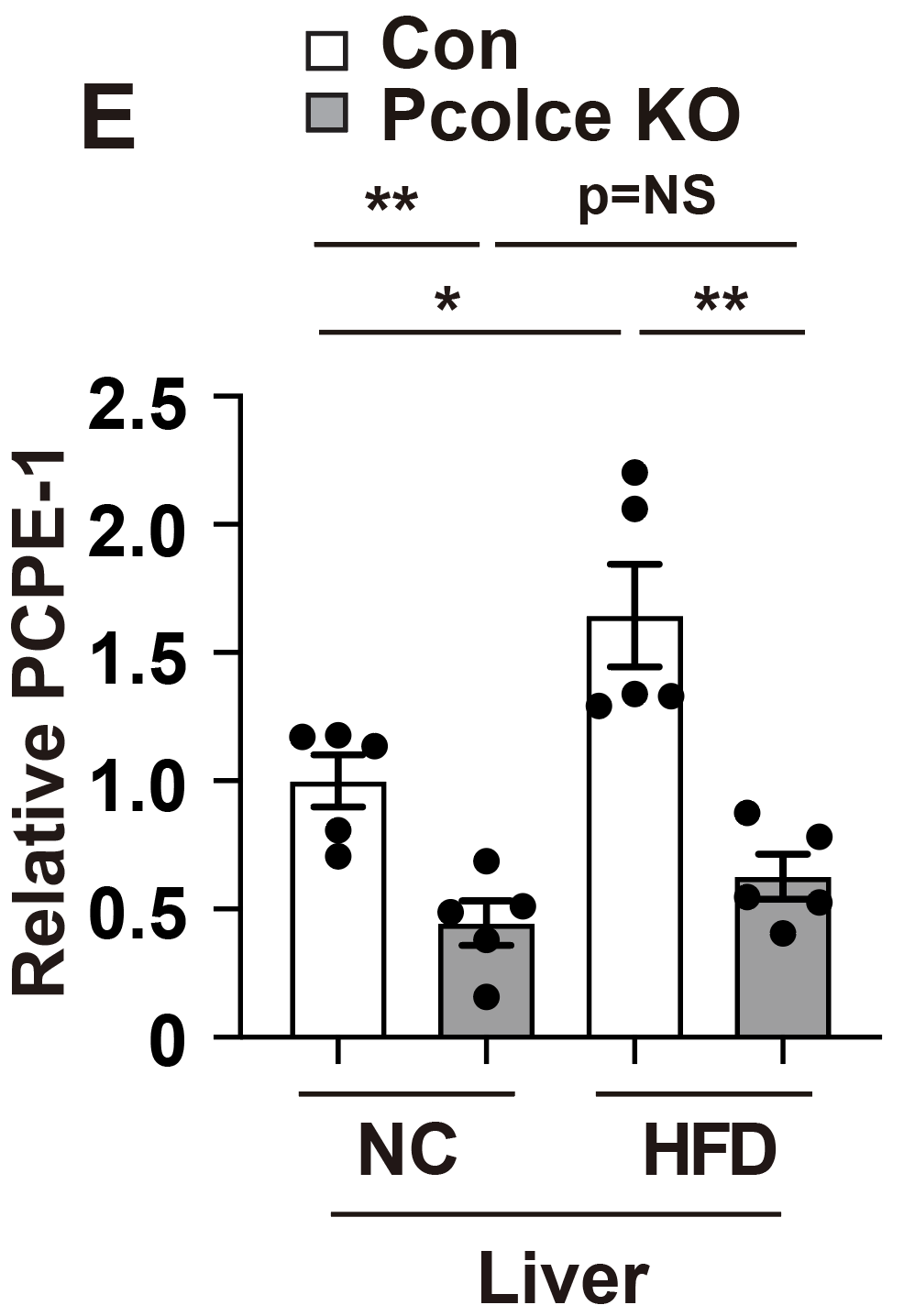

Supplement: Supplementary file 9 — Figure EV3 Source Data [file 44318_2024_196_MOESM9_ESM.zip › Figure EV3/Figure EV3-E/Fig.EV3E.png]

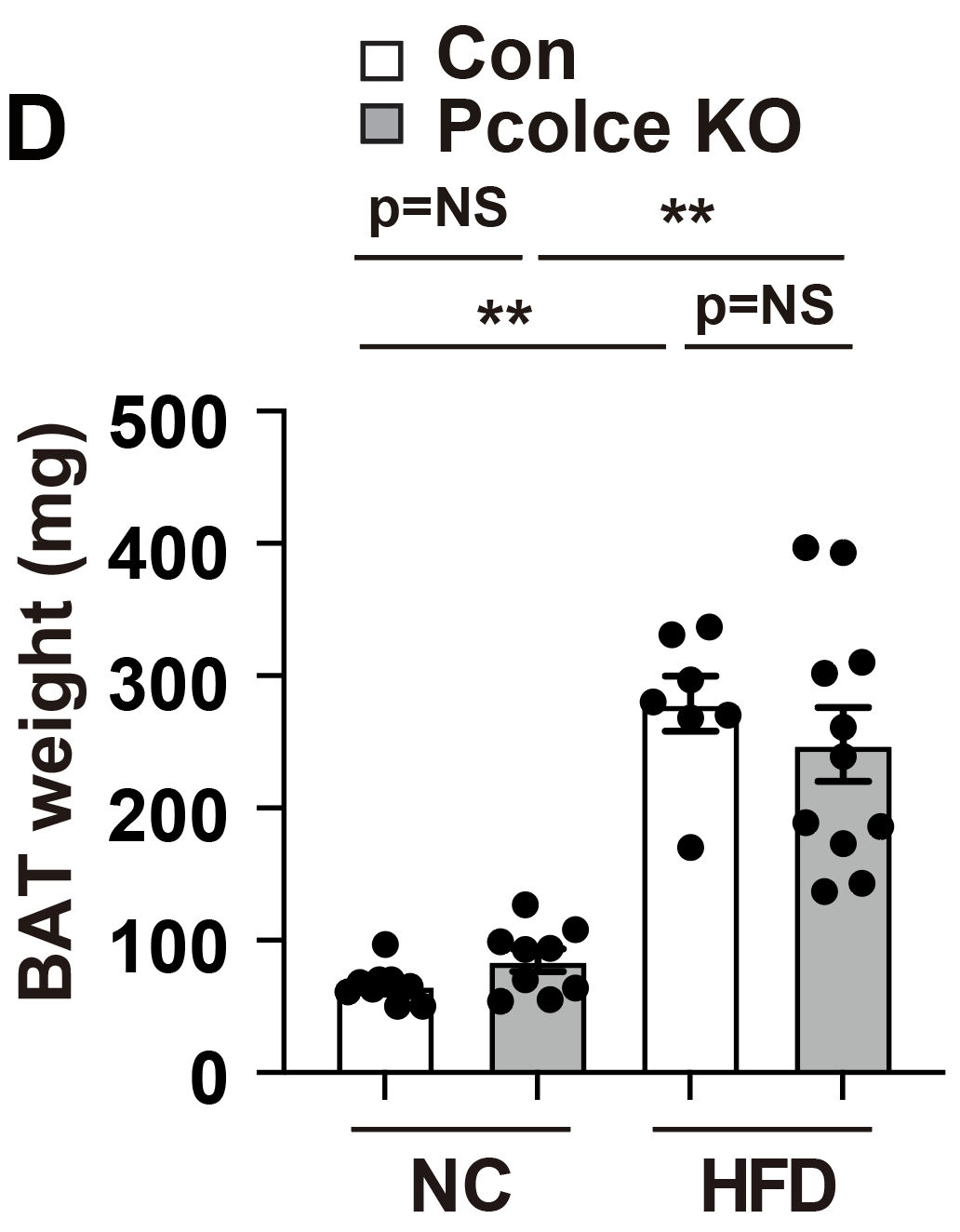

Supplement: Supplementary file 9 — Figure EV3 Source Data [file 44318_2024_196_MOESM9_ESM.zip › Figure EV3/Figure EV3-D/Fig.EV3D.png]

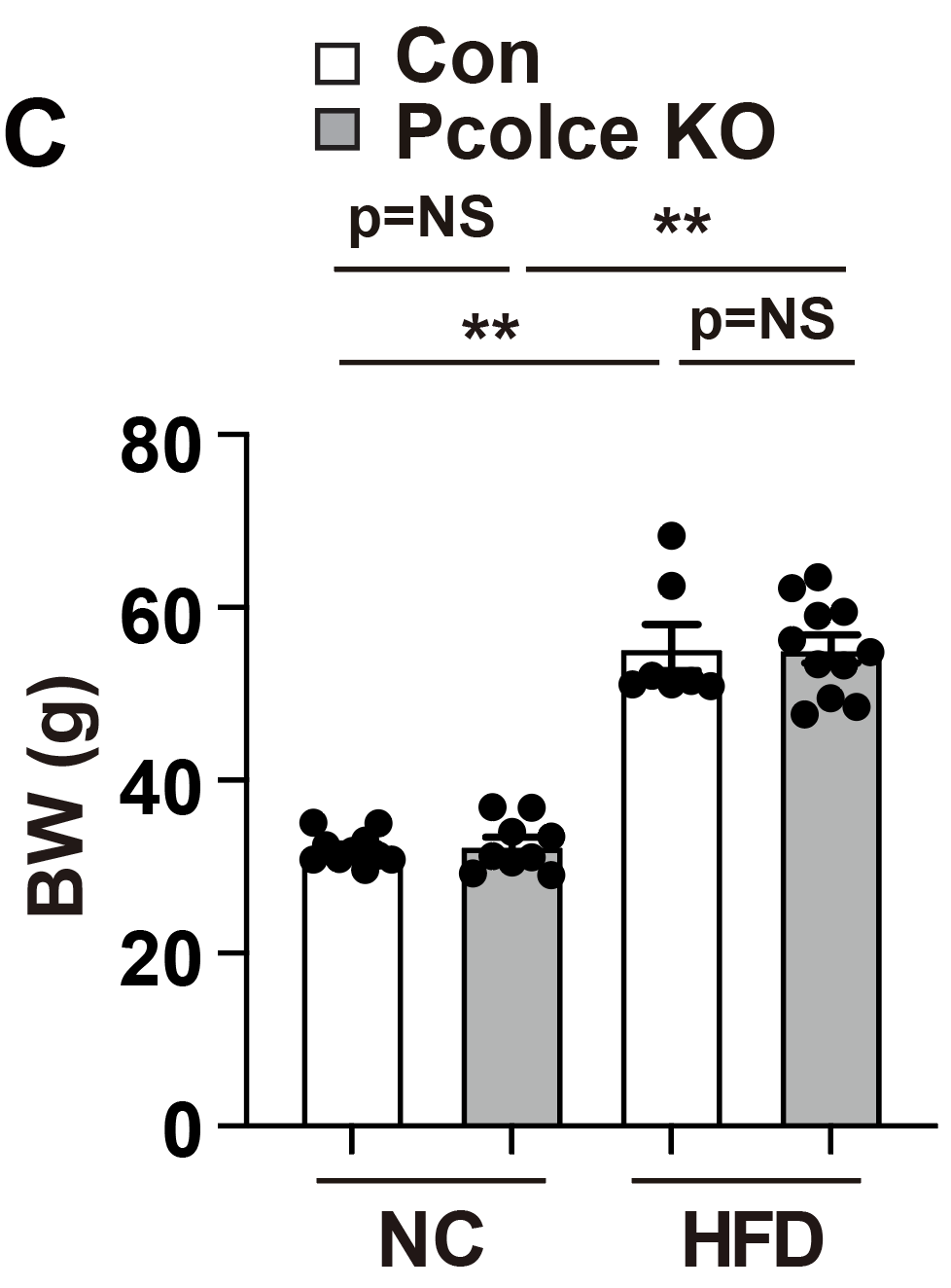

Supplement: Supplementary file 9 — Figure EV3 Source Data [file 44318_2024_196_MOESM9_ESM.zip › Figure EV3/Figure EV3-C/Fig.EV3C.png]

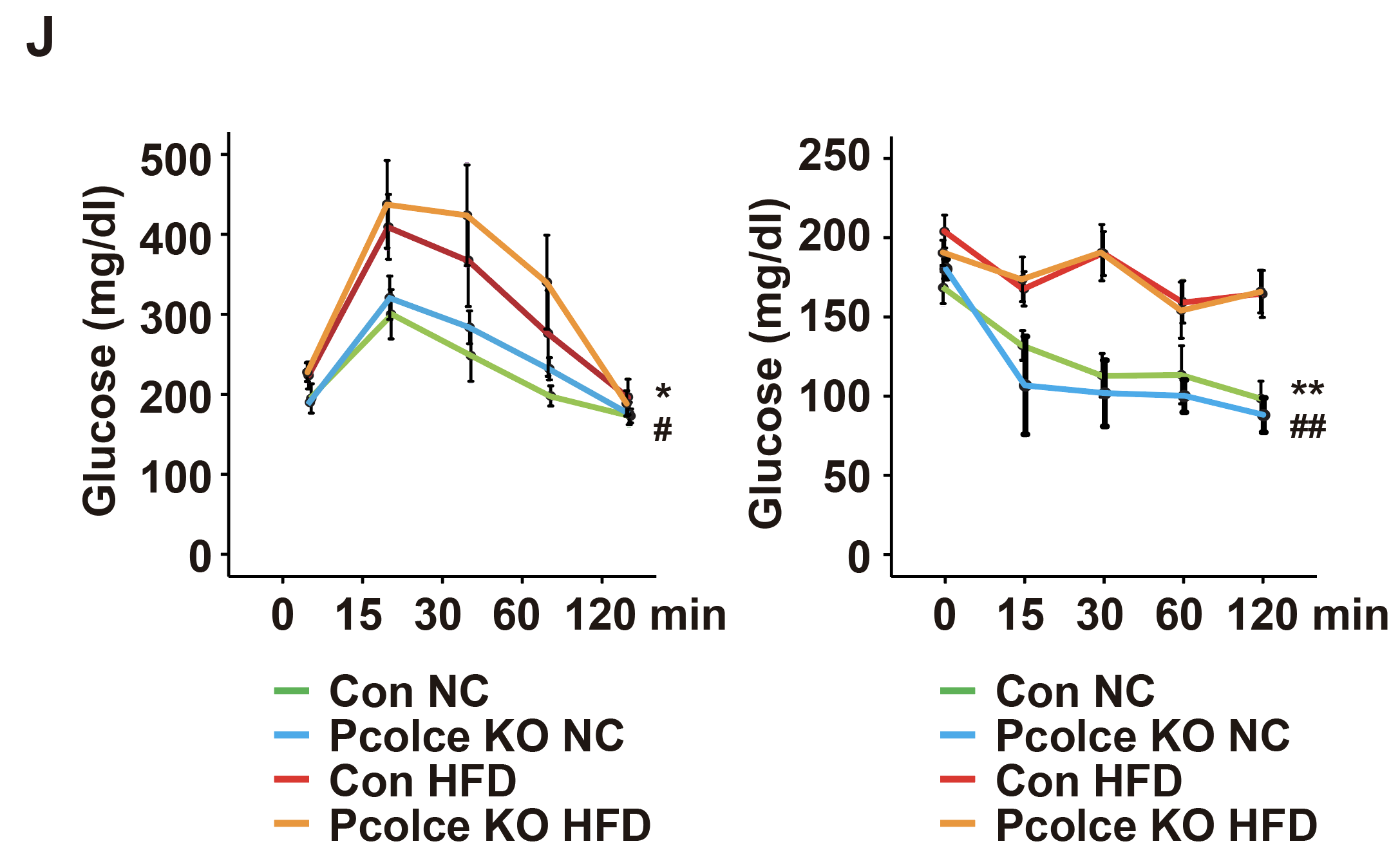

Supplement: Supplementary file 9 — Figure EV3 Source Data [file 44318_2024_196_MOESM9_ESM.zip › Figure EV3/Figure EV3-J/Fig.EV3J.png]

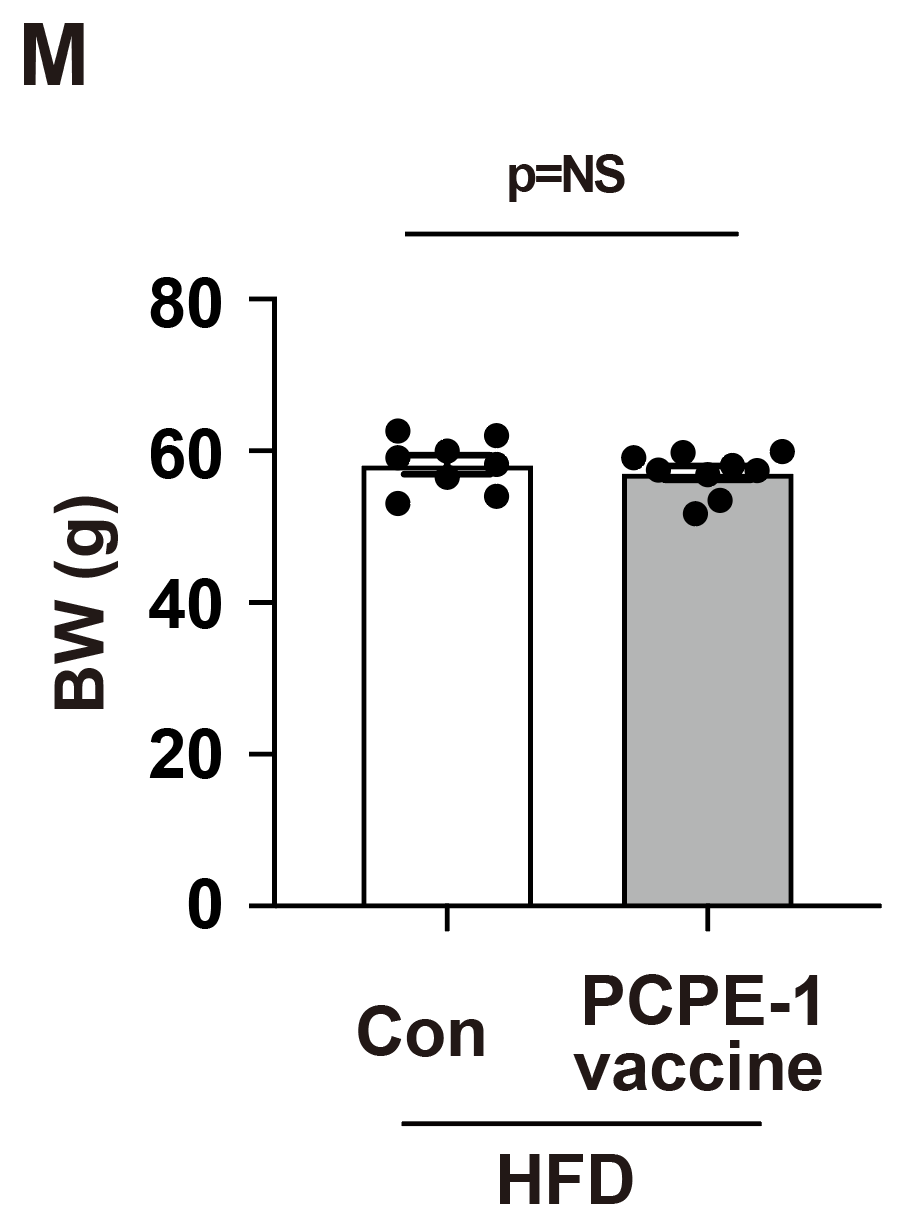

Supplement: Supplementary file 9 — Figure EV3 Source Data [file 44318_2024_196_MOESM9_ESM.zip › Figure EV3/Figure EV3-M/Fig.EV3M.png]

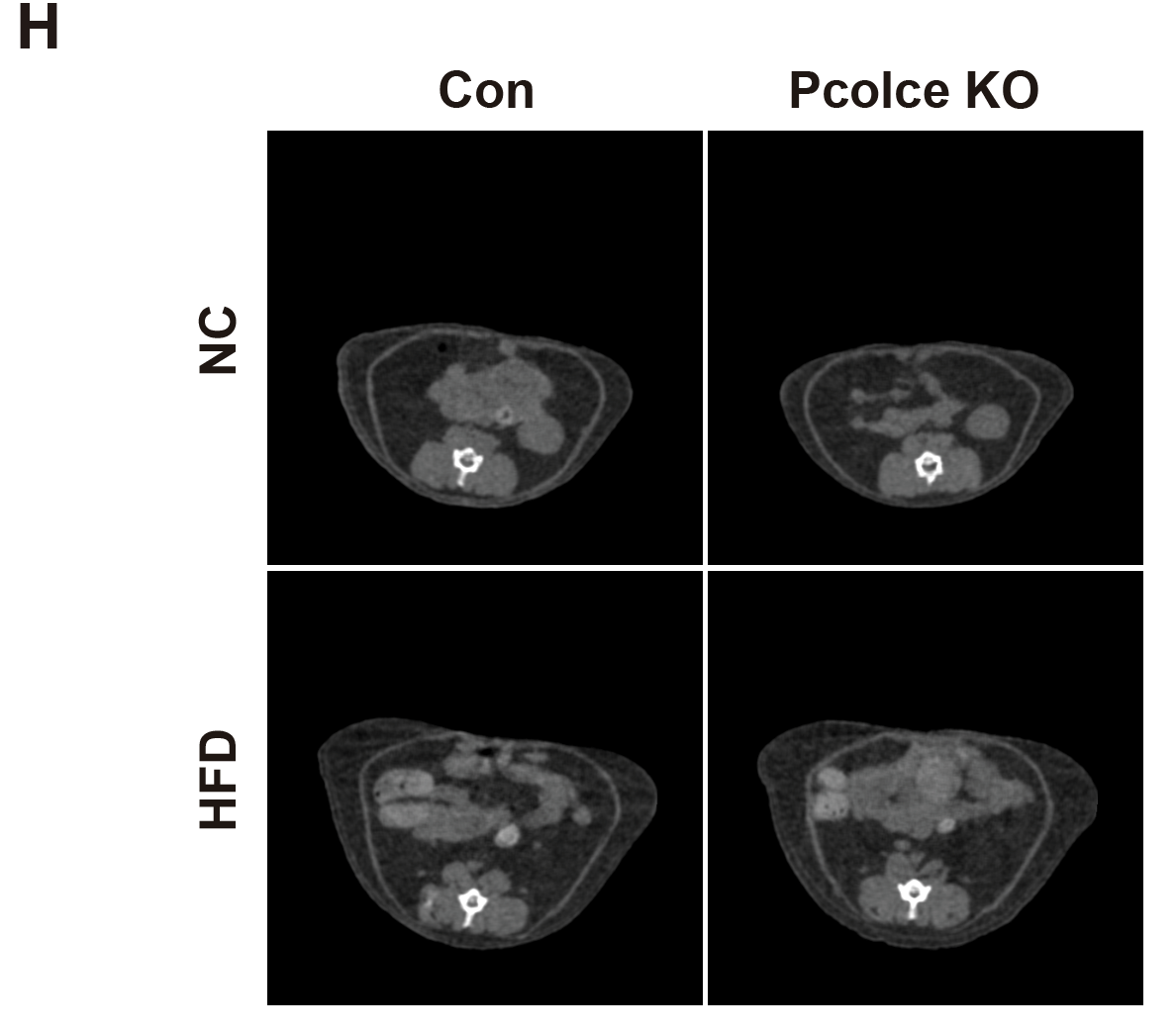

Supplement: Supplementary file 9 — Figure EV3 Source Data [file 44318_2024_196_MOESM9_ESM.zip › Figure EV3/Figure EV3-H/Fig.EV3H.png]

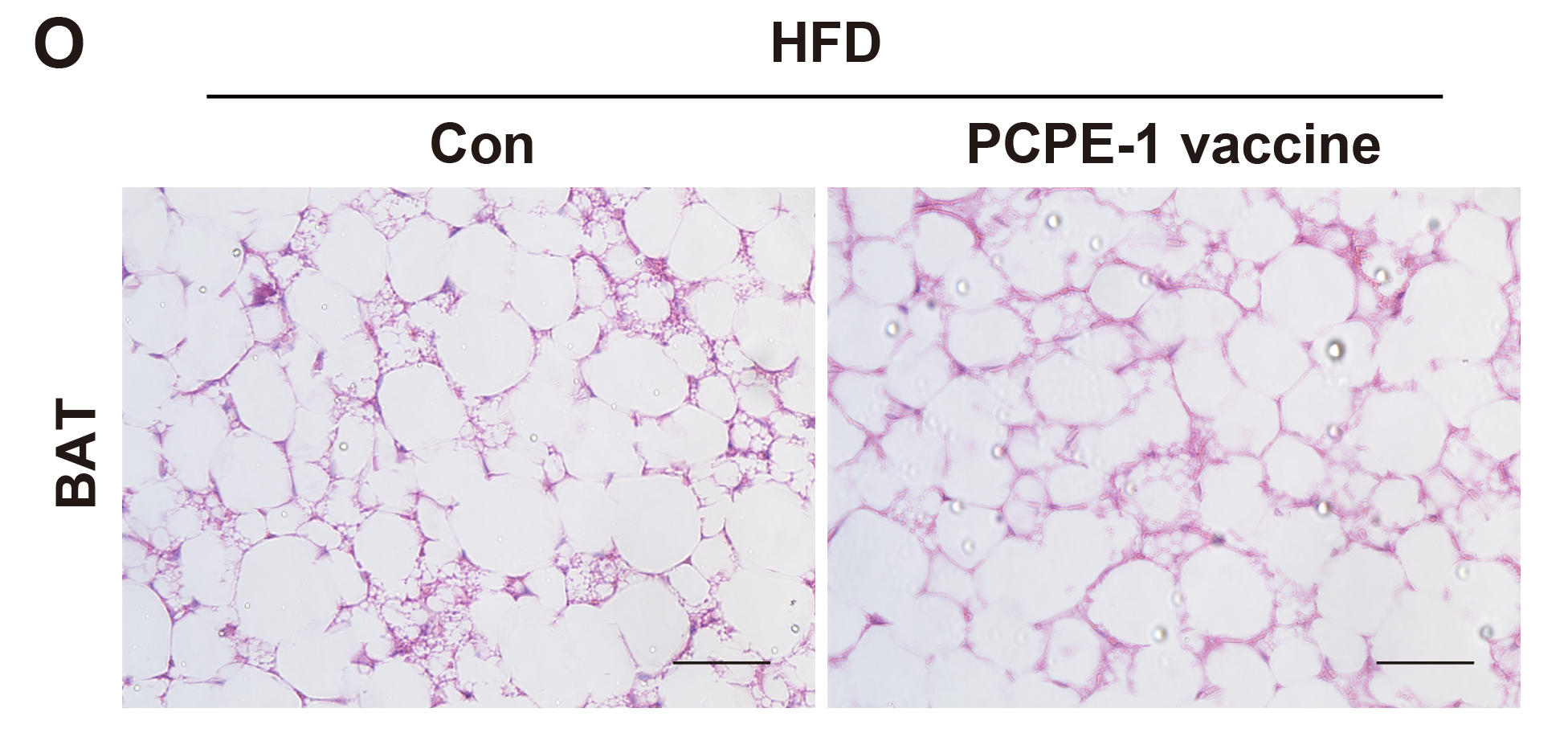

Supplement: Supplementary file 9 — Figure EV3 Source Data [file 44318_2024_196_MOESM9_ESM.zip › Figure EV3/Figure EV3-O/Fig.EV3O.png]

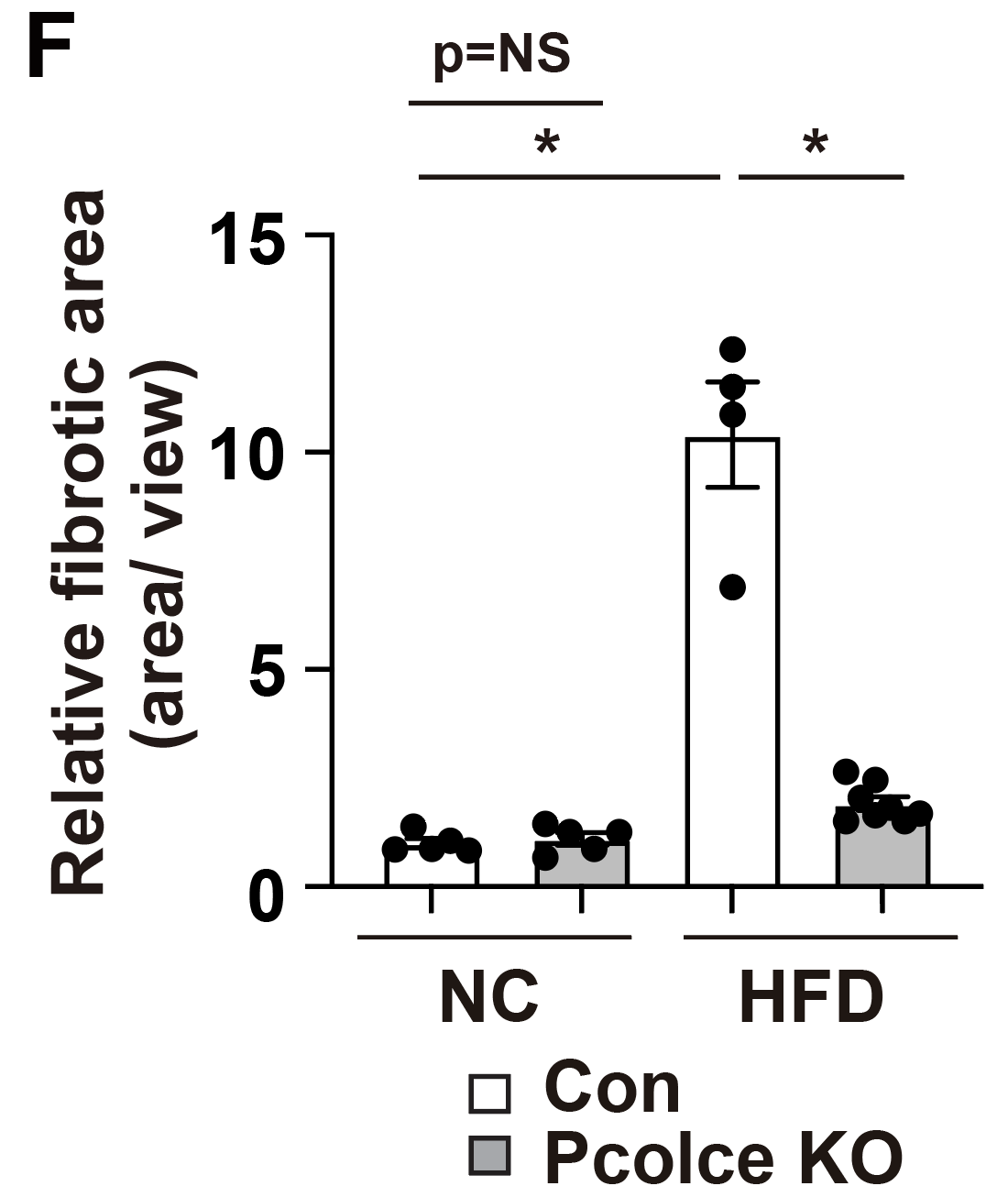

Supplement: Supplementary file 9 — Figure EV3 Source Data [file 44318_2024_196_MOESM9_ESM.zip › Figure EV3/Figure EV3-F/Fig.EV3F.png]

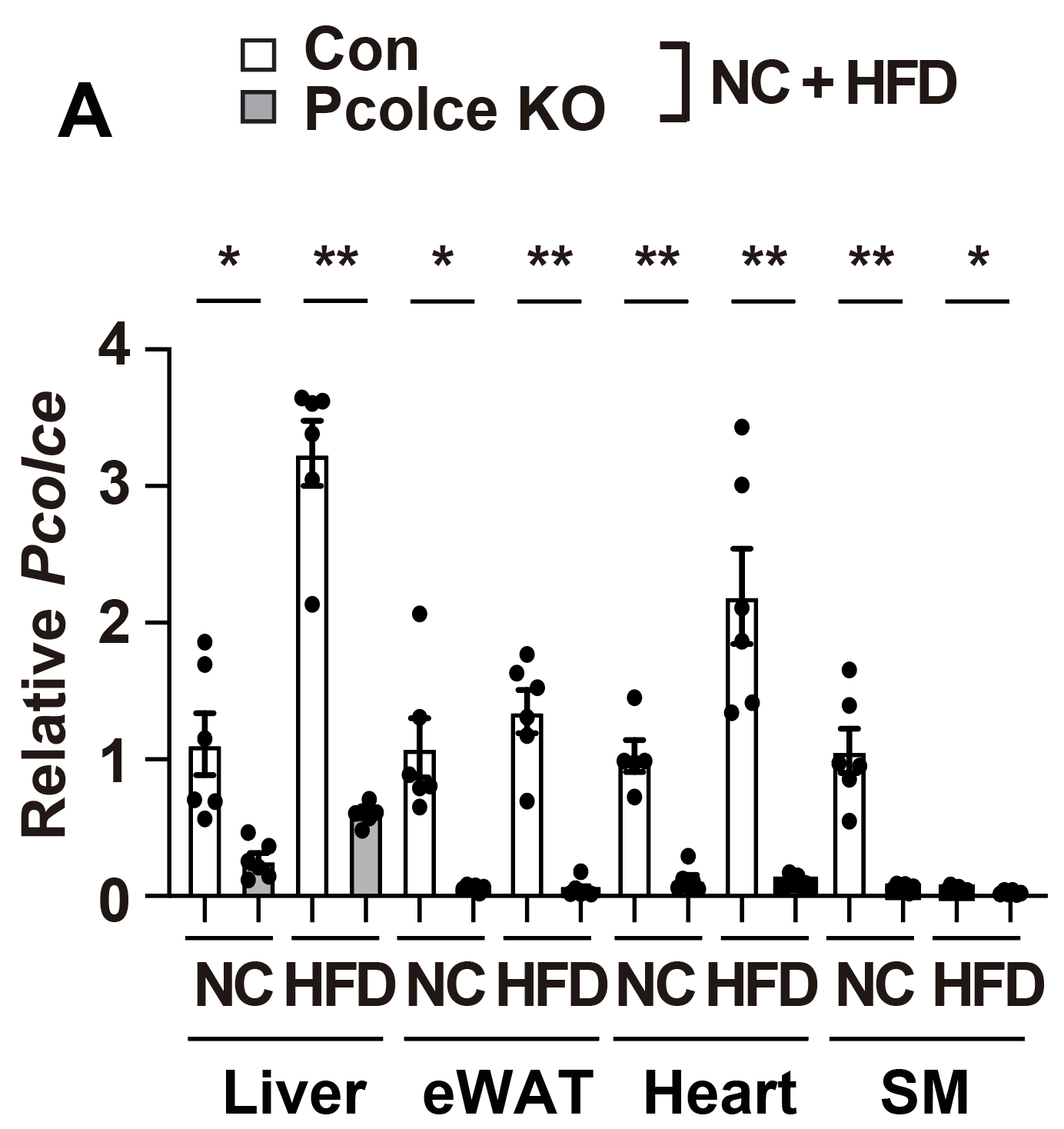

Supplement: Supplementary file 9 — Figure EV3 Source Data [file 44318_2024_196_MOESM9_ESM.zip › Figure EV3/Figure EV3-A/Fig.EV3A.png]

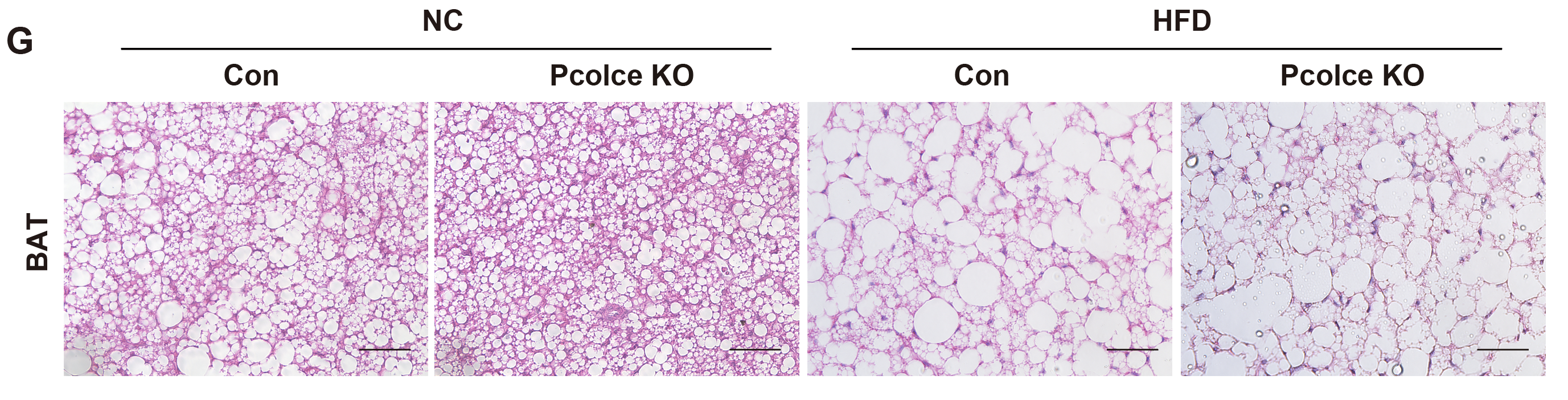

Supplement: Supplementary file 9 — Figure EV3 Source Data [file 44318_2024_196_MOESM9_ESM.zip › Figure EV3/Figure EV3-G/Fig.EV3G.png]

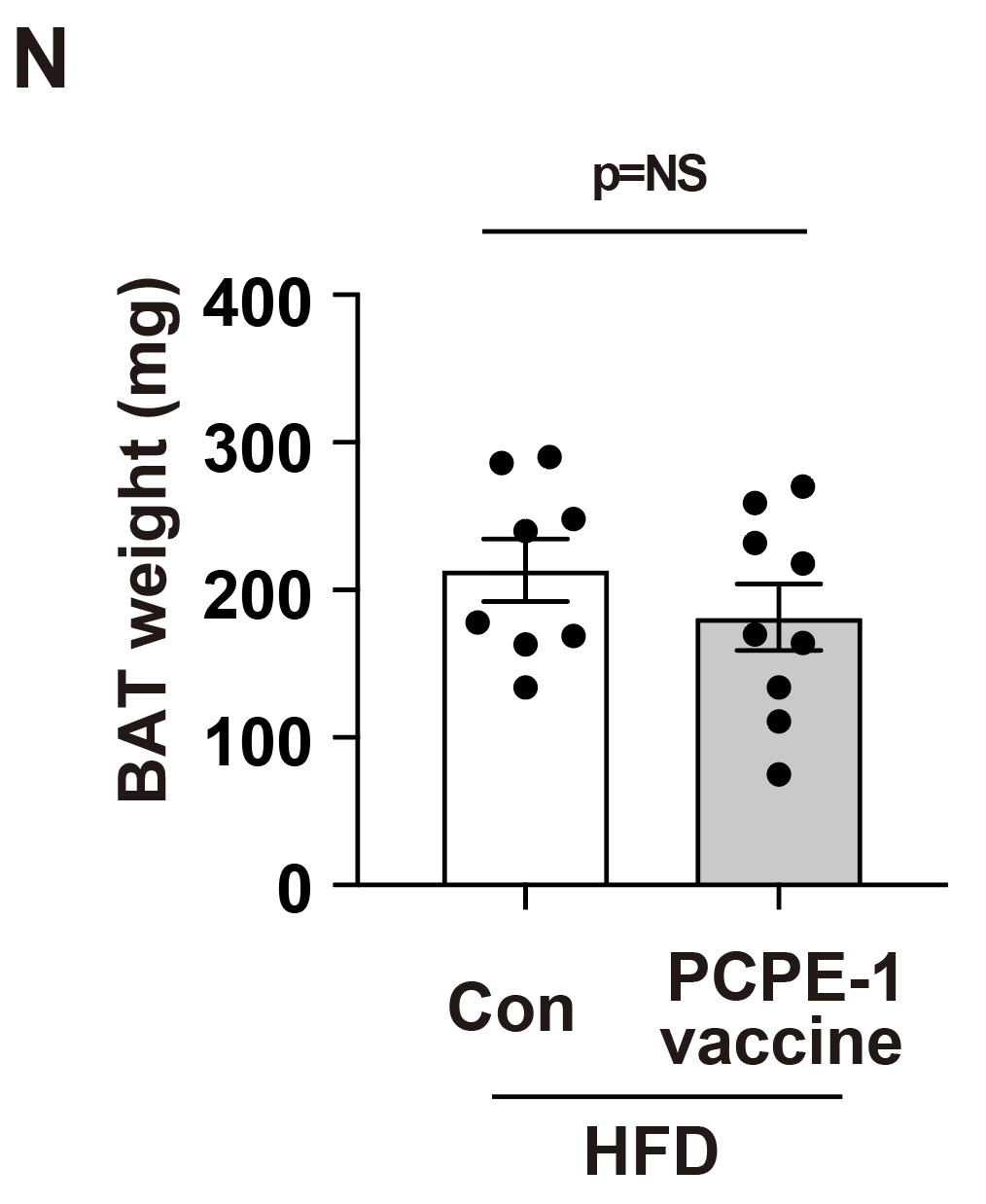

Supplement: Supplementary file 9 — Figure EV3 Source Data [file 44318_2024_196_MOESM9_ESM.zip › Figure EV3/Figure EV3-N/Fig.EV3N.png]

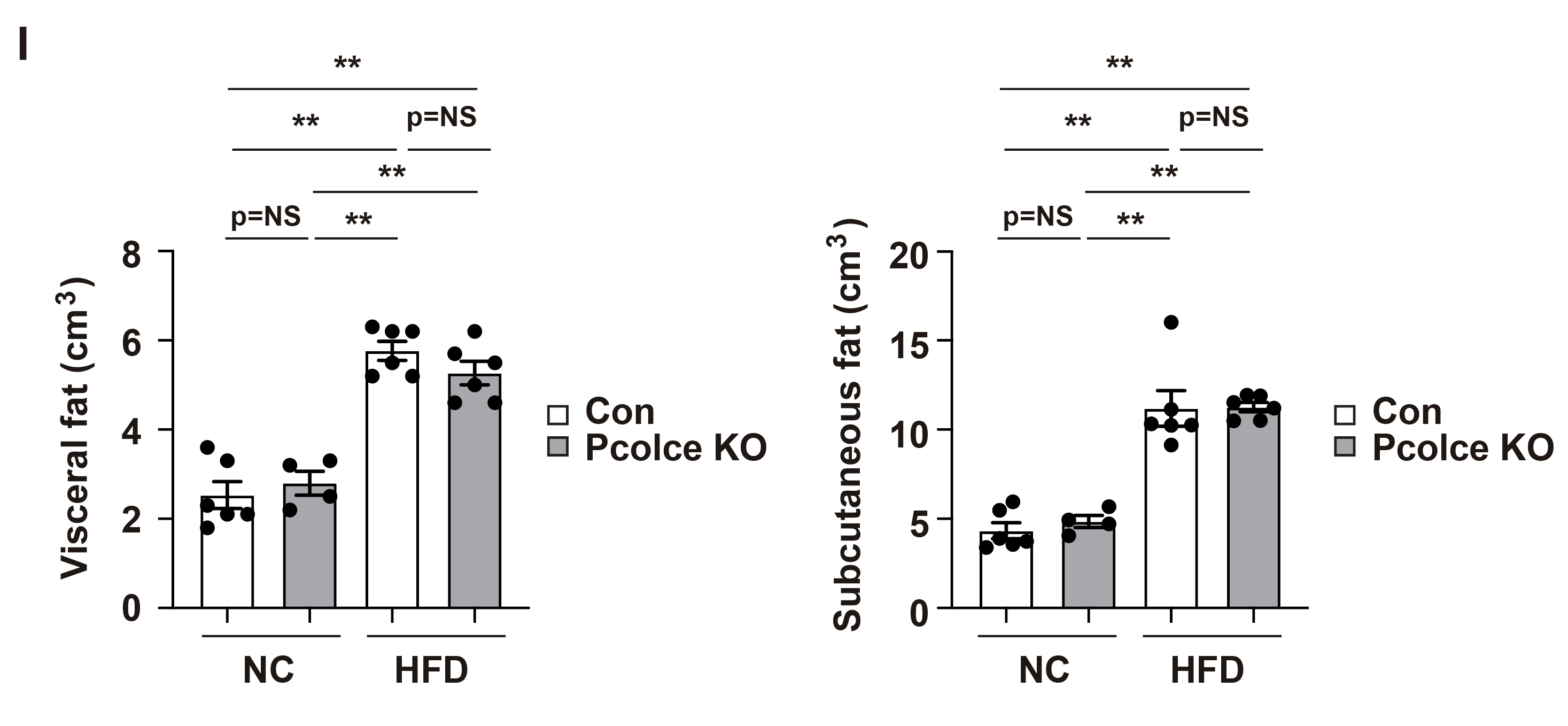

Supplement: Supplementary file 9 — Figure EV3 Source Data [file 44318_2024_196_MOESM9_ESM.zip › Figure EV3/Figure EV3-I/Fig.EV3I.png]

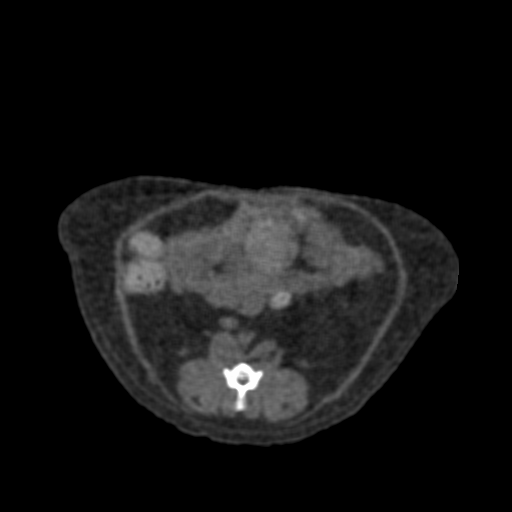

Supplement: Supplementary file 9 — Figure EV3 Source Data [file 44318_2024_196_MOESM9_ESM.zip › Figure EV3/Figure EV3-H/Demonstrated image/HFD Pcolce KO.BMP]

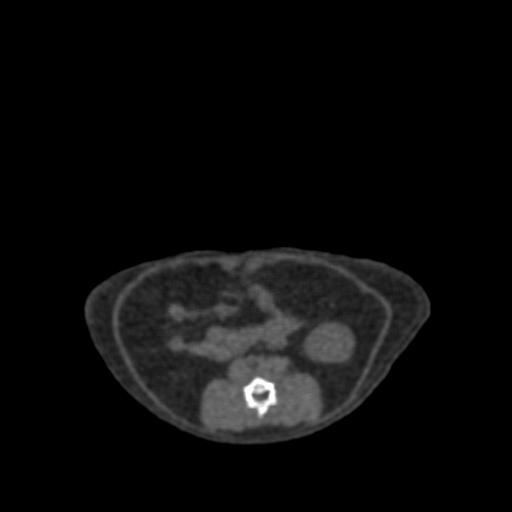

Supplement: Supplementary file 9 — Figure EV3 Source Data [file 44318_2024_196_MOESM9_ESM.zip › Figure EV3/Figure EV3-H/Demonstrated image/NC Pcolce KO.BMP]

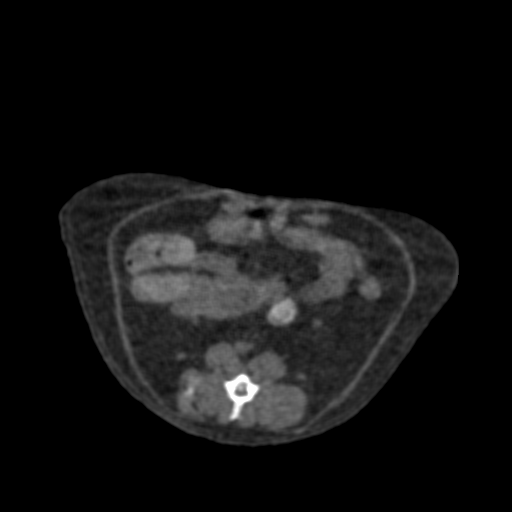

Supplement: Supplementary file 9 — Figure EV3 Source Data [file 44318_2024_196_MOESM9_ESM.zip › Figure EV3/Figure EV3-H/Demonstrated image/HFD Con.BMP]

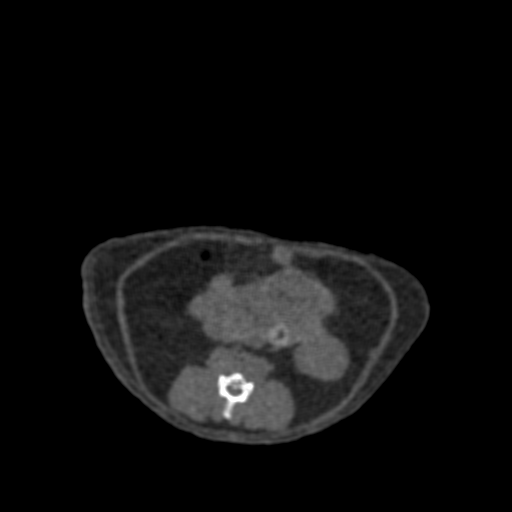

Supplement: Supplementary file 9 — Figure EV3 Source Data [file 44318_2024_196_MOESM9_ESM.zip › Figure EV3/Figure EV3-H/Demonstrated image/NC Con.BMP]

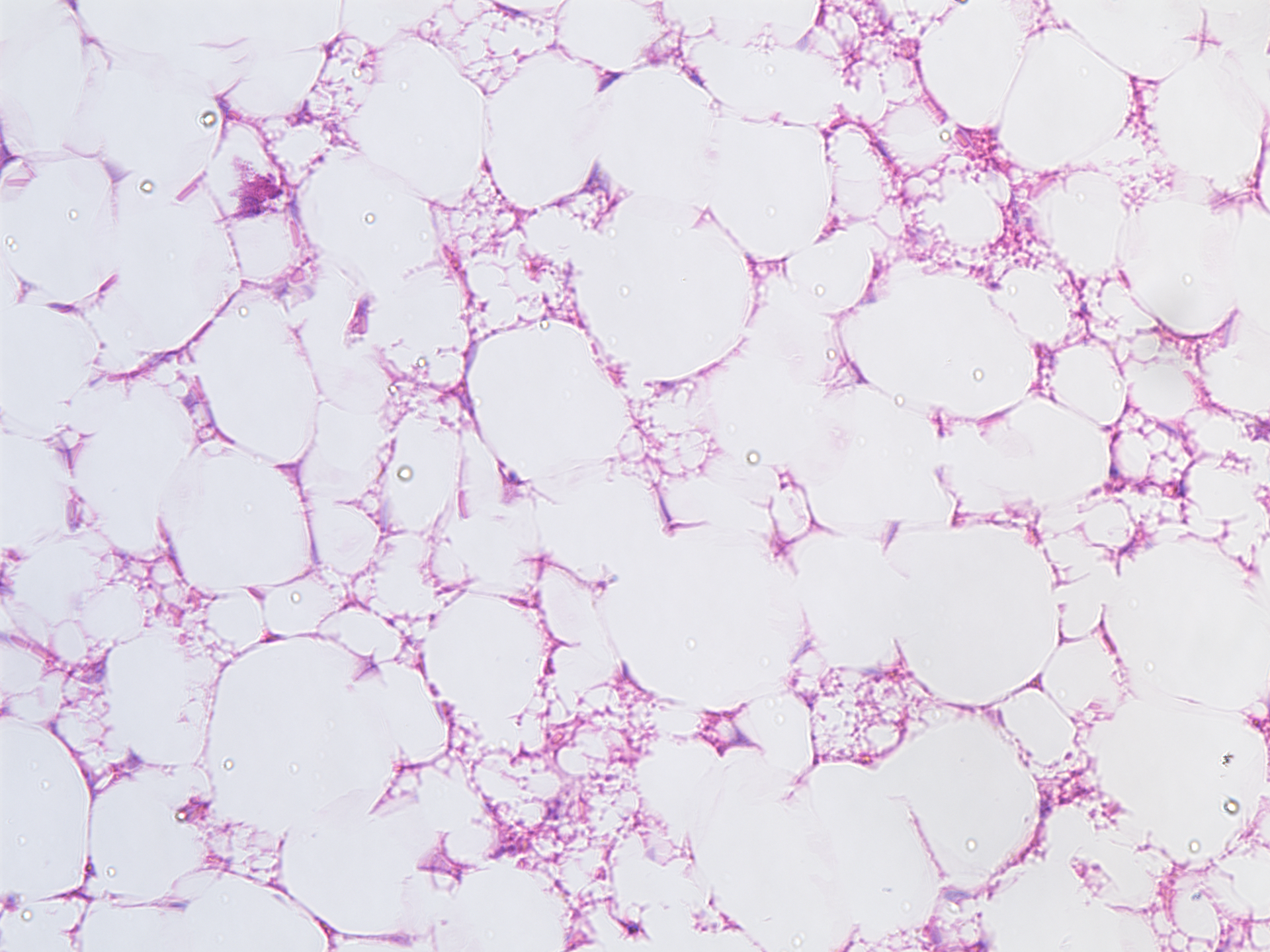

Supplement: Supplementary file 9 — Figure EV3 Source Data [file 44318_2024_196_MOESM9_ESM.zip › Figure EV3/Figure EV3-O/Demonstrated image/Con HFD.tif]

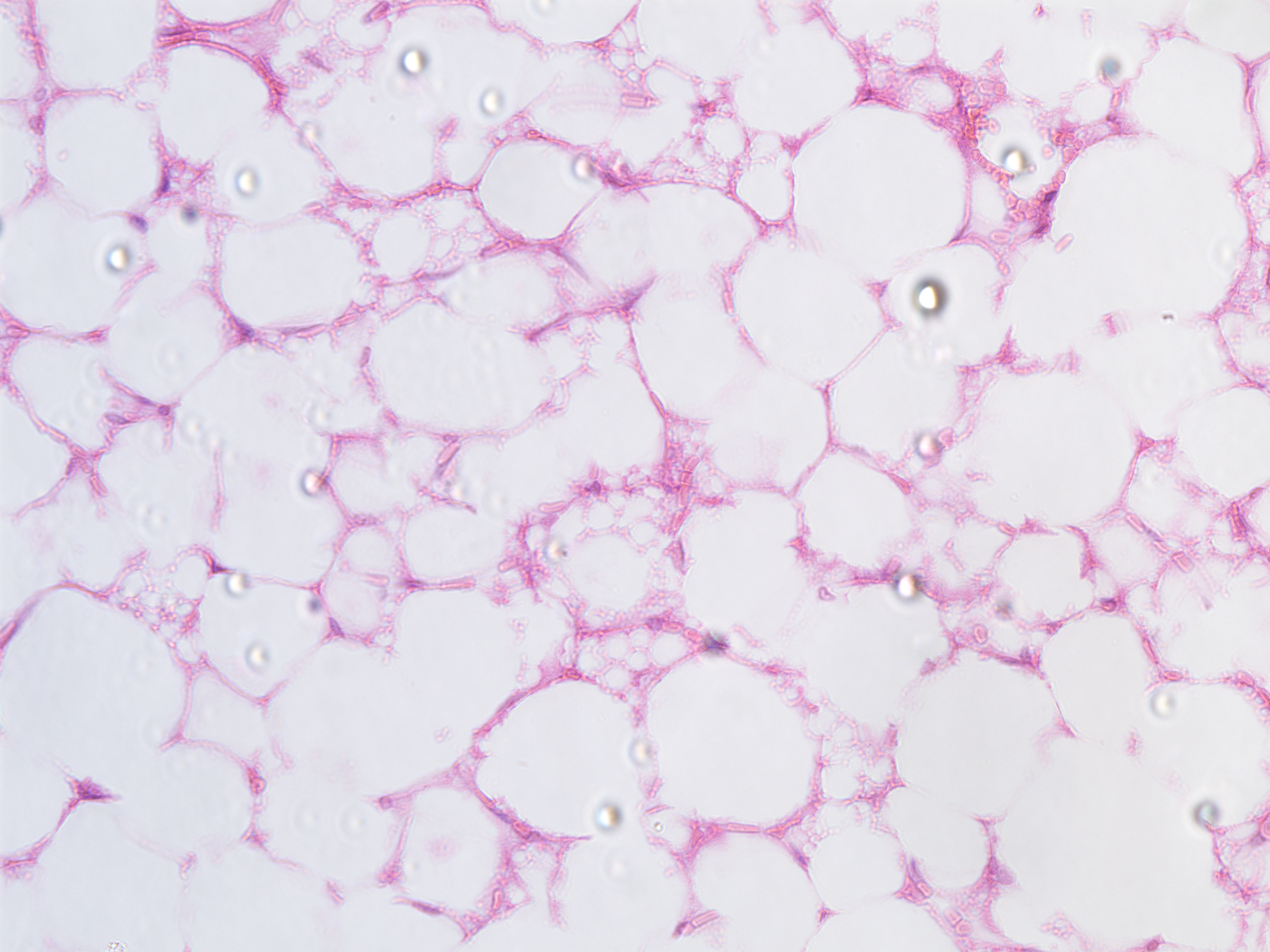

Supplement: Supplementary file 9 — Figure EV3 Source Data [file 44318_2024_196_MOESM9_ESM.zip › Figure EV3/Figure EV3-O/Demonstrated image/PCPE1 vaccine HFD.tif]

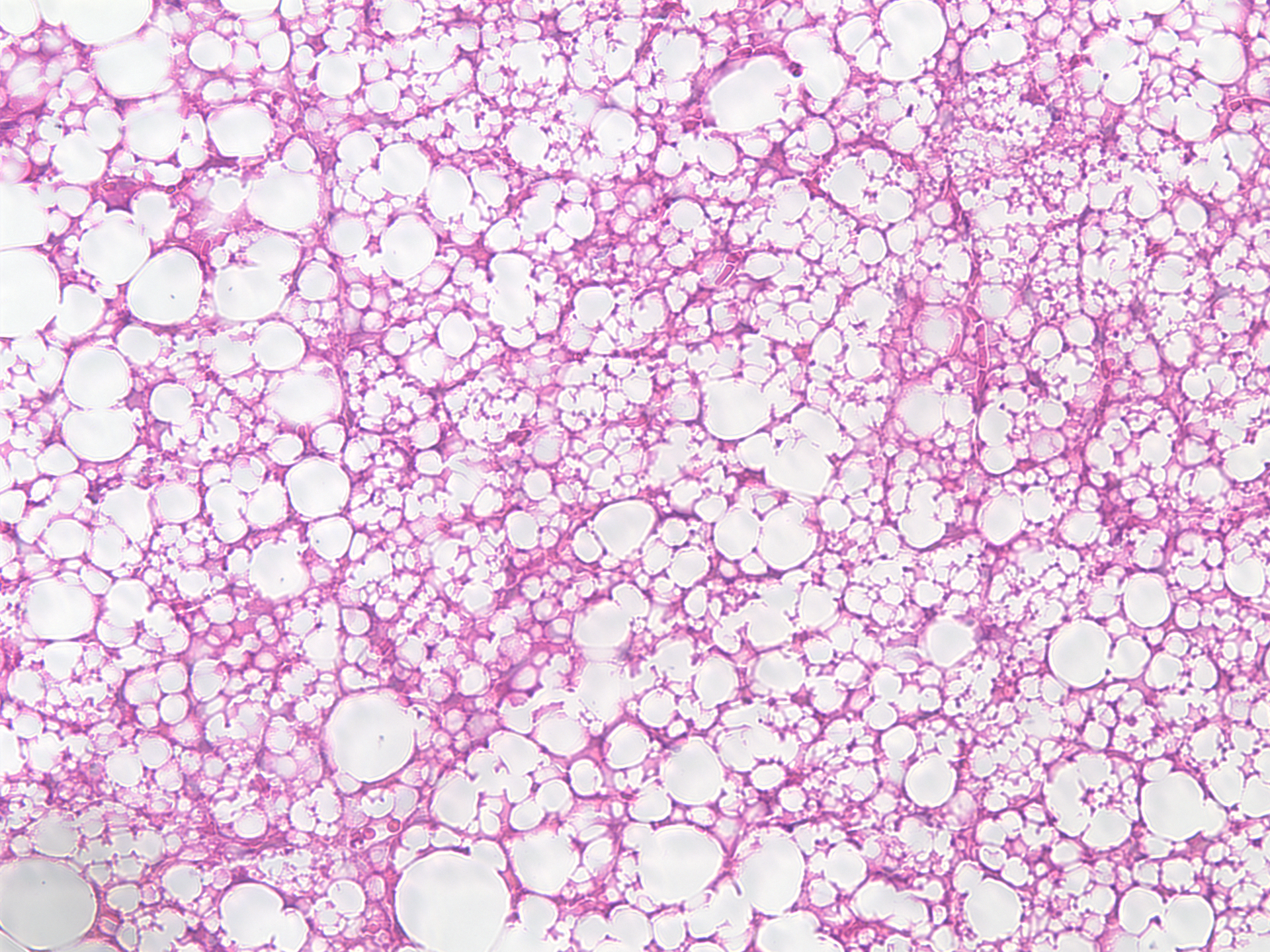

Supplement: Supplementary file 9 — Figure EV3 Source Data [file 44318_2024_196_MOESM9_ESM.zip › Figure EV3/Figure EV3-G/Demonstrated image/NC Con.tif]

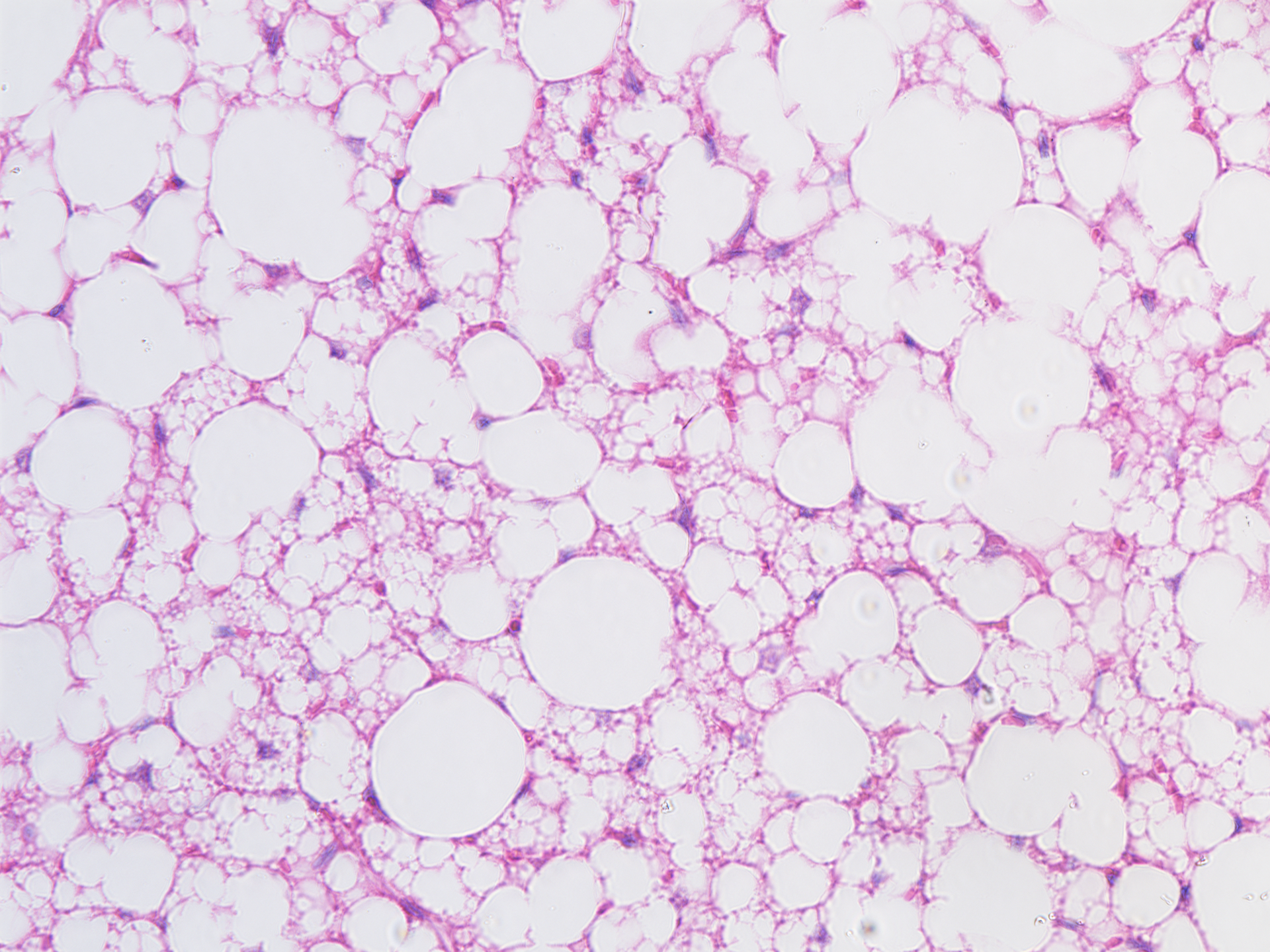

Supplement: Supplementary file 9 — Figure EV3 Source Data [file 44318_2024_196_MOESM9_ESM.zip › Figure EV3/Figure EV3-G/Demonstrated image/HFD Con.tif]

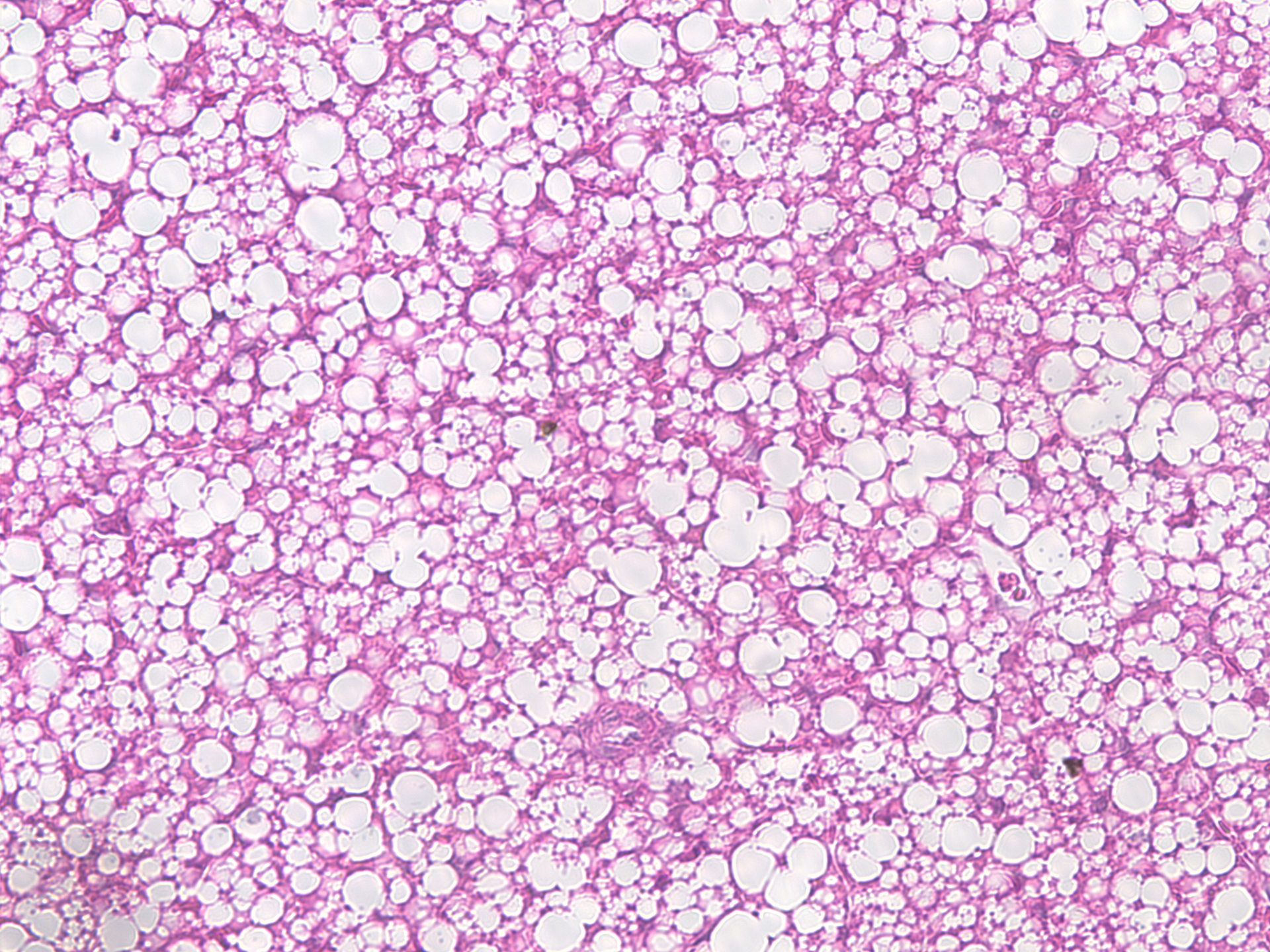

Supplement: Supplementary file 9 — Figure EV3 Source Data [file 44318_2024_196_MOESM9_ESM.zip › Figure EV3/Figure EV3-G/Demonstrated image/NC Pcolce KO.tif]

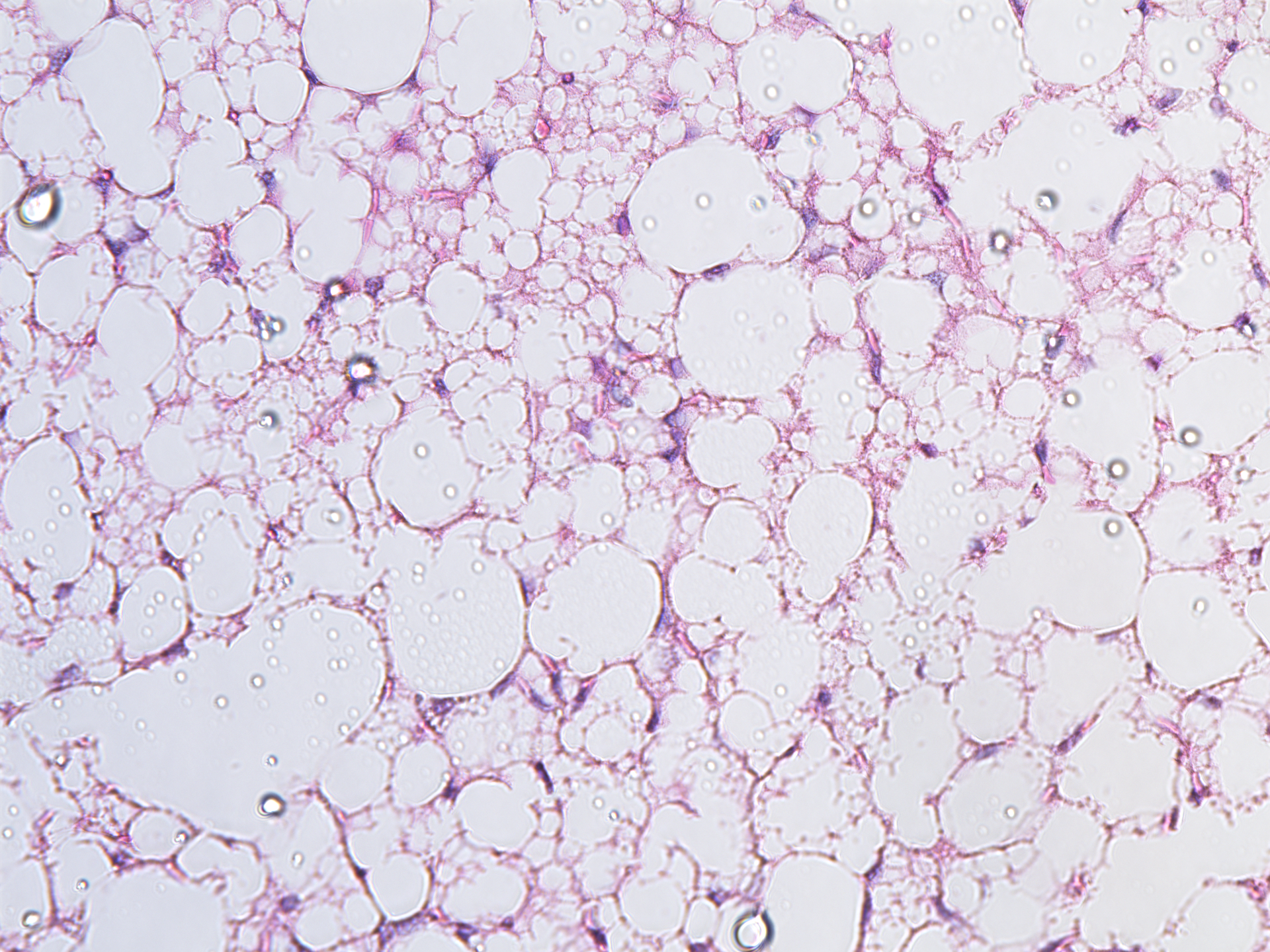

Supplement: Supplementary file 9 — Figure EV3 Source Data [file 44318_2024_196_MOESM9_ESM.zip › Figure EV3/Figure EV3-G/Demonstrated image/HFD Pcolce KO.tif]

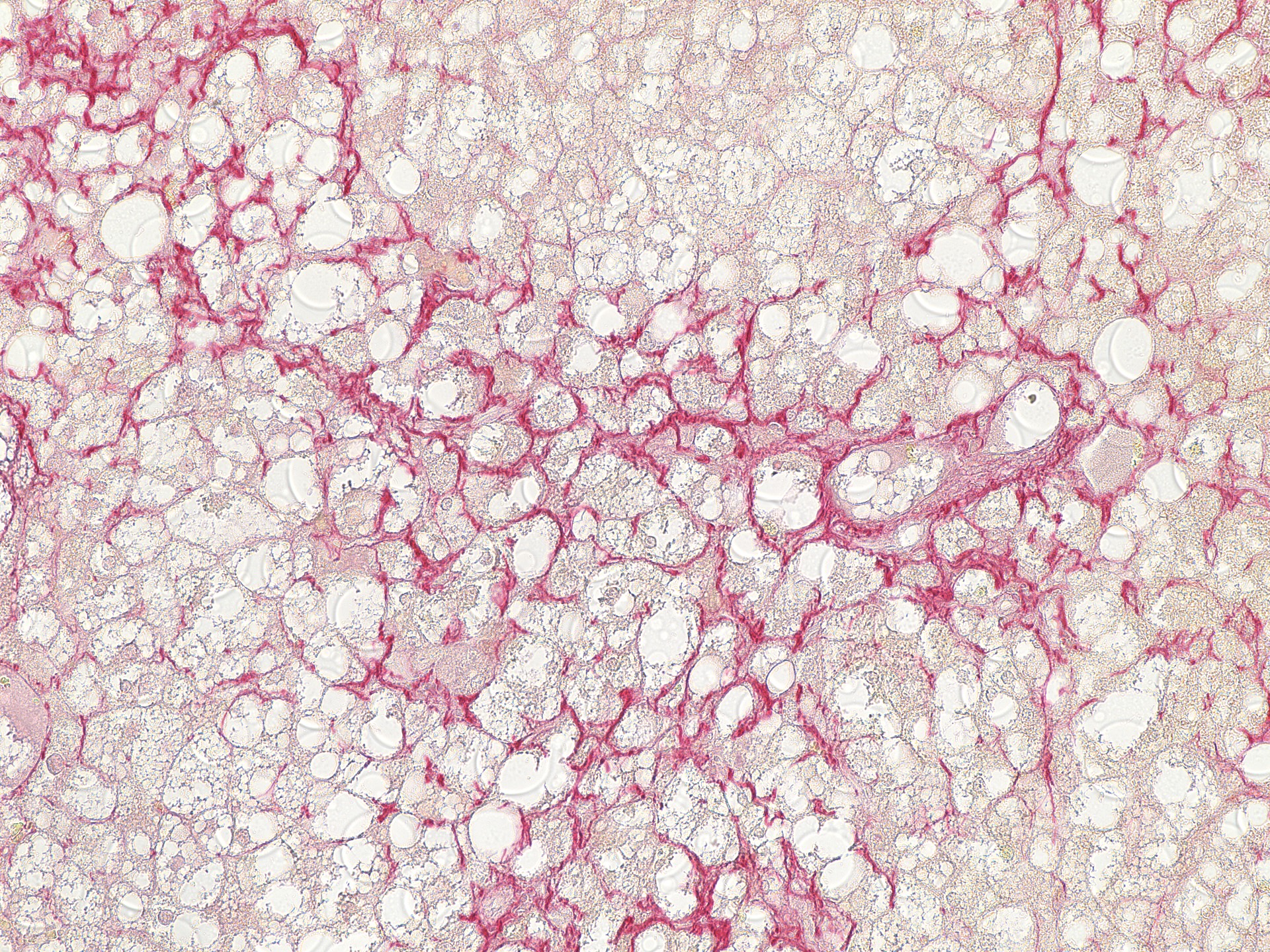

Supplement: Supplementary file 9 — Figure EV3 Source Data [file 44318_2024_196_MOESM9_ESM.zip › Figure EV3/Figure EV3-L/Quantificated image/HFD Con/no.1/Liver-HFD con-no.1-20x-5.jpg]
