# Supplementary figures and images for: PCPE-1, a brown adipose tissue-derived cytokine, promotes obesity-induced liver fibrosis (part 6 of 6)
Source: EMBO J. 2024 Aug 19;43(21):4846–69. doi: 10.1038/s44318-024-00196-0 (PMC11535236; doi:10.1038/s44318-024-00196-0)

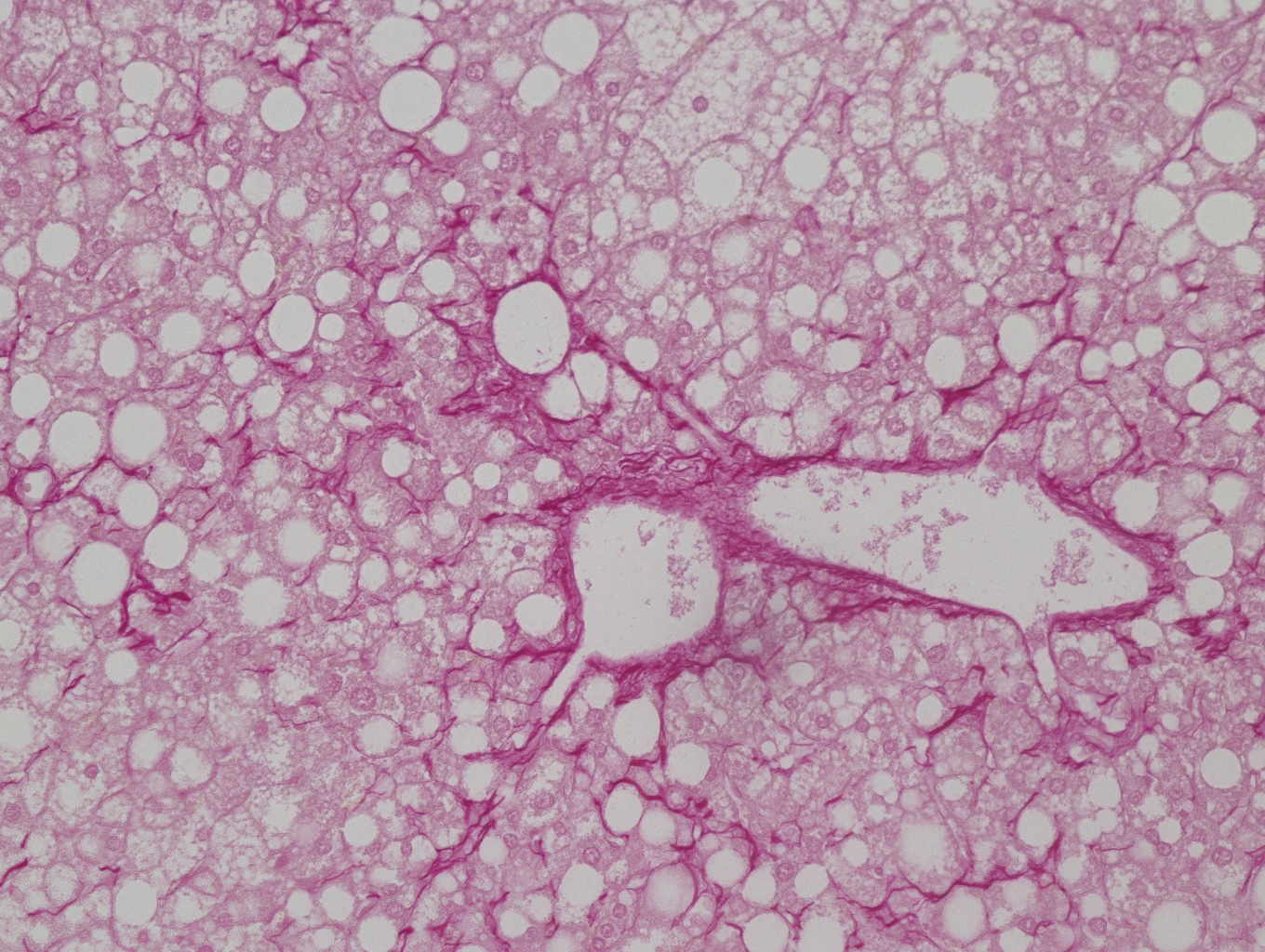

Supplement: Supplementary file 9 — Figure EV3 Source Data [file 44318_2024_196_MOESM9_ESM.zip › Figure EV3/Figure EV3-F/Quantificated image/HFD Con/no.4/HFD Con no.4 x20-1.jpg]

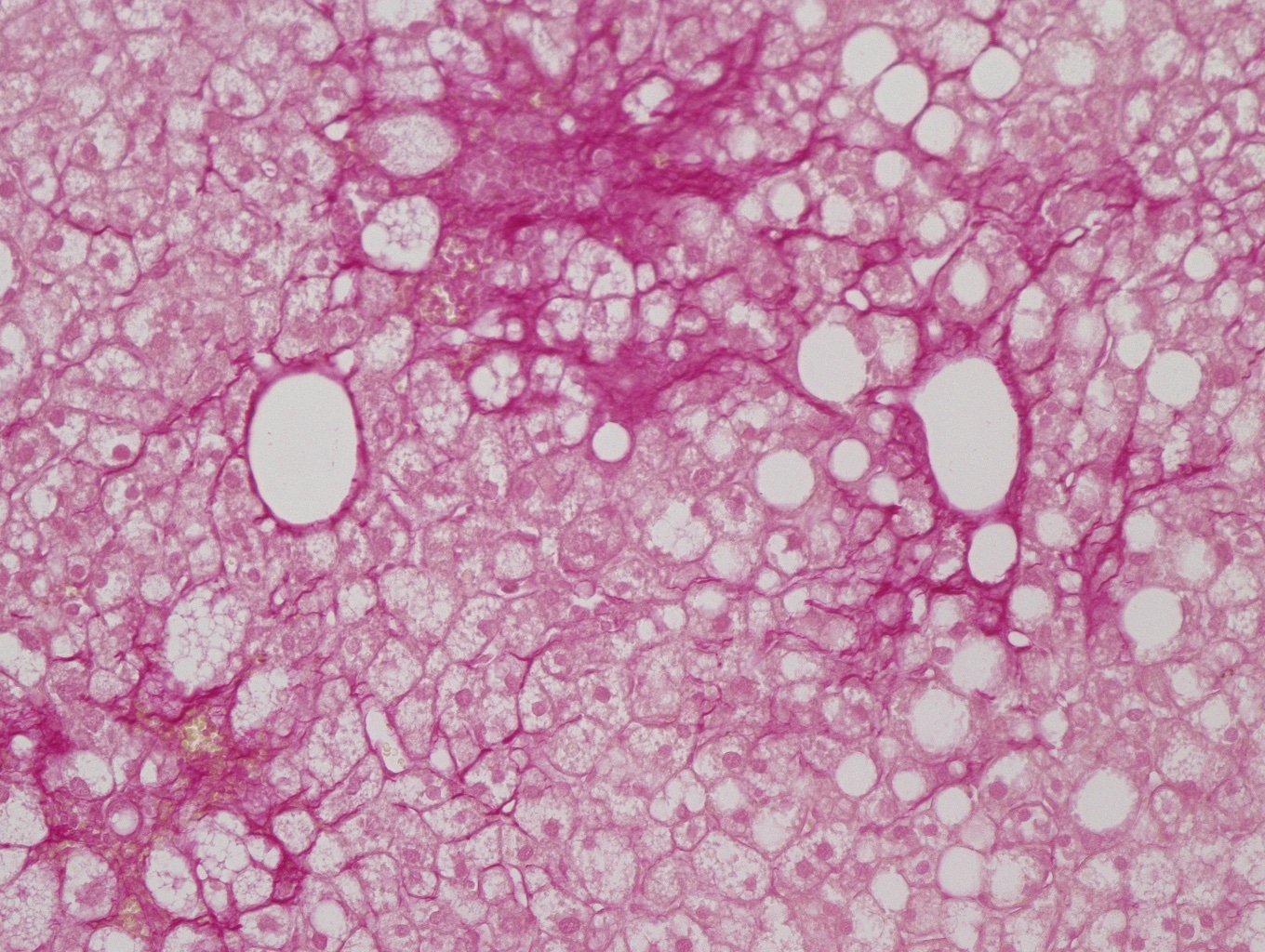

Supplement: Supplementary file 9 — Figure EV3 Source Data [file 44318_2024_196_MOESM9_ESM.zip › Figure EV3/Figure EV3-F/Quantificated image/HFD Con/no.2/HFD Con no.2 x20-2.jpg]

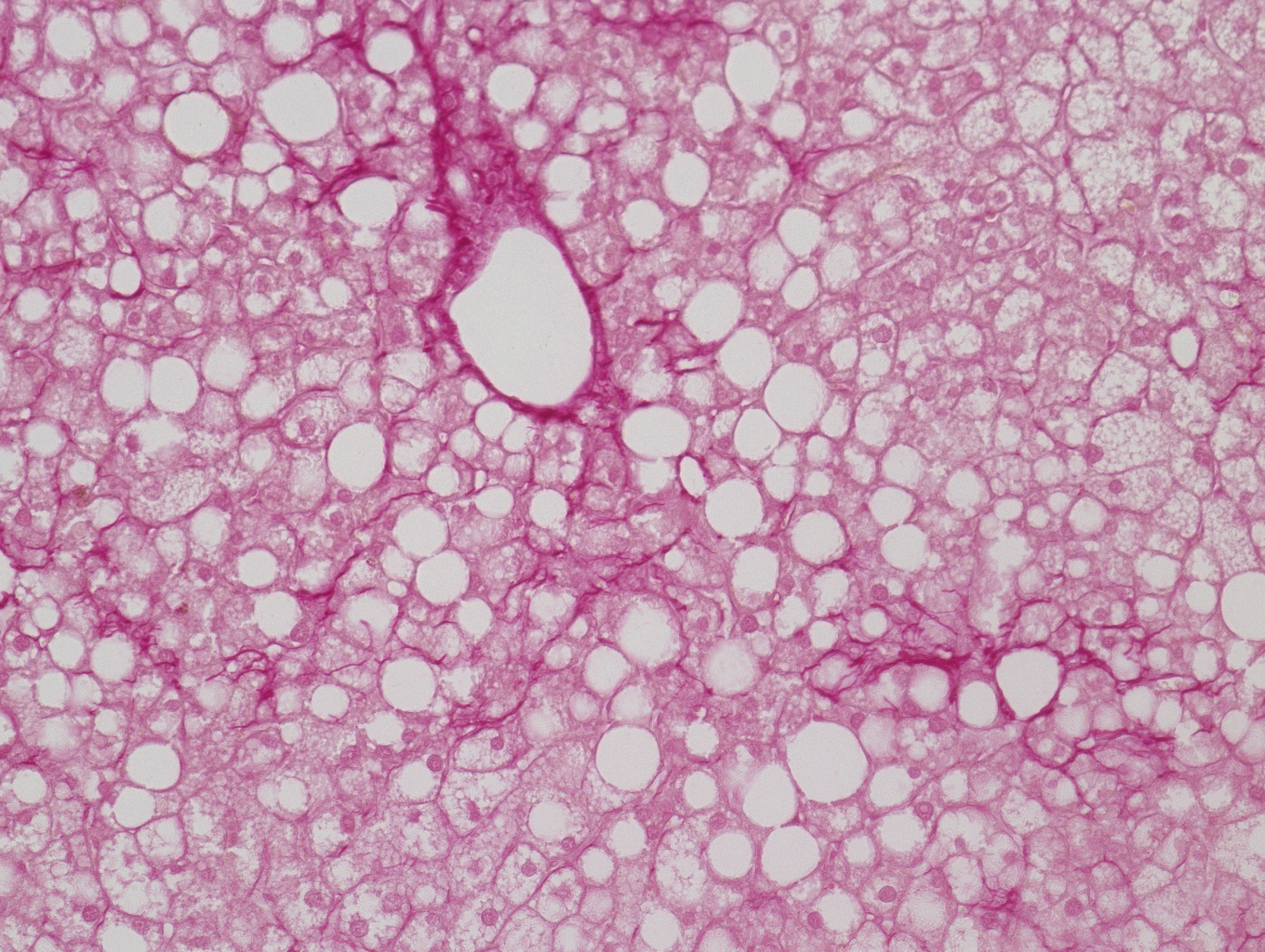

Supplement: Supplementary file 9 — Figure EV3 Source Data [file 44318_2024_196_MOESM9_ESM.zip › Figure EV3/Figure EV3-F/Quantificated image/HFD Con/no.2/HFD Con no.2 x20-3.jpg]

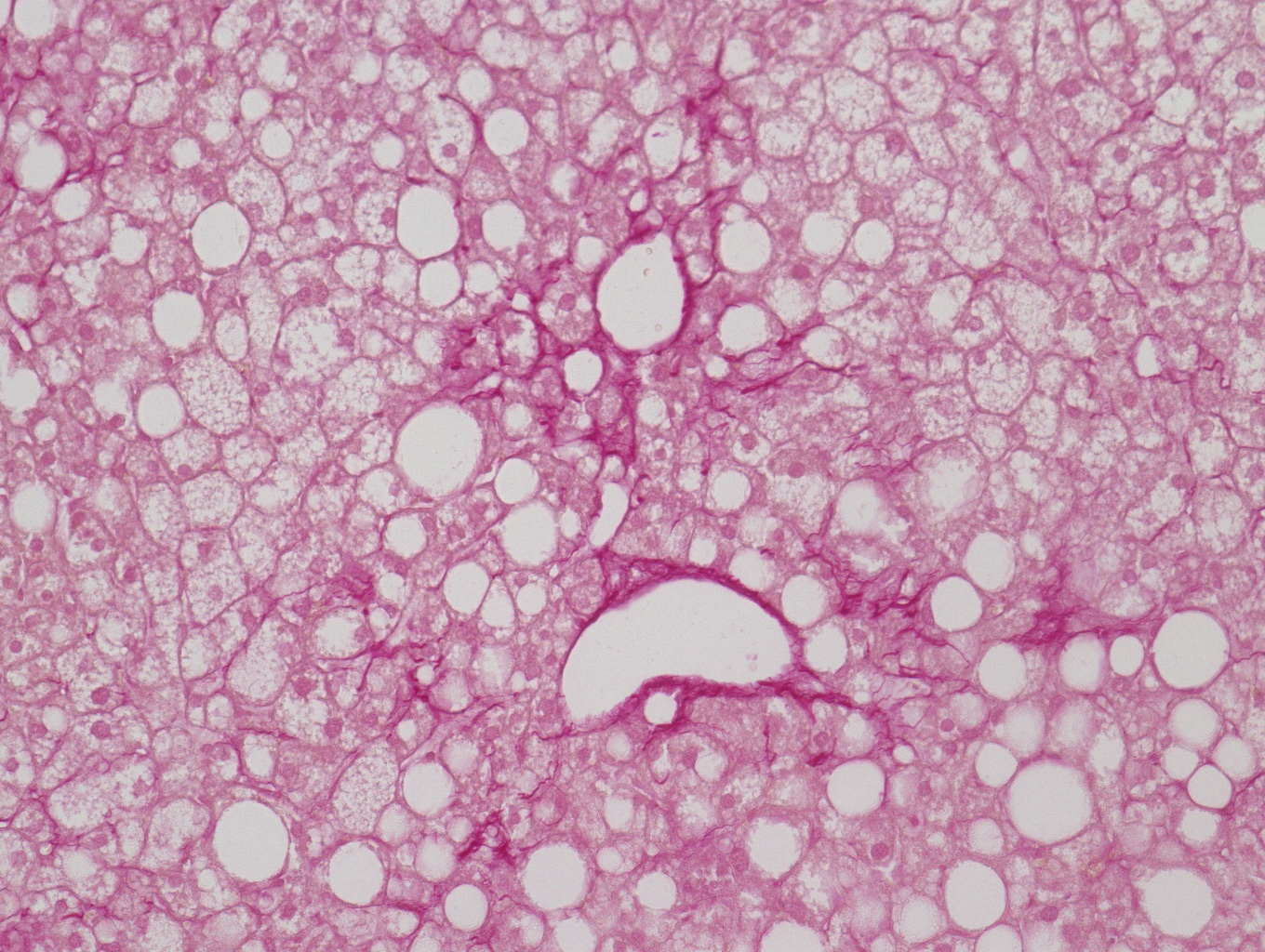

Supplement: Supplementary file 9 — Figure EV3 Source Data [file 44318_2024_196_MOESM9_ESM.zip › Figure EV3/Figure EV3-F/Quantificated image/HFD Con/no.2/HFD Con no.2 x20-1.jpg]

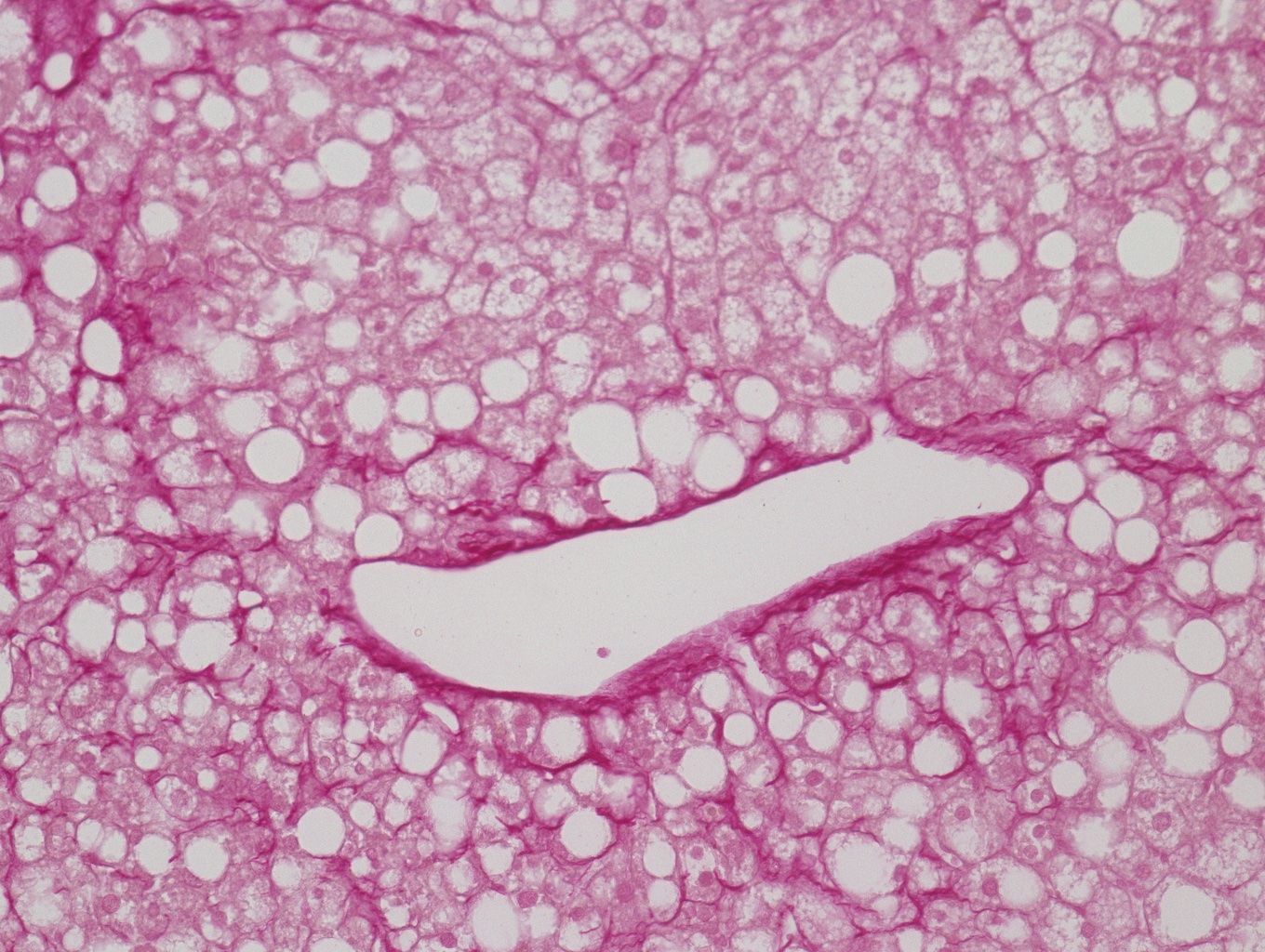

Supplement: Supplementary file 9 — Figure EV3 Source Data [file 44318_2024_196_MOESM9_ESM.zip › Figure EV3/Figure EV3-F/Quantificated image/HFD Con/no.2/HFD Con no.2 x20-4.jpg]

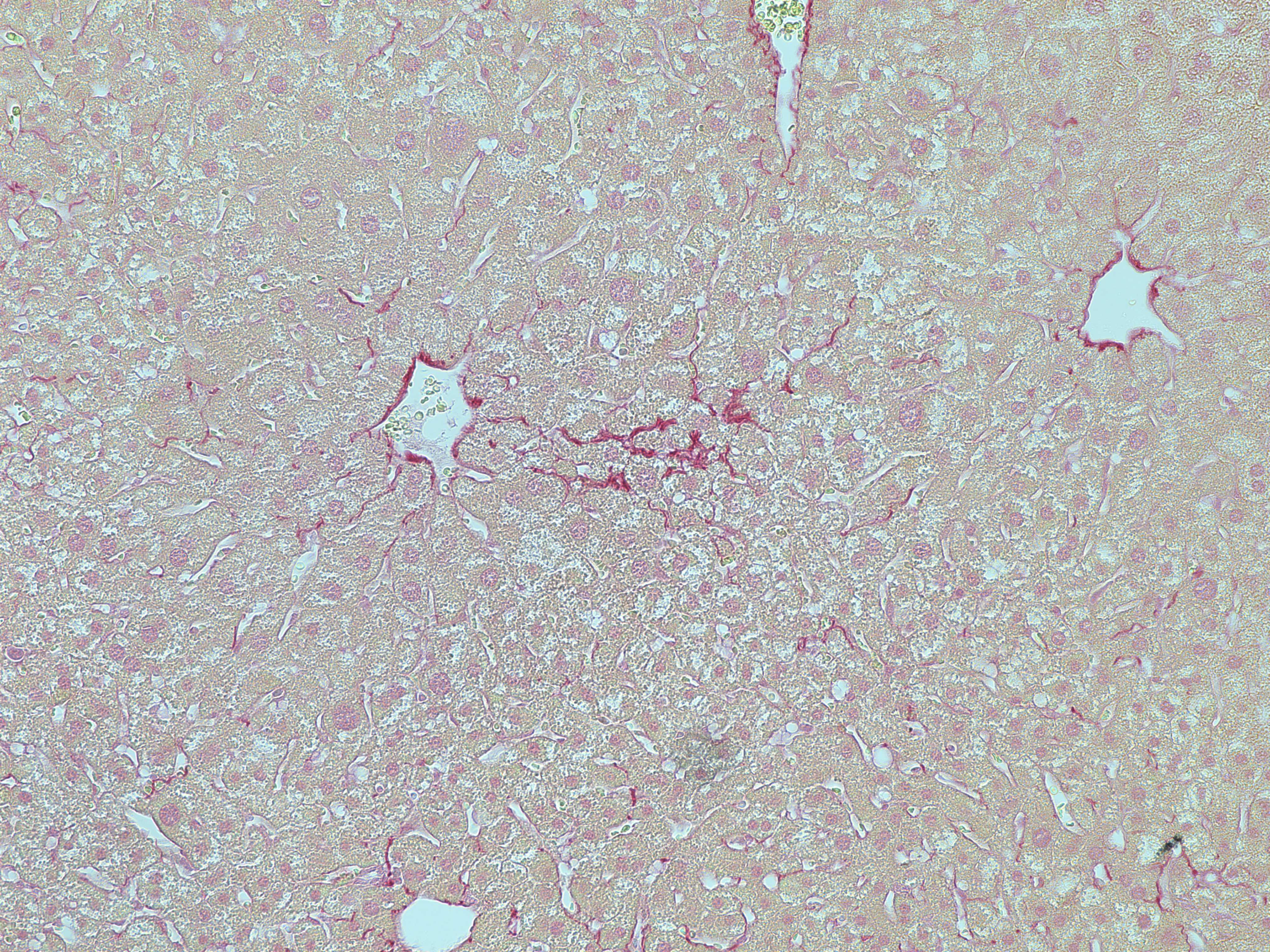

Supplement: Supplementary file 9 — Figure EV3 Source Data [file 44318_2024_196_MOESM9_ESM.zip › Figure EV3/Figure EV3-F/Quantificated image/NC Con/no.1/NC Con no.1-20x-1.jpg]

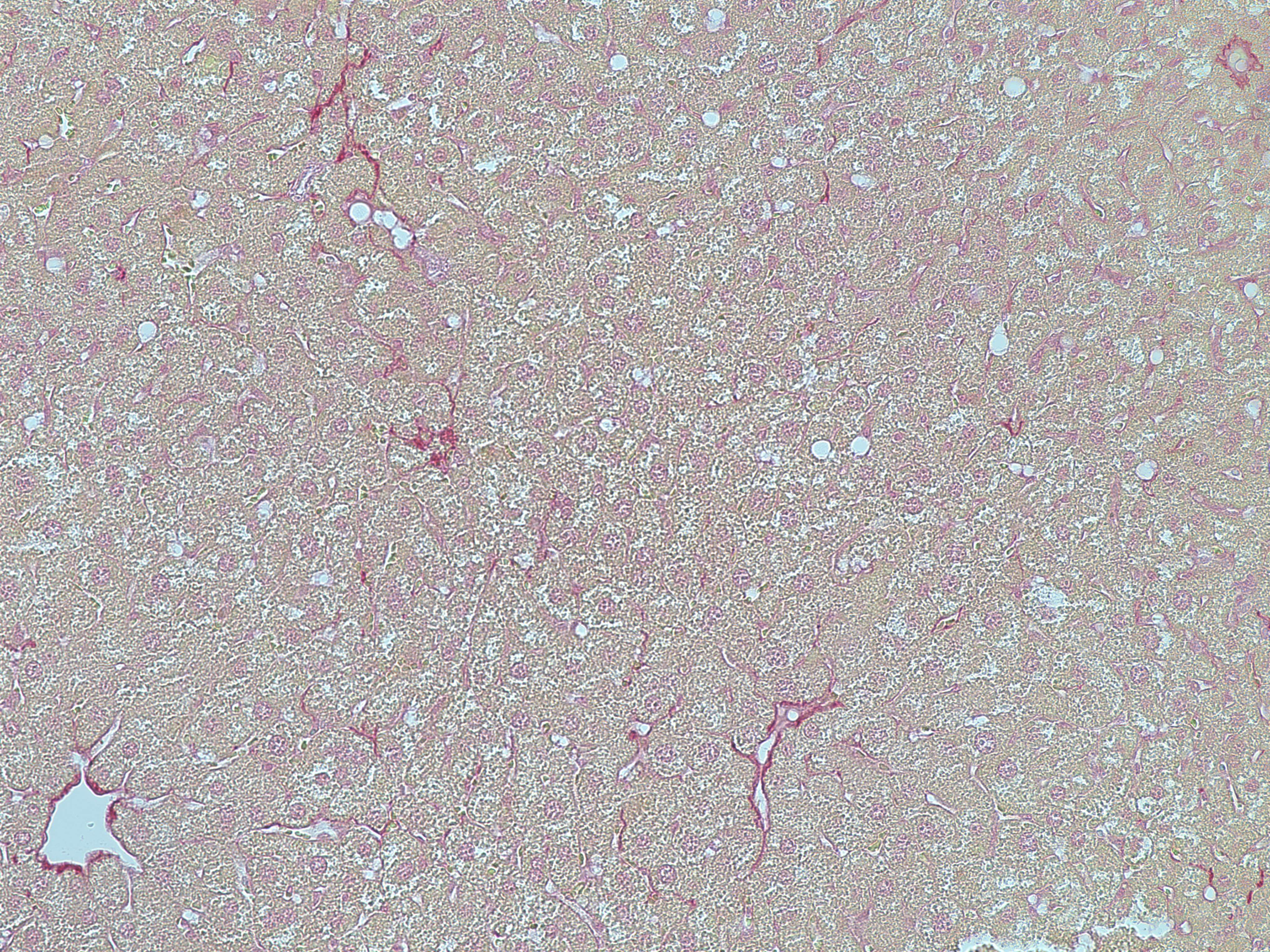

Supplement: Supplementary file 9 — Figure EV3 Source Data [file 44318_2024_196_MOESM9_ESM.zip › Figure EV3/Figure EV3-F/Quantificated image/NC Con/no.1/NC Con no.1-20x-3.jpg]

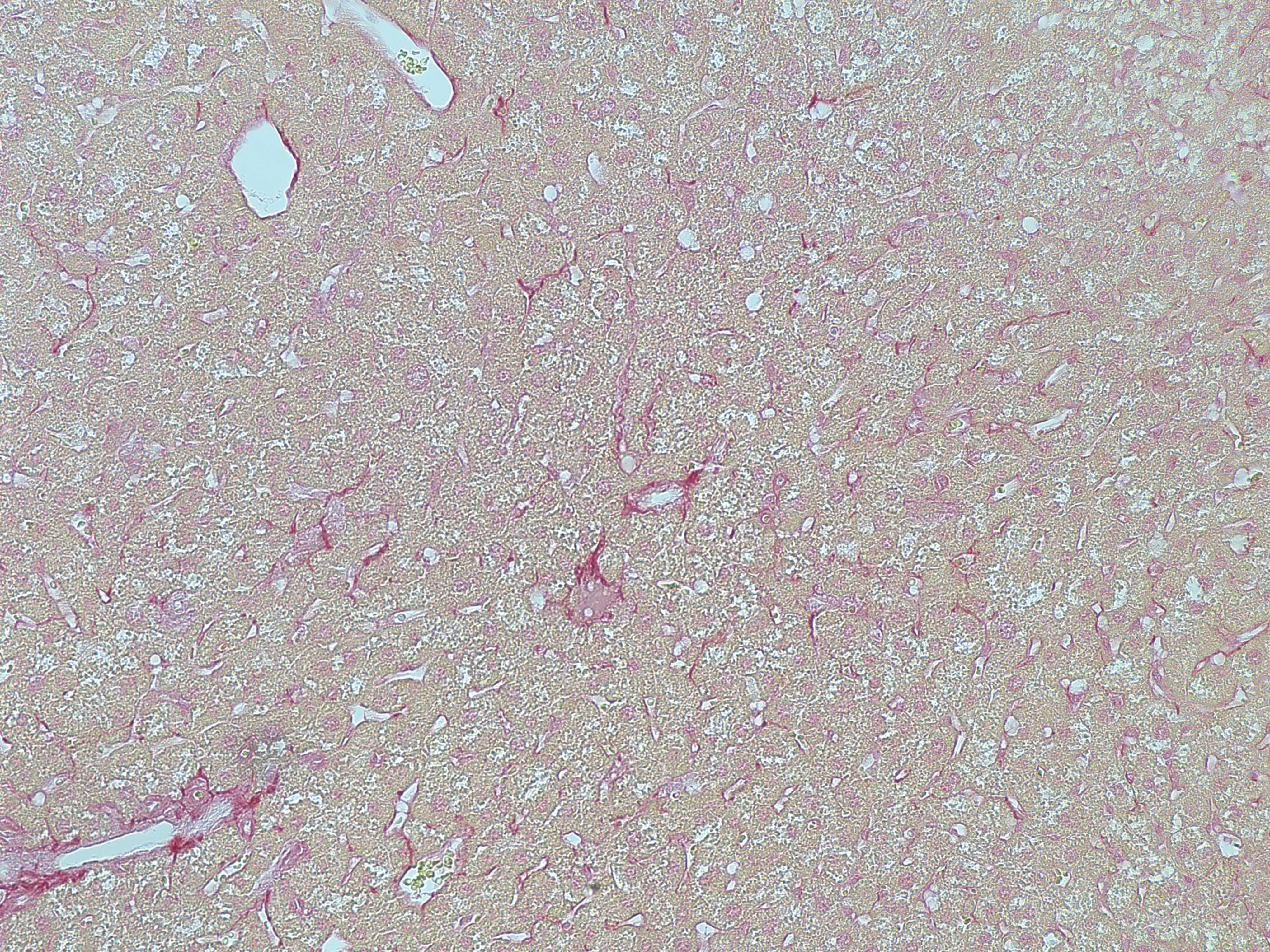

Supplement: Supplementary file 9 — Figure EV3 Source Data [file 44318_2024_196_MOESM9_ESM.zip › Figure EV3/Figure EV3-F/Quantificated image/NC Con/no.1/NC Con no.1-20x-2.jpg]

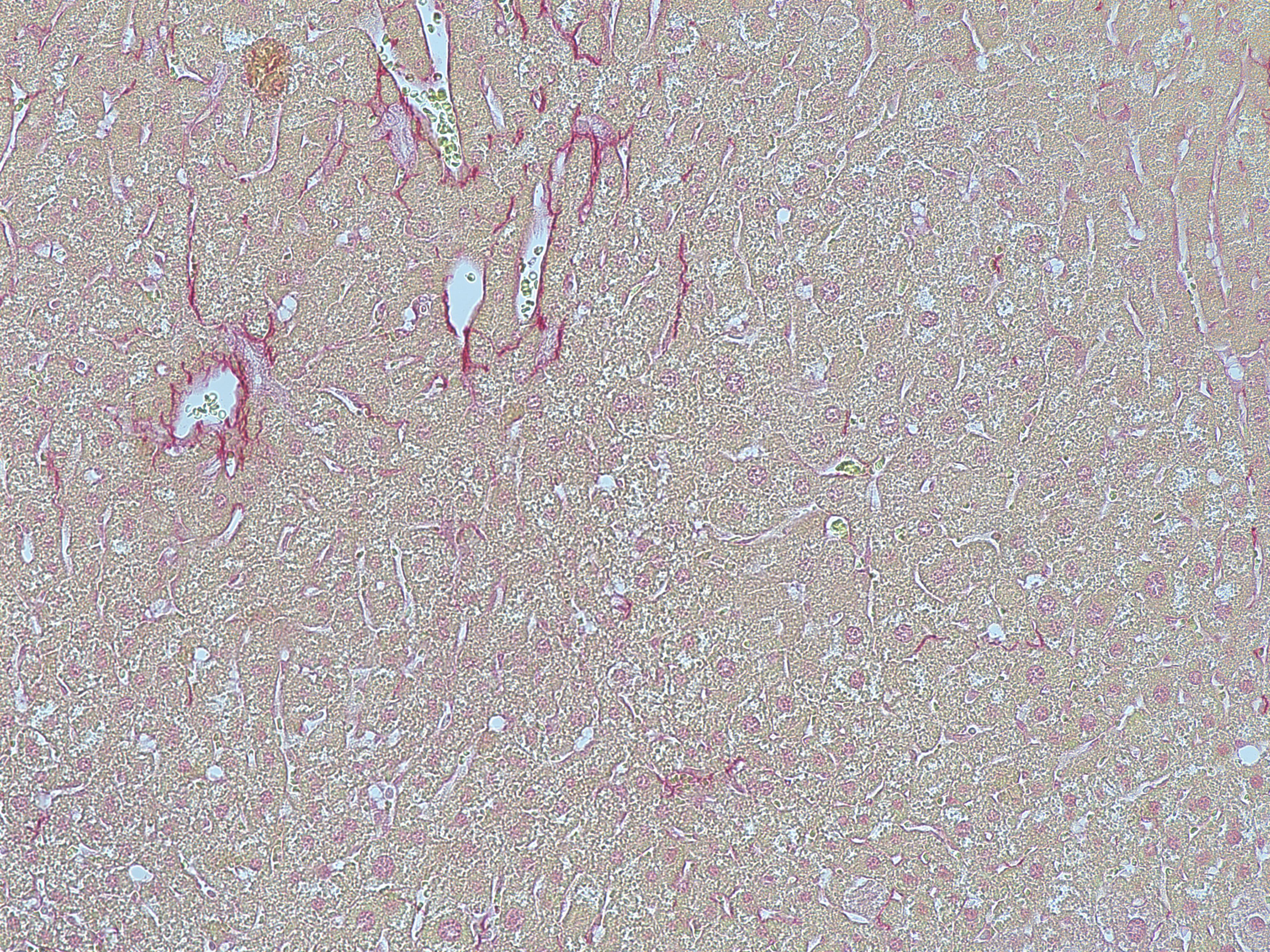

Supplement: Supplementary file 9 — Figure EV3 Source Data [file 44318_2024_196_MOESM9_ESM.zip › Figure EV3/Figure EV3-F/Quantificated image/NC Con/no.1/NC Con no.1-20x-5.jpg]

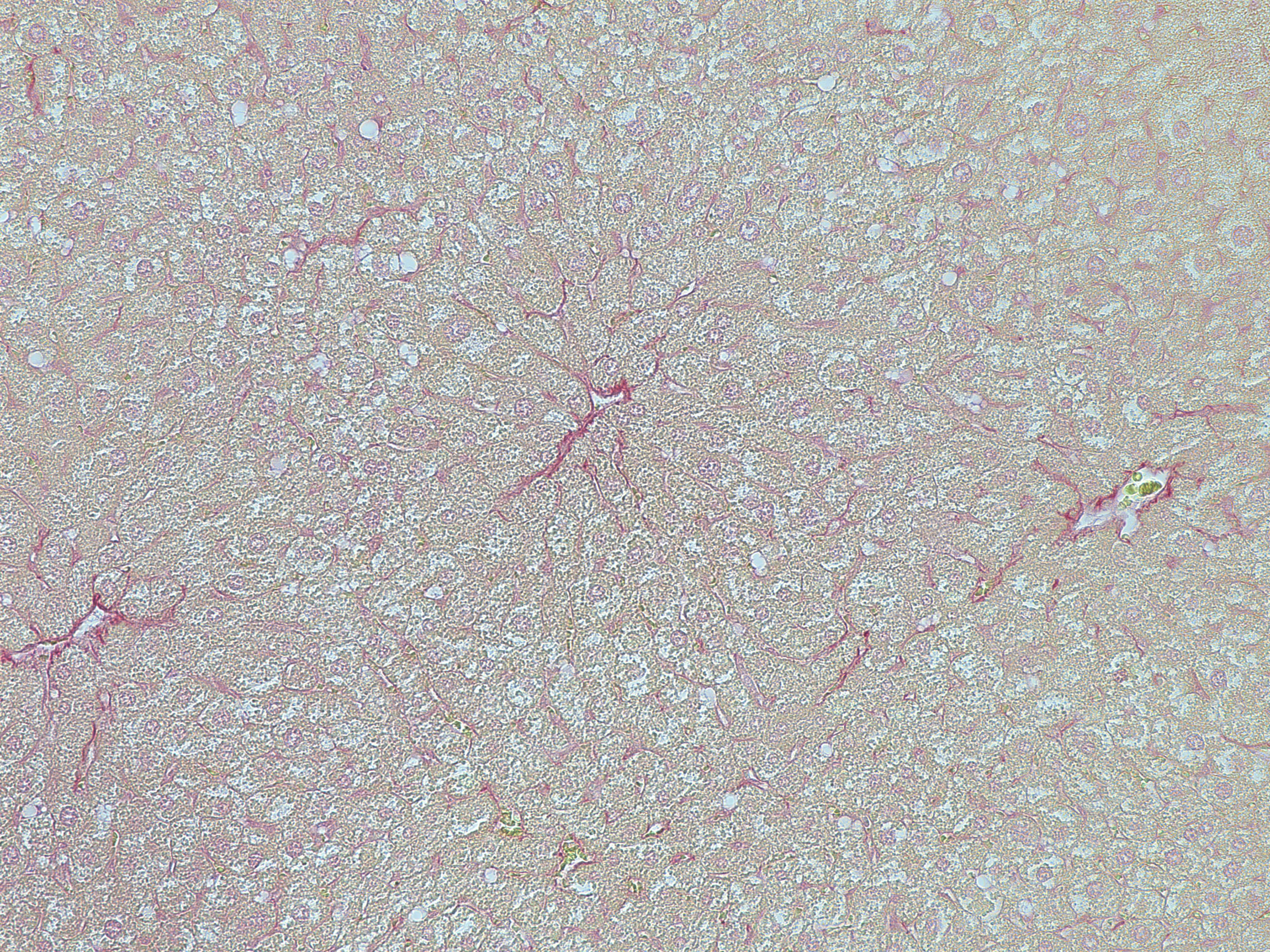

Supplement: Supplementary file 9 — Figure EV3 Source Data [file 44318_2024_196_MOESM9_ESM.zip › Figure EV3/Figure EV3-F/Quantificated image/NC Con/no.1/NC Con no.1-20x-4.jpg]

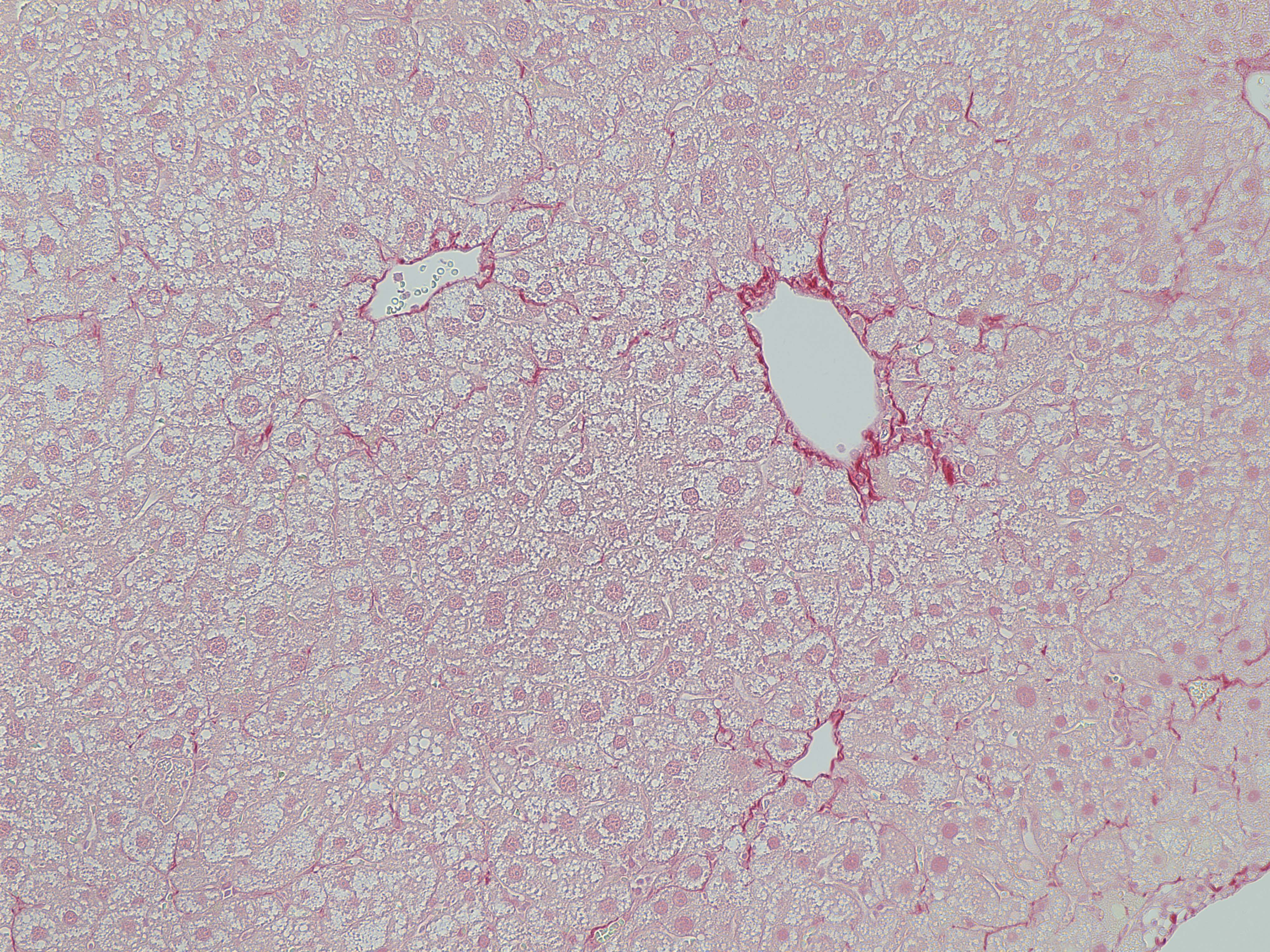

Supplement: Supplementary file 9 — Figure EV3 Source Data [file 44318_2024_196_MOESM9_ESM.zip › Figure EV3/Figure EV3-F/Quantificated image/NC Con/no.3/NC Con no.3-20x-1.jpg]

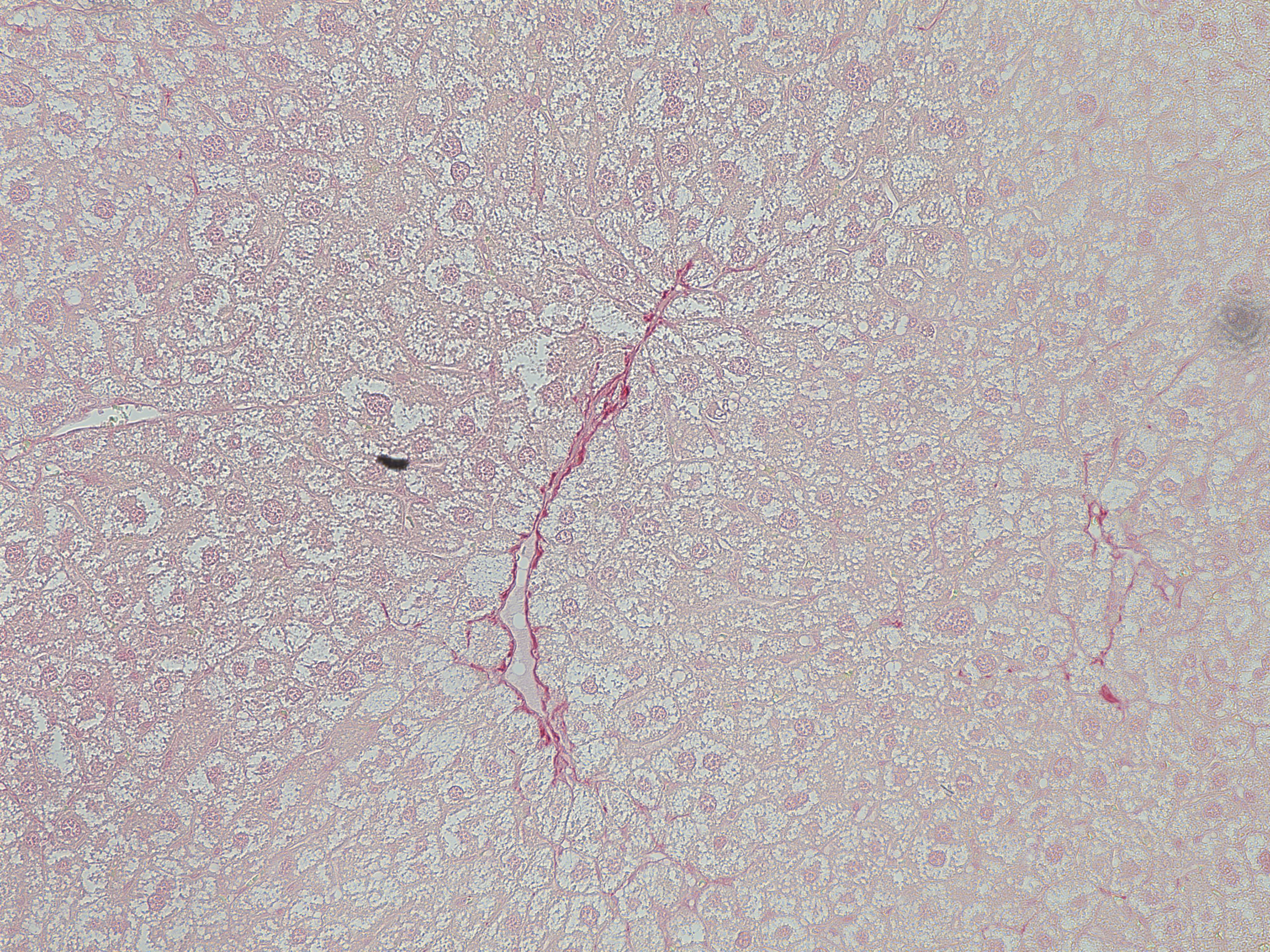

Supplement: Supplementary file 9 — Figure EV3 Source Data [file 44318_2024_196_MOESM9_ESM.zip › Figure EV3/Figure EV3-F/Quantificated image/NC Con/no.3/NC Con no.3-20x-2.jpg]

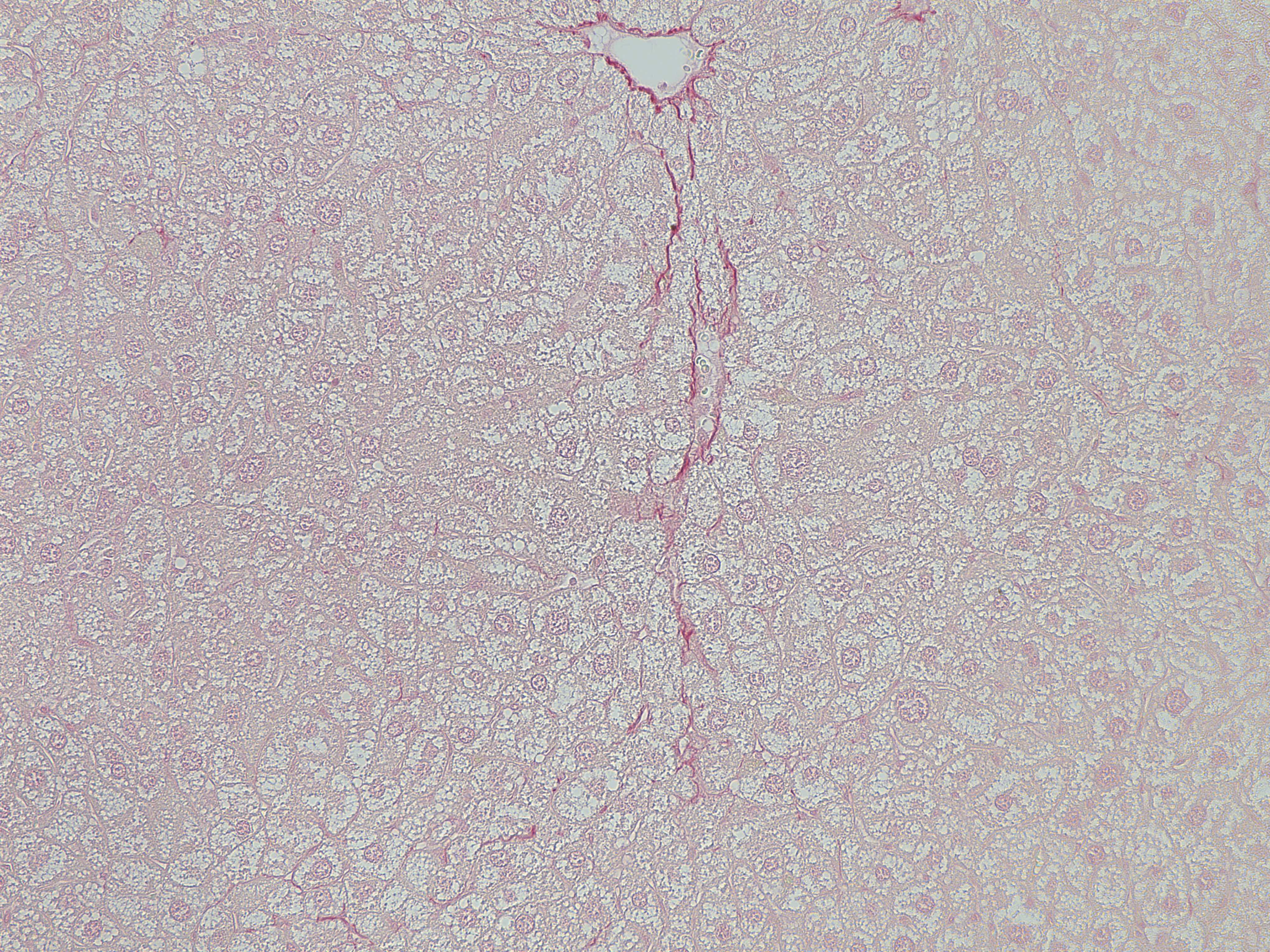

Supplement: Supplementary file 9 — Figure EV3 Source Data [file 44318_2024_196_MOESM9_ESM.zip › Figure EV3/Figure EV3-F/Quantificated image/NC Con/no.3/NC Con no.3-20x-3.jpg]

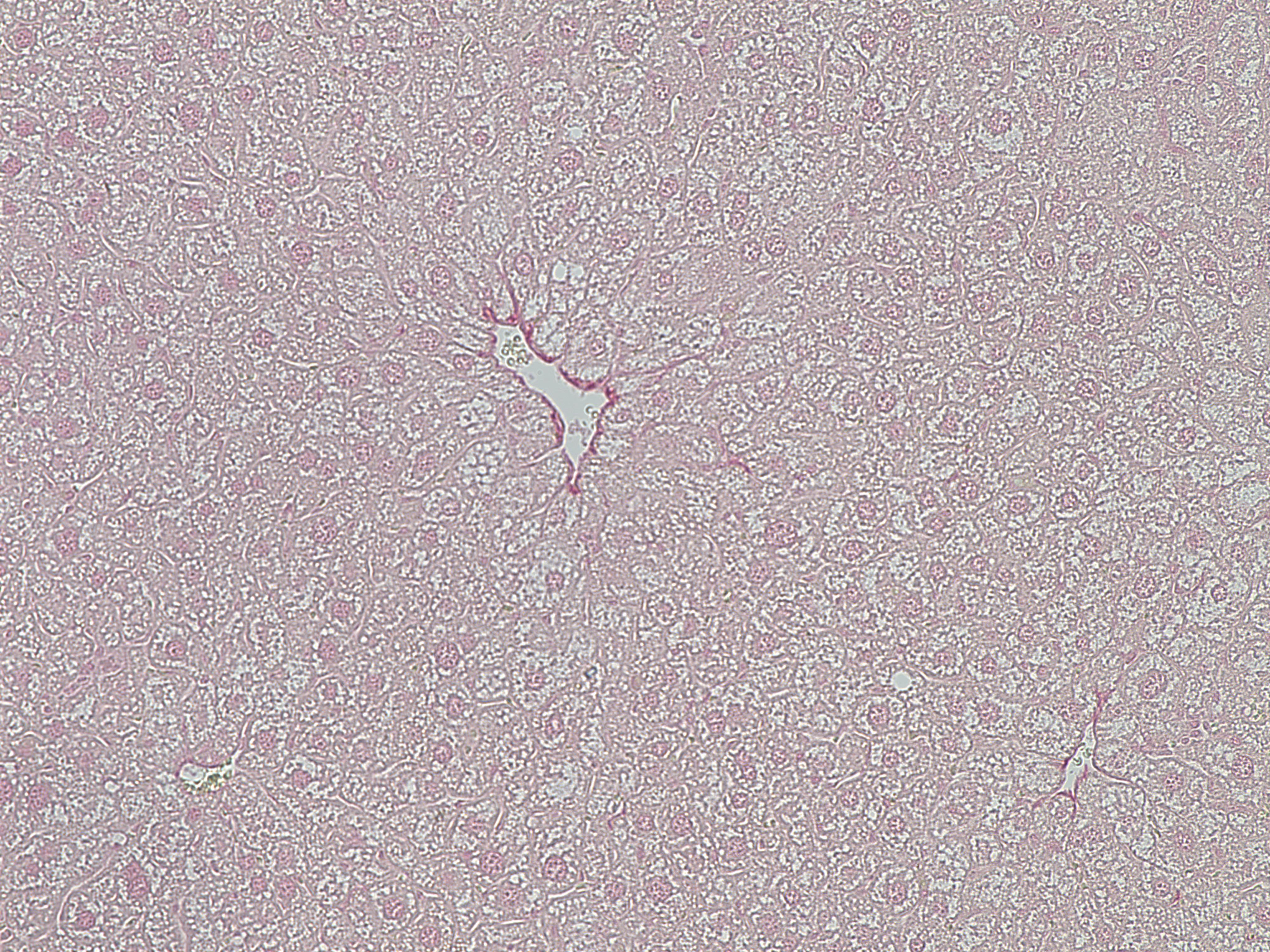

Supplement: Supplementary file 9 — Figure EV3 Source Data [file 44318_2024_196_MOESM9_ESM.zip › Figure EV3/Figure EV3-F/Quantificated image/NC Con/no.3/NC Con no.3-20x-4.jpg]

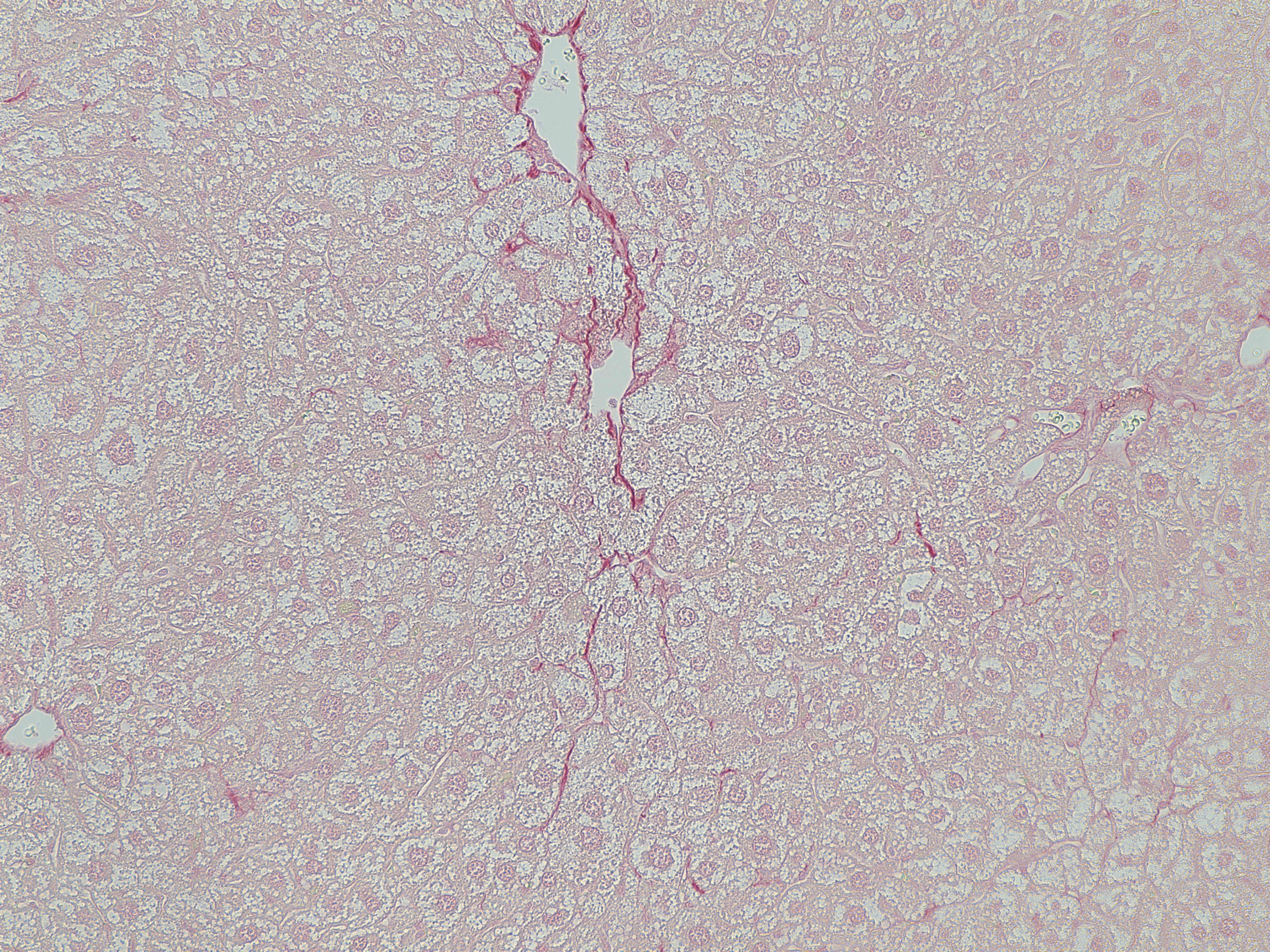

Supplement: Supplementary file 9 — Figure EV3 Source Data [file 44318_2024_196_MOESM9_ESM.zip › Figure EV3/Figure EV3-F/Quantificated image/NC Con/no.3/NC Con no.3-20x-5.jpg]

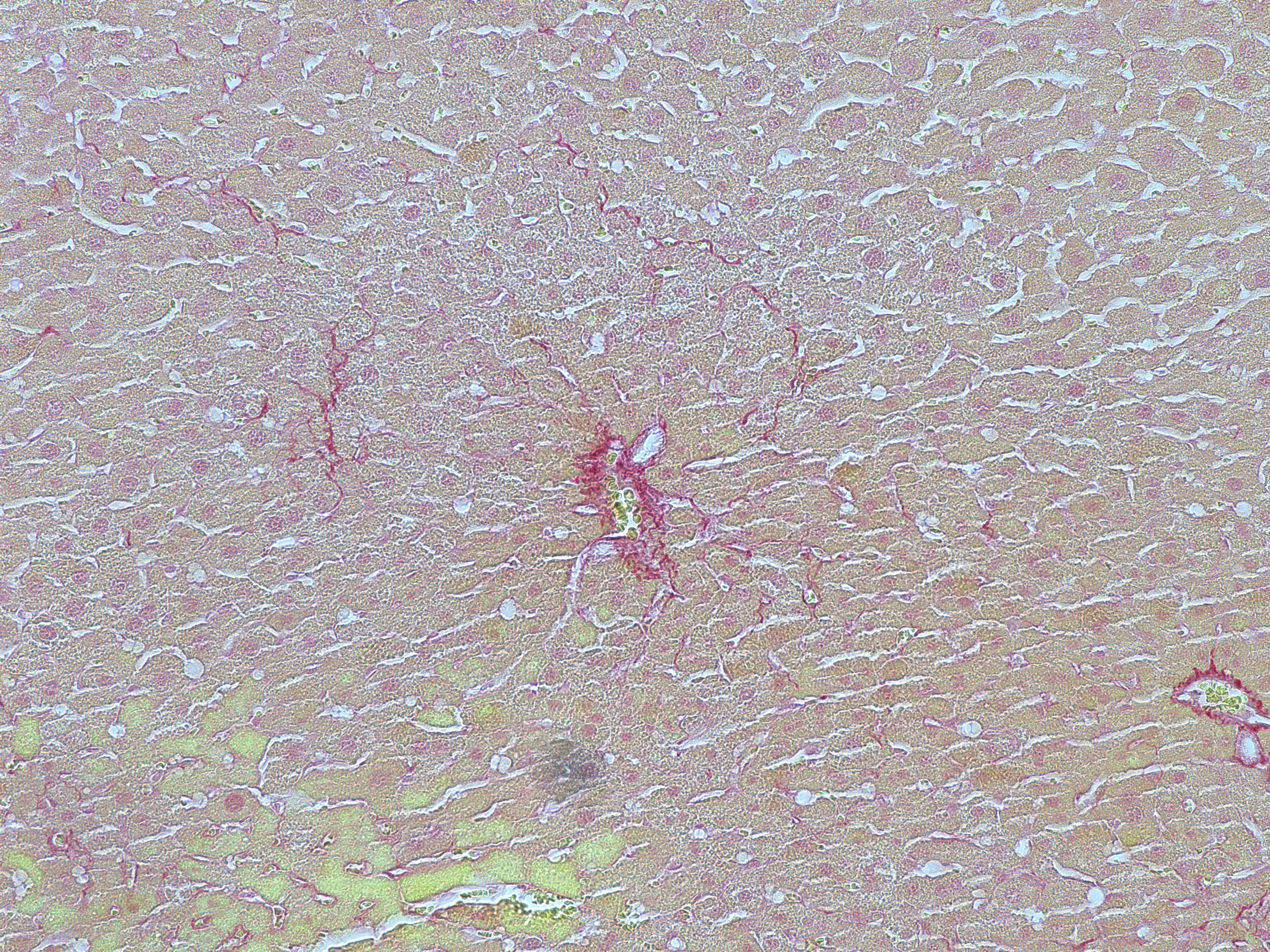

Supplement: Supplementary file 9 — Figure EV3 Source Data [file 44318_2024_196_MOESM9_ESM.zip › Figure EV3/Figure EV3-F/Quantificated image/NC Con/no.4/NC Con no.4-20x-5.jpg]

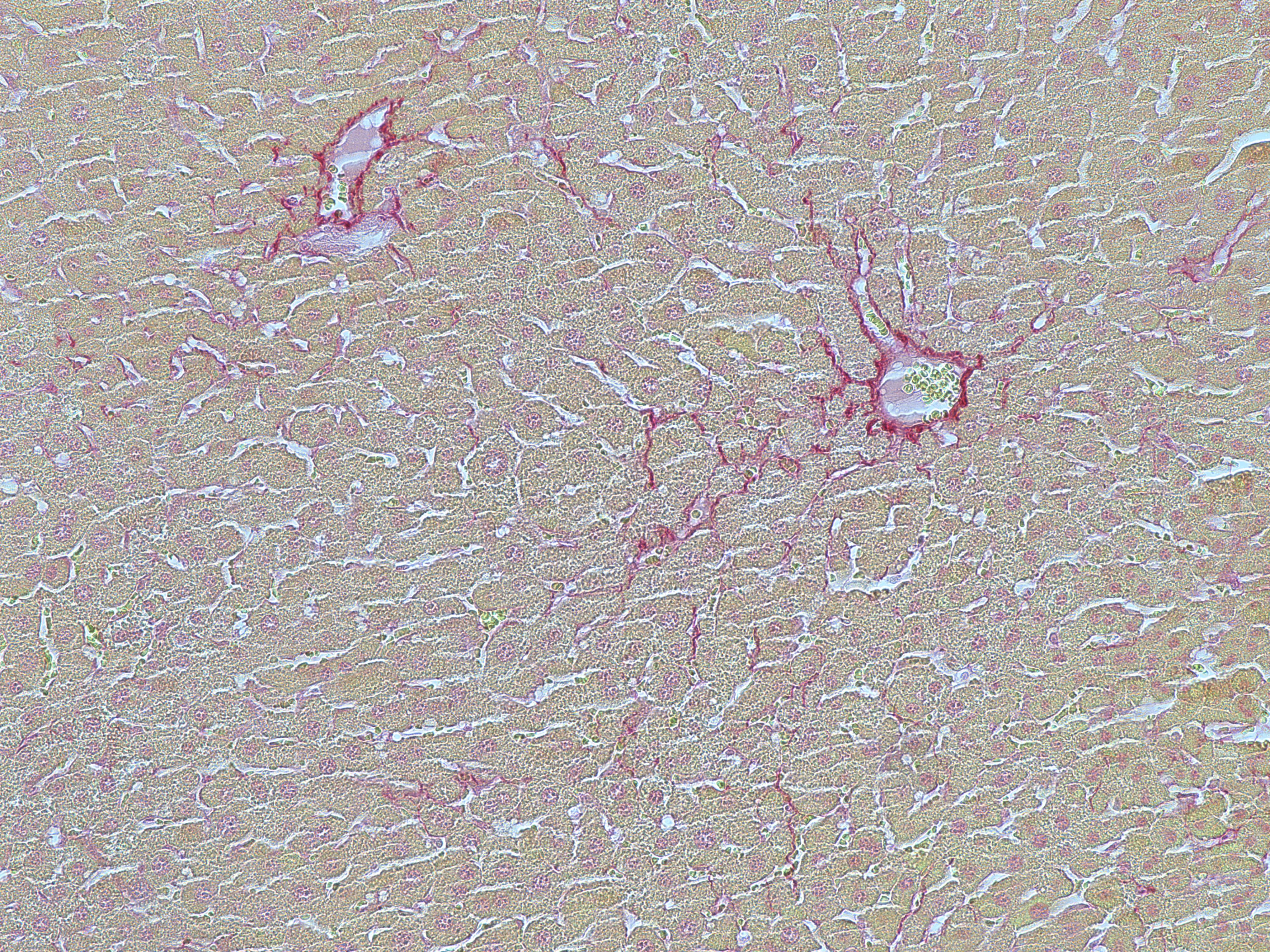

Supplement: Supplementary file 9 — Figure EV3 Source Data [file 44318_2024_196_MOESM9_ESM.zip › Figure EV3/Figure EV3-F/Quantificated image/NC Con/no.4/NC Con no.4-20x-4.jpg]

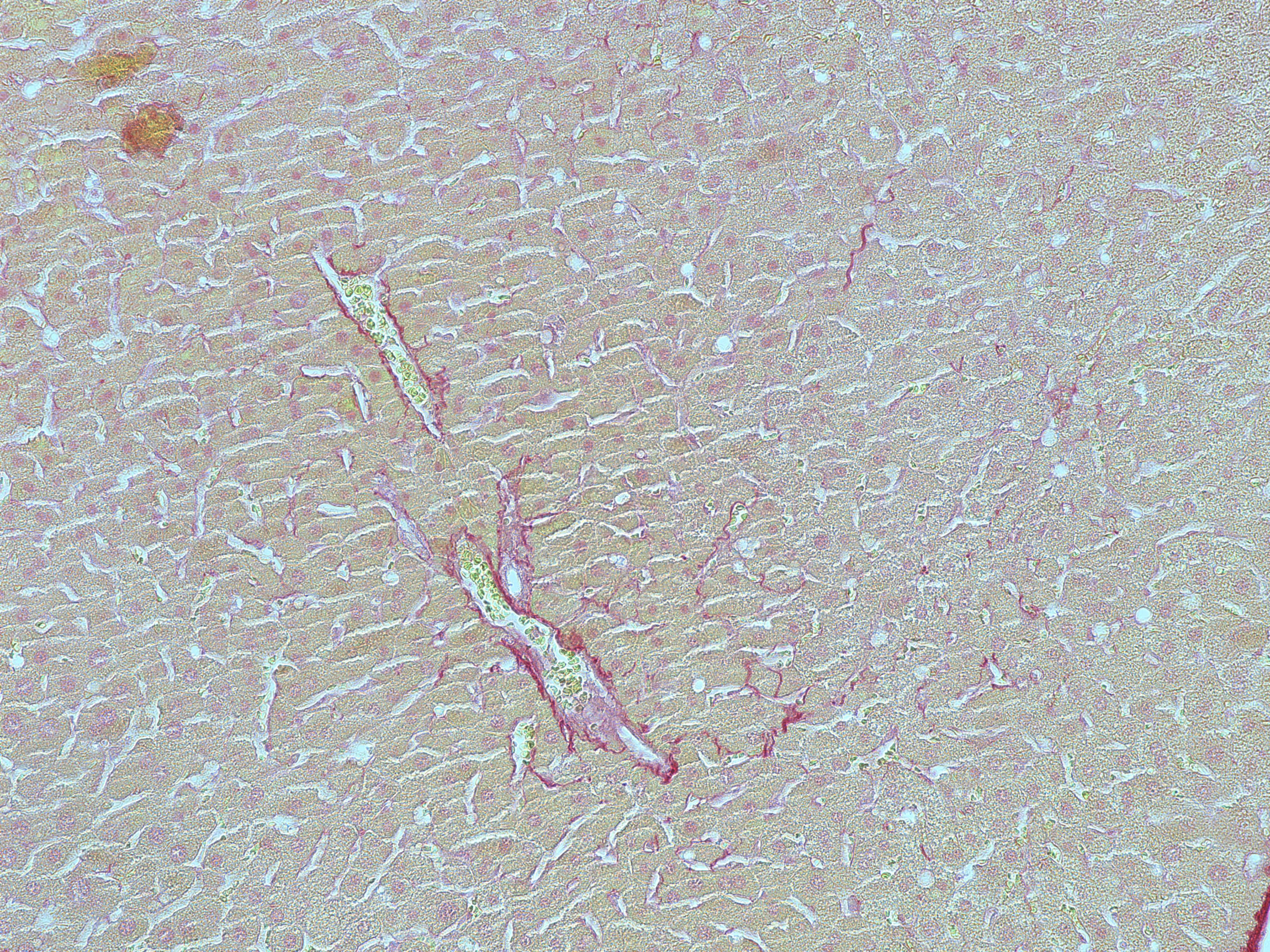

Supplement: Supplementary file 9 — Figure EV3 Source Data [file 44318_2024_196_MOESM9_ESM.zip › Figure EV3/Figure EV3-F/Quantificated image/NC Con/no.4/NC Con no.4-20x-1.jpg]

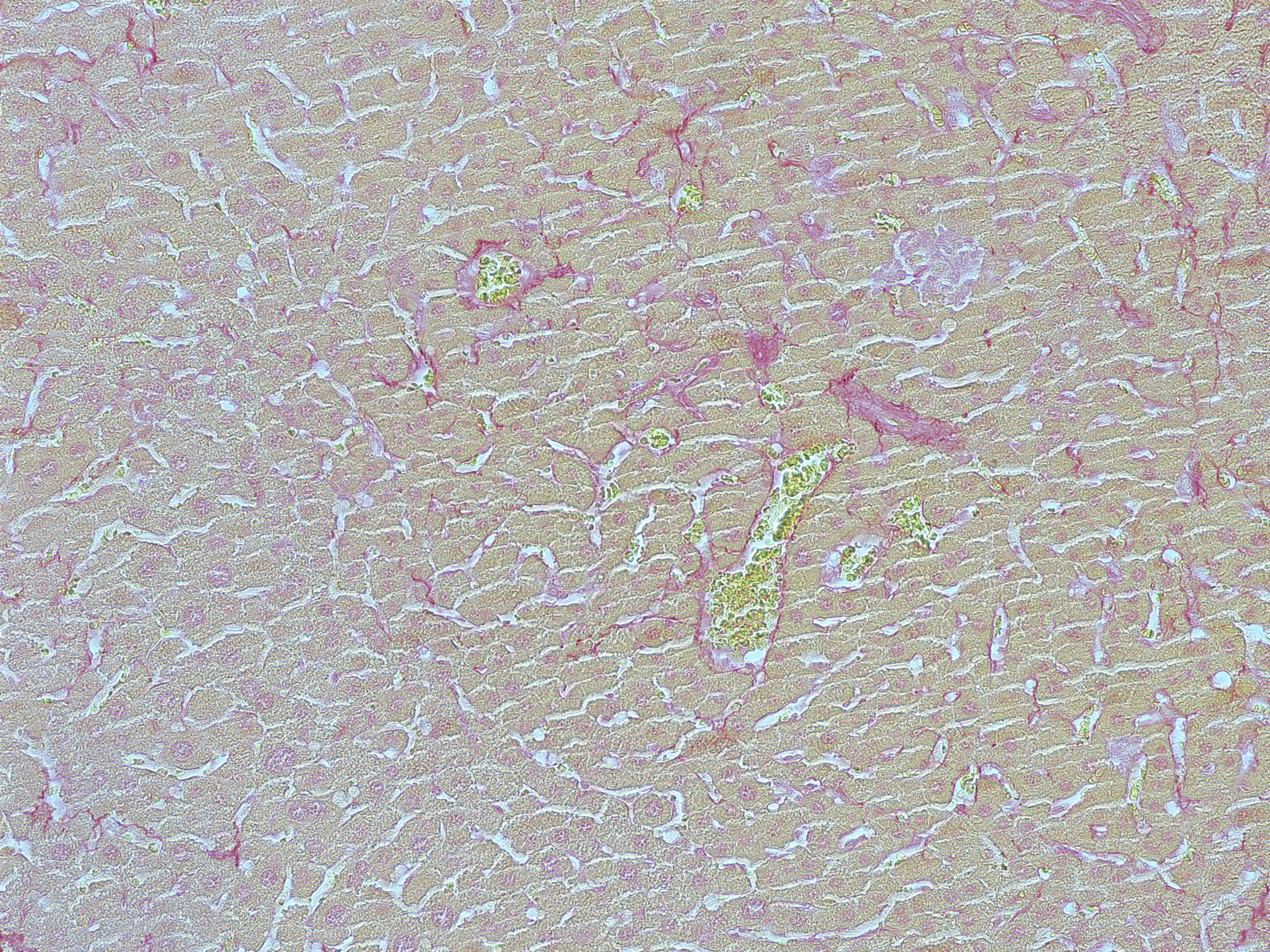

Supplement: Supplementary file 9 — Figure EV3 Source Data [file 44318_2024_196_MOESM9_ESM.zip › Figure EV3/Figure EV3-F/Quantificated image/NC Con/no.4/NC Con no.4-20x-3.jpg]

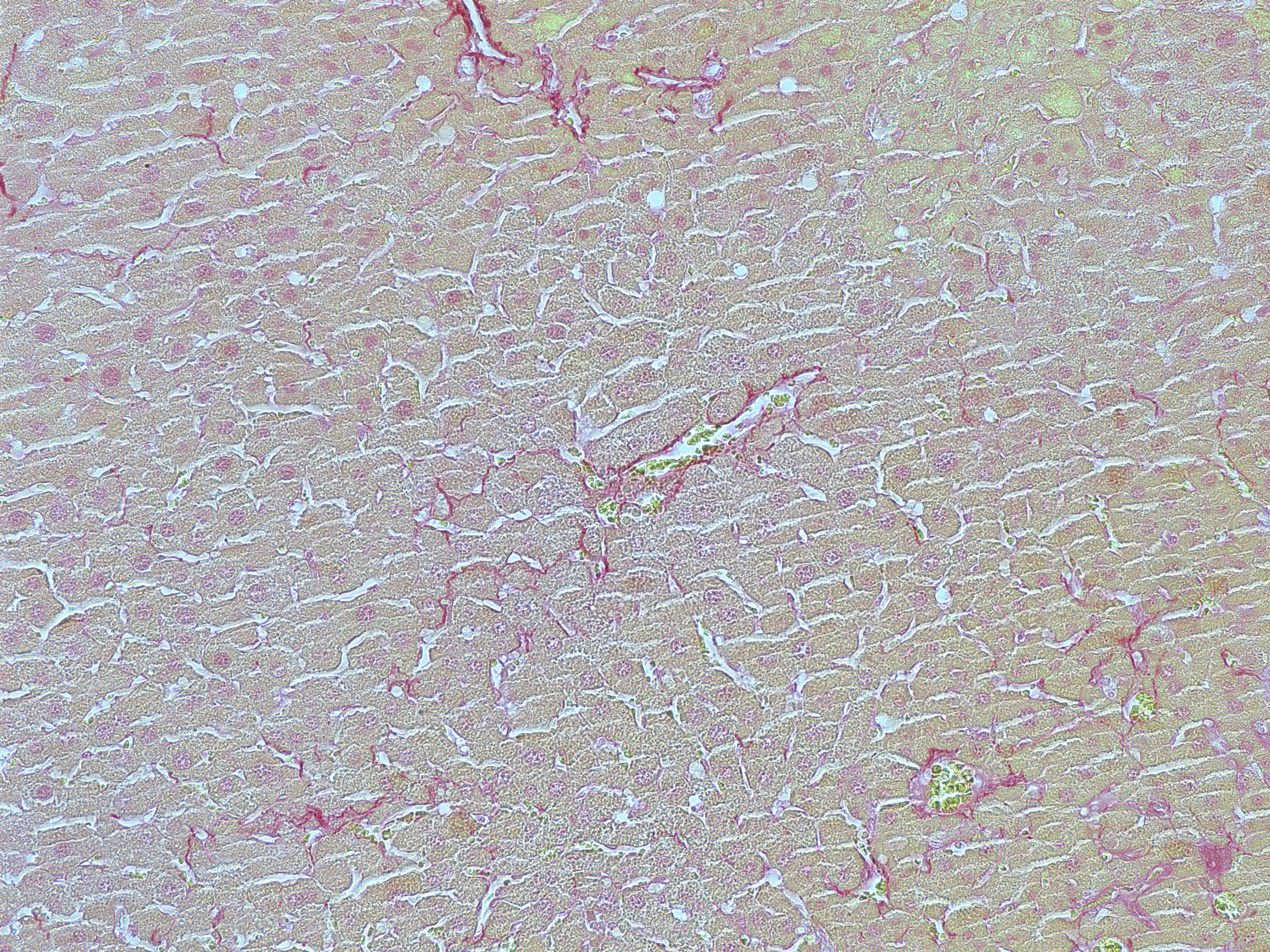

Supplement: Supplementary file 9 — Figure EV3 Source Data [file 44318_2024_196_MOESM9_ESM.zip › Figure EV3/Figure EV3-F/Quantificated image/NC Con/no.4/NC Con no.4-20x-2.jpg]

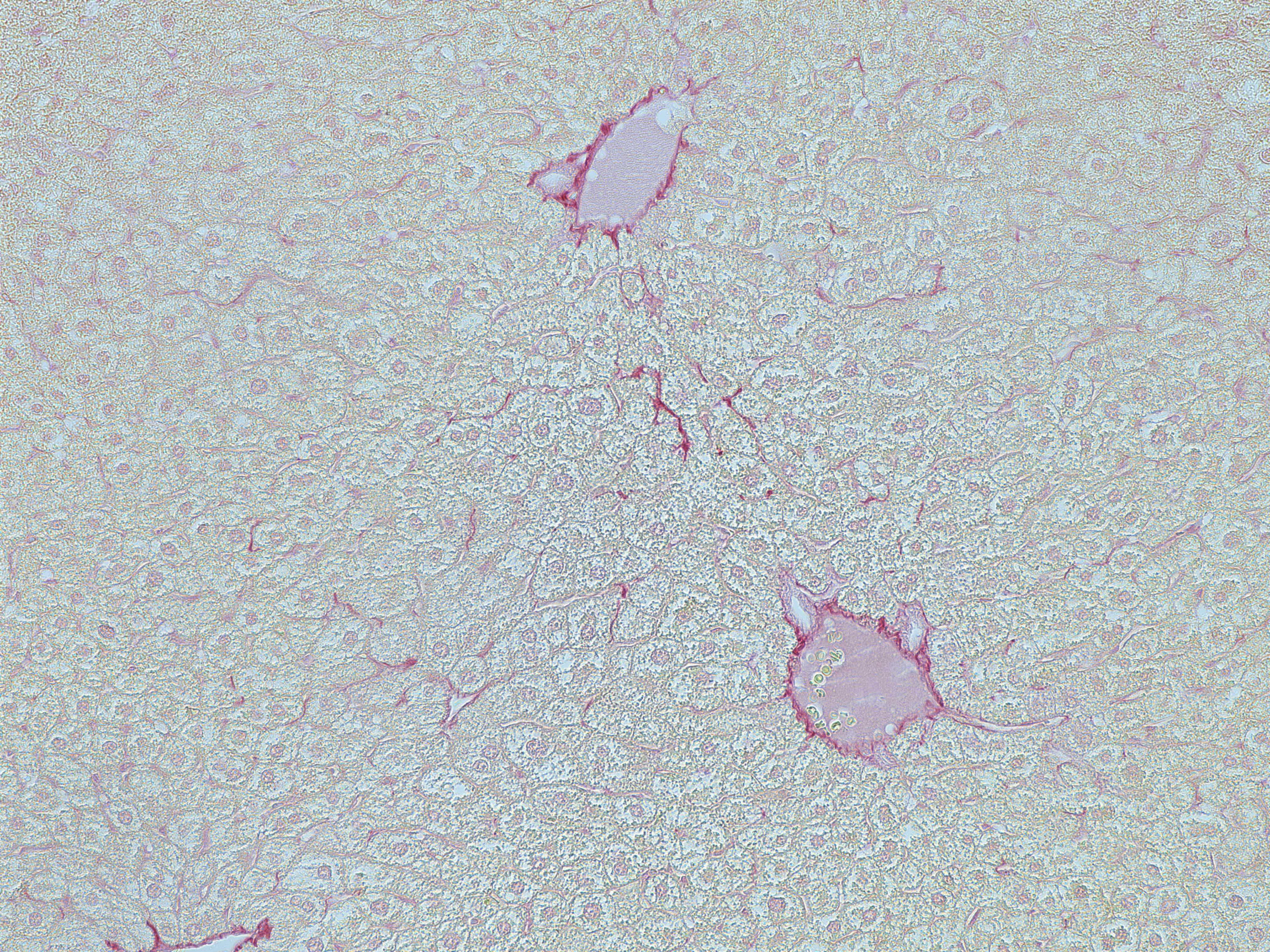

Supplement: Supplementary file 9 — Figure EV3 Source Data [file 44318_2024_196_MOESM9_ESM.zip › Figure EV3/Figure EV3-F/Quantificated image/NC Con/no.5/NC Con no.5-20x-5.jpg]

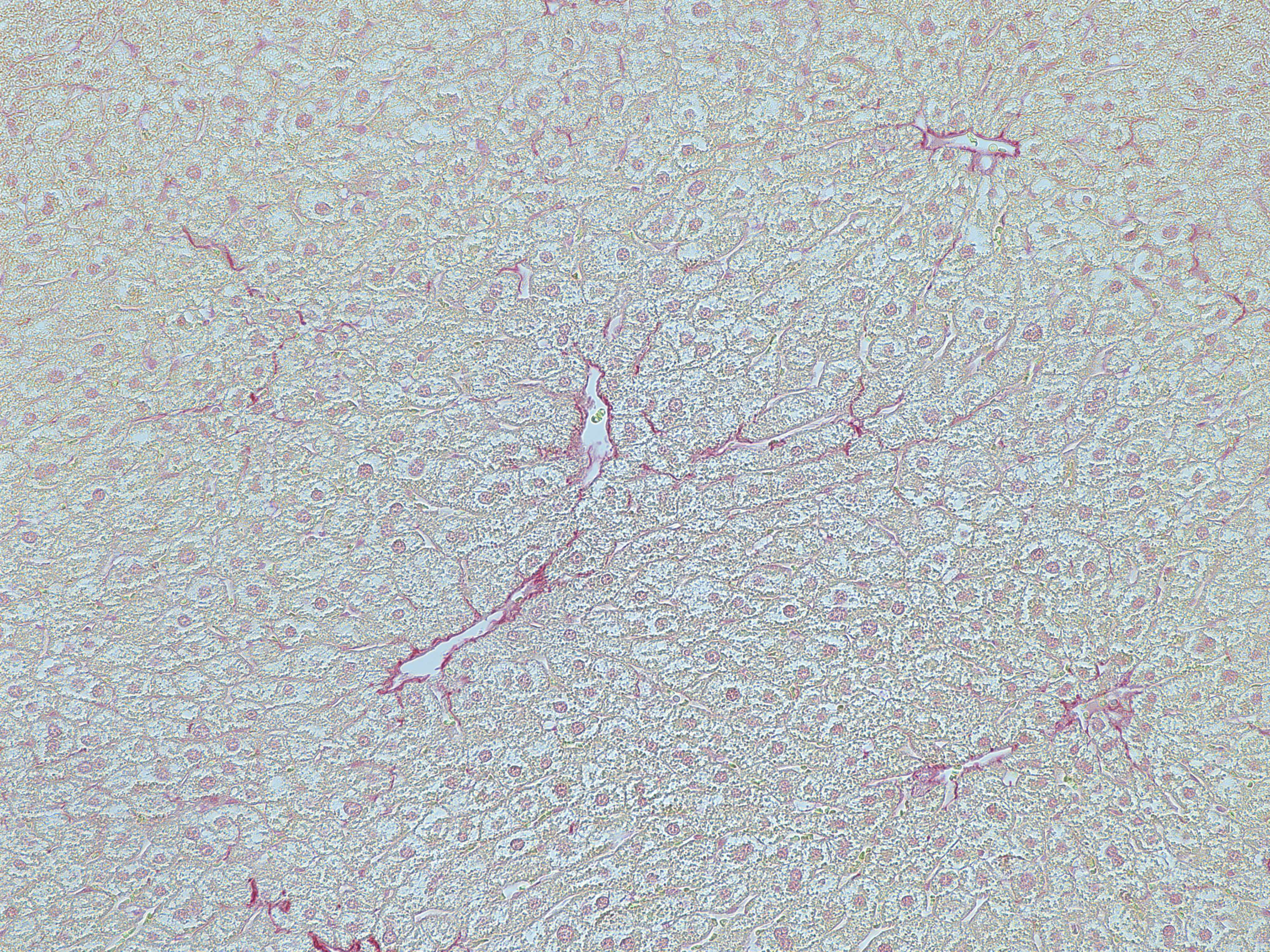

Supplement: Supplementary file 9 — Figure EV3 Source Data [file 44318_2024_196_MOESM9_ESM.zip › Figure EV3/Figure EV3-F/Quantificated image/NC Con/no.5/NC Con no.5-20x-4.jpg]

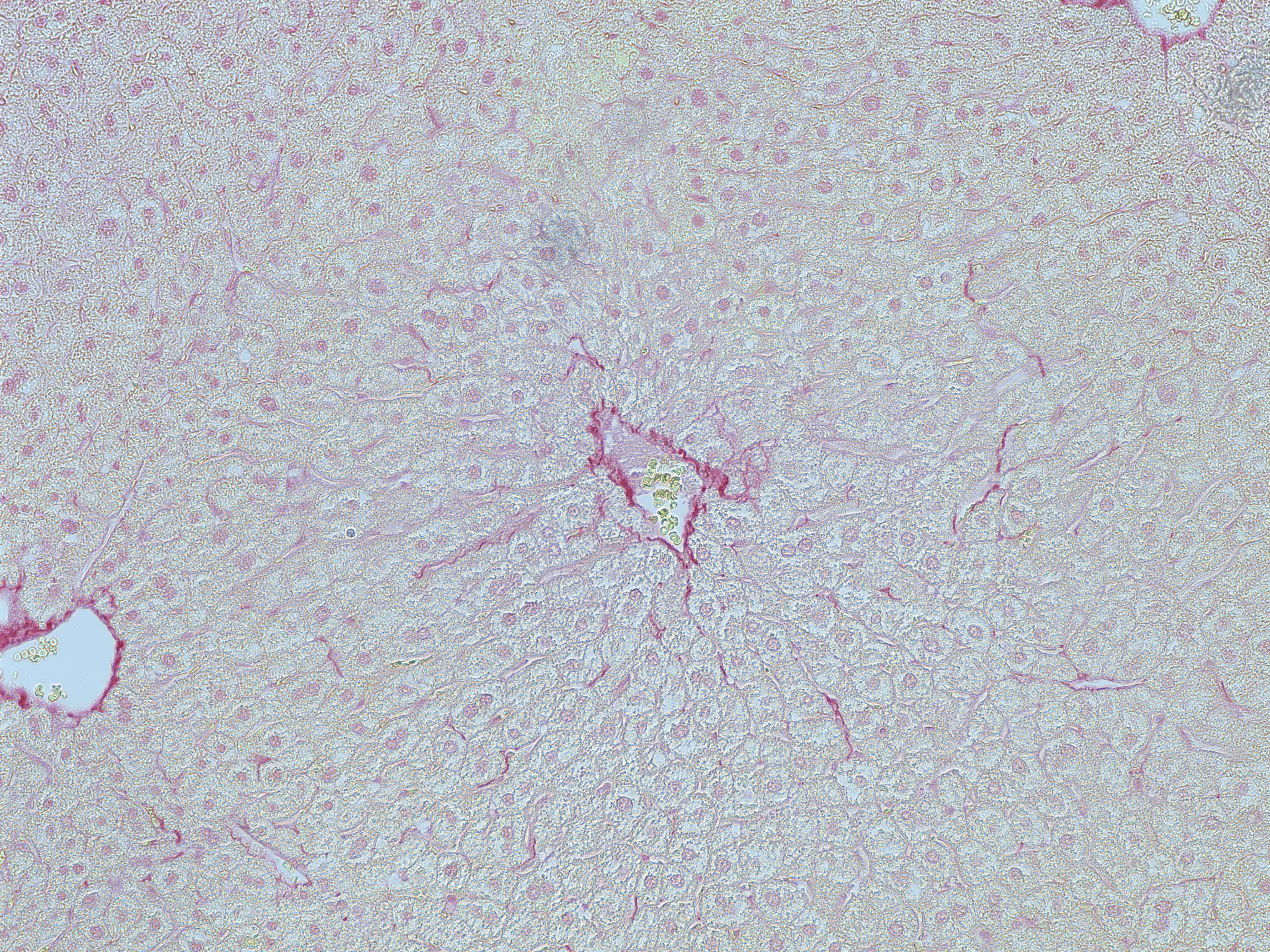

Supplement: Supplementary file 9 — Figure EV3 Source Data [file 44318_2024_196_MOESM9_ESM.zip › Figure EV3/Figure EV3-F/Quantificated image/NC Con/no.5/NC Con no.5-20x-1.jpg]

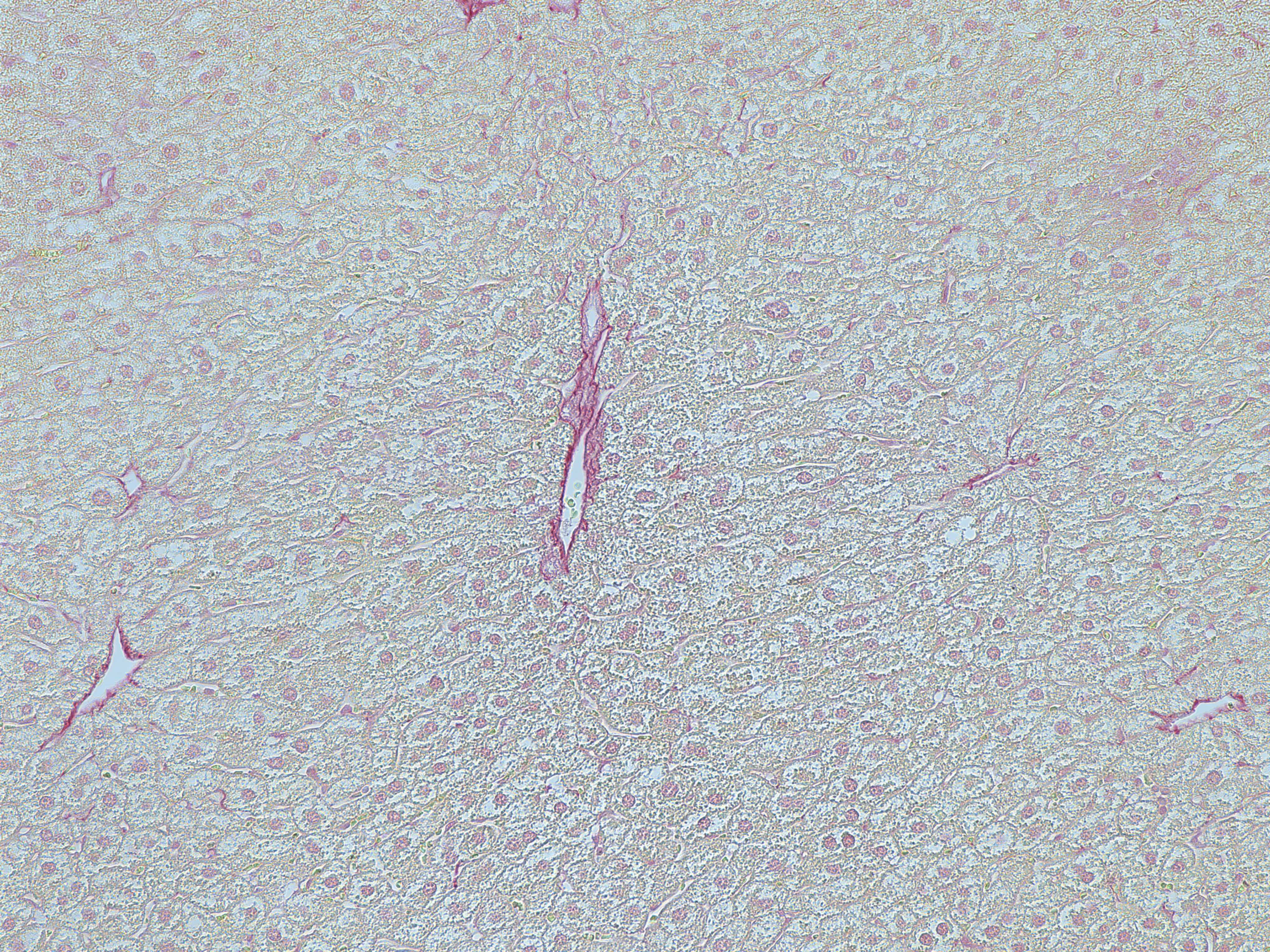

Supplement: Supplementary file 9 — Figure EV3 Source Data [file 44318_2024_196_MOESM9_ESM.zip › Figure EV3/Figure EV3-F/Quantificated image/NC Con/no.5/NC Con no.5-20x-3.jpg]

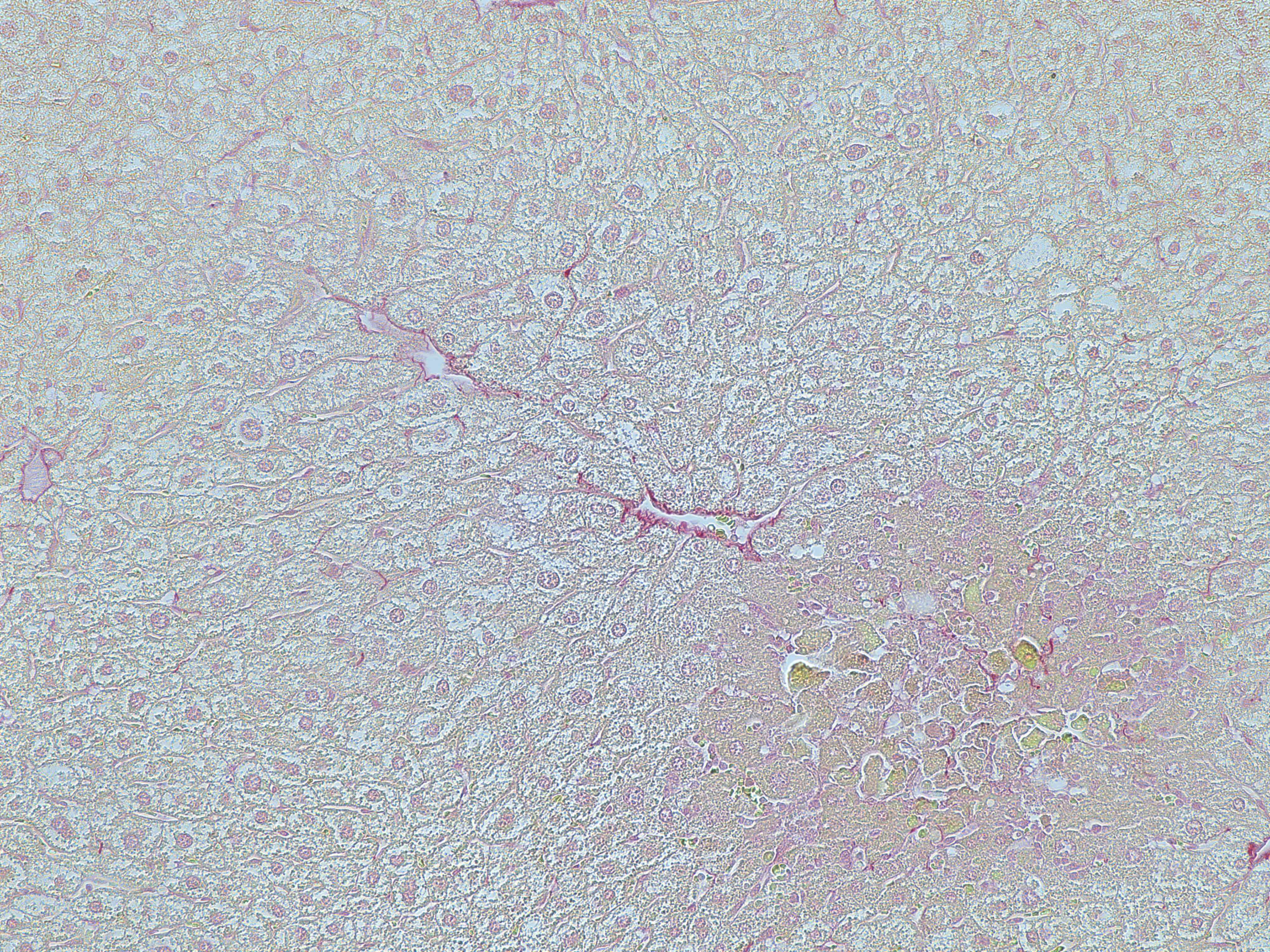

Supplement: Supplementary file 9 — Figure EV3 Source Data [file 44318_2024_196_MOESM9_ESM.zip › Figure EV3/Figure EV3-F/Quantificated image/NC Con/no.5/NC Con no.5-20x-2.jpg]

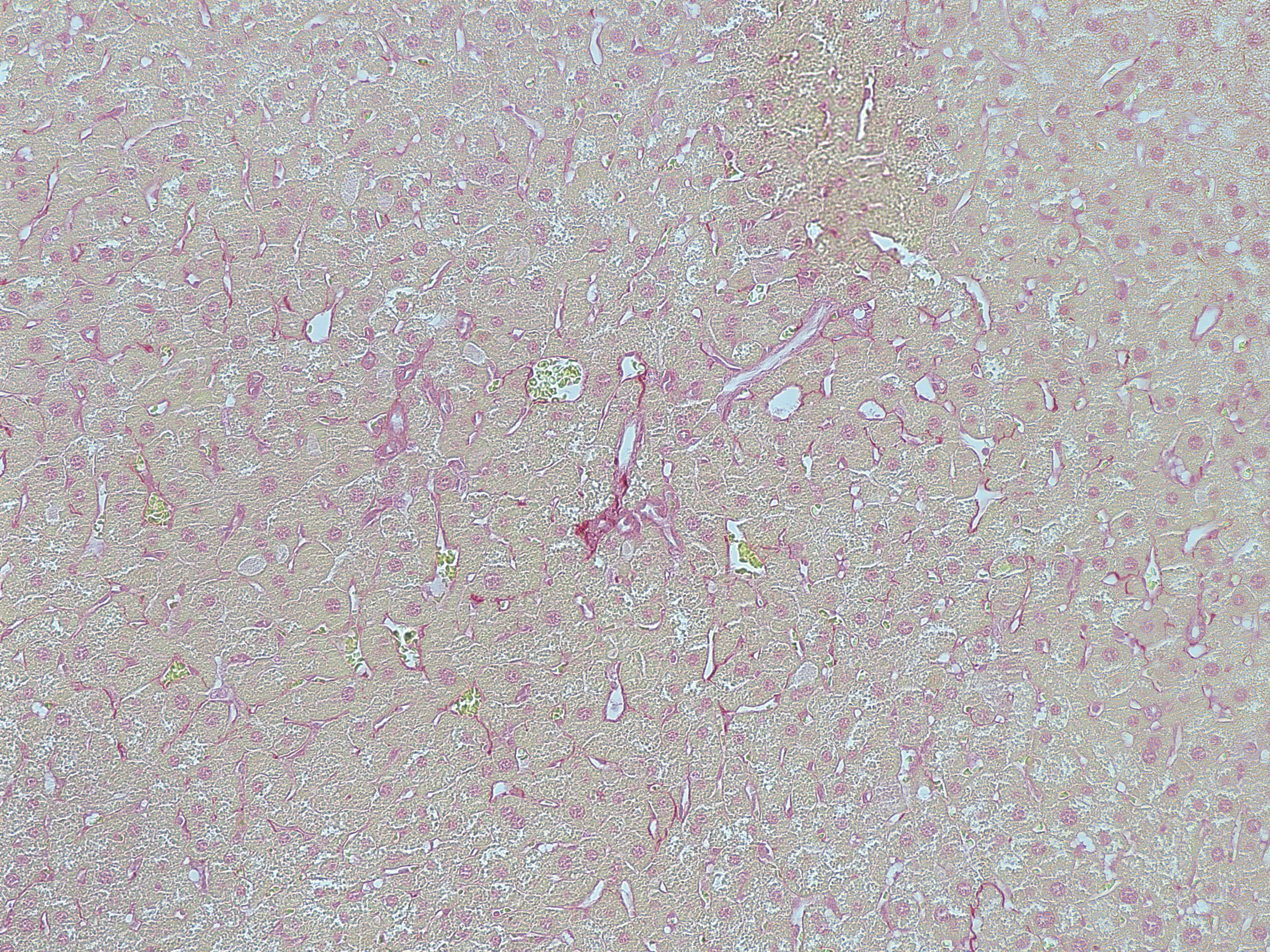

Supplement: Supplementary file 9 — Figure EV3 Source Data [file 44318_2024_196_MOESM9_ESM.zip › Figure EV3/Figure EV3-F/Quantificated image/NC Con/no.2/NC Con no.2-20x-1.jpg]

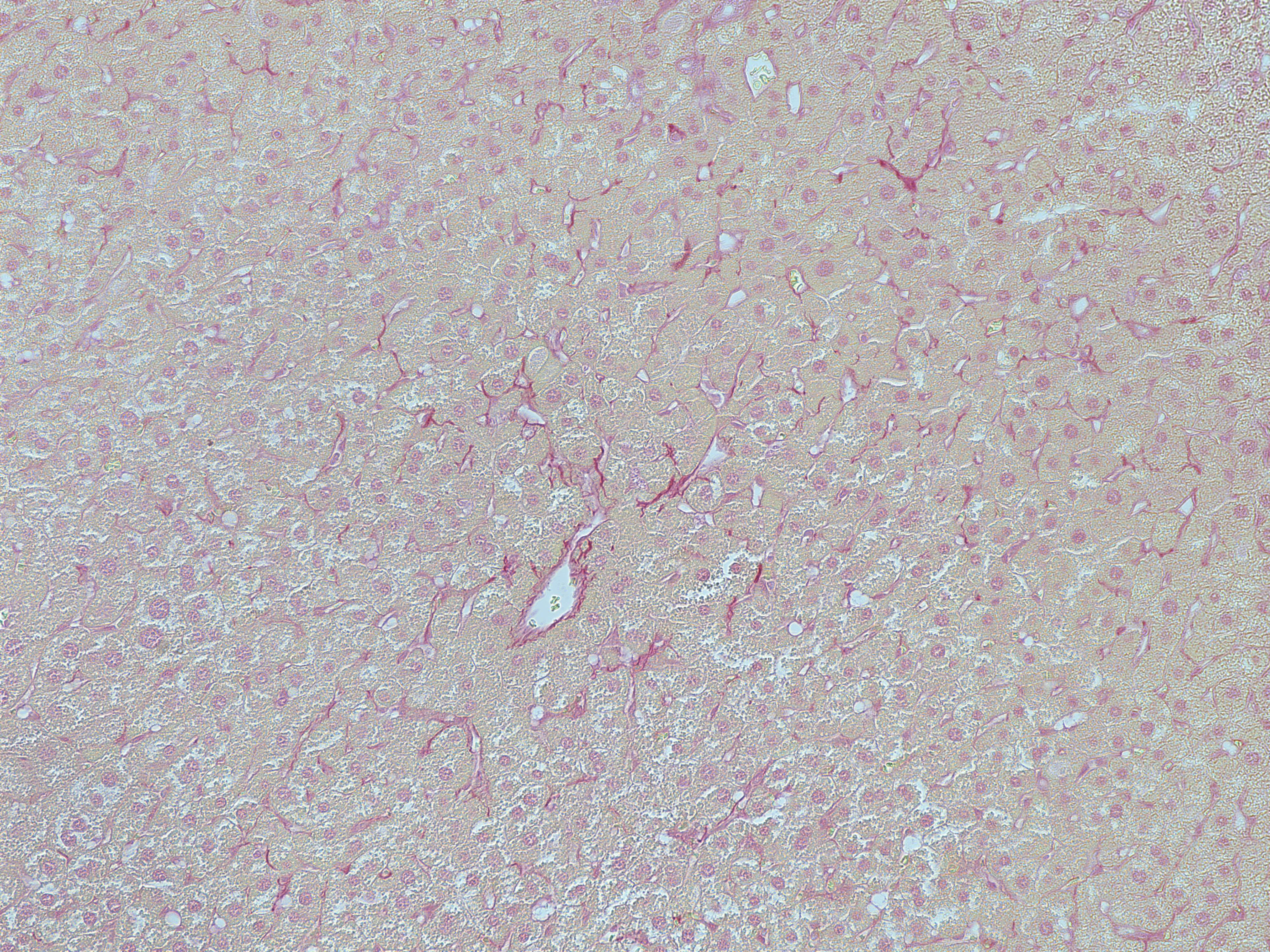

Supplement: Supplementary file 9 — Figure EV3 Source Data [file 44318_2024_196_MOESM9_ESM.zip › Figure EV3/Figure EV3-F/Quantificated image/NC Con/no.2/NC Con no.2-20x-2.jpg]

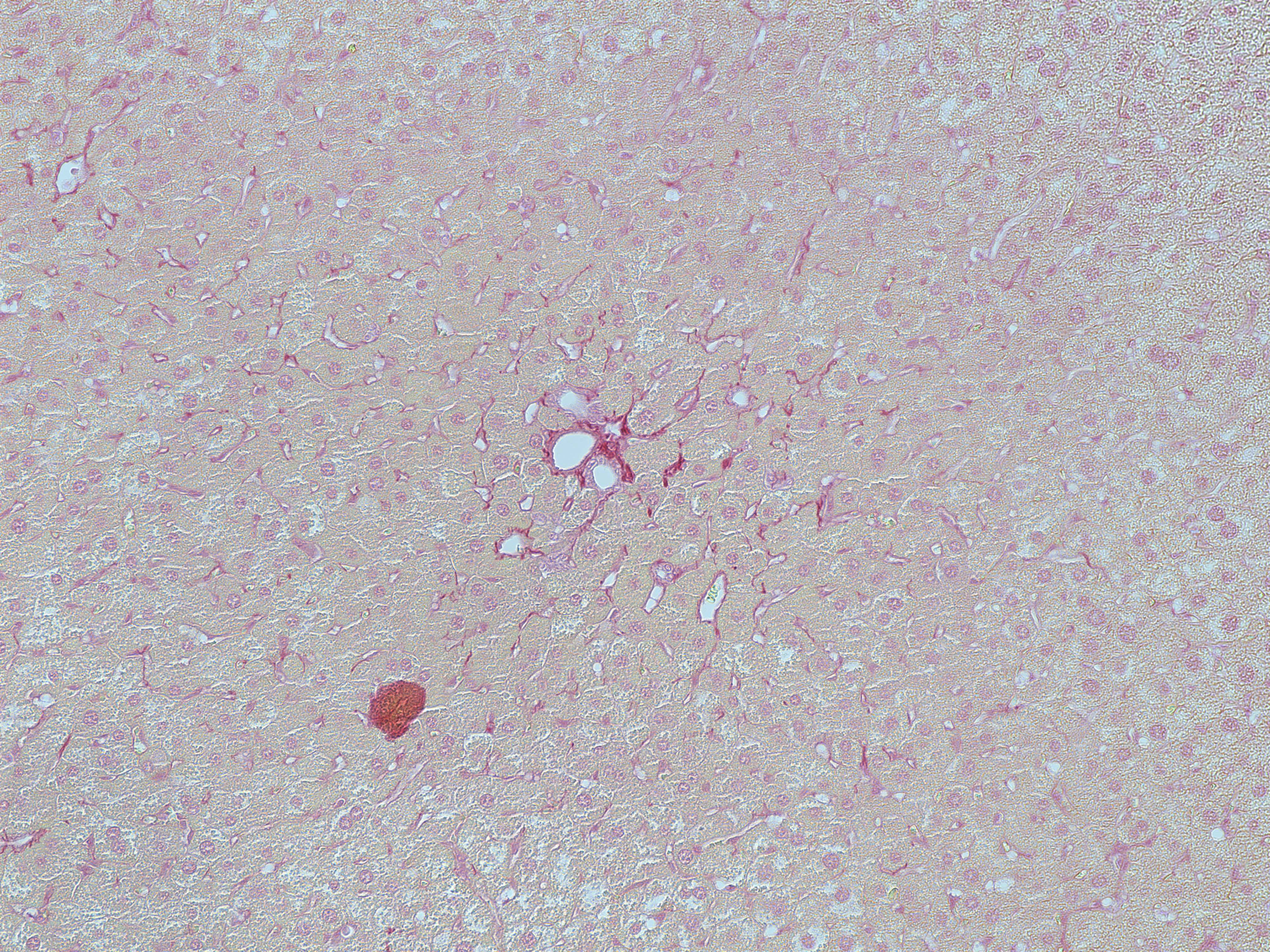

Supplement: Supplementary file 9 — Figure EV3 Source Data [file 44318_2024_196_MOESM9_ESM.zip › Figure EV3/Figure EV3-F/Quantificated image/NC Con/no.2/NC Con no.2-20x-3.jpg]

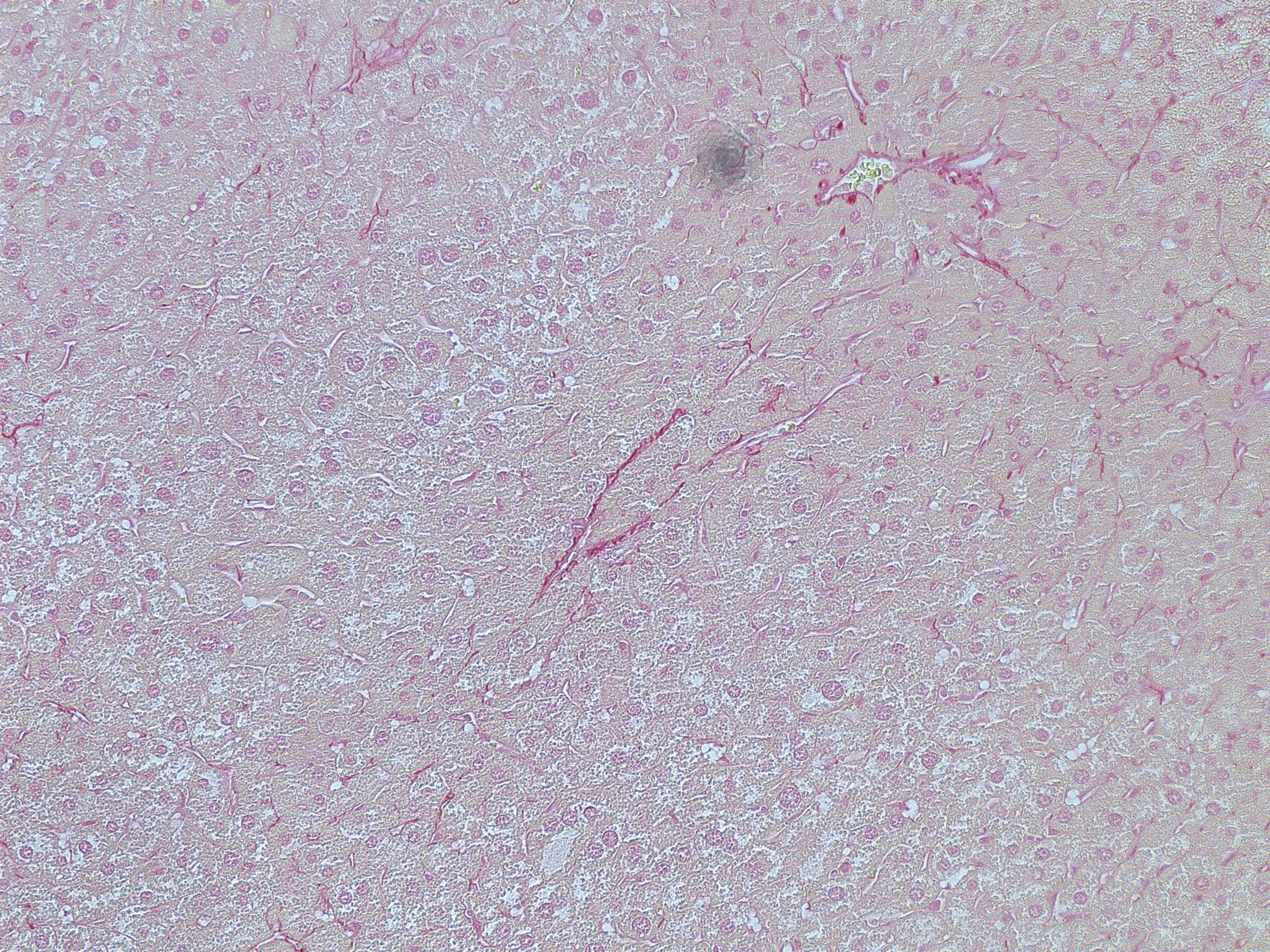

Supplement: Supplementary file 9 — Figure EV3 Source Data [file 44318_2024_196_MOESM9_ESM.zip › Figure EV3/Figure EV3-F/Quantificated image/NC Con/no.2/NC Con no.2-20x-4.jpg]

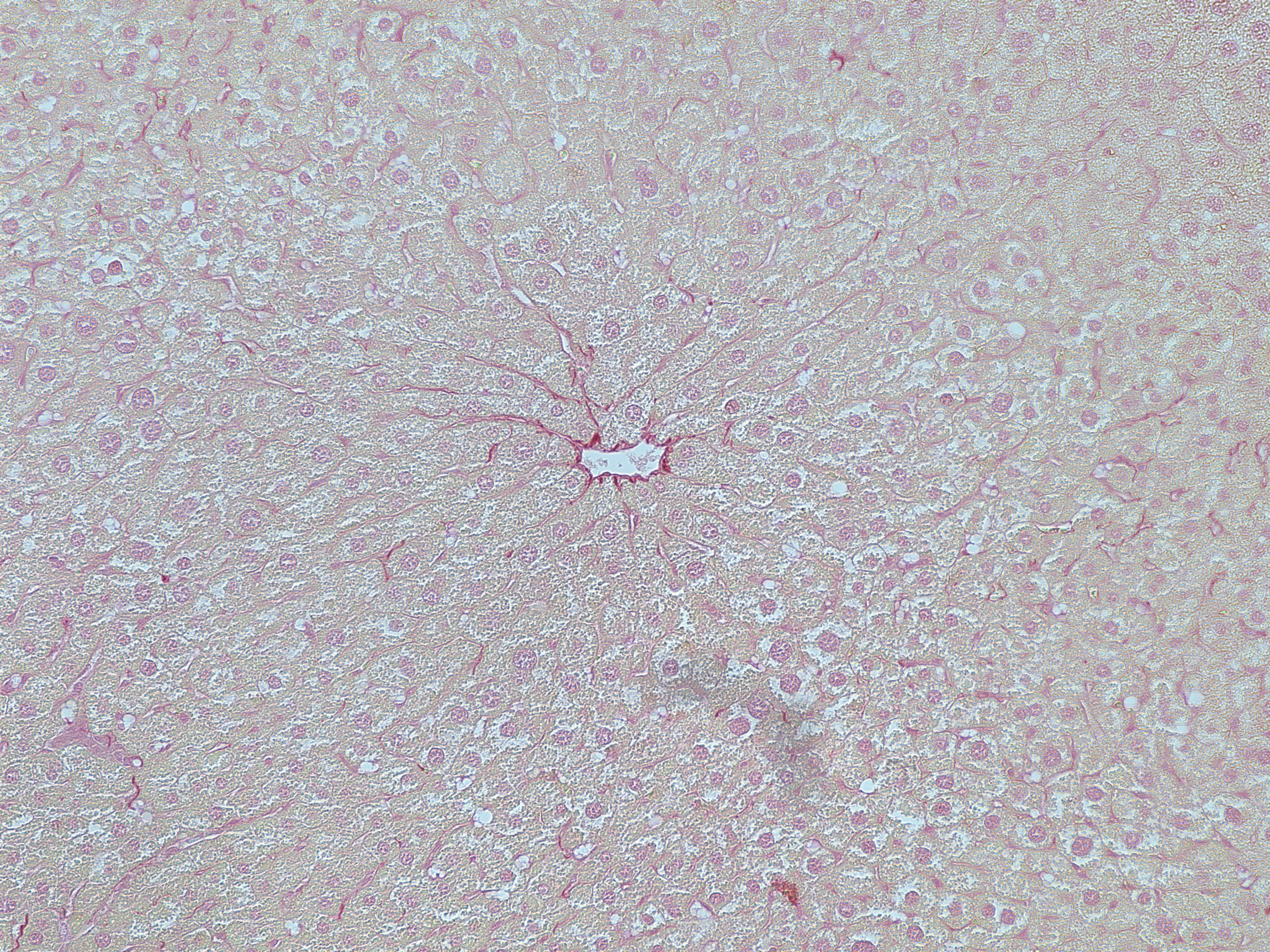

Supplement: Supplementary file 9 — Figure EV3 Source Data [file 44318_2024_196_MOESM9_ESM.zip › Figure EV3/Figure EV3-F/Quantificated image/NC Con/no.2/NC Con no.2-20x-5.jpg]

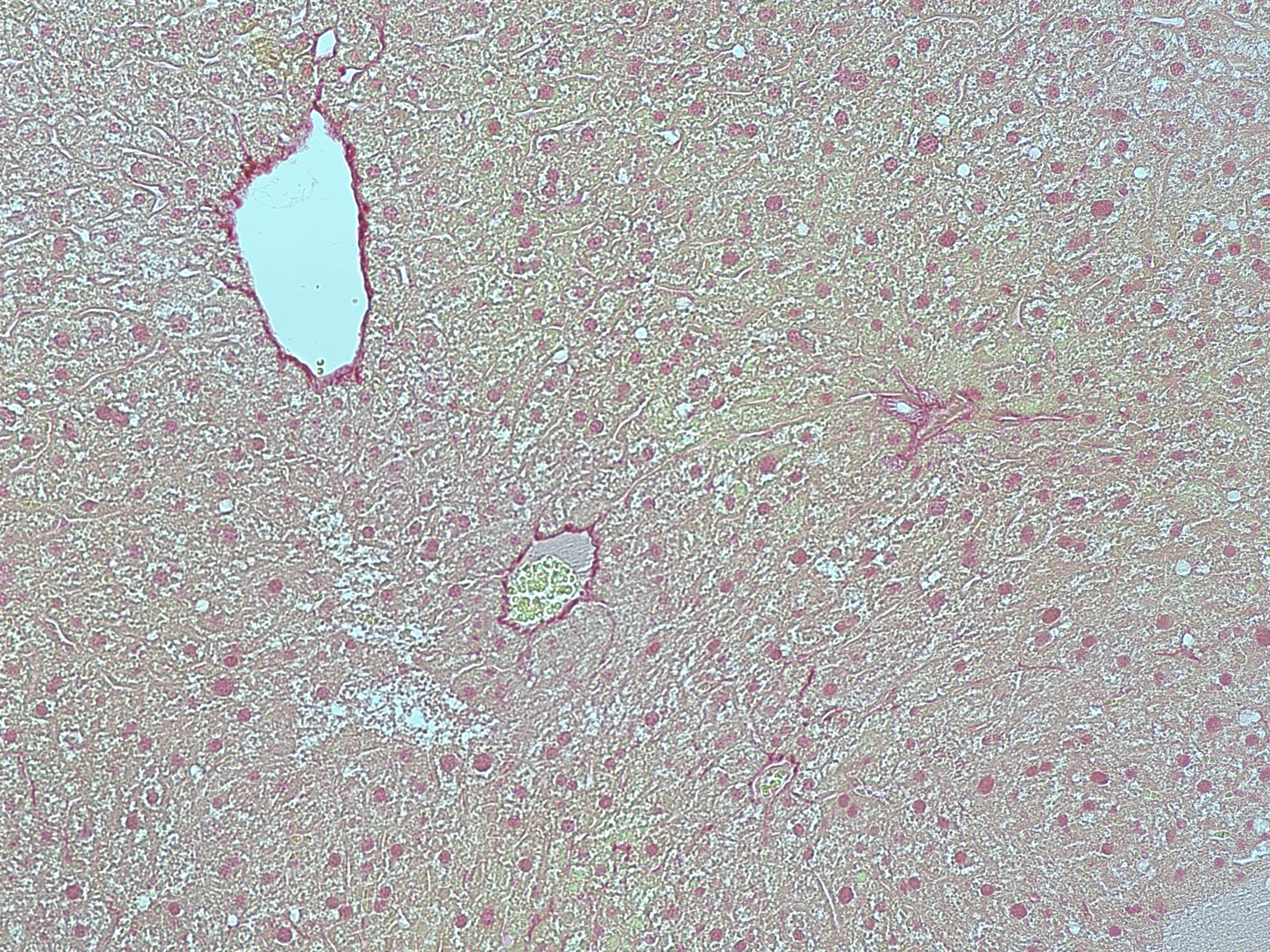

Supplement: Supplementary file 9 — Figure EV3 Source Data [file 44318_2024_196_MOESM9_ESM.zip › Figure EV3/Figure EV3-F/Quantificated image/NC Pcolce KO/no.1/NC Pcolce KO no.1-20x-3.jpeg]

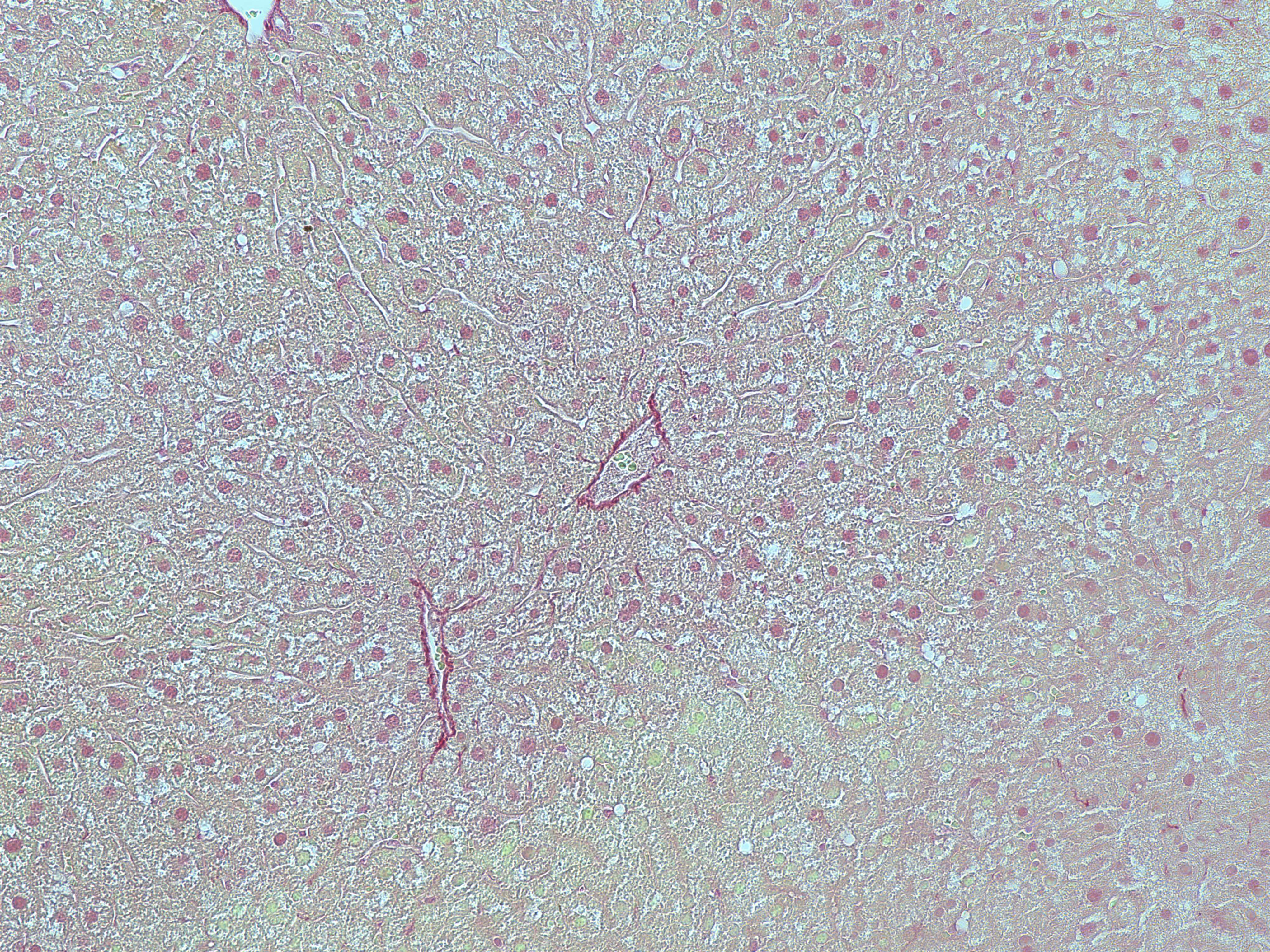

Supplement: Supplementary file 9 — Figure EV3 Source Data [file 44318_2024_196_MOESM9_ESM.zip › Figure EV3/Figure EV3-F/Quantificated image/NC Pcolce KO/no.1/NC Pcolce KO no.1-20x-2.jpeg]

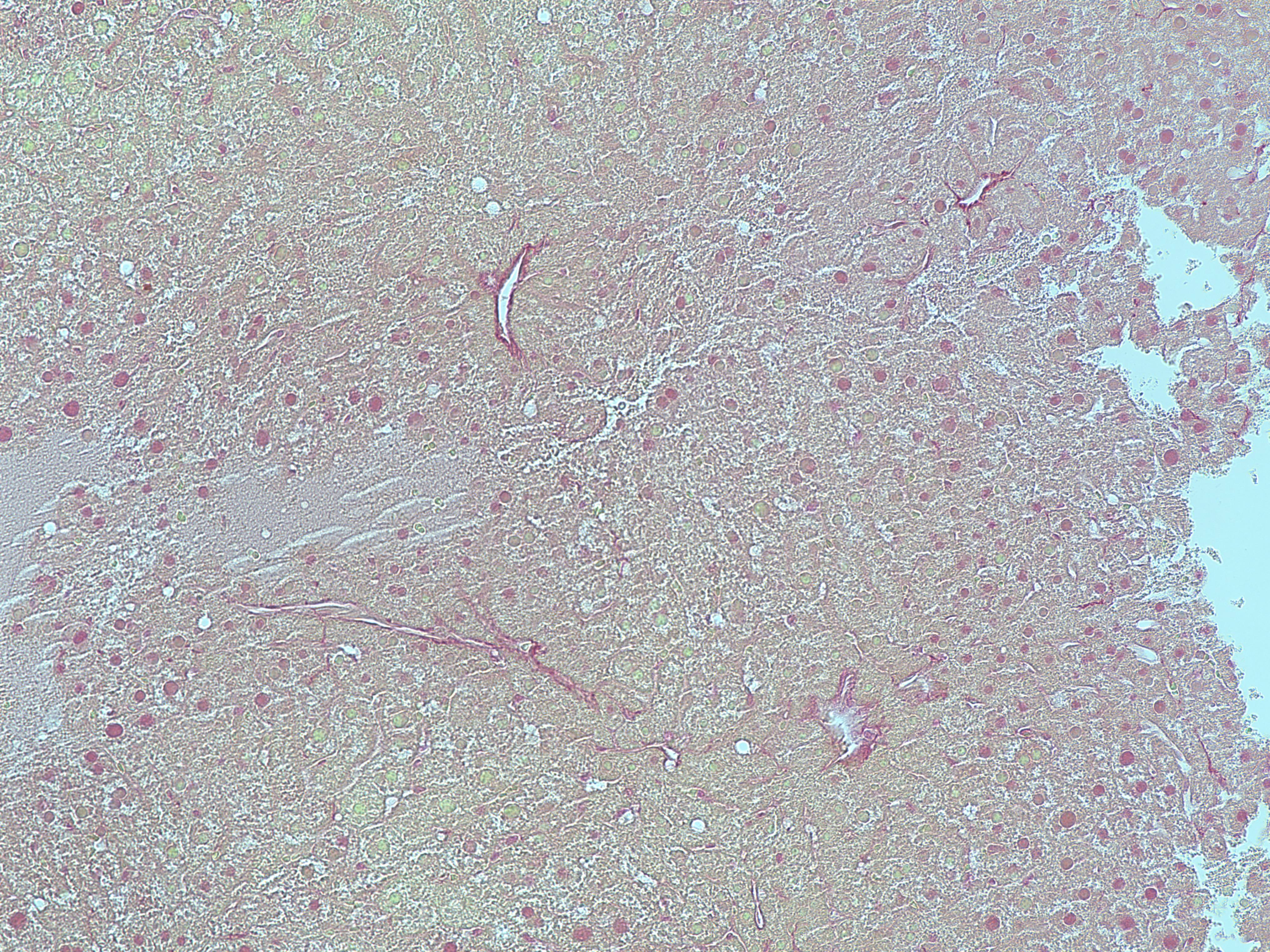

Supplement: Supplementary file 9 — Figure EV3 Source Data [file 44318_2024_196_MOESM9_ESM.zip › Figure EV3/Figure EV3-F/Quantificated image/NC Pcolce KO/no.1/NC Pcolce KO no.1-20x-5.jpeg]

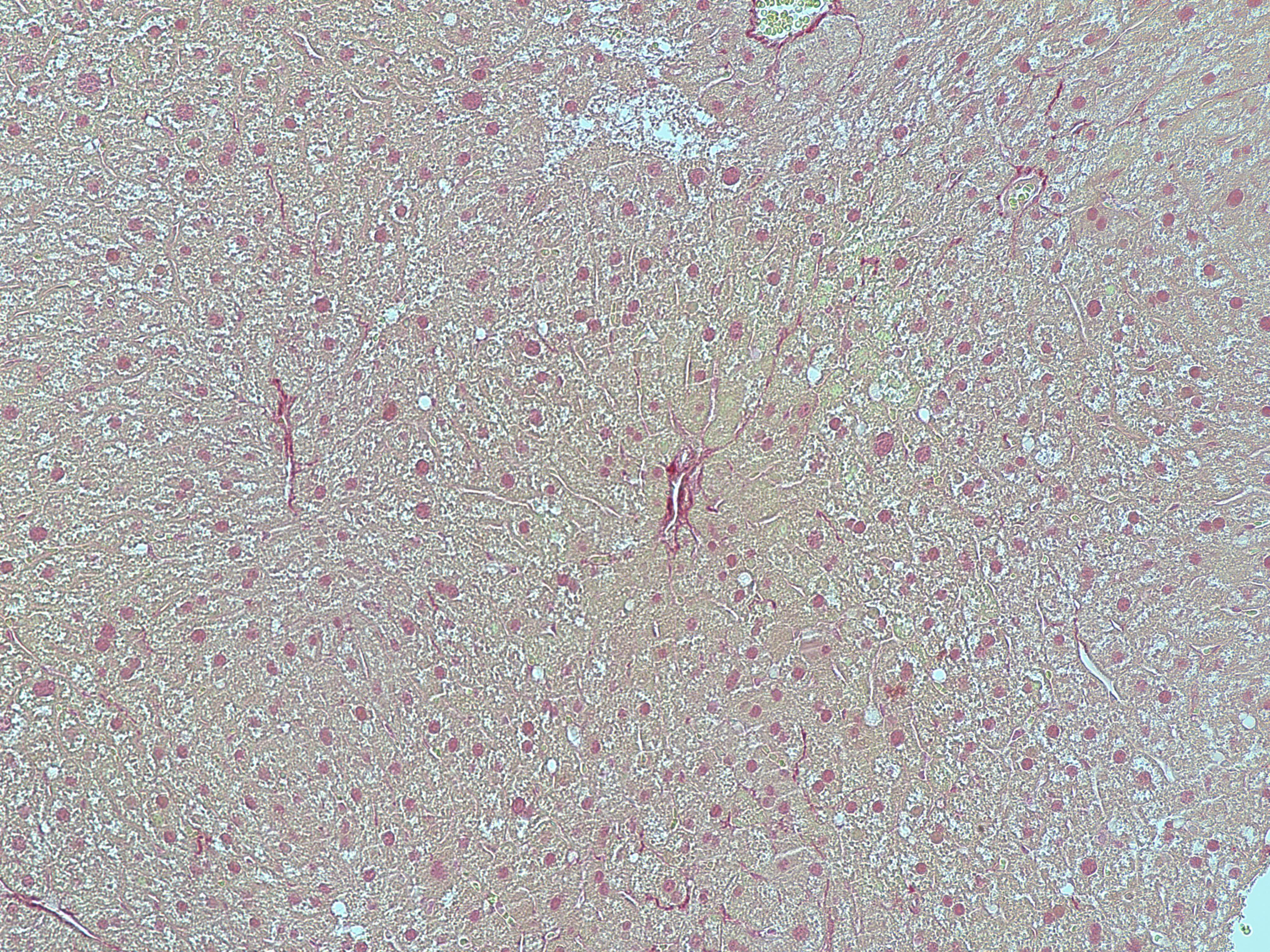

Supplement: Supplementary file 9 — Figure EV3 Source Data [file 44318_2024_196_MOESM9_ESM.zip › Figure EV3/Figure EV3-F/Quantificated image/NC Pcolce KO/no.1/NC Pcolce KO no.1-20x-4.jpeg]

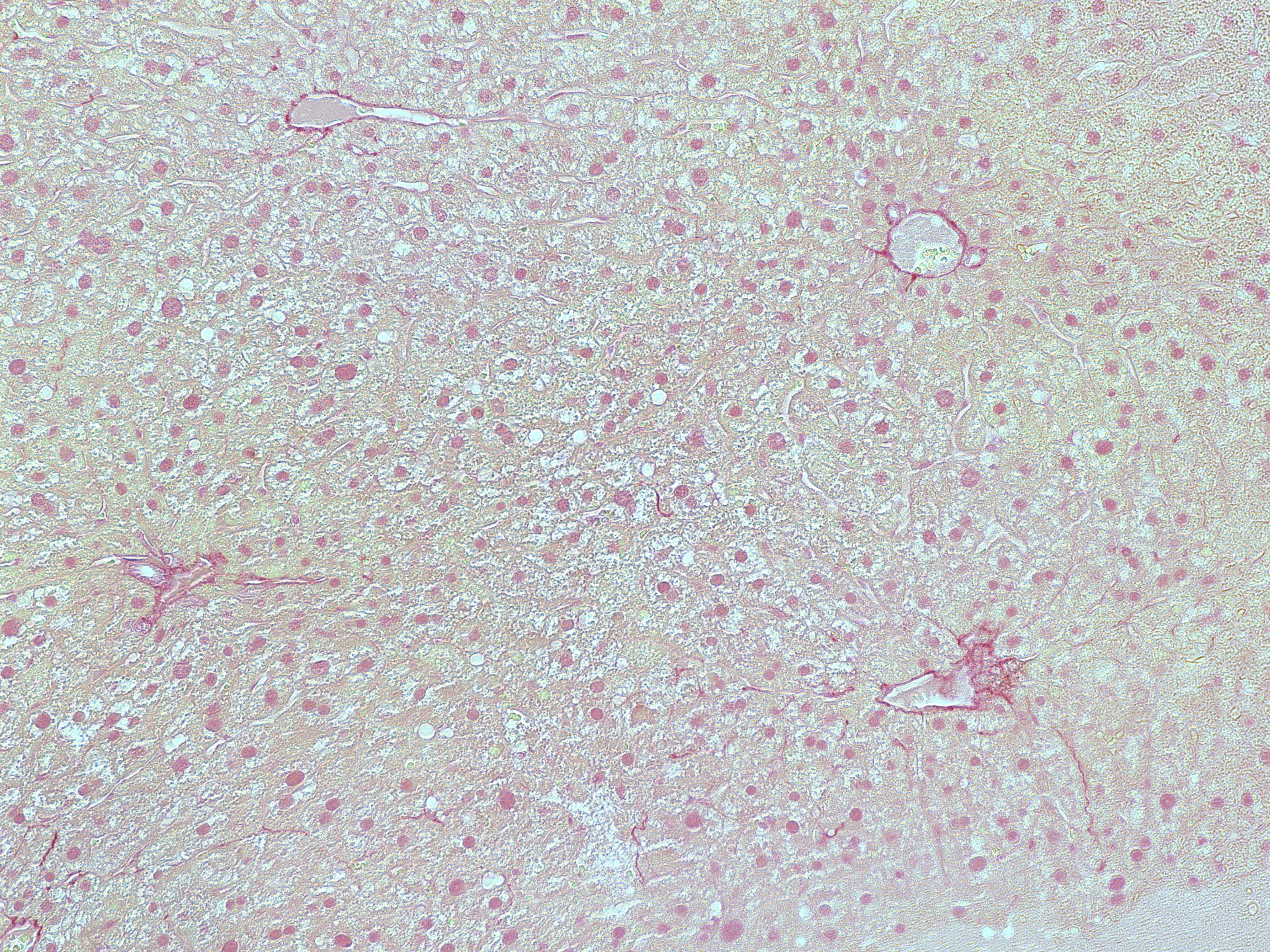

Supplement: Supplementary file 9 — Figure EV3 Source Data [file 44318_2024_196_MOESM9_ESM.zip › Figure EV3/Figure EV3-F/Quantificated image/NC Pcolce KO/no.1/NC Pcolce KO no.1-20x-1.jpeg]

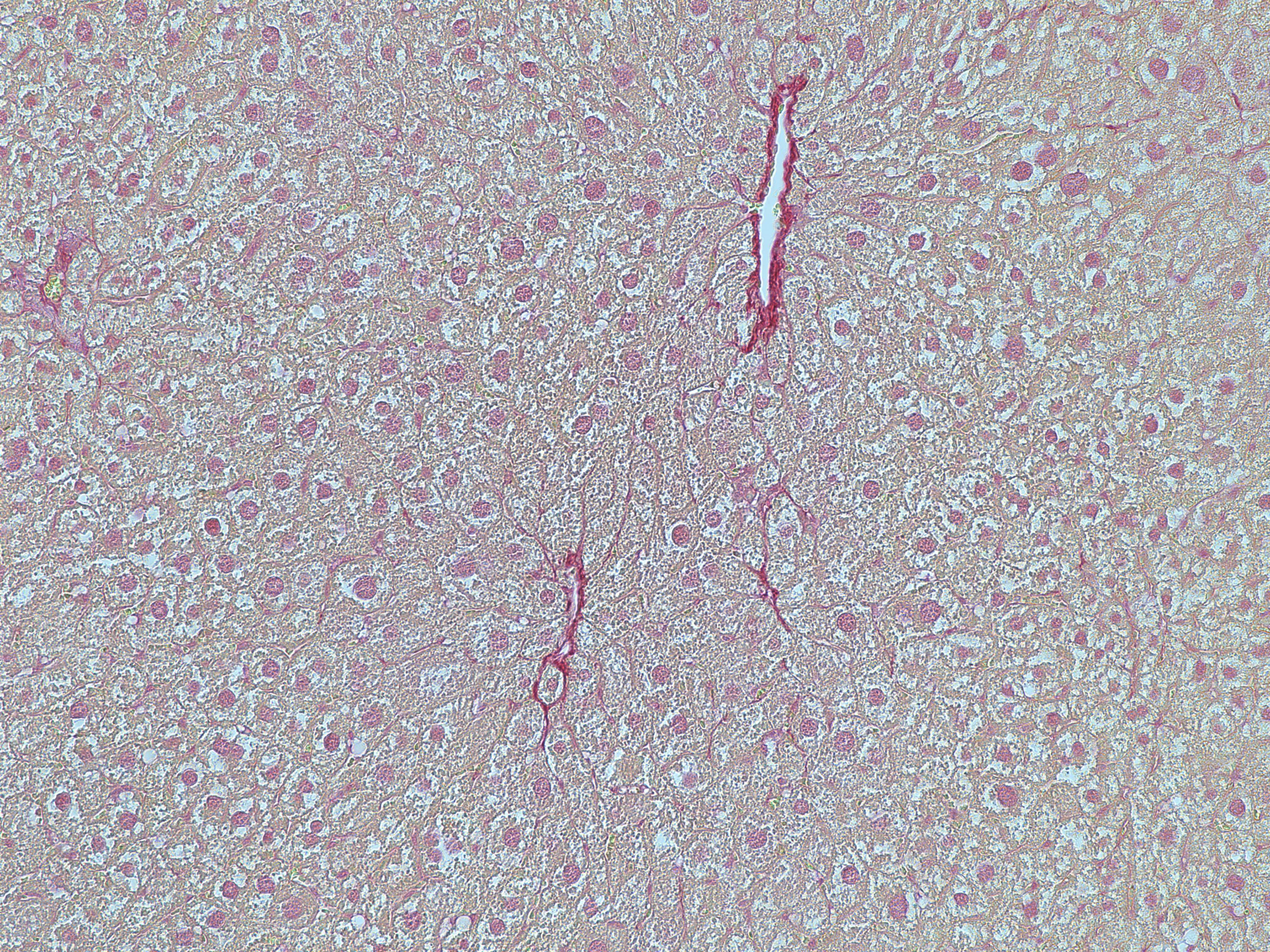

Supplement: Supplementary file 9 — Figure EV3 Source Data [file 44318_2024_196_MOESM9_ESM.zip › Figure EV3/Figure EV3-F/Quantificated image/NC Pcolce KO/no.3/NC Pcolce KO no.3-20x-1.jpg]

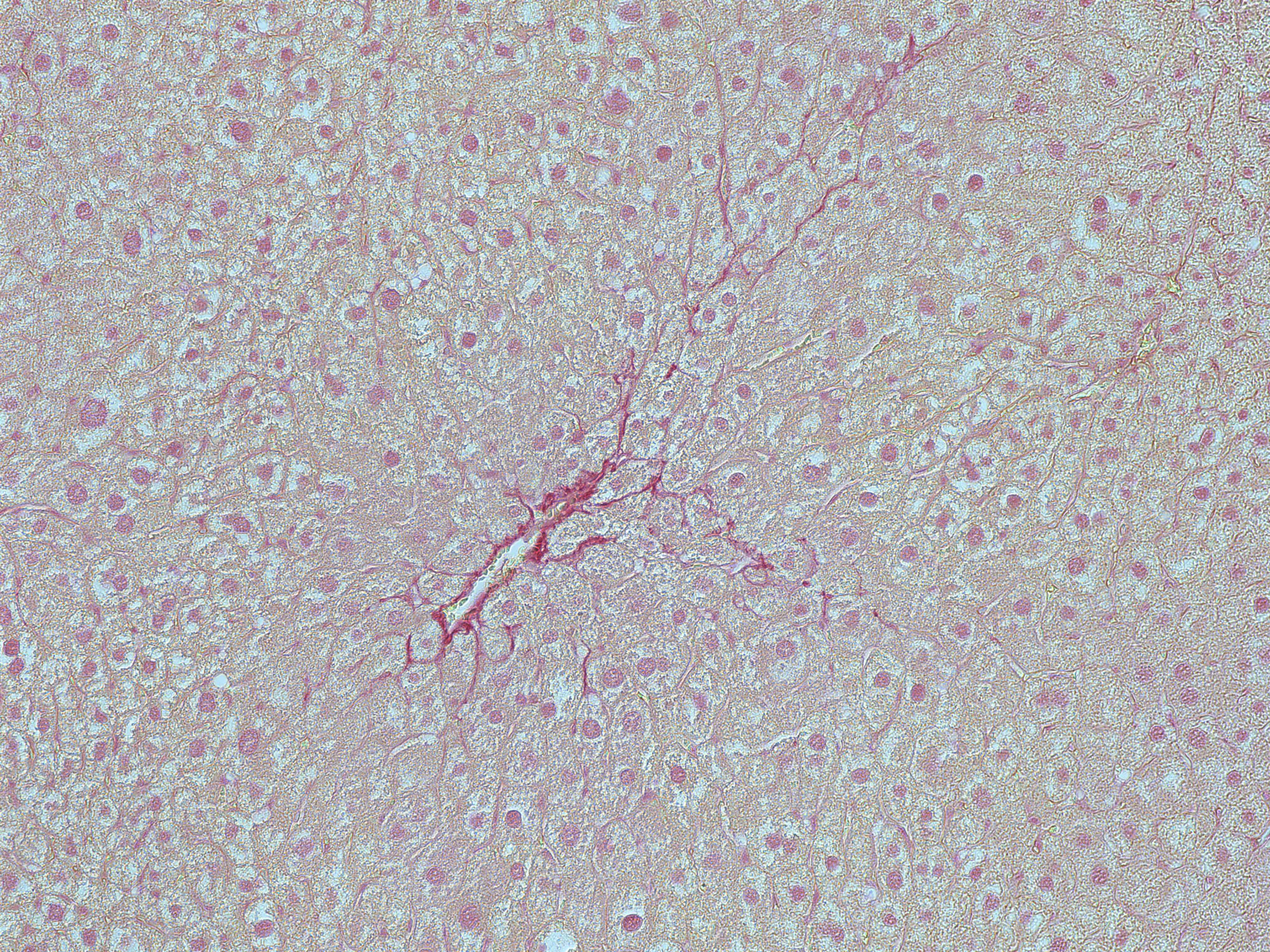

Supplement: Supplementary file 9 — Figure EV3 Source Data [file 44318_2024_196_MOESM9_ESM.zip › Figure EV3/Figure EV3-F/Quantificated image/NC Pcolce KO/no.3/NC Pcolce KO no.3-20x-2.jpg]

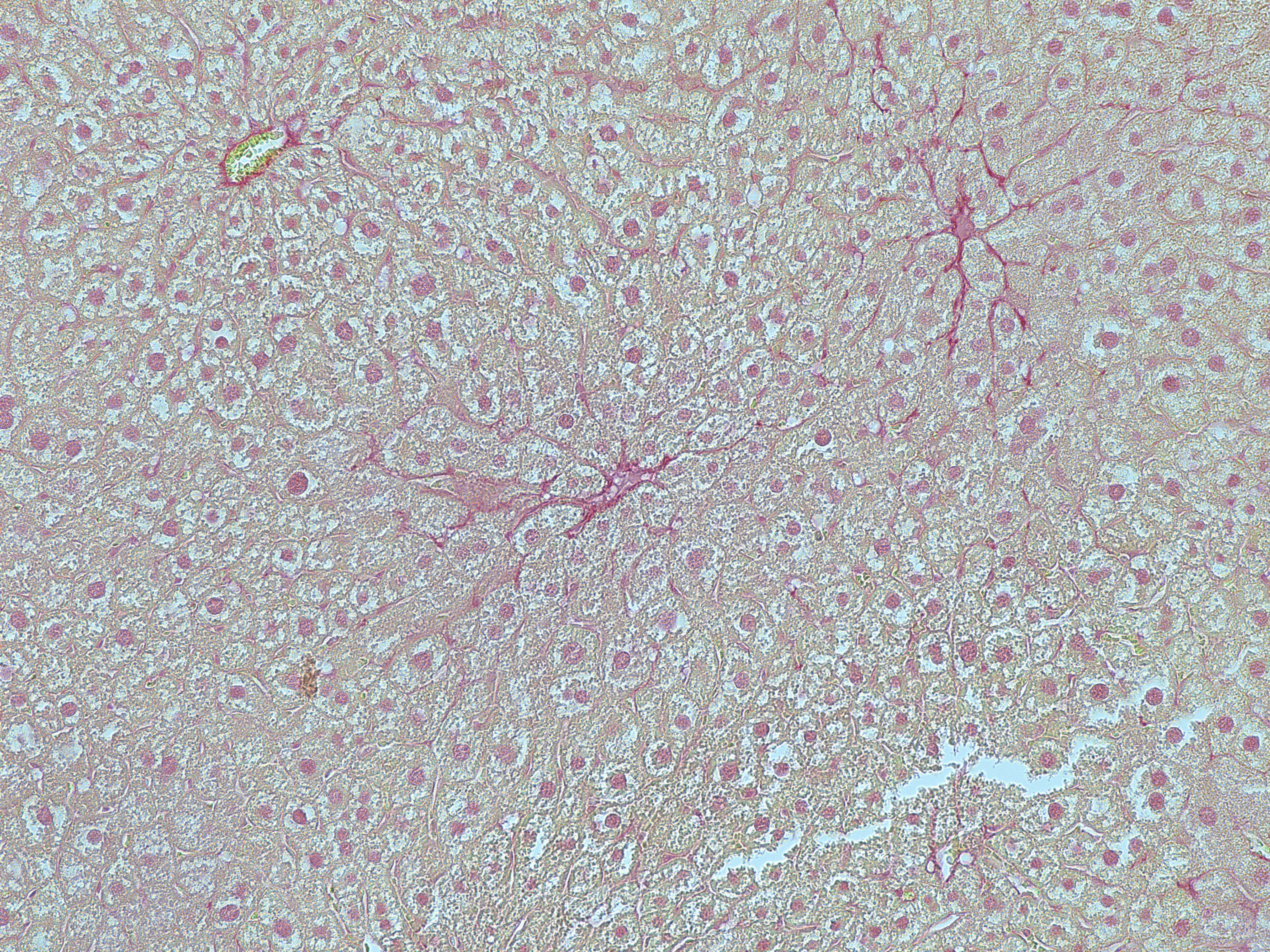

Supplement: Supplementary file 9 — Figure EV3 Source Data [file 44318_2024_196_MOESM9_ESM.zip › Figure EV3/Figure EV3-F/Quantificated image/NC Pcolce KO/no.3/NC Pcolce KO no.3-20x-3.jpg]

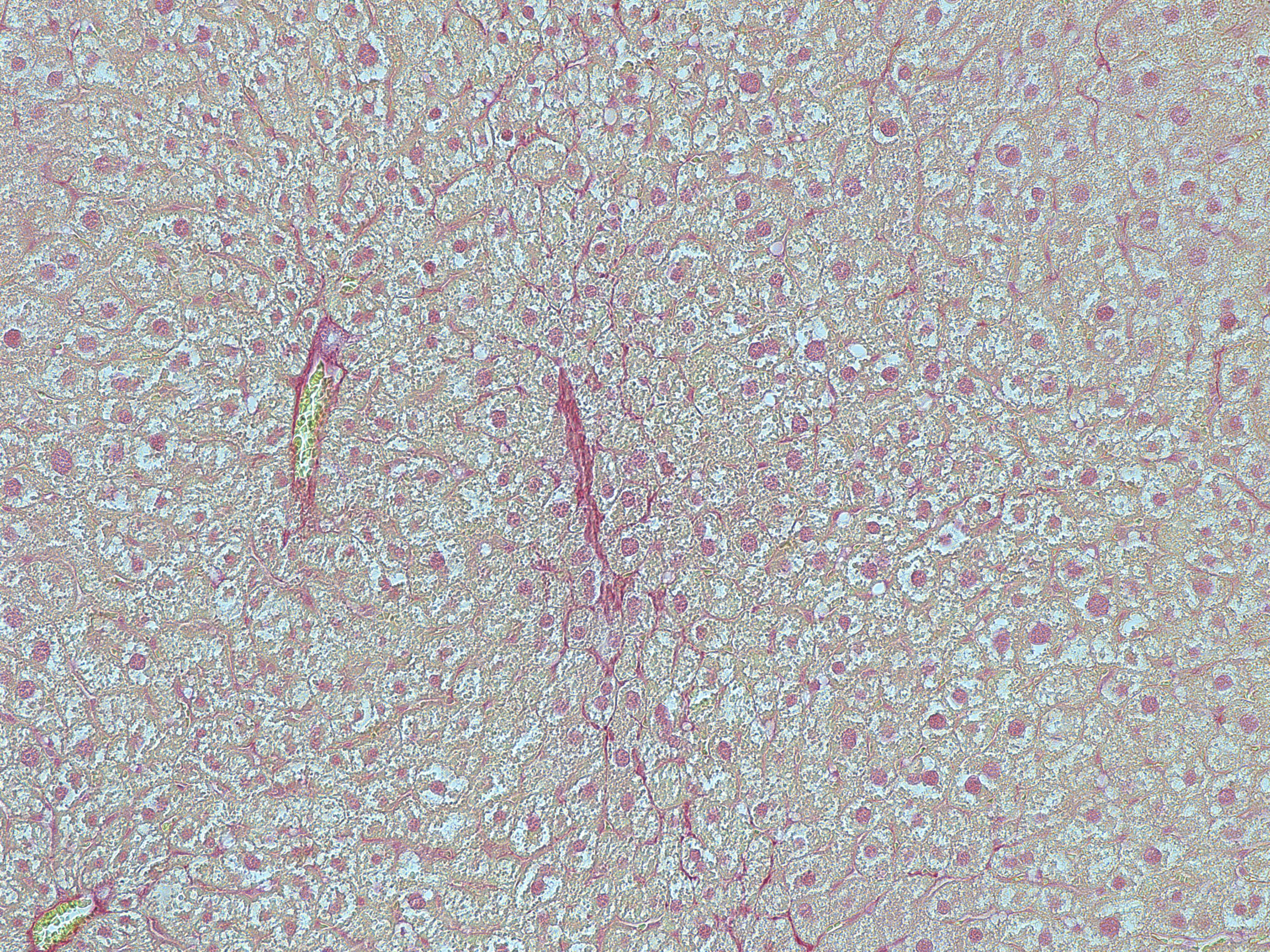

Supplement: Supplementary file 9 — Figure EV3 Source Data [file 44318_2024_196_MOESM9_ESM.zip › Figure EV3/Figure EV3-F/Quantificated image/NC Pcolce KO/no.3/NC Pcolce KO no.3-20x-4.jpg]

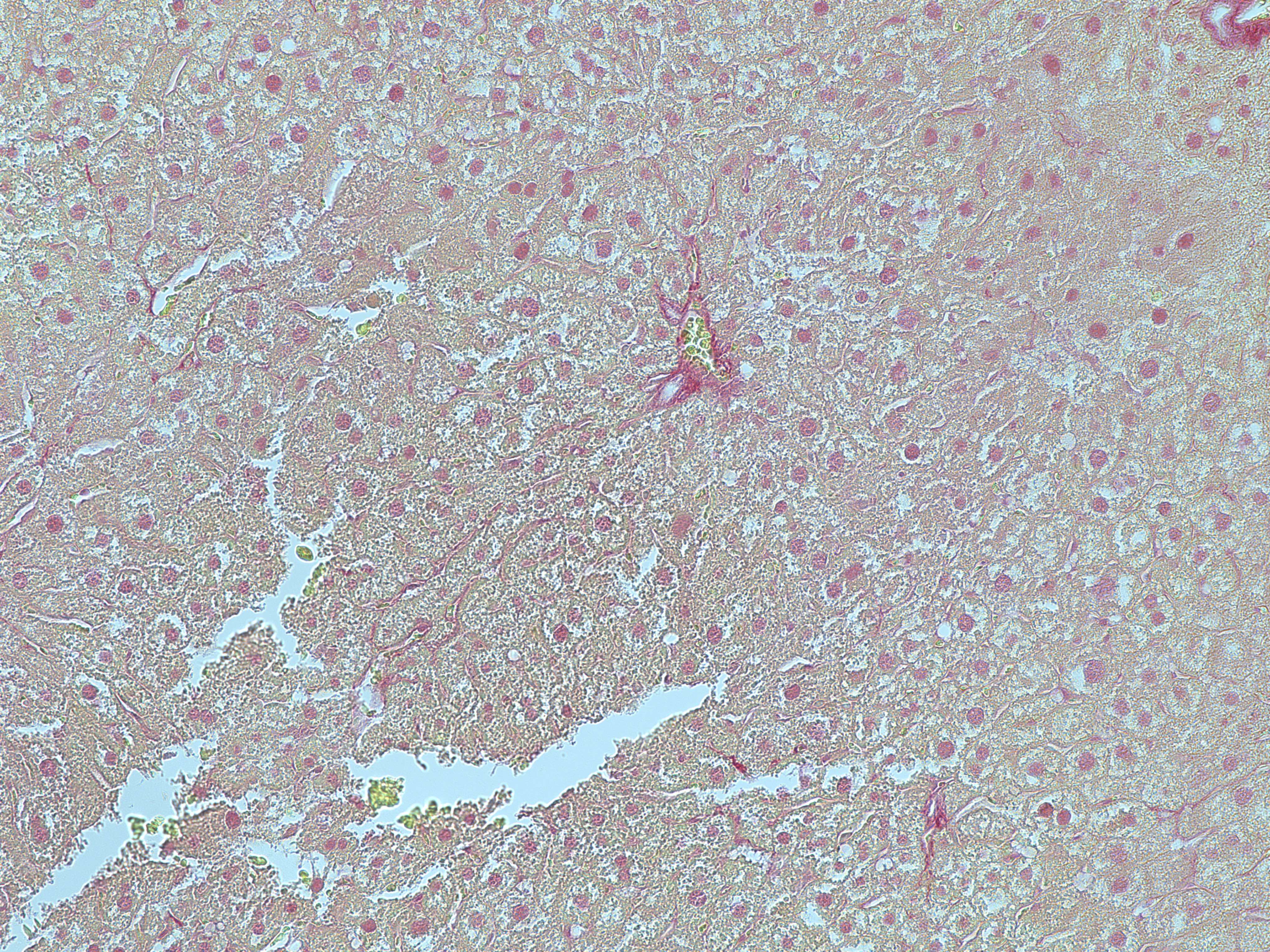

Supplement: Supplementary file 9 — Figure EV3 Source Data [file 44318_2024_196_MOESM9_ESM.zip › Figure EV3/Figure EV3-F/Quantificated image/NC Pcolce KO/no.3/NC Pcolce KO no.3-20x-5.jpg]

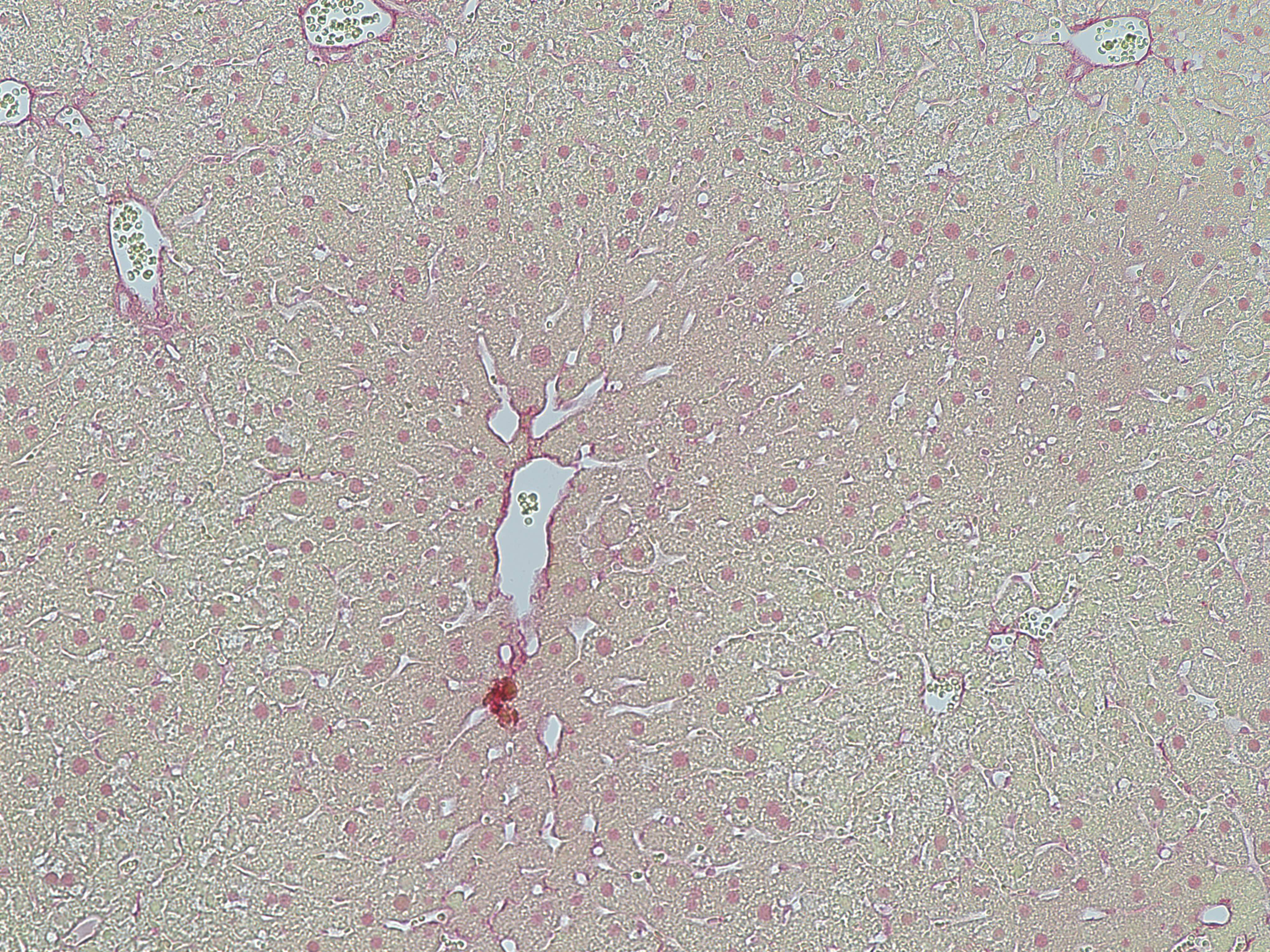

Supplement: Supplementary file 9 — Figure EV3 Source Data [file 44318_2024_196_MOESM9_ESM.zip › Figure EV3/Figure EV3-F/Quantificated image/NC Pcolce KO/no.4/NC Pcolce KO no.4-20x-5.jpg]

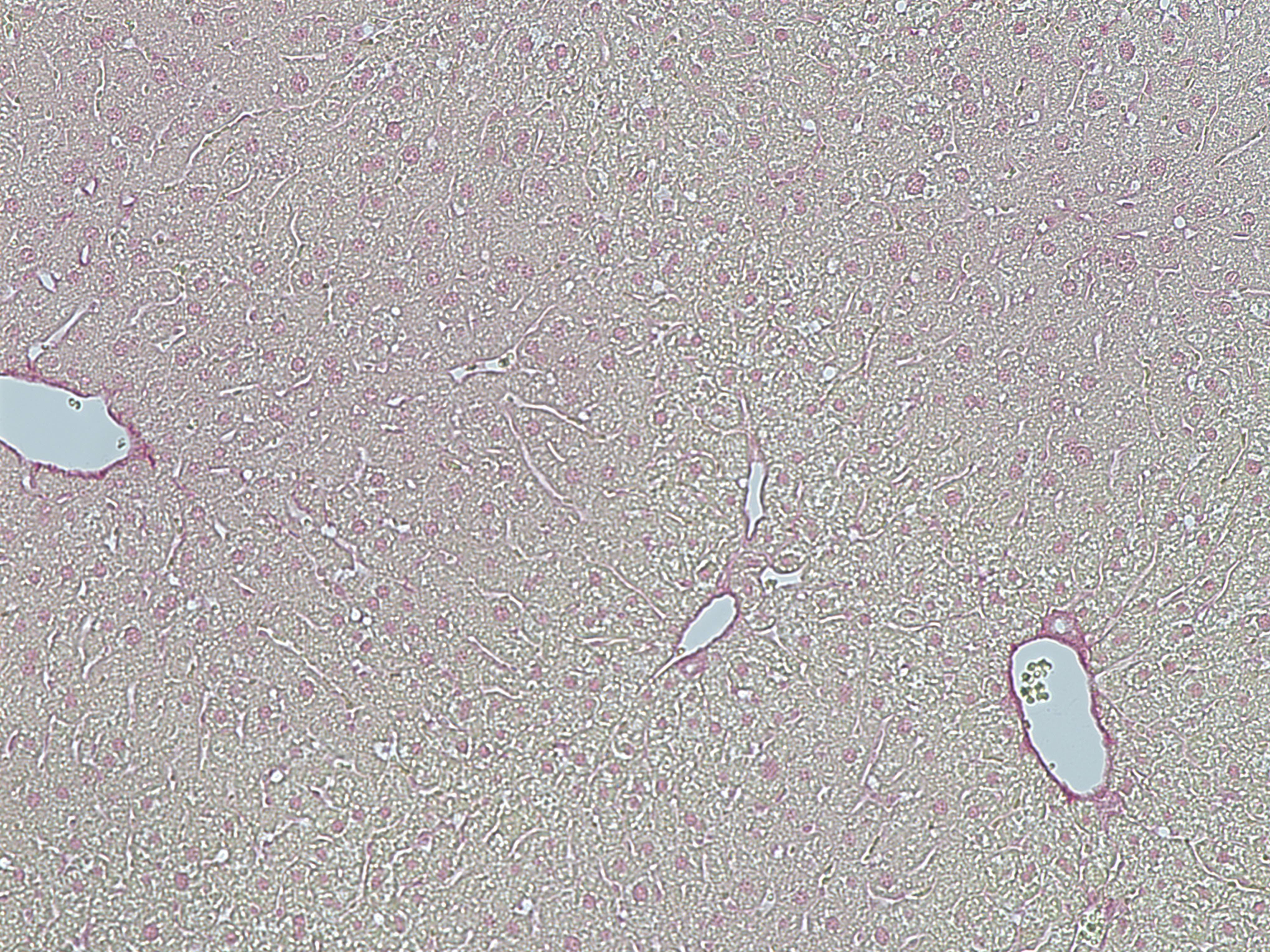

Supplement: Supplementary file 9 — Figure EV3 Source Data [file 44318_2024_196_MOESM9_ESM.zip › Figure EV3/Figure EV3-F/Quantificated image/NC Pcolce KO/no.4/NC Pcolce KO no.4-20x-4.jpg]

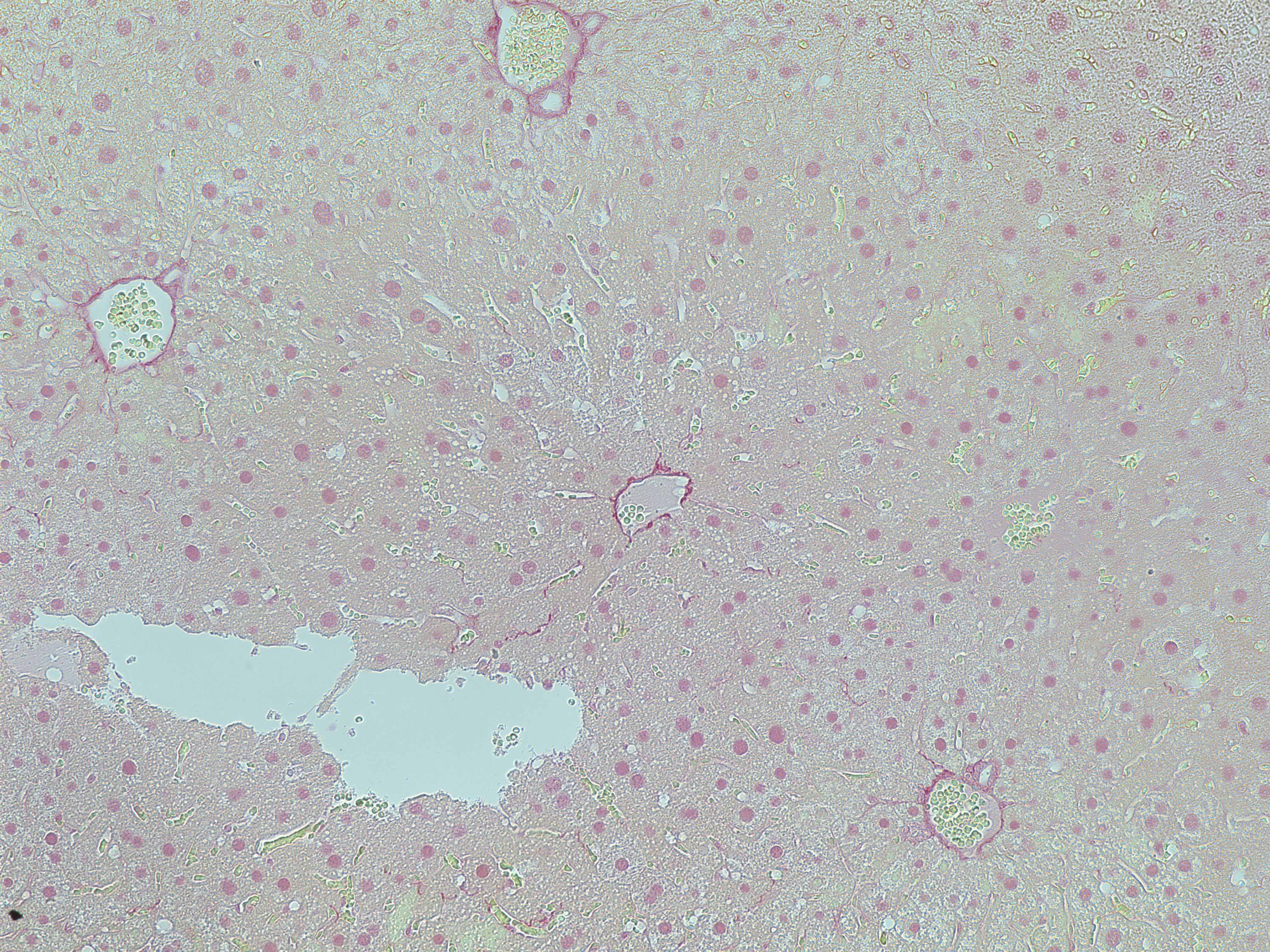

Supplement: Supplementary file 9 — Figure EV3 Source Data [file 44318_2024_196_MOESM9_ESM.zip › Figure EV3/Figure EV3-F/Quantificated image/NC Pcolce KO/no.4/NC Pcolce KO no.4-20x-1.jpg]

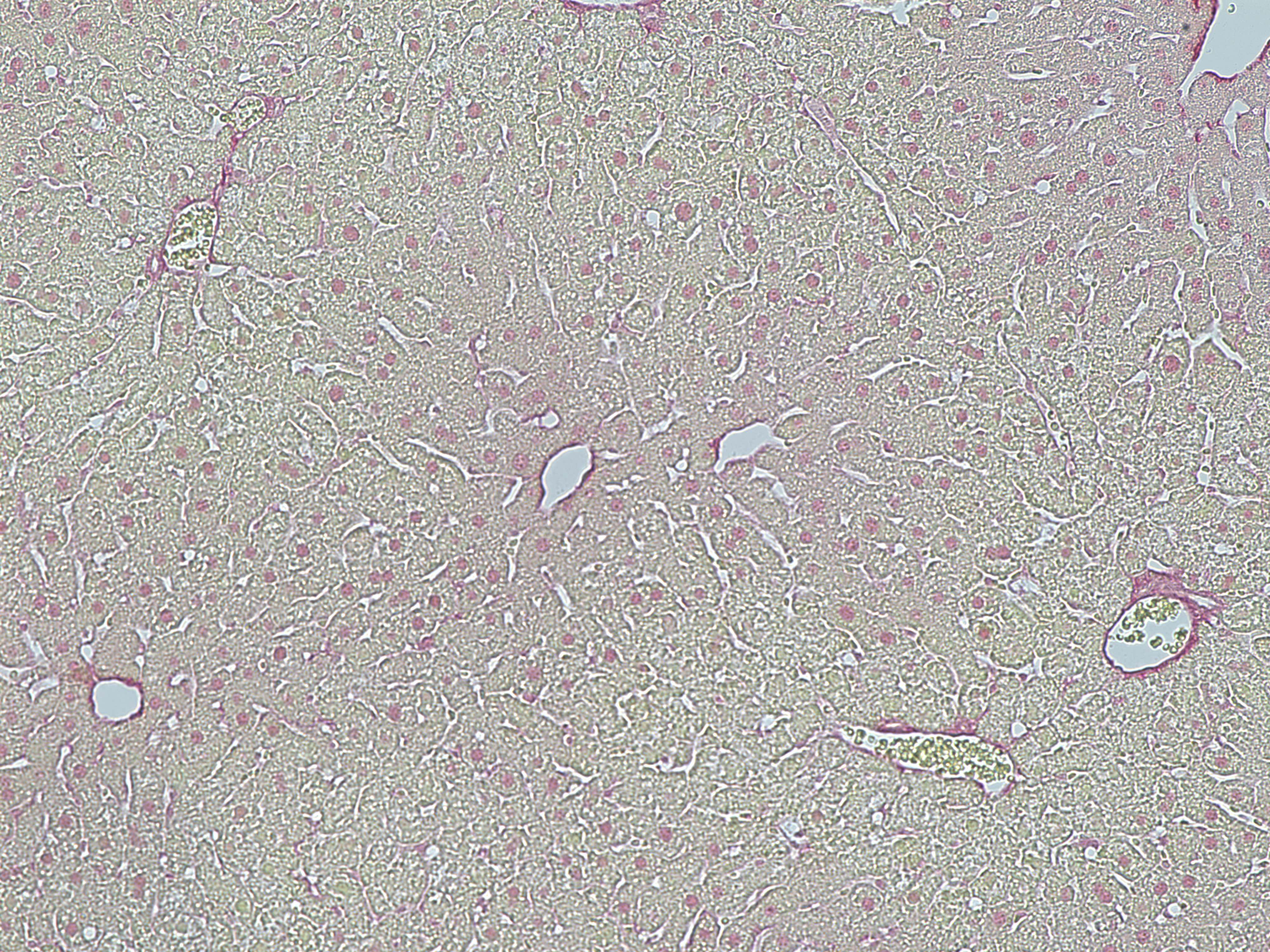

Supplement: Supplementary file 9 — Figure EV3 Source Data [file 44318_2024_196_MOESM9_ESM.zip › Figure EV3/Figure EV3-F/Quantificated image/NC Pcolce KO/no.4/NC Pcolce KO no.4-20x-3.jpg]

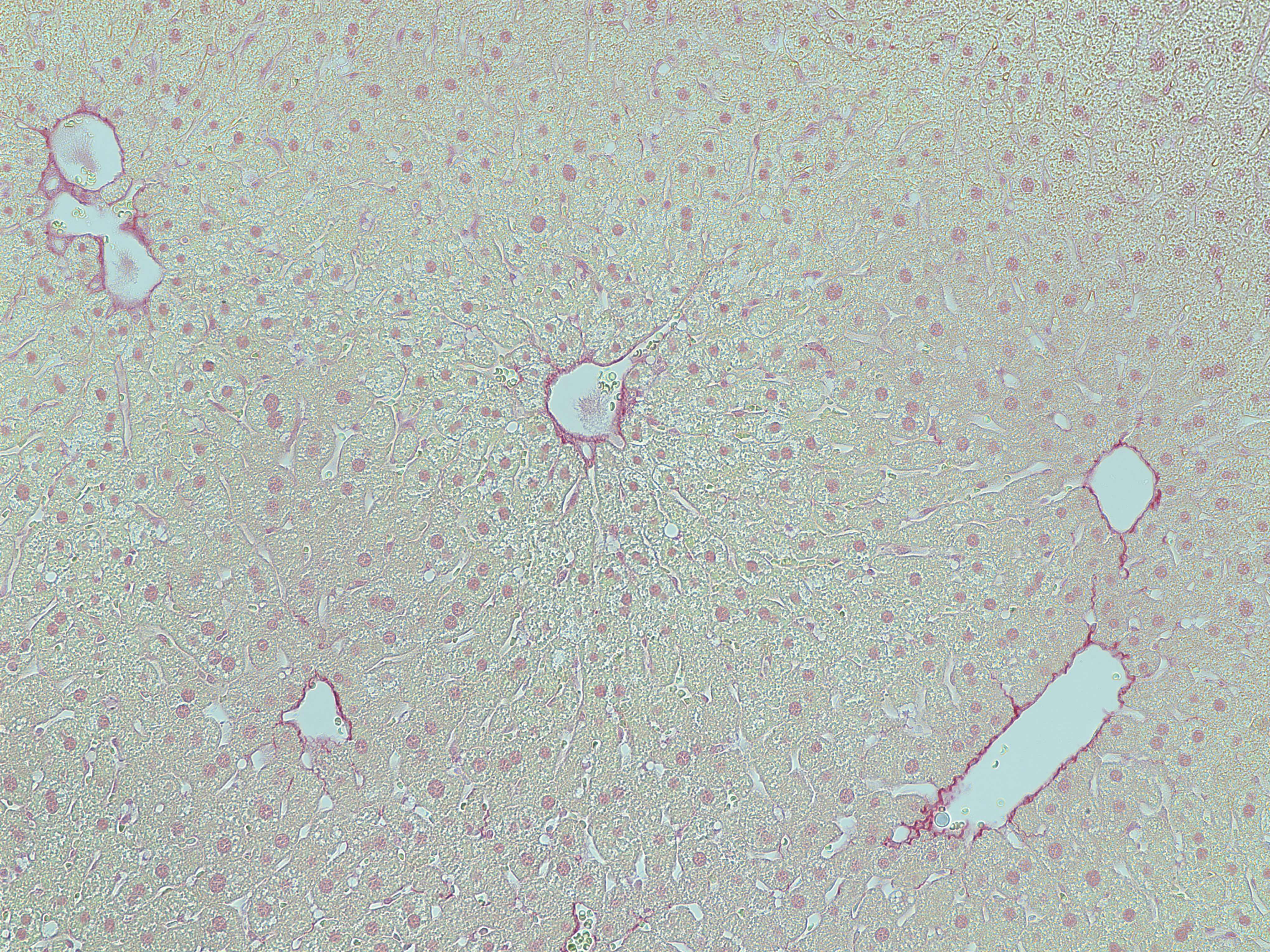

Supplement: Supplementary file 9 — Figure EV3 Source Data [file 44318_2024_196_MOESM9_ESM.zip › Figure EV3/Figure EV3-F/Quantificated image/NC Pcolce KO/no.4/NC Pcolce KO no.4-20x-2.jpg]

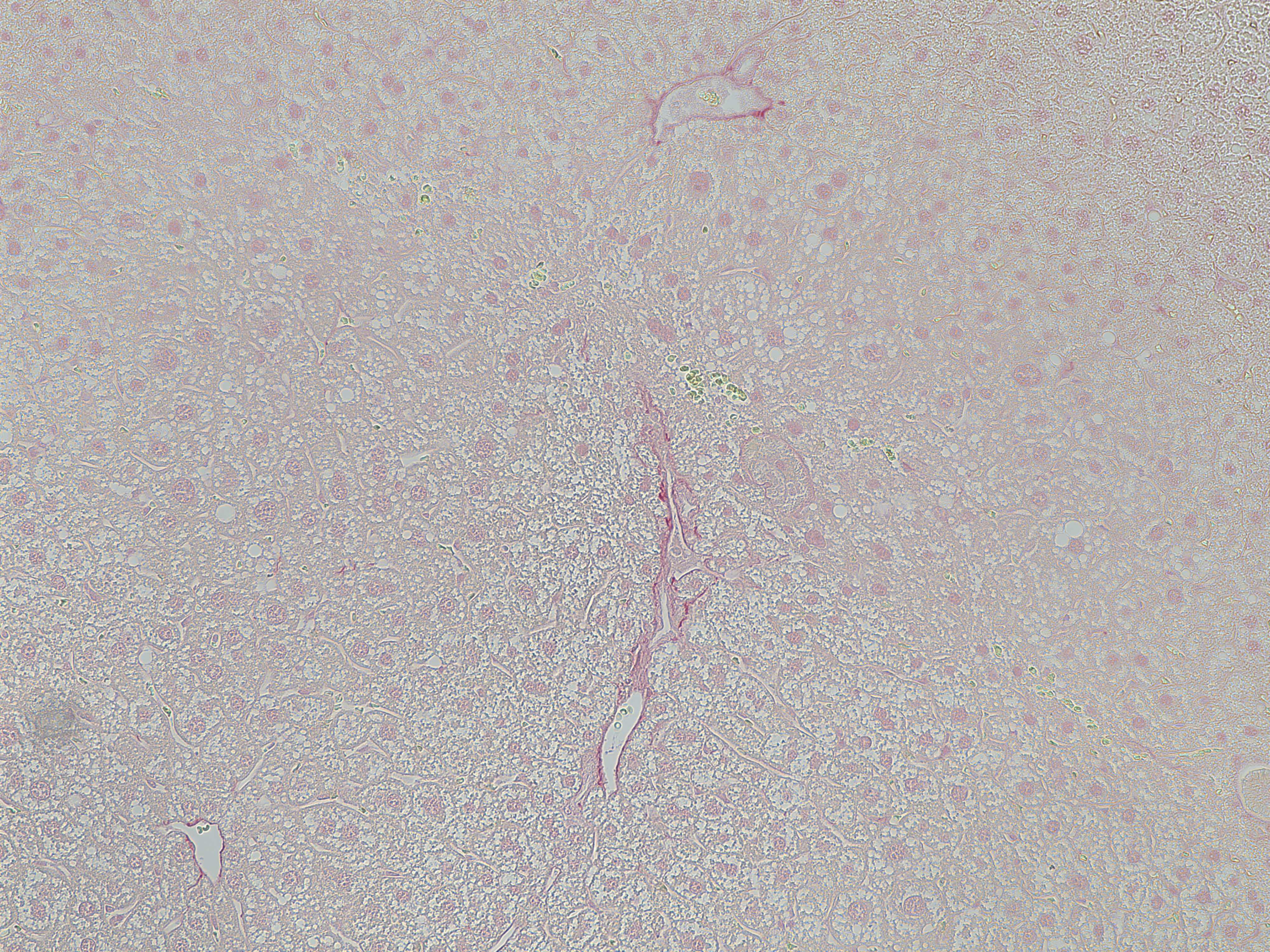

Supplement: Supplementary file 9 — Figure EV3 Source Data [file 44318_2024_196_MOESM9_ESM.zip › Figure EV3/Figure EV3-F/Quantificated image/NC Pcolce KO/no.5/NC Pcolce KO no.5-20x-5.jpg]

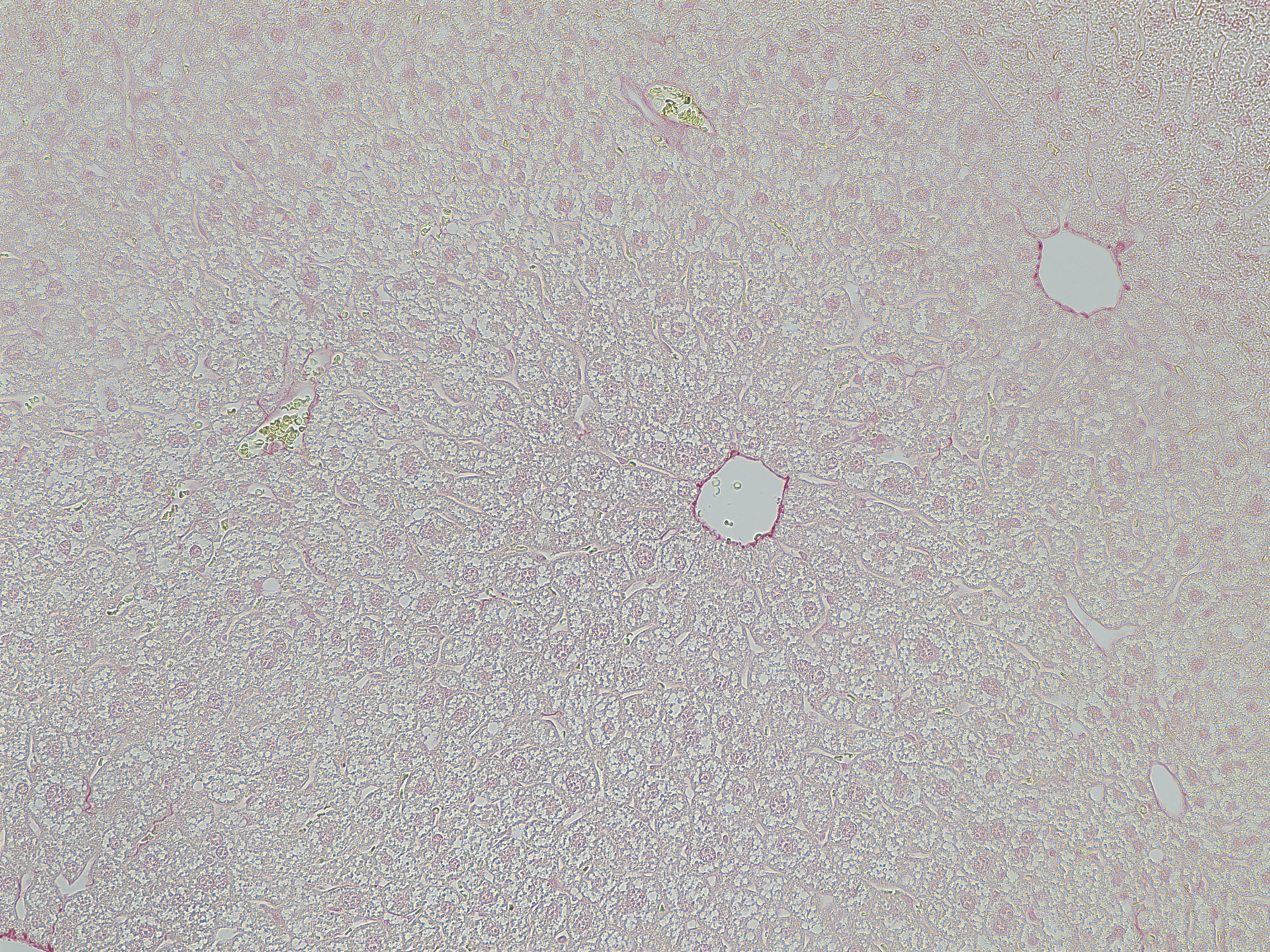

Supplement: Supplementary file 9 — Figure EV3 Source Data [file 44318_2024_196_MOESM9_ESM.zip › Figure EV3/Figure EV3-F/Quantificated image/NC Pcolce KO/no.5/NC Pcolce KO no.5-20x-4.jpg]

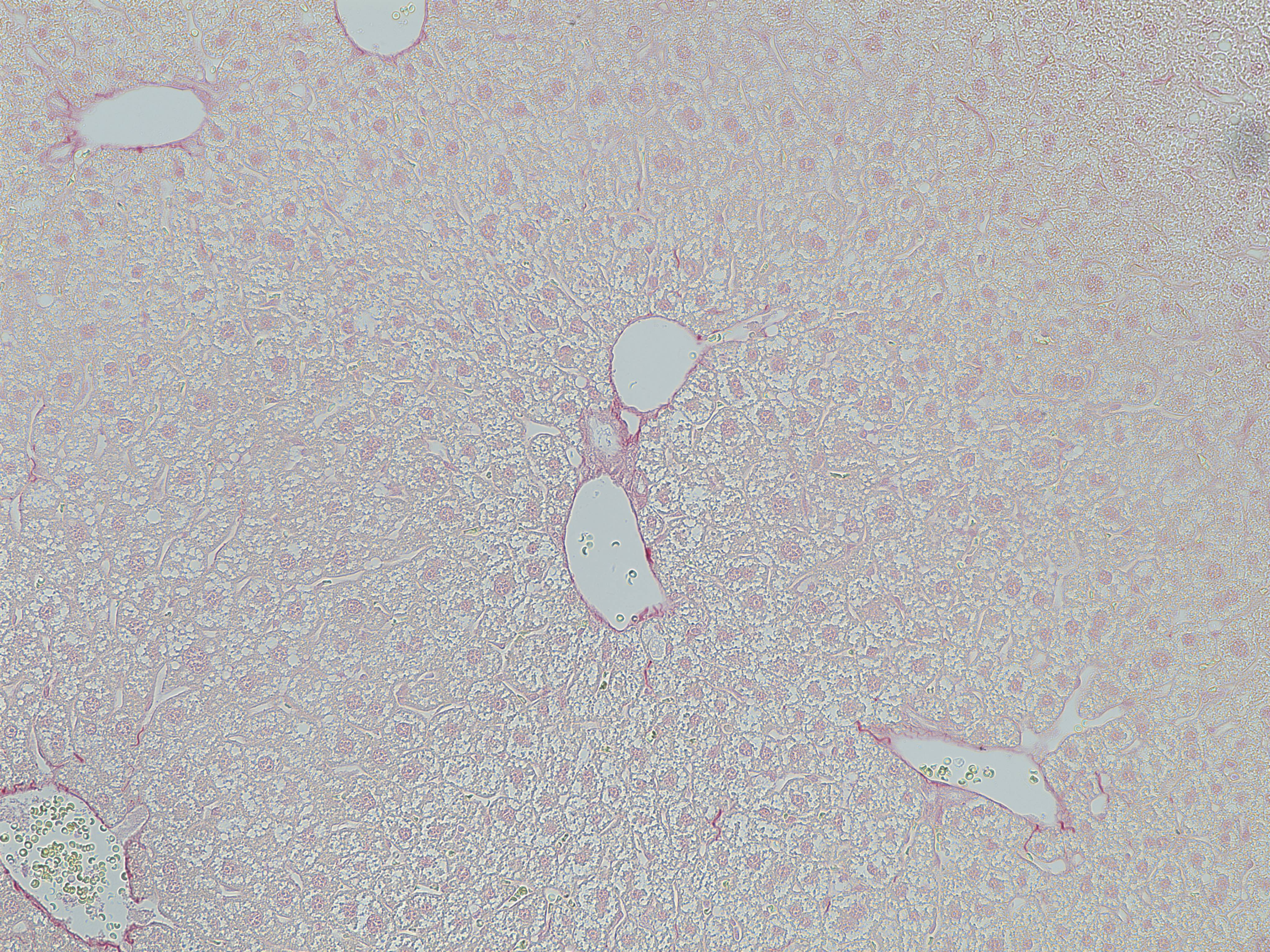

Supplement: Supplementary file 9 — Figure EV3 Source Data [file 44318_2024_196_MOESM9_ESM.zip › Figure EV3/Figure EV3-F/Quantificated image/NC Pcolce KO/no.5/NC Pcolce KO no.5-20x-1.jpg]

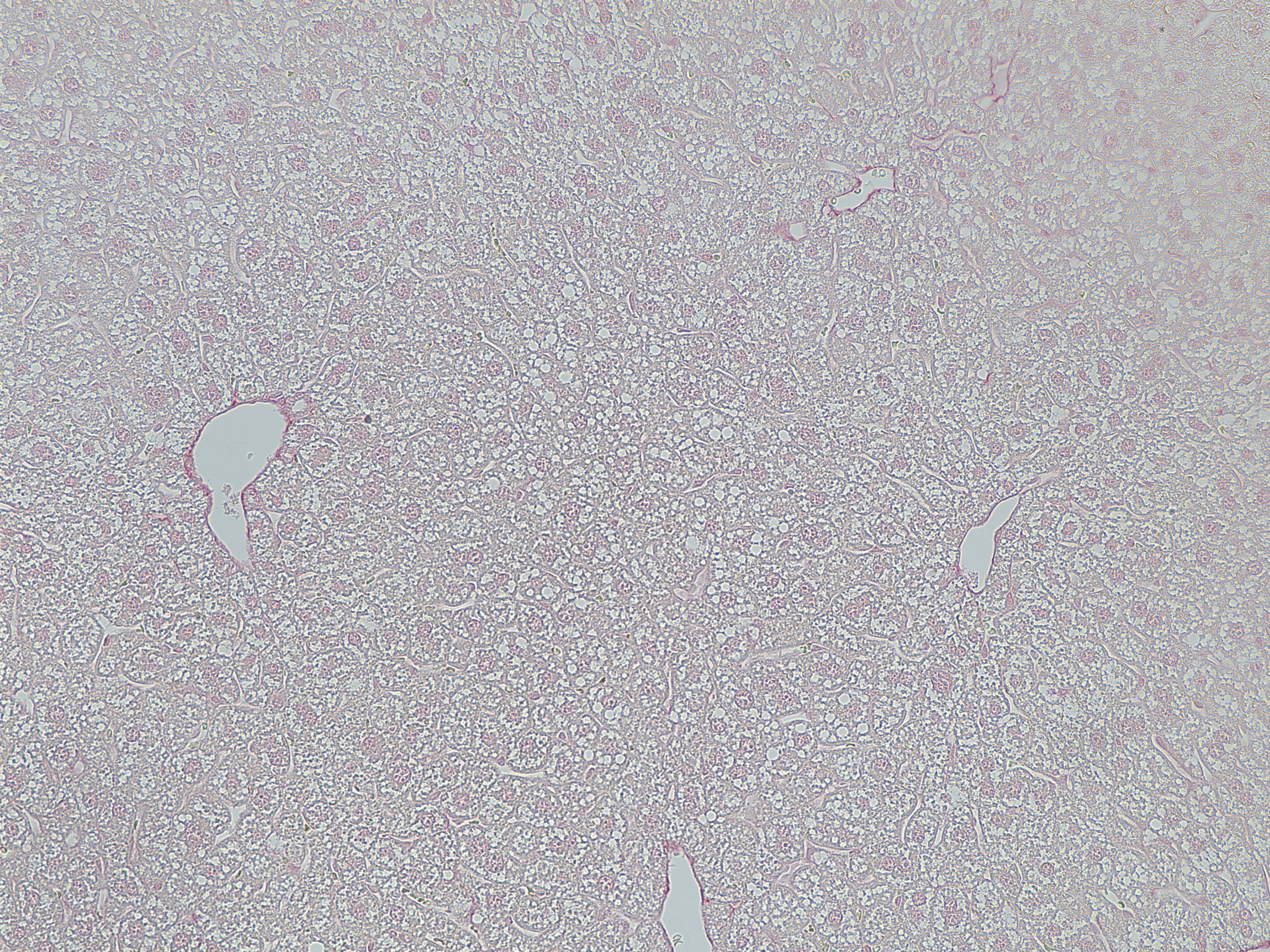

Supplement: Supplementary file 9 — Figure EV3 Source Data [file 44318_2024_196_MOESM9_ESM.zip › Figure EV3/Figure EV3-F/Quantificated image/NC Pcolce KO/no.5/NC Pcolce KO no.5-20x-3.jpg]

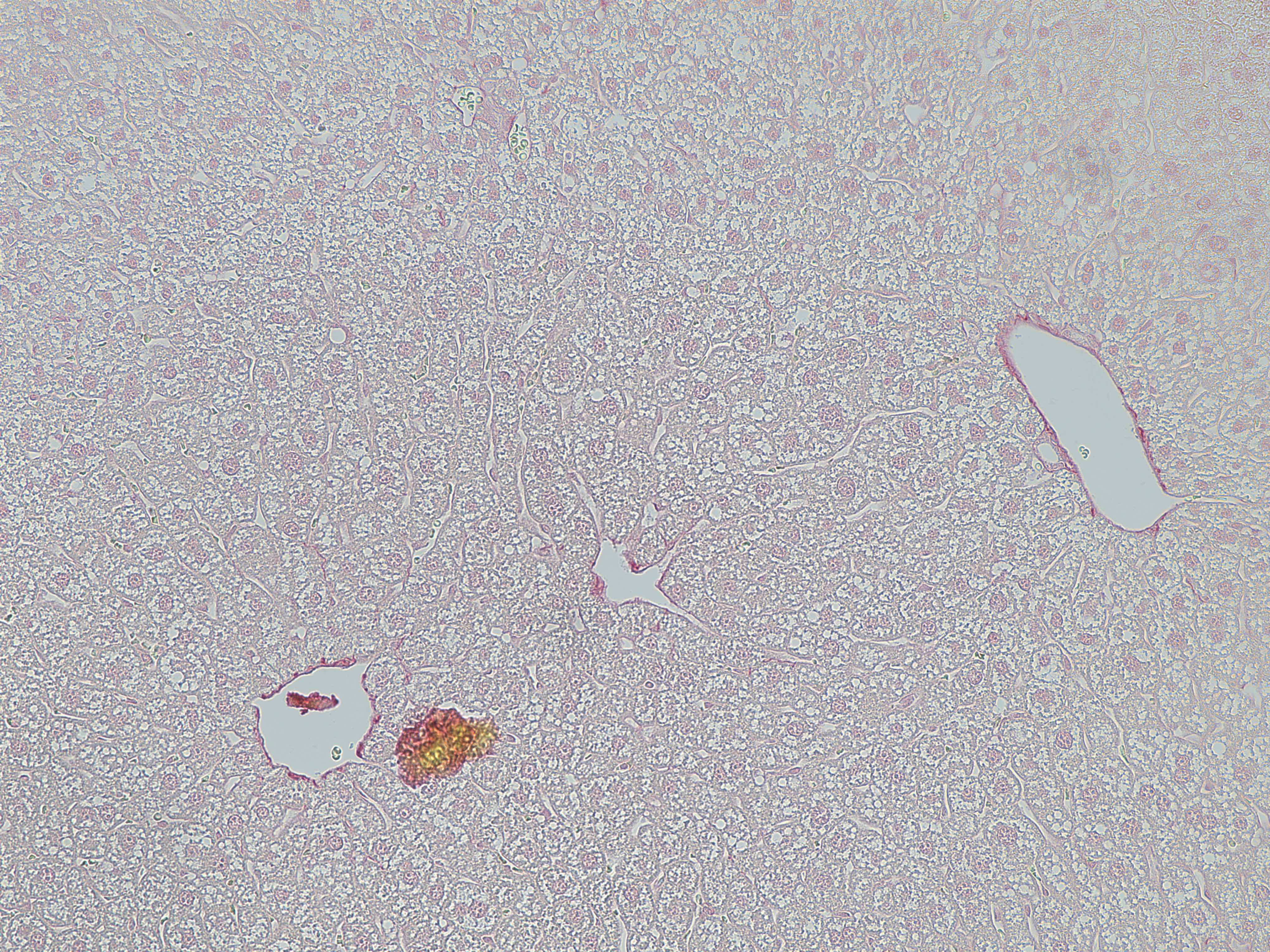

Supplement: Supplementary file 9 — Figure EV3 Source Data [file 44318_2024_196_MOESM9_ESM.zip › Figure EV3/Figure EV3-F/Quantificated image/NC Pcolce KO/no.5/NC Pcolce KO no.5-20x-2.jpg]

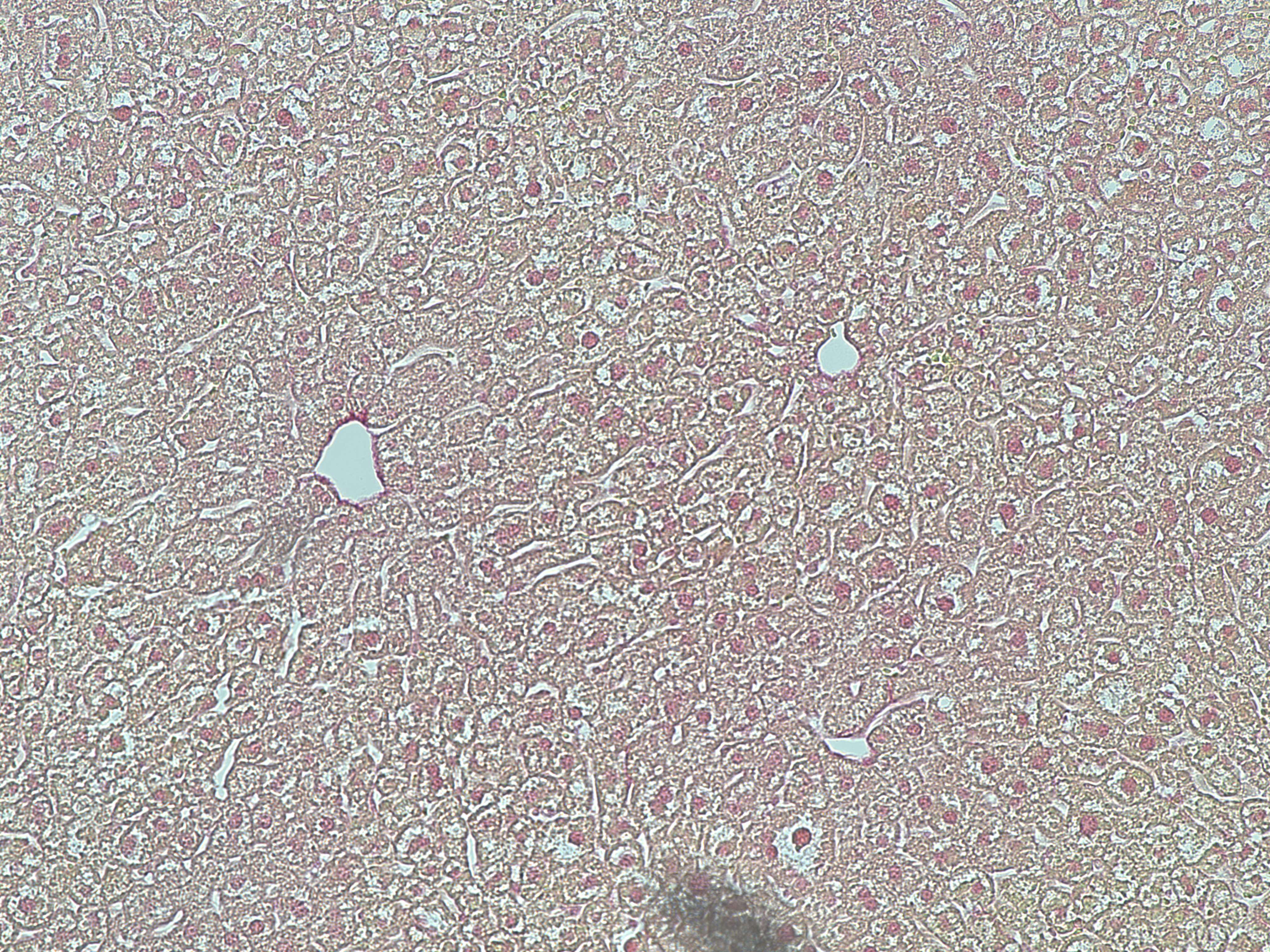

Supplement: Supplementary file 9 — Figure EV3 Source Data [file 44318_2024_196_MOESM9_ESM.zip › Figure EV3/Figure EV3-F/Quantificated image/NC Pcolce KO/no.2/NC Pcolce KO no.2-20x-1.jpg]

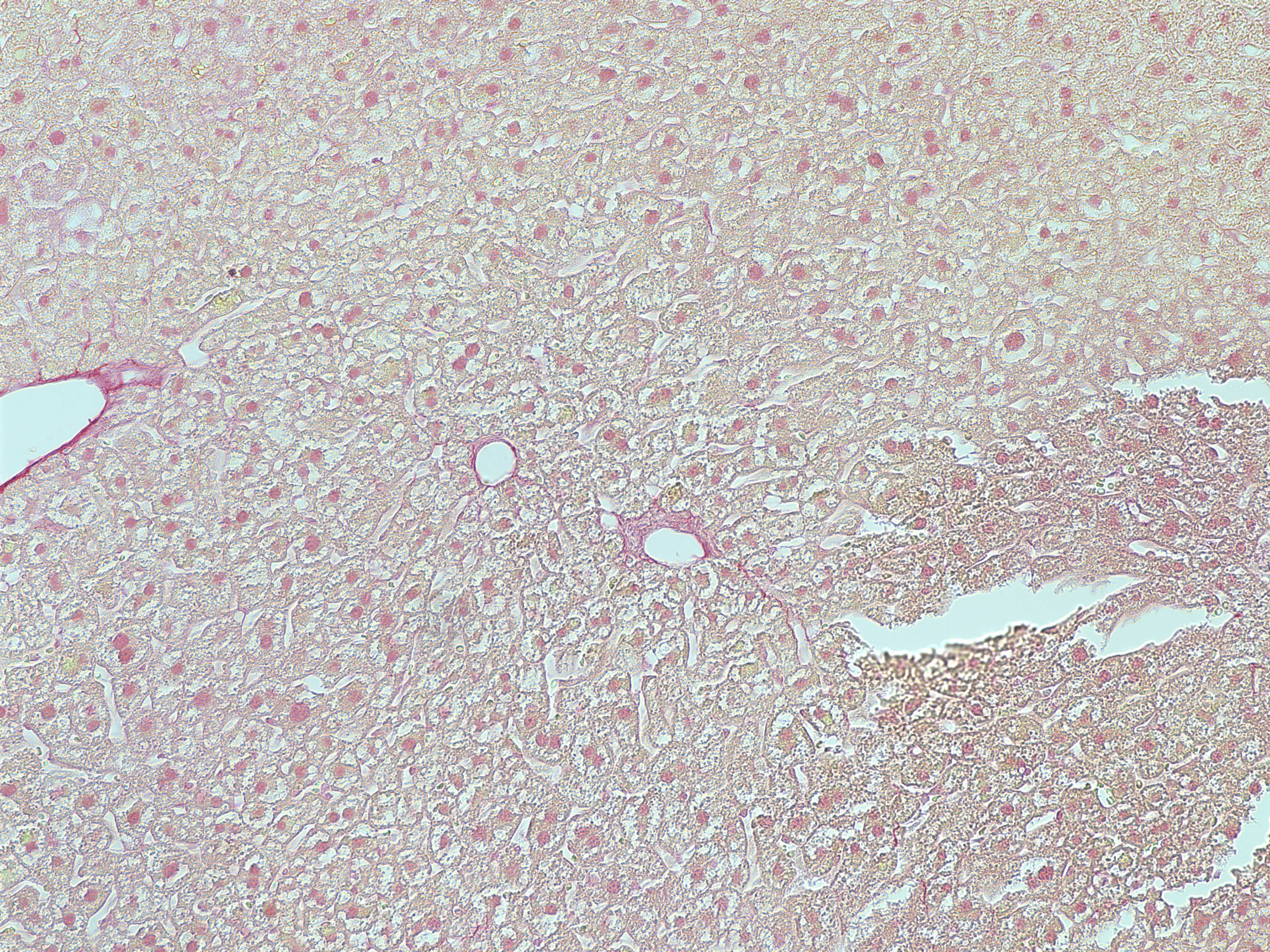

Supplement: Supplementary file 9 — Figure EV3 Source Data [file 44318_2024_196_MOESM9_ESM.zip › Figure EV3/Figure EV3-F/Quantificated image/NC Pcolce KO/no.2/NC Pcolce KO no.2-20x-2.jpg]

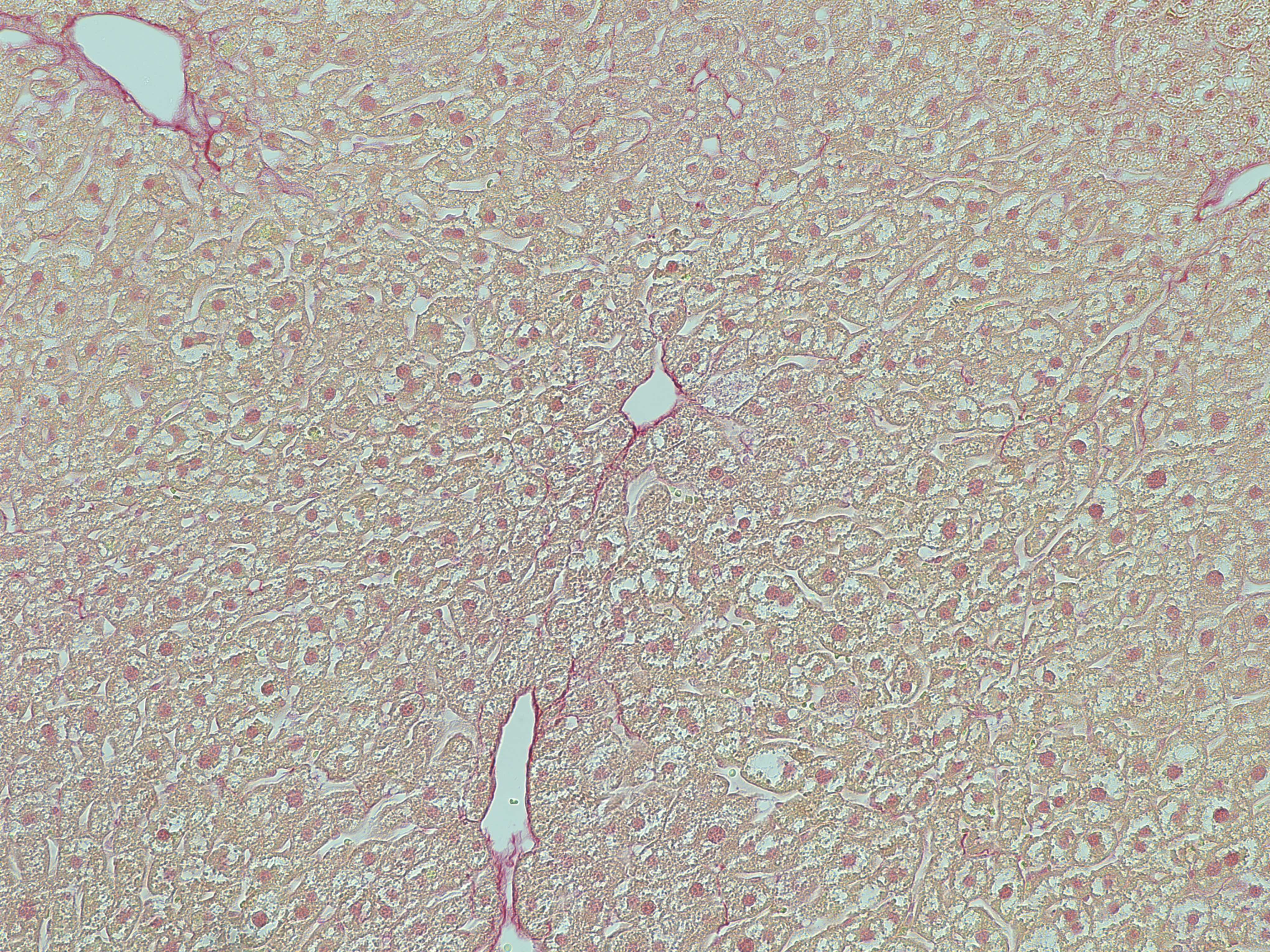

Supplement: Supplementary file 9 — Figure EV3 Source Data [file 44318_2024_196_MOESM9_ESM.zip › Figure EV3/Figure EV3-F/Quantificated image/NC Pcolce KO/no.2/NC Pcolce KO no.2-20x-3.jpg]

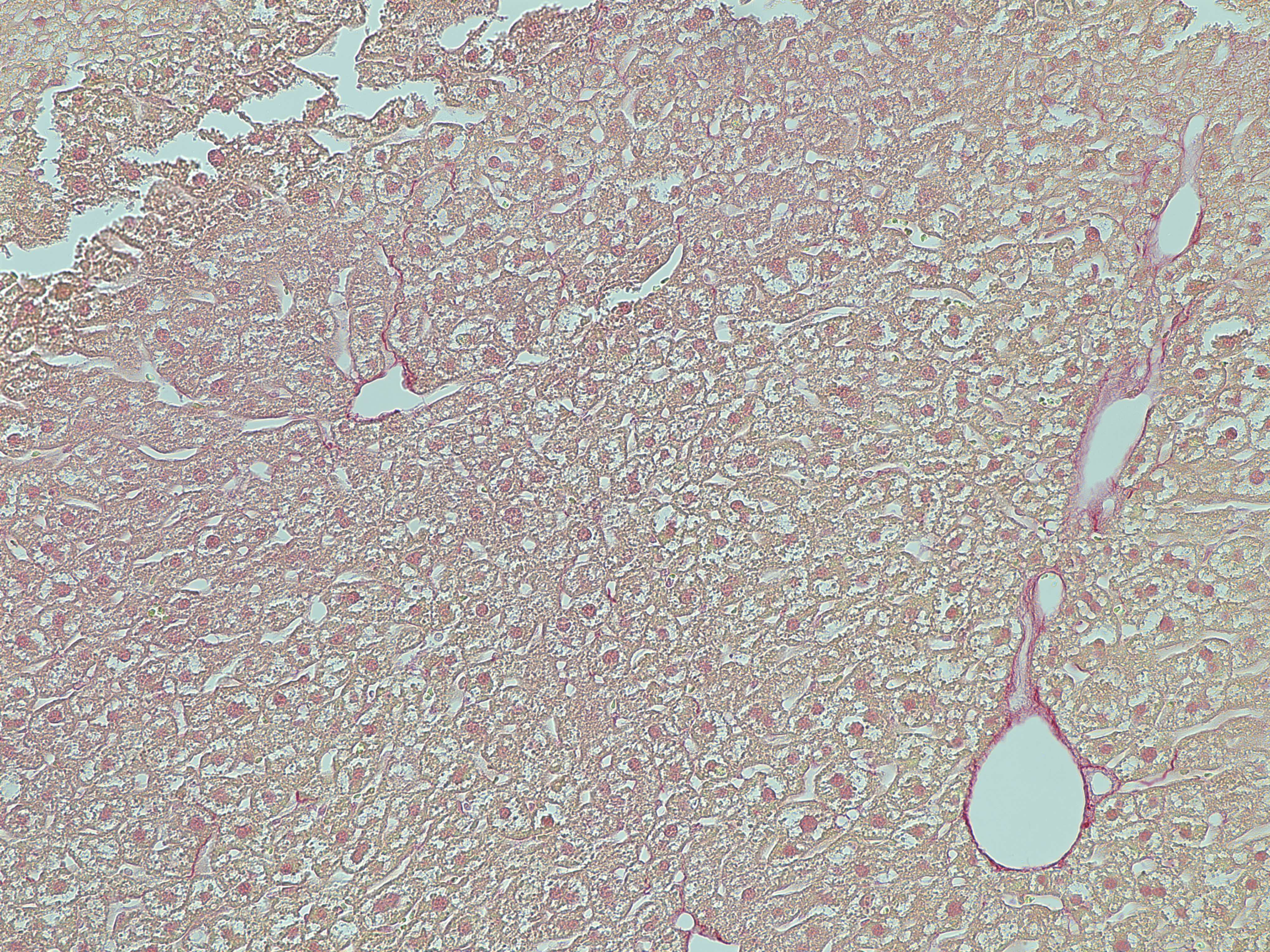

Supplement: Supplementary file 9 — Figure EV3 Source Data [file 44318_2024_196_MOESM9_ESM.zip › Figure EV3/Figure EV3-F/Quantificated image/NC Pcolce KO/no.2/NC Pcolce KO no.2-20x-4.jpg]

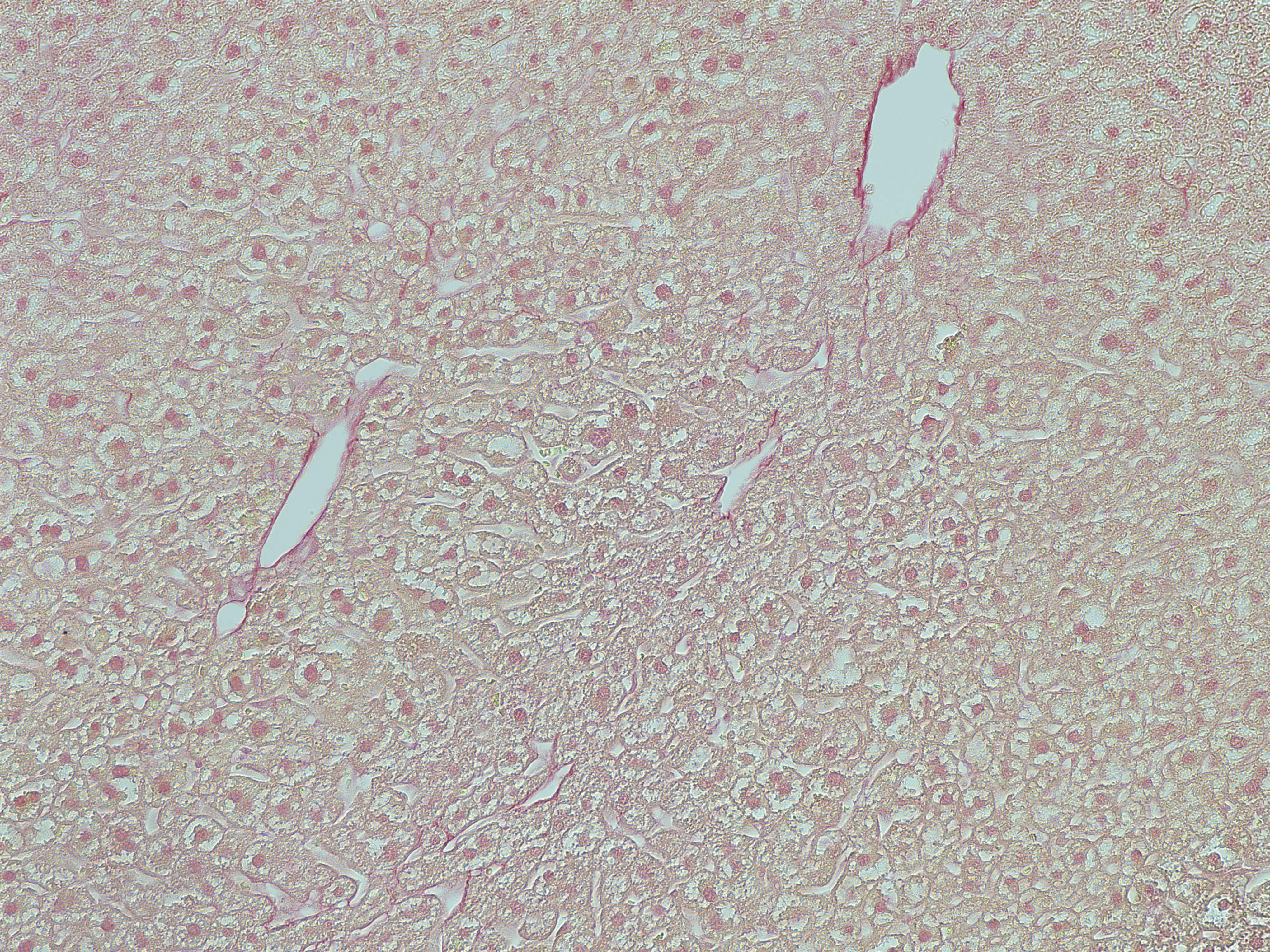

Supplement: Supplementary file 9 — Figure EV3 Source Data [file 44318_2024_196_MOESM9_ESM.zip › Figure EV3/Figure EV3-F/Quantificated image/NC Pcolce KO/no.2/NC Pcolce KO no.2-20x-5.jpg]

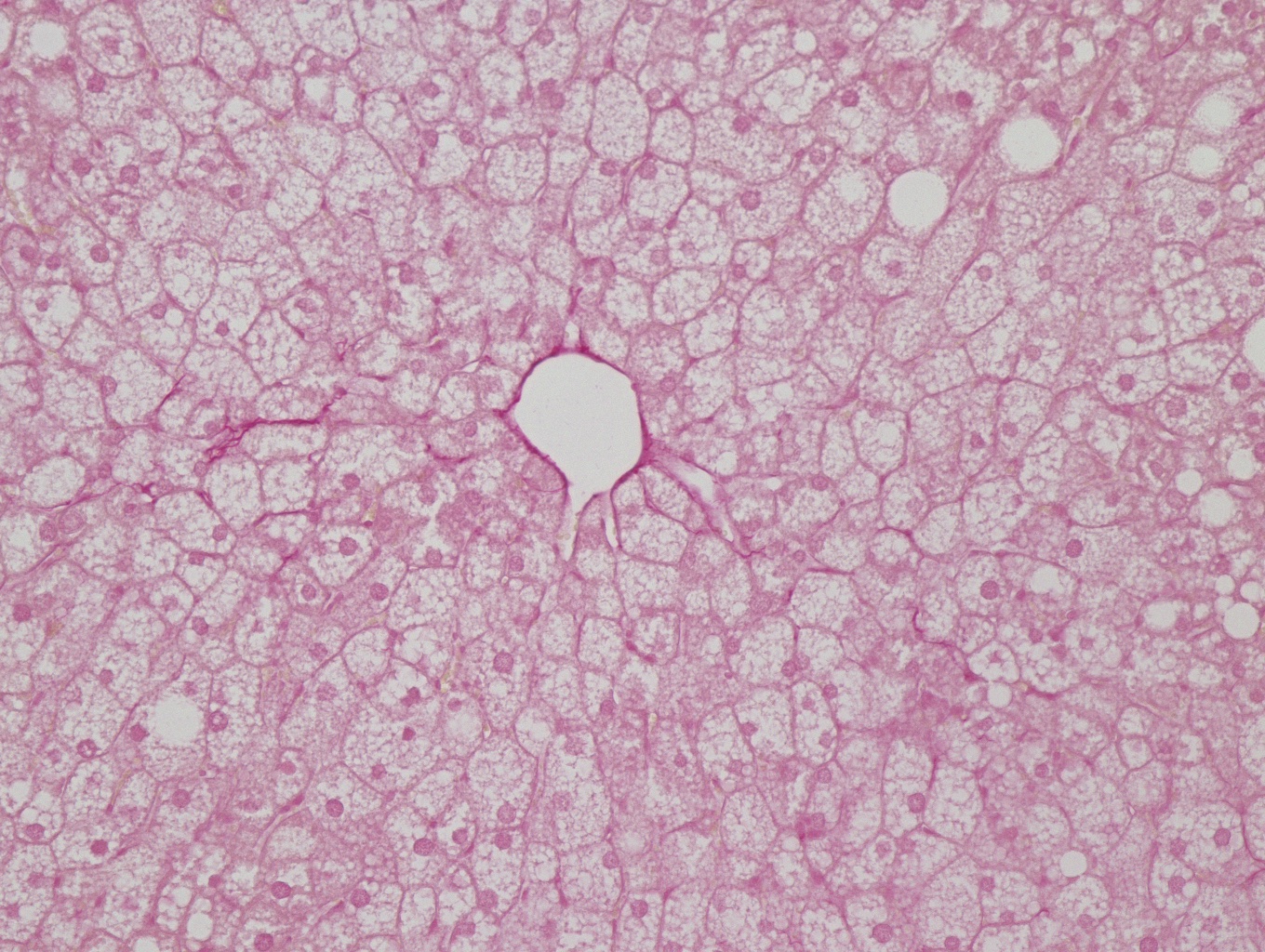

Supplement: Supplementary file 9 — Figure EV3 Source Data [file 44318_2024_196_MOESM9_ESM.zip › Figure EV3/Figure EV3-F/Quantificated image/HFD Pcolce KO/no.7/HFD Pcolce KO no.7 x20-4.jpg]

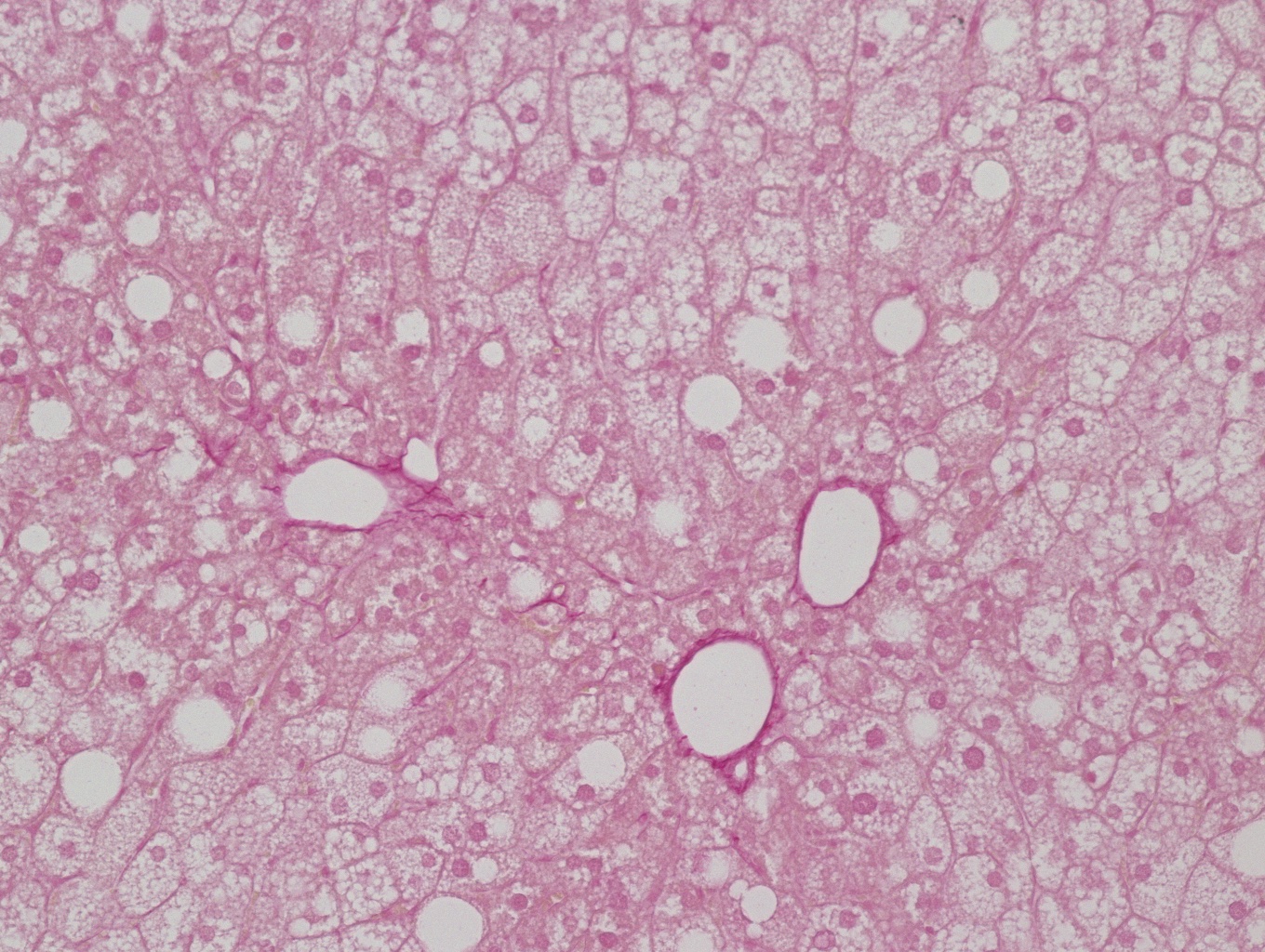

Supplement: Supplementary file 9 — Figure EV3 Source Data [file 44318_2024_196_MOESM9_ESM.zip › Figure EV3/Figure EV3-F/Quantificated image/HFD Pcolce KO/no.7/HFD Pcolce KO no.7 x20-3.jpg]

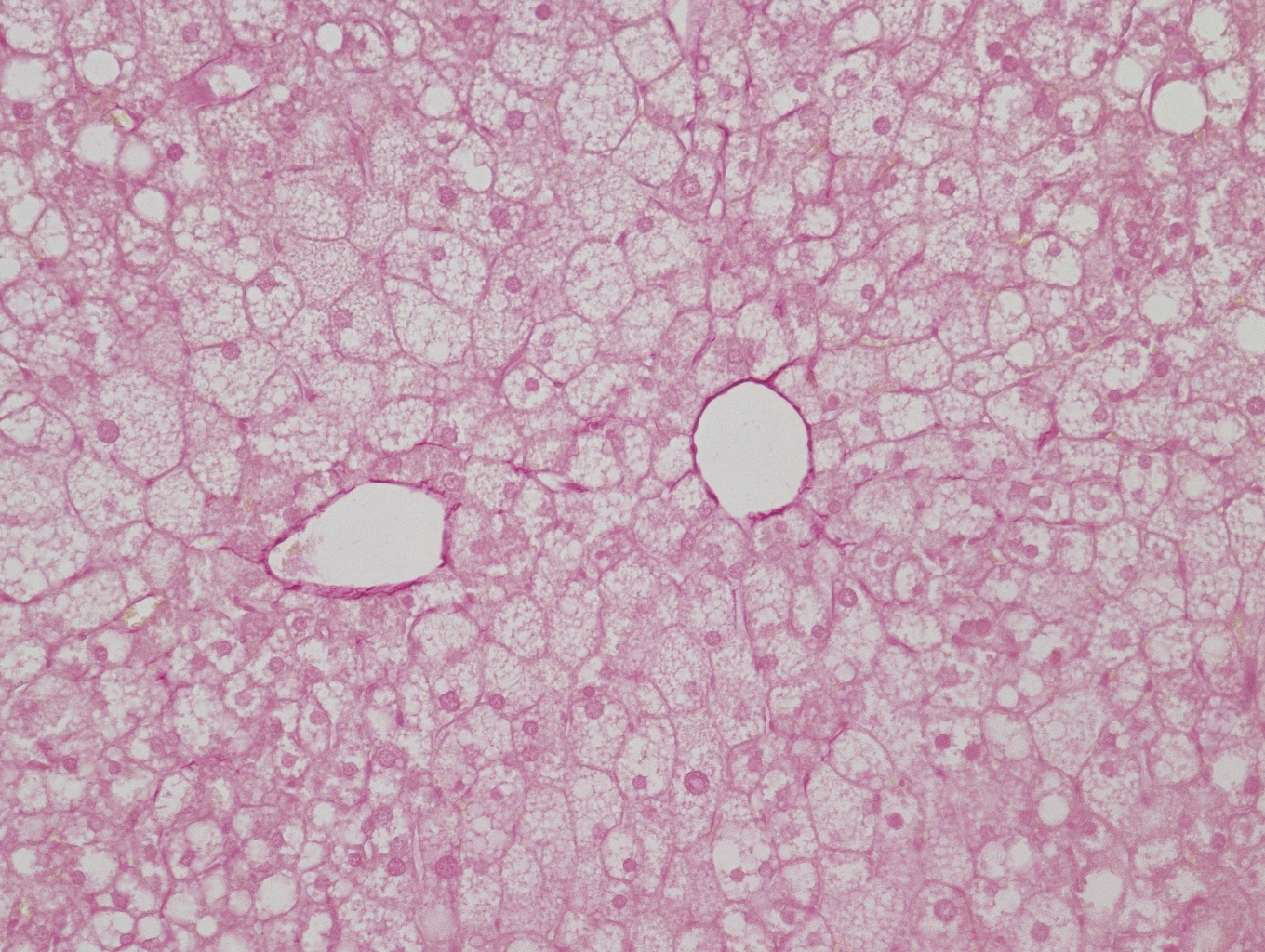

Supplement: Supplementary file 9 — Figure EV3 Source Data [file 44318_2024_196_MOESM9_ESM.zip › Figure EV3/Figure EV3-F/Quantificated image/HFD Pcolce KO/no.7/HFD Pcolce KO no.7 x20-2.jpg]

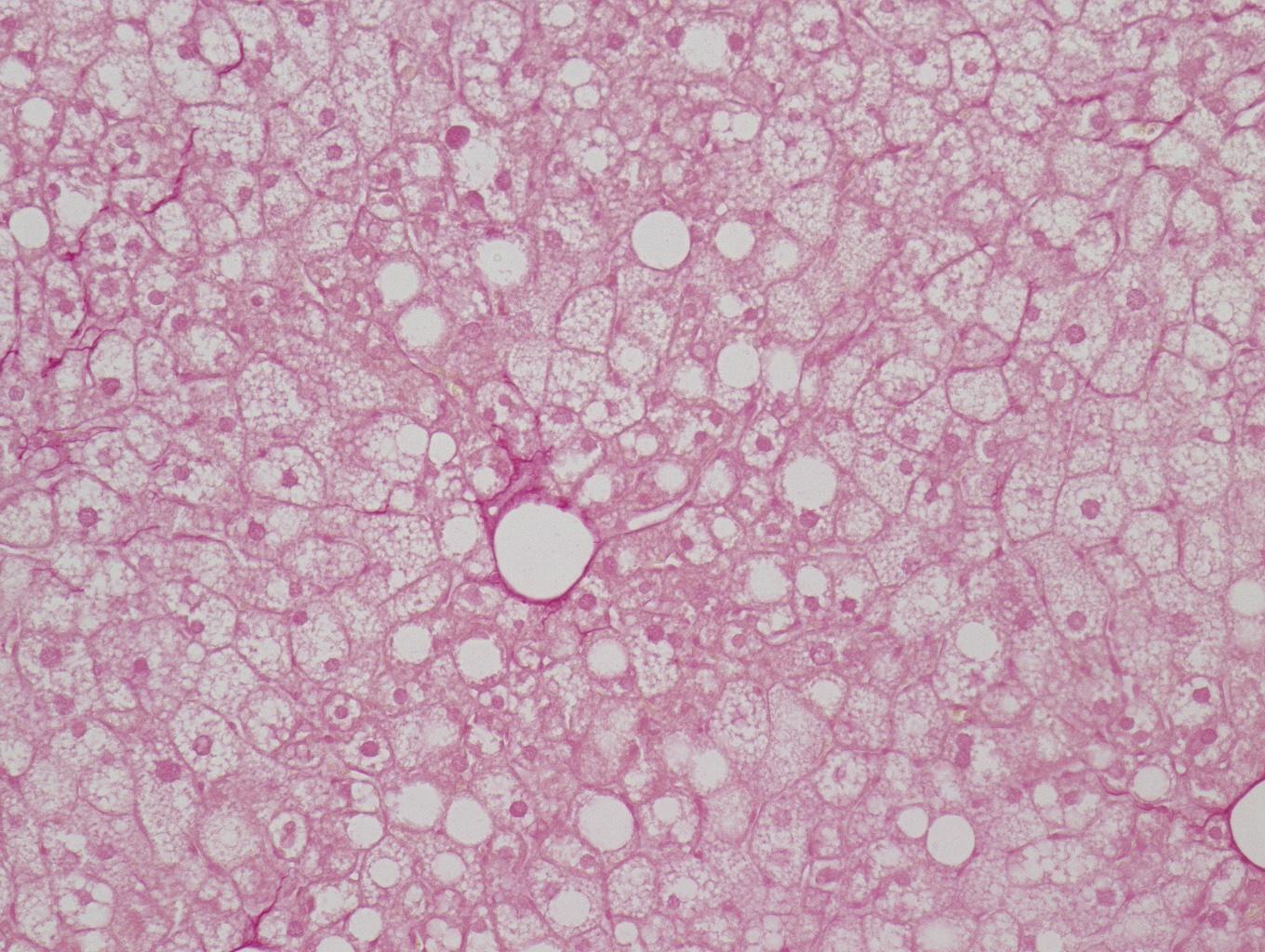

Supplement: Supplementary file 9 — Figure EV3 Source Data [file 44318_2024_196_MOESM9_ESM.zip › Figure EV3/Figure EV3-F/Quantificated image/HFD Pcolce KO/no.7/HFD Pcolce KO no.7 x20-1.jpg]

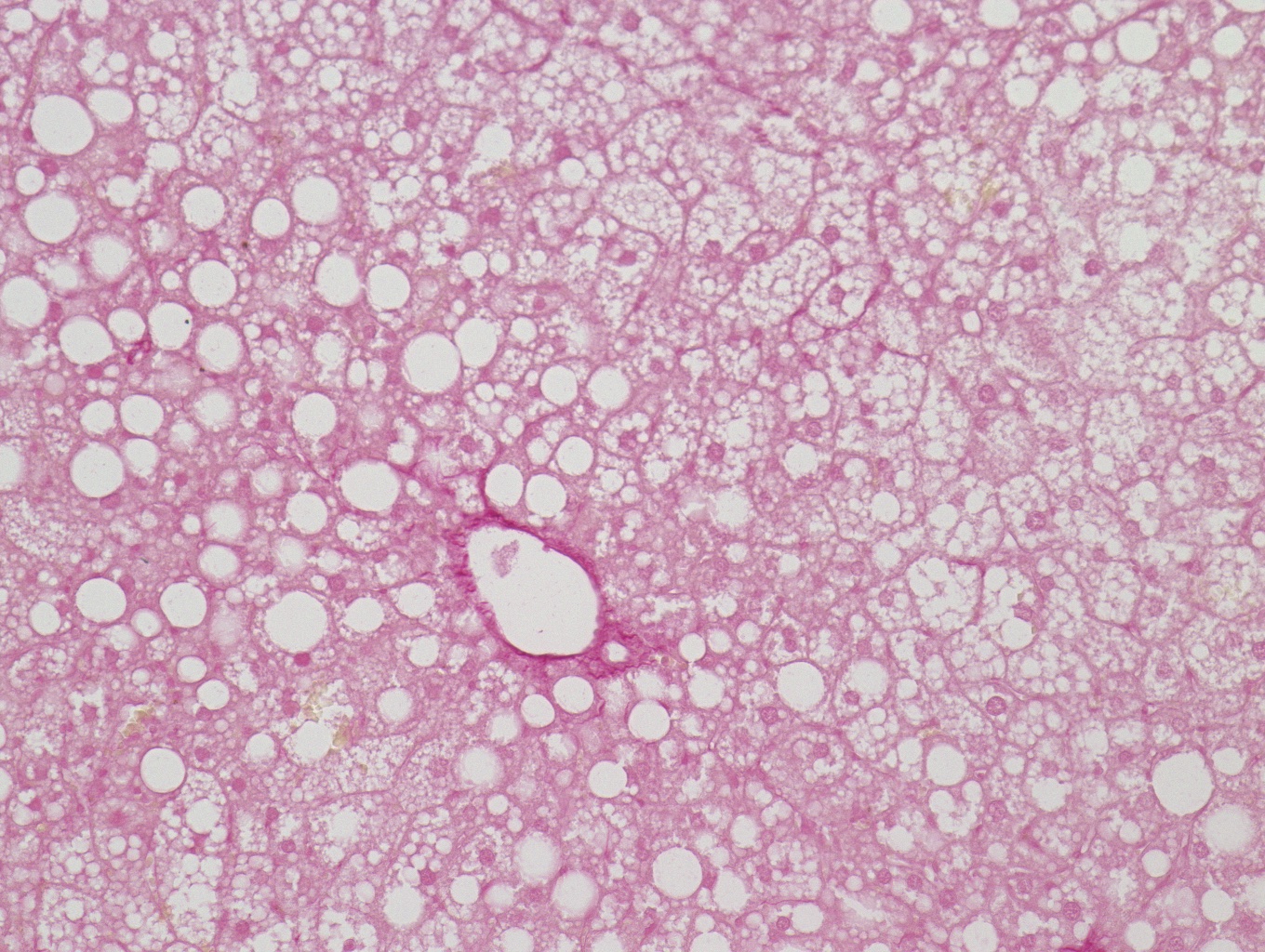

Supplement: Supplementary file 9 — Figure EV3 Source Data [file 44318_2024_196_MOESM9_ESM.zip › Figure EV3/Figure EV3-F/Quantificated image/HFD Pcolce KO/no.8/HFD Pcolce KO no.8 x20-4.jpg]

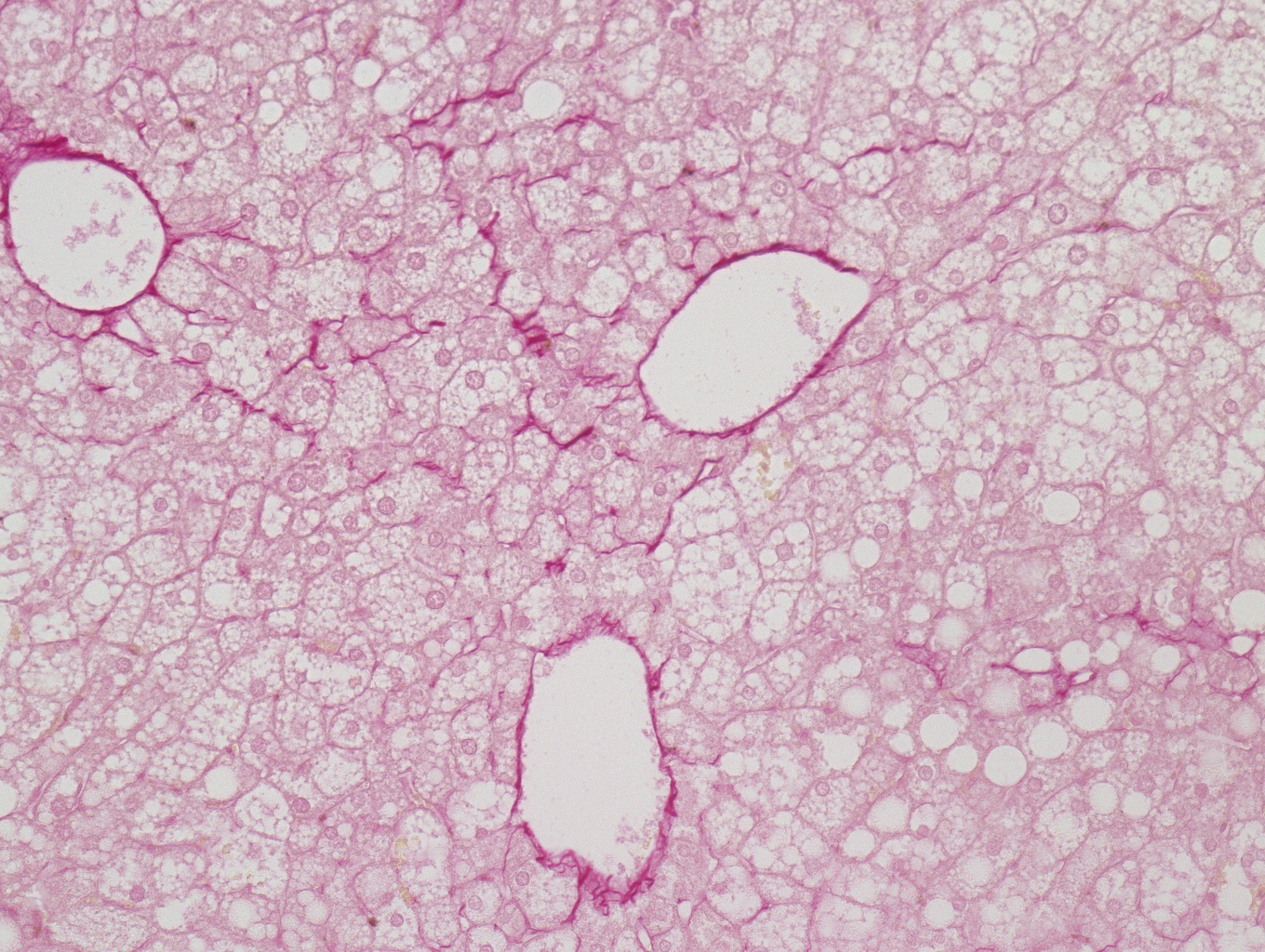

Supplement: Supplementary file 9 — Figure EV3 Source Data [file 44318_2024_196_MOESM9_ESM.zip › Figure EV3/Figure EV3-F/Quantificated image/HFD Pcolce KO/no.8/HFD Pcolce KO no.8 x20-1.jpg]

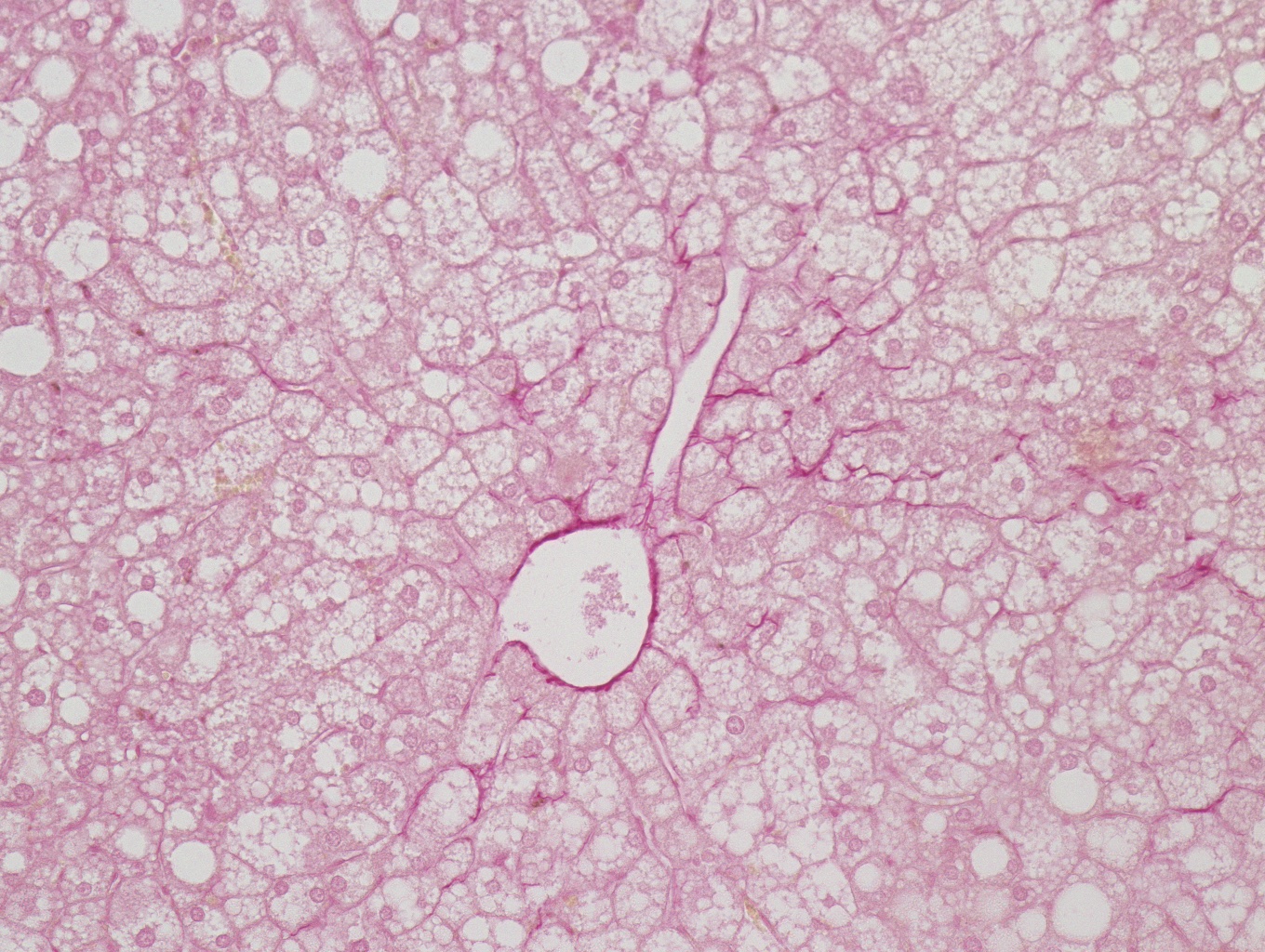

Supplement: Supplementary file 9 — Figure EV3 Source Data [file 44318_2024_196_MOESM9_ESM.zip › Figure EV3/Figure EV3-F/Quantificated image/HFD Pcolce KO/no.8/HFD Pcolce KO no.8 x20-2.jpg]

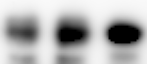

Supplement: Supplementary file 10 — Figure EV4 Source Data [file 44318_2024_196_MOESM10_ESM.zip › Figure EV4/EV4D/Demonstrated data/c-Fos.tif]

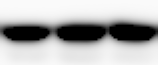

Supplement: Supplementary file 10 — Figure EV4 Source Data [file 44318_2024_196_MOESM10_ESM.zip › Figure EV4/EV4D/Demonstrated data/Actin.tif]

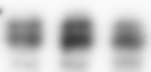

Supplement: Supplementary file 10 — Figure EV4 Source Data [file 44318_2024_196_MOESM10_ESM.zip › Figure EV4/EV4D/Demonstrated data/p-c-Fos.tif]

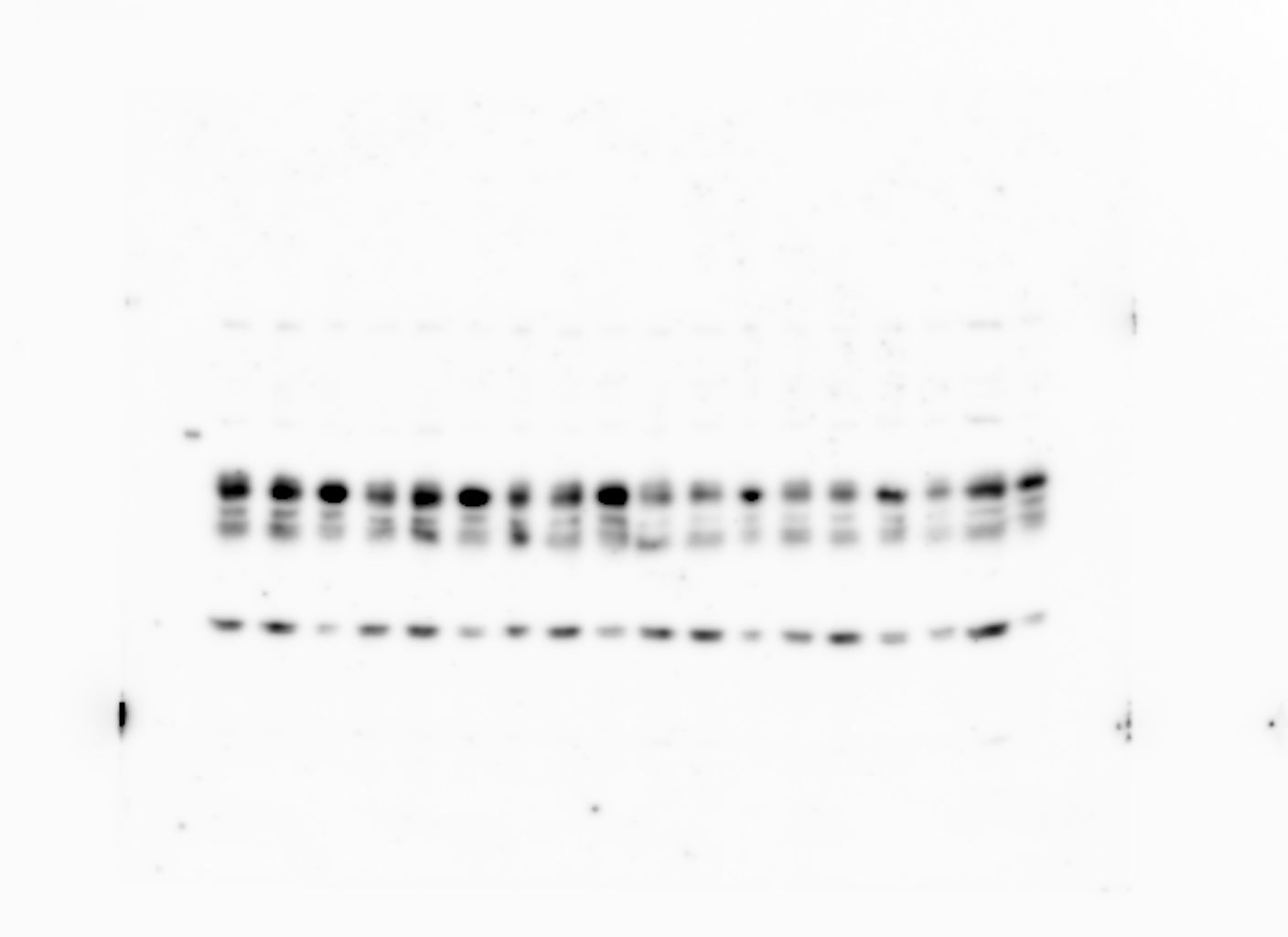

Supplement: Supplementary file 10 — Figure EV4 Source Data [file 44318_2024_196_MOESM10_ESM.zip › Figure EV4/EV4D/Quantification data/c-Fos.tif]

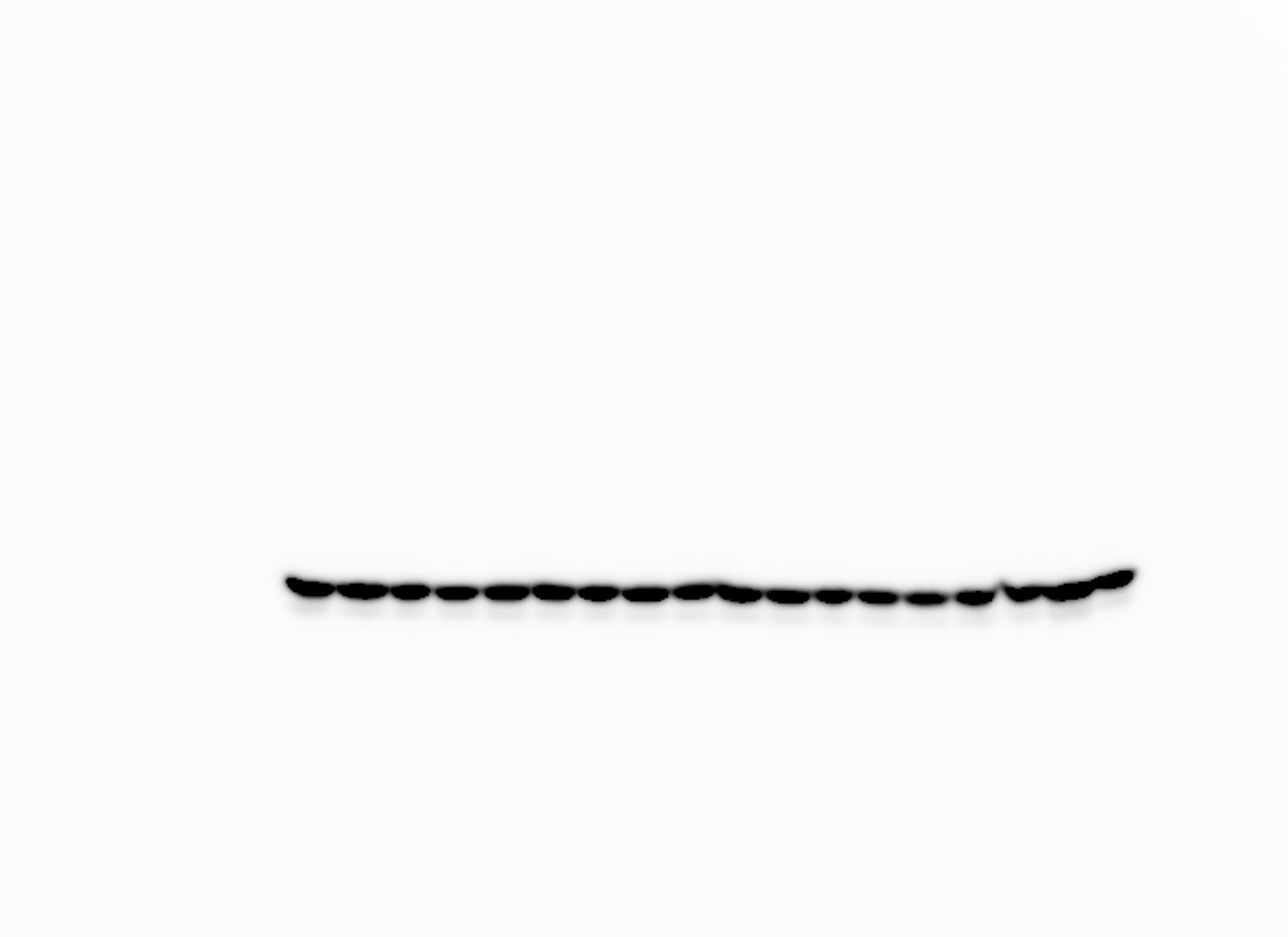

Supplement: Supplementary file 10 — Figure EV4 Source Data [file 44318_2024_196_MOESM10_ESM.zip › Figure EV4/EV4D/Quantification data/Actin.tif]

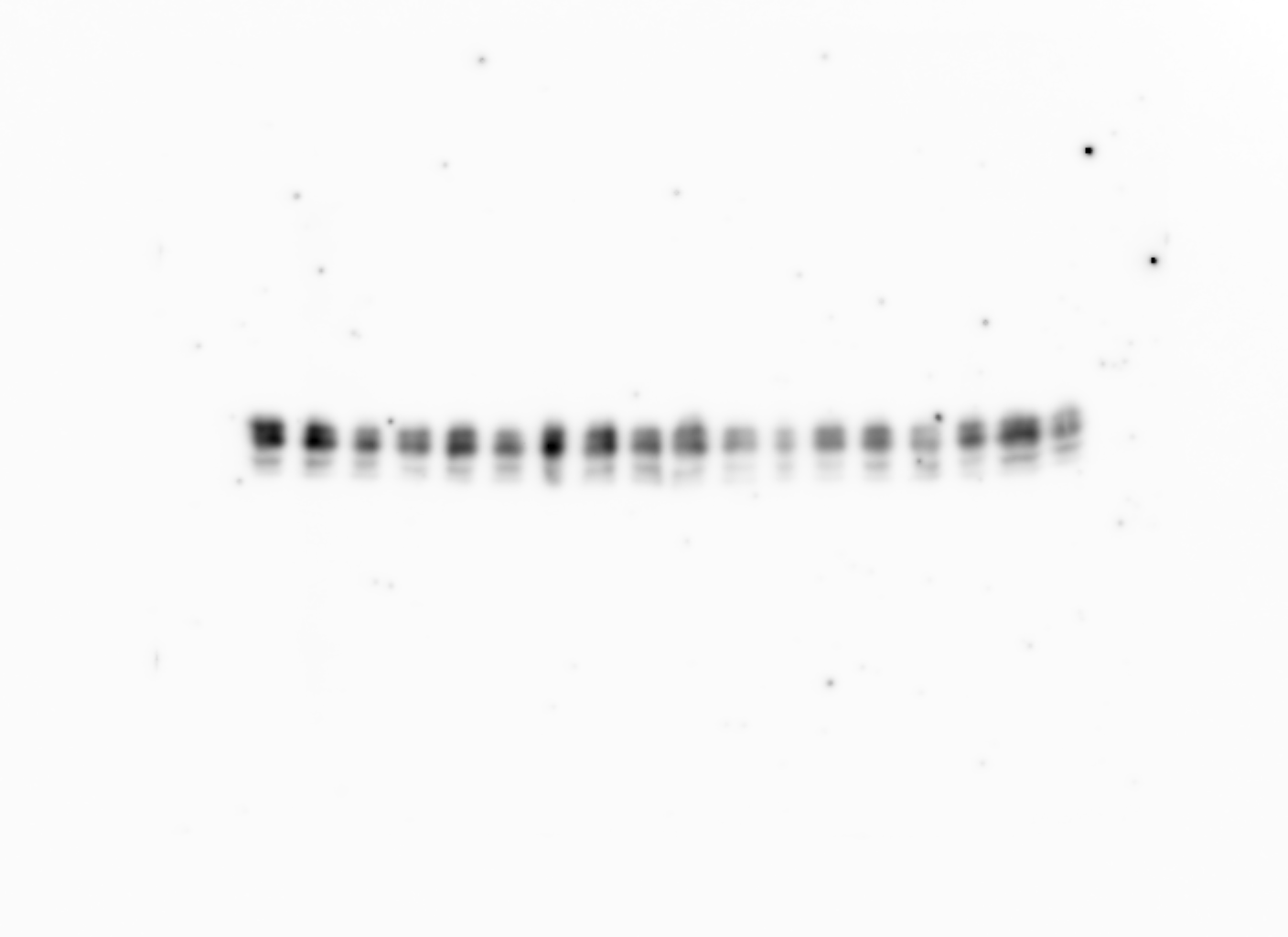

Supplement: Supplementary file 10 — Figure EV4 Source Data [file 44318_2024_196_MOESM10_ESM.zip › Figure EV4/EV4D/Quantification data/p-c-Fos.tif]

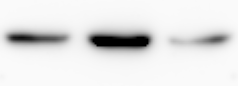

Supplement: Supplementary file 10 — Figure EV4 Source Data [file 44318_2024_196_MOESM10_ESM.zip › Figure EV4/EV4C/Demonstrated data/p-c-Jun.tif]

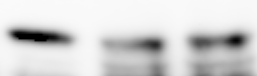

Supplement: Supplementary file 10 — Figure EV4 Source Data [file 44318_2024_196_MOESM10_ESM.zip › Figure EV4/EV4C/Demonstrated data/c-Fos.tif]

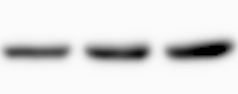

Supplement: Supplementary file 10 — Figure EV4 Source Data [file 44318_2024_196_MOESM10_ESM.zip › Figure EV4/EV4C/Demonstrated data/c-Jun.tif]

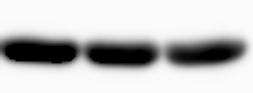

Supplement: Supplementary file 10 — Figure EV4 Source Data [file 44318_2024_196_MOESM10_ESM.zip › Figure EV4/EV4C/Demonstrated data/GAPDH.tif]

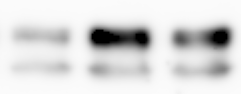

Supplement: Supplementary file 10 — Figure EV4 Source Data [file 44318_2024_196_MOESM10_ESM.zip › Figure EV4/EV4C/Demonstrated data/p-c-Fos.tif]

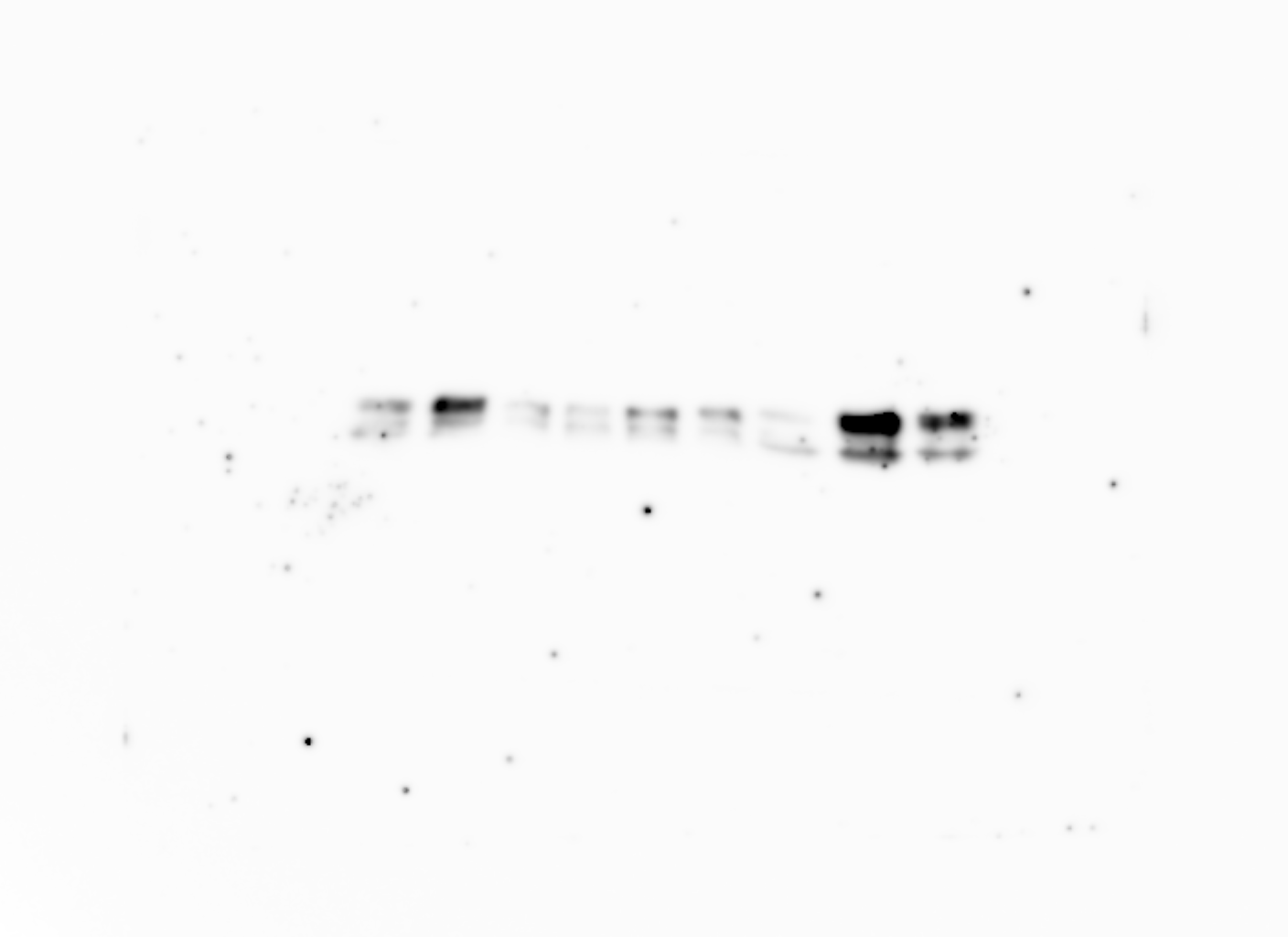

Supplement: Supplementary file 10 — Figure EV4 Source Data [file 44318_2024_196_MOESM10_ESM.zip › Figure EV4/EV4C/Quantification data/p-c-Fos membrane 2.tif]

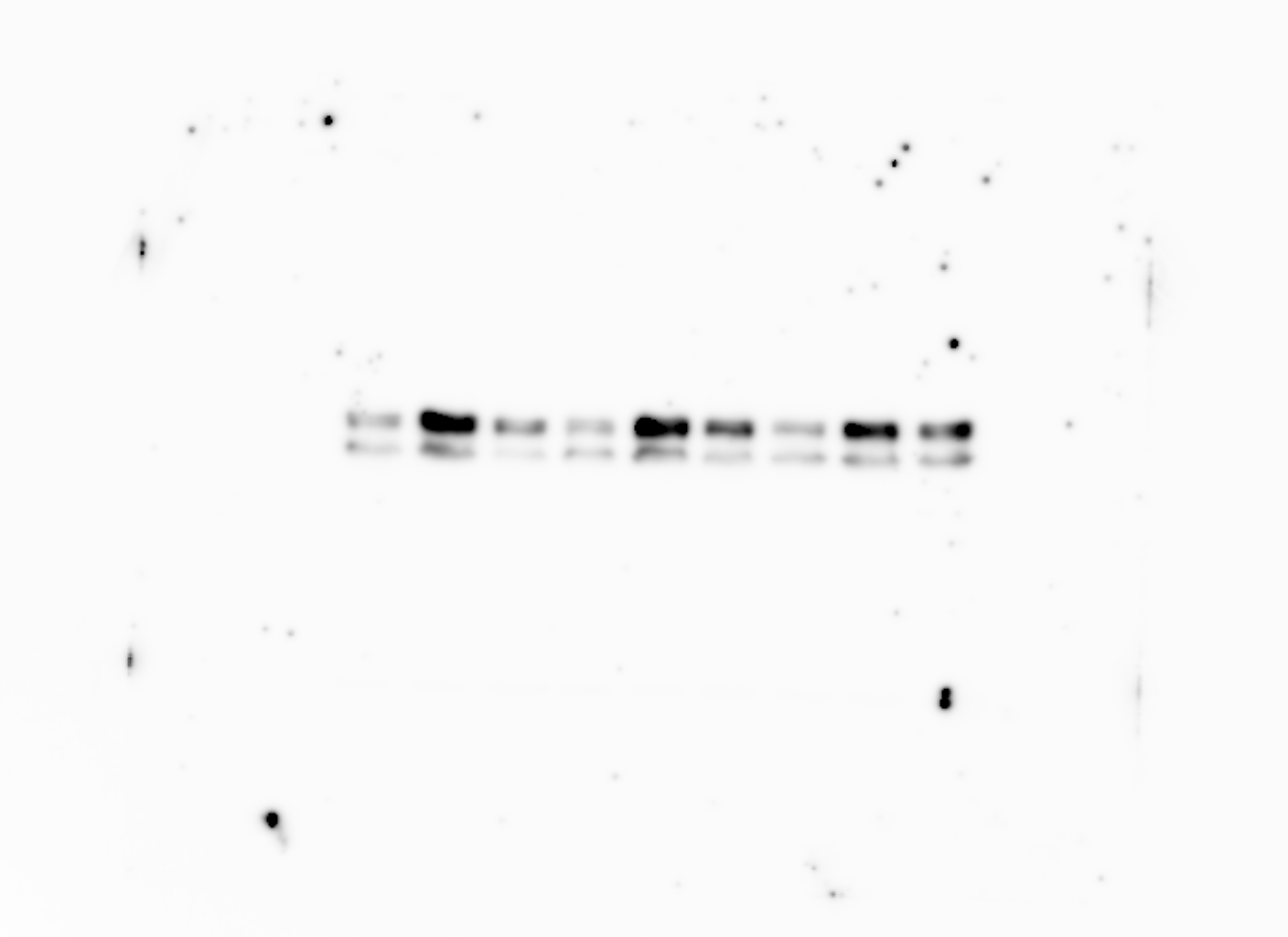

Supplement: Supplementary file 10 — Figure EV4 Source Data [file 44318_2024_196_MOESM10_ESM.zip › Figure EV4/EV4C/Quantification data/p-c-Fos membrane 1.tif]

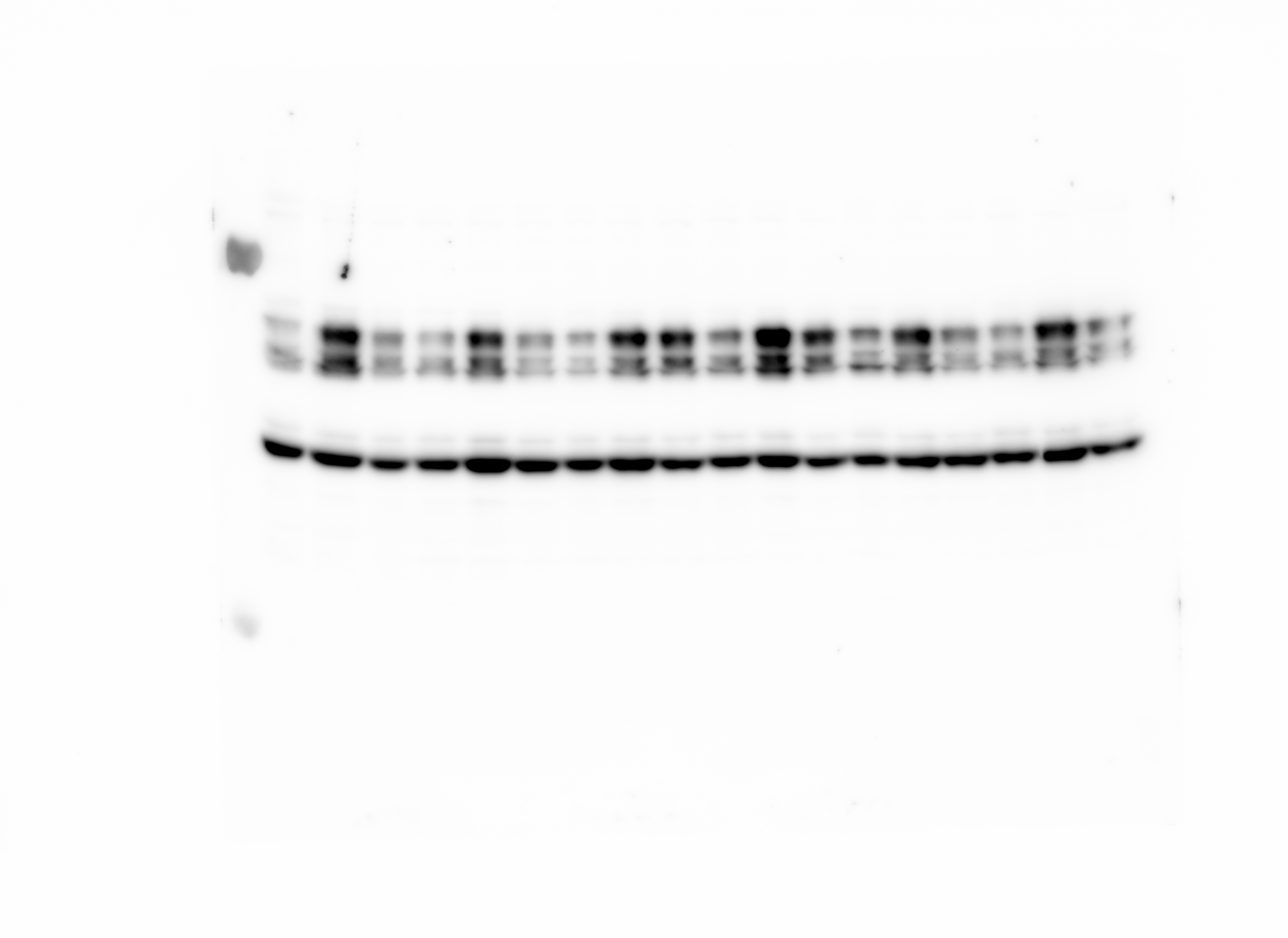

Supplement: Supplementary file 10 — Figure EV4 Source Data [file 44318_2024_196_MOESM10_ESM.zip › Figure EV4/EV4C/Quantification data/p-c-Jun membrane 3.tif]

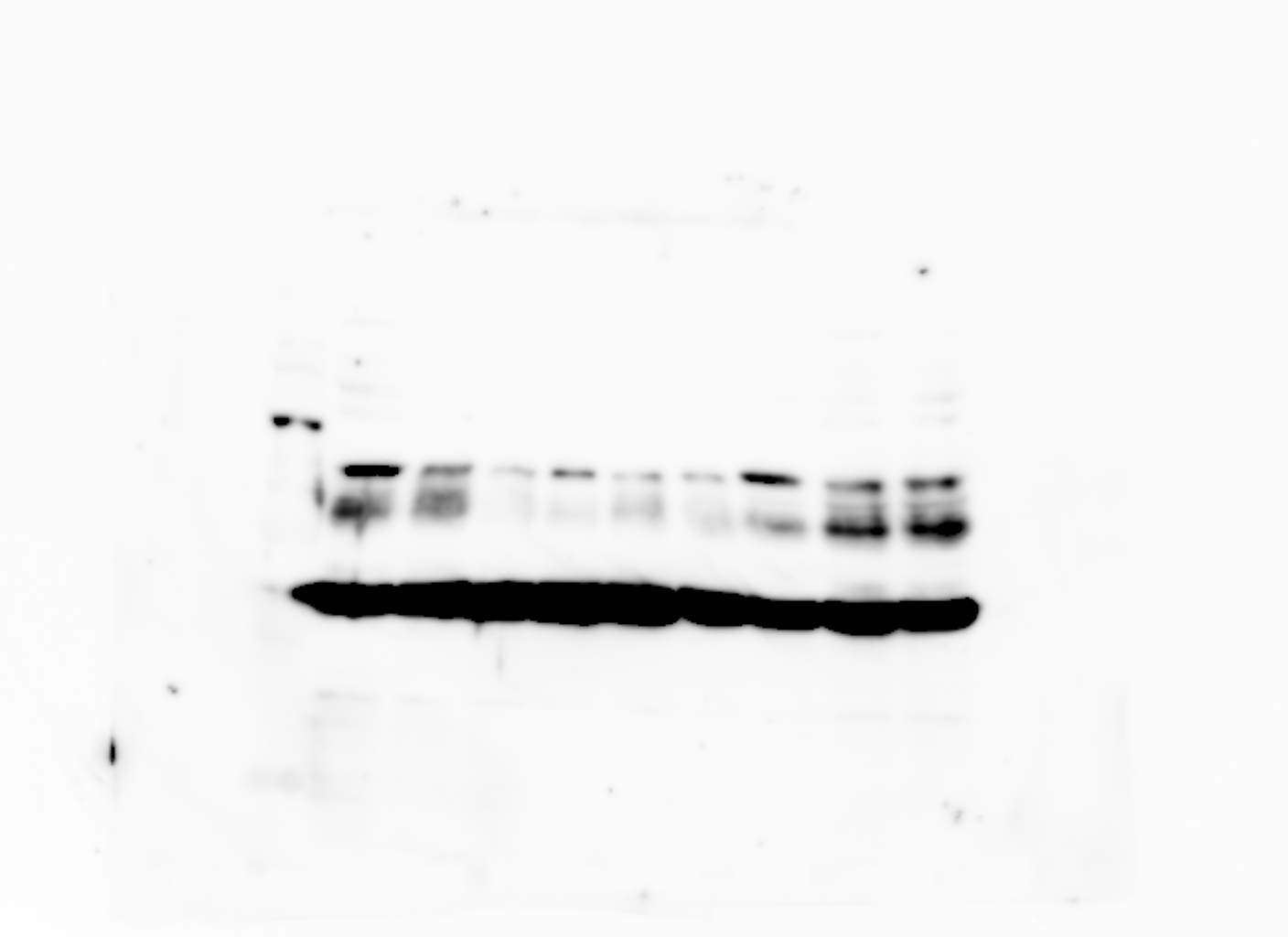

Supplement: Supplementary file 10 — Figure EV4 Source Data [file 44318_2024_196_MOESM10_ESM.zip › Figure EV4/EV4C/Quantification data/c-Fos membrane 1.tif.tif]

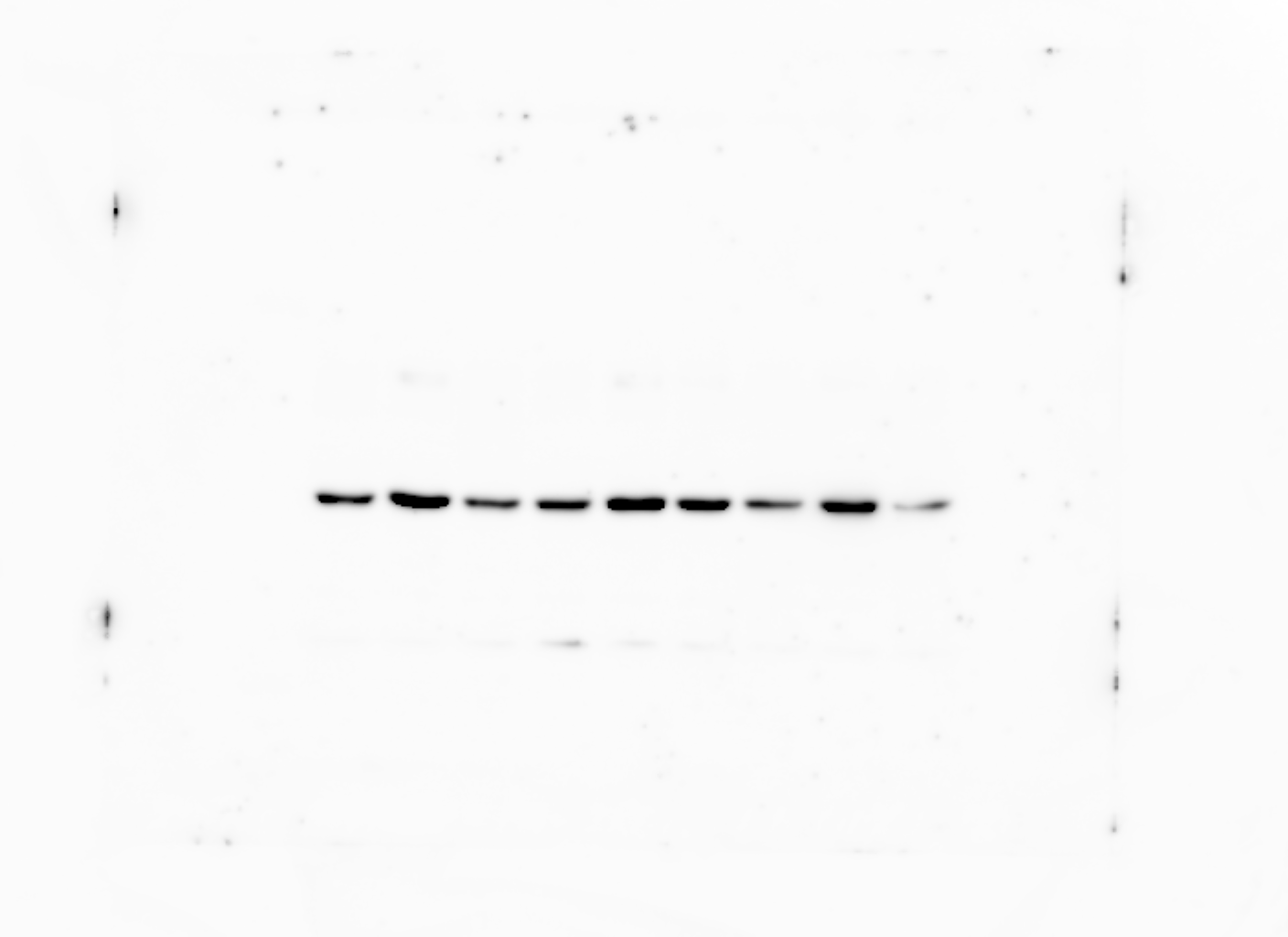

Supplement: Supplementary file 10 — Figure EV4 Source Data [file 44318_2024_196_MOESM10_ESM.zip › Figure EV4/EV4C/Quantification data/p-c-Jun membrane 2.tif]

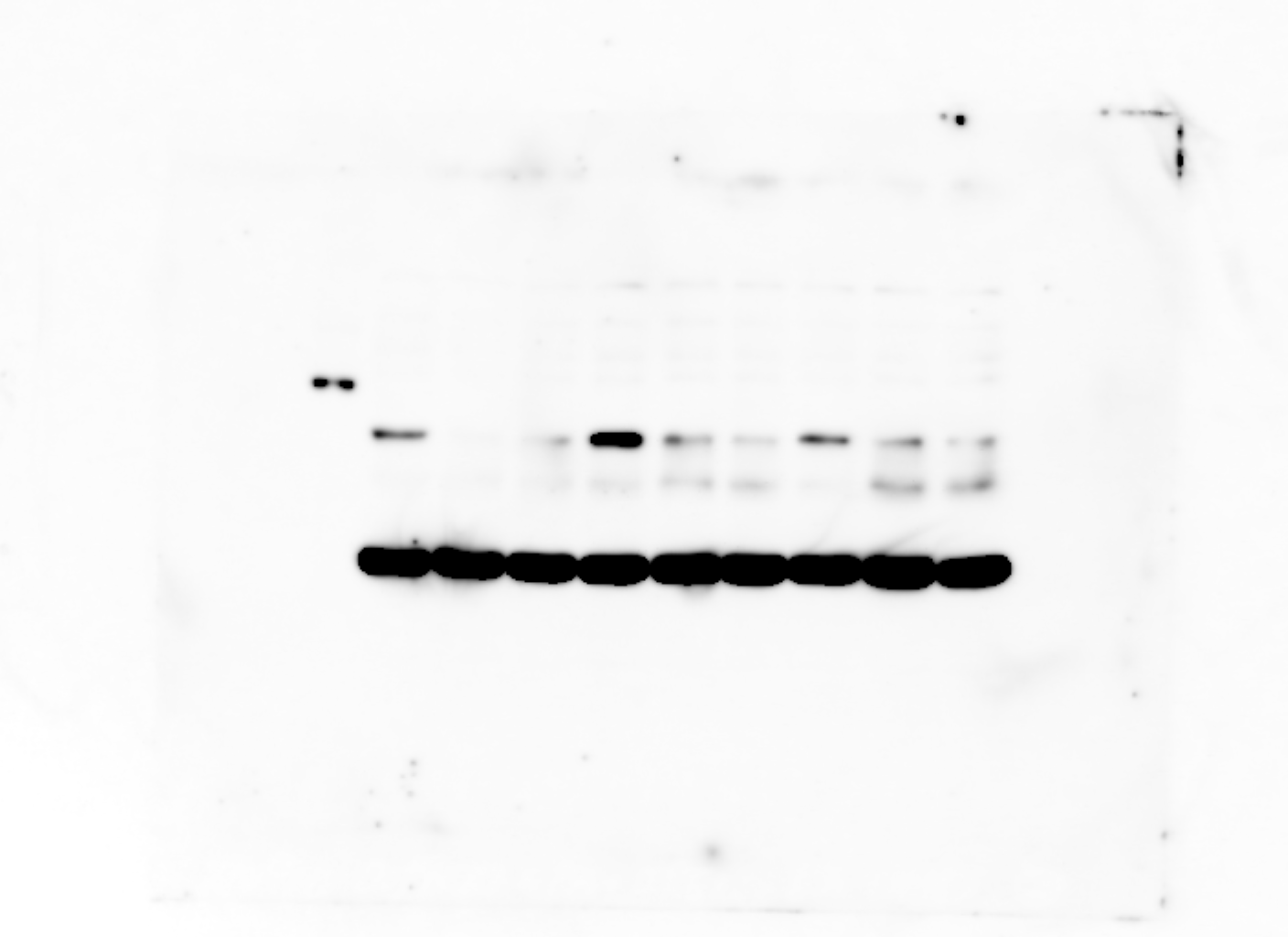

Supplement: Supplementary file 10 — Figure EV4 Source Data [file 44318_2024_196_MOESM10_ESM.zip › Figure EV4/EV4C/Quantification data/c-Fos membrane 2.tif]

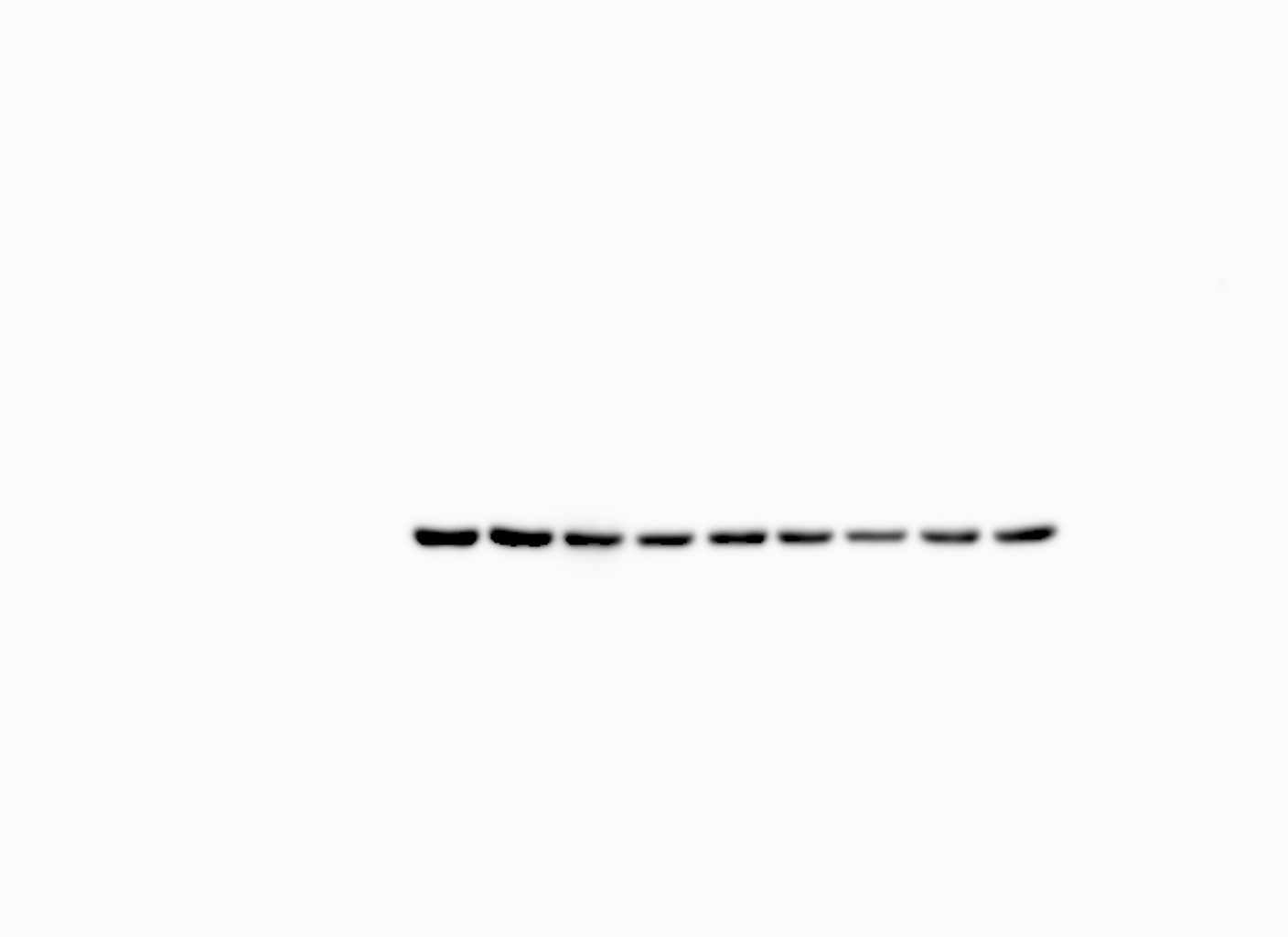

Supplement: Supplementary file 10 — Figure EV4 Source Data [file 44318_2024_196_MOESM10_ESM.zip › Figure EV4/EV4C/Quantification data/c-Jun membrane 1.tif]

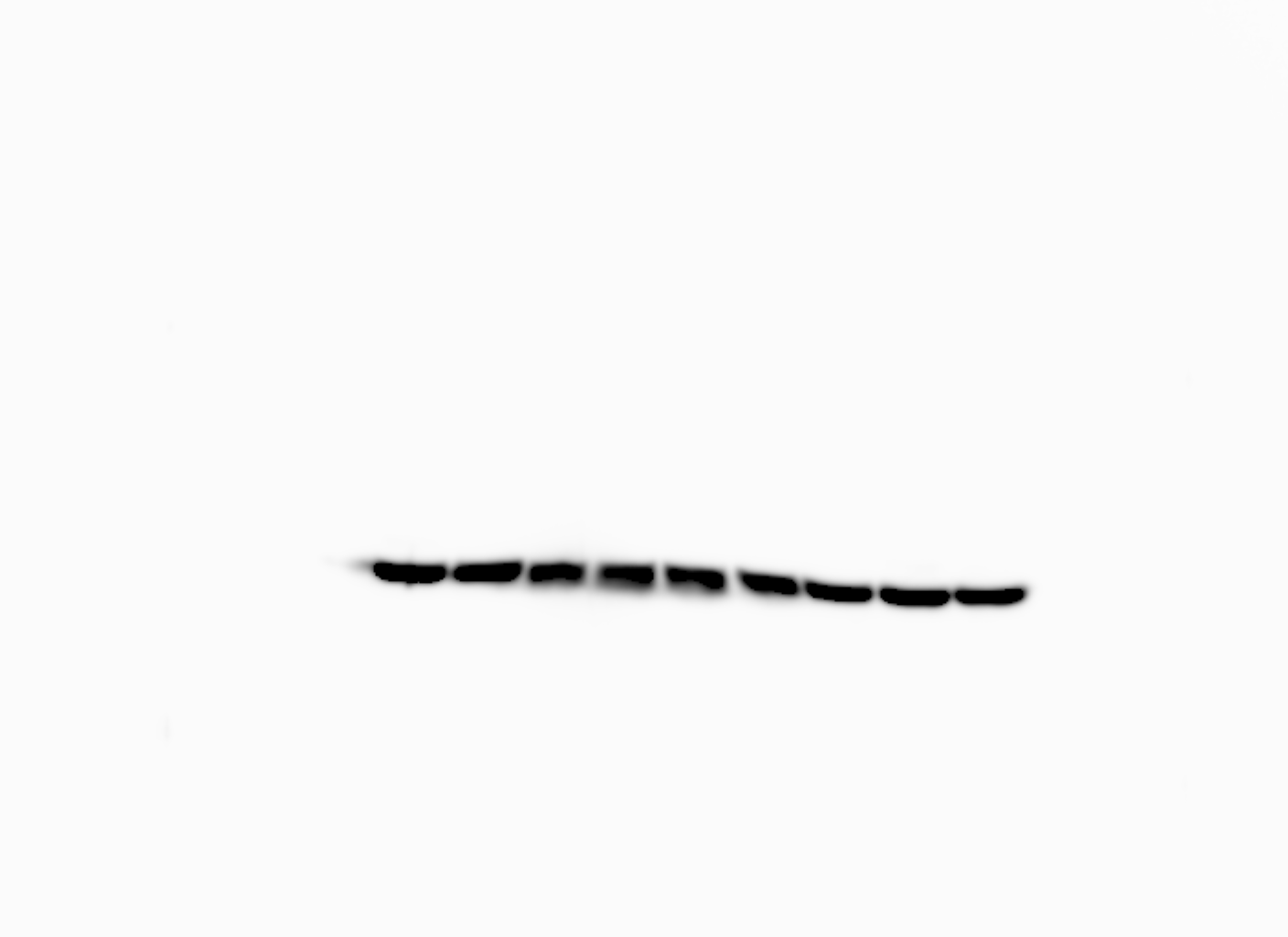

Supplement: Supplementary file 10 — Figure EV4 Source Data [file 44318_2024_196_MOESM10_ESM.zip › Figure EV4/EV4C/Quantification data/c-Jun membrane 2.tif]

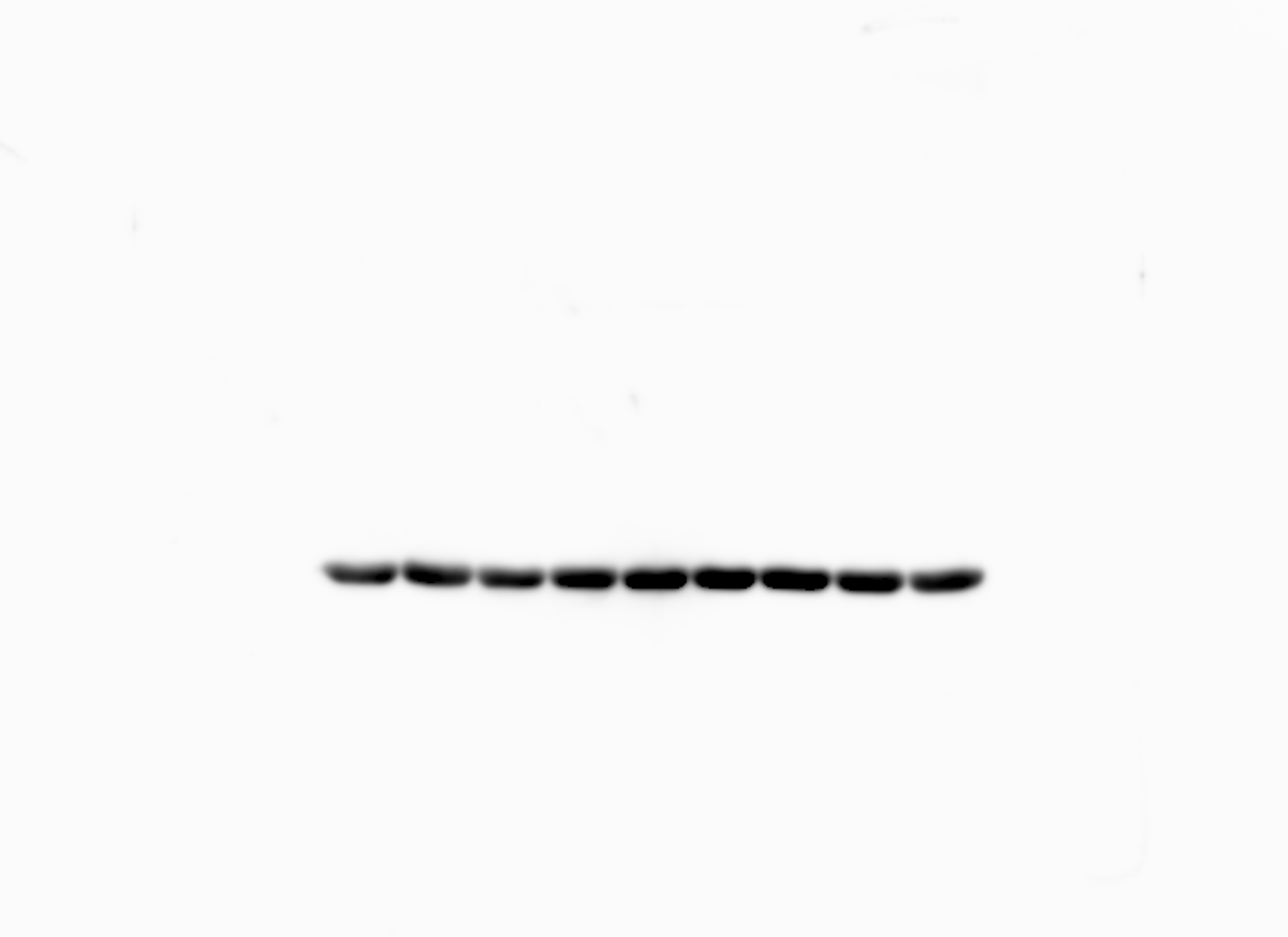

Supplement: Supplementary file 10 — Figure EV4 Source Data [file 44318_2024_196_MOESM10_ESM.zip › Figure EV4/EV4C/Quantification data/GAPDH membrane 1.tif]

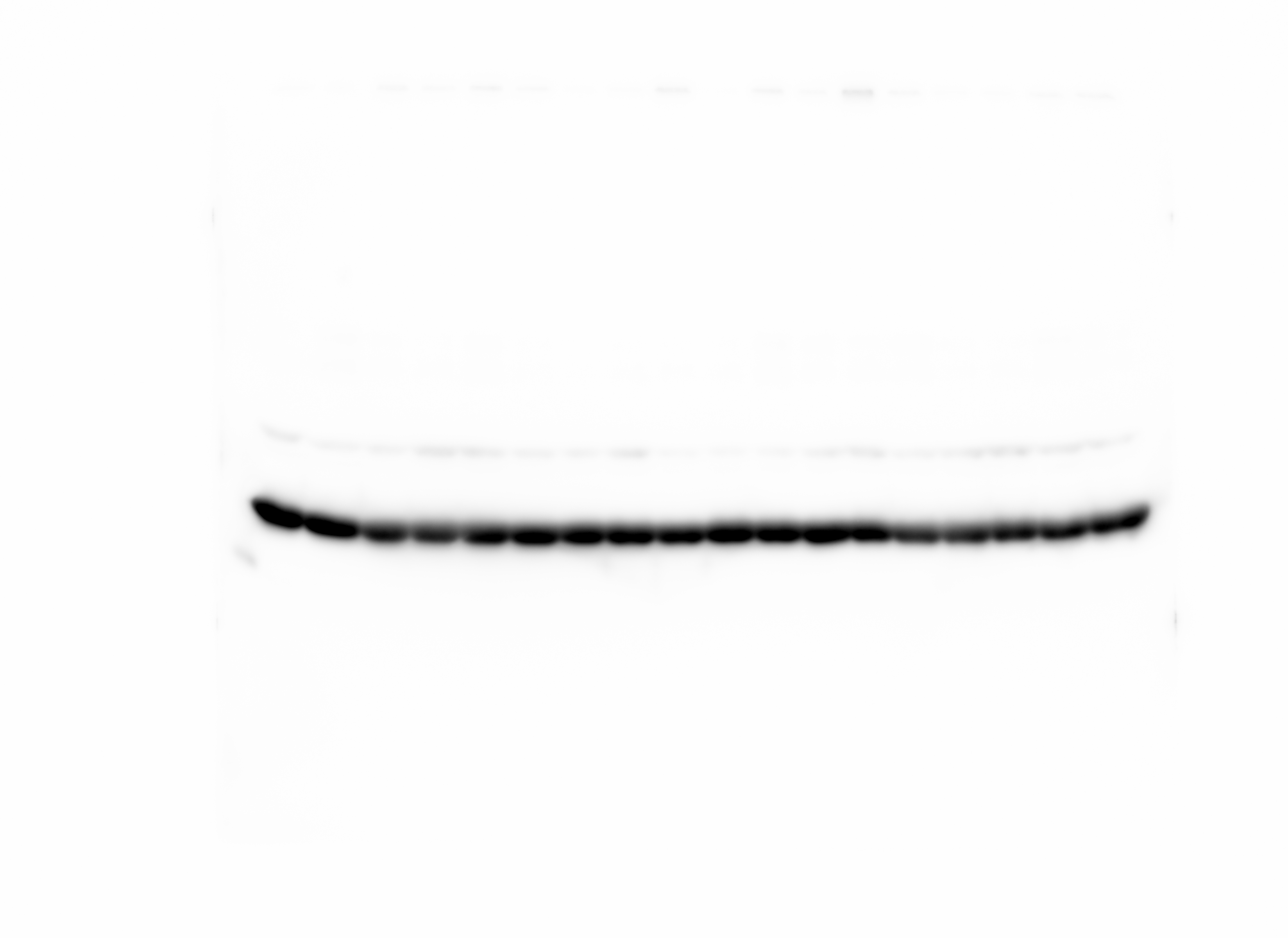

Supplement: Supplementary file 10 — Figure EV4 Source Data [file 44318_2024_196_MOESM10_ESM.zip › Figure EV4/EV4C/Quantification data/GAPDH membrane 3.tif]

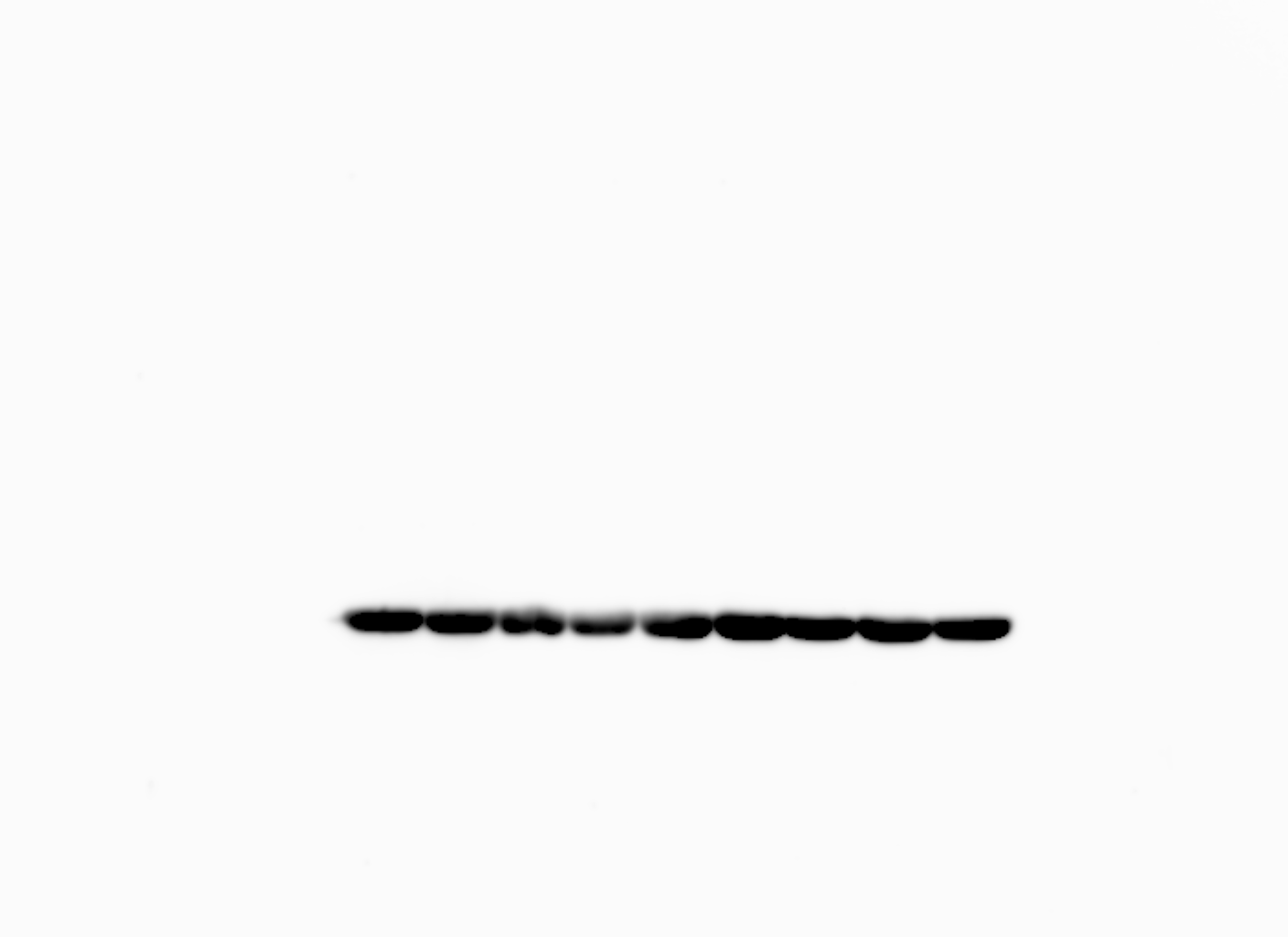

Supplement: Supplementary file 10 — Figure EV4 Source Data [file 44318_2024_196_MOESM10_ESM.zip › Figure EV4/EV4C/Quantification data/GAPDH membrane 2.tif]

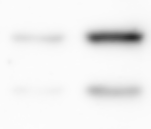

Supplement: Supplementary file 10 — Figure EV4 Source Data [file 44318_2024_196_MOESM10_ESM.zip › Figure EV4/EV4B/Demonstrated data/p-JNK1:2.tif]

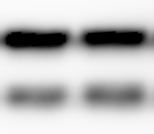

Supplement: Supplementary file 10 — Figure EV4 Source Data [file 44318_2024_196_MOESM10_ESM.zip › Figure EV4/EV4B/Demonstrated data/JNK1:2.tif]

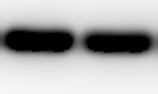

Supplement: Supplementary file 10 — Figure EV4 Source Data [file 44318_2024_196_MOESM10_ESM.zip › Figure EV4/EV4B/Demonstrated data/GAPDH.tif]

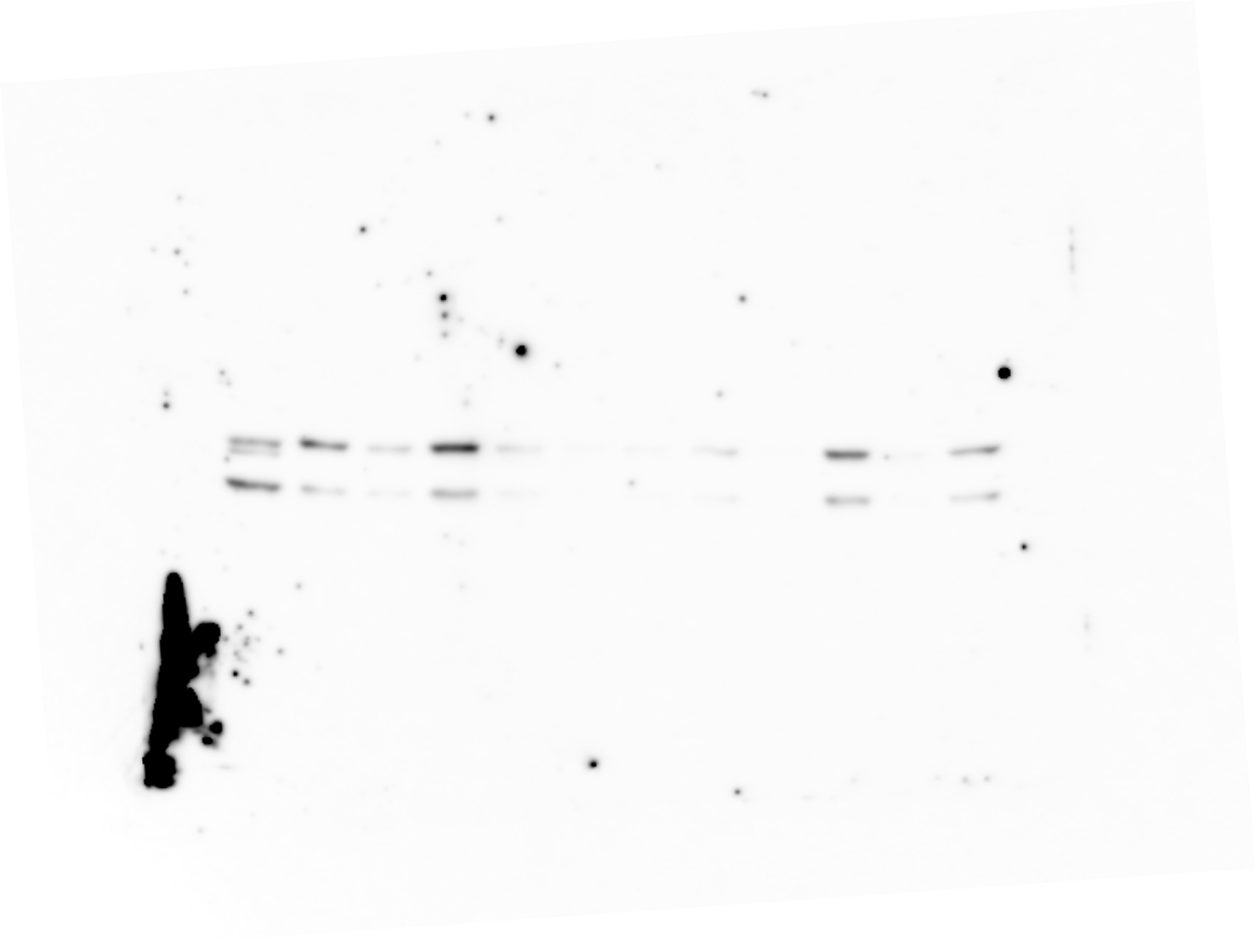

Supplement: Supplementary file 10 — Figure EV4 Source Data [file 44318_2024_196_MOESM10_ESM.zip › Figure EV4/EV4B/Quantification data/p-JNK1:2.tif]

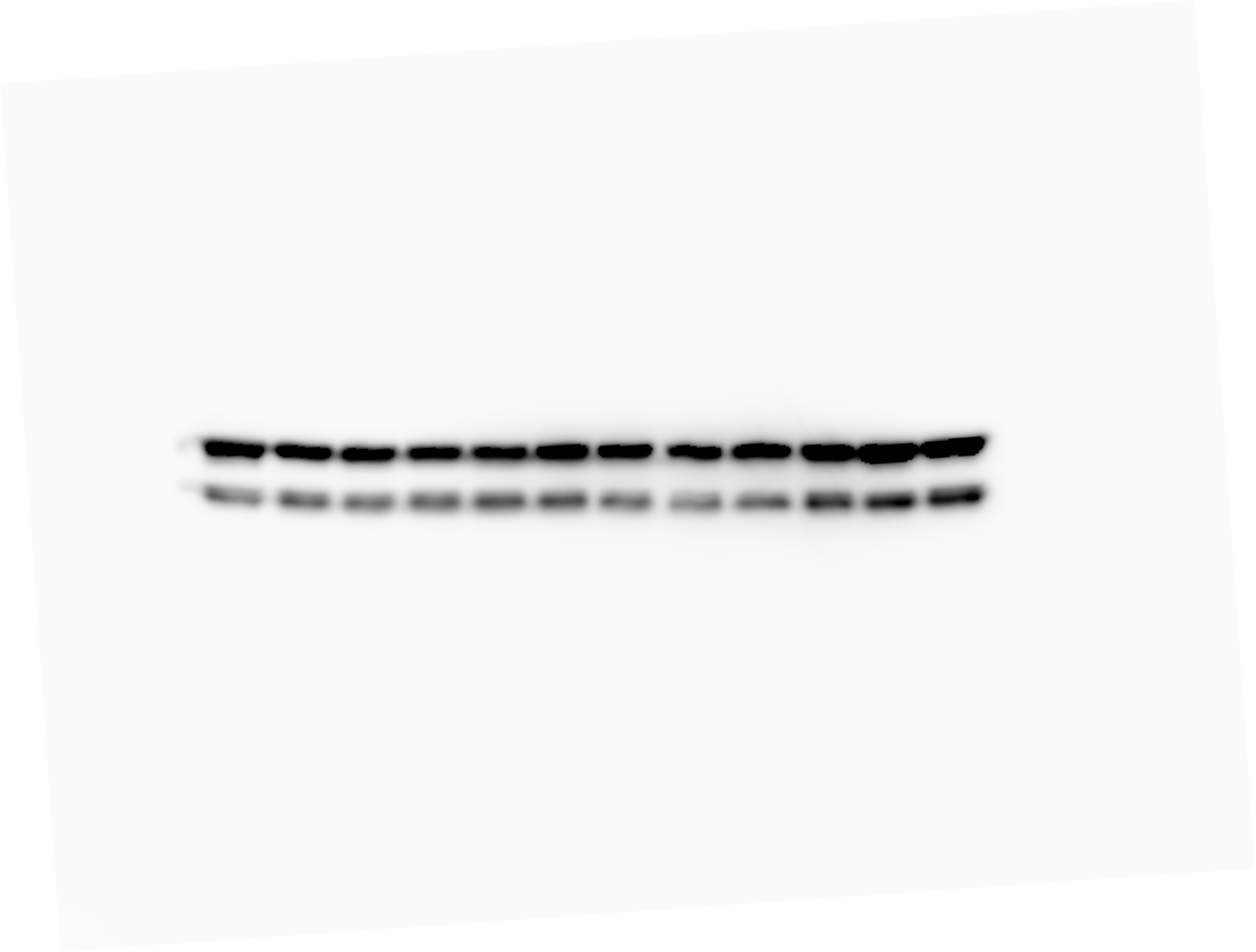

Supplement: Supplementary file 10 — Figure EV4 Source Data [file 44318_2024_196_MOESM10_ESM.zip › Figure EV4/EV4B/Quantification data/JNK1:2.tif]

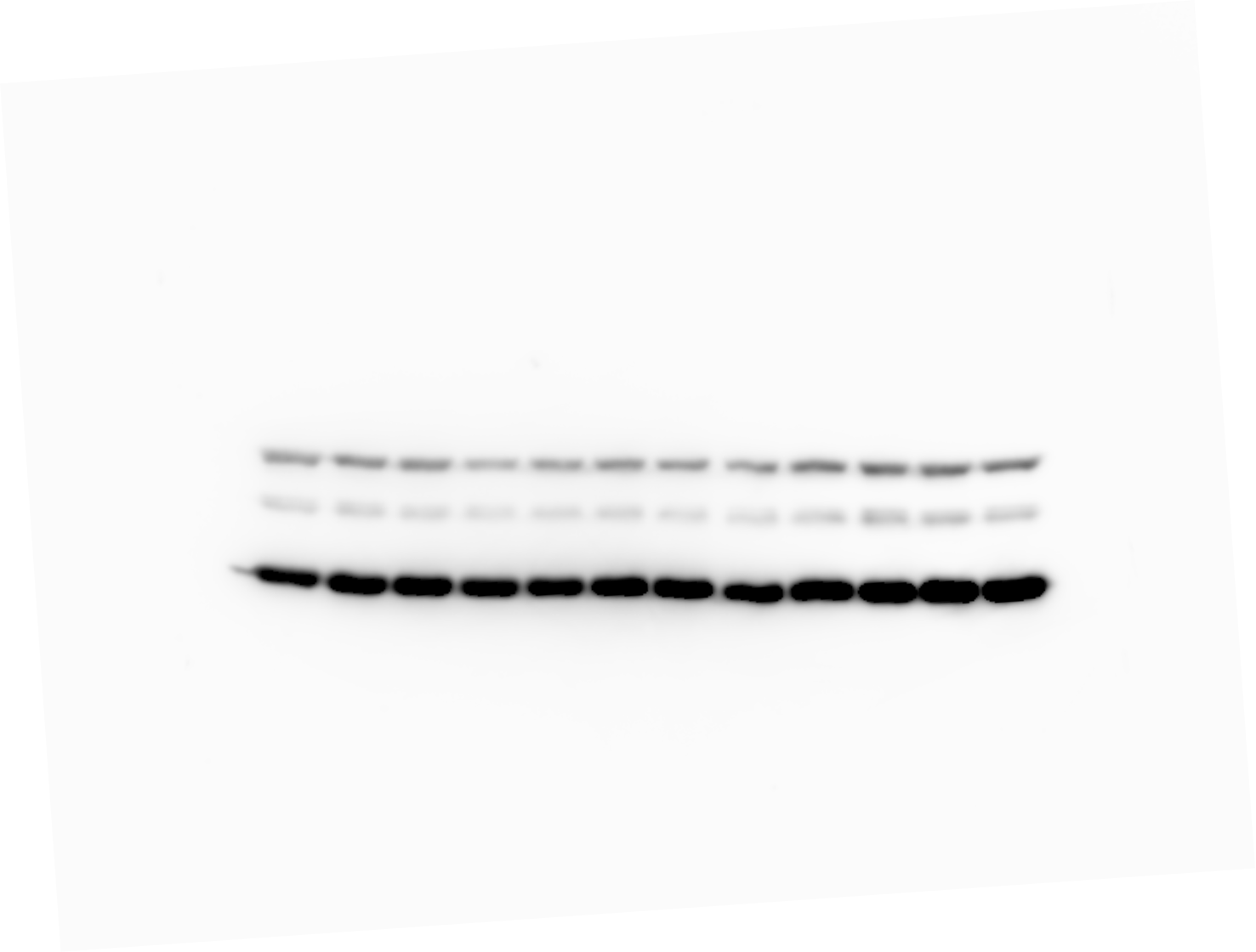

Supplement: Supplementary file 10 — Figure EV4 Source Data [file 44318_2024_196_MOESM10_ESM.zip › Figure EV4/EV4B/Quantification data/GAPDH.tif]

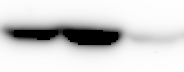

Supplement: Supplementary file 10 — Figure EV4 Source Data [file 44318_2024_196_MOESM10_ESM.zip › Figure EV4/EV4E/Demonstrated data/p-c-Jun.tif]

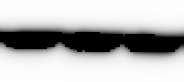

Supplement: Supplementary file 10 — Figure EV4 Source Data [file 44318_2024_196_MOESM10_ESM.zip › Figure EV4/EV4E/Demonstrated data/Actin.tif]

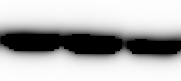

Supplement: Supplementary file 10 — Figure EV4 Source Data [file 44318_2024_196_MOESM10_ESM.zip › Figure EV4/EV4E/Demonstrated data/c-Jun.tif]

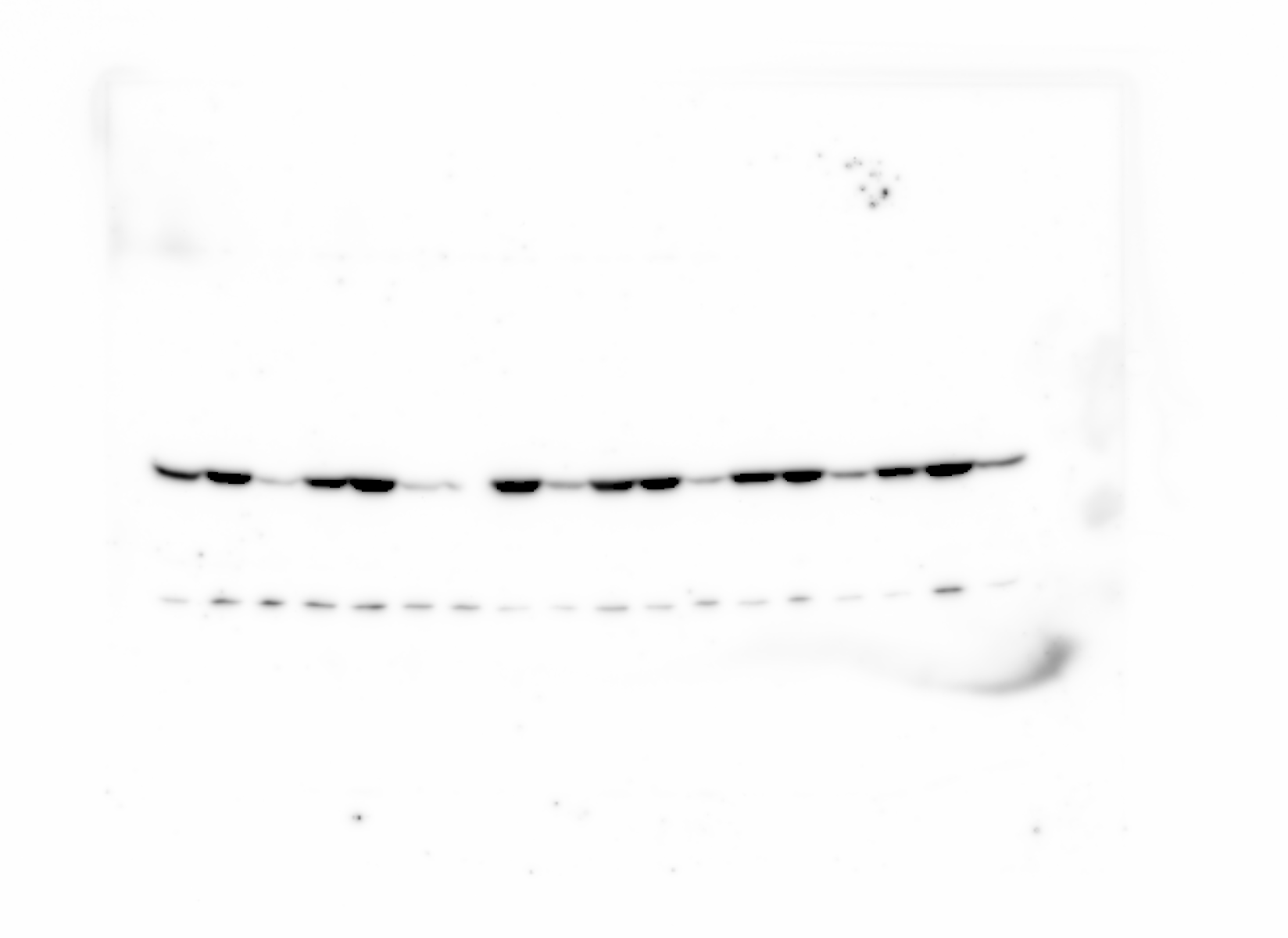

Supplement: Supplementary file 10 — Figure EV4 Source Data [file 44318_2024_196_MOESM10_ESM.zip › Figure EV4/EV4E/Quantification data/p-c-Jun.tif]

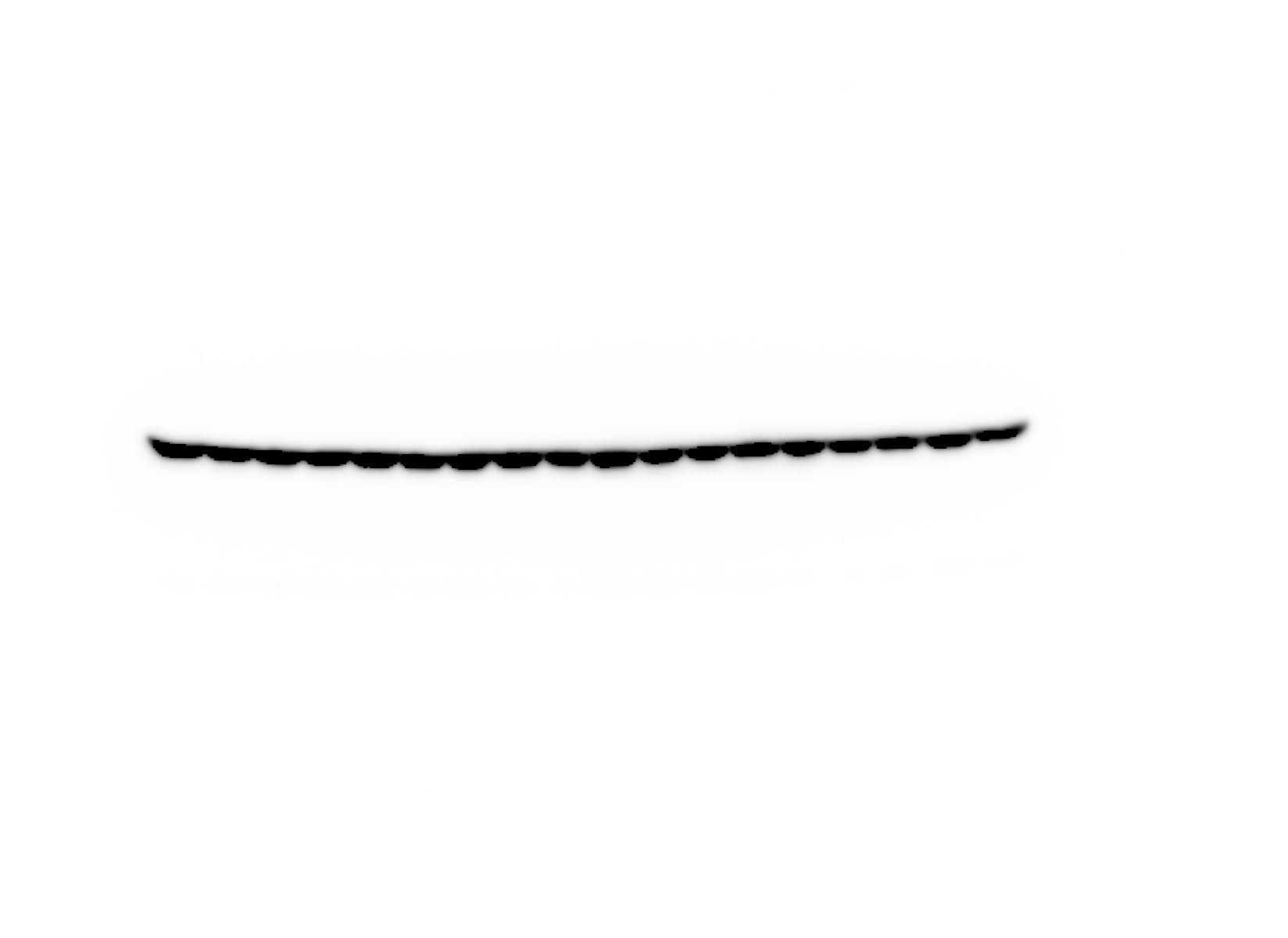

Supplement: Supplementary file 10 — Figure EV4 Source Data [file 44318_2024_196_MOESM10_ESM.zip › Figure EV4/EV4E/Quantification data/Actin.tif]

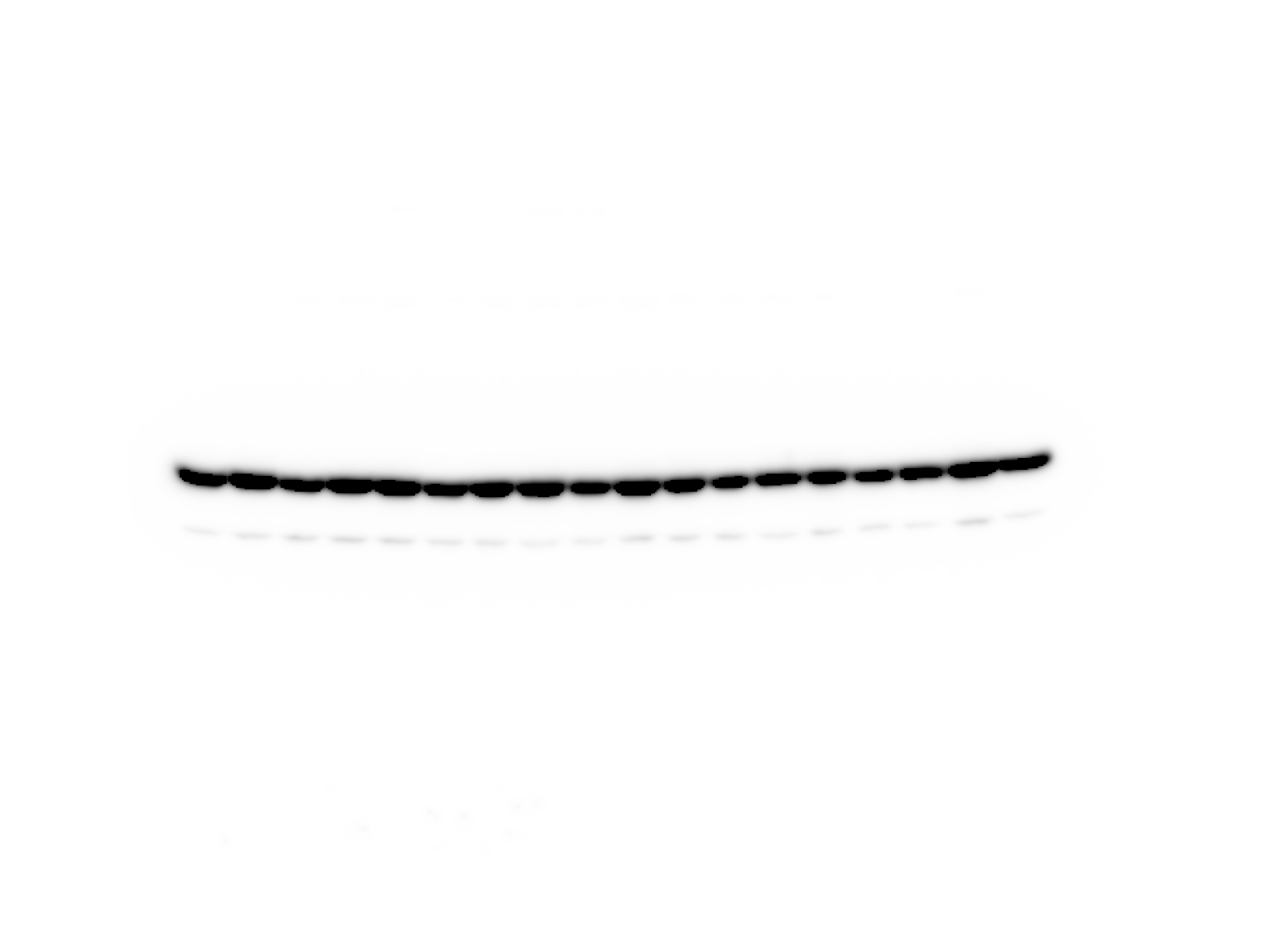

Supplement: Supplementary file 10 — Figure EV4 Source Data [file 44318_2024_196_MOESM10_ESM.zip › Figure EV4/EV4E/Quantification data/c-Jun.tif]
