# Supplementary figures and images for: PCPE-1, a brown adipose tissue-derived cytokine, promotes obesity-induced liver fibrosis (part 5 of 6)
Source: EMBO J. 2024 Aug 19;43(21):4846–69. doi: 10.1038/s44318-024-00196-0 (PMC11535236; doi:10.1038/s44318-024-00196-0)

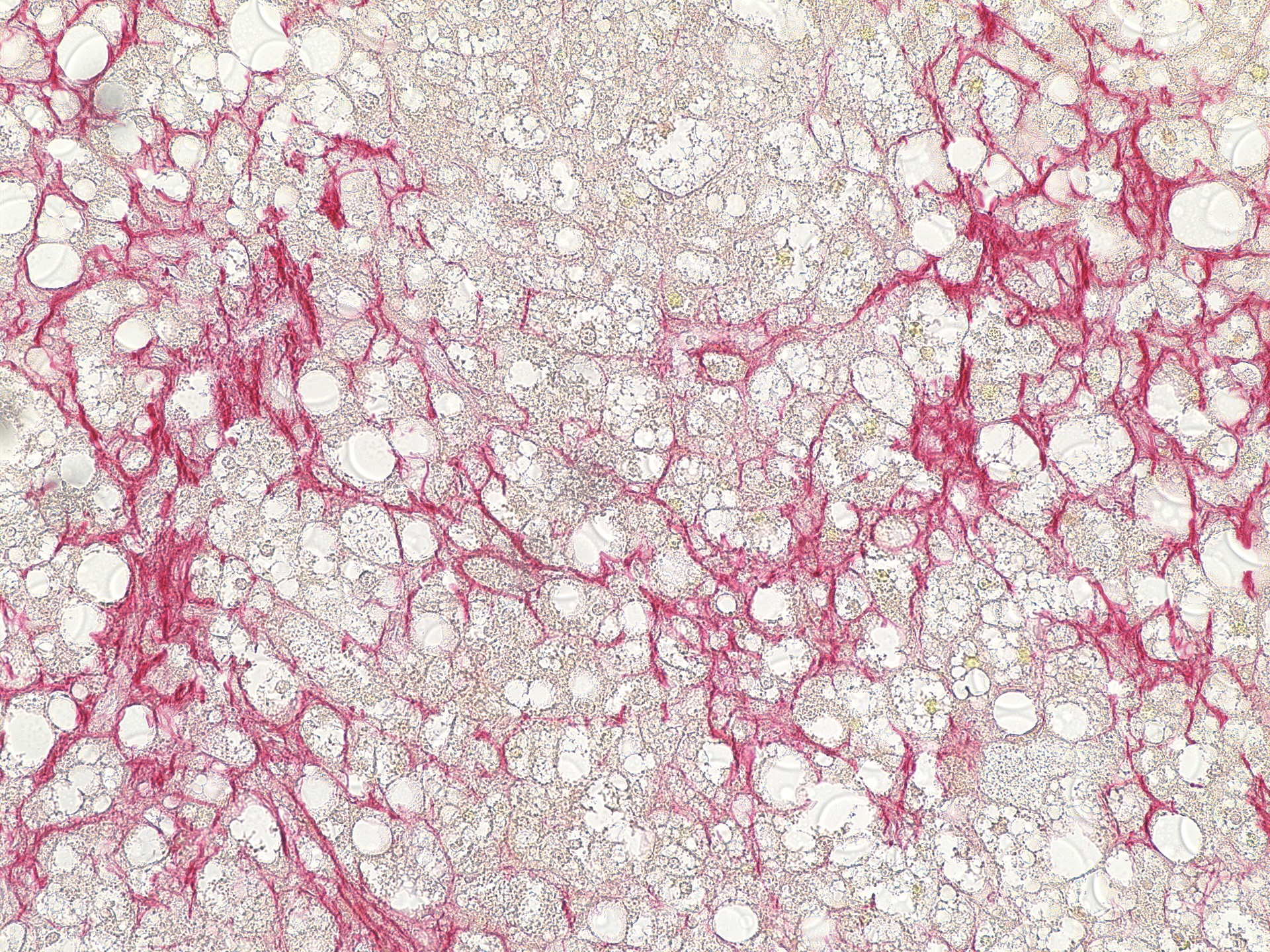

Supplement: Supplementary file 9 — Figure EV3 Source Data [file 44318_2024_196_MOESM9_ESM.zip › Figure EV3/Figure EV3-L/Quantificated image/HFD Con/no.1/Liver-HFD con-no.1-20x-4.jpg]

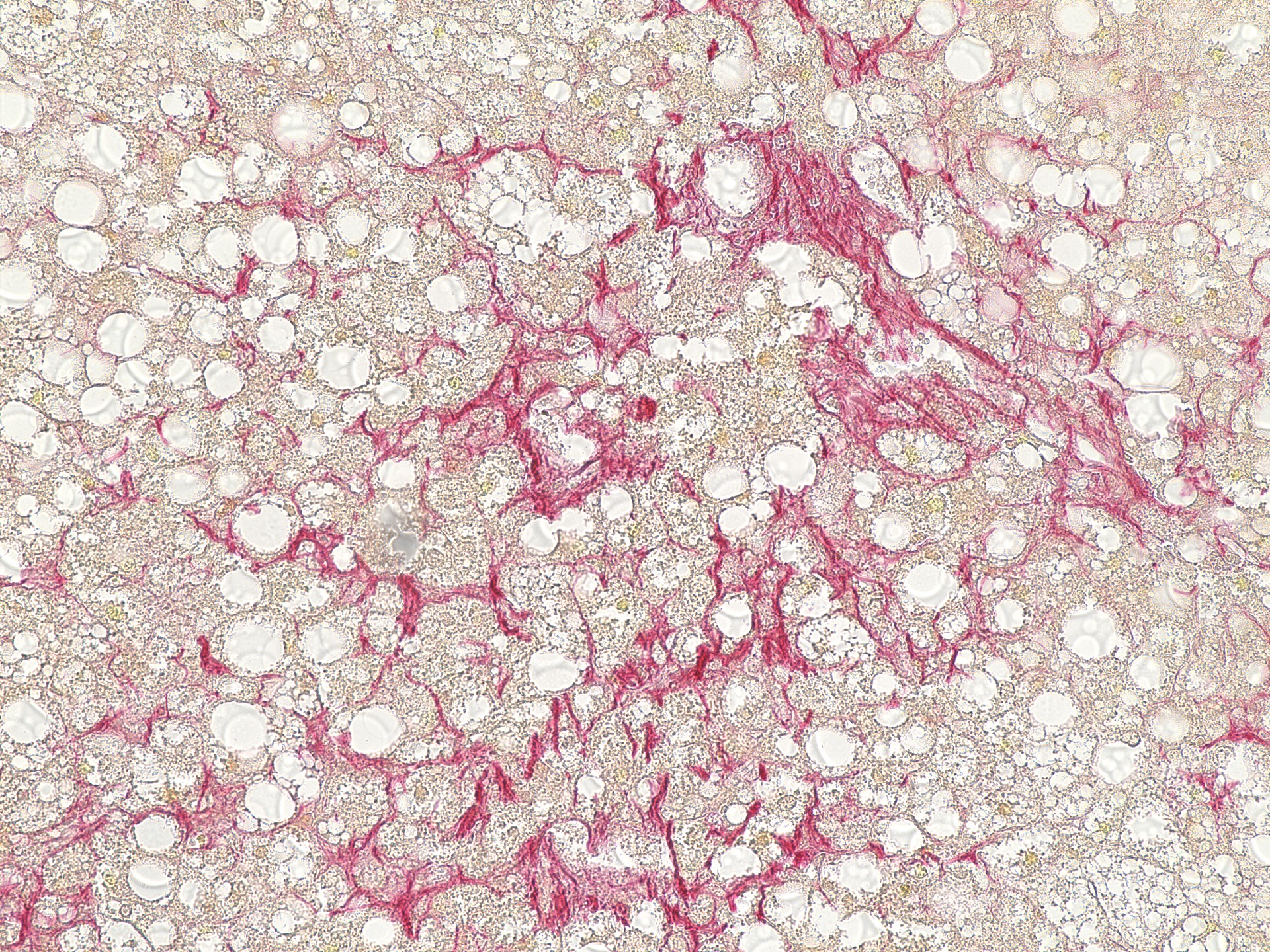

Supplement: Supplementary file 9 — Figure EV3 Source Data [file 44318_2024_196_MOESM9_ESM.zip › Figure EV3/Figure EV3-L/Quantificated image/HFD Con/no.1/Liver-HFD con-no.1-20x-1.jpg]

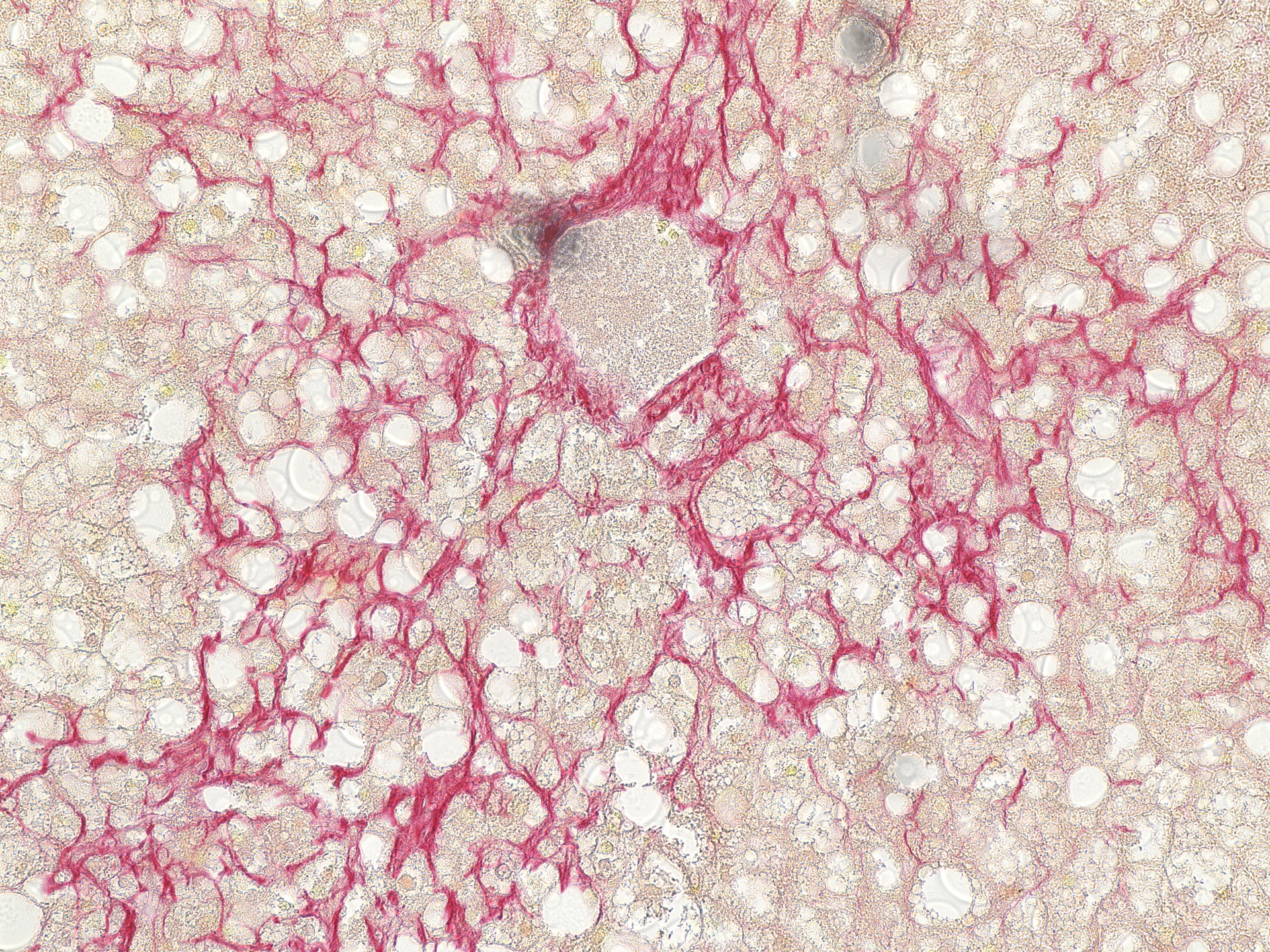

Supplement: Supplementary file 9 — Figure EV3 Source Data [file 44318_2024_196_MOESM9_ESM.zip › Figure EV3/Figure EV3-L/Quantificated image/HFD Con/no.1/Liver-HFD con-no.1-20x-3.jpg]

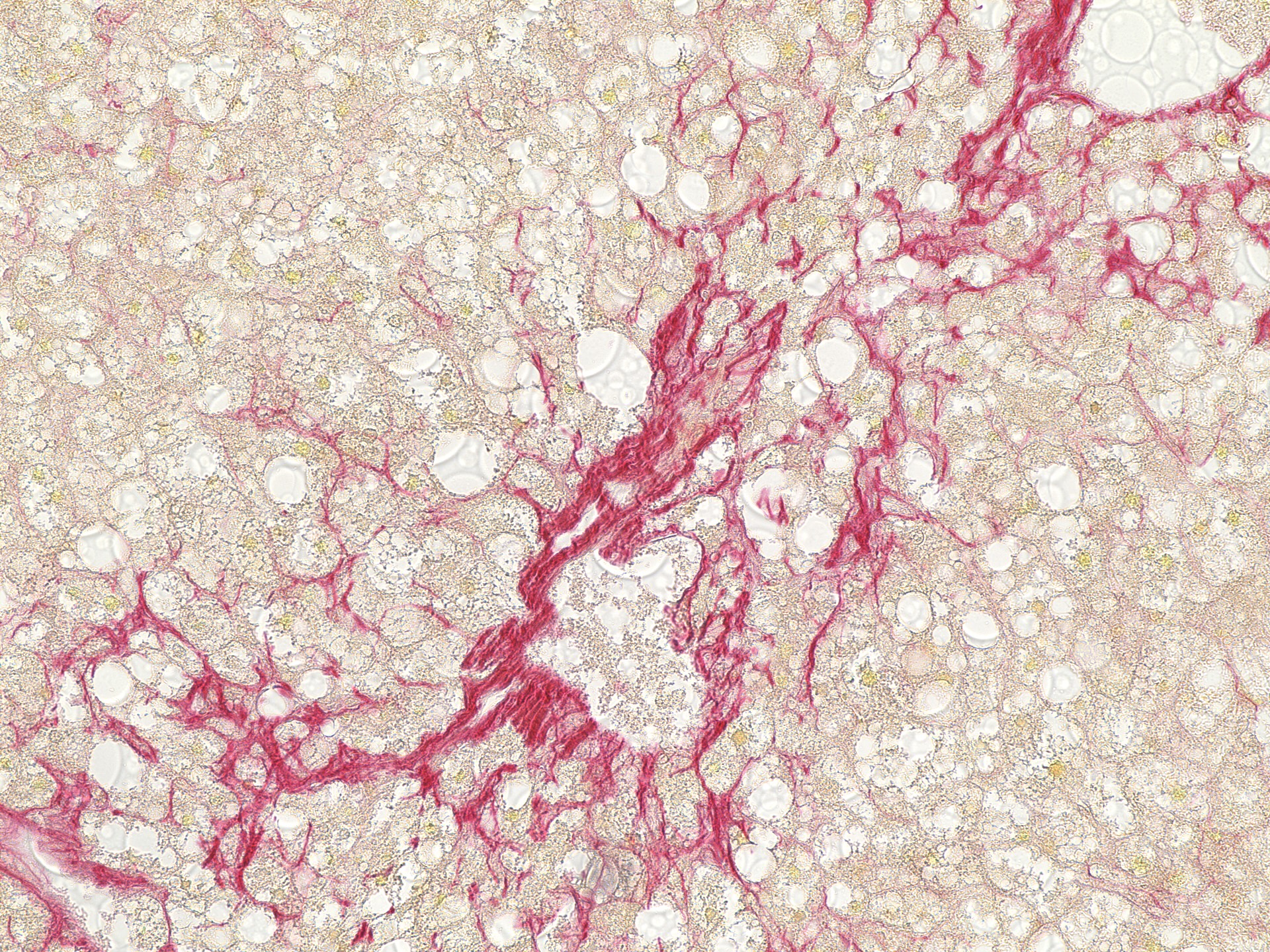

Supplement: Supplementary file 9 — Figure EV3 Source Data [file 44318_2024_196_MOESM9_ESM.zip › Figure EV3/Figure EV3-L/Quantificated image/HFD Con/no.1/Liver-HFD con-no.1-20x-2.jpg]

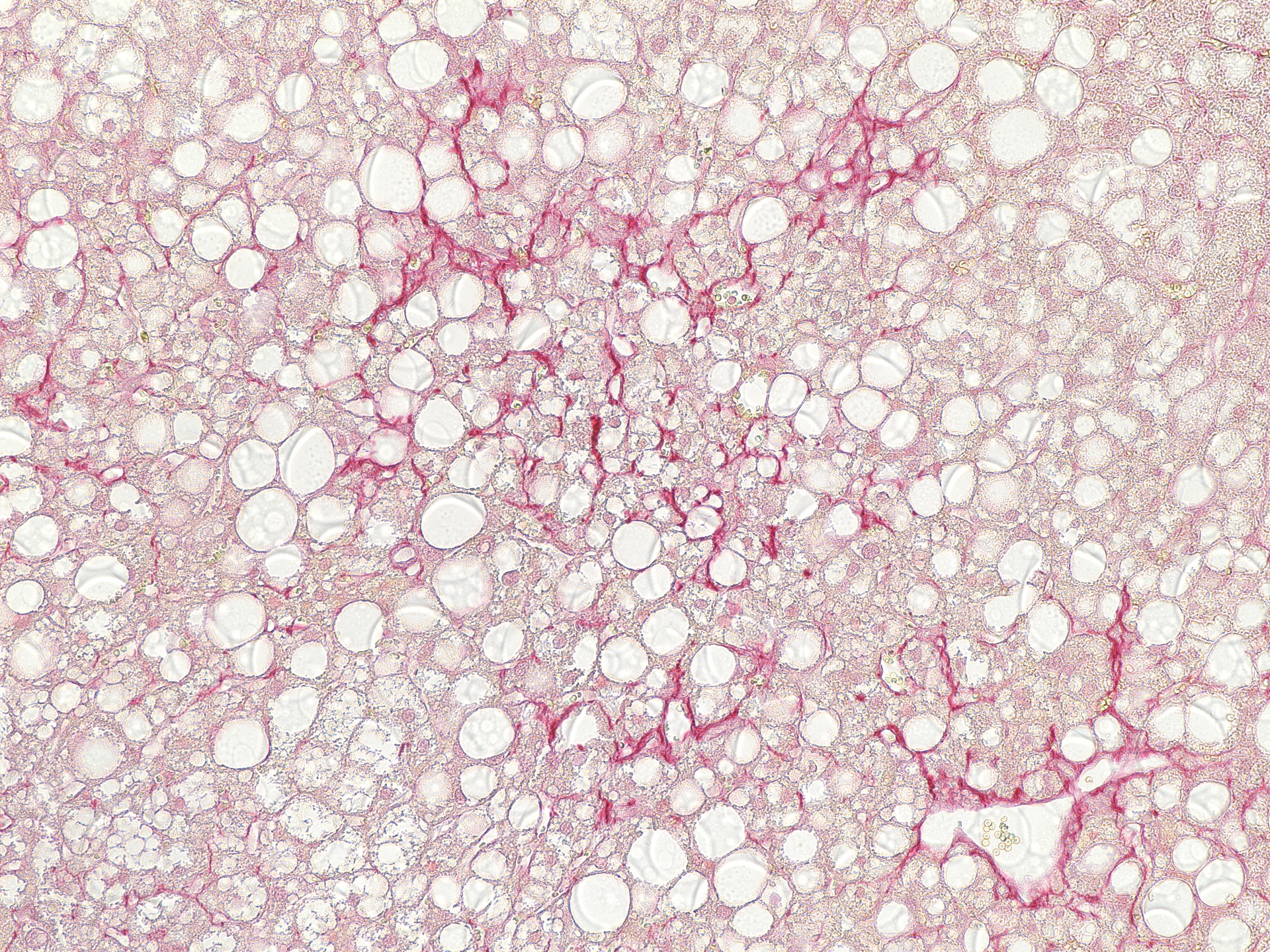

Supplement: Supplementary file 9 — Figure EV3 Source Data [file 44318_2024_196_MOESM9_ESM.zip › Figure EV3/Figure EV3-L/Quantificated image/HFD Con/no.3/Liver-HFD con-no.3-20x-4.jpg]

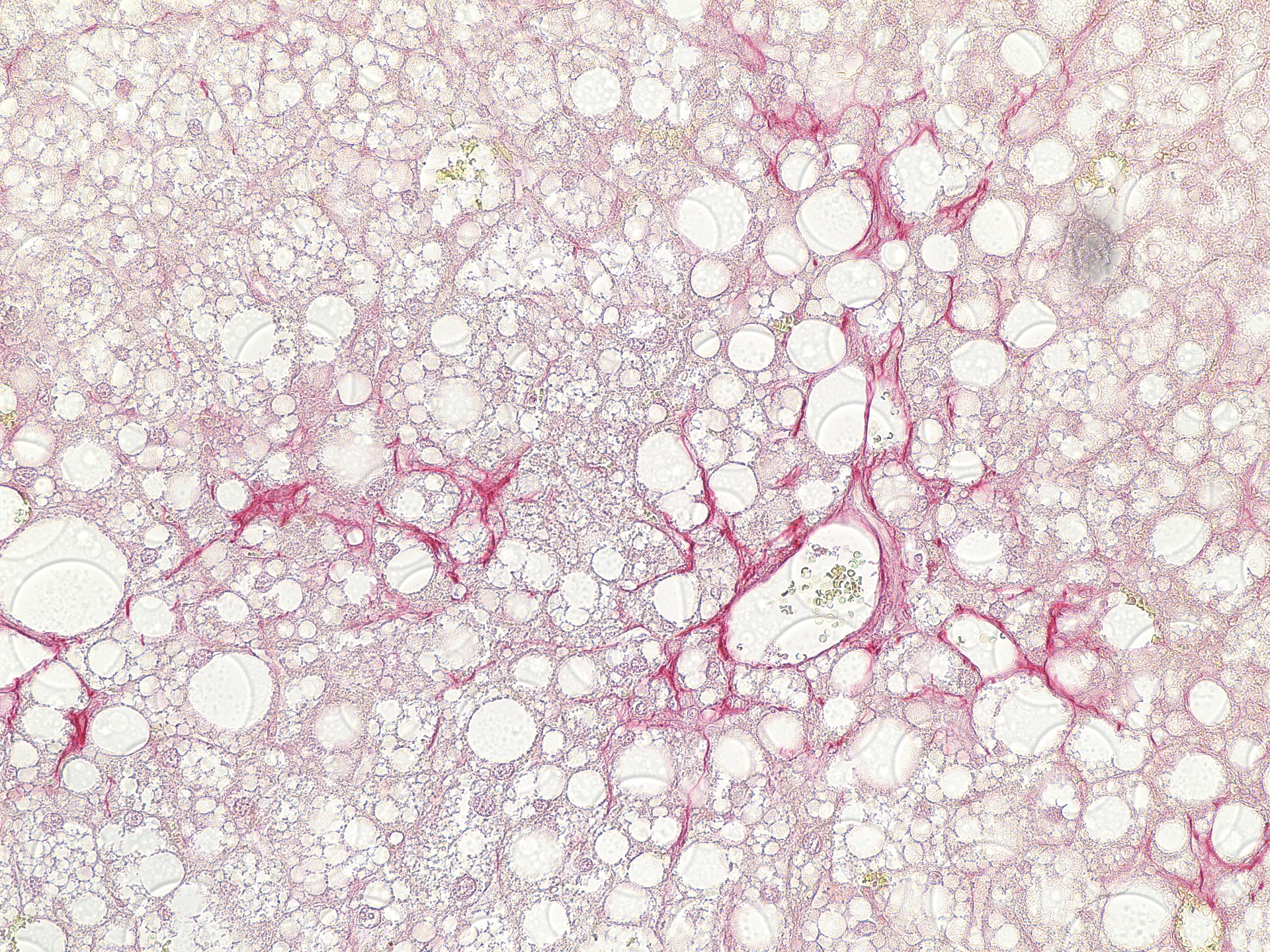

Supplement: Supplementary file 9 — Figure EV3 Source Data [file 44318_2024_196_MOESM9_ESM.zip › Figure EV3/Figure EV3-L/Quantificated image/HFD Con/no.3/Liver-HFD con-no.3-20x-5.jpg]

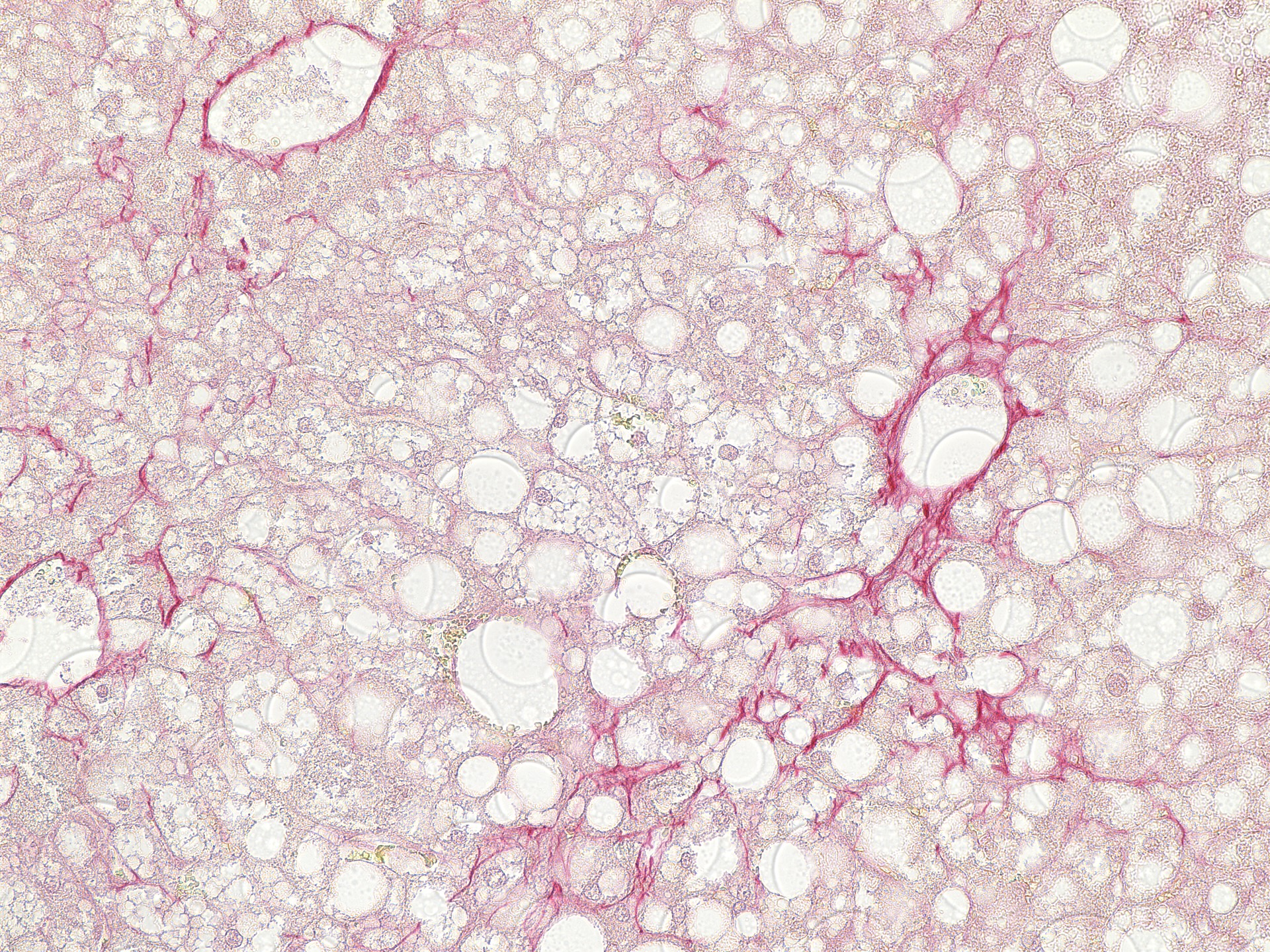

Supplement: Supplementary file 9 — Figure EV3 Source Data [file 44318_2024_196_MOESM9_ESM.zip › Figure EV3/Figure EV3-L/Quantificated image/HFD Con/no.3/Liver-HFD con-no.3-20x-1.jpg]

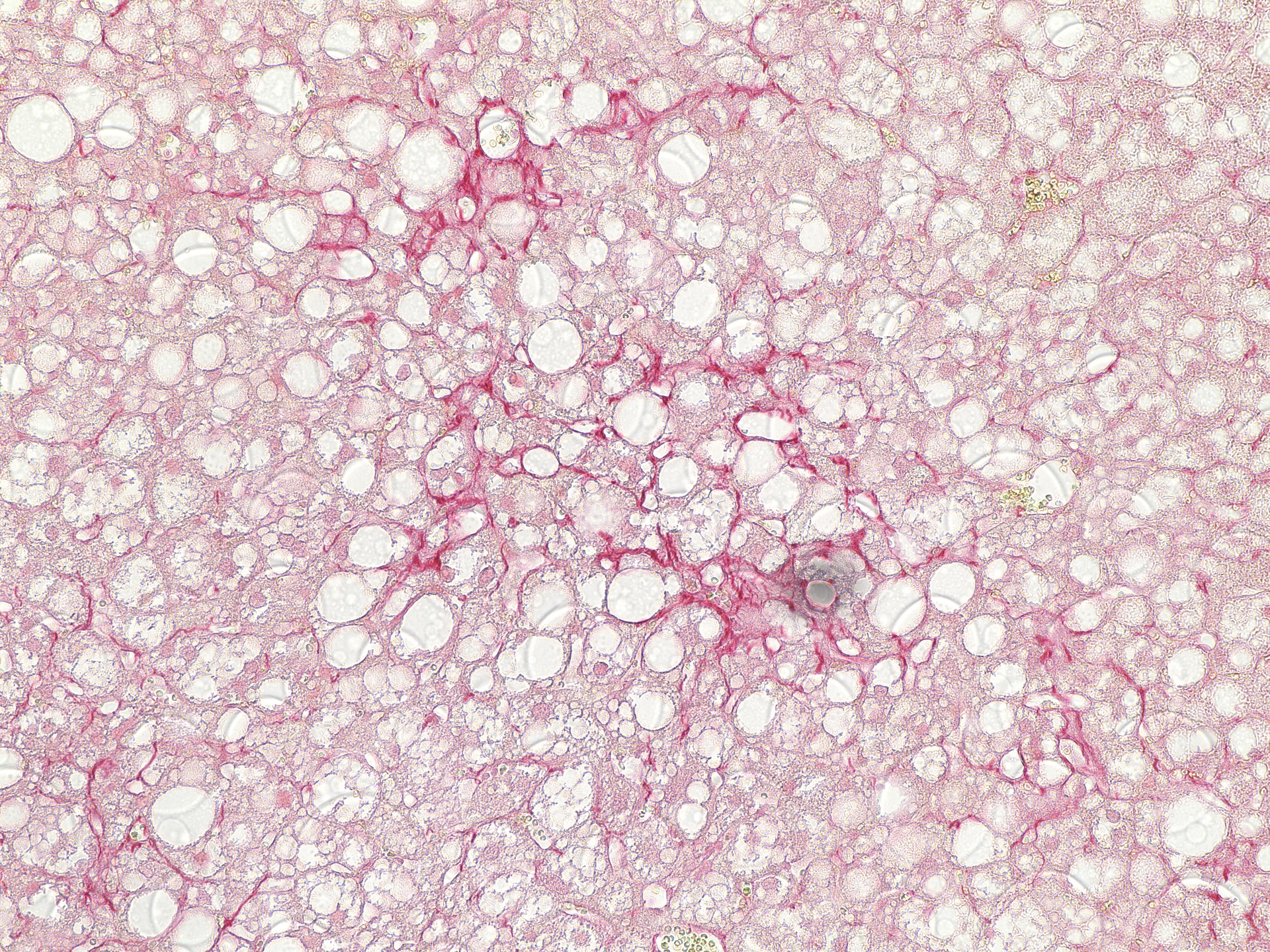

Supplement: Supplementary file 9 — Figure EV3 Source Data [file 44318_2024_196_MOESM9_ESM.zip › Figure EV3/Figure EV3-L/Quantificated image/HFD Con/no.3/Liver-HFD con-no.3-20x-2.jpg]

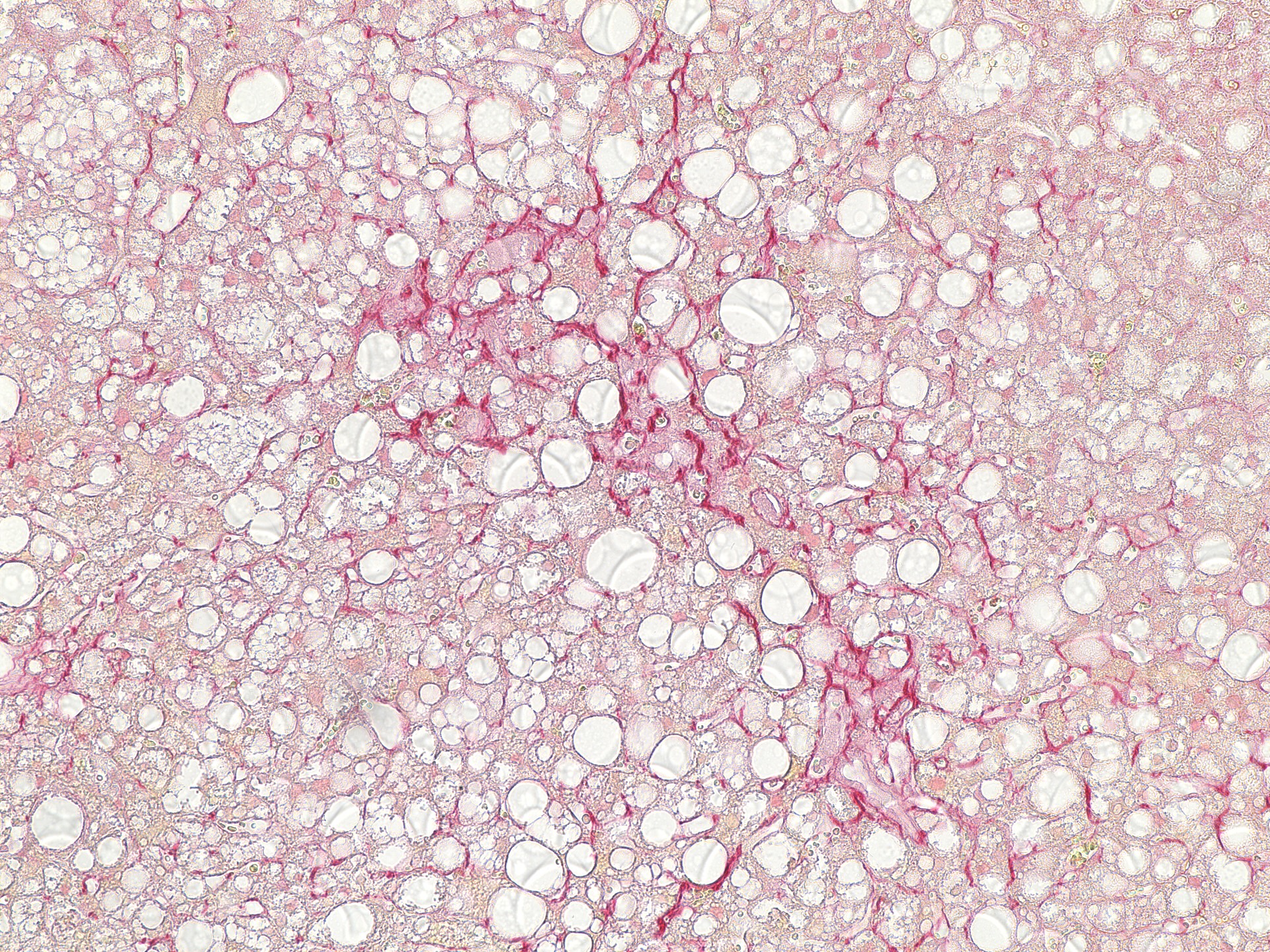

Supplement: Supplementary file 9 — Figure EV3 Source Data [file 44318_2024_196_MOESM9_ESM.zip › Figure EV3/Figure EV3-L/Quantificated image/HFD Con/no.3/Liver-HFD con-no.3-20x-3.jpg]

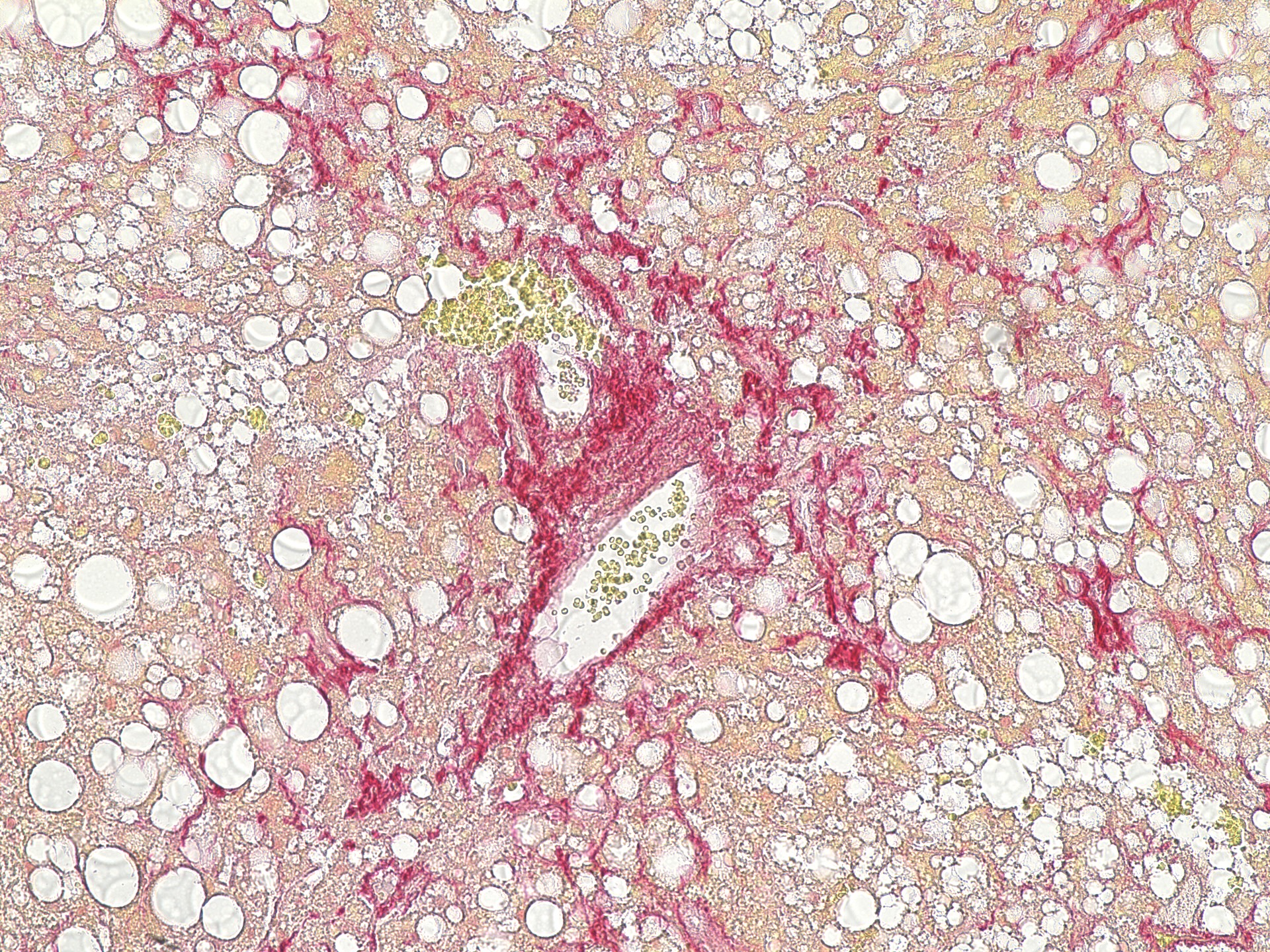

Supplement: Supplementary file 9 — Figure EV3 Source Data [file 44318_2024_196_MOESM9_ESM.zip › Figure EV3/Figure EV3-L/Quantificated image/HFD Con/no.4/Liver-HFD con-no.4-20x-1.jpg]

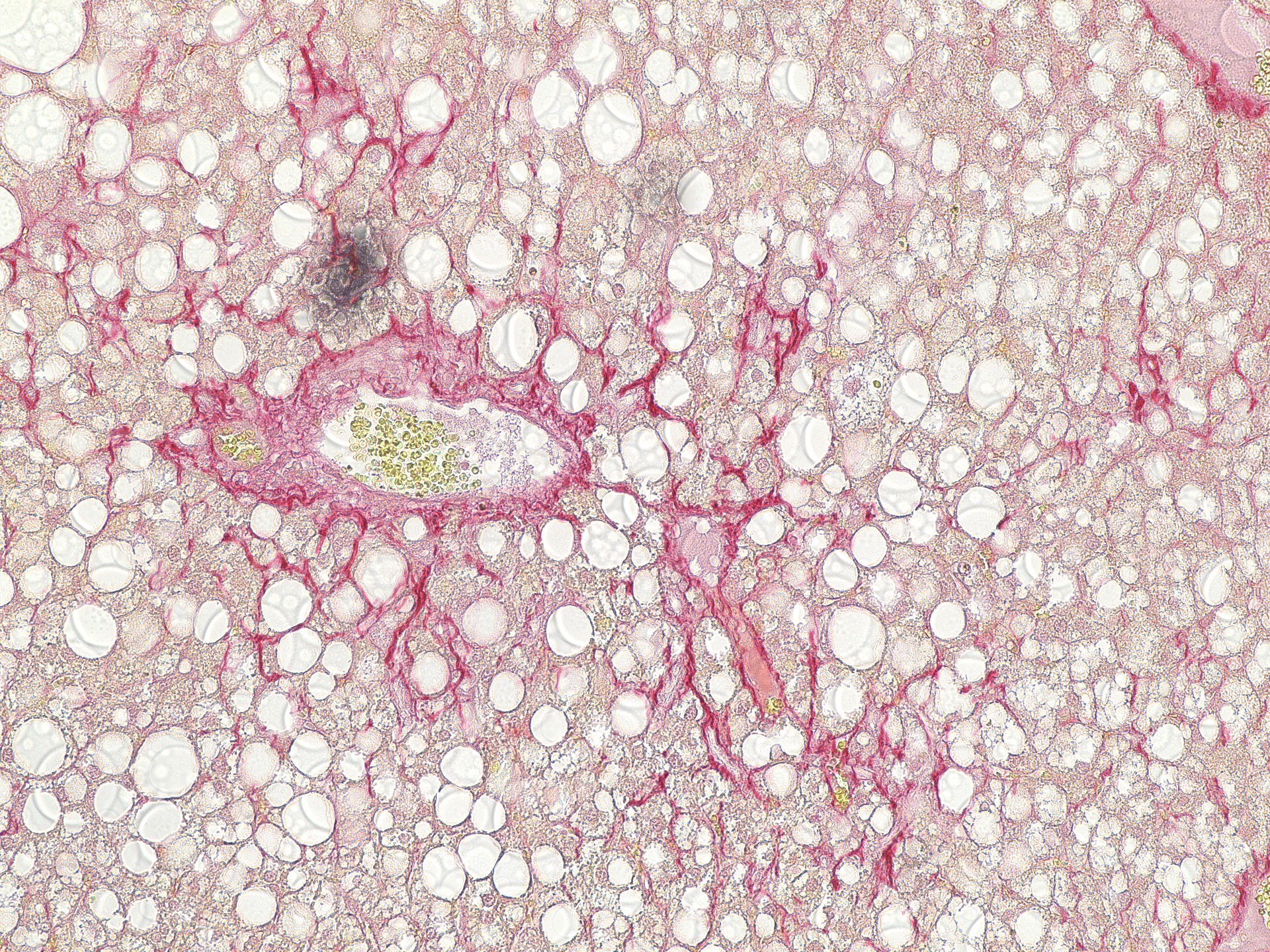

Supplement: Supplementary file 9 — Figure EV3 Source Data [file 44318_2024_196_MOESM9_ESM.zip › Figure EV3/Figure EV3-L/Quantificated image/HFD Con/no.4/Liver-HFD con-no.4-20x-3.jpg]

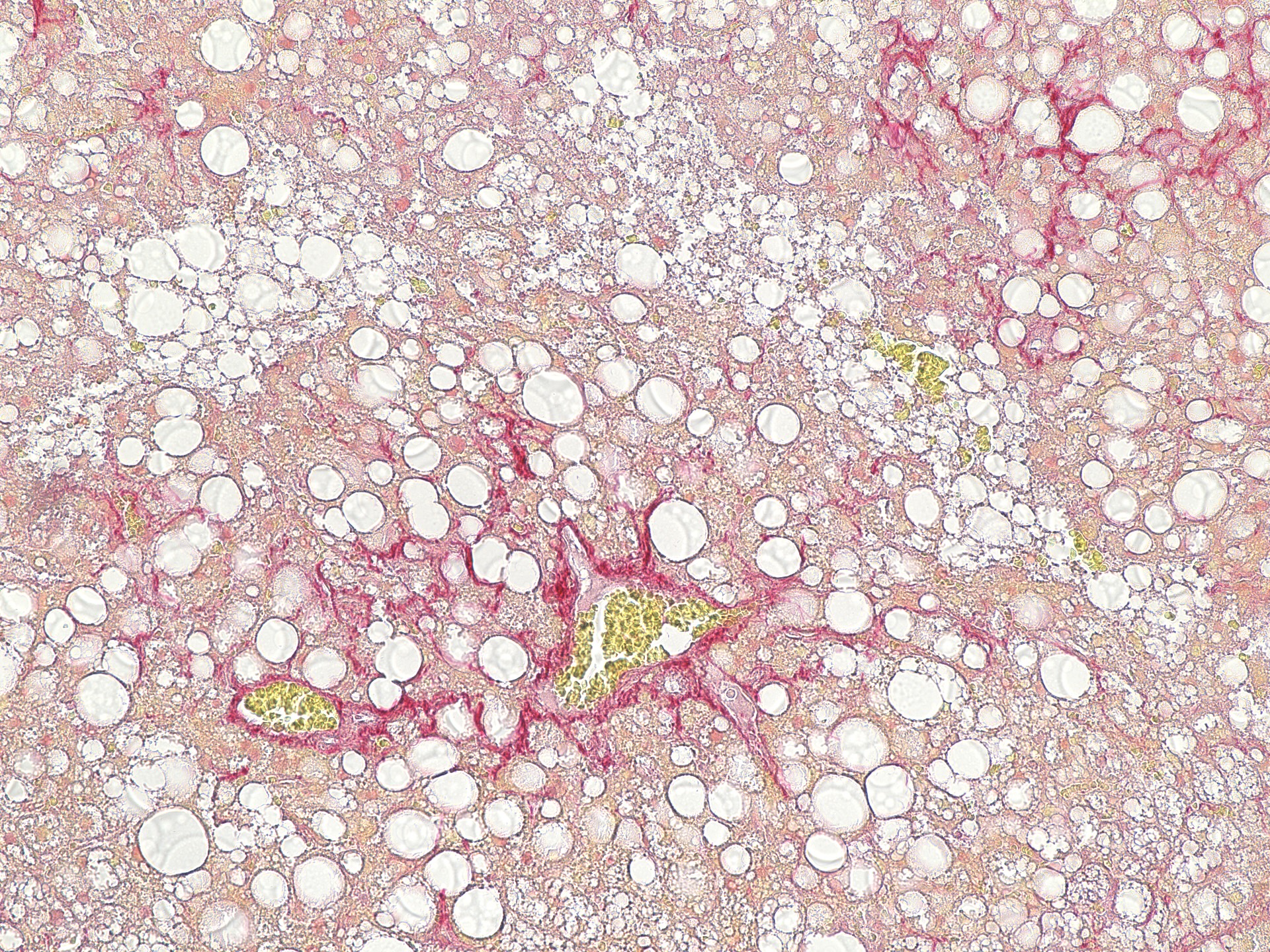

Supplement: Supplementary file 9 — Figure EV3 Source Data [file 44318_2024_196_MOESM9_ESM.zip › Figure EV3/Figure EV3-L/Quantificated image/HFD Con/no.4/Liver-HFD con-no.4-20x-2.jpg]

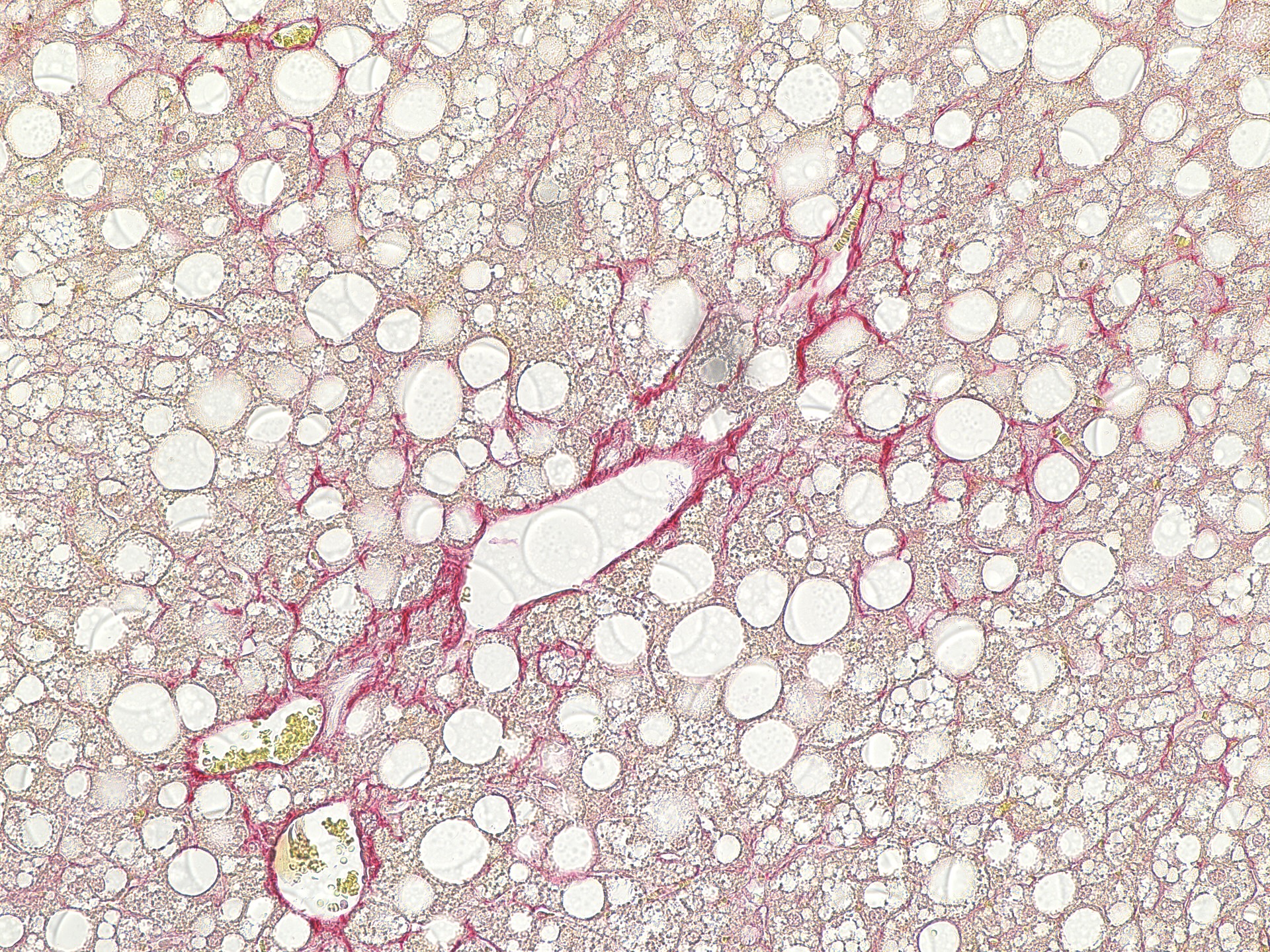

Supplement: Supplementary file 9 — Figure EV3 Source Data [file 44318_2024_196_MOESM9_ESM.zip › Figure EV3/Figure EV3-L/Quantificated image/HFD Con/no.4/Liver-HFD con-no.4-20x-5.jpg]

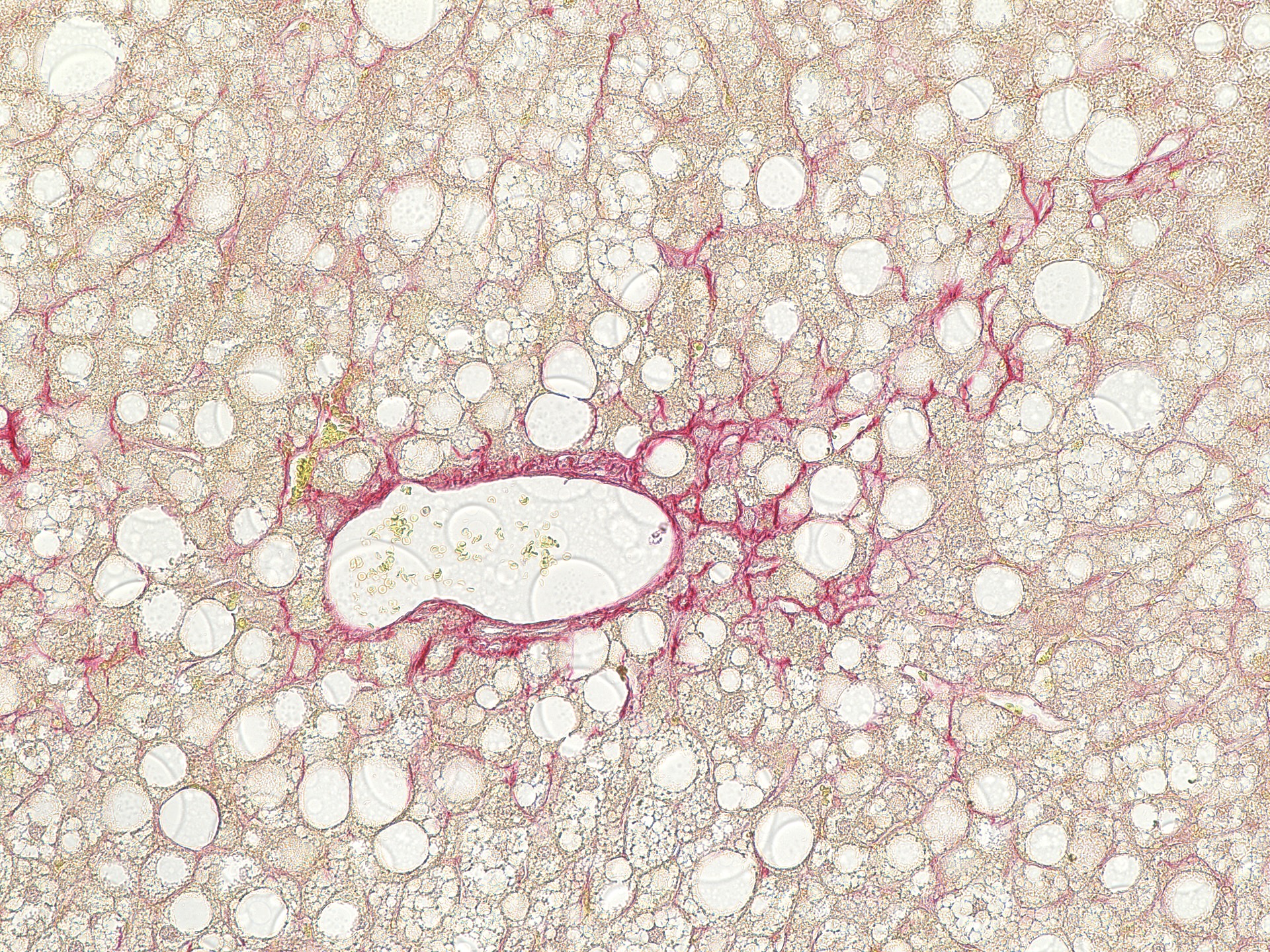

Supplement: Supplementary file 9 — Figure EV3 Source Data [file 44318_2024_196_MOESM9_ESM.zip › Figure EV3/Figure EV3-L/Quantificated image/HFD Con/no.4/Liver-HFD con-no.4-20x-4.jpg]

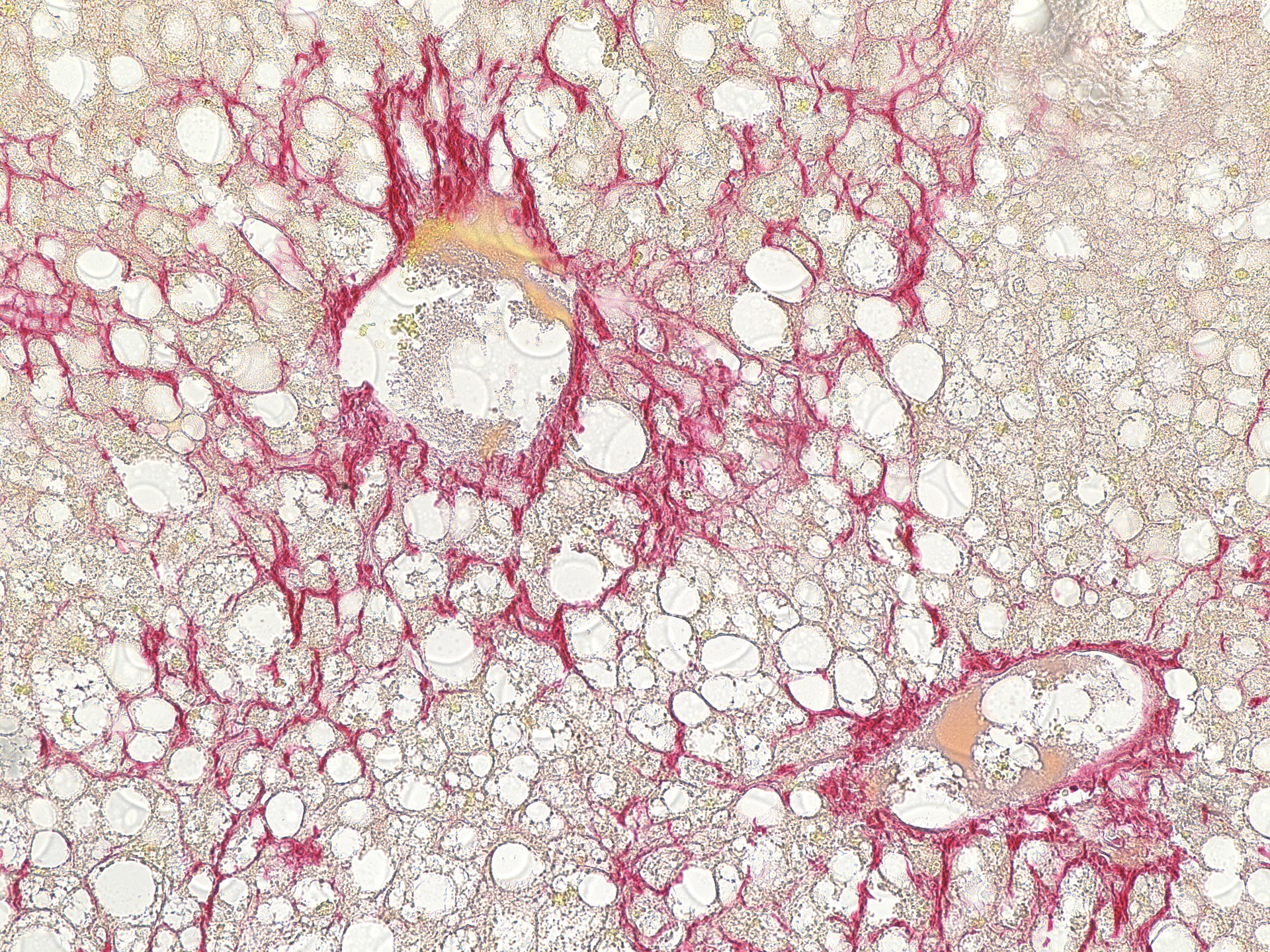

Supplement: Supplementary file 9 — Figure EV3 Source Data [file 44318_2024_196_MOESM9_ESM.zip › Figure EV3/Figure EV3-L/Quantificated image/HFD Con/no.2/Liver-HFD con-no.2-20x-4.jpg]

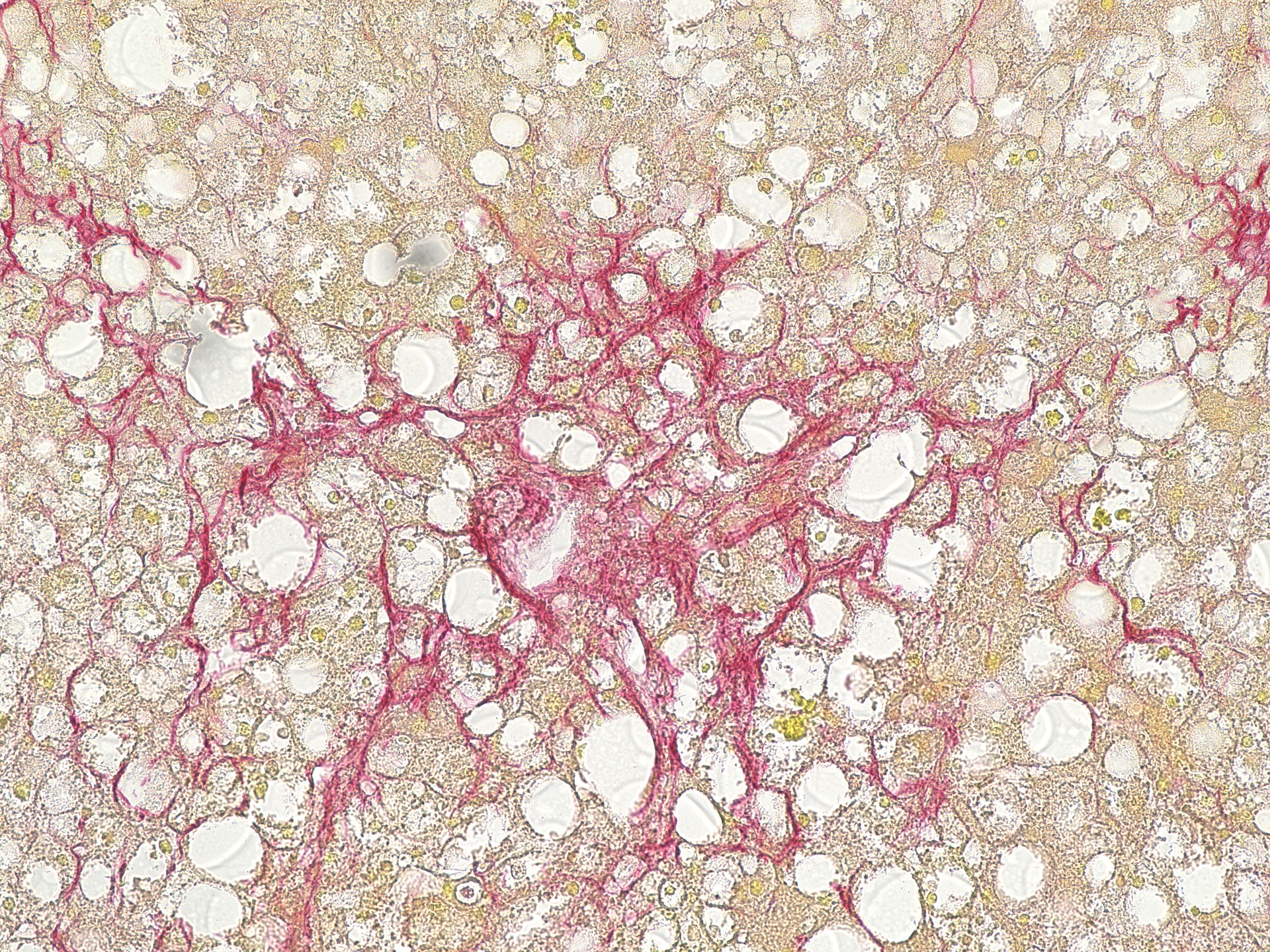

Supplement: Supplementary file 9 — Figure EV3 Source Data [file 44318_2024_196_MOESM9_ESM.zip › Figure EV3/Figure EV3-L/Quantificated image/HFD Con/no.2/Liver-HFD con-no.2-20x-5.jpg]

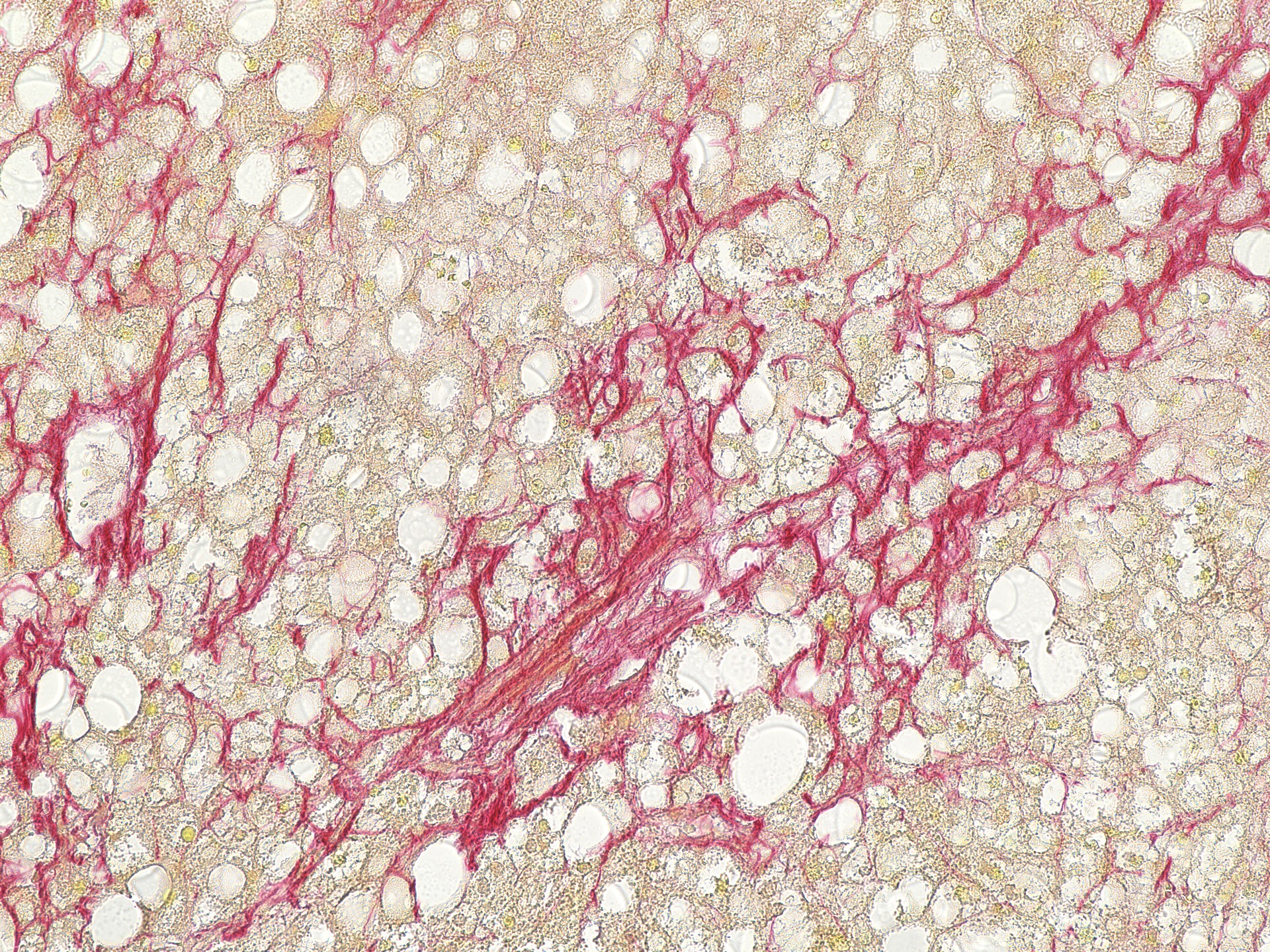

Supplement: Supplementary file 9 — Figure EV3 Source Data [file 44318_2024_196_MOESM9_ESM.zip › Figure EV3/Figure EV3-L/Quantificated image/HFD Con/no.2/Liver-HFD con-no.2-20x-1.jpg]

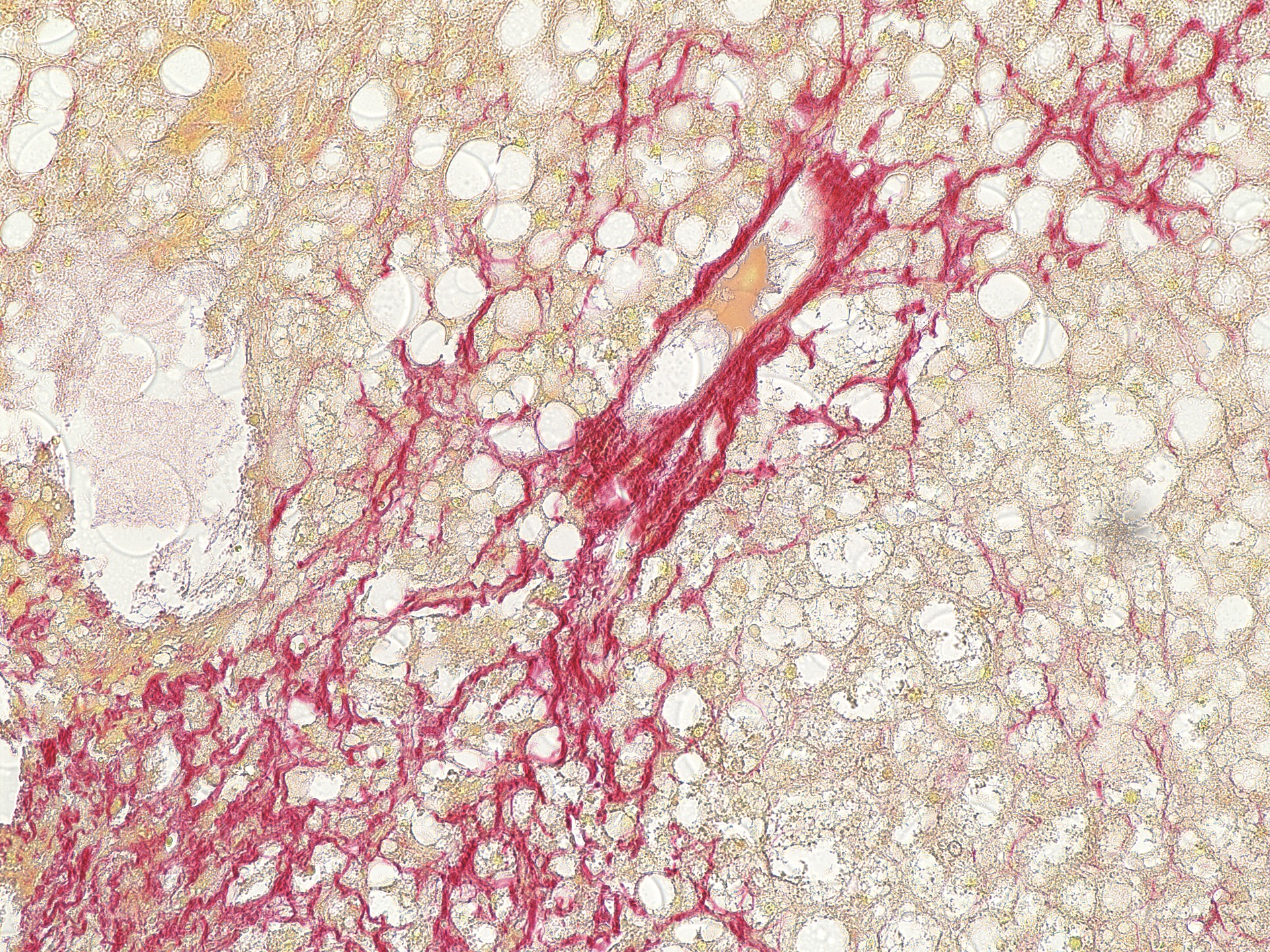

Supplement: Supplementary file 9 — Figure EV3 Source Data [file 44318_2024_196_MOESM9_ESM.zip › Figure EV3/Figure EV3-L/Quantificated image/HFD Con/no.2/Liver-HFD con-no.2-20x-2.jpg]

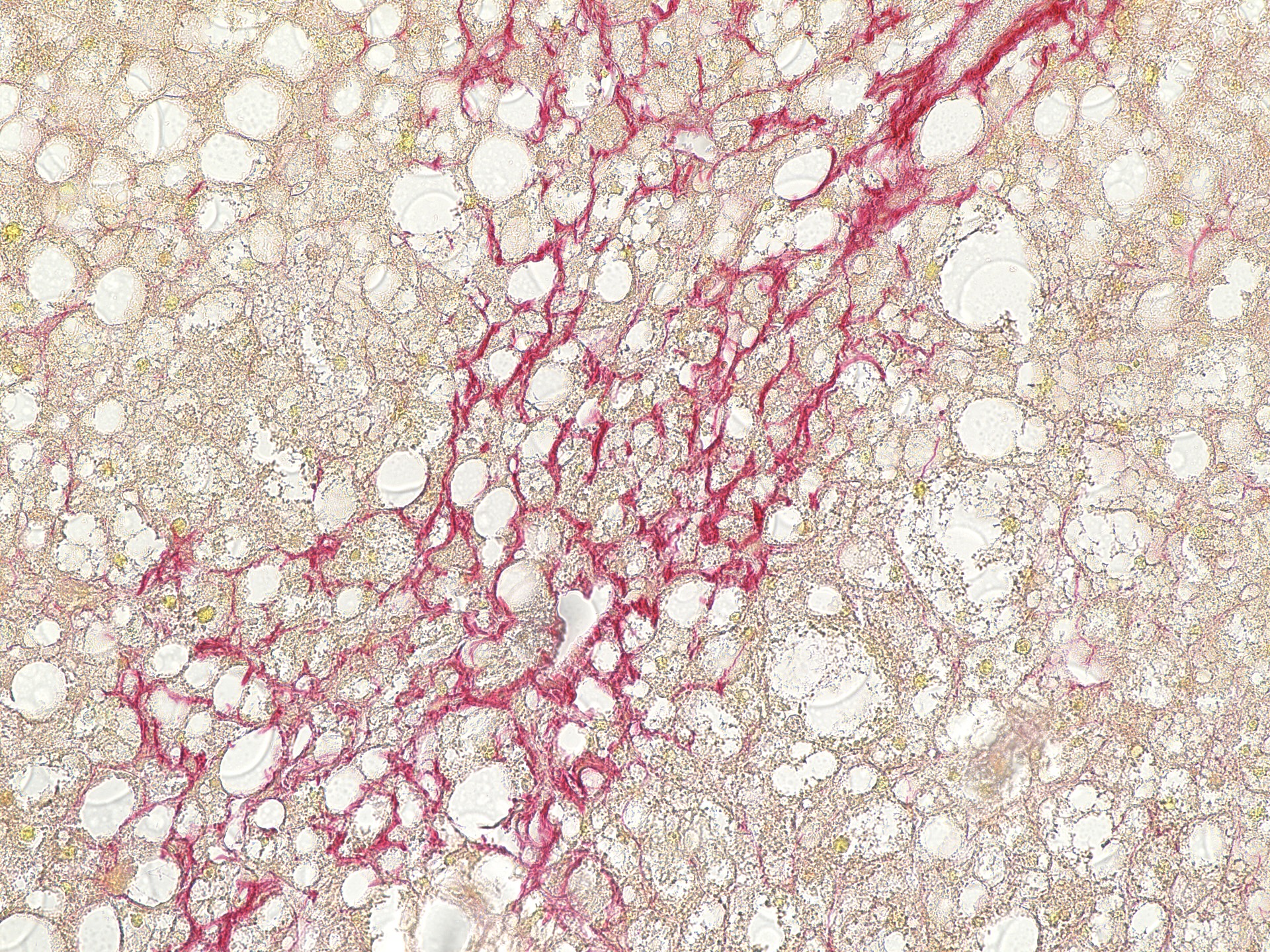

Supplement: Supplementary file 9 — Figure EV3 Source Data [file 44318_2024_196_MOESM9_ESM.zip › Figure EV3/Figure EV3-L/Quantificated image/HFD Con/no.2/Liver-HFD con-no.2-20x-3.jpg]

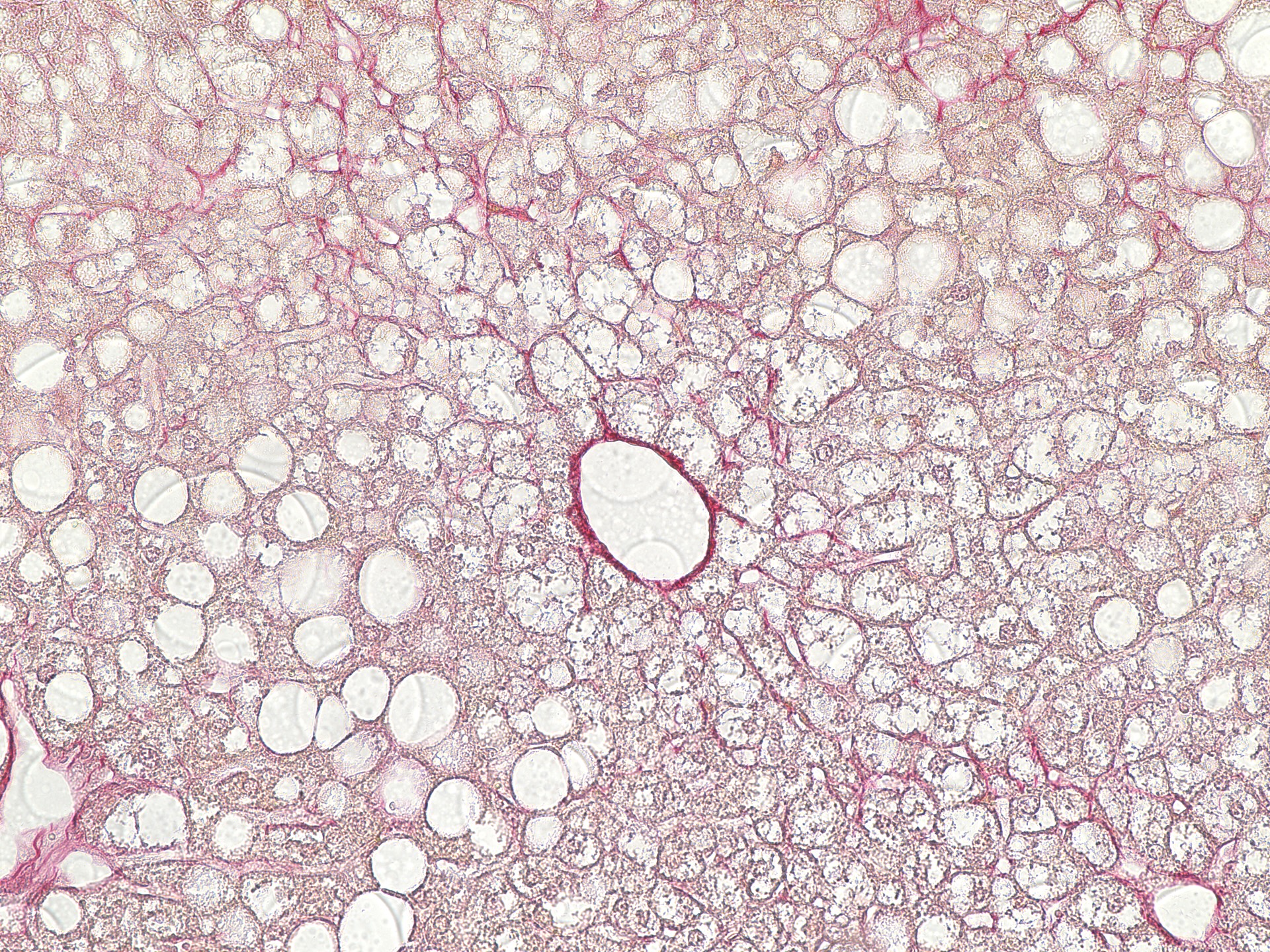

Supplement: Supplementary file 9 — Figure EV3 Source Data [file 44318_2024_196_MOESM9_ESM.zip › Figure EV3/Figure EV3-L/Quantificated image/HFD PCPE-1 vaccine/no.1/Liver-HFD vaccine-no.1-20x-2.jpg]

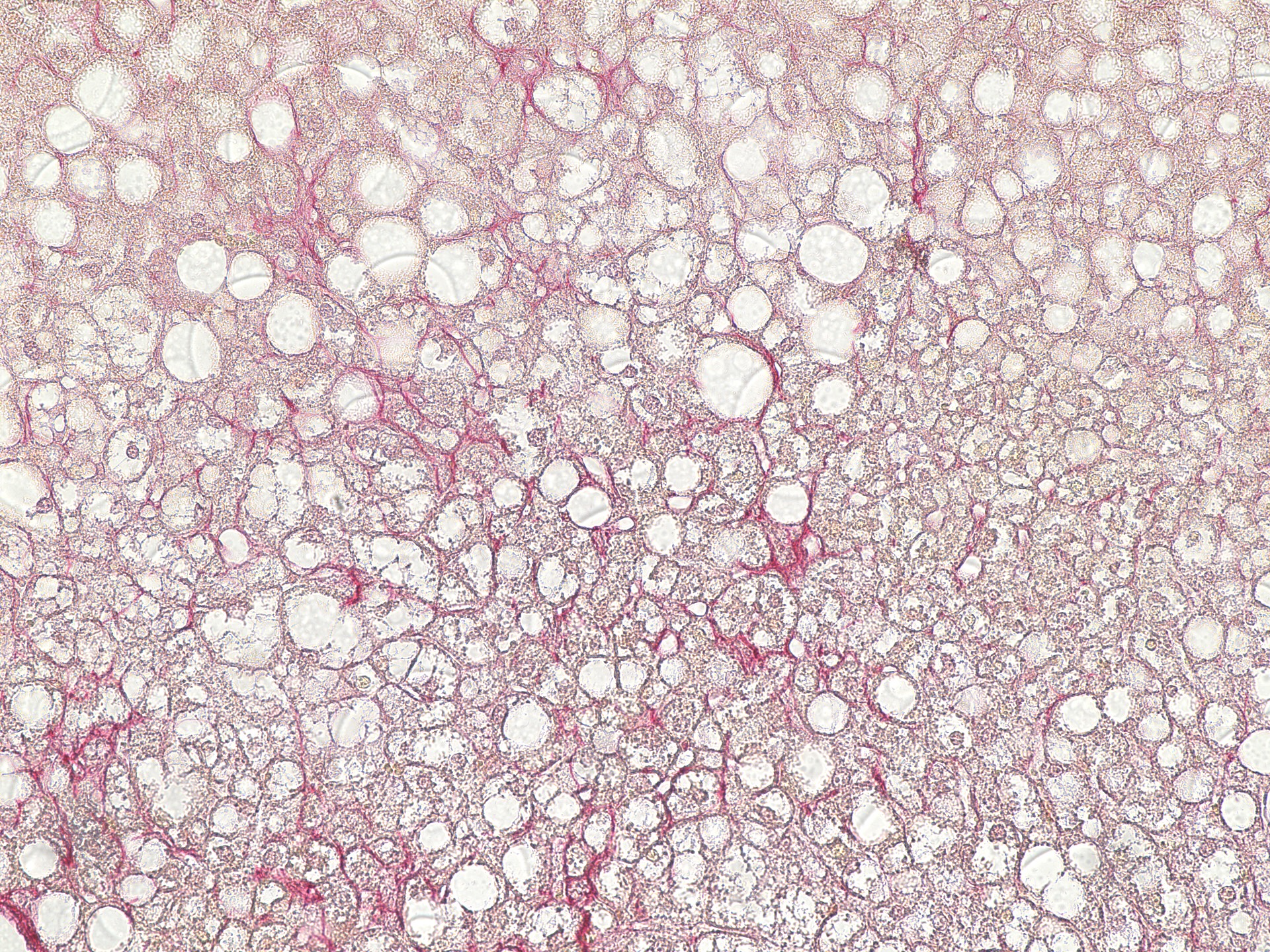

Supplement: Supplementary file 9 — Figure EV3 Source Data [file 44318_2024_196_MOESM9_ESM.zip › Figure EV3/Figure EV3-L/Quantificated image/HFD PCPE-1 vaccine/no.1/Liver-HFD vaccine-no.1-20x-3.jpg]

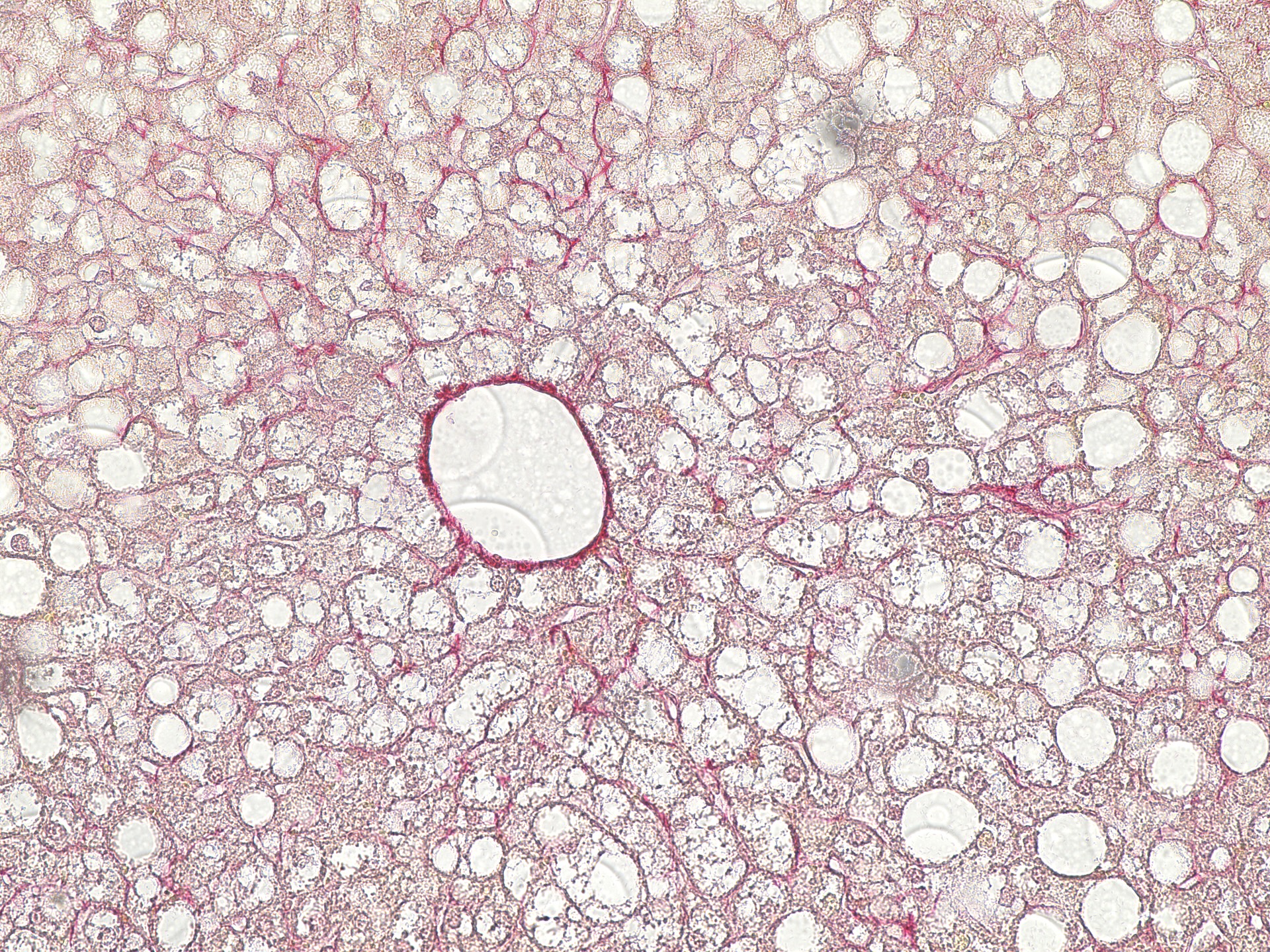

Supplement: Supplementary file 9 — Figure EV3 Source Data [file 44318_2024_196_MOESM9_ESM.zip › Figure EV3/Figure EV3-L/Quantificated image/HFD PCPE-1 vaccine/no.1/Liver-HFD vaccine-no.1-20x-1.jpg]

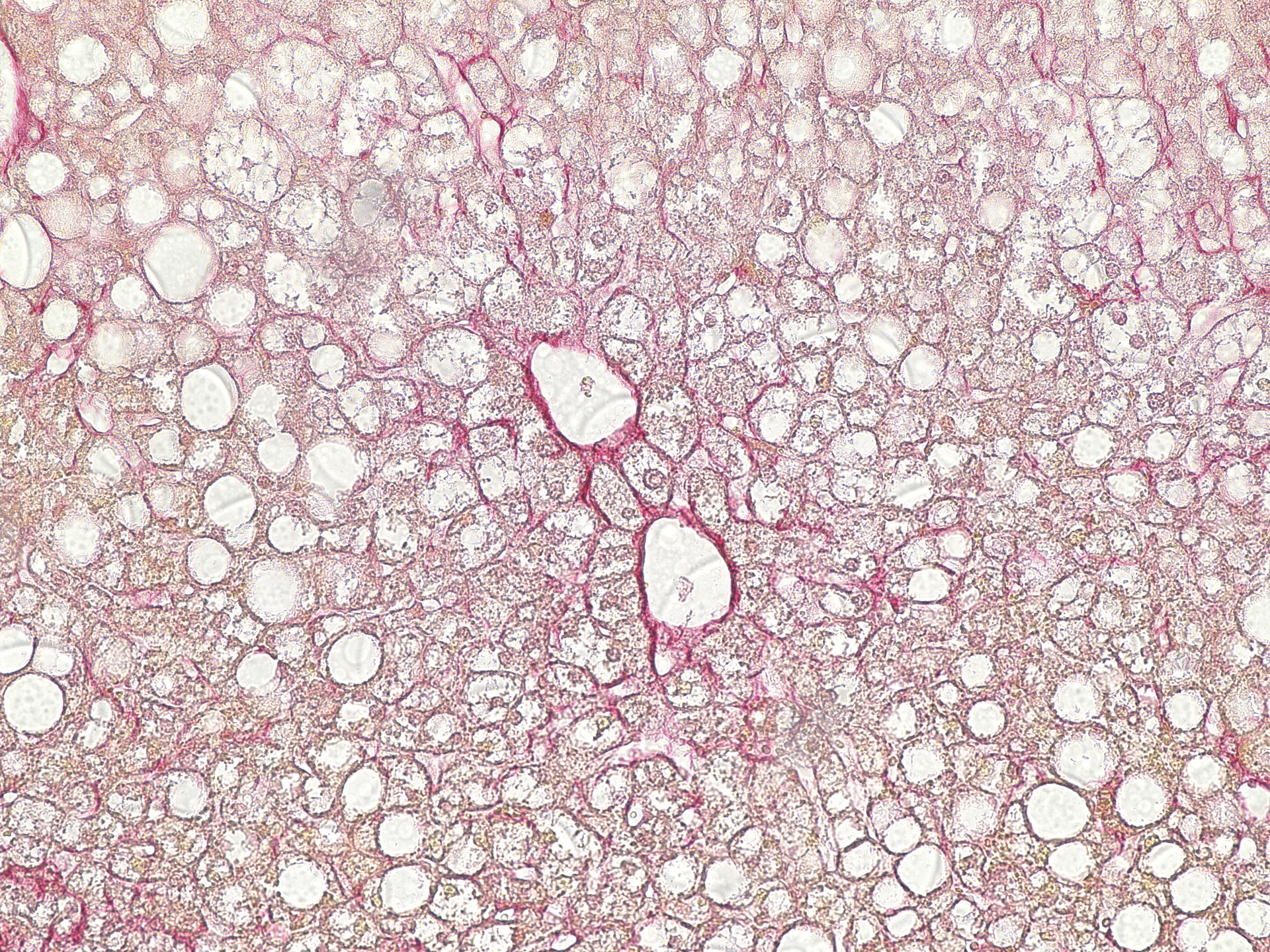

Supplement: Supplementary file 9 — Figure EV3 Source Data [file 44318_2024_196_MOESM9_ESM.zip › Figure EV3/Figure EV3-L/Quantificated image/HFD PCPE-1 vaccine/no.1/Liver-HFD vaccine-no.1-20x-4.jpg]

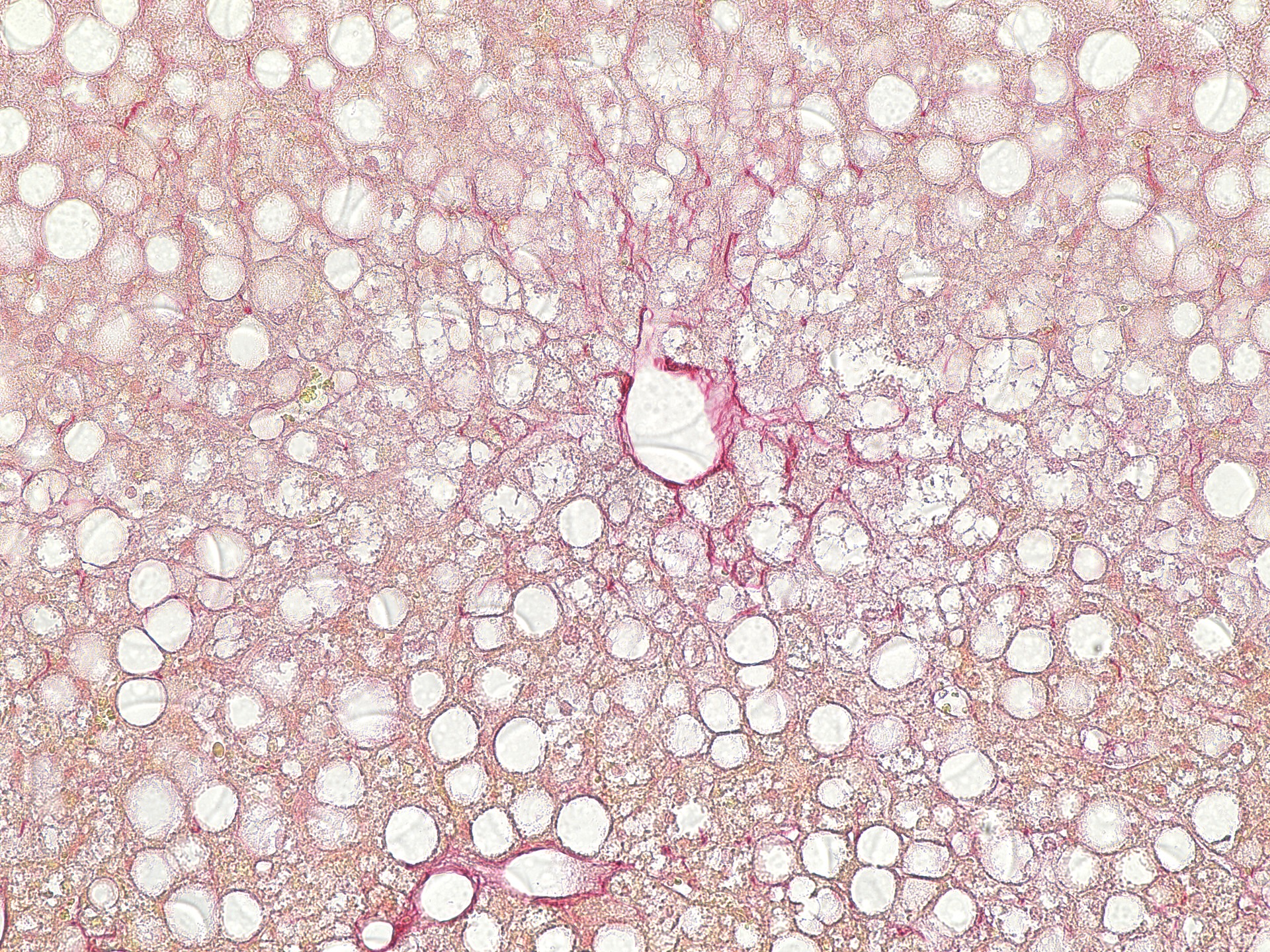

Supplement: Supplementary file 9 — Figure EV3 Source Data [file 44318_2024_196_MOESM9_ESM.zip › Figure EV3/Figure EV3-L/Quantificated image/HFD PCPE-1 vaccine/no.1/Liver-HFD vaccine-no.1-20x-5.jpg]

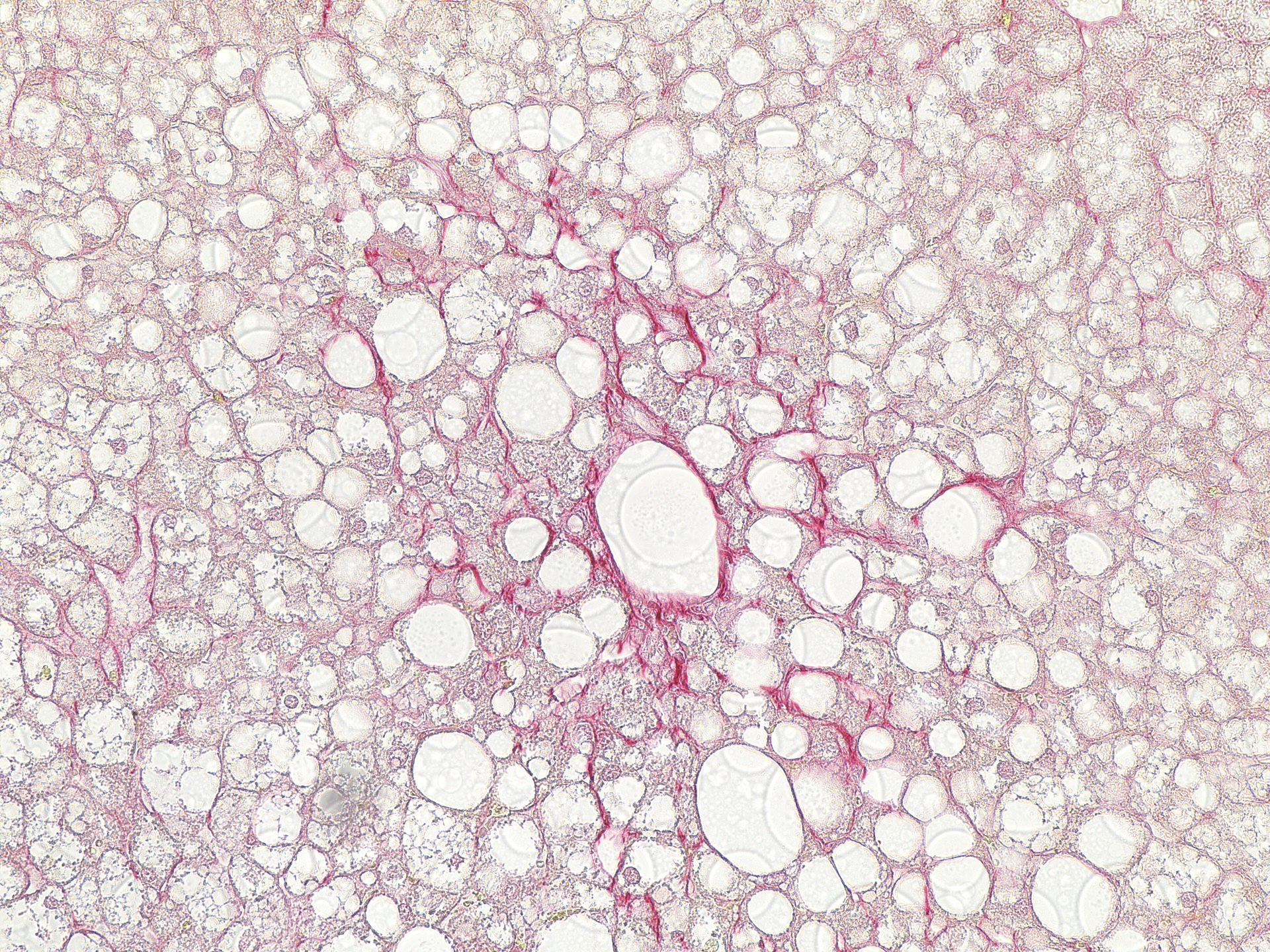

Supplement: Supplementary file 9 — Figure EV3 Source Data [file 44318_2024_196_MOESM9_ESM.zip › Figure EV3/Figure EV3-L/Quantificated image/HFD PCPE-1 vaccine/no.3/Liver-HFD vaccine-no.3-20x-3.jpg]

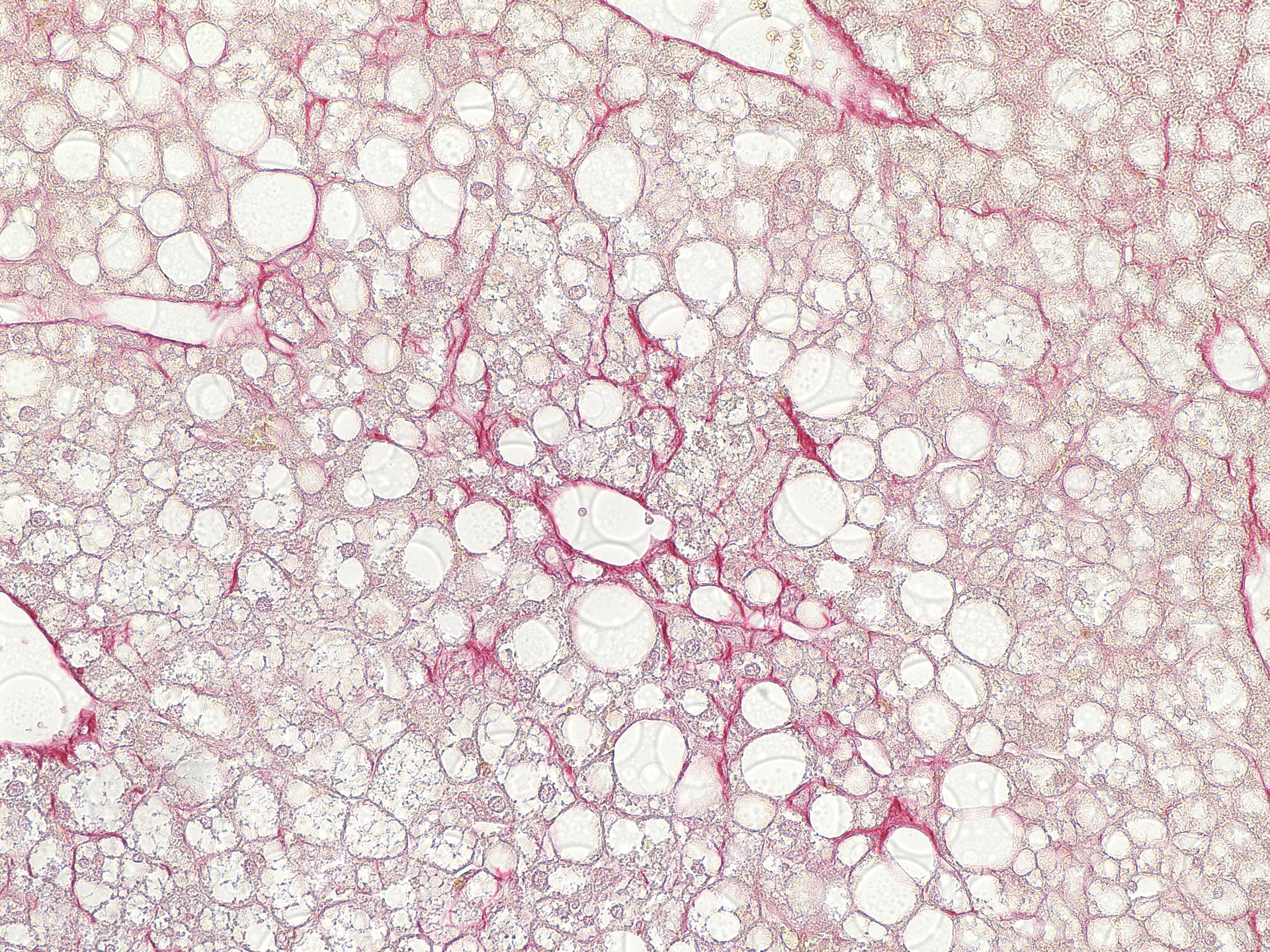

Supplement: Supplementary file 9 — Figure EV3 Source Data [file 44318_2024_196_MOESM9_ESM.zip › Figure EV3/Figure EV3-L/Quantificated image/HFD PCPE-1 vaccine/no.3/Liver-HFD vaccine-no.3-20x-2.jpg]

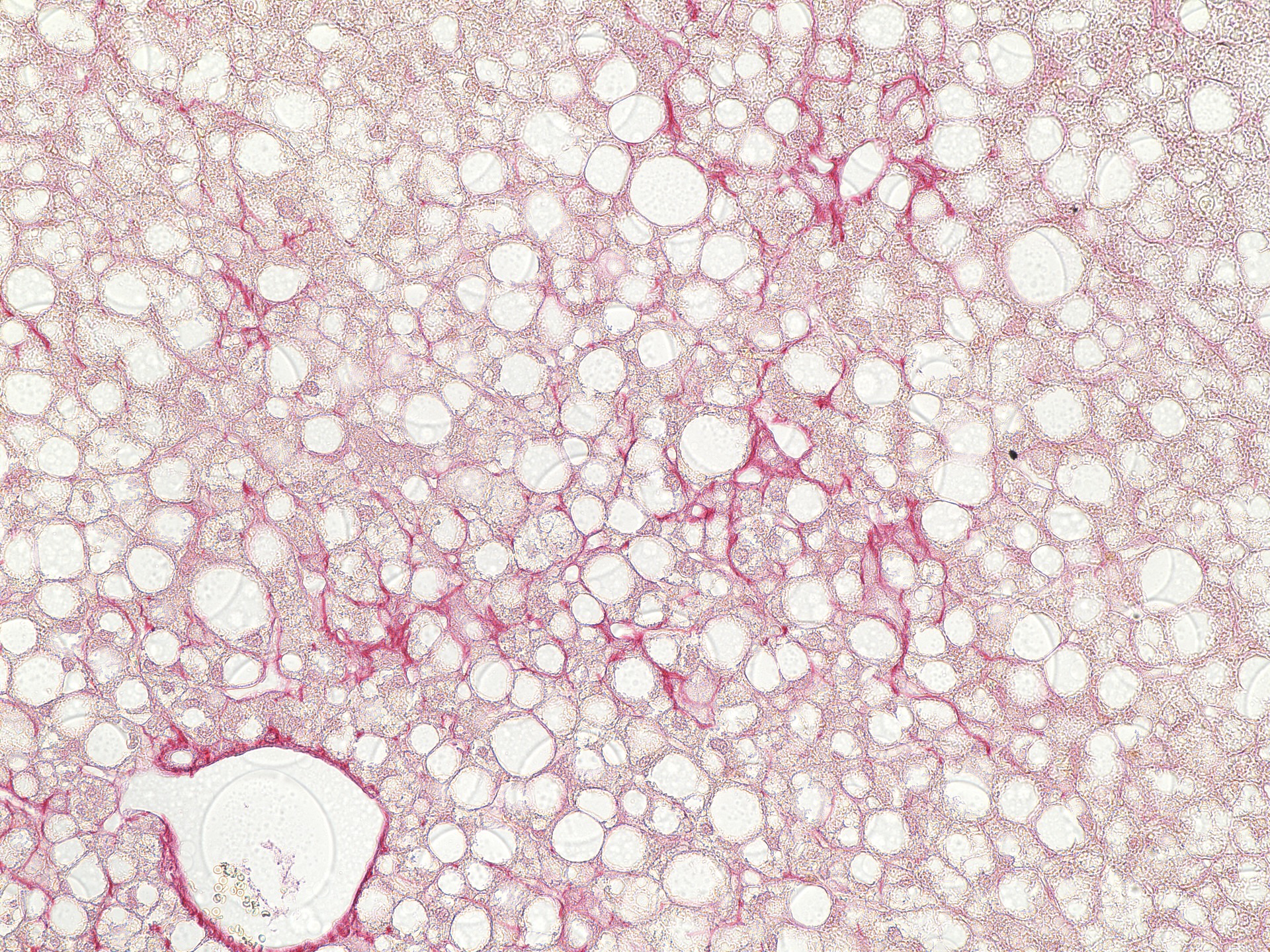

Supplement: Supplementary file 9 — Figure EV3 Source Data [file 44318_2024_196_MOESM9_ESM.zip › Figure EV3/Figure EV3-L/Quantificated image/HFD PCPE-1 vaccine/no.3/Liver-HFD vaccine-no.3-20x-1.jpg]

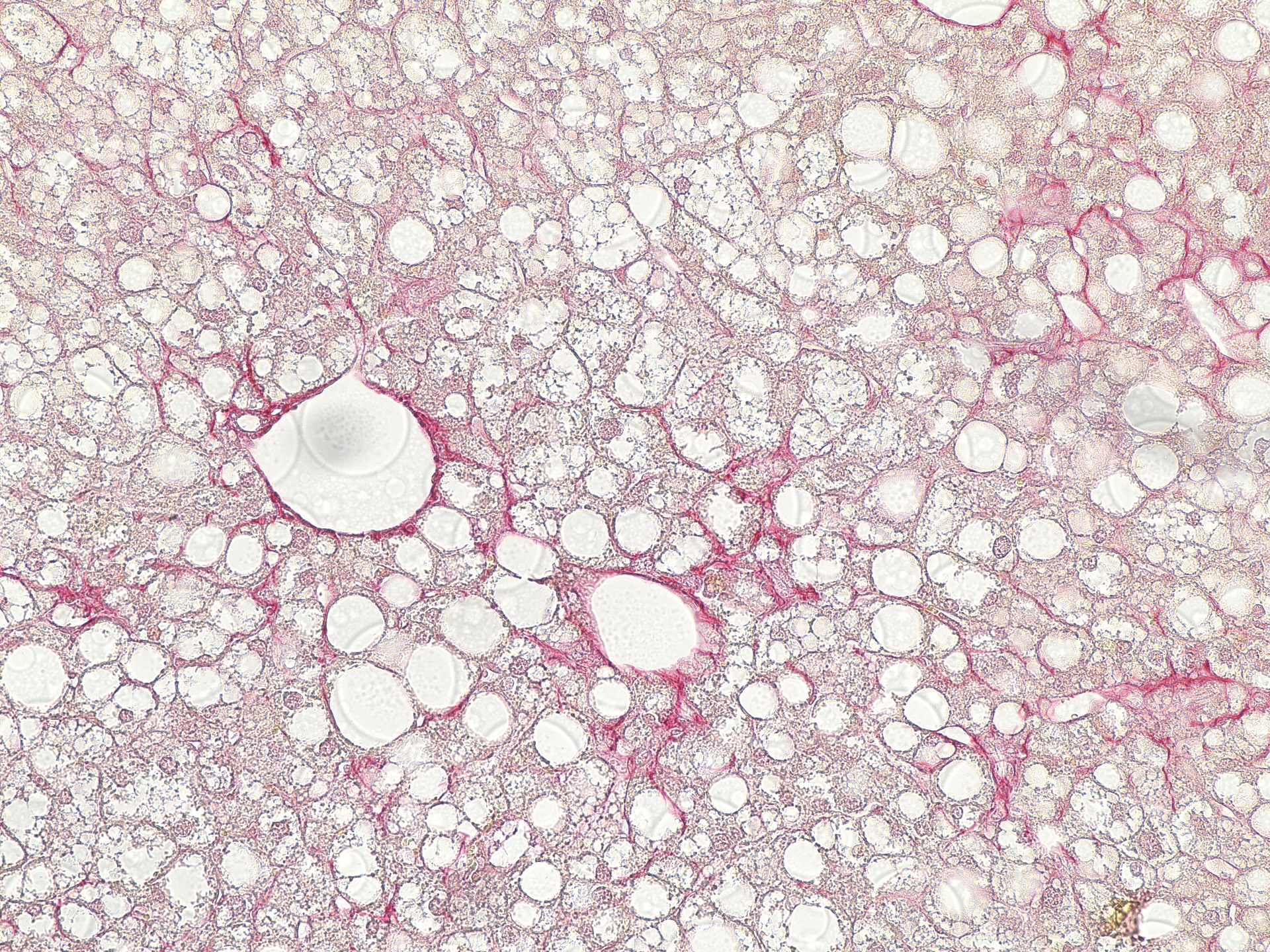

Supplement: Supplementary file 9 — Figure EV3 Source Data [file 44318_2024_196_MOESM9_ESM.zip › Figure EV3/Figure EV3-L/Quantificated image/HFD PCPE-1 vaccine/no.3/Liver-HFD vaccine-no.3-20x-5.jpg]

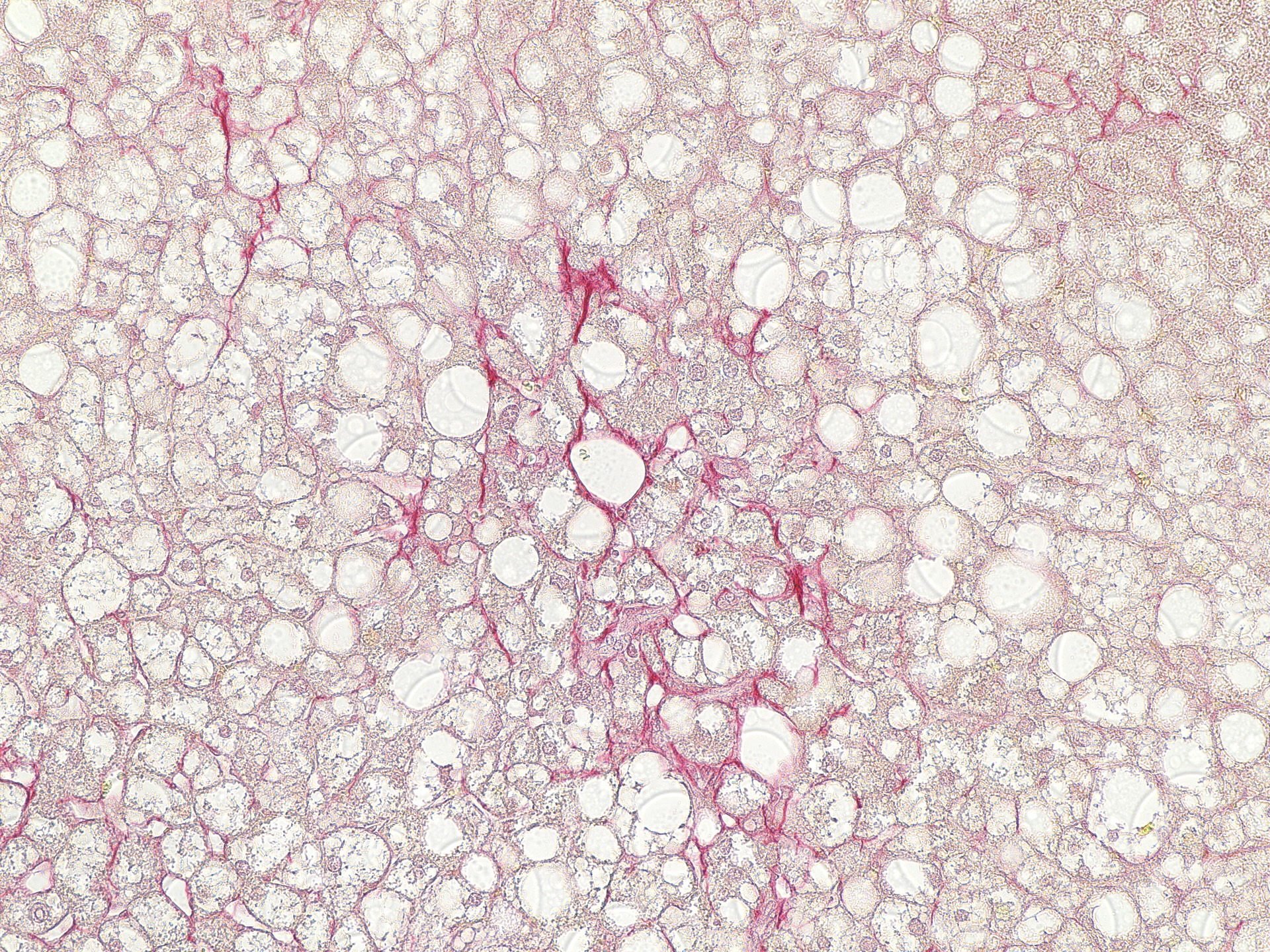

Supplement: Supplementary file 9 — Figure EV3 Source Data [file 44318_2024_196_MOESM9_ESM.zip › Figure EV3/Figure EV3-L/Quantificated image/HFD PCPE-1 vaccine/no.3/Liver-HFD vaccine-no.3-20x-4.jpg]

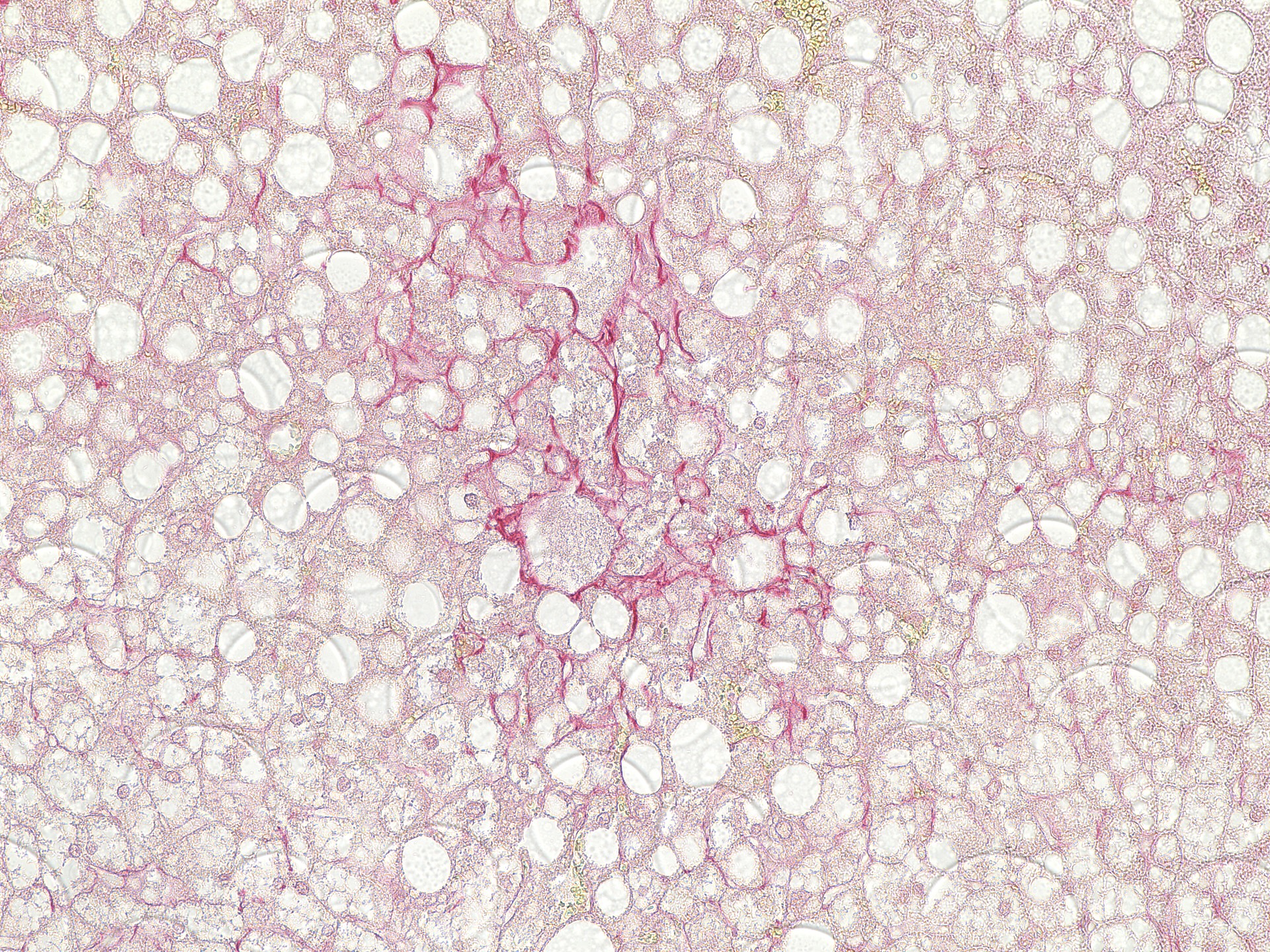

Supplement: Supplementary file 9 — Figure EV3 Source Data [file 44318_2024_196_MOESM9_ESM.zip › Figure EV3/Figure EV3-L/Quantificated image/HFD PCPE-1 vaccine/no.4/Liver-HFD vaccine-no.4-20x-4.jpg]

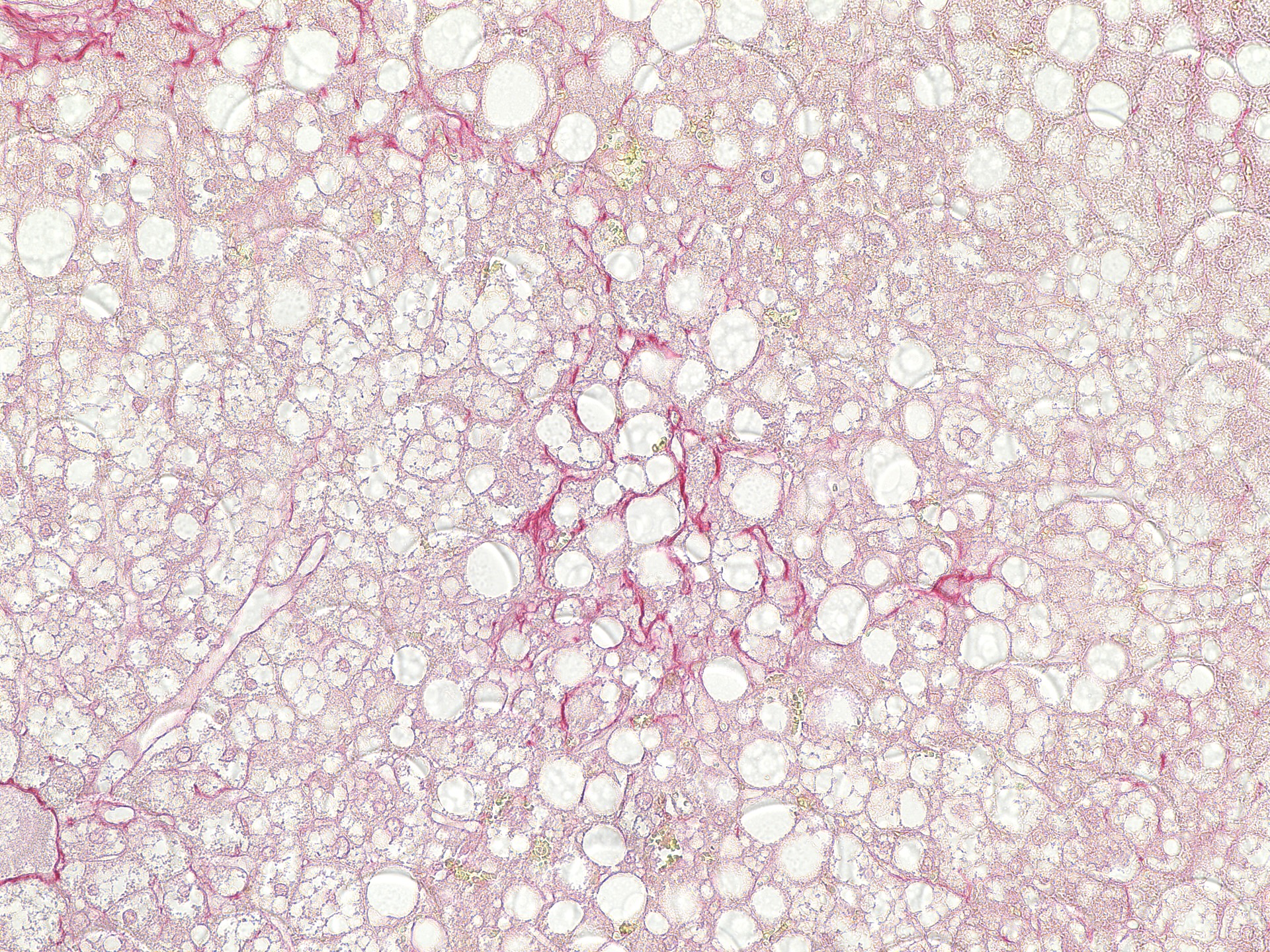

Supplement: Supplementary file 9 — Figure EV3 Source Data [file 44318_2024_196_MOESM9_ESM.zip › Figure EV3/Figure EV3-L/Quantificated image/HFD PCPE-1 vaccine/no.4/Liver-HFD vaccine-no.4-20x-5.jpg]

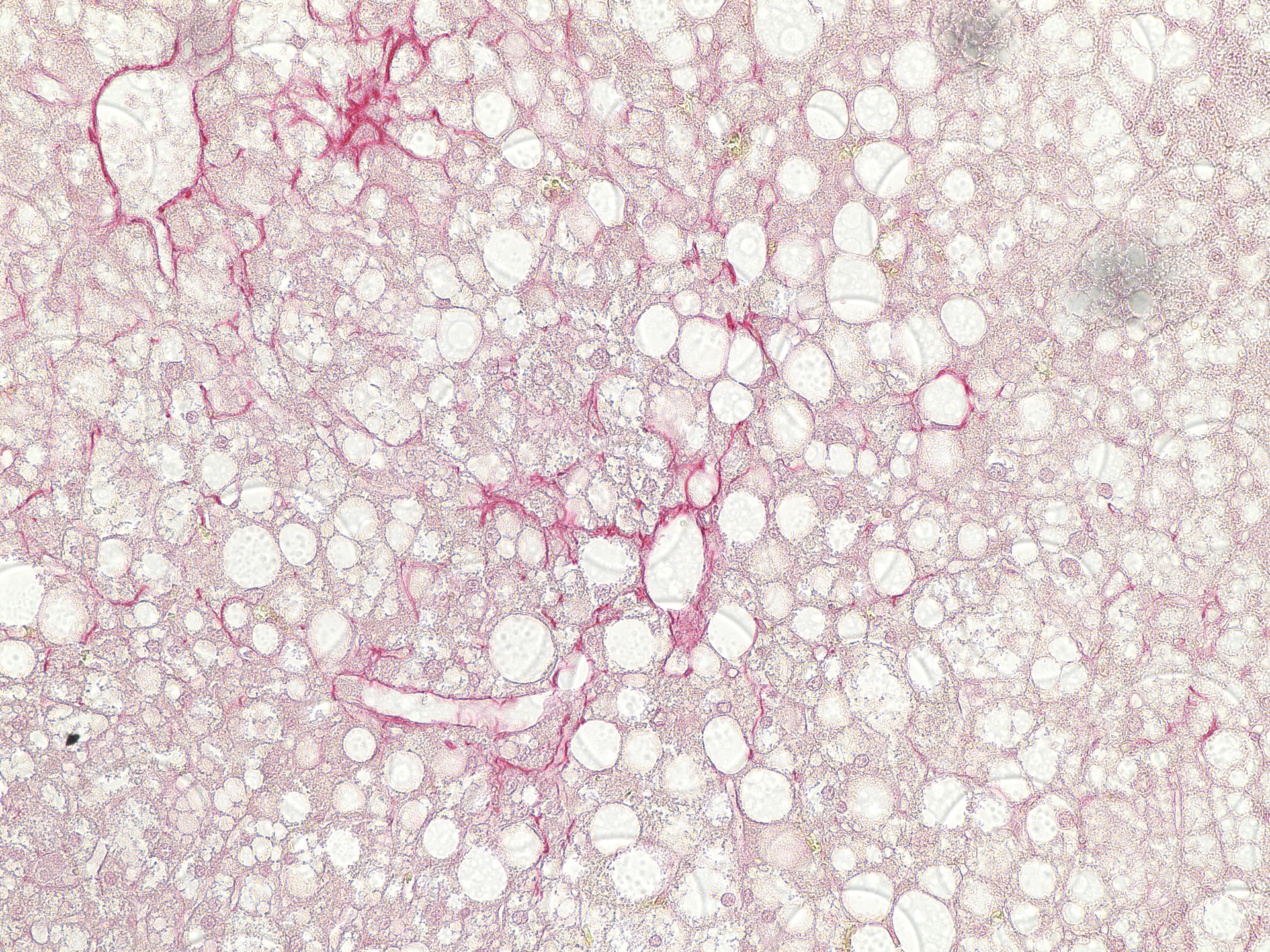

Supplement: Supplementary file 9 — Figure EV3 Source Data [file 44318_2024_196_MOESM9_ESM.zip › Figure EV3/Figure EV3-L/Quantificated image/HFD PCPE-1 vaccine/no.4/Liver-HFD vaccine-no.4-20x-2.jpg]

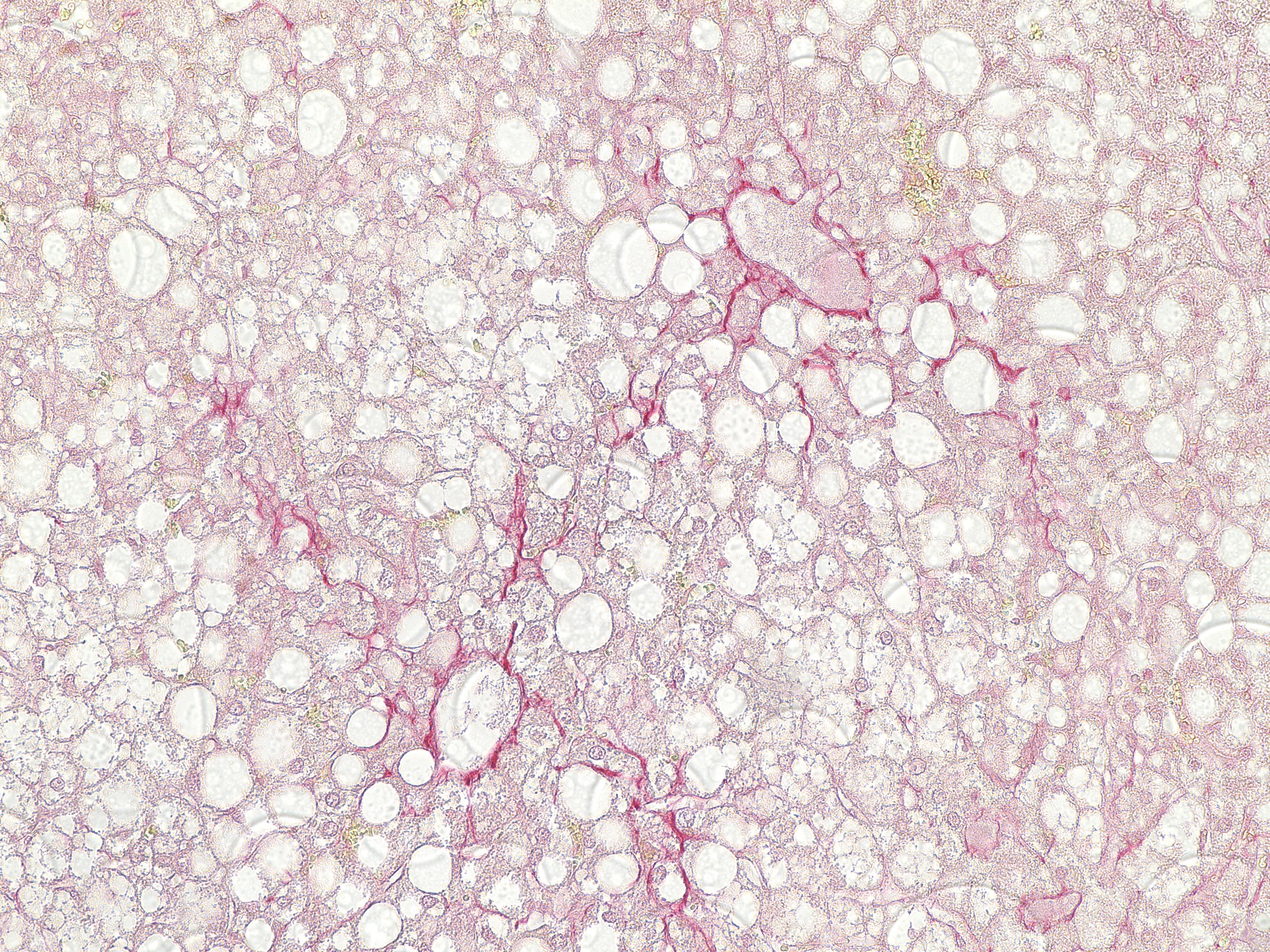

Supplement: Supplementary file 9 — Figure EV3 Source Data [file 44318_2024_196_MOESM9_ESM.zip › Figure EV3/Figure EV3-L/Quantificated image/HFD PCPE-1 vaccine/no.4/Liver-HFD vaccine-no.4-20x-3.jpg]

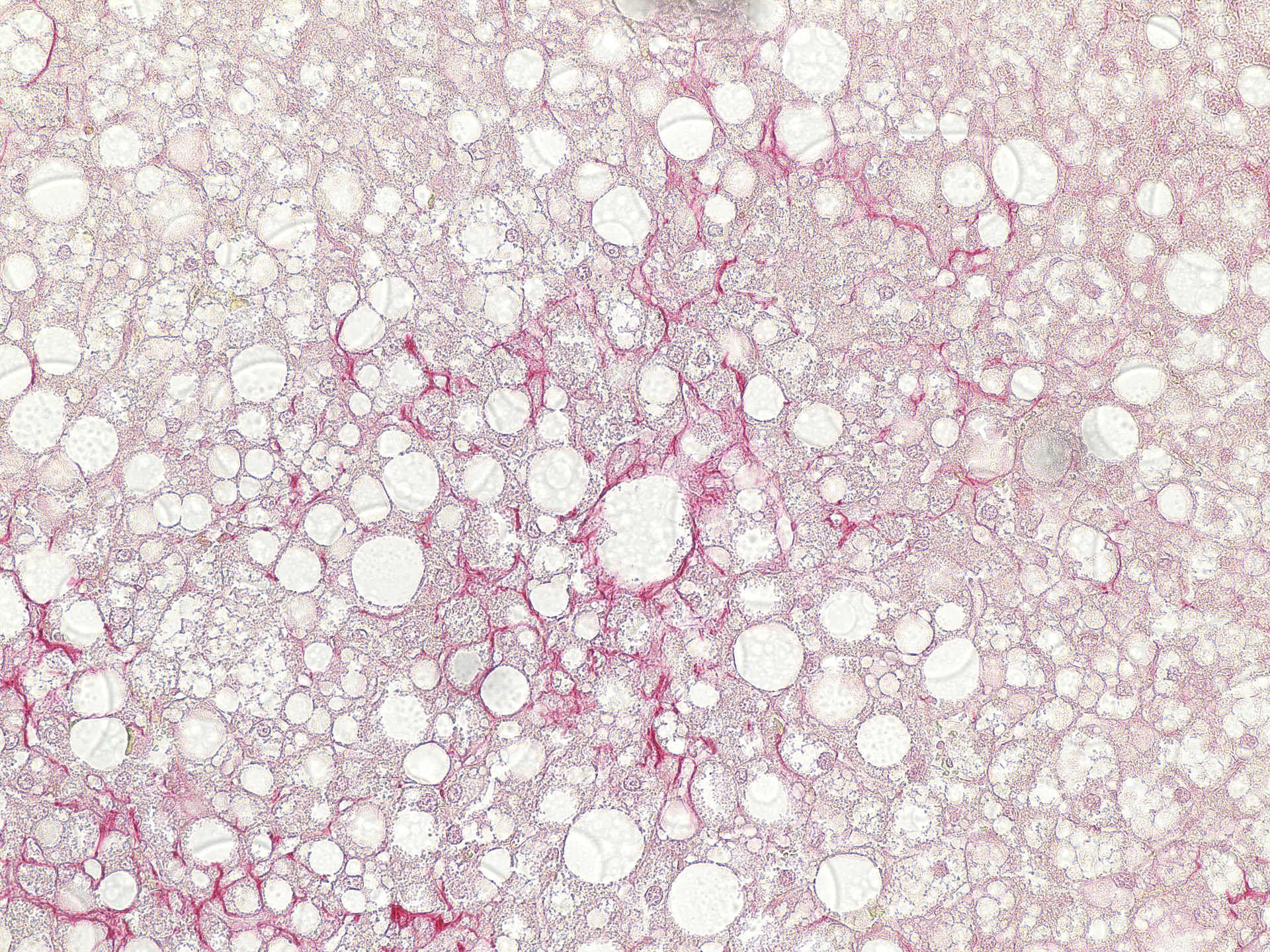

Supplement: Supplementary file 9 — Figure EV3 Source Data [file 44318_2024_196_MOESM9_ESM.zip › Figure EV3/Figure EV3-L/Quantificated image/HFD PCPE-1 vaccine/no.4/Liver-HFD vaccine-no.4-20x-1.jpg]

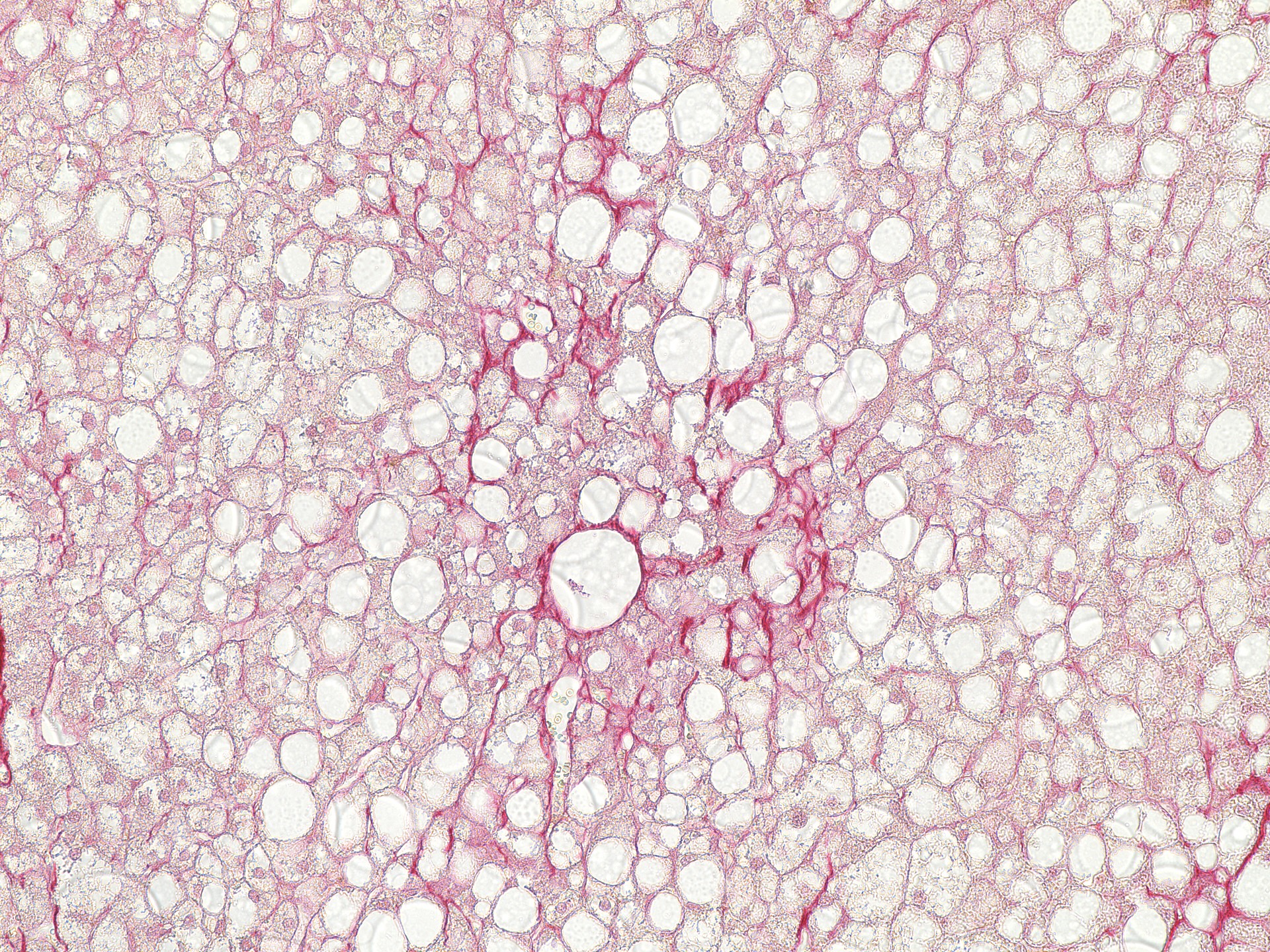

Supplement: Supplementary file 9 — Figure EV3 Source Data [file 44318_2024_196_MOESM9_ESM.zip › Figure EV3/Figure EV3-L/Quantificated image/HFD PCPE-1 vaccine/no.2/Liver-HFD vaccine-no.2-20x-3.jpg]

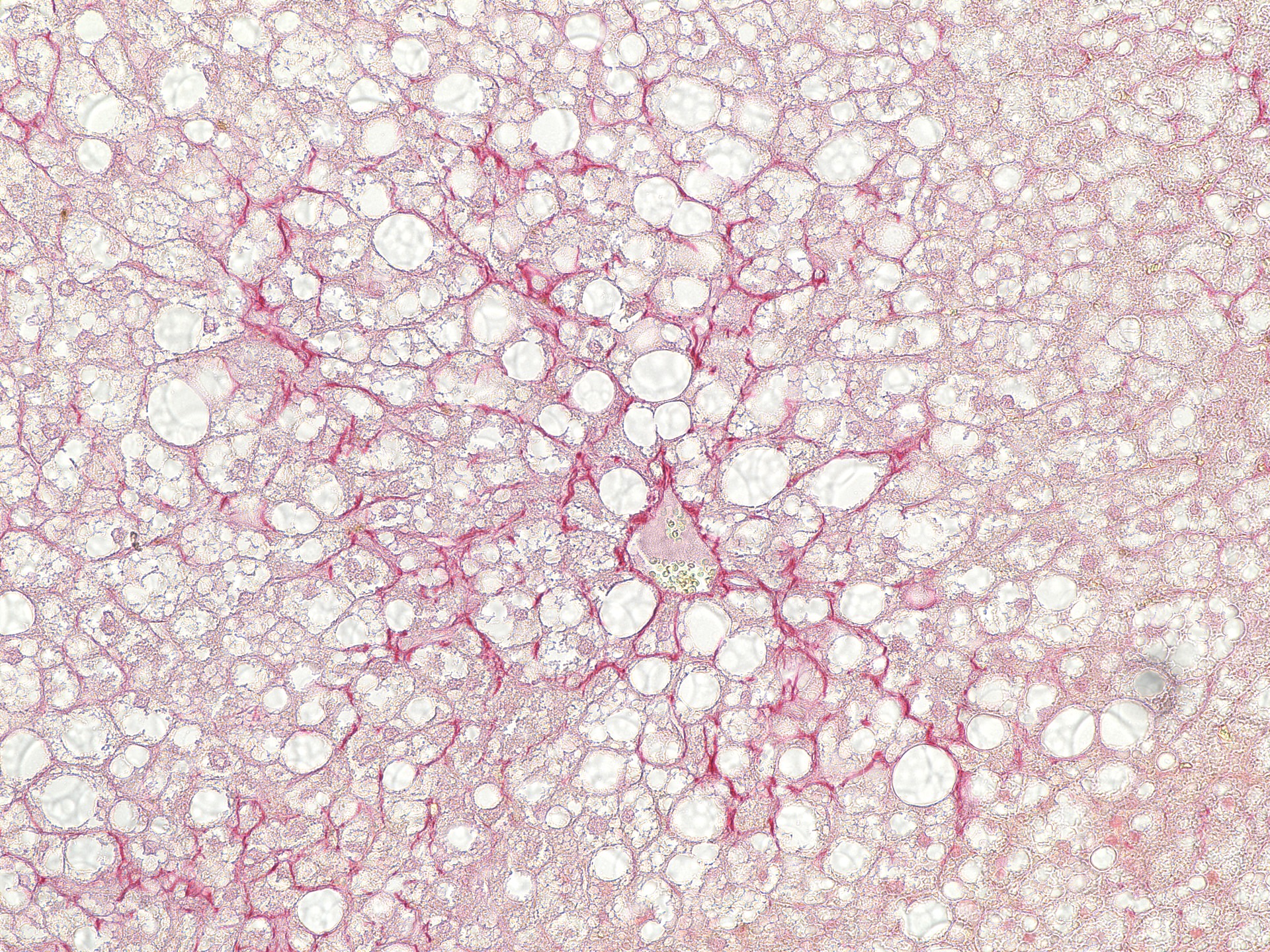

Supplement: Supplementary file 9 — Figure EV3 Source Data [file 44318_2024_196_MOESM9_ESM.zip › Figure EV3/Figure EV3-L/Quantificated image/HFD PCPE-1 vaccine/no.2/Liver-HFD vaccine-no.2-20x-2.jpg]

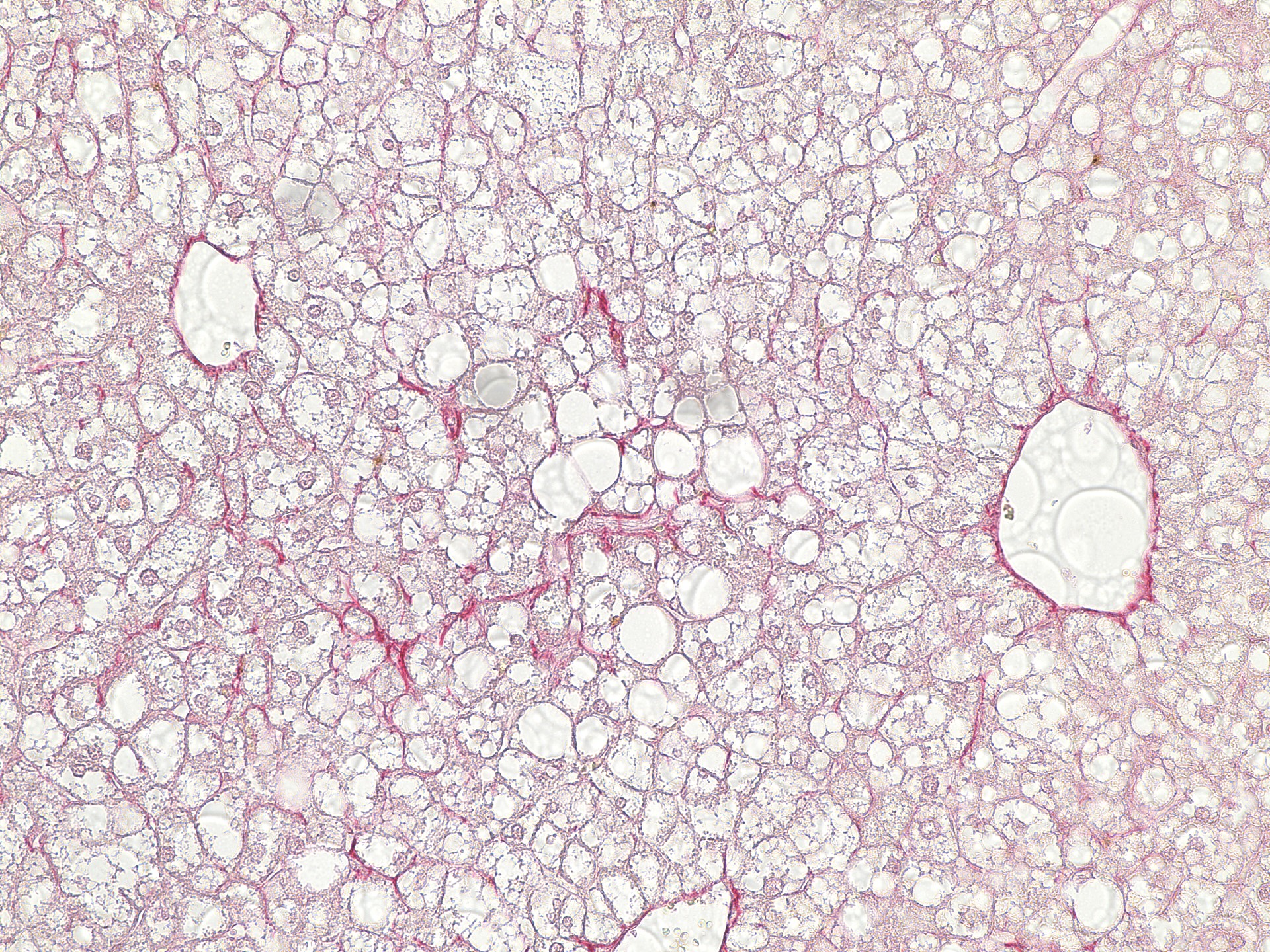

Supplement: Supplementary file 9 — Figure EV3 Source Data [file 44318_2024_196_MOESM9_ESM.zip › Figure EV3/Figure EV3-L/Quantificated image/HFD PCPE-1 vaccine/no.2/Liver-HFD vaccine-no.2-20x-1.jpg]

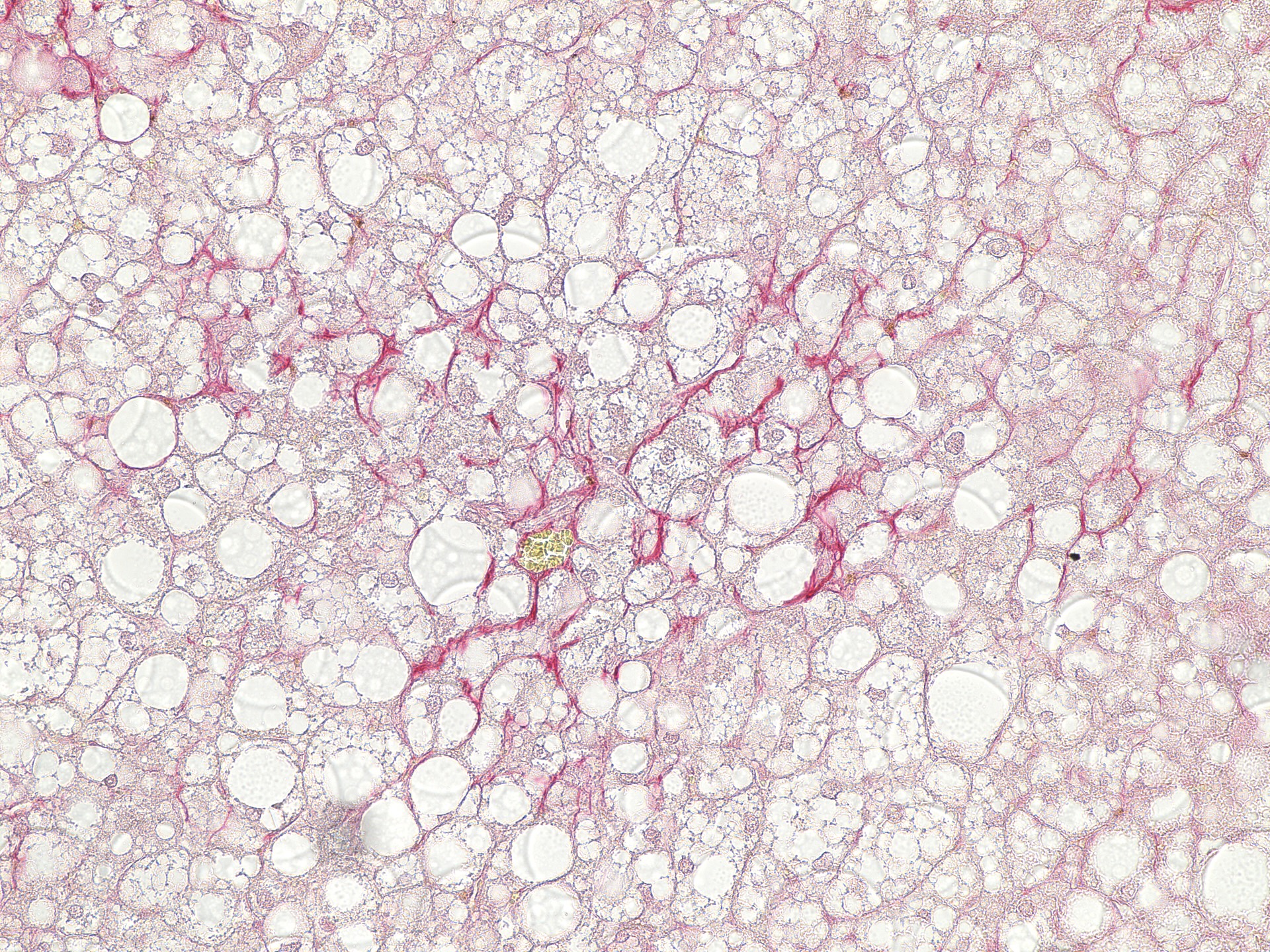

Supplement: Supplementary file 9 — Figure EV3 Source Data [file 44318_2024_196_MOESM9_ESM.zip › Figure EV3/Figure EV3-L/Quantificated image/HFD PCPE-1 vaccine/no.2/Liver-HFD vaccine-no.2-20x-5.jpg]

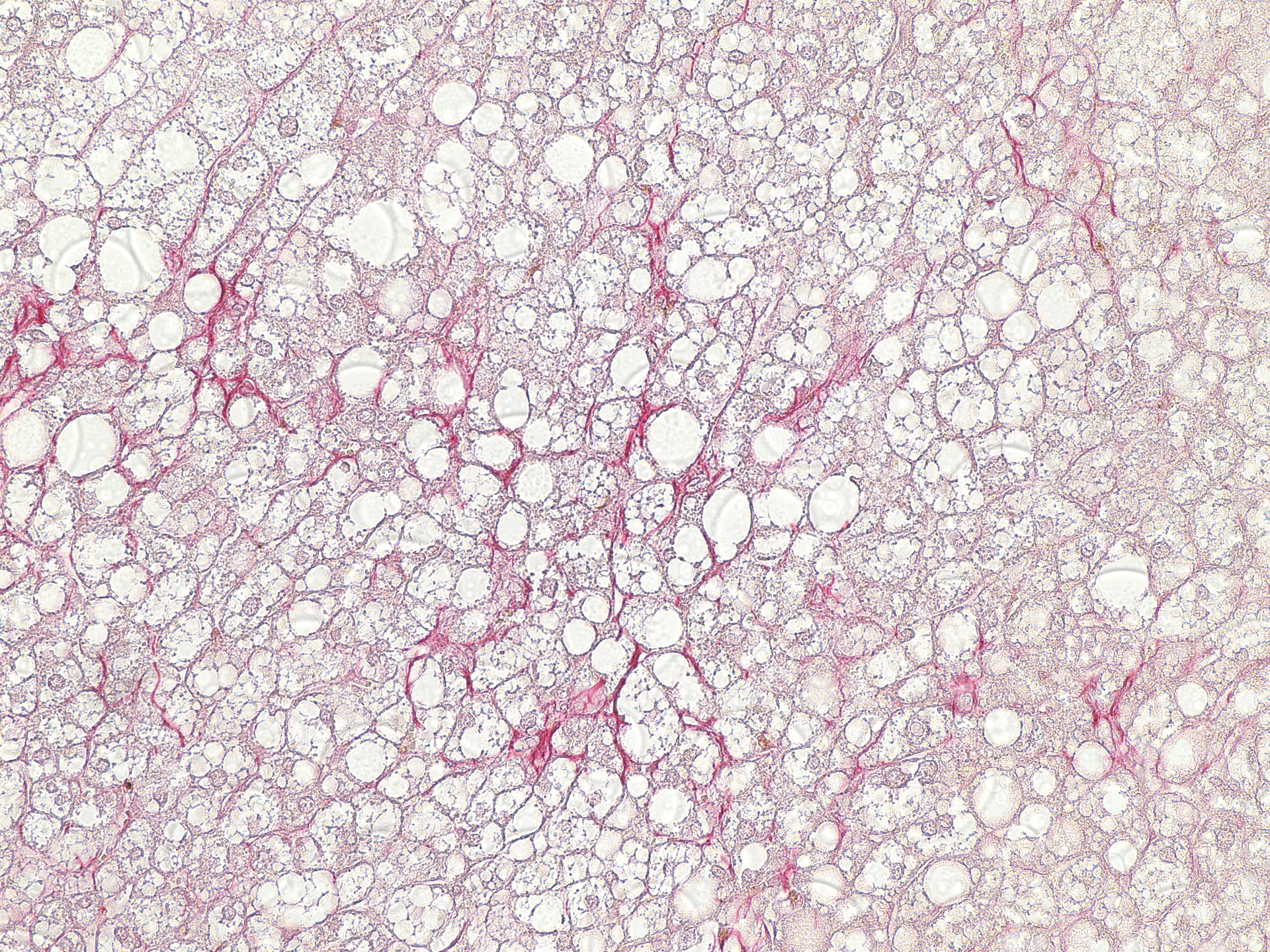

Supplement: Supplementary file 9 — Figure EV3 Source Data [file 44318_2024_196_MOESM9_ESM.zip › Figure EV3/Figure EV3-L/Quantificated image/HFD PCPE-1 vaccine/no.2/Liver-HFD vaccine-no.2-20x-4.jpg]

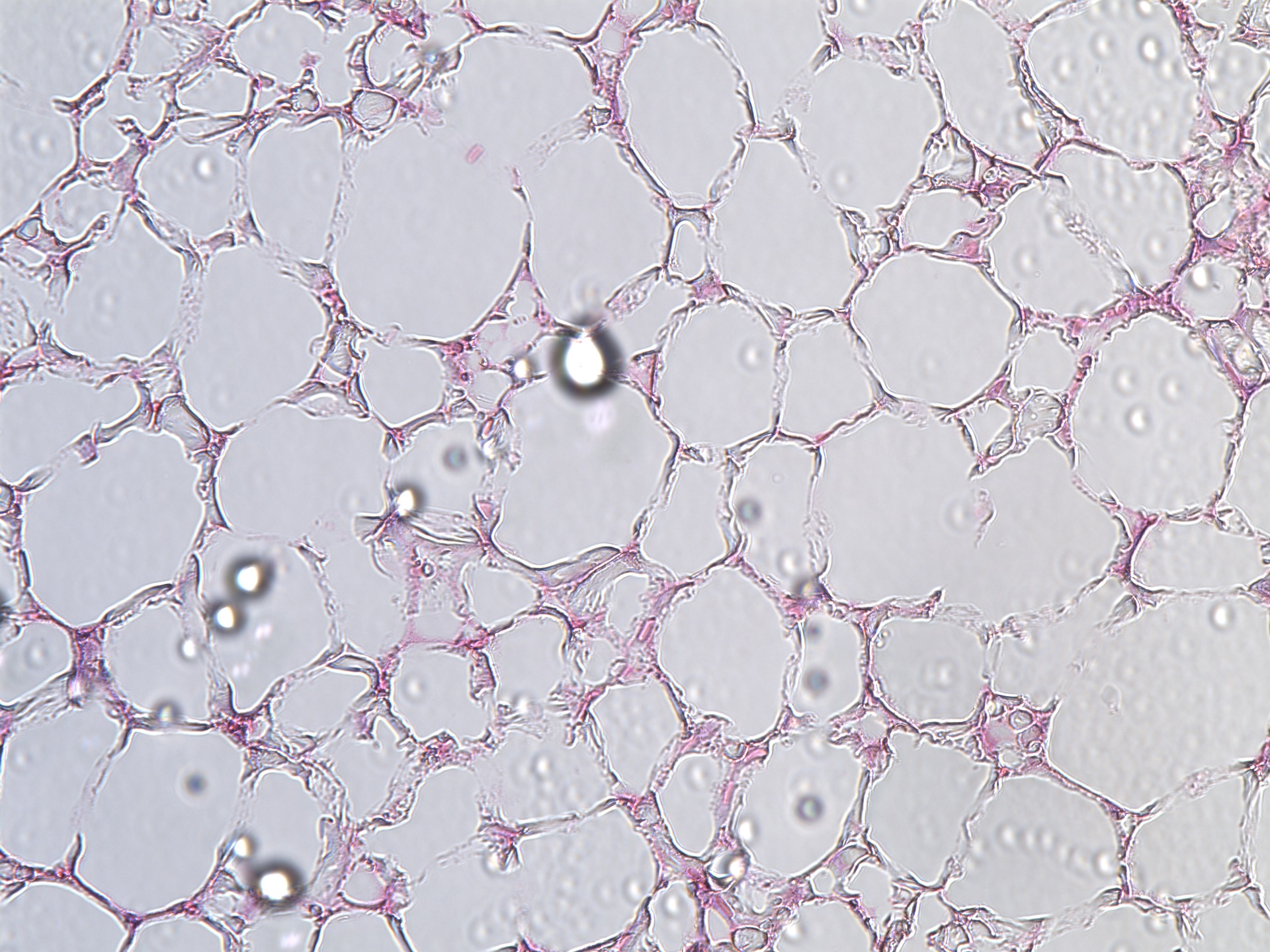

Supplement: Supplementary file 9 — Figure EV3 Source Data [file 44318_2024_196_MOESM9_ESM.zip › Figure EV3/Figure EV3-O/Additional replicate image/HFD Con/no.1/HFD Con-x40_04.jpg]

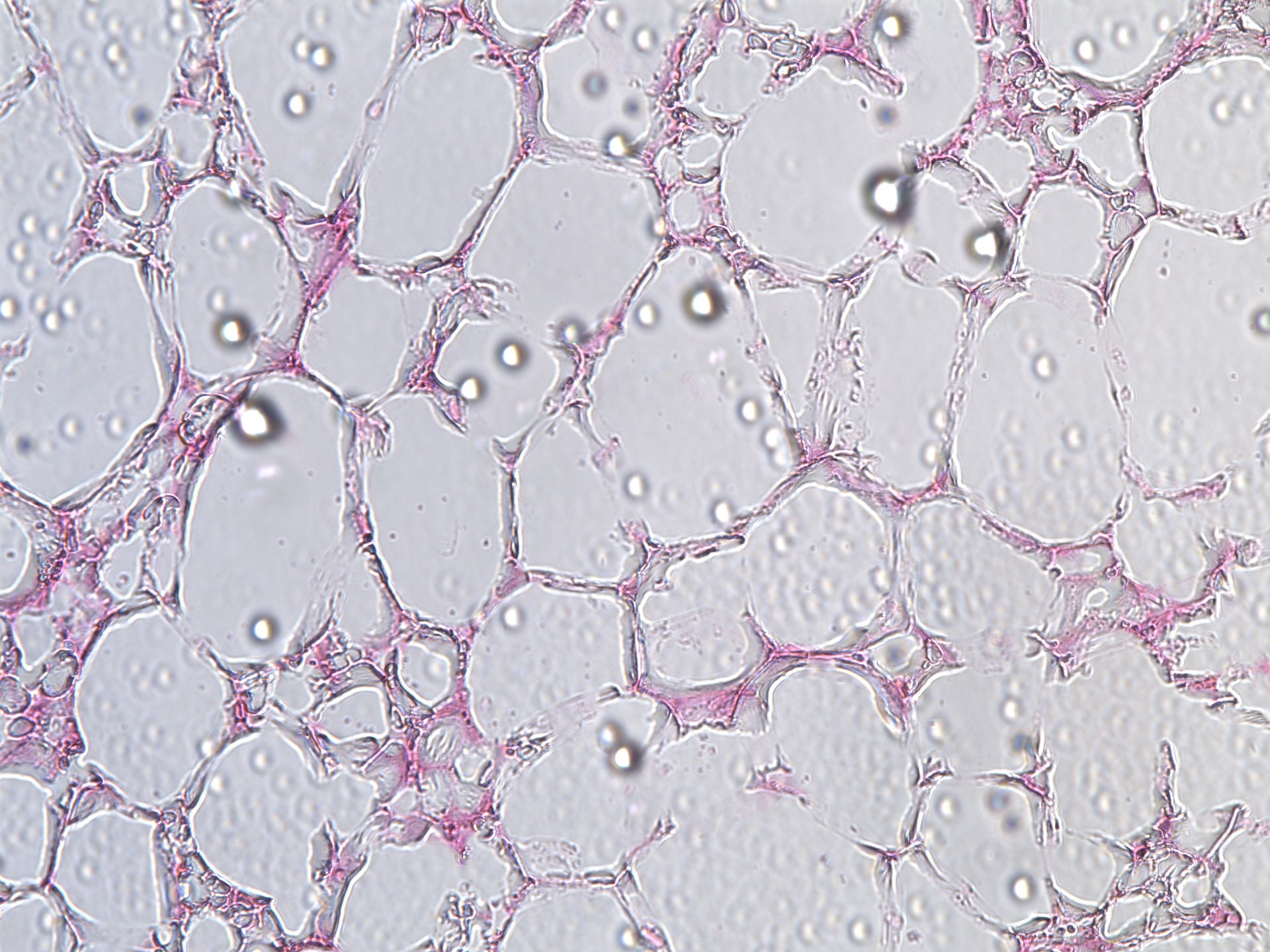

Supplement: Supplementary file 9 — Figure EV3 Source Data [file 44318_2024_196_MOESM9_ESM.zip › Figure EV3/Figure EV3-O/Additional replicate image/HFD Con/no.1/HFD Con-x40_05.jpg]

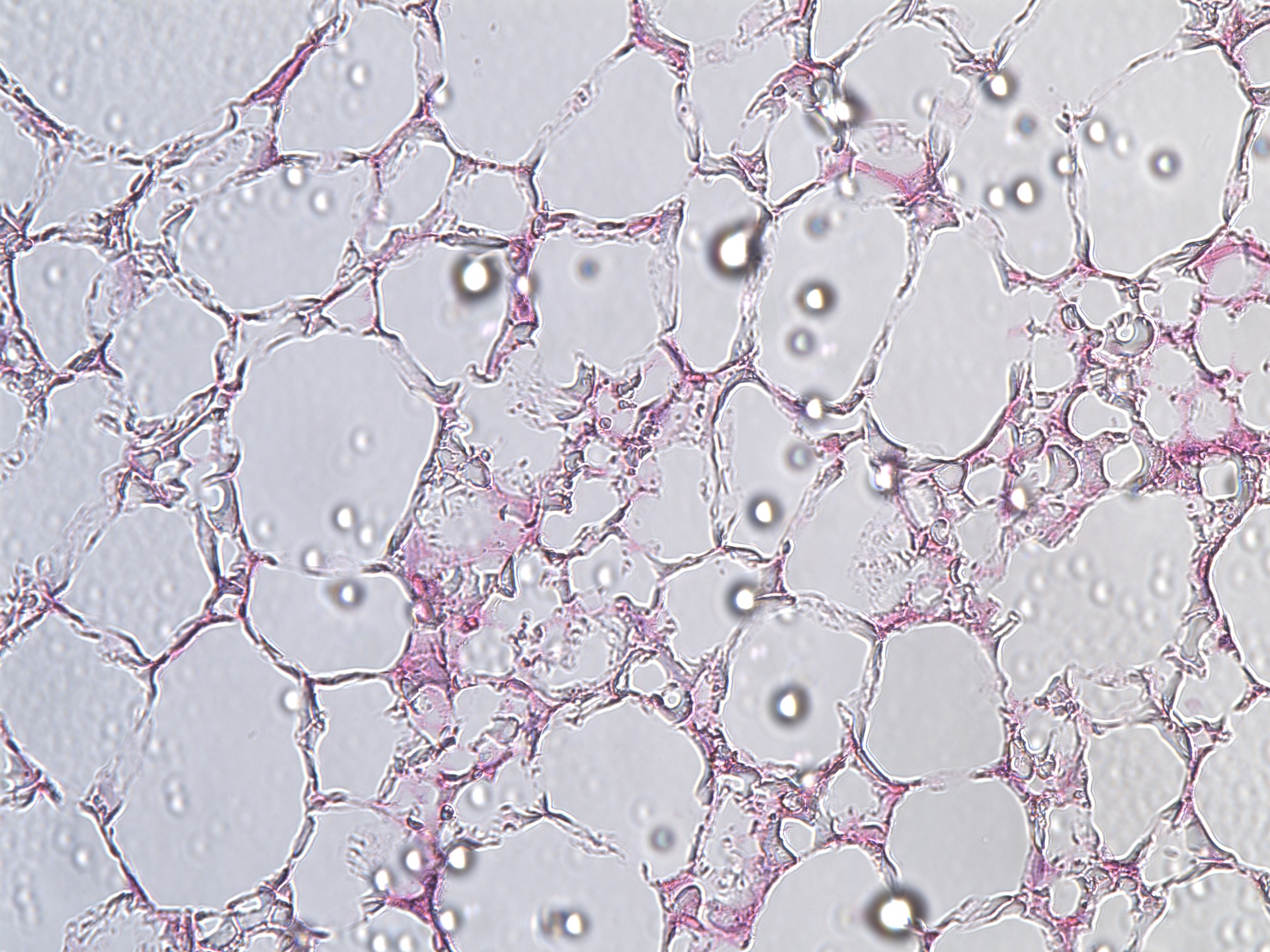

Supplement: Supplementary file 9 — Figure EV3 Source Data [file 44318_2024_196_MOESM9_ESM.zip › Figure EV3/Figure EV3-O/Additional replicate image/HFD Con/no.1/HFD Con-x40_02.jpg]

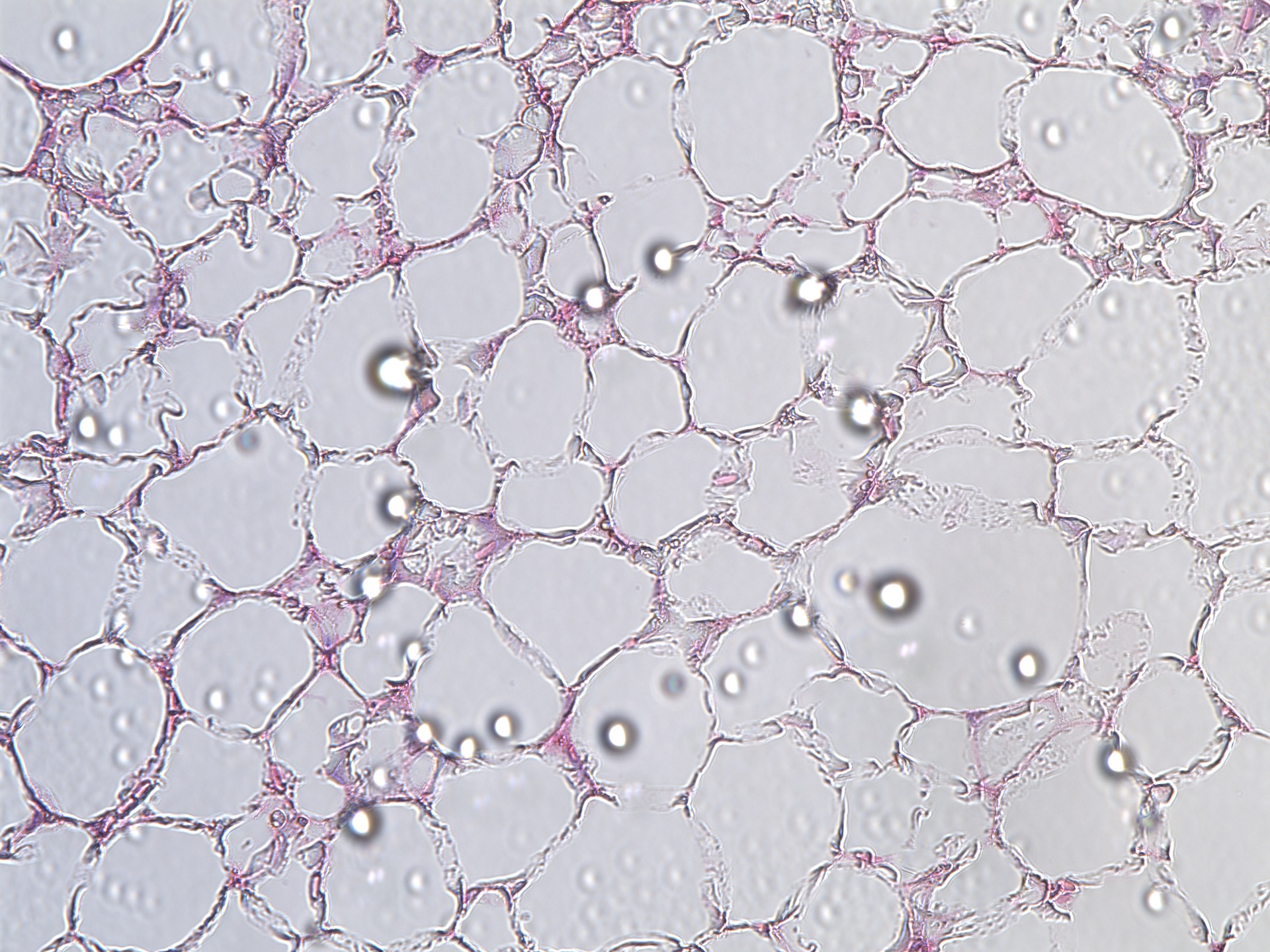

Supplement: Supplementary file 9 — Figure EV3 Source Data [file 44318_2024_196_MOESM9_ESM.zip › Figure EV3/Figure EV3-O/Additional replicate image/HFD Con/no.1/HFD Con-x40_03.jpg]

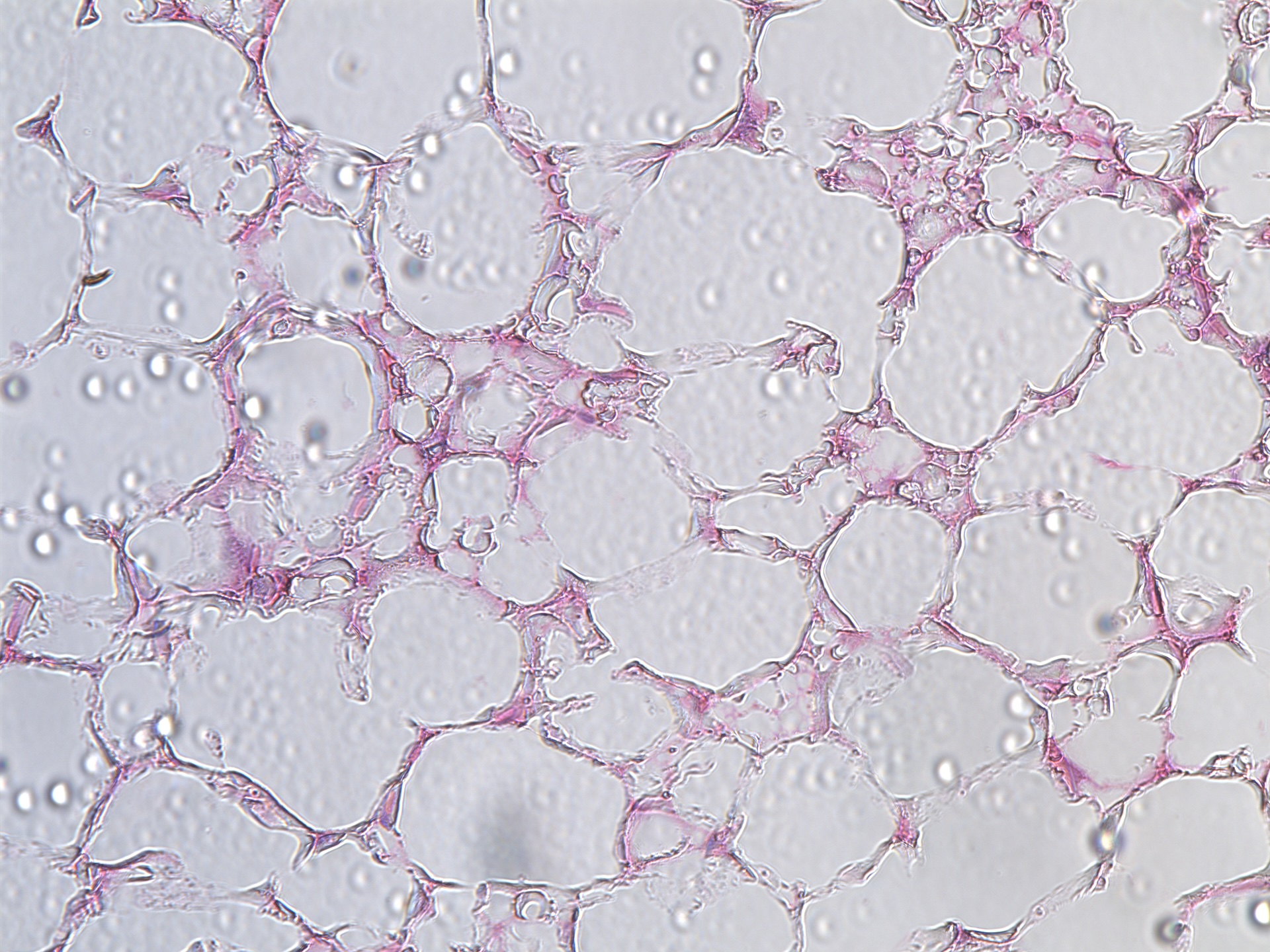

Supplement: Supplementary file 9 — Figure EV3 Source Data [file 44318_2024_196_MOESM9_ESM.zip › Figure EV3/Figure EV3-O/Additional replicate image/HFD Con/no.1/HFD Con-x40_01.jpg]

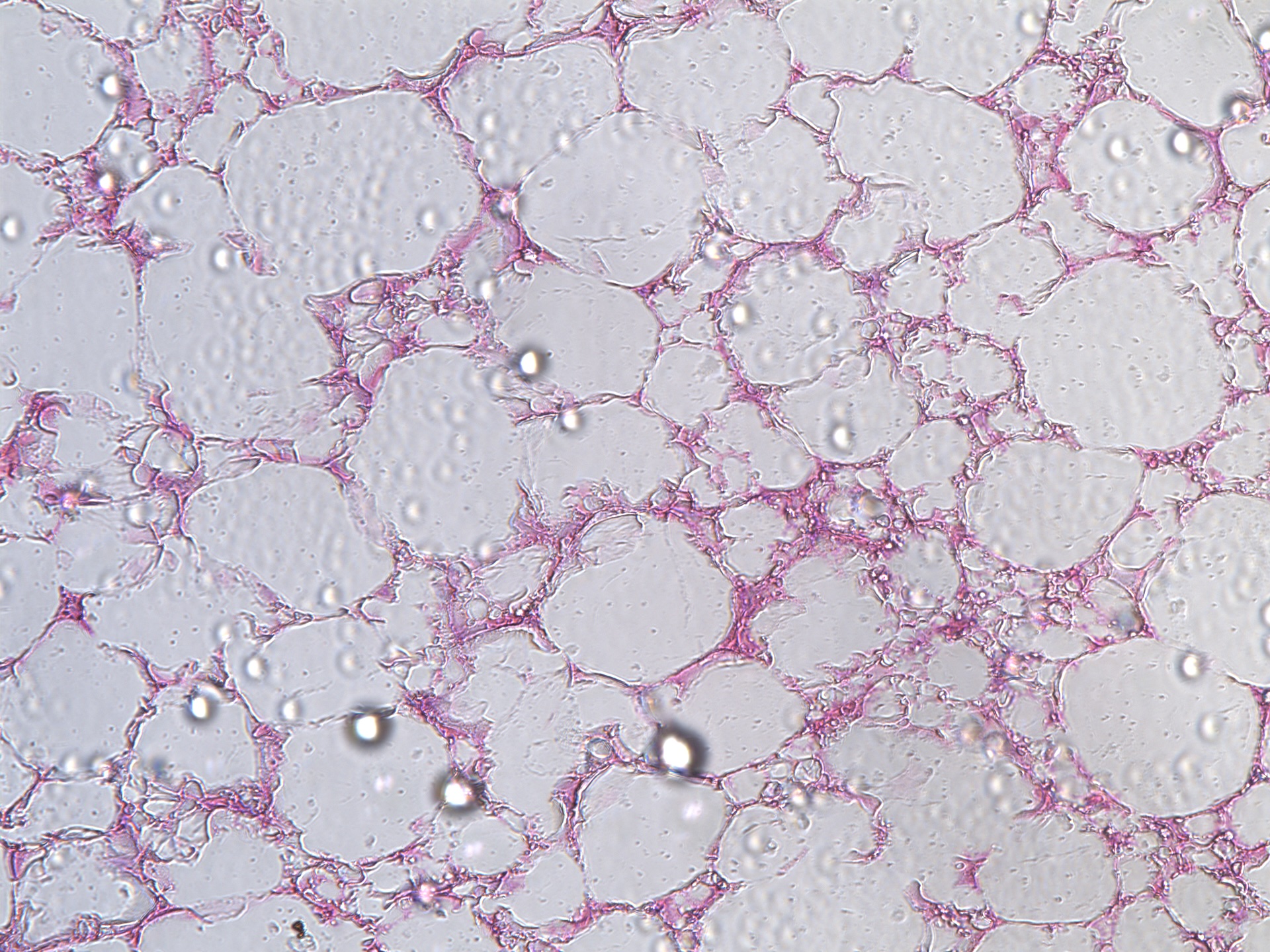

Supplement: Supplementary file 9 — Figure EV3 Source Data [file 44318_2024_196_MOESM9_ESM.zip › Figure EV3/Figure EV3-O/Additional replicate image/HFD Con/no.3/HFD Con-x40_04.jpg]

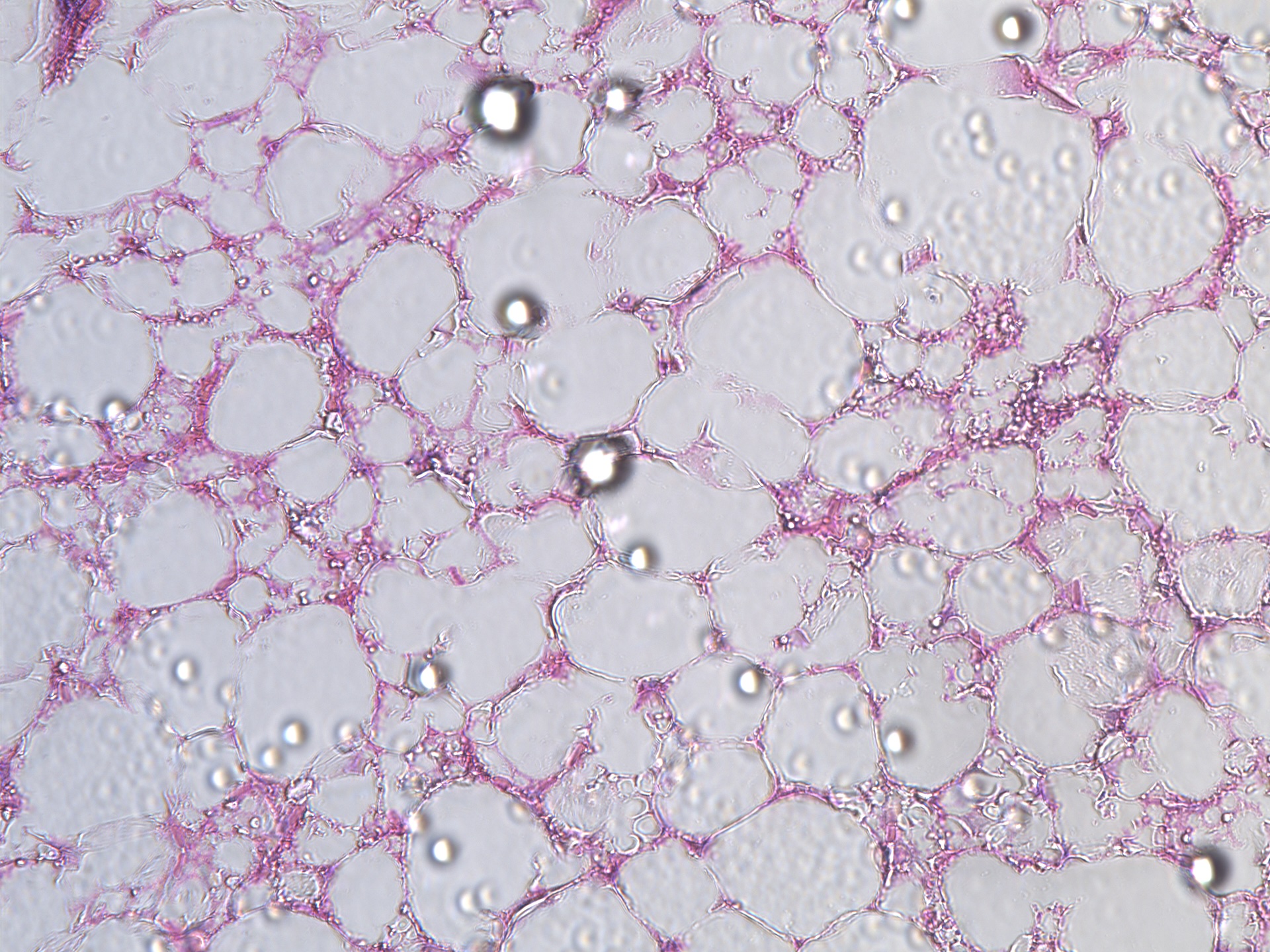

Supplement: Supplementary file 9 — Figure EV3 Source Data [file 44318_2024_196_MOESM9_ESM.zip › Figure EV3/Figure EV3-O/Additional replicate image/HFD Con/no.3/HFD Con-x40_05.jpg]

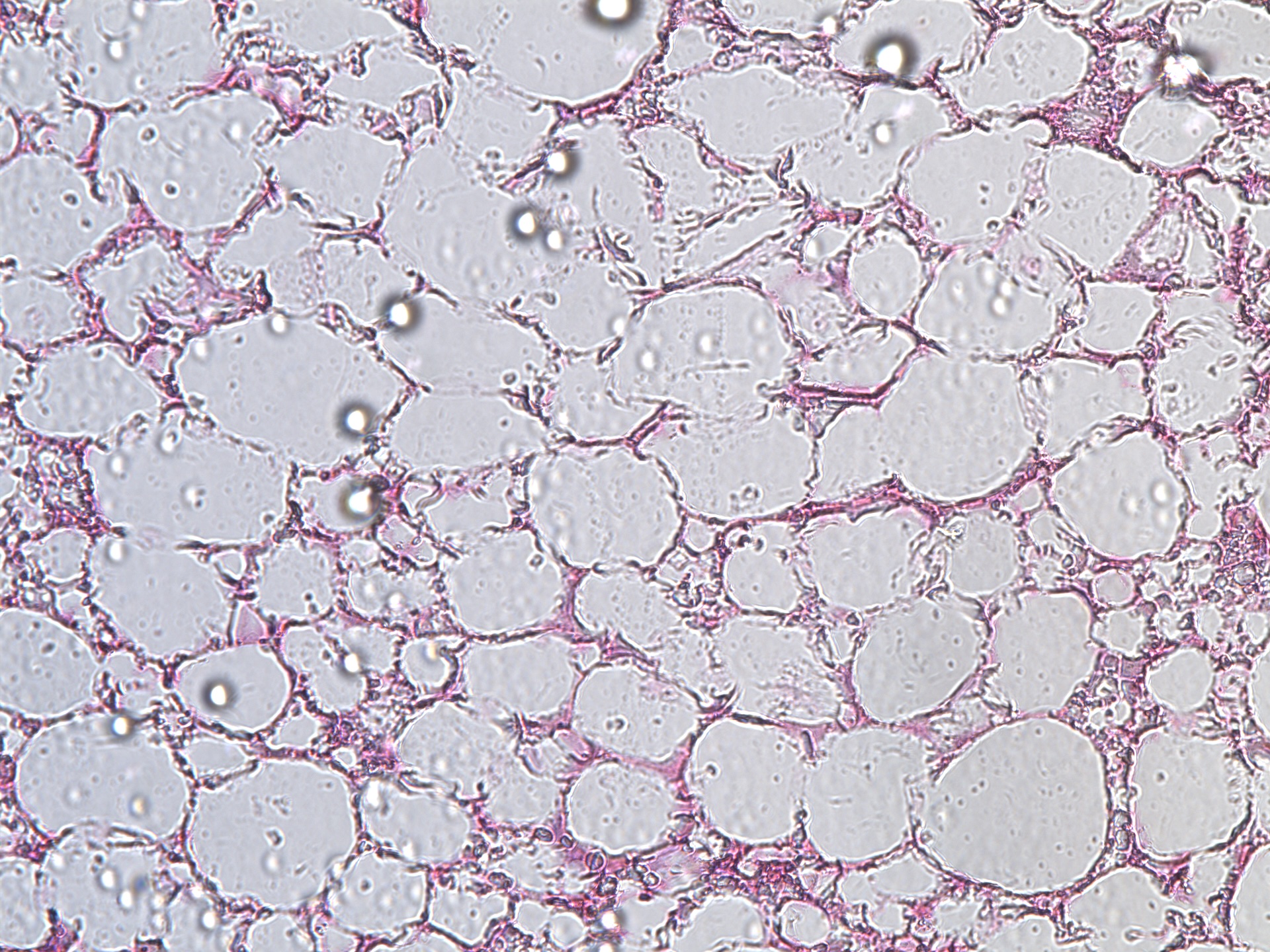

Supplement: Supplementary file 9 — Figure EV3 Source Data [file 44318_2024_196_MOESM9_ESM.zip › Figure EV3/Figure EV3-O/Additional replicate image/HFD Con/no.3/HFD Con-x40_02.jpg]

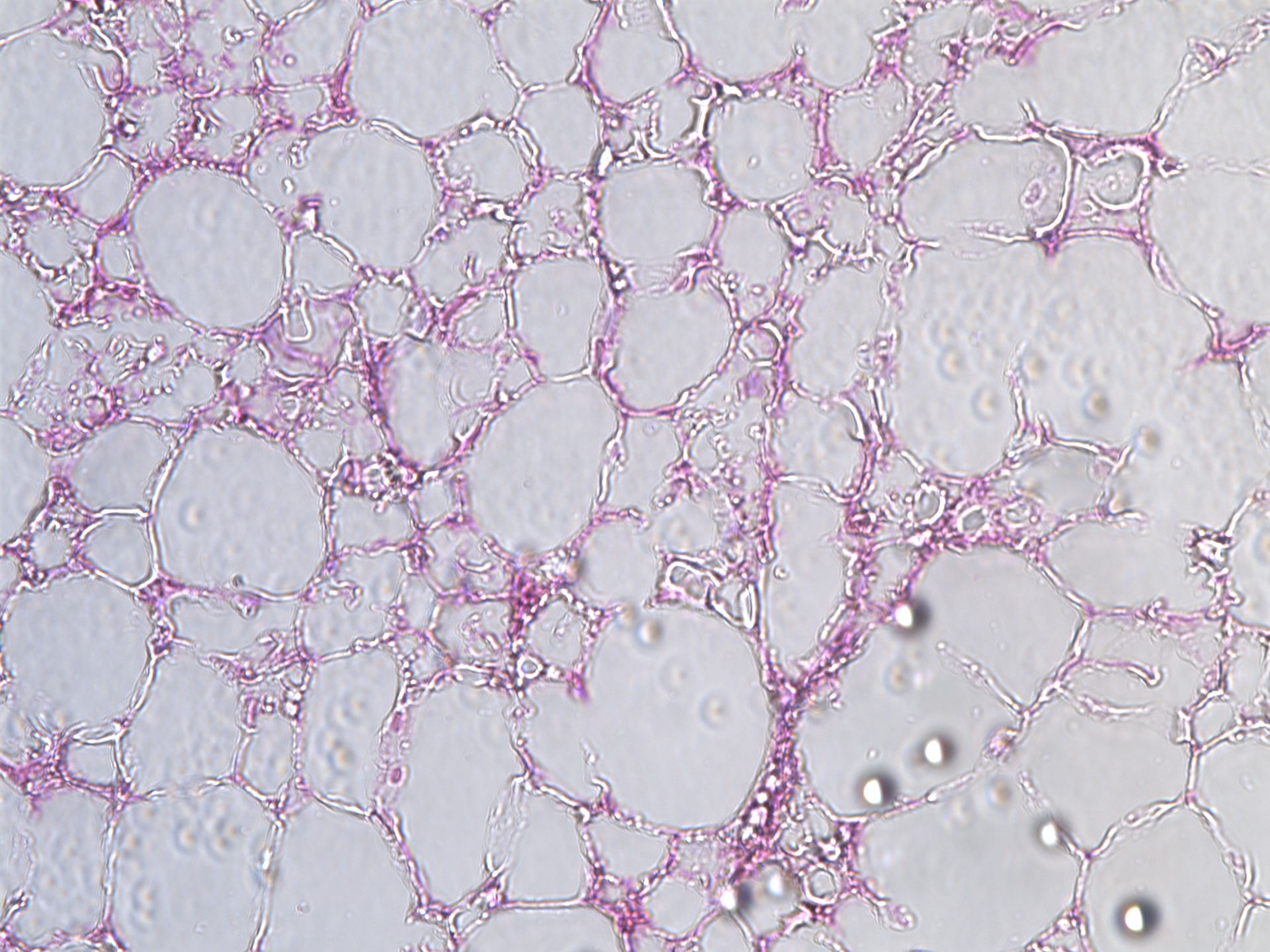

Supplement: Supplementary file 9 — Figure EV3 Source Data [file 44318_2024_196_MOESM9_ESM.zip › Figure EV3/Figure EV3-O/Additional replicate image/HFD Con/no.3/HFD Con-x40_03.jpg]

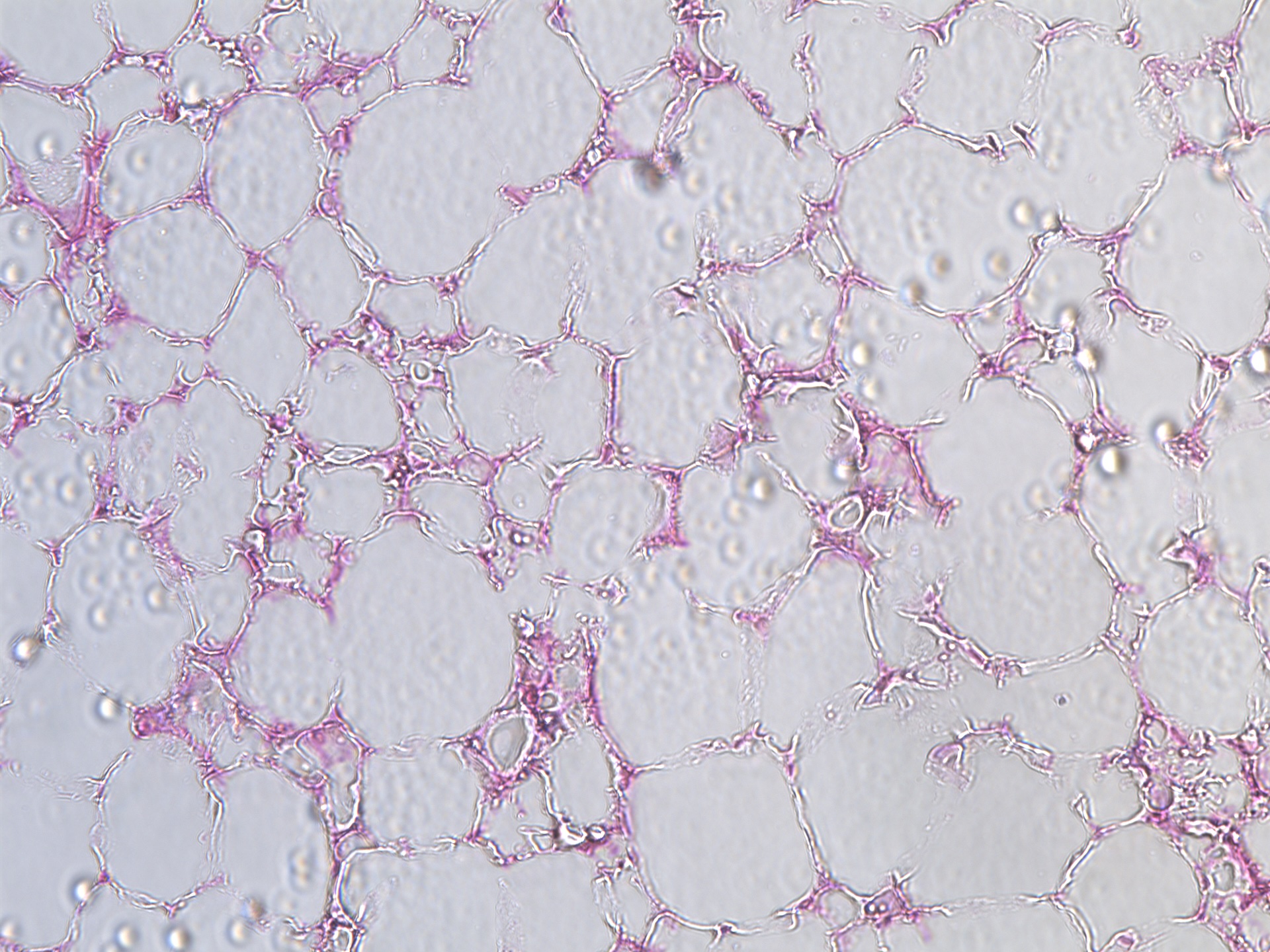

Supplement: Supplementary file 9 — Figure EV3 Source Data [file 44318_2024_196_MOESM9_ESM.zip › Figure EV3/Figure EV3-O/Additional replicate image/HFD Con/no.3/HFD Con-x40_01.jpg]

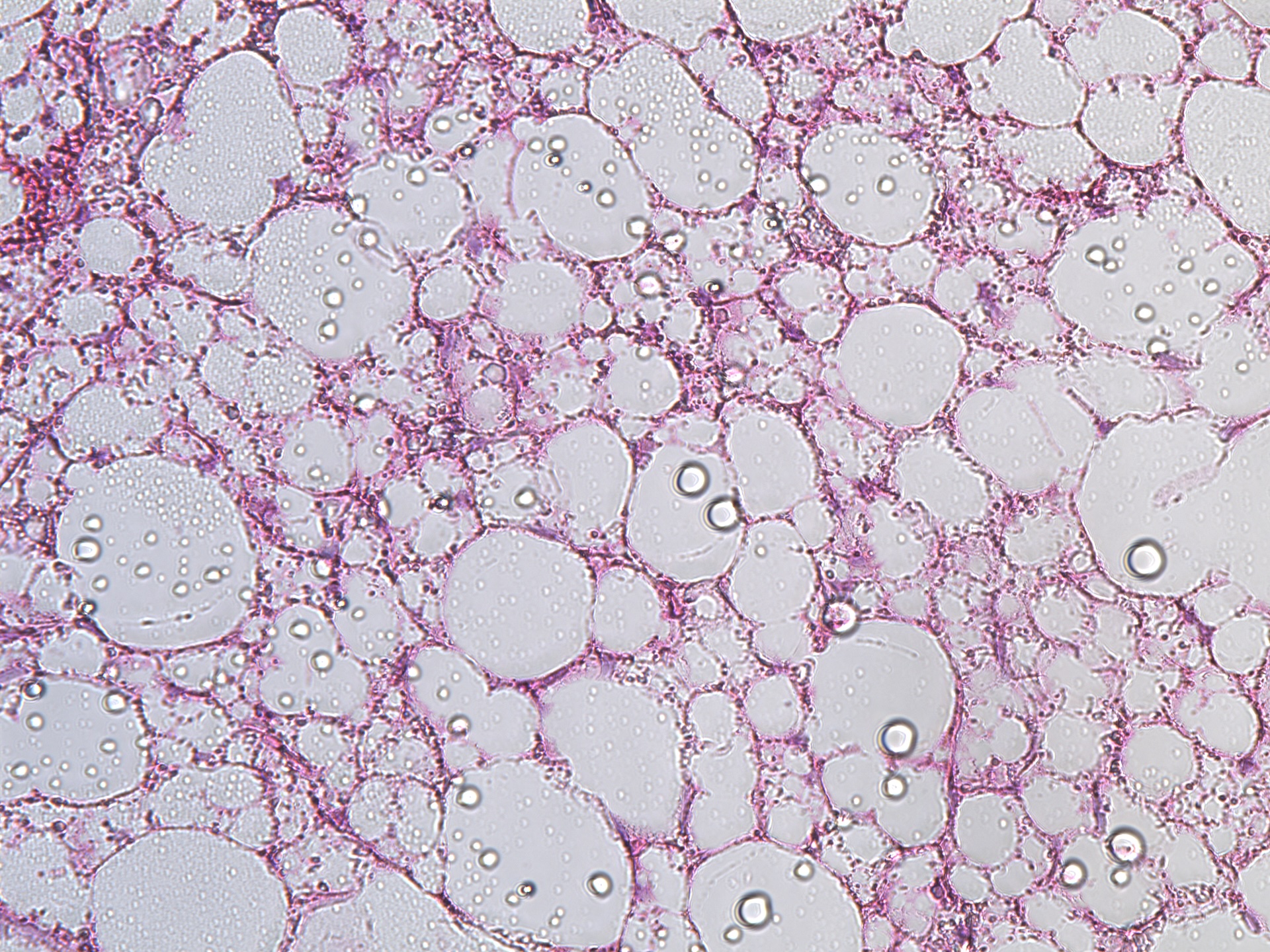

Supplement: Supplementary file 9 — Figure EV3 Source Data [file 44318_2024_196_MOESM9_ESM.zip › Figure EV3/Figure EV3-O/Additional replicate image/HFD Con/no.4/HFD Con-x40_04.jpg]

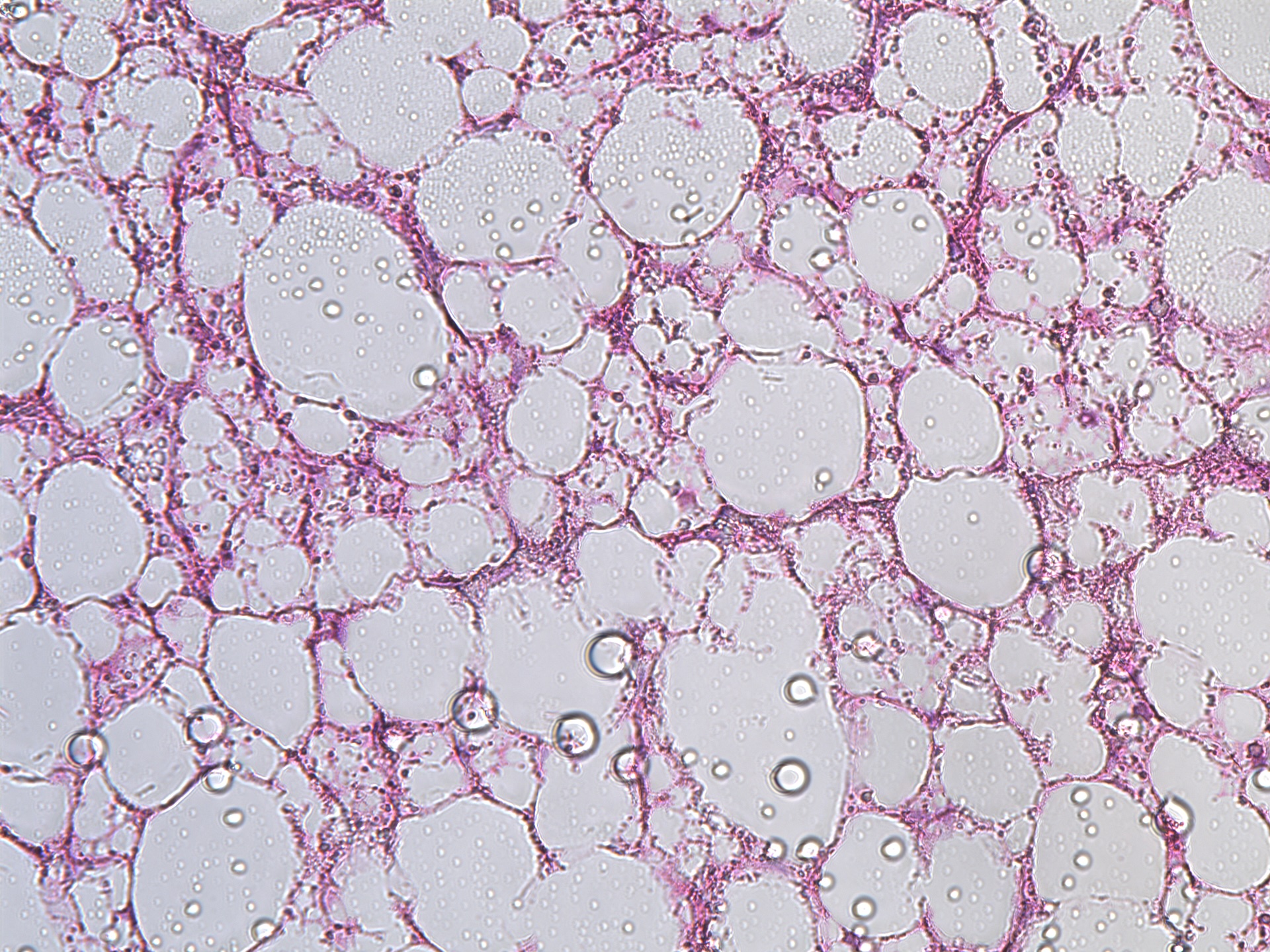

Supplement: Supplementary file 9 — Figure EV3 Source Data [file 44318_2024_196_MOESM9_ESM.zip › Figure EV3/Figure EV3-O/Additional replicate image/HFD Con/no.4/HFD Con-x40_05.jpg]

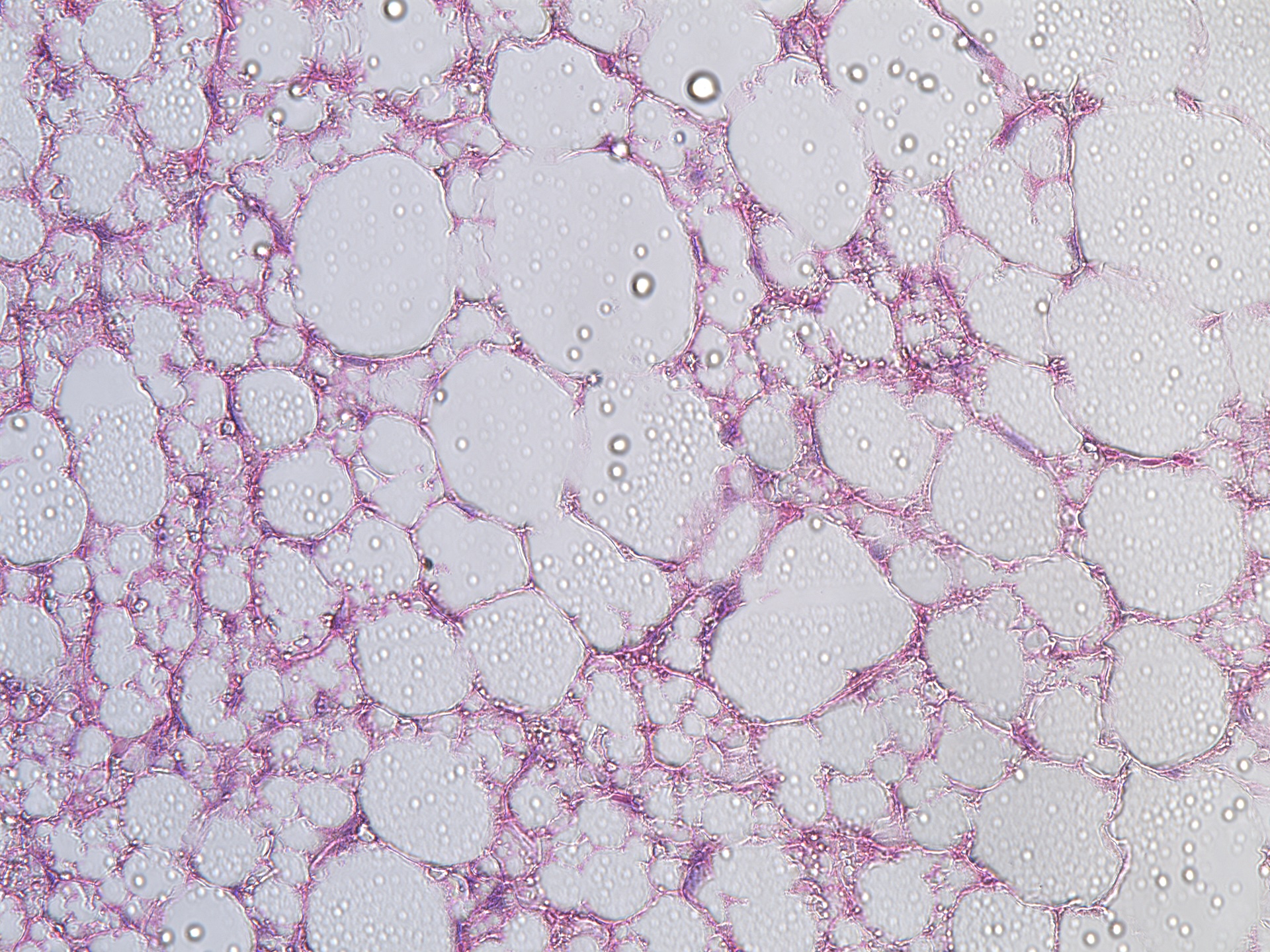

Supplement: Supplementary file 9 — Figure EV3 Source Data [file 44318_2024_196_MOESM9_ESM.zip › Figure EV3/Figure EV3-O/Additional replicate image/HFD Con/no.4/HFD Con-x40_02.jpg]

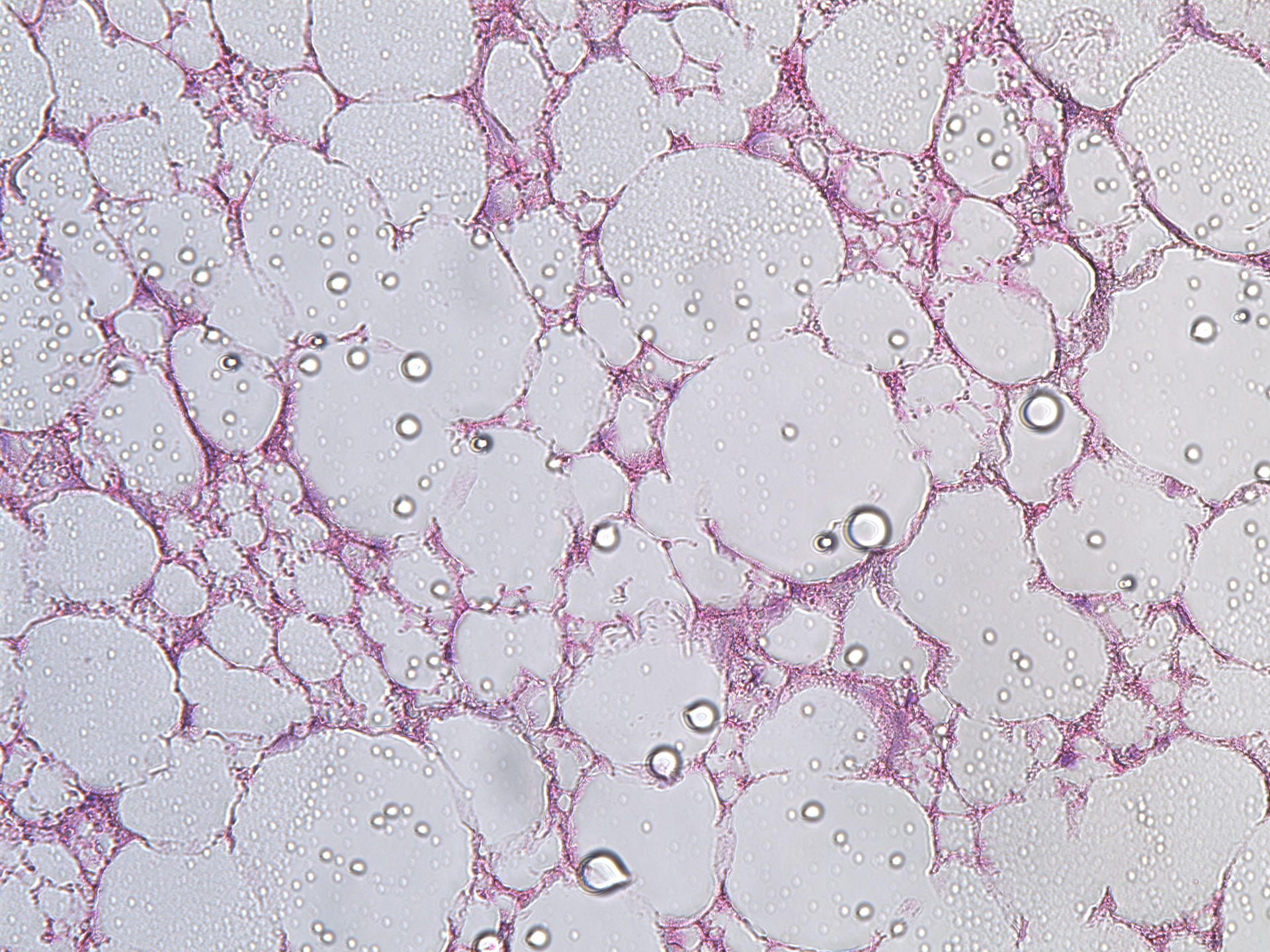

Supplement: Supplementary file 9 — Figure EV3 Source Data [file 44318_2024_196_MOESM9_ESM.zip › Figure EV3/Figure EV3-O/Additional replicate image/HFD Con/no.4/HFD Con-x40_03.jpg]

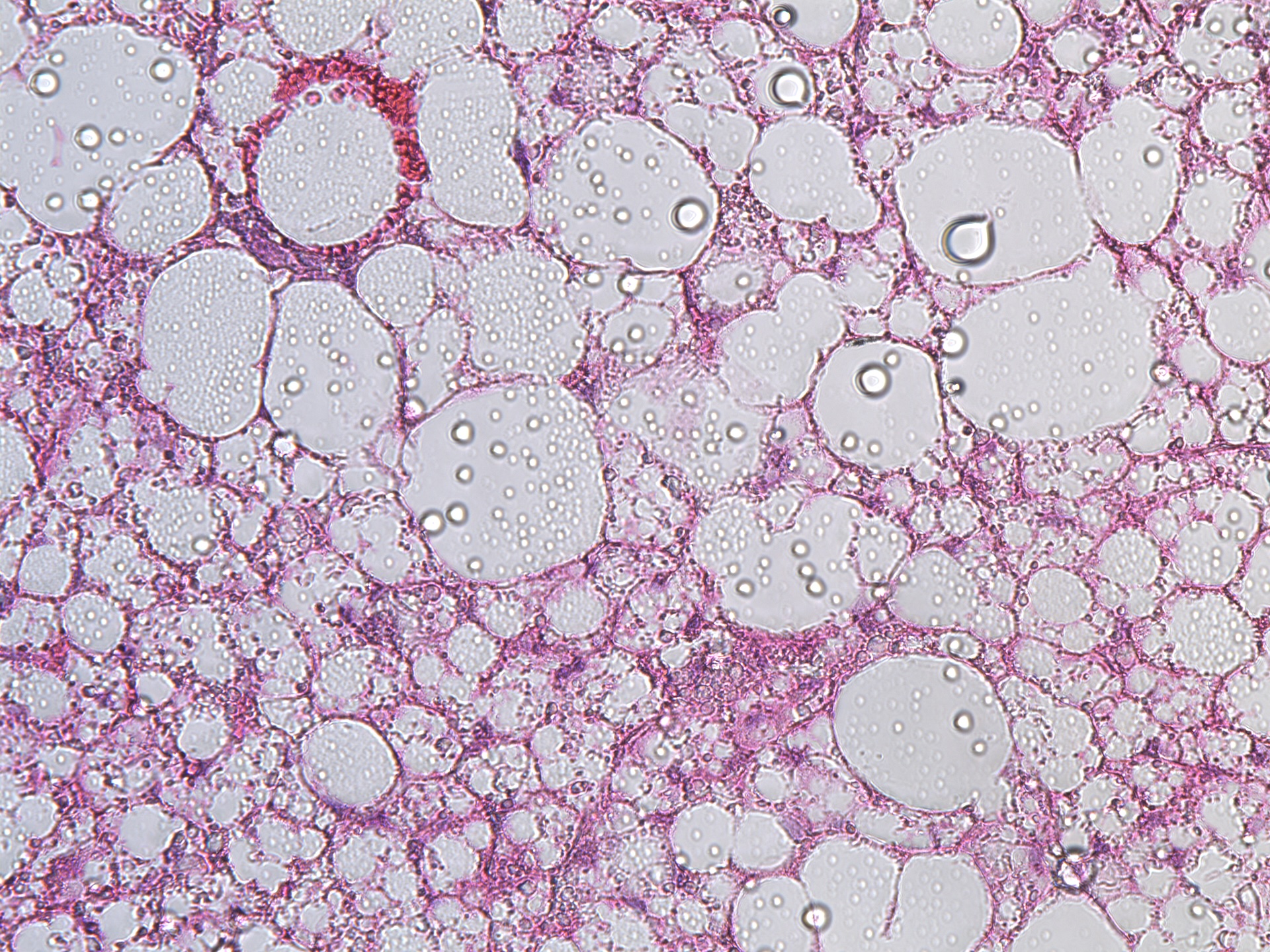

Supplement: Supplementary file 9 — Figure EV3 Source Data [file 44318_2024_196_MOESM9_ESM.zip › Figure EV3/Figure EV3-O/Additional replicate image/HFD Con/no.4/HFD Con-x40_01.jpg]

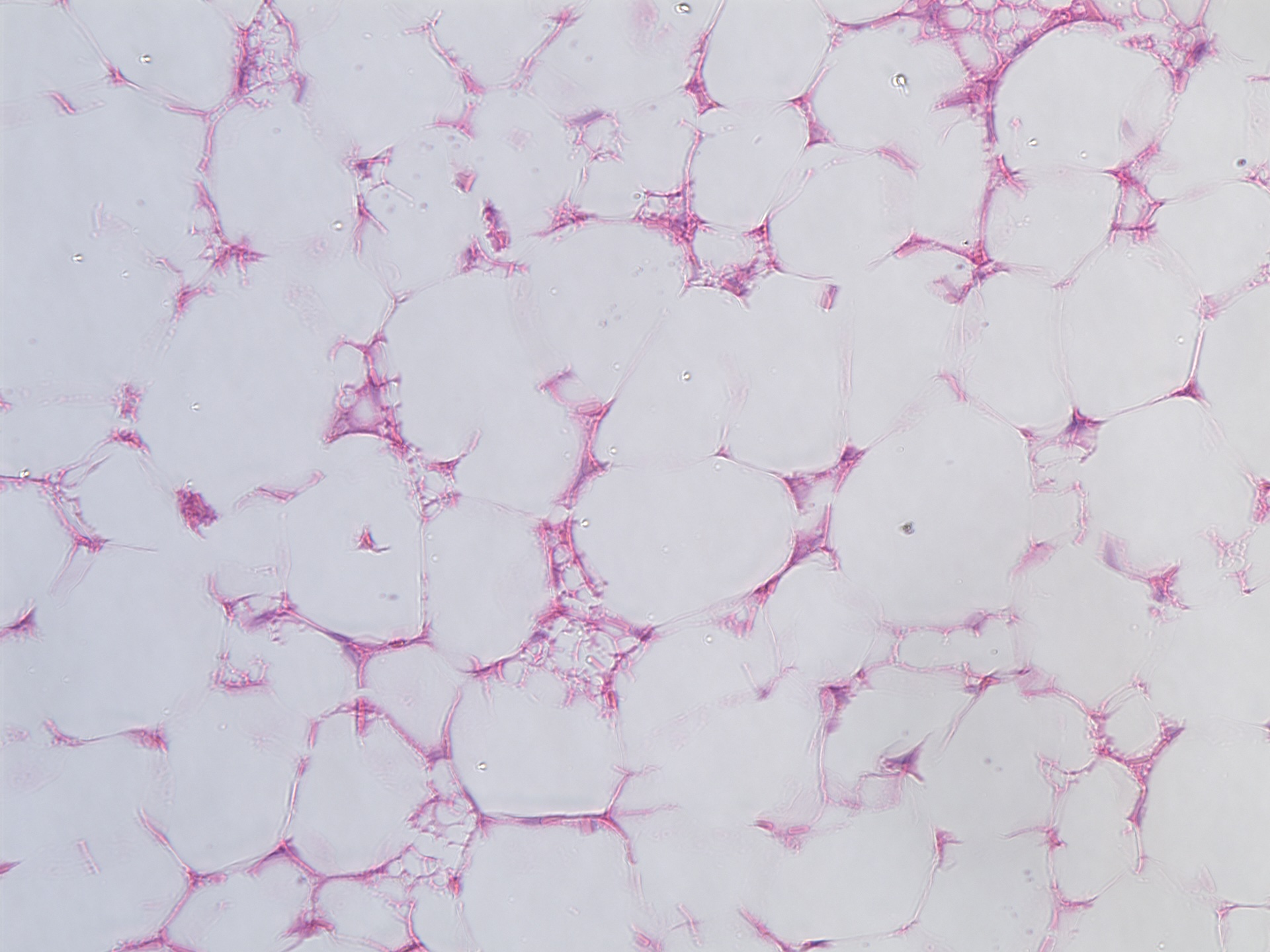

Supplement: Supplementary file 9 — Figure EV3 Source Data [file 44318_2024_196_MOESM9_ESM.zip › Figure EV3/Figure EV3-O/Additional replicate image/HFD Con/no.5/HFD Con-x40_04.jpg]

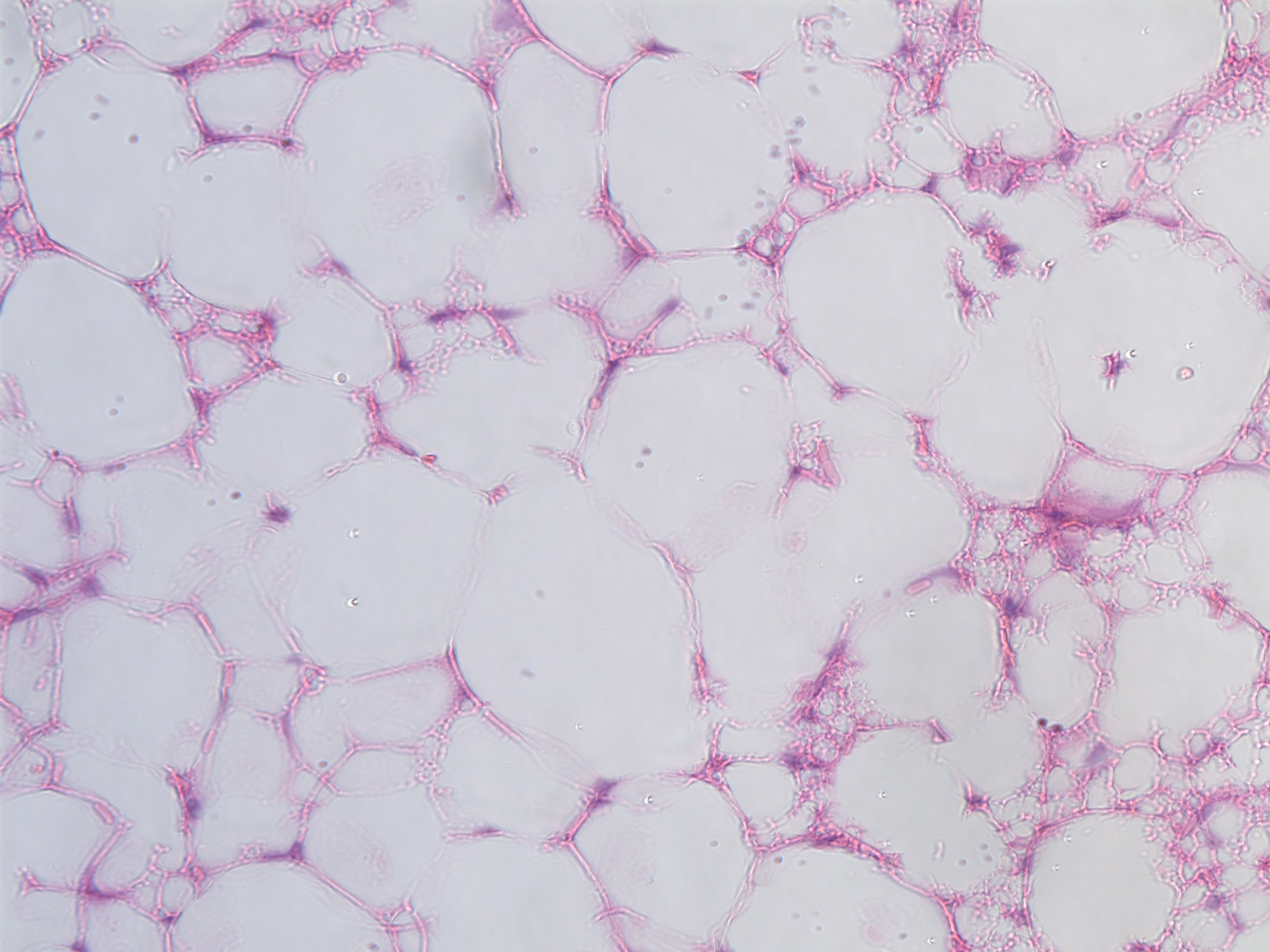

Supplement: Supplementary file 9 — Figure EV3 Source Data [file 44318_2024_196_MOESM9_ESM.zip › Figure EV3/Figure EV3-O/Additional replicate image/HFD Con/no.5/HFD Con-x40_05.jpg]

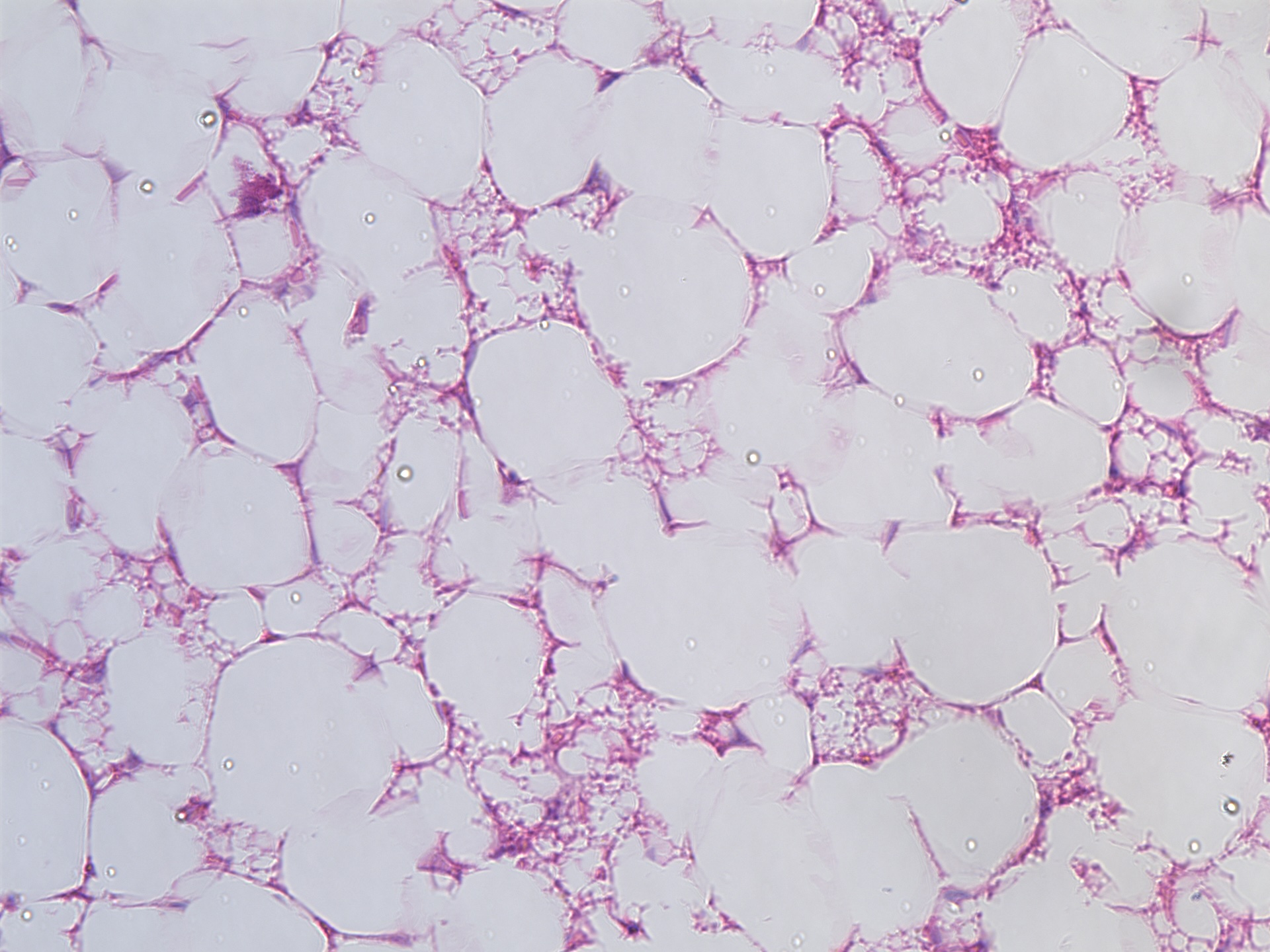

Supplement: Supplementary file 9 — Figure EV3 Source Data [file 44318_2024_196_MOESM9_ESM.zip › Figure EV3/Figure EV3-O/Additional replicate image/HFD Con/no.5/HFD Con-x40_02.jpg]

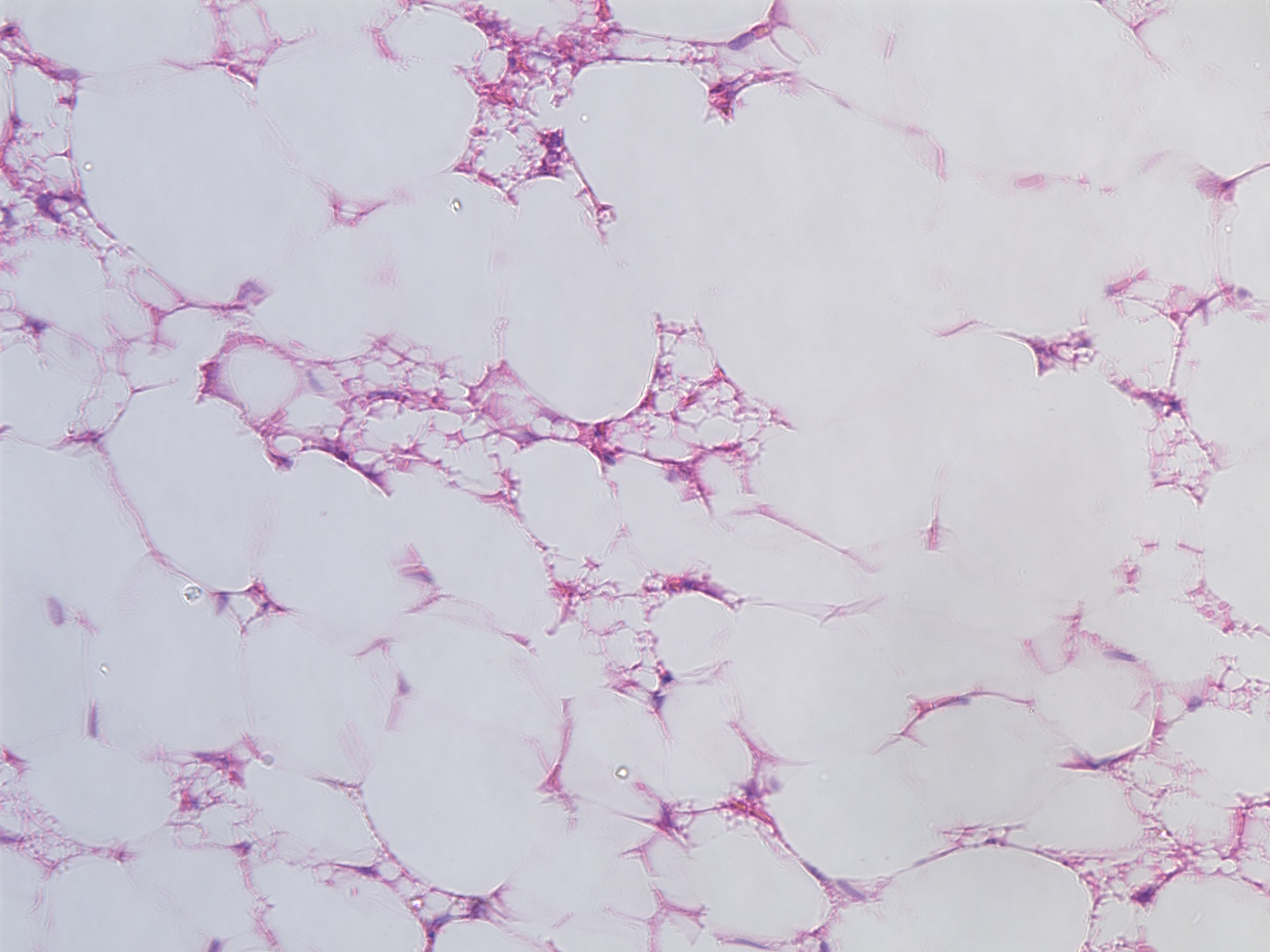

Supplement: Supplementary file 9 — Figure EV3 Source Data [file 44318_2024_196_MOESM9_ESM.zip › Figure EV3/Figure EV3-O/Additional replicate image/HFD Con/no.5/HFD Con-x40_03.jpg]

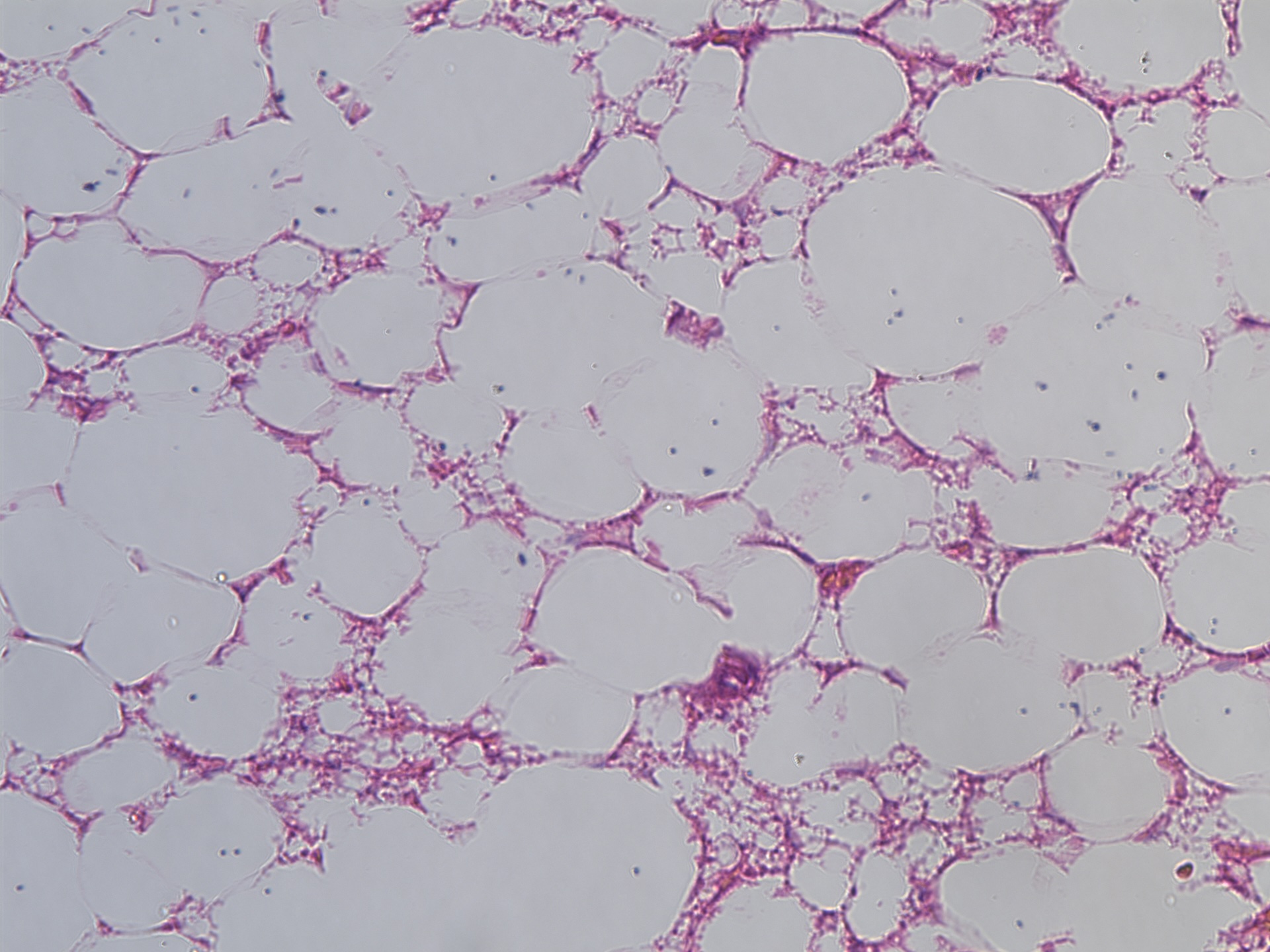

Supplement: Supplementary file 9 — Figure EV3 Source Data [file 44318_2024_196_MOESM9_ESM.zip › Figure EV3/Figure EV3-O/Additional replicate image/HFD Con/no.5/HFD Con-x40_01.jpg]

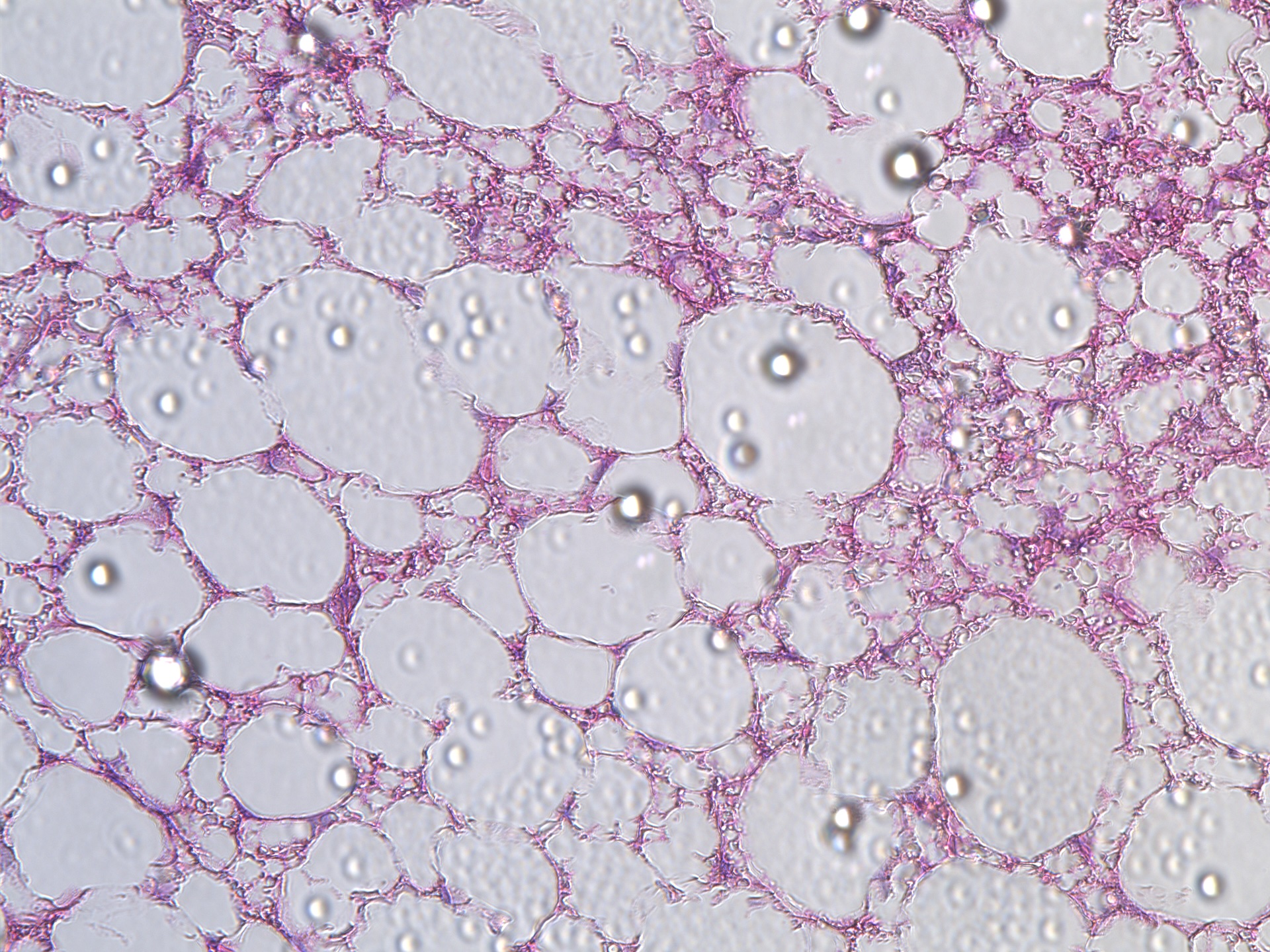

Supplement: Supplementary file 9 — Figure EV3 Source Data [file 44318_2024_196_MOESM9_ESM.zip › Figure EV3/Figure EV3-O/Additional replicate image/HFD Con/no.2/HFD Con-x40_04.jpg]

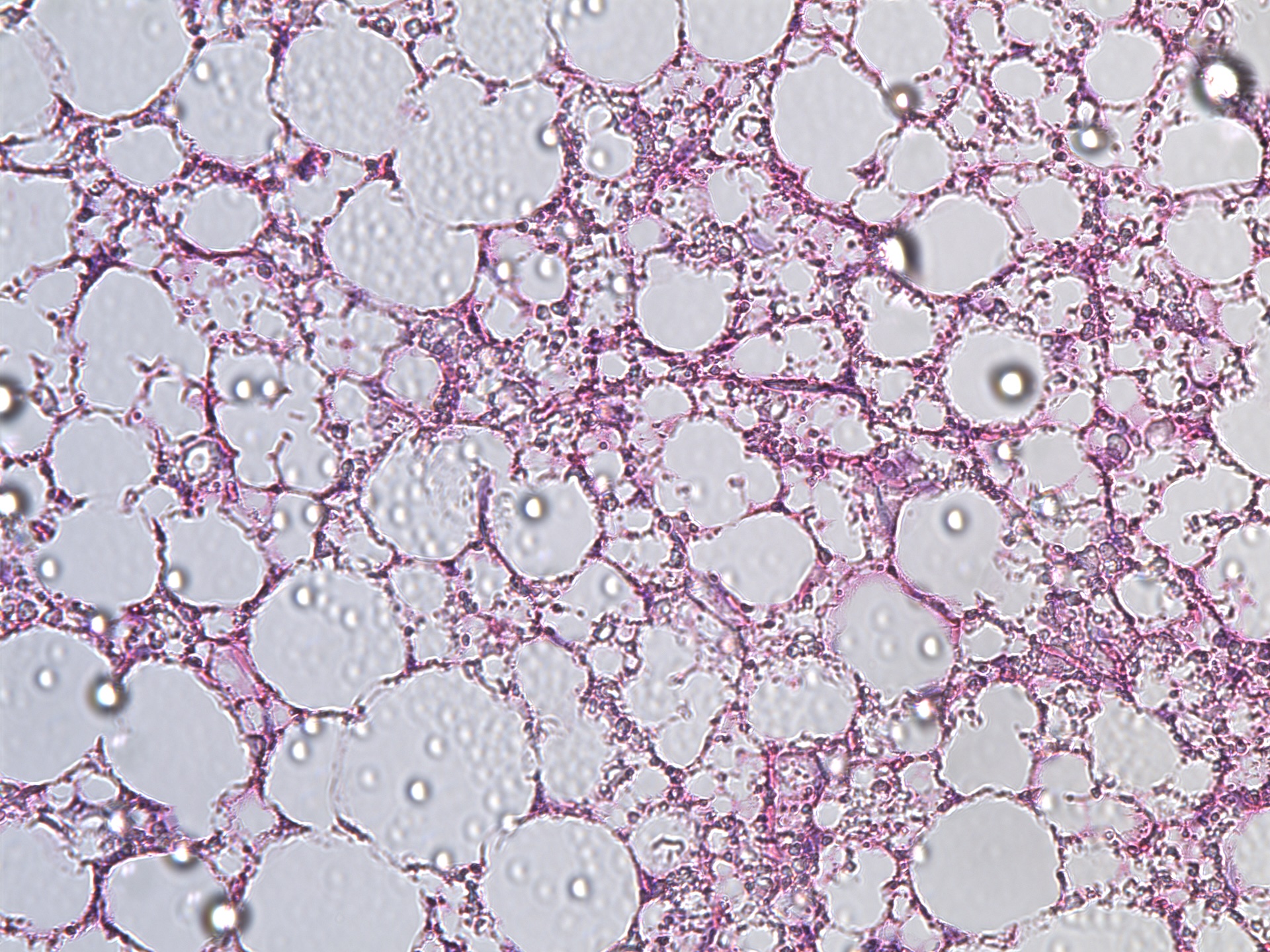

Supplement: Supplementary file 9 — Figure EV3 Source Data [file 44318_2024_196_MOESM9_ESM.zip › Figure EV3/Figure EV3-O/Additional replicate image/HFD Con/no.2/HFD Con-x40_05.jpg]

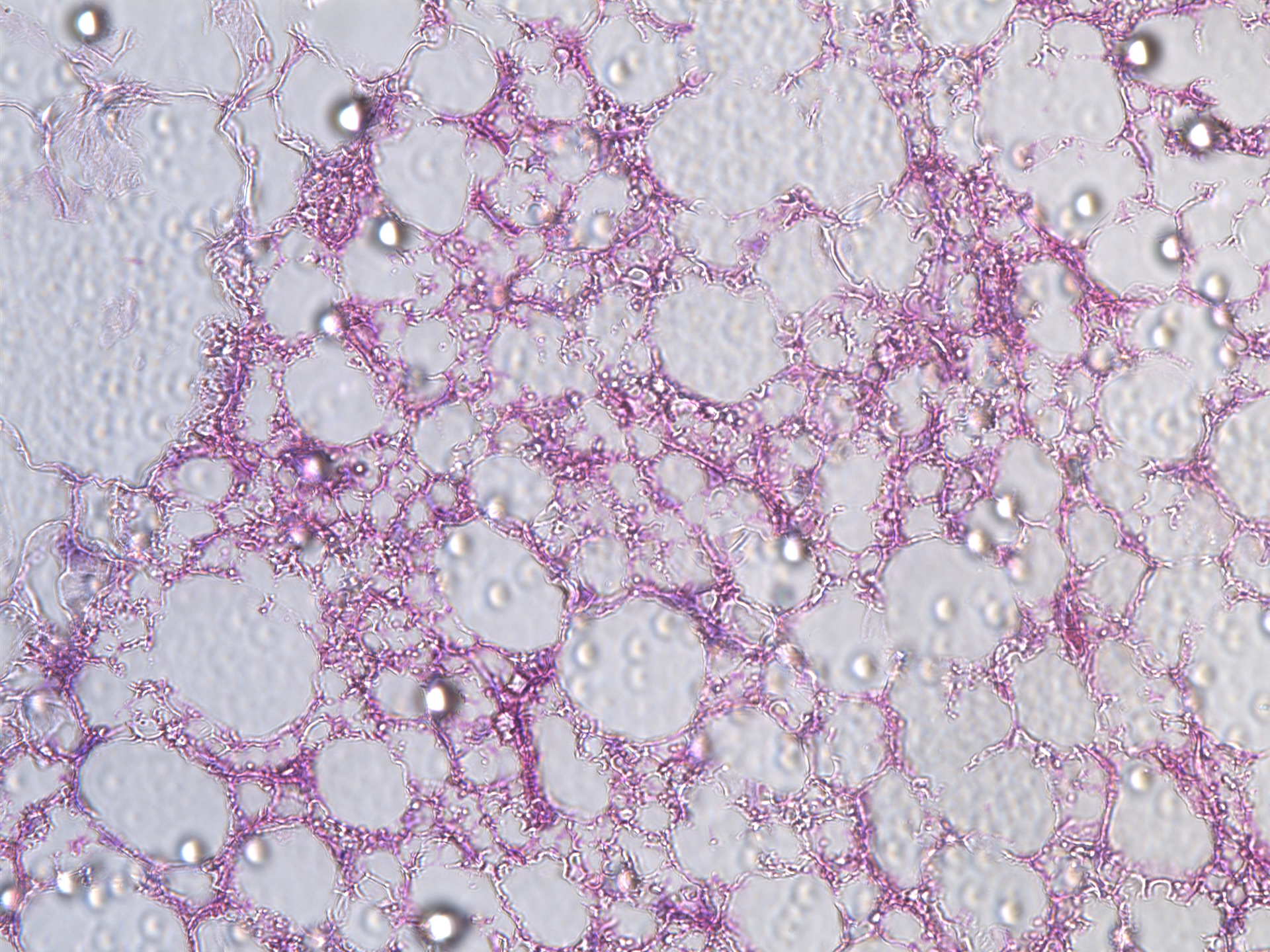

Supplement: Supplementary file 9 — Figure EV3 Source Data [file 44318_2024_196_MOESM9_ESM.zip › Figure EV3/Figure EV3-O/Additional replicate image/HFD Con/no.2/HFD Con-x40_02.jpg]

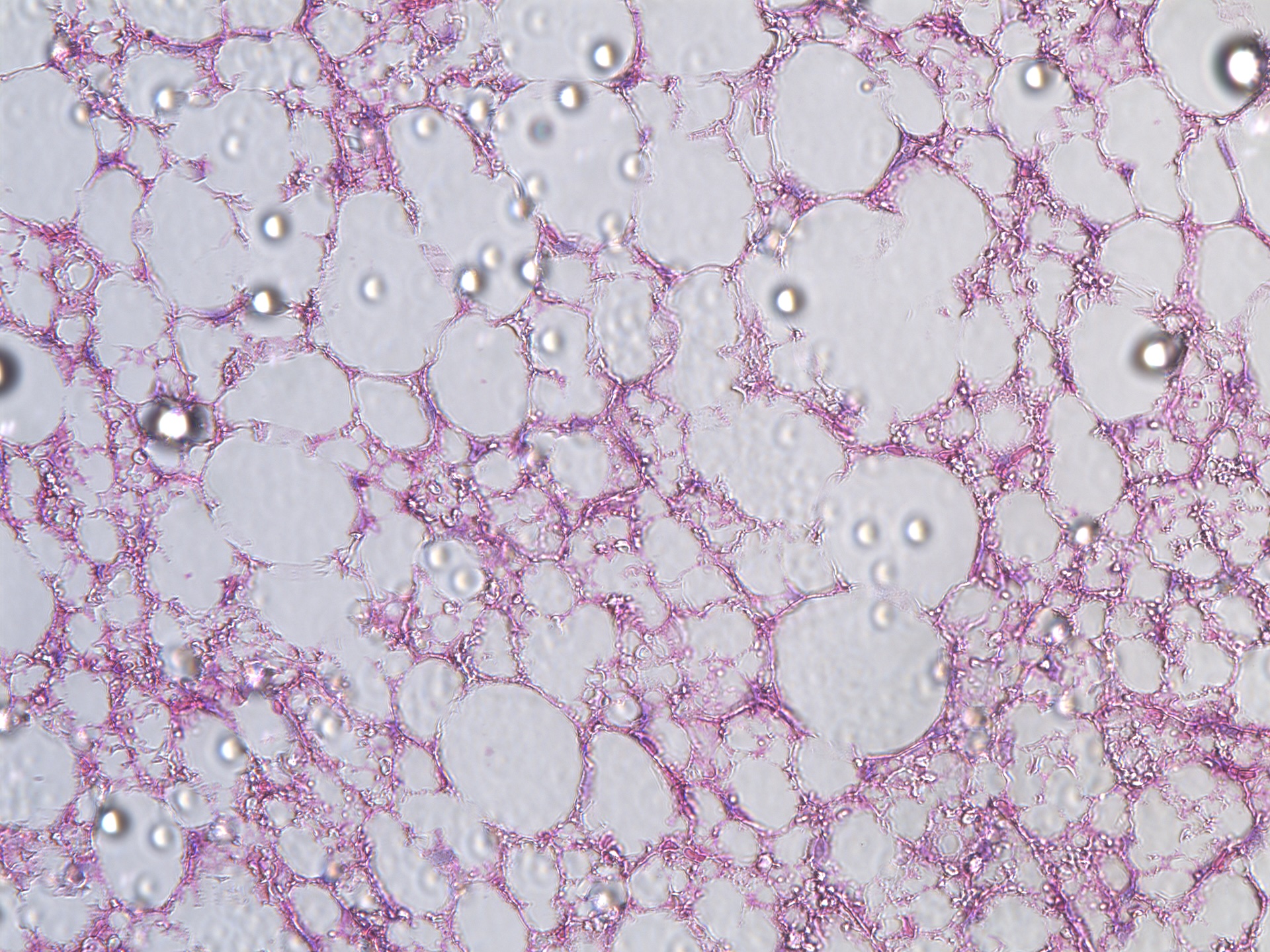

Supplement: Supplementary file 9 — Figure EV3 Source Data [file 44318_2024_196_MOESM9_ESM.zip › Figure EV3/Figure EV3-O/Additional replicate image/HFD Con/no.2/HFD Con-x40_03.jpg]

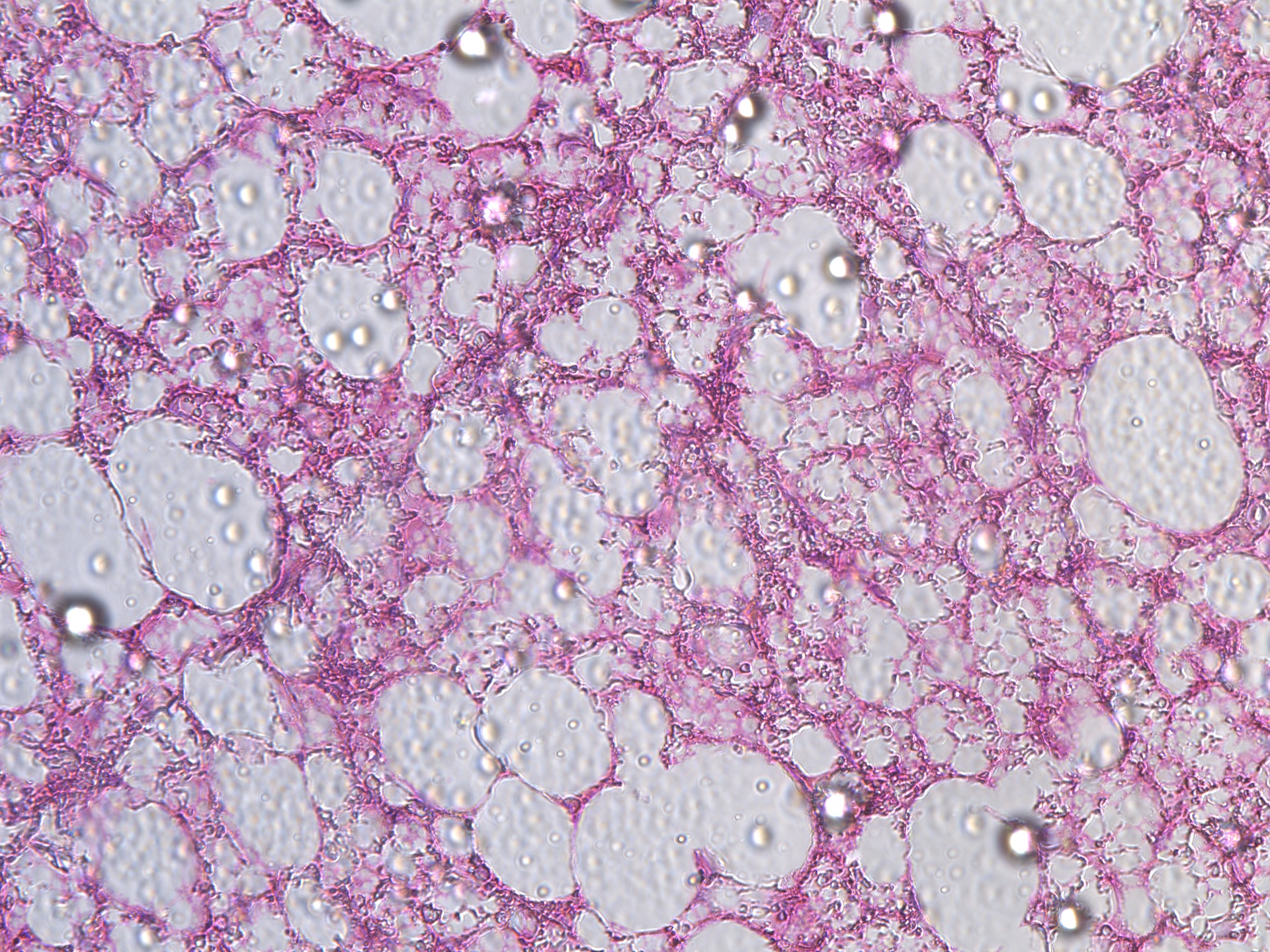

Supplement: Supplementary file 9 — Figure EV3 Source Data [file 44318_2024_196_MOESM9_ESM.zip › Figure EV3/Figure EV3-O/Additional replicate image/HFD Con/no.2/HFD Con-x40_01.jpg]

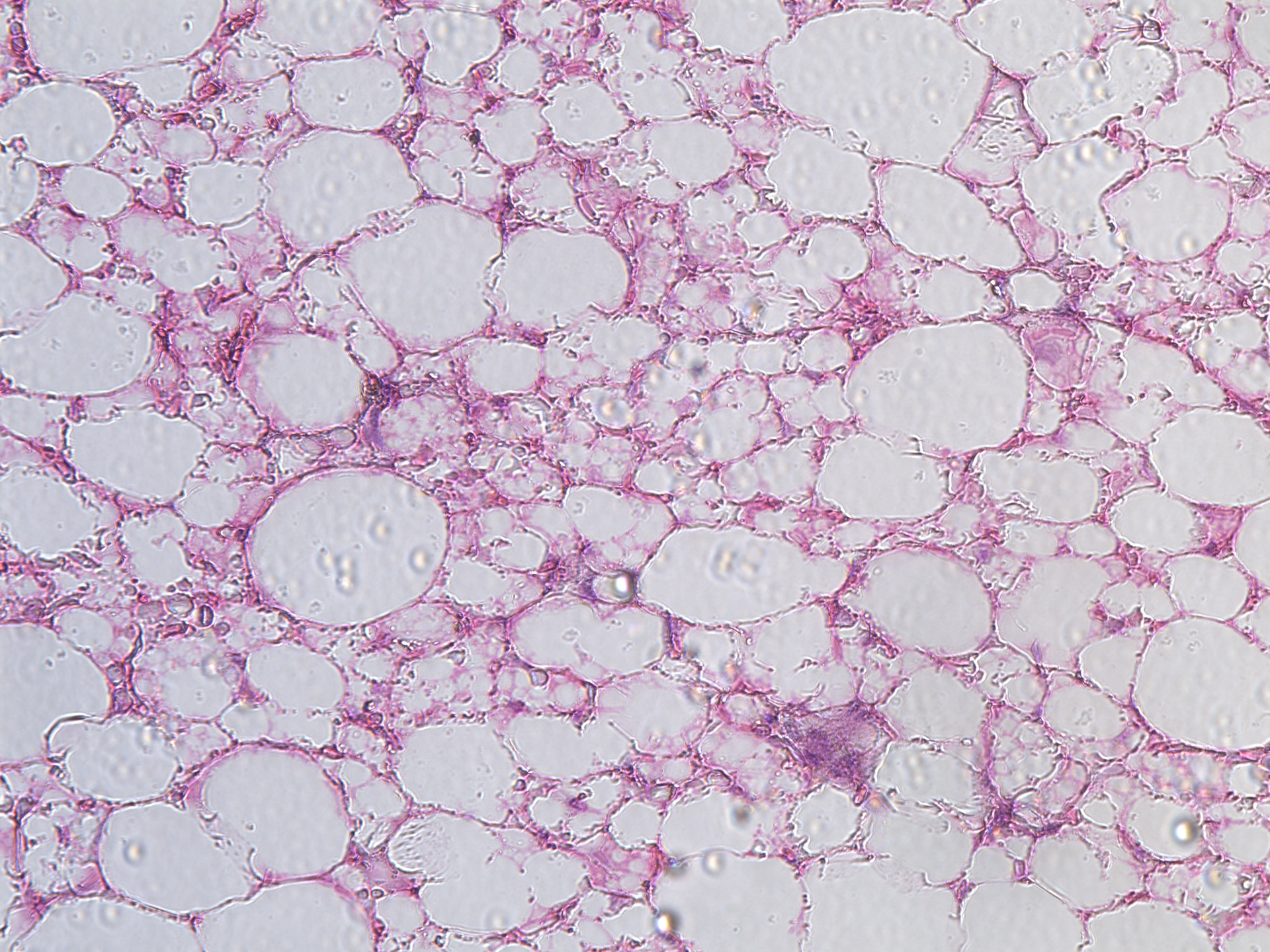

Supplement: Supplementary file 9 — Figure EV3 Source Data [file 44318_2024_196_MOESM9_ESM.zip › Figure EV3/Figure EV3-O/Additional replicate image/HFD PCPE-1 vaccine/no.1/HFD PCPE-1 vaacine-x40_04.jpg]

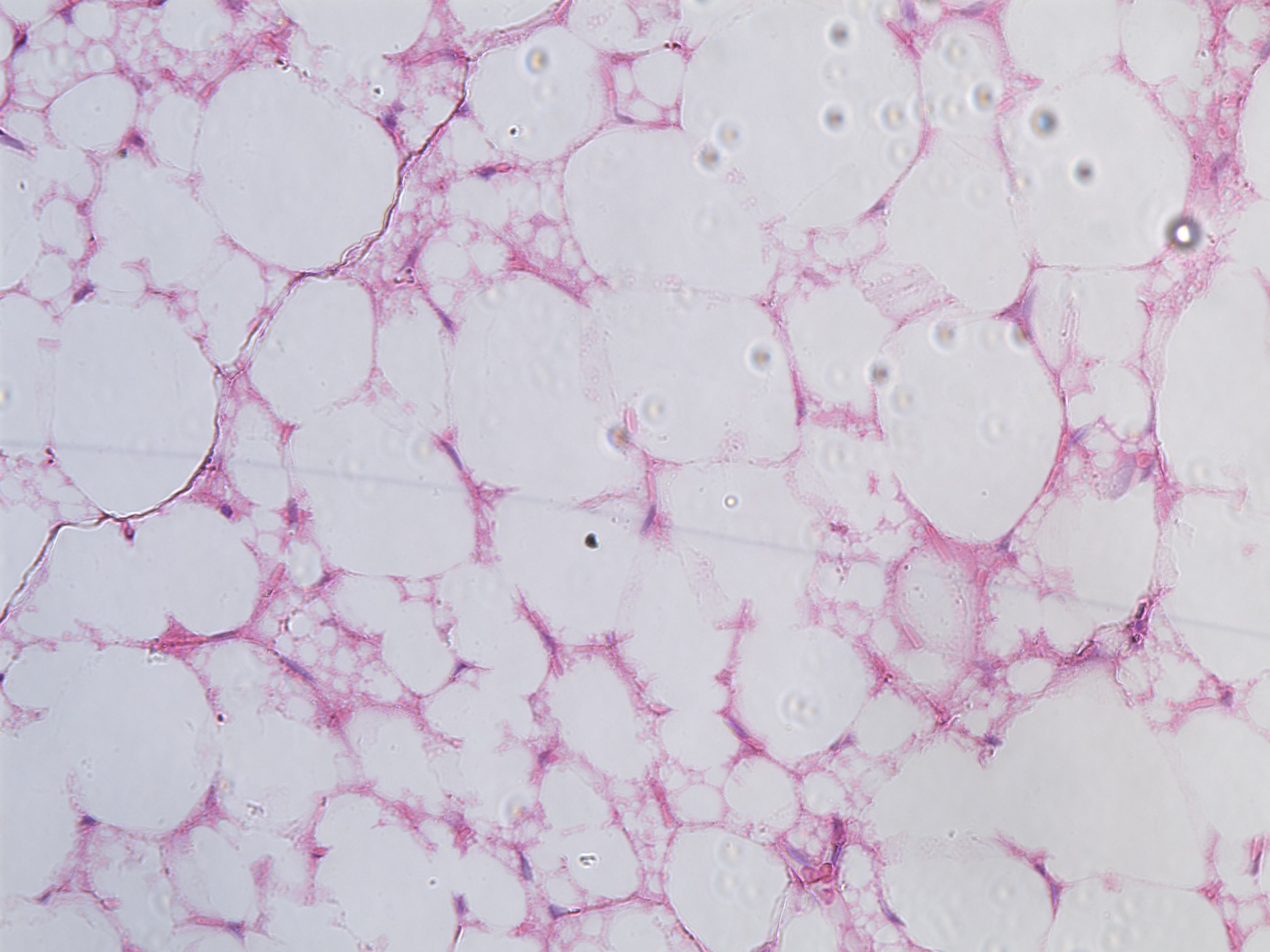

Supplement: Supplementary file 9 — Figure EV3 Source Data [file 44318_2024_196_MOESM9_ESM.zip › Figure EV3/Figure EV3-O/Additional replicate image/HFD PCPE-1 vaccine/no.1/HFD PCPE-1 vaacine-x40_05.jpg]

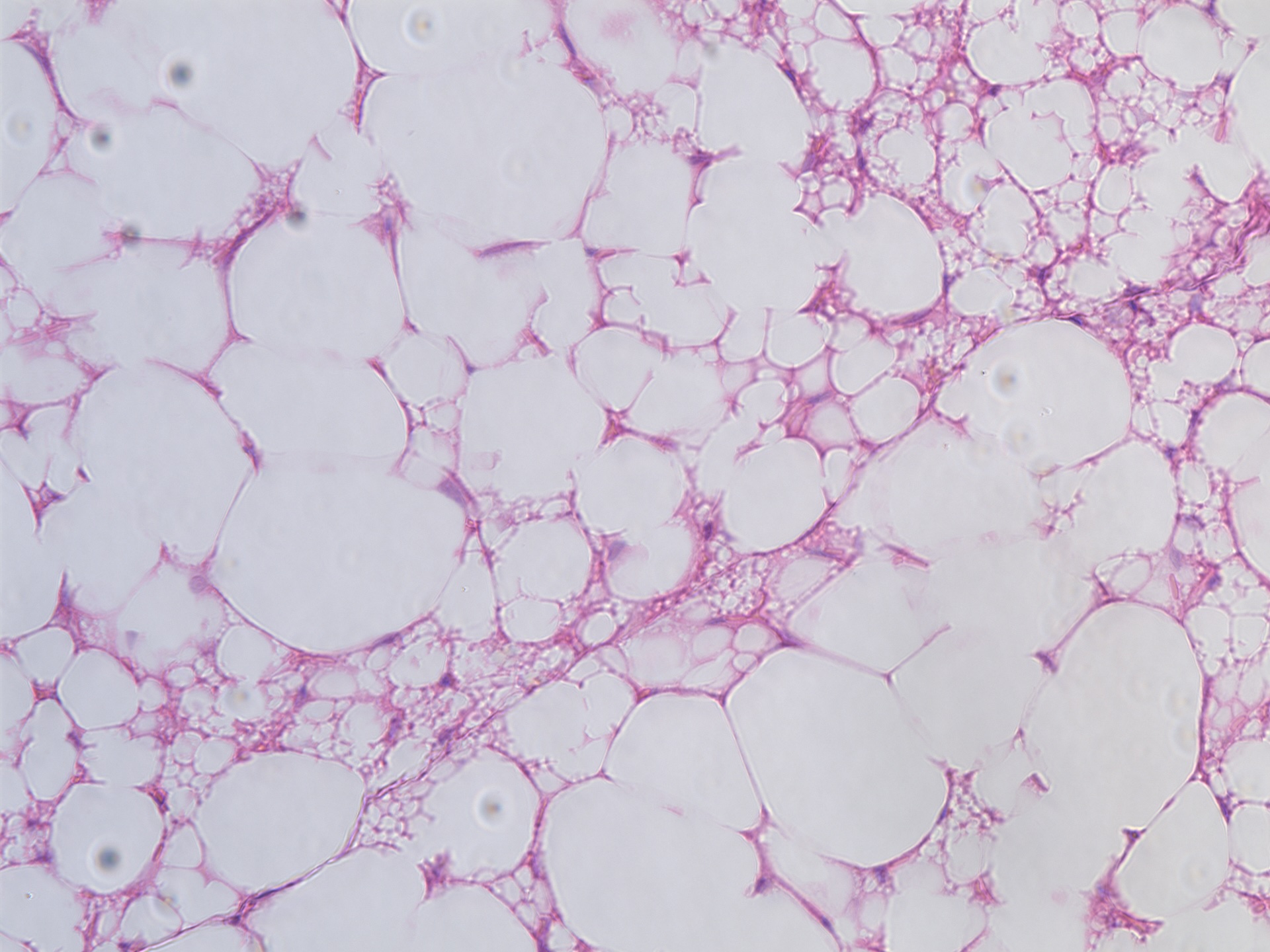

Supplement: Supplementary file 9 — Figure EV3 Source Data [file 44318_2024_196_MOESM9_ESM.zip › Figure EV3/Figure EV3-O/Additional replicate image/HFD PCPE-1 vaccine/no.1/HFD PCPE-1 vaacine-x40_01.jpg]

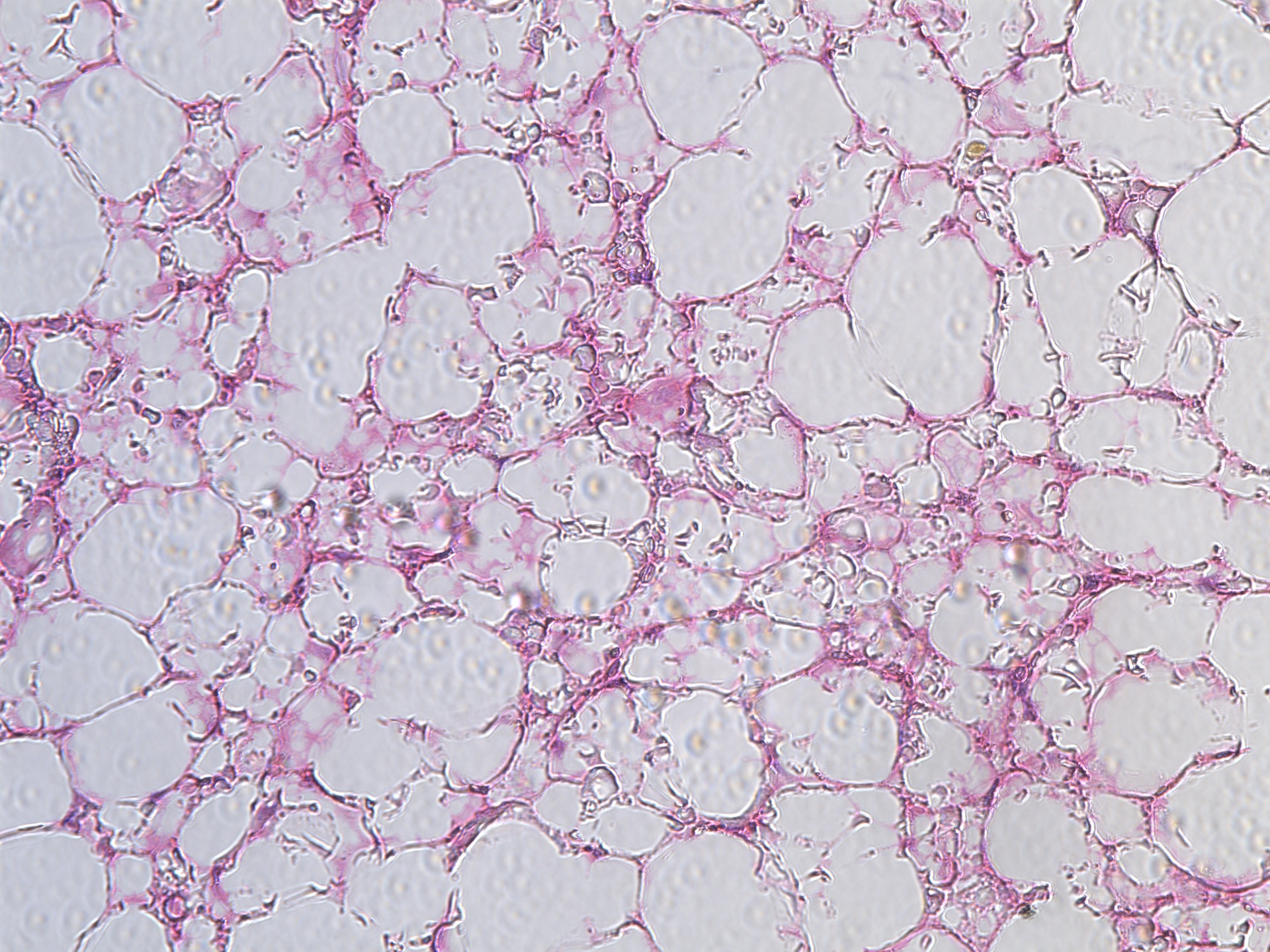

Supplement: Supplementary file 9 — Figure EV3 Source Data [file 44318_2024_196_MOESM9_ESM.zip › Figure EV3/Figure EV3-O/Additional replicate image/HFD PCPE-1 vaccine/no.1/HFD PCPE-1 vaacine-x40_02.jpg]

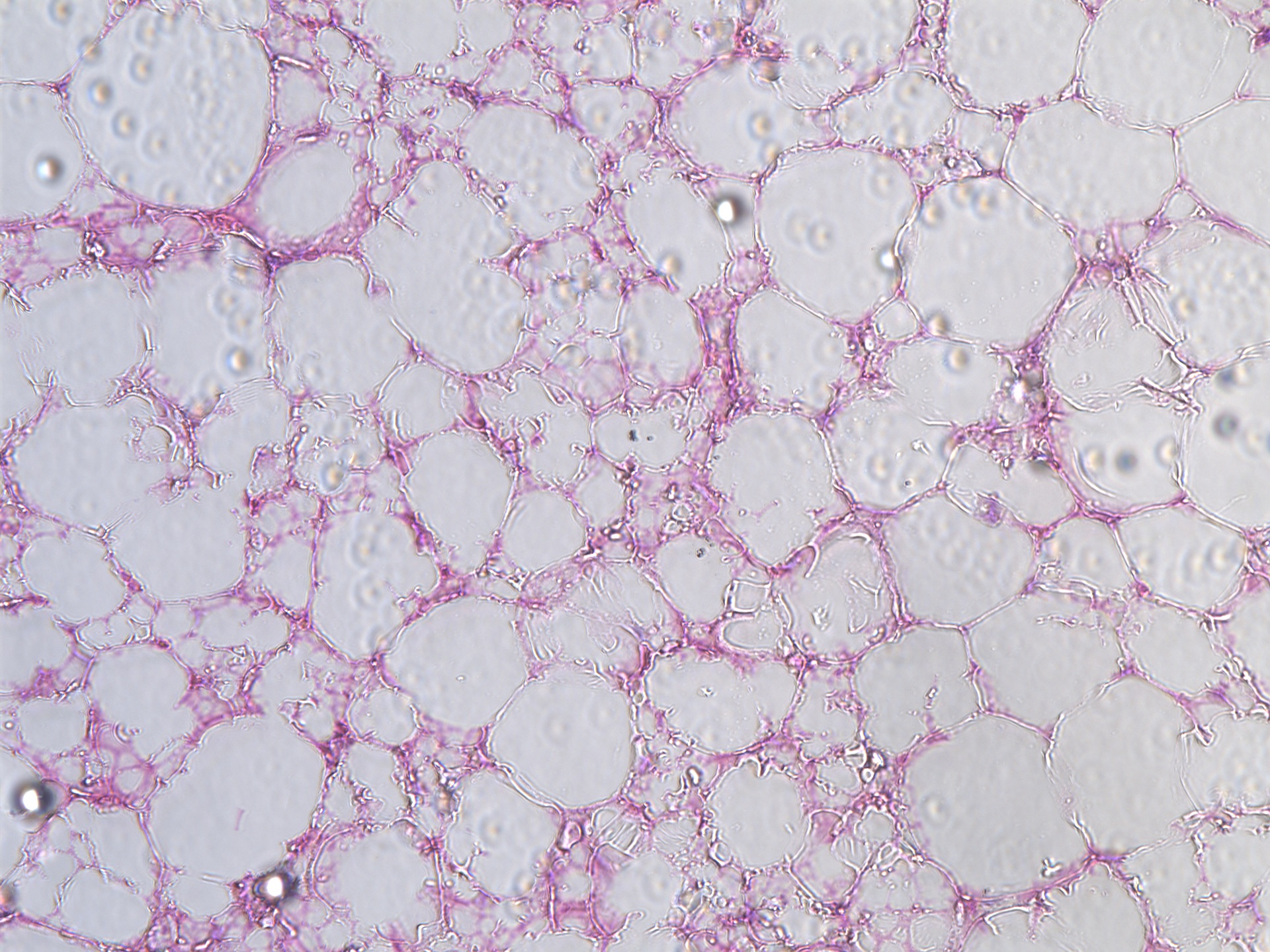

Supplement: Supplementary file 9 — Figure EV3 Source Data [file 44318_2024_196_MOESM9_ESM.zip › Figure EV3/Figure EV3-O/Additional replicate image/HFD PCPE-1 vaccine/no.1/HFD PCPE-1 vaacine-x40_03.jpg]

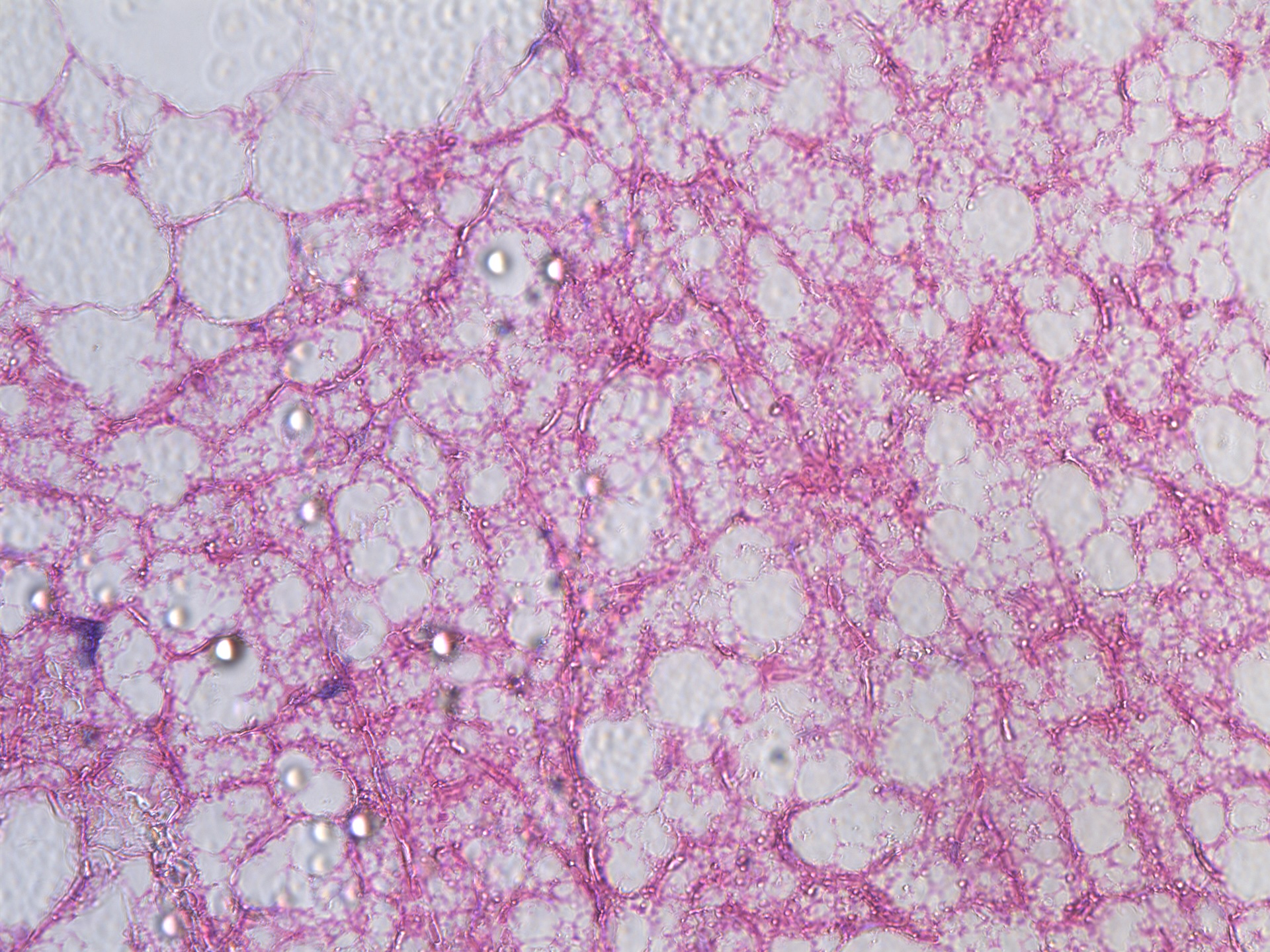

Supplement: Supplementary file 9 — Figure EV3 Source Data [file 44318_2024_196_MOESM9_ESM.zip › Figure EV3/Figure EV3-O/Additional replicate image/HFD PCPE-1 vaccine/no.3/HFD PCPE-1 vaacine-x40_04.jpg]

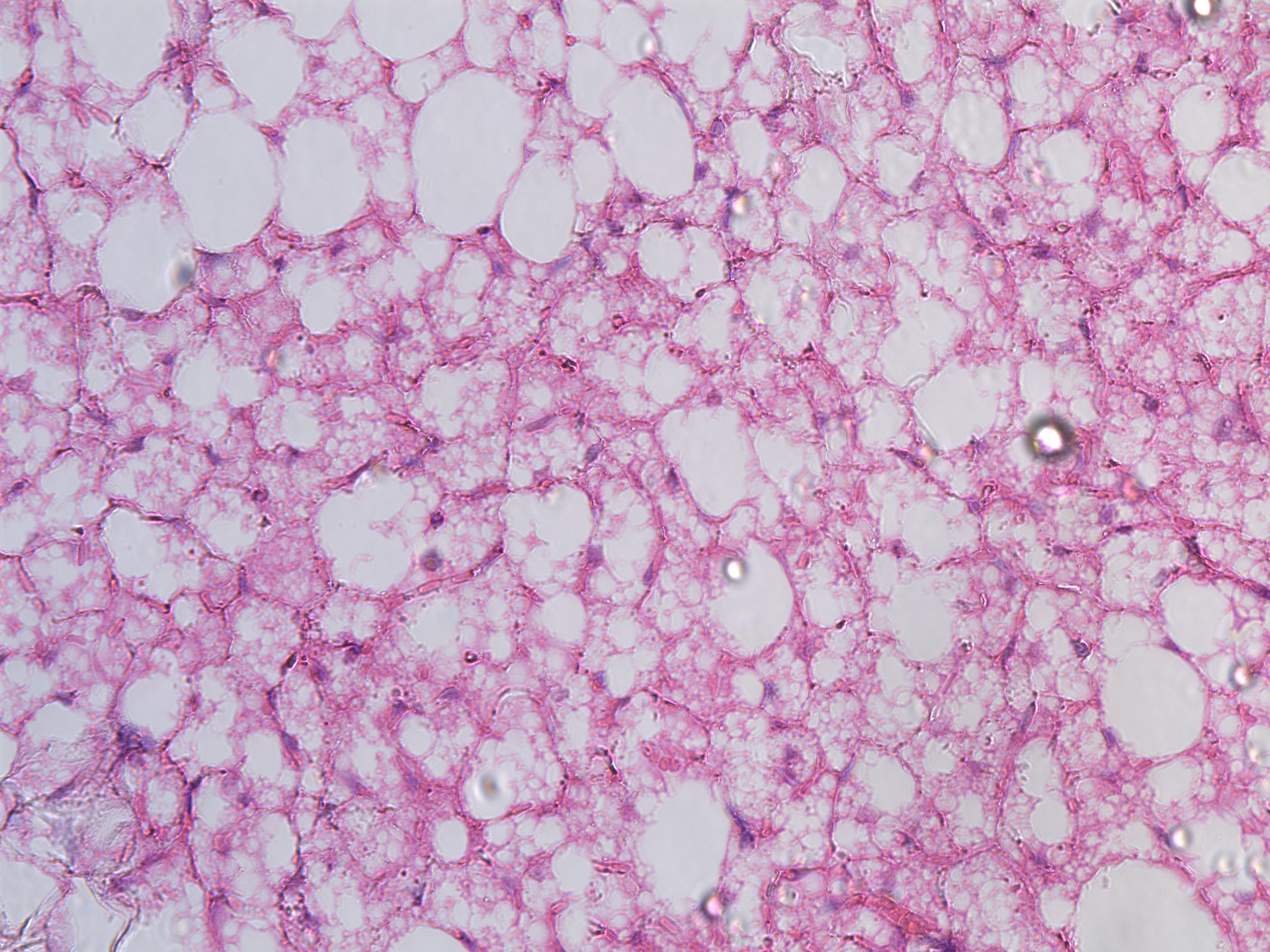

Supplement: Supplementary file 9 — Figure EV3 Source Data [file 44318_2024_196_MOESM9_ESM.zip › Figure EV3/Figure EV3-O/Additional replicate image/HFD PCPE-1 vaccine/no.3/HFD PCPE-1 vaacine-x40_05.jpg]

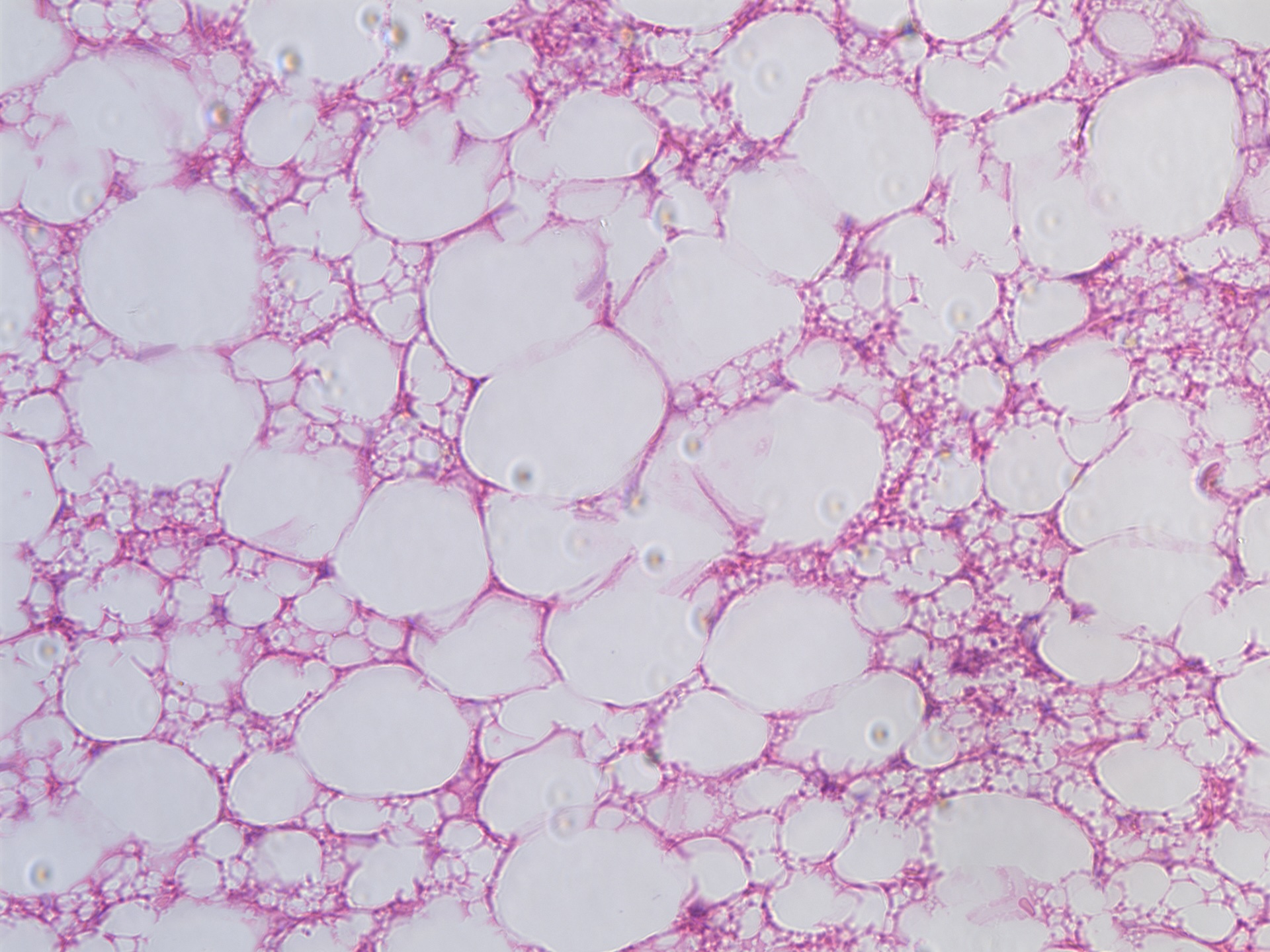

Supplement: Supplementary file 9 — Figure EV3 Source Data [file 44318_2024_196_MOESM9_ESM.zip › Figure EV3/Figure EV3-O/Additional replicate image/HFD PCPE-1 vaccine/no.3/HFD PCPE-1 vaacine-x40_01.jpg]

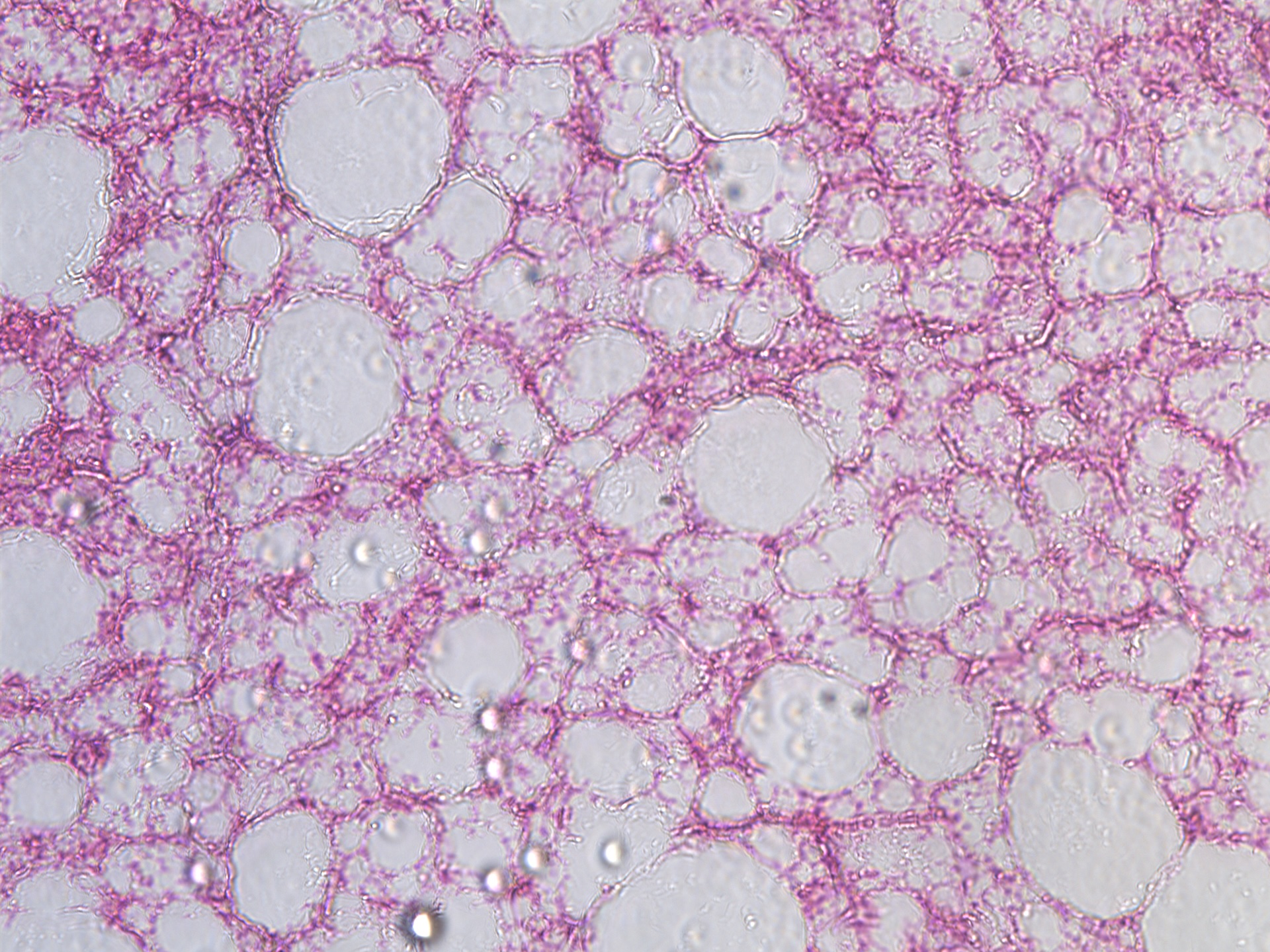

Supplement: Supplementary file 9 — Figure EV3 Source Data [file 44318_2024_196_MOESM9_ESM.zip › Figure EV3/Figure EV3-O/Additional replicate image/HFD PCPE-1 vaccine/no.3/HFD PCPE-1 vaacine-x40_02.jpg]

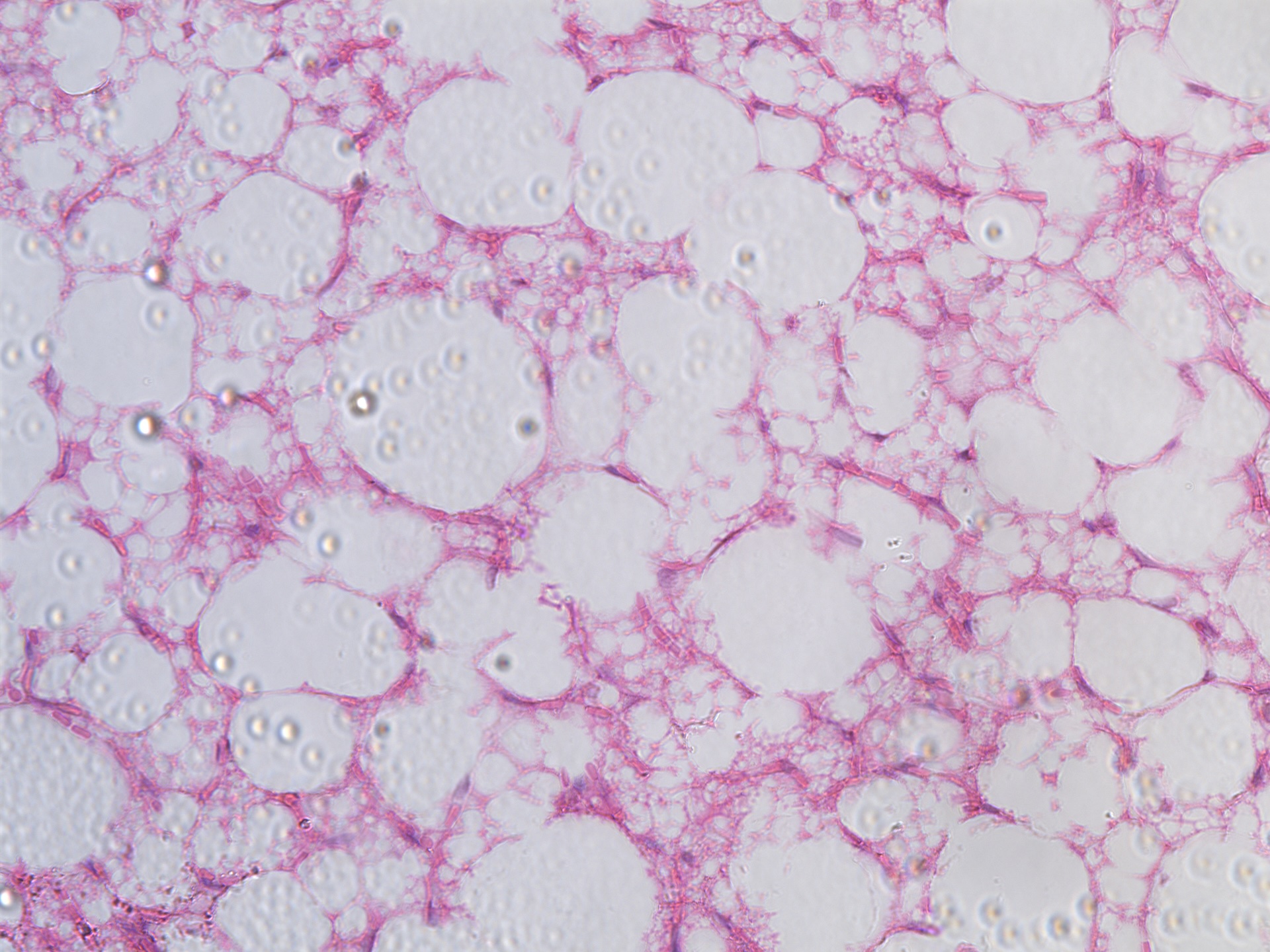

Supplement: Supplementary file 9 — Figure EV3 Source Data [file 44318_2024_196_MOESM9_ESM.zip › Figure EV3/Figure EV3-O/Additional replicate image/HFD PCPE-1 vaccine/no.3/HFD PCPE-1 vaacine-x40_03.jpg]

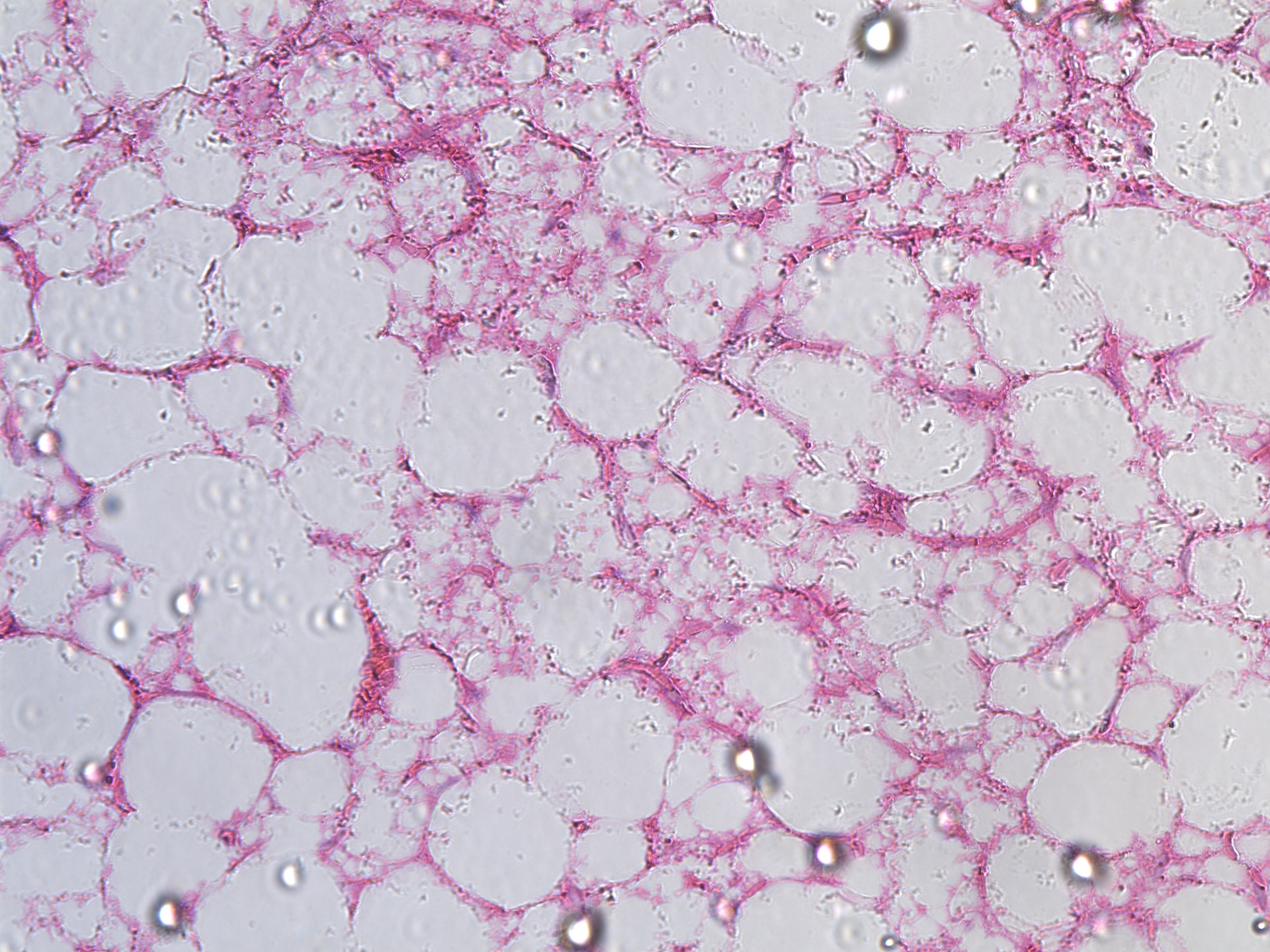

Supplement: Supplementary file 9 — Figure EV3 Source Data [file 44318_2024_196_MOESM9_ESM.zip › Figure EV3/Figure EV3-O/Additional replicate image/HFD PCPE-1 vaccine/no.4/HFD PCPE-1 vaacine-x40_04.jpg]

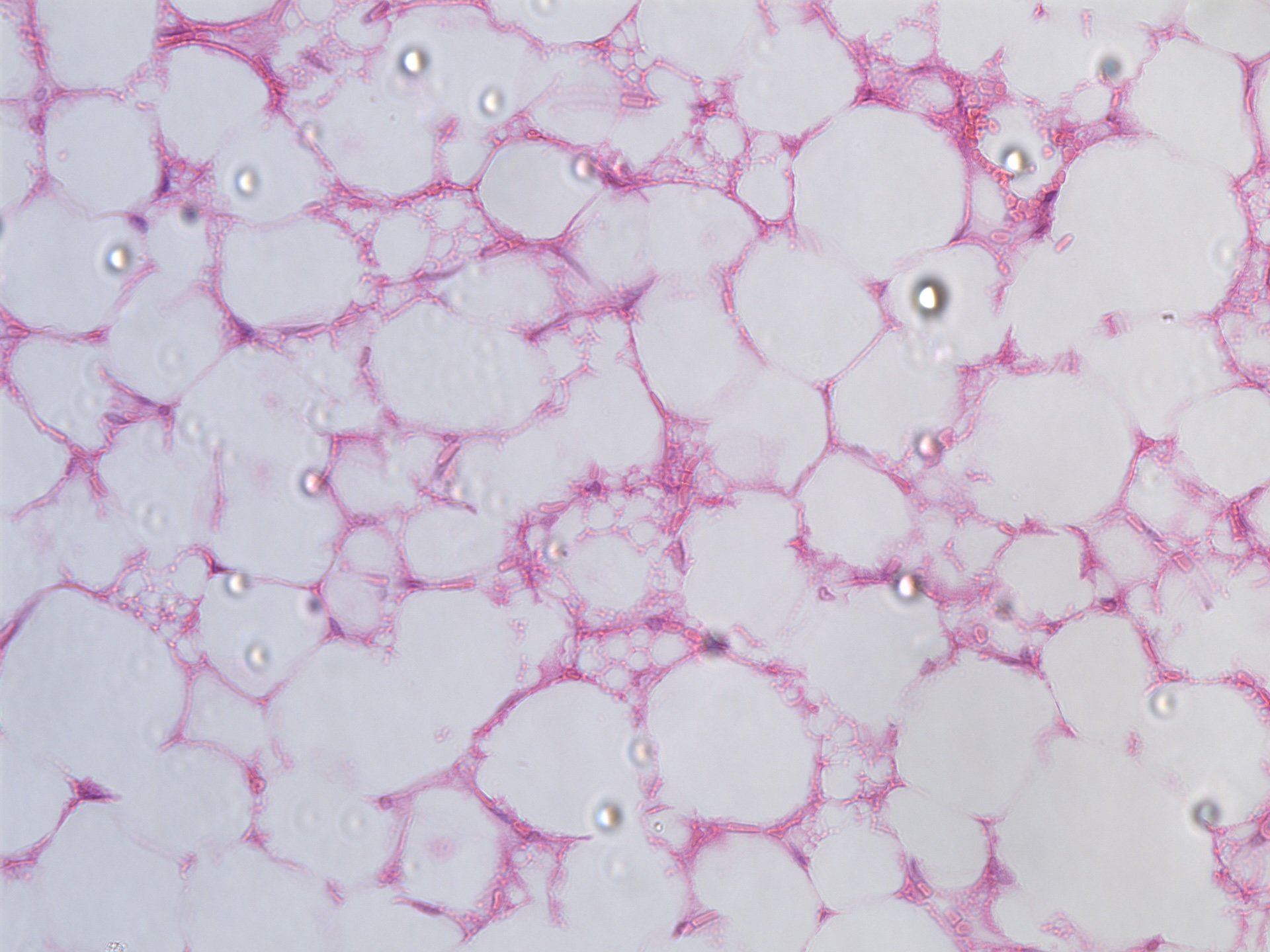

Supplement: Supplementary file 9 — Figure EV3 Source Data [file 44318_2024_196_MOESM9_ESM.zip › Figure EV3/Figure EV3-O/Additional replicate image/HFD PCPE-1 vaccine/no.4/HFD PCPE-1 vaacine-x40_05.jpg]

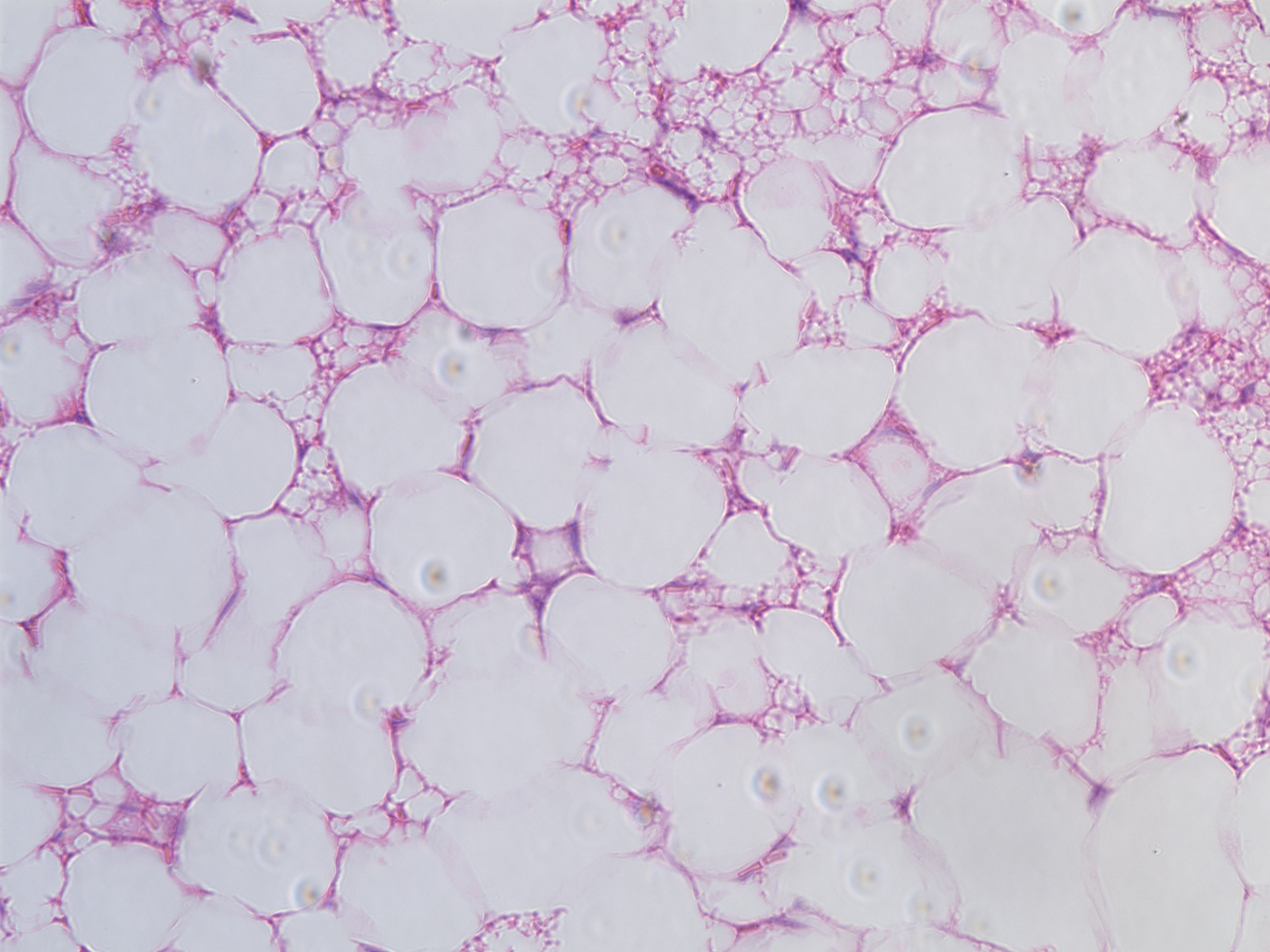

Supplement: Supplementary file 9 — Figure EV3 Source Data [file 44318_2024_196_MOESM9_ESM.zip › Figure EV3/Figure EV3-O/Additional replicate image/HFD PCPE-1 vaccine/no.4/HFD PCPE-1 vaacine-x40_01.jpg]

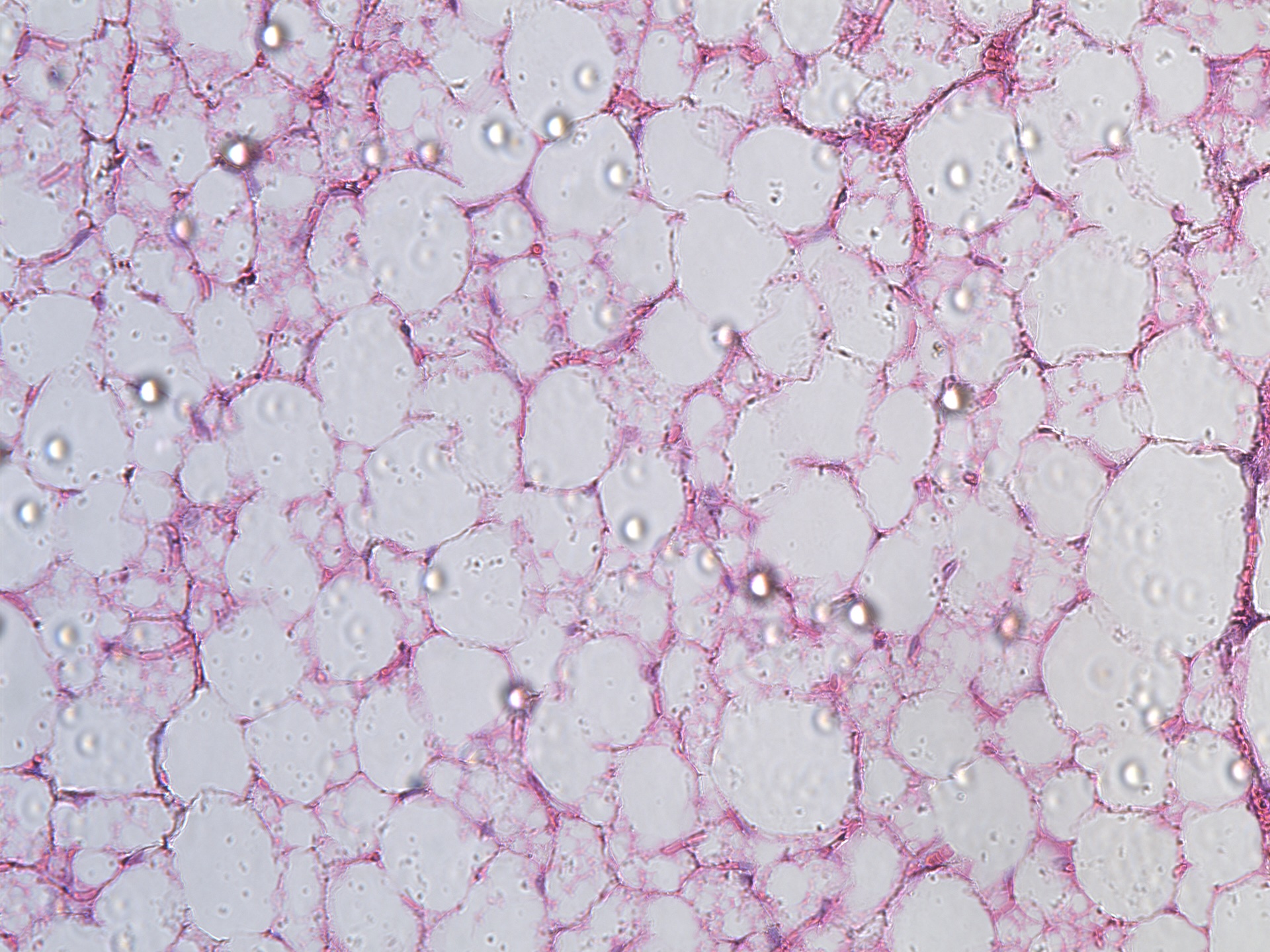

Supplement: Supplementary file 9 — Figure EV3 Source Data [file 44318_2024_196_MOESM9_ESM.zip › Figure EV3/Figure EV3-O/Additional replicate image/HFD PCPE-1 vaccine/no.4/HFD PCPE-1 vaacine-x40_02.jpg]

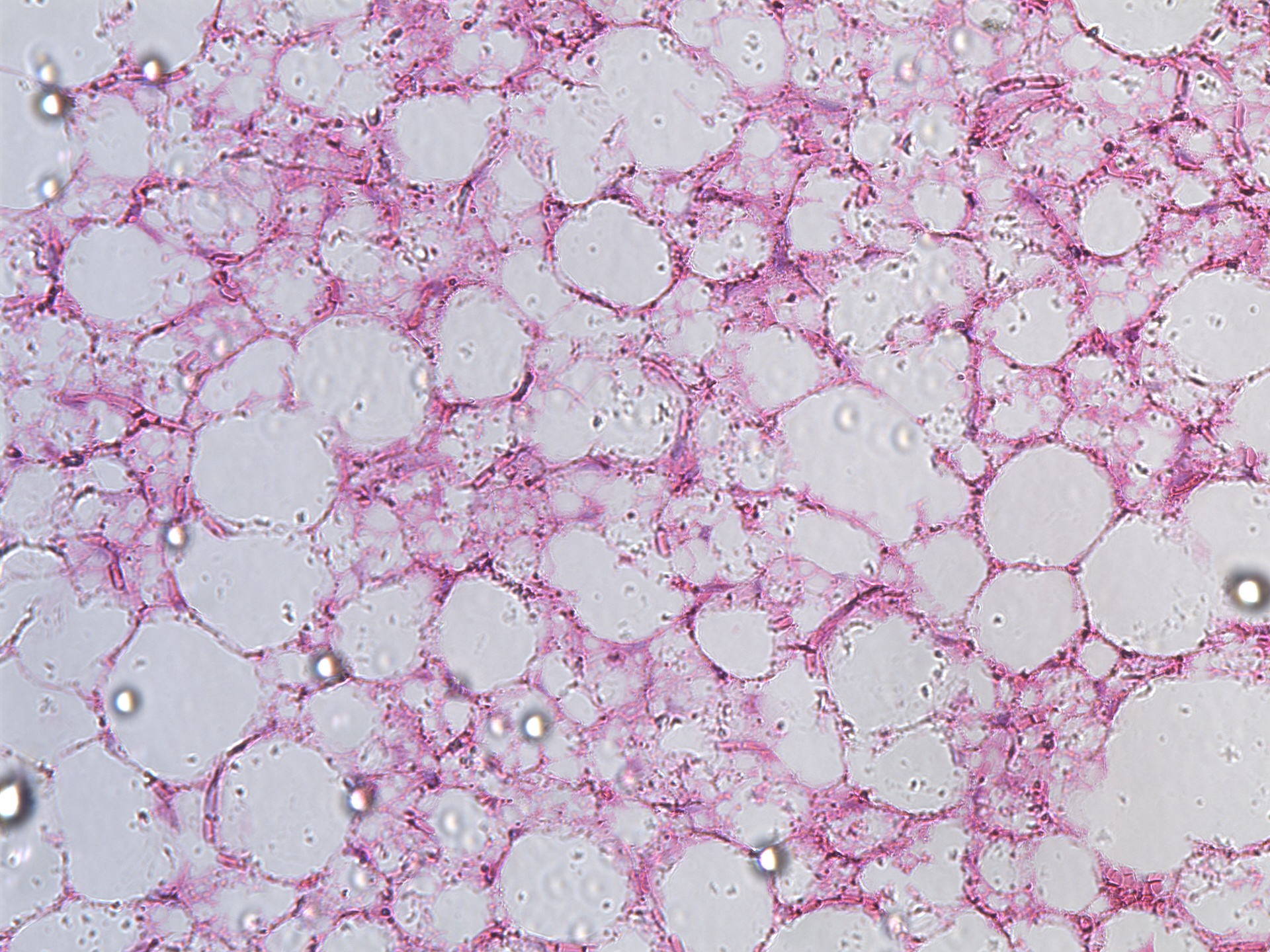

Supplement: Supplementary file 9 — Figure EV3 Source Data [file 44318_2024_196_MOESM9_ESM.zip › Figure EV3/Figure EV3-O/Additional replicate image/HFD PCPE-1 vaccine/no.4/HFD PCPE-1 vaacine-x40_03.jpg]

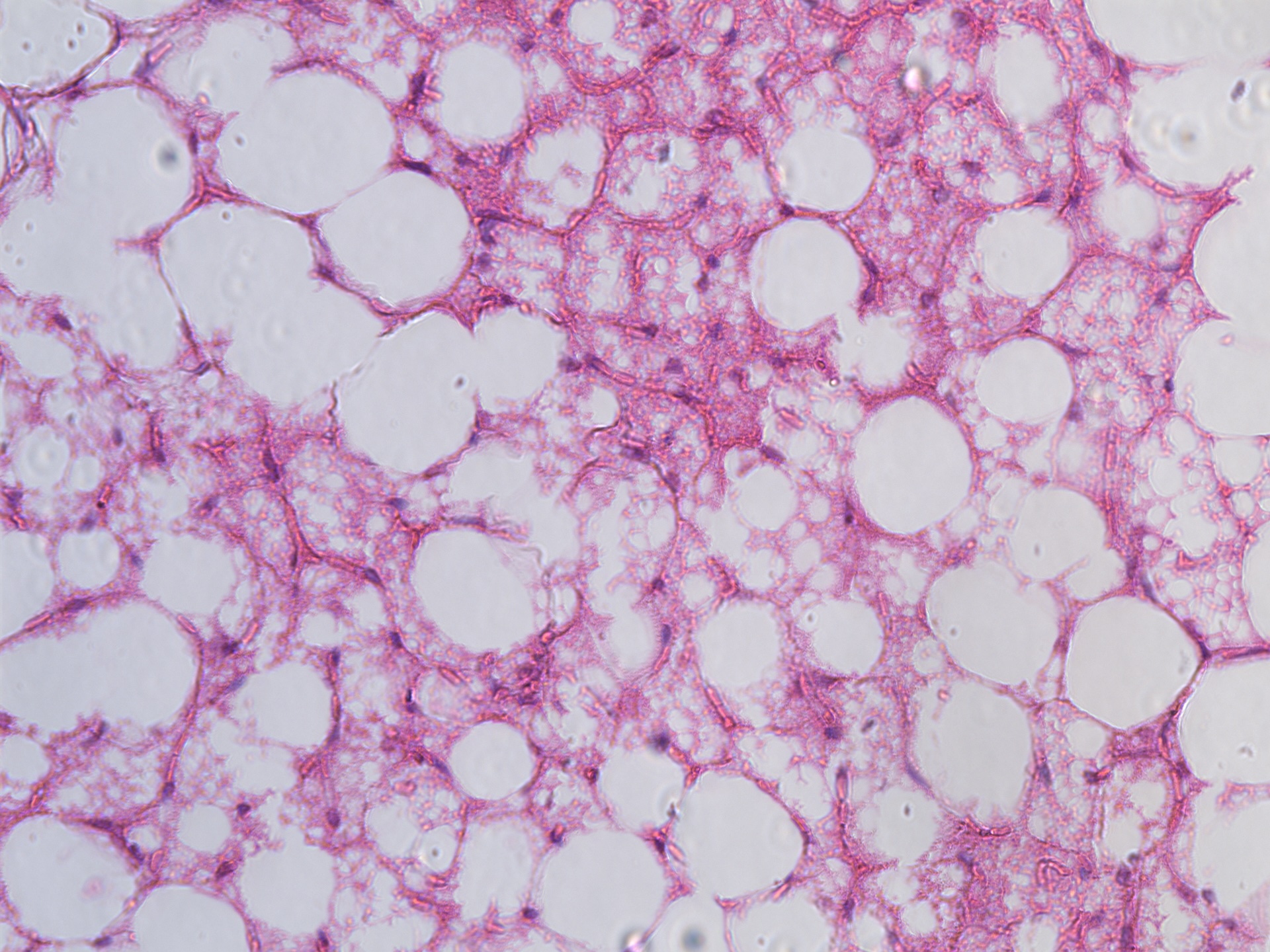

Supplement: Supplementary file 9 — Figure EV3 Source Data [file 44318_2024_196_MOESM9_ESM.zip › Figure EV3/Figure EV3-O/Additional replicate image/HFD PCPE-1 vaccine/no.5/HFD PCPE-1 vaacine-x40_04.jpg]

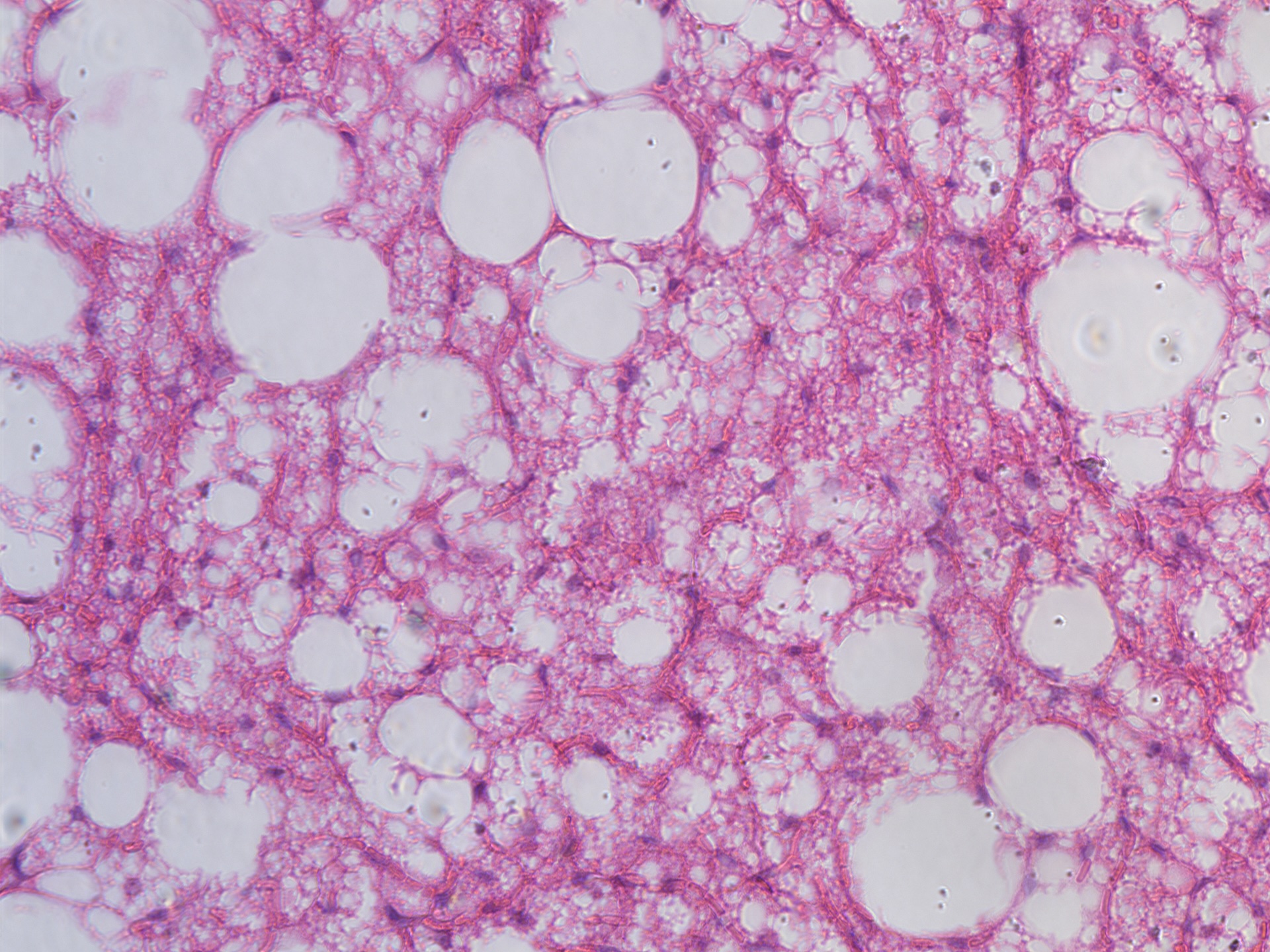

Supplement: Supplementary file 9 — Figure EV3 Source Data [file 44318_2024_196_MOESM9_ESM.zip › Figure EV3/Figure EV3-O/Additional replicate image/HFD PCPE-1 vaccine/no.5/HFD PCPE-1 vaacine-x40_05.jpg]

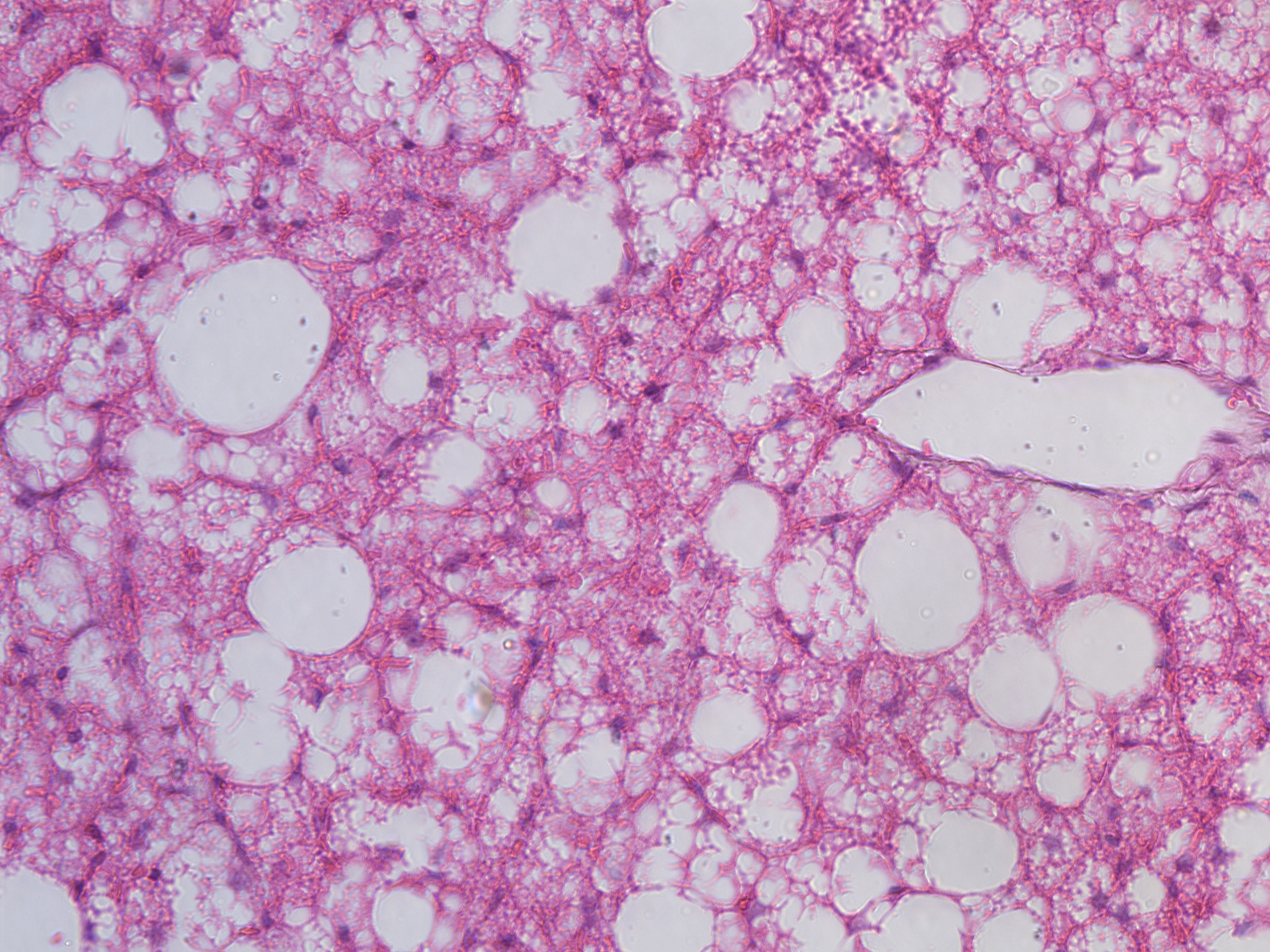

Supplement: Supplementary file 9 — Figure EV3 Source Data [file 44318_2024_196_MOESM9_ESM.zip › Figure EV3/Figure EV3-O/Additional replicate image/HFD PCPE-1 vaccine/no.5/HFD PCPE-1 vaacine-x40_01.jpg]

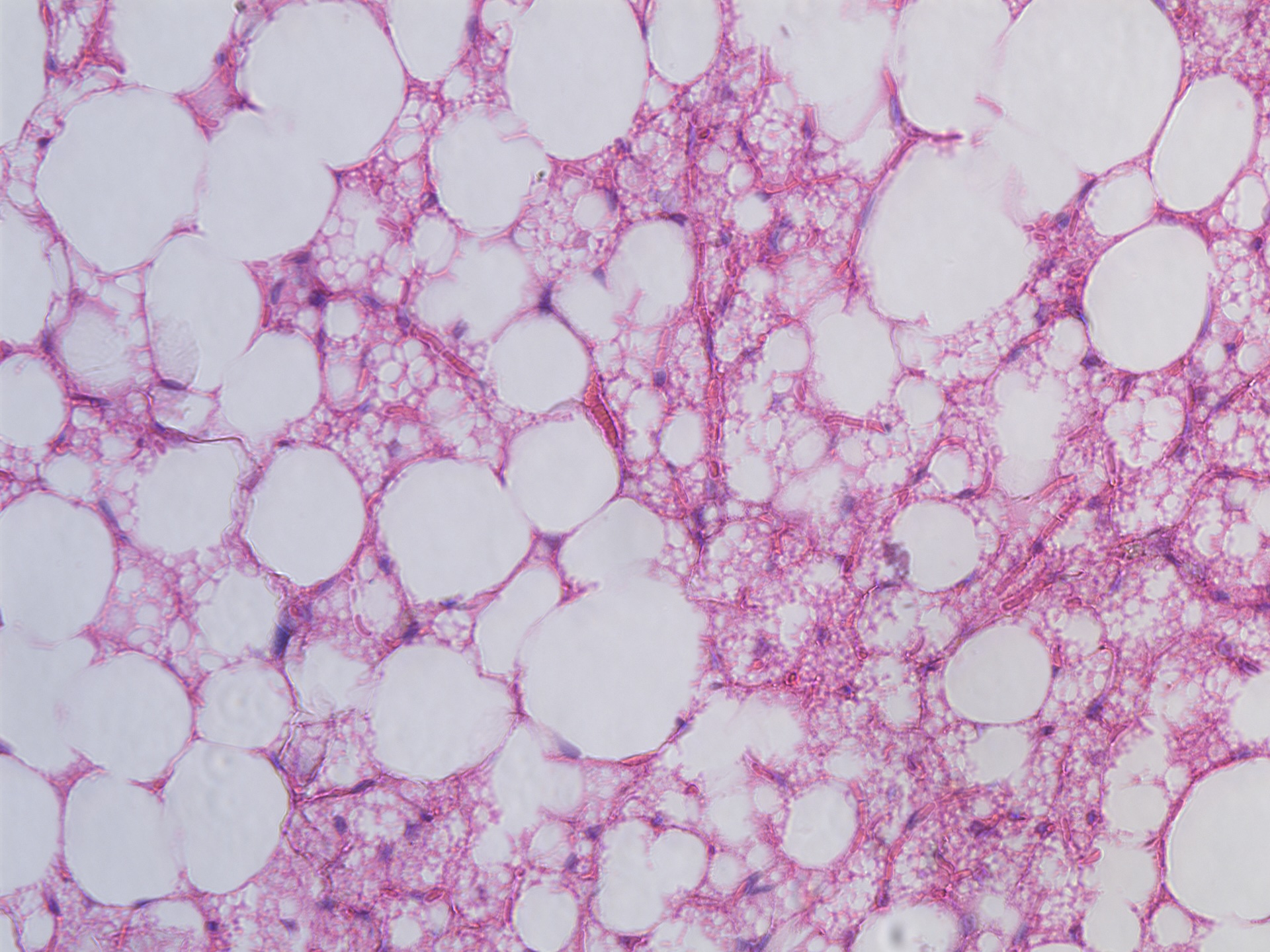

Supplement: Supplementary file 9 — Figure EV3 Source Data [file 44318_2024_196_MOESM9_ESM.zip › Figure EV3/Figure EV3-O/Additional replicate image/HFD PCPE-1 vaccine/no.5/HFD PCPE-1 vaacine-x40_02.jpg]

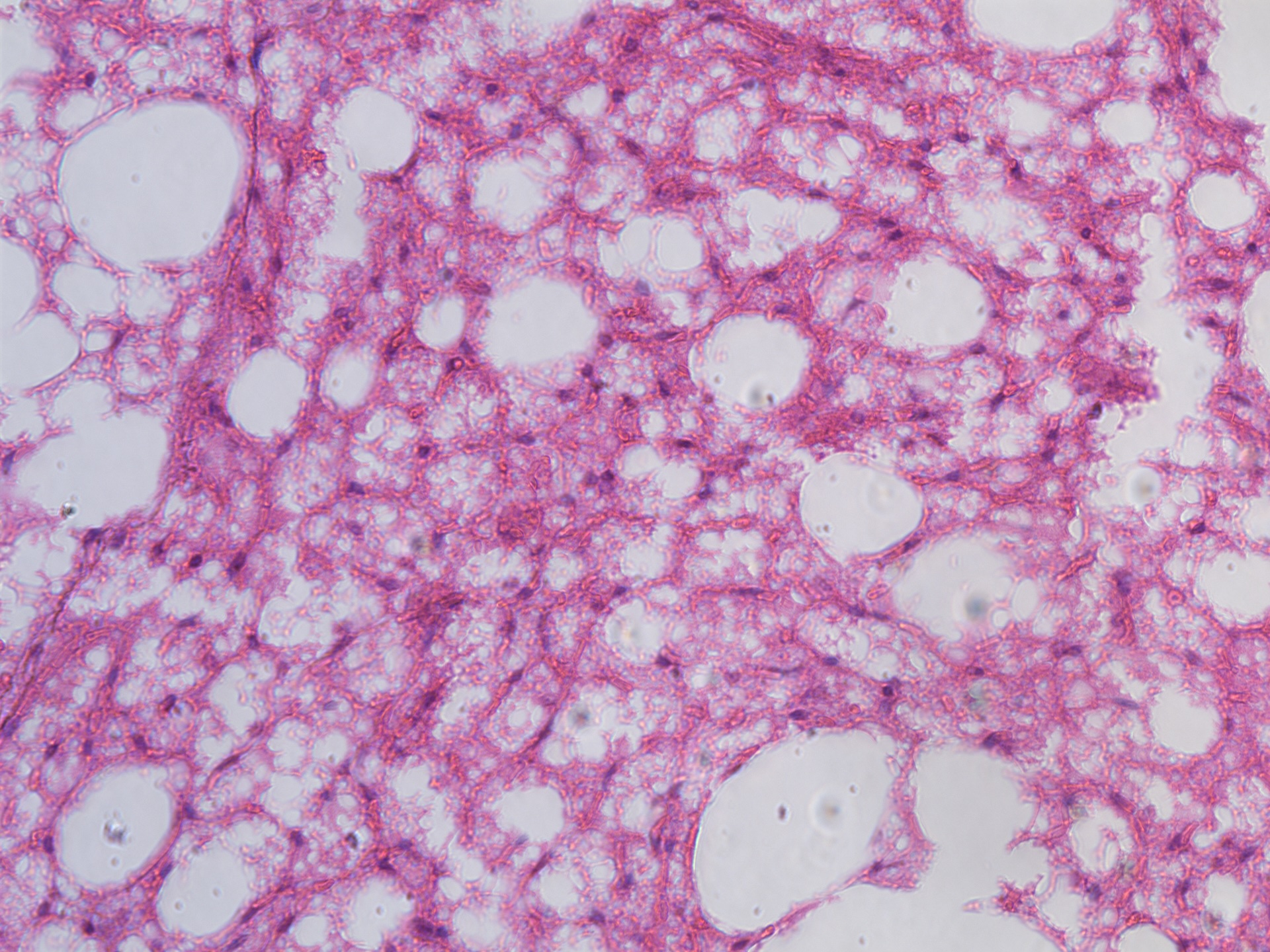

Supplement: Supplementary file 9 — Figure EV3 Source Data [file 44318_2024_196_MOESM9_ESM.zip › Figure EV3/Figure EV3-O/Additional replicate image/HFD PCPE-1 vaccine/no.5/HFD PCPE-1 vaacine-x40_03.jpg]

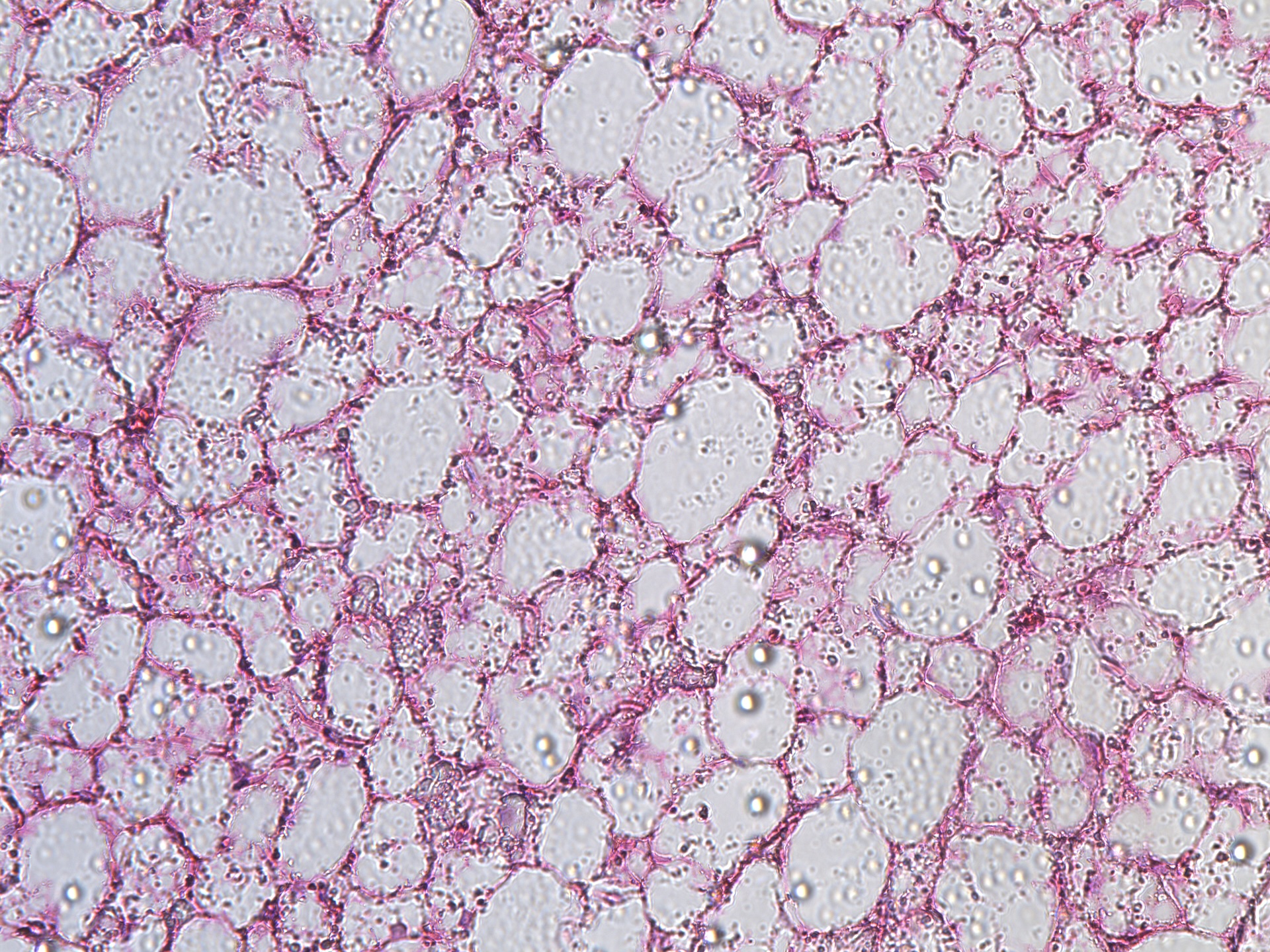

Supplement: Supplementary file 9 — Figure EV3 Source Data [file 44318_2024_196_MOESM9_ESM.zip › Figure EV3/Figure EV3-O/Additional replicate image/HFD PCPE-1 vaccine/no.2/HFD PCPE-1 vaacine-x40_04.jpg]

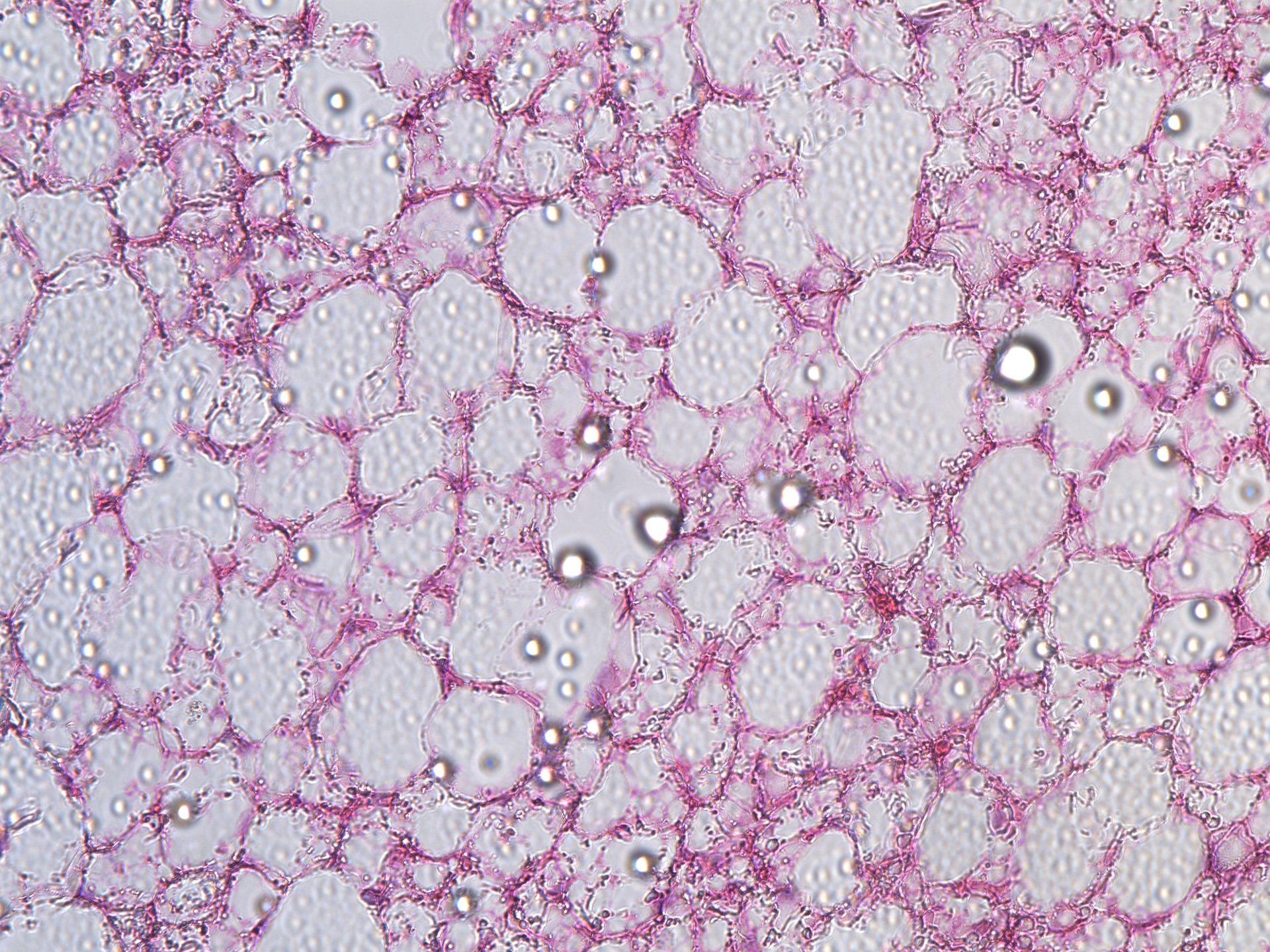

Supplement: Supplementary file 9 — Figure EV3 Source Data [file 44318_2024_196_MOESM9_ESM.zip › Figure EV3/Figure EV3-O/Additional replicate image/HFD PCPE-1 vaccine/no.2/HFD PCPE-1 vaacine-x40_05.jpg]

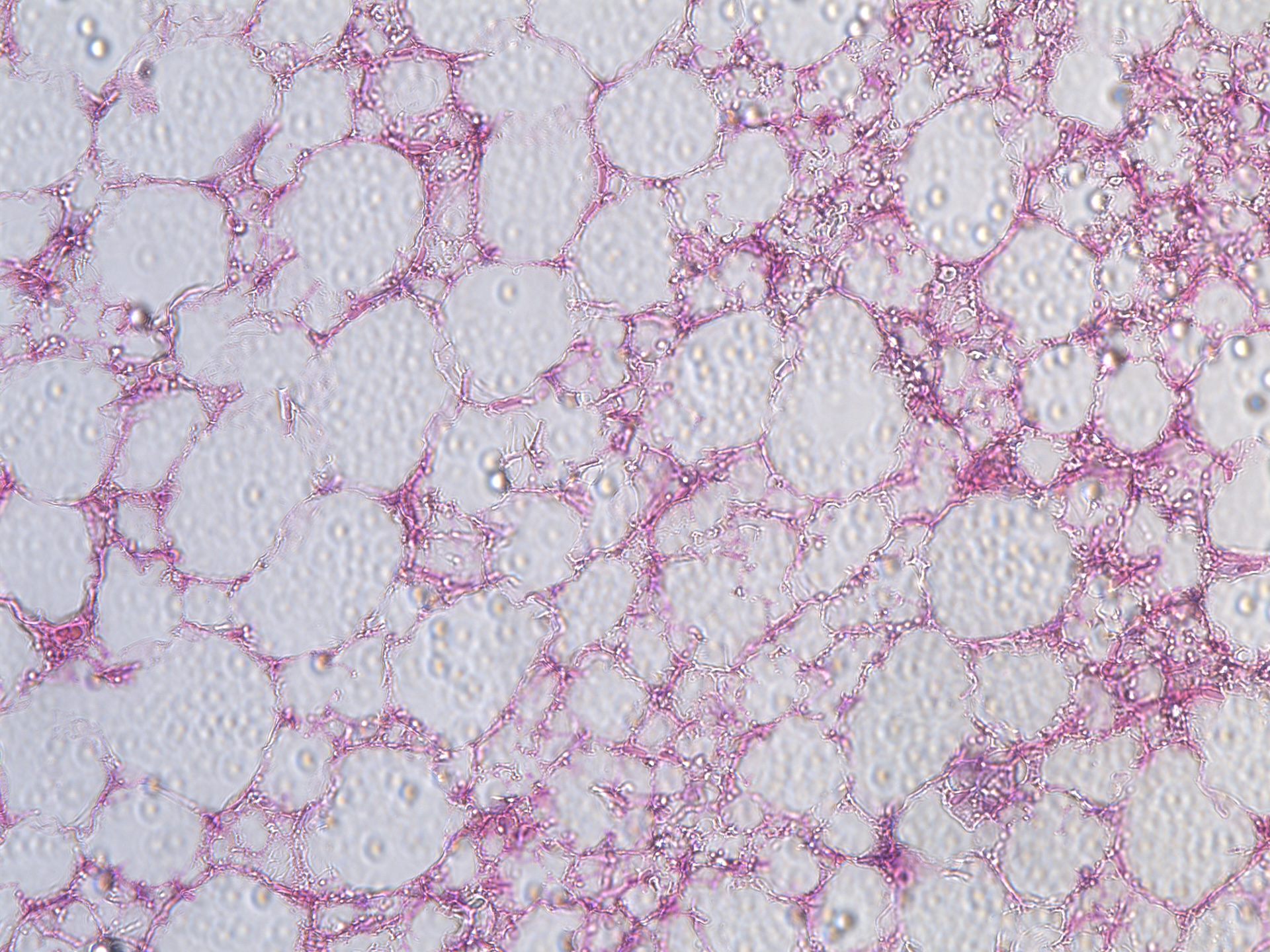

Supplement: Supplementary file 9 — Figure EV3 Source Data [file 44318_2024_196_MOESM9_ESM.zip › Figure EV3/Figure EV3-O/Additional replicate image/HFD PCPE-1 vaccine/no.2/HFD PCPE-1 vaacine-x40_01.jpg]

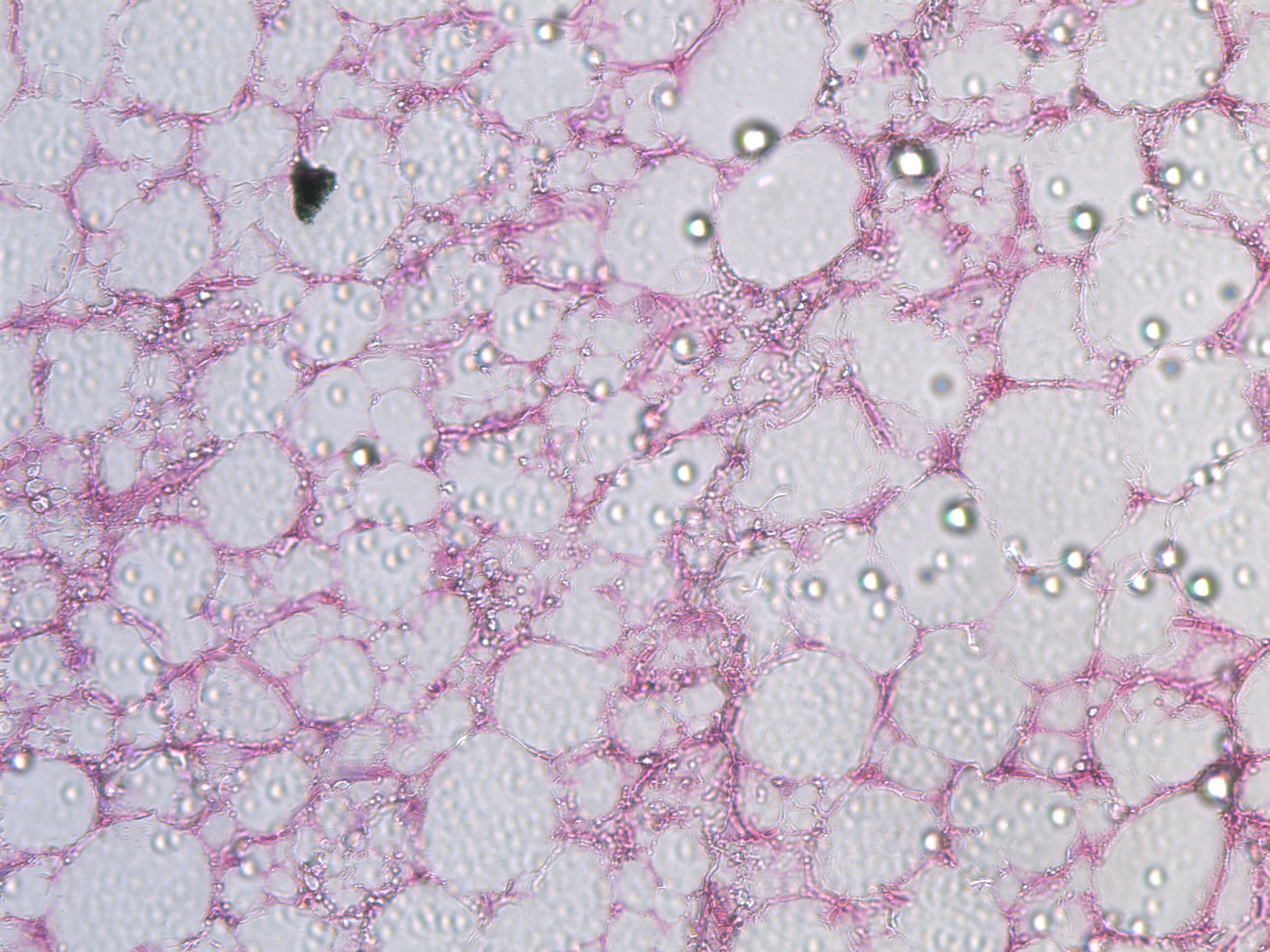

Supplement: Supplementary file 9 — Figure EV3 Source Data [file 44318_2024_196_MOESM9_ESM.zip › Figure EV3/Figure EV3-O/Additional replicate image/HFD PCPE-1 vaccine/no.2/HFD PCPE-1 vaacine-x40_02.jpg]

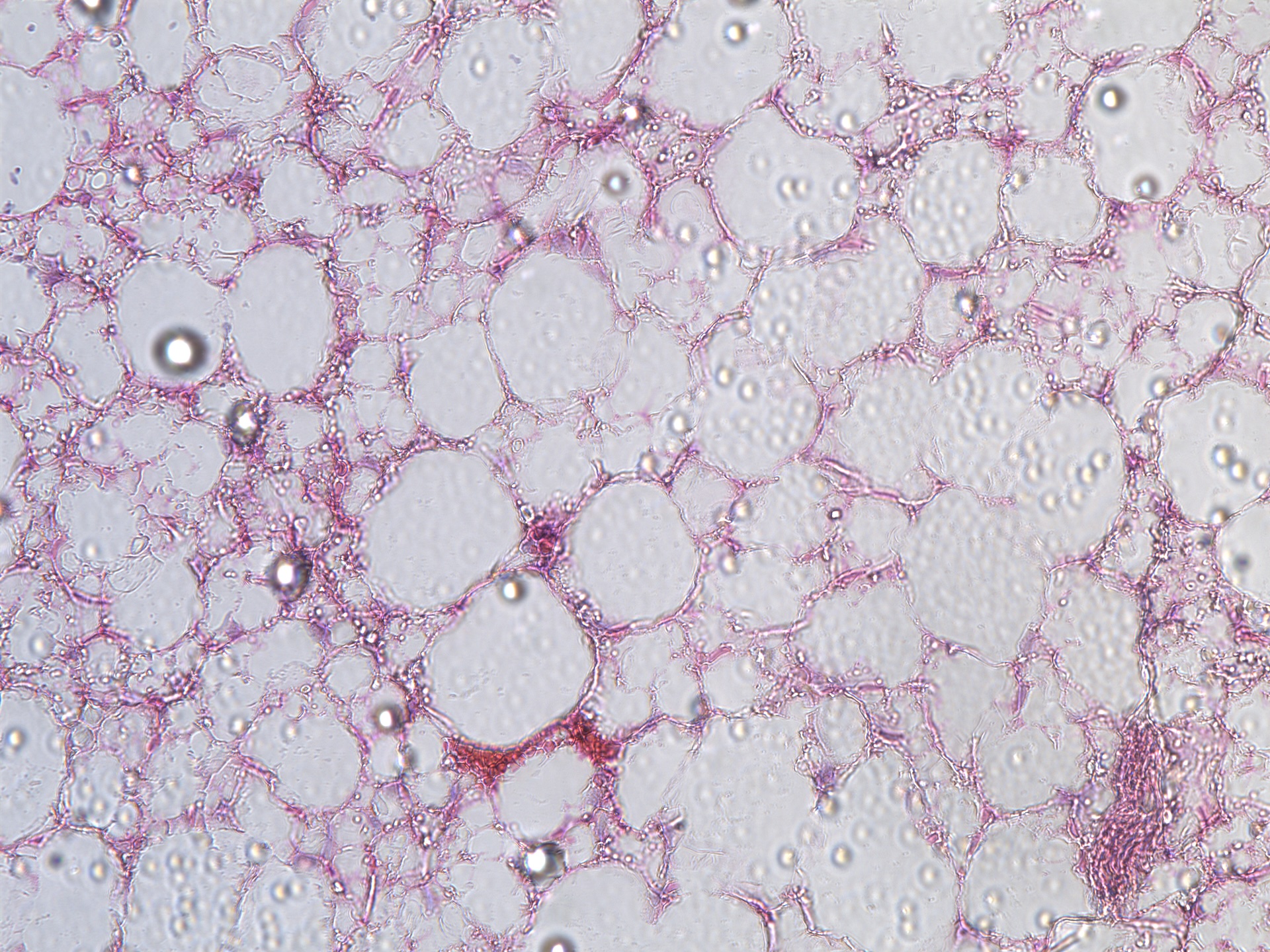

Supplement: Supplementary file 9 — Figure EV3 Source Data [file 44318_2024_196_MOESM9_ESM.zip › Figure EV3/Figure EV3-O/Additional replicate image/HFD PCPE-1 vaccine/no.2/HFD PCPE-1 vaacine-x40_03.jpg]

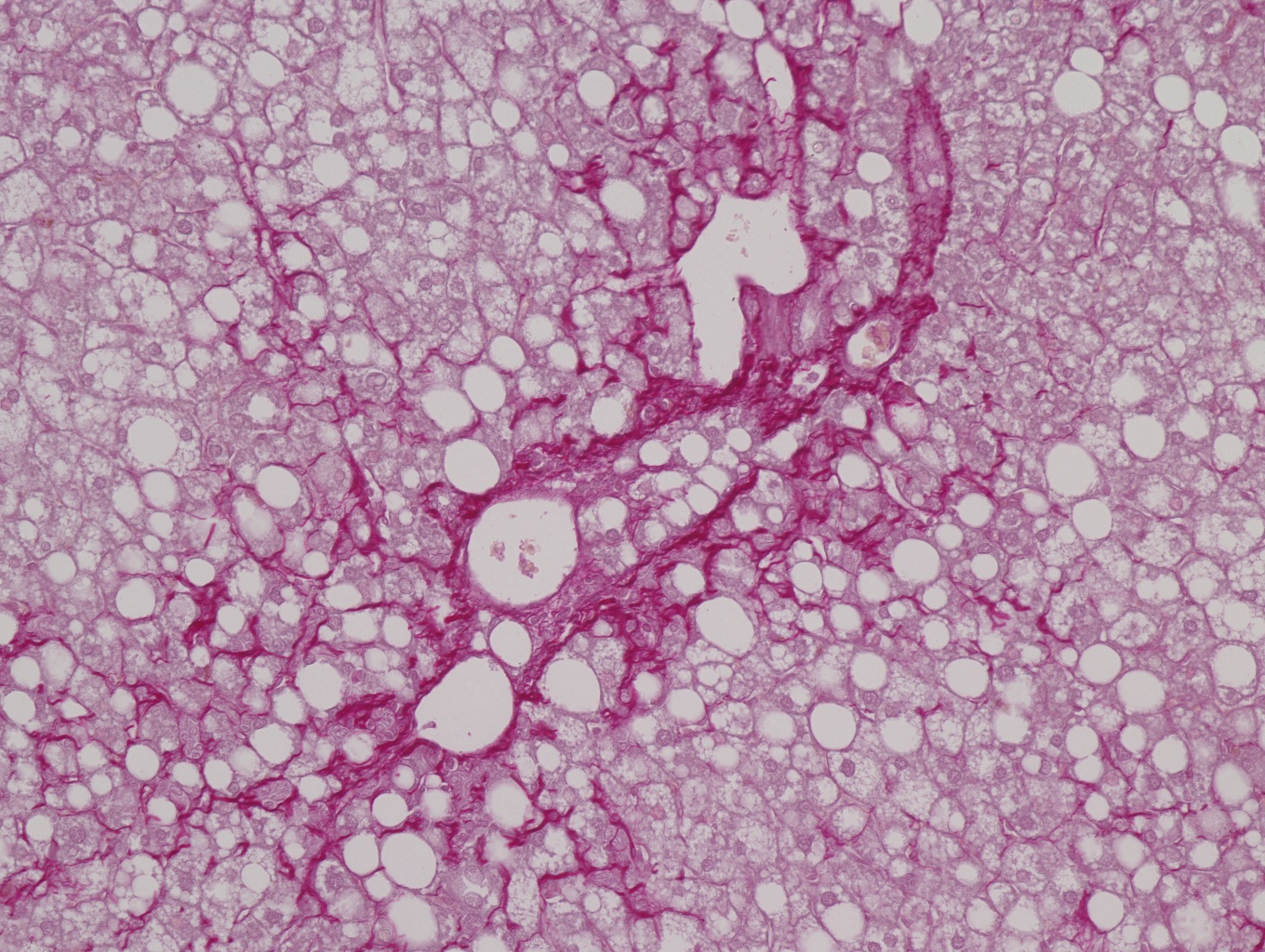

Supplement: Supplementary file 9 — Figure EV3 Source Data [file 44318_2024_196_MOESM9_ESM.zip › Figure EV3/Figure EV3-F/Quantificated image/HFD Con/no.1/HFD Con no.1 x20-3.jpg]

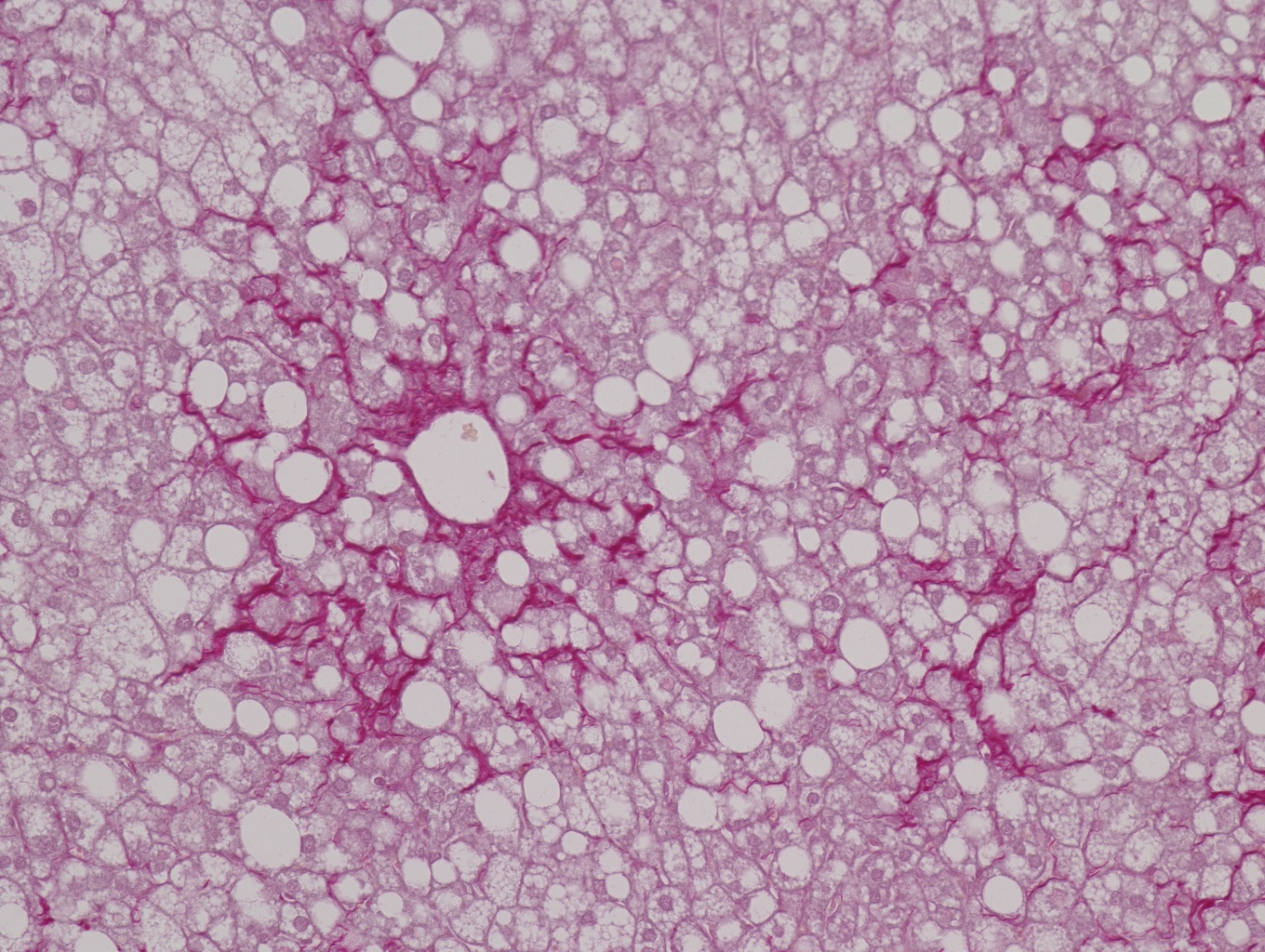

Supplement: Supplementary file 9 — Figure EV3 Source Data [file 44318_2024_196_MOESM9_ESM.zip › Figure EV3/Figure EV3-F/Quantificated image/HFD Con/no.1/HFD Con no.1 x20-2.jpg]

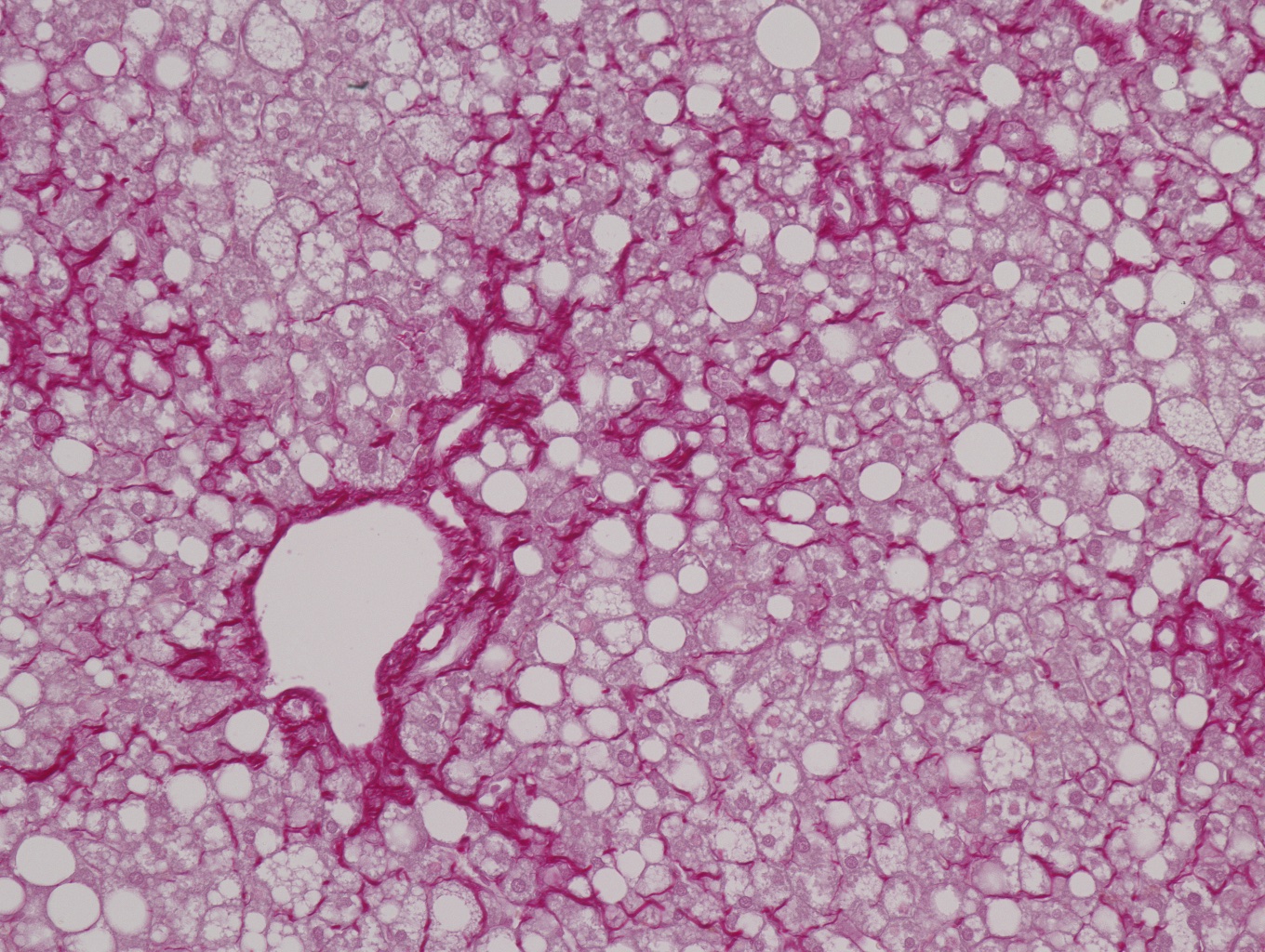

Supplement: Supplementary file 9 — Figure EV3 Source Data [file 44318_2024_196_MOESM9_ESM.zip › Figure EV3/Figure EV3-F/Quantificated image/HFD Con/no.1/HFD Con no.1 x20-1.jpg]

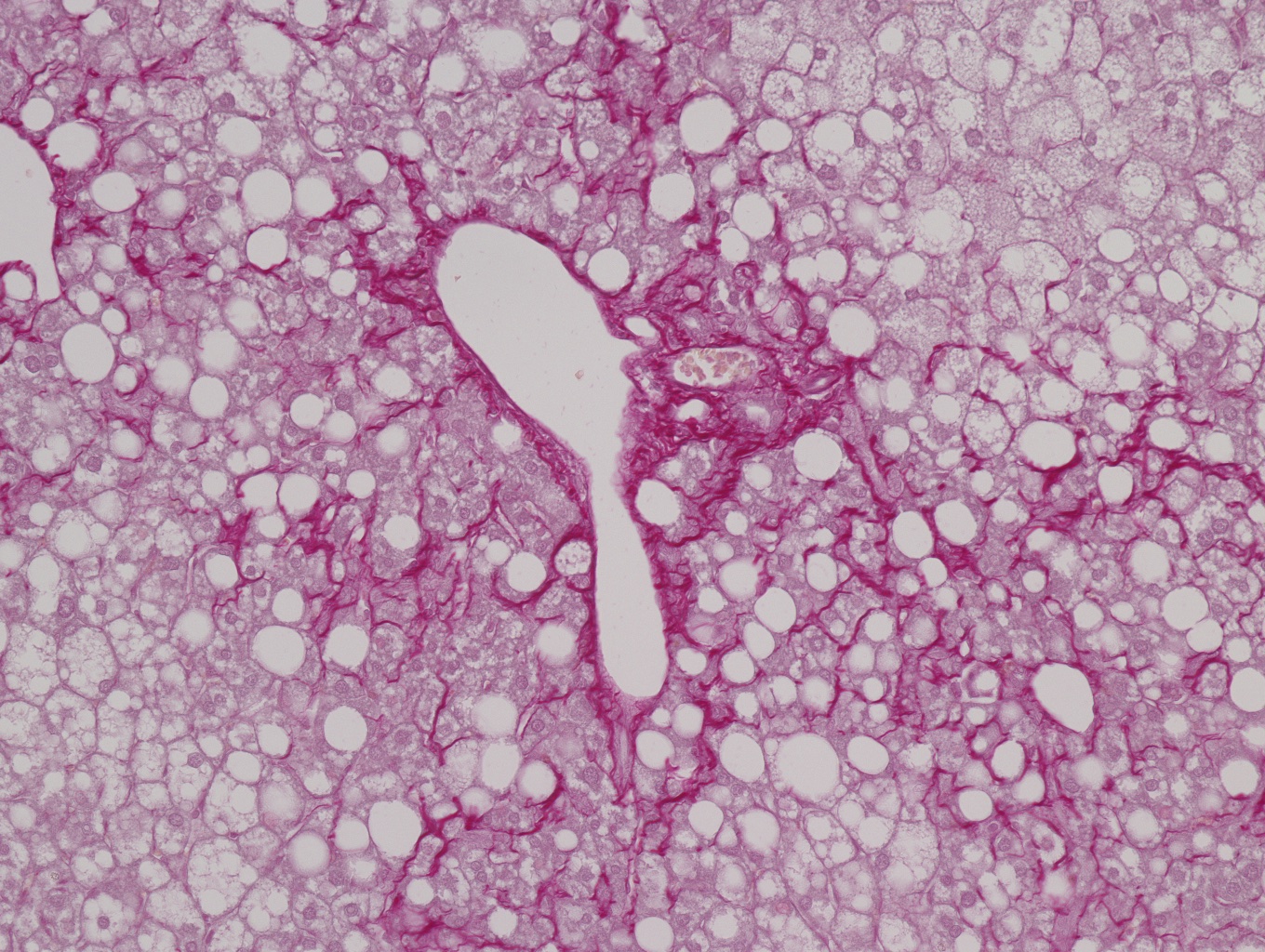

Supplement: Supplementary file 9 — Figure EV3 Source Data [file 44318_2024_196_MOESM9_ESM.zip › Figure EV3/Figure EV3-F/Quantificated image/HFD Con/no.1/HFD Con no.1 x20-4.jpg]

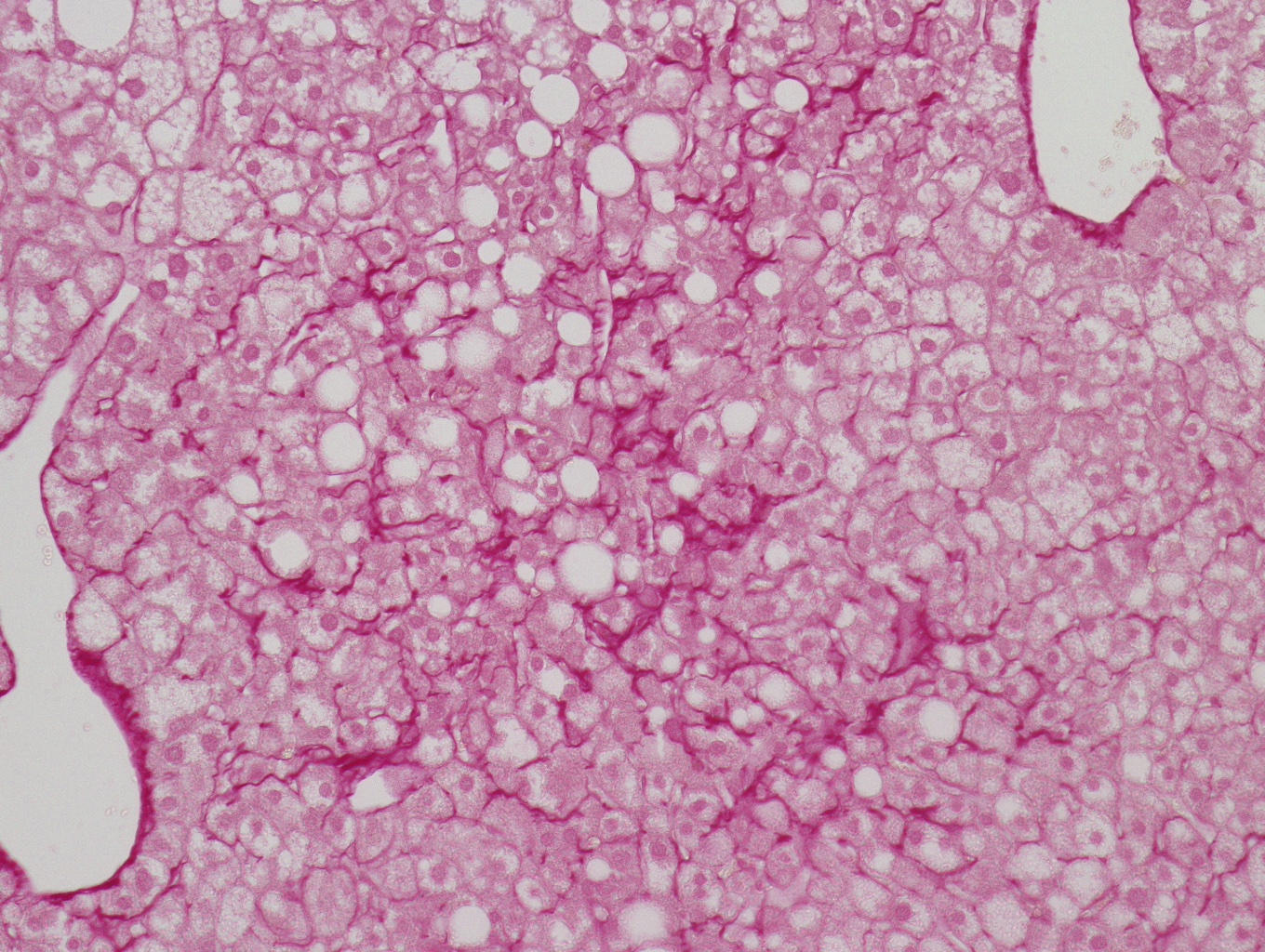

Supplement: Supplementary file 9 — Figure EV3 Source Data [file 44318_2024_196_MOESM9_ESM.zip › Figure EV3/Figure EV3-F/Quantificated image/HFD Con/no.3/HFD Con no.3 x20-2.jpg]

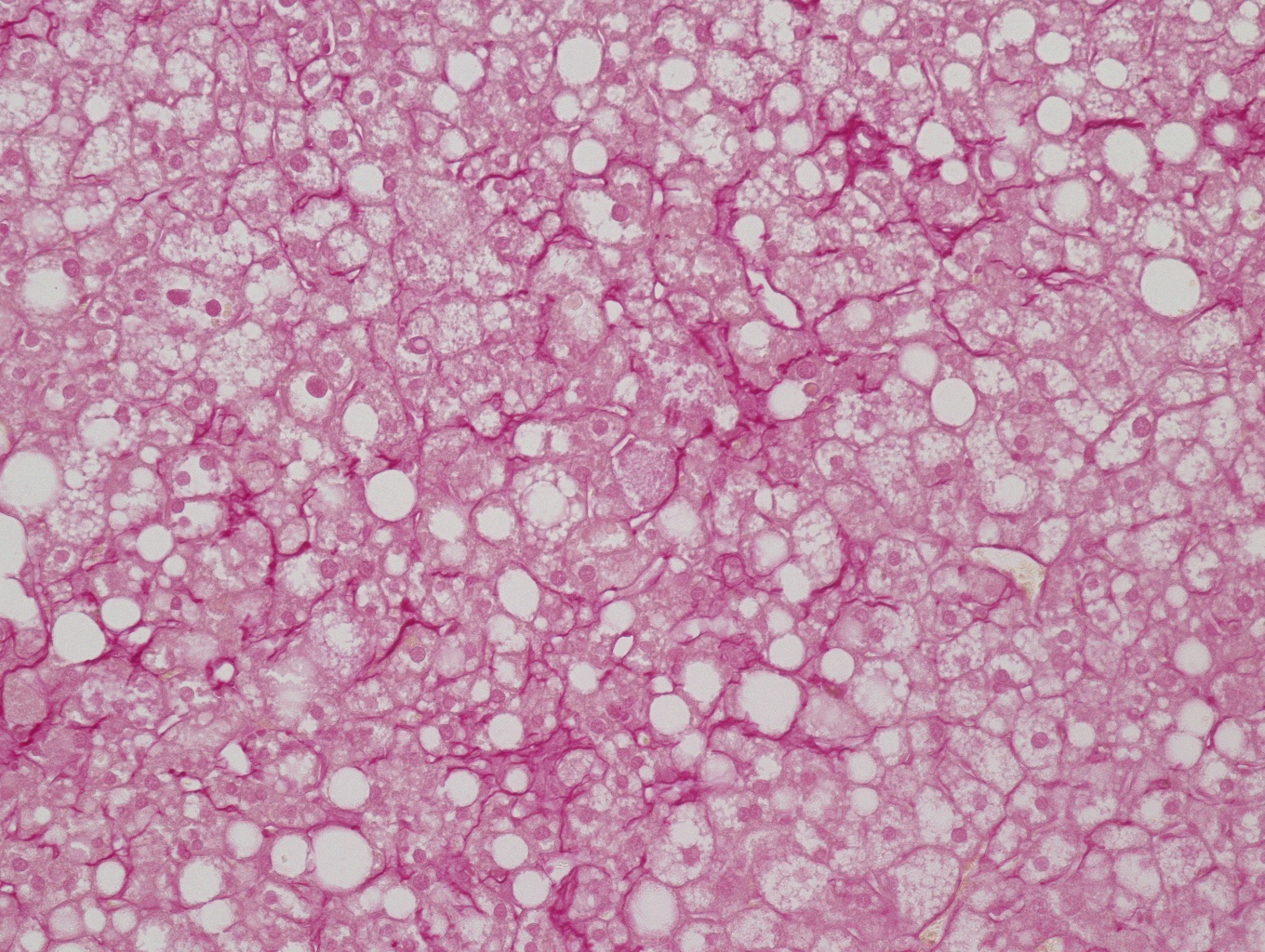

Supplement: Supplementary file 9 — Figure EV3 Source Data [file 44318_2024_196_MOESM9_ESM.zip › Figure EV3/Figure EV3-F/Quantificated image/HFD Con/no.3/HFD Con no.3 x20-3.jpg]

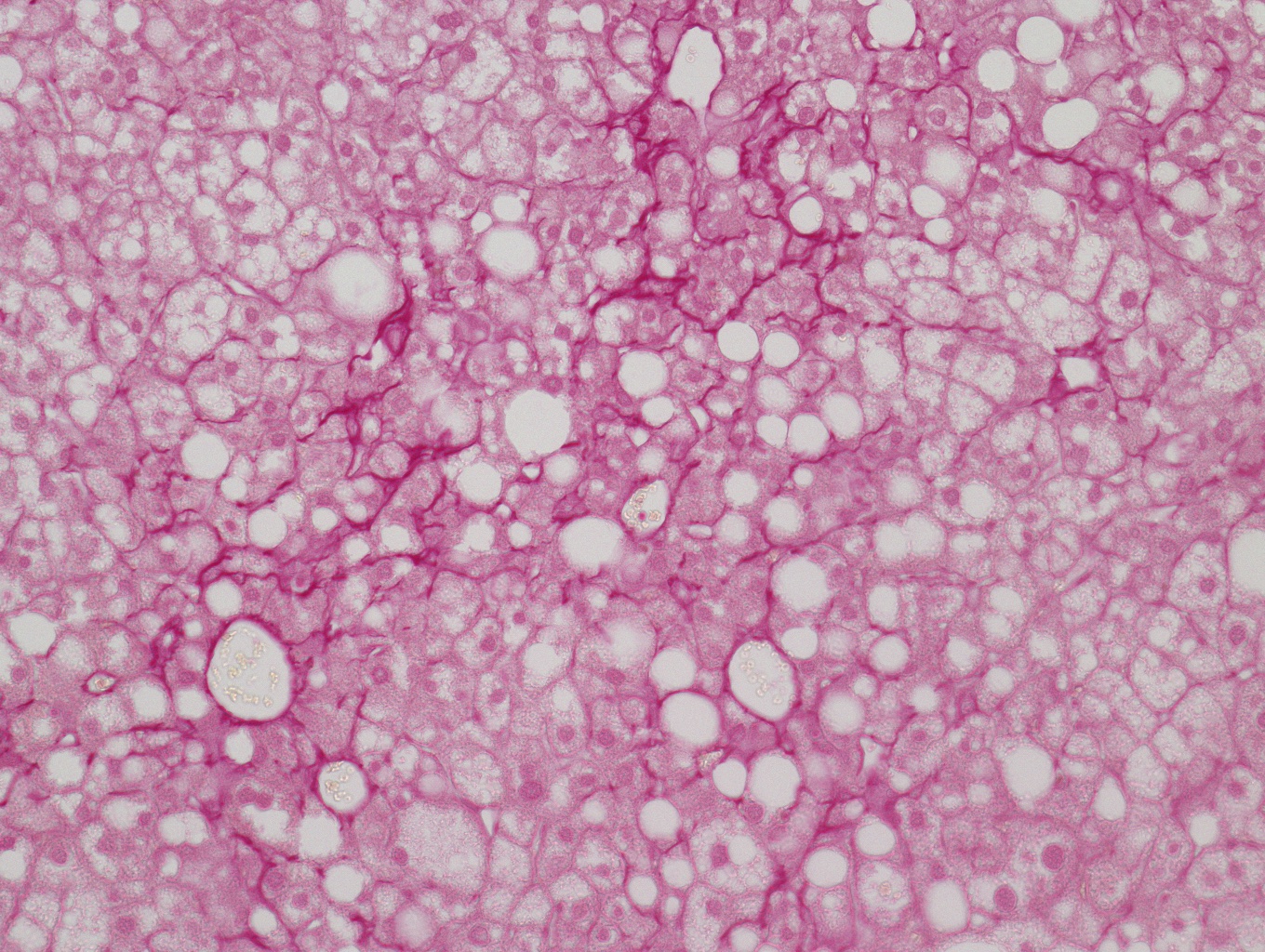

Supplement: Supplementary file 9 — Figure EV3 Source Data [file 44318_2024_196_MOESM9_ESM.zip › Figure EV3/Figure EV3-F/Quantificated image/HFD Con/no.3/HFD Con no.3 x20-1.jpg]

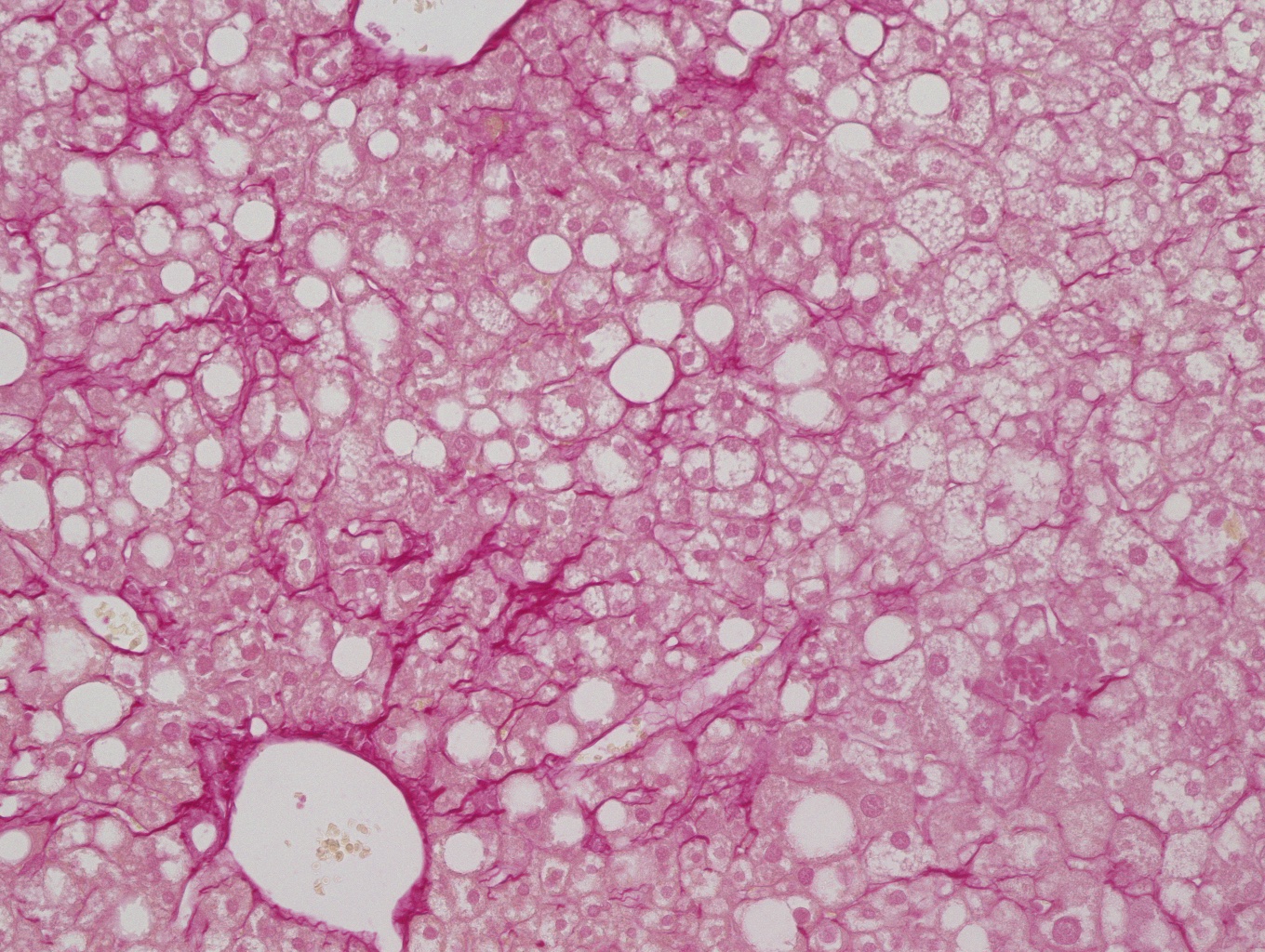

Supplement: Supplementary file 9 — Figure EV3 Source Data [file 44318_2024_196_MOESM9_ESM.zip › Figure EV3/Figure EV3-F/Quantificated image/HFD Con/no.3/HFD Con no.3 x20-4.jpg]

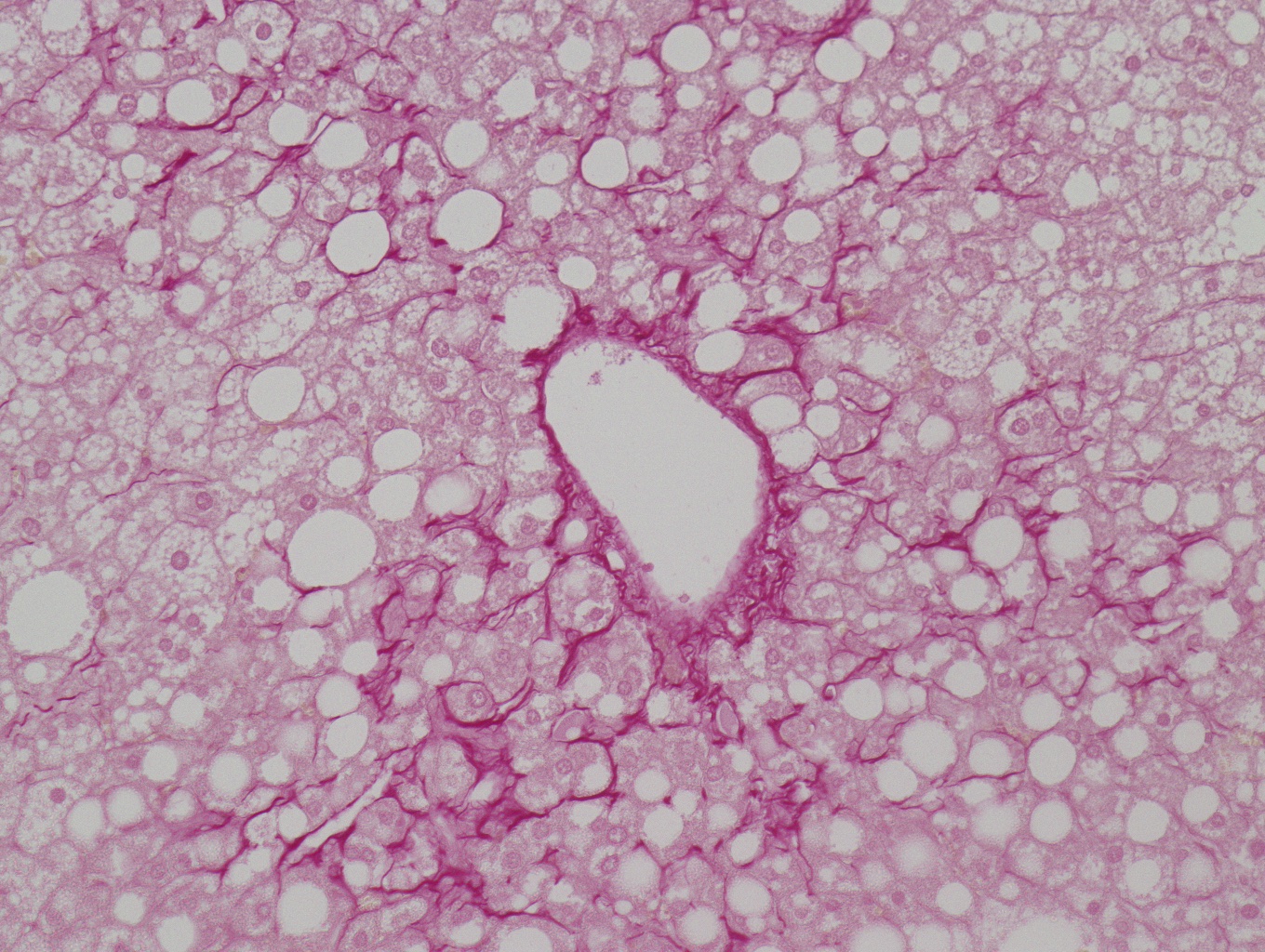

Supplement: Supplementary file 9 — Figure EV3 Source Data [file 44318_2024_196_MOESM9_ESM.zip › Figure EV3/Figure EV3-F/Quantificated image/HFD Con/no.4/HFD Con no.4 x20-4.jpg]

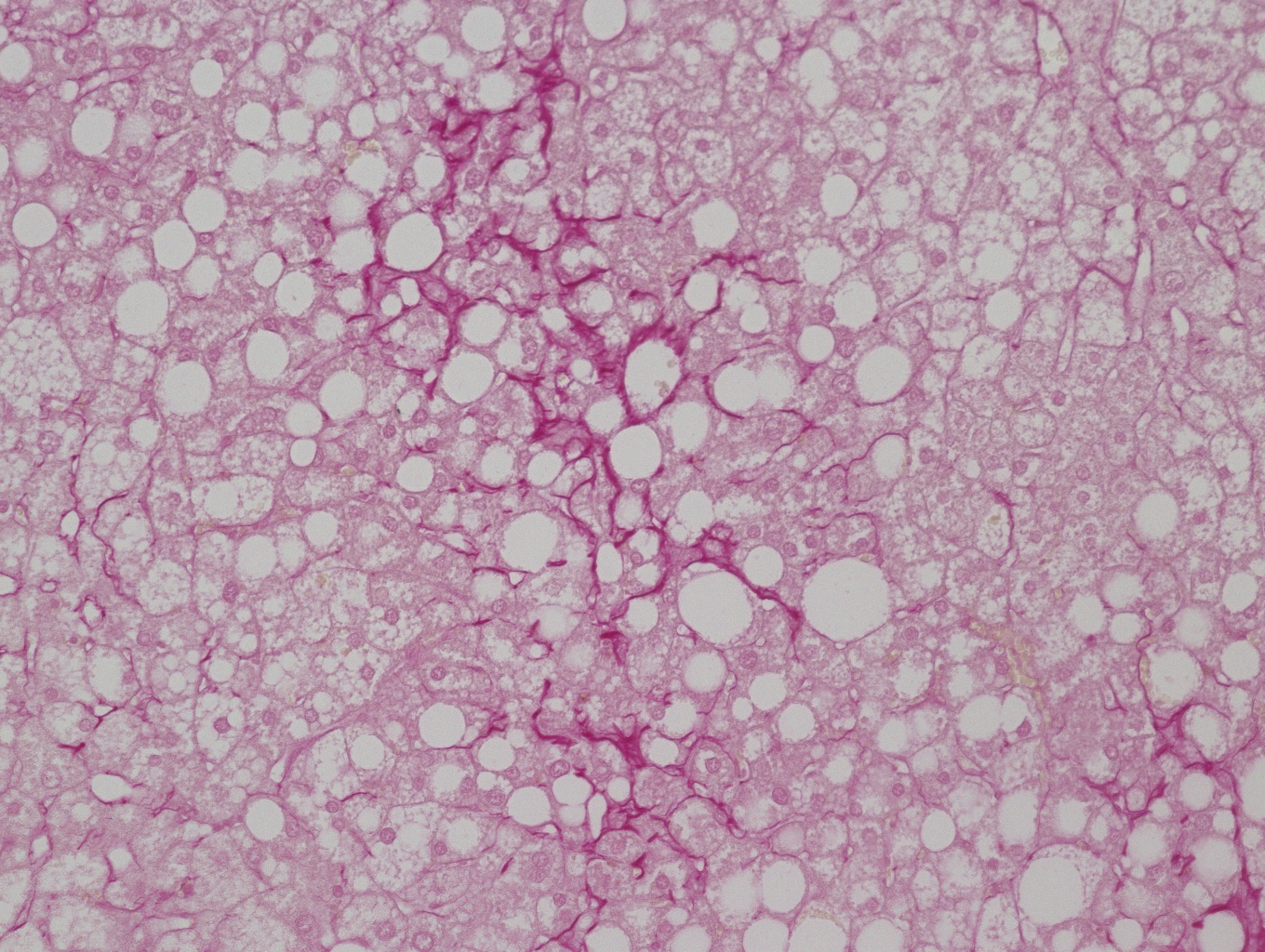

Supplement: Supplementary file 9 — Figure EV3 Source Data [file 44318_2024_196_MOESM9_ESM.zip › Figure EV3/Figure EV3-F/Quantificated image/HFD Con/no.4/HFD Con no.4 x20-3.jpg]

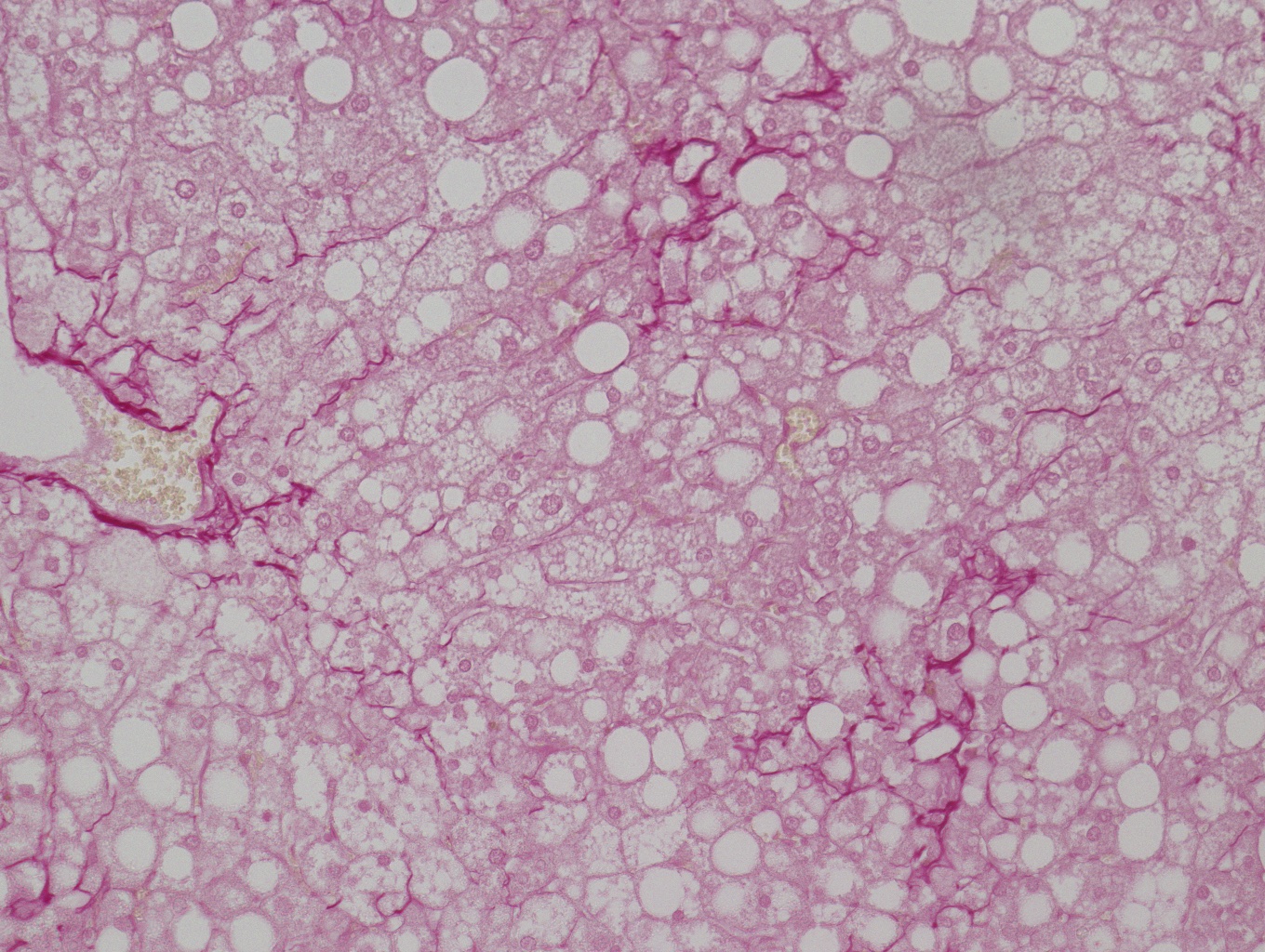

Supplement: Supplementary file 9 — Figure EV3 Source Data [file 44318_2024_196_MOESM9_ESM.zip › Figure EV3/Figure EV3-F/Quantificated image/HFD Con/no.4/HFD Con no.4 x20-2.jpg]
