# Supplementary material for: PCPE-1, a brown adipose tissue-derived cytokine, promotes obesity-induced liver fibrosis
Source: EMBO J. 2024 Aug 19;43(21):4846–69. doi: 10.1038/s44318-024-00196-0 (PMC11535236; doi:10.1038/s44318-024-00196-0)
Supplement: Supplementary file 12 — Expanded View Figures [file 44318_2024_196_MOESM12_ESM.pdf]

# Expanded View Figures

**Figure EV1. Related to Fig. 1.**

C57BL/6NCrSlc wild-type (WT) mice were fed with a normal chow (NC) or a high-fat diet (HFD) from 4 weeks of age and studied at 38–40 weeks of age. (**A**, **B**) Body weight (BW) (**A**) ($n$ = 4, 6) or brown adipose tissue (BAT) weight (**B**) ($n$ = 4, 5) of indicated mice. (**C**) Reference Expression Dataset (RefEx) showing transcript *Pcolce* in systemic organs. (**D–J**) Gene Expression Omnibus studies showing transcript *Pcolce* in eWAT and BAT (**D**) ($n$ = 3, 3 in GSE8044). The transcript was also tested under a HFD fed condition in mice in BAT (**E**) ($n$ = 3, 3 in GSE64718), eWAT ($n$ = 4, 4), liver ($n$ = 4, 4), skeletal muscle (SM) (**F**) ($n$ = 3, 4 in GSE123394), bone (**G**) ($n$ = 3, 4 in GSE194075), adrenal gland (**H**) ($n$ = 4, 4 in GSE216327), skin (**I**) ($n$ = 5, 5 in GSE96932) and in heart (**J**) ($n$ = 4, 4 in GSE171710). (**K–M**) Tabula Muris Senis testing transcript *Pcolce* in systemic organs (**K**), or in cells in the heart (**L**) or BAT (**M**). (**N–P**) Results from quantitative PCR (qPCR) showing transcript *Pcolce* in primary cardiac fibroblasts and primary brown adipocytes (**N**) ($n$ = 6, 6), primary hepatocytes and primary brown adipocytes (**O**) ($n$ = 5, 5), and hepatocyte (AML12) or brown adipocyte cell lines (**P**) ($n$ = 11, 12). (**Q**) Relative fibrotic area (area/view) in the liver of indicated mice related to Fig. 1J ($n$ = 4, 5). Data were analyzed by an independent-samples T-test. Data information: Representative data of two or more independent series (EV1A, B, N, P, Q), one independent series (EV1O). *$P < 0.05$, **$P < 0.01$. NS = not significant. Values represent the mean ± SEM. All data are from different biological replicates. Source data are available online for this figure.

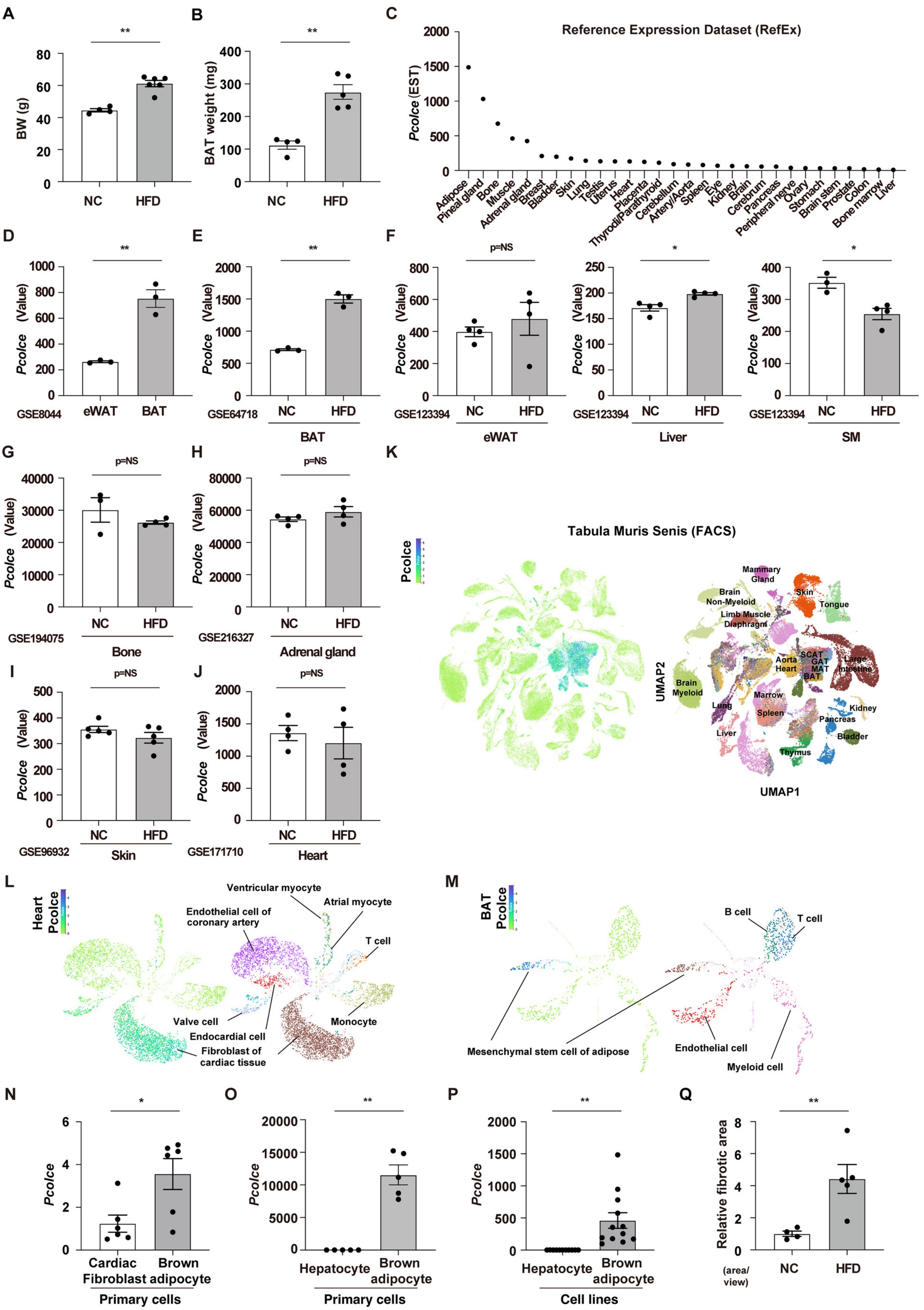
A
BW (g)
NC
HFD
B
BAT weight (mg)
C
Reference Expression Dataset (RefEx)
Pcolce (EST)
Adipose
Pineal gland
Bone
Muscle
Adrenal gland
Breast
Bladder
Skin
Lung
Testis
Uterus
Heart
Placenta
Thyroid/Parathyroid
Cerebellum
Artery/Aorta
Spleen
Eye
Kidney
Brain
Cerebrum
Pancreas
Peripheral nerve
Ovary
Stomach
Brain stem
Prostate
Colon
Bone marrow
Liver
D
Pcolce (Value)
GSE8044
eWAT
BAT
E
GSE64718
F
GSE123394
Liver
SM
G
GSE194075
Bone
H
GSE216327
Adrenal gland
K
Tabula Muris Senis (FACS)
Pcolce
UMAP2
UMAP1
Mammary Gland
Brain Non-Myeloid
Skin
Tongue
Limb Muscle
Diaphragm
Aorta
Heart
SCAT
GAT
MAT
BAT
Large Intestine
Brain Myeloid
Marrow
Lung
Spleen
Kidney
Pancreas
Liver
Bladder
Thymus
I
GSE96932
J
GSE171710
L
Heart Pcolce
Ventricular myocyte
Atrial myocyte
Endothelial cell of coronary artery
T cell
Valve cell
Monocyte
Endocardial cell
Fibroblast of cardiac tissue
M
BAT Pcolce
B cell
Mesenchymal stem cell of adipose
Endothelial cell
Myeloid cell
N
Pcolce
Cardiac Fibroblast
Brown adipocyte
Primary cells
O
Hepatocyte
P
Cell lines
Q
Relative fibrotic area
(area/ view)
p=NS

 © The Author(s)

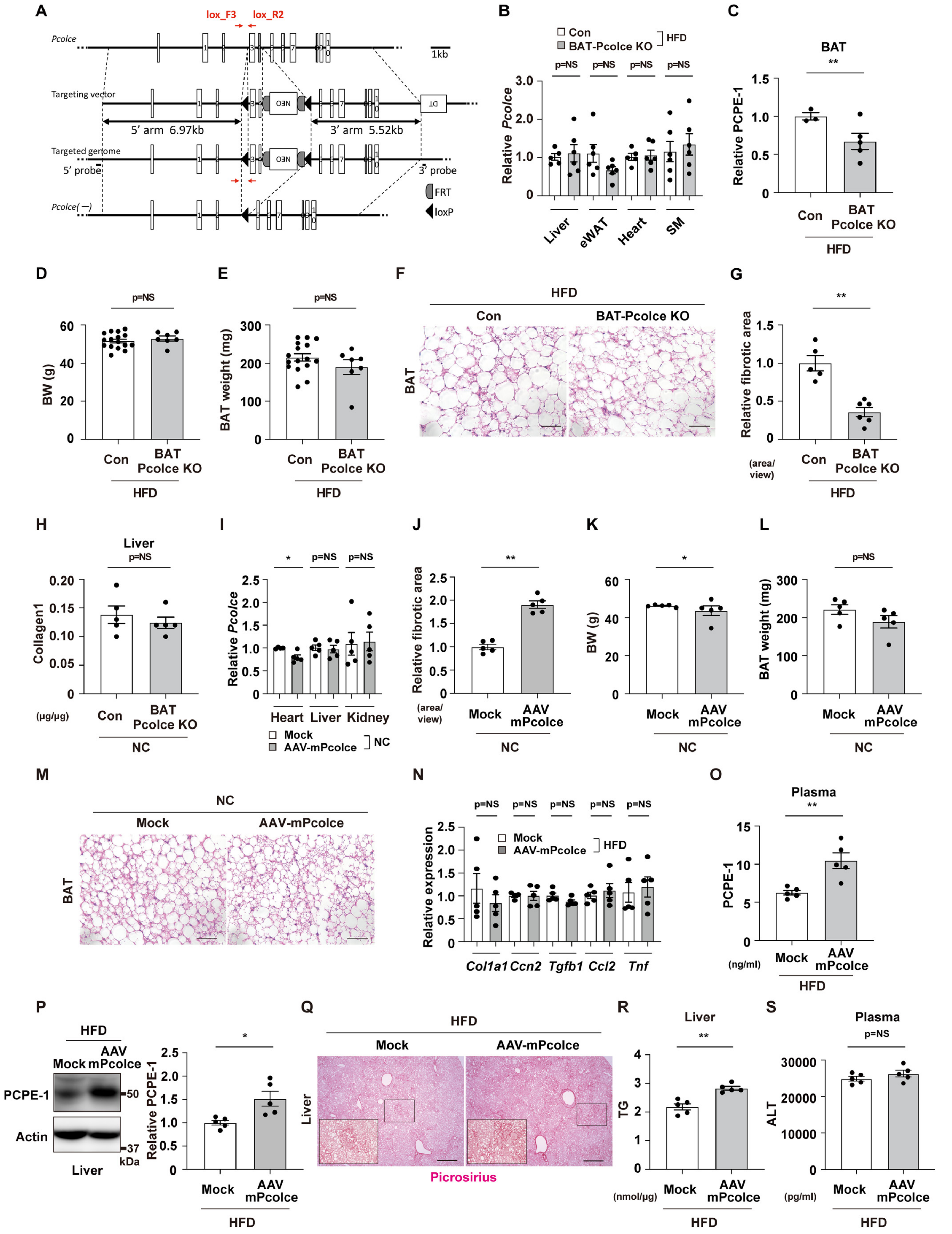
A
lox_F3 lox_R2
Pcolce
1kb
Targeting vector
5' arm 6.97kb
3' arm 5.52kb
Targeted genome
5' probe
3' probe
FRT
loxP
Pcolce(−)
B
Con
BAT-Pcolce KO
HFD
p=NS p=NS p=NS p=NS
Relative Pcolce
Liver eWAT Heart SM
C
BAT
Relative PCPE-1
Con BAT Pcolce KO
HFD
D
BW (g)
E
BAT weight (mg)
F
HFD
Con BAT-Pcolce KO
BAT
G
Relative fibrotic area
(area/view)
H
Liver
Collagen1
(μg/μg)
NC
I
Relative Pcolce
Heart Liver Kidney
Mock
AAV-mPcolce
NC
J
Relative fibrotic area
Mock AAV mPcolce
K
BW (g)
L
BAT weight (mg)
M
NC
Mock AAV-mPcolce
BAT
N
Relative expression
Col1a1 Ccn2 Tgfb1 Ccl2 Tnf
O
Plasma
PCPE-1
(ng/ml)
P
HFD
PCPE-1
Actin
Liver
kDa
Relative PCPE-1
Q
HFD
Mock AAV-mPcolce
Liver
Picrosirius
R
Liver
TG
(nmol/μg)
S
Plasma
ALT
(pg/ml)

**Figure EV2. Related to Fig. 2.**

Brown adipose tissue (BAT)-specific *Pcolce* knockout (KO) mice (UCP1-Cre$^{+/-}$; *Pcolce*$^{flox/flox}$; BAT *Pcolce* KO) or littermate control mice (UCP1-Cre$^{-/-}$; *Pcolce*$^{flox/flox}$; Con) were fed a high-fat diet (HFD) from 4 weeks of age and studied at 42–43 weeks of age. (**A**) Design of the BAT *Pcolce* KO mice. (**B**) Results from quantitative PCR (qPCR) showing transcript *Pcolce* in the liver (*n* = 5, 6), epididymal white adipose tissue (eWAT) (*n* = 6, 6), heart (*n* = 5, 6), and skeletal muscle (SM) (quadriceps) (*n* = 6, 6) from indicated mice fed a HFD. (**C**) The quantification of western blot analysis for PCPE-1 relative to Actin in BAT (*n* = 3, 5) related to Fig. 2B. (**D**, **E**) Body weight (BW) (**D**) (*n* = 16, 7), or BAT weight (**E**) (*n* = 16, 7) of indicated mice. (**F**) HE staining of the BAT from the indicated mice. Scale bar = 50 μm. (**G**) Relative fibrotic area in liver in the indicated mice, related to Fig. 2E (*n* = 5, 6). (**H**) ELISA for liver collagen type I (Collagen1) in the indicated mice (*n* = 5, 5). (**I**) Results from quantitative PCR (qPCR) showing transcript *Pcolce* in heart (*n* = 4, 5), liver (*n* = 5, 5) and kidney (*n* = 5, 5) in the indicated mice. (**J**) Relative fibrotic area of the liver in BAT AAV-Pcolce injection model (AAV-mPcolce) maintained on a normal chow (NC) related to Fig. 2N (58 weeks of age) (*n* = 5, 5). (**K**, **L**) Body weight (BW) (**K**) (n = 5, 5), or BAT weight (**L**) (*n* = 5, 5) of indicated mice. (**M**) HE staining of the BAT from the indicated mice. Scale bar = 50 μm. (**N**) Results from quantitative PCR (qPCR) showing transcripts *Col1a1* (*n* = 5, 5), *Ccn2*(Ctgf) (*n* = 4, 5), *Tgfb1* (*n* = 5, 5), *Ccl2* (*n* = 5, 5), and *Tnf* (*n* = 5, 5) in the liver from the indicated mice. (**O**) ELISA for PCPE-1 in plasma from the indicated mice (*n* = 5, 5). (**P**) Western blot analysis of PCPE-1 expression in liver from the indicated mice. The right panel indicates the quantification of PCPE-1 relative to Actin (*n* = 5, 5). (**Q**) Picrosirius red staining of liver from the indicated mice. Scale bar = 500 μm. The quantification results see Figure Appendix Figure S2H. (**R**, **S**) ELISA for liver triglyceride (TG) (**R**) (*n* = 5, 5) or plasma alanine transaminase (ALT) (**S**) (*n* = 5, 5) in the indicated mice. All data were analyzed by an independent-samples T-test. Data information: Representative data of two or more independent series (EV2B–E, G), one independent series (EV2I–L, N, P), one independent experiment analyzing samples from at least 2 independently prepared samples (EV2H, O, R, S). $^{*}P < 0.05$, $^{**}P < 0.01$. NS = not significant. Values represent the mean ± SEM. All data are from different biological replicates. Source data are available online for this figure.


© The Author(s)

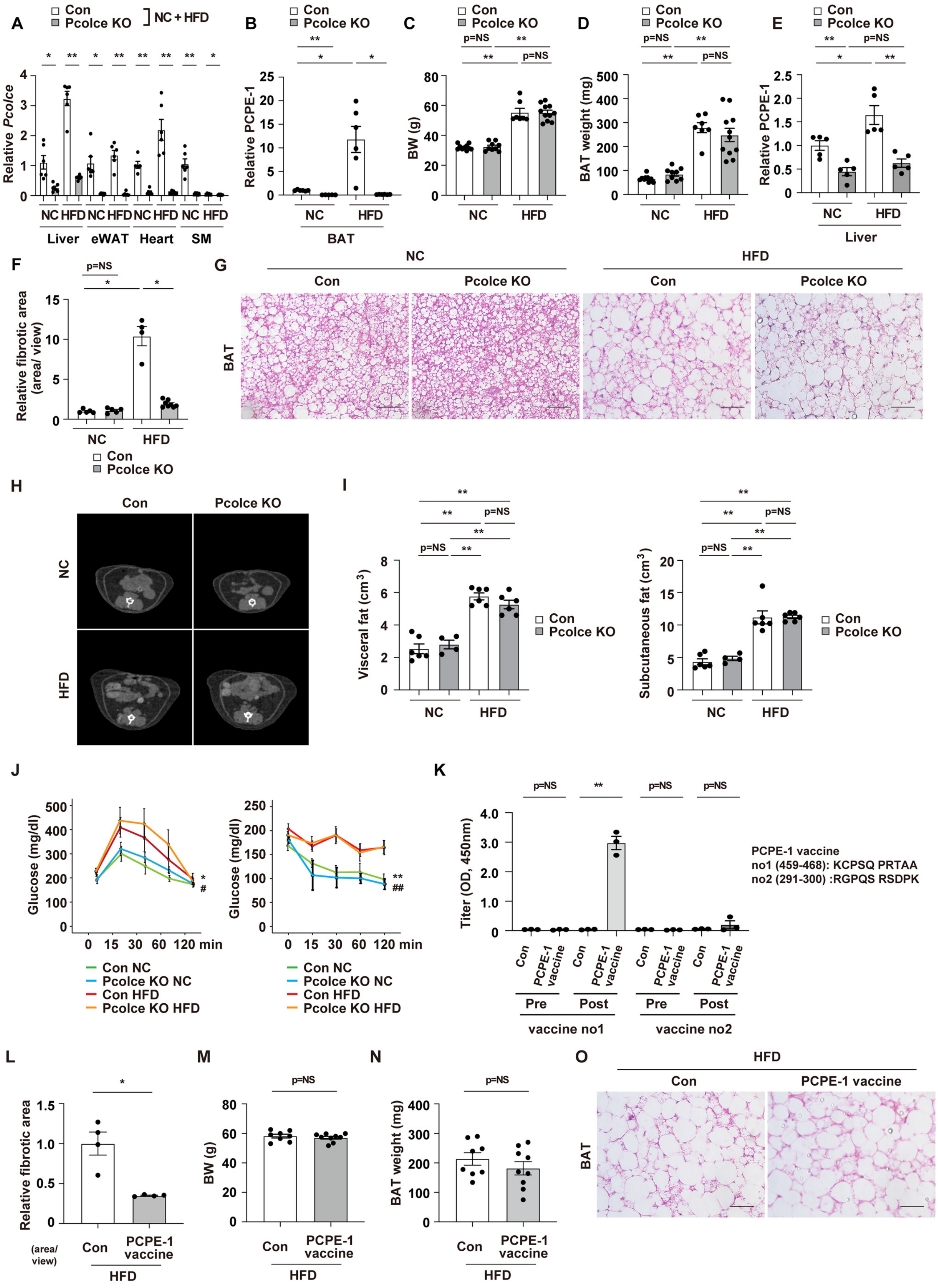
A
Con
Pcolce KO
NC + HFD
Relative Pcolce
NC HFD NC HFD NC HFD NC HFD
Liver eWAT Heart SM
B
Relative PCPE-1
NC HFD
BAT
C
BW (g)
p=NS
D
BAT weight (mg)
E
Relative PCPE-1
Liver
F
Relative fibrotic area (area/ view)
G
NC
HFD
Con
Pcolce KO
BAT
H
I
Visceral fat (cm3)
Subcutaneous fat (cm3)
J
Glucose (mg/dl)
0 15 30 60 120 min
Con NC
Pcolce KO NC
Con HFD
Pcolce KO HFD
K
Titer (OD, 450nm)
PCPE-1 vaccine
no1 (459-468): KCPSQ PRTAA
no2 (291-300) :RGPQS RSDPK
Con
PCPE-1 vaccine
Pre
Post
vaccine no1
vaccine no2
L
Relative fibrotic area
(area/ view)
M
BW (g)
N
BAT weight (mg)
O
HFD

**Figure EV3. Related to Fig. 3.**

Systemic *Pcolce* knockout (*Pcolce* KO) or littermate control (Con) mice were maintained on a HFD from 4 weeks of age, and tissues were harvested and analyzed at 38–44 weeks of age. (**A**) Results from quantitative PCR (qPCR) showing transcript *Pcolce* in the liver, epididymal white adipose tissue (eWAT), heart, and skeletal muscle (SM) (quadriceps) from the indicated mice (heart: $n = 5, 6, 6, 6$, all other organs: $n = 6, 6, 6, 6$). (**B**) The quantification of western blot analysis for PCPE-1 relative to GAPDH in BAT related to Fig. 3B ($n = 5, 6, 6, 6$). (**C**, **D**) Body weight (BW) ($n = 10, 9, 7, 11$) (**C**), or BAT weight ($n = 10, 9, 7, 11$) (**D**) of indicated mice. (**E**) The quantification of western blot analysis for PCPE-1 relative to Actin in liver related to Fig. 3D ($n = 5, 5, 5, 5$). (**F**) Relative fibrotic area in the liver of indicated mice, related to Fig. 3E ($n = 5, 5, 4, 8$). (**G**) HE staining of the BAT from the indicated mice. Scale bar = 50 μm. (**H**) CT scan analyzing the abdominal area of indicated mice aged 29 weeks of age. (**I**) Volume of visceral or subcutaneous fat in the subdiaphragm abdominal area analyzed with a CT scan ($n = 6, 4, 6, 6$). (**J**) Glucose tolerance test (GTT) ($n = 6, 4, 8, 8$) or insulin tolerance test (ITT) ($n = 6, 4, 8, 7$) of the indicated mice. * and ** indicates Con NC vs Con HFD. # and ## indicates Pcolce KO NC vs Pcolce KO HFD. (**K**) The antibody titer in plasma was studied at pre- and post-immunization with two antigenic PCPE-1 peptide sequences (vaccine no.1 (KCPSQ PRTAA) and no.2 (RGPQS RSDPK)) ($n = 3, 3, 3, 3, 3, 3, 3, 3$). (**L**) Relative fibrotic area in the liver of indicated mice, related to Fig. 3L ($n = 4, 4$). (**M**, **N**) Body weight (BW) (**M**) ($n = 8, 9$), and BAT weight (**N**) ($n = 8, 9$) of mice with or without PCPE-1 vaccination (vaccine no.1). (**O**) HE staining of the BAT from the indicated mice. Scale bar = 50 μm. All mice were maintained on a HFD and analyzed at 47 weeks age. The data in (**B**–**D**), (**F**), (**I**) were analyzed by a two-way analysis of variance (ANOVA) followed by Tukey's multiple comparison test (**I**), Dunnett test (**F**), or non-parametric Kruskal Wallis test (**C**, **D**). All the values in (**A**) were analyzed by a two-way ANOVA followed by Dunnett test except for NC in liver and HFD groups in SM analyzed by the independent-samples T-test. In (**B**), Con NC vs Con HFD was analyzed with the independent-samples T-test and other values were analyzed by a two-way ANOVA followed by Dunnett test. The data in (**J**) were analyzed with two-way repeated measures ANOVA. Other data were analyzed by an independent-samples T-test. Data information: Representative data of two or more independent series (EV3A–F, L–N), one independent series (EV3I–K). * and $^{\#}P < 0.05$, ** and $^{\#\#}P < 0.01$. NS = not significant. Values represent the mean ± SEM. All data are from different biological replicates. Source data are available online for this figure.

 © The Author(s)

A

Putative promotor region for Pcolce

| Transcriptional factor | Score | Relative score | Start | End | Strand | predicted site sequence |
|---|---|---|---|---|---|---|
| cJUN::cFOS | 5.682 | 0.818149714 | 15 | 21 | 1 | TGAAAGA |
| cJUN::cFOS | 6.687 | 0.854833858 | 215 | 221 | 1 | TGAAGCA |
| cJUN::cFOS | 6.574 | 0.850709173 | 304 | 310 | −1 | GGACTCA |
| cJUN::cFOS | 5.521 | 0.81227295 | 566 | 572 | −1 | TGCGTCA |
| cJUN::cFOS | 7.794 | 0.89524117 | 566 | 572 | 1 | TGACGCA |
| cJUN::cFOS | 8.185 | 0.90951331 | 600 | 606 | 1 | TGCCTCA |
| cJUN::cFOS | 6.656 | 0.853702308 | 719 | 725 | 1 | TGACTCC |
| cJUN::cFOS | 5.466 | 0.81026536 | 862 | 868 | −1 | GGAATCA |
| cJUN::cFOS | 6.656 | 0.853702308 | 974 | 980 | −1 | TGACTCC |
| cJUN::cFOS | 7.573 | 0.887174308 | 990 | 996 | −1 | TTAATCA |
| cJUN::cFOS | 6.789 | 0.858557025 | 1026 | 1032 | −1 | TGACAGA |
| cJUN::cFOS | 6.201 | 0.837094063 | 1185 | 1191 | 1 | TTCCTCA |
| cJUN::cFOS | 6.696 | 0.855162373 | 1189 | 1195 | −1 | TTACTGA |

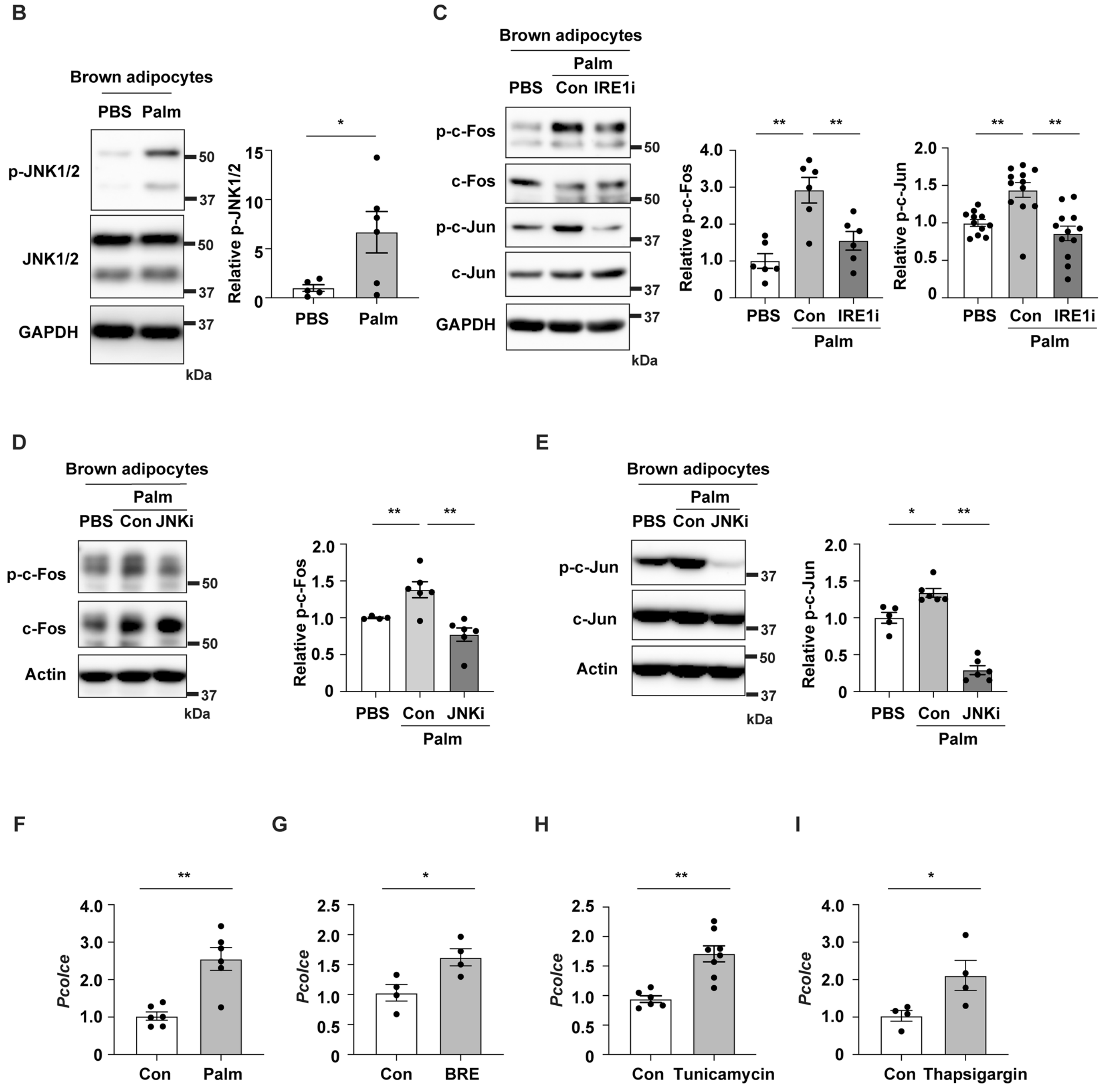

**Figure EV4. Related to Fig. 4.**

(**A**) Results of in silico analysis (DBTSS [http://dbtss.hgc.jp] and JASPAR [http://jaspar.binf.ku.dk]) predicting c-Fos and c-Jun may bind to putative promotor region of *Pcolce*. (**B**) Western blot analysis for phospho-JNK1/2 (p-JNK1/2) and JNK1/2 in fully differentiated brown adipocytes administrated with or without palmitic acid (Palm, 100 μM). The right panel indicates the quantification of p-JNK1/2 ($n = 6, 6$) relative to GAPDH. (**C–E**) Western blot analysis for p-c-Fos, c-Fos, p-c-Jun, and c-Jun in fully differentiated brown adipocytes administrated with PBS, palmitic acid (Palm; 100 μM for 6 h) with or without an IRE1 inhibitor (STF-083010, 30 μM, pre-treated 1 h) (**C**) or a JNK inhibitor (SP600125, 2 μM, pre-treated 1 h) (**D**, **E**). The right panels indicate the quantification of p-c-Fos and p-c-Jun relative to GAPDH or Actin (**C**: $n = 6, 6, 6$ for p-c-Fos, $n = 11, 12, 12$ for p-c-Jun) (**D**: $n = 4, 6, 6$) (**E**: $n = 5,6,6$). (**F–I**) Results from quantitative PCR (qPCR) showing transcript *Pcolce* in the differentiated brown adipocytes administrated with Palm (**E**) ($n = 6, 6$), BRE (Brefeldin A) (**G**) ($n = 4, 4$), Tunicamycin (**H**) ($n = 6, 8$) or Thapsigargin (**I**) ($n = 4, 4$). Data in Fig. EV4C–E were analyzed by a two-way analysis of variance (ANOVA) followed by Tukey's multiple comparison test. Other data were analyzed by an independent-samples T-test. Data information: Representative data of two or more independent series (EV4B–I). $^*P < 0.05$, $^{**}P < 0.01$. NS = not significant. Values represent the mean ± SEM. All data are from different biological replicates. Source data are available online for this figure.

 © The Author(s)
